# A foundation monograph of *Ipomoea* (Convolvulaceae) in the New World

**DOI:** 10.3897/phytokeys.143.32821

**Published:** 2020-03-16

**Authors:** John R.I. Wood, Pablo Muñoz-Rodríguez, Bethany R.M. Williams, Robert W. Scotland

**Affiliations:** 1 University of Oxford, Oxford, UK University of Oxford Oxford United Kingdom; 2 Honorary Research Associate, Royal Botanic Garden, Kew, UK Royal Botanic Garden Kew United Kingdom

**Keywords:** America, *
Batatas
*, Convolvulaceae, distribution, illustrations, keys, lectotypification, monograph, morning glory, new taxa, *
Pharbitis
*, *
Quamoclit
*, revision, storage roots, sweet potato, synonymy

## Abstract

A monograph of the 425 New World species of *Ipomoea* is presented. All 425 species are described and information is provided on their ecology and distribution, with citations from all countries from which they are reported. Notes are provided on salient characteristics and taxonomic issues related to individual species. A full synonymy is provided and 272 names are lectotypified. An extensive introduction discusses the delimitation and history of *Ipomoea* arguing that a broad generic concept is the only rational solution in the light of recent phylogenetic advances. Although no formal infrageneric classification is proposed, attention is drawn to the major clades of the genus and several morphologically well-defined clades are discussed including those traditionally treated under the names *Arborescens*, *Batatas*, *Pharbitis*, *Calonyction* and *Quamoclit*, sometimes as distinct genera, subgenera, sections or series. Identification keys are provided on a regional basis including multi-entry keys for the main continental blocks. Six species are described as new, *Ipomoea nivea* J.R.I. Wood & Scotland from Peru, *I. apodiensis* J.R.I. Wood & Scotland from Brazil, *I. calcicola* J.R.I. Wood & Scotland, *I. pochutlensis* J.R.I. Wood & Scotland, *I. zacatecana* J.R.I. Wood & Scotland and *I. ramulosa* J.R.I. Wood & Scotland from Mexico, while var. australis of *I. cordatotriloba* is raised to specific status as *I. australis* (O’Donell) J.R.I. Wood & P. Muñoz. New subspecies for *I. nitida* (subsp. krapovickasii J.R.I. Wood & Scotland) and for *I. chenopodiifolia* (subsp. bellator J.R.I. Wood & Scotland) are described. The status of previously recognized species and varieties is changed so the following new subspecies are recognized: I. amnicola subsp. chiliantha (Hallier f.) J.R.I. Wood & Scotland, I. chenopodiifolia subsp. signata (House) J.R.I. Wood & Scotland, I. orizabensis subsp. collina (House) J.R.I. Wood & Scotland, I. orizabensis subsp. austromexicana (J.A. McDonald) J.R.I. Wood & Scotland, I. orizabensis subsp. novogaliciana (J.A. McDonald) J.R.I. Wood & Scotland, I. setosa subsp. pavonii (Hallier f.) J.R.I. Wood & Scotland, I. setosa subsp. melanotricha (Brandegee) J.R.I. Wood & Scotland, I. setosa subsp. sepacuitensis (Donn. Sm.) J.R.I. Wood & Scotland, I. ternifolia subsp. leptotoma (Torr.) J.R.I. Wood & Scotland. *Ipomoea angustata* and I. subincana are treated as var. angustata (Brandegee) J.R.I. Wood & Scotland and var. subincana (Choisy) J.R.I. Wood & Scotland of *I. barbatisepala* and *I. brasiliana* respectively. Attention is drawn to a number of hitherto poorly recognized phenomena in the genus including a very large radiation centred on the Parana region of South America and another on the Caribbean Islands, a strong trend towards an amphitropical distribution in the New World, the existence of a relatively large number of species with a pantropical distribution and of many species in different clades with storage roots, most of which have never been evaluated for economic purposes. The treatment is illustrated with over 200 figures composed of line drawings and photographs.

## Introduction

This monograph of *Ipomoea* L. in the New World follows on from our monograph of *Convolvulus* (Wood et al. 2015). *Ipomoea*, as here interpreted, is a large pantropical genus of about 800 species. We have studied the genus worldwide and supporting papers (Wood et al. 2015, 2016a, 2016b, 2017a, 2017b, 2017c, 2017d, 2018; Wood and Scotland 2017a, 2017b, 2017c; Muñoz-Rodríguez et al. 2018, 2019) reflect this comprehensive overview, but for pragmatic reasons this monograph treats in detail only those species recorded from the New World (i.e. the American continent and associated islands including Easter Island, the Galapagos Islands and Hawaii in the Pacific together with Bermuda, Bahamas and the Caribbean Islands on the Atlantic side).

We have developed the ‘foundation monograph’ concept at Oxford as an approach to overhauling the taxonomy of species-rich groups of tropical plants since many of these groups have never been studied across their entire geographical distribution as a consequence of the pragmatic and local nature of much taxonomy. Inevitably, these groups contain undiscovered species, high levels of undetected synonymy, and identification keys are absent or limited. A major challenge in monographing these groups is the size of the task given the number of species, their global distribution and extensive synonymy, the large and increasing number of specimens, the numerous and dispersed herbaria where specimens are housed and an extensive, scattered and often obscure literature. Our approach seeks to focus on those tasks that are tractable and can offer the maximum improvement in taxonomic knowledge in a given period of time. It is novel in the sense that we combine standard taxonomic techniques with the use of online digital images and molecular sequence data to focus on species level taxonomic problems across the entire distribution range of individual species. A detailed account of our approach is available in Muñoz-Rodríguez et al. (2019).

**Figure 1.**
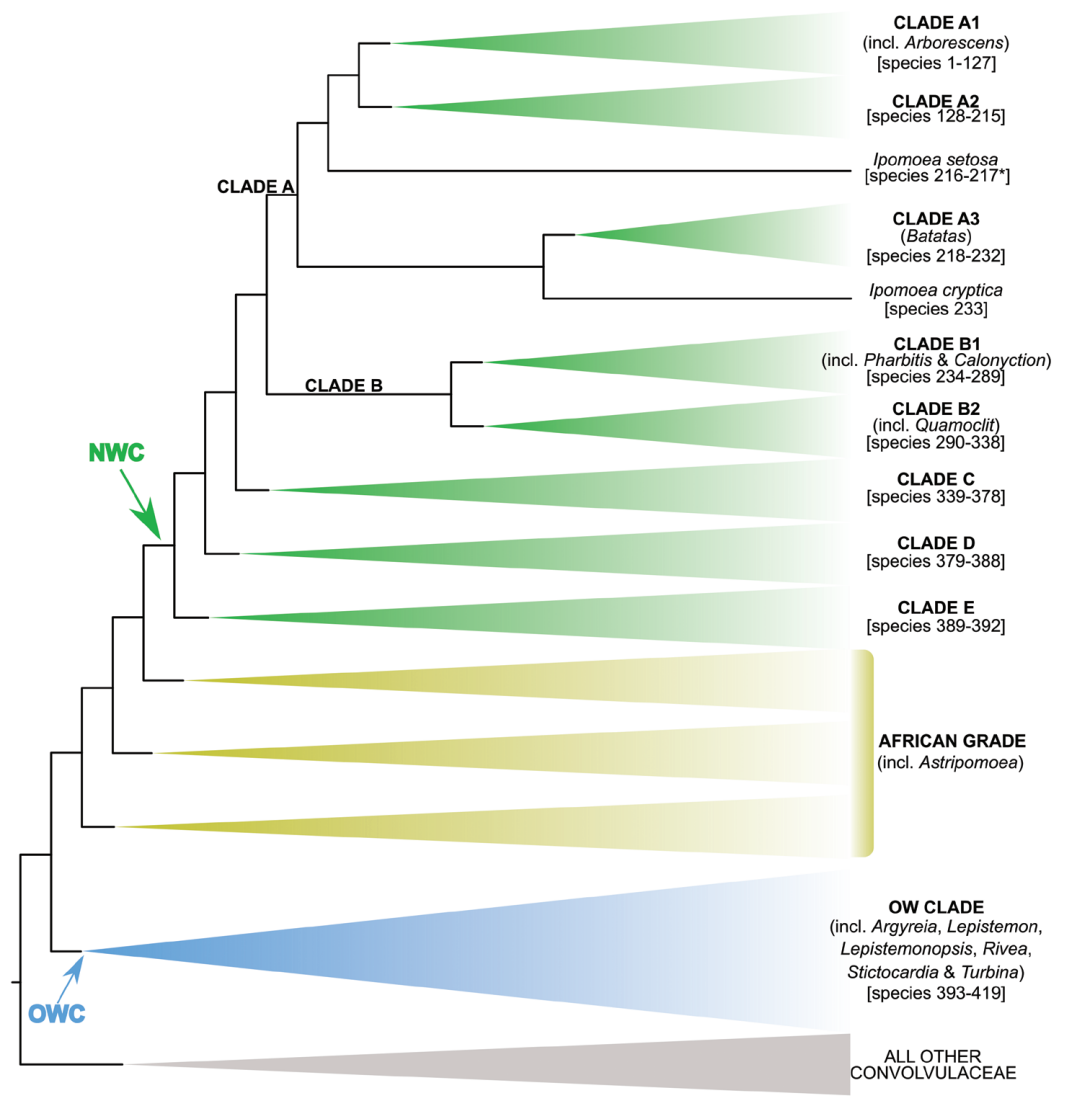
Cladogram showing the principal clades into which *Ipomoea* can be divided, the two main clades indicated as OWC (Old World Clade) and NWC (New World Clade). The placement of traditionally recognised genera and groups is shown in the corresponding clade.

Although there are some problems of species delimitation in *Ipomoea*, particularly in Clade A (Figure 1; Muñoz-Rodríguez et al. 2019), we have been able to provide descriptions of all accepted species in New World *Ipomoea*, identify types and provide outline details of distribution and ecology for nearly all taxa. We have described six further new species and provided a complete synonymy, keys, illustrations and notes to facilitate identification as well as to highlight infraspecific variation and areas of taxonomic uncertainty. To avoid unnecessary redundancy authorities are not provided for taxa mentioned in the introductory section and notes, as these are all provided in the taxonomic account. Exceptions are made for first occurrence of taxa which do not feature in the taxonomic account or in situations where authorities are needed to distinguish between different applications of the same name.

## History

### Generic delimitation

*Ipomoea* as constituted by Linnaeus was based on *Ipomoea pes-tigridis* L. and contained various elements, including *I. quamoclit* and *I. coccinea* (Quamoclit Clade, page 556), *I. triloba* and *I. lacunosa* (Batatas Clade, page 387), *I. violacea*, *I. alba* and *I. carolina* as well as species of *Merremia* Dennst. ex Endl. and *Jacquemontia* Choisy and even a species of Hydrophyllaceae, *I. nyctelea* L. (=*Ellisia nyctelea* (L.) L.). It was not clearly defined and several species since treated as belonging to *Ipomoea* were placed in *Convolvulus* L. by Linnaeus including *I. purpurea* and *I. pes-caprae*.

Jacquin, Vahl, Willdenow and others of Linnaeus’ successors in the later part of the 18^th^ century continued placing species of Convolvulaceae rather arbitrarily in either *Convolvulus* L. or *Ipomoea*. Only Cavanilles’ placements came close to coinciding with a modern concept of *I.omoea*. Some authors, like Desrousseaux (1792), maintained a wide concept of *Convolvulus* that included all species of *Ipomoea* and it was only in 1810 that a clear distinction between the two genera, based on stigma morphology, was established by Robert Brown (1810: 484). He contrasted the 2–3-lobed, capitate stigma of *Ipomoea* with the two filiform stigmas of *Convolvulus*. Brown recognized the ovary of *Ipomoea* as being 2–3 locular but made no attempt to subdivide the genus based on the number of ovary cells. Although Roemer and Schultes (1819) followed Brown’s classification, a wide circumscription of *Convolvulus* remained current for some time. Both Kunth (1819) and [Bibr B569][Bibr B570]) included *Ipomoea* within *Convolvulus* and it was not until the various publications of Choisy (1834, 1838, 1845) that *Ipomoea* was permanently separated from *Convolvulus*.

Choisy (1834, 1845) subdivided *Ipomoea* s.l. into several genera based on a series of ovary and fruit characters. He recognized a tribe Argyreieae Choisy comprising a heterogeneous group of genera including *Argyreia*, *Rivea*, *Legendrea* (=*Turbina*) and *Marcellia* on the basis of their having indehiscent fruits. The tribe Convolvuleae Choisy, in contrast, was characterized by having dehiscent fruiting capsules. In this second group, Choisy recognized *Quamoclit*, *Mina*, *Batatas*, *Pharbitis*, *Calonyction*, *Exogonium* and *Lepistemon* as distinct from but related to *Ipomoea* based on characters of the ovary and corolla. *Quamoclit* was recognized as distinct because of the 4-locular ovary, each cell with a single seed. *Mina* was separated from *Quamoclit* because of the suburceolate corolla shape. Together these two genera comprise what we recognize as the Quamoclit Clade (page 556). *Pharbitis* was separated on the basis of having a 3-lobed stigma and 3-locular ovary, each cell with two seeds, this genus constituting the Pharbitis Clade (page 430). Choisy’s *Batatas* was vaguely defined and is very heterogeneous comprising many extraneous elements besides *I. batatas* and *I. triloba*. *Calonyction* and *Exogonium* were separated from *Ipomoea* on the basis of their corolla, large, showy, white or pale lilac in the case of *Calonyction* but merely tubular in the case of *Exogonium*. A small clade of species of which *I. alba* is the best known more or less coincides with Choisy’s *Calonyction*, which was redefined and extended by Hallier (1897b). *Exogonium* was accepted by House (1908a) and other authors but is very heterogeneous and therefore polyphyletic, so bearing no clear relationship to the clades recognized in our molecular studies. *Lepistemon* was separated because of the large scales at the base of the stamens, a character that sometimes appears elsewhere in the genus, for example in some specimens of *I. batatoides*.

Choisy’s system continued in use until the 1890s when it was essentially reproduced in the account of Convolvulaceae in *Die Natürlichen Pflanzenfamilien* (Peter 1891). However, acceptance was never universal and Grisebach (1862b) reduced many of Choisy’s genera to sections of *Ipomoea*, a decision in which he was followed by Meisner (1869), Gray (1878, 1886) and others. Nevertheless, it was only in 1893 that a major generic reorganization was proposed by Hallier. The major innovation in Hallier’s (1893a, b) system was the use of pollen. He divided the Convolvulaceae into two pollen groups based on whether the pollen was smooth or spiny. The spiny pollen group, which included *Ipomoea*, was itself divided into two subgroups essentially on the basis of the fruit distinction proposed by Choisy for his tribes Argyreieae and Convolvuleae. The first subgroup (Echinoconieae subgroup Ipomoeeae) was composed of species with a dehiscent capsular fruit and comprised *Lepistemon*, *Calonyction* and *Quamoclit* as well as *Ipomoea* (in which Hallier included *Exogonium*, *Pharbitis*, *Marcellia* and *Legendrea*). The second subgroup (Echinoconieae subgroup Argyreieae) was characterized by its indehiscent fruit and comprised *Argyreia*, *Rivea*, *Ipomoea tiliifolia* and *Blinkworthia*. Two new genera in this second group were established: *Stictocardia* to accommodate *I. tiliifolia* and a few related species based on the prominent black leaf glands and strongly accrescent sepals and *Astrochlaena* (=*Astripomoea* Meeuse) to accommodate a group of mostly erect South African plants with stellate hairs and shortly oblong stigmas (Hallier 1893b: 159). For the first time *Merremia* Dennst. ex Endl. was clearly distinguished to accommodate species previously placed in *Ipomoea* but distinct because of their non-spiny pollen and generally white, cream or yellow flowers (Hallier 1893a). In fact, *Merremiasensu* Hallier represents a heterogeneous collection of species although dividing it up into natural genera is problematic (Simões et al. 2015, Simões and Staples 2017).

Hallier’s system has endured with only a few, relatively minor changes for about 125 years. A handful of new genera were established to include small, morphologically distinct splinter groups from the Ipomoeeae such as *Lepistemonopsis* with fleshy scales at the base of the filaments and *Pentacrostigma* with a 5-lobed stigma and 5-locular ovary. On the other hand the genera *Calonyction*, *Mina* and *Quamoclit*, all recognized by Hallier, were gradually abandoned; none was recognized by O’Donell in his various publications (O’Donell 1959a, b) although *Mina* is still occasionally accepted (Deroin 2001). Within the Argyreieae there has been uncertainty about the limits of various genera, notably *Rivea* and *Turbina*, the latter reincorporated in this subgroup and unique for its nearly pantropical distribution. A new genus, *Paralepistemon* was established to include two African species with thickened filament bases. (Lejoly and Lisowski 1986). In passing, it should be noted that a rather eccentric attempt to reclassify Convolvulaceae by Roberty (1952, 1964) has been universally rejected, like a similar earlier attempt by Rafinesque (1837, 1838a,b).

Recent phylogenetic studies point towards the acceptance of a broad concept of *Ipomoea* to include all Hallier’s Echinoconieae. Initial studies by Wilkin (1999) and confirmed by our own more extensive sampling (Muñoz-Rodríguez et al. 2019) have shown that even when smaller genera recognized within the tribe Ipomoeeae have strong phylogenetic support, they are nested within *Ipomoea*. Manos et al. (2001) showed that species with spiny pollen split into two major clades, a result confirmed by Muñoz-Rodríguez et al. (2019). One clade consists of species placed in *Stictocardia*, *Rivea* and *Argyreia* together with a superficially heterogeneous group of species from *Ipomoea* and *Turbina*, composed mainly (but not exclusively) of Old World species. The second clade consists of mostly (but not exclusively) New World species but includes *Astripomoea*, some species hitherto treated as *Turbina* and all Australian endemics we have sampled. Neither clade can be diagnosed by specific morphological features and it seems that there are multiple origins for many of the characters in *Ipomoea* including both the capsular and indehiscent fruit types as well as the different number of ovary cells, many characters thus being homoplastic. It is clear from these studies that *Ipomoea* as hitherto understood is not monophyletic and an expanded circumscription of *Ipomoea* is required to secure its monophyly (Stefanovic et al. 2003).

Our own extensive studies (Muñoz-Rodríguez et al. 2019) confirm earlier papers and support an Old World origin of *Ipomoea* s.l. They indicate that the recognition of a broad *Ipomoea* based on the presence of spiny pollen is the only logical solution that integrates monophyly and diagnosability. The alternative of dividing the whole clade into many small, formally recognized groups is not recommended due to high levels of homoplasy, lack of diagnostic characters and a complex tree model in which it is not obvious to which clades some species, not sampled for molecular data, should be placed. Consequently, we are adopting a wide concept of the genus to include *Argyreia*, *Astripomoea*, *Blinkworthia*, *Lepistemon*, *Lepistemonopsis*, *Rivea*, *Stictocardia* and *Turbina*, which are all nested within *Ipomoea*. However, as the genus is so large, we informally recognize certain diagnosable clades within *Ipomoea* to facilitate discussion and reflect the phylogenetic history of the genus. These informal clades include some traditionally recognized genera as well as newly discovered groups that contain similar looking plants and are geographically coherent. Where we have recognized informal taxa, we have been as explicit as possible about what species belong to those clades.

### Infrageneric classifications

An inevitable result of the situation described in the previous paragraphs is that all existing infrageneric classifications of *Ipomoea* are to a degree unnatural and many subgroups are neither monophyletic nor well-defined, something that goes far towards explaining the instability of all previous infrageneric classifications.

Choisy (1845) divided *Ipomoea* into groups based on habit but, while superficially practical, this is clearly artificial and so has only been occasionally and partially adopted by subsequent authors, such as Meisner (1869) and Matuda (1964, 1965, 1966a,b). Grisebach (1862b) reincorporated *Quamoclit*, *Calonyction* and *Pharbitis* into *Ipomoea* as sections and this treatment was followed by Bentham in Bentham and Hooker (1876), although these authors also incorporated other elements such as *Aniseia* Choisy within *Ipomoea*. Clarke (1883) followed Grisebach but treated the sections as subgenera. Hallier (1893b) began the introduction of a hierarchy of infrageneric taxa by recognizing subsections as well as sections and this process was continued by House (1908b), who multiplied the number of subsections. There was then a lull in attempts at an infrageneric classification of American species (O’Donell (1941 and *passim*) appears to have had no interest) until a major reformulation was made by Austin (1979, 1980), who recognized an even more extensive hierarchy with sections, subsections and series. Austin’s work culminated in a detailed and very complex hierarchical classification published 16 years later (Austin and Huáman 1996).

Apart from the repeated changes of status that these infrageneric taxa have undergone resulting in many groupings being re-graded from subgenus to section to series, the increasing multiplication of infrageneric taxa illustrates the difficulties of achieving a satisfactory classification. Apart from Quamoclit, Pharbitis, Calonyction, Old World Astripomoea and eventually Batatas (Austin 1975a, 1978b), none of these groupings are entirely natural, not even Stictocardia or Arborescens (McPherson 1981), both of which comprise a diagnostic and readily identifiable core of species but with a varying number of other heterogeneous elements. The essential instability of these classifications is well illustrated by the history of series Anisomeres. This was recognized by Austin and Huáman (1996) as a distinct series but was discarded a year later in a paper dissolving the Anisomeres series (Austin 1997). We believe that any attempt to provide a subgeneric classification following a traditional Linnaean model is bound to be artificial, impractical and doomed to failure (Carine and Scotland 2002).

### Species delimitation

The history of species recognition in *Ipomoea* is somewhat chequered. Good taxonomic decisions always require an awareness of previous publications as well as a good understanding of the relative value of different taxonomic characters. Access to a good range of specimens and images as well as field knowledge are also useful but only a few taxonomists have been able to benefit from these. Most have worked with limited material. The new species of early authors have not always stood the test of time (Wood 2017). However, amongst those publishing a significant number of new species of neotropical *Ipomoea*, it is clear that both Desrousseaux and Kunth demonstrated a high degree of competence, avoiding excessive duplication of names and so most of their species have endured.

The legacy of the most important 19^th^ century expert on *Ipomoea*, the Swiss botanist Choisy, is very mixed and he was criticised even during his own lifetime. He saw a wide range of specimens in many European herbaria and was well aware of previous publications, but neither his generic nor his species concepts have lasted well. He described the same species under different names often in different genera (Choisy 1845). *Ipomoea indica* was described under at least eight different names, mostly in *Pharbitis* and *I. batatas* under at least four names in *Batatas* but also in *Ipomoea*. Similarly *I. asarifolia* was described under three names in *Ipomoea*, *I. eriocalyx* twice, once in *Pharbitis* once in *Batatas*, *I. delphinioides* three times in *Ipomoea* and so on.

The next major work was the account of Convolvulaceae prepared by Meisner (1869) for the Flora Brasiliensis and this was virtually a monograph of the family in South America. It was as unsatisfactory as Choisy’s works but for different reasons. Meisner appears to have largely discounted the names of earlier authors including those of Choisy. He superfluously redescribed species as if earlier publications did not exist - *Ipomoea chrysotricha* Meisn. instead of *I. hirsutissima* Gardner, *I. obtusiloba* Meisn. and *I. heterotricha* Meisn. instead of *I. bonariensis* Hook., *I. riedelii* Meisn. instead of *I. batatoides* Choisy, *I. graminiformis* Meisn. instead of *I. schomburgkii* Choisy, *I. llaveana* Meisn. instead of *I. funis* Schltd. & Cham., *I. cardiosepala* Meisn. instead of *I. philomega* (Vell.) House) and many others. The other major problem with his approach was the recognition of a large number of varieties under each species, a process that tended to obscure the boundaries of recognized species. These varieties often recognize trivial variation but, in contrast, in other cases treat distinct, often very distinct, species as mere varieties of unrelated species.

The inadequate species level taxonomy of Choisy and Meisner was not an inevitable consequence of the epoch in which they worked. Near contemporaries such as Grisebach (1862a, b, 1879) or Gray (1878, 1886 were far more reliable. However, the excessive recognition of varieties continued to bedevil species level taxonomy into the 20^th^ century in the publications of Kuntze (1891, 1898), Hallier (1899c) and especially of Chodat and Hassler (1905) and Hassler (1911). Hassler took this interest in infraspecific taxa to an obsessive level, providing names for every slight variant found in Paraguay. However, the publications of the North Americans, especially House (1907 and passim), Brandegee (1889 and passim) and Robinson (1891 and passim) saw a welcome decline in the number of described infraspecific taxa associated with the species they described from Mexico. However the new century did not bring a marked improvement in species delimitation. Urban (1902–3 and passim) and Standley (1924 and *passim*) between them described over 50 new species of *Ipomoea*, of which only a quarter are recognized today (Wood 2017.

From the mid-20^th^ century onwards, the situation has improved, partly as the result of the outstanding achievement of the Argentinian botanist Carlos O’Donell (1941, 1948a, 1948b, 1950a, 1950b, 1950c, 1952, 1953a, 1953b, 1959a, 1959b, 1959c, 1960). He described 56 species of *Ipomoea* of which 45 are accepted today, a success rate far higher than that of any of his predecessors (Wood 2017). Almost everything he described from Argentina is still accepted. He did much to rationalize the chaos left by Hassler, sorted out the Quamoclit Clade throughout the Americas and added significantly to our knowledge of *Ipomoea* in Bolivia, Peru and Brazil. The vast majority of his taxonomic decisions stand today and his judgement in an era before the internet is remarkable.

After O’Donell’s premature death in 1954, there has been a slow but steady increment of new species from the Americas. Isolated species from different countries have been published by various authors but the contributions of Dan Austin and Andy McDonald are the most significant. Accounts of *Ipomoea* in Panama, Ecuador, Venezuela by Austin (1976, 1982a, 1982b) have provided a framework for the study of *Ipomoea* in these countries. McDonald (1987a, 1995, 2001) has revised several groups of *Ipomoea* principally from Mexico but has also contributed with Austin and Murguía-Sánchez to the account in the Flora Mesoamerica (Austin et al. 2012). Other important contributions have been made by Carranza (1998 and passim) on Mexican *Ipomoea* and Liogier (1955 and passim) on those of Cuba and Hispaniola.

### Discovery of *Ipomoea* species in America

Inevitably the first species of *Ipomoea* to be recognized and catalogued from the New World were species of economic importance (*I. batatas*), of horticultural value (*I. alba*, *I. indica*, *I. nil*, *I. purpurea*), or were widespread conspicuous species of accessible habitats, such as *I. pes-caprae*, or *I. violacea*, which grow on seashores. All were known to pre-Linnean botanists and featured in Species Plantarum and other near contemporary works.

Geographically, the first region from where a reasonably comprehensive inventory of *Ipomoea* emerged was the eastern United States. Nearly all the localized species from this area had been found and described by around 1800, including species like *I. pandurata*, *I. lacunosa* and *I. macrorhiza*. The United States Southwest had to wait until the 1850s after the United States-Mexican war. It was only then that species from this region were discovered, principally by Wright, Lindheimer and Torrey. Most were described a quarter of a century later by Gray (1878, 1886) including *I. leptophylla*, *I. tenuiloba*, *I. barbatisepala*, *I. lindheimeri* and *I. cardiophylla*. After the mid 19^th^ century there was little new to discover in the United States, and most (but not all) described novelties were ephemeral, being shown subsequently to be conspecific with earlier species.

The Caribbean had been one of the earliest regions of botanical exploration and many of its endemic species including *Ipomoea ternata*, *I. tenuifolia*, *I. repanda*, *I. digitata*, *I. clausa* and *I. desrousseauxii* were discovered in the 18^th^ century, as both Jamaica and Hispaniola were visited by botanists from different European countries. Cuba was somewhat different. Although Humboldt and Bonpland visited Cuba, they did not find any of its endemic species. A few were discovered by Sagra in the 1840s, but it was only in the 1860s that the rich diversity of *Ipomoea* in Cuba became known after Grisebach (1862a, 1866) wrote up the many new species found by Charles Wright (the same Wright who had been active in the United States Southwest). In the years after 1870 there was a slow increment of new species from the Caribbean culminating in the collections of Eric Ekman on Cuba (*I. baliocalda*, *I. erosa*) and on Hispaniola (*I. luteoviridis*), although many of his supposed novelties described by Urban (1924a and *passim*) have proved to be synonyms of other species. The occasional new species has been found since but the inventory of Caribbean *Ipomoea* is now largely complete.

There were collections from Mexico in the Spanish colonial era but those of Sessé and Moçiño were not published until a hundred years later. Nevertheless, seeds sent to Spain by them and by Née were cultivated in Madrid enabling Cavanilles to describe several attractive and interesting Mexican species including *Ipomoea tricolor*, *I. stans* and *I. bracteata* at the end of the 18^th^ century. The expedition of Humboldt and Bonpland constituted the next step forward in revealing the wealth of Mexican *Ipomoea*. *Ipomoea cholulensis*, *I. suffulta* and *I. hastigera* were amongst their discoveries, as was *I. arborescens*, the first tree *Ipomoea* to be described. During the first half of the 19^th^ century Mairet, Andrieux, Hartweg and others added to the list of species known from Mexico, but the most important advance came with the collections of Galeotti, which greatly increased the number of known species. His discoveries were published in 1845 and included many well-known Mexican species such as *I. lindenii*, *I. minutiflora*, *I. chenopodiifolia*, *I. pauciflora*, *I. suaveolens* and *I. proxima* (Martens and Galeotti 1845).

After 1845 there was a lull in the discovery of new Mexican species for almost fifty years. However, the end of the 19^th^ century proved to be a golden age for botanical exploration in Mexico thanks to a series of collectors mostly from the United States, especially Palmer, Pringle, Purpus, Nelson and Brandegee, and the Mexican-Italian Casiano Conzatti. The number of recognized species doubled during this era. However, these collections did not exhaust the riches of Mexican *Ipomoea* and the 20^th^ century has seen the regular discovery of new species by collectors from both Mexico and the United States, notably H.S. Gentry, G.B. Hinton and Rogers McVaugh from the United States and the Japanese-Mexican Eizi Matuda. This trend has continued into the new century with at least eight new species described since 2000. It is too early to say whether this trend is ending but it is perhaps significant that rather few new Mexican species has been found during the course of our studies in *Ipomoea*.

It was mostly during the 20^th^ century that the *Ipomoea* flora of Central America was discovered and described. Although not as rich as the Mexican flora, there has been a steady increment of new species since the middle of the century including *I. chiriquensis* from Panama, *I. magniflora* from Costa Rica, *I. riparum* from Honduras, *I. heterodoxa* from Belize and *I. steerei* from Yucatán, mostly found by North American collectors. However, the inventory of species seems to be nearly complete, since, as with the Caribbean, nothing new has been reported since the turn of the 21^st^ century.

The earliest collections from South America of any importance were made by Ruiz and Pavón in Peru at the end of the 18^th^ century. They noted surprisingly few new species of *Ipomoea* but amongst them were *I. ramosissima*. Of far greater importance was the expedition of Humboldt and Bonpland. Having found new species in Mexico they went on to find a series of new species in the northern Andes including *I. discolor* and *I. parasitica* in Venezuela, *I. capillacea* in Colombia and *I. abutiloides* in Ecuador.

Essentially little more was discovered or described from the Andean region for well over a century apart from a few species from Venezuela (Pittier 1927, 1931), a few from Peru by Weberbauer (Ooststroom 1933), two from Bolivia (Rusby 1896, 1899) and a couple from Argentina (Grisebach 1879, Kuntze 1891).

This situation only changed after the Second World War initially as a result of O’Donell’s short career (O’Donell 1941 and *passim*). He significantly increased our knowledge of species in the southern Andes, describing at least six new species from Argentina (*Ipomoea jujuyensis*, *I. rubriflora*, *I. lilloana*, *I. oranensis* etc.) as novelties, two from Bolivia (*I. tarijensis*, *I. suburceolata*), two from Peru (*I. velardei*, *I. peruviana*), three from Colombia (*I. colombiana*, *I. killipiana and I. reticulata*) and one from Venezuela (*I. pittieri*); these all still recognised. Fieldwork by Danish botanists led to the discovery and description of three new species from Ecuador by Dan Austin. The diversity in Bolivia was, however, only revealed recently (Wood et al. 2015, 2018, Wood and Scotland 2017b), with the description of 21 new species, mostly Andean which took the total number of *Ipomoea* species known from that country to 109, thus putting it in third place after Brazil and Mexico for the total number of *Ipomoea* species recorded.

The 19^th^ century, in contrast, was a golden age for plant discovery in Brazil, mostly under the stimulus of the production of Martius’ Flora Brasiliensis. The roll call of collectors finding new species of *Ipomoea* in Brazil is composed of most famous plant collectors in Brazil in the 19^th^ century. They include the Germans Martius, Riedel and Sellow, the Brazilians Vellozo and Silva Manso, Blanchet and Glaziou from France, Regnell from Sweden, Gardner, Spruce and Burchell from Britain and Pohl from Austria, their achievement commemorated in species such as *I. burchellii*, *I. regnellii*, *I. blanchetii*, *I. spruceana* and *I. pohlii*, all still recognized Brazilian species. The result was that by about 1870 our knowledge of *Ipomoea* was greater in Brazil than elsewhere in South America.

After the publication of Flora Brasiliensis (Meisner 1869), there was lull in the process of discovery in Brazil, which did not really pick up again for more than a hundred years. It has only been since the 1980s that significant numbers of new species have been found and described from Brazil (Austin 1981, [Bibr B558][Bibr B177], Vasconcelas et al. 2016, Wood et al. 2017a,d, Wood and Scotland 2017a,b). It is clear that Brazil is the richest country in South America for *Ipomoea* but it remains the least explored and it is the only country in the Americas from where we would expect significant numbers of new species to emerge.

As in other aspects of its history, Paraguay (and neighbouring parts of Argentina) has followed a somewhat different trajectory. Until the 1870s, the flora of this region was essentially unknown. Then came a publication by Parodi (1877) listing around 15 species of *Ipomoea* but in the absence of associated specimens, these names cannot mostly be linked to recognized names. Expeditions by Morong and Balansa began to reveal the diversity of *Ipomoea* in Paraguay, but it was a long-term Swiss resident, Emile Hassler, and Teodoro Rojas, the Paraguayan curator of his herbarium, who really discovered the Paraguayan flora and revealed the number of *Ipomoea* species in the country. Between them they added some 20 recognised species, all of which are endemic to the region, some extending into nearby parts of Argentina. Those species that were not recognized by Hassler himself were described subsequently by Carlos O’Donell (1948a, 1950b, 1953a), together with a number of species from Misiones and Corrientes provinces in neighbouring Argentina.

## Materials and methods

### Specimens

This monograph is based fundamentally on the study of herbarium specimens of *Ipomoea* informed by observations from morphology and molecular sequence data, fieldwork, photographs and information from literature and individual contacts throughout the Americas.

We have depended heavily on herbarium collections at Kew (K) and the Natural History Museum in London (BM), which together with material at Oxford (OXF) have formed the basis of our study. We have visited various European herbaria including Edinburgh (E), Leyden (L), Paris (P), Madrid (MA) and Stockholm (S) to view their collections of *Ipomoea*. During the course of visits to the United States we have seen material at (GH) and (A) at Harvard, the New York Botanical Garden (NY), the Smithsonian Institution in Washington (US), the Field Museum (F), Missouri Botanical Garden (MO) and Arizona University (ARIZ), including extensive material from Fairchild in Florida (FTG). Within Latin America, visits have been made to see herbarium collections in Cuba (HACB, HAJB), Mexico (IEB, MEXU), Ecuador (Q, QAP, QCA, QCNE, GUAY, LOJA), Peru (CIP, CUZ, USM), Bolivia (BOLV, HSB, LPB, USZ), Paraguay (FCQ, PY, SCP), Argentina (CTES, LIL) and Brazil (CEN, CPAP, HUEFS, IPA, JPB, MBM, PEUFR, R, RB, SP, UB). Help received from individuals in all these institutions is detailed in the acknowledgements at the end of this monograph. We have also received important loans of material from most of these institutions as well as from G and GOET. Photographs of herbarium specimens have also been a valuable source of information. The most important have been the images of types available through Jstor (www.jstor.org), but the websites of CRIA (splink.cria.org.br) and Reflora (reflora.jbrj.gov.br) and those associated with herbaria, including ARIZ (SEInet; swbiodiversity.org), B, BR, C, COL, E, F, MO, NY, P, PMA, US and W have all provided valuable information. We have also been sent images of important material from Geneva (G), Turin (TO), St Petersburg (LE), Vienna (W), Göttingen (GOET), Rancho Santa Ana (RSA), Montevideo (MVM) and Cambridge University (CGE). All cited acronyms are in accordance with the Index Herbariorum (http://sweetgum.nybg.org/science/ih/).

Much of the material we have been loaned has been type material or old or rare specimens and this has had important limitations on our ability to provide complete and accurate descriptions. In particular, details of the habit of many species is missing and can only be inferred. Flower colour has often been lost or modified during the drying process. It is often impossible, or at least undesirable, to dissect corollas, where only one or two are present pasted to the sheet and fragile in nature. Finally, it must be emphasized that the fruit of many species is unknown.

It is important to stress that herbarium specimens are not only a source of basic taxonomic information and an indispensable tool for species delimitation but also an essential resource for phylogenetic, ecological and other information. We have been able to use specimens for DNA sequencing, even from collections over a hundred years old, if they have been rapidly dried and retain their natural colouring. More recent, heat-dried specimens nearly always yield high-quality DNA, but there are striking exceptions, such as specimens of *Ipomoea chondrosepala*, which have mostly resisted repeated attempts to extract DNA. Specimen labels are another invaluable source of data. They can provide information that is not apparent from the specimen, such as flower colour, habit and size. Label information can also contain information about the general and specific habitat of the plant and can provide important facts about flowering patterns and ecology. We have used all available information of this kind to inform our descriptions and notes.

### Fieldwork

The first author has had many years of fieldwork during which he has collected *Ipomoea*. However, it is only since about 2008 that he has made careful efforts to collect, photograph and study the genus. Most of his fieldwork in South America has been carried out in Bolivia but important visits have been made to Argentina with the help of Hector Keller, to Brazil with the help of Luciano de Queiroz and to Paraguay with the help of Rosa Degen. This fieldwork has been very important in enhancing our understanding of the variation in species and in providing details of their habit and habitat. A consequence is that Bolivia is the only country from where we have near complete molecular sampling, a near complete collection of photographs of living plants and a good understanding of the phenology of different *Ipomoea* species. It is fortunate that there are 109 recorded species in Bolivia making it the third most species-rich country for *Ipomoea* in the Americas after Mexico and Brazil.

### Images

We have also benefitted from observations and in particular images sent to us by individuals over the years. We are particularly grateful to Maira Tatiana Martinez, Alfredo Fuentes, Alexander Parada, Julia Gutiérrez, Modesto Zarate and Daniel Soto (Bolivia), Moises Mendoza and Hibert Huaylla (Bolivia and Brazil), Hector Keller and Keith Ferguson (Argentina), Gilberto Morillo (Venezuela), Regis E. Bastian, Teresa Buril and Ray Harley (Brazil), Mario Giogetta (Bolivia and Argentina), Erin Tripp (Mexico), Jhon Infante Betancour (Colombia), Rémi Girault (French Guiana), Ramona Oviedo and José Luis Gómez (Cuba). We have also benefitted from images of living plants shown on a number of websites, especially Tropicos (tropicos.org) and SEInet.

### Literature

We have made full use of a wide range of literature as cited in the list of references. This includes regional, national and local floras and checklists (WCSP (1917), for example) as well as taxonomic works. We have consulted field guides and similar works when we have become aware of their existence. They often provide specific habitat and field identification information not readily available elsewhere. We have also made occasional use of information on the internet, but only if it seems reliable. We have scanned literature for examples of illustrations of species to supplement those prepared specifically for this project.

## DNA sequencing

Perhaps the most significant element in our methodology has been the integration of morphological and molecular data. During the course of the five years that we have been studying *Ipomoea* we have been able to sequence 1,560 specimens and approximately 450 species of *Ipomoea* from all over the world for *ITS* and two chloroplast markers (matK and trnH-psbA), 3,035 DNA barcode sequences in total (Muñoz-Rodríguez et al. 2019, supplementary data 3–8). A smaller number of 211 selected species were sequenced for 605 nuclear genes and the whole chloroplast genome (Muñoz-Rodríguez et al. 2019, supplementary data 3–8). Figure 1 summarises the results showing the main clades into which *Ipomoea* divides. We are particularly grateful to Kew and the Natural History Museum in London for allowing us to sequence large numbers of specimens in their collections and it is from these herbarium collections that the main bulk of our sequence data has been taken. We have had permission to sequence selected examples of species from other herbaria including E, L, MA and P and in the Americas from A, ARIZ, F, GH, HUEFS, IEB, LPB, MEXU, MO, NY, US. Obviously, field collections have provided additional samples for sequencing and we are grateful to several botanists for sending us samples including George Staples, Deng Yunfei (SCIB), Moises Mendoza (UB) and Barbara Kennedy (BISH).

### Species concept

Nowhere in biology is the disparity between theory and practice more evident than at the level of species. In an influential and widely cited contribution Kevin de Queiroz (2005, 2007) proposed the ‘unified species concept’ to treat existence of species as ‘separately evolving metapopulation lineages’ as the only necessary property of species and that the plethora of species concepts in existence merely represent different lines of evidence relevant to assessing lineage separation. In this way Queiroz (2005, 2007) separated the theoretical idea of what species are from the operational criteria of how to discover them. An important issue for the recognition of species is that as lineages diverge they can become distinguishable as separate species with diagnostic characters of fixed traits. Species can evolve distinctive ecologies and they can pass through polyphyletic, paraphyletic, and monophyletic stages in terms of their component genes. The problem is that these changes do not all necessarily occur, or if they do occur, do not do so at the same time and they do not even necessarily occur in a regular order (Queiroz et al. 1998). What this dynamic system of divergence means is that there is a certain pragmatic and heuristic nature to species delimitation whereby, although the expectation is that many species are clearly monophyletic, there will be other situations where the estimate of the degree of separation comes down to taxonomic judgement. Therefore, in this monograph we consider species to be *separately evolving metapopulation lineages* and in the discussion that follows we describe the *operational criteria* we used to infer, delimit and make taxonomic judgements about species boundaries. From experience gained studying *Ipomoea* we consider that species delimitation is usually relatively straightforward, given a representative sample of specimens and an understanding of the important diagnostic characters in the genus. Thus, a first task is to gain access to a wide range of specimens for comparison purposes, followed by a study of publications by reliable taxonomists who have worked on the genus. At the same time as specimens are studied morphologically, representative leaf samples from each putative species are sequenced for a few DNA barcode markers to provide an independent data source to corroborate or refute a species hypothesis based on morphological analysis or literature sources. Species delimitation is facilitated if DNA sequencing and specimen examination take place nearly simultaneously but this is only possible when DNA samples can be extracted from the available material. Conceptually as discussed above, we follow Queiroz (2005, 2007), whose framework includes the idea that disagreements about the limits of species are especially prevalent in those species at the active interface of speciation. This explains why many species have universally agreed boundaries whereas others are more difficult to interpret. We take the view that, in the context of taxonomy, for those instances where species delimitation is particularly problematic, it is best to flag up the variation, make a pragmatic, discursive and explicit taxonomic decision and move onto those other species delimitation problems that taxonomy can readily solve. This is especially true in large tropical groups such as *Ipomoea* in which many of the taxonomic problems can be readily solved by having a good sample of specimens combined with good knowledge of the group’s literature and nomenclature. Our view is that species and species delimitation can be viewed as a heuristic allowing an approach to problem solving or discovery that employs a practical method not guaranteed to be optimal or perfect, but sufficient for the immediate goals. To a greater extent than in *Convolvulus* (Wood et al. 2015), *Evolvulus L.* or even *Jacquemontia*, most species of *Ipomoea* are, as a generalization, well-defined. Hybridization is rarely reported, and that principally in the *Batatas* Clade. Claims by Austin that *I. leucantha* and *I. grandifolia* are of hybrid origin require corroboration. There are, of course, species complexes where delimitation is difficult, as intermediates occur between recognized species and these may be hybrids but a lot of the difficulty faced by the taxonomist arises from the lack of available material for study. Almost 20 species in the following account are only known from the type collection, another 50 or so are only known from less than five collections, and not all of these have been available for study. In about 20 cases, we have not seen authentic material and have relied on images and original descriptions to describe and delimit species. Inevitably, we are tentative in our decisions on the validity of some individual species. Examples include *I. leucantha* mentioned above as well as several species in Clade A1 (Figure 1; Muñoz-Rodríguez et al. 2019), such as *I. vivianae* or *I. pseudomalvaeoides*. A particular case is the pantropical *I. indica*. Molecular studies show that this is polyphyletic (Muñoz-Rodríguez et al. 2019, supplementary data 3), but it is clear that a more exhaustive study with extensive sampling is necessary before the components of this entity can be unravelled.

We have tried to make use of so-called conservative characters in accepting species and the value of these is discussed in the notes that follow. Pollination syndromes as reflected in corolla shape and to some extent in colouring and the structure of the androecium are seen as important for species delimitation (Rosas-Guerrero et al. 2011). Ideally each species will be delimited by a combination of distinctive characters. Species defined by a single character, unless it is exceptionally strong, are regarded with suspicion. We do take into account ecological and geographical factors. We would expect different species to have different ecological requirements or occupy a different geographical area from obviously related species. Where morphology, ecology and geography intergrade we have indicated by notes and in some cases by the recognition of subspecies. We have used insights from molecular sequence data, keeping separate apparently similar species like *I. marginisepala* and *I. cardiophylla* or *I. asarifolia* and *I. paludicola* where uniting them would render them non-monophyletic. We have been reassured in our species level decisions by molecular data in the recognition of numerous species, for example, *I. huayllae*, *I. graniticola*, *I. chiquitensis* and *I. juliagutierreziae* from Bolivia and *I. kraholandica* and *I. chapadensis* from Brazil. Molecular data has been less helpful for species delimitation within some of the relatively large clades such as Clades A, 1–2 (Figure 1) where multiple accessions of several species are not resolved partly due to the limited variation provided by regions used.

### Subspecies

The concept of a subspecies is retained for taxa which are morphologically distinct throughout most of their range but whose characters overlap in regions where the ranges of the two taxa meet. We accept that some species recognized in the following account could have been treated as subspecies of a closely related species, but in many cases, the number of specimens seen is so few that it would be premature to make this decision. Subspecific status is, therefore, reserved pragmatically for taxa of which we have seen many examples. Subspecies are keyed out when there are three more recognized subspecies for a particular species.

### Variety

Apart from subspecies, other infraspecific categories are not formally recognized in this account with the exception of two varieties. Most varieties, formas and subformas recognized by previous authors have little value and often do little more than recognize minor variations of corolla colour, indumentum or leaf shape. Some varieties, however, have long been recognized and, where these are historically significant or readily recognized, we have drawn attention to them in the notes that follow the species descriptions and made comments about their distinctive characters and distribution. We accept that some readers may wish to continue recognizing and using these varietal names. We understand varieties as morphologically distinct populations that occur sporadically over part or all of the range of a species. Although varieties may be restricted to a specific area they do not occupy a distinct geographical region with populations overlapping with those from another distinct geographical area. Sporadic occurrence is an important criterion in the recognition of varietal, rather than subspecific status.

## Structure of the monograph

In the following taxonomic account species are arranged in a linear order, reflecting phylogenetic relationships as far as is possible (Muñoz-Rodríguez et al. 2019). The 605 nuclear regions we sequenced provided an overall framework for the major clades we recognise but as the number of sequenced species was relatively small, this provided information on the placement of only about 140 species from the Americas into major clades. We have added to this analysis with information from chloroplast sequences and *ITS*, thus increasing the coverage to well over half the American species. Where there were differences between nuclear and chloroplast results we generally followed the nuclear data because of the larger data set and the support it gave for major clades. In cases of incongruence (*Ipomoea parasitica*, for example) we considered the size and increased support offered by the NGS nuclear data to be more reliable than *ITS* (Muñoz-Rodríguez et al. 2019). The order of species was then expanded based on obvious morphological similarities, thus all species in the Quamoclit Clade were grouped together as were all the inferred relatives of *I. malvaeoides*, even though we had not obtained sequences for some species in these groups. There remained a residue of species (± 40) whose placement was somewhat arbitrary as it was based on uncertain interpretation of morphological characters. Only five species are included at the end of the treatment as we were unable to suggest any likely placement.

The process described above was not always straightforward as the resolution of some parts of the phylogeny is poor, particularly in Clade A (Figure 1, Muñoz-Rodríguez et al. 2019). Nevertheless we are confident that the order of species presented in this monograph is a reasonable approximation reflecting the phylogenetic history of *Ipomoea* (Muñoz-Rodríguez et al. 2019).

The accepted names of species and subspecies are given in bold italics, followed by their author and place of publication. Where a recognized taxon is based on a nomenclatural combination, the basionym is given in plain italics immediately following the accepted names. This is followed in chronological order by any other names based on the same basionym. Heterotypic synonyms are then listed in chronological order of their basionym, each followed by subsequent combinations based on each basionym. Finally any commonly used name misapplied to the species is listed but only very common misapplications are cited. Authorities are not cited in the notes and other discussion sections for taxa that are treated in the monograph unless needed to clarify some typification or nomenclatural issue.

Types are cited for all listed taxa. The location of all types is indicated by the appropriate acronym following Index Herbariorum (http://sweetgum.nybg.org/science/ih/), the only exception being CIP (Centro Internacional de La Papa at Lima), whose herbarium is not included in Index Herbariorum but does contain some types. We have tried to indicate the holotype (or lectotype) in each case and we have seen all holotypes and lectotypes unless indicated with n.v. (not seen). We have not necessarily seen all isotypes but have listed herbaria where they are reported to be present. The list of isotypes may not be complete in every case and we have uncovered numerous isotypes during the course of our visits to different herbaria. Many more are likely to be found in herbaria we have not visited. We have designated lectotypes in many cases where no holotype existed or where it was ambiguous. It is hoped this will help achieve nomenclatural stability.

Descriptions all follow the same sequence and should be comparable although some details (fruits and seeds for example) are not always known. Subspecies are treated diagnostically following the main species description. With two exceptions varieties are not formally accepted and are included within the synonymy of individual species. However, those varieties we consider particularly significant are highlighted in bold in the notes that follow each species and we indicate what their distinctive characteristics are.

References are provided to illustrations after the descriptive text. These include all illustrations in the present work and selected illustrations from other publications. We have only selected illustrations from relatively recent publications with an emphasis on those from publications related to the Americas. However, we have included references to Bosser and Heine’s (2000) Flora of the Mascarenes and Deroin’s (2001) Flore de Madagascar 2001, although these works are not American, The two floras have illustrations of outstanding quality, showing most of the widespread species and including details which are not shown in other drawings. Systematic references to photographs have not been made. These are increasingly available on websites such as Tropicos, Reflora, SEInet and those of individual herbaria and on the websites of individual research workers.

Geographical information is provided country by country. Continental countries are ordered from south to north as follows: Eastern non-Andean, South America: Uruguay, Argentina, Paraguay, French Guiana, Surinam, Guyana; Western South America northwards, Chile north to Venezuela and then northwards from Panama to Canada. The islands are ordered from Bermuda to Bahamas, Turks and Caicos, Cuba, Cayman Islands and Jamaica, then from Haiti in an arc east and south to Trinidad, with the Netherlands Group at the end. Hawaii is placed in final position. Although apparently rather eccentric, this order ensures to a very large extent that plants whose range extends into adjacent countries or along mountain or island chains are arranged into logical distribution patterns.

Citations of occurrence are provided for all countries and, where possible, for major areas (states, provinces or departments), highlighted in bold face, in the larger countries. All South American countries except Uruguay and the Guianas are treated as “large countries”, together with Mexico, the United States and Canada. Major areas within larger countries are arranged alphabetically. The small Caribbean islands are treated as “major areas” of the Lesser Antilles. Citations are based on specimens seen or, in a few cases, identified by an established authority who is known to have understood the species well. Records from checklists and, especially data bases without images, have not been used as they contain many errors (Goodwin et al. 2015) and, if included, are indicated with the word *fide.* As a general rule at least one specimen is cited for every country and major area in larger countries. The purpose of the citations is to provide evidence of the presence of a species in a particular territory, not to provide a complete list of specimens seen, but in the case of rarer species, all specimens we have seen may be cited. If a user of this monograph wishes to confirm a record this can be traced through the cited herbarium. Photographs of many but not all cited specimens are available on line through the web sites of the relevant herbaria. Many individual records can also be traced through our project website “www.ipomoeaproject.org”.

Ecological information is included within distributional information. Our knowledge of the ecology of individual species varies from zero to good. It is particularly poor in cases of very localized species. Many of the widespread species occur as garden escapes, weeds or adventives in and around settlements and by roads. The only *Ipomoea* species reported to be invasive is *I. aquatica* and that only in Florida and Cuba. No troublesome weed of cultivation has been noted.

Explanations for lectotypifications are provided separately from other notes. In cases where no explanation is provided, it should be assumed that the most complete specimen seen and cited by the original author was chosen.

Notes are mostly related to taxonomic issues. They often summarise distinctive characteristics of a species and indicate how it can be distinguished from other species with which it is often confused. Some information has been given about traditional and economic uses but this has not been a focus of attention in this monograph.

## Results

### Molecular sequence data

Results from molecular sequencing and phylogenetic analysis have been of great value in our research at many levels (Wood et al. 2015; 2017a, b, d; 2018) and have enabled us to delimit *Ipomoea* as a genus, facilitating the study of its evolution (Muñoz-Rodríguez et al. 2018, 2019). It has enabled us to recognize major radiations in South America within Clade A1 (Figure 1) and in the Caribbean within Clade A2. It has confirmed the monophyly of some groups previously recognized on morphological grounds, such as *Calonyction*, *Quamoclit*, *Astripomoea* and *Batatas* (Muñoz-Rodríguez et al. 2019, supplementary data 3–8). Other accepted groupings, such as *Argyreia*, *Pharbitis*, *Stictocardia* and the *Arborescens* group are shown only to be monophyletic if certain species are excluded. Conversely, it has demonstrated that some recognized groups are not monophyletic (*Turbina* for example) and that *Rivea* is nested within *Argyreia*. Importantly the phylogenetic framework we have developed provides a context in which to interpret and understand the evolution of the many species of *Ipomoea* that lay outside the previously recognized segregate genera.

DNA sequencing and phylogenetic analysis has been valuable at the species level too. It has confirmed the monophyly of many species and has also drawn attention to the existence of unrecognized new species. We have many examples of this, such as the “discovery” of *Ipomoea kraholandica* in Brazil or *I. lactifera* in Bolivia, this last especially interesting as DNA confirmed it as belonging to the Batatas Clade and sister to the Old World species, *Ipomoea littoralis*. Sequence data has shown some species thought to be distinct are conspecific with others from different geographical areas, for example, *I. acanthocarpa* from Africa is the same species as *I. piurensis* from America, while *I. lindenii* from mainland America is the same as the Jamaican endemic *I. cyanantha*. In both these examples multiple specimens of the supposedly distinct species form a largely unresolved single clade confirming our morphological observations that the species are indistinguishable. DNA has also demonstrated that some species pairs thought to be possibly conspecific are indeed different; *I. paludicola* is distinct from *I. asarifolia*, *I. marginisepala* from *I. cardiophylla* and *I. pterocaulis* from *I. jalapa*. In these examples specimens from the two species do not form a clade separate from all other species. DNA has also shown that *Ipomoea indica* is not monophyletic and so consists of more than one entity, although we have not yet been able to unravel this complex. It has highlighted misidentifications when wrongly named specimens appear in parts of the tree separate from the clade where they belong. It has also provided a phylogenetic context to enable the interpretation of incomplete specimens, which lack diagnostic morphological information. However, molecular sequencing using *ITS* has severe limitations which are well documented, not least lack of resolution and support. For this reason, we have always used our *ITS* phylogeny in conjunction with hypotheses based on morphological characters. Nevertheless, we have been reassured that all major clades identified in our *ITS* tree are also inferred from the analysis of single-copy nuclear regions and of whole chloroplast genomes (Muñoz-Rodríguez et al. 2019).

### Major clades of *Ipomoea*

Figure 1 summarises the phylogeny of *Ipomoea* and shows the genus is divided into two clades of similar size. These are labelled for ease of reference but are not formally recognized. The two clades are dominated by species from the Old World and New World respectively but with many exceptions. The Old World Clade (OWC) consists of species previously placed in *Argyreia*, *Rivea*, *Stictocardia* and *Lepistemon* as well as many always included in *Ipomoea*. The New World Clade (NWC) consists of an early diverging grade of Old World elements and a species-rich clade dominated by species from the Americas, but which also includes all species endemic to Australia. The existence of this fundamental split within *Ipomoea* had been posited by previous research ([Bibr B411][Bibr B368]) although based on much poorer taxon sampling.

Both NWC and OWC contain elements that were recognized previously as genera and appear as smaller clades within NWC or OWC. The only previously recognized genus that is represented by native species in both NWC and OWC is *Turbina*, although most of its species are in OWC. *Turbina* is polyphyletic containing several heterogeneous elements and is consequently rejected. Similarly, we reject the New World genus *Exogonium* as it was founded on a hypocrateriform corolla adapted for bird pollination and this character is homoplastic occurring in various different clades within NWC. In NWC, species formerly grouped under the names *Arborescens*, *Batatas*, *Pharbitis*, *Calonyction* and *Quamoclit* all form small clades which more or less coincide with their traditional circumscription and so are used by us as names for the corresponding clades. In OWC the generic names *Argyreia*, *Astripomoea*, *Stictocardia* and *Lepistemon* form clades of varying sizes and we continue to use these names for these distinct clades. Unlike the New World clades recognized above, these Old World clades vary considerably in size, *Argyreia* having around 125 species (including *Rivea*), *Stictocardia* around ten and *Lepistemon* only two so barely meriting recognition. All these nine clades which are assigned traditional names are more or less diagnosable using combinations of morphological characters.

NWC comprises about 450 species. Apart from a few species previously placed in *Turbina*, all species belong to *Echinoconieae* subgroup *Ipomoeeae* in Hallier’s (1893a, b) classification. Most (All?) Australian endemics belong to this clade and it is also well represented in Africa. Within NWC, two very large clades are recognizable. One clade (mostly South American but including *Batatas*) roughly coincides with subgenus Eriospermum (Hallier f.) Verdc. ex D.F. Austin as defined by Austin and Huáman (1996), although Austin included many elements which do not belong, such as *Ipomoea rubens*, *I. lindenii*, *I. violacea*, *I. imperati*, *I. magnifolia*, *I. habeliana* etc. (Muñoz-Rodríguez et al. 2019). We refer to this as Clade A. There is a second very large, mainly Mexican, clade that has not previously been recognized which includes Austin’s subgenera *Ipomoea* and *Quamoclit* as well as some other species. We refer to this as Clade B. Clades C, D, E and F represent smaller clades within NWC, this last essentially African with one New World endemic (*I. habeliana*).

Our studies have revealed many smaller clades to which a traditional name cannot be readily attached. The two largest are both in Clade A of NWC and we refer to these as Clades A1 and A2. Some of the species in Clade A1 were treated as series *Jalapa* by Austin and Huáman, but in a very inconsistent way. It is found throughout the neotropics but is most diverse in South America. The *Arborescens* group form a small clade within A1. Clade A2 is also found throughout the neotropics but is particularly important in the Caribbean, as nearly all the 25 endemic species of that region belong to it. Elements of this clade were referred to as *Microsticta* by McPherson (1980) and as series *Eriospermum* by Austin and Huáman (1996). Both Clade A1 and Clade A2 are usually recognizable morphologically, the former by its pubescent corolla and rather soft, flattish sepals and the latter by its usually glabrous corolla and coriaceous, often convex, ovate to elliptic sepals. We note that there are a few exceptions in both these clades and that several of these diagnostic characters are homoplastic in other parts of *Ipomoea*. Clade A3 is a small clade comprising the Batatas group. *Ipomoea cryptica* is sister to this clade in the nuclear phylogeny but not in the chloroplast phylogeny.

Apart from *Pharbitis*, *Calonyction* and *Quamoclit*, there are several small clades which are more or less diagnosable morphologically within Clade B. There is a small clade (Species 328–334) of seven species centred on *Ipomoea costellata* assigned the name *Pedatisecta* by House (1908b) characterized by digitately divided leaves. These were treated as part of Sect. Leptocallis by McDonald (1995) but the name *Leptocallis* has to refer to a quite different small clade (Species 280–288) centred on *I. capillacea*, perhaps characterized by tuberous storage roots. The most distinct small clade in Clade B consists of five species characterized by pinnatifid leaves and centred on *I. stans* (Species 275–279). These were included in Sect Tyrianthinae by McDonald (2001) but this name cannot be used for this clade as the type, *I. orizabensis*, belongs to a different clade. Since the six small clades discussed here account for only a small proportion of the species in Clade B, we have avoided any formal recognition of these names.

Clade C also contains a number of small clades which are more or less diagnosable morphologically or geographically, although the best known species, *Ipomoea pes-caprae*, belongs to a clade of Australian species. A small clade of four species (Species 345–348) centred on *I. asarifolia* can be recognized by their very unequal, transversely muricate sepals. Another small clade of South American species consisting of perhaps eight species centred on *I. maurandioides* (Species 356–363) that can be recognized by their glabrous indumentum, unequal sepals and often trailing habit.

The Old World Clade (OWC) contains around 350 species mostly from the palaeotropics. It includes most species treated as *Echinoconieae* subgroup *Argyreieae* by Hallier including all species placed in *Argyreia*, *Rivea* (which is nested within *Argyreia*), *Stictocardia* and some species placed in *Turbina*. Only a few relatively small clades are composed of neotropical species. Much the largest is the clade of around 12 species centred on *I. corymbosa* but with morphologically very disparate elements, including *I. ochracea*, *I. regnellii*, *I. crinicalyx*, *I. cuscoenesis* and *I. daturiflora*.

There are important practical implications from our molecular results. Since there is no obvious or close correlation between morphological characters and the *Ipomoea* phylogeny, it is currently impossible to propose an infrageneric classification along traditional lines. Although most clades cannot be defined morphologically, they do have certain morphological tendencies, which we have highlighted and discussed in the notes that precede the description of the species in each clade. As noted above, some of the smaller clades are well-defined and, where this is the case, their distinctive morphological features are indicated. We have also tentatively used molecular results to inform the placement of individual species within clades.

It should be stressed that we have faced a problem that we share with previous botanists working on the classification of *Ipomoea*. Some species are not available for study or sequencing and so cannot be assigned unequivocally to a clade. In this situation, we have inferred the position of species from their morphology. Most placements will be uncontroversial but in a few cases they are little more than guesses. The notes following each species indicate where placement is particularly uncertain.

### Geographical distribution

*Ipomoea* is a tropical genus and this is reflected in its distribution in the Americas with few species found north or south of 30 degrees latitude. The main exception lies in the Eastern United States where several species, *I. coccinea*, *I. lacunosa*, *I.sagittata* and *I. pandurata*, extend north to at least 35 degrees, *I. repanda* as far as 43°N in Ontario, Canada. The complete absence of *Ipomoea* from California in the west apart from a few introduced ornamentals (as well as in central Chile) suggests that it cannot tolerate a Mediterranean climate with arid summers and cool wet winters.

Within the neotropics *Ipomoea* is widely distributed but is noticeably less diverse in the equatorial region with relatively few species in Amazonia, Ecuador (Austin 1982a) or Colombia (Bernal et al. 2015). Although a partial explanation lies in the low diversity of *Ipomoea* species generally in rain forest, it does not account for the lack of species diversity in the dry forests of the Caribbean coasts or of the inter-Andean valleys such as the Colombian Magdalena. Species diversity rises as one moves away from the Equator and the countries with the greatest diversity of species lie mostly within the 15 to 30 degrees of latitude, notably Mexico and Brazil, both large countries with extensive subtropical dry forest. Some smaller countries in these latitudes, such as Paraguay (Wood et al. 2017c), Bolivia (Wood et al. 2015) or Cuba (Wood and Scotland 2017c) are proportionately as rich.

Most species of *Ipomoea* are relatively localized in their distribution often being found in a single region or country. However, there is a large set of species (*I. alba*, *I. batatas*, *I. cairica*, I. carnea subsp. fistulosa, *I. corymbosa*, *I. hederifolia*, *I. indica*, *I. muricata*, *I. nil*, *I. purpurea*, *I. quamoclit* and *I. tricolor*) that occur around cultivation or in disturbed places near settlements throughout the tropics and are found in almost every country of the Americas with a tropical climate. To this group should be added some other pantropical species that are also widespread but absent from many countries including *I. acanthocarpa*, *I. aquatica*, *I. asarifolia*, *I. fimbriosepala*, *I. mauritiana*, *I. setifera* and *I. triloba*. All these pantropical species occur sporadically, occasionally abundantly, in different neotropical countries but there is little geographical patterning to their distribution. A similar pattern can be observed in the palaeotropics. Of the 26 species recorded for the Flora of the Mascarenes (Bosser and Heine 2000 all but one also occur in the Americas. Equally all but two species recorded from Hawaii are also present on the American continent.

Of species never found in the Old World, *Ipomoea aristolochiifolia* is probably the most widespread, being found from Argentina north to Mexico, although it is absent from the Caribbean islands. Other very widespread species include *I. philomega*, *I. batatoides*, *I. ramosissima* and *I. regnellii* but, apart from *I. ramosissima*, none extends into Argentina and all peter out as they enter Mexico. Two species, *I. dumetorum* and *I. clavata* extend along the Andean Chain from Argentina or Bolivia north to Mexico but are absent elsewhere. More frequent are species that extend from the United States or Mexico southwards to northern South America. These include *I. capillacea*, *I. cholulensis* and *I. lindenii* that are restricted to the mountain chains and *I. minutiflora*, *I. meyeri*, *I. trifida* and *I. tiliacea* which are common in the Caribbean (except *I. minutiflora*) and Central America extending into northern South America, in the case of *I. tiliacea* south along the eastern edge of Brazil almost to Uruguay. Of some interest are two upland species, *I. plummerae* and *I. pubescens*, common around the 20–30° latitude in both hemispheres but largely absent from intermediate equatorial regions.

*Ipomoea plummerae* and *I. pubescens* are not the only species with disjunct distributions. *Ipomoea crinicalyx* and *I. amnicola* are also amphitropical in distribution but there is suspicion that the latter has been introduced into the northern hemisphere. Several annual species like *I. parasitica*, *I. heptaphylla*, *I. longeramosa* and *I. neurocephala* are very scattered in their distribution, being known from many countries but, with the exception of *I. longeramosa* in NE Brazil, from only one or few collections in each case. The occurrence of the South American *I. subrevoluta* on the Isla de Juventud (Pinos) in Cuba and also on Trinidad is remarkable but it perhaps arrived as a result of the movement of migratory water birds. *Ipomoea thurberi* also has a curious distribution with isolated populations in Guatemala and Nicaragua which are disjunct from each other as well as from the main population in northern Mexico and Arizona. In South America remarkable disjunctions are noted for species found on isolated granite domes around the Amazon. *Ipomoea chiquitensis* and *I. graniticola* are known from a few locations separated by many thousands of kilometres (Wood et al. 2017c). The apparent disjunctions in the distribution of two species found in the Amazon basin, *I. amazonica* and *I. velutinifolia* can be explained by inadequate collecting in areas separating known locations. The most inexplicable disjunction, however, is that of *I. eremnobrocha* known from the Cerro Campana in Panama and from a number of locations in NE Brazil.

### Endemism

Throughout the Americas many species are endemic to single countries with a good number of species endemic to single localities or to a very restricted area. Clearly the two largest countries, Brazil and Mexico, each with about 60 endemic species, have the greatest numbers of single country endemics. Scattered endemic species are found in most Andean countries with much the greatest numbers in Bolivia (c. 20) but the arbitrary nature of political boundaries tends to reduce the gross figures for individual countries. There are few species endemic to the small Central American republics although four are endemic to the Panama-Guatemala region. The large Caribbean islands are also major centres of endemism. We recognize 17 species as endemic to Cuba, seven to Hispaniola and four to Jamaica. Additionally there are a number of near endemics on these islands. In contrast, species endemic to small islands or island groups are few and we recognize only four, *Ipomoea sphenophylla* on St Eustatius, *I. steudelii* on Puerto Rico, *I. tuboides* on Hawaii and *I. habelana* on the Galapagos, the last two on several islands in their respective archipelagos and, perhaps coincidentally, both adapted for moth pollination.

It is harder to discern concentrations of endemic species in particular regions of the large continental countries, particularly in Mexico, where endemic species occur in scattered locations over much of the country. However, there is evidence that the greatest concentrations of endemics are in the seasonally arid regions of South West Mexico (McDonald 1991), with a lesser centre in the central northern plateau. Much the same is true for South America but the Chapada de Veadeiros (Brazil) is home to at least four endemic species and the Sierra de Amambay (Paraguay) to at least three. Both these locations are also home to several other very rare species which extend only to a few nearby locations. Another very rich area comprises the lower eastern slopes of the Andes near the border of Argentina and Bolivia. This is exceptionally diverse in terms of local endemic species with at least nine species endemic to the area.

It is equally difficult to discern clear examples of endemism in particular biomes except for some extreme examples such as seashores. Clearly there are many species endemic to Seasonally Dry Forest and to Cerrado but as the former includes many distinct variants and the latter very different physiognomies from campo limpo to cerradão, the notion of endemism is not very easy to apply except in a very loose sense. Specific examples of habitat preferences are indicated after species descriptions, where these are reliably known.

### Ecology

Precise information about the ecology of many species is unavailable so it is difficult to provide anything approaching a comprehensive account of the habitat requirements of many neotropical species. Certainly, *Ipomoea* species grow in many different habitats and it is clear that most habitats host species specific to that habitat.

The most typical beach species are *Ipomoea pes-caprae*, *I. imperati* and *I. littoralis* (in Hawaii) but others occur on coastal sands including *I. tiliifolia* and some forms of *I. batatas*. There is some evidence that the fruits of some of these species can survive for long periods in salt water (Miryeganeh et al. 2014) and it has been suggested that in the case of *I. pes-caprae* the persistent pedicel actually aids seed dispersal. The world distribution of these species and that of *I. violacea*, which often grows in mangrove swamp, strongly suggests that their dispersal is mediated through ocean currents. This may be the explanation of how the salt marsh species, *I. sagittata* made it to Europe in prehistoric times. Ocean currents may also partially explain the distribution of *Ipomoea indica* and *I. triloba* as both show a predilection for islands, although the former is also readily spread by broken shoots as a result of trampling by cattle. There is also an interesting group composed of species that are not strictly maritime but are often found in the proximity of the coast, although all occur, sometimes abundantly inland; these include *Ipomoea tiliacea*, *I. mauritiana*, *I. digitata*, *I. asarifolia*, *I. macrorhiza and I. jalapa*.

Some species are characteristic of freshwater habitats and are often specialized in their requirements. The only true aquatic is the introduced *Ipomoea aquatica*, which roots on mud and sometimes has extensive floating stems. *Ipomoea subrevoluta* usually grows by small streams in grassy plain whereas *I. rubens* is more typical of the borders of larger rivers or small lakes. *Ipomoea paludicola*, *I. schomburgkii* and *I. pittieri* favour flooded pampa whereas *I. paludosa* is characteristic of swampy hollows in the cerrados. *Ipomoea fimbriosepala*, *I. setifera* and *I. neei* are often found near water. The widespread species *I. alba* appears to favour disturbed scrubby gullies which are permanently or seasonally moist, when it grows as an apparently native species. The natural distribution of I. carnea subsp. fistulosa is obscured by its presence as an escape from cultivation but it appears native in swamp in the Parana basin of South America and perhaps elsewhere.

The lack of diversity of *Ipomoea* in rain forest does not mean that there are no characteristic species in this habitat. The best indicator of rainforest in the genus is *I. philomega*, which is found in evergreen forest at low altitudes throughout the Americas. Other typical species that are more local in their distribution include *I. amazonica*, *I. velutinfolia*, *I. santillanii*, *I. splendor-sylvae* whereas *I. aurantiaca*, *I. chondrosepala*, *I. regnellii*, *I. squamosa*, *I. batatoides* and *I. reticulata* also occur in rainforest but are not restricted to this habitat. The near absence of several otherwise widespread species from the Amazon basin is also interesting. *Ipomoea hederifolia* and I. carnea subsp. fistulosa are almost completely absent from Amazonia.

Cloud forest is another wet forest habitat where *Ipomoea* is relatively poorly represented. Cloud forest occurs from slightly below 1000 m to at least 2500 m along the Andes from Bolivia northwards, in the Brazilian Atlantic forest and in Central America. Probably the most widespread cloud forest species is *I. lindenii*, which grows from Bolivia to southern Mexico with an outlying station in Jamaica. Other cloud forest species are much more local but include *I. austrobrasiliensis* from the Brazilian Atlantic Forest, *I. magnifolia*, *I. inaccessa* and *I. odontophylla* from the Bolivian Andes, *I. retropilosa* from Colombia and Venezuela, *I. chiriquensis*, *I. isthmica* from Panama and Costa Rica and *I. chenopodiifolia* from Guatemala and Mexico. Other species may occur in coffee plantations, which are often created from areas of former cloud forest including the widespread *I. aristolochiifolia*.

High altitude species are even rarer and very few species occur above about 2500 m. The only species that might occur in paramo is *Ipomoea capillacea* while, in puna or at least subpuna, the only species recorded are *I. plummerae* and *I. pubescens*. Both have a disjunct amphitropical distribution occurring in Mexico and the United States Southwest as well as South America. *Ipomoea plummerae* reaches 4000 m in Bolivia.

*Ipomoea* species are tolerant of drought and several are recorded from desert. In South America *I. incarnata* is the best adapted to arid conditions occurring in the coastal deserts of Peru and the Colombian Guajira as well as the Caatinga of NE Brazil. Other indicators of very arid conditions in South America are *I. nationis* from Peru, *I. verruculosa* from Venezuela and *I. sericosepala* from Brazil and Bolivia. In North America, Felger et al. (2012) record some 13 species from the Sonora desert region of Mexico-Arizona, listing *I. cardiophylla*, *I. costellata*, *I. cristulata* and *I. ternifolia* as typical of this habitat. Most tree species from the *Arborescens* clade in both South and North America favour arid habitats but are more typical of dry deciduous forest than true desert.

Of some interest are morphological adaptations found in several species growing in dry habitats. One such occurs in the coastal lomas of Peru and the northern Atacama of Chile. Here forms of *Ipomoea dumetorum*, *I. nil* and *I. purpurea* occur with short, erect stems, very unlike the normal long twining stems found in other habitats. The Galapagos Islands comprise another arid habitat where there occur extreme forms of *I. muricata* and *I. incarnata*, once treated as distinct species under the names respectively of *I. tubiflora* and *I. linearifolia*. In the former the fleshy teeth of the stems are largely suppressed while the latter presents with very narrow leaves. In the Sonora Desert in Mexico, forms of *I. cristuluta* occur with erect, woody virgate stems, a facies very different from the normal herbaceous, twining stems. Perhaps the most remarkable is the dwarf form of the usually lowland *I. platensis* which grows in arid situations at over 2000 m in the Argentinian Andes. (Figure 83).

Desert merges into dry grassland, particularly in North America. Erect and, less commonly, trailing species of *Ipomoea* are characteristic of grassland habitats. There are relatively few examples from North America, *I. leptophylla* being the only widespread prairie species but several other North American species are clearly adapted to the grassland habitat, including *I. longifolia* and the Mexican endemic *I. durangensis*. However, it is in the South American cerrados that a great number of grassland species have evolved. Erect species occur in different clades and include *I. hirsutissima*, *I. malvaeoides* and *I. cuneifolia* and several others from Clade A1, *I. argentea* and *I. paulistana* from Clade A2 and *I. squamisepala* and *I. pinifolia* from Clade C. Trailing species are also common including *I. descolei*, *I. psammophila* and *I. langsdorfii*, *I. burchellii*, *I. goyazensis* and *I. procumbens*.

Thorn scrub merging into seasonally dry forest is another important semi-arid habitat, which is common throughout much of tropical America. *Ipomoea* is at its most diverse in this habitat. In South America the relatively widespread species *I. amnicola*, *I. megapotamica*, *I. incarnata* and *I. abutiloides* are good indicators of this habitat. However, each of these dry forest regions has its own set of localized species, *I. argentinica*, *I. oranensis* and *I. schulziana* where the chaco meets the Andes, *I. brasiliana*, *I. longibracteolata*, *I. marcellia* and others in NE Brazil. *Ipomoea verruculosa* in the dry coastal woodland of Venezuela, *I. pauciflora* and *I. velardei* in Ecuador and Peru. Dry forest species are also noted from the Caribbean Islands, *I. carolina* from Cuba, for example, but it is in Mexico and Central America that very large numbers are recorded as growing in dry forest, usually pine or oak woodland, either wholly deciduous or partially so. All the tree species (from both North and South America), lianas like *I. bombycina* and numerous other species are recorded from this habitat. The roll call of dry forest species from Mexico is long and includes such relatively common species as *I. orizabensis*, *I. pedicellaris*, *I. praecana*, *I.seducta*, *I. lobata* and many others.

*Ipomoea* species tend to avoid closed forest but occur along streams, by tracks and roads and often favour rock outcrops where the forest cover is broken. Species diversity is greatest in deciduous forest, possibly because there is more plentiful light during the dry season (McDonald 1991). This could be an explanation for why some dry forest species flower in the dry season at a time when they are leafless. This is a particular feature of the tree species in general, some Mexican species such as *I. tehuantepecensis*, *I. pseudoracemosa*, *I. concolor* and *I. pruinosa*, but of relatively few South American species wth the exception of *I. juliagutierreziae* and *I. schulziana*.

Rocks provide a specialized habitat for some species. In Mexico, cliffs or “crags” are often cited as the habitat for *Ipomoea rupicola*, *I. chilopsidis*, *I. teotitlanica*, *I. seeania* and *I. concolor* whereas in South America the only species cited from a similar habitat is *I. killipiana*. The geological composition of the cliffs is not usually recorded but volcanic rocks are mentioned for *I. seeania* and limestone for *I. teotitlanica*. Limestone, however, is often cited for plants from the Caribbean including *I. montecristina*, *I. praecox* and *I. fuchsioides* from Cuba, the last two characteristic of limestone towers locally known as mogotes. It is also cited for several species from Hispaniola including *I. digitata* and *I. desrousseauxii*. *Ipomoea luteoviridis* is recorded from serpentine outcrops in Hispaniola but we are unaware of any other American species with this habitat preference. A few species are noted from lava flows, notably *I. tuboides* from Hawaii, but several Mexican species are recorded on pedregales including *I. orizabensis* and *I. dumetorum*. In South America the most commonly recorded specialized rock habitat consists of granite domes and platforms, which outcrop sporadically in dry forest and cerrados on the pre-Cambrian shield. The commonest species of this habitat are *I. bonariensis* and *I. maurandioides*, but neither is restricted to granite. More restricted geographically and geologically and often very disjunct in their distribution are *I. caloneura*, *I. chiquitensis* and *I. graniticola*, the last being found in isolated locations in Bolivia, Brazil and Paraguay. *Ipomoea leprieurii* is locally frequent on granite outcrops in French Guiana and neighbouring parts of Brazil while *I. marabaensis*, *I. scopulina* and *I. fasciculata* are currently known only as pin-point endemics.

*Ipomoea* species are also frequent in secondary scrub and in disturbed places around settlements. This is the kind of habitat where the widespread pantropical species are often found. *Ipomoea indica*, *I. nil*, *I. hederifolia*, *I. purpurea* and *I. cairica* are rarely found far away from human habitation and *I. alba*, *I. cairica*, *I. tricolor*, *I. indica*, *I. quamoclit* and I. carnea subsp. fistulosa are sometimes clearly garden escapes. The same is true for many species of the *Batatas* clade. *Ipomoea tiliacea*, *I. triloba*, *I. cordatotriloba*, *I. australis*, *I. leucantha*, *I. grandifolia* and *I. trifida* are all recorded as characteristic of disturbed bushy ground and are rare in truly natural habitats.

### Phenology

Many species have a distinct, relatively short flowering season. The only country where details are documented, albeit superficially is Bolivia (Wood et al. 2015). Similar details are largely unknown from other countries although information about 12 Mexican species is provided by Chemás-Jaramillo and Bullock (2005). The short flowering season is at least a partial explanation for why some species are rarely collected and so are only known from one or two examples.

Certain generalisations, however, are possible. The erect cerrado and grassland species with a stout xylopodium often come into flower soon after the start of the spring rains, possibly being stimulated into growth and flowering by the fire that often precedes the onset of rain. Annual species, in contrast, use the moist summer season for growth and come into flower towards the end of the summer, their flowers often persisting long into the winter dry season (see Chemás-Jaramillo and Bullock (2005) for examples from Mexico). Most dry forest and semi-desert species flower during the summer rainy season, taking advantage of the short wet period to produce their flowers. One subset, however, prefers to flower in the height of the dry season when they are leafless so their seeds are mature when the rains eventually begin (*Ipomoea schulziana*, *I. juliagutierreziae*). Plants of flooded pampas flower after the waters recede during the winter. There is no clear pattern amongst species of moist forest. The archetypical rain forest species, *I. philomega* flowers at the height of the summer but other moist forest species such as *I. regnellii* and *I. cryptica* prefer the winter.

There are many individual subtleties, which need careful observation and recording before any explanation can be provided. In Eastern Bolivia in areas of a similar altitude and climate, the first author has observed the following sequence, although these observations may be partially dependent on the date of the onset of rain. To see flowering specimens of *I. hirsutissima*, *I. cerradoensis* and *I. psammophila*, it is best to visit in October and November; to find *I. schomburgkii*, *I. aprica*, *I. caloneura* and *I. paulistana* it is best to look in December or January; to find *I. graniticola* and *I. densibracteata* February to early March would be best; March to early April would be good for *I. amnicola*, *I. abutiloides* and *I. megapotamica*; April to June would be good to find *I. bonariensis*, *I. argentinica*, *I. rubens*, *I. bahiensis* and *I. cordatotriloba*; to find *I. ramosissima*, *I. setifera*, *I. paludicola* or *I. eriocalyx* June or July would be best, while July or August might be best for *I. regnellii*, *I. lactifera* and *I. cryptica*. Finally you should note that you might find *I. maurandioides* in flower at almost any season.

### Anthesis

*Ipomoea* species are commonly named “Morning Glory” because the flowers of several cultivated species, notably *I. indica*, open at dawn and close before midday. However, while this observation may be a useful generalization, it is only a partial truth. Much depends on the strength of the sun and many morning-flowering species will continue in flower well into the afternoon on a dull day. Conversely night-flowering species, such as *I. alba*, *I. muricata* and *I. violacea* may remain open during clouded, sunless days. These observations indicate that research suggesting different species flower for a specific number of hours (Chemás-Jaramillo and Bullock 2005) should be treated with caution. However, there is no doubt about the truth of their observation that the flowers of some species, especially robust perennials, such as *I. ampullacea*, *I. bracteata* and *I. pedicellaris*, remain open for much longer periods than those of more slender species.

### Economic uses

Much the most important species of *Ipomoea* economically is *I. batatas*, the sweet potato, which is reported to be amongst the ten most important staple food crops worldwide (Woolf 1992, FAO 2017). Although clearly of American origin it is widely cultivated in almost all tropical and subtropical countries for its root tubers (storage roots). The largest contemporary producer is China but much of Chinese production is used as animal fodder (FAO 2017). It has a number of important advantages as a human food. It is second only to the potato in productivity per hectare. It is more drought resistant than many important staple crops such as maize. The common orange-fleshed varieties are an outstanding source of Vitamin A and have significant quantities of Beta-carotene, potassium and various other elements important for human nutrition (Kurabachew 2015). Indeed per gram it is richer in potassium than bananas (USDA 2017. The purple-fleshed varieties have enjoyed a recent vogue as brain food but it is unclear whether this is merely a fashion fad or based on sound evidence.

Other species of *Ipomoea* produce root tubers but there are only occasional reports of their use, usually as a famine food. Amongst species whose tubers are reported to be used for food are *I. leptophylla*, *I. pubescens*, *I. pandurata* (Haddock et al. 2015), *I. plummerae* (Gutiérrez-R 2016) and *I. serrana* (Vasconcelas et al. 2016).

The leaves of some species of *Ipomoea* are used as a vegetable. Much the most important is *I. aquatica*, the water spinach or kangkong, which is widely used as a stir-fry vegetable in South East Asia, although it has not achieved much popularity outside the region. The leaves of other species are occasionally used as vegetables, including *I. batatas* itself and apparently *I. littoralis* (Austin 1991b), although it is unclear whether they enjoy general use or are a resort at times of famine. It is possible that the leaves of other species could be used as a vegetable but the leaves of some species are potentially harmful (Meira et al. 2012). *Ipomoea malvaeoides* and I. carnea subsp. fistulosa, for example, are avoided even by goats and are unpalatable, if not actually poisonous, to animals and presumably to humans.

Various species of *Ipomoea* are cultivated as garden ornamentals. In extra-tropical countries, relatively quick growing annual species are favoured, particularly *I. indica*, *I. purpurea*, *I. nil*, *I. quamoclit* and *I. tricolor*. In tropical countries, perennials are more common. The most conspicuous is I. carnea subsp. fistulosa, which is widely cultivated for its erect habit and profuse flowers. *Ipomoea cairica* is often planted to cover walls and unattractive bushes. *Ipomoea alba* and *I. muricata* are also sometimes grown in gardens and on boundary fences. *Ipomoea horsfalliae* is a widely planted liana that is grown in many tropical countries for its attractive red flowers, but is not reported to set seed and so is never naturalized. *Ipomoea quamoclit* and, less commonly, *I. lobata* are also grown quite frequently and sometimes become naturalised. There are occasional reports of the cultivation of other species including *I. nervosa*, *I.pauciflora* and *I. intrapilosa* but this is not common practice.

Various species of *Ipomoea* have had medicinal uses since pre-Colombian times, broadly for two purposes. The seeds of several species are known for their hallucinogenic properties as they contain small quantities of LSD-like substances (Steiner and Leistner 2018). Amongst the species used as a hallucinogen are *I. tricolor* “Heavenly Blue”, *I. purpurea*, *I. alba*, *I. corymbosa* and *I. nervosa*. The roots of several species have been used as a purgative and marketed under the name “jalapa”. *Ipomoea purga* is the best-known species used for this purpose but others such as *I. simulans*, *I. orizabensis* and *I. jalapa* are sometimes reported as having similar properties, although their medical value requires confirmation. Meira et al. (2012) document many actual and potential medical uses of *Ipomoea* species.

## Morphological characters and their use in species delimitation

In the following section we discuss the range of characters which have proved useful in species delimitation and have indicated some of the pitfalls in their use. Taxonomic decisions often have to be made using incomplete material. Many species of *Ipomoea* are extremely localized in their distribution and many of their morphological characters are unknown, particularly the roots and the fruit characters, which are unknown for perhaps a third of species.

### Habit and lifeform

Species of *Ipomoea* may be annual or perennial, herbaceous or woody, twining (or at least scrambling), erect, decumbent or prostrate. All of these characters are potentially useful in species delimitation and are used in the keys. It is useful, for example, to distinguish between lianas and scandent herbs or between prostrate or erect herbs but the distinctions need to be treated with caution. Many species have a woody rootstock and herbaceous stems, which may or may not be woody at the base. Stems may become somewhat woody with age. Twining plants may be trailing in the absence of shrubs to climb on. We have also avoided the use of the term *vine* as it is sometimes used to mean a woody climber (like the grape vine), so almost a synonym of liana, and sometimes to mean a relatively slender twining plant.

Annual species are characterized by having fibrous roots and typically flower in the late rainy season (tropical summer) as they require sufficient time to reach maturity after the onset of rains. In the herbarium, in the absence of roots, annuals can often be identified by their slender habit and the presence of mature capsules on flowering specimens. Perennial species, in contrast, are relatively stout and often lack mature capsules on flowering specimens or are almost entirely without corollas on fruiting specimens. It is possible that some normally annual species perenniate under suitable circumstances, especially in areas with no distinct dry season. There are no known erect annual species. Annual species are not found in Clades A1 or A2. In contrast they are well-represented in the Batatas (A3 in part), Pharbitis (B1 in part), Quamoclit (B2 in part) and the Pedatisecta Clades (B2 in part).

The majority of species are twining perennial herbs or lianas with petiolate, ovate, cordate leaves. The inflorescence is formed of pedunculate axillary cymes, the cymose structure usually being very obvious, although the cymes are sometimes reduced to single flowers. There is a tendency for some of the lianas to develop inflorescences on short leafy branchlets, rather than from the axils of the stem leaves.

Somewhat similar is a less well-defined assembly of essentially trailing plants. At one extreme these species root at the nodes and form extensive mats, in one case (*Ipomoea aquatica*) extending its stems to float on shallow water. Two widespread submaritime species, *I. pes-caprae* and *I. imperati*, are good examples of this growth form. More common are trailing species that do not root at the nodes. They usually grow in open, often sandy inland habitats. These trailing species often have shortly petiolate, elliptic leaves rounded to truncate at the base combined with axillary cymose inflorescences, these sometimes being shortly pedunculate. These trailing plants are, thus, apparently intermediate morphologically between the true climbers and the erect species. Some trailing species are morphologically indistinguishable from the climbers, the prostrate habit apparently the consequence of the absence of suitable plants to climb. *Ipomoea maurandioides*, a South American species principally of rock outcrops, is one such example.

The erect habit is usually associated with subsessile, oblong, lanceolate, or oblong-elliptic cuneate-based leaves with a terminal inflorescence, the upper leaves clearly bract-like and the pedicels and peduncles reduced so the inflorescence is subracemose or even subspicate in form. Species with this habit occur mostly in open grasslands and especially in the cerrados of South America. Most species produce annual stems from a tough woody perennial subterranean xylopodium, which is resistant to fire, a characteristic and perhaps defining feature of these habitats. Erect species are found in many different clades but are unknown in the Batatas, Quamoclit and Pharbitis Clades and rare in Clade B.

The erect habit is also associated with a number of shrubs and small trees often treated as Section Arborescens. These usually (always?) have white latex and often flower when leafless or nearly leafless. The inflorescence often develops on short branchlets and is not obviously axillary and cymose in structure. The corolla is white with a dark centre, subcampanulate to funnel-form in shape and possibly bat-pollinated (Felger and Austin 2005). Species with these characteristics mostly occur in very dry forest along the mountain chains of Mexico, Central America and the Andes and are completely absent from Brazil and the Caribbean.

Much the most widespread and common erect species, Ipomoea carnea subsp. fistulosa fits none of the above characteristics, having ovate cordate leaves and pink flowers in axillary cymes but its uniqueness is perhaps a consequence of its close relationship with Ipomoea carnea subsp. carnea which is a characteristic climbing species, from which it is presumably diverged.

### Underground parts

Although annual species are generally known to have fibrous roots, little reliable information is available about most of the perennial species. Erect species of the cerrado nearly always arise from a woody xylopodium but this is known to vary considerably in form and development from species to species. *Ipomoea hirsutissima*, for example, has very large somewhat woody tuberous roots. Similar storage roots are seen in other species in Clade A1 including *I. lilloana* (Figure 15D) and *I. opulifolia* (Figure 15E). The best-known species for its tuberous rootstock is, of course, the edible *I. batatas*, but storage roots occur in many different clades throughout the genus, such as *I. bonariensis* and *I. platensis* in Clade A2, this last sometimes cultivated as a succulent. Those of *I. pubescens* and *I. plummerae* in Clade B are sometimes eaten, while those of *I. pandurata* and *I. leptophylla* in Clade C are noted for their size. Other species have tubers which can be used medicinally, notably *I. purga* and *I. jalapa*. However, for the vast majority of species there is no accurate information about their rootstock. Although this character may prove to be of economic importance in the future and is significant in discussions around the origin of the sweet potato (Muñoz-Rodríguez et al. 2019), it can be little used at the present time in species delimitation.

### Latex

White latex is recorded as present in many species and is sometimes abundant, notably in trees and lianas, including species in the Arborescens and Calonyction Clades as well as in the aptly named *Ipomoea lactifera*. However, its presence often goes unrecorded and it may be more or less obvious according to climatic conditions.

### Stem

Stems may be entirely herbaceous, woody in the lower parts and herbaceous above, or entirely woody except for the new growth. Stems may be glabrous or variously hirsute, the indumentum usually being similar to that of the peduncles, petioles and leaves, especially the abaxial surface of the leaves. There is a tendency for older stems to be somewhat glabrescent. Unusual features of the stem include distinct wings (*Ipomoea pterocaulis*, *I. splendor-sylvae*, *I. subalata*, *I. kahloae*), squamose dark glands (*I. balioclada*), warty protuberances (*I. verruculosa*, *I. tuboides*), spinules (*I. spinulifera*), soft spines (*I. setosa*), soft fleshy teeth (*I. muricata*, *I. alba*, *I. parasitica*) and granulose protuberances (*I. granulosa*).

### Indumentum

Species may be glabrous or variously hirsute. There is a good deal of intra-species variation and this has often proved to be an unsatisfactory character in species delimitation. Many species or varieties have been recognized over the years based on the presence or absence of hairs and have subsequently been abandoned. Despite this important proviso, many species have a characteristic indumentum which is readily recognized. Species which are always glabrous in their vegetative parts form a long list, as do those which are characteristically sericeous or tomentose. A sericeous indumentum is characteristic of almost all species previously placed in *Argyreia*, *Rivea*, *Turbina* and *Stictocardia* as well as many that have always been included in *Ipomoea*. Some unusual indumentum types include:

• Stellate hairs. These are characteristic of certain species notably *Ipomoea bonariensis* from South America, *I. scopulorum* from Mexico and *I. luteoviridis* from Hispaniola. In cases where they are mixed with simple hairs they may be very difficult to observe and pass unnoticed. They are also characteristic of the Astripomoea Clade, which is restricted to Africa.

• T-shaped hairs. *Ipomoea malpighipila* was named on the basis of the presence of T-shaped hairs. They are not reported from other species, except the related *I. aemilii*, and are difficult to observe even in these species.

• Scattered long fine hairs. *Ipomoea clavata*, *I. dolichopoda*.

• Density and appearance. Many species are densely hairy especially on young stems and the abaxial surface of leaves but sometimes on all vegetative parts. Where hairs are dense the leaves are often white or grey in colour and characteristic of the species. This kind of indumentum is not always easy to define and is sometimes described as canescent, sericeous, tomentellous, tomentose or densely pubescent by different authors.

• Gland dots. Distinct gland dots are found in some species, especially on the abaxial leaf surface but sometimes on other vegetative parts or even the corolla. They usually appear as dark dots and are so characteristic of *I. tiliifolia* that they are often regarded as a defining characterstic of the Stictocardia Clade (Austin and Demissew 1997). They occur sporadically elsewhere as in some specimens of *I. megapotamica*, *I. reticulata* and *I. batatoides*. As white dots they are characteristic of *I. eremnobrocha* and the related species *I. isthmica* and *I. peteri*.

### Extrafloral nectaries

These have been reported in many species including *Ipomoea alba*, *I. batatas*, *I. bonariensis*, *I. carnea*, *I. indica*, *I. leptophylla*, *I. mauritiana*, *I. muricata*, *I. pes-caprae* and *I. tuboides* (Keeler 1977, 1980, 1985, Keeler and Kaul 1979, Mondal et al. 2013, Meeuse and Welman 2000). These are usually found on the petioles, at the base of the leaf where it joins the petiole or on the sepals. It is postulated that they attract ants which help to protect the plant from predators. However, they are not readily observed and their taxonomic value is uncertain as they are not necessarily constant in a particular species (for an example, see the discussion about *I. indica* in Keeler (1985). The case of *I. tuboides* is particularly interesting as there are no native ants in Hawaii, suggesting perhaps that the nectaries evolved in the ancestor of this species before it was dispersed to Hawaii from the American mainland.

### Leaves

Leaves are exstipulate but a few species have pseudo-stipules (notably *Ipomoea cairica*, *I. fissifolia* and *I. quamoclit*), formed by modified leaves or prophylls. Leaf size can be distinctive but difficult to quantify diagnostically. Large leaves are a feature of a few species such as *Ipomoea ampullacea*, *I. magnifolia* and *I. philomega* whereas small leaves are characteristic of many annual species but also of some perennials such as *I. hartwegii* and *I. rupicola*.

Leaf shape is mostly related to habit with almost all climbing species having ovate to deltoid leaves with a truncate, cordate or sagittate base. Elliptic leaves are rare and mostly found in trailing species. Lanceolate, oblong or oblong-elliptic leaves are mostly a feature of erect species. Some unusual shapes occur, such as the strap-shaped leaves of *I. tenuissima*.

Leaves may be entire or variously divided. Pinnate leaves are only present in *Ipomoea quamoclit*, and pinnatifid to lyrate-dentate leaves in a few Mexican species (*I. ancisa*, *I. sescossiana*, *I. tacambarensis*, *I. stans*). A much larger number of species have leaves palmately lobed. The number of lobes, usually 3 or 5, occasionally more, and the depth of lobing are often characteristic of a particular species. However, leaf lobing is often an inconstant character, many species having entire-leaved forms or forms that intergrade with the normally lobed forms. The leaves of some, such as *I. bonariensis*, *I. clausa*, *I. microdactyla* or *I. mauritiana* are notoriously variable in form. A relatively small number of species have leaves palmately divided into separate leaflets and this character is usually constant. Species which present forms with both lobed leaves and leaves divided into separate leaflets occur in only a very few species (*I. cairica*, *I. bonariensis*, *I. homotrichoidea*).

The leaf base is sometimes distinctive, particularly in those species that have leaves with strongly cordate or strongly cuneate bases. Sagittate or hastate leaves are also often distinct but may intergrade with the more common cordate leaf base. Rounded leaf bases often intergrade with shallowly cordate or truncate leaf bases and are difficult to characterize.

The leaf margins are usually entire to slightly undulate but a few species have distinctly dentate leaves (*I. odontophylla*, *I. schaffneri*, *I. noctuliflora*, *I. ignava*, *I. peruviana*, *I. descolei* and *I. erosa*). A few species may have 1–several rather large teeth on the margins, usually towards the base (*I. acanthocarpa*, *I. dumetorum*, *I. eriocalyx*). In the majority of species the leaf apex is acute to acuminate, although the actual tip may be somewhat obtuse. The tips are commonly mucronate but in a few cases the midrib extends as a mucro several millimetres in length (*I. walteri*). In a few species the apex is distinctly retuse (*I. pes-caprae*).

In general, petiole length is of little significance except that short or absent petioles correlate with an erect habit and elongate leaf shape as noted earlier. One curious feature is the fusion of the petiole and the peduncle at least for part of their length (*I. connata*, *I. bracteata*, *I. dumosa*).

### Inflorescence

Most inflorescences consist of cymes that arise from the leaf axils. Cymes are nearly always solitary but are very variable in the number of flowers. In many species the cymes are reduced to a single flower while in others the cymes may be compounded with up to 15 or more flowers. The number of flowers in the cyme is often a useful although somewhat imprecise taxonomic character.

Not all inflorescences are obviously cymose in structure, some are more or less corymbose (especially in the Quamoclit Clade) or racemose (e.g. *Ipomoea bombycina*, *I. reticulata*, *I. corymbosa*) or umbellate (some forms of *I. batatas*), even appearing paniculate in some forms of *I. lineolata* or *I. philomega*. In quite a few species, the pedicels are very short so the inflorescence is subcapitate in form. In the Arborescens Clade and also in a number of woody lianas, the inflorescence arises on short leafy (bracteate) branchlets with no obvious cymose structure.

### Bracts and bracteoles

We have generally avoided using the term bract since in most twining or trailing species, the bracts are not clearly differentiated from the leaves, the cymes arising in the axils of the leaves which function as bracts. In the erect species and also in some or the arborescent species where the inflorescence is either terminal or borne on small branchlets bracts are more clearly differentiated from leaves, typically smaller and narrower and diminishing in size towards the branch tips and, in this situation, we have used the term bract. Some authors, however, use the term bract for the very different structures that arise at the inflorescence branching points or at the base of the pedicel in unbranched inflorescences. We refer to these as bracteoles, only rarely differentiating between primary bracteoles (at the first branching point) or secondary bracteoles (at the higher branching points) as these rarely differ in any significant way. In many species the bracteoles are inconspicuous and caducous (and have never been observed in a few species), but in others they are prominent and persistent, especially in the Pharbitis Clade, and occasionally even forming an involucre around the flowers where the pedicels are very short, notably in *I. neurocephala* and *I. involucrata*.

In the majority of species the bracteoles are small (< 3 mm long), often linear, lanceolate or scale-like and caducous. In a few species, *Ipomoea blanchetii* is an example, we have not observed bracteoles in any specimen available to us. In others, they are relatively persistent, particularly in species, with a subcapitate inflorescence. These include *I. indica*, *I. villifera*, *I. mairetii*, *I. argentinica*, *I. asplundii*, *I. chrysocalyx*, *I. racemosa*, *I. amazonica*, *I. eriocalyx*, *I. setifera*, *I. fimbriosepala*, *I. burchellii*, *I. pohlii* and *I. mcvaughii*. In a very few species the bracteoles are expanded, persistent and form an involucre around the inflorescence as in *I. neurocephala*, *I. involucrata*, *I. bracteata and I. suffulta*.

### Peduncles and pedicels

Peduncles may be short or long and the length is sometimes significant. Most species with a terminal inflorescence have very short peduncles and pedicels. However, some trailing or twining species are also remarkable for their relatively short peduncles. These include *Ipomoea eriocalyx* and a miscellaneous group of other species, such as *I. lindenii*, *I. chapadensis*, *I. riparum* and *I. chrysocalyx* but is most common in Clade A2. Species in this clade with very short peduncles include *I. microdonta*, *I. lachnea* and *I. calophylla* from the Caribbean, *I. goyazensis* from South America, *I. isthmica* and *I. heterodoxa* from Central America and *I. pseudoracemosa*, *I. pruinosa*, *I. conzattii* and *I. tehuantepecensis* from Mexico. Many of these species with short peduncles also have short pedicels so the whole axillary inflorescence is very compact. However, there is also a group of species with relatively long peduncles but a subcapitate inflorescence in which the flowers are borne on short pedicels. This is particularly characteristic of the Pharbitis Clade (*I. indica*, *I. neurocephala*, *I. mairetii*, *I. lambii* and *I. villifera*) but is also noteworthy amongst many unrelated species including *I. racemosa*, *I. amazonica*, *I. argentinica*, *I. bahiensis*, *I. eriocalyx*, *I. fasciculata*, *I. exserta* and *I. batatas*. Species with pedunculate subcapitate inflorescences often but not always have a bracteolate inflorescence. Very long peduncles are also distinctive in species such as *I. marcellia*, *I. macdonaldii*, *I. longibarbis*, and *I. austrobrasiliensis*. Unusually long pedicels are rarely apparent but are a feature of *I. pedicellaris* and its allies which include *I. regnellii*, *I. lindenii* and *I. tentaculifera*, these inflorescences appearing very lax. Unusual features of the peduncle include the winged peduncles of *I. decemcornuta* and *I. kahloae*, the peduncle fused with the petiole for some of its length (*I. connata*, *I. dumosa*, *I. bracteata*) and the peduncle that passes through the leaf sinus (*I. aristolochiifolia*, *I. huayllae*). Very occasionally pedicels are unusually slender and coiled (*I. heptaphylla*, *I. tenera*).

### Sepals (Figures 2–7)

The calyx is formed of five overlapping, free sepals. The two outer sepals are usually similar in size and form as are the two inner sepals, which often have relatively broad, scarious, glabrous margins. The middle sepal is intermediate in size and shape and is commonly asymmetrically scarious. The sepals are often of considerable taxonomic significance and constitute important conserved characters at the species level. The differences in size and shape between the inner and outer sepals are often of great significance. The apex is frequently especially diagnostic. Many species have mucronate sepals, but the mucros are often caducous so some or even all sepals may appear muticous or retuse. Also important is the abaxial surface of the outer sepals which may show all kinds of variation in indumentum, venation and surface which can be smooth, muricate or armed with soft spines. In a few species notably in the Arborescens Clade, the presence of hairs on the adaxial surface is significant. As observed by Hallier (1893a), the sepals are accrescent in fruit, more especially so in the lianas such as *I. brasiliana* or *I. tiliifolia*, sometimes doubling their size after anthesis and becoming wider so sepals which were lanceolate at anthesis may become ovate in fruit. They may also enclose or nearly enclose the capsule.

Many sepals display unusual features including:

• Very unequal sepals: *I. anisomeres*, *I. cryptica*, *I. squamosa*, *I. asarifolia*, *I. paludicola*, *I. maurandioides*, *I. macedoi*.

• Adaxial (inner) surface hirsute: Arborescens Clade, *I. longibracteolata*, *I. magna*.

• Subterminal awns: all species in the Quamoclit Clade.

• Sepals terminating in a long awn: *I. alba*, *I. muricata*, *I. nil*, *I. hederacea*. Sepals of some other species, such as *I. incarnata*, may be interpreted as terminating in an awn.

• Sepals with fleshy spine-like trichomes: *I. crinicalyx*, *I. echinocalyx*, *I. altoamazonica*, *I. silvicola*, *I. setosa*, *I. tentaculifera*, *I. lozanii* (smaller than in other species),

• Sepals with a prominent abaxial appendage, *I. rosea*, *I. bahiensis*; *I. decemcornuta*.

• Sepals with swollen abaxial tumour: *I. appendiculata*.

• Sepals with 1–2 prominent black abaxial glands: *I. hieronymi*, *I. megapotamica*.

• Sepals muricate: *I. plummeae*, *I. capillacea*, *I. madrensis*, *I. aristolochiifolia*, *I. pedicellaris*, *I. obscura*, *I. ochracea*, *I. cairica*, *I. asarifolia*, *I. paludicola*, *I. procurrens*, *I. coriacea*.

• Sepals with prominent longitudinal ribs: *I. fimbriosepala*, *I. setifera*, *I. parvibracteolata*, *I panduata*.

• Sepals with fimbriate margins: *I. tenera*, *I. sidifolia* (sometimes).

• Sepals with a prominent cordate base: *I. macedoi*, *I. apodiensis*, *I. pantanalensis*, *I. pubescens*, *I.lindheimeri*.

The great diversity of sepal form is curious and not easily explained. It has been suggested that the development of coriaceous and large sepals may have evolved in response to the need to protect nectar glands from robber insects. (McDonald 1991).

**Figure 2.**
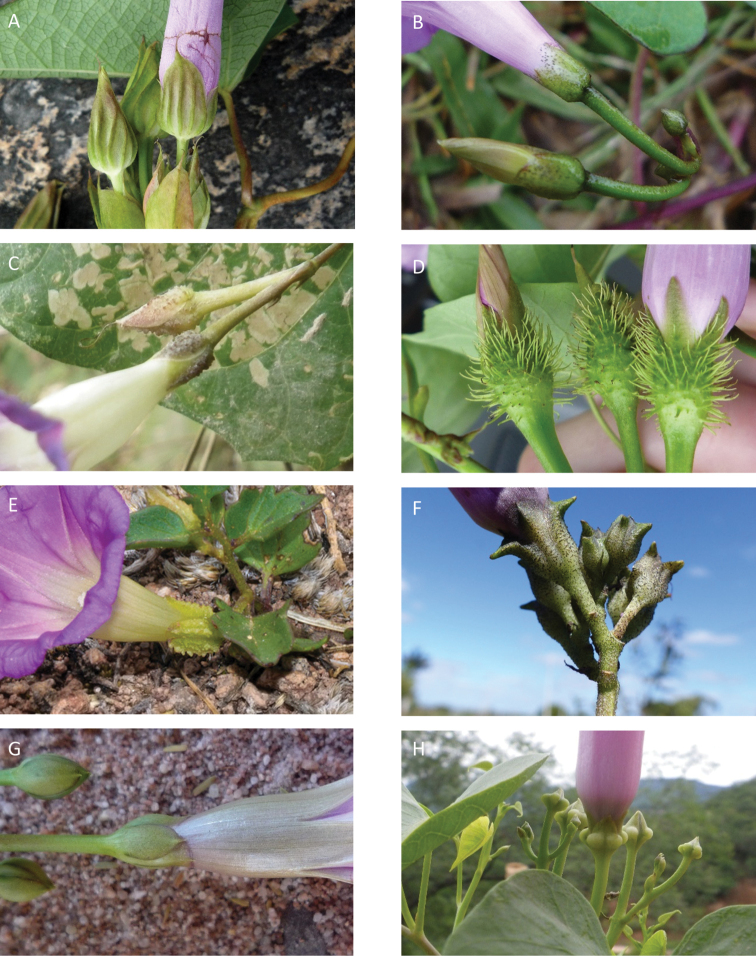
Sepals of *Ipomoea* species. **A***I. setifera***B***I. dumetorum***C***I. aristolochiifolia***D***I. crinicalyx***E***I. plummerae***F***I. bahiensis***G***I. amnicola***H***I. appendiculata*. Photographs of **A** (*Wood et al.* 27771) **B** (*Wood et al.* 27654) **D** (*Wood et al.* 27606) and **G** (*Wood et al. 27706*) by Beth Williams **C** (*Wood* 27926) **H** (*Wood et al.* 28024) by John Wood **E** by Mario Giorgetta **F** (*Queiroz et al.* 15950) by Hibert Huaylla.

**Figure 3.**
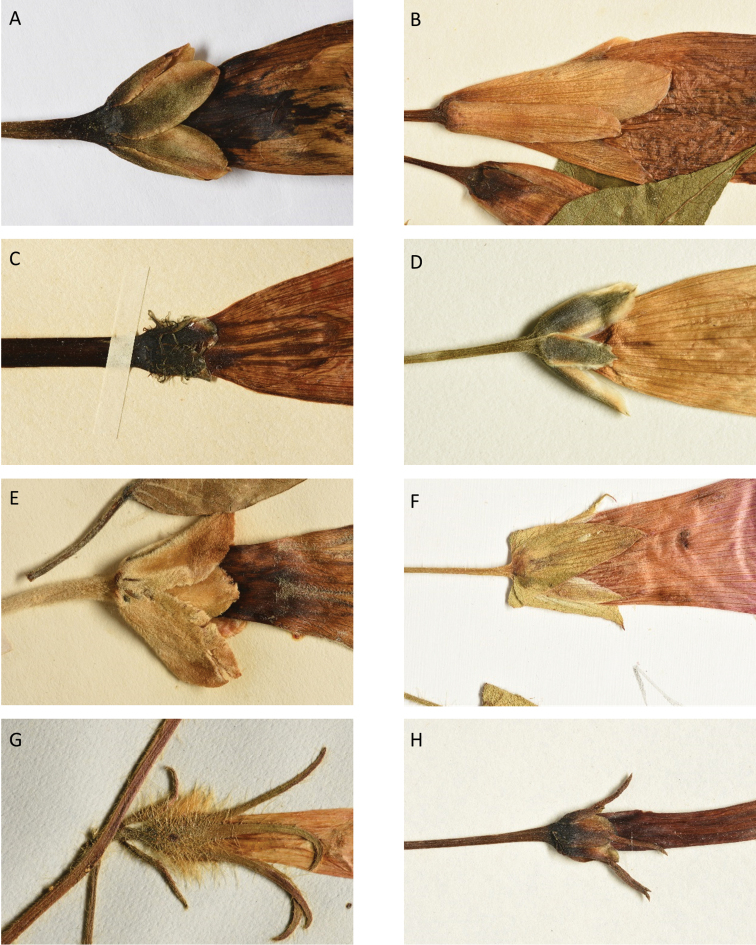
Sepals of *Ipomoea* species. **A***I. pauciflora***B***I. bernoulliana***C***I. tentaculifera***D***I. hartwegii***E***I. murucoides***F***I. pantanalensis***G***I. hederacea***H***I. funis*. Photographs of **A** (*Harling et al.* 15403) **B** (*Standley* 27496) **C** (*Pringle* 6702) **D** (*Santos Martínez* 2228 **E** (*Pringle* 6066) **F** (*Pott* 6399) **G** (*McCarthy* s.n.) **H***Andrieux* 600 by John Baker.

**Figure 4.**
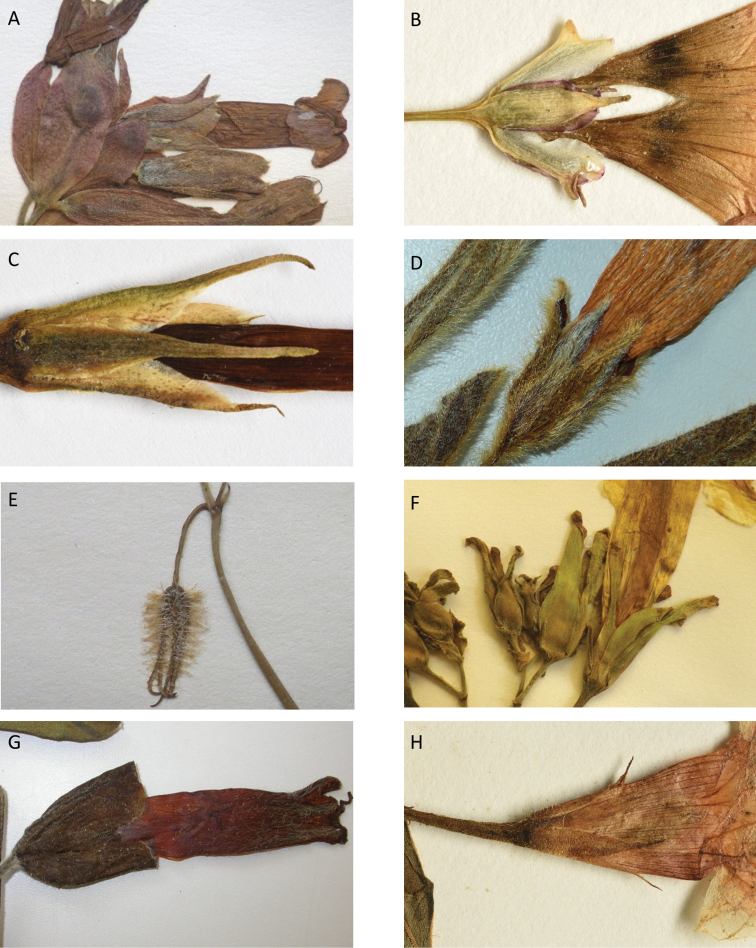
Sepals of *Ipomoea* species. **A***I. racemosa***B***I. rosea***C***I. alba***D***I. hirsutissima***E***I. barbatisepala***F***I. ampullacea***G***I. gigantea***H***I. longeramosa*. Photographs of **A** (*R.A. & E.S. Howard* 8863) **E** (*González Ortega* 874) and **F** (*Lott & Wendt* 2192) by John Wood; **B** (*Harley et al.* 54830); **C** (*Fendler* 589) and **H** (*Pickersgill et al*. RU72-400) by John Baker; **D** (Mendoza 4365) and **G** (*Mendoza* 4645) by Moises Mendoza.

**Figure 5.**
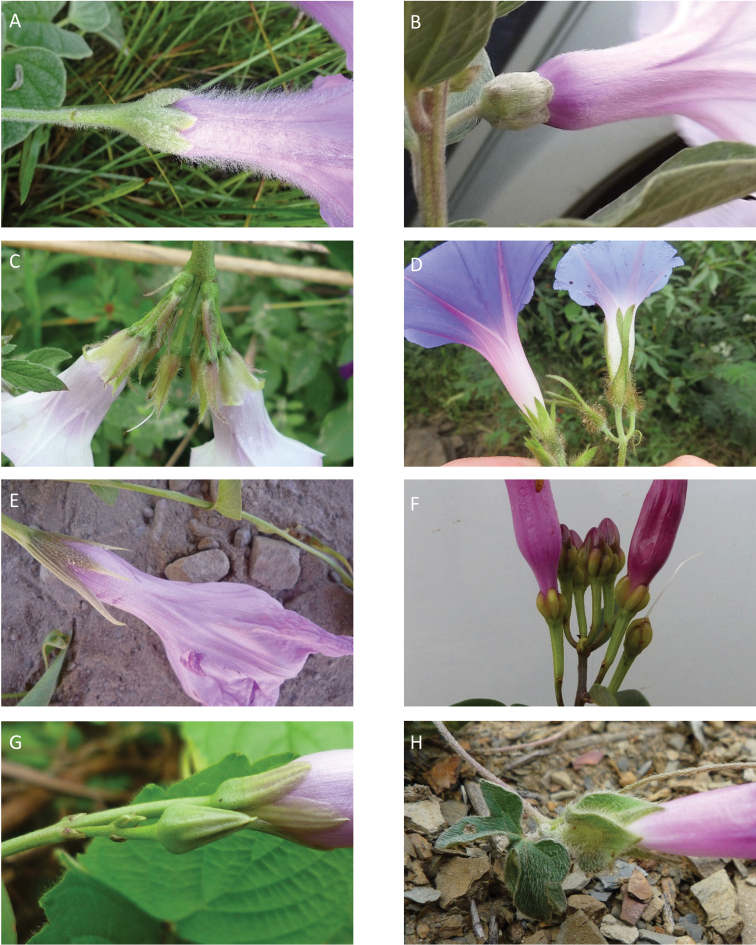
Sepals of *Ipomoea* species. **A***Ipomoea descolei***B***I. paraguariensis***C***I. australis***D***I. purpurea* (left), *I. nil* (right) **E***I. incarnata***F***I. pintoi***G***I. maurandioides***H***I. pubescens*. Photographs of **A** by Hector Keller; **B** and **G** by T. Carruthers; **C** (*Wood et al.* 27708); **E** (*Wood* 27756) and **H** (*Wood* 27675) by Beth Williams; **D** by John Pink; **F** (*Queiroz* 15956) by Hibert Huaylla.

**Figure 6.**
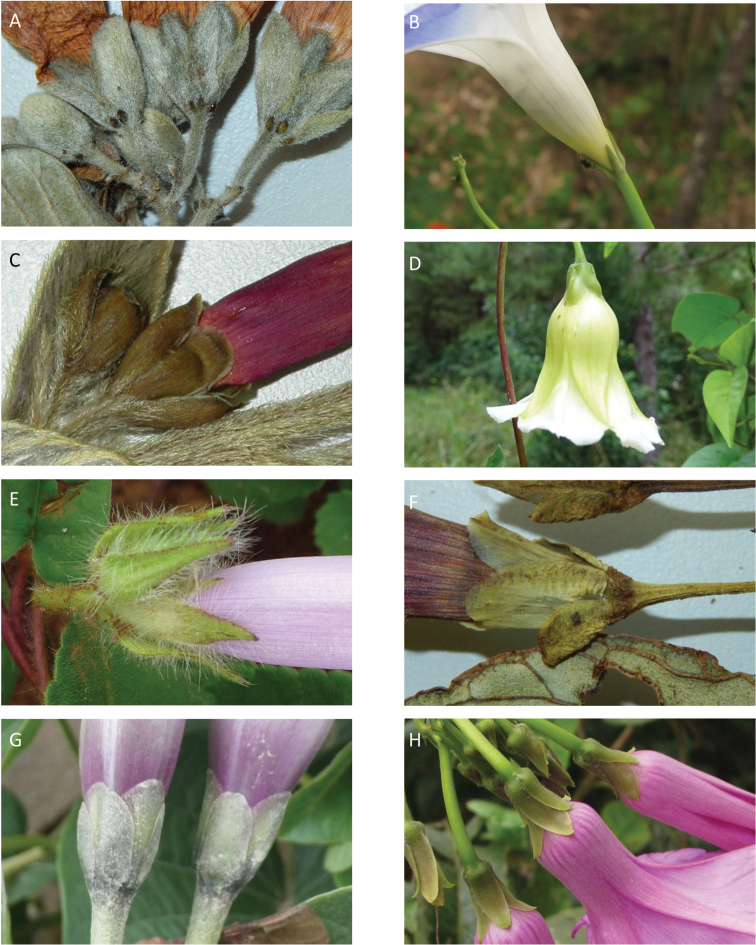
Sepals of *Ipomoea* species. **A***I. argyreia***B***I. tricolor***C***I. argentea***D***I. syringiifolia***E***I. eriocalyx***F***I. procurrens***G***I. tarijensis***H***I. regnellii*. Photographs of **A** (*Mendoza* 4899); **C** (*Mendoza* 4705) and **F** (*Mendoza* 4900) by Moises Mendoza; **B** (*Wood & Soto* 27960) and **H** (*Wood & Soto* 27951) by Daniel Soto; **D** by Hector Keller; **E** (*Wood et al.* 27809) by Beth Williams; **G** (*Wood* 27920) by John Wood.

**Figure 7.**
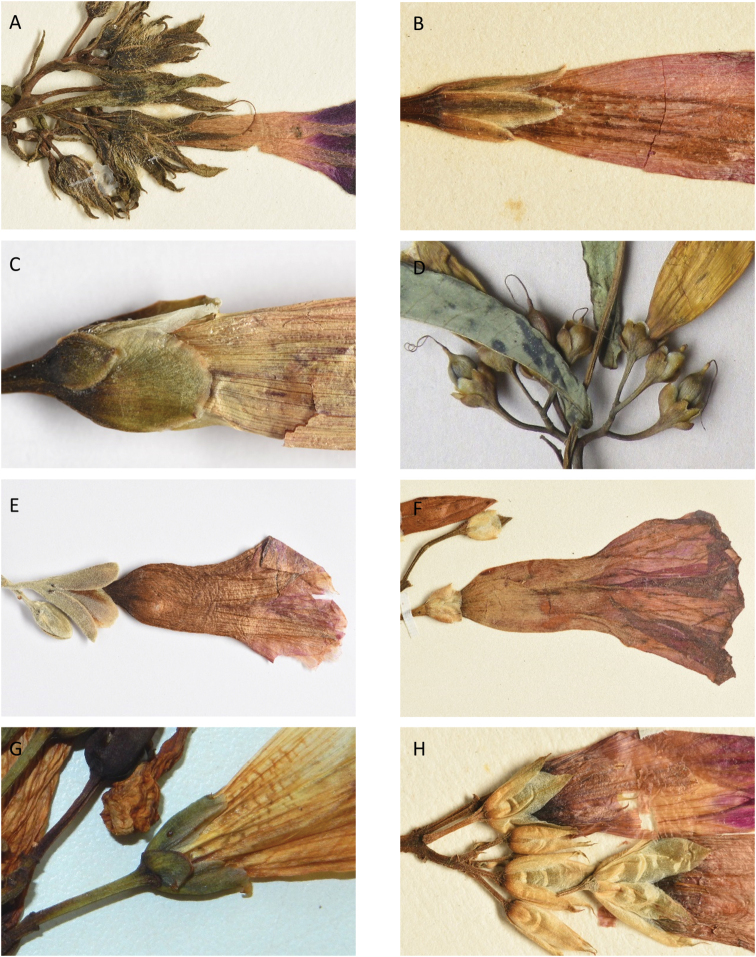
Sepals of *Ipomoea* species. **A***I. meyeri***B***I. ternifolia***C***I. cryptica***D***I. heterodoxa***E***I. sericosepala***F***I. splendor-sylvae***G***I. squamisepala***H***I. trifida*. Photographs of **A** (*Anderson* 1895) **B** (*Pringle* 4439) **C** (*Soto et al.* 1331) **E** (*Wood & Soto* 27550) **F** (*Wilkin* 472) and **H** (*Smith* 1570) by John Baker; **D** (*Wallnöfer* 9506) by John Wood; **G** (*Mendoza* 4902) by Moises Mendoza.

### Corolla (Figure 8)

The corolla is most commonly funnel-shaped, but is quite often campanulate, or hypocrateriform, or sometimes suburceolate, the limb usually prominent, entire or shallowly lobed but occasionally deeply lobed, or much reduced and present only as five indistinct teeth. The corolla exterior has five prominent midpetaline bands, which may be more darkly coloured and/or more pubescent than other parts of the corolla exterior. The corolla is very variable in size from less than 1 cm long in species like *I. eriocarpa* or *I. minutiflora* to over 10 cm in length in species like *I. jalapa*, *I. megalantha*, *I. parvibracteolata*, *I. subalata* and *I. pterocaulis*. Size is an unsatisfactory character at one level because of its variability within individual species, but is nonetheless often characteristic of a particular species.

**Figure 8.**
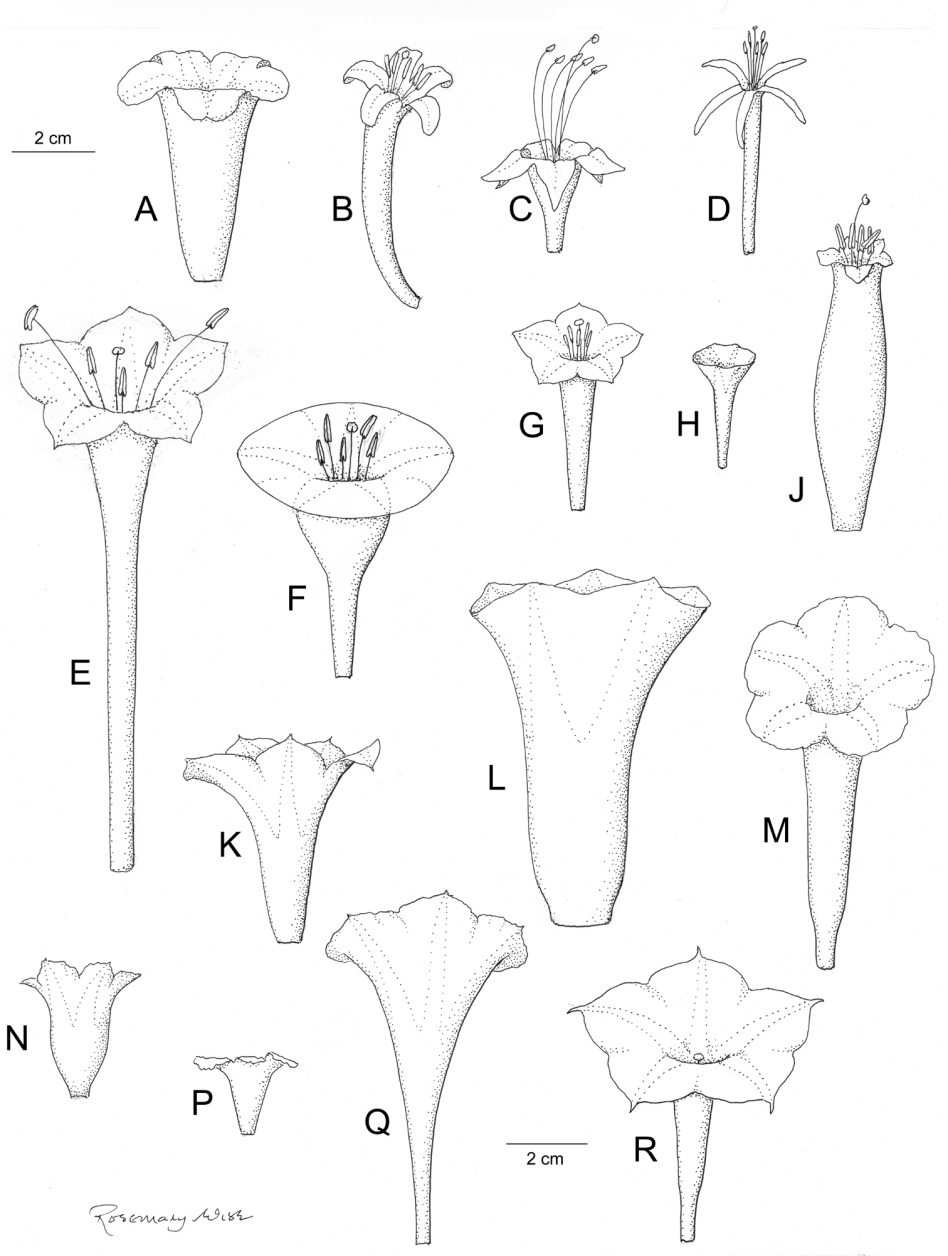
Corollas showing variations in form (side view), size, limb lobing and stamen exsertion. **A***Ipomoea argentea***B***I. repanda***C***I. neei***D***I. electrina***E***I. habeliana***F***I. santillanii***G***I. nationis***H***I. rubriflora***J***I. longistaminea***K***I. megapotamica***L***I. megalantha***M***I. neriifolia***N***I. syringifolia***P***I. ramosissima***Q***I. elongata***R***I. mucronatoproducta*. **A** from *Wood et al.* 25639 and photo; **B** from *Whitefoord* 5244; **C** from *Skutch* 2043; **D** from *Breedlove* 27626; **E** from *Bentley* 203; **F** from *Bourgeau* 3024; **G** from *Saunders* 987; **H** from *Wood et al.* 27678; **J** from *Pastore et al.* 2678; **K** from *Wood et al.* 28060; **L** from *Hassler* 9114; **M** from *Rezende et al.* 1011; **N** from *Stutz* 1426 and photo; **P** from *Bang* 2246; **Q** from *Purpus* 3904; **R** from *Wood & Villarroel* 25474. Drawn by Rosemary Wise.

Corolla shape is usually, perhaps always, related to pollination. The commonest corolla shape consists of a very short subcylindrical basal tube which is then gradually widened to the mouth. Corollas of this type are described as funnel-shaped, are usually, pink, sometimes blue or white, in colour and are apparently pollinated by bees. The limb is entire, undulate or shallowly (very rarely deeply) lobed. When the corolla is very short, the tube is more abruptly widened from the base and is campanulate in form. This is characteristic of some species in the Batatas Clade and also of small-flowered species with a cream corolla, such as *Ipomoea reticulata*, *I. corymbosa* and *I. syringiifolia*. This kind of corolla tends to intergrade with the common funnel-shaped corolla. The corolla of the Arborescens Clade and some other, mostly woody liana species is shortly funnel-shaped (almost campanulate), white or white with a dark purple centre. These flowers may be bat-pollinated (McDonald 1991: 73, Felger and Austin 2005, Queiroz et al. 2015) but confirmation is needed in most cases.

Other corolla shapes are less common. A hypocrateriform or salver-shaped corolla in which the nearly cylindrical corolla tube is only slightly widened at the mouth is associated with red flowers, exserted stamens and bird pollination. This corolla type is characteristic of the Quamoclit Clade but is also fairly common in the Clade A2 in South America (*Ipomoea exserta*, *I. longistaminea*, *I. ana-mariae*, *I. verruculosa*), and especially the Caribbean (*I. argentifolia*, *I. digitata*, *I. microdactyla*, *I. steudelii*). In Mexico and northern South America it is more commonly associated with Clade B in the Pharbitis Clade (*I. jamaicensis*) and elsewhere (*I. bracteata*, *I. dumosa*, *I. chenopodiifolia*, *I. retropilosa*, *I. tubulata*). Occasionally the corolla limb is very deeply lobed as in *I. repanda*, *I. hastigera*, *I. electrina* (which is orange, rather than red). An occasional variation is the suburceolate corolla, in which the corolla tube is essentially cylindrical but somewhat swollen in the middle and with a short corolla limb consisting of small teeth. *Ipomoea suburceolata* from Bolivia, *I. lobata* and *I. tehuantepecensis* from Mexico and *I. praecox* from Cuba have flowers of this kind. nother variation is found in plants with a white or pale blue corolla in which the tube is exceptionally long. This type of corolla is associated with night-flowering hawk moth pollinated species. The best-known species of this type is *I. alba* but there are various others with similar corollas including *I. habeliana*, *I. violacea*, *I. tuboides*, *I. scopulorum*, *I. riparum*, *I. santillanii*, *I. chiriquensis*, *I. ampullacea*, *I. macdonaldii and I. lottiae*. Species with this kind of corolla are notably more common on oceanic islands and in Mesoamerica and Mexico than elsewhere.

Corolla colour. Field and herbarium observations of flower colour need to be treated with caution. Flowers change colour during the course of the day, most obviously in the case of *Ipomoea nil*, which is blue when fresh but turns pink as it ages and appears pink in herbarium specimens. Equally, one collector’s purple is another collector’s pink or lilac or even red. Although the great majority of species have a corolla colour that is generally described as pink, there are many exceptions. White flowers (often with a dark centre) are characteristic of the Arborescens Clade and of several other woody liana species, such as *I. magna*, *I. longibracteolata*, *I. brasiliana* and *I. paradae*, and are in some cases pollinated by bats. Night-flowering moth pollinated species typically with a hypocrateriform corolla, such as *I. alba*, *I. santillanii*, *I. habeliana*, *I. violacea*, *I. ampullacea* have pure white corollas. Campanulate or funnel-shaped white flowers are noted for many different species in different clades but are more common in the Batatas Clade (*I. lactifera*, *I. lacunosa*), Clade A1 (*I. cerradoensis*, *I. macrorhiza*, *I. langsdorfii*, *I. vivianae*, for example) and Clade A2 (*I. proxima*, *I. suaveolens*, *I. pruinosa*) but occasionally occur elsewhere (*I. imperati*). Many usually pink-flowered species are recorded as sometimes being white-flowered (*I. acanthocarpa*, *I. bahiensis*, *I. carnea*). Slightly different are those species with creamy or violet-tinged flowers such as *I. lindenii*, *I. corymbosa*, *I. saopaulista*, *I. minutiflora* and *I. syringiifolia*. Truly yellow flowers are rare in American *Ipomoea* but include *I. ochracea*, *I. longeramosa* and *I. lutea*. There are many subtle variations between red and pink. Red flowers being principally a feature of the Quamoclit Clade, some Caribbean species (*I. montecristina*, *I. microdactyla*, *I. repanda* and a few South American species notably *I. cavalcantei*). Some corollas are described as purple and include forms of *I. indica*, *I. cuzcoensis* and *I. magnifolia*. Blue flowers also occur and are often associated with a white corolla tube. *I. hederacea*, *I. nil*, *I. aristolochiifolia*, *I. tricolor*, *I. marginisepala* and *I. cardiophylla* are species with this corolla colour.

Corolla indumentum. The indumentum of the corolla exterior is best observed on buds as there is some evidence that hairs are caducous in some species as the corolla matures. Hairs are often difficult to see on open corollas but are best searched for at the tips of the midpetaline bands. Although previous studies have not seen corolla indumentum as particularly important taxonomically, we have found it of great significance both at species and clade level. It is nearly always constant in a particular species, exceptions being very rare and their existence raising doubts about the circumscription of the species in the few cases where it has been noted (*Ipomoea lindenii*, *I. wolcottiana*, *I. brasiliana*). All species of the Quamoclit and Batatas Clades have corollas glabrous on the exterior. All species in Clade A2 have coriaceous sepals and glabrous corollas (except *I. discolor*). All species in the very large *Jalapa* radiation (Species 1–83) have pubescent corollas.

### Androecium

The stamens are of little taxonomic value. They are always five and may be included or exserted. If they are included they are unequal with two noticeably longer than the other three but, if exserted or near exserted, they are subequal in length. The filaments are slightly expanded near the base but are occasionally thickened and subtriangular as in *Lepistemon* and some forms of *Ipomoea batatoides*. The filaments are always glandular pilose at the base. In a few species hairs are reported to extend upwards along the filament and this has been used as a diagnostic character in the Batatas Clade. (Austin 1978b).

### Pollen (Figures 9, 10)

The pollen of *Ipomoea* is always globose and pantoporate with large supratectal elements that form acute or blunt spines. The presence of these echinulate supratectal elements is the diagnostic synapomorphy for *Ipomoea* within Convolvulaceae. Within this general pollen-type subtle variations are visible in the size of the pollen grains, in the number and shape of pores, in the number and structure of the supratectal elements, in the structure of the area surrounding the pores, the presence or absence of ‘basal cushions’ *sensu*Wilkin (1993) at the base of the supratectal elements and the extent of columellae in different parts of the pollen (Sengupta 1972, Pedraza 1983, Wilkin, 1993). In addition, individual pollen grains may look very different depending on whether the opercula or aperture membranes remain intact (Figure 10A, C–H) or not (Figure 10B) after acetolysis.

**Figure 9.**
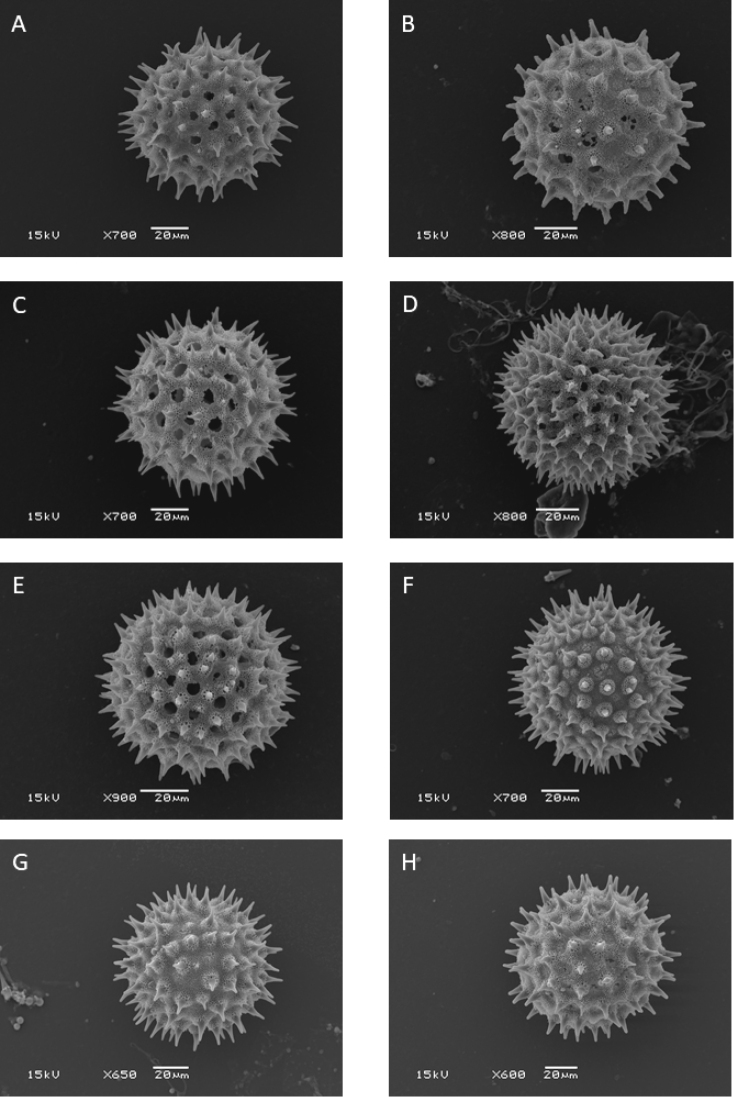
Pollen of *Ipomoea* species. **A***I. hieronymi* (*Wood et al.* 28055) **B***I. wolcottiana* (*Hughes et al.* 1911) **C***I. bonariensis* (*Wood et al.* 27871) **D***I. bahiensis* (*Queiroz* 15975) **E***I. maurandioides* (*Krapovickas & Cristóbal* 1573) **F***I. corymbosa* (Jurgensen 612) **G***I. sericosepala* (*Wood* 28122) **H***I. tiliifolia* (*Beddome* 5581). Photos by Robert Scotland.

**Figure 10.**
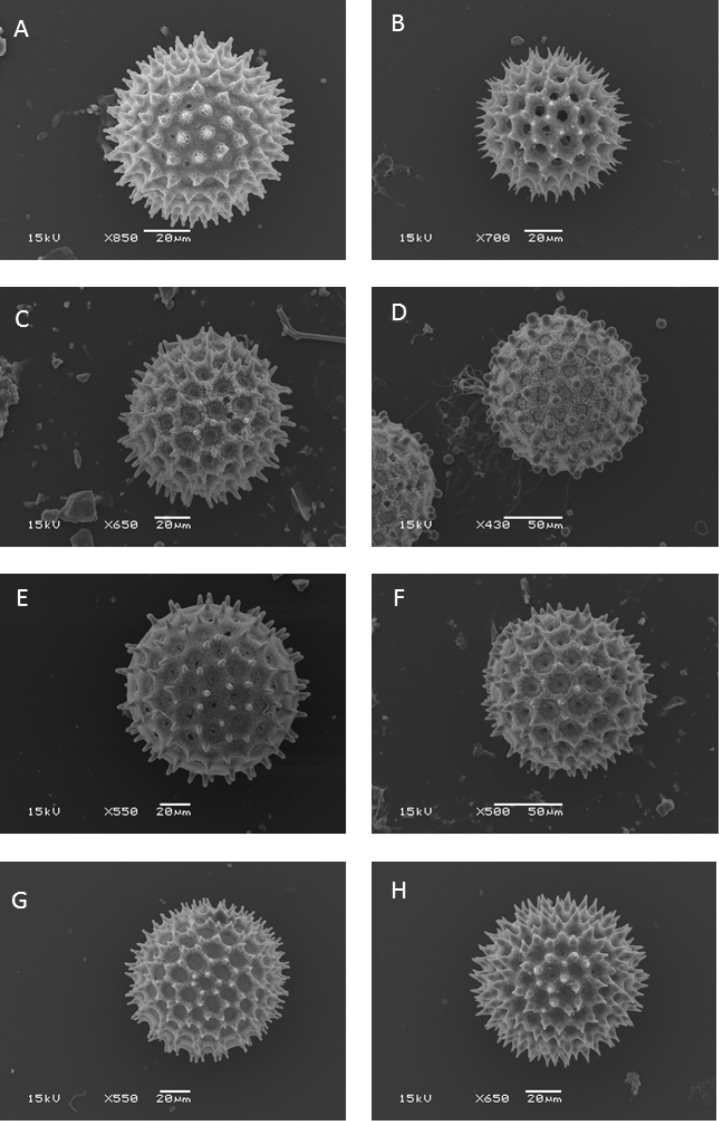
Pollen of *Ipomoea* species. **A***I. triloba* (*D’Arcy* 317) **B***I. cryptica* (*Steinbach* 6311) **C***I. purpurea* (*Parada & Rojas* 2664) **D***I. alba* (Wood et al. 27828) **E***I. dumosa* (*Hinton et al.* 9479) **F***I. hederifolia* (*Queiroz* 15975) **G***I. stans* (*Y. Mexia* 275112) **H***I. suffulta* (*Pringle* 4755). Photos by Robert Scotland.

Our own survey of *Ipomoea* pollen confirms these previous studies demonstrating continuous variation in pollen morphology with little, if any, discrete variation that correlates with phylogeny. The attempt by Wilkin (1993) to correlate results with a broader infrageneric classification of *Ipomoea* was made in the pre-molecular era and does not correspond closely with our molecular results. Nevertheless, although pollen in itself is of little phylogenetic or taxonomic value within *Ipomoea*, a few broad generalisations can be cautiously made. The pollen of species in Clade A (Figure 9, A–C) usually has fewer supratectal elements (spines) than the pollen of species in other clades. Pollen in Clades B and C (Figures 9D, E, 10C–H) often shows a regular pattern of 4–6 supratectal elements per pore as exemplified by Figure 10C and 10G but this is not always the case (Figure 10A, H). The pollen of *I. alba* (Figure 10D) and related species in the Calonyction Clade (species 271–274) have characteristic stout, rounded gemmiform spines rather than the usual acute spines, but similar blunt spines are also found in other more distantly related species such as *I. dumosa* (Figure 10E).

In summary, the pollen of *Ipomoea* is characterised by echinulate supratectal elements, showing a number of features that vary continuously and some specific morphologies that are homoplastic.

### Gynoecium

The style is elongate, equalling or extended slightly beyond the anthers and nearly always glabrous, even in species with a hirsute ovary. The only exception we are aware of is *Ipomoea sidifolia*, in which the hairs extend for a short distance upwards from the ovary. The style is usually included in the corolla but is exserted in species with a hypocrateriform corolla. The stigmas are characteristically biglobose, that is they are bilobed with each lobe globose and appearing fused. They sometimes appear simply globose. Triglobose stigmas are characteristic of the Pharbitis Clade but are not reported from all species in the clade. Somewhat elongate stigmas are reported from African species placed in Astripomoea Clade but also occur in three species of the Arborescens Clade: *I. pauciflora*, *I. populina* and *I. wolcottiana*.

The ovary is narrowly ovoid in shape and usually glabrous. A pubescent or comose ovary is rare and only commonly found in the Batatas Clade. Most species have a bilocular ovary with two ovules in each chamber. This correlates with a biglobose stigma. A few species (Pharbitis Clade) have a trilocular ovary each chamber with two ovules, this correlating with a trilobed stigma. In species of the Quamoclit Clade, in *Rivea*, *Stictocardia* and most species placed in *Argyreia*, the ovary is 4-locular but with a single ovule in each chamber. Very rarely other arrangements are noted. In *Ipomoea decasperma* (and *I. longituba* Hallier f. from Madagascar) the ovary is 5-locular with two ovules per chamber but it is not clear whether this is constant in all examples of these species. *Ipomoea gilana* is reported to have a trilocular ovary.

### Fruit

The fruit may be an indehiscent, woody or somewhat fleshy structure or formed by a dehiscent capsule. In species with an indehiscent fruit, this is usually globose to ellipsoid in shape and may contain up to four seeds except in those species placed in *Turbina* where 1–2 seeds only are present. Indehiscent fruits are glabrous but some species placed in *Argyreia* have mealy fruits. In those species with a capsular fruit, the capsules may be globose, ovoid or conical in shape. Capsules are usually muticous but species with a prominent rostrate apex formed by the persistent style base are common. Most capsules are completely glabrous but in a few species, they are pubescent, pilose or comose, this correlating with a hirsute ovary (*Ipomoea velutinifolia*, *I. dubia*, *I. sidifolia*, *I. dasycarpa*, many annual species of the Batatas Clade). In the majority of species the capsule is bilocular with up to four seeds, though often less as a result of abortion. There are several exceptions. In the Pharbitis Clade capsules are usually trilocular and 6-seeded. Very rarely capsules have up to 10 seeds (*I. decasperma*). In the Quamoclit Clade the capsules are 4-locular but with only four seeds.

**Figure 11.**
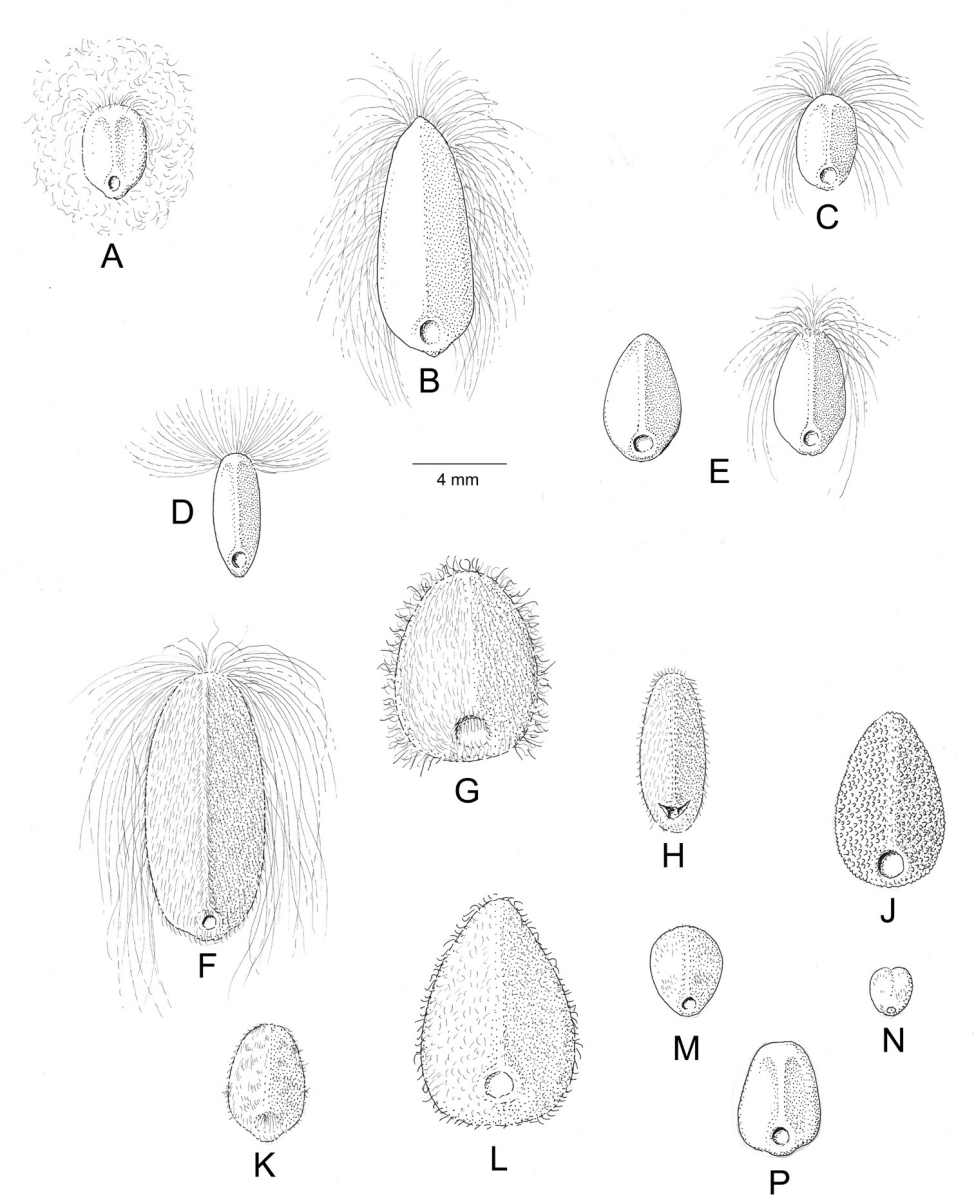
Seeds of *Ipomoea***A***I. peteri***B***I. murucoides***C***I. carolina***D***I. eggersiana***E***I. longibarbis* (with and without marginal hairs) **F***I. clavata***G***I. violacea***H***I. acanthocarpa***J***I. parvibracteolata***K***I. meyeri***L***I. jujuyensis***M***I. cholulensis***N***I. minutiflora***P***I. tiliacea*. **A** from *Wallnöfer & Tut-Tesucun* 9662; **B** from *Pringle* 6066; **C** from *Gillis* 12906; **D** from *Urote* 35; **E** from *Killeen et al.* 4199; **F** from *Fuentes & Miranda* 10895; **G** from *Stearn* 322; **H** from *Wurdack & Monachino* 39830; **J** from *Silva et al.* 18; **K** from *Smith* 1573; **L** from *Rose et al.* 23251; **M** from *Hinton* 11166; **N** from *Stevens & Montiel* 26592; **P** from *Curtiss* 249. Drawn by Rosemary Wise.

Seeds (Figure 11) are typically broadly oblong in outline and vary in size from species to species. Their colour (when ripe) can vary from black to varying shapes of brown, sometimes being distinctly reddish-brown. They can be completely glabrous, minutely covered in very short hairs (tomentellous), only visible under a microscope, pubescent, tomentose or, in many species, with prominent, usually white hairs which develop on the angles of the seeds, In a few cases the seeds are completely covered in matted woolly hairs (*Ipomoea bombycina*, *I. eremnobrocha*, *I. isthmica*, *I. macrorhiza*, *I. jalapa*). Although important in diagnosing species and species groups, the value of seeds as a taxonomic character is somewhat diminished by a number of factors. The seeds of many species are unknown; in some the marginal hairs are caducous so may appear absent (*I. psammophila*) and in others there may be more variability than can be demonstrated from the few fruiting specimens known (*I. jalapa*).

## Dichotomous keys

Keys are provided in a somewhat unconventional way and it is recommended that users follow the suggested steps in the order provided. Species in Steps 1–3 below also appear in the appropriate geographical keys. Note that species may enter several times in different places in the keys.

**Step I.** Does the plant fit any of the following distinct groups?

1. Plants of seashore (rarely inland in saline habitats): *Ipomoea pes-caprae* (pink flowers, retuse leaves), *I. violacea* (white to pale violet flowers, exserted stamens), *I. imperati* (white flowers, creeping herb), *I. littoralis* (Hawaii), *I. sagittata* (Caribbean and North American–sagittate lvs), *I. macrorhiza* (United States–white flowers, pubescent sepals).

2. Plants with a hirsute ovary and capsule: *Ipomoea sidifolia*, *I. dasycarpa*, *I. velutinifolia*, species in the Batatas Clade (page 387).

**Step II.** Is the plant one of the following very distinctive widespread common species?

An erect plant with ovate cordate leaves and pink flowers: 84b. I. carnea subsp. fistulosa.

A slender plant with pinnate leaves, pseudo-stipules and dark red corollas: 312. *I. quamoclit*.

A twining vine with pure white flowers, a narrowly cylindrical corolla tube and strongly awned sepals: 272. *I. alba*.

**Step III.** Does the plant belong to one of the following distinctive clades?

The Arborescens Clade (page 263). Trees, shrubs or lianas with white latex. Leaves entire. Sepals ovate or oblong, somewhat coriaceous. Corolla white, often with dark centre, glabrous or pubescent anthers included; seeds with long white marginal hairs.

The Batatas Clade (page 387) Annual or perennial herbs. Leaves entire or lobed. Sepals thin, often papery, usually distinctly mucronate. Corolla always glabrous, white or pink, often with a dark throat, often small and campanulate. Ovary and capsule often hirsute.

The Pharbitis Clade (page 430) Annual or perennial herbs, often hirsute. Leaves lobed or entire. Bracteoles often persistent. Sepals usually relatively large, usually with elongate, somewhat accrescent apex, sometimes leafy in texture. Corolla usually showy, pink, blue or violet, glabrous or (less commonly) pubescent. Stigma usually 3-lobed and ovary 3-locular. Capsule up to 6-seeded.

The Quamoclit Clade (page 556) Slender, twining usually annual, herbaceous herbs. Sepals characteristically awned, the awn subterminal on the abaxial surface, often equalling the sepal proper. Corolla red, orange or yellow, suburceolate or hypocrateriform, glabrous, stamens exserted or at least held at mouth of corolla. Ovary and capsule 4-locular.

**Step IV.** If your plant cannot be placed using Steps 1–3, go to the appropriate geographical key:

A. South American continent including the Galapagos Islands (page 54)

B. The North American Continent from Panama northwards (page 78)

C. The Caribbean Islands including Bermuda, Trinidad and the Netherlands Antilles (page 93)

D. Hawaii (page 99)

The two continental keys are divided into a series of subkeys to facilitate access as they would otherwise be very large. Some species can be accessed through different routes so individual species may occur in several subkeys.

### A. Keys to South American species

Key A1: Species with soft fleshy spines on the sepals and/or peduncles

Key A2: Species with erect stems

Key A3: Species with leaves divided digitately to, or near the base, into five or more lobes or segments

Key A4: Species with very long sepals, mostly exceeding 2 cm in length

Key A5: Species with coriaceous, convex, usually glabrous sepals

Key A6: Species with a subcylindrical corolla tube and (usually) exserted stamens

Key A7: Species with small flowers, the corolla < 3 cm long

Key A8: Plants with a glabrous white corolla > 3 cm long (check buds).

Key A9: Plants with subcapitate inflorescences

Key A10: Trailing, climbing or twining plants with a pubescent corolla > 3.5 cm long

**Key A1**

Species with soft fleshy spines on the sepals and/or peduncles (Figure 15B). Excluded are species where soft spines are only on the stem, such as *Ipomoea muricata* and *I. parasitica* as these teeth occur occasionally in other species such as *I. alba*.

**Table:** 

1	Leaves 3 (–5)-lobed	**2**
–	Leaves entire	**3**
2	Outer sepals 14–17 mm long, covered in long white hairs and soft spines; corolla white	**411. *I. altoamazonica***
	Outer sepals 8–10 mm long, glabrous or with soft spines; corolla pink	**216. *I. setosa***
3	Outer sepals 15–25 cm long; peduncles < 5 cm long; corolla white	**409. *I. echinocalyx***
	Outer sepals 12–14 cm long; peduncles 0.5–8 cm long; corolla pink	**408. *I. crinicalyx***

**Key A2**

Erect species. Perennial herbs or subshrubs growing in open habitats. Leaves subsessile (petioles usually < 1 cm), linear, lanceolate, ovate or oblong in shape, base attenuate or cuneate, rarely rounded, never cordate. Sepals various. Inflorescence usually terminal on the stem, often subspicate or subracemose in form but occasionally branched and arising from the upper leaf axils. Corolla shape and colour varied but never hypocrateriform (except *I. cavalcantei*) or suburceolate. Capsule and seeds varied.

**Table:** 

1	Corolla glabrous on the exterior	**2**
–	Corolla hirsute on the exterior at least in bud	**18**
2	Leaves divided nearly to the base into linear segments; sepals > 2 cm long	**13. *I. theodori***
–	Leaves entire or shallowly lobed	**3**
3	Sepals subequal, coriaceous, convex	**4**
–	Sepals equal or unequal, never coriaceous or convex	**7**
4	Leaves and stem glabrous	**5**
–	Leaves and stem hirsute	**6**
5	Herb; leaves linear, 1–3 mm wide	**169. *I. schomburgkii***
–	Subshrub; leaves oblong or oblanceolate, 5–25 mm wide	**155. *I. franciscana***
6	Leaves green, pubescent, imbricate, diminishing in size upwards; corolla weakly lobed	**168. *I. paulistana***
–	Leaves silvery-sericeous, especially below, not conspicuously imbricate or diminishing in size upwards; corolla lobed	**167. *I. argentea***
7	Sepals pubescent	**8**
–	Sepals glabrous	**11**
8	Corolla hypocrateriform, deep red; stamens exserted	**96. *I. cavalcantei***
–	Corolla funnel-shaped, pink; stamens included	**9**
9	Outer sepals 6–10 mm long; leaves pubescent beneath	**10**
–	Outer sepals 12–15 mm; leaves glabrescent beneath	**97. *I. marabaensis***
10	Leaves linear, 3–5 mm wide	**102. *I. neriifolia***
–	Leaves mostly oblong, 5–14 mm wide	**101. *I. queirozii***
11	Leaves pubescent beneath	**101. *I. queirozii***
–	Leaves glabrous	**12**
12	Stems conspicuously granulose	**368. *I. granulosa***
–	Stems smooth	**13**
13	Sepals subequal (Guianas and Amapá)	**385. *I. leprieurii***
–	Sepals markedly unequal	**14**
14	Sepals abaxially muricate	**15**
–	Sepals abaxially smooth	**16**
15	Leaves oblong or ovate; plant only woody basally	**345. *I. procurrens***
–	Leaves oblong-elliptic to suborbicular; woody subshrub	**344. *I. coriacea***
16	Outer sepals 7–11 mm long	**367. *I. rupestris***
–	Outer sepals 2–6 mm long	**17**
17	Leaves linear, < 3 mm wide	**364. *I. pinifolia***
–	Leaves oblong, > 5 mm wide	**363. *I. squamisepala***
18	Leaves all entire	**19**
–	Leaves 3–5-lobed	**43**
19	Leaves linear to very narrowly oblong; inflorescence clearly terminal (*I. campestris* might key out here but inflorescence is axillary)	**20**
–	Leaves oblong or ovate, > 5 mm wide; inflorescence clearly terminal only or with flowers also in the leaf axils	**23**
20	Leaves 16–27 cm long, coarsely tomentose	**6. *I. aemilii***
–	Leaves 1.5–12 cm long, variously hirsute but not coarsely tomentose	**21**
21	Leaves acute, mucronate (widespread, cerrados)	**47. *I. aprica***
–	Leaves obtuse, prominently mucronate	**22**
22	Leaves with 3 prominent longitudinal veins, abaxially floccose (Paraguay)	**49. *I. oblongifolia***
–	Leaves with a single longitudinal vein, abaxially puberulent to subsericeous (Brazil)	**48. *I. uninervis***
23	Inflorescence of unbranched terminal spikes or poorly differentiated cymose clusters	**24**
–	Inflorescence clearly branched, the lower part clearly cymose in structure, sometimes appearing paniculate	**38**
24	Leaves elliptic or ovate, up to three times as long as broad	**25**
–	Leaves oblong, lanceolate or oblanceolate, at least three times as long as broad	**30**
25	Pedicels absent or very short so bracteoles immediately below calyx; peduncles 2.5–5 cm long	**50. *I. guaranitica***
–	Pedicels 2–7 mm long, bracteoles arising at least 5 mm below calyx; peduncles	**26**
26	Sepals 6–8 (–10) mm long; flowers in cymes, rarely solitary	**27**
–	Sepals 9–15 mm long; flowers usually solitary	**29**
27	Abaxial leaf surface and outer sepals densely silvery-tomentose; corolla pink (Paraguay)	**55. *I. paraguariensis***
–	Abaxial leaf surface and outer sepals pubescent but not densely silvery-tomentose; corolla white or pink	**28**
28	Corolla white or pale pink; leaves 6 × 3.5 cm; plant ±herbaceous	**33. *I. cerradoensis***
–	Corolla pink; leaves up to 15.5 × 7 cm; plant distinctly shrubby	**34. *I. sp*** . **B**
29	Peduncles very short; leaves with white “highlighted” ciliolate margins (Amambay, Paraguay)	**54. *I. estrellensis***
–	Peduncles 0.8–4 cm; leaves without distinct white margins (Cordillera, Paraguay)	**8. *I. cordillerae***
30	Plant inconspicuously hirsute, often appearing glabrous except when using a hand lens	**31**
–	Plant conspicuously hirsute	**32**
31	Plant usually > 50 cm in height; flowers in compact cymes, rarely solitary; wet places in Argentina, Paraguay and the Pantanal	**9. *I. paludosa***
–	Plant usually < 30 cm high; flowers mostly solitary; dry places in the Brazilian cerrados	**35. *I. campestris***
32	Bracts ±equalling leaves, nearly concealing flowers; leaves and bracts imbricate	**103. *I. pohlii***
–	Flowers not concealed by bracts; leaves and bracts not imbricate, or, if somewhat imbricate, flowers and calyx clearly visible	**33**
33	Inflorescence elongate, up to 30 cm in length; leaves tomentose on both surfaces (Amambay, Paraguay)	**53. *I. rojasii***
–	Inflorescence nor elongate, usually < 10 cm long; leaves not tomentose on both surfaces	**34**
34	Outer sepals mostly 15–20 × 5–7 mm, often somewhat foliose, much larger than inner sepals	**83. *I. burchellii***
–	Outer sepals < 16 × 4 mm, usually much less, not conspicuously unequal	**35**
35	Sepals acute to acuminate	**36**
–	Sepals obtuse	**37**
36	Inflorescence very compact, clustered at apex of stem; sepals 8–11 mm long (Sierra de Pireneus in Brazil)	**31. *I. pyrenea***
–	Flowers not clustered at stem apex; sepals 12–16 mm long (widespread in cerrado)	**29. *I. hirsutissima***
37	Leaves lanceolate	**30. *I. aurifolia***
–	Leaves oblong	**32. *I. subspicata***
38	Leaves abaxially white, appressed tomentellous	**38. *I. argyreia***
–	Leaves greyish, usually tomentose with spreading hairs	**39**
39	Leaves oblanceolate to obovate, widest above the middle	**40**
–	Leaves ovate, oblong elliptic or oblong, widest in the middle	**41**
40	Leaves mostly < 2 cm wide, densely pubescent adaxially; inflorescence simple, side branches absent or very short	**39. *I. cuneifolia***
–	Leaves mostly 2–4 cm wide, thinly pilose to glabrous, adaxially; inflorescence with long side branches below	**40. *I. haenkeana***
41	Leaves slightly longer than broad, adaxially much less hirsute than abaxially	**41. *I. virgata***
–	Leaves 3 or more times longer than broad, both surfaces equally hirsute	**42**
42	Sepals acute, 10–12 mm long; ovary and capsule glabrous	**42. *I. verbasciformis***
–	Sepals acuminate, submucronate, ±15 mm long; ovary and capsule comose	**43. *I. dasycarpa***
43	Leaves divided to near the base into linear segments, all or most less than 3 mm wide	**44**
–	Leaves shallowly lobed or, if lobed to near the base, segments oblong, not linear	**46**
44	All leaf segments < 5 cm long	**45**
–	Some or all leaf segments 5–7 cm long	**15. *I. itapuaensis***
45	Sepals 5–8 mm, obtuse to rounded; inflorescence usually terminal and cymose in form	**17. *I. angustissima***
–	Sepals 9–11 mm, acute; inflorescence axillary; flowers solitary in the leaf axils	**16. *I. fiebrigii***
46	Leaves shallowly lobed, often with some entire leaves	**47**
–	Leaves deeply lobed into oblong segments	**49**
47	Plant roughly hirsute with long spreading hairs; flowers solitary; corolla very large, > 9 cm long	**28. *I. megalantha***
–	Plant pubescent to subglabrous, hairs appressed; flowers usually in cymes; corolla < 6.5 cm long	**48**
48	Lower leaves entire, upper leaves usually 3-lobed	**10. *I. morongii***
–	All leaves divided into 3–5 lobes	**11. *I. malvaeoides***
49	Inflorescence terminal, formed of few-flowered cymes	**7. *I. malpighipila***
–	Inflorescence of solitary axillary flowers, these occasionally in axillary cymes	**50**
50	Corollas 6–9 cm long	**51**
–	Corollas 5–6 cm long	**11. *I. malvaeoides***
51	Sepals obtuse, mucronate; inner sepals 11–16 mm long	**14. *I*. sp. A**
–	Sepals acute; inner sepals 8–11 mm long	**12. *I. pseudomalvaeoides***

**Key A3**

Digitate-leaved species with leaves divided to or near the base into 5 or more segments. Excluded are species with all or most leaves 3-lobed or divided to halfway or less.

**Table:** 

1	Corolla up to 3 cm long; plants slender annuals or perennials	**2**
–	Corolla 3.5–9 cm long; plants perennial	**8**
2	Corolla 1–1.2 cm long; sepals apiculate; introduced weed in dry areas of Venezuela.........	**328. *I. costellata***
–	Corolla 1.7–3 cm long; sepals not apiculate	**3**
3	Perennials from a bulb-like corm; sepals muricate, scarious margined (high altitude Andean species)	**4**
–	Annual or perennial lowland herbs lacking a corm-like rootstock; sepals neither muricate, nor prominently scarious-margined; plants not usually occurring above 2500 m	**5**
4	Leaves imbricate, the segments filiform; sepals outer sepals 4–5 mm long; plant usually erect	**288. *I. capillacea***
–	Leaves scarcely imbricate, the segments linear 1–3 mm wide; outer sepals 5.5–7 mm long; plant usually decumbent to ascending	**287. *I. plummerae***
5	Peduncle coiled or at least twisted; leaflets all arising from the same origin	**6**
–	Peduncle straight or nearly so; leaflets pedate or some forked	**7**
6	Sepal base abruptly truncate, margin fimbriate below	**373. *I. tenera***
–	Sepal base, rounded, margin entire, not fimbriate	**374. *I. heptaphylla***
7	Corolla yellow with violet centre; sepals > 7 mm long, acuminate; dry habitats	**383. *I. longeramosa***
–	Corolla pink; sepals 3–3.5 mm, obtuse; wetlands in Venezuela and Colombia	**280. *I. pittieri***
8	Sepals with a prominent appendage on the abaxial surface (NE Brazil)	**90. *I. rosea***
–	Sepals lacking an appendage on the abaxial surface	**9**
9	Leaf petioles with conspicuous pseudo-stipules	**392. *I. cairica***
–	Leaf petioles clearly lacking pseudo-stipules	**10**
10	Leaf segments linear to oblong, ±parallel-sided, mostly < 5 mm wide	**11**
–	Leaf segments elliptic, ovate or obovate, clearly not parallel-sided	**23**
11	Corolla glabrous	**12**
–	Corolla pubescent	**16**
12	Sepals > 1.5 cm long	**13**
–	Sepals 0.5–1 cm long	**14**
13	Sepals truncate at base; slender herb, variable in habit but never erect	**377. *I. pantanalensis***
–	Sepals narrowed at base; erect herb	**13. *I. theodori***
14	Sepals obovate suborbicular, about as long as broad	**156. *I. platensis***
–	Sepals ovate or oblong, twice as long as broad	**15**
15	Sepals ovate, apiculate, 5–6 mm long (stream sides)	**378. *I. subrevoluta***
–	Sepals oblong, rounded, rounded (granite domes)	**89. *I. graniticola***
16	Twining plant	**18. *I. revoluta***
–	Erect or ascending herbs	**17**
17	All or most leaf segments less than 3 mm wide	**18**
–	All or most leaf segments oblong, not linear	**20**
18	All leaf segments < 5 cm long	**19**
–	Some or all segments 5–7 cm long	**15. *I. itapuaensis***
19	Sepals 5–8 mm, obtuse to rounded; inflorescence usually terminal and cymose in form	**17. *I. angustissima***
–	Sepals 9–11 mm, acute; inflorescence axillary; flowers solitary in the leaf axils	**16. *I. fiebrigii***
20	Inflorescence terminal, formed of few-flowered cymes	**7. *I. malpighipila***
–	Inflorescence of solitary axillary flowers, these occasionally in axillary cymes	**21**
21	Corolla 6–9 cm long	**22**
–	Corolla 5–6 cm long	**11. *I. malvaeoides***
22	Sepals obtuse, mucronate; inner sepals 11–16 mm long	**14. *I*. sp. A**
–	Sepals acute; inner sepals 8–11 mm long	**12. *I. pseudomalvaeoides***
23	Sepals > 20 cm long, bracteoles large, persistent, often concealing the calyx	**107. *I. gigantea***
–	Sepals < 1.5 cm, bracteoles small, caducous, never concealing the calyx	**24**
24	Corolla and sepals glabrous	**25**
–	Corolla and sepals pubescent	**29**
25	Sepals papery, flat, subacute to mucronate	**95. *I. killipiana***
–	Sepals coriaceous, convex, rounded	**26**
26	Inflorescence of compound, many-flowered axillary cymes, 10–30 cm in length (Peru)	**158. *I. maranyonensis***
–	Inflorescence of simple or doubled axillary cymes, 10 cm long	**27**
27	Leaf lobes linear-oblong	**156. *I. platensis***
–	Leaf lobes (oblong-)elliptic	**28**
28	Leaves large, 5–14 × 6–16 cm (wetlands in tropical lowlands)	**157. *I. mauritiana***
–	Leaves relatively small, mostly 4–6 × 5–7 cm (mostly dry habitats in the inter-Andean valleys and the Chaco lowlands)	**159. *I. cheirophylla***
29	Leaves digitately lobed to base	**3. *I. pampeana***
–	Leaves not digitately divided to base	**30**
30	Corolla almost glabrous; leaves 6–9-palmatisect with elliptic to oblanceolate lobes	**1. *I. stuckertii***
–	Corolla conspicuously pubescent; leaves 3–5-palmatilobed with ovate lobes	**2. *I. padillae***

**Key A4**

Species with very long sepals, mostly exceeding 2 cm in length

**Table:** 

1	Corolla pure white, the tube narrowly cylindrical, sepals with a long terminal awn	**272. *I. alba***
–	Corolla pink, blue, or yellowish with a coloured tube, tube not cylindrical; sepals not awned	**2**
2	Leaves lobed or divided into segments	**3**
–	Leaves entire, ovate, cordate	**6**
3	Leaf divided into linear-filiform segments; erect plant (Paraguay)	**13. *I. theodori***
–	Leaf segments or lobes broad; trailing or climbing plant	**4**
4	Leaf divided into 5–10 oblong segments (Brazil)	**107. *I. gigantea***
–	Leaf lobed, not divided into separate segments	**5**
5	Leaves and sepals glabrous; corolla purple (Cusco area, Peru)	**402. *I. cuscoensis***
–	Leaves and sepals hirsute; corolla blue when fresh	**236. *I. nil***
6	Corolla pubescent on the exterior	**7**
–	Corolla glabrous on the exterior	**9**
7	Corolla pink; pedicels very short, < 10 mm long; bracteoles relatively persistent	**8**
–	Corolla yellowish with purple tube; pedicels 10–25 mm; bracteoles short, caducous (Venezuela)	**109. *I. yaracuyensis***
8	Peduncles 3–5 cm long; corolla 6–7 cm long (Bolivia and Brazil)	**98. *I. calyptrata***
–	Peduncles < 1.2 cm; corolla 12 cm long (Peru)	**113. *I. nivea***
9	Sepals obovate to suborbicular; stamens exserted from corolla	**269. *I. mirandina***
–	Sepals lanceolate or oblong, much longer than broad; stamens included in corolla	**10**
10	Leaves sagittate with acute auricles; sepals with prominent longitudinal vein	**355. *I. incarnata***
–	Leaves cordate with rounded auricles; sepals lacking prominent longitudinal veins	**11**
11	Leaves pubescent (Brazil)	**405. *I. daturiflora***
–	Leaves glabrous	**12**
12	Sepals very unequal in size	**360. *I. paranaensis***
–	Sepals equal or nearly so	**13**
13	Flowers solitary (rarely paired); stem with scattered long spreading white hairs; corolla pale blue	**401. *I. clavata***
–	Inflorescence a cyme of up to 7 flowers; stem glabrous; corolla pale lilac	**217. *I. peruviana***

**Key A5**

Species with coriaceous sepals. Perennial erect, trailing or twining herbs or woody lianas, erect, trailing or twining, stellate hairs sometimes present. Leaves lobed or entire. Sepals coriaceous, convex, subequal, usually glabrous but sometimes indumentum from pedicels extends onto lower half of outer sepals. Corolla glabrous (except *I. discolor*), funnel-shaped with included stamens or hypocrateriform or suburceolate with exserted stamens. Capsule 4-seeded, seeds commonly with prominent, long marginal hairs.

**Table:** 

1	Corolla pubescent on the exterior (Venezuela and Guianas)	**172. *I. discolor***
–	Corolla glabrous on the exterior	**2**
2	Stellate (branched) hairs present on leaves and stem	**3**
–	Hairs all unbranched	**6**
3	Stellate hairs conspicuous, unbranched hairs absent or very few	**4**
–	Stellate hairs inconspicuous, mixed with and partly concealed by unbranched hairs	**5**
4	Stellate hairs with long branches 0.5–1.5 mm long	**163. *I. homotrichoidea***
–	Stellate hairs with short branches <0.5 mm long	**162. *I. bonariensis***
5	Corolla funnel-shaped; stamens included	**164. *I. oranensis***
–	Corolla hypocrateriform; stamens exserted	**165. *I. exserta***
6	Stems erect; petioles < 1 cm long; leaves linear, oblong or obovate	**7**
–	Stems twining or trailing; petioles > 1 cm long; leaves varied but if oblong, plant a liana	**10**
7	Leaves and stem glabrous	**8**
–	Leaves and stem hirsute	**9**
8	Herb; leaves linear, 1–3 mm wide	**169. *I. schomburgkii***
–	Subshrub; leaves oblong or oblanceolate, 5–25 mm wide	**155. *I. franciscana***
9	Leaves green, pubescent, imbricate, diminishing in size upwards; corolla weakly lobed	**168. *I. paulistana***
–	Leaves silvery-sericeous, especially below, not conspicuously imbricate or diminishing in size upwards; corolla lobed	**167. *I. argentea***
10	Leaves 5–7-lobed to near base; vigorous cultivated liana of tropical gardens	**211. *I. horsfalliae***
–	Leaves entire or lobed, but, if lobed, not lobed to near base or plant herbaceous; naturally growing herbaceous or woody climbers	**11**
11	Corolla suburceolate or hypocrateriform with a relatively narrow tube, sometimes leafless at anthesis; stamens exserted	**12**
–	Corolla funnel-shaped, leaves present at anthesis; stamens included	**16**
12	Stems and leaves glabrous	**13**
–	Stems and leaves hirsute	**15**
13	Leaves dimorphic, commonly 3-lobed, often absent at anthesis; corolla limb with ovate lobes up to 5 mm long; stem often warted (Venezuela)	**171. *I. verruculosa***
–	Leaves entire, uniform in shape, present at anthesis; corolla limb very short, the lobes < 3 mm long; stem not warted	**14**
14	Leaves oblong-elliptic, < 2.5 cm wide (Brazil)	**153. *I. ana-mariae***
–	Leaves ovate, 4–8 cm wide (Bolivia)	**150. *I. suburceolata***
15	Leaves white canescent on both surfaces, usually absent at anthesis (Brazil)	**154. *I. longistaminea***
–	Leaves adaxially green, present or absent at anthesis (Bolivia)	**165. *I. exserta***
16	Leaves all conspicuously 3–7-lobed	**17**
–	Leaves entire or occasionally with a few leaves shallowly lobed	**23**
17	Leaves abaxially densely silvery sericeous; corolla campanulate, < 2.5 cm long, white	**176. *I. eremnobrocha***
–	Leaves abaxially glabrous or thinly pubescent; corolla funnel-shaped > 4 cm long, pink	**18**
18	All or most leaves 5–7-lobed	**19**
–	All or most leaves 3-lobed	**22**
19	Leaf lobes linear-oblong, not widest in the middle	**156. *I. platensis***
–	Leaf lobes (oblong-)elliptic, widest in the middle	**20**
20	Inflorescence of compound, many-flowered axillary cymes, 10–30 cm in length	**158. *I. maranyonensis***
–	Inflorescence of simple or doubled axillary cymes, 10 cm long	**21**
21	Leaves large, 5–14 × 6–16 cm; humid tropical lowlands	**157. *I. mauritiana***
–	Leaves relatively small, mostly 4–6 × 5–7 cm; mostly dry habitats in the inter-Andean valleys and the Chaco lowlands	**159. *I. cheirophylla***
22	Leaves glabrous	**160. *I. blanchetii***
–	Leaves pubescent	**161. *I. caloneura***
23	Inflorescence with large persistent bracteoles which conceal calyx and capsule	**170. *I. densibracteata***
–	Bracteoles small, caducous or briefly persistent, never concealing calyx and capsules	**24**
24	Peduncles and pedicels very short, < 7 mm long	**148. *I. goyazensis***
–	Peduncles and/or pedicels at least 1 cm long, usually much more	**25**
25	Leaves glabrous	**26**
–	Leaves hirsute at least beneath	**31**
26	Leaves oblong-ovate to oblong-obovate, base cuneate to weakly cordate; woody lianas of dry country	**27**
–	Leaves broadly lanceolate to ovate, base truncate to cordate; plants of relatively moist areas, stems not obviously woody	**29**
27	Leaves oblong-ovate, base truncate to subcordate (Argentina and Bolivia)	**149. *I. schulziana***
–	Leaves oblong-elliptic to obovate, base cuneate to attenuate, 0.7–2.5 cm wide (Brazil)	**28**
28	Leaves with 4–5 pairs of veins, apex rounded to emarginate	**152. *I. serrana***
–	Leaves with 9–12 pairs of veins, apex acute to obtuse	**151. *I. pintoi***
29	Leaves 10–22 × 9–16 cm, commonly with a distinct angle or tooth on the margin; sepals 9–12 mm long (Southern Brazil)	**147. *I. austrobrasiliensis***
–	Leaves mostly < 14 × 10 cm long, lacking a distinct marginal angle or tooth; sepals usually < 9 mm long	**30**
30	Widespread species of lowland forest; leaves ovate, usually entire	**145. *I. batatoides***
–	Andean species; leaves subdeltoid, often shallowly 3-lobed	**146. *I. volcanensis***
31	Stem and leaves with stiff spreading bulbous white hairs	**142. *I. pogonocalyx***
–	Stem and leaves variously hirsute but never as above	**32**
32	Leaf base broadly cuneate, leaves oblong-ovate	**143. *I. sp*** . **C**
–	Leaf base cordate; leaves ovate, sometimes lobed	**33**
33	Bracteoles caducous	**34**
–	Bracteoles persistent (Amazonia)	**166. *I. asplundii***
34	Lowland species; indumentum usually sparse, stellate hairs absent	**145. *I. batatoides***
–	Andean species (Bolivia and Argentina); indumentum dense with some stellate hairs	**164. *I. oranensis***

**Key A6**

Species with a subcylindrical corolla tube and exserted stamens. Perennial or annual herbs of varying habit and leaf shape. Corolla subcylindrical, the tube scarcely widened upwards; stamens exserted.

**Table:** 

1	Corolla white or white flushed very pale blue	**2**
–	Corolla variously coloured but never white or white flushed bluish	**6**
2	Corolla suburceolate, the limb very short	**423. *I. scopulina***
–	Corolla hypocrateriform with a conspicuous limb	**3**
3	Sepals obtuse; peduncle very long, 22–40 cm	**82. *I. marcellia***
–	Sepals awned or mucronate; peduncles < 20 cm long	**4**
4	Outer sepals with long awns 5–12 mm long; stems often with fleshy spines	**272. *I. alba***
–	Outer sepals mucronate but lacking long awns; stems lacking fleshy spines	**5**
5	Leaves lanceolate, base rounded to cuneate (Galapagos Islands)	**390. *I. habeliana***
–	Leaves ovate or suborbicular, cordate (widespread on coasts)	**389. *I. violacea***
6	Sepals terminating in a distinct awn; corolla bright red, yellow or orange; plants annual, slender	**Go to Key to *Quamoclit* clade (page 556)**
–	Sepals obtuse or acute, sometimes mucronate, but the mucro < 1 mm long; corolla dark red, pink or purple; perennial herbs, lianas or subshrubs	**7**
7	Corolla pubescent at least on the exterior	**8**
–	Corolla glabrous on theexterior	**9**
8	Sepals unequal, the inner 16–17 mm long; ovary and capsule hirsute	**404. *I. sidifolia***
–	Sepals subequal, 10–12 mm long; ovary (and presumably capsule) glabrous	**96. *I. cavalcantei***
9	Leaves lanceolate, up to 1 cm wide (Peru)	**425. *I* . sp. D** .
–	Leaves of varied shape, usually ovate, at least 1.5 cm wide	**10**
10	Sepals coriaceous, convex; glabrous (Key A5)	**11**
–	Sepals varied but nor coriaceous or convex, glabrous or pubescent	**14**
11	Leaves glabrous	**12**
–	Leaves densely hirsute, especially abaxially	**13**
12	Leaves oblong-elliptic, < 2.5 cm wide (Brazil)	**153. *I. ana-mariae***
–	Leaves ovate, 4–8 cm wide (Bolivia)	**150. *I. suburceolata***
13	Leaves white canescent on both surfaces, usually absent at anthesis (Brazil)	**154. *I. longistaminea***
–	Leaves adaxially green, usually present at anthesis (Bolivia)	**165. *I. exserta***
14	Sepals obovate, 1.8–2.5 cm long (Venezuela)	**269. *I. mirandina***
–	Sepals < 11 mm long, ovate, oblong or lanceolate	**15**
15	Corolla tube < 3.5 cm long (Peru and Ecuador)	**16**
–	Corolla tube > 3.5 cm long (Colombia, Venezuela and Brazil)	**17**
16	Sepals very unequal, the outer 3–4 mm long	**310. *I. alexandrae***
–	Sepals subequal 9–10 mm long	**309. *I. nationis***
17	Sepals obtuse to rounded; leaves commonly lobed; stems usually warted (Venezuela)	**171. *I. verruculosa***
–	Sepals acute, usually mucronate; leaves always unlobed; stems not warted	**18**
18	Petioles < 2.2 cm; sepals unequal (Brazil)	**291. *I. dumosa***
–	Petioles > 4 cm; sepals subequal (Venezuela and Colombia)	**265. *I. retropilosa***

**Key A7**

Species with small flowers, the corolla < 3 cm long

**Table:** 

1	Leaves divided to base into 5 or more digitate segments	**2**
–	Leaves entire, or, if divided, only 3-lobed, the lateral lobes sometimes forked	**8**
2	Corolla 1–1.2 cm long; sepals apiculate; introduced weed of dry areas in Venezuela	**328. *I. costellata***
–	Corolla 1.7–3 cm long; Sepals various (apiculate only in *I. longeramosa*)	**3**
3	Perennials from a bulb-like corm; Sepals muricate, scarious-margined (High altitude Andean species)	**4**
–	Annual or perennial lowland herbs lacking a corm-like rootstock; sepals neither muricate, nor prominently scarious-margined	**5**
4	Leaves imbricate, the segments filiform; outer sepals 4–5 mm; plant usually erect	**288. *I. capillacea***
–	Leaves scarcely imbricate, the segments linear 1–3 mm wide; outer sepals 5.5–7 mm; plant usually decumbent to ascending	**287. *I. plummerae***
5	Peduncle coiled or at least twisted; leaflets all arising from the same origin	**6**
–	Peduncle straight or nearly so; leaflets pedate or some forked	**7**
6	Sepal base abruptly truncate, margin fimbriate below	**373. *I. tenera***
–	Sepal base, rounded, margin entire, not fimbriate	**374. *I. heptaphylla***
7	Corolla pale yellow with violet centre; sepals > 7 mm long, acuminate, apiculate; dry habitats	**383. *I. longeramosa***
–	Corolla pink; sepals 3–3.5 mm, obtuse; wetlands in Venezuela and Colombia	**280. *I. pittieri***
8	Corolla pubescent on the exterior	**9**
–	Corolla glabrous on the exterior	**10**
9	Flowers arranged in dense heads surrounded by persistent bracteoles	**244. *I. neurocephala***
–	Flowers not in dense bracteate heads	**311. *I. velardei***
10	Flowers in bracteolate clusters, the bracteoles 7–25 mm long, persistent	**305. *I. meyeri***
–	Inflorescence clearly cymose or flowers solitary; bracteoles inconspicuous, often caducous	**11**
11	Corolla white, cream or yellowish, sometimes with a dark centre	**12**
–	Corolla pink	**18**
12	Leaves 3-lobed	**13**
–	Leaves entire	**14**
13	Outer sepals 13–20 mm, ovate, basally cordate and auriculate; flowers usually solitary	**375. *I. macedoi***
–	Outer sepals 4–5 mm, oblong, basally cuneate; inflorescence of condensed axillary cymes	**176. *I. eremnobrocha***
14	Corolla c. 0.5 cm long; sepals 2–3 mm long	**336. *I. minutiflora***
–	Corolla > 1.5 cm long; sepals 5–7 mm long	**15**
15	Sepals oblong, > 10 mm long	**403. *I. corymbosa***
–	Sepals ovate or elliptic, < 10 mm long	**16**
16	Sepals white-margined; capsule strongly rostrate; cymes congested, the pedicels < 5 mm long	**382. *I. acanthocarpa***
–	Sepals not white-margined; capsule muticous; cymes lax, the pedicels 5–15 mm long	**17**
17	Annual herb; sepals ovate, acute, often mucronate; corolla 1.5–2.5 cm long (Caribbean)	**413. *I. obscura***
–	Perennial herb: sepals elliptic, obtuse; corolla 2.3–3.5 cm long (moist forest, often Andean)	**87. *I. reticulata***
18	Leaves 3-lobed with the two laterals forked (Brazil)	**384. *I. kraholandica***
–	Leaves entire or 3-lobed but, if 3-lobed, the laterals undivided	**19**
19	Low Andean herb; leaves cuneate, entire, bi- or trilobed	**287. *I. plummerae***
–	Twining herbs; leaves ovate, cordate or 3-lobed	**20**
20	Whole plant softly grey-canescent (Bolivia near Brazil)	**387. *I. deminuta***
–	Plant glabrous or pubescent, but never grey-canescent/tomentellous	**21**
21	Subshrub with somewhat succulent leaves; plant completely glabrous	**340. *I. amnicola***
–	Slender herbs, not succulent; plants glabrous or variously hirsute	**22**
22	Sepals with dark blotches on abaxial surface	**281. *I. dumetorum***
–	Sepals lacking dark blotches on abaxial surface	**23**
23	Peduncle passing through sinus of leaf base; sepals 3–5 mm long, corolla blue when fresh	**298. *I. aristolochiifolia***
–	Peduncle not passing through sinus of leaf base; sepals mostly more than 5 mm long, but, if less, corolla pink when fresh	**24**
24	Sepals acute, not mucronate or aristate, lanceolate-deltoid; corolla, when fresh, blue with white throat	**255. *I. marginisepala***
–	Sepals variously shaped (but never lanceolate-deltoid), always mucronate; corolla pink or pink with a dark throat	**25**
25	Flowers solitary (rarely paired); sepals ovate, gradually narrowed to an aristate point	**26**
–	Flowers usually several in axillary cymes; sepals variously shaped but not gradually narrowed to an aristate point	**28**
26	Completely glabrous trailing herb (Colombia)	**357. *I. colombiana***
–	Stem, leaves and/or sepals variously hirsute (Bolivia and Brazil)	**27**
27	Leaves entire; stem glabrous; capsule rostrate	**406. *I. chiquitensis***
–	Leaves commonly lobed; stem pilose; capsule acute, not rostrate	**407. *I. melancholica***
28	Capsule strongly rostrate; seeds pilose; sepals thick in texture with white margins; leaf auricles commonly acute	**382. *I. acanthocarpa***
–	Capsule muticious, style rarely persistent; sepals thin in texture, lacking white margins; leaf auricles usually rounded (Batatas Clade)	**29**
29	Outer sepals broadly oblong-elliptic, usually glabrous; capsule glabrous or hirsute	**30**
–	Outer sepals lanceolate or ovate, usually hirsute; capsule usually hirsute	**31**
30	Ovary and capsule glabrous; capsule compressed	**230. *I. ramosissima***
–	Ovary and capsule pubescent; capsule conical	**231. *I. cynanchifolia***
31	Corolla < 1.8 cm long	**229. *I. triloba***
–	Corolla 2–2.5 cm long	**228. *I. grandifolia***

**Key A8**

Plants with a white or yellow glabrous corolla. Included are tree-like shrubs or lianas with white flowers and dark purplish or pinkish centres, which have not been keyed out earlier.

If sepals with fleshy spines go to Key A1.

If corolla with cylindrical tube and exserted stamens: Go to Key A6.

If corolla < 3 cm long go to Key A7.

If an erect plant with sessile/subsessile leaves go to Key A2.

If plant with coriaceous, convex sepals, go to Key A5.

**Table:** 

1	Sepals very unequal, the outer conspicuously shorter than the inner sepals	**2**
–	Sepals equal or only slightly unequal	**5**
2	Stems trailing, often rooting at the nodes	**3**
–	Stems twining or clambering over vegetation or arborescent	**4**
3	Leaves linear or oblong, rectangular or 5-lobed (coastal)	**388. *I. imperati***
–	Leaves ovate or subreniform	**347. *I. asarifolia***
4	Leaves tomentellous to tomentose; outer sepals 10–12 mm long	**94. *I. sulina***
–	Leaves usually glabrous; outermost sepal <3 mm long	**381. *I. anisomeres***
5	Small trees	**6**
–	Lianas or perennial somewhat woody climbers	**7**
6	Leaves and sepals completely glabrous	**117. *I. pauciflora***
–	Abaxial leaf surface and sepals thinly pubescent	**119. *I. wolcottiana***
7	Liana leafless at anthesis, flowers borne towards the apex of slender branches, many metres high	**116. *I. juliagutierreziae***
–	Plant with leaves present at anthesis, the flowers borne in axillary cymes, corymbs or racemes	**8**
8	Corolla large, 9–12 cm long	**9**
–	Corolla < 6.5 cm long	**10**
9	Corolla white with dark pinkish-purple centre; leaves abaxially greyish or whitish with prominent reticulate venation	**106. *I. paradae***
–	Corolla pure white or with a very pale pink centre; leaves abaxially pale green, not conspicuously reticulate-veined	**85. *I. inaccessa***
10	Peduncles short, < 1.5 cm long so inflorescence appearing compact	**11**
–	Peduncles 1.5–8 cm long	**12**
11	Sepals densely pubescent; pedicels 3–5 mm long	**110. *I. chrysocalyx***
–	Sepals usually glabrous; pedicels 7–27 mm long	**400. *I. lindenii***
12	Bracteoles 2–3 cm long, persistent, asperous-pilose	**105. *I. longibracteolata***
–	Bracteoles < 1.5 cm long, usually caducous, glabrous	**13**
13	Corolla 2.5–3.5 cm long	**14**
–	Corolla 3.5–5 cm long	**15**
14	Sepals 10–14 mm long, spreading in fruit	**403. *I. corymbosa***
–	Sepals 5–7 mm long, not spreading in fruit	**87. *I. reticulata***
15	Sepals obtuse or rounded, not mucronate; inflorescence commonly compound	16 Sepals mucronate or very acute; inflorescence of simple axillary cymes	**17**
16	Pedicels very long, 1.5–2.5 cm; corolla campanulate, pendulous; plant glabrous	**371. *I. syringiifolia***
–	Pedicels mostly less than 1.5 cm long; corolla funnel-shaped, not pendulous; plant glabrous or hirsute	**86. *I. saopaulista***
17	Slender annual herb with yellow flowers, usually somewhat hirsute at least abaxially on the leaves; white latex absent	**412. *I. ochracea***
–	Robust perennial with white flowers, sometimes with pink centre, almost completely glabrous; white latex usually abundant (Bolivia)	**223. *I. lactifera***

**Key A9**

Plants with flowers in subcapitate inflorescences. Inflorescence pedunculate but flowers on reduced pedicels so clustered in a head-like inflorescence, the bracteoles often persistent.

**Table:** 

1	Corolla subcylindrical, suburceolate (Brazil)	**423. *I. scopulina***
–	Corolla funnel-shaped with expanded limb	**2**
2	Corolla white; peduncle up to 40 cm long; trailing liana	**82. *I. marcellia***
–	Corolla pink; peduncles usually < 10 cm long	**3**
3	Leaves, stem and sepals grey-tomentose	**4**
–	Leaves, stem and sepals glabrous or pubescent	**5**
4	Bracteoles ovate-rhomboid, 2–4 mm wide; corolla with a few hairs at tips of midpetaline bands	**353. *I. amazonica***
–	Bracteoles filiform, < 1 mm wide; corolla pubescent	**69. *I. argentinica***
5	Bracteoles forming a spathe-like involucre around the flowers	**6**
–	Bracteoles narrow or broad but not forming a spathe-like involucre	**7**
6	Bracteoles basally united to form a boat-shaped involucre, paler basally but not prominently veined	**419. *I. involucrata***
–	Bracteoles free at the base, not forming a boat-like structure, pale green with prominent dark veins	**244. *I. neurocephala***
7	Corolla glabrous	**8**
–	Corolla pubescent at least in bud	**386. *I. eriocalyx***
8	Bracteoles inconspicuous, caducous or somewhat persistent, up to 5 mm long	**9**
–	Bracteoles conspicuous, persistent, > 5 mm long	**10**
9	Sepals ovate, very shortly mucronate, abaxially pubescent, inconspicuously veined	**422. *I. fasciculata***
–	Sepals oblong to oblong-elliptic, acuminate, conspicuously mucronate, ciliate-margined or glabrous, prominently veined	**220. *I. batatas***
10	Leaves glabrous; bracteoles narrowly ovate, boat-shaped; inflorescence hispid-pilose	**240. *I. spruceana***
–	Leaves usually hirsute, at least abaxially; bracteoles linear, not boat-shaped; inflorescence pubescent but not hispid-pilose	**234. *I. indica***

**Key A10**

Trailing, climbing or twining plants not in Keys A1–9 with corolla > 3.5 cm long, pubescent on the exterior. Buds should be checked carefully as pubescence is more obvious at this stage. On mature flowers check near the apex of the midpetaline bands.

**Table:** 

1	Leaf base truncate, rounded, cuneate or attenuate, never cordate or sagittate; plant trailing	**2**
–	Leaf base cordate or sagittate; plant erect, climbing, twining or trailing	**21**
2	Leaves all or mostly 3-lobed	**3**
–	Leaves all simple, rarely a few weakly 3-lobed	**8**
3	Leaves white-tomentose or sericeous at least on the lower surface	**4**
–	Leaves pubescent or pilose but not whitish on either surface	**6**
4	Flowers solitary	**68. *I. pseudocalystegia***
–	Flowers in cymes	**5**
5	Inner sepals obtuse; adaxial leaf surface green, thinly pilose; corolla 7–8 cm long (Bolivia)	**63. *I. opulifolia***
–	Inner sepals acute; adaxial leaf surface thinly floccose-tomentose; corolla c. 4.5 cm long (Brazil)	**52. *I. malvaviscoides***
6	Sepals obtuse to subacute	**21. *I. delphinioides***
–	Sepals finely acuminate	**7**
7	Flowers solitary; corolla 8.5–9.5 cm long	**28. *I. megalantha***
–	Flowers in cymes; corolla 5.5–6.5 cm long	**20. *I. acutisepala***
8	Flowers solitary or paired	**9**
–	At least some inflorescences of 3- or more-flowered cymes	**12**
9	Leaves ovate to suborbicular	**23. *I. chodatiana***
–	Leaves oblong to oblong-elliptic	**10**
10	Sepals finely acuminate (Paraguay)	**19. *I. valenzuelensis***
–	Sepals obtuse to acute	**11**
11	Sepals acute; leaves broadly oblong, > 1.5 cm wide	**32. *I. subspicata***
–	Sepals obtuse; leaves narrowly oblong, < 1.2 cm wide	**36. *I. ensiformis***
12	Leaves white-tomentose or sericeous abaxially	**13**
–	Leaves hirsute but not whitish abaxially	**17**
13	Leaves white-sericeous on both surfaces	**26. *I. altoparanaensis***
–	Leaves distinctly discolorous, the adaxial surface green even if with some white hairs	**14**
14	Leaves broadly ovate to elliptic, scarcely longer than broad	**15**
–	Leaves narrowly ovate to oblong-ovate, 2–3 times longer than broad	**16**
15	Inflorescence from upper leaf axils only; leaves subrhomboid with petioles < 2 cm long (Andean Bolivia)	**56. *I. mendozae***
–	Inflorescence clearly axillary; leaves ovate with petioles 1–4.5 cm long (Southern Brazil)	**22. *I. uruguayensis***
16	Leaves asperous-pilose (Brazil)	**51. *I. langsdorfii***
–	Leaves white woolly, not asperous (Argentina)	**27. *I. lanuginosa***
17	Leaves all < 8 mm wide; sepals finely acuminate, 12–14 mm long	**37. *I. attenuata***
–	Leaves all > 15 mm wide; sepals obtuse or acute, up to 12 mm long	**18**
18	Leaves glabrous or thinly pubescent	**19**
–	Leaves conspicuously sericeous or pubescent, at least beneath	**20**
19	Petioles < 1 cm long; leaves completely glabrous; cymes simple (Bolivia)	**25. *I. psammophila***
–	Petioles up to 4.5 cm long; leaves glabrous or thinly pubescent abaxially; cymes usually compounded (Argentina)	**24b. I. nitida subsp. krapovickasii**
20	Leaves sericeous; sepals acute (Argentina)	**24a. I. nitida subsp. nitida**
–	Leaves pubescent; sepals obtuse to acute (Brazil)	**21. *I. delphinioides***
21	Leaves mostly 3-lobed to about halfway	**22**
–	Leaves unlobed or a few leaves 2–3-lobed	**27**
22	Sepals 15–20 mm long, pale green, minutely puberulent	**5. *I. cardenasiana***
–	Sepals < 15 mm long, grey-tomentose or pubescent	**23**
23	Sepals pilose with spreading hairs; plant of wetlands	**399. *I. rubens***
–	Sepals appressed hairy to sericeous; plants of dry habitats	**24**
24	Leaves dimorphic with some entire and some lobed on the same plant; inflorescence subterminal	**4. *I. prolifera***
–	Leaves all lobed on the same plant; inflorescence clearly axillary	**25**
25	Lobes acute to acuminate	**63. *I. opulifolia***
–	Lobes rounded to obtuse, mucronate	**26**
26	Flowers solitary or subsessile at the apex of a long peduncle	**68. *I. pseudocalystegia***
–	Flowers in cymes, clearly pedicellate	**67. *I. mucronifolia***
27	Leaves conspicuous grey- or white-tomentose or sericeous abaxially	**28**
–	Leaves green abaxially, not strongly grey- or white-tomentose	**53**
28	Inflorescence borne on long peduncles 20–42 cm in length	**29**
–	Inflorescence borne on peduncles < 25 cm long; corolla pink	**30**
29	Corolla white; stamens shortly exserted	**82. *I. marcellia***
–	Corolla pink; stamens included	**100. *I. descolei***
30	Flowers all or mostly solitary (rarely up to 3)	**31**
–	Flowers in axillary cymes of 3 or more flowers	**34**
31	Leaves mostly > 7 × 6 cm	**32**
–	Leaves very small, < 5 × 5 cm	**33**
32	Trailing herb; sepals acute, not markedly accrescent in fruit (Bolivia)	**57. *I. gypsophila***
–	Liana; sepals rounded to obtuse, accrescent to 2.8 cm (Galapagos)	**418. *I. tiliifolia***
33	Bracteoles caducous; pedicels 6–15 mm (Venezuela, Guyana)	**172. *I. discolor***
–	Bracteoles persistent; pedicels very short, < 5 mm long (Brazil)	**44. *I. geophilifolia***
34	Sepals relatively large, > 14 mm long, especially in fruit; bracteoles usually > 15 mm long	**35**
–	Sepals <13 mm long (sometimes more in glabrous leaved *I. chondrosepala*); bracteoles short, usually < 12 mm long	**39**
35	Leaves tomentellous adaxially; capsule large, 1.5–2 cm	**36**
–	Leaves glabrous adaxially; capsule unknown	**37**
36	Bracteoles persistent adpressed to calyx; leaves grey-tomentose adaxially	**98. *I. calyptrata***
–	Bracteoles caducous, not adpressed to calyx; leaves green-tomentose adaxially	**108. I. brasiliana var. subincana**
37	Outer sepals 14–16 mm long	**38**
–	Outer sepals 18–25 mm long	**396. *I. pearceana***
38	Leaves large, > 9 cm long; peduncles long, mostly > 15 cm long (Cultivated)	**393. *I. nervosa***
–	Leaves small, < 6 cm long (Peru); peduncles < 4.5 cm	**114. *I. mathewsiana***
39	Sepals short, < 8 mm long	**40**
–	Sepals 8–15 mm long	**42**
40	Sepals rounded, lacking black glands at base (Andes south to Peru)	**84a. I. carnea subsp. carnea**
–	Sepals acute to apiculate, commonly with dark glands at base	**41**
41	Vegetative parts all shortly and finely sericeous; ovary hirsute	**397. *I. velutinifolia***
–	Vegetative parts, subglabrous, pubescent or appressed pilose but never uniformly sericeous; ovary glabrous	**61. *I. megapotamica***
42	Sepals with conspicuous spreading hairs	**43**
–	Sepals appressed hairy, tomentose or sericeous	**44**
43	Bracteoles caducous; corolla c. 5 cm long (Wet places)	**399. *I. rubens***
–	Bracteoles somewhat persistent; corolla c. 8 cm long (Dry places, Bolivia)	**70. *I. longibarbis***
44	Sepals glabrous or nearly so	**45**
–	Sepals tomentose, sericeous or uniformly pubescent	**46**
45	Cymes simple; sepals ovate to elliptic	**73. *I. jalapa***
–	Cymes commonly compounded and inflorescence subracemose or corymbose; sepals oblong or oblong-obovate	**394. *I. abutiloides***
46	Leaves obtuse with a 3 mm apical mucro	**66. *I. walteri***
–	Leaves not as above	**47**
47	Bracteoles 12–20 mm long, persistent till after the flowers have fallen	**69. *I. argentinica***
–	Bracteoles usually < 10 mm long, usually deciduous at anthesis	**48**
48	Leaves dimorphic, some lobed, some entire; inflorescence subtermina	**4. *I. prolifera***
–	Leaves all entire; inflorescence axillary	**49**
49	Abaxial leaf surfaces with long appressed hairs; cymes usually few-flowered (Colombia)	**64. *I. macarenensis***
–	Abaxial leaf surface tomentose but hairs not appressed nor long	**50**
50	Corolla large 9–11 cm long (Ecuador, Colombia, Venezuela)	**73. *I. jalapa***
–	Corolla 4.5–7 cm long	**51**
51	Sepals oblong; inflorescence often formed on leafy branchlets	**395. *I. sericosepala***
–	Sepals ovate; inflorescence of leafless cymes	**52**
52	Sepals acute, not mucronate, eglandular, peduncles and pedicels usually short so inflorescence crowded (Central Brazil)	**65. *I. sericophylla***
–	Sepals mucronate, usually with two large basal glands; inflorescence lax (Southern Andes)	**45. *I. hieronymi***
53	Corolla < 4 cm long (Ecuador and Peru)	**311. *I. velardei***
–	Corolla > 5 cm long	**54**
54	Stem and inflorescence bearded with yellowish hairs; bracteoles persistent; pedicels < 10 mm long (Ecuador)	**245. *I. harlingii***
–	Stem and inflorescence not bearded with yellowish hairs; bracteoles persistent or not; pedicels mostly > 10 mm long	**55**
55	Stem with fleshy teeth; corolla limb deeply lobed; violet with white tube	**270. *I. parasitica***
–	Stem unarmed; corolla limb at most weakly lobed; tube coloured	**56**
56	Sepals lanceolate, much longer than broad	**57**
–	Sepals ovate to elliptic, only slightly longer than broad	**58**
57	Flowers solitary (rarely paired); peduncle < 5 mm long	**417. *I. chapadensis***
–	Flowers in cymes; peduncles well-developed, usually exceeding 10 mm	**416. *I. regnellii***
58	Flowers solitary (very rarely up to 3); sepals strongly accrescent and enveloping the capsule	**418. *I. tiliifolia***
–	Flowers several in cymes, rarely reduced to single flowers	**59**
59	Sepals with a prominent swollen abaxial appendage (Bolivia)	**58. *I. appendiculata***
–	Sepals lacking a prominent swollen abaxial appendage	**60**
60	Trailing perennial with stout stem; leaves undulate to dentate (very dry inter-Andean valleys of Bolivia and Argentina)	**71. *I. lilloana***
–	Twining or climbing perennials, stems stout to slender; leaves occasionally lobed but not undulate or dentate	**61**
61	Corolla very large, 7–12 cm long; sepals mostly > 10 mm long	**62**
–	Corolla 4–6.5 cm long; sepals mostly < 8 m long	**65**
62	Sepals glabrous; stem often winged	**72. *I. subalata***
–	Sepals thinly to densely pubescent; stems unwinged	**63**
63	Stems minutely spinulose, thinly pilose with long white hairs; abaxial surface of leaves glabrous apart from highlighted veins (Bolivia)	**46. *I. spinulifera***
–	Stems smooth, lacking spinules and long white hairs; veins not highlighted on abaxial leaf surface	**64**
64	Sepals 10–15 mm long (Central America, Caribbean and Ecuador)	**73. *I. jalapa***
–	Sepals 8–10 mm long (Brazil)	**59. *I. cearensis***
65	Sepals rounded to obtuse; base of calyx truncate (Brazil)	**62. *I. decipiens***
–	Sepals acute; base of calyx cuneate to rounded	**66**
66	Inflorescence clearly cymose; corolla pink; old stems not corky	**61. *I. megapotamica***
–	Inflorescence often subracemose; corolla usually white; old stems corky (Chaco)	**60. *I. vivianae***

**Key A11**

Trailing and twining plants not in Keys A1–9 with a glabrous corolla, > 3.5 cm long.

**Table:** 

1	Creeping seashore plant with fleshy stems and leaves; leaves apically retuse; pedicel persistent on fallen capsule	**339. *I. pes-caprae***
–	Plant not growing on seashores; leaves not apically retuse and rarely fleshy; pedicel not persistent on fallen capsule	**2**
2	Night flowering species with dull lilac, somewhat salver-shaped corolla; stems commonly armed with soft fleshy spines	**271. *I. muricata***
–	Day flowering species with pink corolla; stems lacking soft spines	**3**
3	Leaf base cuneate to attenuate; trailing plants of the Cerrado	**4**
–	Leaf base truncate, cordate, hastate or sagittate; plants of varying habit and habitat	**9**
4	Leaves linear or narrowly oblong, attenuate at base, the petiole not clearly differentiated	**5**
–	Leaves oblong to ovate, cuneate at base, the petiole distinct from the lamina	**6**
5	Sepals narrowed at base; leaves linear, 0.5–1 mm wide; stem, pedicels and leaves glabrous	**365. *I. graminifolia***
–	Sepals with a broad truncate base; leaves narrowly oblong, at least 2 mm wide; stem, pedicels and leaves with long white hairs	**420. *I. dolichopoda***
6	Sepals abaxially muricate	**7**
–	Sepals abaxially smooth	**8**
7	Leaves oblong or ovate; plant only woody basally	**345. *I. procurrens***
–	Leaves oblong-elliptic to suborbicular; woody subshrub	**344. *I. coriacea***
8	Flowers solitary (rarely paired); inflorescence leafless	**366. *I. procumbens***
–	Flowers in cymes, often somewhat leafy	**367. *I. rupestris***
9	Peduncle fused with petiole for part of its length	**92. *I. connata***
–	Peduncles and petioles not fused	**10**
10	Sepals with a prominent abaxial appendage	**379. *I. bahiensis***
–	Sepals smooth, ribbed or muricate but lacking a prominent abaxial appendage	**11**
11	Sepals with prominent abaxial muricate ribs	**12**
–	Sepals abaxially smooth	**14**
12	Bracteoles linear 3 × 0.5 mm; corolla c. 10 cm long	**343. *I. parvibracteolata***
–	Bracteoles 8–20 × 3–15 mm; corolla 2.5–8 cm long	**13**
13	Annual herb; corolla 2.5–3.5 cm long	**341. *I. fimbriosepala***
–	Perennial herb; corolla 5.5–8 cm long	**342. *I. setifera***
14	Sepals conspicuously truncate or cordate at base, often with a lateral tooth	**15**
–	Sepals narrowed or rounded at base, lacking teeth	**16**
15	Sepals ovate, cordate; leaves entire or shallowly 3-lobed	**376. *I. apodiensis***
–	Sepals deltoid, truncate; leaves usually 3–5-lobed to near the base, rarely ovate-deltoid	**377. *I. pantanalensis***
16	Sepals very unequal in length, the outer conspicuously shorter than the inner	**17**
–	Sepals all equal or slightly unequal in length	**29**
17	Leaves and stem white-tomentellous (Peru)	**115. *I. pulcherrima***
–	Leaves and stem not white-tomentellous	**18**
18	Abaxial surface of sepals commonly muricate; plants of seasonally wet areas	**19**
–	Abaxial surface of sepals smooth; plants of dry or moist habitats	**20**
19	Leaves ovate, sagittate	**346. *I. paludicola***
–	Leaves subreniform, hastate	**347. *I. asarifolia***
20	Outermost sepal very short, < 3 mm long; plant glabrous	**233. *I. cryptica***
–	Outermost sepal > 5 mm long; plant glabrous or pubescent	**21**
21	Leaves tomentose on both surfaces, cordate	**144. *I. mirabilis***
–	Leaves glabrous or thinly pubescent, sagittate or cordate	**22**
22	Sepals all < 10 mm long, the margins usually white	**380. *I. squamosa***
–	Inner sepals usually > 10 mm long, often somewhat scarious but not distinctly white-margined	**23**
23	Inner sepals < 12 mm long; leaves ovate to deltoid	**24**
–	Inner sepals > 13 mm long; leaves varied in shape	**25**
24	Inner sepals acuminate; corolla < 3. 5 cm long (Colombia)	**357. *I. colombiana***
–	Inner sepals obtuse to rounded, mucronate; corolla 4–6 cm long	**356. *I. maurandioides***
25	Sepals relatively large, the inner 15–28 mm long;; leaves usually rounded at apex	**360. *I. paranaensis***
–	Inner sepals < 16 mm long; leaves narrowed to an obtuse or acute apex	**26**
26	Leaves oblong, the margins undulate	**361. *I. variifolia***
–	Leaves linear, lanceolate or ovate, the margin entire	**27**
27	Leaves narrowly oblong, the base hastate to sagittate; peduncles very short, < 2 mm long	**362. *I. tacuaremboensis***
–	Leaves ovate-deltoid or linear, sagittate; peduncles mostly more than 2 cm long	**28**
28	Corolla lobes terminating in a distinct mucro 5–6 mm long; lamina narrowly ovate-deltoid with prominent deltoid auricles	**359. *I. mucronatoproducta***
–	Corolla unlobed or lobes not terminating in a distinct mucro; lamina linear, similar to the auricles	**358. *I. aequiloba***
29	Flowers solitary; leaves deltoid with very slender pedicels	**370. *I. longirostra***
–	Flowers several in axillary cymes; leaves ovate, cordate, not strikingly deltoid or with disproportionately slender pedicels	**30**
30	Sepals relatively short, all < 12 mm long	**31**
–	Sepals relatively long, some > 12 mm long	**40**
31	Perennial or annual herbs; sepals always mucronate, usually of papery texture; corolla usually with a dark centre, 3.5–5.5 cm long; leaves lobed or not; ovary and capsule hirsute or not	**Batatas Clade (Species 218–233)**
–	Perennial herbs or subshrubs; sepals mucronate or not but never papery in texture; corolla usually lacking a dark centre, 3.5–9 cm long; leaves unlobed; ovary and capsule glabrous	**32**
32	Stem, petioles or abaxial leaf surface tomentose, pubescent or puberulent	**33**
–	Plant completely glabrous	**36**
33	Leaves dentate (Bolivia)	**299. *I. odontophylla***
–	Leaves entire or undulate	**34**
34	Peduncle passing through sinus of leaf base; corolla pale blue (Bolivia)	**300. *I. huayllae***
–	Peduncle not passing through sinus of leaf base; corolla pink	**35**
35	Leaves with overlapping auricles; stamens held at corolla mouth, 2.5 cm long; seeds lanate	**88. *I. tarijensis***
–	Leaf auricles not overlapping; stamens held within corolla tube, 4–5 cm long; seeds tomentellous	**289. *I. jujuyensis***
36	Corolla blue with cream tube; sepals lanceolate, acute with white margins	**37**
–	Corolla pink (rarely white), the tube similar or darker in colour; sepals of varied shape but mostly mucronate, always lacking white margins	**38**
37	Corolla < 4 cm long (Andean Argentina and Bolivia)	**255. *I. marginisepala***
–	Corolla 5.5–7 cm (Mexico, but widely cultivated elsewhere)	**257. *I. tricolor***
38	Leaves usually sagittate; aquatic herb rooting at nodes on mud	**391. *I. aquatica***
–	Leaves ovate, cordate; subshrubs climbing to several metres	**39**
39	Corolla 4–5.5 cm long	**340b. I. amnicola subsp. chiliantha**
–	Corolla < 3.5 cm long	**340a. I. amnicola subsp. amnicola**
40	Leaves white-tomentose abaxially	**41**
–	Leaves variously hirsute or glabrous, but if ±tomentose abaxially, indumenm not white or bracts linear, persistent	**43**
41	Pedicels 1–4 mm, bracteoles persistent, appressed to calyx	**99. *I. veadeirosii***
–	Pedicels mostly > 10 mm, not appressed to calyx, caducous	**42**
42	Corolla 10–12 cm long; marginal hairs on seeds up to 20 mm long	**104. *I. magna***
–	Corolla 5–8 cm long; hairs on seeds up to 5 mm long	**108. *I. brasiliana***
43	Bracteoles linear to oblong, persistent; sepals commonly tapered to an elongated apex; leaves 3-lobed or, less commonly, entire; ovary trilocular; often weedy hirsute species of disturbed places (Pharbitis Clade)	**44**
–	Bracteoles filiform to linear, caducous; sepals varied but lacking an elongated apex; leaves unlobed; ovary bilocular, glabrous or hirsute herbs or subshrubs	**49**
44	Sepals deltoid with a distinct truncate base	**242. *I. pubescens***
–	Sepals linear-oblong, narrowed at base	**45**
45	Sepals 15– 35 mm long, tapering to a long point, lanceolate with a broad base, often conspicuously pilose at base; leaves 3-lobed	**46**
–	Sepals < 20 mm long, linear-oblong, pubescent but not conspicuously pilose near base; leaves simple or 3-lobed	**47**
46	Sepals with fleshy recurved tips (southern USA, adventive elsewhere)	**237. *I. hederacea***
–	Sepals with erect, herbaceous tips (very widespread)	**236. *I. nil***
47	Leaves very large, 11–20 × 7–20 cm; corolla 7–9 cm long; bracteoles usually caducous (Bolivia and Peru)	**247. *I. magnifolia***
–	Leaves < 11 × 11 cm; corolla 4–6 cm; bracteoles always persistent (widespread)	**48**
48	Vegetative parts softly pubescent; sepals oblong or lanceolate; corolla usually pink; leaves usually unlobed; flowers not clustered	**238. *I. purpurea***
–	Vegetative parts usually hirsute but not softly pubescent; sepals ovate; corolla usually bluish-purple; leaves commonly lobed; flowers commonly clustered	**234. *I. indica***
49	Pedicels and sepals with long shaggy hairs	**105. *I. longibracteolata***
–	Pedicels and sepals glabrous or shortly pubescent	**50**
50	Sepals acuminate to a fine point	**51**
–	Sepals obtuse, rounded or retuse	**52**
51	Sepals prominently veined; leaves sagittate; plant glabrous	**355. *I. incarnata***
–	Sepals not prominently veined; leaves cordate; plant thinly pubescent	**247. *I. magnifolia***
52	Stems winged (caatinga of Bahia)	**91. *I. pterocaulis***
–	Stems not winged (moist forest)	**53**
53	Corolla bluish or greenish or yellow; sepals oblong-lanceolate; peduncles very short, usually < 1 cm long	**400. *I. lindenii***
–	Corolla pink; sepals oblong, ovate or suborbicular; peduncles usually > 1 cm long or flowers borne on leafy side shoots	**54**
54	Corolla tube narrow, often constricted below limb; sepals opaque, commonly reddish; inflorescence often compound, much branched	**352. *I. philomega***
–	Corolla broadly funnel-shaped, not constricted upwards; sepals somewhat transparent, pale green; inflorescence of simple cymes	**369. *I. chondrosepala***

### B. Keys to Central and North American species

This key includes all species from continental North America from Panama northwards. For plants from the Caribbean islands, go to Key C and for plants from Hawaii, go to Key D.

Several North American species are notable for having dentate leaves, often in the form of one or two lateral teeth on otherwise entire leaves. The following species are noted as having dentate leaves, at least to some degree: *Ipomoea tastensis*, *I. jicama*, *I. noctulifolia*, *I. schaffneri*, *I. ignava*, *I. stans*, *I. jacalana*, *I. tacambarensis*, *I. acanthocarpa*, *I. rupicola*, *I. calcicola*, *I. dumetorum* (at least sometimes).

**Note.** Options in Keys B1 to B8 should be considered before proceeding to Keys B9–10.

Key B1: Species with oblong, lanceolate or elliptic leaves, the base narrowed, cuneate to rounded, margin entire or toothed, sometimes pinnatifid, or pinnate.

Key B2: Species with leaves divided digitately to, or near to the base, into five or more free or nearly free lobes or segments.

Key B3: Species with soft fleshy spines/protuberances on the sepals.

Key B4: Species with a subcylindrical corolla tube and (usually) exserted stamens

Key B5: Species with small flowers, the corolla < 3 cm long (or calyx < 5 mm long)

Key B6: Species with white, cream or yellowish flowers > 3 cm long

Key B7 Species with very long sepals, mostly exceeding 1.8 cm in length

Key B8: Plants with subcapitate inflorescences.

Key B9: Trailing, climbing or twining plants with pubescent or hirsute sepals < 1.8 cm long

Key B10. Trailing, climbing or twining plants with glabrous sepals <1.8 cm long.

**Key B1**

Species with oblong, lanceolate or elliptic leaves, the base narrowed, cuneate to rounded, margin entire or toothed, sometimes pinnate or pinnatifid. Leaves never cordate, hastate, sagittate or truncate or palmately lobed or palmately divided into leaflets. Stems commonly erect, less commonly trailing or climbing.

**Table:** 

1	Leaves pinnatifid or strongly dentate	**2**
–	Leaves entire or obscurely dentate, serrate or crenate	**6**
2	Anthers exserted; leaves with pseudo-stipules	**312. *I. quamoclit***
–	Anthers included; leaves lacking pseudo-stipules	**3**
3	Leaves oblong with lyrate-dentate margin, usually abaxially pubescent	**4**
–	Leaves pinnatifid with narrow segments 1–2 mm wide, glabrous	**5**
4	Flowers solitary or paired from the leaf axils; sepals unequal; petioles < 5 mm long	**276. *I. stans***
–	Inflorescence of terminal and axillary cymes with 5–15 flowers; sepals subequal; petioles 2–4.5 cm long	**277. *I. tacambarensis***
5	Leaf segments 1–6 cm long; peduncles 3–12 cm long at anthesis; corolla white or pale lilac	**278. *I. ancisa***
–	Leaf segments 0.3–2.5 cm long; peduncles 0.8–3.5 cm at anthesis; corolla purplish-blue	**279. *I. sescossiana***
6	Low, often prostrate plants of high altitudes; stems short usually < 20 cm long; sepals muricate; leaves often dimorphic	**7**
–	Erect or climbing plants; stems > 20 cm long; sepals smooth; leaves all similar in form	**8**
7	Corolla 5–5.5 cm long; sepals 6–10 mm long; leaves 2–10 cm long, commonly dimorphic with some simple, some forked and, occasionally, some lobed	**286. *I. madrensis***
–	Corolla 2–3 cm long; sepals 4–6 mm long; leaves < 2 cm long, all of the same form	**287. I. plummerae forma adiantifolia**
8	Corolla glabrous on the exterior	**9**
–	Corolla pubescent on the exterior at least in bud	**15**
9	Leaves oblong-elliptic or ovate; plants decumbent, climbing or twining	**10**
–	Leaves oblong or lanceolate; plants erect	**13**
10	Leaves abaxially appressed pilose, silvery in colour	**179. *I. steerei***
–	Leaves glabrous	**11**
11	Woody liana; bracteoles 15–26 mm long, oblong-elliptic; peduncles very short, 2–8 mm long	**174. *I. robinsonii***
–	Perennial herbs, woody at base; bracteoles < 3 mm long; peduncles up to 7 cm long	**12**
12	Corolla pink, trumpet-shaped (United States)	**349. *I. shumardiana***
–	Corolla orange or yellow, funnel-shaped (Mesoamerica)	**173. *I. aurantiaca***
13	Sepals very unequal, the outer 10–16 mm long, the inner 16–23 mm long	**93. *I. longifolia***
–	Sepals subequal, 5–12 mm long	**14**
14	Leaves pubescent, 4–8 × 1.5–2.5 cm	**128. *I. petrophila***
–	Leaves glabrous, 3–10 × 0.5 cm	**348. *I. leptophila***
15	Peduncles very short, <5 mm long	**16**
–	Peduncles >1.5 cm long	**17**
16	Leaves, 1 cm long; sepals 7–10 mm long, obtuse	**129. *I. lenis***
–	Leaves 2–3.5 cm long; sepals 12–16 mm long, acuminate	**130. *I. durangensis***
17	Twining plant; leaves finely acuminate, well-spaced; corolla pink or lilac	**78. *I. kruseana***
–	Erect undershrub; leaves acute or obtuse, imbricate; corolla white	**131. *I. ciervensis***

**Key B2**

Species with leaves palmately divided into free or almost free leaflets. This key includes species where the leaflets are pedatisect. All species have glabrous corollas.

**Table:** 

1	Sepals elliptic to obovate, obtuse or rounded, coriaceous; robust lianas or perennials	**2**
–	Sepals oblong, lanceolate or ovate, commonly acuminate and apiculate; plants mostly slender, annual or perennial herbs	**3**
2	Corolla red, > 4 cm long; cultivated woody liana	**211. *I. horsfalliae***
–	Corolla greenish-white, <3.5 cm long; native perennial of Mesoamerica	**178. *I. heterodoxa***
3	Corolla ±salver-shaped; anthers strongly exserted; sepals awned	**313. *I. fissifolia***
–	Corolla ±funnel-shaped; anthers included; sepals lacking a subterminal awn	**4**
4	Corolla 4.5–7 cm long, pink or white; leaves with or without pseudo-stipules	**5**
–	Corolla usually < 4.5 cm, pink, white or yellow; leaves lacking pseudo-stipules	**8**
5	Decumbent or ascending plant with short stems, commonly < 10 cm long; leaves lacking pseudo-stipules, usually dimorphic with some palmately lobed and some entire or bifid	**286. *I. madrensis***
–	Twining plants, stems usually much more than 25 cm long; leaves of one kind, with or without conspicuous pseudo-stipules	**6**
6	Cylindrical basal part of corolla > 2 cm in length; leaves without conspicuous pseudo-stipules	**285. *I. tenuiloba***
–	Cylindrical basal part of corolla very short, < 5 mm long; leaves usually with conspicuous pseudo-stipules	**7**
7	Annual herb; leaves with up to 14 linear or ensiform leaflets	**332. *I. diegoae***
–	Perennial herb; leaves usually with 5 oblong-elliptic leaflets	**392. *I. cairica***
8	Outer sepals with cordate base and prominent soft spines on abaxial surface	**333. *I. sororia***
–	Outer sepals neither basally cordate nor with soft spines on adaxially surface	**9**
9	Peduncle coiled or twisted; sepals obtuse	**374. *I. heptaphylla***
–	Peduncle straight or suppressed; Sepals acute or acuminate	**10**
10	Plants erect 5–20 cm high, not twining; leaf segments filiform, <1 mm wide	**288. *I. capillacea***
–	Plants with twining or trailing stems; leaf segments at least 2 mm wide	**11**
11	Corolla < 1.2 mm long	**328. *I. costellata***
–	Corolla > 1.5 cm long	**12**
12	Corolla entirely yellow	**329. *I. chamelana***
–	Corolla pink, white or bluish	**13**
13	Leaves twice divided, the palmate lobes pinnatifid	**331. *I. perpartita***
–	Leaf segments simple, not pinnatifid	**14**
14	Cylindrical basal part of corolla very short, often < 5 mm	**15**
–	Cylindrical basal part of corolla elongated, often > 10 mm long	**285. *I. tenuiloba***
15	Outer sepals usually muricate, glabrous, obtuse to acute; root a globose tuber; corolla deep pink; leaf segments obtuse	**287. *I. plummerae***
–	Outer sepals smooth, glabrous or pubescent, acute to acuminate; root a small tap root; corolla pale lilac; leaf segments acute	**334. *I. ternifolia***

**Key B3**

Plants with sepals covered in soft slightly fleshy spines or protuberances

**Table:** 

1	Spines and protuberances present only on the sepals	**2**
–	Spines present on petioles, peduncles and/or stem as well as on the sepals	**216. *I. setosa***
2	Sepals with abundant spreading soft spines, resembling fleshy trichomes	**3**
–	Sepals with few to many fleshy protuberances, wings or similar structures, not resembling fleshy trichomes	**4**
3	Sepals up to 35 mm long; peduncles 0–0.4 cm long; pedicels 2–4 cm long	**410. *I. silvicola***
–	Sepals 12–15 mm long; peduncles 0.5–8 cm long; pedicels 0.8–2.1 cm long	**408. *I. crinicalyx***
4	Pedicels short, < 4.5 mm; corolla 2.5–3 cm long; peduncle winged	**372. *I. decemcornuta***
–	Pedicels elongate, > 15 mm long; corolla 5–8 cm long; peduncle unwinged	**5**
5	Flowers usually numerous, cymes often much branched; corolla pubescent in bud and at tips of midpetaline bands	**414. *I. pedicellaris***
–	Flowers usually solitary, rarely paired, inflorescence simple; corolla glabrous even in bud	**6**
6	Peduncles 4.5–7.5 cm long; pedicels notably stouter than peduncle; sepals c. 8 mm long	**415. *I. tentaculifera***
–	Peduncles < 4 cm long; pedicels similar to peduncles in width; sepals 12–14 mm long	**132. *I. lozanii***

**Key B4**

Corolla tube cylindrical for at least half its length, often to the base of the limb; stamens equal or nearly so, anthers exserted or held at mouth of corolla.

**Table:** 

1	Leaves pinnate; pseudo-stipules present	**312. *I. quamoclit***
–	Leaves not pinnate but if pinnatifid, pseudo-stipules absent	**2**
2	Sepals with a distinct subterminal awn; corolla red, orange or yellow	**Go to the Quamoclit Clade (312–327)**
–	Sepals lacking a subterminal awn or, if awn present; corolla pure white or blue	**3**
3	Corolla white, blue or pale lilac	**4**
–	Corolla pink or red	**16**
4	Corolla tube cylindrical to below the limb	**5**
–	Corolla tube cylindrical for about half its length, then gradually expanded in upper half	**8**
5	Sepals terminating in a prominent awn	**272. *I. alba***
–	Sepals acute or obtuse, sometimes with a short mucro but never terminating in a long awn	**6**
6	Sepals 16–23 mm long; anthers weakly exserted or included; sea shore species	**389. *I. violacea***
–	Sepals <9 mm; anthers clearly exserted; inland species	**7**
7	Peduncle furnished with prominent setae at base; corolla limb undulate; sepals acute, mucronate	**421. *I. discoidea***
–	Peduncle glabrous at base; corolla limb distinctly 5-lobed; sepals obtuse	**138. *I. macdonaldii***
8	Corolla pubescent on the exterior at least in bud	**9**
–	Corolla glabrous on the exterior even in bud	**11**
9	Sepals obtuse, equal; leaves weakly lobed, abaxially pubescent at least on the veins (Mexico)	**10**
–	Sepals aristate, unequal; leaves entire, glabrous (Costa Rica)	**274. *I. magniflora***
10	Flowers solitary; peduncle < 2.5 cm long; sepals 13–16 mm long	**77. *I. zimmermanii***
–	Flowers in cymes; peduncles very long, 11–20 cm; sepals 25–40 cm long	**248. *I. ampullacea***
11	Corolla blue orlilac; stems armed with soft spines; sepals with an aristate tip	**271. *I. muricata***
–	Corolla white, occasionally pale lilac; stems unarmed; sepals sometimes mucronate but never aristate	**12**
12	Sepals < 7 mm long; anthers scarcely exserted	**13**
–	Sepals > 10 mm long; anthers strongly exserted	**14**
13	Flowers solitary; leaves entire; corolla bluish (drying pink)	**307. *I. expansa***
–	Inflorescence formed of cymes with up to 7 flowers; leaves 3-lobed; corolla white	**136. *I. lottiae***
14	Leaves with prominent lateral teeth; sepals 2–3 cm long	**297. *I. tastensis***
–	Leaves entire or palmately lobed; sepals <1.6 cm long	**15**
15	Corolla pale blue; flowers solitary	**262. *I. gilana***
–	Corolla pure white; flowers usually several	**273. *I. santillanii***
16	Corolla limb short and inconspicuous (except *I. electrina*), the tube cylindrical	**17**
–	Corolla limb formed of broad obovate lobes, the tube often not strictly cylindrical	**21**
17	Flowers enclosed within two conspicuous persistent bracteoles forming a spathe-like inflorescence	**338. *I. bracteata***
–	Flowers naked, bracteoles inconspicuous, often caducous	**18**
18	Exterior of the corolla conspicuously sericeous or pubescent	**181. *I. concolor***
–	Exterior of the corolla glabrous or nearly so	**19**
19	Corolla lobes linear, > 15 mm long	**294. *I. electrina***
–	Corolla lobes very short, ovate to elliptic, c. 5 mm long	**20**
20	Pedicels and sepals pubescent	**180. *I. conzattii***
–	Pedicels and sepals glabrous	**182. *I. tehuantepecensis***
21	Sepals broadly obovate, 18–25 × 12–16 mm (Panama)	**269. *I. mirandina***
–	Sepals lanceolate, ovate or oblong, < 6 mm wide	**22**
22	Leaves sagittate	**284. *I. caudata***
–	Leaves ovate-cordate	**23**
23	Limb clearly lobed, the lobes short, c. 1.5 cm diameter	**293. *I. tubulata***
–	Limb subentire, 3.5–5 cm diameter	**24**
24	Petiole and peduncle fused for part of their length, peduncle usually passing through leaf sinus; calyx usually concealed by folded lamina	**291. *I. dumosa***
–	Petiole and peduncle free to their base; peduncle not passing through leaf sinus; calyx not concealed by folded leaf	**25**
25	Sepals lanceolate, 3–5 times longer than broad, unequal, the outer noticeably shorter than the inner	**266. *I. chenopodiifolia***
–	Sepals ovate, only slightly longer than broad, subequal	**290. *I. purga***

**Key B5**

Species with small flowers; this includes species with a calyx less than 5 mm long or a corolla less than 3 cm long. Mostly slender herbs but includes a few species of liana habit.

**Table:** 

1	Leaves palmatisect into separate segments	**Go to Key B2**
–	Leaves entire or lobed	**2**
2	Sepals very short, < 4 mm long	**3**
–	Sepals > 4 mm long	**5**
3	Corolla purple; sepals with 3 distinctive wings/ protuberances	***I. decemcornuta***
–	Corolla yellow; sepals smooth, unwinged	**4**
4	Corolla 4–5 mm long, not obviously lobed; stems pilose	**336. *I. minutifolia***
–	Corolla 25–30 mm long, deeply lobed; stems glabrous or nearly so	**335. *I. microsepala***
5	Ovary and capsule pubescent	**Go to key to Batatas Clade (218–232)**
–	Ovary and capsule glabrous	**6**
6	Sepals 10–15 mm long, narrowly lanceolate, nearly always with long, spreading stiff hairs; corolla blue	**258. *I. barbatisepala***
–	Sepals glabrous or with a few short hairs, if more than 10 mm long, not narrowly lanceolate; corolla cream or pink	**7**
7	Corolla cream-coloured, ± campanulate; inflorescence often developing into a raceme-like structure; stems woody	**8**
–	Corolla pink (rarely white), funnel-shaped; inflorescence of axillary cymes; stems herbaceous except at base	**9**
8	Sepals 5–7 mm long, deciduous in fruit	**87. *I. reticulata***
–	Sepals 10–14 mm long, often persistent and speading in fruit	**403. *I. corymbosa***
9	Plant vigorous, somewhat fleshy, clearly perennial, completely glabrous; sepals oblong-elliptic, mucronate	**340. *I. amnicola***
–	Plant relatively slender, not fleshy, annual or short-lived perennial; sepals not as above	**10**
10	Leaves strap-shaped (Florida)	**232. *I. tenuissima***
–	Leaves variously shaped but never strap-shaped	**11**
11	Low perennial, decumbent, with short stems < 10 cm long; leaves cuneate	**287. *I. plummerae***
–	Twining annual (?always) herbs, the stems usually at least 1 m long	**12**
12	Outer sepals obovate, mucronate; capsule depressed-globose, muticous	**230. *I. ramosissima***
–	Outer sepals oblong-lanceolate to oblong-ovate; capsule ovate, rostrate	**13**
13	Sepals lanceolate or ovate, acute, the margins whitish, scarious	**14**
–	Sepals not as above	**15**
14	Corolla blue with white tube; sepals lanceolate, 2–4 mm wide; pedicels relatively long, 1–3 cm; leaves lacking a lateral tooth	**256. *I. cardiophylla***
–	Corolla pink (rarely white); sepals ovate, 3.5–7 mm wide; pedicels 2–5 mm long; leaves commonly with a lateral tooth	**382. *I. acanthocarpa***
15	Peduncle passing through leaf sinus; sepals often muricate, sometimes pubescent, never with dark spots	**298. *I. aristolochiifolia***
–	Peduncle not passing through leaf sinus; sepals smooth, glabrous, the abaxial surface with dark spots	**281. *I. dumetorum***

**Key B6**

Plants with white, cream or yellowish flowers more than 3 cm in length, often much more, the throat occasionally dark.

**Table:** 

1	Small trees or erect, woody, often multi-stemmed shrubs, often leafless at anthesis	**Go to key to the Arborescens Clade (Species117–126)**
–	Twining or trailing herbs or lianas	**2**
2	Stamens exserted; corolla hypocrateriform or salverform or nearly so	**Go to Key B4**
–	Stamens included; Corolla funnel-shaped or campanulate	**3**
3	Corolla campanulate, not more than 3.5 cm long	**4**
–	Corolla funnel-shaped, hypocrateriform or salver-shaped, usually much more than 3.5 cm long	**5**
4	Sepals 5–7 mm long, deciduous in fruit	**87. *I. reticulata***
–	Sepals 10–14 mm long, often persistent and spreading in fruit	**403. *I. corymbosa***
5	Prostrate seashore plant rooting at the nodes; leaves shortly oblong, linear, lanceolate or 3–5-lobed, small, 1.5–3 × 0.8–2 cm	**388. *I. imperati***
–	Climbing herbs or lianas of inland areas; leaves ovate, mostly large or absen	t	**6**
6	At least some sepals 13 mm or more in length	**7**
–	All sepals < 13 mm in length	**12**
7	Corolla and sepals tomentose or pubescent on the exterior	**8**
–	Corolla and sepals glabrous on the exterior	**9**
8	Trailing herb of coastal regions of the United States; inflorescence clearly axillary Liana of Mexico and Central America; inflorescence arising on short shoots	**79. *I. praecana***
9	Leaves 3-lobed; sepals aristate; inflorescence paniculate	**330. *I. ramulosa***
–	Leaves entire; sepals muticous or at most shortly mucronate; inflorescence of axillary cymes	**10**
10	Cymes borne on long peduncles usually > 5 cm long	**259. *I. chiriquensis***
–	Cymes very shortly pedunculate; the peduncles usually < 1 cm long	**11**
11	Pedicels very short, cymes dense, subcapitate; bracteoles conspicuous, persistent	**112. *I. riparum***
–	Pedicels 2–4 cm long, cymes relatively lax; bracteoles inconspicuous, caducous	**400. *I. lindenii***
12	Bracteoles 1.5–2.5 cm long, oblong or oblong-elliptic, persistent; corolla relatively large, 7–8 cm long	**174. *I. robinsonii***
–	Bracteoles relatively small and inconspicuous, < 5 mm long, usually linear, filiform or squamose: corolla varied in size, often < 7 cm long	**13**
13	Corolla orange or yellow; leaves ovate, unlobed, the base truncate	**173. *I. aurantiaca***
–	Corolla white or cream, sometimes with dark throat or blue-flushed; leaves ovate, usually cordate, sometimes lobed, sometimes absent at anthesis	**14**
14	Corolla white with pink throat; sepals with prominent veins on abaxial surface; leaves often pandurate (United States)	**350. *I. pandurata***
–	Corolla white with a dark centre; sepals lacking prominent veins on abaxial surface; leaves entire, shallowly lobed; rarely pandurate	**15**
15	Outermost sepal much shorter than inner sepals, <5 mm long	**16**
–	Sepals equal or nearly so, or if somewhat unequal, outer sepal at least 5 mm long	**17**
16	Peduncles 5–10 cm long; bracteoles caducous; outer sepal c. 3 mm long, green	**381. *I. anisomeres***
–	Peduncles < 1 cm long; bracteoles persistent; outer sepal c. 5 mm long, whitish-green	**111. *I. pochutlensis***
17	Sepals oblong to oblong-obovate, not coriaceous nor convex; flowers usually solitary (rarely up to 3); leaves typically very small < 4.5 cm long (if flowers several and sepals oblong-lanceolate see *I. lindenii*)	**133. *I. hartwegii***
–	Sepals ovate to elliptic, coriaceous, usually convex; flowers solitary or in cymes; some leaves > 4.5 cm long or leaves absent	**18**
18	Corolla sericeous; plant leafless at anthesis, stem and leaves velutinous	**140. *I. pruinosa***
–	Corolla glabrous or almost so; Plant leafy or leafless at anthesis; stem and leaves glabrous or variously hirsute but not velutinous	**19**
19	Stem, leaves, (and typically) pedicels and sepals pilose with stiff spreading, bristly hairs	**141. *I. suaveolens***
–	Hairs, if present, neither spreading nor bristly	**20**
20	Sepals distinctly unequal; outer sepals oblong to oblong-elliptic, inner sepals up to 12 mm long; indumentum with at least some branched hairs	**135. *I. scopulorum***
–	Sepals equal or slightly unequal, varied in shape but about as broad as long; plants glabrous or with simple hairs	**21**
21	Corolla hypocrateriform	**22**
–	Corolla funnel-shaped	**23**
22	Leaves lobed; stem, leaves and sepals pubescent; peduncles < 3 cm long	**136. *I. lottiae***
–	Leaves entire; stem, leaves and sepals glabrous except on the leaf margins; peduncles > 10 cm long	**138. *I. macdonaldii***
23	Woody lianas; leaves entire, never lobed	**24**
–	Perennial herb, leaves lobed or entire, or, if woody, absent at anthesis	**25**
24	Leaves broadly ovate, 7–14 × 6–10 cm, pubescent	**134. *I. cuprinacoma***
–	Leaves obscurely puberulent	**118. *I. populina***
25	Plant leafless at anthesis	**139. *I. pseudoracemosa***
–	Leaves present at anthesis	**26**
26	Leaves pubescent, usually lobed; peduncles < 1 cm long	**27**
–	Leaves glabrous; peduncles > 3 cm long	**28**
27	Leaves entire, usually glabrous; sepals oblong-lanceolate, 5–18 mm long; corolla often flushed violet	**400. *I. lindenii***
–	Leaves commonly lobed, usually pubescent; sepals ovate to orbicular up to 8 mm long; corolla white	**137. *I. proxima***
28	Peduncles up to 13 cm long; leaves basally truncate; plant of central Mexico	**139. *I. pseudoracemosa*** form
–	Peduncles usually 3–6 cm long; leaves basally cordate, flowers often pink; plant of southern Mexico	**145. *I. batatoides***

**Key B7**

Plants with long sepals > 18 mm in length.

**Table:** 

1	Sepals covered in soft spines	**410. *I. silvicola***
–	Sepals lacking soft spines	**2**
2	Sepals terminating in a prominent awn; corolla white, the tube long, narrow, cylindrical	**272. *I. alba***
–	Sepals acute or obtuse, sometimes with a short mucro but never terminating in a long awn; corolla tube not long, narrow and cylindrical, white, blue or pink	**3**
3	Liana with winged stem; leaves palmatilobed; peduncles < 11 mm long; corolla subcampanulate, magenta	**127. *I. kahloae***
–	Plants of varied habit but stems unwinged and corolla never magenta; peduncles usually > 15 mm long	**4**
4	Small trees or lianas; corolla white	**5**
–	Perennial or annual herbs; corolla pink or blue, rarely white	**6**
5	Liana	**79. *I. praecana***
–	Tree	**119. *I. wolcottiana***
6	Leaves with marginal teeth	**7**
–	Leaves entire or lobed but lacking marginal teeth	**8**
7	Corolla 9–12 cm long, white; anthers exserted	**297. *I. tastensis***
–	Corolla 5–6 cm, pale pink; anthers included	**296. *I. jicama***
8	Sepals distinctly pubescent or tomentose	**9**
–	Sepals glabrous or nearly so	**18**
9	Corolla glabrous on the exterior	**10**
–	Corolla pubescent, sericeous on tomentose on the exterior	**15**
10	Sepals with distinct white margins	**11**
–	Sepals uniformly green	**12**
11	Leaves entire	**261. *I. orizabensis***
–	Leaves deeply lobed	**261b. I. orizabensis subsp. collina**
12	Flowers 1(–2); leaves usually 3–5 lobed; corolla pink	**13**
–	Flowers usually of 2 or more flowers; leaves entire or shallowly lobed; corolla blue or pink	**14**
13	Corolla 7–9 cm long; sepals lanceolate, cuneate, much longer than broad	**243. *I. lindheimeri***
–	Corolla < 5 cm long; sepals ovate, cordate, c. twice as long as broad	**242. *I. pubescens***
14	Corolla pink (rarely white or blue); sepals oblong-lanceolate, obtuse or acute; leaves entire or 3–5-lobed	**238. *I. purpurea***
–	Corolla blue with a white tube (drying pink): sepals ovate with an elongate apex, notably accrescent in fruit	**236. *I. nil***
15	Base of sepals truncate or subcordate; leaves palmately lobed or entire	**253. *I. laeta***
–	Base of sepals cuneate to rounded; leaves entire or 3-lobed	**16**
16	Flowers in cymes of 3–5; stigma 3-lobed; capsule 15 mm wide, not enclosed by accrescent sepals	**17**
–	Flowers usually solitary, rarely 2–3; stigma bilobed; capsule subglobose, 20–25 mm wide, enclosed by accrescent sepals (near the sea)	**418. *I. tiliifolia***
17	Leaves entire; bracteoles deciduous	**250. *I. mairetii***
–	Leaves 3-lobed; bracteoles persistent	**249. *I. temascaltepecensis***
18	Abaxial surface of outer sepals with prominent longitudinal veins	**19**
–	Abaxial surface of outer sepals lacking prominent longitudinal veins	**21**
19	Bracteoles prominent, persistent; veins on sepals denticulate; corolla pink	**20**
–	Bracts minute, deciduous; veins on sepals smooth; corolla white with a pink throat	**350. *I. pandurata***
20	Annual herb; corolla 2.5–3.5 cm long	**341. *I. fimbriosepala***
–	Perennial herb; corolla 5.5–8 cm long	**342. *I. setifera***
21	Inflorescence with large boat-shaped, chartaceous, oblong-elliptic bracteoles 2–2.5 × 0. 5–1.2 cm	**22**
–	Inflorescence with small, inconspicuous, often caducous bracteoles	**23**
22	Corolla glabrous	**251. *I. invicta***
–	Corolla pubescent	**252. *I. lambii***
23	Sepals broadly (ob)ovate, elliptic or suborbicular, scarcely longer than broad	**24**
–	Sepals ovate, lanceolate or oblong, distinctly longer than broad	**25**
24	Corolla hypocrateriform; anthers exserted (Panama)	**269. *I. mirandina***
–	Corolla funnel-form; stamens included	**352. *I. philomega***
25	Sepals narrowly lanceolate, acuminate; leaves lobed	**254. *I. thurberi***
–	Sepals oblong, oblong-lanceolate or oblong-ovate; leaves usually entire	**26**
26	Sepals with prominent white hyaline margins	**261. *I. orizabensis***
–	Sepals lacking distinct white hyaline margins	**27**
27	Flowers solitary (rarely paired); inner sepals 22–30 mm long (Mexico southwards)	**28**
–	Flowers several to many in cymes; inner sepals usually < 22 mm long (United States)	**350. *I. pandurata***
28	Flowers blue; stem thinly pilose with long white hairs	**401. *I. clavata***
–	Flowers pink; stem glabrescent (puberlous when young)	**295. *I. bernoulliana***

**Key B8**

Inflorescence subcapitate, flowers in compact heads, never solitary; bracteoles usually persistent.

**Table:** 

1	Corolla pubescent, at least in bud; bracteoles somewhat chartaceous	**2**
–	Corolla glabrous, even in bud; bracteoles not chartaceous	**4**
2	Corolla, stem, bracteoles and leaves sparsely hairy	**252. *I. lambii***
–	Corolla, stem, bracteoles and leaves densely hairy	**3**
3	Outer bracteoles ovate to suborbicular, 7–20 × 7–24 mm, pale green with darker veins	**244. *I. neurocephala***
–	Outer bracteoles lanceolate to ovate, 20–25 × 5 mm, uniformly green	**246. *I. villifera***
4	Corolla white	**112. *I. riparum***
–	Corolla pink or violet	**5**
5	Bracteoles linear/filiform, < 1 m wide	**305. *I. meyeri***
–	Bracteoles expanded, ovate or oblong > 2 mm wide	**6**
6	Leaves forming a spathe-like structure around the terminal inflorescence	**268. *I. mcvaughii***
–	Leaves not forming a spathe-like structure around the flowers; inflorescence clearly axillary	**7**
7	Bracteoles up to 2.5 cm long; sepals 20–23 mm long (Mexico)	**251. *I. invicta***
–	Bracteoles up to 10 mm long; sepals 11–20 mm (widespread)	**234. *I. indica***

**Key B9**

Plants not in Keys B1–8 with pubescent, pilose or tomentose sepals, < 18 mm long.

**Table:** 

1	Small erect trees or shrubs	**2**
–	Perennial or annual herbs	**3**
2	Flowers pink; sepals < 6 mm long, densely tomentellous	**84b. I. carnea subsp. fistulosa**
–	Flowers white; Sepals > 5.5 mm long, sparsely pubescent	**Go to Arborescens Clade (Species117–126)**
3	Corolla glabrous on the exterior	**4**
–	Corolla pubescent on the exterior at least when in bud	**13**
4	Sepals abruptly terminating in a distinct mucro; slender, usually annual herbs	**Go to the Batatas Clade (Species 218–233)**
–	Sepals obtuse or narrowed into a terminal mucro, margin not clearly ciliate	**5**
5	Leaves dentate with conspicuous teeth	**6**
–	Leaves entire	**7**
6	Leaf margin with numerous small teeth; sepals foliose, 1–4.5 cm long	**275. *I. jacalana***
–	Leaf margin with few large teeth; bracteoles small, 3–7 mm	**302. *I. schaffneri***
7	Sepals pilose with conspicuous long spreading hairs	**8**
–	Sepals pubescent or very shortly pilose	**11**
8	Sepals lanceolate, acuminate, > 9 mm long; corolla pink or blue	**9**
–	Sepals elliptic, obtuse, < 8 mm long; corolla white	**141. *I. suaveolens***
9	Sepals glabrous in the upper half; leaves always entire; ovary bilocular	**306. *I. mitchelliae***
–	Sepals hirsute to apex; leaves entire or lobed; ovary trilocular	**10**
10	Corolla blue, drying pink; sepals recurving, the tips strongly accrescent in fruit; leaves usually 3-lobed	**237. *I. hederacea***
–	Corolla pink; sepals remaining erect, not strikingly accrescent in fruit; leaves entire or lobed	**238. *I. purpurea***
11	Pedicels 10–35 mm long, so inflorescence usually lax; leaves thinly pubescent	**12**
–	Pedicels very short, < 12 mm, so inflorescence dense; leaves densely appressed white-pilose to tomentose abaxially	**177. *I. peteri***
12	Corolla pink; flowers in cymes; stamens included	**261. *I. orizabensis***
–	Corolla pale blue; flowers solitary; stamens exserted (United States)	**262. *I. gilana***
13	Leaves absent at anthesis; corolla white with pink midpetaline bands	**140. *I. pruinosa***
–	Leaves present at anthesis; corolla pink or lilac	**14**
14	Leaves polymorphic, some entire, some digitately 7-lobed	**75. *I. leonensis***
–	Leaves all ± of the same shape, none digitately 7-lobed	**15**
15	Leaves slightly paler abaxially but essentially green on both surfaces	**16**
–	Leaves distinctly discolorous; the abaxial surface whitish and strongly contrasting with the greenish adaxial surface	**20**
16	Leaves small, < 5 cm long and wide, margins undulate or dentate	**17**
–	Leaves mostly > 5 cm long, margins entire	**18**
17	Sepals ovate, acuminate, subequal, all 12–13 mm long	**239. *I. zacatecana***
–	Sepals oblong to oblong-elliptic, obtuse, unequal, the outer 8–10 mm long	**76. *I. rupicola***
18	Sepals oblong-lanceolate three times longer than broad, < 4 mm wide	**416. *I. regnellii***
–	Sepals ovate to elliptic, > 4 mm wide	**19**
19	Flowers in cymes of 3 or more flowers; seeds long-pilose	**73. *I. jalapa***
–	Flowers solitary; seeds densely pubescent	**260. *I. decasperma***
20	Sepals with prominent white margins; leaves deeply 3-lobed	**241. *I. calcicola***
–	Sepals lacking prominent white margins; Leaves entire or shallowly lobed	**21**
21	Perennial herbs; sepals with spreading hairs	**22**
–	Lianas; sepals sericeous with appressed hairs	**23**
22	Outer sepals < 10 mm long, grey-sericeous	**263. *I. leucotricha***
–	Outer sepals > 10 mm long, shortly pilose	**399. *I. rubens***
23	Corolla urceolate, the tube greenish with pinkish midpetaline bands; seeds densely woolly	**24**
–	Corolla funnel-shaped, uniformly pink; seeds pubescent	**25**
24	Sepals 5–7 mm long; corolla 3–3.5 cm long	**81. *I. bombycina***
–	Sepals 11–15 mm long; corolla c. 4.5 cm long	**80. *I. gesnerioides***
25	Bracteoles papery, persistent, 2.5–6 cm long; flowers several in cymes; sepals 12–16 mm long	**393. *I. nervosa***
–	Bracteoles small, caducous; flowers usually solitary, rarely up to 3; sepals strongly accrescent to 4 cm in fruit	**418. *I. tiliifolia***

**Key B10**

Plants not in Keys B1–8 with glabrous sepals < 18 mm long; i.e. sepals without hairs, but some species may have fleshy teeth

**Table:** 

1	Prostrate seaside plant, rooting at the nodes; corolla white; flowers solitary	**388. *I. imperati***
–	Plants of various habits but if maritime, corolla pink, solitary or in cymes	**2**
2	Prostrate seaside plant with pink flowers and large somewhat fleshy rounded to retuse leaves	**339. *I. pes-caprae***
–	Usually twining inland plants but if maritime, not as above	**3**
3	Corolla (buds) sericeous or pubescent	**4**
–	Corolla glabrous on the exterior even in bud	**6**
4	Leaves conspicuously white sericeous on lower surface (Panama)	**394. *I. abutiloides***
–	Leaves green abaxially	**5**
5	Corolla 6–8 cm long, pink; stem always lacking soft spines; flowers numerous; sepals often winged or muricate	**414. *I. pedicellaris***
–	Corolla 2.5–4 cm long, bluish with white tube; stem often with scattered soft spines; flowers few; sepals abaxially smooth, occasionally with a few hairs	**270. *I. parasitica***
6	Sepals aristate with a long attenuate mucro up to 7 mm in length; corolla lilac or bluish, open at night; stem muricate with soft spines	**271. *I. muricata***
–	Sepals varied in shape, sometimes acuminate but not aristate; corolla pink or white, not lilac; stem not muricate with soft spines	**7**
7	Peduncles very short, < 1.5 cm long; corolla dark violet (or creamy); sepals elongate, with scarious margins	**400. *I. lindenii***
–	Peduncles short or long, but if short, corolla not dark violet or creamy, nor sepals elongate with scarious margins	**8**
8	Pedicels very short, < 1.5 cm, calyx concealed or not by leaves or bracteoles	**9**
–	Pedicels at least 1.5 cm long, usually much longer, the calyx exposed	**18**
9	Flowers in compact cymes with small, inconspicuous bracteoles	**10**
–	Flowers solitary or, if numerous, with conspicuous large bracteoles	**12**
10	Leaves abaxially white, sericeous or tomentose	**11**
–	Leaves abaxially green	**305. *I. meyeri***
11	Leaves entire	**175. *I. isthmica***
–	Leaves deeply lobed	**176. *I. eremnobrocha***
12	Bracteoles ovate to suborbicular, spathe-like, completely enclosing the calyx	**13**
–	Bracteoles not spathe-like, calyx concealed by leaves or large bracteoles	**14**
13	Inflorescence pedunculate, axillary	**337. *I. suffulta***
–	Inflorescence terminal on the branches, subsessile	**268. *I. mcvaughii***
14	Corolla white; flowers numerous	**112. *I. riparum***
–	Corolla pink; flowers in cymes of 1–4	**15**
15	Corolla hypocrateriform; stamens exserted, flowers in cymes of 1–5 flowers	**291. *I. dumosa***
–	Corolla funnel-shaped; stamens included; flowers solitary	**16**
16	Leaves partially enclosing the calyx; peduncle and petiole fused basally; leaves 1–6 cm long, acuminate	**292. *I. seducta***
–	Leaves distant from calyx; peduncle and petiole not fused; Leaves < 2 cm long, obtuse	**17**
17	Leaves rounded in outline, the margin with large teeth	**267. *I. noctulifolia***
–	Leaves broadly ovate, margin entire or obscurely dentate	**304. *I. eximia***
18	Leaves with large marginal teeth	**303. *I. ignava***
–	Leaves entire or lobed but lacking marginal teeth	**19**
19	Sepals very unequal in length	**20**
–	Sepals equal or only slightly unequal in length	**26**
20	Flowers solitary (rarely paired); leaves strongly sagittate; corolla gradually widened from a narrow base	**21**
–	Flowers several in cymes, rarely solitary; leaves varied in shape but, if sagittate, corolla not widened as above	**22**
21	Corolla funnel-shaped, 4–7 cm long; sepals oblong-elliptic, 3–7 mm wide, smooth	**351. *I. sagittata***
–	Corolla very narrowly funnel-shaped, 6–10 cm long; sepals lanceolate to oblong, < 3 mm wide, muricate	**301. *I. elongata***
22	Sepals up to 10 mm long, abaxially smooth	**23**
–	Inner sepals 8–15 mm long; outer sepals often transversely muricate	**25**
23	Corolla pink; outermost sepal at least 5 mm long	**380. *I. squamosa***
–	Corolla white; outermost sepal 2–5 mm long	**19**
24	Outer sepals < 3 mm long, obtuse to rounded; corolla 5–6 cm long	**381. *I. anisomeres***
–	Outer sepals 2–5 mm acute; corolla 3.5–4 cm long	**308. *I. puncticulata***
25	Leaves ovate, sagittate; corolla pink 7–8.5 cm long	**346. *I. paludicola***
–	Leaves subreniform, usually hastate; Corolla, white with dark centre or pale pink, 5–6 cm long	**347. *I. asarifolia***
26	Aquatic plant rooting at the nodes; leaves usually hastate or sagittate	**391. *I. aquatica***
–	Terrestrial plants, usually climbing, not rooting at nodes	**27**
27	Flowers solitary, rarely paired	**28**
–	Inflorescence formed of cymes of 3 or more flowers	**31**
28	Sepals with dark blotches, ovate, 3–8 mm long	**29**
–	Sepals lacking dark blotches, oblong or, if ovate > 12 mm long	**30**
29	Corolla 4–6 cm long, reddish-purple with pale tube	**283. *I. miquihuanensis***
–	Corolla 2.5–4.5 cm long, blue	**282. *I. simulans***
30	Sepals oblong to oblong-obovate, < 10 mm long, abaxially smooth; corolla white or pale pink	**133. *I. hartwegii***
–	Sepals ovate, 12–14 mm long, abaxially often with a few teeth; corolla reddish-purple	**132. *I. lozanii***
31	Outer sepals scarious, papery in texture	**218. *I. splendor-sylvae***
–	Outer sepals varied in texture, but not papery	**32**
32	Sepals oblong-deltoid, dark green with white margin; corolla blue with yellowish throat and white tube	**257. *I. tricolor***
–	Sepals and corolla not as above	**33**
33	Sepals thin in texture, flat, conspicuously mucronate	**34**
–	Sepals coriaceous, elliptic, usually obtuse and convex, inconspicuously mucronate	**35**
34	Flowers in a lax cyme	**221. *I. tiliacea***
–	Flowers in a subumbellate pedunculate inflorescence	**220. *I. batatas***
35	Leaves palmately lobed	**157. *I. mauritiana***
–	Leaves entire	**36**
36	Corolla pink	**37**
–	Corolla white	**134. *I. cuprinacoma***
37	Sepals broadly oblong to elliptic, rounded, not more than twice as long as broad, usually < 8 mm long	**145. *I. batatoides***
–	Sepals lanceolate to oblong-lanceolate, c. 3 times longer than broad, usually 8–12 mm long	**266. I. chenopodiifolia subsp. bellator**

### C. Key to Caribbean Island species

**Table:** 

1	Erect undershrub, usually cultivated	**84. I. carnea subsp. fistulosa**
–	Trailing or twining herbs or lianas	**2**
2	Leaves pinnate	**312. *I. quamoclit***
–	Leaves entire, lobed or digitately divided into leaflets	**3**
3	Leaves borne on short brachyblasts, very small, <3 cm long	**4**
–	Leaves not borne on brachyblasts, usually much > 3 cm long	**7**
4	Leaves digitately lobed (Cuba, Jamaica)	**5**
–	Leaves reniform, bilobed, or some leaves trifoliate, the terminal leaflet bilobed (Puerto Rico and Lesser Antilles)	**6**
5	Leaves divided into 3 leaflets; corolla red (Cuba)	**201. *I. microdonta***
–	Leaves divided mostly into 5–7 leaflets; corolla with green tube and pale pink limb (Jamaica)	**204. *I. tenuifolia***
6	Corolla funnel-shaped (Virgin Islands to Barbuda)	**202. *I. eggesiana***
–	Corolla hypocrateriform (Puerto Rico)	**203. *I. steudelii***
7	Leaves palmately divided almost to or completely to the base, the leaflets free or joined only near the base	**8**
–	Leaves entire, or shallowly lobed but, if palmately 3-lobed, divided to not more than three quarters of their length and bracteoles persistent, prominent	**21**
8	Sepals lanceolate, oblong, acute to mucronate, clearly longer than broad, not coriaceous	**9**
–	Sepals elliptic to obovate, occasionally mucronate but never acute, about as broad as long, coriaceous	**12**
9	Corolla 4–5 cm long, pink	**10**
–	Corolla < 3 cm long, pink or creamy yellow	**11**
10	Petioles usually with pseudo-stipules at base; leaflets lanceolate to oblong-lanceolate	**392. *I. cairica***
–	Petioles lacking pseudo-stipules; leaflets linear to narrowly oblong (Cuba, Trinidad)	**378. *I. subrevoluta***
11	Corolla creamy yellow with dark centre; peduncle usually straight; sepals acuminate and mucronate	**383. *I. longeramosa***
–	Corolla pink; peduncle twisted and commonly coiled; sepals obtuse	**374. *I. heptaphylla***
12	Corolla white or greenish-white, sometimes with pale pink lobes	**13**
–	Corolla pink or red	**15**
13	Leaflets filiform; corolla small, < 2 cm long (Hispaniola)	**207. *I. nematoloba***
–	Leaflets relatively broad oblong-elliptic, ovate or elliptic; corolla 3–5 cm long	**14**
14	Leaflets 7.5–14 cm long; leavesalways 3-lobed (Jamaica)	**212. *I. ternata***
–	Leaflets < 7 cm long, leaces 3–5-lobed (Hispaniola)	**215. *I. clausa***
15	Corolla 2.5–4 cm; sepals 4–6 mm long; (Hispaniola)	**16**
–	Corolla > 4 cm long; some or all sepals > 7 mm long	**17**
16	Sepals red-margined; leaflets oblong	**214. *I. digitata***
–	Sepals green margined; leaflets oblanceolate	**213. *I. desrousseauxii***
17	Leaflets completely sessile or partially fused at base; plant cultivated or growing in disturbed places	**18**
–	Leaflets with a short but distinct basal petiole; plants growing in natural situations	**19**
18	Woody liana, cymes commonly compound	**211. *I. horsfalliae***
–	Trailing or climbing herb; cymes usually simple	**157. *I. mauritiana***
19	Corolla 5–6 cm long; leaflets usually broadest towards the base or in the middle, mostly oblong-elliptic (Jamaica)	**210. *I. lineolata***
–	Corolla 4–5 cm long; leaflets mostly oblong, oblanceolate or obovate, rather narrow and broadest near apex	**20**
20	Leaflets up to 6.5 × 2.2 cm long; peduncles stout < 4 cm long (Cuba and Bahamas)	**208. *I. carolina***
–	Leaflets up to 11 × 3.5 cm long; peduncles (Hispaniola)	**209. *I. furcyensis***
21	Corolla pubescent on the exterior (best seen in bud)	**22**
–	Corolla glabrous on the exterior	**30**
22	Weedy annual herb with subsessile cymes, the peduncles < 10 mm long; corolla 7–9 mm long	**398. *I. eriocarpa***
–	Annual or perennial herbs, relatively robust in habit; inflorescence pedunculate; corolla > 2.5 cm long	**23**
23	Sepals 5–7 mm long	**24**
–	Sepals at least 8 mm long, often much more in fruit	**25**
24	Sepals about as broad as long, uniformly pubescent; corolla pink, 6–7 cm long	**84a. I. carnea subsp. carnea**
–	Sepals longer than broad, nearly glabrous but with a few scattered hairs; corolla blue with white tube, 2.5–4 cm long	**270. *I. parasitica***
25	Bracteoles caducous, absent at anthesis; corolla relatively large, > 5 cm long, usually much longer	**26**
–	Bracteoles relatively persistent, conspicuous, 1.5–4 cm long; corolla < 5 cm long	**28**
26	Corolla white, cream or bluish; sepals narrowly ovate, much longer than broad; sepals and leaves usually glabrous (Jamaica)	**400. *I. lindenii***
–	Corolla pink; sepals broadly ovate or ovate elliptic, not much longer than broad; sepals and leaves pubescent or sericeous	**27**
27	Woody liana; flowers solitary (rarely to 3); sepals strongly accrescent in fruit and enclosing the capsule	**418. *I. tiliifolia***
–	Perennial herb; flowers usually in cymes of 3–5 flowers (sometimes more); sepals not strongly accrescent in fruit	**73. *I. jalapa***
28	Leaves borne in fascicles; flowers subsessile, borne on peduncles < 1.5 cm long	**205. *I. lachnaea***
–	Leaves solitary, petiolate; flowers in pedunculate cymes	**29**
29	Bracteoles papery, pale yellow-green; sepals 12–16 mm long, elliptic to obovate (cultivated or an escape)	**393. *I. nervosa***
–	Bracteoles not papery, reddish to mauve in colour; sepals 18–25 mm, ovate to lanceolate (Cuba and Hispaniola)	**354. *I. racemosa***
30	Corolla 8–11 cm long; anthers at least weakly exserted	**31**
–	Corolla < 8 cm long; anthers included or exserted	**32**
31	Sepals lanceolate, terminating in a long awn-like structure	**272. *I. alba***
–	Sepals elliptic to suborbicular, obtuse, sometimes shortly mucronate	**389. *I. violacea***
32	Sepals pubescent or tomentose; perennials with coriaceous, obtuse sepals and densely sericeous or pubescent leaves	**33**
–	Sepals glabrous or, if hirsute, plants annual and weedy, leaves glabrous or pubescent; sepals acute to strongly mucronate	**39**
33	Corolla yellow-green; indumentum of stellate hairs (Hispaniola)	**206. *I. luteoviridis***
–	Corolla pink or purple; indumentum of unbranched hairs (Cuba)	**34**
34	Stamens strongly exserted; corolla hypocrateriform	**35**
–	Stamens included; corolla funnel-shaped	**37**
35	Plant leafless at anthesis; inflorescence of axillary clusters; sepals reddish, pubescent near base only (Cuba)	**193. *I. praecox***
–	Plant leafy at anthesis; onflorescece cymose; sepals uniformly tomentose, grey	**36**
36	Leaves basally subcordate; bracteoles linear-lanceolate, not foliose (Cuba	**195. *I. jalapoides***
–	Leaves basally cuneate; bracteoles obovate to oblanceolate, foliose (Cuba)	**192. *I. argentifolia***
37	Leaves 3-lobed (Cuba)	**188. *I. passifloroides***
–	Leaves entire	**38**
38	Sepals pubescent only near base; flowers several in cymes (Cuba)	**196. *I. montecristina***
–	Sepals uniformly tomentose; flowers solitary (Cuba)	**194. *I. calophylla***
39	Sepals elliptic to obovate, obtuse to rounded, coriaceous, glabrous; plants perennial	**40**
–	Sepals varied, usually lanceolate, ovate or oblong, acute to acuminate, often mucronate, glabrous or hirsute; plants annual or perennial	**54**
40	Corolla greenish-yellow to white	**41**
–	Corolla red, purple or pink	**45**
41	Leaves dentate, abaxially pubescent (Cuba)	**186. *I. erosa***
–	Leaves entire or lobed but not dentate; glabrous	**42**
42	Stamens exserted; leaves lobed with acute lobes (Cuba)	**184. *I. cubensis***
–	Stamens included; leaves entire or variously lobed	**43**
43	Corolla 3.5–6 cm long; seeds with long marginal hairs	**44**
–	Corolla 1.5–1.7 cm long; seeds uniformly pilose (Cuba)	**185. *I. merremioides***
44	Leaves ovate to ovate elliptic, rarely shallowly lobed (Cuba)	**183. *I. alterniflora***
–	Leaves usually deeply lobed or palmately divided into leaflets but if entire, ovate-deltoid (Hispaniola)	**215. *I. clausa***
45	Leaves pubescent or sericeous	**46**
–	Leaves glabrous	**49**
46	Leaves green, pubescent or pilose abaxially; sepals often reddish	**47**
–	Leaves silvery sericeous abaxially; sepals not reddish	**48**
47	Leaves large, 4–16 cm long; peduncles 3–7 cm long	**190. *I. clarensis***
–	Leaves small, 1.2–5.5 cm long; peduncles < 0.6 cm long	**197. *I. fuchsioides***
48	Leaves large 5–12 cm long, cordate, sericeous below but not silvery; sepals completely glabrous	**189. *I. hypargyreia***
–	Leaves up to 6.5 cm long, cuneate to weakly cordate, silvery; sepals pubescent near base	**196. *I. montecristina***
49	Stamens included	**50**
–	Stamens exserted	**51**
50	Stem, peduncles and petioles with conspicuous squamose glands (Eastern Cuba)	**187. *I. balioclada***
–	Plant lacking conspicuous squamose black glands (Western Cuba	**183. *I. alterniflora***
51	Corolla limb deeply divided into oblong lobes (Hispaniola)	**199. *I. repanda***
–	Corolla limb entire or undulate	**52**
52	Leaves oblong, mostly absent at anthesis; flowers in dense clusters (Cuba)	**191. *I. incerta***
–	Leaves of varied shape, present at anthesis; flowers in lax cymes	**53**
53	Leaves wedge-shaped (St. Eustatius)	**200. *I. sphenophylla***
–	Leaves usually ovate, somewhat polymorphic (Cuba, Bahamas, Florida)	**198. *I. microdactyla***
54	Corolla hypocrateriform; stamens exserted	**55**
–	Corolla funnel-shaped or campanulate; stamens included	**56**
55	Sepals c. 3 mm long with a subterminal awn of similar length	**321. *I. hederifolia***
–	Sepals 10–15 mm long, without a prominent subterminal awn (Jamaica)	**235. *I. jamaicensis***
56	Trailing plants rooting at the nodes growing in wet places near the sea or in and around cultivation	**57**
–	Twining, climbing or trailing plants, not rooting at the nodes and not usually found in wet places or on sea shores	**61**
57	Leaves ovate, suborbicular, linear, oblong, rectangular or 5-lobed, not, or scarcely, basally cordate; seashore plants	**58**
–	Leaves lanceolate, ovate, subreniform or suborbicular but with cordate or sagittate base, plants of freshwater or dry habitats	**59**
58	Leaves shortly oblong, linear, lanceolate or 3–5-lobed, small, 1.5–3 × 0.8–2 cm; sepals very unequal; corolla white, 3.5–4 cm long	**388. *I. imperati***
–	Leaves ovate to suborbicular, rounded or emarginate, 3.5–9 × 3–10 cm; sepals subequal; corolla pink, 4–5 cm long	**339. *I. pes-caprae***
59	Sepals strongly mucronate, usually ciliate or pilose; plant of cultivation or waste ground	***I. batatas***
–	Sepals not mucronate, glabrous; plants usually of wetland	**60**
60	Sepals subequal, smooth; leaves acuminate, sagittate or hastate	**391. *I. aquatica***
–	Sepals very unequal, often transversely muricate; leaves rounded, obtuse or acute, never acuminate	**347. *I. asarifolia***
61	Sepals with prominent abaxial muricate ribs; bracteoles prominent, 8–20 × 3–15 mm	**62**
–	Sepals abaxially smooth; bracteoles prominent or not	**63**
62	Annual herb; corolla 2.5–3.5 cm long	**341. *I. fimbriosepala***
–	Perennial herb; corolla 5.5–8 cm long	**342. *I. setifera***
63	Flowers grouped into bracteolate clusters	**64**
–	Inflorescence clearly cymose, but, if clustered, bracteoles caducous	**65**
64	Corolla 2–3 cm long; stigma bilobed; capsule 4-seeded	**305. *I. meyeri***
–	Corolla 5–6 cm; stigma trilobed; capsule 6-seeded	**234. *I. indica***
65	Sepals more than 10 × 10 mm in size, commonly reddish; plant a vigorous liana	**352. *I. philomega***
–	Sepals < 10 mm wide, not reddish; plant herbaceous	**66**
66	Sepals glabrous	**67**
–	Sepals hirsute, or at least ciliate	**74**
67	Corolla white or cream, rarely bluish; sepals oblong or oblong-lanceolate	**68**
–	Corolla pink or blue; Sepals variable in shape	**69**
68	Corolla campanulate, 2.5–3 cm long; sepals oblong, nearly completely scarious, < 15 mm long	**403. *I. corymbosa***
–	Corolla funnel-shaped, 5–6 cm long; sepals oblong-ovate, scarious only on the margins, often exceeding 14 mm (Jamaica)	**400. *I. lindenii***
69	Sepals < 11 mm long, equal in length or nearly so	**70**
–	Sepals > 12 mm long or if less, very unequal in length	**71**
70	Sepals lanceolate, acute but not mucronate, scarious-margined; corolla blue with white tube and yellowish throat (cultivated or an escape)	**257. *I. tricolor***
–	Sepals oblong or oblong-ovate, conspicuously mucronate, not scarious-margined; corolla pink, often with a dark centre	**221. *I. tiliacea***
71	Flowers usually solitary; leaves strongly sagittate to hastate	**72**
–	Flowers usually several in cymes, very rarely solitary; leaves cordate or sagittate	**73**
72	Sepals lanceolate, 17–21 mm long, acuminate, subequal with prominent longitudinal veins (Netherlands Antilles)	**355. *I. incarnata***
–	Sepals oblong-elliptic, rounded, < 12 mm long, unequal in size, not prominently veined	**351. *I. sagittata***
73	Corolla bluish; peduncles short, usually < 1.5 cm; sepals narrowly ovate acute to acuminate (Jamaica)	**400. *I. lindenii***
–	Corolla usually pink or pale pink; peduncles 4–12 cm; sepals obovate to suborbicular, (Hispaniola, Trinidad)	**380. *I. squamosa***
74	Corolla white, yellow or cream, sometimes with a dark centre	**75**
–	Corolla pink, blue or purplish	**77**
75	Ovary and capsule pilose; corolla white	**224. *I. lacunosa***
–	Ovary and capsule glabrous; corolla yellowish, sometimes with a dark centre	**76**
76	Corolla large, 3–4 cm long	**412. *I. ochracea***
–	Corolla small, 1.5–2.5 cm long	**413. *I. obscura***
77	Sepals obtuse, acute or acuminate but not mucronate; stigma 3-lobed; capsule 6-seeded, glabrous	**78**
–	Sepals oblong or lanceolate, distinctly mucronate; stigma 2-lobed; capsule 4-seeded, often pilose	**80**
78	Corolla pink (rarely white or blue); sepals oblong-lanceolate, obtuse or acute; leaves entire or 3–5-lobed	**238. *I. purpurea***
–	Corolla blue with a white tube (drying pink): sepals ovate with an elongate apex, notably accrescent in fruit	**79**
79	Corolla < 3.5 cm long; sepals < 2 cm long at anthesis, the tips recurving; peduncle very short	**237. *I. hederacea***
–	Corolla 4–4.5 cm long; sepals c. 3 cm long at anthesis, the tips erect; peduncles long or short	**236. *I. nil***
80	Corolla < 2.5 cm long; plants annual, always slender	**81**
–	Corolla > 2.5 cm long; plants perennial or annual, usually relatively robust	**82**
81	Sepals oblong, 5–6 mm long	**229. *I. triloba***
–	Sepals lanceolate, 10–14 mm long	**225. *I. leucantha***
82	Slender, 1–2-flowered herb with pubescent strap-shaped sagittate leaves (Cuba, Florida, Hispaniola, Mona Island)	**232. *I. tenuissima***
–	Slender or robust herbs, 1–many-flowered; leaves not strap-shaped, rarely sagittate, but, if so, completely glabrous	**83**
83	Sepals oblong-lanceolate; sepals chartaceous even at anthesis, unequal, the outer shorter than the inner	**219. *I. trifida***
–	Sepals obovate, ovate or elliptic; sepals not chartaceous at anthesis, equal in length or nearly so	**84**
84	Annual herb, not rooting at nodes; cymes always lax and few-flowered, never umbellate in form	**226. *I. cordatotriloba***
–	Perennial herb, often decumbent and rooting at the nodes; cymes compact, umbellate or subcapitate in form	**220. *I. batatas***

### D. Key to Hawaiian species

**Table:** 

1	Leaves pinnate	**312. *I. quamoclit***
–	Leaves simple or palmately lobed	**2**
2	Erect undershrub to c. 3 m; corolla pubescent	**84b. I. carnea subsp. fistulosa**
–	Trailing or twining herbs; corolla glabrous except in. *I. tiliifolia*	**3**
3	Leaves 5-lobed to or near the base	**4**
–	Leaves entire or shallowly 3-(5)-lobed	**5**
4	Woody liana; leaves lacking pseudo-stipules; corolla orange-red	**211. *I. horsfalliae***
–	Twining herb; Leaves with cnspicuous pseudo-stipules; corolla pink	**392. *I. cairica***
5	Corolla hypocrateriform, red, white or pale blue; stamens exserted or held at corolla mouth; twining plants	**6**
–	Corolla funnel-shaped, pink, yellowish or white, stamens included; twining or prostrate plants	**8**
6	Corolla red; leaves usually shallowly lobed	**321. *I. hederifolia***
–	Corolla white or pale blue, usually entire	**7**
7	Sepals terminating in a prominent awn 5–12 mm in length; habitats with fresh water	**272. *I. alba***
–	Sepals obtuse, sometimes mucronulae; saline habitats	**389. *I. violacea***
8	Corolla yellowish, white or lilac tinged	**9**
–	Corolla pink, sometimes with a dark centre	**11**
9	Corolla yellowish; capsule rostrate; twining anual herb	**413. *I. obscura***
–	Corolla white or lilac tinged; usually trailing perennial herbs	**10**
10	Creeping seashore plant, rooting at the nodes; leaves linear to oblong usually basally truncate	**388. *I. imperati***
–	Prostrate or twining plant of lava flows; leaves simple or lobed but characteristically cordate at base	**264. *I. tuboides***
11	Leaves rounded to retuse; creeping seahore species	**339. *I. pes-caprae***
–	Leaves obtuse, acute or acuminate; plants of varied hábitats	**12**
12	Corolla pubescenrtin bud; leaves grey-tomentose when young, dotted with black glands beneath; sepals strongly accrescent and enclosing the capsule	**418. *I. tiliifolia***
–	Corolla glabrous; leaves eglandular, rarely grey-tomentose, not gland-dotted beneath; sepals not strongly accrescent in fruit	**13**
13	Creeping freshwater aquatic herb	**391. *I. aquatica***
–	Twining or prostrate herb, but if creeping, not growing in freshwater aquatic habitats	**14**
14	Stigma 3-lobed; sepals obtuse to acute but not mucronate	**15**
–	Stigma bilobed; sepals mucronate	**16**
15	Flowers clustered in a subcapitate bracteolate inflorescence; pedicels very short	**234. *I. indica***
–	Flowers in lax cymes; pedicels > 10 mm long; bracteoles linear, inconspicuous	**238. *I. purpurea***
16	Twining annual herb; corolla < 2.5 cm long	**229. *I. triloba***
–	Perennial herb, usually prostrate; corolla > 2.5 cm long	**17**
17	Flowers in subumbellate pedunculate clusters; sepals usually ciliate; plant often pubescent; cultivated or escaped from cultivation	**220. *I. batatas***
–	Flowers in 1–3-flowered cymes; sepals and leaves glabrous or nearly so; native species of seashores or near the sea	**222. *I. littoralis***

## Taxonomic account

### Ipomoea

Taxon classificationPlantaeSolanalesConvolvulaceae

L. Sp. Pl. 1: 159. 1753 nom. cons.

Acmostemon Pilg., Notizbl. Bot. Gart. Berlin-Dahlem 13: 106. 1936. (Pilger 1936: 106). Type. Acmostemon angolensis Pilg. (= Ipomoea viscoidea Choisy).
Adamboe Raf., Fl. Tellur. 4: 79. 1836 [pub. 1838]. (Rafinesque 1838a: 79). Type. Adamboe bicolor Raf. (= Ipomoea campanulata L.).
Amphione Raf., Fl. Tellur. 4: 79. 1836 [pub. 1838]. (Rafinesque 1838a: 79). Type. Amphione lobata Raf. (= Ipomoea triloba L.).
Apopleumon Raf., Fl. Tellur. 4: 72. 1836 [pub. 1838]. (Rafinesque 1838a: 72). Type. Apopleumon bignonioides (Sims) Raf. (= Ipomoea bignoniodes Sims).
Argyreia Lour., Fl. Cochinch. 1: 95, 134. 1790. (Loureiro 1790: 134). Type. Argyreia obtusifolia Lour. (= Ipomoea obtusifolia (Lour.) J.R.I. Wood & Scotland).
Argyryon St.-Lag., Ann. Soc. Bot. Lyon. 7: 120. 1880. (Saint-Lager 1880: 120). Orth. Variant of Argyreia.
Astripomoea A. Meeuse, Bothalia 6: 709. 1958. (Meeuse 1958: 709). Type. Based on Astrochlaena Hallier f.
Astrochlaena Hallier f., Bot. Jahrb. Syst. 18: 120. 1894 [pub. 1893]. (Hallier 1893b: 120), nom. illeg., non Astrochlaena Corda (1845). Type. Astrochlaena lachnosperma (Choisy) Hallier f., lectotype designated by Meeuse, Taxon 4: 199. 1955. (= Ipomoea lachnosperma Choisy).
Batatas Choisy, Mém. Soc. Phys. Genève 6: 434 [52]. 1833 [pub. 1834]. (Choisy 1834: 434). Type. Batatas edulis (Thunb.) Choisy, lectotype designated by Roberty, Boissiera 10: 147 (1964). (= Ipomoea batatas (L.) Lam.).
Blinkworthia Choisy, Mém. Soc. Phys. Genève 6: 430 [48]. 1833 [pub. 1834]. (Choisy 1834: 430). Type. Blinkworthia lycioides Choisy (= Ipomoea lycioides (Choisy) J.R.I. Wood & Scotland).
Bombycospermum J. Presl, Reliq. Haenk. 2: 137.1835. (Presl 1831–5: 137). Type. Bombycospermum mexicanum J. Presl (= Ipomoea bombycina (Choisy) Benth. & Hook f.).
Bonanox Raf., Ann. Gén. Sci. Phys. 8: 272. 1821. (Rafinesque 1821: 272). Type. Ipomoea bonanox (=Ipomoea alba L.).
Calboa Cav., Icon. 5: 51. 1799. (Cavanilles 1799: 51). Type. Calboa vitifolia Cav. (= Ipomoea neei (Spreng.) O’Donell).
Calonyction Choisy, Mém. Soc. Phys. Genève 6: 441. 1833. [pub. 1834]. (Choisy 1834: 441) Type. Based on Bonanox Raf.
Calycanthemum Klotzsch in W.C.H. Peters, Naturw. Reise Mossambique 6 (Bot. 1): 243. 1861. (Peters 1861: 243). Type. Calycanthemum leucanthemum Klotzsch (= Ipomoea leucanthemum (Klotzsch) Hallier f.).
Cleiemera Raf., Fl. Tellur. 4: 77. 1836 [pub. 1838]. (Rafinesque 1838a: 77). Type. Several syntypes cited including Cleimera hederacea (Jacq.) Raf. (= Ipomoea hederacea Jacq.).
Cleiostoma Raf., Fl. Tellur. 4: 80. 1836 [pub. 1838]. (Rafinesque 1838a: 80). Type. Cleiostoma villosum Raf. (= Ipomoea pes-tigridis L.).
Clitocyamos St.-Lag., Ann. Soc. Bot. Lyon 7: 128. 1880 (Saint-Lager 1880: 128). Type. Clitocyamos pinnatifidus Raf. (= Ipomoea quamoclit L.)
Coiladena Raf., Fl. Tellur. 2: 12 1836 [pub.1837]. (Rafinesque 1837: 12). Type. Coiladena hyemalis Raf. = Ipomoea sp. incert.
Cryptanthela Gagnep., Notul. Syst. (Paris) 14: 24. 1950. (Gagnepain 1950: 24). Type. Cryptanthela sericea Gagnep. (= Ipomoea thorelii Gagnep.)
Decaloba Raf., Fl. Tellur. 4: 76 1836 [pub. 1838]. (Rafinesque 1838a: 79). Type. Several syntypes cited, including Decaloba mutabilis (Ker-Gawl) Raf. = Ipomoea indica (Burm.) Merrill
Diatremis Raf., Ann. Gén. Sci. Phys. 8: 271. 1821. (Rafinesque 1821: 271). Type. Convolvulus nil L. (= Ipomoea nil (L.) Roth).
Dimerodiscus Gagnep., Notul. Syst. (Paris) 14: 25. 1950. (Gagnepain 1950: 25). Type. Dimerodiscus fallax Gagnep. (= Ipomoea polymorpha Roem. & Schult.).
Doxema Raf., Fl. Tellur. 4: 75. 1836 [pub. 1838]. (Rafinesque 1838a: 75). Type. Doxena sanguinea (Vahl) Raf. (= Ipomoea hederifolia L.).
Elythrostamna Bojer ex Desjardins, Rapp. Annuel Trav. Soc. Hist. Nat. île Maurice 1: 31. 1836. (Desjardins 1836: 31). Type. Elythrostamna convolvulacea Bojer ex Desjardins (= Ipomoea pileata Roxb.).
Euryloma Raf., Fl. Tellur. 4: 75. 1836 [pub. 1838]. (Rafinesque 1838a: 75). Type. Convolvulus grandiflorus L. (= Ipomoea alba L.).
Exallosis Raf., Fl. Tellur. 4: 83 1836 [pub.1838]. (Rafinesque 1838a: 83). Type. Exallosis biflora (L.) Raf. (= Ipomoea biflora (L.) Pers.).
Exocroa Raf., Fl. Tellur. 4: 80. 1836 [pub. 1838]. (Rafinesque 1838a: 80). Type. Exocroa egyptiaca Raf. (= Ipomoea cairica (L.) Sweet).
Exogonium Choisy, Mém. Soc. Phys. Genève 6: 443. 1834. (Choisy 1834: 443). Type. Exogonium bracteatum (Cav.) Choisy ex G. Don, lectotype designated by House, Bull. Torrey Bot. Club 35: 98. (1908). (= Ipomoea bracteata Cav.).
Fraxima Raf., Fl. Tellur. 4: 83. 1836 [pub. 1838]. (Rafinesque 1838a: 83). Type. Fraxima ebractea (Desr.) Raf. (= Ipomoea ebracteata (Desr.) Choisy).
Gynoisa Raf., Fl. Tellur. 4: 75 1836 [pub. 1838]. (Rafinesque 1838a: 75). Type. Gynoisia carolina (L.) Raf. (= Ipomoea cordatotriloba Dennst.).
Kolofonia Raf., Fl. Tellur. 4: 73 1836 [pub. 1838]. (Rafinesque 1838a: 73). Type. Kolofonia albivenia (Lindl.) Raf. (= Ipomoea albivenia Lindl.)
Latrienda Raf. Fl. Tellur. 4: 81. 1836 [pub. 1838]. (Rafinesque 1838a: 81). Type. Various syntypes cited including Latrienda soldanella (L.) Raf. (= Calystegia soldanella (L.) R.Br. and Latrienda imperati (Vahl) Raf. (=Ipomoea imperati (Vahl) Griseb.)
Legendrea Webb & Berth, Hist. Nat. Iles. Canar., Bot. 3, 2: 26. 1844. (Webb and Berthelot 1844–50 : 26). Type. Legendrea mollissima Webb. & Berthel. (= Ipomoea corymbosa (L.) Roem. & Schult.).
Lepistemon Blume, Bijdr. Fl. Ned. Ind. 722. 1826. (Blume 1826: 722). Type. Lepistemon flavescens Blume (= Ipomoea binectarifera (Wall.) J.R.I. Wood & Scotland).
Lepistemonopsis Dammer, Pflanzenw. Ost-Afrikas C: 331. 1895. (Engler 1895). Type. Lepistemonopsis volkensii Dammer (= Ipomoea volkensii (Dammer) J.R.I. Wood & Scotland).
Lettsomia Roxb., Fl. Ind. 2: 75. 1824. (Roxburgh 1824: 75). Type. Lettsomia cuneata (Willd.) Roxb., designated by Pfeiffer (1874: 95). (= Ipomoea cuneata (Willd.) J.R.I. Wood & Scotland).
Marcellia Choisy, Mém. Soc. Phys. Genève 10: 443. 1844. (Choisy: 1844: 443). Type. Marcellia villosa Mart. ex Choisy (= Ipomoea marcellia Meisn.).
Melascus Raf., Fl. Tellur. 4: 81 1836 [pub. 1838]. (Rafinesque 1838a: 81). Type. Melascus latifolius Raf. (= Ipomoea alba L.).
Mina Cerv. in La Llave & Lexarza, Nov. Gen. Descr. 1: 3. 1824. (La Llave and Lexarza 1824: 3). Type. Mina lobata Cerv. (= Ipomoea lobata (Cerv.) Thell.).
Modesta Raf., Fl. Tellur. 4: 76. 1836 [1838]. (Rafinesque 1838a: 76). Type. Modesta paniculata (L.) Raf. (= Ipomoea mauritiana Jacq.).
Moorcroftia Choisy, Mém. Soc. Phys. Genève 6: 431. 1833 [pub.1834]. (Choisy 1834: 431). Type. Moorcroftia pinangiana Choisy, lectotype designated by Roberty (1964: 143) = Ipomoea pinangiana (Choisy) J.R.I. Wood & Scotland.
Navipomoea Roberty, Boissiera 10: 147. 1964. . (Roberty 1964: 147). Type. Navipomoea involucrata (P. Beauv.) Roberty (= Ipomoea involucrata P. Beauv.).
Neorthosis Raf., Fl. Tellur. 4: 125 1836 [pub. 1838]. (Rafinesque 1838a: 125). Type. Not clearly cited, possibly Neorthosis coccinea (L.) Raf. (= Ipomoea coccinea L.).
Ornithosperma Raf., Fl. Ludov.: 149. 1817. (Rafinesque 1817: 149). Type. Ornithosperma serotina (DC.) Raf. (= Ipomoea orizabensis (G. Pelletan) Ledeb. ex Steud.).
Paralepistemon Lejoly & Lisowski, Bull. Jard. Bot. Belg. 56: 196. 1986. (Lejoly and Lisowski 1986: 196). Type. Paralepistemon shirensis (Oliv.) Lejoly & Lisowski (= Ipomoea shirensis Oliv.).
Pentacrostigma K. Afzel., Svensk. Bot. Tidskr. 23: 181. 1929. (Afzelius 1929: 181). Type. Pentacrostigma nyctanthum K. Afzel. (= Ipomoea longituba Hallier f.).
Pharbitis Choisy, Mém. Soc. Phys. Genève 6: 438. 1833 [pub.1834]. (Choisy 1834: 438). Type. Pharbitis hispida (Zuccagni) Choisy (= Ipomoea purpurea (L.) Roth).
Plesiagopus Raf., Fl. Tellur. 4: 78. 1836 [1838]. (Rafinesque 1838a: 78). Type. Convolvulus pes-caprae L. (= Ipomoea pes-caprae (L.) R.Br.).
Pseudipomoea Roberty, Boissiera 10: 147. 1964. (Roberty 1964: 147). Type. Pseudipomoea repens (L.) Roberty (= Ipomoea repens (L.) Lam.).
Quamoclit Mill., Gard. Dict. Abr. ed. 4(3). 1754. (Miller 1754). Type. Based on Ipomoea quamoclit L.
Quamoclita Raf., Fl. Tellur. 4: 74. 1836 [pub. 1838]. (Rafinesque 1838a: 74). Type. Various heterogeneous species cited.
Rivea Choisy, Mém. Soc. Phys. Genève 6: 407. 1833 [pub.1834]. (Choisy 1834: 407). Type. Rivea hypocrateriformis (Desr.) Choisy. Lectotype designated by Manitz (1976: 313). (= Ipomoea hypocrateriformis (Desr.) J.R.I. Wood & Scotland)
Samudra Raf., Fl. Tellur. 4: 72 1836 [pub. 1838]. (Rafinesque 1838a: 72). Type. Samudra speciosa (L.f.) Raf. (= Ipomoea nervosa (Burm. f.) J.R.I. Wood & Scotland)
Stictocardia Hallier f., Bot. Jahrb. Syst. 18: 159. 1894 [pub. 1893]. (Hallier 1893b: 159). Type. Stictocardia tiliifolia (Desr.) Hallier f. (= Ipomoea tiliifolia (Desr.) Roem. & Schult.)
Stomadena Raf., Fl. Tellur. 2: 12 1836 [pub.1837]. (Rafinesque 1837: 12). Type. Stomadena violacea Raf. (= Ipomoea sp. incert.)
Tereietra Raf., Fl. Tellur. 4: 124 1836 [pub.1838]. (Rafinesque 1838a: 124). Type. Tereietra violacea (L.) Raf. (= Ipomoea violacea L.)
Tirtalia Raf., Fl. Tellur. 4: 71. 1836 [pub. 1838]. (Rafinesque 1838a: 71). Type. Various syntypes cited.
Turbina Raf., Fl. Tellur. 4: 81. 1836 [pub. 1838]. (Rafinesque 1838a: 81). Type. Turbina corymbosa (L.) Raf. (= Ipomoea corymbosa (L.) Roth ex Roem. & Schult.).

#### Type.

*Ipomoea pes-tigridis* L.

#### Description.

Annual or perennial herbs, subshrubs, lianas, shrubs or small trees, very varied in habit but, most commonly, twining, less commonly decumbent or erect; vegetative parts glabrous or variously hirsute. Leaves without true stipules, alternate, usually petiolate, entire, lobed or compound with separate leaflets; pseudostipules sometimes present. Inflorescence characteristically of axillary cymes, but sometimes very dense and subcapitate or reduced to single flowers or corymbose to foliose paniculate in form, or subterminal and racemose to spicate in erect species; peduncles variable in length, rarely absent; bracts usually indistinguishable from leaves except in species with a terminal inflorescence; bracteoles very small to large, persistent or caducous, scarious, chartaceous or foliaceous, occasionally forming an involucre; pedicels short or long, rarely absent; calyx of five equal or unequal sepals, very variable in texture, coriaceous, herbaceous, scarious, persistent, often enlarging in fruit; corolla ±often showy, small or (usually) large, commonly funnelform, sometimes hypocrateriform, campanulate or suburceolate, pink or white with 5 distinct darker midpetaline bands, the limb distinct from the tube; stamens 5, usually included, equal or unequal in length, dilated and glandular-pilose at base, inserted near base of corolla tube; anthers usually narrowly oblong; pollen spheroidal, pantoporate, echinulate, the grains relatively large; disc annular, ovary 2(–5) locular, 4 (–10)-ovulate, glabrous or pubescent; style simple, filiform; stigma subglobose, 2(–3)-lobed, rarely (*Astripomoea* and some species in the Arborescens Clade) lobes somewhat elongate. Fruit a globose, 4 (–10) valved capsule or indehiscent; seeds (1–)4–6(–10), triquetous, ovoid or subglobose, glabrous or variously hirsute.

#### Distribution.

A mainly tropical genus, which is almost absent from temperate regions. In its widest circumscription (that is including *Argyreia* and *Stictocardia*), it is about equally common in all three tropical regions although the greatest numbers are found in the Americas. A feature of the genus is the existence of a group of around 30 species which are pantropical in distribution, many as the result of early or prehistoric dispersal. There are significant numbers of endemic species on some large islands including Cuba, Hispaniola, Madagascar and Australia but endemics on small islands or island groups are uncommon.

••• Clade A. (Species 1–233). This enormous clade includes over half the species found in the Americas. There is no obvious morphological character that unites the clade but it divides into three smaller clades. Species in the first two of these, Clade A1 (species 1–127) and Clade A2 (Species 128–215), appear always to have pollen with relatively few echinulae (Figure 9A–C) while that of Clade A3 (Species 218–232) has more numerous echinulae (Figure 10A, B). In addition, there are 3 species within clade A that lie outside Clades A1–3. These are *Ipomoea setosa* and *I. peruviana* (species 216–217) that are sister to each other and together sister to Clades A1–2. Lastly, *Ipomoea cryptica* (Species 233) is sister to Clade A3. Figure 1.

•• Clade A1 (Species 1–127) is very heterogenous morphologically although notable for the absence of annual species and of species with a hypocrateriform corolla and exserted stamens. It includes a number of smaller clades, which are indicated in the text, as well as the following major, principally South American, radiation, which we refer to as the Jalapa radiation after its most widespread species.

• The Jalapa radiation (Species 1–83) is centred on Paraguay, Bolivia, southern Brazil and the extreme north of Argentina. It is very poorly represented in North America. The exact boundaries of the radiation are unclear but evidence suggests that *Ipomoea carnea* (Species 84) and subsequent species should be excluded (Muñoz Rodríguez et al. 2019). Species in this radiation are very varied in habit but the corolla is always pubescent on the exterior. The sepals are usually ±flat, somewhat soft in texture and pubescent.

The radiation appears to be actively evolving and there are several clusters of species, which are difficult to delimit or are bridged by intermediates. To date molecular studies have not shed much light on these relationships or on species monophyly. Most species are unresolved with samples of some species, notably *Ipomoea malvaeoides* and *I. hirsutissima* appearing in several places, although in other cases samples from multiple accessions indicate monophyletic species. Results from the few species for which we have extensive sequence data confirm some species relationships suggested by morphology such as *Ipomoea malvaeoides* with *I. paludosa*, or *I. argentinica* with *I. longibarbis* but raise serious questions over others that are suggested by morphology, such as *I. megapotamica* with both *I. hieronymi* and *I. opulifolia* or *I. nitida* with *I. psammophila*.

### 1. Ipomoea stuckertii

Taxon classificationPlantaeSolanalesConvolvulaceae

O’Donell, Lilloa 14: 188. 1948. (O’Donell 1948a: 188)

#### Type.

ARGENTINA. Córdoba, Dept. Tulumba, *B. Balegno* 1199 (lectotype LIL001355, designated here; isolectotype LIL).

#### Description.

Perennial with napiform rootstock and usually trailing, rarely twining, lanate stems, which become sparsely pilose when old. Leaves petiolate, 2.5–11 × 2.5–8 cm, deeply palmatisect with 6–9 narrowly elliptic to oblanceolate crenate acute lobes, both surfaces tomentose to thinly pilose, base cuneate; petioles 2.5–4 cm, white-pubescent. Flowers 1–3 in axillary, pedunculate cymes; peduncles 7–18 mm, pubescent; bracteoles deltoid. 2–3 mm long, caducous; pedicels 2–10 mm, pubescent; sepals subequal, 8–11 × 4–6 mm, oblong-elliptic, obtuse, white-pubescent, the inner with glabrous margins; corolla 3.5–6 cm long, funnel-shaped, pink, glabrous or with a few short hairs in bud, limb c. 2.5 cm diam. Capsules 15 × 15 mm, subglobose, rostrate; seeds 7–8 mm, long-pilose.

#### Illustration.

O’Donell (1959b: 143).

#### Distribution.

Endemic to the sub-Andean region of NW Argentina, growing on rocky mountains at around 1000 m, apparently most common in Córdoba.

**ARGENTINA. Catamarca**: La Paz, *J. Brizuela* 108 (P). **Córdoba**: sine data, *E. Fielding* (BM); camino de Carlos Paz a Pampa de Achala, 12 km antes de Copina, *A.L. Pastore* 367 (P, SI, US); Copina, *A. Burkart* 7460 (SI); San Alberto, *T. Stuckert* 10762 (CORD). **San Luis**: Ayacucho, Ruta 146 a S de Luján, *R. Kiesling* 4736 (SI); *C. Galander* s.n. [15/3/1882] (CORD). **Santiago del Estero**: Choya, *A.T. Hunziker & A.E. Cucucci* 17909 (CORD).

#### Note.

The palmatisect leaves, lanate stems and pubescent sepals are distinctive.

### 2. Ipomoea padillae

Taxon classificationPlantaeSolanalesConvolvulaceae

O’Donell, Lilloa 29: 207. 1959. (O’Donell 1959b: 207)

#### Type.

ARGENTINA. Misiones, Dept. Candelaria, Gramajo, *G.J. Schwarz* 5552 (lectotype LIL001267, designated here; isolectotypes LIL, P, S, SI).

#### Description.

Prostrate perennial herb; stems trailing, several metres long, pilose, glabrescent. Leaves petiolate, 3–17 × 3–20 cm, 3–7-palmatilobed, the segments elliptic to obovate, narrowed towards the base, apex obtuse and mucronate, base shallowly cordate, both surfaces thinly pubescent, the lower sometimes sericeous; petioles 1–11 cm. Inflorescence of 1–8-flowered, axillary, pedunculate often compounded cymes; peduncles 2–18 cm long; bracteoles 3–5 mm long, lanceolate, caducous; secondary peduncles 1.5–5 mm; pedicels 9–30 mm long; sepals 7.5–10 × 4–6 mm, subequal, ovate, acute and mucronate, sericeous, the inner with glabrous, scarious margins; corolla 5.5–8 cm long, pink, funnel-shaped, sericeous, limb c. 4 cm diam. Capsules subglobose, 7–8 mm wide, glabrous; seeds not seen.

#### Illustration.

O’Donell (1959b: 210).

#### Distribution.

An uncommon plant of degraded cerrado in NE Argentina (Misiones) and neighbouring Rio Grande do Sul in Brazil.

**ARGENTINA. Misiones**: Leandro, *A. Krapovikas et al*. 15023 (CTES); Candelaria, Posadas-Bonpland, *W.A. Archer* 4611 (US); Ruta Nacional 12.2 km del peaje, *M.E. Rodríguez & A. Gachez* (CTES, FCQ); Apóstoles, *H. Keller & Franco* 4907 (CTES); Concepción, *H. Keller & Franco* 5732 (CTES).

**BRAZIL. Rio Grande do Sul**: Roque Gonzales, Rincão Vermelho, *P.P.A. Ferreira & J. Durigon* 590 (S); São Borja caminho para Garruchos, *P.P.A. Ferreira & J. Durigon* 582 (CTES).

#### Notes.

The palmatilobed pubescent leaves and sericeous exterior of the corolla help to identify this species.

*S. Heinonen et al.* 117 (CTES) collected in Corrientes, Dept. Ituzaingo at Puerto Valle may represent an undescribed related species. It has trifurcate, thinly appressed pilose leaves divided to near the strongly truncate base. The leaf lobes are oblong, 3–5.5 × 0.5–1.2 cm, the flowers are solitary, borne on a 3–4 cm long, pubescent peduncle with caducous bracteoles and 5–7 mm long pedicels. The corolla is pubescent and the sepals narrowly ovate, 7–8 × 3 mm, subacute and pubescent. The leaf base is very different from that of *Ipomoea padillae* and other species with trifurcate leaves, such as *I. delphinioides*.

### 3. Ipomoea pampeana

Taxon classificationPlantaeSolanalesConvolvulaceae

P.P.A. Ferreira & Miotto, Kew Bull. 66(2): 289. 2011. (Ferreira and Miotto 2011: 289)

#### Type.

BRAZIL. Rio Grande do Sul, Manoel Viana, *P.P.A. Ferreira* 279 (holotype ICN, isotypes K, P, SP).

#### Description.

Perennial twiner to 3 m, stems woody, grey-tomentose. Leaves petiolate, divided palmately to the base into five segments, 4–10 × 0.7–3 cm, narrowly elliptic to oblanceolate, acute or obtuse and mucronate, the basal lobes sometimes only lobed, noticeably larger, both surfaces grey-tomentose; petiole 2–5 cm long, grey-tomentose. Inflorescence of compound axillary cymes; peduncles 2–13 cm, tomentose; bracteoles 3–6 mm, lanceolate, caducous; secondary peduncles 1–2.5 cm; pedicels 7–10 mm, tomentose; sepals slightly unequal, outer 10–12 mm, ovate, acute, grey-tomentose, inner 11–13 mm, the margins glabrous; corolla 5–7 cm long, funnel-shaped, sericeous, pink with purple throat, limb 5–6 cm diam. Capsules 11–12 × 10 mm, subglobose, glabrous; seeds black, shortly tomentose, 7–8 mm long.

#### Illustration.

Ferreira and Miotto (2011: 291).

#### Distribution.

Grassy pampa. Endemic to the area around Manoel Viana in Rio Grande do Sul, Brazil.

**BRAZIL. Rio Grande do Sul** (Ferreira and Miotto 2011).

#### Note.

This species is probably close to *Ipomoea padillae* and the species represented by *Heinonen et al.* 117 discussed after *I. padillae*.

### 4. Ipomoea prolifera

Taxon classificationPlantaeSolanalesConvolvulaceae

J.R.I. Wood & Scotland, Kew Bull. 73 (57): 1. 2018. (Wood et al. 2018: 1)

#### Type.

BOLIVIA. Vallegrande, on descent to Pampa Negra, *J.R.I. Wood*, *M. Martinez & G. Aramayo* 28441 (holotype USZ, isotypes LPB, OXF).

#### Description.

Perennial herb, clambering over shrubs or, less commonly, decumbent; stems up to c. 3 m long, pubescent with long appressed hairs. Leaves petiolate, dimorphic; upper leaves and bracts 2.5–8 × 2–10 cm, diminishing in size upwards, entire, broadly ovate-elliptic to suborbicular, rounded, base shallowly cordate to truncate, margins undulate; lower leaves 7–13 × 7–14 cm, 3–5-lobed to about halfway (rarely unequally bilobed), the lobes oblong, obtuse to acute, base shallowly cordate; both leaf forms adaxially dark green, pubescent, abaxially grey-tomentose; petioles 2.5–7.2 cm, pubescent. Inflorescence of pedunculate axillary cymes usually with 7–8 flowers, mainly near the branch tips, somewhat proliferating; peduncles (0.5) –3–4.5 cm, pubescent, often somewhat bent or twisted, diminishing in length towards apex; bracteoles caducous, not seen; secondary peduncles 0.5–2 cm; pedicels 13–20 mm, pubescent, often bent; sepals subequal, 8–9 × 5–6 mm, oblong-elliptic, densely pubescent, outer rounded with narrow scarious margins, inner with rounded or retuse with broader scarious margins; corolla 5.5–6 cm long, funnel-shaped, pale pink, pubescent, limb c. 4 cm diam.; ovary glabrous. Capsules and seeds not seen.

#### Illustration.

Figure 12.

**Figure 12.**
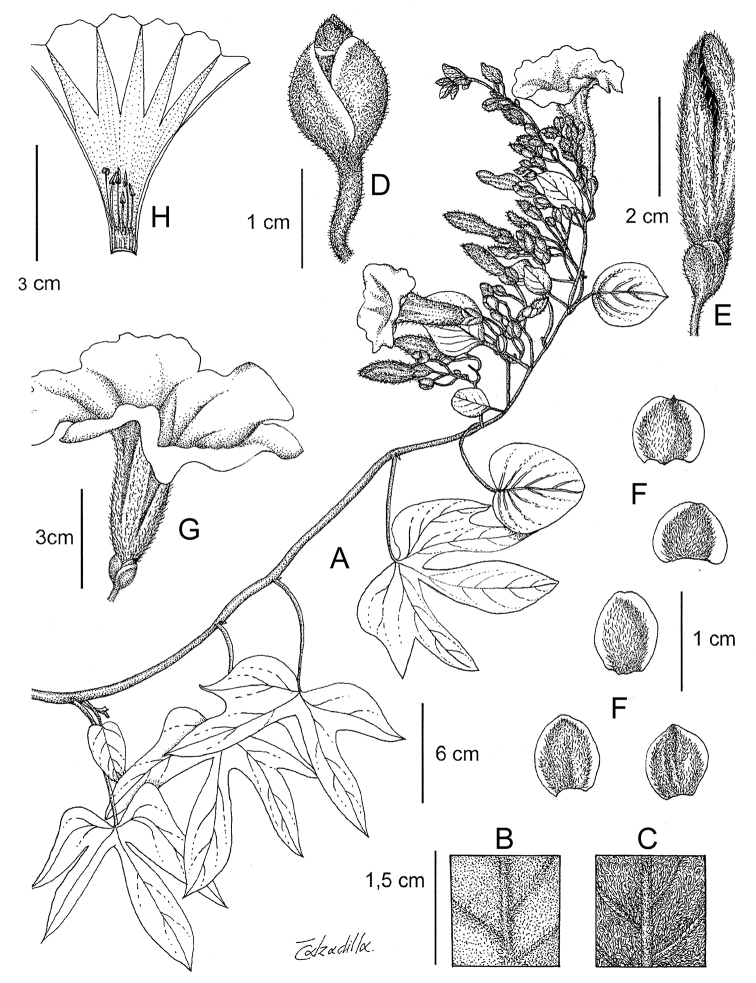
*Ipomoea prolifera***A** habit **B** adaxial surface of leaf **C** abaxial leaf surface **D** calyx **E** bud **F** sepals **G** corolla **H** corolla opened out to show stamens and style. Drawn by Eliana Calzadilla from *Wood et al.* 28441.

#### Distribution.

A narrow endemic restricted to seasonally very arid spiny bushland on descent to Pampa Negra in Vallegrande Province in Bolivia between 1650 and 1800 m.

**BOLIVIA. Santa Cruz**: Vallegrande, *J.R.I. Wood et al.* 28443 (LPB, OXF, USZ).

#### Note.

A scrambling or decumbent species with dimorphic leaves and stems which distinctly proliferate.

### 5. Ipomoea cardenasiana

Taxon classificationPlantaeSolanalesConvolvulaceae

O’Donell, Dusenia 1: 375. 1950. (O’Donell 1950c: 375)

#### Type.

BRAZIL. Mato Grosso do Sul, Urucúm, *M. Cárdenas* 4448 (holotype LIL001235).

#### Description.

Vigorous twining perennial to 3 m; stems stout, glabrous. Leaves petiolate, 4–10 × 3–8 cm, mostly 3-lobed to half way with acute lobes but some leaves ovate with one or two marginal teeth, base broadly cordate, apex shortly acuminate and mucronate, adaxially glabrous apart from veins pubescent near base, abaxially paler, pubescent especially on the veins; petioles 2–5 cm. Inflorescence of pedunculate, axillary cymes; peduncles 2–5 cm, stout, glabrous; bracteoles c. 5 mm long, oblong, muconate, papery, caducous; secondary and tertiary peduncles 0.8–1.5 cm; pedicels 5–10 mm, pubescent; sepals slightly unequal, outer 15–20 × 10–12 mm, ovate, narrowed to an obtuse apex, minutely puberulent, pale green; inner sepals 18–22 × 12 mm, elliptic, acuminate to an obtuse apex, sericeous, palid; corolla 7–9 cm long, funnel-shaped, pale pink, pubescent in bud, limb 5 cm diam., shallowly lobed. Capsules ovoid, 15 × 10 mm, glabrous, brown, enclosed by sepals; seeds 11 × 6 mm (possibly immature), brown, pilose with very long marginal hairs.

#### Illustration.

Wood et al. (2015: 53, photo).

#### Distribution.

A narrow endemic restricted to the Bolivia-Brazil border around Corumbá and Puerto Suárez at the edge of the Pantanal where it is locally common on scrubby roadsides around 100–150 m.

**BRAZIL. Mato Grosso do Sul**: Corumbá, *Dorrien Smith* 80 (K); Estrada da Codrasa, Ladãrio, *Bartolotto et al.* 8 (MBM).

**BOLIVIA. Santa Cruz**: Germán Busch, Puerto Suárez area, *J.R.I. Wood & D. Villarroel 25902* (K, LPB, UB, USZ); *J.R.I. Wood et al.* 27885 (K, LPB, USZ).

#### Note.

A very distinctive species because of its large corolla, acutely 3-lobed leaves and large pale green sepals.

### 6. Ipomoea aemilii

Taxon classificationPlantaeSolanalesConvolvulaceae

(O’Donell) J.R.I. Wood & R. Degen, Kew Bull. 71, 25: 3. 2016. (Wood et al. 2016b: 3)

Ipomoea malpighipila var. aemilii O’Donell, *Arq. Mus. Paranaense* 9: 228. 1952. (O’Donell 1952: 228). Type. PARAGUAY. Alto Paraná, 1909/10, *K. Fiebrig* 5684 (holotype SI001300, isotypes G? n.v., GH, LIL, SI, US).
Ipomoea aurita Hassl., nom. nud., Add. Plantae Hasslerianae 18. 1917. (Hassler 1917: 18).

#### Type.

Based on Ipomoea malpighipila var. aemilii O’Donell

#### Description.

Perennial of a pale green colour from a woody xylopodium; stems erect to 1 m high, apparently unbranched, densely hirsute with somewhat rough mostly appressed hairs. Leaves sessile, 16–27 × 0.4–0.8 cm, narrowly oblong, slightly narrowed to a cuneate base, apex obtuse and mucronate, coarsely tomentose on both surfaces, abaxially prominently 3–5-veined. Inflorescence terminal, rather short and dense < 7 cm long, formed of (1–)3-flowered cymes in the axils of leaf bracts; bracts 2–6.5 cm long, diminishing in size upwards, apparently deciduous and absent from uppermost cymes; peduncles 2–4 mm, relatively stout, densely hirsute; bracteoles c. 3 × 0.5 mm, lanceolate, acuminate, almost hidden by the indumentum; pedicels 5–7 mm, densely hirsute; sepals 7–8 × 4–5 mm, broadly elliptic, densely hirsute, slightly unequal, outer obtuse, inner rounded to retuse with glabrous, scarious margins; corolla 4–5 cm long, pink, funnel-shaped, densely pubescent on mid-petaline bands, limb 2.5–3 cm diam. Capsules glabrous; seeds not seen.

#### Illustration.

Figure 13.

**Figure 13.**
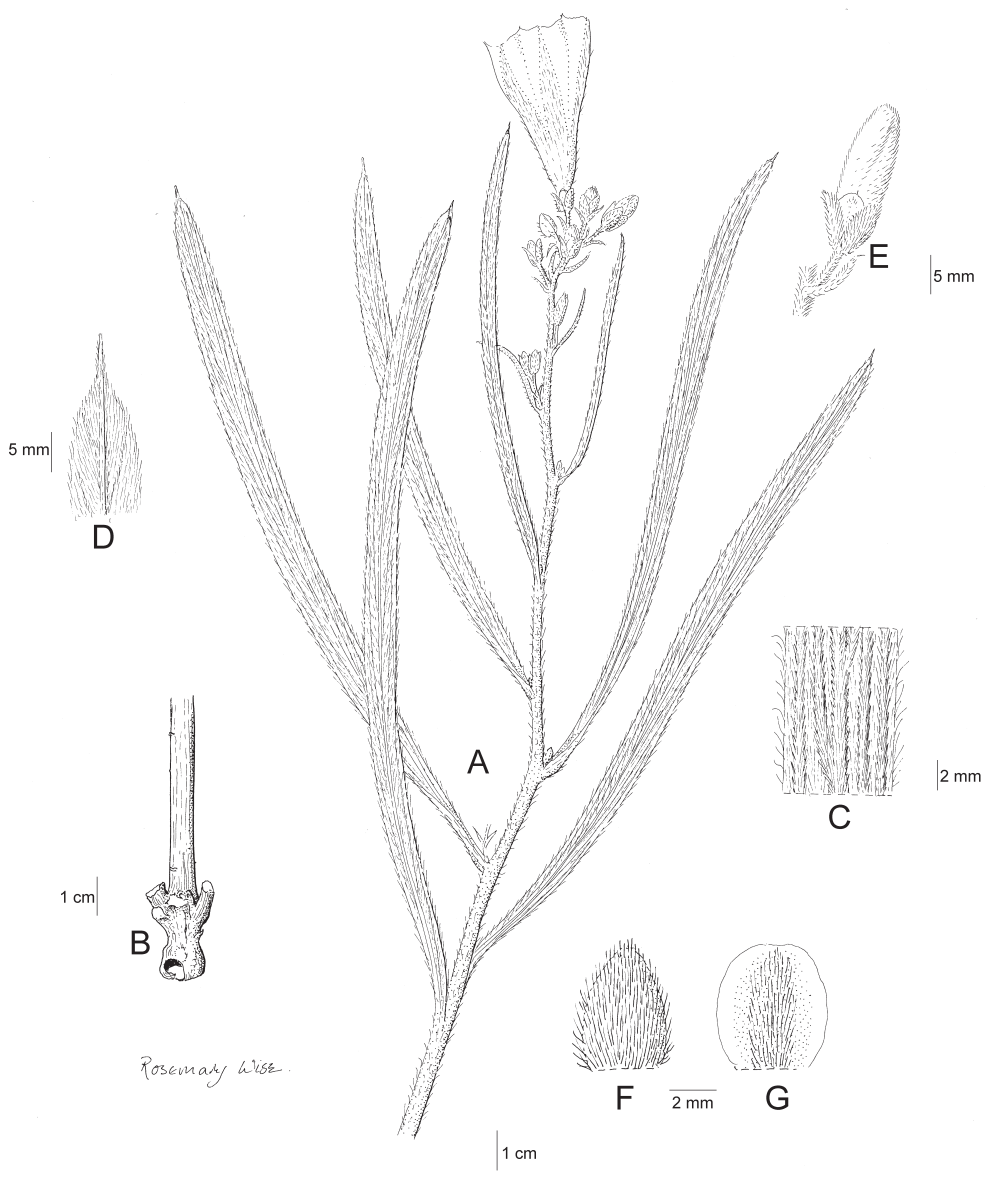
*Ipomoea aemilii*. **A** habit **B** stem base **C** leaf surface **D** leaf apex **E** flower bud **F** outer sepal **G** inner sepal. Drawn by Rosemary Wise **A** from *Itapú Binacional* 1046; **B–G** from *Fiebrig* 5684.

#### Distribution.

Endemic to Paraguay. In sabanas in the area north of Hernandarias, especially in the Reserva Tatí Yupí.

**PARAGUAY. Alto Paraná**: Reserva Tatí Yupí, *Itaipú Binacional* 1046 (MO); *G. Caballero Mamori* 1423 (CTES); Com. Puerto Palma, *C. Romero Pereira* 14 (SCP); Pirá Pytá, *A. Schinini et al*. 18152 (CTES).

#### Note.

Distinguished from *Ipomoea malpighipila* by the simple leaves and distinct indumentum.

### 7. Ipomoea malpighipila

Taxon classificationPlantaeSolanalesConvolvulaceae

O’Donell, Lilloa 23: 448. 1950. (O’Donell 1950a: 448)

#### Type.

ARGENTINA. Misiones, Dept. San Ignacio, Gob. Roca, 22 Nov. 1947, *G.J. Schwarz* 2338 (holotype LIL001259).

#### Description.

Erect perennial herb or subshrub from a xylopodium, stems 0.5–1 m long, usually simple, distinctly angled, adpressed pubescent with t-shaped hairs. Leaves shortly petiolate, 3-fid from near base, lobes 7.5–15 × 0.2–1.2 cm, narrowly oblong, shortly mucronate, base attenuate, both surfaces adpressed-pubescent, abaxially prominently veined; petioles 1–1.5 cm. Inflorescence elongate (to 10 cm), terminal, formed of shortly pedunculate cymes from the axils of leaf-like bracts, these absent in the upper part of inflorescence; peduncles 0.4–1.5 cm, adpressed pubescent; bracteoles ovate, caducous; pedicels 3–8 cm, adpressed pubescent; sepals equal, 6–8 × 4–6 mm, elliptic to suborbicular, obtuse and often mucronate, subsericeous; corolla 3.5–5 cm long, pink, funnel-shaped, adpressed pubescent. Capsules 7–10 × 7–8 mm, subglobose, glabrous; seeds 6 × 4 mm, blackish-brown, margins lanate.

#### Illustration.

O’Donell (1959b: 177).

#### Distribution.

Almost endemic to the province of Misiones in Argentina where it grows in seasonally flooded grassy pampa. There appear to be no recent records from Paraguay or Brazil.

**ARGENTINA. Misiones**: San Ignacio, *D. Giambaggio* s.n. (SI); *G.J. Schwarz* 5334 (E, LIL, S); *M.E. Rodríguez & A. Gochez* 1179 (MA); *H. Keller et al.* 6464 (CTES); *J.E. Montes* 458 (LIL, S).

**PARAGUAY. Itapúa**: Encarnación, *T. Rojas* 29 (SCP).

**BRAZIL. Rio Grande do Sul**: *Agusto* s.n. (ICN18804), fide Ferreira and Miotto (2009: 446).

#### Note.

The T-shaped hairs are difficult to observe but are distinctive. *Ipomoea malpighipila* is usually easily identified by the terminal inflorescence and obscurely pubescent, trifid leaves with narrowly oblong lobes.

### 8. Ipomoea cordillerae

Taxon classificationPlantaeSolanalesConvolvulaceae

J.R.I. Wood & Scotland, Kew Bull. 72 (9): 9. 2017. (Wood and Scotland 2017a: 11)

Ipomoea malveoides Meisn. var. ovata Hallier f., Bull. Herb. Boiss. 7(5): append. 1: 152. 1899. (Hallier 1899b: 52). Type. PARAGUAY. [Cordillera], Cordillera de Peribebuey, 6 April 1883, *B. Balansa* 4391 (lectotype G00174792, designated by Wood and Scotland (2017a: 11), isolectotypes G, P).

#### Type.

Based on Ipomoea malveoides Meisn. var. ovata Hallier f.

#### Description.

Erect subshrub to at least 50 cm; stems woody below, ± glabrescent; above herbaceous, softly white-tomentose. Leaves very shortly petiolate, 2.4–7 × 3.2–5 cm, ovate, oblong or oblong-elliptic, acute and mucronate, base broadly cuneate, margin entire, both surfaces softly pubescent, abaxially more densely so, paler, adaxially somewhat glabrescent on very old leaves; petioles 0–4 mm, densely pubescent to villous. Inflorescence usually of solitary, pedunculate axillary flowers forming a long terminal raceme; occasionally of axillary cymes with up to five flowers from the uppermost leaf axils; bracts leaf-like except the uppermost of which are much reduced; peduncles 0.8–4 cm, densely white-pubescent; bracteoles 6 mm long, linear filiform; pedicels 0.6–7 cm, densely pubescent; sepals with a dark gland near base, somewhat unequal, outer 9–15 × 2–4 mm, narrowly to broadly ovate, acuminate or acute and mucronate, tomentose, inner similar bur with broad scarious margins; corolla 6–6.5 cm long, funnel-shaped, pink, pubescent, limb c. 5 cm diam. Capsules c. 1.2 × 0.8 cm, ovoid, glabrous; seeds 7 × 4 mm, blackish, glabrous.

#### Illustration.

Figure 14.

**Figure 14.**
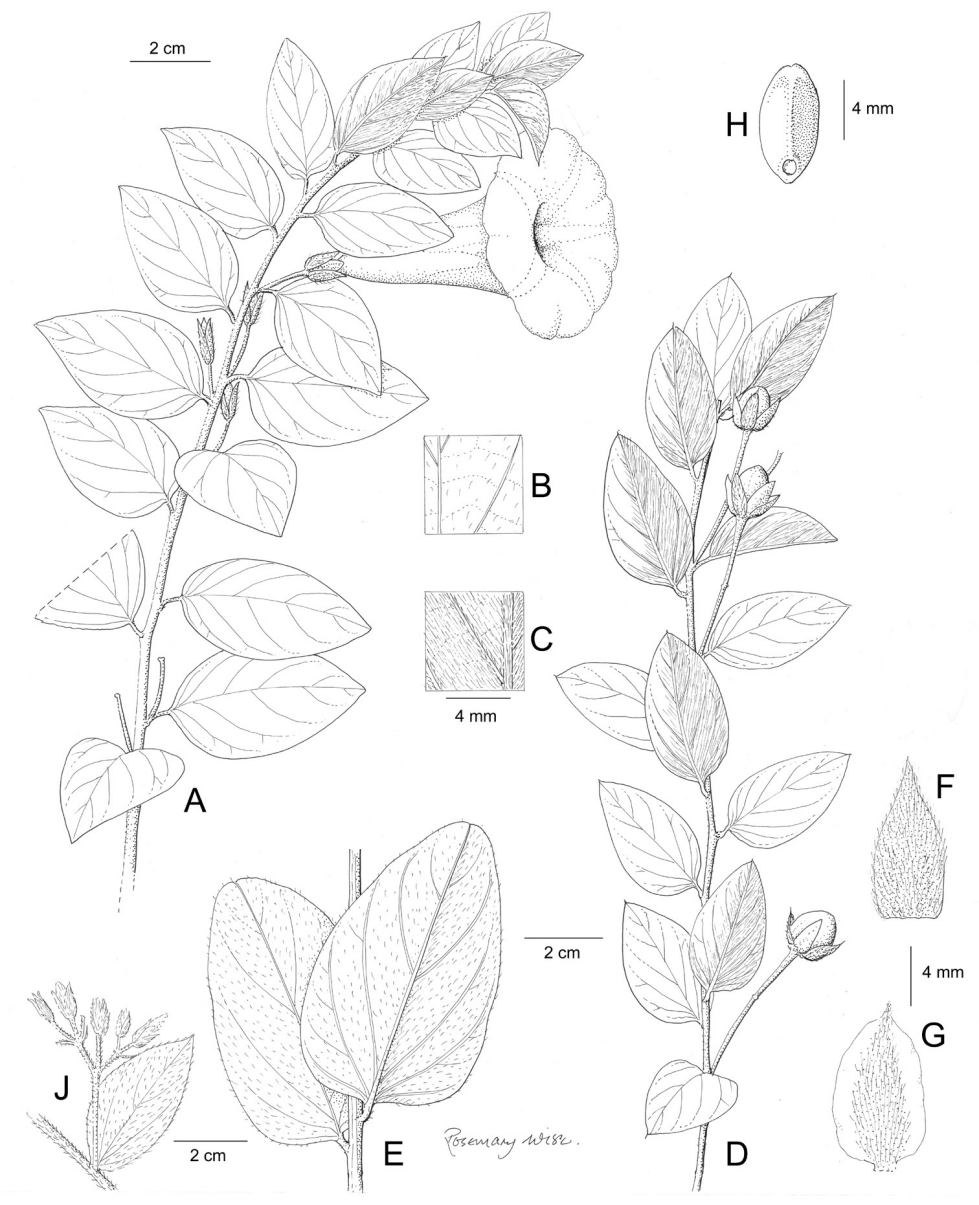
*Ipomoea cordillerae*. **A** habit (flowering plant) **B** adaxial leaf surface **C** abaxial leaf surface **D** habit (fruiting plant) **E** portion of stem and leaves **F** outer sepal **G** inner sepal **H** seed **J** form with branched inflorescence. Drawn by Rosemary Wise **A–C** from *Hassler* 8714 (GH); **D–H** from *Balansa* 4391: **J** from *Hassler* 485. Drawn by Rosemary Wise.

#### Distribution.

Endemic to Paraguay and growing in forest clearings (fide *Balansa* 4391). **PARAGUAY. Cordillera**: *E. Hassler* 285 (K, P), 1903 (K, P), 8714 p.p. (BM, K).

#### Note.

Characterised by the relatively long acuminate or acute and mucronate sepals usually around 12 mm in length combined with the softly tomentose indumentum and ovate-elliptic leaves. In the type the leaves are silvery beneath but this is less obvious in the other cited collections. *Ipomoea paraguariensis* differs in the much shorter silvery sepals and more strictly terminal inflorescence and *I. estrellensis* differs in the shorter, obtuse to subacute sepals, the shorter peduncles and the ciliolate leaf margins. We have seen no modern collections of this species.

Specimens of *Hassler* 8714 are mixed, those at BM and K are this species but some specimens with this number are *Ipomoea paraguariensis*. They are all labelled as from Villarrica where *Ipomoea paraguariensis* grows but the specimens of *I. cordillerae* presumably came from the Pirebebuy area.

• Speces 9–18 form a complex in which *Ipomoea malvaeoides* is the best-known and most common species.

### 9. Ipomoea paludosa

Taxon classificationPlantaeSolanalesConvolvulaceae

O’Donell, Lilloa 23: 495. 1950. (O’Donell 1950b: 495)

Ipomoea malvaeoides var. integrifolia Chodat & Hassl., Bull. Herb. Boiss. Ser. 2, 5: 690. 1905. (Chodat and Hassler 1905: 690). Type. PARAGUAY. Canindeyú, Río Jezuí Guazú, *E. Hassler* 5734 (lectotype G00175132, designated here; isolectotypes BM, G, GH, K, MPU, P).
Ipomoea malvaeoides forma apiculata Chodat & Hassl. [as var. uliginosa forma apiculata ], Bull. Herb. Boiss., ser. 2, 5: 691. 1905. (Chodat and Hassler 1905: 691). Type. PARAGUAY. Cordillera, Tobatí, *E. Hassler* 6274 (?G, n.v.).
Ipomoea malvaeoides var. uliginosa Chodat & Hassl., Bull. Herb. Boiss. Ser. 2, 5: 691. 1905. (Chodat and Hassler 1905: 691). Type. PARAGUAY. Cordillera, Tobatí, *E. Hassler* 6405 (lectotype G, n.v., designated by O’Donell (1953a: 373), isolectotype BM000089442).
Ipomoea paludosa var. uliginosa (Chodat & Hassl.) O’Donell, Lilloa 26: 373. 1953. (O’Donell 1953a: 373). Type. Based on Ipomoea malvaeoides var. uliginosa Chodat & Hassl.

#### Type.

ARGENTINA. Misiones, Dept. San Ignacio, Gob. Roca, *G. J. Schwarz* 5283 (lectotype LIL001271, designated here; isolectotypes CTES, LIL).

#### Description.

Erect undershrub 0.5–1.5 m from a woody rhizome, stems glabrous or with a few scattered hairs, sparingly branched, often simple. Leaves shortly petiolate, 2.5–11 × 0.6–2.2 cm, oblanceolate, acute or rounded and strongly apiculate, cuneate at base, adaxially glabrous to thinly adpressed pilose, abaxially adpressed pilose, veins prominent on both surfaces, esp. abaxially; petioles 0.5–1 cm long, thinly pubescent. Inflorescence long, terminal, raceme-like, formed of mostly2–3-flowered cymes, commonly reduced to single flowers; bracts leaf-like but diminishing in size upwards; peduncles 0.2–3 cm long; bracteoles 3–4 mm, lanceolate, caducous; pedicels 2–10 mm, pubescent; sepals 5–8 mm, ovate, acute to obtuse and apiculate, sericeous to pubescent, inner sepals similar but obtuse and with glabrous, scarious margins; corolla 3.5–5.5 cm long, pink, funnel-shaped, sericeous on midpetaline bands, limb 2–2.5 cm diam., undulate. Capsules c. 8 × 6 mm, ovoid, glabrous; seeds long-pilose.

#### Illustration.

**Figure 15.**
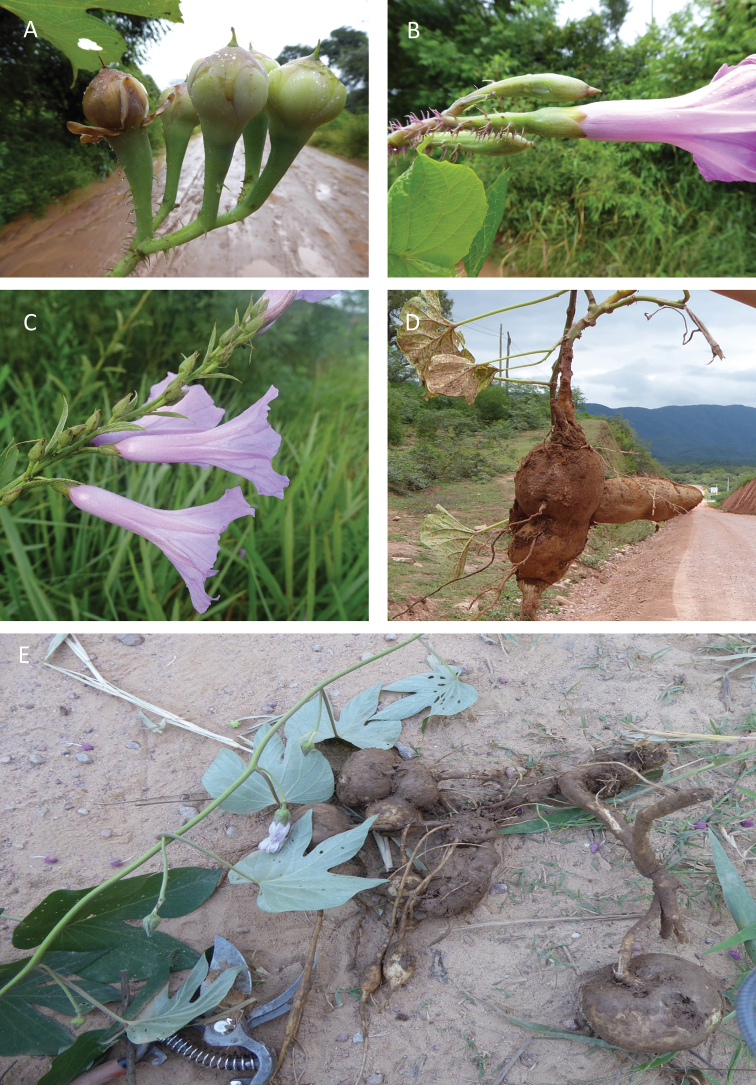
Photographs of *Ipomoea* species. **A–B**I. setosa subsp. pavonii**A** fruit **B** flower **C***I. paludosa*, subspicate inflorescence **D***I. lilloana*, storage root **E***I. opulifolia*, storage root, leaves and flower. **A**, **B**, **D**, **E** Maira Martinez; **C** Hector Keller.

O’Donell (1959b: 213); Figure 15C.

#### Distribution.

Flooded plain in the Paraná basin in Argentina, Brazil and Paraguay. **ARGENTINA. Misiones**: San Ignacio, *F.O. Zuloaga & M. Kostlin* 9948 (SI); Candelaria, *H. Keller & Paredes* 10563 (CTES); Bonpland, *E.L. Ekman* 1432 (K, S); Capital, *T.M. Pedersen* 13661 (C, CTES).

**PARAGUAY. Alto Paraná**: Est. Río Bonito, *E. Zardini & Vieira* 41978 (FTG, PY). **Amambay**: Est. Carmen de la Sierra, *N. Soria* 4725 (CTES, FCQ). **Caaguazú**: Coronel Oviedo, *A. Krapovickas et al.* 13848 (CTES). **Caazapá**: Enramadita, *I. Basualdo* 001902 (FCQ, MO, FTG). **Canindeyú**: Reserva Mbaracuyú, *B. Jiménez & G. Marín* 1962 (BM, MA). **Central**: *A. Schinini* 5717 (CTES). **Concepción**: Est. Ybyraty, *F. Mereles* 8580 (CTES, FCQ). **Cordillera**: Peribebuy, *B. Balansa* 4392 (P); Tobatí, *R.O. Vanni et al.* 185 (CTES, PY). **Guairá**: Cordillera de Ybyturuzú, *F. Mereles* 3724 (FCQ). **Itapúa**: Yacyreta Island Reserve, *E. Zardini & Gamarra* 55715 (ARIZ); Trinidad, *M. Ortiz* 850 (FCQ). **Paraguarí**: 3 km antes de Caballero, *Calviño et al.* 3774 (FCQ). **San Pedro**: Est. San Antonio, *N. Soria* 5363 (CTES, FCQ).

**BRAZIL. Mato Grosso do Sul**: Faz. Campo Alto, Corumbá, *A. Pott et al.* 5576 (CPAP, CTES); *Hatschbach et al.* 76514 (MBM).

#### Note.

Plants from Argentina are relatively uniform but in Paraguay they are more variable, the leaves sometimes strongly apiculate and/or the inflorescence rather lax and few-flowered.

### 10. Ipomoea morongii

Taxon classificationPlantaeSolanalesConvolvulaceae

Britton in Morong, T. & Britton, N.L., Ann. New York Acad. Sc. 7: 171. 1892. (Morong and Britton 1892: 171)

Ipomoea malvaeoides var. trifida Hallier f., Bull. Herb. Boiss. 7 (5), append. 1: 52. 1899. (Hallier 1899b: 52). Type. PARAGUAY. Cordillera de los Altos, *E. Hassler* 1938 (lectotype G00174972, designated here).
Ipomoea malvaeoides var. heterophylla Hallier f., Bull. Herb. Boiss. 7 (5), append. 1: 52. 1899. (Hallier 1899b: 52). Type. PARAGUAY. [Cordillera], San Bernardino, *E. Hassler* 1796 (lectotype G00174971, designated here).
Ipomoea malvaeoides forma intermedia Chodat & Hassl. [as var. heterophylla forma intermedia ], Bull. Herb. Boiss., ser. 2, 5: 690. 1905. (Chodat and Hassler 1905b: 690). Type. PARAGUAY. Cordillera de Los Altos, *E. Hassler* 3456 (lectotype G00174963, designated here; isolectotypes G).

#### Type.

PARAGUAY. [Central], Luque, *T. Morong* 303 (holotype NY00319204, isotypes GH, MO, PH, US, WIS).

#### Description.

Erect undershrub to 1.2 m, stems below woody, glabrous, reddish above herbaceous, densely puberulent. Leaves petiolate, lower leaves 9–10 × 2–4 cm, entire, ovate obtuse to acute and mucronate, base cuneate, upper leaves (2–)3-lobed with the laterals much shorter than the central lobe which is usually lanceolate, acuminate, the uppermost leaves noticeably smaller and with narrower lobes, both surfaces finely tomentellous, abaxially paler; petioles1–2.5 cm, puberulent. Inflorescence of shortly pedunculate cymes from the upper leaf axils; peduncles 2–4 (–9) cm, puberulent; bracteoles 3–4 × 1 mm, oblong-lanceolate, caducous; secondary peduncles 0.7–1.8 cm; pedicels 6–10 mm, puberulent; sepals subequal, tomentellous, outer 7–9 × 5–6 mm, ovate, acute to obtuse, inner similar but with scarious, less hirsute margins; corolla 4.5–6.5 cm long, pink, pubescent, funnel-shaped; limb 3–5 cm diam., entire. Capsules and seeds not seen.

#### Illustration.

Figures 16, 17B.

**Figure 16.**
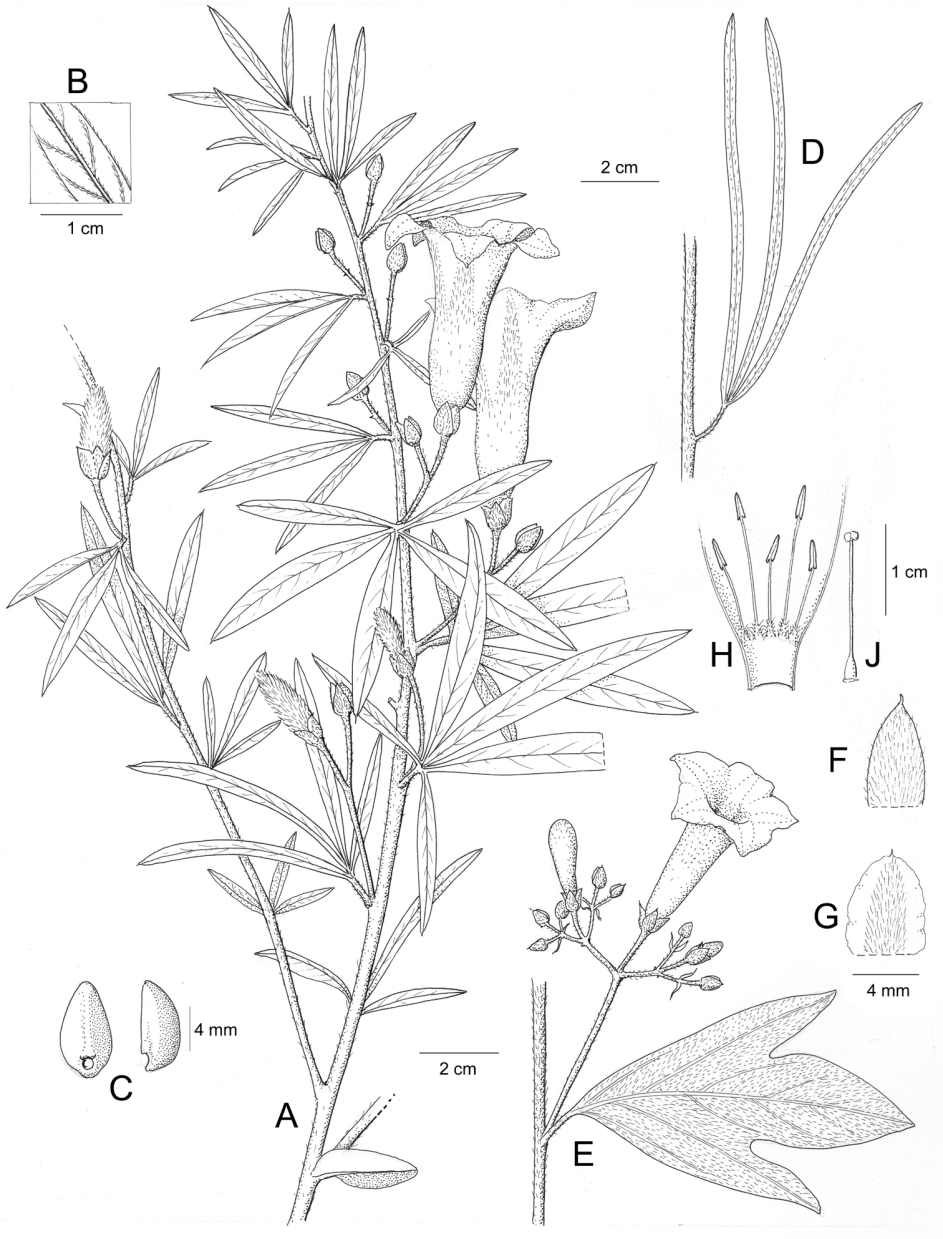
**A–D***Ipomoea malvaeoides*. **A** habit **B** abaxial leaf surface **C** seed **D** leaves (var. lineariloba). **E–J***Ipomoea morongii*. **E** habit **F** outer sepal **G** inner sepal **H** corolla opened out to show stamens **J** ovary and style. Drawn by Rosemary Wise **A, B** from *Krapovickas et al.* 412477; **C** from *Schinini* 30429; **D** from *St. Hilaire* 2703, **E–J** from *Hassler* 3307.

**Figure 17.**
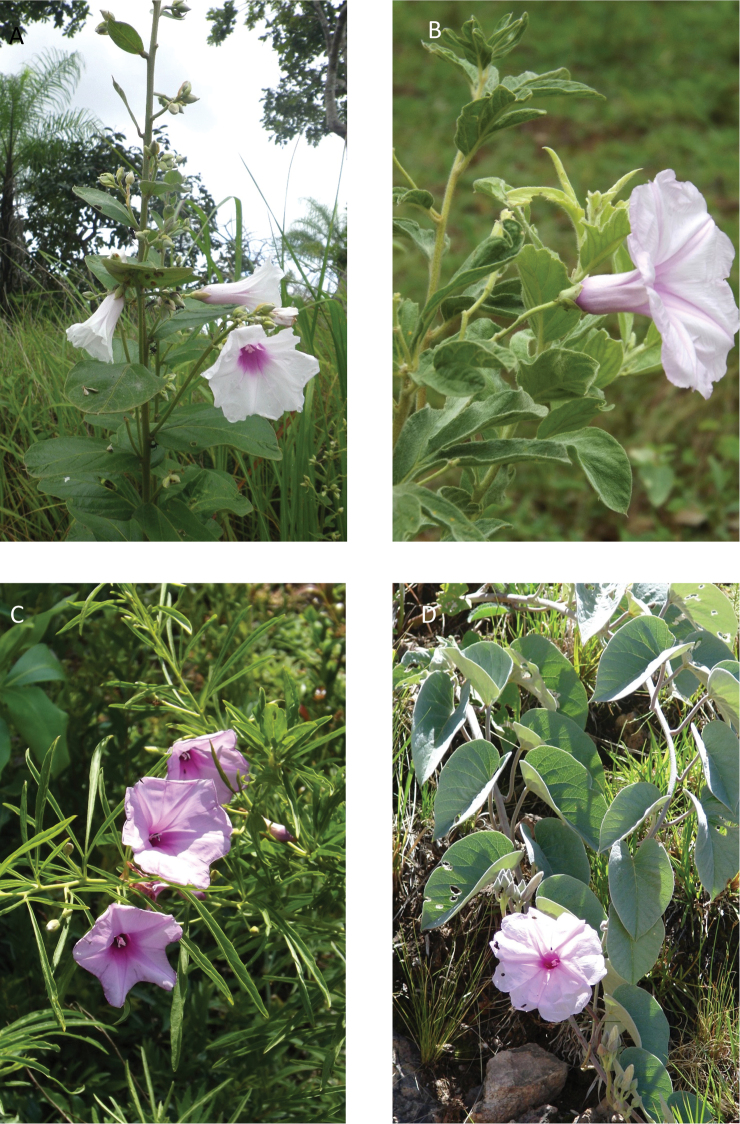
Photographs of *Ipomoea* species. **A***I. haenkeana***B***I. morongii***C***I. malvaeoides***D***I. hieronymi*. **A** John Wood; **B, C** Tom Carruthers; **D** Keith Ferguson.

#### Distribution.

Endemic to the area around Lago Ypacaraí in Central and Cordillera departments in eastern Paraguay.

**PARAGUAY. Central**: Ypacaraí, *E. Hassler in Rojas* 11473 (BM, K, NY). **Cordillera**: Emboscada, *I. Basualdo* 1021 (CTES, FCQ); Emboscada hacia Nueva Colombia, *R. Degen* 1385 (CTES, FCQ); Nueva Colombia, *J.R.I. Wood et al.* 28147 (FCQ); costa del Lago Ypacaraí, *C. Quarin et al.* 1488 (CTES); San Bernardino, *E. Hassler* 3307 (P), *T. Rojas* 1694 (LIL, SI), *T. Rojas* 14136 (SCP).

#### Typification.

*Ipomoea morongii* is heterophyllous on the same plant with some leaves entire and some trifurcate. In lectotypifying the synonyms of *Ipomoea morongii*, we have endeavoured to choose specimens which show heterophylly and at least some trifurcate leaves. G00174972 is the only specimen at G annotated *trifida* by Hallier, although he also, confusingly, annotated it as *I. heterophylla*. The portion in the envelope which is clearly trifid should be treated as the lectotype in the event of any dispute. The specimen G00174971 of *Hassler* 1796 is designated as the lectotype of Ipomoea malvaeoides var. heterophylla because it has some trifurcate leaves even though it was not annotated by Hallier.

#### Note.

Although this species is clearly closely related to *I. malvaeoides* and could possibly be treated as a variety of it, it is usually easily distinguished by the trifurcate tomentose stem leaves with broad segments conspicuously fused in their lower half. The type is less hairy than most specimens.

### 11. Ipomoea malvaeoides

Taxon classificationPlantaeSolanalesConvolvulaceae

Meisn. in Martius et al., Fl. Brasil. 7: 251. 1869. (Meisner 1869: 251)

Ipomoea malvaeoides var. digitata Hallier f., Bull. Herb. Boiss. 7 (5), append. 1: 53. 1899. (Hallier 1899b: 53), nom. illeg., autonymic var.
Ipomoea malvaeoides var. lineariloba Hallier f., Bull. Herb. Boiss. 7 (5), append. 1: 53. 1899 (Hallier 1899b: 53). Type. PARAGUAY. *B. Balansa* 1073 (lectotype G00175984, designated here; isolectotypes BR, G, GOET, K, P).
Ipomoea malvaeoides var. albiflora Hallier f., Bull. Herb. Boiss. 7 (5), append. 1: 53. 1899. (Hallier 1899b: 53). Type. PARAGUAY. *B. Balansa* 4395 (lectotype P03536099, designated here; isolectotypes G).
Ipomoea malvaeoides var. argentea O’Donell, Lilloa 29: 179. 1959. (O’Donell 1959b: 179). Type. ARGENTINA. Corrientes, Dept. Mburucuyá, Est. Santa Teresa, *G.J. Schwarz* 8811 (holotype LIL, n.v.).
Ipomoea pinifolioides Arachav., An. Mus. Nac. Montevideo 7: 197. 1911. (Arechavaleta y Balpardo 1911: 197). Type. URUGUAY. Artigas, “Campos de San Eugenio, diciembre 1901”, *J. Arechaveleta* 455 (holotype MVM).

#### Type.

BRAZIL. [Rio Grande do Sul, between Rio Santa Bárbara and Alegrete], *F. Sello(w)* 3386 (Photo F ex B, holotype†), epitype Brazil, Rio Grande do Sul, *A. St Hilaire* 2714 (P00746402), designated here).

#### Description.

Erect (rarely decumbent) undershrub to 50 cm; stems puberulent, rootstock tuberous. Leaves shortly petiolate, numerous, mostly 3–5(–7)-fid to near base (some lower leaves entire and up to 2.5 cm wide), lobes 4–9 × 0.15–1.5 cm, oblong to narrowly oblong-oblanceolate, obtuse and mucronate, tapering at base, abaxially (greyish-)sericeous to pubescent only on the veins; petioles 0.2–1.5 cm. Inflorescence of few-flowered pedunculate cymes, from the upper leaf axils,these often reduced to solitary flowers in many populations; peduncles 0.7–2.5(–4)cm, glabrous or puberulent, rarely glabrous; bracteoles 1–2 mm, lanceolate, caducous; pedicels 5–15 mm, puberulent or glabrous; sepals somewhat unequal, outer (5–)7–8(–10) × 3–6 mm, ovate, obtuse to subacute, thinly to very densely pubescent, inner sepals elliptic, rounded, very slightly shorter, pubescent but with scarious glabrous margins; corolla 4.5–6 cm long, pink, funnel-shaped, thinly pilose, limb 3–4 cm diam. Capsules 1.3 × 0.7 cm, ovoid, glabrous; seeds 7 × 5 mm, blackish, glabrous.

#### Illustration.

Figures 16, 17C; O’Donell (1959b: 181, 183).

#### Distribution.

Cerrado and cerrado-like pampas in NE Argentina, southern Brazil, eastern Paraguay and Uruguay, probably declining in frequency throughout its range.

**URUGUAY.***F. Felippone* s.n. (SI).

**ARGENTINA. Corrientes**: Ituzaingó, Santa Rita, *A. Krapovickas et al.* 41247 (CTES, K); Mburucuyá, Est. Santa Teresa, *T.M. Pedersen* 198 (C, P, S); Manantiales, *T.S. Ibarrola* 3678 (LIL, S); Capital, Riachuelo, *A. Schinini* 30429 (CTES, MA). **Misiones**: Posadas, *M.E. Rodríguez* 1177 (CTES); *A. Barbero* (SCP); *E.L. Ekman* 1424 (S).

**PARAGUAY. Caaguazú**: Arroyo Yakare’i, *E. Zardini & Aguayo* 10744 (FCQ). **Canendiyú**: *B. Jiménez et al.* 1873 (CTES); Mbaracayú Natural Reserve, *E. Zardini & Benítez* 51288 (ARIZ). **Central**: Campo Grande de San Lorenzo, *T. Rojas* 10351 (SCP); Limpio, Ribera de Río Salado, *F. Mereles* 3886 (FCQ); road to Luque, *L. Pérez et al.* 32 (PY). **Concepción**: 3.2 km NW of Loreto, *M. Dematteis et al.* 3137 (CTES, FCQ); Est. Villa Sana, *R. Degen* 2280 (CTES, FCQ). **Cordillera**: *E. Hassler* 6116 (BM, G); Piribebuy, *N. Soria* 3212 (FCQ); Tobatí, *E. Zardini & Velázquez* 26714 (FCQ); Caacupé, *Bordas* 4078 (CTES). **Itapúa**: Isla Yaciretá, *M. Pena-Chacarro et al.* 1789 (BM, FCQ). **Misiones**: 12 km W of San Ignacio, *M.M. Arbo et al.* 1917 (CTES, MO). **Paraguarí**: Colonia Achotei, Est. Lago Ypoá, *F. Mereles et al*. 8050 (CTES, FCQ); Ybicuí, *Bernardi* 18086 (BM, G). **San Pedro**: Est. Chaparral, *S. Keel & L. Spinzi* 1793 (FCQ).

**BRAZIL. Rio Grande do Sul**: São Francisco de Assis, *L.P. Queiroz & M.C. Machado* 12612 (HUEFS); ibid., *P.P.A. Ferreira* 488 (NY); Santana de Livramento, *E. Barbosa et al.* 2542 (MBM, RB).

#### Lectotypification.

None of the syntypes of Ipomoea malvaeoides var. lineariloba are annotated with this name by Hallier but we have selected the Geneva specimen, G00175984, of *Balansa* 1073 as it is only sheet we have seen with any annotation by Hallier. However, in the case of Ipomoea malvaeoides var. albiflora, we have designated the Paris specimen as none of the syntypes are annotated by Hallier and the Paris specimen of *Balansa* 4395 is much the best available.

#### Note.

*Ipomoea malvaeoides* is a notoriously variable species, especially in Paraguay, and a number of varieties have been recognised. Variation is most marked in the length and width of the leaflets, their indumentum and in the degree of branching of the inflorescence. The type and most specimens from Argentina have solitary axillary flowers whereas most specimens from Paraguay have a branched cymose inflorescence. Plants from Corrientes in Argentina were recognised as **var. argentea** by O’Donell and can be recognised by the relatively broad leaflets which are silvery-pubescent on the abaxial surface. These plants occur rarely in Paraguay. Very narrow-leaved forms are found in Uruguay, Rio Grande do Sul and in Paraguay and can be recognised as **var. lineariloba** Hallier f. There is some variation in sepal size; *Pena-Chacarro et al.* 1789, for example, has longer sepals than usual but forms from eastern Paraguay with consistently longer sepals are treated below as *Ipomoea pseudomalvaeoides*.

### 12. Ipomoea pseudomalvaeoides

Taxon classificationPlantaeSolanalesConvolvulaceae

Chodat & Hassl., Bull. Herb. Boiss., ser. 2, 5: 691. 1905. (Chodat and Hassler 1905: 691)

Ipomoea pseudomalvaeoides forma sericea Chodat & Hassl., Bull. Herb. Boiss., ser. 2, 5: 691. 1905. (Chodat and Hassler 1905: 691). Type. PARAGUAY. [Canindeyú], Apepú, *E. Hassler* 4345 (lectotype G00175054, designated here; isolectotypes BM, G, K, NY, P).
Ipomoea pseudomalvaeoides forma palmata Chodat & Hassl., Bull. Herb. Boiss., ser. 2, 5: 691. 1905. (Chodat and Hassler 1905: 691). Type. PARAGUAY. San Pedro, Río Corrientes, *E. Hassler* 5840 (lectotype G00175060, designated here; isolectotypes G).
Ipomoea pseudomalvaeoides forma trispathulata Chodat & Hassl., Bull. Herb. Boiss., ser. 2, 5: 691. 1905. (Chodat and Hassler 1905: 691). Type. PARAGUAY. Canindeyú, Río Carimbatay, *E. Hassler* 4540 (lectotype G00175052, designated here; isolectotypes BM, G).

#### Type.

PARAGUAY. San Pedro, Río Corrientes, *E. Hassler* 5857 (lectotype G00175058, designated here; isolectotypes, F, G, K, NY, P, UC).

#### Description.

Erect herb to 0.75 cm from a xylopodium; stems adpressed pilose. Leaves subsessile, mostly trifurcate but occasionally simple above, base cuneate, segments (and simple leaves) 4–10 × 0.5–1.7 cm, oblong-oblanceolate, acute, mucronate, adaxially with scattered long, appressed hairs, abaxially the veins and margins pilose with white appressed hairs, the intercostal areas glabrous; petioles 0–6 mm, thinly pilose. Inflorescence of solitary pedunculate flowers from the upper leaf axils; peduncles 0–35 mm, diminishing in length upwards, adpressed pilose; bracteoles early caducous, not seen; pedicels 4–5 mm, very constant in length, adpressed pilose; sepals slightly unequal, outer 9–15 × 3–4.5 mm, ovate, acute, adpressed pilose, inner similar but with broad, glabrous, scarious margins; corolla 7–9 cm long, pink, pubescent, funnel-shaped, limb 5–6 cm diam., undulate. Capsules and seeds not seen.

#### Distribution.

Scrubby cerrado. Probably endemic to Canindeyú and neighbouring parts of San Pedro departments in Paraguay.

**PARAGUAY. Canindeyú**: Mbaracayú Natural Reserve, *E. Zardini & I. Chaparro* 50723 (ARIZ, AS, MO), 60302 (MO), 60327 (MO); *E. Zardini & S. Ramírez* 51089 (ARIZ, AS, MO), 51288 (ARIZ, AS, MO); *A. Schinini & M. Dematteis* 33313 (CTES, FCQ, MO); Reserva de Campo Comunal del asientamiento Mandu’ara, *O.A. Torres Figueredo* 43 (FCQ); 25 km W of Curuguaty, *J.R.I. Wood & G. González* 28465 (FCQ). **San Pedro**: south of Arroyo Gasory, *S. Keel & L. Spinzi* 1738 (FCQ).

#### Notes.

The exact location of Apepú is uncertain. The name refers to a citrus fruit and appears as a place name for a number of different locations.

*Ipomoea pseudomalvaeoides* is very close to *I. malvaeoides* and may prove to be only a variety of it but it has distinctive longer sepals and is restricted geographically to Canindeyú and the surrounding area.

### 13. Ipomoea theodori

Taxon classificationPlantaeSolanalesConvolvulaceae

O’Donell, Lilloa 14: 191. 1948. (O’Donell 1948a: 191)

#### Type.

PARAGUAY. Caaguazú, Estancia Primera, April 1927, *T. Rojas* 5036 (holotype LIL001288).

#### Description.

Perennial herb, stems erect or decumbent, glabrous, to 50 cm long. Leaves subsessile, (1–)3 partite almost to base, segments linear, acute, 3–7 × 0.1–0.2 cm, glabrous. Inflorescence of solitary, long-pedunculate, axillary flowers; peduncles 6–10 cm, glabrous; bracteoles 1.5–1.7 cm, linear, caducous; pedicels 10–16 mm, relatively stout; sepals subequal, outer 20–23 × 6–8 mm, broadly lanceolate, acute, glabrous, inner slightly narrower; corolla 5–6 cm long, funnel-shaped, deep pink, glabrous, the limb 4 cm diam., unlobed; ovary glabrous. Capsules and seeds unknown.

#### Distribution.

Endemic to Paraguay. Known only from the type.

**PARAGUAY. Caaguazú**: the type collection.

#### Note.

Outstanding for the large sepals and glabrous vegetative parts. It is only distinguishable from the following, unnamed species by the very long sepals.

### 14. Ipomoea sp. A aff. theodori

Taxon classificationPlantaeSolanalesConvolvulaceae

#### Remarks.

Erect perennial undershrub from a xylopodium; stems several, below woody, glabrescent, above, herbaceous, thinlysoftly pilose. Leaves shortly petiolate, mostly 3-lobed almost to base but a few lower leaves entire, base cuneate, segments 4–13 × 0.2–0.6, linear-oblong, obtuse to acute, shortly mucronate, both surfaces thinly pilose to subglabrous; petioles 2–10 mm. Inflorescence of solitary pedunculate axillary flowers; peduncles 2.5–10 cm; bracteoles caducous, not seen; pedicels 10–15 mm, slightly thickened upwards; sepals subequal to unequal, outer 8–15 × 5–6, ovate, obtuse, mucronate, thinly pubescent to subglabrous, inner larger, 11–16 mm, ovate to elliptic, mucronate, more densely pubescent but with broad, glabrous margins; corolla 6–8 cm long, pink, pubescent, funnel-shaped, limb 4–6.5 cm diam., undulate. Capsules and seeds not seen.

#### Distribution.

Endemic to Caaguazú in Paraguay and recorded as growing in cerrado.

**PARAGUAY. Caaguazú**: Río Yhú, *E. Hassler* 9689 (BM, K), 9689a (BM, K), 9689b (NY); Vic. Caaguazú, *E. Hassler* 9229 (BM, K, NY); Colonia Pindo, camino entre Itakyry y Curuguaty, *A. Schinini & G. Caballero Mamori* 30164 (CTES, K).

#### Note.

This plant comes from the same region as *Ipomoea theodori* and may eventually prove to be only a form of it. It differs in the somewhat broader leaflets and the distinctly shorter calyx, although the calyx is still longer than other species in this group.

### 15. Ipomoea itapuaensis

Taxon classificationPlantaeSolanalesConvolvulaceae

J.R.I. Wood & R. Degen, Kew Bull. 71, 25: 2. 2016. (Wood et al. 2016b: 2)

#### Type.

PARAGUAY. March 1931, *P. Jorgensen* 4662 (holotype US, isotypes F, GH, S).

#### Description.

Perennial herb from a woody base; stems 30–40 cm long, probably erect, woody below, subglabrous but with a few adpressed trichomes arranged bifariously. Leaves subsessile, lamina subdigitately divided into (3–)5(–7) linear segments 2–7 × 0.1–02 cm, apex acute (apiculate), both surfaces glabrous (or abaxially pubescent on midvein and margins); petioles 2–4 mm long, glabrous (thinly pubescent). Inflorescence of solitary, axillary flowers; peduncles 2.5–3.8 cm; bracteoles caducous, not seen; pedicels 3–7 mm; sepals slightly unequal, outer 6–7 × 3.5–5 mm, ovate-elliptic, slightly convex, apex obtuse, mucronulate, the margins glabrous, inner elliptic-suborbicular, mucronate, rounded, 7–8 × 3.5–6 mm. pubescent in central area, margin glabrous, scarious; corolla 4.5–6 cm long, pink or white, funnel-shaped, densely pubescent in bud; limb 3.5–4 cm diam., unlobed; ovary glabrous. Capsules and seeds not seen.

#### Illustration.

Figure 18.

**Figure 18.**
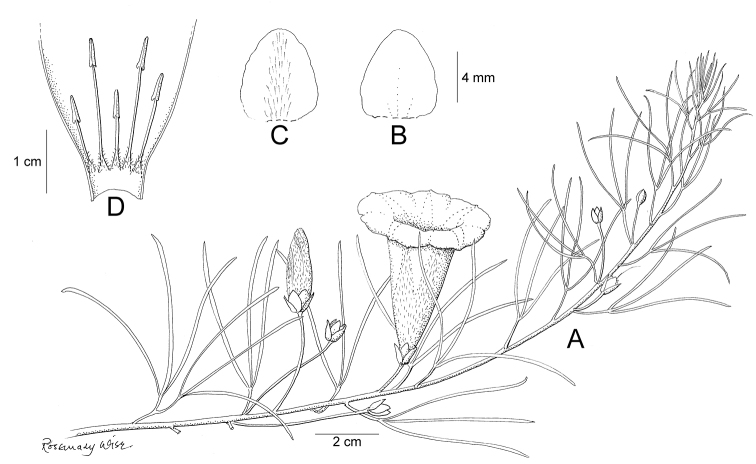
*Ipomoea itapuaensis*. **A** habit **B** outer sepal **C** inner sepal **D** corolla opened out to show stamens. Drawn by Rosemary Wise from *Jorgensen* 4662.

#### Distribution.

Endemic to Paraguay, where it grows in cerrado grassland on the border area between Itapúa and Caazapá.

**PARAGUAY. Caazapá**: Reserva Tapytá, *B. Jiménez* 208 (FCQ); ibid., *M. Vera* 167 (FCQ). **Itapúa**: Alta Verá, *A. Parra et al.* 116 (FCQ), ibid., *Parra et al.* 117 (FCQ); P.N. San Rafael, *G. Caballero Marmori* 3906 (MBM).

#### Note.

This species resembles *Ipomoea theodori* and *I. fiebrigii* in the subsessile, digitately divided leaves with linear segments. From the former it is distinguished by the pubescent (not glabrous) corolla and much shorter sepals, which in *I. theodori* are 20–24 mm long. From *I. fiebrigii* it is readily distinguished by the glabrous (not pilose), stem, leaves, peduncles and sepals and by the much longer peduncles, which scarcely reach 5 mm in *I. fiebrigii*.

### 16. Ipomoea fiebrigii

Taxon classificationPlantaeSolanalesConvolvulaceae

Hassl. ex O’Donell, Lilloa 14: 169. 1948. (O’Donell 1948a: 169)

#### Type.

PARAGUAY. Río Alto Paraná, Ñucañy, Feb. 1908, *K. Fiebrig* 5675 (holotype LIL001244, isotypes SI, US).

#### Description.

Perennial from a xylopodium, stems erect, 30–60 cm high, pilose. Leaves with very short internodes, subsessile, divided into 5–7 segments, segments 2–4.5 × 0.1–0.3 cm, linear, acute, pilose, strongly inrolled; petioles 0–1 mm. Inflorescence of solitary, shortly pedunculate, axillary flowers, peduncles 2–3 mm; bracteoles 4 mm, lanceolate, caducous; pedicels 3–7 mm; sepals slightly unequal, outer 9–11 × 4–4.5 mm, oblong-lanceolate, acute, pubescent with white hairs, inner sepals c. 5 mm wide, oblong-ovate, obtuse, densely white-piloset, the margins slightly scarious; corolla 4.5–5.5 cm long, funnel-shaped, pink, tomentose with long white hairs outside, limb 4.5 cm diam., unlobed. Capsules ovoid, c. 10 mm wide, glabrous; seeds glabrous.

#### Illustration.

Figure 19.

**Figure 19.**
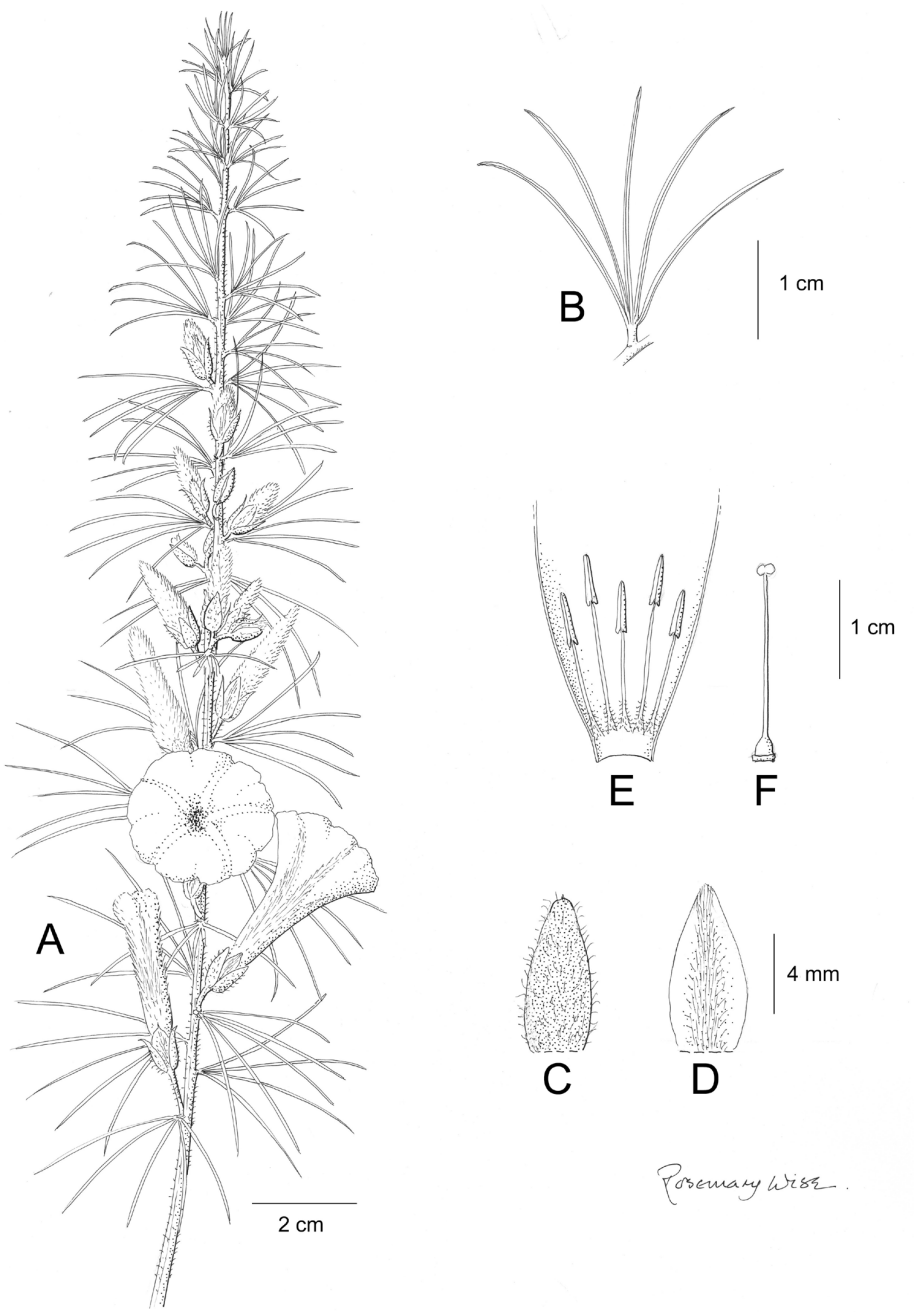
*Ipomoea fiebrigii*. **A** habit **B** leaf **C** outer sepal **D** inner sepal **E** corolla opened out **F** ovary and style. Drawn by Rosemary Wise from *Itaipú Binacional* 1081.

#### Distribution.

A rare species endemic to eastern Paraguay.

**PARAGUAY. Alto Paraná**: Itakyry, *K. Fiebrig* 6706 (LIL); Reserva Tatí Yupí, *Itaipú Binacional* 1081 (MO).

#### Note.

This species is distinguished by its linear, almost filiform leaf segments, shortly pedunculate axillary flowers and the white-pilose indumentum of the corolla and inner sepals.

### 17. Ipomoea angustissima

Taxon classificationPlantaeSolanalesConvolvulaceae

J.R.I. Wood & Scotland, Kew Bull. 72 (9): 13. 2017. (2017a: 13)

#### Type.

BRAZIL. Goiás, 16 km N of Alto Paraíso *Gates & Estabrook* 106 (holotype RB223038, isotype FTG).

#### Description.

Perennial herb to 40 cm from a tuberous rootstock, apparently unbranched or branched near the base only; stems erect, asperous-pubescent. Leaves sessile or very shortly petiolate, 1–7 segments radiating out from the base, segments 0.8–5 × c.0.1 cm linear, acute, 1-veined, thinly pilose to ±glabrous; petioles 0–2 mm, thinly pilose. Inflorescence terminal consisting of single flowers or compact few-flowered cymes from the uppermost leaf axils; peduncles 1–9 mm, pubescent; bracteoles 3 × 1.5–2 mm, oblong, rounded to retuse, thinly pubescent, margin scarious, caducous; pedicels 3–7 mm, pubescent; sepals subequal, 5–8 × 5–6 mm, elliptic, obtuse to rounded, pubescent except for the scarious margins, outer sometimes mucronulate, reddish, margins narrow, inner more rounded with broader scarious margins; corolla 3.5–4 cm long, funnel-shaped, pink, pubescent, limb c. 2.5 cm diam., somewhat lobed. Capsules and seeds not seen.

#### Illustration.

Figure 20.

**Figure 20.**
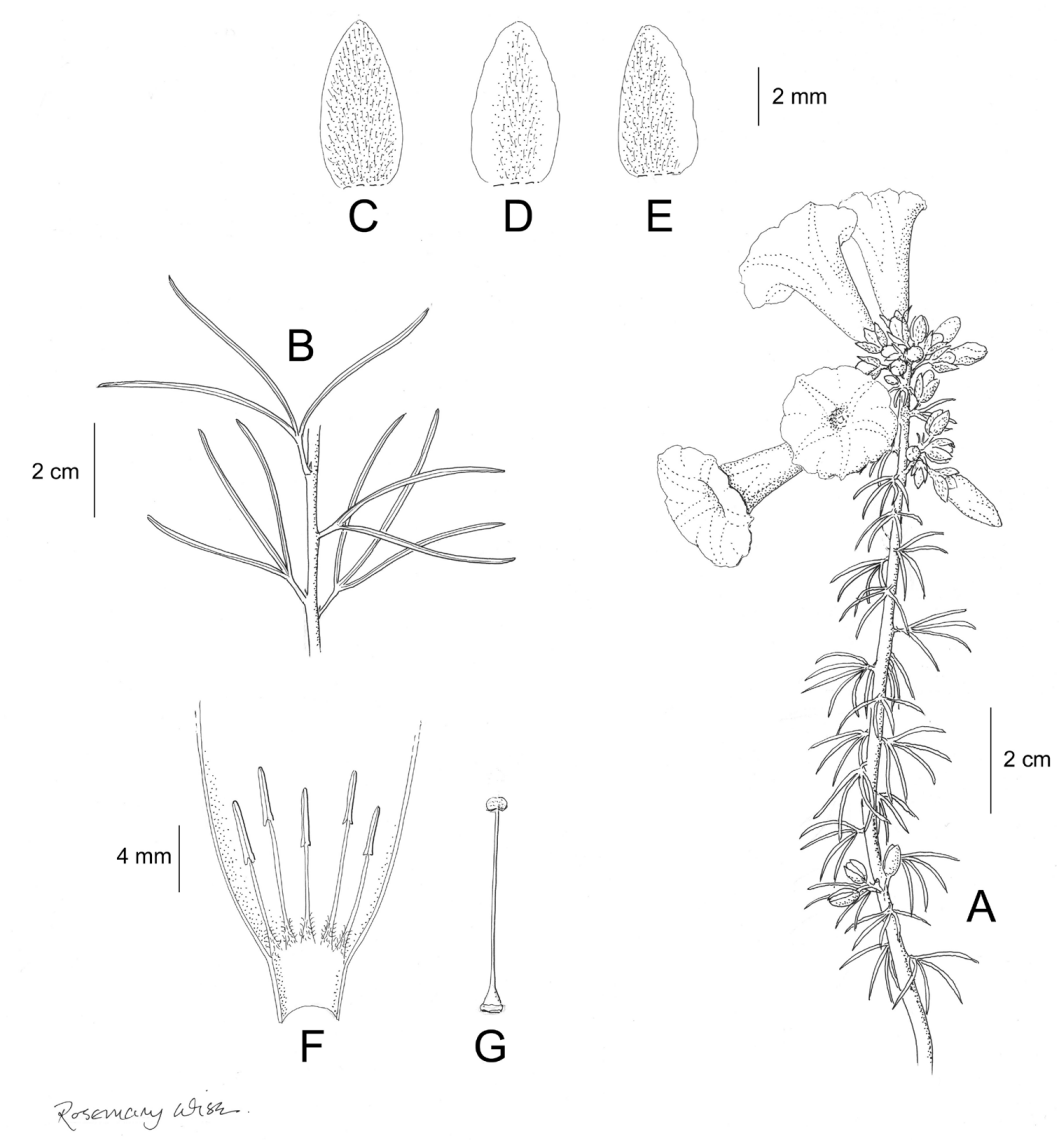
*Ipomoea angustissima*. **A** habit **B** stem showing leaves **C** outer sepal **D** middle sepal **E** inner sepal **F** corolla opened out **G** ovary and style. Drawn by Rosemary Wise **A** from *Harley et al.* 11361; **B** from *Gates & Esterbrook* 106; **C–G** from *Irwin et al.* 12542.

#### Distribution.

Campo húmedo at relatively high altitudes in the Chapada dos Veadeiros. **BRAZIL. Goiás**: Alto de Paraíso area, *H.S. Irwin et al.* 32976 (MO, NY); ibid., *Gates & Estabrook* 106 (FAU, RB223038); ibid., *J.R. Pirani et al.* 1765 (K, SPF); c. 65 km due N of Brasilia, *R.M. Harley et al.* 11361 (K); ibid., *M.J. Graziela & A. Lima* 829-68 (IPA, OXF); Mun. Cavalcante, frente entrada a Faz. Vicente, *J.F.B. Pastore et al.* 816 (CEN).

#### Note.

This species is often identified as *Ipomoea fiebrigii* in error but is immediately distinguished by the terminal inflorescence, shorter, rounded or obtuse sepals and the absence of long white hairs on the inner sepals and corolla. It is also sometimes identified as *Ipomoea stenophylla* (= *I. campestris*) but differs in the terminal inflorescence and elliptic, rounded, not acute sepals. The upper part of stem and peduncles appear to be sticky as granules of sand stick to the hairs, the stem appearing superficially to be granulose.

*J.R. Pirani et al.* 1765 differs from other specimens in having some entire, lanceolate or ovate, ±obtuse leaves 1.5–3.5 × 0.3–0.8 cm. *R. Romero et al.* 4796 (SP) from P.N. Serra de Canastra, São Roque de Minas, Minas Gerais might also belong to this species but the inflorescence is axillary and is only known to us from an image.

### 18. Ipomoea revoluta

Taxon classificationPlantaeSolanalesConvolvulaceae

J.R.I. Wood & Scotland, Phytokeys 88: 25. 2017. (Wood et al. 2017d: 25)

#### Type.

BRAZIL. Mato Grosso do Sul, Serra de Maracaju, 17 Feb. 1970, *G. Hatschbach* 23761 (holotype MBM, isotypes CTES, F, MICH, S).

#### Description.

Slender twining liana of unknown height; stem woody, c. 2–3 mm thick, pale brown, shortly pubescent. Leaves petiolate, digitately divided into 5–7 free leaflets; leaflets 5–9 × 0.15–0.4 cm, linear, attenuate to a mucronate apex, basally tapered, margin inrolled; adaxally glabrous, midvein strongly impressed; abaxially white-tomentose, the midvein prominent, nearly glabrous; petioles 8–13 mm, thinly pubescent;. Inflorescence of 1–3-flowered axillary cymes; peduncles 7–9 mm, very thinly pubescent with scattered hairs; bracteoles c. 1 mm long, scale-like, caducous; pedicels 8–10 mm long, very thinly pubescent with scattered hairs; sepals subequal, 8–10 × 6–7 mm, ovate to elliptic, acute to shortly mucronate, sericeous with narrow, scarious, glabrous margins, inner sepals white-sericeous with wider scarious margins; corolla 5–6 cm long, pink, sericeous in bud, funnel-shaped from a short basal cylindrical tube, limb c. 2 cm diam., lobes rounded. Capsules ovoid, apiculate, c. 10 mm long (immature), glabrous, ±enclosed by the sepals; seeds not known.

#### Illustration.

Figure 21.

**Figure 21.**
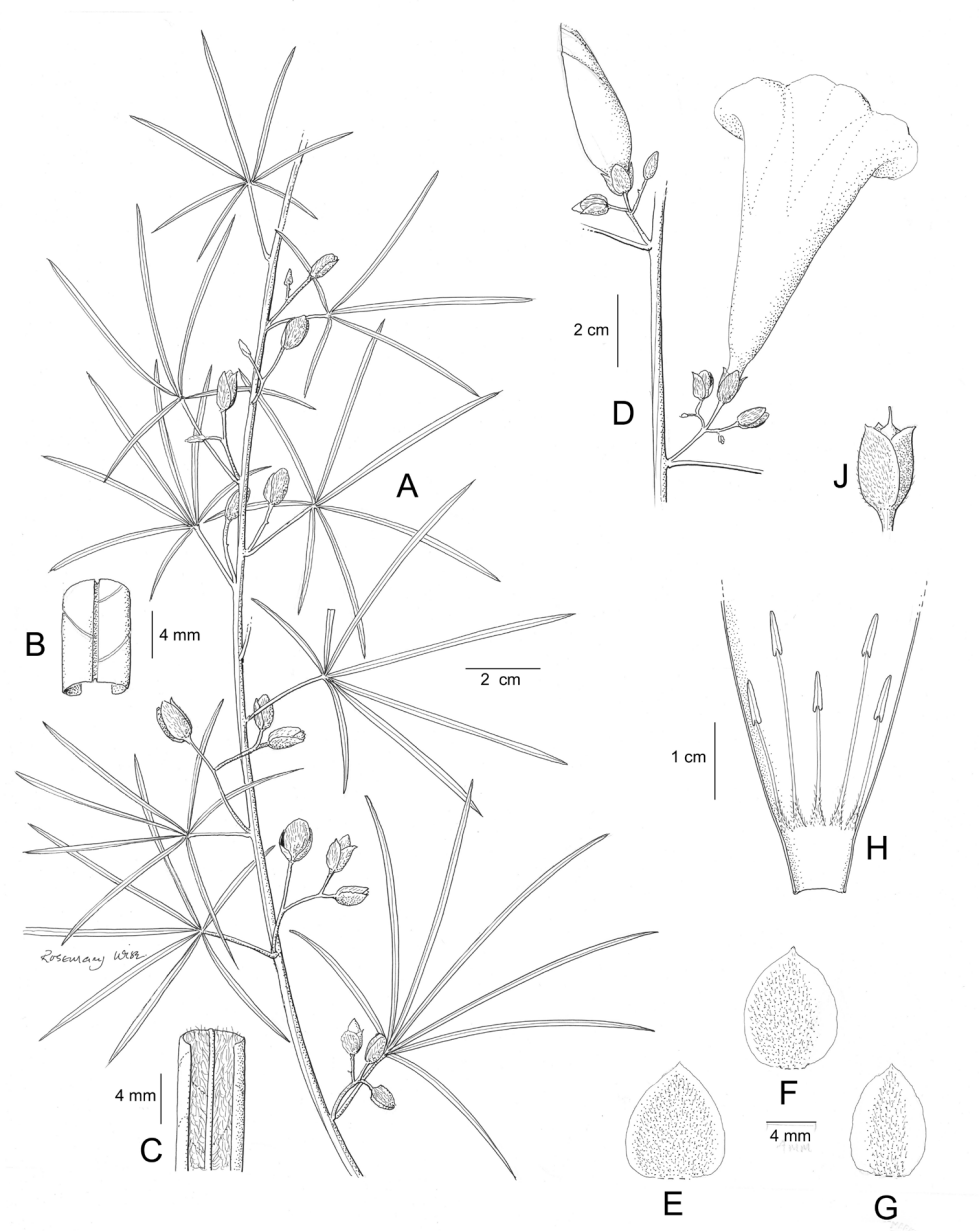
*Ipomoea revoluta*. **A** habit **B** adaxial leaf surface **C** abaxial leaf surface **D** inflorescence **E** Outer sepal **F** middle sepal **G** inner sepal **H** corolla opened out to show stamens **J** calyx enclosing capsule. Drawn by Rosemary Wise from *G. Hatschbach* 23761.

#### Distribution.

Apparently endemic to the Serra de Maracaju in Mato Grosso do Sul, where it grows on sandstone rock outcrops.

**BRAZIL. Mato Grosso do Sul**: *G. & M. Hatschbach & J.M. Silva* 60724 (MBM).

#### Note.

This species is almost certainly related to *Ipomoea malvaeoides* and its allies but is distinguished from all of these by its twining (not erect) habit and distinctly petiolate leaves. Related species in which the leaves have linear leaflets, such as *I. fiebrigii*, *I. itapuaensis* and *I. theodori*, have sessile or near sessile leaves. The linear leaflets recall those of the unrelated *Ipomoea subrevoluta*, which it has been wrongly named in many herbaria. It is easily distinguished from that species by the sericeous exterior of the corolla and the large, abaxially pubescent sepals.

### 19. Ipomoea valenzuelensis

Taxon classificationPlantaeSolanalesConvolvulaceae

Chodat & Hassl., Bull. Herb. Boiss. Ser. 2: 5: 687. 1905. (Chodat and Hassler 1905: 687)

Ipomoea valenzuelensis forma glabrescens Chodat & Hassl., Bull. Herb. Boiss. Ser. 2: 5: 687. 1905. (Chodat and Hassler 1905: 687). Type. PARAGUAY. Cordillera, Valenzuela, Jan. 1900, *E. Hassler* 7035 (? holotype G n.v., isotypes BM, K, NY, P).

#### Type.

PARAGUAY. Dept. Cordillera, Valenzuela, Jan. 1900, *Hassler* 7036 (? holotype G n.v., isotypes BM, F, GH, K, LIL, MO, MPU, NY, P, S, UC).

#### Description.

Trailing perennial with densely coarsely hirsute stems. Leaves shortly petiolate, 5–12 × 2–5 ovate, ovate-deltoid to oblong-elliptic (rarely shallowly 3-lobed), acute, base cuneate to rounded, densely hirsute on both surfaces, abaxially paler; petioles 5–13 mm, hirsute. Inflorescence of 1–3-flowered, pedunculate axillary cymes; peduncles 2.5–11 cm, hirsute; bracteoles filiform, 5–12 mm, caducous; pedicels 5–15 mm, hirsute; sepals 14–18(–20) × 4–8 mm, slightly unequal, ovate, caudate, densely hirsute, inner with subglabrous, slightly scarious margins; corolla 5.5–6.5 cm long, funnel-shaped, pink, densely pubescent, limb 4.5–5 cm diam., weakly lobed. Capsules 11 × 8 mm, ovoid, glabrous, shortly rostrate; seeds 6 × 3.5 mm, ovoid, blackish-brown, glabrous.

#### Distribution.

Endemic to Paraguay where it grows in cerrado-like vegetation. **PARAGUAY.** Sine data, *Jorgensen* 3475 (F, S). **Cordillera**: Valenzuela, *R.O. Vanni et al.* 1154 (MO, CTES, K). **Guairá**: Villarrica, *E. Hassler* 8577 (BM), 8827 (BM); Villa Rica-Independencia, *N. Soria* 4233 (FCQ, MA, MO); *F. Mereles & M. Soloaga* 7561 (CTES, FCQ); Cordillera del Ybytyrusú, *E. Zardini & A. Aguayo* 14896 (FTG, MO, FCQ).

#### Typification.

We have not seen specimens of the type nor of var. glabrescens at Geneva, so have not made any lectotypifications.

#### Note.

This species is characterised by its decumbent habit, coarsely hispid indumentum and long, caudate sepals. It is similar to *Ipomoea pseudocalystegia* but the leaves are simple, the sepals and bracteoles are shorter and the pedicels much longer. It also somewhat resembles *Ipomoea langsdorffii* but the flowers are often solitary and never in many-flowered cymes, and the leaves are not whitish beneath.

### 20. Ipomoea acutisepala

Taxon classificationPlantaeSolanalesConvolvulaceae

O’Donell, Lilloa 23: 478, 1950. (O’Donell 1950b: 478)

#### Type.

ARGENTINA. Misiones, *G.J. Schwarz* 5098 (lectotype LIL001225, designated here; isolectotypes LIL, P).

#### Description.

Decumbent (rarely climbing) perennial with stems to 4 m long; stems thinly hispid. Leaves shortly petiolate, 5–11 × 1–10, elliptic to obovate in outline, 3-lobed to about halfway, base broadly cuneate, apex obtuse to rounded, strongly mucronate, both surfaces thinly to densely hispid; petioles 0.5–2.5 cm, hispid pilose. Inflorescence of long-pedunculate, compact axillary cymes with up to c. 8 flowers; peduncles 3–12 cm, hispid; bracteoles 5–15 × 0.5–1 mm, linear or lanceolate, acuminate, hispid, margins scarious; secondary peduncles very short or absent, up to 1 cm long; pedicels 3–8 mm, hispid; sepals subequal, 10–16 × 3–4 mm, lanceolate to ovate, finely acuminate, densely hispid-pilose; corolla 5.5–6.5 cm long, funnel-shaped, pink, pilose; limb c. 4 cm diam.; stigma bilobed with globose lobes. Capsules and seeds not seen.

#### Illustration.

Figure 22; O’Donell (1959b: 102).

**Figure 22.** *Ipomoea acutisepala*. **A** habit **B** inflorescence **C** outer sepal **D** middle sepal **E** inner sepal **F** corolla opened out to show stamens **G** ovary and style. Drawn by Rosemary Wise from *Cavalcanti et al.* 3675.

#### Distribution.

Scattered over southern Brazil and neighbouring parts of Argentina and Paraguay.

**ARGENTINA. Misiones**: Dept. Candelaria, *Rodríguez* 1187 (CTES); Posadas, *E.L. Ekman* 1417 (LIL, S).

**PARAGUAY. Itapúa**: Trinidad, *A. Krapovickas et al.* 46153 (CTES, K).

**BRAZIL. Paraná**: *A. Krapovickas & C. Cristóbal* 39719 (CTES, FTG, K), 40802 (CTES, FTG); *P. Dusen* 2661 (S); Campo Largo, *G. Hatschbach* 3674 (US). Parque Iguaçu, *L. R. Landrum* 4045 (ARIZ, MO, NY); Jaguariaíva, *T.B. Cavalcanti et al.* 3675 (CEN); *A. Krapovickas & A. Schinini* 38237 (CTES); *J.C. Lindeman & J.F.M. Valls* 9502 (CTES, ICN); *B. Rambo 34977* (S), 51633 (S). **Santa Catarina**: *A. Krapovickas & C. Cristóbal* 42007 (CTES, FTG), 43574 (CTES); *L.B. Smith & R.M. Klein* 8116 (S); *L. B. Smith & R. Reitz* 8632 (US), 9048 (MO, US); Mafra, *R. Reitz* 5370 (US). **São Paulo**: *A. St. Hilaire* 1525 (P).

#### Notes.

Similar to *Ipomoea valenzulensis* but the leaves are trifurcate and the inflorescence is many-flowered. It differs from *Ipomoea langsdorffii* in the trifurcate leaves, which are not whitish beneath, and from *I. delphinioides* in the finely acuminate sepals. *Landrum* 4045 has some leaves entire, some trifurcate.

O’Donell’s concept of this species contained elements of *Ipomoea megalantha* as he identified *Hassler* 9114 as *I. acutisepala* in 1953. Consequently, in the protologue he provided larger sepal and floral dimensions than are correct. The type (*Schwarz* 5098) itself is mostly 1-flowered and is not characteristic of the species.

*P. Dusen* 7385 (F, GH, MICH, P, S) from Serrinha, Paraná State is similar to *Ipomoea acutisepala* except for the subacute sepals. It is thus intermediate with *I. delphinioides* and has been identified with both species on different occasions.

### 21. Ipomoea delphinioides

Taxon classificationPlantaeSolanalesConvolvulaceae

Choisy, Mém. Soc. Phys. Genève 8(1): 53 [131]. 1838. (Choisy 1838: 53 [131])

Ipomoea polymorpha var. delphinioides (Choisy) Meisn. in Martius et al., Fl. Brasil. 7: 252. 1869. (Meisner 1869: 252).
Convolvulus campestris Vell., Fl. Flumin.74. 1825 [pub. 1829]. (Vellozo 1829: 74), non Ipomoea campestris Meisn. (1869). Type. BRAZIL. [São Paulo], Cunha (lectotype, original parchment plate of Flora Fluminensis in the manuscript section of the Biblioteca Nacional, Rio de Janeiro [cat. no.: mss1198651-066], designated here; later published in Vellozo, Fl. Flum. Icon. 2: t. 66. 1827 [pub. 1831]).
Ipomoea trifurcata Choisy, Mém. Soc. Phys. Genève 8(1): 53 [131]. 1838. (Choisy 1838: 53 [131]). Type. BRAZIL. São Paulo, Mugi das Cruzas, N. Lund (isotypes G, possible isotype P03390576).
Ipomoea polymorpha var. heteromorpha Meisn. in Martius et al., Fl. Brasil. 7: 252. 1869. (Meisner 1869: 252). Type. Based on Ipomoea trifurcata Choisy
Ipomoea aspersa Mart. ex Choisy in A.P. de Candolle, Prodr. 9: 368. 1845. (Choisy 1845: 368). Type. BRAZIL. “ex Cam Raben n. 275” (lectotype BR00005307272, designated here).
Ipomoea polymorpha var. calvescens Meisn. in Martius et al., Fl. Brasil. 7: 252. 1869. (Meisner 1869: 252), nom. illeg., superfl. Type. BRAZIL. Based partly on Ipomoea aspersa and partly on *Martius* Obs. 571 (M).

#### Type.

BRAZIL. São Paulo, Taubaté, *N. Lund* 771 (holotype G00135575).

#### Description.

Perennial with prostrate to ascending, appressed pubescent stems, rootstock tuberous with subcylindrical tubers. Leaves shortly petiolate, 2.5–6.5 × 0.8–3.5 cm, oblong-elliptic, obtuse to rounded, mucronulate, base cuneate, usually 3-lobed to half way (occasionally entire, rarely 5-lobed more deeply), finely pubescent on both surfaces; petioles 0.1–1.2 cm. Inflorescence of pedunculate axillary cymes, often reduced to solitary flowers; peduncles 0.3–6 (–13) cm, pubescent; bracteoles filiform, 4 mm, caducous; pedicels 5–10 mm, pubescent; sepals 7–12 mm, subequal, narrowly ovate, subacute to obtuse, pubescent, the inner with broad, scarious, glabrous margins; corolla 5–6 cm long, white or pale pink, funnel-shaped, sericeous, limb c. 3–4 cm diam. Capsules c. 11 × 7 mm, ovoid, glabrous; seeds not seen.

#### Distribution.

Endemic to Brazil, where it is recorded principally from cerrados in Paraná, São Paulo and Minas Gerais states but is perhaps most common in São Paulo and Paraná.

**BRAZIL.** sine data, *W.J. Burchell* A285 (K); sine data, *J.B. Pohl* (OXF). **Minas Gerais**: *A.F. Regnell* Ser. 3, 203 (K); *C.W. Mosén* 4286 (S); *M.M. Arbo et al.* 3936 (CTES, SPF); *P. Clausen* s.n. (K); Santa Bárbara, *L. Duarte* 969 (HB, K). **Paraná**: Laranjeiras do Sul, *G. Hatschbach* 15546 (MB, US); Sengés, *J.R.V. Iganci et al.* 751 (ICN, S); Mun. Tibagí, Fda. Monte Alegre, Harmonia, *G. Hatschbach* 2984 (US); Parque Vila Velha, *G. Hatschbach* 13107 (F); Mun. Arapoti, *G. Hatschbach* 8370 (US); ibid., Rio das Perdizes, *G. Hatschbach* 18838 (CTES, F); Mun. Castro, Carambei, Rio São João, *L. B. Smith et al.* 14475 (US); ibid., *R. Reitz & R.M. Klein* 17887 (F, P, US). **Rio Grande do Sul**: Sengés, *J.R.V. Iganci et al.* 751 (S). **São Paulo**: *I.S. Gottesberger* 930 (FTG); *L. Riedel* 1672 (LE, K); Botucatu, *G. Edwall* 3386 (SP); Mun. Itarare, *V. Souza* 4482 (SPF, CTES); Jabaquara, *M. Kuhlmann* 10.440. (K); Congonhas, *W. Hoehne* 13706 (F, K); Cachambu, *J. Weir* 338 (BM, K).

#### Note.

This species was aptly named *Ipomoea polymorpha* by Meisner because of the very varied leaf form. It is usually with 3-lobed leaves but in specimens with entire leaves, the leaves are oblong. *Hatschbach* 8370 is abnormal in being nearly glabrous. The obtuse sepals are a useful distinguishing feature. Entire-leaved forms (*I. aspersa*) may resemble *I. uruguayensis* but can be recognised by the pubescent leaves which are not grey-tomentose beneath (or only very slightly so), and the inner sepals which have broad, glabrous, scarious margins. They are more common in Paraná State.

### 22. Ipomoea uruguayensis

Taxon classificationPlantaeSolanalesConvolvulaceae

Meisn. in Martius et al., Fl. Brasil. 7: 272. 1869. (Meisner 1869: 272)

Ipomoea megapotamica var. pauciflora Meisn. in Martius et al., Fl. Brasil. 7: 259. (Meisner 1869: 259). Type. SOUTHERN BRAZIL (without exact location). *F. Sello(w*) (possible syntypes BM001125482, F, photo of *F. Sello* 1776 (B†).
Ipomoea lurida Hassl., nom. nud., Addenda ad Plantas Hasslerianas 18. 1917. (Hassler 1917: 18).

#### Type.

URUGUAY or SOUTHERN BRAZIL. *J. Tweedie* s.n. (lectotype K000899637, designated here).

#### Description.

Trailing perennial (but appears to be able to climb fide Rambo collection labels); stems at least 1 m long, shortly crisped-pubescent. Leaves petiolate, 5–13 × 2.5–9 cm, ovate or ovate-elliptic, rounded, truncate or broadly cuneate, apex subacute and mucronate, adaxially pubescent, abaxially paler, more densely pubescent; petioles 1–4.5 cm, pubescent. Inflorescence od long-peduculate (1–)3(–4)-flowered axillary cymes, very occasionally branched and compound; peduncles 5.5–16 cm long, pubescent; bracteoles linear-lanceolate, 6–8 mm long; pedicels 6–30 mm, pubescent; sepals subequal, 10–12 × 5–7 mm, elliptic, acute and shortly mucronate, densely pubescent, inner sepals white tomentose with scarious subglabrous margins; corolla c. 5 cm long, pink, funnel-shaped, pubescent, limb c. 3.5 cm diam., apparently lobed. Capsules and seeds not seen.

#### Distribution.

Apparently restricted to southern Brazil and adjacent eastern Paraguay.

**PARAGUAY. Alto Paraná**: *K. Fiebrig* 6346 (GH, US); cerca de Hernandarias, junto al arroyo Pirapitá, *Fernández Casas et al.* 7326 (NY); Reserva Biológica Tatí Yupí, Itaipú Binacional, *G. Caballero Marmori* 1421 (CTES).

**BRAZIL. Rio Grande do Sul**: *C. Gaudichaud*, Herb. Imp. 668 (P), 672 (P); *O. Bueno* 10668 (CTES, F); *Fox* 62 (K); Morro da Gloria, *B. Rambo* 70 (LIL); Morro de Polizia, near Puerto Alegre, *B. Rambo* 39193 (LIL), Fazenda do Arroio, near Osorio, *B. Rambo* 45240 (P); Mun. Lagoa Vermelha, *A. Krapovickas & C. Cristóbal* 41934 (CTES); Porto Alegre, *P. Ferreira* 119 (CTES).

#### Typification.

In choosing a lectotype for this species, we have selected the only extant *Tweedie* collection. No suitable material was found at B, BR or M.

#### Note.

This species is characterised by the inflorescence that consists of long-pedunculate, usually 3-flowered cymes and by the large ovate leaves, pubescent to subtomentose on both surfaces.

### 23. Ipomoea chodatiana

Taxon classificationPlantaeSolanalesConvolvulaceae

O’Donell, Lilloa 23: 484. 1950. (O’Donell 1950b: 484)

Ipomoea uruguayensis var. glabrata Chodat & Hassl., Bull. Herb. Boiss. Ser. 2: 5: 693. 1905. (Chodat and Hassler 1905: 693). Type. PARAGUAY. Canindeyú, Yeruti, *E. Hassler* 5747 (? holotype G n.v., isotype BM).
Ipomoea uruguayensis forma retusa Chodat & Hassl., Bull. Herb. Boiss. Ser. 2: 5: 693. 1905. (Chodat and Hassler 1905: 693). Type. PARAGUAY. Canindeyú, Ygatimí, *E. Hassler* 4681 (? holotype G n.v., isotypes BM, F, GH, K, P).
Ipomoea uruguayensis var. sericea Chodat & Hassl., Bull. Herb. Boiss. Ser. 2: 5: 693. 1905. (Chodat and Hassler 1905: 693). Type. PARAGUAY. Canindeyú, Curuguaty, *E. Hassler* 4667 (? holotype G n.v. isotypes BM, K, NY).
Ipomoea uruguayensis var. elliptica Chodat & Hassl., Bull. Herb. Boiss. Ser. 2: 5: 693. 1905. (Chodat and Hassler 1905: 693). Type. PARAGUAY. Canindeyú, Ygatimí, *E. Hassler* 4667a (? holotype G n.v.).
Ipomoea polymorpha var. discolor Hassl., Fedde, Repert. Spec. Nov. Regni Veg.9: 155. 1911. (Hassler 1911: 155). Type. PARAGUAY. Canindeyú, Yeruti, *E. Hassler* 5747 (G, BM) and *E. Hassler* 4681 (G, BM, F, K, NY, P), syntypes.
Ipomoea polymorpha forma canescens Hassl. [as var. discolor forma canescens], Fedde, Repert. Spec. Nov. Regni Veg.9: 155. 1911. (Hassler 1911: 155). Type. PARAGUAY. *E. Hassler* 5747 (G, BM) and *E. Hassler* 4681 (G, BM, F, K, P), syntypes.
Ipomoea polymorpha forma argentea Hassl. [as var. discolor forma argentea], Fedde, Repert. Spec. Nov. Regni Veg.9: 155. 1911. (Hassler 1911: 155). Type. PARAGUAY. *E. Hassler* 4667 (BM, G, K, NY) and *E. Hassler* 4667a (G), syntypes.
Ipomoea polymorpha subforma elliptica (Chodat & Hassl.) Hassl. [as var. discolor forma argentea subforma elliptica], Fedde, Repert. Spec. Nov. Regni Veg.9: 155. 1911. (Hassler 1911: 155). Type. Based on Ipomoea uruguayensis var. elliptica Chodat & Hassl.
Ipomoea polymorpha subforma sericea (Chodat & Hassl.) Hassl. [as var. discolor forma argentea subforma sericea], Fedde, Repert. Spec. Nov. Regni Veg.9: 156. 1911. (Hassler 1911: 156). Type. Based on Ipomoea uruguayensis var. sericea Chodat & Hassl.

#### Type.

PARAGUAY. Canindeyú, Ygatimí, *E. Hassler* 4681 (isotypes BM, F, G, GH, K, P).

#### Description.

Trailing or climbing perennial; stems 1–2 m long, pubescent. Leaves shortly petiolate, 3.5–10 × 1.5–7 cm, ovate, obtuse to retuse, mucronate, base rounded to weakly cordate, adaxially green, pubescent, abaxially white-sericeous with long hairs, the veins prominent and mostly without hairs; petioles 3–23 mm, densely pubescent. Flowers solitary, axillary, pedunculate; peduncles (0.5–)3–8 cm, thinly pubescent; bracteoles 2 mm, lanceolate, pubescent, caducous; pedicels 4–13 mm, densely pubescent; sepals subequal, 9–12 mm, ovate or ovate-elliptic, subacute, mucronate, sericeous, base with a conspicuous gland, inner sepals more densely hairy centrally but margin scarious and nearly glabrous; corolla 6–7 cm long, pink, funnel-shaped, midpetaline bands sericeous; limb 4–4.5 cm diam., somewhat lobed. Capsules and seeds not seen.

#### Distribution.

Apparently endemic to Canindeyú in eastern Paraguay, where it appears to be very rare:

**PARAGUAY. Canindeyú**: Col. Ita Poty, *I. Basualdo* 5609 (FCQ).

#### Typification.

We have not seen specimens of the type or of the infraspecific taxa at Geneva, so have not made any lectotypification. The Geneva specimens may, in fact, serve as holotypes.

#### Note.

Resembles *Ipomoea nitida* but the flowers are solitary and the leaves strongly discolorous and abaxially silvery.

### 24. Ipomoea nitida

Taxon classificationPlantaeSolanalesConvolvulaceae

Griseb., Symb. Fl. Argent. 264. 1879. (Grisebach 1879: 264)

Ipomoea malveoides var. nitida (Griseb.) Hallier f., Bull. Herb. Boiss. 7 (5), append. 1: 52. 1899. (Hallier 1899c: 52).

#### Type.

ARGENTINA. Entre Ríos, weiden bei Concordia, 15 Feb. 1876, *Lorentz* 719 (holotype GOET002520, photo of isotype from B† at F).

#### Description.

Trailing perennial; stems 1–3 m long, glabrous to pubescent. Leaves petiolate, 4.5–18 × 1.5–17 cm, oblong-lanceolate to elliptic-rhomboid, base truncate to cuneate, apex subacute to rounded, mucronate, both surfaces green, subglabrous, adpressed pubescent to sericeous; petioles 1.3–4.5 cm, glabrous to pubescent. Inflorescence of pedunculate axillary, usually compounded cymes with up to 8 flowers; primary peduncles 1–11 cm, glabrous or pubescent; secondary peduncles 2.5–6.7 cm; tertiary peduncles sometimes present; bracteoles lanceolate, 1.5–2 mm, nearly glabrous to sericeous, caducous; pedicels 8–23 mm, glabrous or pubescent; sepals subequal, 8–10 × 5–6 mm, outer sepals ovate, acute, pubescent, inner sepals oblong-elliptic, obtuse, more densely pubescent with nearly glabrous margins; corolla 4.5–6.5 cm long, pink, funnel-shaped, pubescent; limb 4–5 cm diam.,undulate. Capsules 10–13 × 9 mm, ovoid, glabrous; seeds 6–7 × 3.5 mm, black, obscurely pubescent.

#### Variation.

This species is divisable into two relatively well-marked geographical subspecies based principally on leaf shape and inflorescence development.

### 24a. Ipomoea nitida subsp. nitida

Taxon classificationPlantaeSolanalesConvolvulaceae

#### Diagnosis.

Leaves oblong-lanceolate, 4.5–11 × 1–3.5 cm, base cuneate, both surfaces finely sericeous; inflorescence of 1–3-flowered cymes.

#### Illustration.

O’Donell (1959b: 205).

#### Distribution.

Apparently endemic to the Department of Entre Ríos in Argentina.

**ARGENTINA. Entre Ríos**: Concordia, *Meyer* 10997 (LIL); ibid., *A. Krapovickas & C. Cristóbal* 46563 (CTES); ibid., Parque Rivadavia, *A. Burkart & Gamerr* 21873 (K, SI); Federación: *A. Burkart* 26713 (F); ibid., Santa Ana, *A. Burkart & S. Crespo* 23090 (SI).

#### Note.

This subspecies is very localised and morphologically uniform.

### 24b. Ipomoea nitida subsp. krapovickasii

Taxon classificationPlantaeSolanalesConvolvulaceae

J.R.I. Wood & Scotland subsp. nov.

urn:lsid:ipni.org:names:77208065-1

#### Type.

ARGENTINA. Prov. Corrientes, Depto. Santo Tomé, 16 km N de Santo Tomé, *A. Schinini* 19971 (holotype CTES, isotypes K, MO).

**Diagnosis.** Resembling subsp. nitida but leaves 7–18 × 4.5–17 cm, elliptic-rhomboid, base truncate to very broadly cuneate, adaxially usually glabrous, abaxially usually thinly pubescent, sometimes densely so or glabrous. Inflorescence commonly of compounded cymes with up to 8 flowers.

#### Distribution.

In “hilly” grassland in NE Argentina and adjacent parts of Brazil. **ARGENTINA. Corrientes**: Ituzaingó, *H. Keller et al.* 5366 (CTES); Mercedes, *T.M. Pedersen* 5359 (A, C, K, S); San Martín, *Medina et al. 289* (CTES, K); Santo Tomé, *T.S. Ibarrola* 1597 (LIL, S). **Misiones**: Capital, *H. Keller et al.* 12033 (CTES); Apóstoles, *C. Cristóbal et al.* 1910 (CTES).

**BRAZIL. Mato Grosso do Sul**: 7 km de Ponta Pora, a Dourados, *A. Krapovickas & C. Cristóbal* 34289 (CTES). **Rio Grande do Sul**: 11 km E of Sâo Borja, *J.C. Lindemann & A. Pott* 21094, (CTES, F); Sâo Borja, *P.P.A. Ferreira & J. Durigon* 575 (K); ibid., *P.P.A. Ferreira* 270 (CTES).

#### Notes.

This subspecies is very variable in indumentum and the number of flowers per cyme but is never sericeous on both surfaces of the leaf and the leaves are characteristically elliptic-rhomboid. It might merit recognition as a distinct species.

*Krapovickas et al.* 18066 (CTES) from Ituzaingo in Corrientes (Argentina) is near glabrous but the leaves are often 3-lobed so approaching forms of *Ipomoea padillae*, another indication of introgression in this clade.

### 25. Ipomoea psammophila

Taxon classificationPlantaeSolanalesConvolvulaceae

J.R.I. Wood & Scotland, Kew Bull. 70 (31): 48. 2015. (Wood et al. 2015: 31)

#### Type.

BOLIVIA. Santa Cruz, Prov. Chiquitos, entrando hacia Motacú por San Juanama, near Santiago de Chiquitos, *J.R.I. Wood, D. Soto, P. Pozo, W. Hawthorne & D. Villarroel* 25122 (holotype USZ, isotypes K, LPB, UB).

#### Description.

Vigorous trailing perennial herb; rootstock, woody, forked; stems angled, obscurely bifariously puberulent, glabrescent. Leaves shortly petiolate, 3–7.5 × 1–4.5 cm, ovate, elliptic to suborbicular, apex emarginate and mucronate, obtuse or rounded, base truncate to very shallowly cordate, margin entire, green and glabrous on both surfaces; petioles 3–9 mm, glabrous to pubescent. Inflorescence of (1–)3(–5)-flowered axillary cymes; peduncles 1.5–9 cm, glabrous to very thinly pubescent; secondary peduncles (when present) 7–8 mm; bracteoles 1.5 × 0.5 mm, lanceolate, obtuse, caducous; pedicels 3–10 mm, pubescent; sepals subequal, 11–12 mm long, outer sepals narrowly ovate, obtuse to subacute, puberulent to pubescent, inner sepals ovate-elliptic, thinly to densely pubescent, c. 1 mm longer, margins scarious; corolla 5 –7 cm long, pink, funnel-shaped, in bud pubescent, limb c. 7 cm diam., shallowly lobed. Capsules c. 13 × 10 mm, ovoid, rostrate with mucro 1.5 mm long, glabrous; seeds 7 × 3.5 mm, oblong, brown, obscurely puberulent but appearing glabrous, minutely scaly on margin.

#### Illustration.

Figure 23.

**Figure 23.**
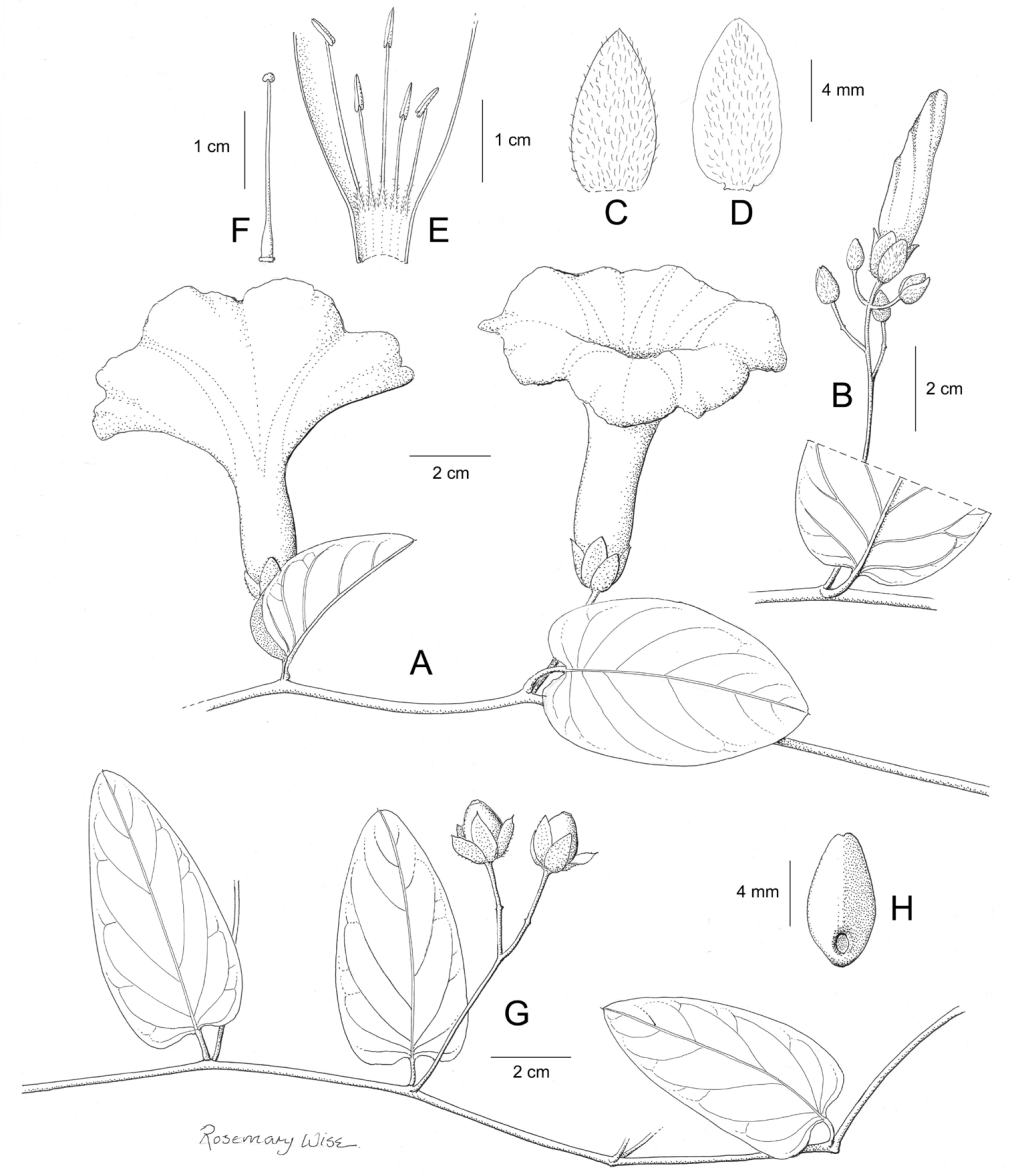
*Ipomoea psammophila*. **A** habit **B** flowering shoot **C** outer sepal **D** inner sepal **E** corolla opened out to show stamens **F** ovary and style **G** habit showing fruiting inflorescence with capsule **H** seed. Drawn by Rosemary Wise **A**, **C–F** from *Wood* 20691; **B** from *Wood et al.* 23578; **G–H** from *Wood* 27910.

#### Distribution.

Endemic to Bolivia, where it grows in cerrado on sandy soil in two areas of Santa Cruz Department.

**BOLIVIA. Santa Cruz**: Chiquitos, around Santiago de Chiquitos *R. Guillén et al.* 4799 (MO, USZ); *J.R.I. Wood et al.* 20171 (BOLV, K, LPB, USZ); south of Taperas *J.R.I. Wood et al.* 23578 (K, LPB, UB, USZ); south of San José de Chiquitos, *J.R.I. Wood et al.* 29159 (LPB, USZ).

#### Note.

Resembles Ipomoea nitida Griseb., particularly subsp. krapovickasii, but the leaves are glabrous or obscurely pubescent, their base cordate to truncate, rather than truncate to cuneate, the petioles very short (0.3–0.9 cm, not 2–4 cm), the cymes usually 1–3-flowered (not up to 7-flowered) and the sepals green, pubescent, rather than grey-tomentellous, 11–12 mm (not 7–9 mm) long. Molecular data suggest the two species are not closely related.

### 26. Ipomoea altoparanaensis

Taxon classificationPlantaeSolanalesConvolvulaceae

O’Donell, Arq. Mus. Paranaense 9: 210. 1952. (O’Donell 1952: 210)

Ipomoea paranaensis Hassl., nom. nud. Add. Plantae Hasslerianae 18. 1917. (Hassler 1917: 18).

#### Type.

PARAGUAY. Alto Paraná, *K. Fiebrig* 5812 (holotype LIL001231, isotype GH, K, SI).

#### Description.

Trailing perennial; stems stout, densely tomentellous. Leaves petiolate. 5–11 × 2–8 cm, ovate to elliptic, obtuse, mucronate, margin entire to slightly undulate, base broadly cuneate to shallowly cordate, both surfaces sericeous-tomentose, the venation highlighted; petioles 2–5 cm,tomentose. Inflorescence of long-pedunculate 3–5-flowered cymes; peduncles 5–19 cm, tomentose; bracteoles 9–10 × 2.5 mm, tomentose, caducous; secondary and tertiary peduncles 2–4.5 cm; pedicels 6–25 mm, densely tomentose; sepals subequal or interior slightly shorter, 10–14 × 8–11 mm, broadly elliptic to subglobose, obtuse and mucronate, tomentose; corolla 7–9 cm long, funnel-shaped, pink, tomentose, limb 5 cm diam. Capsules and seeds unknown.

#### Distribution.

Endemic to Paraguay and only known from two collections. It grows in open cerrado.

**PARAGUAY. Alto Paraná**: Hernandias, Prop. Takurú Pukú de la Itaipú Binacional, *M. Vera et al.* 2384 (FCQ).

#### Note.

Very distinct are the silvery sericeous leaves with highlighted veins. It is very like forms of *Ipomoea nitida* from Corrientes but with the distinct sericeous-tomentose indumentum.

### 27. Ipomoea lanuginosa

Taxon classificationPlantaeSolanalesConvolvulaceae

O’Donell, Lilloa 23: 445. 1950. (O’Donell 1950a: 445)

#### Type.

ARGENTINA. Misiones, San Ignacio, 31 March 1948, *C. O’Donell* 5611 (lectotype LIL001249, designated here).

#### Description.

Decumbent perennial with thick root tubers, stems 3–6 m long, densely lanate but eventually glabrescent. Leaves petiolate, 4–10 × 1.5–4.5 cm, broadly to narrowly ovate-elliptic, usually simple, sometimes weakly 1–2-lobed, rarely 5-partite, obtuse to acute, base rounded to cuneate, both surfaces woolly, the lower surface densely so; petioles 1–2(–6) cm. Inflorescence of compact pedunculate, axillary cymes; peduncles 4–12 (–20) cm, lanate; bracteoles 5–12 × 2–3 mm, lanceolate, lanate, moderately persistent; secondary peduncles, if present, 2–4.5 cm; tertiary peduncles (if present) up to 2.5 cm; pedicels often short, 0 –13 mm, densely lanate; outer sepals 10–14 mm, elliptic, lanate, obtuse; corolla 5–8 cm long, pink, the tube purplish inside, midpetaline bands woolly, limb c. 5 cm diam. Capsules glabrous; seeds densely tomentose, black.

#### Illustration.

O’Donell (1950a: t. 9).

#### Distribution.

A very rare, possibly extinct species known from single locations in Argentina, Paraguay and Brazil. Not recorded from Paraguay since 1943 or from Argentina since 1949 despite search in the San Ignacio area by Hector Keller. Probably a cerrado species.

**ARGENTINA. Misiones**: San Ignacio, *E.L. Ekman* 1420 (NY, S); ibid., *G. J. Schwarz* 5446 (K, P).

**PARAGUAY. Itapúa**: Encarnación, *Spagazzini* 23/1/1907 (LPS); ibid., *L. Jiménez* 37 (SCP).

**BRAZIL. Rio Grande do Sul**: *Hagelund* 3300C (ICN), fide Ferreira and Miotto (2009: 446).

#### Note.

This species is characterised by the white lanate indumentum, very short, densely lanate pedicels and the moderately persistent, relatively large bracteoles.

### 28. Ipomoea megalantha

Taxon classificationPlantaeSolanalesConvolvulaceae

J.R.I. Wood & Scotland, Kew Bull. 72 (9): 18. 2017. (Wood and Scotland 2017a: 18)

#### Type.

PARAGUAY. In viciniis Caaguazú, *E. Hassler* 9114 (holotype BM00089494, isotypes, G, K, MO, NY, P, SI, S, US).

#### Description.

Perennial subshrub; root a woody xylopodium of unknown size but at least 2 cm thick and 8 cm long; stems decumbent or ascending, woody, pilose, glabrescent when old, 10–40 cm long. Leaves shortly petiolate, 1.5–9.5 × 0.5–5, oblong to ovate, obovate or elliptic, often trifurcate on the same plant, apex obtuse or acute, mucronate, base broadly to narrowly cuneate, margin entire, both surfaces pilose, more densely so on the veins; petioles 2–9 mm, pilose. Inflorescence of solitary, axillary flowers arising from towards the base of the stem; peduncles 2.5–6 cm, pilose; bracteoles 13–27 × 1–3 mm, linear-lanceolate, pilose, persistent; pedicels 3–11 mm, pilose; sepals slightly unequal, lanceolate, finely acuminate, outer 17–20 × 3–6 mm, abaxially pilose, inner up to 22 mm long, the central area pilose, the margins scarious, glabrous; corolla 8.5–9.5 cm long, ±funnel-shaped, gradually widened from base, midpetaline bands densely pilose; limb 5–6 cm, diam., unlobed. Capsules and seeds unknown.

#### Illustration.

Figures 8L, 24.

**Figure 24.**
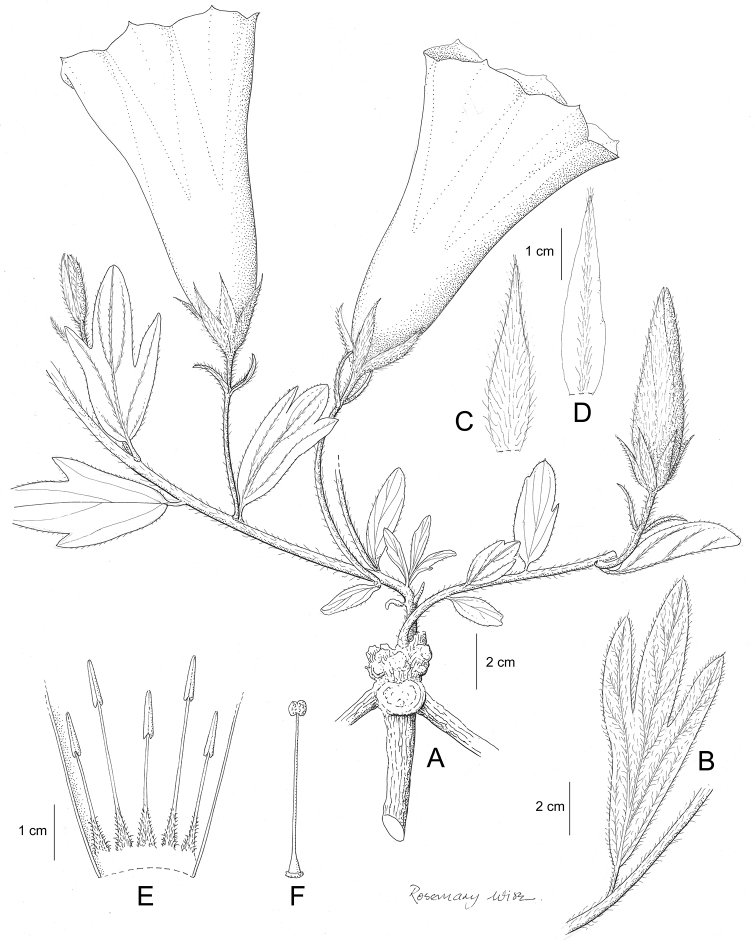
*Ipomoea megalantha*. **A** habit **B** leaf **C** outer sepal **D** inner sepal **E** corolla opened out **F** ovary and style. Drawn by Rosemary Wise **A**, **C–F** from *Hassler* 9114; **B** from *Jorgensen* 4859.

#### Distribution.

Only known from the Department of Caaguazú in Paraguay, where it grows in cerrado.

**PARAGUAY. Caaguazú**: *B. Balansa* 1174 (P); *P. Jorgensen* 4859 (A, F, S); *A. Krapovickas et al.* 45769 (CTES, K).

#### Note.

*Ipomoea megalantha* is distinguished by its large corolla about 9 cm in length. It is similar in habit and indumentum to *I. hirsutissima* but also differs in its trifurcate leaves. *Ipomoea acutisepala* has longer trailing stems, leaves with petioles 1–3 cm long, shorter, somewhat caducous bracteoles, a usually branched inflorescence, shorter sepals (13–17 mm long) and shorter corolla.

### 29. Ipomoea hirsutissima

Taxon classificationPlantaeSolanalesConvolvulaceae

Gardner, Icon. Pl. sub t. 471. 1842. (Gardner 1842a: t. 471)

Ipomoea chrysotricha Meisn. in Martius et al., Fl. Brasil. 7: 243. 1869. (Meisner 1869: 243). Type. BRAZIL. São Paulo, “in campis R. Pardo,” L. Riedel 610 (lectotype LE, sheet with Convolvulus crossed out and replaced with “Ipomoea chrysotricha Meißn. n. sp.”, designated by Wood and Scotland 2017a: 18), isolectotype NY).
Ipomoea chrysotricha Meisn. var. ovata Meisn. in Martius et al., Fl. Brasil. 7: 243. 1869. (Meisner 1869: 243). Type. BRAZIL. Serra de Christaes, J.B. Pohls.n. (BR0000530689, possible isotype).
Ipomoea chrysotricha Meisn. var. boliviana Meisn. in Martius et al., Fl. Brasil. 7: 243. 1869. (Meisner 1869: 243). Type. BOLIVIA. Santiago de Chiquitos, *A. D’Orbigny 928* (lectotype P03878901, designated by Wood et al. 2015: 38).
Ipomoea punicea var. rariflora Meisn. in Martius et al., Fl. Brasil. 7: 242. 1869. (Meisner 1869: 242). Type. BRAZIL. Minas Gerais, L. Riedels.n. (lectotype LE01025979, designated here).
Ipomoea hirsutissima var. integrifolia Chodat & Hassl., Bull. Herb. Boiss. Ser. 2: 5: 688. 1905. (Chodat and Hassler 1905: 688). Type. PARAGUAY. Canindeyú, Ipe Hú, Sierra de Maracayú, *E. Hassler* 5007 (lectotype G00174906, designated by Wood and Scotland 2017a: 18, isolectotypes F, G, K, P, S, UC).
Ipomoea hirsutissima var. repens Glaz. Bull. Soc. Bot. France 57, mém. 3e: 481. 1910. (Glaziou 1910: 481). Type. BRAZIL. *A.F.M. Glaziou* 21791 (holotype P03536444, isotypes BR, G, K).

#### Type.

BRAZIL. Goiás, Mision of Duro, Oct. 1839, *G. Gardner* 3355 (lectotype K000612806, designated by Wood and Scotland 2017a: 18, isolectotypes BM, F, GH, K, NY, P, SP).

#### Description.

Erect herb to about 40 cm with a large woody tuberous root, the whole plant densely pilose with rather stiff white hairs swollen at the base. Leaves subsessile, 3–8 × 1–3.5 cm, oblong–obcuneate, obtuse, base cuneate, both surfaces pilose, green; petioles 0–2 mm. Inflorescence of solitary (rarely paired), pedunculate axillary flowers arising from the upper leaf axils; peduncles 1–4 cm; bracteoles 10–25 × 1–1.5 mm, linear-lanceolate, finely acuminate, caducous; pedicels 3–6 mm; sepals slightly unequal, narrowly ovate, acuminate, pilose, 13–16 × 4 mm, inner sepals similar but with broad, glabrous margins; corolla 6–7 cm long, funnel-shaped, gradually widened from base, pink, pilose, the hairs with dark bases, limb c. 5 cm diam., shallowly lobed. Capsules 12 × 5 mm, narrowly ovoid, glabrous; seeds 7 × 2–3 mm, dark brown, glabrous except for shortly pilose angles.

#### Illustration.

**Figure 25.**
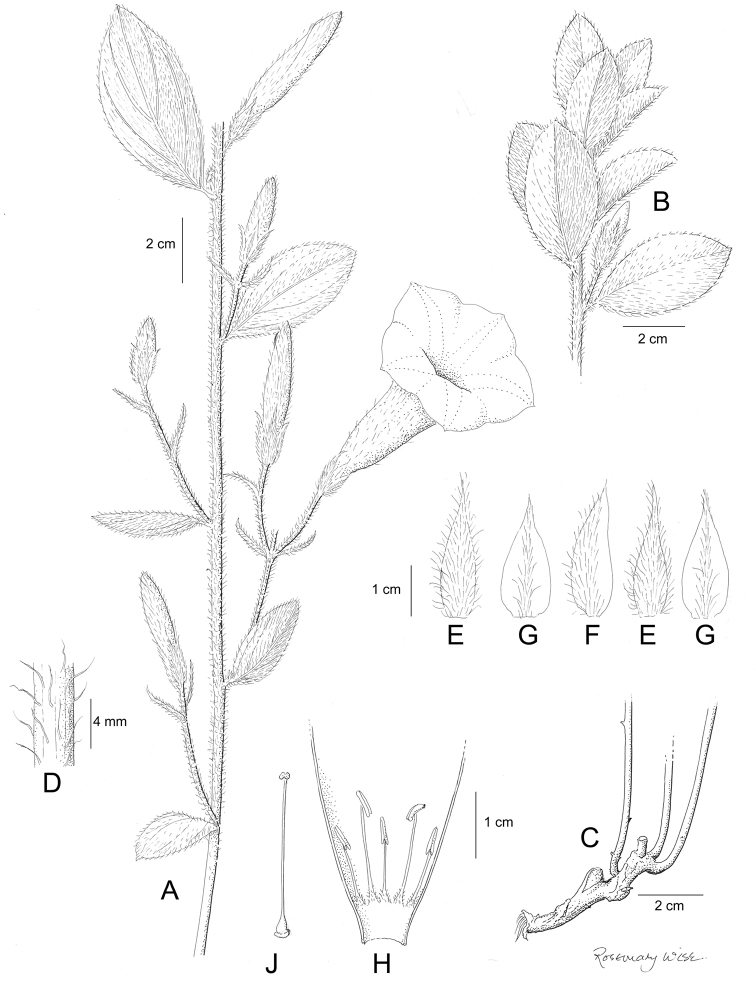
*Ipomoea hirsutissima*. **A** habit (lower stem) **B** habit (stem apex) **C** stem base **D** stem indumentum **E** outer sepals **F** middle sepal **G** inner sepals **H** corolla opened out **J** ovary and style. Drawn by Rosemary Wise from *Wood & Guzmán* 17405.

Figures 4D, 25; Wood et al. (2015: 40).

#### Distribution.

Widely distributed in the cerrados of Brazil, Paraguay and Bolivia but nowhere very frequent.

**PARAGUAY. Alto Paraná**: *G. Caballero* s.n. (G). **Canindeyú**: type of Ipomoea hirsutissima var. integrifolia.

**BRAZIL. Dist. Fed.**: Brasilia, *E. Pereira* 4854 (RB). **Goiás**: *A.F.M. Glaziou* 21791a (K); Serra dos Pireneus, *H.S. Irwin et al.*10815 (MO, NY); Alto Paraíso, *da Silva et al.* 2428 (IBGE, K); c. 5 km de Niquelândia, *M.L. Fonseca et al.* 1234 (IBGE, K); Mimosa de Goiás, *M. Mendoza* 4365 (CEN); Minacú, *B.M.T. Walter* 793 (CEN); Mun. Água Fria, *G. Hatschbach et al.* 58314 (MBM). **Mato Grosso do Sul**: *A. Pott* 15189 (UFMA). **Minas Gerais**: Serra da Anta, c. 2 km N of Paracatú, *H.S. Irwin* 26055 (NY). **Pernambuco**: Petrolândia, *E.P. Heringer et al.* 12822 (NY). **São Paulo**: type of *Ipomoea chrysotricha*.

**BOLIVIA. Santa Cruz**: Santiago de Chiquitos, *J.R.I. Wood & E. Guzmán* 17405 (K, LPB, USZ); Germán Busch, Cerro Mutún, *I.G. Vargas et al.* 3240 (F, NY).

#### Note.

A very distinct species because of its erect habit, subsessile leaves and stiff spreading hairs, which cover almost all parts of the plant including the corolla.

### 30. Ipomoea aurifolia

Taxon classificationPlantaeSolanalesConvolvulaceae

Dammer, Bot. Jahrb. 23, Beibl. 57: 39. 1897. (Dammer 1897: 39)

Ipomoea stenophylla var. aurifolia (Dammer) Hallier f., Jahrb. Hamburg. Wiss. Anst. 16, beiheft 3: 54. 1899. (Hallier 1899a: 54).

#### Type.

BRAZIL. Goiás, Rasgão, Corumbá [de Goiás], *A.F.M. Glaziou* 21798 (holotype B†, photo F, isotypes BR, G, R).

#### Description.

Erect, usually branched perennial from woody xylopodium 20–40 cm high, stem asperous-pilose especially when young. Leaves subsessile, 3–6.5 × 0.5–2 cm, lanceolate to narrowly oblong-ovate, obtuse and mucronate, cuneate at base, densely adpressed asperous pilose on both surfaces. Flowers 1–3 (often solitary) in shortly pedunculate, dense axillary cymes from the uppermost leaf axils, all parts densely hirsute; peduncles 0.5–2 cm; bracteoles linear-lanceolate, acuminate 8–20 × 2–3.5 mm, persistent; pedicels 0–2 mm; sepals subequal, 8–10 mm, ovate, obtuse, densely pilose with stiff golden hairs, inner more obtuse, the margins glabrous, scarious; corolla 5–5.5 cm long, funnel-shaped, pink, densely stiffly adpressed pilose; limb c. 2.5 cm diam. Capsules and seeds not seen.

#### Illustration.

Figure 26.

**Figure 26.**
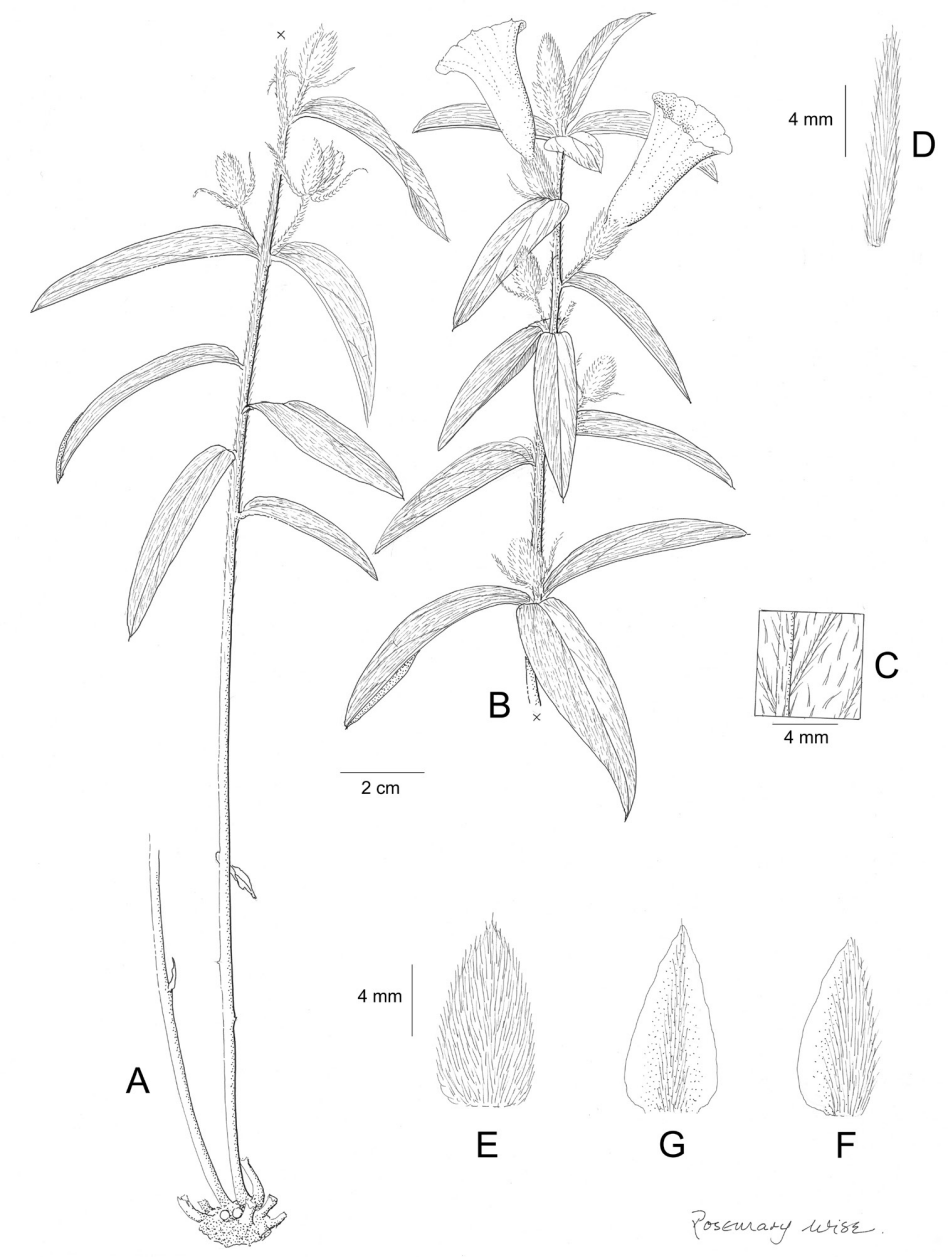
*Ipomoea aurifolia*. **A** habit with xylopodium **B** habit and inflorescence **C** abaxial leaf surface **D** bracteole **E** outer sepal **F** middle sepal **G** inner sepal. Drawn by Rosemary Wise from *Heringer et al.* 5912.

#### Distribution.

Endemic to Brazil, growing in cerrado in and around the Distrito Federal and neighbouring parts of Goiás.

**BRAZIL. Dist. Fed./ Goiás**: 12 km E of Brazlândia on road to Brasilia, 1225 m, 22 Nov. 1965, *H.S. Irwin et al*.10585 (NY, MO); *Pereira* 861 (RB); Luzuânia, *E.P. Heringer* 14887 (UB); IBGE Reserva Ecológica, *E.P. Heringer et al.* 5912 (IBGE, K); Faz. Água Limpa, *G. Kirkbride* 1573 (F).

#### Note.

Similar in general facies to *Ipomoea hirsutissima* with which it may intergrade but often more slender in habit, the indumentum appressed, rather than spreading, leaves lanceolate, flowers mostly in the uppermost leaf axils the sepals rounded to obtuse (never acuminate to a fine point) and densely covered in golden hairs.

### 31. Ipomoea pyrenea

Taxon classificationPlantaeSolanalesConvolvulaceae

Taub., Bot. Jahrb. 21: 449. 1895. (Taubert 1895: 449)

#### Type.

BRAZIL. Goiás, Serra dos Pyreneus, *Ule* 3011 (holotype B†, isotypes HBG 506564, P03551472, R000040279).

#### Description.

Erect subshrub to c. 30 cm from a woody xylopodium, stem densely asperous-pilose. Leaves subsessile, 2.5–5 × 0.3–0.7 cm, narrowly oblong-oblanceolate, base narrowly cuneate, apex acute and mucronate, thinly but roughly pilose on margin and veins of both surfaces; petioles <2 mm long. Inflorescence congested, terminal, the flowers solitary, subsessile, from the uppermost leaf axils; peduncles 0–4 mm; bracteoles 7–8 mm, linear-lanceolate, thinly pubescent, ±equalling the sepals; pedicels absent; sepals subequal, 8–10 × 4 mm long, ovate, acuminate, appressed-pilose, inner obtuse to subacute and mucronate, the margins scarious, subglabrous; corolla 3.5–4.5 cm long, funnel-shaped, pink, appressed pilose, limb c. 2 cm diam. Capsules and seeds not seen.

#### Illustration.

Figure 27.

**Figure 27.**
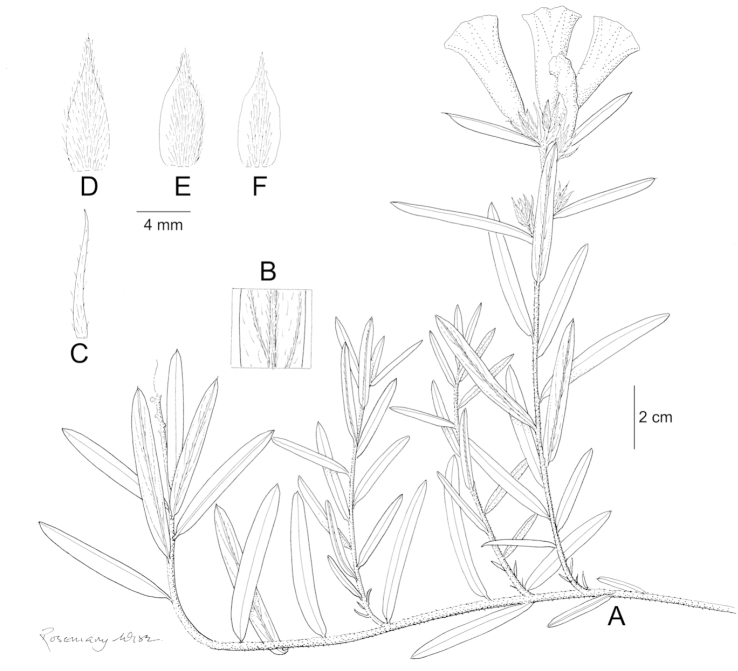
*Ipomoea pyrenea*. **A** habit **B** abaxial leaf surface **C** bracteole **D** outer sepal **E** middle sepal **F** inner sepal. Drawn by Rosemary Wise from *Irwin et al.* 34376.

#### Distribution.

Endemic to the Serra de Pireneus in Goiás State, Brazil, growing at relatively high altitudes of 1000–1300 m.

**BRAZIL. Goiás**: Serra de Pireneus, *A.Macedo* 3501 (NY); ibid., *H.S. Irwin* et al. 24377 (NY); ibid., *W.R. Anderson et al.* 34376 (FTG, NY, SP); ibid., *G. Hatschbach et al.* 70081 (MBM); ibid., *D.P. Saraiva et al.* 275 (RB, SP).

#### Note.

Somewhat similar to *Ipomoea aurifolia*, but leaves oblong, rather than lanceolate, narrower (<7 mm wide) and much more thinly hairy, outer sepals acuminate and the inflorescence more strictly terminal.

### 32. Ipomoea subspicata

Taxon classificationPlantaeSolanalesConvolvulaceae

(Meisn.) O’Donell, Lilloa 23: 501. 1950. (O’Donell 1950b: 501)

Ipomoea virgata var. subspicata Meisn. in Martius et al., Fl. Brasil. 7: 241. 1869. (Meisner 1869: 241). Type. BRAZIL. Minas Gerais, Caldas, *Lindberg 163* (lectotype BR00005306435, designated here; isolectotype S12-2160).

#### Type.

Based on Ipomoea virgata var. subspicata Meisn.

#### Description.

Erect undershrub to 80 cm with tuberous rootstock, stems densely pubescent, glabrescent, branched at base but otherwise simple. Leaves subsessile, 2–6 (–9) × 0.5–2 (–3.5) cm, broadly oblong to oblong-elliptic, subacute (sometimes mucronulate), entire or undulate, base broadly cuneate, thinly to densely pubescent on both surfaces but especially below; petioles 0–3 mm, pubescent. Flowers in a leafy terminal raceme, solitary or in 2–3 flowered cymes, peduncles 2–6 mm, pubescent; bracteoles 1–3 mm long, lanceolate, caducous; pedicels 4–7 mm, pubescent; sepals 7–12 mm, almost equal, lanceolate to oblong, obtuse to subacute, tomentose, the inner with scarious, glabrous margins; corolla 4.5–6 cm long, deep pink, funnel-shaped, sericeous in bud and on midpetaline bands, limb unlobed, 2.5–3 cm diam. Capsules 9–10 × 6 mm long, ellipsoid, glabrous; seeds 5 × 2.5 mm long, lanate with reddish marginal hairs.

#### Distribution.

An uncommon cerrado species from south-central Brazil.

**BRAZIL. Dist. Fed.**: *Freitas & Freitas* s.n. [1996] (UB). **Minas Gerais**: *C.W. Mosén* 958 (S), 4290 (S); Paracatu, *A. Glaziou* 21788 (K, P); Caldas,); ibid., *W.H. Stubblebine et al.* 503 (UEC), 598 (UEC); ibid., *Leitão Filho et al.* 1916 (UEC); P.N. Grande Sertão Veredas, *D. Alvarenga et al.* 1129 (IBGE, OXF). **Paraná**: *G. Hatschbach* 13291 (RB). **São Paulo**: near Brotas, *Weir* 153 (K); *C.W. Mosén* 4289 (S); *A. Saint-Hilaire* 1068 (K, P); Mun. Moji-Guaçu, *G. Eiten & Machado de Campos* 1493 (NY, SP); ibid., *J. Mattos* 9629 (SP).

#### Notes.

This species resembles *Ipomoea hirsutissima* in habit and leaves but lacks the spreading hairs and has less acute sepals. It was treated as *Ipomoea campestris* in Flora do Brasil 2020 under construction and is undoubtedly closely related but differs in the broader, shortly acute leaves and less finely acute sepals. The two species may intergrade but more detailed study is needed.

*R.M. Harley et al.* 24982 (FTG, K) from Minas Gerais, Mun. Buenópolis, Serra do Cabral in Brazil is very similar but appears to be be prostrate and may represent a different taxon.

Two collections from Santiago de Chiquitos in Bolivia (*A. D’Orbigny* 927, P035360730, and *J.R.I. Wood & D. Soto* 23444 [K, USZ]), collected about 170 years apart, are also similar in facies to *Ipomoea subspicata*. The leaves of these specimens somewhat resemble those of *I. psammophila* but the habit and more acute sepals suggest an affinity with *I. hirsutissima* and the indumentum is somewhat intermediate between these two species. As these are the only two species from this clade occurring at Santiago, it is possible that these collections represent a hybrid, something possibly corroborated by the nuclear data which places *Wood & Soto* 23444 as sister to *I. psammophila*. If this supposition eventually proves correct, hybridisation could turn out to be a factor complicating species delimitation in a number of the species clusters in this clade.

### 33. Ipomoea cerradoensis

Taxon classificationPlantaeSolanalesConvolvulaceae

J.R.I. Wood & Scotland, Kew Bull. 70 (31): 39. 2015. (Wood et al. 2015: 39)

#### Type.

BOLIVIA. Santa Cruz, Prov. Velasco, Parque Nacional Noel Kempff Mercado, la meseta, camino al Camp. Huanchaca 2, *J.R.I. Wood, D. Villarroel & M. Mendoza* 27017 (holotype K, isotypes USZ, LPB).

#### Description.

Erect or ascending herb to 50 cm, rootstock a woody xylopodium with small tubers, stem adpressed pubescent. Leaves shortly petiolate, 3–6 × 1–3.5 cm, ovate to elliptic, base broadly cuneate to rounded, apex obtuse to rounded, minutely mucronate, margin entire, both surfaces densely pubescent with slightly asperous hairs, abaxially paler with prominent dull red veins; petiole 2–5 mm, pubescent. Inflorescence of shortly pedunculate axillary cymes, commonly reduced to 1–2 flowers; peduncles 1–15 mm, pubescent; bracteoles 2–3 × 1 mm, oblong-lanceolate, caducous; pedicels 2–4 mm, pubescent; sepals subequal, outer 6–7 × 4–5 mm, ovate, acute to obtuse, mucronulate, pubescent, inner 6 × 4–5 mm, ovate-suborbicular, obtuse, mucronulate, pubescent, margin narrow, scarious; corolla pubescent in bud, somewhat glabrescent, white (rarely very pale pink), 5–6 cm long, funnel-shaped, limb c. 3 cm diam, indistinctly lobed. Capsules and seeds not seen.

#### Illustration.

Figure 28.

**Figure 28.**
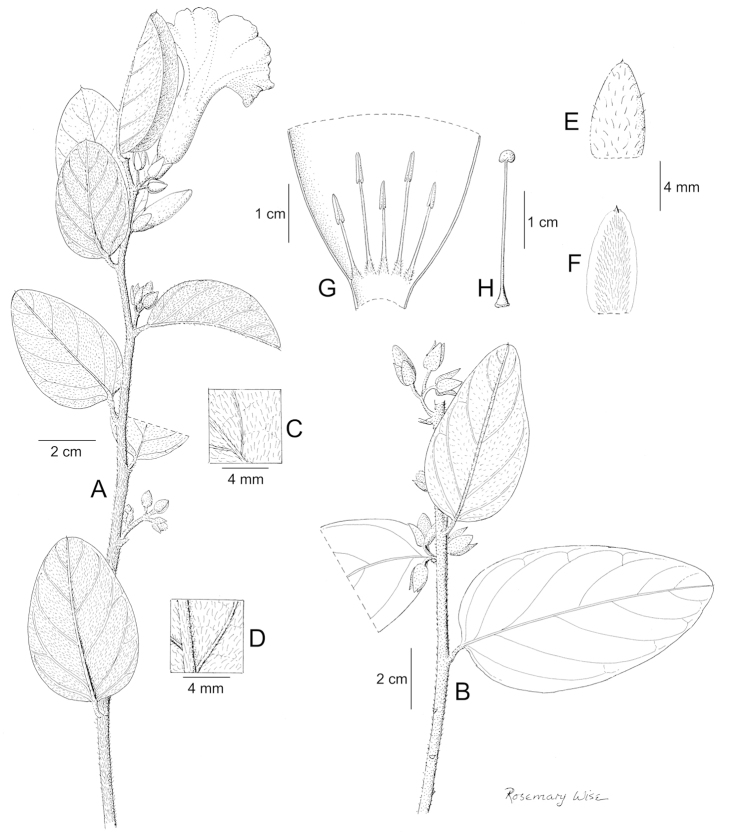
*Ipomoea cerradoensis*. **A** habit **B** habit **C** adaxial leaf surface **D** abaxial leaf surface **E** outer sepal **F** inner sepal **G** corolla opened out to show stamens **H** ovary and style. Drawn by Rosemary Wise **A, C–H** from *Wood et al.* 27017; **B** from *Irwin & Soderstrom* 7399.

#### Distribution.

Open cerrado (*campo sujo*) with scattered shrubs, often near rock outcrops on the serranias and chapadas of the Precambrian shield of Bolivia and Brazil between 700 and 1000 m.

**BRAZIL. Goiás**: Serra do Caiapó *H.S. Irwin & T.R. Soderstrom* 7399 (NY); Chapada dos Veadeiros, *H.S. Irwin et al.* 24544 (NY); Goiânia, *A. Luna Peixoto et al.* 746 (RB); Cavalcante, *G. Pereira-Silva et al.* 5772 (CEN). **Minas Gerais**: Selviria, *O. Tiritan & M. Paiva 436* (RB). **Rondônia**: Velhena, *M.G. Veira et al.* 783 (US).

**BOLIVIA. Santa Cruz**: Velasco, P.N. Noel Kempff Mercado, Las Gamas, *R. Guillen & T. Centurión* 859 (MO, USZ).

#### Notes.

A relatively distinct cerrado species with the characteristically shortly petiolate leaves of an erect or ascending species. It is similar to *Ipomoea hirsutissima* and *I. aurifera* in habit but is distinguished by the pubescent indumentum, ovate leaves and sepals, and shortly pedicellate white flowers borne in small axillary cymes.

The Rondônia collection, *Veira et al.* 783, is somewhat anomalous having slightly larger sepals and pink flowers (according to the collection label). In habit and other details it fits *Ipomoea cerradoensis* and is probably correctly placed here unless further collections from Rondônia prove otherwise.

### 34. Ipomoea

Taxon classificationPlantaeSolanalesConvolvulaceae

sp. B (E. Hassler 6760)

#### Description.

Subshrub 1–1.5 m high; stems adpressed pubescent. Leaves very shortly petiolate, 4–15.5 × 1.5–7 cm, ovate to ovate-elliptic, apex obtuse and shortly mucronate, base broadly cuneate, both surfaces thinly pubescent, green, abaxially slightly paler; petioles 0–5 mm, puberulent. Inflorescence of axillary cymes, occasionally compounded or reduced to single flowers; peduncles 0.8–3.8 cm, stout, puberulent; bracteoles 1–2 mm long, ovate, caducous, puberulent; secondary peduncles 0.3–1.8 cm; pedicels 4–14 mm, puberulent; sepals subequal, ovate-elliptic, outer 6–7 × 3.5–4 mm, obtuse and shortly mucronate, puberulent with narrow scarious margins; inner c. 1 mm longer, rounded, pubescent with broad, glabrous, scarious margins; corolla c. 6 cm long, pink, funnel-saped, pubescent in bud; limb 4–4.5 cm diam. Capsules and seeds not seen.

#### Distribution.

Only known from a single collection.

**PARAGUAY. Cordillera**: *E. Hassler* 6760 (BM, F, MO, P, S).

#### Note.

*Ipomoea* sp. B is most similar to *Ipomoea cerradoensis* but is easily distinguished by its pink corollas, woody stems, much larger leaves and, sometimes, compounded inflorescence. It also resembles *Ipomoea paludosa* in the simple leaves and form of the sepals but is distinguished by the clearly woody stems, large, ovate leaves and, especially, by the lateral, not terminal inflorescence. It was originally named I. malvaeoides var. ovata by Chodat and Hassler (1905: 690).

### 35. Ipomoea campestris

Taxon classificationPlantaeSolanalesConvolvulaceae

Meisn. in Martius et al., Fl. Brasil. 7: 254. 1869. (Meisner 1869: 254)

Ipomoea virgata var. angustata Meisn. in Martius et al., Fl. Brasil. 7: 241. 1869. (Meisner 1869: 241). Type. BRAZIL. [Minas Gerais], Serra do Cristaës, J.B. Pohls.n. (?B†, n.v.).
Ipomoea stenophylla Meisn. in Martius et al., Fl. Brasil. 7: 240. 1869. (Meisner 1869: 240). Type. BRAZIL. Minas Gerais, Curvello, L. Riedel 2758 (lectotype NY00319227, designated here; isolectotype LE).
?Ipomoea stenophylla var. laciniata Meisn. in Martius et al., Fl. Brasil. 7: 249. 1869. (Meisner 1869: 249). Type. BRAZIL. São Paulo, Rio Pardo, L. Riedel [805] (lectotype LE01025981, designated here)

#### Type.

BRAZIL. Minas Gerais, Lagoa Santa, *E. Warming s.n.* (lectotype BR0000005307203, designated here; isolectotypes P, NY).

#### Description.

Erect or decumbent subshrub with woody xylopodium, stems somewhat woody, pubescent to pilose, eventually glabrescent. Leaves subsessile, ±imbricate, 4–10 × 0.1–1 cm, linear or oblong, acute, mucronate, base broadly cuneate, adaxially thinly pubescent to glabrous, abaxially thinly pubescent, veins somewhat prominent; petioles 1–3 mm, pubescent. Inflorescence of shortly pedunculate cymes from the upper leaf axils, these often reduced to single flowers; peduncles 2–15 mm, pubescent; bracteoles 1–2 mm, triangular, acute, caducous; pedicels 3–9 mm, thickened upwards; sepals subequal, 8–11 × 3–4 mm, oblong-ovate, finely acute, thinly pubescent, inner with scarious margins, pubescent along midrib only, strongly mucronate; corolla 3.5–6 cm long, pink, pubescent, funnel-shaped, limb c. 2 cm diam. Capsules and seeds not seen.

#### Distribution.

A cerrado species of central Brazil, apparently rare and with few modern collections.

**BRAZIL. Dist. Fed.**: Rio Belchior, *G. Pereira-Silva et al.* 7291 (CEN). **Goiás**: Cocalzinho de Goiás, *H.S. Irwin* 18770 (NY). **Minas Gerais**: *G. Hatschbach* 27787 (MBM, RB), *P. Clausen* 290 (P); *A. Saint-Hilaire* B1/1949 (K, P), C1-1060 (P); Lagoa Santa, Palacios et al. 3224 (LIL); Serra de Cipó, *H.S. Irwin* 20554 (NY); ibid., *A. Duarte* 2170 (RB); ibid., *L.S. Kinoshita & J.C. Galvão* 220 (UEC); São Roque da Minas, *R. Romero* 4956 (HUFU).

#### Typification.

In designating a lectotype of *Ipomoea stenophylla*, we have chosen the NY specimen as it appears to have a label in Meisner’s handwriting annotated as “Ipomoea stenophylla nob. (29./12./67.)”

#### Notes.

This species is distinguished by its linear to oblong, acuminate, mucronate leaves and distinctly acute sepals. The type of *Ipomoea stenophylla* represents a form with very narrow, linear leaves.

The type of Ipomoea stenophylla var. laciniata is very similar to *Hassler* 5023a (NY, P) from Río Tapiraguay (Canindeyú, Paraguay), which was also treated as this variety by Chodat and Hassler (1905: 690), even though plants with 3-lobed leaves were mixed with plants with simple leaves. The specimens have something of the appearance of *Ipomoea granulosa* because of their short, erect, slightly granular stems and subsessile flowers, but differ in the 3-lobed leaves and pubescent corolla. They are included here with doubt. Unfortunately, we have seen no modern collections, which could help elucidate the status of this variety.

### 36. Ipomoea ensiformis

Taxon classificationPlantaeSolanalesConvolvulaceae

J.R.I. Wood & Scotland, Phytokeys 88: 16. 2017. (Wood et al. 2017d: 16)

#### Type.

BRAZIL. Goiás, 5 km Alto Paraíso, Chapada dos Veadeiros, 1450 m, *Gates & Estabrook* 4 (holotype UB62303, isotypes MICH, RB).

#### Description.

Procumbent perennial herb, stems thinly pubescent, to 50 cm; rootstock a knotted woody xylopodium. Leaves shortly petiolate, 2–6 × 0.3–1.2 cm, oblong to oblong-lanceolate, base rounded, apex subacute to obtuse, very shortly mucronate, margin entire to undulate, glabrescent, the very young leaves pubescent; petioles 1–4 mm, puberulent. Inflorescence of solitary (rarely paired), axillary flowers borne on slender peduncles; peduncles 1.4–3.2 cm, slender, puberulent; bracteoles 3 × 1 cm, ovate, acuminate, relatively persistent; pedicels 5–6 mm, thinly puberulent; sepals subequal, outer 6–7 × 2.5–3 mm, oblong-ovate, obtuse, glabrous, inner similar but narrowly oblong-ovate, 7–8 mm long, abaxial surface sparsely pubescent centrally; corolla 3–4 cm long, pink, very sparsely pubescent on midpetaline bands, funnel-shaped, limb 3.5 cm diam. Capsules and seeds not seen.

#### Illustration.

Figure 29.

**Figure 29.**
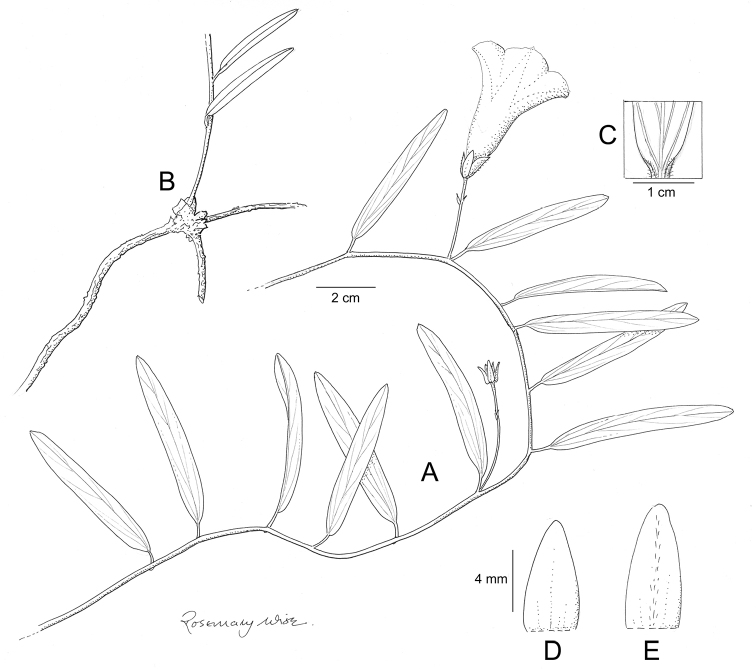
*Ipomoea ensiformis*. **A** habit **B** rootstock **C** base of young leaves showing indumentum **D** outer sepal **E** inner sepal. Drawn by Rosemary Wise from *Gates & Estabrook* 4.

#### Distribution.

Endemic to Goiás State in central Brazil. It is one of several *Ipomoea* species, which are apparently restricted to the Chapada dos Veadeiros and, like *Ipomoea graminifolia*, was found at the exceptionally high altitude of 1450 m.

BRAZIL. Goías: only known from the type collection.

Notes. Similar to *Ipomoea campestris* Meisn. but prostrate, glabrescent (pubescent only on young parts), the leaves petiolate (not subsessile), with an obtuse apex (not strongly acute). The sepals are < 8 mm long, the outer glabrous (not 8–11 mm long, pubescent). The corolla is smaller (3–4 cm long) and relatively widely funnel-shaped.

This has the appearance of a nearly glabrous prostrate form of Ipomoea campestris. Ipomoea campestris is quite variable in leaf shape but is always readily distinguished by the longer, narrower corolla, which reaches 6 cm, and the conspicuous pubescent indumentum of the inflorescence and corolla.

### 37. Ipomoea attenuata

Taxon classificationPlantaeSolanalesConvolvulaceae

J.R.I. Wood & Scotland, Phytokeys 88: 5. 2017. (Wood et al. 2017d: 5)

#### Type.

BRAZIL. Distrito Federal, Loc. Gama, BR 60, ca. 8.2 km do Tevo, DF-180 SO, disturbed campo sujo, dispersed locally, 15.5756S, 48.1059W, 1030 m, 26 Feb. 2015, *M. Mendoza, J.B.A. Brugel, A.A. Santos, T. Reis & T.K.M. Arquelão* 4802 (holotype UB, isotypes CEN, K).

#### Description.

Perennial herb; rootstock a woody xylopodium; stems up to 80 cm long, 2 mm diam., decumbent, weakly ascending or, fide field notes, climbing, pubescent with relatively long, often twisted spreading and appressed hairs. Leaves shortly petiolate, 4–10 × 0.3–0.7 cm, narrowly oblong, entire, apex acute and shortly mucronate, base cuneate, both surfaces thinly pubescent but more densely abaxially; petioles 3–7 mm long, pubescent. Inflorescence of lax, compounded axillary cymes from the middle and upper leaf axils; cymes up to 15 cm long, rather narrow, diminishing in size upwards, irregularly racemose in form; peduncle 2–7 cm long, often extending into a rhachis, pubescent; primary bracteoles foliose, 9–12 × 1–3 mm, linear, acuminate, persistent; secondary peduncles 0.5–2 cm long, thinly pubescent; ultimate bracteoles 4–7 × 0.5–1 mm, linear lanceolate, finely acuminate, persistent; pedicels very short, 3–5 mm long, a few scattered hairs present; calyx ovate in outline; sepals subequal, 11–14 × 4–5 mm, ovate with distinct truncate base and long-attenuated acuminate apex, glabrous, the inner very slightly longer than outer sepals; corolla 4–5 cm long, funnel-shaped, pink or reddish-purple, pubescent on the midpetaline bands, limb c. 2.5–3 cm diam. Capsules 13–15 × 8 mm, ovoid, glabrous; seeds 7 × 3.5 mm, ellipsoid, blackish-brown, glabrous except for pubescence along the angles.

#### Illustration.

Figure 30.

**Figure 30.**
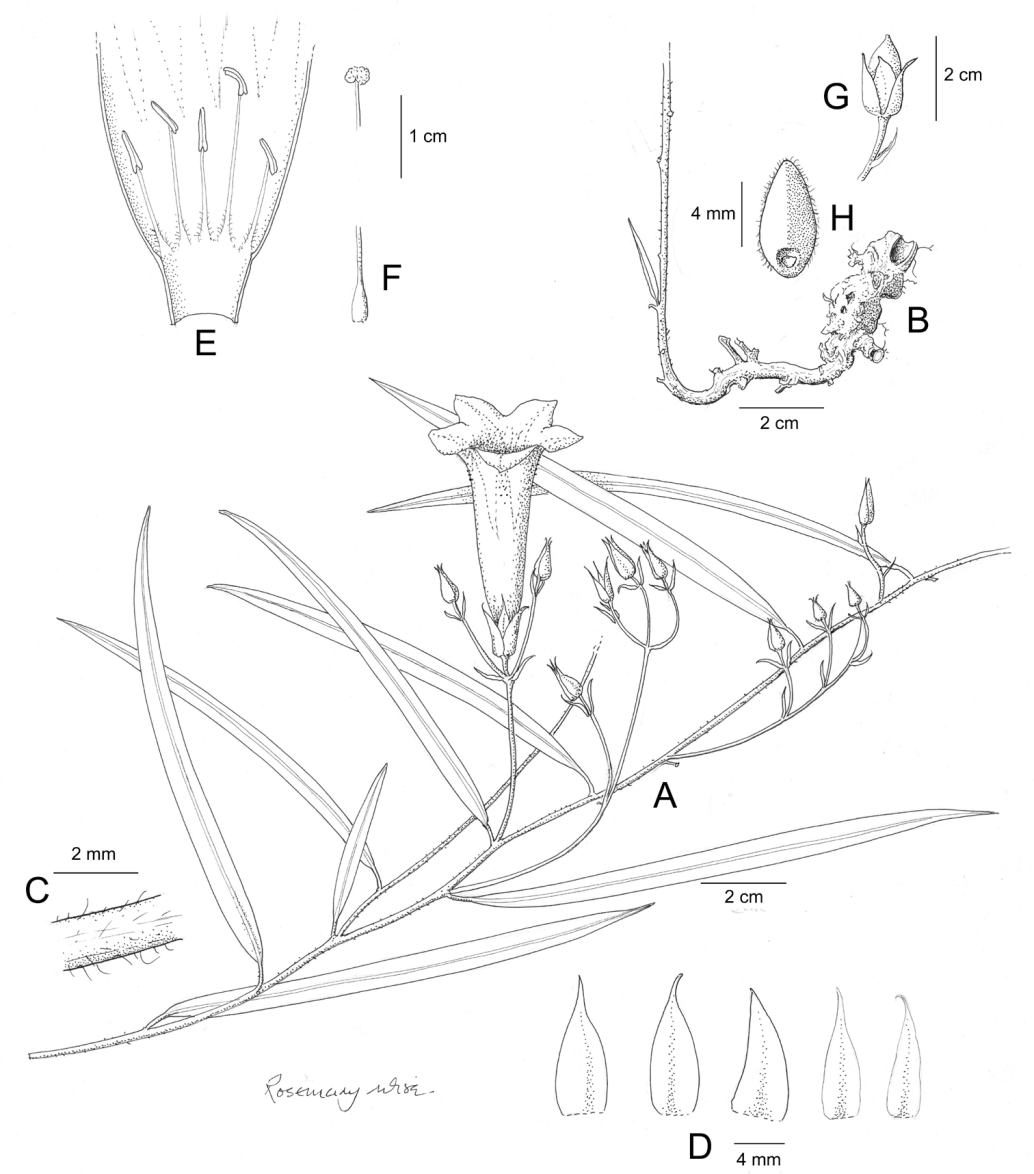
*Ipomoea attenuata*. **A** habit **B** xylopodium **C** section of stem showing indumentum **D** sepals outermost (left) to innermost (right) **E** corolla opened out to show stamens **F** ovary and style **G** capsule **H** seed. Drawn by Rosemary Wise **A–E** from *H.S. Irwin et al.* 11019; **F–H** from *M. Mendoza et al.* 4802.

#### Distribution.

Endemic to the Distrito Federal and Goiás State in Brazil, where it appears to be a rare species of cerrado.

**BRAZIL. Dist. Fed.**: type collection. **Goiás**: Samambaia, Rio Corumbá, *E.P. Heringer* 11283 (NY); Mun. Luziânia, Santo Antonio do Descoberto, *R.C. Mendonça* 93 (IBGE, NY); Serra dos Pireneus, c. 20 km S of Corumbá de Goiás, *H.S. Irwin et al.* 11019 (NY).

#### Note.

The attenuate sepal tips raise doubts about this tentative placement as this shape is atypical of species in this clade. *Ipomoea attenuata* has generally been treated in herbaria as *Ipomoea campestris* Meisn. because of the similar leaves and the pubescent exterior of the corolla, but is readily distinguished by the distinctive ovate sepals with truncate base and long attenuate apex. Additionally the inflorescence is of elongate complex cymes, somewhat racemose in form and with distinctive persistent linear-lanceolate bracteoles. The form of the inflorescence (axillary cymes) combined with the oblong leaf shape strongly suggests this is essentially a decumbent species even though this is not indicated in field notes.

### 38. Ipomoea argyreia

Taxon classificationPlantaeSolanalesConvolvulaceae

(Choisy) Meisn. in Martius et al., Fl. Brasil. 7: 246. 1869. (Meisner 1869: 246)

Rivea argyreia Choisy in A.P. de Candolle, Prodr. 9: 327. 1845. (Choisy 1845: 327). Type. BRAZIL. J.B. Pohls.n. (lectotype BR00005792573, designated here; isolectotypes BR, K, M).
Ipomoea argyreia var. burchellii Hassl., Repert. Spec. Nov. Regni Veg. 9: 196. 1911. (Hassler 1911: 196). Type. BRAZIL. *W.J. Burchell* 6700-9 (lectotype BR00005792214, designated here).

#### Type.

Based on *Rivea argyreia* Choisy

#### Description.

Erect subshrub to 1.5 m of grey appearance, stem woody, white-villous above, pubescent below. Leaves sessile, numerous, imbricate, sometimes appearing opposite or verticillate, 2.5–6 × 0.5–2 cm, oblong to oblanceolate, base cuneate, apex acute and mucronate, adaxially minutely tomentellous, abaxially shortly silvery-grey tomentellous. Flowers aggregated above into a terminal racemose inflorescence simple or branched, 5–15 cm long, flowers solitary or in few-flowered pedunculate cymes, peduncles 0.5–2 cm long, tomentellous; bracteoles 4–6 mm, ovate, acute, deciduous; pedicels 3–6 mm, grey-tomentellous; sepals nearly equal, 7–8 × 3–6 mm, ovate to elliptic, grey-tomentellous, inner sepals similar but with broad scarious margins; corolla 3–3.5 cm long, funnel-shaped, pubescent, limb lobed, c. 2 cm diam. Capsules ovoid, 5–9 mm long, glabrous, shortly rostrate; seeds c. 5 × 2.5 mm, black with long silky marginal hairs.

#### Illustration.

Figure 6A.

#### Distribution.

Almost endemic to the Distrito Federal and Goiás State in Brazil. It appears to grow always in campo rupestre from around 800 m to over 1100 m. It is recorded from Mato Grosso in Flora do Brasil 2020 under construction but we have seen no specimen.

**BRAZIL. Dist. Fed.**: 4 km W of Rio Preto *G. Kirkbride* 7383a (FTG). **Goiás**: 7–20 km E of Pireopolis. Serra de Pireneus, *M.M. Arbo et al*. 3793 (CTES, FTG); 35 km N of Formosa on road to São Gabriel, *H.S. Irwin et al.* 14198 (NY, FTG); Chapada dos Veadeiros, *H.S. Irwin et al.* 24670 (NY, FTG); 13 km S of São Joao de Alianca, *W.R. Anderson* 7581 (NY, FTG); 13 km E of Cristalina, *W.R. Anderson* 8310 (NY, FTG); Serra Dourada, 20 km S E, of Goiás Velho, *H.S. Irwin et al.* 11778 (FTG, NY, MO); Serra dos Cristais, 10 km W of Cristalina, 4 March 1966, *H.S. Irwin et al.* 13464 (FTG, NY); Serra dos Pirineus (Mun. Pienopolis), *P.I.Oliveira & W.R. Anderson* 465 (MBM, FTG); Mun. Planaltina, 16 km N de São Gabriel, *G. Hatschbach & Silva* 59993 (CTES, MBM, S). **Minas Gerais**: Cabeceira Grande, *A.A.Santos & J.B. Pereira* 1814 (CEN).

### 39. Ipomoea cuneifolia

Taxon classificationPlantaeSolanalesConvolvulaceae

Meisn. in Martius et al., Fl. Brasil. 7: 245. 1869. (Meisner 1869: 245)

Ipomoea cuneifolia var. acutifolia Meisn. in Martius et al., Fl. Brasil. 7: 245. 1869. (Meisner 1869: 245). Type. BRAZIL. Minas Gerais, Lagoa Santa, *E. Warming* [1757] (holotype BR00005307227, isotype C, n.v.).

#### Type.

BRAZIL. Goiás, 17/1/1829, *W.J. Burchell* 8501-2 (holotype BR0000006972578, isotype K).

#### Description.

Erect undershrub to 1.5 m, stem woody, hispid-pilose with multicelular hairs, roots tuberous. Leaves subsessile, 3–6 × 1.2–2 cm oblong-oblanceolate, apex rounded and mucronate, base cuneate and slightly asymmetric, adaxially densely grey-pubescent, abaxially hispid-hirsute and gland-dotted; petioles 0–5 mm. Inflorescence terminal, simple, short to somewhat elongate, formed of shortly pedunculate cymes from the uppermost leaf axils; peduncles 0.5–1 cm, diminishing in size upwards; bracteoles up to 6 × 2 mm, linear-lanceolate, caducous; pedicels 3–5 mm so cymes congested; sepals 5–7 mm, ovate-elliptic, obtuse, grey-tomentellous, similar, slightly accrescent in fruit; corolla c. 4 cm long, funnel-shaped, pink, appressed pilose, limb c. 3 cm diam., shallowly lobed. Capsules c. 10 × 6 mm, narrowly ovoid, glabrous; seeds woolly.

#### Distribution.

Scattered through the cerrados of central Brazil (most common in Mato Grosso), extending west to a single location in eastern Bolivia.

**BRAZIL. Goiás**: Aragarças, *D. Philcox & Ferreira* 4030 (K); Novo Alegre-Taguatinga, *J.R.Pirani et al.* 1909 (K, SPF); *G. Gardner* 3904 (K); Natividade, *G. Gardner* 3353 (K); *H.S. Irwin et al.* 32032 (NY). **Mato Grosso**: *C.A.M. Lindman* 3313 (S); *G. Hatschbach* 34008 (MBM); *J. Ratter et al*. 4129 (E); Novo Mundo, P. Est. Cristalino, *D. Sasaki et al.* 1907 (K); Santa Cruz do Xingu, *J.H. Piva & V. Marine* 56 (K, RB); Xavantina, *D. Philcox et al.* 3170 (K, MO, P), 3604 (K, MO, NY, P), 4367 (K, MO, P, S); ibid., *R.M. Harley & Souza* 11048 (K). **Mato Grosso do Sul**: *Pott & Pott* 6701 (CPAP); Coxim, *G. Hatschbach* 34008 (MBM, MO). **Tocantins**: Reserva Indigena Krahó, *A. Amaral-Santos* 722 (CEN); Paraná, *G. Pereira-Silva* 11546 (CEN). In Flora do Brasil 2020 under construction *Ipomoea cuneifolia* is recorded from Pará and Maranhão, but we have seen no specimens and these records require confirmation.

**BOLIVIA.** Santa Cruz: P.N. Noel Kempff Mercado, *E. Gutiérrez* 1144 (ARIZ, USZ).

#### Note.

Close to *Ipomoea haenkeana* but leaves < 2 cm wide, densely pubescent adaxially and inflorescence simple, side branches absent or very short so raceme-like in form.

### 40. Ipomoea haenkeana

Taxon classificationPlantaeSolanalesConvolvulaceae

Choisy in A.P. de Candolle, Prodr. 9: 358. 1845. (Choisy 1845: 358)

#### Type.

BOLIVIA. “Cochabamba”, *T. Haenke* (lectotype BR006973261, designated here; isolectotype BR).

#### Description.

Erect perennial to 2 m, branched towards the apex, stems woody, tomentellous. Leaves subsessile, mostly 3–6 × 2–4 cm, oblong-obovate, apex rounded and apiculate, base rounded to truncate, slightly asymmetric, adaxially dark green and thinly pilose to subglabrous, abaxially grey-tomentose; petioles 0–4 mm. Inflorescence of shortly pedunculate cymes from the uppermost leaf axils forming a terminal panicle of raceme-like branches ; peduncles 1–3 cm; bracteoles 9–12 × 1–2 mm, lanceolate, acute, ± persistent; pedicels 2–5 mm (so cymes very dense); sepals subequal, 7–9 × 3–4 mm, oblong-ovate, acuminate to shortly apiculate, grey-sericeous; corolla 3.5–4 cm long, funnel-shaped, pale pink with a darker centre, pubescent outside, the limb 2.5–3.5 cm diam. Capsules and seeds not known.

#### Illustration.

Figure 17A; Wood et al. (2015: 40, photo).

#### Distribution.

Locally common in cerrados in Santa Cruz Department in Bolivia and adjacent areas of Mato Grosso extending sporadically eastwards to Minas Gerais.

**BRAZIL. Mato Grosso**: Cuaibá, *L. Riedel* (NY); Serra de Roncador, *H.S. Irwin et al.* 16026 (NY); Córrego da Palha, *D.L. Amaral* 175 (LE, RB); Parque Estadual Cristalina, *D. Sasaki* 11907 (RB). **Mato Grosso do Sul**: *G. Hatschbach* 58891 (CTES, MBM, SP). **Minas Gerais**: Lagoa Santa, *E. Warming* (NY, P); Ituiutaba, *A. Macedo* 665 (BM, NY). **São Paulo**: Fazenda Campininha, *O. Handro* 448 (UEC).

**BOLIVIA. Santa Cruz**: Chiquitos, Santiago de Chiquitos, *J.R.I. Wood* 17972 (K, LPB, USZ); Florida, Laguna Volcánes near Bermejo, *A. Fuentes* 348 (LPB, NY, USZ); Guarayos, Ascensión de Guarayos, *A. Krapovickas & A. Schinini* 31838 (CTES, FTG); Ichilo, Buenavista, *J. Steinbach* 5583 (GH, LPB, NY, F); Ñuflo de Chávez, 40 km S. of Concepción, *T.J. Killeen* 2345 (LPB, NY, F, MO, USZ). Sara, N.of La Bélgica, *M. Nee & M. Sundue* 52213, (LPB, NY, USZ); Ángel Sandoval, San Matías, *A. Krapovickas & A. Schinini* 36185 (G, LIL); Velasco, San Ignacio hacia El Recreo, *J.R.I. Wood et al.* 24788 (K, LPB, UB, USZ).

#### Notes.

*Ipomoea haenkeana* is most likely to be confused with *I. cuneifolia* which has narrower leaves and a much shorter, more compact, unbranched terminal inflorescence.

The cited type locality of “Cochabamba” must be wrong as this is a plant of lowland cerrado vegetation, not inter-Andean dry valleys.

### 41. Ipomoea virgata

Taxon classificationPlantaeSolanalesConvolvulaceae

Meisn. in Martius et al., Fl. Brasil. 7: 241. 1869. (Meisner 1869: 241)

Ipomoea virgata var. paniculata Meisn. in Martius et al., Fl. Brasil. 7: 241. 1869. (Meisner 1869: 241), nom. illeg, autonymic var.

#### Type.

BRAZIL. Minas Gerais, *A.F. Regnell* Ser. 3, 192 (lectotype BR0000005305797, designated by Wood et al. (2015: 43), isolectotypes K, S).

#### Description.

Ascending or erect undershrub from a woody xylopodium, stems woody, somewhat lanate. Leaves sessile, 3–7 × 2.5–5 cm, broadly ovate to narrowly elliptic, obtuse and apiculate, broadly cuneate at base, adaxially pubescent, abaxially whitish-floccose. Inflorescence of lax axillary cymes, forming an elongate terminal raceme, often somewhat compound below with branches to 7 cm in length, so appearing paniculate; peduncles 1–4.5 cm, villous; bracteoles lanceolate, acuminate, caducous; pedicels 5–8 mm; sepals subequal, 8–12 mm, ovate, acute, grey-tomentose; corolla 3–6 cm long, subcampanulate to broadly funnel-shaped, white(?), densely pilose with appressed hairs, limb 2.5–3.5 cm diam. Capsules (immature) 6–7 × 3–4 mm, narrowly ovoid, glabrous.

#### Distribution.

Apparently uncommon in both the cerrados of Brazil and Bolivia. In Bolivia only known from a single collection and in Brazil from scattered collections, mostly old, from three states.

**BRAZIL. Mato Grosso**: Santa Ana da Chapada, *Robert* 674 (BM), 701 (BM), 715 (BM). **Minas Gerais**: *St Hilaire* 354 (P); *A.F.M. Glaziou* 2179 (P); *A. Macedo* 1329 (S); Uberlandia, *A. A. Barbosa* 31776 (HUFU). **São Paulo**: *Gaudichaud* 316 (P); *C.W. Mosén* 1498 (P, S); *A.F. Regnell* 192 (S), 4289 (P).

**BOLIVIA. Santa Cruz**: Velasco, P.N. Noel Kempff Mercado, *S. Jiménez & E. Gutiérrez* 1274 (USZ).

#### Note.

A little known species with a paniculate inflorescence distinguished from *Ipomoea haenkeana* by its more woolly indumentum, ovate leaves and longer sepals.

### 42. Ipomoea verbasciformis

Taxon classificationPlantaeSolanalesConvolvulaceae

(Meisn.) O’Donell, Lilloa 23: 502. 1950. (O’Donell 1950b: 502

Ipomoea virgata var. verbasciformis Meisn. in Martius et al., Fl. Brasil. 7: 241. 1869. (Meisner 1869: 241). Type. BRAZIL. Minas Gerais, Caldas, A.F. Regnell Ser.1, 305 (lectotype BR0000530742, designated here; isolectotypes BR, R, RB, S, US).

#### Type.

Based on Ipomoea virgata Meisn. var. verbasciformis Meisn.

#### Description.

Erect undershrub to 1.5 m, the whole plant tomentose. Leaves shortly petiolate, 3–5.5 × 1–2.5 cm, diminishing in size upwards, ovate-elliptic, obtuse, mucronulate, broadly cuneate to subtruncate at base, paler abaxially, tomentose on both surfaces; petioles 2–5 mm, tomentose. Inflorescence terminal, elongate, formed of dense, few-flowered pedunculate cymes from the middle leaf axils, often with solitary flowers from the upper axils; peduncles 1–6 cm, tomentose; bracteoles 5–12 mm, ovate, acute, persistent; pedicels 0–5 mm, densely tomentose; sepals subequal, 10–12 × 5 mm, ovate-elliptic, acute,submucronate, lanate, inner with paler hyaline margins; corolla 5–7 cm long, pink, funnel-shaped, pilose; limb c. 3 cm diam. Capsules and seeds not seen.

#### Distribution.

Possibly endemic to Minas Gerais in Brazil, gowing in cerrado.

**BRAZIL. Minas Gerais**: *C.W.H. Mosén* 4288 (S); *J.F. Widgren* 226 (S); Santa Rosália, Caldas, *L.S.K. Gouvea et al*. 776 (IPA); São José de Barreiro, entrando por Babilônia, *R. Simão-Bianchini & S. Bianchini* 1203 (NY, SP); São Roque de Minas, *J.N. Nakajima* 1731 (HUFU).

#### Typification.

Meisner cited three syntypes, *Regnell* Ser.1, 305, *Widgren* 304 and *Widgren* 226. The Regnell specimen from Martius’ herbarium at Brussels is here selected as lectotype. It must be presumed to have been seen by Meisner and is excellent material, duplicated at R and US.

#### Note.

This species is distinct because of the erect habit and the persistent ovate bracts, which almost clasp the calyx as the pedicels are very short.

### 43. Ipomoea dasycarpa

Taxon classificationPlantaeSolanalesConvolvulaceae

J.R.I. Wood & Scotland, Phytokeys 88: 12. 2017. (Wood et al. 2017d: 12)

#### Type.

BRAZIL. Goiás, P.N. Chapada dos Veadeiros, ca. 1100 m, perto da sede do parque, *J.R. Pirani, R.M. Harley, B.L. Stannard, A. Furlan & C. Kameyama* 1715 (holotype SPF00049438, isotype K).

#### Description.

Erect perennial subshrub to 1 m, rootstock unknown, presumably a xylopodium, stem densely tomentose with white hairs. Leaves very shortly petiolate, 2.5–11 × 1–3.5 cm, oblong to narrowly-oblong-elliptic, margin entire, base cuneate, apex acute, mucronate, the mucro often bent, adaxially green, tomentose, abaxially whitish, tomentose, veins prominent; petioles 2–5 mm, tomentose. Inflorescence terminal formed of shortly pedunculate, 3-flowered cymes arising in the axils of the reduced uppermost leaves; peduncles 1–5.5 cm, grey-tomentose; lower bracteoles 15–20 × 4–7 mm, foliose, elliptic, acuminate to a fine point and ±mucronate, tomentose, persistent; upper bracteoles similar, but slightly smaller; pedicels 0–11 mm, tomentose; sepals subequal, outer 15–18 × 6–8 mm, ovate, acuminate, submucronate, tomentose, inner 14–15 × 5–7 mm, tomentose with broad glabrous margins; corolla 4.5–5 cm long, funnel-shaped, pink, tomentose in bud, limb c. 4 cm diam., entire. Capsules 9 × 5 mm, ovoid, muticous, comose with shaggy, somewhat deciduous hairs; seeds 6 × 3 mm, glabrous apart from the fine white marginal hairs c. 5–6 mm long.

#### Illustration.

Figure 31.

**Figure 31.**
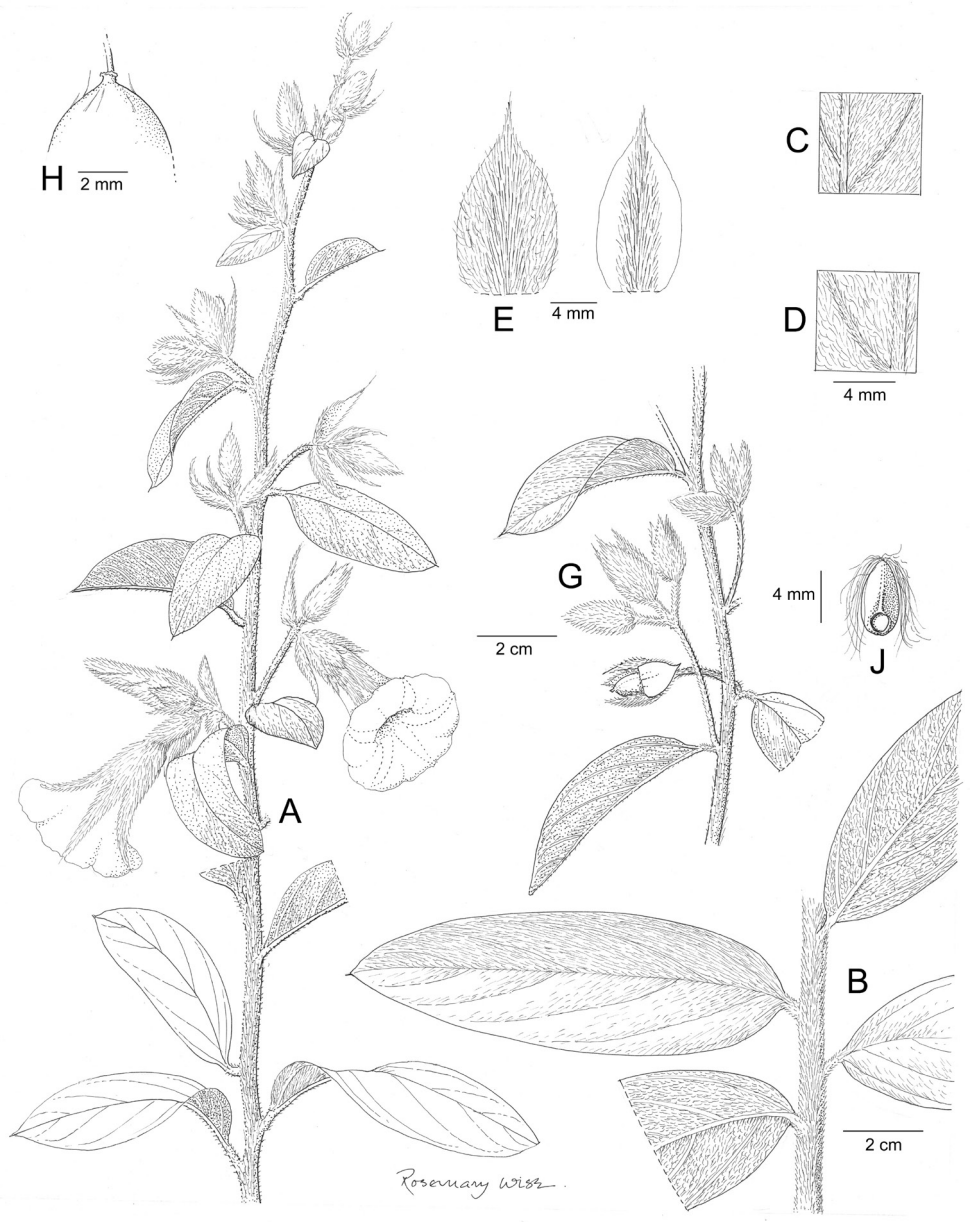
*Ipomoea dasycarpa*. **A** habit **B** leaves and stem **C** adaxial leaf surface **D** abaxial leaf surface **E** outer sepal **F** inner sepal **G** fruiting inflorescence with fallen bracteoles **H** apex of capsule **J** seed. Drawn by Rosemary Wise **A–D** from *J.R. Pirani et al.* 1715; **E, F, H** J from *H.S. Irwin et al.* 32875; **G** from *H.S. Irwin et al.* 24946.

#### Distribution.

Endemic to relatively high altitudes between 1000 and 1250 m in the Chapada dos Veadeiros in Goiás, Brazil, apparently growing in rocky cerrado.

**BRAZIL. Goiás**: Chapada dos Veadeiros, c. 20 km W of Veadeiros, *H.S. Irwin et al*. 12407 (FTG114226); 10 km S of Alto do Paraíso, *H.S. Irwin et al*. 24946a (FTG114228); 18 km N of Alto do Paraíso, *H.S. Irwin et al*. 32875 (FTG114227); perto da sede do Parque, *J.R. Pirani et al.* 1715 (K, SPF).

#### Note.

*Ipomoea dasycarpa* appears close to *Ipomoea verbasciformis* but is distinguished by the larger dimensions of the leaves, bracteoles and sepals, by the strongly mucronate leaves, acuminate, submucronate (not obtuse) sepals and the comose (not glabrous) ovary. Hirsute capsules are rare in *Ipomoea* and found outside the Batatas Clade in only a few species such as the unrelated *I. sidifolia* and *I. velutinifolia*.

### 44. Ipomoea geophilifolia

Taxon classificationPlantaeSolanalesConvolvulaceae

K. Afzelius, Svensk Bot. Tidskr. 60: 484. 1966. (Afzelius 1966: 484)

#### Type.

BRAZIL. Distrito Federal, Cabeça do Veado, March 1961, *E.P. Heringer 8029* (whereabouts unknown, possibly number cited erroneously, neotype *E.P. Heringer 8030* (UB), designated here).

#### Description.

Relatively slender twining or trailing herb, stems pubescent. Leaves petiolate, 2 –5.5 × 2–5 cm, ovate to suborbicular, mucronate, cordate with narrow basal sinus, pubescent to subtomentose on both surfaces, paler beneath; petioles 1.5–3.5 cm, pubescent. Flowers in 1–3-flowered axillary cymes; peduncles 2–3 cm; bracteoles linear, 6–10 mm, pilose, persistent; pedicels c. 5 mm, densely pubescent; sepals subequal, 12–16 × 4–6 mm, densely pubescent, lanceolate, acuminate, inner paler and less hairy on paler margins; corolla 5–6.5 cm long, pilose, pink, funnel-shaped, the tube purple within, limb 4 cm diam., slightly lobed. Capsules and seeds not seen.

#### Distribution.

A local endemic species of cerrado in the Brazilian planalto at around 1100–1200 m near Brasilia.

**BRAZIL. Dist. Fed.**: *H. Irwin et al*. 12261 (FTG, NY, MO); Campo Experimental UB, *G. Kirkbride* 1444 (F, K); *E.P. Heringer* 15396 (FTG). **Goiás**: Mun. Cristalina, *G. Hatschbach & J. Cordeiro* 51799 (MBM); *H.S. Irwin et al.* 13773 (FTG).

#### Typification.

Afzelius cited *Heringer* 8029 as the type but as this is neither at S nor UB, it is possible the number was cited erroneously. *Heringer* 8030 was cited as a paratype and is at UB, so is here designated as neotype.

#### Note.

This species is distinguished by its relatively slender habit, suborbicular leaves, persistent bracteoles and lanceolate sepals 12–16 mm long.

### 45. Ipomoea hieronymi

Taxon classificationPlantaeSolanalesConvolvulaceae

(Kuntze) O’Donell, Lilloa 14: 171. 1948. (O’Donell 1948a: 171)

Mouroucoa hieronymi Kuntze, Revis. Gen. Pl. 3(3): 217. 1898. (Kuntze 1898: 217). Type. ARGENTINA. Salta, San José, *Lorentz & Hieronymus* 220 (holotype B†, isotypes GOET, CORD00003758).
Argyreia megapotamica Griseb., Symb. Fl. Argent. 263. 1879. (Grisebach 1879: 263), non Ipomoea megapotamicaChoisy (1845). Type. ARGENTINA. Córdoba, Ascochinga, *Lorentz*s.n. (possible lectotype (fide O’Donell 1948a: 179) CORD00006091).
Ipomoea kurtziana O’Donell, Lilloa 14: 179. 1948 (O’Donell 1948a: 179). Type. Based on Argyreia megapotamica Griseb.
Ipomoea hieronymi var. kurtziana (O’Donell) O’Donell, Lilloa 29: 163. 1959. (O’Donell 1959b: 163).
Ipomoea hieronymi var. calchaquina O’Donell, Lilloa 29: 165. 1959. (O’Donell 1959b: 165). Type. ARGENTINA. Catamarca, Dept. Andalgalá, camino de Capillitas a Santa María, *O’Donell & T. Meyer* 5198 (holotype LIL202799).

#### Type.

Based on *Mouroucoa hieronymi* Kuntze

#### Description.

Vigorous perennial, sometimes growing as a liana, stems pubescent to tomentellous. Leaves petiolate, 4–10(–15) × 4–10(–15) cm, ovate, cordate with rounded auricles, apex rounded and mucronate to acute or very shortly acuminate, adaxially dark green and densely puberulent, abaxially white-tomentose; petioles 2–11 cm, densely puberulent or tomentose. Inflorescence of long-pedunculate axillary cymes, usually 3–5-flowered; peduncles 3–20 cm, tomentose; bracteoles 5 mm long, ovate, caducous; secondary peduncles mostly 6–10 mm; pedicels 5–12 mm, tomentose; sepals subequal, 9–11 × 6–7 mm, ovate, grey-tomentose, often with a dark gland at base, acute to obtuse, inner slightly shorter with scarious margins; corolla 4.5–7 cm long, funnel-shaped, pink, tomentellous, limb c. 4 cm diam. Capsules ovoid, 8–10 mm long, glabrous; seeds 7–8 mm long, glabrous except sericeous angles.

#### Illustration.

**Figure 32.**
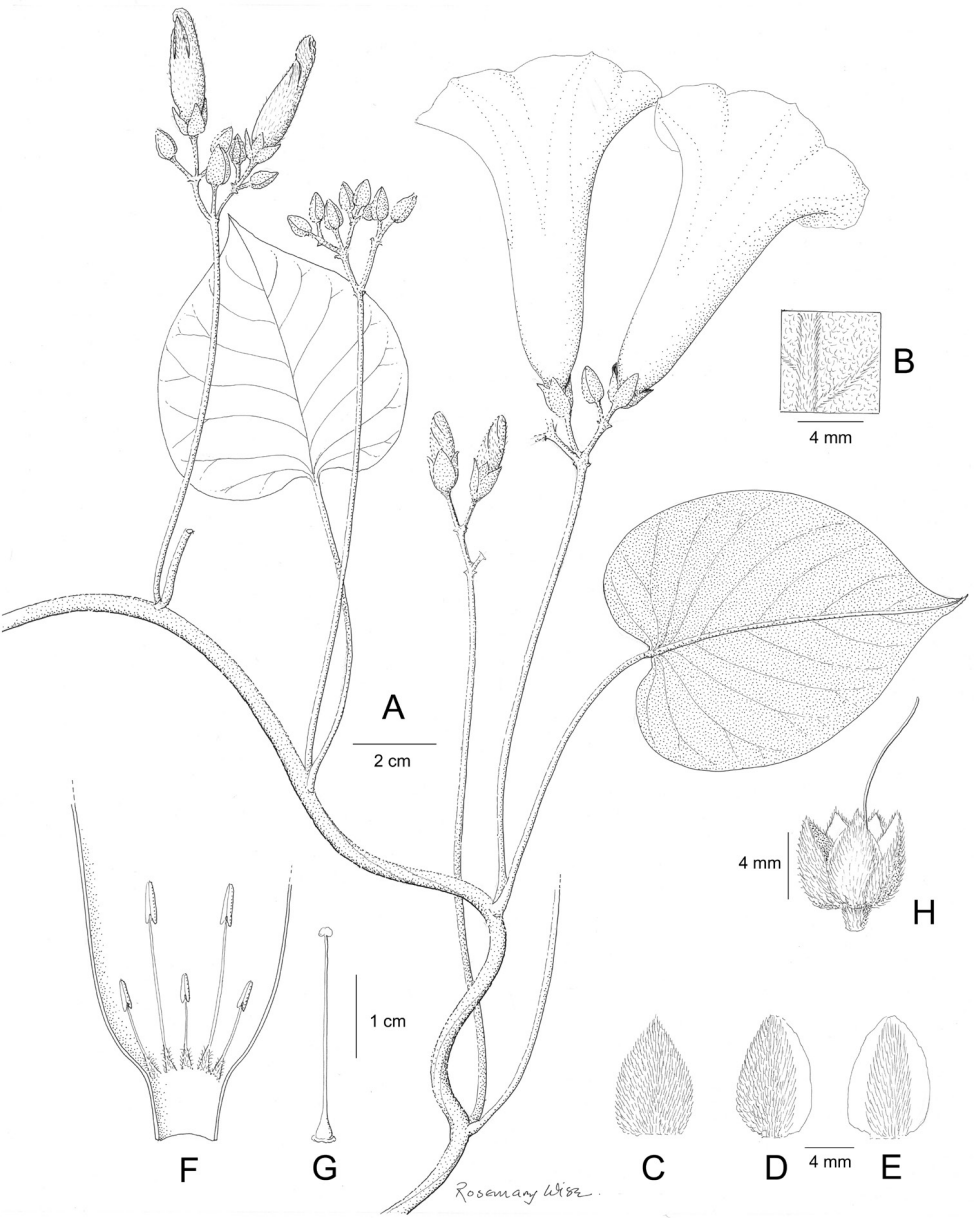
*Ipomoea hieronymi*. **A** habit **B** abaxial leaf surface **C** outer sepal **D** middle sepal **E** inner sepal **F** corolla opened out to show stamens **G** ovary and style **H** fruiting calyx. Drawn by Rosemary Wise **A, B** from *Brooks* MS206A; **C–G** from *Venturi* 5837 **H** from *Novara* 4206.

Figures 9A, 17D, 32.

#### Distribution.

Common in the Andean region of northwestern Argentina extending into the south of Bolivia. It is found from around 700 to 2000 m in scattered locations by roadsides and along forest margins.

**ARGENTINA. Catamarca**: Santa Rosa, Alijilán, *S. Pierotti* 28348 (BM); *E. Wall* s.n. [29/11/1946] (S). **Córdoba**: *Pierotti* s.n. [27/1/1944] (LIL, S); Yacanto Calamuchita, *Tirel* 60 (G, P); Punilla, *G. Seijo* 1902 (CTES). **Jujuy**: Tumbaya, Volcán, *J.G. Hawkes et al.* 3758 (C, MO); Capital, *A.L. Cabrera et al.* 29926 (MO, SI). La Rioja: General Belgrano, *F.N. Biurrun & E. Pagliari* 2659 (CORD). **Salta**: La Caldera, *L.J. Novara* 6043 (G). **San Luis**: Villa Carmen, *D.O. King* 549 (BM); Chacabuco, *R. Pozner & M.J. Belgrano* 206 (CTES, SI). **Tucumán**: Burragaco, Cerro del Campo, *S. Venturi* 10346 (BM, MO); Tafí, *T.M. Pedersen & J. Hjerting* 921 (MO).

**BOLIVIA. Chuquisaca**: Calvo, 31 km SW of Cuevo, *M. Mendoza et al.* 2739 (USZ). **Tarija**: Cercado, *K. Fiebrig* 2655 (BM, NY, P). Gran Chaco, Serranía San Alberto, *R. Chávez & R. Meneses* 2954 (LPB). O’Connor, Cuesta de San Simón, *A. Krapovickas & A. Schinini* 38036 (CTES, LPB).

#### Note.

This species resembles *Ipomoea argentinica* and similar species in having leaves abaxially white-tomentose. It is close to *I. megapotamica*, the sepals often with a dark gland near the base, but differs in the tomentose leaves and longer sepals, which are about 10 mm in length.

### 46. Ipomoea spinulifera

Taxon classificationPlantaeSolanalesConvolvulaceae

J.R.I. Wood & Scotland, Kew Bull. 50 (31): 47. 2015. (Wood et al. 2015: 47)

#### Type.

BOLIVIA. Tarija, Prov. O’Connor, on descent from Caneletas to Narvaéz, on road from Tarija to Entre Ríos, *J.R.I. Wood* 27923 (holotype LPB, isotypes K, LPB).

#### Description.

Very vigorous liana-like perennial to 5 m; stems relatively stout, thinly pilose with long white hairs, spinulose with short triangular spines on angles. Leaves petiolate, 9–11 × 8–10 cm, ovate, base cordate with rounded auricles, apex acute to shortly mucronate, margin entire, adaxially green, glabrous, abaxially paler, veins pilose and highlighted with whitish hairs, intercostal regions glabrous; petioles 5–9 cm, thinly pilose. Inflorescence of long-pedunculate, lax, compound cymes comprising 5–10 flowers; primary peduncles very long, 17–21 cm, thinly pilose and with a few scattered stalked glands and spinules; secondary peduncles 3–3.5 cm, pilose; tertiary peduncles 2–3 cm; bracteoles 1.5 × 0.5 mm, oblong, caducous; pedicels 12–23 mm, densely white-pilose, bearded below flower; outer sepals 10–11 × 7 mm, ovate, obtuse to retuse, dark green when fresh, pubescent at centre near base, glabrous upwards and at margins, the scarious margins thin; inner sepals 10–11 × 8 mm, broadly elliptic, glabrous except near base, scarious margins broad; corolla 7.5–9 cm long, gradually widened from base, pink, in bud pubescent, limb c. 5 cm diam., undulate to weakly lobed. Capsules and seeds not seen.

#### Illustration.

Figure 33.

**Figure 33.**
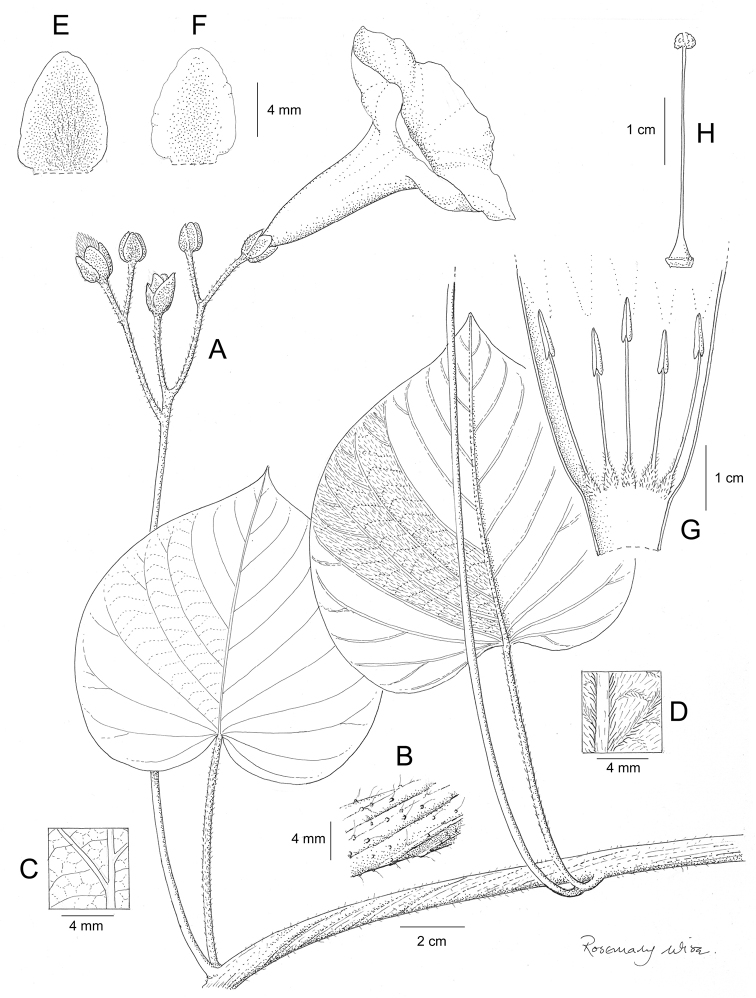
*Ipomoea spinulifera*. **A** habit **B** section of stem showing spinules **C** adaxial leaf surface **D** abaxial leaf surface **E** outer sepal **F** inner sepal **G** corolla opened out to show stamens **H** ovary and style. Drawn by Rosemary Wise **A, B** from *Villalobos et al.* 1307; **C–H** from *Wood* 27923.

#### Distribution.

Endemic to the Andes in Tarija Department, Bolivia, where it is locally common between 1600 and 2100 m in scrub and forest relics derived from former moist Tucuman-Bolivian forest.

**BOLIVIA. Tarija**: Arce, *M. Serrano et al.* 6038 (ARIZ, MO); O’Connor, *J. Villalobos et al*. 1307 (OXF, HSB, MO); *J.R.I. Wood et al.* 28047 (LPB, USZ).

#### Notes.

*Ipomoea spinulifera* appears to be related to *I. hieronymi* but is distinguished by the dark green, near glabrous sepals, very large corolla 7.5–9 cm long and spinulose stems.

*M.A. Negritto et al.* 502 (MA, CORD, n.v.) from an unspecified location in Prov. Arce in Tarija Department appears to be intermediate between this species and *Ipomoea jujuyensis*. It was identified by the collectors as *I. lilloana* to which it would key following O’Donell’s (1959b) key to Argentinian species of *Ipomoea* because of the pubescent midpetaline bands which are clearly visible on the buds but it is not *I. lilloana*, which is a trailing plant with distinct undulate leaves. The somewhat truncate calyx and large corolla suggests an affinity with *I. spinulifera* but the leaves are very different.

### 47. Ipomoea aprica

Taxon classificationPlantaeSolanalesConvolvulaceae

House, Ann. New York Acad. Sci. 18: 243. 1908. (House 1908b: 243)

Ipomoea angustifolia Choisy in A.P. de Candolle, Prodr. 9: 355. 1845. (Choisy 1845: 355), non Ipomoea angustifolia Jacq. (1791). Type. BRAZIL. J.B. Pohl in Herb. Mart. (holotype M0184918, isotypes BR, K).
Ipomoea angustifolia var. villosula Meisn. in Martius et al., Fl. Brasil. 7: 249. 1869. (Meisner 1869: 249). Type. BRAZIL. Minas Gerais, near Riego, L. Riedel 1368 (lectotype NY00039151, designated here).

#### Type.

Based on *Ipomoea angustifolia* Choisy

#### Description.

Erect undershrub from a xylopodium to c. 75 cm, stems strigose, woody, not usually branched. Leaves sessile, rather numerous, 1.5–12 × 0.2–0.5 cm, linear to narrowly oblong, base cuneate, apex acute and mucronate, adpressed pubescent. Inflorescence terminal, usually short (c. 5 cm long) with a distinct rhachis, somewhat compact; flowers solitary from the upper leaf axils or in very shortly pedunculate cymes; peduncles 0–5 cm, pubescent; bracteoles c. 2 mm, lanceolate, fugacious; pedicels 3–10 mm, pubescence more spreading than on peduncles; sepals subequal, 4–6 mm (accrescent to 7 mm in fruit), ovate to suborbicular, obtuse to subacute, densely pubescent, the inner c. 1 mm longer than outer, rounded with wide, glabrous, scarious margins; corolla 4–4.5 cm long, funnel-shaped, pink, adpressed pilose, limb 2.5–3 cm diam. Capsules ovoid, 5–7 mm long, glabrous, shortly rostrate; seeds not seen.

#### Illustration.

Figure 34.

**Figure 34.**
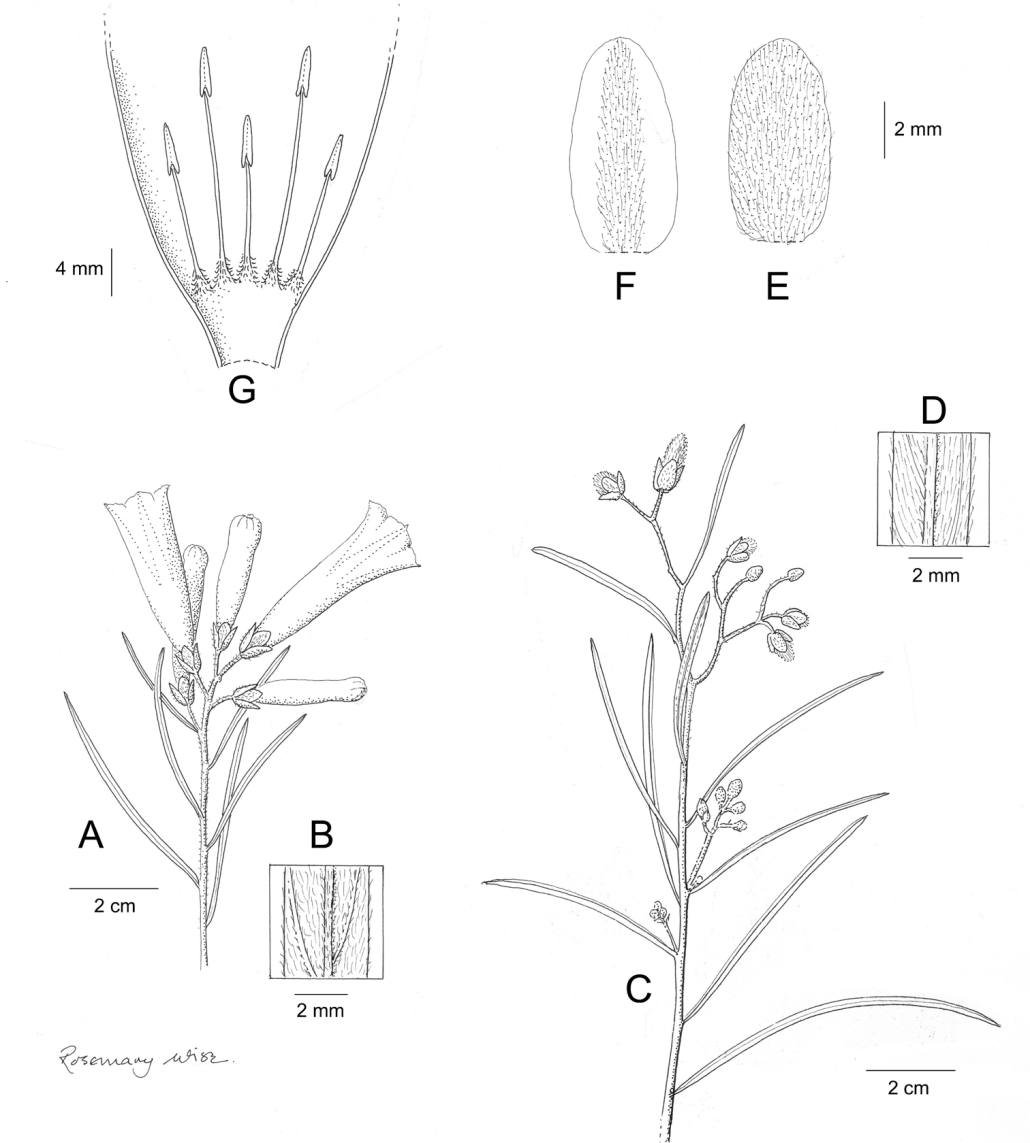
*Ipomoea aprica*. **A** habit **B** abaxial leaf surface **C** habit **D** abaxial leaf surface **E** outer sepal **F** inner sepal **G** corolla opened out to show stamens. Drawn by Rosemary Wise **A, B** from *Pohl* s.n; **C, D** from *Heringer et al.* 2982; **E–G** from *Menezes et al.* 4912.

#### Distribution.

A characteristic cerrado species, which is quite common in central Brazil but very localised in Paraguay and Bolivia.

**PARAGUAY. Amambay**: *E. Zardini & Baez* 52211 (ARIZ); *Hahn* 1707 (FTG, MO, PY), *L. Bernardi* 18972 (G); *M.S. Ferrucci et al.* 1445 (CTES, MBM); Cerro Corá, *N. Soria* 7386 (CTES, FCQ, MO); ibid., *F. Mereles* 3440 (FCQ); *R. Fortunato et al.* 922 (PY). **Canindeyú**: Reserva Natural, Bosque Mbaracayú, *A. Schinini & M. Dematteis* 33269 (CTES).

**BRAZIL. Dist. Fed.**: *A.F.M. Glaziou* 17710 (K); *E.P. Heringer et al.* 2982 (NY). **Goiás**: Caldas Novas, *A. Macedo* 3532 (NY, S); ibid., *N.L. Menezes 643* (SPF, K). Serra dos Cristais, *H.S. Irwin et al.* 13310 (NY). **Mato Grosso**: Mun. Rio Brilhante, *G. Hatschbach* 26117 (RB). **Mato Grosso do Sul**: Bela Vista, Faz. Novo Recanto, *A. Pott* 14025 (CGMS). **Minas Gerais**: *P. Clausen*, 1840 (BM, K); *B.M.T. Walter et al*. 5088 (CEN); *A.F. Regnell* Ser. 3, 196 (S); Serra do Ouro Branco, *A.M. Giulietti et al.* 13766 (K, USF); Serra da Anta, *H.S. Irwin et al.* 26036 (NY); Niquelândia, *H.S. Irwin et al.* 34880 (NY).

**BOLIVIA. Santa Cruz**: Velasco, P.N. Noel Kempff Mercado, *A. Soto et al.* 415 (FTG, MO, USZ).

#### Typification.

We have designated the NY specimen of *Riedel* 1368 as lectotype of Ipomoea angustifolia var. villosula as a suitable lectotype at LE could not be found.

#### Note.

This species might be confused with *Ipomoea schomburgkii* because of its linear-oblong leaves but both the corolla and sepals are hirsute. From *I. pinifolia*, it is distinguished by the subequal sepals and hirsute corolla and sepals.

### 48. Ipomoea uninervis

Taxon classificationPlantaeSolanalesConvolvulaceae

J.R.I. Wood & Scotland, Phytokeys 88: 28. 2017. (Wood et al. 2017d: 28)

#### Type.

BRAZIL. Distrito Federal, próximo ao posto Colorado Chacara FTRC, Centro Oeste, 15°41'S, 47°52'W, 6 Feb. 1999, *C. Proença, R.S. Oliveira, C.M. Clemente, J.F. Ribeiro* 2074 (holotype UB8208-2, isotype E).

#### Description.

Perennial undershrub; stems erect, to 1.2 m, sparingly branched, grey-puberulent to subsericeous. Leaves subsessile, 4–12 × 0.1–0.5 cm, linear to narrowly oblong, obtuse, shortly mucronate, both surfaces grey-puberulent to subsericeous, abaxaially paler with one prominent longitudinal vein; petioles 0–3 mm, tomentellous. Inflorescence of few-flowered cymes from the upper leaf-axils, forming a terminal usually elongate inflorescence up to 15 cm in length; bracts formed of reduced leaves, caducous so inflorescence appearing naked; peduncles 1–4 mm, grey-tomentellous; bracteoles 1.5 mm, linear, tomentellous, caducous; pedicels 3–7 mm, grey-tomentellous; sepals subequal, 7.5–8 × 3–4 mm, broadly oblong, obtuse to rounded, grey-tomentose, the inner with broad glabrous, scarious margins; corolla c. 4.5 cm long, pink, pubescent, funnel-shaped; limb c. 4 cm diam.; ovary conical. Capsules and seeds not seen.

#### Illustration.

Figure 35.

**Figure 35.**
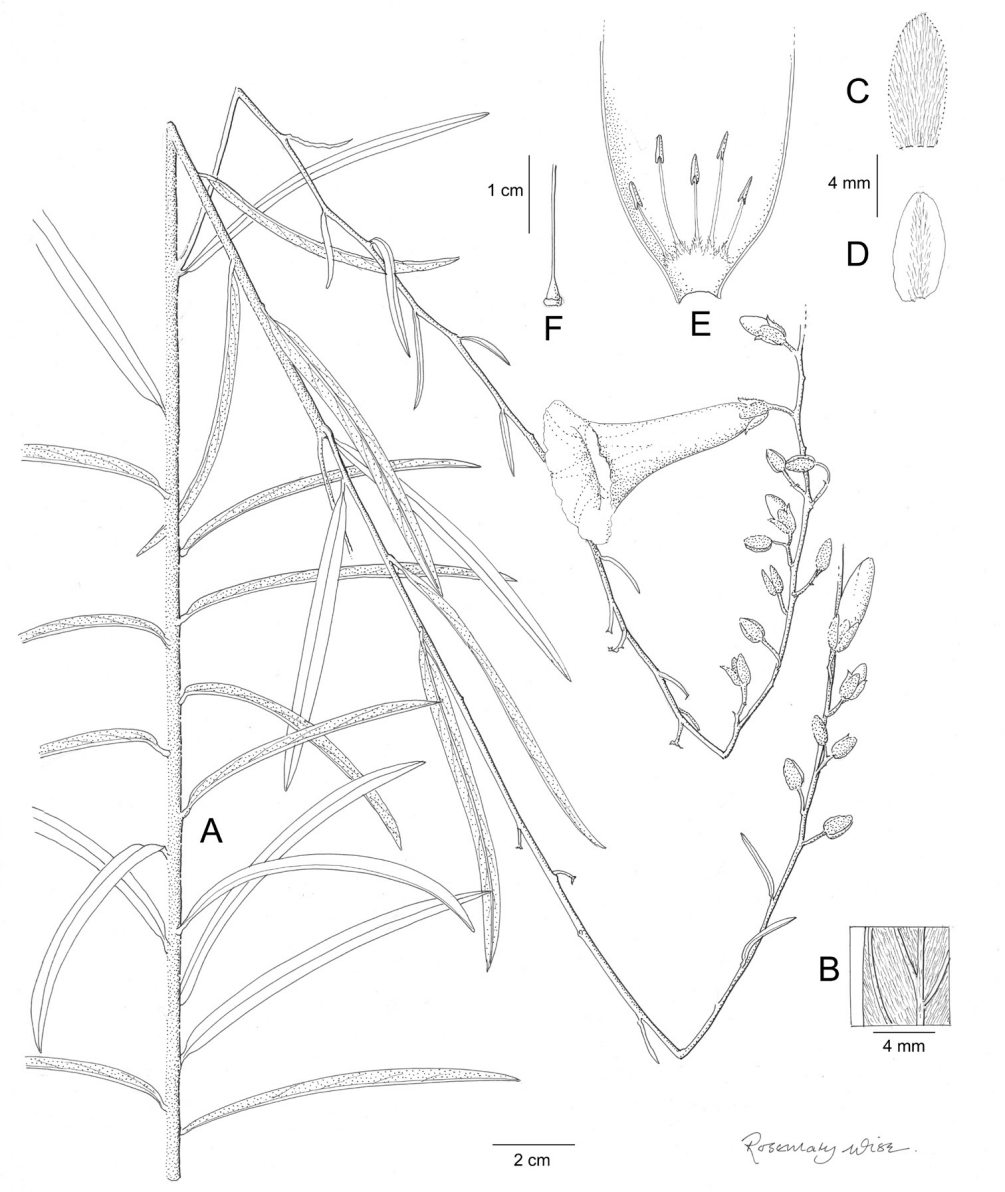
*Ipomoea uninervis*. **A** habit **B** abaxial leaf **C** outer sepal **D** inner sepal **E** corolla opened out to show stamens **F** ovary and base of style. Drawn by Rosemary Wise from *C. Proença et al.* 2074.

#### Distribution.

Endemic to the Distrito Federal and Goiás State in Brazil, where it appears to be a rare species of cerrado.

**BRAZIL. Dist. Fed.**: type collection. **Goiás**: Cristalina, 5 km along estrada para Paracatu, 16°46'S, 47°37'W, 1050 m, *J.R. Pirani et al.* 1560 (SPF, K).

#### Note.

*Ipomoea uninervis* appears close to *I. aprica* but differs in the grey-tomentellous, oblong outer sepals 7.5–8 mm long (these are green-tomentose, broadly ovate to suborbicular and 5–6 mm long in *I. aprica*) and the elongate inflorescence with deciduous bracts so appearing naked (not leafy with persistent bracts). It is also close to *Ipomoea oblongifolia* but differs in the 1-veined leaves and oblong, not elliptic bracts and relatively long inflorescence.

### 49. Ipomoea oblongifolia

Taxon classificationPlantaeSolanalesConvolvulaceae

(Hassl.) O’Donell, Lilloa 23: 493. 1950. (O’Donell 1950b: 493)

Ipomoea argyreia var. lanata Hassl., Repert. Spec. Nov. Regni Veg. 9: 196. 1911. (Hassler 1911: 196). Type. PARAGUAY. Sierra de Amambay, Punta Porá, T. Rojas in Hassler 9821, (lectotype G00175126, designated here; isolectotypes BM, F, G, K, LIL, MPU, P).
Ipomoea argyreia forma oblongifolia Hassl. [as var. lanata forma oblongifolia], Repert. Spec. Nov. Regni Veg. 9: 196. 1911. (Hassler 1911: 196). Type. PARAGUAY. Sierra de Amambay, Punta Porá, T. Rojas in Hassler 9821, (lectotype G00175126, designated here; isolectotypes BM, F, G, LIL, MPU).
Ipomoea argyreia forma linearifolia Hassl. (as var. lanata forma linearifolia], Repert. Spec. Nov. Regni Veg. 9: 196. 1911 (Hassler 1911: 196). Type. PARAGUAY. Sierra de Amambay, Punta Porá, *E. Hassler* 9821b (lectotype G00228031, designated here; isolectotype BM, MPU).

#### Type.

Based on Ipomoea argyreia forma oblongifolia Hassl.

#### Description.

Erect perennial herb or subshrub from a xylopodium; stems to 0.75 cm, unbranched or branched at the base, yellow-brown, woody and glabrous below, pubescent above. Leaves subsessile, 3–12 × 0.5–1.2 cm, oblong, base cuneate, apex obtuse and strongly mucronate with a deflexed falcate tip, shortly floccose on both surfaces, adaxially grey-green, abaxially paler, prominently 3–5-veined; petioles 0–2 mm. Inflorescence terminal, compact and subcapitate, 3–5 cm long, composed of 1–3-flowered subsessile cymes; bracts rarely present, linear, foliose, < 1.5 cm long, peduncles 2 mm, white-tomentose; bracteoles 2 × 1 mm, obovate, retuse, papery, caducous; pedicels 4–5 mm, denselytomentose; sepals subequal, 7–7.5 × 7 mm, suborbicular to broadly elliptic, rounded, densely white tomentose; corolla 4–5 cm long, pink, broadly funnel-shaped, pubescent, limb 5 cm diam., unlobed.

#### Distribution.

Endemic to the Sierra de Amambay.

**PARAGUAY. Amambay**: Alredores de P.J. Cabellero, camino a Cerro Corá, *A. Schinini et al.* 36029 (CTES, PY); ibid., Ruta 5, *A. Krapovickas & C. Cristóbal* 44964 (CTES).

#### Note.

The oblong, shortly floccose, abaxially prominently veined leaves with deflexed mucronate apex, compact terminal inflorescence with tomentose suborbicular sepals are distinctive.

### 50. Ipomoea guaranitica

Taxon classificationPlantaeSolanalesConvolvulaceae

Chodat & Hassl., Bull. Herb. Boiss. Ser. 2: 5: 688. 1905. (Chodat and Hassler 1905: 688)

Ipomoea patula var. villosa Meisn. in Martius et al., Fl. Brasil. 7: 240. 1869. (Meisner 1869: 240). Type. BRAZIL (south), *F. Sello(w*) 5089 (photo F of specimen at B destroyed in 1943).
Ipomoea cornucopia Chodat & Hassl., Bull. Herb. Boiss., ser. 2, 5: 688 (Chodat and Hassler 1905: 688). Type. PARAGUAY. Canindeyú. Río Capabary, Yerbales, Sierra de Maracayú, Sept. 1898, *E. Hassler* 4474 (lectotype G00288030, designated by Wood and Scotland 2017a: 11).

#### Type.

PARAGUAY. Canindeyú, Ipé hú, Yerbales, Sierra de Maracayu, Oct. 1898, *E. Hassler* 5008 (lectotype G00174894, designated by Wood and Scotland (2017a: 11), isolectotypes BM, G, K, NY, P, UC).

#### Description.

Erect undershrub to 1 m with a stout stem, the whole plant densely grey-tomentose. Leaves very shortly petiolate, 10–14 × 3–6 cm, oblong-oblanceolate, obovate or narrowly elliptic, subacute, very shortly mucronate, cuneate at base; petioles 2–3 mm. Flowers solitary, arising in the upper leaf axils; peduncles 2.5–5 cm; bracteoles 1.5–3.5 cm, linear-lanceolate, acuminate, born below the calyx; pedicels absent or nearly so; sepals subequal, 18–22 mm, narrowly ovate to elliptic, obtuse, densely villous-tomentose; corolla 7–9 cm long, pink, funnel-shaped, pilose on midpetaline bands, limb 6 cm diam., undulate. Capsules and seeds not seen.

#### Illustration.

Figure 36.

**Figure 36.**
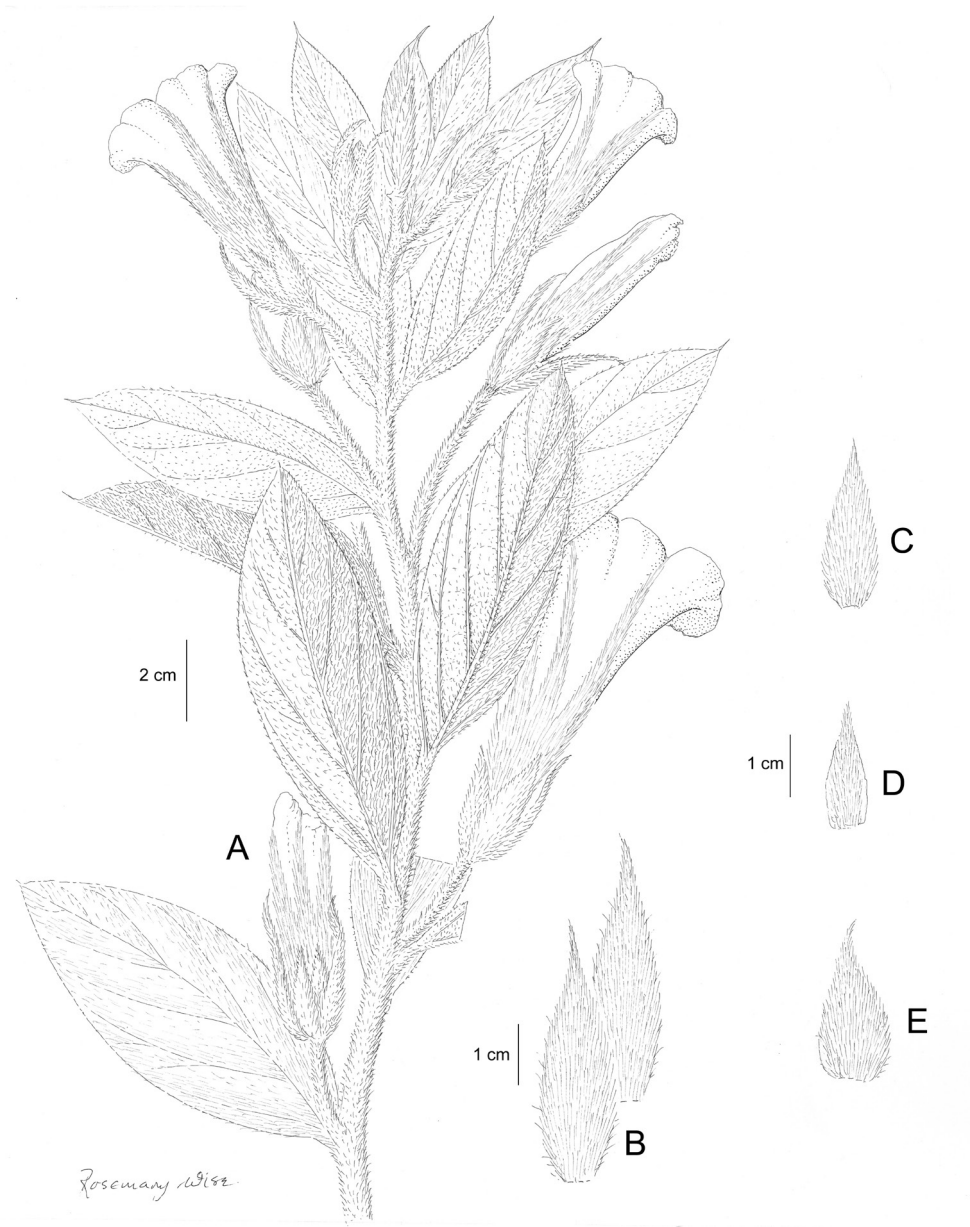
*Ipomoea guaranitica*. **A** habit **B** bracteoles **C** outer sepal **D** middle sepal **E** inner sepal. Drawn by Rosemary Wise from *Balansa* 1075.

#### Distribution.

Eastern Paraguay and neighbouring parts of Brazil in “campo”. There have been no records from Paraguay for about a hundred years.

**PARAGUAY. Alto Paraná**: *K. Fiebrig* 6037 (GH). **Caaguazú**: *B. Balansa* 1075 (P); Río Yhu, *E. Hassler* 9510 & 9510a (MO, BM, P, S). **Canindeyú**: type collection.

**BRAZIL. Paraná**: km 127, Laranjeiras do Sul, *G. Hatschbach et al.* 23119 (MO, NY, S, US).

**Rio Grande do Sul**: entre Panamba & Palmeiras, *Lima* 64-4234 (IPA); Neu Württemberg, Palmeraquelle, *A. Bornmüller* 768 (GH); Palmeira, *B. Rambo* 51964 (S). **Santa Catarina**: 8–13 km W. of Chapecó, *L.B. Smith & R.M. Klein* 14056 (NY, US).

#### Note.

This species is distinguished by the dense floccose indumentum, obovate –oblanceolate leaves, and the long pedunculate solitary flowers lacking pedicels.

### 51. Ipomoea langsdorffii

Taxon classificationPlantaeSolanalesConvolvulaceae

Choisy in A.P. de Candolle, Prodr.9: 368. 1845. (Choisy 1845: 368)

Ipomoea elegans Meisn. in Martius et al., Fl. Brasil. 7: 244. 1869. (Meisner 1869: 244), nom. illeg., non A. Dietrich (1836: 313). Type. BRAZIL. (lectotype J.F.Widgren 309 (BR[00000583768], designated by Wood and Scotland (2017a: 9), isolectotypes S).
Ipomoea patula var. monticola Meisn. in Martius et al., Fl. Brasil. 7: 240. 1869. (Meisner 1869: 240). Type. BRAZIL. Minas Gerais, Vila Rica, *Martius* obs. 788 (holotype M[0185028]).
Ipomoea monticola (Meisn.) O’Donell, Lilloa 26: 371. 1953. (O’Donell 1953a: 371).

#### Type.

BRAZIL. “Rio de Janeiro”, *Langsdorff* (holotype P03560903 ex Herb. Richard).

#### Description.

Trailing perennial herb; stems asperous-hirsute, at least 80 cm long. Leaves shortly petiolate, 4–10 × 2–4 cm, broadly oblong, less commonly ovate, apex obtuse and mucronate, base broadly cuneate to rounded, both surfaces roughly pubescent, abaxially whitish; petioles 0.6–1.6 cm, hirsute. Inflorescence of rather compact, pedunculate cymes arising in the axils of leaf-like bracts towards the apex of the stems; bracts resembling small leaves diminishing markedly in size towards the tips; peduncles 0.5–9 cm, sometimes extended to form the rhachis of a racemose inflorescence; bracteoles 12–15 mm, linear, finely acuminate, persistent, hirsute with white or reddish hairs; secondary peduncles c. 5 mm; pedicels 5–12 mm, hirsute; sepals subequal, 12–18 × 4–5 mm, lanceolate to narrowly ovate, densely villous, outer densely brownish villous, inner paler the central hairs brownish, the marginal hairs whitish; corolla 4–5 cm long, white with dark centre, funnel-shaped, pubescent, limb c. 3–3.5 cm diam. Capsules and seeds not seen.

#### Illustration.

Figure 37.

**Figure 37.**
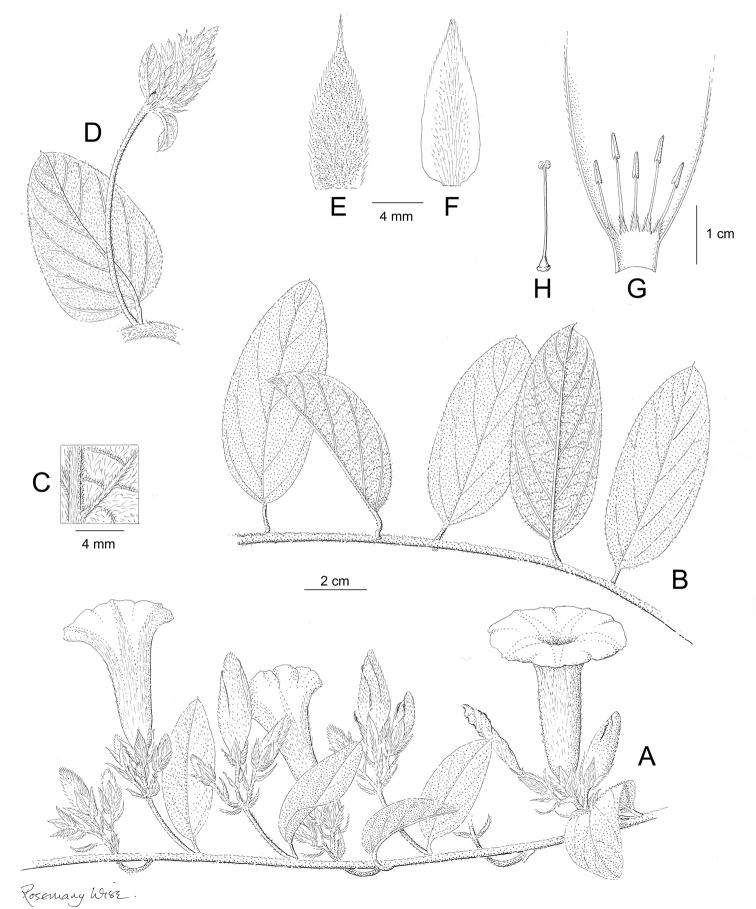
*Ipomoea langsdorfii*. **A, B** habit **C** abaxial leaf surface **D** peduncle and inflorescence **E** outer sepal **F** inner sepal **G** corolla opened out **H** ovary and style. Drawn by Rosemary Wise **A–C, E–H** from *Weddell* 1912; **D** from *Clausen* s.n.

#### Distribution.

Apparently endemic to Minas Gerais State in Brazil, where it grows in cerrado.

**BRAZIL. Minas Gerais**: *P. Clausen* s.n. (BM); Bello Horizonte, Villa Cruzeiro do Sul, *M. Barreto* 2312 (F); Betim, Contagem, Faz. do Cabuí, *L.O. Williams* 5101 (GH); San Francisco, *M. Weddell* 1175 (P), 1912 (P); Serra do Itabirito, 45km SW of Belo Horizonte *H.S. Irwin et al.* 19706 (FTG).

#### Note.

See Wood and Scotland (2017a) for a discussion of the problematic typification of *Ipomoea patula* Choisy and its implications. The type location is given as “Rio de Janeiro” but this seems improbable, given that this is a cerrado species, otherwise only known from Minas Gerais.

This species resembles *Ipomoea valenzuelensis* but the leaves are whitish abaxially and never lobed, and the cymes are usually more than 3-flowered.

### 52. Ipomoea malvaviscoides

Taxon classificationPlantaeSolanalesConvolvulaceae

Meisn. in Martius et al., Fl. Brasil. 7: 284. 1869. (Meisner 1869: 284)

#### Type.

BRAZIL. Minas Gerais, Caldas, *A.F. Regnell* Ser. 3, 202 (holotype BR00005837670, isotypes S, US, ?P03524169).

#### Description.

Twining herb to 1 m, stems tomentellous and somewhat glabrescent. Leaves petiolate, 3–6.5 × 2.5–5.5 cm, entire and ovate or 3-lobed to half way with the sides almost parallel, base weakly cordate or truncate with rounded auricles, apex rounded on central lobe, acute on laterals, strongly mucronate, adaxially thinly tomentose, greenish, abaxially densely white-tomentose with long appressed hairs; petioles 2.5–5 cm, densely pubescent. Inflorescence of moderately dense, few-flowered, axillary cymes, peduncles 4–9 cm, tomentellous; bracteoles 8–12 mm, linear to linear-lanceolate, tomentellous, somewhat persistent; pedicels 2–14 mm, tomentellous; sepals subequal, 9–13 mm, broadly lanceolate, acuminate, densely softly pilose, inner with pale, glabrous margins; corolla c. 4.5 cm long, funnel-shaped, pink, pubescent; limb c. 3 cm diam. Capsules and seeds not seen.

#### Distribution.

Apparently endemic to Minas Gerais State in Brazil.

**BRAZIL. Minas Gerais**: only known from the type collection.

#### Notes.

Resembling *Ipomoea verbasciformis* in the short pedicels and indumentum, but twining in habit, the leaves 3-lobed and distinctly petiolate and the inflorescence clearly axillary, not terminal. The parallel-sided leaves are also distinctive.

The Paris specimen cited above is ambiguously labelled but is probably an isotype.

### 53. Ipomoea rojasii

Taxon classificationPlantaeSolanalesConvolvulaceae

Hassl., Repert. Spec. Nov. Regni Veg. 9: 152. 1911. (Hassler 1911: 152)

#### Type.

PARAGUAY. Sierra de Amambay, *Rojas* in *Hassler* 10752 (lectotype G00175159, designated here; isolectotypes BM, G, K, NY, F, MVM, P, S, UC).

#### Description.

Erect perennial undershrub to 1 m, stems stout, white-lanate. Leaves subsessile, 4.5–12 × 1.5–2 cm, oblong, apex falcate, acute and strongly apiculate, base attenuate, softly tomentose on both surfaces, veins beneath prominent; pedicels 0–5 mm. Inflorescence terminal, elongate, up to 30 cm long, flowers in sessile or shortly pedunculate few-flowered cymes, (often solitary) in the axils of leaf-like bracts which diminish in size upwards; peduncles 0–0.7 cm; bracteoles 7–11 mm, linear-lanceolate, finely acuminate, deciduous; pedicels 3–10 mm, densely tomentose; sepals slightly unequal, 9–12 × 6–7 mm, ovate, acute, mucronate, densely white-tomentose, inner sepals broader and slightly shorter; corolla 5.5–7.5 cm long, pink, densely pilose in bud and on midpetaline bands, limb c. 4 cm diam., entire. Capsules and seeds not seen.

#### Distribution.

A cerrado species endemic to the Sierra de Amambay.

**PARAGUAY. Amambay**: *Rojas in Hassler* 10891 (F, P, BM); *A. Schinini & M. Dematteis* 33798 (FCQ, CTES); Cerro Coro, *D.R. Brunner* 1416 (MO, PY); camino a la Colonia Naranja Hai, *N. Soria* 7667 (FCQ, MO, G); camino al Cerro Muralla, *N. Soria* 6377 (FCQ); Cerro Alambique, *N. Soria* 6400 (FCQ).

#### Note.

Distinguished by the prominently mucronate, falcate (and bent down) leaf tips, oblong, tomentose laminas, and elongate inflorescence with shortly pedunculate flowers with short pedicels.

### 54. Ipomoea estrellensis

Taxon classificationPlantaeSolanalesConvolvulaceae

Hassl. ex O’Donell, Arq. Mus. Paranaense 9: 220. 1952. (O’Donell 1952: 220)

Ipomoea chrysotrichoides Hassl., nom. nud., Add. Plantae Hasslerianae 18. 1917. (Hassler 1917: 18).

#### Type.

PARAGUAY. Amambay, Cabecera Estrella, Pedro Juan Caballero, Sept. 1933, *T. Rojas* 6260 (holotype LIL190807).

#### Description.

Subshrub with erect stems from a xylopodium to c. 60 cm, stems pilose with long soft hairs. Leaves subsessile, ovate to broadly elliptic, acute and mucronate, rounded to subcordate at base, prominently veined especially abaxially, both surfaces densely adpressed asperous-pilose, the hairs bulbous-based; borders highlighted, densely white-ciliolate; petioles 2–3 mm, pubescent. Flowers solitary from the upper leaf axils; peduncles suppressed or very short, 0–4 mm, pilose; bracteoles 6–7 mm, linear; pedicels 4–8 mm, pilose; sepals 10–13 × 4 mm long, subequal, ovate, acuminate, sericeous, similar but inner subacute and mucronate, c. 5 mm wide; corolla 6–9 cm long, pink, midpetaline bands sericeous, limb 4–6 cm diam., undulate. Capsules and seeds not seen.

#### Distribution.

Endemic to the Sierra de Amambay in Paraguay, where it was probably found growing in cerrado. There have been no confirmed records for over eighty years.

**PARAGUAY. Amambay**: *T. Rojas* 6362 (LIL); *E. Hassler* 9819 (BM), 10052 (BM, G, K, P).

#### Note.

Characterised by the subsessile, broadly elliptic leaves with highlighted ciliolate margins and the solitary axillary flowers, the peduncles nearly suppressed and the pedicels short.

*U. Eskuche & Z. Ahumada 06177* (G) from 36 km N of San Estansilao in Dept. San Pedro may belong to this species but differs in the longer peduncles (mostly 6–10 mm).

### 55. Ipomoea paraguariensis

Taxon classificationPlantaeSolanalesConvolvulaceae

Peter, Die Naturlichen Pflanzenfamilien 4(3a): 29. 1897 [pub. 1891]. (Peter 1891: 29)

Ipomoea argyreia var. paraguariensis (Peter) Chodat & Hassl., Bull. Herb. Boiss., ser. 2, 5: 689. 1905. (Chodat and Hassler 1905: 689).
Ipomoea argyreia forma paraguariensis (Peter) Hassl. [as var. discolor forma paraguariensis ], Repert. Spec. Nov. Regni Veg. 9: 195. 1911. (Hassler 1911: 195).
Ipomoea argyreia forma grandiflora Chodat & Hassl., Bull. Herb. Boiss., ser. 2, 5: 689. 1905. (Chodat and Hassler 1905: 689). Type. PARAGUAY [Canindeyú], Ipé Hú, Sierra de Maracayú, *E. Hassler* 5229 (lectotype G00166317, designated here; isolectotype G).
Ipomoea argyreia forma intermedia Chodat & Hassl. [as var. paraguariensis forma intermedia], Bull. Herb. Boiss., ser. 2, 5: 689. 1905. (Chodat and Hassler 1905: 689). Type. PARAGUAY. [Canindeyú], Yerbales de Sierra de Maracayu, 1898/9, *E. Hassler* 5748 (lectotype G00175069, designated here; isolectotypes BM, G, K, NY, P).
Ipomoea argyreia forma salicifolia Chodat & Hassl. [as var. paraguariensis forma salicifolia], Bull. Herb. Boiss., ser. 2, 5: 68. 1905. (Chodat and Hassler 1905: 689). Type. PARAGUAY. [Canindeyú], Caruguaty, *E. Hassler* 4599 (lectotype G00175072, designated here; isolectotypes BM, G, NY).
Ipomoea nitens Chodat & Hassler, Bull. Herb. Boiss. Ser. 2 5: 689. 1905 (Chodat and Hassler 1905: 689). Type. PARAGUAY. [Canindeyú], fl. Jezui Guazu [Río Jejuí Guazú], Dec. 1898, *E. Hassler* 5691 (holotype G00175121).
Ipomoea argyreia forma nitens (Chodat & Hassler) Hassl. [as var. discolor forma nitens]., Repert. Spec. Nov. Regni Veg. 9: 196. 1911. (Hassler 1911: 196).
Ipomoea argyreia var. martii Hassl., Repert. Spec. Nov. Regni Veg. 9: 195. 1911. (Hassler (Hassler 1911: 195). Type. PARAGUAY. [Canindeyú], Ipé Hu, Sierra de Maracayu, *E. Hassler* 5229 (lectotype G00166317, designated here; isolectotypes G).
I.omoea. argyreia var. discolor Hassl., Repert. Spec. Nov. Regni Veg. 9: 195. 1911. (1911: 195). Type. PARAGUAY. [Canindeyú], Yerbales de Sierra de Maracayu, 1898/9, *E. Hassler* 5748 (lectotype G00175069, designated here; isolectotypes BM, G, K, NY, P).

#### Type.

PARAGUAY. Villarrica, *B.Balansa* 1074 (lectotype GOET005546, designated by Staples et al. 2012: 673, isolectotypes GOET, G, K, P).

#### Description.

Erect subshrub from a woody rhizome, stems tomentose, eventually glabrescent. Leaves very shortly petiolate, 1.5–5 × 0.7–2.5 cm, oblong-elliptic or ovate-elliptic, mucronate, base rounded to cuneate, adaxially densely pubescent, green, abaxially silvery tomentose with long appressed hairs, veins moderately prominent; petioles 2–3 mm long. Inflorescence terminal, panicle-like formed of 1–3-flowered cymes; peduncles up to 1.5 cm; bracteoles 5–9 mm, lanceolate, caducous; pedicels 2–7 mm; sepals 6–8(–10 mm in fruit), ovate to suborbicular, obtuse, mucronate, tomentose, inner with glabrous, scarious margins; corolla 3.5–6 cm long, funnel-shaped, pink, tomentose, limb 3.5 cm diam. Capsules 10–12 × 7 mm, ellipsoid, glabrous seeds 5–6 × 3.5 mm, blackish, pilose on margins, the hairs c. 8 mm long, deciduous.

#### Illustration.

Figure 5B.

#### Distribution.

Endemic to cerrados in Paraguay and recorded from three departamentos but apparently rare.

**PARAGUAY. Caazapá**: Est. Nu Pyajhú, próximo a San Juan Nepomuceno, *C.V. Pavetti* s.n. (SCP); Coronel Oviedo, *T. Carruthers et al.* 105 (FCQ). **Canindeyú**: Curuguaty, *T. Carruthers et al. 99* (FCQ). **Guairá**: Villarrica, *B. Balansa* 1074 (P), ibid., *Jorgensen* 4297 (F), ibid., *Jorgensen* 4297 (S); Col. Independencia, *A. Schinini & E. Bordas* 25218 (CTES); ibid., *F. Mereles* 3376 (FCQ, G); ibid., *R. Degen et al.* 4010 (FCQ).

#### Note.

Characterised by the discoloured elliptic leaves. *Hassler* 4599 (BM, G, NY), the type of Ipomoea argyreia forma salicifolia Chodat & Hassler (1905: 689), is very close to *Ipomoea rojasii* but the leaves are discoloured, narrowly ovate and shortly acuminate, reaching only to 6.5 cm long, and the sepals are shorter. It looks like an intermediate between *Ipomoea rojasii* and *I. paraguariensis*.

### 56. Ipomoea mendozae

Taxon classificationPlantaeSolanalesConvolvulaceae

J.R.I. Wood & Scotland, Kew Bull. 70 (31): 44. 2015. (Wood et al. 2015: 44)

#### Type.

BOLIVIA. Santa Cruz, Prov. Vallegrande, Guadalupe, 350 m de la represa sobre senda a La Estancia Collana, *M. Mendoza & E. Calzadilla* 416 (holotype USZ, isotypes K, LPB).

#### Description.

Perennial herb, stems decumbent or ascending, 0.5–1.5 m long, relatively stout and slightly woody, densely white-tomentose. Leaves petiolate, 5–11.5 × 2.5–7 cm, ovate to subrhomboid, acute and shortly mucronulate, base truncate to broadly cuneate, adaxially grey-green, densely pubescent with long hairs, abaxially grey-tomentose; petioles 0.5–2 cm, tomentose. Inflorescence subterminal formed of pedunculate 1–3(–5)-flowered cymes from the upper leaf axils; bracts similar to the leaves but smaller, diminishing in size upwards, peduncles 6–12 cm, white-tomentose; secondary peduncles 1–1.5 cm; bracteoles 5–8 mm, linear to filiform; pedicels 5–12 mm, tomentose; sepals subequal, 8–10 × 3–4 mm, ovate-elliptic, obtuse, outer densely tomentose, the inner similar but with scarious, glabrous margins; corolla 5.5–8 cm long, pink, funnel-shaped, in bud tomentose on exterior, at maturity somewhat glabrescent but with pubescent midpetaline bands, limb 5.5–6 cm diam., shallowly lobed. Capsules and seeds not seen.

#### Illustration.

Figure 38.

**Figure 38.**
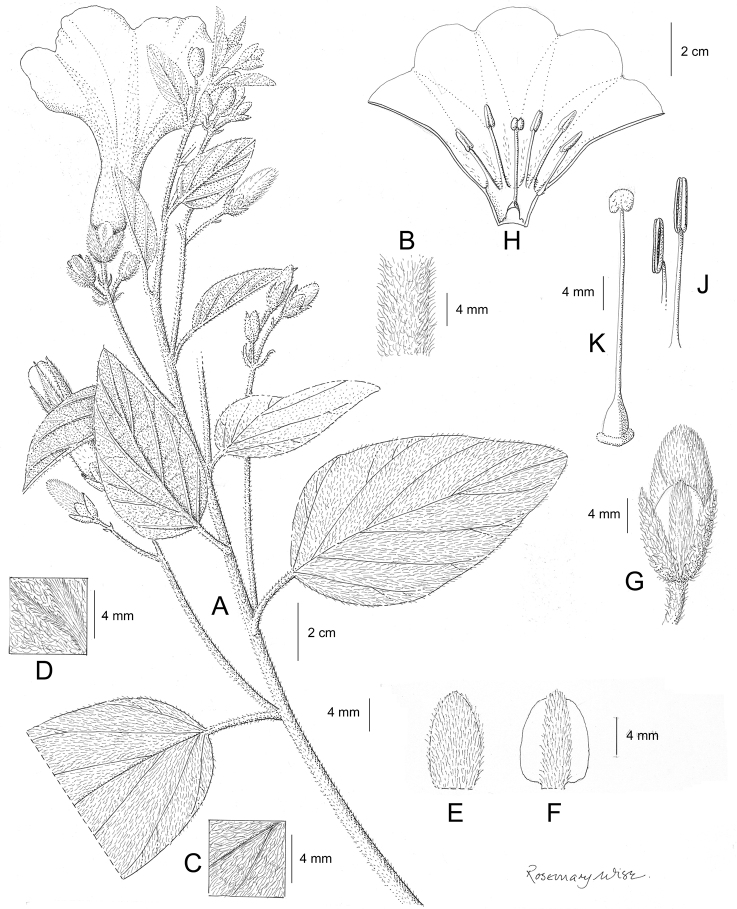
*Ipomoea mendozae*. **A** habit **B** portion of stem showing indumentum **C** adaxial leaf surface **D** abaxial leaf surface **E** outer sepal **F** inner sepal **G** calyx with flower bud **H** corolla opened out to show stamens **J** anthers **K** ovary and style. Drawn by Rosemary Wise from *Mendoza & Calzadilla* 416.

#### Distribution.

Endemic to the Vallegrande area in the Bolivian inter-Andean valleys where it is uncommon in open grassy scrubland on hillsides from 1900 to 2200(–2500) m.

**BOLIVIA. Santa Cruz**: Vallegrande area, *I. Vargas* 33 (NY); road to Tierras Nuevas, *M. Nee et al.* 37406 (NY); on descent to Piraimiri, *J.R.I. Wood et al.* 21743 (LPB); Vallegrande-Postrervalle, *G.A. Parada et al.* 5326 (MO, USZ).

#### Note.

This appears to be a rather isolated species morphologically. The subterminal inflorescence suggests it is essentially erect or ascending in habit, as indicated by most field notes, but it is unlike most erect species in South America in its broad leaves and Andean habitat.

### 57. Ipomoea gypsophila

Taxon classificationPlantaeSolanalesConvolvulaceae

J.R.I. Wood & Scotland, Kew Bull. 70 (31): 61. 2015. (Wood et al. 2015: 61)

#### Type.

BOLIVIA. Tarija, Prov. Aniceto Arce Ruiz, La Merced, 30 km de Padcaya hacia Bermejo, *S.G. Beck, R. Kiesling & D. Metzing* 22139 (holotype LPB, isotypes SI n.v., K [leaves only]).

#### Description.

Stout trailing or weakly ascending plant; stems lanate. Leaves petiolate, 7–10 × 6–10 cm, ovate, base cordate with rounded overlapping auricles, apex acute, adaxially appressed white-villous, abaxially densely white lanate-tomentose; petiole 3–6 cm. Flowers 1(–3) in pedunculate, axillary cymes; peduncles 5–7 cm, lanate, straight or nearly so; bracteoles 2–3 mm, lanceolate, somewhat persistent; pedicels 8 mm; sepals subequal, 15 × 5 mm, oblong-lanceolate to oblong-ovate, obtuse, lanate; corolla 7–8 cm long, funnel-shaped, uniformly pink, tomentose at base and on midpetaline bands, limb c. 5 cm diam. Capsules and seeds not seen.

#### Illustration.

Wood et al. (2015: 63).

#### Distribution.

Endemic to Southern Andean Bolivia at around 2000 m; rare and only known from five collections.

**BOLIVIA. Chuquisaca**: Zudañez, between Puca Pampa and Presto, *J. Gutiérrez et al.* 2863 (HSB, OXF). **Tarija**: Cercado, Yesera, *T. Meyer* 17334 (LIL), 17981 (LIL); *E. Bastian* 416 (LPB).

#### Note.

This species bears a superficial resemblance to *Ipomoea descolei* O’Donell but is Andean in distribution and immediately distinguished by the indumentum of the corolla, sepals, stem and peduncles, which is appressed, not spreading. The leaves are not strongly reticulate beneath, have white hairs on both surfaces (not dark green above) and the flowers are usually solitary and the peduncles reach only 7 cm long.

### 58. Ipomoea appendiculata

Taxon classificationPlantaeSolanalesConvolvulaceae

J.R.I. Wood & Scotland, Kew Bull. 70 (31): 57. (Wood et al. 2015: 57)

#### Type.

BOLIVIA. Santa Cruz, Prov. Gran Chaco, 10–20 km from Villamontes towards Palos Blancos, *J.R.I. Wood, D. Villarroel & B. Williams* 27607 (holotype USZ, isotypes OXF, K, LPB).

#### Description.

Vigorous liana climbing over other plants to c. 3 m, stems woody, pale brown, glabrous. Leaves petiolate, slightly succulent and often transversely folded, 5–7 × 4–5 cm, broadly ovate, shallowly cordate with rounded auricles, shortly acuminate to a mucronate apex, margin entire, both surfaces pale green and glabrous; petioles 2–3.5 cm, glabrous. Inflorescence of shortly pedunculate axillary cymes with up to five flowers; peduncles 2–3.5 cm, rigid, glabrous; bracteoles 2–4 × 1 mm, lanceolate, boat-shaped, scurfy puberulent, caducous; secondary and tertiary peduncles 1–2.5 cm; pedicels (1–)2.2–3 cm, straight, glabrous below, upwards thickened, scurfy puberulent; sepals subequal, 5–7 × 3–5 mm, ovate, puberulent, each with two swollen glabrous appendages on each side towards the base, outer sepals acute to obtuse, mucronate, inner sepals obtuse to rounded, minutely mucronate, margins scarious, glabrous; corolla 6.5–7 cm long, funnel-shaped, uniformly pink, puberulent in bud, glabrescent at anthesis, limb 5 cm diam., undulate but not lobed. Capsules ovoid, 6 × 7 mm, glabrous; seeds 1.6 × 1 mm. ovoid, obtuse, brown, glabrous.

#### Illustration.

Figures 2H, 39.

**Figure 39.**
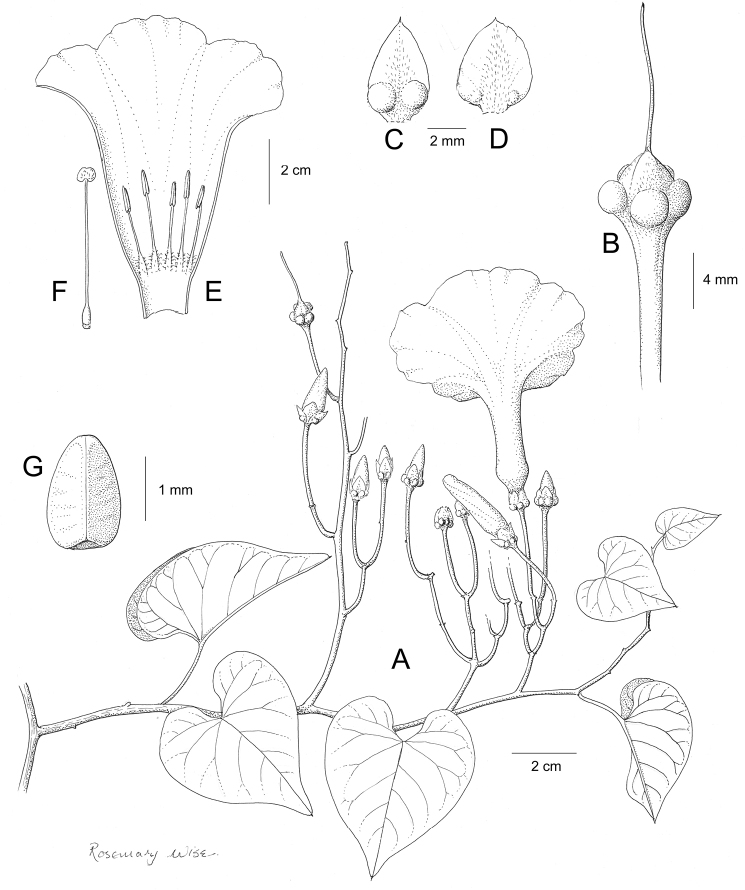
*Ipomoea appendiculata*. **A** habit **B** immature fruit showing appendages **C** outer sepal **D** inner sepal **E** corolla opened out to show stamens **F** ovary and style **G** seed. Drawn by Rosemary Wise from *Wood et al.* 27633.

#### Distribution.

Endemic to southern Bolivia where it grows in chaco scrub between Villamontes and Palos Blancos in the Andean foothills at 500–650 m.

**BOLIVIA. Tarija**: Prov. Gran Chaco, *J.R.I. Wood et al.* 28024 (LPB, USZ), 28027 (LPB, OXF, USZ).

#### Note.

This species shows some similarity to *Ipomoea amnicola* Morong in the somewhat succulent leaves, these often being deciduous on herbarium species, and also to *I. tarijensis* O’Donell in the commonly folded leaves. The 5–6 mm long sepals are shorter than those of *I. hieronymi* and lack the dark glands sometimes found in that species and in *I. megapotamica*. The distinctive swollen appendages on the dorsal surface of the sepals immediately separate this species from all others known to us.

### 59. Ipomoea cearensis

Taxon classificationPlantaeSolanalesConvolvulaceae

O’Donell, Lilloa 26: 363. 1953. (O’Donell 1953a: 363)

#### Type.

BRAZIL. Ceará, Salvarão, *A. Löfgren* 158 (holotype S07-4422).

#### Description.

Vigorous liana-like twiner of unknown height; stems stout, herbaceous, glabrous to thinly pilose. Leaves petiolate, 10–12 × 8–13 cm, broadly ovate, shortly acuminate, mucronate, base cordate with rectangular sinus and rounded auricles, margin entire to obscurely undulate; both surfaces glabrous or abaxial veins thinly pubescent; petioles 6–8.5 cm, glabrous. Inflorescence of axillary pedunculate cymes, sometimes compound, peduncles 1.3–7.5 cm, glabrous; bracteoles caducous, not seen; secondary peduncles 2.5–8 cm; tertiary peduncles up to 6.5 cm; pedicels 6–26 mm, glabrous; sepals slightly unequal, glabrous or almost so, outer 8–9 × 5 mm, elliptic, mucronate, the margin narrow, scarious; inner 9–10 × 7–8 mm, the margins broad, scarious; corolla 11–12 cm long, pale pink with darker centre, funnel-shaped, pilose on the midpetaline bands, limb 8–9 cm diam. Capsules and seeds unknown.

#### Distribution.

A rare species of northeastern Brazil.

**BRAZIL. Ceará**: type of *Ipomoea cearensis*. **Maranhão**: Mun. Lorêto, Ilha de Balsas, *G & L. T. Eiten* 4077A (K, NY, SP).

#### Note.

Clearly part of the *Ipomoea megapotamica* complex but immediately recognised by its very large corolla. The (near) glabrous sepals are also distinct.

### 60. Ipomoea vivianae

Taxon classificationPlantaeSolanalesConvolvulaceae

Krapov., Bonplandia (Corrientes) 18 (1): 57. 2009. (Krapovickas 2009: 57)

#### Type.

ARGENTINA. Salta, Dept. Rivadavia, Pluma del Pato, 13 Feb. 2005, *V. Solis Neffa, J.G. Seijo, J.G. Grabiele & W. Reynoso* 1985 (holotype CTES0013270, isotypes LIL, SI).

#### Description.

Twining perennial liana to at least 3 m, stems glabrous or sparsely pubescent when young, becoming woody with corky bark when old. Leaves petiolate, 2–4 × 2.5–5.5 cm, broadly ovate to subreniform, abruptly acuminate, shallowly cordate, glabrous or very thinly pubescent, abaxially somewhat paler; petioles 2–4 cm, slender. Inflorescence of shortly pedunculate axillary cymes, often raceme-like on short side branches; peduncles short, 1–2 cm, commonly somewhat woody; bracteoles 2 mm, caducous; secondary peduncles 5–10 mm; pedicels 10–16 mm; sepals subequal, outer 6–8 × 3–4 mm, ovate-elliptic, subacute, thinly pubescent, inner sepals c. 1 mm longer, rounded, the central part pubescent but with glabrous scarious margins; corolla 4–5 cm long, funnel-shaped, white, sometimes with pink centre, pubescent in bud and on midpetaline bands, limb 3–4 cm diam., unlobed. Capsules ovoid, 8 × 6 mm, glabrous, rostrate, the style base persistent; seeds 5 mm long, long-pilose.

#### Illustration.

Figure 40.

**Figure 40.**
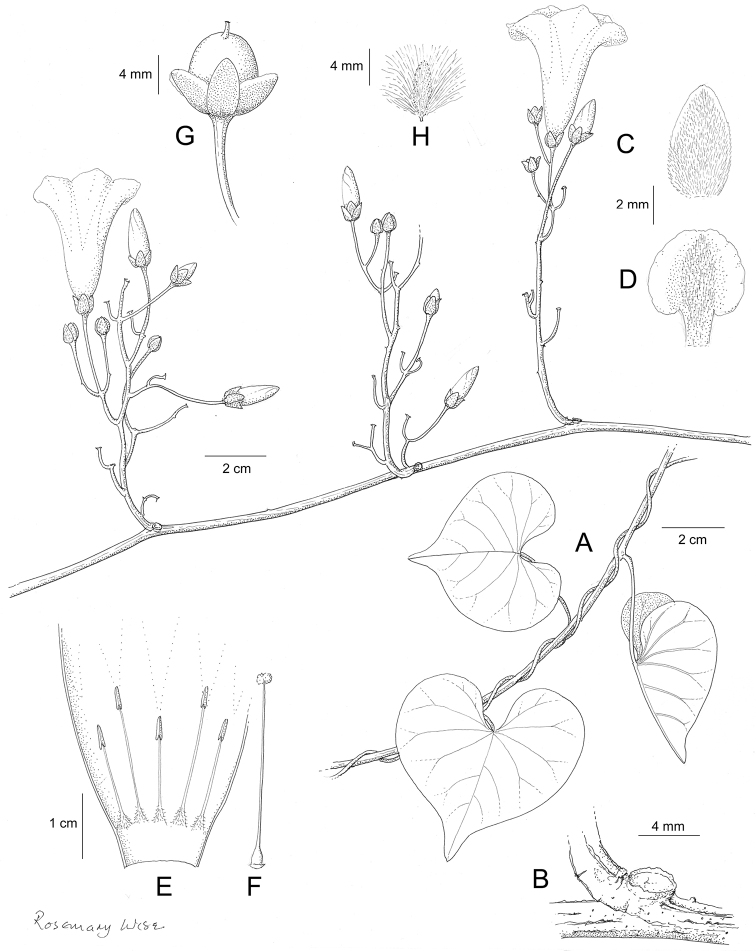
*Ipomoea vivianae*. **A** habit **B** woody stem **C** outer sepal **D** inner sepal **E** corolla opened out to show anthers **F** ovary and style **G** fruit and calyx **H** seed. Drawn by Rosemary Wise **A–F** from *Petersen* 12909; **G, H** from *Navarro* 2122.

#### Distribution.

A western Chaco species found in NW Argentina, western Paraguay and southern Bolivia.

**ARGENTINA. Formosa**: *T.M. Petersen* 12909 (C, CTES, G). **Salta**: type of *Ipomoea vivianae*.

**PARAGUAY. Boquerón**: Mayor Pedro Lagerenza, *Schinini & Bordas* 15091 (CTES); Col. Fernheim, Filadelfia, *August & Ulmke* 48 (CTES); Picada 104, Ruta Transchaco, *R. Degen & F. Mereles* 2979 (FCQ); Colonia 4 de Mayo, *F. Mereles & R. Degen* 5148 (CTES, FCQ).

**BOLIVIA. Santa Cruz**: Prov. Cordillera, *A. Fuentes & G. Navarro* 2418 (BOLV, LPB, NY, MO, USZ). **Tarija**: Gran Chaco, *P. Zuñiga et al.* 175 (HSB).

#### Note.

Some of the cited paratypes of this species including *Krapovickas & Cristóbal* 44938 (CTES), 44944 (CTES, SI), 45042 (CTES, SP) and *Schinini et al.* 29283 (CTES) from Amambay in eastern Paraguay are *Ipomoea megapotamica*. Plants from the true Chaco in western Paraguay, Argentina (Salta, Formosa) and Bolivia (Tarija, Santa Cruz) differ in the nearly glabrous leaves, usually white corolla, distinctly corky stems and, in particular, the often raceme-like inflorescence that develops on short shoots. These characters serve to separate *Ipomoea vivianae* from *I. megapotamica* but this species may eventually be shown to be only an adaptation of *I. megapotamica* to the arid climate of the Chaco. Krapovickas seems not to have known *Ipomoea megapotamica*.

### 61. Ipomoea megapotamica

Taxon classificationPlantaeSolanalesConvolvulaceae

Choisy in A.P. de Candolle, Prodr. 9: 375. 1845. (Choisy 1845: 375)

Argyreia megapotamica var. puberula Griseb., Symb. Fl. Argent. 263. 1879. (Grisebach 1879: 263). Type. Based on Ipomoea megapotamica Choisy

#### Type.

“URUGUAY” (possibly south Brazil fide O’Donell 1948a: 182), *Otto* s.n. (syntype B, not found, presumably destroyed in 1943), neotype BRAZIL. Mato Grosso do Sul, *G. Hatschbach* 23711 (NY01013991, designated by Wood et al. (2015: 59), isoneotypes F, MBM?, RB).

#### Description.

Twining perennial herb reaching 2 m, stems thinly pubescent to subglabrous. Leaves petiolate, 4–10 × 4–10 cm, broadly ovate, cordate, acute and apiculate, minutely scabridulous to thinly appressed pubescent on both surfaces, abaxially paler, often dark gland-dotted, sometimes densely appressed pilose and somewhat velutinous; petioles 2.5–5 cm. Inflorescence of long pedunculate, many-flowered, lax, compound cymes; peduncles 2.5–20 cm, glabrous to puberulent; bracteoles linear, 3–4 mm, caducous; secondary peduncles 1–5.5 cm; tertiary peduncles 1–1.5 cm; quaternary peduncles 0.5–1 cm; pedicels 3–5 mm long, puberulent; sepals subequal 5–7.5 × 3.5–4.5 mm, ovate, acute to shortly apiculate, the apex erect (often slightly bent backwards), tomentellous, often dotted with dark glands, inner elliptic, obtuse to subacute, the margins scarious; corolla 4.5–6 cm long, pale pink with a darker centre, pubescent, funnel-shaped, limb 3–4 cm diam., unlobed. Capsules subglobose, 7 × 6 mm, rostrate with mucro c. 3 mm long, glabrous; seeds 4 × 2 mm, long pilose on margins with hairs to 8 mm.

#### Illustration.

Figures 8K, 41.

**Figure 41.**
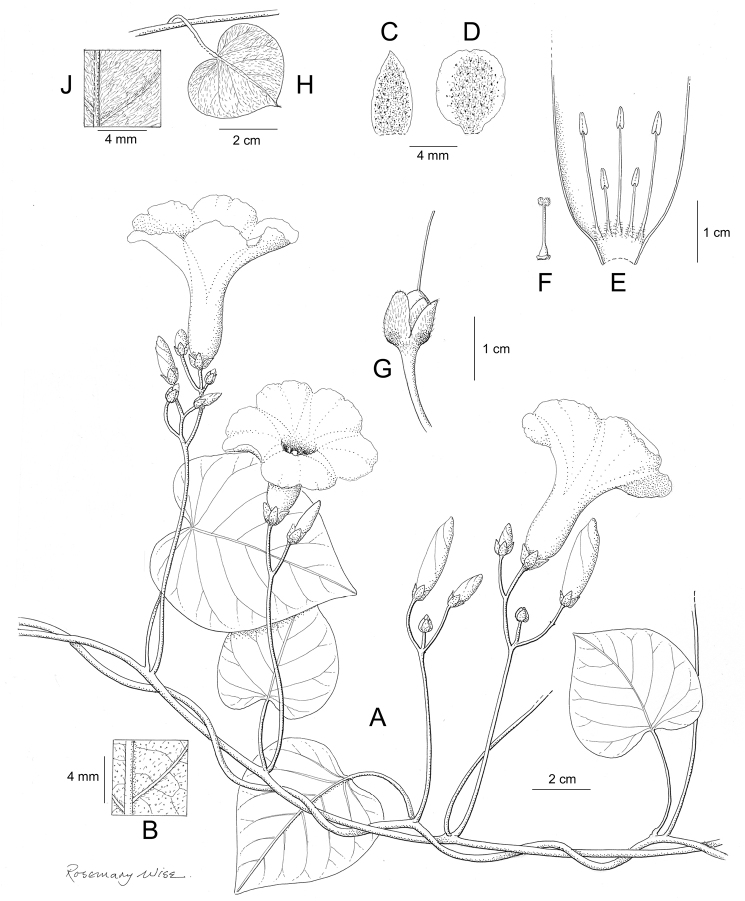
*Ipomoea megapotamica subsp. megapotamica*. **A** habit **B** abaxial leaf surface **C** outer sepal **D** inner sepal **E** corolla opened out to show anthers **F** ovary and style **G** Young fruit and calyx showing glands at base of sepals. *Ipomoea megapotamica subsp. velutina*. **H** leaf **J** abaxial leaf surface. Drawn by Rosemary Wise **A, B** from *G. Hatschbach* 23711; **C–G** from *Fernández Casas & Molero* 4302; **H–J** from *A. Fernández-R*. et al. 9612.

#### Variation.

*Ipomoea megapotamica* is widely distributed in the South American lowlands and quite variable. Specimens from the southern part of its range have leaves abaxially glabrous to thinly pubescent while those from Venezuela and NE Brazil have leaves abaxially softly appressed pilose. These two forms are here recognised as subspecies, which intergrade in central Brazil, for example in Mato Grosso (*D. Philcox* 3722 (K, NY, RB) from Xavantina and *B. Dubs* 1840 (ARIZ, S, Z) from the Chapada dos Guimarães).

### 61a. Ipomoea megapotamica subsp. megapotamica

Taxon classificationPlantaeSolanalesConvolvulaceae

Ipomoea megapotamica var. cordifolia Hassl., Repert. Spec. Nov. Regni Veg. 9: 157. 1911. (Hassler 1911: 157). Type. PARAGUAY. Concepción, Naranjati, *Hassler* 10401 (lectotype G00175106, designated here).
Ipomoea riograndensis P.P.A. Ferreira & Miotto, Kew Bull. 66(2): 290. 2011. (Ferreira and Miotto 2011: 290). Type. BRAZIL. Rio Grande do Sul, Puerto Alegre, *P.P.A. Ferreira* 118 (holotype ICN; isotypes K, LIL, SP).

#### Diagnosis.

This subspecies is distinguished by its leaves, which are abaxially glabrous to thinly pubescent. The sepals are relatively long, usually 6–7.5 mm in length.

#### Distribution.

Found around the north and east of the Chaco in Bolivia, Paraguay and Brazil and, like a number of Chaco species, also present in NE Brazil. In Bolivia it has been mostly found at low altitudes along the line of the new road from Santa Cruz to Brazil and was notably more common immediately following its construction, becoming less common in subsequent years.

**ARGENTINA. Salta**: Rivadavia, *A. Maranta & P. Arenas* 118 (CTES); ibid., *M.E. Suarez* 12 (CTES).

**PARAGUAY. Alto Paraguay**: Fortín Teniente Martínez, *Fernández Casas & Molero* 4302 (G, MA, NY); P.N. Defensores del Chaco, *E. Zardini & J. Godoy* 50415 (ARIZ, MO); ibid., *F. Mereles et al.* 8899 (FCQ). **Amambay**: Cerro Corá, *Fernández Casas & Molino* 6017 (G, NY), ibid., 6081 (G, NY); *Krapovickas & Cristóbal* 44944 (CTES, FCQ), 45042 (CTES, FCQ). **Boquerón**: Filadelfia, *R.O. Vanni et al.* 2521 (CTES, G); Colonia Fernheim, *P. Arenas* 3311 (FCQ). **Presidente Hayes**: Com. Armonia, *O. Aquino et al*. 436 (FCQ); camino a Riacho González, *R. Degen* 3467 (FCQ). **San Pedro**: Com. 25 de Diciembre, *J.R.I. Wood & G. González* 28471 (FCQ).

**BRAZIL. Dist. Fed.**: *Irwin et al.* 12043 (NY, MO). **Mato Grosso do Sul**: *V.J. Pott* 229 (CPAP, CTES); Rondonopolis, *G. Hatschbach* 34061 (CTES). **Minas Gerais**: Ituiutaba, *A. Macedo* 673 (S), 1701 (MO, RB). **Pernambuco**: *E.P. Heringer et al.* 478 (RB, UB); P. *Gomes* 463 (RB). **Rio Grande do Norte**: *A.C. Sarmento 761* (NY, RB). **Rio Grande do Sul**: type of *Ipomoea riograndensis*. **Sergipe**: *R. Simão-Bianchini* 1757 (ASE).

**BOLIVIA. Santa Cruz**: Chiquitos, San José de Chiquitos, *J.R.I. Wood et al.* 22862 (HSB, K, LPB, USZ); Germán Busch, Rincón del Tigre, *J.R.I. Wood et al.* 27269 (K, LPB, USZ); ibid., near Puerto Suárez, *J.R.I. Wood & D. Villarroel* 25516 (K, LPB, UB, USZ). **Tarija**: Gran Chaco, near Palos Blancos, *J.R.I. Wood et al.* 27617 (OXF, LPB, USZ).

### 61b. Ipomoea megapotamica subsp. velutina

Taxon classificationPlantaeSolanalesConvolvulaceae

J.R.I. Wood & Scotland, Kew Bull. 72 (10): 13. 2017. (Wood and Scotland 2017b: 13)

Ipomoea nyctaginea var. cordifolia Choisy in A.P. de Candolle, Prodr. 9: 369. 1845. (Choisy 1845: 369).

#### Type.

BRAZIL. Pernambuco. Tapera, *B. Pickel* 3037 (holotype RB, isotypes NY, P).

**Diagnosis.** Leaves adpressed pilose on the abaxial surface; sepals usually only 5–6 mm long.

#### Distribution.

The principal variety in NE Brazil and the only variety in Venezuela. **BRAZIL. Alagoas**: Pão de Açucar, *Lyra-Lemos et al.* 6889 (RB). **Ceará**: Planalto de de Ibíapaba, *Figueirido* 574 (RB). **Maranhão**: *P. Martins* 18/4/79 (RB). **Paraíba**: *Coêlho de Moraes* 2126 (MO, US). **Pernambuco**: Serra Talhada, *E.P. Heringer et al.* s.n. (RB). **Piauí**: *Rizzini* s.n.12/4/74 (RB); Caracol, P.N. Serra das Confusões, *G. Martinelli et al.* 16358 (RB).

**VENEZUELA.** Sine data: *Moritz* 497. **Cojedes**: Las Peonías, *Delascio* 3401 (FTG). **Guarico**: Mesa de el Sombrero, *H. Pittier* 12486 (US). **Monagas**: Mun. Freitas, *Fernández et al.* 9612 (US). **Portuguesa**: Araure, orillas del Río Auro, *G. Aymard & Ortega* 3078 (NY).

#### Note.

*Ipomoea megapotamica* is, usually recognisable by the much-branched but clearly cymose structure of the inflorescence and the sepals with distinct dark glands near their base. It differs from *Ipomoea hieronymi* in the shorter sepals and distinctly branched, compound inflorescences. The sepals, pedicels and, sometimes, the leaves are gland-dotted. This species is also close to *Ipomoea opulifolia* but it is almost always distinguished easily by the entire (rarely very shallowly lobed) leaves which, in subsp. megapotamica, are relatively small and sparsely pubescent beneath.

### 62. Ipomoea decipiens

Taxon classificationPlantaeSolanalesConvolvulaceae

Dammer, Bot. Jahrb. Syst. 23, beiheft 57: 40. 1897. (Dammer 1897: 40)

#### Type.

BRAZIL. Minas Gerais, Congonhas do Campo, *A.F.M. Glaziou* 13100 (holotype B†, photo F, isotypes G, K, P, R).

#### Description.

Twining perennial herb of unknown height, stems thinly pubescent to glabrous. Leaves petiolate, 4–13 × 4–10.5 cm, broadly ovate, cordate, acute or subacute and apiculate, adaxially glabrous, abaxially paler, glabrous, thinly pubescent or puberulent on the veins only; petioles 2–9 cm, glabrous below, puberulent upwards. Inflorescence of pedunculate, many-flowered, lax, compound cymes; peduncles 2.5–9 cm, glabrous; bracteoles caducous, not seen; secondary peduncles 0.5–2.5 cm, spreading at right angles to the peduncle; tertiary peduncles 0.5–1.5 cm; pedicels 7–13 mm long, pubecent; sepals slightly unequal, outer 6–7 × 3–4 mm, ovate, obtuse to rounded, thinly pubescent, inner 7–11 × 6–7 mm, obovate, rounded, nearly completely scarious, glabrous; corolla 5.5–6.5 cm long, pink, pubescent, funnel-shaped, limb 4–4.5 cm diam., unlobed, midpetaline bands ending in a point. Capsules and seeds unknown.

#### Distribution.

A rare species of Caatinga in the the Brazilian planalto.

**BRAZIL. Minas Gerais**: type collection. **Bahia**: Rodovia BR-116, 34 km N de Poções en trecho a Jequié, *S.A. Mori et al*. 9540 (CEPEC, NY).

#### Note.

Obviously part of the *Ipomoea megapotamica* complex differing principally in the obtuse outer sepals and rounded scarious inner sepals. The subtruncate base of the calyx and sparse indumentum should also be noted.

### 63. Ipomoea opulifolia

Taxon classificationPlantaeSolanalesConvolvulaceae

Rusby, Bull. Torrey Bot. Club 26: 150. 1899. (Rusby 1899: 150)

#### Type.

BOLIVIA. *M. Bang* 2506 (lectotype NY00319206, designated here; isolectotype US).

#### Description.

Vigorous twining species 3–4 m high, stems relatively stout, adpressed pilose; rootstock large tuberous. Leaves petiolate, 5–14 × 4–16 cm, 3-lobed to about half way, apex shortly acuminate and mucronate, base broadly cuneate to subtruncate to weakly cordate with rounded auricles, central lobe slightly narrowed to base, adaxially punctate with hair bases and scattered hairs, abaxially softly adpressed silvery-grey pilose, usually gland-dotted; petioles 2–11 cm, pubescent. Inflorescence of lax pedunculate, axillary cymes; peduncles 2–10 cm, densely pubescent; bracteoles 2 mm, scale-like, silvery-pilose, caducous; secondary peduncles 1.5 cm; pedicels 7–8 mm, densely silvery-pilose; sepals slightly unequal, sericeous, outer 10–11 × 4–6 mm, ovate, acute, grey-sericeous, the inner sepals c. 6 mm wide. oblong-elliptic, rounded to truncate, the margin scarious and glabrous; corolla 7–8 cm long, funnel-shaped, mauve, sericeous, limb c. 4 cm diam. Capsules and seeds not known.

#### Illustration.

Figures 15E, 42.

**Figure 42.**
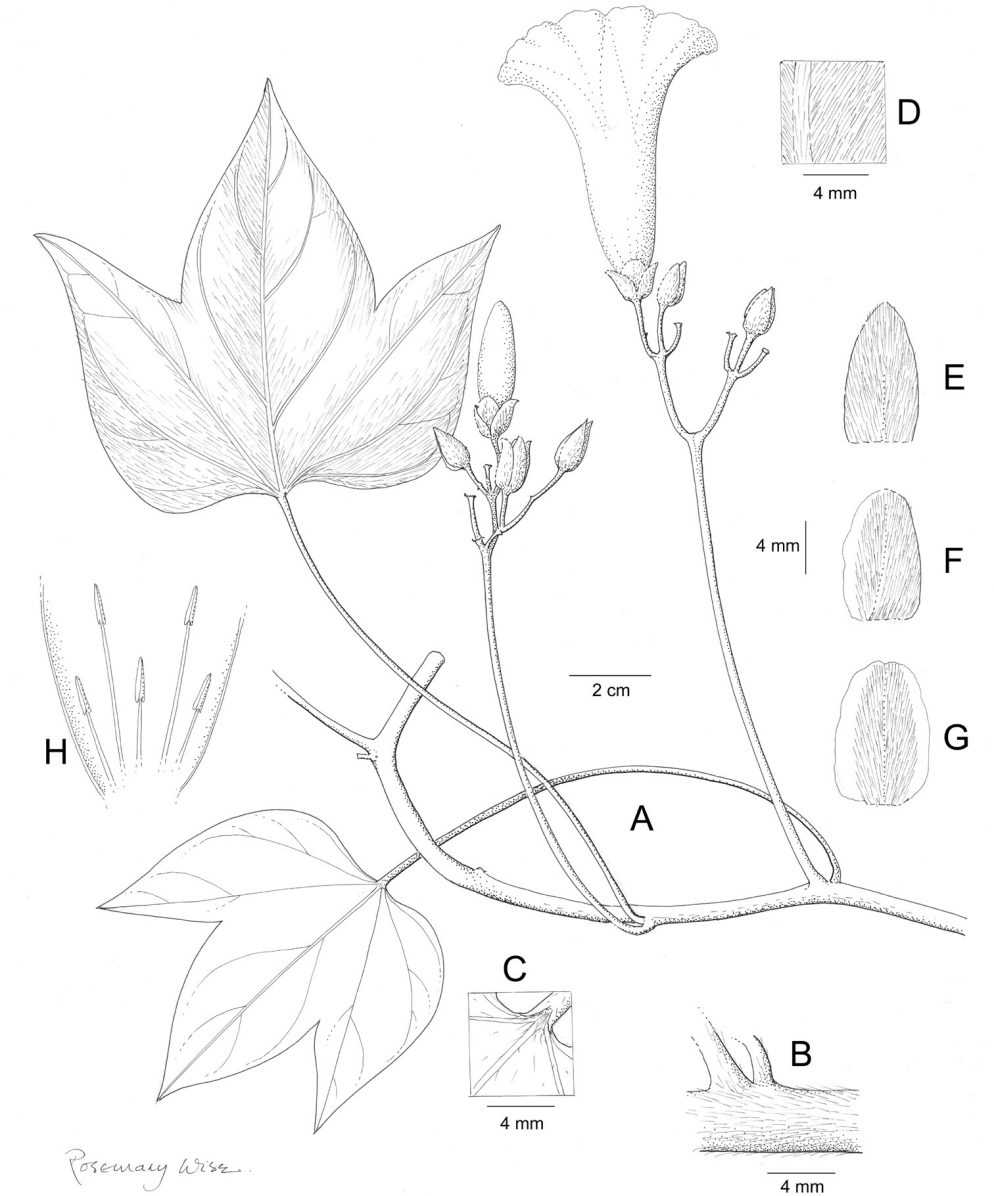
*Ipomoea opulifolia*. **A** habit **B** stem showing indumentum **C** adaxial leaf surface **D** abaxial leaf surface **E** outer sepal **F** middle sepal **G** inner sepal **H** corolla opened out to show stamens **J** fruiting inflorescence. Drawn by Rosemary Wise from *Wood* 16278.

#### Distribution.

Endemic to NW Bolivia. This is a local species of moist forest and forest relics in the Andean foothills below 700 m.

**BOLIVIA. Beni**: Ballivián, east of Puente Quiquebey, *J.R.I. Wood* 16278 (HSB, K, LPB, USZ); Marbán: San Pablo, *J.P. Coulleri et al.* 166 (CTES); Cercado, T.C.O. Ibiato, *M. Martinez* 9 (USZ); Yacuma, Est. Biológica del Beni, *E. Gutiérrez et al.* 1567 (FTG, MO, USZ). **Cochabamba**: Chapare, P.N. Isiboro-Sécure, *E. Thomas* 699 (BOLV, LPB, K). **La Paz**: Iturralde, Alto Madidi, *A. Gentry & S. Estensoro* 70653 (LPB, MO, SP); San Buenaventura, *A. Fuentes* 4387 (BOLV, LPB, MO, USZ); Larecaja, Guanay, *H.H. Rusby* 1999 (MICH, NY).

#### Typification.

The syntype from Guanay (NY 00319205) is labelled as holotype but this is not correct. We have selected *Bang* 2506 (NY00319206) as lectotype as Rusby clearly states that the description of the flowering plant is based on this collection and it is, in any case, a much better specimen.

#### Note.

This species is morphologically close to *Ipomoea megapotamica* differing by the acutely 3-lobed leaves and the silvery-grey appressed pilose abaxial surface of the leaves. *Coulleri et al.* 166 differs in the spreading indumentum of the sepals and the more persistent bracteoles, so approaching *Ipomoea macarenensis*.

### 64. Ipomoea macarenensis

Taxon classificationPlantaeSolanalesConvolvulaceae

J.R.I. Wood & Scotland, Kew Bull. 72 (10): 6. 2017. (Wood and Scotland 2017b: 6)

#### Type.

COLOMBIA. Meta, El Mico airstrip, last savannah before Río Guajar, 6 Nov. 1949, *W.R. Philipson, J.M. Idrobo & A. Fernández* 1322 (holotype BM001191225, isotypes COL, US).

#### Description.

Climbing perennial herb of unknown height; stems densely pubescent to subtomentose. Leaves petiolate, 2.5–5.5 × 2.8–5 cm, ovate, entire or shallowly 2–3-lobed, apex acute, mucronate, base truncate to shallowly cordate, adaxially green, thinly adpressed-pilose, abaxially densely silvery-tomentose with rather long appressed hairs; petioles 2–3.8 cm, pubescent. Inflorescence of few-flowered axillary cymes; peduncles 1.2–5 cm; bracteoles 12–20 × 1–7 mm, linear to oblanceolate-narrowly elliptic, foliose, variable in size and shape; secondary peduncles 6 mm; pedicels 5–6 mm; sepals subequal, densely appressed-pilose, outer 11–14 × 7–8 mm, ovate, acute, inner similar but obtuse and margins scarious, glabrous; corolla 5.5–6 cm long, white with pale pink centre, pubescent, funnel-shaped; limb c. 4 cm diam., entire; longer filaments c. 25 mm, shorter 12–14 m. Capsules and seeds not seen.

#### Illustration.

Figure 43.

**Figure 43.**
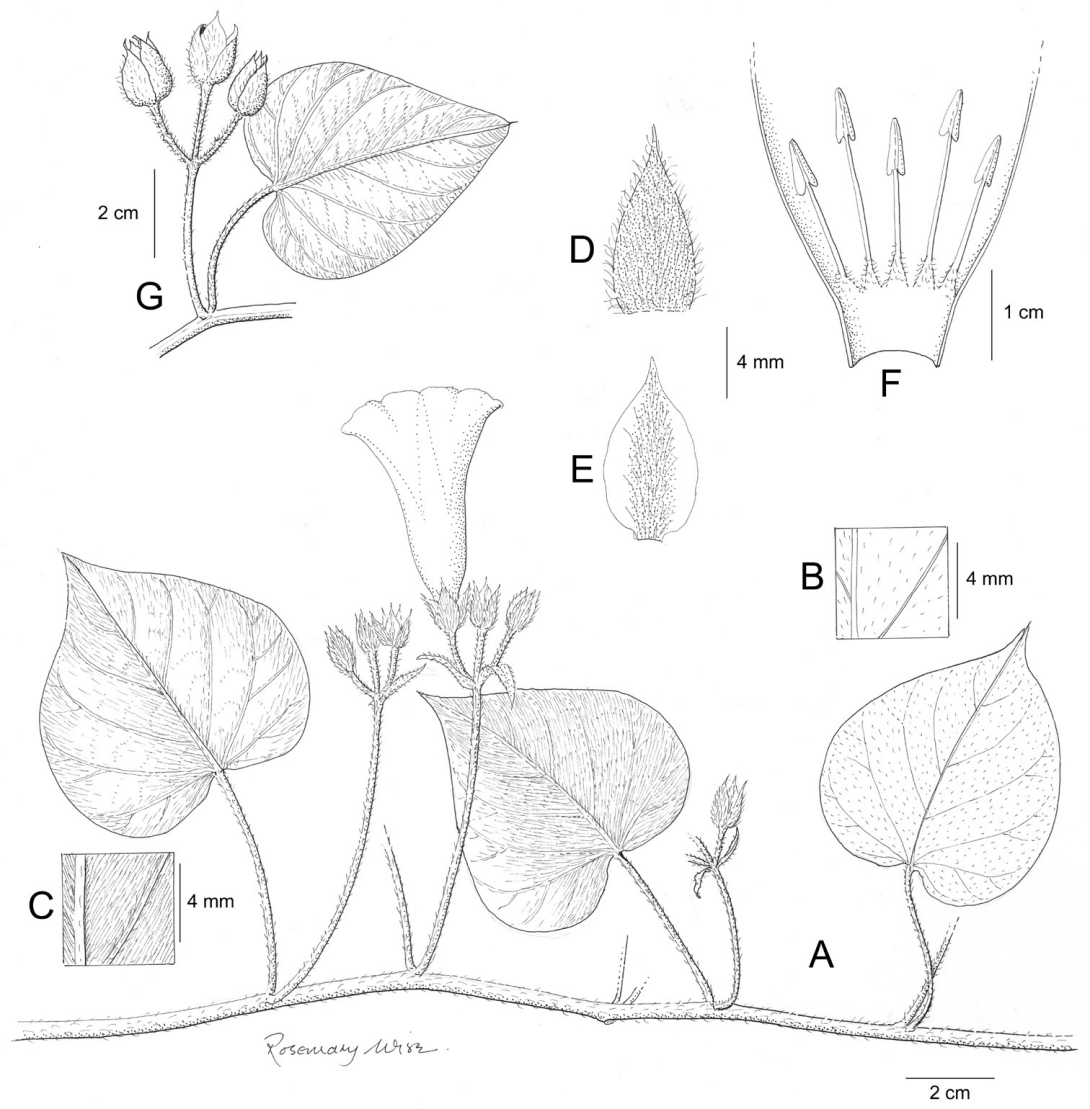
*Ipomoea macarensis***A** habit **B** adaxial leaf surface **C** abaxial leaf surface **D** outer sepal **E** inner sepal **F** corolla opened out to show stamens **G** fruiting inflorescence. Drawn by Rosemary Wise **A–C** from *W. R. Philipson et al.* 1322; **D–G** from *J. Cuatrecasas* 7778.

#### Distribution.

Only known from the plains below the Sierra de Macarena.

**COLOMBIA. Meta**: *J. Cuatrecasas* 7778 (US, COL).

**Notes**. This species has been identified as *Ipomoea sericophylla* Meisn. and it has a very similar leaf indumentum. It is, however, readily distinguished by the much larger sepals (11–14 mm long, not 6–8 mm, larger corolla c. 6 cm long, not 4.5 cm, and the much laxer, fewer-flowered cymes with foliose bracteoles. It is also similar to I. megapotamica subsp. velutina but differs in the indumentum, size and shape of the sepals. It is perhaps closest to *I. opulifolia* but the leaves are unlobed or only shallowly lobed and the sepals distinctly larger. The bracteoles are larger than in all these related species even when of relatively reduced size.

*Lindman* 3189 (S) from Santa Cruz da Barra, Mato Grosso, Brazil may belong to *Ipomoea macarenensis*. The size of the corolla and the sepals is similar but the bracteoles are linear filiform, although persistent, and the leaves are deeply 3-lobed. It may perhaps represent yet another species or some kind of intermediate with *I. opulifolia*.

### 65. Ipomoea sericophylla

Taxon classificationPlantaeSolanalesConvolvulaceae

Meisn. in Martius et al., Fl. Brasil. 7: 260. 1869. (Meisner 1869: 260)

#### Type.

BRAZIL. Minas Gerais, *P. Clausen* [289] (lectotype BR00005837199, designated here; isolectotypes BR, NY01043511P, K, S).

#### Description.

Liana with thick stems. Leaves petiolate, 4–7 × 3.5–6.5 cm, ovate, broadly cuneate to ±truncate, obtuse and apiculate, adaxially green and thinly appressed pilose above, beneath grey-tomentose with long, appressed hairs; petioles 2.5–4.2 cm, densely pubescent. Inflorescence of dense compact pedunculate cymes; peduncles often short, 1–4 cm, usually grey-tomentose; bracteoles 5–10 mm long, filiform, grey-tomentose, somewhat persistent; secondary peduncles 0.5–1 cm; pedicels 3–8 mm, rather short; sepals subequal, 9–10 mm, oblong-lanceolate, acute, silvery-sericeous; corolla 6.5–7 cm long, pink, adpressed sericeous with long hairs. Capsules glabrous; seeds glabrous, shiny blackish-brown with long silky hairs on margins.

#### Distribution.

Endemic to the cerrados of the planalto of Brazil at c. 700–1000 m.

**BRAZIL.** Sin. loc., *W.J. Burchell* 6692 (K). **Goiás**: 20 km S. of Cavalcante, *H.S. Irwin et al.* 24228 (FTG, MO, NY); Niquelândia, *H.S. Irwin et al.* 34998 (FTG, NY); Corumbá de Goiás, *E.P. Heringer et al.* 17003 (IBGE, US); Luziania, *E.P. Heringer et al.* 17768 (IBGE, FTG); *B. Walter* 1329 (CEN, RB); Minaçu, *T.B. Cavalcanti* 1076 (RB). **Minas Gerais**: S.E. of Paracatú, *H.S. Irwin et al.* 26192 (NY, FTG, MO); Serra Bom Jardim, *A. Macedo* 5800 (US).

#### Typification.

We have selected the Clausen collection at BR as the lectotype and this is duplicated in various other herbaria. We specifically exclude NY00319222 as it appears to be a mixed collection with *Ipomoea sericophylla* near the top of the sheet and another species below. The exceptionally large corolla pasted to this sheet may be from a third species, such as *I. cearensis*.

#### Note.

*Ipomoea sericophylla* is a poorly understood and possibly poorly defined species. As understood here and illustrated in Plate 98 of Meisner (1869), it is characterised by its relatively short, compact cymes with persistent filiform bracteoles. Unlike *Ipomoea megapotamica* and its allies, glands are apparently absent from the sepals, which are acute, not mucronate, and strongly tomentose and the peduncles are short so the inflorescence is characteristically shorter than the leaves.

### 66. Ipomoea walteri

Taxon classificationPlantaeSolanalesConvolvulaceae

J.R.I. Wood & Scotland, Phytokeys 88: 34. 2017. (Wood et al. 2017d: 34)

#### Type.

BRAZIL. Goiás: Colinas do Sul, arredores da Serra de Jipe, 500 m, *B.M.T. Walter et al.* 4734 (CEN).

#### Description.

Liana of unknown height, stems thinly pubescent; leaves petiolate, 3–5 × 3.5–5.5 cm, ovate, apex obtuse and long-cuspidate (mucro c. 3–4 mm), base cordate with rounded auricles, adaxially very sparsely pubescent to subglabrous, abaxially grey-tomentose, gland-dotted; petioles 2.5–3.5 cm. Inflorescence of long-pedunculate lax axillary cymes; peduncles 7–11 cm; bracteoles caducous, not seen; secondary peduncles 0.3–2.2 cm; tertiary peduncles c. 10 mm; pedicels 4–5 mm; sepals unequal, outer 11–12 × 8–9 mm, obovate-elliptic, rounded, thinly tomentellous; inner 8–9 × 6 mm, densely tomentose in central part but with broad, glabrous scarious margins; corolla 5–5 cm long, appearing broadly tubular but not fully open, probably funnel-shaped when open, pale pink. Capsules and seeds unknown.

#### Illustration.

Figure 44.

**Figure 44.**
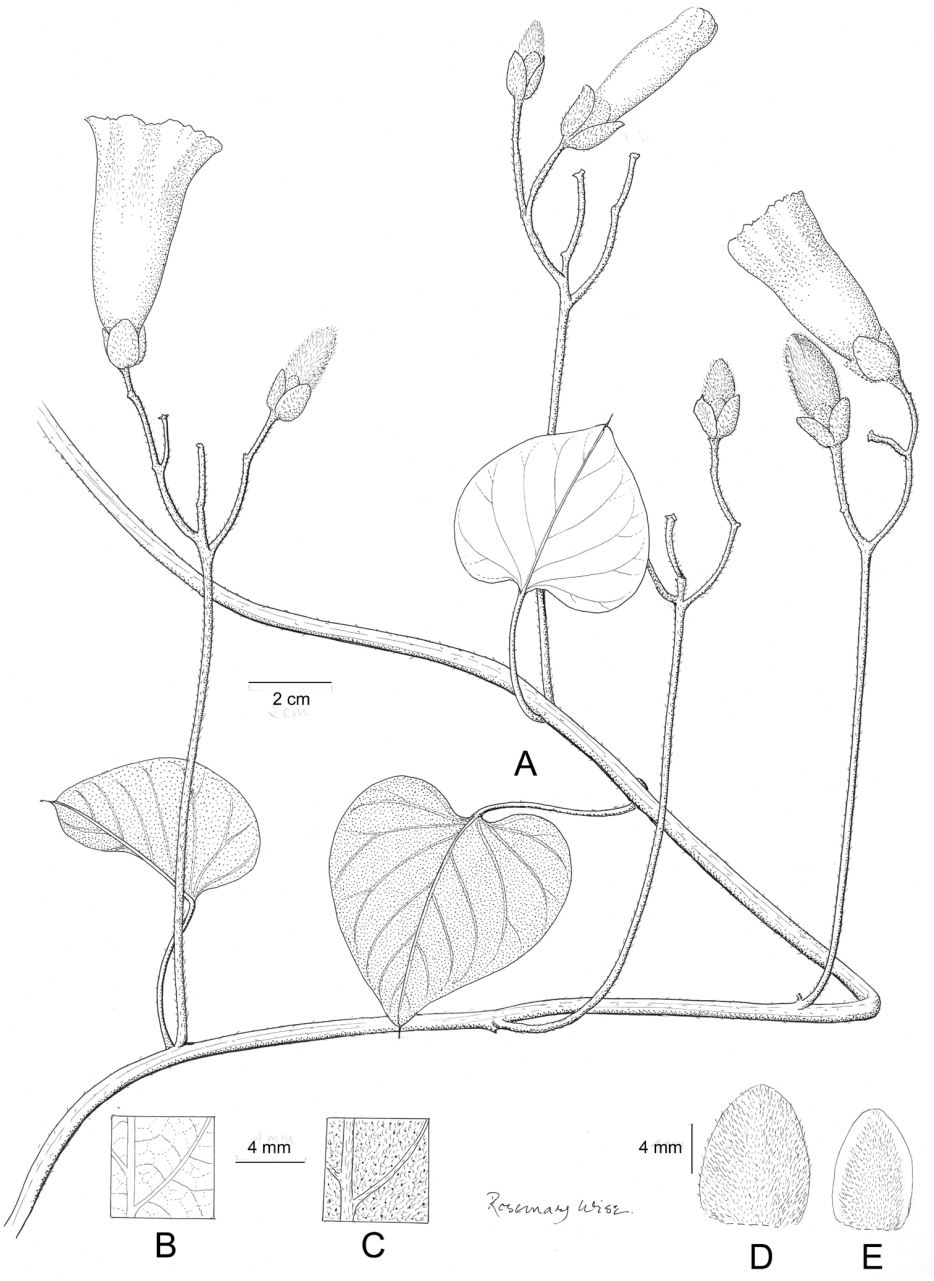
*Ipomoea walteri***A** habit **B** adaxial leaf surface **C** abaxial leaf surface **D** outer sepals **E** inner sepal. Drawn by Rosemary Wise from *Walter et al.* 4734.

#### Distribution.

Cerrados of central Brazil but only known from the type collection.

**BRAZIL. Goiás**: the type collection.

#### Note.

*Ipomoea walteri* appears close to *Ipomoea sericophylla* but is distinct because of the long-pedunculate lax inflorescence, adaxially nearly glabrous leaves and relatively large sepals. The strongly cuspidate leaves with a distinct apical mucro c. 3 mm long are particularly distinct and are only matched in a few other unrelated species, especially *I. daturiflora*. Also somewhat unusual are the inner sepals, which are noticeably shorter than the outer.

### 67. Ipomoea mucronifolia

Taxon classificationPlantaeSolanalesConvolvulaceae

J.R.I. Wood & Scotland, Kew Bull. 50 (31): 46. 2015. (Wood et al. 2015: 46)

#### Type.

BOLIVIA. Santa Cruz, Prov. Chiquitos, entre Limoncito y Roboré, *J.R.I. Wood & P. Pozo* 25064 (holotype USZ, isotypes K, LPB).

#### Description.

Trailing perennial; stem densely villous, glabrescent when old. Leaves petiolate, mostly 4–8 × 4–8 cm, shallowly cordate with the base broadly cuneate, auricles rounded, 3(–5)-lobed, the 4^th^ and 5^th^ lobes often poorly developed, lobes broadly ovate, elliptic or obovate, often overlapping, acute or obtuse and strongly mucronate wth mucro 2–3 mm long, densely grey appressed-pilose on both surfaces but abaxially paler; petioles 2.5–7 cm, softly pilose. Inflorescence of pedunculate, (2–)5-flowered, axillary cymes; peduncles 5.5–14 cm, pilose; bracteoles 3–7 × 1 mm, lanceolate, scarious, pilose, somewhat persistent; secondary peduncles 0.6–1.8 cm; pedicels 0.6–1.2 cm, pilose; sepals minutely gland-dotted on the exterior, unequal, outer 12–14 × 4 mm, broadly lanceolate, shortly acuminate, adpressed-pilose; inner 13–14 × 5 mm, oblong-obovate, rounded to acute, the central region pubescent, marginal part broad, glabrous, margin sparsely ciliate; corolla 5.5–6 cm long, pink, funnel-shaped, the limb c. 5 cm diam., distinctly lobed with ovate acute lobes, densely pilose in bud but somewhat glabrescent, the midpetaline bands thinly pilose on open corollas. Capsules and seeds not seen.

#### Illustration.

Figure 45.

**Figure 45.**
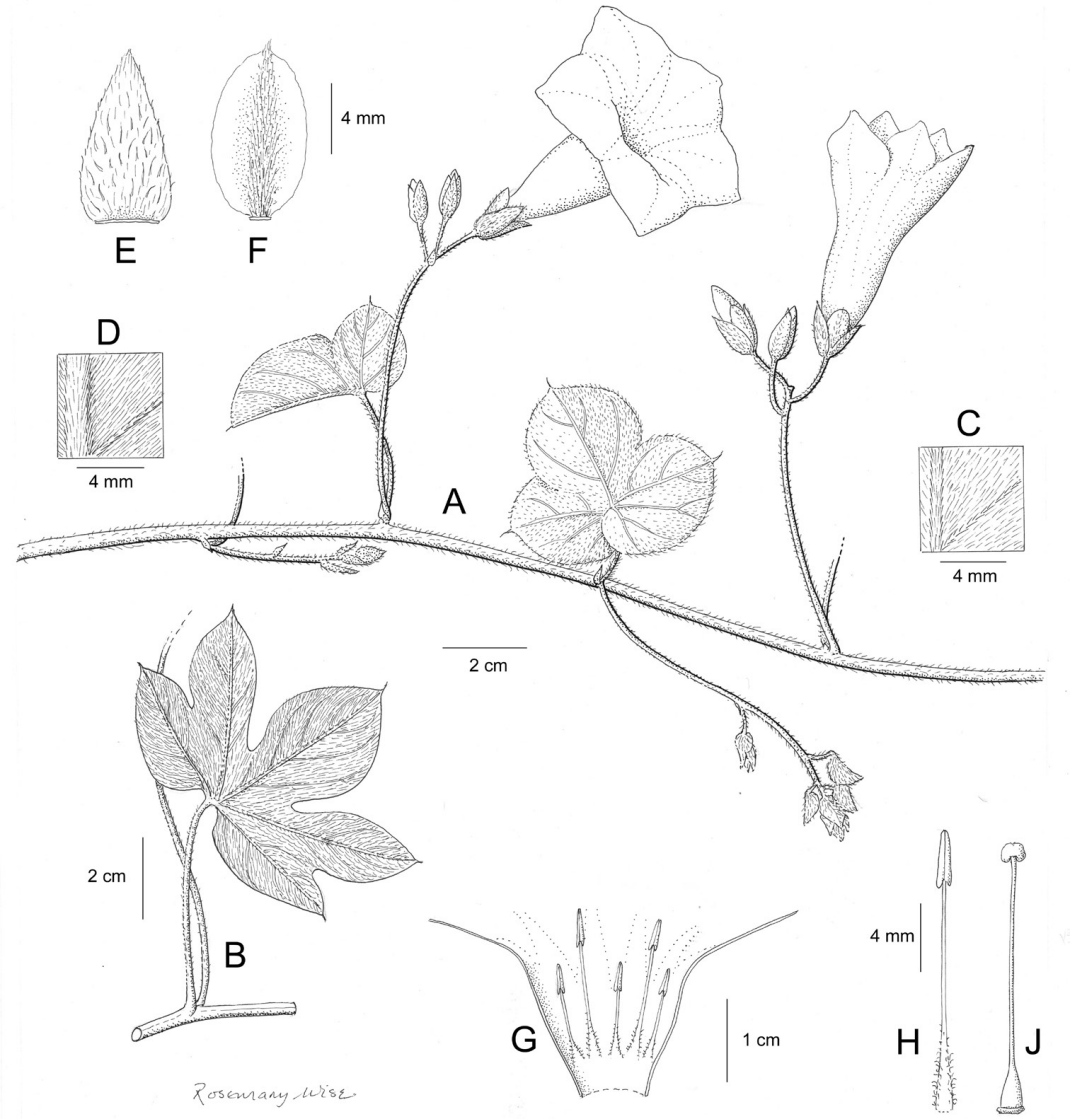
*Ipomoea mucronifolia*. **A** habit **B** leaf **C** adaxial leaf surface **D** abaxial leaf surface **E** outer sepal **F** inner sepal **G** corolla opened out to show stamens **H** stamen **J** ovary and style. Drawn by Rosemary Wise **A, C–J** from *Wood & Pozo* 25064; **B** from *Fuentes & Navarro* 2319.

#### Distribution.

A species of the northern Chaco growing in somewhat degraded bushland in Bolivia and the extreme north of Paraguay.

**PARAGUAY. Alto Paraguay**: Madrejón, *F. Mereles* 6696 (FCQ). **Boquerón**: Fortin Platanillos *F. Mereles & R. Degen 6193* (CTES).

**BOLIVIA. Santa Cruz**: Cordillera, P.N. Kaa-Iya, *A. Fuentes & G. Navarro* 2319 (CTES, MO, NY, USZ).

#### Note.

*Ipomoea mucronifolia* is somewhat similar to *Ipomoea pseudocalystegia* in its palmately-lobed, softly hirsute, strongly mucronate leaves, combined with the lanceolate, acuminate sepals. It differs in the smaller, more deeply divided, less silvery leaves, the inflorescence of several-flowered cymes and the shorter deciduous bracteoles (up to 7 mm long, not > 20 mm).

### 68. Ipomoea pseudocalystegia

Taxon classificationPlantaeSolanalesConvolvulaceae

Hassl., Repert. Spec. Nov. 9: 151. 1911. (Hassler 1911: 151)

#### Type.

PARAGUAY. Sierra de Amambay, *Rojas* in *Hassler* 10723 (holotype G00175048, isotypes BM, K).

#### Description.

Trailing perennial, the whole plant densely sericeous-pilose, often silvery in colour; rootstock unknown but probably woody. Leaves petiolate, 3.5–13 × 2.5–15 cm, usually weakly 3–5-palmately lobed,(sometimes entire, broadly ovate), base broadly cuneate to subtruncate, lobes oblong-deltoid, the laterals often poorly developed, apex obtuse and mucronate; petioles 2–12 cm. Inflorescence of long-pedunculate solitary or clustered, axillary flowers; peduncles 5–20 cm; bracteoles 1.5–2.5 cm long, usually filiform but sometimes lanceolate (to 4 mm wide) or, even, as in type, foliose, spathulate-elliptic, reaching 5 × 1.5 cm; pedicels 1–8 mm, the hairs more patent than on peduncle; sepals lanceolate, long-acuminate, 18–25 × 3–5 mm; corolla 7–10 cm long, pink, funnel-shaped, pilose, limb undulate, 3–4 cm diam. Capsules and seeds not seen.

#### Illustration.

Figure 46.

**Figure 46.**
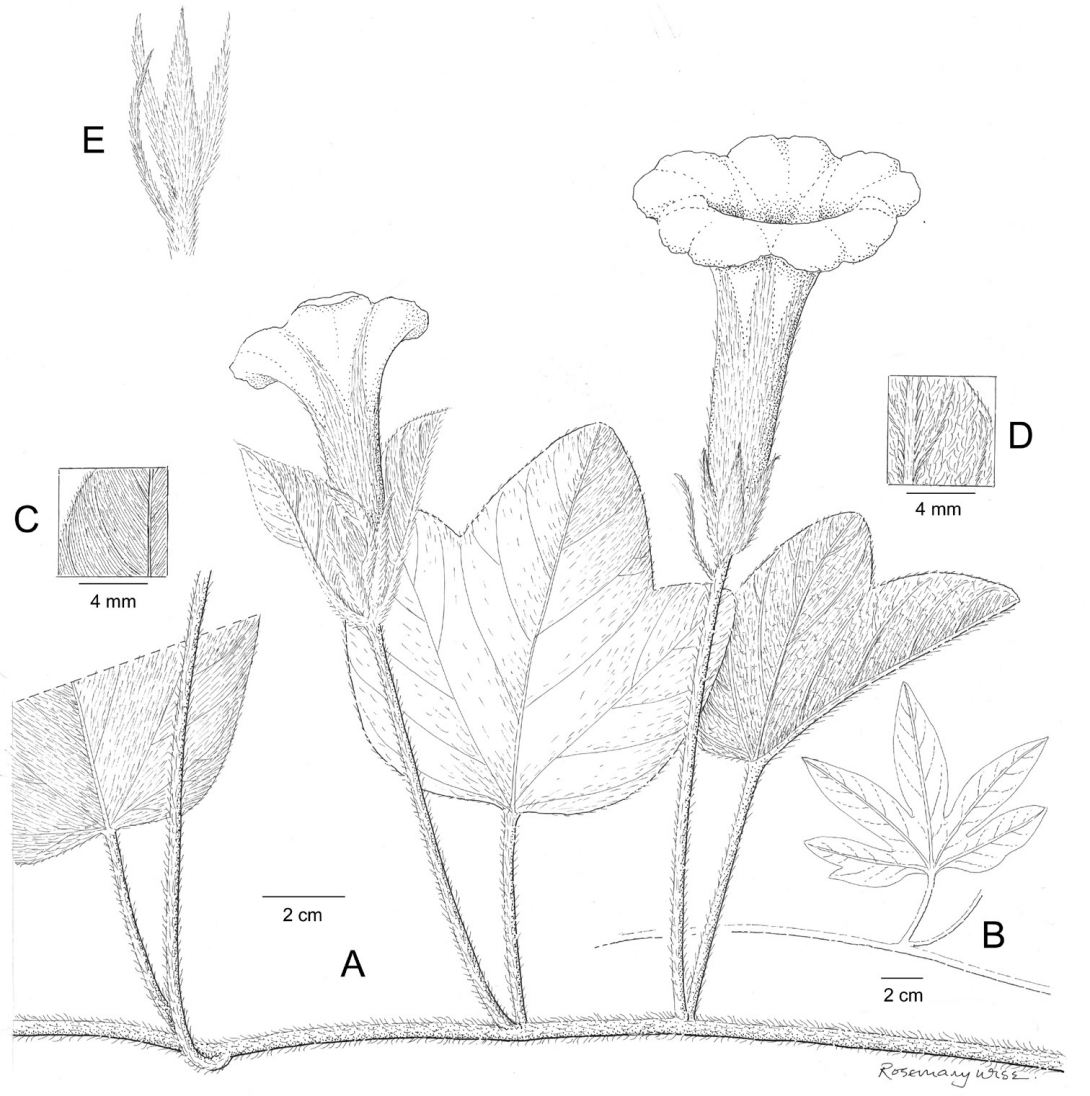
*Ipomoea pseudocalystegia*. **A** habit **B** variation in leaf shape **C** adaxial leaf surface **D** abaxial leaf surface **E** bracteole and calyx. Drawn by Rosemary Wise **A, C–E** from *Hassler* 10723; **B** from *Hassler* 10620.

#### Distribution.

A local species endemic to the Sierra de Amambay in Paraguay and neighbouring parts of Rio Grande do Sul in Brazil, apparently always growing in cerrado. **PARAGUAY. Amambay**: *Rojas* in *Hassler* 10620 (BM); *K. Mizoguchi & T. Sano* 1139 (MO); Chiriguelo, *A. Schinini & M. Dematteis* 33482 (CTES, FCQ), 33647 (FCQ, CTES); *Krapovickas et al.* 45907 (CTES, K); Cerro Corá, *Krapovickas & Cristóbal* 44958 (CTES, F, FCQ); ibid., *N. Soria* 5740 (CTES, FCQ); ibid., *N. Soria & E. Zardini* 1952 (FCQ). **San Pedro**: Rancho Laguna Blanca, *F. González & M.J. López* 757 (FCQ), 817 (FCQ), Yaguarete Sustainable Forest, *E. Zardini & L. Guerrero* 43282 (MO, PY).

**BRAZIL. Rio Grande do Sul**: Pacari, Mun. Ponta Porã, *G. Hatschbach* 45924 (MBM).

#### Note.

*Hassler* 5009 (NY, F, G, K, P) from Canindeyú (Nandurucay, Sierra de Maracayu), differs slightly in its less silvery appearance with leaf lobes oblong-lanceolate in shape. *Krapovickas & Cristóbal* 44958 (CTES, F, FCQ) from P.N. Cerro Corá, Amambay, appears identical to *Ipomoea pseudocalystegia* in its inflorescence but the leaves are 3-lobed to half way, the central lobe broadly elliptic and base truncate and very shortly cuneate onto the petiole. Further collections are needed to elucidate these forms.

### 69. Ipomoea argentinica

Taxon classificationPlantaeSolanalesConvolvulaceae

Peter, Nat. Pflanzenfam.4 (3a): 30. 1897 [pub.1891]. (Peter 1891: 30)

Mouroucoa juramenti Kuntze, Revis. Gen. Pl. 3(2): 217. 1898. (Kuntze 1898: 217). Type. ARGENTINA. Salta, pasaje del Río Juramento, *Lorentz & Hieronymus* 285 (holotype B†, isotypes GOET, CORD, S, US).
Argyreia juramenti (Kuntze) K. Schum., Bot. Jahrsber. (Just) 26 (1): 382. 1900. (Schumann 1900: 382)
Ipomoea juramenti (Kuntze) O’Donell, Lilloa 14: 177. 1948. (O’Donell 1948a: 177).
Ipomoea lorentzii Kuntze, Revis. Gen. pl. 3(2): 217. 1898. (Kuntze 1898: 217), nom. illeg. superfl. Type. As for Murucoa juramenti Kuntze

#### Type.

ARGENTINA. Salta, pasaje del Río Juramento, *Lorentz & Hieronymus* 285 (lectotype GOET 005548, designated by O’Donell (1959b: 110), isolectotype US).

#### Description.

Twining or, less commonly, trailing perennial, roots with small tubers, stems densely pubescent. Leaves petiolate, mostly 2–8 × 3–10 cm, broadly ovate to suborbicular, shallowly cordate to ±truncate with rounded auricles, apex acute and apiculate, adaxially green and appressed pilose, abaxially grey, tomentose with long, appressed hairs; petioles 1–8 cm. Inflorescence of compact pedunculate cymes; peduncles 4–7(–11) cm, usually grey-tomentellous; bracteoles 1.2–2 × 0.1–0.3 cm long, linear-lanceolate, long-acuminate, grey-tomentose, persistent; secondary peduncles 0.3–4 cm; pedicels 0–10 mm, often very short, tomentellous; sepals subequal, 9–10 × 4–5 mm, broadly lanceolate, acute to acuminate, silvery-sericeous, the inner ovate with scarious, glabrous margins; corolla 5–7 cm long, pale pink, adpressed-pilose, funnel-shaped, limb 3–4 cm diam., undulate to very shallowly lobed. Capsules ovoid, 8–9 × 7 mm, glabrous; seeds 6–7 mm long, long-pilose.

#### Illustration.

**Figure 47.**
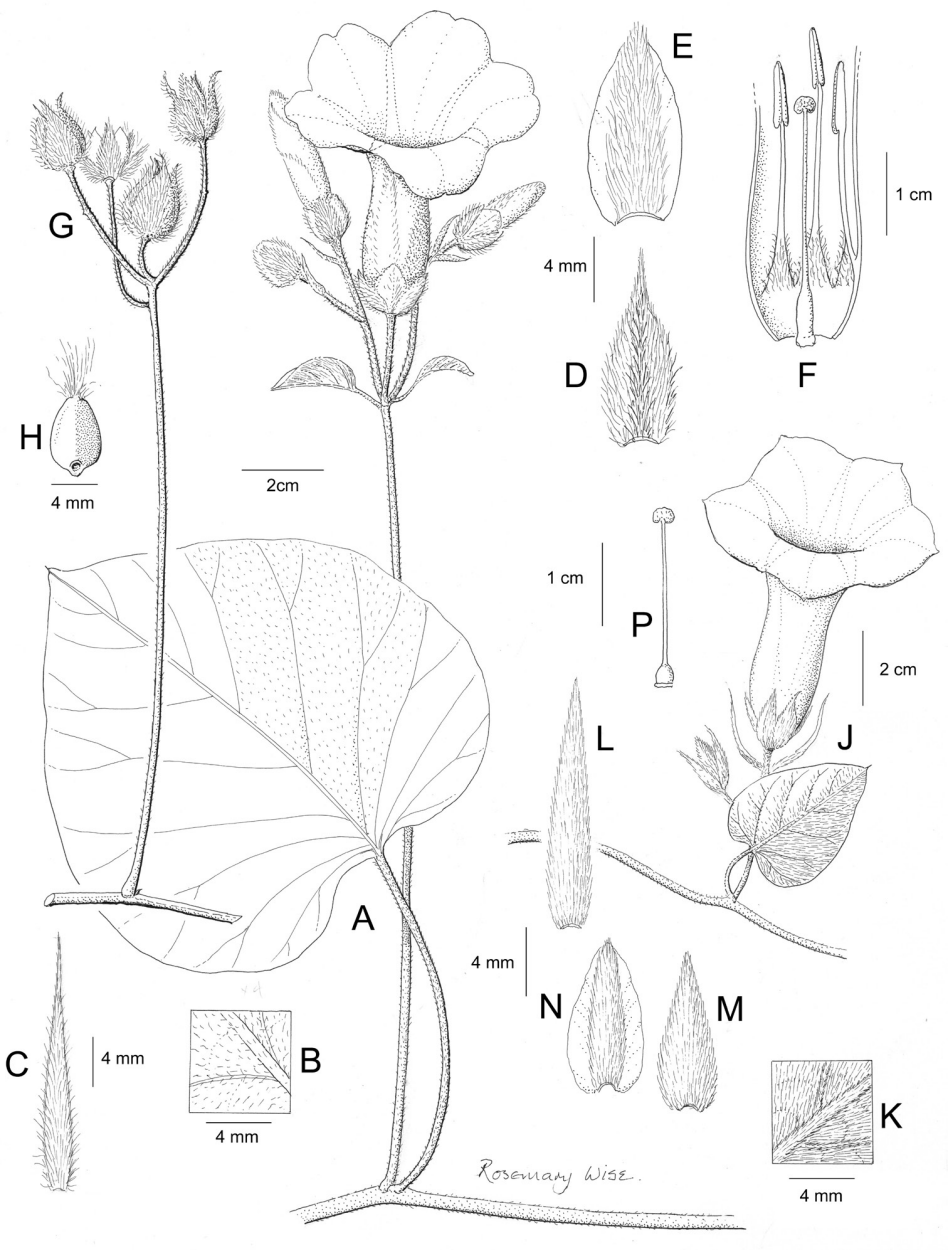
**A–H***Ipomoea longibarbis*. **A** habit **B** abaxial leaf surface **C** bracteole **D** outer sepal **E** inner sepal **F** section of corolla showing 3 stamens, ovary and style **G** fruiting inflorescence **H** seed. **J–P***Ipomoea argentinica***J** inflorescence **K** abaxial leaf surface **L** bracteole **M** outer sepal **N** inner sepal **P** ovary and style. Drawn by Rosemary Wise **A–F** from *Nee & Linneo* 54148; **G, H** from *Killeen et al.* 4199; **J–P** from *Wood & Mamani* 27502.

Figure 47J–P; O’Donell (1959b: 111).

#### Distribution.

A species of the western Chaco in northern Argentina, western Paraguay and southern Bolivia. It is a lowland species of roadsides and disturbed bushy habitats, not found above 600 m. It is particularly common around the city of Santa Cruz in Bolivia. We have not traced the record from Brazil (Austin and Huáman 1996), which seems improbable.

**ARGENTINA. Jujuy**: *H.H. Bartlett* 20301 (SI, US); *A. Krapovickas & A. Schinini* 30639 (CTES). **Salta**: *Cabrera et al.* 34479 (SI); *B.B. Simpson* s.n. [20/1/1986] (MO).

**PARAGUAY. Alto Paraguay**: *Perez de Molas & G. Navarro* 9092 (CTES). **Boquerón**: Picada Sirascuas, *F. Mereles & R. Degen* 5476 (FCQ, MO); *D.R. Brunner* 1559 (G, MO, PY); *L. Bernardi* 20268 (G).

**BOLIVIA. Santa Cruz**: Chiquitos, Tres Cruces, *J.R.I. Wood & B. Williams* 27908 (OXF, K, LPB, USZ); Cordillera, *A. Fuentes* 2869 (USZ); Florida, La Angostura, *J.R.I. Wood et al.* 24101 (K, LPB, UB, USZ); Ibañez, *M. Nee* 49033 (CTES, LPB, MO, NY, OXF, USZ); Ichilo, *J. Steinbach* 1272 (LIL); Sara, La Bélgica, *M. Nee & M.A. Sundue* 52222 (LPB, NY, MO, USZ); Velasco, Santa Rosa de la Roca, *J.R.I. Wood et al*. 27791 (OXF, K, LPB, USZ). **Tarija**: Gran Chaco. *Abrahamczck* s.n. (LPB).

#### Note.

Distinguished from all similar species (*Ipomoea hieronymi*, *I. longibarbis*, *I. megapotamica*) by the very long bracteoles, which persist till corollas have fallen, those immediately below the calyx being particularly persistent. Additionally from *I. longibarbis* it can be separated by the adpressed, ± sericeous hairs of the sepals and from *I. hieronymi* by the more acuminate sepals. It has been treated as a synonym of the Brazilian *Ipomoea sericophylla* (Staples et al. 2012: 674) but differs in the much longer, persistent bracteoles, much less dense abaxial leaf indumentum and longer sepals.

### 70. Ipomoea longibarbis

Taxon classificationPlantaeSolanalesConvolvulaceae

J.R.I. Wood & Scotland, Kew Bull. 50 (31): 56. 2015. (Wood et al. 2015: 56)

#### Type.

BOLIVIA. Santa Cruz, Prov. Cordillera, Pie de la Muela del Diablo, Boyuibe-Camiri, *J.R.I. Wood, D. Villarroel & B. Williams* 27633 (holotype USZ, isotypes OXF, K, LPB).

#### Description.

Robust twining perennial usually 2–5 m high, stems pubescent, somewhat woody. Leaves petiolate, 4–13 × 3–12 cm, ovate, acute or shortly acuminate, terminating in a fine hair point, base shallowly cordate to truncate, margin slightly undulate, adaxially green, thinly adpressed-pubescent, abaxially grey, densely pubescent; petioles 3–10 cm, pubescent. Inflorescence of pedunculate axillary cymes with 1–8 flowers, somewhat dense; peduncle 4.5–26 cm, usually rather stout, pubescent; lower bracteoles 2–2.5 × 0.2–0.8 cm, oblong to oblong-elliptic; secondary peduncles 1.5–4 cm; upper bracteoles 9–18 × 1 mm, linear-lanceolate, terminating in a long fine point, pubescent, somewhat persistent; pedicels 2–10 mm, pubescent; sepals slightly unequal, outer 11–16 × 6–7 mm, ovate, acuminate to a fine point, grey-pilose with conspicuous spreading hairs, inner ovate-elliptic, acute, silvery-pilose with hairs weakly spreading; corolla c. 8 cm long, uniformly pink, silky pubescent on the exterior, funnel-shaped, limb 5 cm diam., shallowly-lobed. Capsules 10–11 × 10 mm, ovoid, glabrous; seeds 7 × 3–3.5 mm, brown, glabrous apart from the 10 mmlong white marginal hairs.

#### Illustration.

Figures 11E, 47A–H.

#### Distribution.

Endemic to Bolivia, growing in dry chaco scrub woodland along the Andean foothills from Camiri south to the Villamontes area, between 500 and 1500 m, largely replacing *Ipomoea argentinica* in this region.

**BOLIVIA. Chuquisaca**: Boeto, Río Grande valley, *J.R.I. Wood* 28128 (LPB, OXF, USZ); Calvo, 80 km E of Boyuibe, *T. Killeen, et al.* 4199 (MO); Siles, between Monteagudo and Rosario del Ingre, *M. Serrano* 2087 (HSB). **Santa Cruz**: Cordillera, SE of Salinas, *M. Nee & I. Linneo* 54148 (MO, NY, USZ); between Camiri and Boyuibe, *M. Mendoza et al.* 2765 (K, LPB, USZ). **Tarija**: Gran Chaco, cañón del Río Pilcomayo, *J.R.I. Wood et al.* 27593 (OXF, K, LPB, USZ); O’Connor, Alta de Soledad, *F. Zenteno et al. 4357* (LPB).

#### Note.

Similar to *Ipomoea argentinica* in habit and leaf indumentum but differing in the laxer inflorescence with longer peduncles, broader outer sepals with conspicuous speading hairs and less persistent bracteoles. Herbarium specimens resemble *Ipomoea rubens* very closely in facies and indumentum but molecular studies indicate there is no close affinity. *Ipomoea longibarbis* is a plant of dry habitats, not stream banks.

### 71. Ipomoea lilloana

Taxon classificationPlantaeSolanalesConvolvulaceae

O’Donell, Lilloa 14: 182. 1948 (O’Donell 1948a: 182)

#### Type.

ARGENTINA. Salta, Dept. Campo Santo, Juramento, *C. O’Donell* 4910 (lectotype LIL001253, designated here; isolectotypes LIL).

#### Description.

Trailing perennial herb, stems sparsely pubescent, somewhat stout and slightly fleshy, up to 2 m long, rootstock stout, often 10 × 10 cm or more, tuberous. Leaves petiolate, 3–7 cm, ovate-deltoid, ovate or suborbicular, obtuse to acute, base broadly cordate to subtruncate, the margin undulate to dentate, white-canescent when young but when mature adaxially dark green and glabrous, abaxially puberulent especially on the veins; petioles 1.5–3.5 cm, thinly pubescent. Inflorescence of shortly pedunculate 1–3-flowered cymes; peduncles 1–7.5 cm; bracteoles not known, fugacious; pedicels 5–10 mm; sepals slightly unequal, 8–10 × 6–7 mm at anthesis but accrescent to 13 mm in fruit, ovate-elliptic, pubescent, outer sepals subacute, inner sepals slightly longer, scarious-margined, obtuse to rounded, sometimes mucronate; corolla 4–7 cm long, funnel-shaped, pink, densely adpressed pilose, limb 5–6.5 cm diam., unlobed. Capsules 1.5 × 0.8 mm, ovoid, acute to rostrate, glabrous; seeds 9 × 4 mm, densely woolly.

**Illustration**: Figure 15D; O’Donell (1959b: 175); Wood et al. (2015: 49, photo).

#### Distribution.

Inter-Andean dry valleys of northern Argentina and southern Bolivia between about 650 m and 2600 m in small, scattered populations on open stony or sandy slopes. **ARGENTINA. Catamarca**: Andalgalá, Cuesta de la Chilca, *G.E. Barboza et al.* s.n. [30/1/2008] (SI). **Salta**: Campo Santo, *C. O’Donell* 5509 (LIL); Virgilio Tedin, *Peirano* s.n. [20/11/1933] (GH, LIL).

**BOLIVIA. Chuquisaca**: Oropeza, near Chuquichuqui, *J.R.I. Wood* 10252 (HSB, K, LPB). **Cochabamba**: Campero, Lagar Pampa, *J.R.I. Wood & M. Mendoza* 21515 (BOLV, OXF, K, LPB, USZ); between Omereque and Totora, *J.R.I. Wood & N.P. Taylor* 22521 (K, LPB). **Santa Cruz**: Caballero, near Abra de Quine, *M. Nee* 46632 (NY, USZ). **Tarija**: Gran Chaco, between Palos Blancos and Yacuiba, *J.R.I. Wood et al.* 28322 (LPB, OXF, USZ); O’Connor, Río Pilaya, *M. Serrano et al*. 7114 (HSB).

#### Note.

Readily identified by its trailing habit, stout stem, thinly pubescent, undulate leaves, pubescent sepals and corolla. Although the leaves are variable in shape, there is no other similar species in the inter-Andean valleys.

### 72. Ipomoea subalata

Taxon classificationPlantaeSolanalesConvolvulaceae

Hassl., Fedde, Repert. Spec. Nov. Regni Veg. 9: 157. 1911. (Hassler 1911: 157)

#### Type.

PARAGUAY. [Concepción], San Luis, *K. Fiebrig* 4485 p.p. (holotype G00175183, isotype G001751820).

#### Description.

Robust perennial reaching 6 m; stems trailing or twining, glabrous, usually slightly winged, the wings muricate. Leaves petiolate, 5–11 × 5–9 cm, ovate, base broadly cordate to subtruncate, apex shortly acuminate, margin entire to undulate, often denticulate near base, adaxially glabrous, abaxially puberulent especially on the veins, sometimes glabrescent; petioles 3–10 cm, slightly winged below. Inflorescence of few-flowered, pedunculate, axillary cymes; peduncles often erect, straight, subglabrous, 3–10 cm; bracteoles minute, lanceolate, caducous; secondary peduncles stout, 2–8 cm; pedicels 1–3 cm; sepals subequal, glabrous to very sparsely pubescent, margins scarious, outer sepals 10–12 × 7–9 mm, broadly ovate or elliptic, obtuse to rounded; inner sepals 11–13 × 8–9 mm, accrescent to 15 mm in fruit elliptic or suborbicular, rounded to retuse (sometimes mucronulate), with broader scarious margins; corolla 9–11 cm long, funnel-shaped, pink, pubescent in bud and at tips of midpetaline bands, limb 4–5 cm diam., weakly lobed; stamens included, slightly unequal, very short, c. 8–10 mm long, style biglobose. Capsules 15–16 × 11–12 mm, ovoid to ellipsoid, very shortly rostrate, glabrous; seeds 6–11 × 3–4 mm, pilose on the angles.

#### Illustration.

Figure 48.

**Figure 48.**
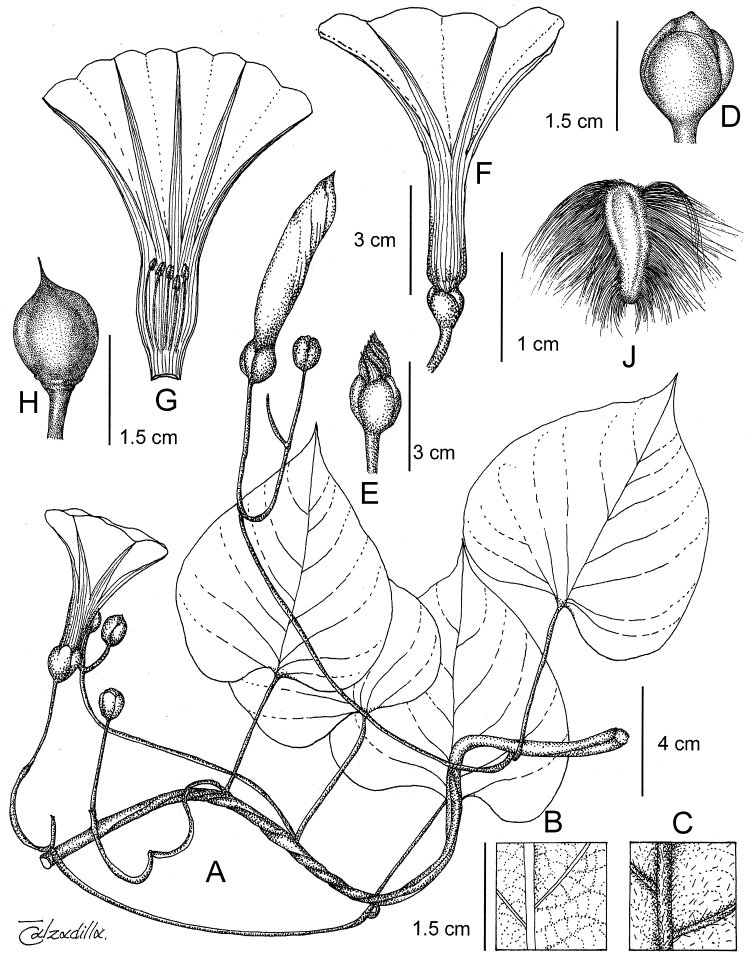
*Ipomoea subalata*. **A** habit **B** adaxial surface of leaf **C** abaxial leaf surface **D** calyx **E** bud **F** corolla **G** corolla opened to show stamens **H** capsule **J** seed. Drawn by Eliana Calzadilla **A–G** from *Wood et al.* 27637; **H–J** from *Wood et al.* 28398.

#### Distribution.

Fairly common in the Andean foothills of the Chaco region of Bolivia below 1000 m, most commonly near the town of Camiri, but with a single collection from northern Paraguay.

**PARAGUAY. Concepción**: the type collection.

**BOLIVIA. Chuquisaca**: Luis Calvo, Serranía de Inca Huasi, *A. Lliully & Portal* 725 (OXF, HSB, MO). **Santa Cruz**: Cordillera, Tatarenda, *R.E. Fries* 1451 (S); Ipati-Lagunillas, *J.R.I. Wood et al.* 27637 (K, USZ, LPB); Abapó-Tatarenda, *J.R.I. Wood et al.* 27590 (K, LPB, USZ); Abapo, *J.R.I. Wood & F. Mamani* 27477 (K, LPB, UZ); Ichilo, Buenavista, *J.R.I. Wood & B. Williams* 27735 (K, LPB, USZ). **Tarija**: Gran Chaco, Palos Blancos, *M. Mendoza et al.* 2662 (K, USZ); Yacunda, carretera hacia Campo Largo, *F. Zenteno et al.* 4454 (CTES, LPB).

#### Note.

This species has been the source of much confusion in Brazil and elsewhere. Wood et al. (2015) treated it as a synonym of *Ipomoea megapotamica* while assigning the Bolivian records to *Ipomoea chondrosepala*. With so many errors on our part and the part of others, we identify the Bolivian material with *I. subalata* with some trepidation. However, the very large pubescent corolla (usually 9–10 cm long), the usually winged stems and the leaves puberulent beneath make it impossible to distinguish Bolivian material from the Paraguayan type. Additionally the habitat is essentially one of the Chaco fringes so the disjunct distribution is not really anomalous.

### 73. Ipomoea jalapa

Taxon classificationPlantaeSolanalesConvolvulaceae

(L.) Pursh, Fl. Amer. Sept. 146. 1813. (Pursh 1813: 146)

Convolvulus jalapa L., Mant. Pl. 43. 1767. (Linnaeus 1767: 43). Type. MEXICO. Veracruz, *McDonald* 2430 (neotype BM000953190, designated by McDonald (1989: 137), isoneotypes K, MEX, TEX).
Batatas jalapa (L.) Choisy, Mém. Soc. Phys. Genève 8(1): 47 [125]. 1838. (Choisy 1838: 47 [125]).
Ipomoea jalapa var. rosea Ker-Gawl., Bot. Reg. 8: t. 621. 1822. (Ker-Gawler 1822: t.621), var. illeg., autonymic variety based on Convolvulus jalapa L.
Ipomoea purshii G. Don, Hort. Brit., ed. 3: 483, 1839. (Sweet 1839: 483). Type based Ipomoea jalapa var. rosea Ker-Gawl.
Ipomoea calantha Griseb., Cat. Pl. Cub. 202. 1866. (Grisebach 1866: 202). Type. CUBA. Bahia Honda, *C. Wright* 3091[1637] (holotype GOET002505, isotypes BM, G, GOET, HAC, K, MO, US).
Ipomoea carrizalia Brandegee, Univ. Calif. Publ. Bot. 4(19): 382. 1913. (Brandegee 1913: 382). Type. MEXICO. Veracruz, Baños de Carrizal, C.A. Purpus 6241 (holotype UC167863, isotypes BM, F, GH, NY, US).
Ipomoea fendleriana Kuntze, Revis. Gen. Pl. 2: 444. 1891. (Kuntze 1891: 444). Type. VENEZUELA. Aragua, Tovar, *A. Fendler* 2083 (lectotype K000612881, designated here).
Ipomoea perichnoa Urban, Symb. Antill. 9: 426. 1925. (Urban 1925: 426). Type. CUBA. Pinar del Río, Guanahacabibes Peninsular, *E.L. Ekman* 18781 (holotype S07-4768, isotypes A, NY, G, HAC–fragment).

#### Type.

Based on *Convolvulus jalapa* L.

#### Description.

Vigorous climbing perennial; stem somewhat woody, pubescent, rootstock a swollen tuber. Leaves petiolate, 6–13 × 5–10 cm, ovate (rarely irregularly lobed to halfway), shortly but finely acuminate, mucronate, base subtruncate to cordate with rounded auricles, glabrous above, abaxially glabrous to tomentellous; petioles 7–9 cm, thinly to densely pubescent. Inflorescence of axillary, pedunculate cymes with mostly 3–5(–10) flowers; peduncles 4–8 cm, relatively stout; bracteoles caducous, not seen; secondary peduncles 1.2–1.8 cm; pedicels 1–3 cm, thickened upwards, puberulent, with tendency to recurve; sepals subequal, outer 8–13 × 4–7 mm, ovate to elliptic, acute or obtuse, uniformly puberulent to tomentellous, occasionally nearly glabrous, inner sepals more obovate to sunorbicular, rounded, the central area more densely hirsute and the wide margins scarious and glabrous; corolla (7–)9–11 cm long, pink, sericeous in bud and on midpetaline bands, narrowly funnel-shaped, limb undulate, c. 6 cm diam. Capsules ovoid, 10–14 × 9–10 mm, glabrous; seeds 8–10 × 4 mm, brown, densely pilose to woolly, hairs white, 5–12 mm long, of different lengths.

#### Illustration.

Acevedo-Rodríguez (2005: 168) as *Ipomoea calantha*.

#### Distribution.

*Ipomoea jalapa* grows at altitudes of up to 1700 m, but often at low altitudes not far from the coast. The distribution is similar to that of *Ipomoea trifida* but *I. jalapa* is nowhere very common and it is unrecorded in a number of countries, where it might be expected to occur including the Dominican Republic, Panama and Guatemala.

**ECUADOR. Guayas**: Chongón, *E. Asplund* 5219 (AAU, K, NY, S, US); Río Daule, *G.W. Harling* 4796 (MO, S). **Loja**: Hac. Banderones, *B. Klitgaard et al.* 531 (AAU, LOJA, NY, QCNE). **Napo**: Misahuallí, F. *Ervik* 36876 (AAU).

**COLOMBIA.***J. Cuatrecasas* 25431(US). **Bolívar**: Isla Mucura, *C.A. Florez* 103 (COL). **VENEZUELA.** sine data, *Moritz* 1242 (BM); *Engstedt* 8/10/1947 (S). **Dist. Fed.**: Macarao, *H. Pittier* 13649 (MO). **Lara**: Jiménez, Represa de Yacambú, *J. Steyermark* 108776 (MO). **Miranda**: Carenero, *J. Steyermark & G. S. Bunting* 102315 (MO). **Yaracuy**: 10 km al N. de Marín, *J. Steyermark* 105352 (MO).

**COSTA RICA.** Puntarenas, *D.F. Austin* 7826 (CR, FTG, MO); ibid., Garabito, *B. Hammel* 19972 (K, MO); *A. Rodríguez & A. Estrada* 371 (K, MO).

**NICARAGUA.** Matagalpa, *W. D. Stevens & R. Riviere* 20937 (MO); Chontales, Tawa, *W. D. Stevens & O.M. Montiel* 35018 (MO).

**HONDURAS.** Comayagua, Chicipates, *C.H. Nelson et al.* 6603 (MO).

**BELIZE.** Cayo, Chiquibul Forest Reserve, *C. Whitefoord* 10522 (BM)

**MEXICO. Campeche**: Calkiní, *E. F. Cabrera* 14402 (IEB, MEXU). **Guanajuato**: El Llanete, *S. Zamudio et al*. 10462 (IEB); Humuchil, *J. Rzedowski* 52937 (IEB). **Hidalgo**: San Cristóbal, *S. Zamudio* 10887 (IEB). **Jalisco**: fide Carranza (2007: 58). **Nuevo León**: Iturbide, *J.C & G.S. Hinton* 21456 (GBH); Aramberri, *P. Carrillo-Reyes & V. Sosa* 4655 (IEB). **Querétaro**: La Mora, *E. Carranza & I. Silva* 6250 (IEB); Cañon del Río Estórax, *S. Zamudio & L. Beltrán* 14194 (IEB). **Quintana Roo**: *O. Télez* 3689 (MEXU). **San Luís Potosí**: *D.F. Austin & F. de la Puente* 7698 (FTG); Rayón, *E. Carranza & E. Pérez* 5637 (IEB). **Sonora**: Cerro Prieto, *A.C. Sanders et al.* 9261 (MO). **Tamaulipas**: *H.H. Bartlett* 11115 (MICH); *M.C. Johnston* 5609 (MICH); *J.N. Labat* 542 (P). **Veracruz**: Baños de Carrizal, *C.A. Purpus* 6241 (MO). **Yucatán**: *C. Vargas* 143 (CICY).

**CUBA. La Habana**: *Bro. León* 6826 (HAC), 14703 (HAC, NY). **Pinar del Río**: *E.L. Ekman* 18176 (HAC, NY, S); *J. Bisse et al.* 51285 (HAJB).

**HAITI.** Massif des Matheux, *E.L. Ekman* H7093 (NY, S), Nouvelle Touraine. *E.L. Ekman* H1471 (S).

**PUERTO RICO.** Coamo, *P. Sintenis* 3128 (K, MO, NY, P, S), 3684 (BM, NY).

**LESSER ANTILLES. U.S. Virgin Islands**: St Croix and St John fide Acevedo-Rodríguez (2005). **Martinique**: *Berlanger* s.n. (P).

#### Notes.

*Ipomoea jalapa* and *I. macrorhiza* are unusual in this large clade as their distribution is centred on the Caribbean rather than South America. It is also highly variable in leaf shape and corolla size, and *ITS* suggests it is polyphyletic. Intensive studies are needed to resolve these uncertainties.

*Ipomoea jalapa* is most likely to be confused with I. carnea subsp. carnea but is distinguished by the longer outer sepals. These are usually < 7 mm long in *I. carnea*. The corolla is also larger. Historically this species has also been confused with *I. macrorhiza*, which is a coastal night-flowering species of the SE United States with white flowers and often 3-lobed leaves.

*Ipomoea perichnoa* is included as a synonym of *I. jalapa*. It differs in the woolly seeds with hairs to 15 mm long covering the whole surface but, in the absence of any other obvious distinguishing character, there seems no good reason to accept *I. perichnoa* as a distinct species.

*Ipomoea jalapa* is quite variable, plants from Haiti and Puerto Rico, for example, have very long stamens, while specimens from the interior of Mexico quite commonly have irregularly lobed leaves and relatively small sepals.

An extract from the roots is used medicinally.

### 74. Ipomoea macrorhiza

Taxon classificationPlantaeSolanalesConvolvulaceae

Michx., Fl. Bor.-Amer. 1: 141. 1803. (Michaux 1803: 141)

Ipomoea jalapa var. macrorhiza (Michx.) Ker-Gawl., Bot. Reg. 8: t. 621. 1822. (Ker-Gawler 1822: t. 621).
Ipomoea michauxii Sweet, Hort. Brit. 288.1826. (Sweet 1826: 288), nom. illeg., superfl.
Modesta macrorhiza (Michx) Raf., Fl. Tel. 4: 76. 1838. (Rafinesque 1838a: 76).
Ipomoea jalapa forma macrorhiza (Michx.) Matuda, Anales Inst. Biol. Univ. Nac. Mex. 35: 51. 1965. (Matuda 1966a: 51).

#### Type.

UNITED STATES. In maritimis Georgiae et Floridae, (lectotype P00625543, designated here).

#### Description.

Vigorous trailing perennial of sea shores; stems puberulent, rootstock a stout tuber. Leaves petiolate, 4–18 × 4.5–17 deltoid in outline, 3-lobed or (less commonly) entire, obtuse to shortly falcate-acuminate, base truncate and then cuneate onto the petiole, margin undulate to serrate, adaxially minutely punctate, thinly pubescent, glabrescent, abaxially grey-tomentellous; petioles 1.5–9.5 cm, pubescent and sometimes muricate. Inflorescence of few-flowered axillary cymes, flowers often solitary; peduncles 1.3–10 cm, tomentose, glabrescent; bracteoles caducous, not seen; secondary peduncles 7–24 mm; pedicels 10–30 mm, thickened upwards; sepals obtuse, sometimes mucronate with a broad point, tomentellous, unequal, outer oblong-lanceolate, 13–15 × 4–5 mm, inner oblong-ovate, 14 –16 × 6–7 mm, the margins glabrous, scarious, strongly accrescent in fruit to 22 × 10 mm; corolla 10–11 cm long, white with a pink throat, tomentellous on mid-petaline bands, tube cylindrical and only slightly widened for c. 5 cm, then abruptly flared and funnel-shaped, limb c. 8 cm diam., apparently entire. Capsules 15–20 × 12–15 mm, ovoid with short persistent style, glabrous; seeds 12 × 5 mm densely lanate with hairs 10–15 mm long.

#### Illustration.

Figure 49.

**Figure 49.**
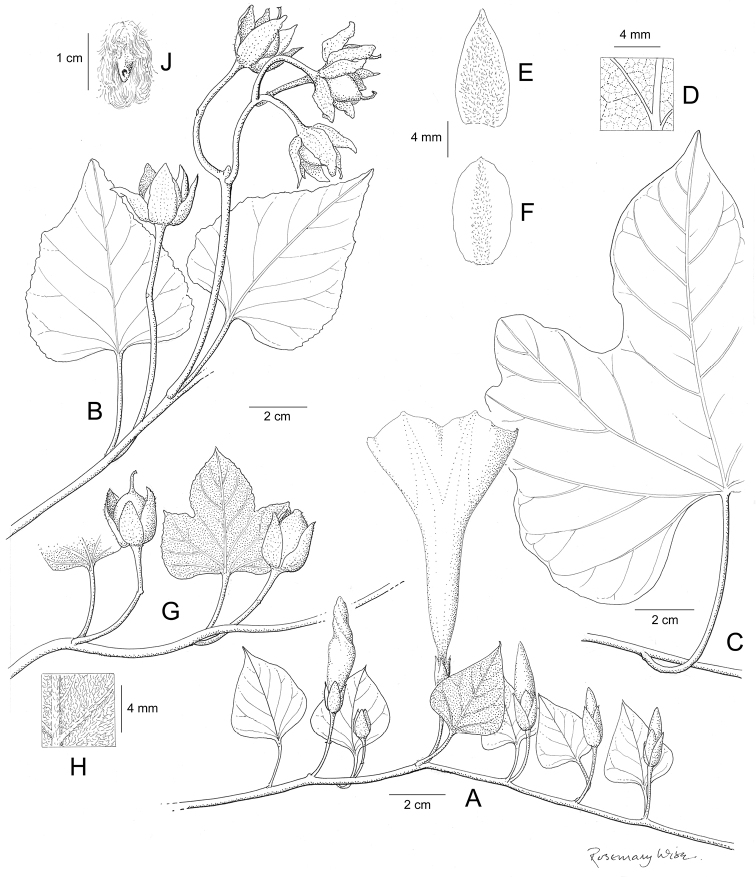
*Ipomoea macrorhiza*. **A** habit **B** habit **C** leaf **D** leaf, abaxial surface **E** outer sepal **F** inner sepal **G** fruiting inflorescence and capsule **H** leaf, abaxial surface **J** seed. Drawn by Rosemary Wise **A, E, F** from *Carey* s.n.; **B** J from *Curtiss* 2165; **C, D** from *Ritchie Bell* 18568; **G, H** from *Genelle & Fleming* 351.

#### Distribution.

Endemic to the south eastern coasts of the USA from North Carolina to Florida and west to Mississippi.

**UNITED STATES. Alabama**: *J.R. McDonald* 9845 (IBE, MO). **Florida**: *Genelle & Fleming* 351 (BM, USF); *F. Rugel* 1845 (BM); *A.H. Curtiss* 2165 (BM, K); *T. Nuttall* (OXF). **Georgia**: *W. Faircloth 5388* (GA). **Mississippi**: Pearl River, sine data (FSU); Jackson, *R.L. Diener* s.n. (MISSA). **North Carolina**: *C. Ritchie Bell* 18568 (UNC, BM). **South Carolina**: *S.W. Leonard* 4321 (UNC, S).

#### Typification.

In designating a lectotype, we have chosen the only original specimen at Paris with a corolla.

#### Note.

A coastal species resembling *Ipomoea jalapa* but differing in the more hirsute, usually 3-lobed leaves and white, usually solitary flowers as well as the distinctive habitat. It is reported to be a night-flowering moth pollinated species (Austin and Huáman 1996).

### 75. Ipomoea leonensis

Taxon classificationPlantaeSolanalesConvolvulaceae

B.L. Rob., Proc. Amer. Acad. Arts 26: 170. 1891. (Robinson 1891: 170)

#### Type.

MEXICO. Nuevo León, Monterrey, *C.G. Pringle* 2840 (holotype GH00054512, isotype VT).

#### Description.

Perennial with woody, tuberous rootstock; stems probably trailing. Leaves petiolate, polymorphic, young leaves up to 7 × 4 cm, ovate-deltoid, obtuse, base very broadly cuneate so some leaves subrhomboid; older leaves up to 12 × 14 cm, digitately 7-lobed to just halfway, the central lobe oblong-elliptic, the inner four oblong, the outermost two ovate with a broad, basal appendage, base subcordate and cuneate onto the petiole, adaxially thinly punctate, abaxially tomentose when young, glabrescent; petioles 2–6 cm, glandular-tuberculate near base of older leaves. Inflorescence of solitary (or paired) pedunculate flowers arising in the axils of foliose 7-partite bracts; peduncles 3–5 cm; bracteoles scale-like, caducous; pedicels 1.5–2 cm; sepals subequal c. 8 5 × 5 mm, ovate, rounded, canescent; corolla 5–7 cm long, pubescent in bud, purple, limb c. 5 cm diam. Capsules and seeds unknown.

#### Distribution.

Endemic to north east Mexico, apparently only known from the type.

**MEXICO. Nuevo León**: type collection.

#### Note.

The shape of the mature leaves is very distinct as is the tuberculate lower part of the petiole.

### 76. Ipomoea rupicola

Taxon classificationPlantaeSolanalesConvolvulaceae

House, Ann. New York Acad. Sci. 18: 230. 1908. (House 1908b: 230)

#### Type.

MEXICO. Tamaulipas, Jonmave Valley, *E.W. Nelson* 4448 (holotype US332519, isotype GH).

#### Description.

Trailing or twining perennial from woody enlarged rootstock, stems thinly pubescent, eventually glabrescent. Leaves petiolate, small, 2–5 × 2–5 cm, ovate-deltoid, acuminate, cordate-hastate with relatively large, rounded, acute or shallowly bifid auricles, margin often undulate, pubescent, especially beneath; petioles 1–4 cm. Inflorescence of solitary flowers; peduncles 1–3 cm; bracteoles minute; pedicels 7–15 mm; sepals slightly unequal, oblong or oblong-elliptic, obtuse, puberulous, outer 8–10 mm, inner 10–13 × 6–8 mm with scarious margins; corolla 6–9 cm long, funnel-shaped, pubescent, limb 6 cm diam., bluish-purple. Capsules globose, rostrate, glabrous; seeds 8 × 5 mm, ellipsoid, densely lanate.

#### Distribution.

Arid rocky slopes and cliff faces, NE Mexico and adjacent parts of Texas.

**MEXICO. Coahuila**: Sierra del Pino, *I.M. Johnston & C.H. Muller* 385 (GH); Torreón, *G.S. Hinton* 25751 (GBH, TEX); zona de Laguna de la Leche, *T. Wendt & E.J. Lott* 1884 (MEXU). **Nuevo León**: Salinas Victoria, *G.S. Hinton* 24248 (GBH); Sierra de Lampazos, *J.A. Villarreal et al.* 9149 (IEB). **Tamaulipas**: Pueblo Viejo, 2 km S of Tampico, *E. Palmer* 428 (US); 25 km S of Tula, *M.C. Johnston et al*. 11134 (MEXU, MO); Tula, *E. Pérez Calix* 4259 (IEB).

**UNITED STATES. Texas**: Brewster, Brushy Creek, *B.L. Turner & W. Dodson* 23-167 (TEX); ibid., Mt Emory, *B.H. Warnock* 476 (TEX); Cameron Co., *W.R. Carr & M. Pons* 29898 (TEX); Hidalgo, La Joya, *R. Runyon* 2751 (TEX).

#### Note.

This species is characterised by the small pubescent leaves with undulate margins, solitary flowers and oblong puberulous sepals.

### 77. Ipomoea zimmermanii

Taxon classificationPlantaeSolanalesConvolvulaceae

J.A. McDonald, Brittonia 39: 108. 1987. (McDonald 1987b: 108)

#### Type.

MEXICO. Coahuila, Sierra de la Paila, *A.D. Zimmerman* 1948 (holotype TEX00372576, isotypes NY, TEX).

#### Description.

Trailing or twining perennial; stems woody, glabrous or thinly pubescent at nodes. Leaves petiolate, 3.8–4.5 × 3.5–4.5 cm, ovate to subtrilobate, apex obtuse or acute, margin sinuate, base cordate and cuneate onto the petiole, the auricles rounded, both surfaces glabrous; petioles 2.8–5.4 cm. Inflorescence of solitary, axillary flowers; peduncles 1.5–2.2 cm, glabrous or pubescent basally; bracteoles caducous, not seen; pedicels 18–23 mm; sepals equal, 13–16 × 4–5 mm, oblong-elliptic, outer with a few minute appressed hairs, inner with scarious margins; corolla opening at night, 4.5–6 cm long, hypocrateriform, tube purple inside, limb white c. 4 cm in diam., pilose on midpetaline bands; stamens exceeding corolla but not reported as exserted. Capsules and seeds unknown.

#### Distribution.

Only known from the type collected from the slopes of an arid inselberg at 1400 m.

**MEXICO. Coahuila**: type collection.

#### Note.

Reported as related to *Ipomoea rupicola* but differing in the white hypocrateriform corolla.

### 78. Ipomoea kruseana

Taxon classificationPlantaeSolanalesConvolvulaceae

Matuda 36: 115. 1966, Anales Inst. Biol. Univ. Nac. México 36: 115. 1966. (Matuda 1966b: 115)

#### Type.

MEXICO. Guerrero, Mun. Mochitlán, Agua de Obispo, *H. Kruse* 744 (holotype MEXU00093332, isotypes CAS, ENCB, IEB).

#### Description.

Twining perennial from a tuberous rootstock, stem somewhat woody, tomentellous, up to 3 m long. Leaves shortly petiolate, 3–6.5 × 0.7–2.5 cm, oblong to narrowly elliptic, acute, base cuneate, adaxially green, obscurely tomentellous, abaxially white-sericeous to tomentellous; petioles 5–12 mm. Inflorescence of solitary (rarely paired) axillary flowers; peduncles 1.5–4 cm, obscurely sericeous; bracteoles 5–10 mm, linear; pedicels 10–15 mm, sericeous; sepals subequal, 14–20 × 3–5 mm, narrowly ovate, finely acuminate, white-sericeous, the inner with sericeous margins; corolla 5–6 cm long, funnel-shaped, pink or bluish, sericeous, limb 3–3.5 cm diam. Capsules globose, glabrous; seeds unknown.

#### Distribution.

Mixed oak and pine forest on stony soil at 1100 m.

**MEXICO.** Sine data, *Bourgeau* s.n. (P03538332). **Guerrero**: Mun. Mochitlán, Agua de Obispo, *H. Kruse* 6368 (IEB).

#### Note.

The Bourgeau collection differs somewhat from the type in its narrower leaves and slightly shorter sepals but in other ways conforms to this very distinctive species, which is characterised by the sericeous or tomentellous indumentum, persistent linear bracteoles and relatively large, narrowly ovate, acuminate sepals.

The placement of this species is provisional. The pubescent corolla and calyx strongly support its placement in the *Jalapa* radiation but a final decision cannot be made until this species has been successfully sequenced.

### 79. Ipomoea praecana

Taxon classificationPlantaeSolanalesConvolvulaceae

House, Ann. New York, Acad. Sci. 18: 227. 1908. (House 1908b: 227)

#### Type.

MEXICO. Oaxaca, near Reyes, *E.W. Nelson* 1823 (holotype US00111447, isotypes GH, NY).

#### Description.

Vigorous twining or sprawling liana to 4 m; stems and all vegetative parts densely white-tomentose. Leaves petiolate, 8–20 × 6–20 cm, ovate to suborbicular, obtuse or acute, base subtruncate to shallowly cordate with rounded auricles, densely white-tomentose on both surfaces but abaxially paler; petioles 4–35 mm. Inflorescence of shortly pedunculate 3–6-flowered cymes borne on side branches so appearing to form elongate bracteate racemes; bracts resembling small leaves; peduncles very short, 0.5–3 cm, tomentose; bracteoles 10–20 × 4–6 mm, oblong-elliptic, caducous; pedicels 2–3.5 cm, sulcate, thickened upwards, tomentose; sepals 15–20 × 7–10 mm at anthesis but strongly accrescent in fruit to 25 × 15 mm, ovate to elliptic, obtuse, densely tomentose; corolla 6–10 cm long, white, subhypocrateriform, tomentose, more densely so on midpetaline bands, limb c. 5 cm diam., undulate. Capsules 20–25 × 15–18 mm, ovoid, glabrous; seeds 11–14 × 5–6 mm, black with long marginal hairs 12–20 mm long.

#### Illustration.

Figure 50.

**Figure 50.**
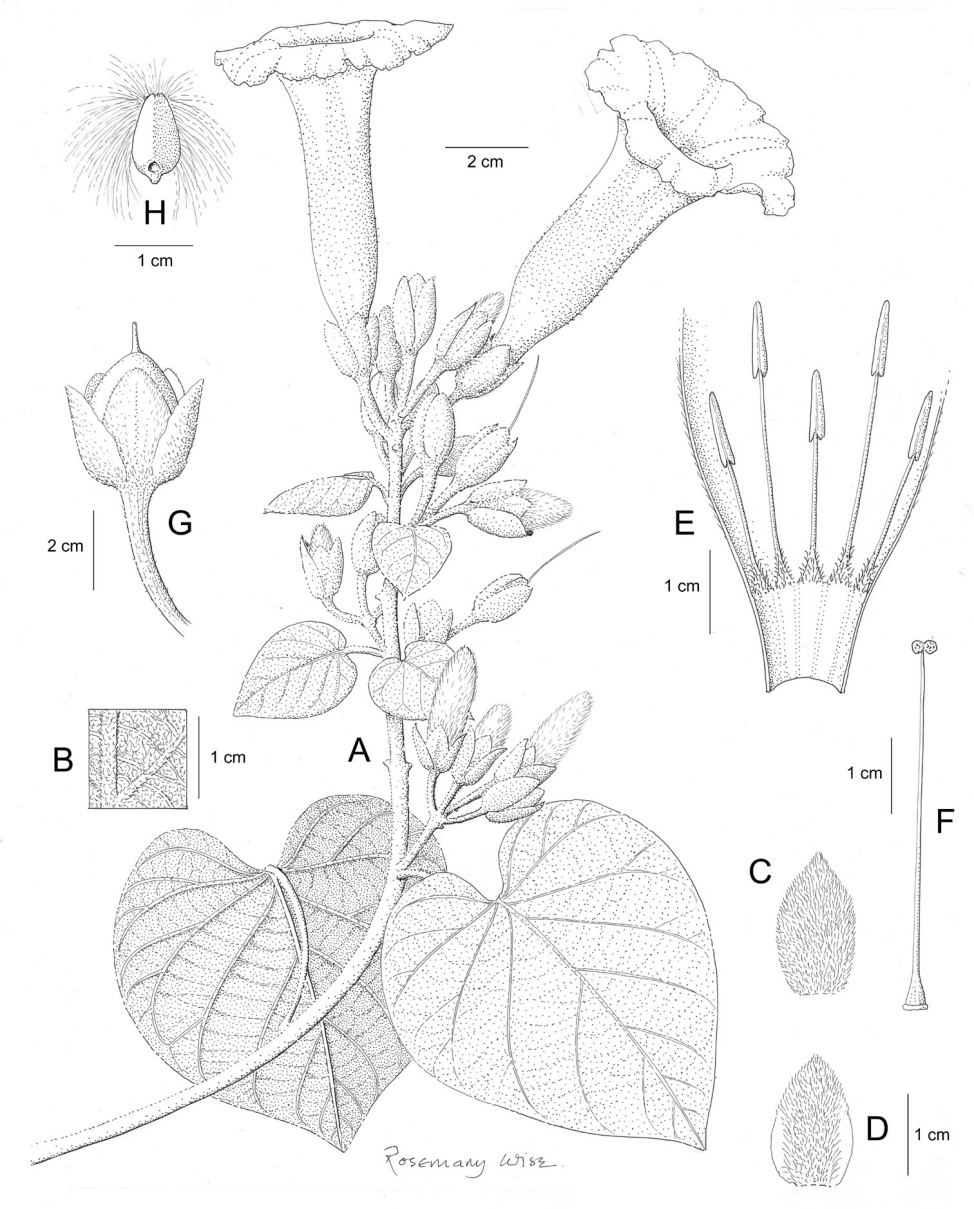
*Ipomoea praecana***A** habit **B** abaxial leaf surface **C** outer sepal **D** inner sepal **E** corolla opened out to show stamens **F** ovary and style **G** calyx and capsule **H** seed. Drawn by Rosemary Wise **A–F** from *Mendoza* 112; **G, H** from *Stafford et al.* 235.

#### Distribution.

Dry deciduous forest and scrub, often on rocky soils below 1100 m from central Mexico south to Nicaragua.

**NICARAGUA.** Estelí, Cerro El Almendro, Condega, *P. Moreno* 25334 (BM); Matagalpa, Loma Chichigua, *W.D. Stevens et al.* 5672 (BM, MO); *A. Molina* 23332 (F).

**HONDURAS.** Francisco Morazán, *V. Mendoza* 112 (BM); *J.V. Rodríguez* 3272 (F).

**EL SALVADOR.** Área protegída San Juan Buenavista, *R.A. Carballo* 17 (MO, W).

**GUATEMALA**. *W. Kellerman* 5645 (US); *J.J. Castillo Mont et al.* 1694 (MO).

**MEXICO**. **Chiapas**: Tuxtla Gutiérrez-San Cristóbal, *P.J. Stafford et al.* 235 (BM). **Colima**: *R. McVaugh* 26236 (MICH). **Est. México & Dist. Fed.**: Temascaltepec, Guayabal, *G.B. Hinton* 3360 (BM, K, MEXU); ibid., Calera, *G.B. Hinton* 5887 (K); ibid., Salitre, *G.B. Hinton* 8739 (K, MO). **Guerrero**: Petalán, *J. Soto Nuñez* 12121 (MEXU); Tierra Colorada, *Kruse* 992 (MEXU). **Michoacán**: *J.V. Dieterle* 3176 (MICH); Aquila, *E. Carranza & I. Silva* 6659 (IEB, MEXU). **Morelos**: Cuernavaca, *C.G. Pringle* 7229 (GH). **Oaxaca**: Cuicatlán, *R. & M.L. Torres* 6908 (MEXU, MO); San Juan Bautista, Cuicatlán, *J.P. Abascal* 118 (MEXU). **Querétaro**: Cerro La Pedrera, *L.M. Chávez* 6 (IEB, MEXU).

#### Note.

This species was placed in the *Arborescens* group by McPherson (1981) but both nuclear data and *ITS* indicate its more correctly placed in the *Jalapa* radiation, to which it conforms morphologically.

### 80. Ipomoea gesnerioides

Taxon classificationPlantaeSolanalesConvolvulaceae

J.A. McDonald, Sida 15: 173. 1992. (McDonald 1992: 173)

#### Type.

MEXICO. Oaxaca, 10.4 miles W of Santiago Astata, *M. Luckow* 2605 (holotype TEX00372566, isotypes: A, MEXU, US).

#### Description.

Woody vine; stems erect, eventually twining and twisted, 0.5–3 m long and up to 1 cm thick, villous when young but glabrescent when old, the stem base swollen and succulent. Leaves petiolate, 2–8 × 1.5–5 cm, broadly elliptic to subrhomboid, base cuneate, rounded or truncate, apex acute to acuminate, adaxially dark green puberulent, abaxially canescent; petioles 0.5–3.5 cm long. Inflorescence of axillary and terminal, bracteate pseudoraceme; flowers solitary in the axils of the petiolate bracts; peduncles absent; bracteoles triangular, stipule-like; pedicels 2–9 mm, puberulent; sepals subequal, 11–15 × 5–7 mm, oblong-elliptic, acute to obtuse, grey-green-canescent; corolla 3.5–4 cm long, urceolate, pubescent, basal cylindrical part 6–8 × 4–8 mm, greenish, then abruptly dilated for 2.5–3.5 cm. 1.5–2 cm wide, limb flared, lobed, 2.5–3 cm diam., midpetaline bands green between purplish petaline regions, stamens included. Capsules ellipsoid, 11–13 × 8–10 mm, glabrous; seeds 6–7 × 3.5 mm, puberulent and densely lanate from the marginal hairs.

**Illustration**: McDonald (1992: 174).

#### Distribution.

Endemic to the Tehuantepec region of SE Oaxaca in southern Mexico.

**MEXICO. Oaxaca**: *A. Saynes et al.* 2657 (IEB, MEXU, MO); *J.F. Castrejón et al.* 1094 (MEXU, MO); ibid., *M. Elorsa* 2485 (IEB, MEXU); Pochutla, *A. Nava Zafra et al.* 780 (MEXU).

#### Note.

Apparently very similar to *Ipomoea bombycina*, differing in the smooth, terete hypocotyl, sepals 10–15 mm and the corolla 3.5–4 cm long with a pale green and purple limb. The two species may intergrade but neither are very well-known.

### 81. Ipomoea bombycina

Taxon classificationPlantaeSolanalesConvolvulaceae

(Choisy) Benth. & Hook f. ex Hemsl., Biol. Cent.-Amer., Bot. 2: 384. 1882. (Hemsley 1882: 384)

Bombycospermum mexicanum C. Presl, Reliq. Haenk. 2: 137, t. 71. 1835. (Presl 1831–35: 137), non Ipomoea mexicana A. Gray (1878). Type. MEXICO (west). Haenke s.n. (PR?, n.v.).
Batatas bombycina Choisy in A.P. de Candolle, Prodr. 9: 340. 1845. (Choisy 1845: 340). Type. Based on Bombycospermum mexicanum C. Presl

#### Type.

Based on *Bombycospermum mexicanum* C. Presl

#### Description.

Woody liana from a rough, furrowed hypocotyl, stem with yellowish bark, pubescent and scabrous-pustulate. Leaves petiolate, 2.5–7.5 cm, ovate-rhomboid, acute, margin somewhat undulate, base subtruncate and cuneate onto the petiole (sometimes asymmetric), adaxially glabrous, abaxially grey-tomentose, puncticulate, veins prominent; petioles 2–5 cm, pubescent, sometimes pustulate. Inflorescence of short leafy axillary racemes, sometimes reduced to tight clusters; rhachis 2–8 cm long, densely pubescent; bracteoles c. 5 mm long, linear, fugacious; pedicels 3–4(–8) mm; sepals 5–8 mm, grey-tomentose, subequal, outer ovate, acute, inner elliptic, obtuse; corolla 2.5–3.5 cm long, basal cylindrical tube 7–10 mm, then expanded, urceolate, tube cream with purplish veins, adpressed pilose, limb with short triangular lobes, c. 3 × 3 mm, yellowish-green. Capsules 15 × 8–10 mm, ellipsoid, glabrous; seeds 7 × 4 mm, blackish, densely woolly with hairs 2 cm or more long.

#### Distribution.

An uncommon endemic of southern Mexico.

**MEXICO. Chiapas**: Mun. Ocozocoautla de Espinoza, *A. Shilom Tom* 3761 (F). **Guerrero**: Acapulco, *E. Palmer* 370 (F, K, MO); ibid., *F. Miranda* 3342 (MEXU); Tecpan, *E. Langlassé* 939 (K). **Jalisco**: Tomatlán, Puerto Vallarta-Barra de Navidad, *E.J. Lott* 678 (FTG, MEXU, MO); La Huerta, *M.G. Ayala* 442 (MEXU); Coyuca-El Zapote, *G.L. Webster & G.J. Breckon* 16227 (MEXU). **Oaxaca**: Tapanatepec, *D. Thomatis* s.n. (K). **Zacatecas**: El Calabazal, *E. Langlassé* 479bis (K, P).

**Notes**. The corolla is consistently 3–3.5 cm long, not 2.5 cm, as stated by McDonald (1992) so corolla size is unreliable in separating this species from *Ipomoea gesneriodes*.

A night-flowering, possibly bat-pollinated species.

The specimen at MO (*H.C. Cutler* 8414) from Ceará, Brazil, identified as *Ipomoea bombycina* by McPherson is leafless and flowerless and is almost certainly not this species. It might, for example, be *I. eremnobrocha*, which has similar seeds and is known from several states in NE Brazil.

### 82. Ipomoea marcellia

Taxon classificationPlantaeSolanalesConvolvulaceae

Meisn. in Martius et al., Fl. Brasil. 7: 257. 1869. (Meisner 1869: 257)

Marcellia villosa Mart. ex Choisy, Mém. Soc. Phys. Genève 10: 443. 1844. (Choisy: 1844: 443), non Ipomoea villosa Ruiz & Pav. (1799). Type. BRAZIL. Piauí, inter Capoculo et Serrinha, *C.F. Martius* 2437 (lectotype M0184915, designated by Delgado Junior et al. [2017]).
?Calystegia discolor Dammer, Bot. Jahrb. Syst. 23(5), Beibl. 57: 42. 1897. (Dammer 1897: 42). Type. BRAZIL. Minas Gerais, Ayucuroa, *A.F.M. Glaziou* 11260 (holotype B† (photo F), isotypes K000612831, C10009678).

#### Type.

Based on *Marcellia villosa* Mart. ex Choisy

#### Description.

Usually trailing liana; stems stout, woody, pubescent to tomentose. Leaves petiolate, 9–17 × 5.5–14 cm, broadly ovate, acute to broadly mucronate, base shallowly cordate to truncate, often with a square sinus, margins often undulate, adaxially tomentellous, abaxially white-tomentose; petioles 9–10 cm. Inflorescence woody, long-pedunculate, formed of compound cymes, usually subcapitate; peduncle 20–42 cm, stout, often woody, white-felted to tomentellous; secondary, tertiary, quaternary peduncles often present, 1.5–4 cm diminishing in length and thickness upwards; bracteoles 10–26 × 7–11 mm, oblong-oblanceolate, acute to obtuse, somewhat boat-shaped and partially enclosing calyx, tardily caducous; pedicels 0–6 mm, tomentellous; sepals slightly unequal 13–15 × 8–10 mm, tomentellous, outer elliptic, obtuse, inner obovate-elliptic, pubescent but less so at scarious margins; corolla 4–5 cm long, white to pale lilac, pilose, funnel-shaped; stamens shortly exserted. Capsules 10–12 mm, ellipsoid, glabrous; seeds 6 mm, dark brown, long-pilose.

#### Distribution.

Endemic to Brazil and almost restricted to caatinga in the north east.

**BRAZIL. Alagoas**: Agua Branca, *K. Costa & Magalhães* 561 (SP). **Bahia**: Jeremoabo, *L.P. de Queiroz et al.* 4651 (HUEFS, RB); Santa Maria da Vítoria, *L.P. de Queiroz et al.* 6114 (HUEFS, OXF). **Ceará**: Estrada de Quichará, *A.P. Duarte* 1487 (RB); Chapada do Araripe, *A. Castellanos & L. Duarte* 536 (MO). **Paraíba**: Mun. Campina Grande, *M.F. Agra* 1271 (K, MO); *Costa & de Brito* 145 (JPB). **Pernambuco**: Mun. Caruaru, *Oliveira & Miranda 15* (PEUFR, SP); Alagoinha, *D. Andrade-Lima* 92 (ASE, SP, SPF). **Rio Grande do Norte**: Caiçara do Rio do Vento, *R.L. Soares Neto* 60 (UFRN). **Sergipe**: *D.M. Coelho* 435 (RB).

#### Notes.

*Pereira Neto et al.* 234 (RB), from Mun. Patrocinio in Minas Gerais, is atypically densely tomentose and distant from the main population and may represent a distinct species.

*Calystegia discolor* is included in this synonomy with some doubt. The extant isotypes are of poor quality and do not show the distinct inflorescence of *Ipomoea marcellia*. The type was collected in Minas Gerais and may correspond to the form represented by *Pereira Neto et al.* 234.

### 83. Ipomoea burchellii

Taxon classificationPlantaeSolanalesConvolvulaceae

Meisn. in Martius et al., Fl. Brasil. 7: 271. 1869. (Meisner 1869: 271)

#### Type.

BRAZIL. Goiás: Rio Tocantins, Porto Imperial, *W.J. Burchell* 8738 (isotype K000612855).

#### Description.

Subshrub with trailing stems, the whole plant softly tomentose to pubescent. Leaves shortly petiolate, 3–7.5 × 0.4–2 cm, oblong, acute, mucronate, base truncate to cordate, margin often inrolled, adaxially green, pubescent, abaxially whitish, gland-dotted, densely pubescent especially on the veins; petioles pubescent, 2–9 mm. Inflorescence of dense, 1–3-flowered subsessile bracteolate clusters, often reduced to single flowers, forming a subterminal inflorescence; peduncles 3–10 mm densely hirsute; bracteoles 6–13 mm, linear, finely acuminate, pilose; sepals very unequal, outer 15–20 × 5–7 mm, oblong, ovate or oblanceolate, obtuse or rounded and mucronate, long-pilose especially near base, pale green and somewhat foliose, inner 11–12 × 3–4 mm, ovate, acuminate, densely lanate but with glabrous, scarious margins; corolla 5.5–7.5 cm long, very narrowly funnel-shaped, only slightly widened upwards, pink with white tube, pilose, the limb undulate, 2.5–3 cm diam. Capsules c. 8 × 7 mm, subglobose, glabrous; seeds 5 × 3 mm, shortly pilose on the angles.

#### Illustration.

Figure 51.

**Figure 51.**
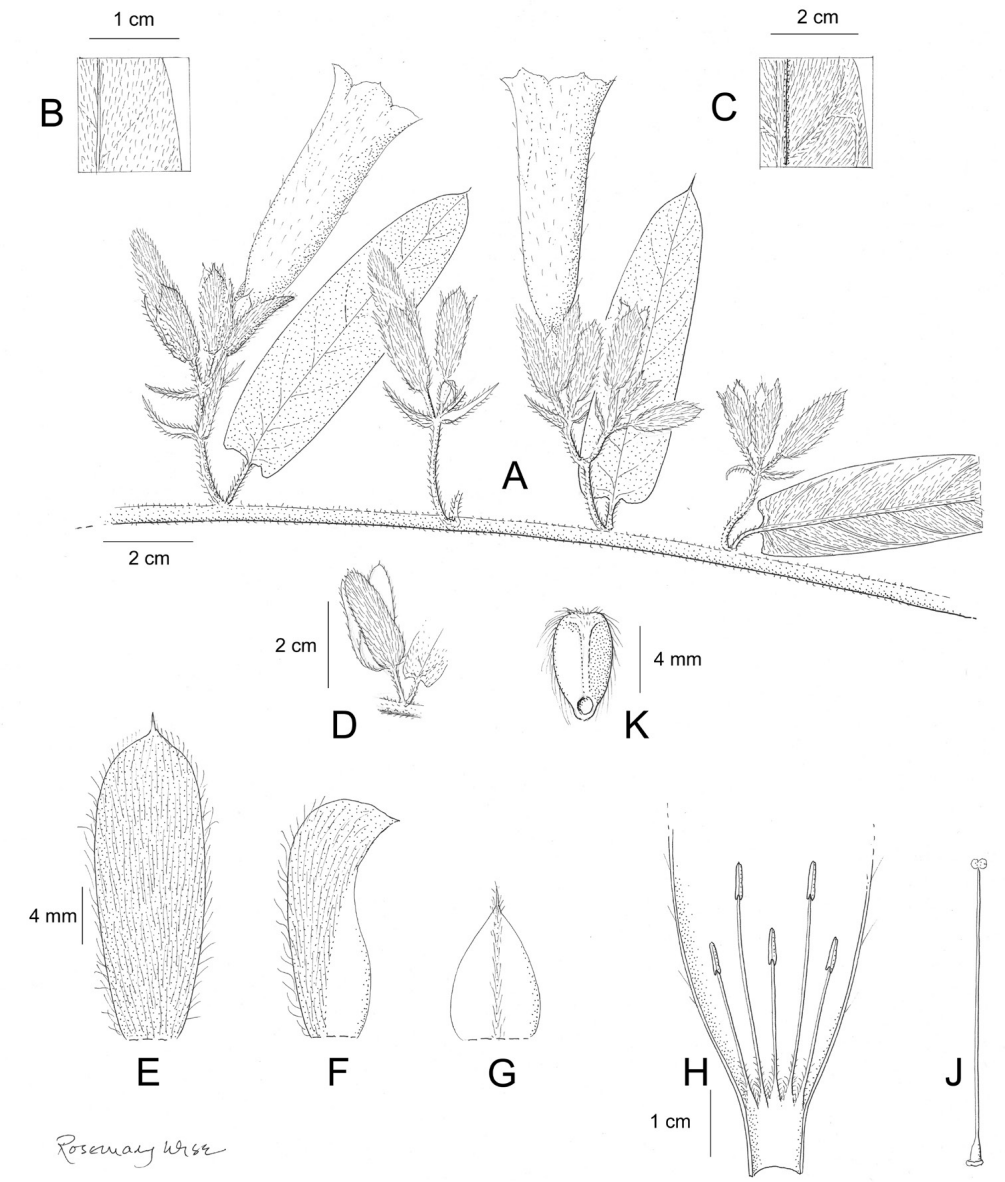
*Ipomoea burchellii*. **A** habit **B** adaxial leaf surface **C** abaxial leaf surface **D** leaf and single flower **E** outer sepal **F** middle sepal **G** inner sepal **H** corolla opened out to show stamens **J** ovary and style **K** seed. Drawn by Rosemary Wise **A–C, F–J** from *Irwin et al.* 21323; **D** K from *Amaral et al.* 835.

#### Distribution.

Endemic to the cerrados of north central Brazil.

**BRAZIL. Bahia**: fide Flora do Brasil (2020). **Goiás**: 24 km S of Alto Paraíso, *H.S. Irwin et al.* 21745, (FTG, HUEFS, NY, MO); Itacajá, *W.N. Fonseca* 109 (RB); Mun. Tupiratins, *G. Hatschbach & R. Kummrow* 38491 (MBM). **Maranhão**: *Eiten & Eiten* 3908 (UB, US); Mun. Mirador, *L.P. Féliz* 8136 (RB). **Mato Grosso**: Mun. Colider, *I.L. Amaral et al.* 835 (ARIZ, FTG, RB). **Piauí**: Ribeiro Gonçalves, *E.M. Saddi et al.* 339 (RB). **Tocantins**: 10 km S. of Guará, *H.S. Irwin et al.* 21323 (NY, RB, FTG); Mun. Goiatins, *S. Pereira-Silva et al.* 15352 (CEN).

#### Note.

The position of this species in the sequence is uncertain.

• The following species (84–127) of Clade A1 are not part of the *Jalapa* radiation.

### 84. Ipomoea carnea

Taxon classificationPlantaeSolanalesConvolvulaceae

Jacq., Enum. Syst. Plants 13. 1760. (Jacquin 1760: 13)

#### Type.

Icon, Jacquin, Stirp. Amer. Hort. Pl. t. 18 (1763), lectotype designated by Austin (1977: 237; possible type specimen BM000953169).

#### Description.

Erect (subsp. fistulosa) or climbing (subsp. carnea) undershrub to 4 m, often growing in clumps, stems stout, hollow, canescent when young, becoming glabrous. Leaves petiolate, 8–20(–30) × 3–10(–12)cm, ovate or elongate-ovate-deltoid, base cordate to subtruncate with rounded auricles, apex acuminate to long-acuminate, both surfaces grey-canescent when young, glabrescent, veins prominent abaxially; petioles 3–8 cm. Inflorescence of long-pedunculate axillary, somewhat compact cymes; peduncles 2–12 cm; bracteoles 3–4 mm, ovate or elliptic, caducous; secondary peduncles 3–7 mm; pedicels 5–15 mm, puberulent; sepals subequal, 5–6 × 7–8 mm, ovate to suborbicular, rounded, tomentellous, margins scarious; corolla 6–7 cm long, funnel-shaped, pink, tomentellous in bud, ±glabrescent, limb 4.5–5 cm diam., shallowly lobed. Capsules 18 × 10 mm, ellipsoid, glabrous; seeds 10–11 × 3–4 mm, woolly with very long hairs on the angles.

#### Variation.

Two distinct subspecies are generally recognised, sometimes as distinct species. The type subspecies is a twining liana with ovate, cordate, shortly acuminate leaves, whereas subsp. fistulosa is an erect, commonly cultivated subshrub, in which the ovate cordate leaves are long-acuminate. Occasional intermediate occurs, such as *J. Schunke & G. Edwin* 3718 (BM, F) from Cajamarca in Peru, which combines the leaf shape of subsp. carnea with the habit of subsp. fistulosa.

### 84a. Ipomoea carnea subsp. carnea

Taxon classificationPlantaeSolanalesConvolvulaceae

Convolvulus pareirifolius Bertol. ex Spreng., Syst. Veg. 1: 613. 1825 [pub. 1824]. (Sprengel 1824: 613). Type. COLOMBIA. [Magdalena], Santa Marta, *S. Bertero*s.n. (lectotype TO, sheet numbered 1615 with four corollas and numerous seeds, designated here).
Batatas pareirifolia (Berthol. ex Spreng.) Choisy, Mém. Soc. Phys. Genève 8(1): 123 [45]. 1838. (Choisy 1838: 123[45]).
Ipomoea pareirifolia (Bertol. ex Spreng.) G. Don, Gen. Syst. 4: 273. 1838. (Don 1838: 273).
Ipomoea carnea forma albiflora Moldenke, Phytologia 2: 224. 1947. (Moldenke 1947: 224). Type. ECUADOR. Loja, La Toma, R. Espinosa 490 (holotype NY00319167).

#### Diagnosis.

Characterised by its climbing habit and ovate, shortly acuminate, almost orbicular leaves.

#### Distribution.

Distributed along the mountain chain from northern Peru to Mexico, this subspecies is perhaps most characteristic of dry woodland. We have seen no specimens from Brazil, the Guianas, Guatemala, El Salvador or Honduras and very few from the Caribbean Islands. Records from Bolivia (Austin and Huáman1996: 6) were presumably based on misidentifications as no specimens have been traced.

**PERU. Cajamarca**: Jaén, *P.C. Hutchinson & Wright* 6376 (UC, MO, S); Pucara, *A. Gentry et al*. 22703 (USM). **Huánuco**: Tingo Maria, *R. Ferreyra* 12782 (USM). **Pasco**: Oxapampa, *R. Rojas et al.* 4272 (MO, OXF). **Piura**: Paimas-Sullana, *A. Gentry et al.* 74921 (MO, USM).

**ECUADOR. El Oro**: Chacras, *H. Vargas et al.* 1169 (MO). **Guayas**: *R. Spruce* 6499 (BM); *Fraser* s.n. (BM); Santa Elena, *L.B. Holm-Nielson et al.* 2449 (AAU, S). **Loja**: *G. Harling & L. Andersson* 18251 (FTG, S). **Manabí**: Montecristí, *L.B. Holm-Nielson et al.* 7210 (AAU, NY).

**COLOMBIA. Antioquia**: San Luis, *J.G. Ramírez & D. Cárdenas* 1748 (MO). **Boyacá**: Puerto Romero-Otanche, *J. Betancur* 6791 (COL). **Cesar**: La Jagua de Ibirico, *J.L. Fernández* 13382 (COL). **Cundinamarca**: Nariño, *J.L. Fernández* 7814 (COL); Tocaima, *J.J. Triana* 3805 (COL). **Magdalena**: Santa Marta, *H.H. Smith* 1583 (BM).

**VENEZUELA. Lara**: *A.H.G. Alston* 6348 (BM, S); *E. Asplund* 15003 (S). **Nueva Esparta**: Margarita Island, *O.O. Miller & J.R. Johnston* 79 (BM, MO). **Maracaibo**: *Moritz* 1241 (BM).

**PANAMA.** Los Santos, Pocri, *D. Burch et al.* 1266 (MO, RB).

**COSTA RICA.** Guanacaste, P.N. Palo Verde, *U. Chavarría* 892 (BM, MO); ibid., P.N. Santa Rosa, *R. Espinoza & U. Chavarría* 1273 (K, MO); *P. Wilkin* 443 (BM).

**NICARAGUA.** Granada, Isla Zapatero, *J.C. Sandino* 1889 (MO, BM).

**BELIZE.** Belize River, *J. Lyon* 12A (MO).

**MEXICO. Baja California**: *J.I. Calzada* 25086 (K, MEXU). **Campeche**: Ciudad de Carmen, *E.F. & H. Cabrera* 15887 (MO, MEXU). **Chiapas**: *D. Breedlove & E. McClintock* 23563 (MEXU). **Quintana Roo**: Solidaridad, Cobá, *O. Téllez* 1382 (BM, MEXU, MO). **Tabasco**: *A. Novelo et al.* 275 (MEXU). **Yucatán**: *M. Peña-Chocarro & Tun* 416 (BM).

**JAMAICA.***Bancroft* s.n. (K); *Marsh* s.n. (K).

**LESSER ANTILLES. St Vincent**: *H.H. & G.W. Smith* 1308 (K).

### 84b. Ipomoea carnea subsp. fistulosa

Taxon classificationPlantaeSolanalesConvolvulaceae

(Mart. ex Choisy) D.F. Austin, Taxon 26: 337. 1977. (Austin 1977: 237)

Ipomoea fistulosa Mart. ex Choisy in A.P. de Candolle, Prodr. 9: 349. 1845. (Choisy 1845: 349). Type. BRAZIL. *C. F. Martius* 2398 (lectotype M0184890, designated by D.F. Austin 1977: 237).
Convolvulus batatilla Kunth, Nov. Gen. Sp. Pl. 3: 106. 1818 [pub.1819]. (Kunth 1819: 106). Type. VENEZUELA. Valles de Aragua, Caracas, Cumaná, Humboldt & Bonpland 723 (holotype P00670761).
Ipomoea batatilla (Kunth) G. Don, Gen. Syst. 4: 275. 1838. (Don 1838: 275).
Batatas crassicaulis Benth., Voy. Sulphur 5: 134.1845. (Bentham 1845: 134). Type. ECUADOR. Guayaquil, Sinclair (holotype K000612883, isotypes BM)
Ipomoea crassicaulis (Benth.) B.L. Rob., Proc. Amer. Acad. Sci. 51: 530. 1916. (Robinson 1916: 530).
Ipomoea fruticosa Kuntze, Rev. Gen. 2: 444. 1891. (Kuntze 1891: 444). Type. BRAZIL. *R. Spruce* 6499 (lectotype K000395032, designated by Austin (1977: 237, isolectotypes K, MPU).
Ipomoea tragulifera Miers, Proc. Roy. Hort. Soc.4: 160. 1864. (Miers 1864: 160). Type. COLOMBIA. Río Magdalena, Weir 20 (holotype BM000953166).
Ipomoea gossypioides D. Parodi, Contr. Fl. Parag. 15. 1877. (Parodi 1877: 15). Type. “Paraguay et Corrientes [Argentina] in humidis et uliginosis frequentissimma”, no specimen cited or found.
Ipomoea texana Coult., Contrib. U.S. Natl. Herb. 1(2): 45. 1890. (Coulter 1890: 45). Type. UNITED STATES. Texas, *G.C. Neally*s.n. (holotype US00147753, isotypes GH, K, US).
Ipomoea fistulosa var. nicaraguensis Donn. Sm., Bot. Gaz. 19(7) 256. 1894. (Donnell Smith 1894: 256). Type. NICARAGUA. Rivas, Río de Las Lajas, *W.C. Shannon* 5046 (holotype US246468).
Ipomoea nicaraguensis (Donn. Sm.) House, Bot. Gaz. 43(6): 409. 1907. (House 1907b: 409). Type. Based on Ipomoea fistulosa var. nicaraguensis Donn. Sm.
Ipomoea fistulosa forma albiflora Chodat & Hassl., Bull. Herb. Boiss., ser. 2, 5: 687. 1905. (Chodat and Hassler 1905: 687). Type. PARAGUAY. [Cordillera], Tobatí, *E. Hassler* 6180 (?G, n.v.).
Ipomoea crassicaulis var. goodellii Degener, Fl. Hawaii. sine pag. 1936. (Degener 1932–1940). Type. HAWAII. No specimen cited.

#### Type.

Based on *Ipomoea fistulosa* Mart. ex Choisy

**Diagnosis**. This subspecies is characterised by its erect habit and elongate, long-acuminate leaves.

#### Illustration.

**Figure 52.**
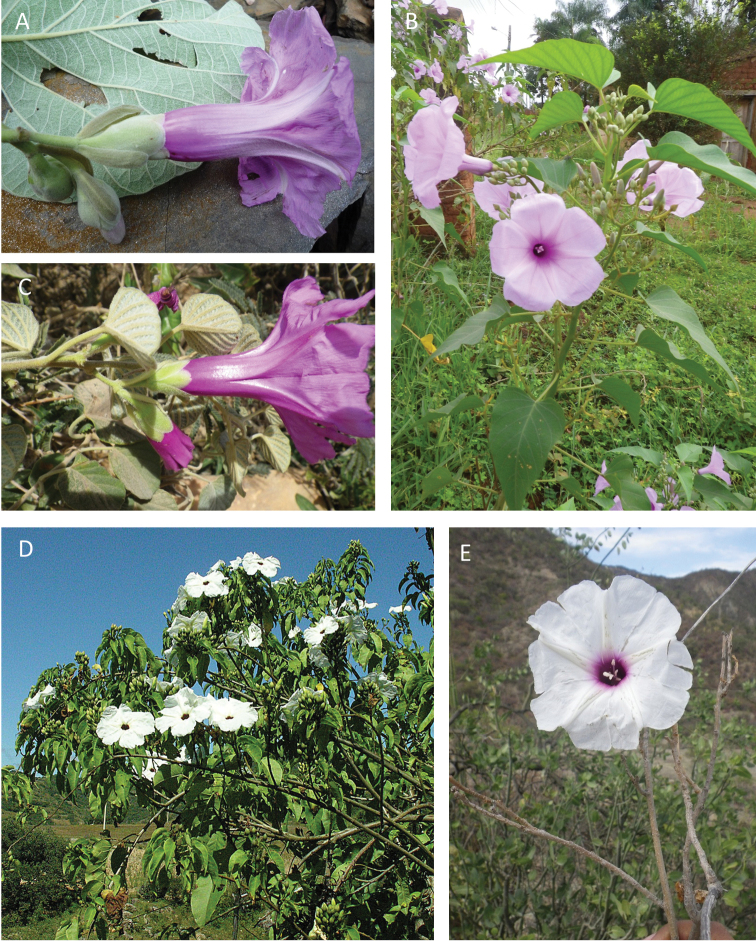
Photographs of *Ipomoea* species. **A***I. calyptrata***B**I. carnea subsp. fistulosa**C**I. brasiliana var. subincana**D***I. pauciflora***E***I. juliagutierreziae*. **A** Beth Williams; **B, C** E John Wood; **D** Dick Culbert.

Figure 52B; O’Donell (1959b: 159); Austin (1998: 400); Deroin (2001: 195) as *Ipomoea fistulosa*.

#### Distribution.

Probably native in swamp and flooded pampas in eastern Bolivia, northern Argentina (Formosa, Corrientes), Eastern Paraguay and southern Brazil in the Pantanal and Rio Paraguay-Parana systems but also possibly so in seasonally dry swampy areas elsewhere. It is apparently rare or absent in the Amazon forest region, not being recorded from Pando in Bolivia or Rôndonia in Brazil and with few records from Brazilian Amazonas. It is also widely cultivated as an ornamental in gardens up to at least 1000 m and it is not always easy to decide whether a population is spontaneous or planted. It seems that all collections from the Caribbean, the United States and probably Mexico are cultivated or recently naturalised plants.

**ARGENTINA. Corrientes**: *A. Schinini* 13248 (CTES, MO). **Formosa**: *L. Morel* 1730 (LIL, RB); *R.H. Fortunato et al.* 6083 (MO).

**PARAGUAY. Alto Paraguay**: Puerto Casado, *F. Mereles & R. Degen* 6150 (CTES, FCQ, MO). **Boqueron**: Mariscal Estigarribia, Escuela Agricola, *B. Garcete* 15 (FCQ). **Central**: *B. Balansa* 1077 (P); Limpio, *E. Zardini* 2678 (FCQ, MO). **Cordillera**: *E. Zardini* 22224 (MO). **Guairá**: Colonia 14 de Mayo, *F. Mereles et al.* 10016 (FCQ). **Pres. Hayes**: Riacho Heê, *J. de Egea et al.* 783 (BM, FCQ). **Ñeembucú**: Est. San Antonio, *J. de Egea et al.* 367 (BM, FCQ). **Pres. Hayes**: Est. Loma Porá, W. of Puente Concepción, *F. Mereles & R. Degen* 6015 (FCQ, MO).

**BRAZIL. Acre**: *J.U. Santos* 66 (RB). **Amapá**: *D.F. Austin* 6969 (MO). **Amazonas**: *P.J. & H. Maas* 522 (MO). **Bahia**: *R.M. Harley et al.* 16278 (K, MO, NY). **Ceará**: *A. Löfgren* 707 (S). **Maranhão**: *N.A. Rosa* 2510 (NY); *B.A. Krukoff* 2028 (NY, S). **Mato Grosso**: *S. Moore* 908 (BM); Transpantaneira highway, *G. Prance* 26158 (NY). **Mato Grosso do Sul**: *G. Hatschbach* 29552 (MBM, NY, S); *E.P. Heringer* 860 (NY). **Pará**: Santarém, *R. Spruce* s.n. [3/1850] (BM); *A. Ducke* s.n. (RB). **Paraíba**: *M.F. Agra* 661 (RB). **Paraná**: *M.G. Caxambú* 221 (MBM). **Pernambuco**: *L.P. Féliz* 5661 (RB). **Piauí**: *B.M.T. Walter* 6678 (CEN, RB). **Rio de Janeiro**: *A.M. Miranda* 3728 (RB). **Rio Grande do Norte**: *A.M. Marinho* 65 (RB). **Santa Catarina**: *L.A. Funez* 3642 (FURB). **São Paulo**: *J.M. Camargo* 2510 (RB). Throughout Brazil except Rondônia fide Flora do Brasil (2020).

**FRENCH GUIANA.***D.W. Roubik* (MO).

**SURINAM.** No record or specimen seen.

**GUYANA.** Fide Austin and Huáman (1996: 6).

**BOLIVIA. Beni**: Cercado, *N. & M. Ritter* 3335 (BOLV, MO); Mamoré, *M. Moraes et al.* 1523 (LPB, USZ). **Santa Cruz**: Germán Busch, *M. Toledo et al.* 591 (USZ). Velasco, *J.R.I. Wood & B. Williams* 27736 (OXF, LPB, USZ). *N. Ritter & P.F. Foster* 2391 (MO, USZ); Warnes, *M. Nee* 45170 (LPB, MO, NY, USZ).

**PERU. Amazonas**: *P.J. Barbour* 4226 (MO). **Cusco**: *P. Nuñez & S. Walsh* 6321 (CUZ, MO, USM). Ica: *J. Roque* 100 (USM). **Lima**: Canta, *G. Vilcapoma* 7777 (USM). **Loreto**: M. *Rimachi* 8593 (MO)

**ECUADOR.***Fagerlind & Wibom* 159 (S). **Guayas**: Colimes-Balzar, *C. Bonifaz* 678 (GUAY). **Loja**: *J. Jaramillo & V. Winnerskjold* 5826 (GB).

**COLOMBIA. Chocó**: *E. Ferrero & R. Jaramillo* 2485 (MO). **Magdalena**: *C. Allen* 51 (MO). **Tolima**: *L. Aguirre* 203 (COL, RB). **Valle**: *L.E. Forero & N. Hernández* 1613 (MO).

**VENEZUELA. Apure**: *G. Davidse & A.C. González* 14800 (MO). **Bolívar**: J. *Velazco* 71. **Guárico**: *R. Rondeau* 160 (MO). **Miranda**: *K.R. Robertson & D.F. Austin* 149 (MO).

**PANAMA.***B.L. Seeman* 177 (BM); *W.H. Lewis et al.* 296 (MO).

**COSTA RICA.***D. Hernández & R. Chacón* 9025 (K).

**NICARAGUA.***W.D. Stevens* 9421 (BM, MO), 22900 (BM, MO); *A.A. Beetle* 26253 (K, UC).

**EL SALVADOR.***J.M. Tucker* 922 (K, UC).

**HONDURAS.** Fide Nelson and Proctor (1994).

**BELIZE.***E.G.F. Campbell* 87 (K).

**GUATEMALA.***H. Pittier* 355 (BM).

**MEXICO. Campeche**: *B. Faust & P. Ucan* 0522 (CICY, MO). **Chiapas**: *Espinosa* 153 (MO). **Guerrero**: *E. Palmer* 431 (BM, K). **Guanajuato**: *E. Carranza & R.M. García* 5330 (IEB). **Michoacán**: *G.B. Hinton et al.* 12514 (K). **Nuevo León**: *J.A. Villarreal* 9191 (IEB). **Oaxaca**: *M. Elorsa* 2804 (IEB). **Querétaro**: *E. Pérez* 4356 (IEB). **Quintana Roo**: *E.F. & H. Cabrera* 6847 (MEXU, MO). **Sonora**: fide Felger et al. (2012). **Tabasco**: *J.N. Rovirosa* 226 (K). **Tamaulipas**: *E. Palmer* 222 (BM, K). **Veracruz**: *F. Chiang* 419 (K, MEXU, MO). **Yucatán**: *J.S. Flores* 08146 (MO).

**UNITED STATES. Florida**: fide Wunderlin and Hansen (2011: 391). **Texas**: type of *Ipomoea texana*.

**CUBA.***R.A. Howard* 4841 (A, BM, S); *E.L. Ekman* 445 (S); *C.F. Baker* 14 (K, MO).

**JAMAICA.***G.R. Proctor* 25573 (BM); *L. Wynter* 2192 (K).

**HAITI.***E.L. Ekman* H9151 (S)

**DOMINICAN REPUBLIC.***H.F.A. von Eggers* 1839 (BM, K); *H. von Türckheim* 2544 (BM, K); *E.J. Valeur* 467 (K, MO)

**PUERTO RICO.***J.S. Miller & C.D. Sherman* 6602 (MO).

**LESSER ANTILLES: British Virgin Islands**: Anegada, *M.A. Hamilton* 126 (K). **Dominica**: *C. Whitefoord* 5821 (BM).).

**TRINIDAD**. *W.E. Broadway* s.n. [24/11/1932] (BM, MO). **Tobago**: *W.E. Broadway* 2450 (K).

**NETHERLANDS ANTILLES. Curaçao**: fide Proosdij (2012).

**HAWAII.** Type of Ipomoea crassicaulis var. goodellii.

#### Note.

Immediately recognised by the tall erect habit combined with the cordate, acuminate leaves. The tomentellous sepals are unexpectedly small.

• Species 85–93. These nine species form a clade in both the nuclear and chloroplast sequences. They are very heterogenous morphologically and it is difficult to see any common character.

### 85. Ipomoea inaccessa

Taxon classificationPlantaeSolanalesConvolvulaceae

J.R.I. Wood & Scotland, Kew Bull. 73 (57): 2. 2018. (Wood et al. 2018: 2)

#### Type.

BOLIVIA. Caranavi, Serrania de Bellavista, west side above Carrasco, *J.R.I. Wood & S.G. Beck* 28539 (holotype LPB, isotypes K, OXF, USZ).

#### Description.

Liana, 15–20 m high, the flowers covering the tops of trees; stems when young green, minutely puberulous, weakly angled; when mature woody, grey, somewhat muricate; rootstock (juvenile) tuberous. Leaves petiolate, 5.5–14 × 3–8 cm, ovate, cordate, acuminate, both surfaces, minutely and densely puberulent, abaxially paler with rather prominent, raised veins; petioles 2.5–6 cm, minutely puberulent. Inflorescence of (1–)2–4(–7)-flowered, pedunculate, axillary cymes; peduncles 2.5–11 cm, minutely puberulent; bracteoles at base of cyme resembling small leaves, upwards caducous and not seen; secondary peduncles 0.5–4 cm; pedicels 1.5–3.5 cm, minutely puberulent; sepals slightly unequal, somewhat convex, outer 13–16 × 10 mm, inner 18–20 × 15–18 mm, elliptic to subovate, rounded, rigid, glabrous, pale green with scarious margins; corolla 9–9.5 cm long, funnel-shaped, white with pale pink throat or pure white, glabrous; limb unlobed, c. 6 cm wide; filaments unequal, 15–24 mm long, anthers 10 mm long; style 3 cm long; stigma biglobose. Capsules subglobose, 18 × 15 mm, glabrous; seeds 8 × 4 mm, pilose on the margins with hairs up to 12 mm long.

#### Illustration.

Figure 53.

**Figure 53.**
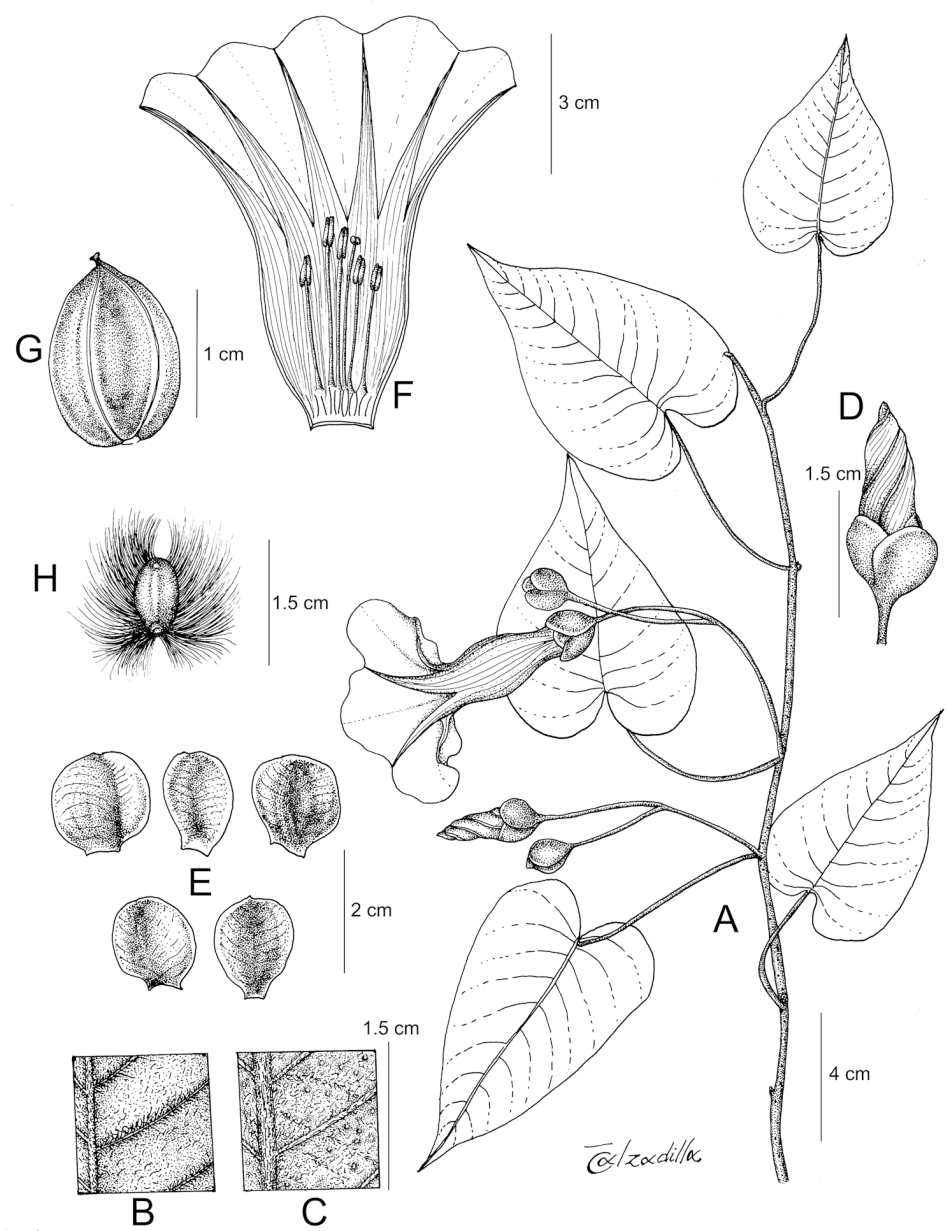
*Ipomoea inaccessa*. **A** habit **B** adaxial surface of leaf **C** abaxial leaf surface **D** bud **E** sepals **F** corolla opened out to show stamens and style **G** capsule **H** seed. Drawn by Eliana Calzadilla **A–C** from *Wood & Beck* 28539; **D–F** from *Wood & Beck* 28543; **G, H** from *Feuerer & Höhne* 4662.

#### Distribution.

Endemic to moist hill forest with frequent cloud 1400–1500 m on the west side of the Serrania de Bellavista.

**BOLIVIA. La Paz**: Caranavi, *T. Feuerer & N. Höhne* 4662 (LPB); *S.G. Beck* 17205 (LPB, K, SP).

#### Note.

A very vigorous liana reaching heights unattained by most species of *Ipomoea*. Herbarium specimens are most likely to be confused with *Ipomoea philomega* but that species has smaller, deep pink corollas, 5–6 cm in length and, usually, glabrous leaves and distinctive reddish sepals (Figure 167A). *Ipomoea inaccessa* has a larger corolla about 9 cm long, which is white or white with a pale pink throat. The leaves are uniformly densely puberulent on both surfaces and the sepals are pale green. The flower colour and sepal shape suggest it is related to *I. reticulata* O’Donell and *I. saopaulista* O’Donell and this is confirmed by molecular sequence data using *ITS*. However, the sepals (13–20 mm long) and corolla (9–9.5 mm long) are much larger.

### 86. Ipomoea saopaulista

Taxon classificationPlantaeSolanalesConvolvulaceae

O’Donell, Lilloa 26: 392. 1953. (O’Donell 1953a: 392)

Ipomoea floribunda Moric. var. martii Meisn. in Martius et al., Fl. Brasil. 7: 262. 1869. (Meisner 1869: 262). Type. BRAZIL. A.F. Regnell [1]11: 198, lectotype BR000005748655 designated here).
Ipomoea batatoides var. tomentosa Glaz., Bull. Soc. Bot. France 57, mém. 3e: 484. 1910. (Glaziou 1910: 484). Type. BRAZIL. Serra de Pragaos a Theresopolis, Rio de Janeiro, *A.F.M. Glaziou* 4143 (B, C, K, P), nom. nud.
Ipomoea paulistana O’Donell, Dusenia 3: 278. 1950. (O’Donell 1950c: 278), non Ipomoea paulistana (Silva Manso) Stellfeld (1945).

#### Type.

Based on Ipomoea floribunda Moric. var. martii Meisn.

#### Description.

Variable twining perennial or liana to 6 m, stems (and leaves) glabrous to tomentellous. Leaves petiolate, 4–12 × 4–12 cm, ovate, shortly acuminate, cordate, with rounded auricles, adaxially glabrous, abaxially glabrous, pubescent or tomentellous; petioles 2.5–5.5 cm. Inflorescence typically many-flowered, subcorymbose in form or a raceme of umbels; peduncles 2–9(–20) cm; bracteoles caducous, scale-like; secondary peduncles 3–5 cm; tertiary peduncles 1–1.5 cm; pedicels 5–20 mm; sepals unequal, outer 7–8 × 3–4 mm, ovate, obtuse, scarious-margined, glabrous, inner 8–10 × 4–5 mm, oblong-elliptic, rounded to retuse, margins broad, scarious; corolla (3–)3.5–4.5 cm long, white, funnel-shaped, glabrous, limb 2.5 cm diam. Capsules 9–10 × 8–12 mm, subglobose, glabrous; seeds 6 × 2–3 mm, pilose with long cilia c. 8 mm in length.

#### Illustration.

Figure 54.

**Figure 54.**
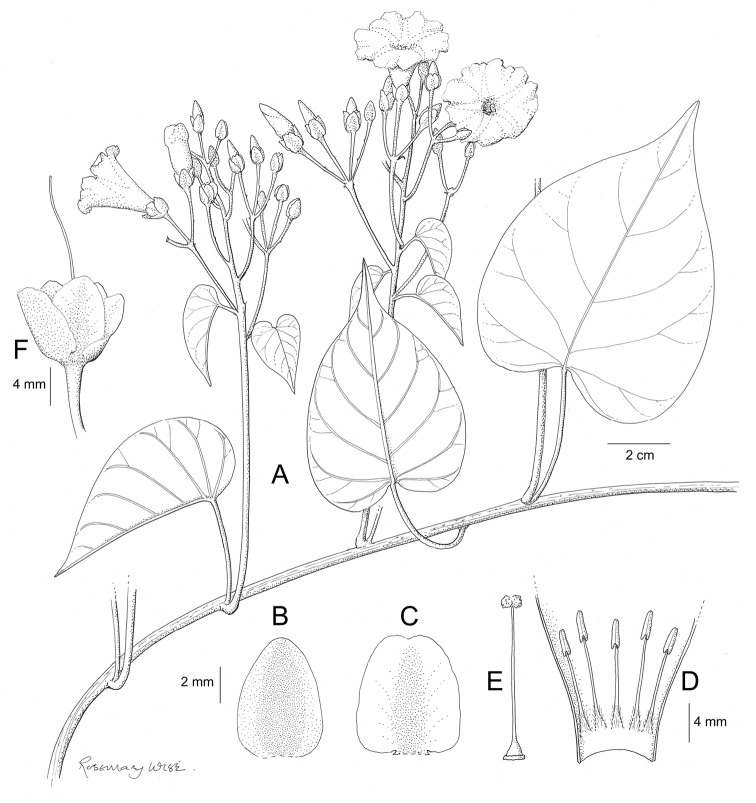
*Ipomoea saopaulista***A** habit **B** outer sepal **C** inner sepal **D** corolla opened out to show stamens **E** ovary and style **F** calyx in fruit. Drawn by Rosemary Wise from *de Queiroz et al.* 15967.

#### Distribution.

Common in scrub and woodland and on woodland borders in the São Paulo area extending north to Bahia and south to NE Argentina and Rio Grande do Sul. Records from Mato Grosso and Pará (Flora do Brasil 2020 under construction require confirmation).

**ARGENTINA. Corrientes**: Ituzaingo, *A. Schinini et al.* 11144 (CTES). **Misiones**: Belgrano, *H. Keller & Franco* 9733 (CTES); San Pedro, *Rodríguez* 1165 (CTES).

**BRAZIL. Bahia**: Estrada de Catuaba para Bonito, *L.P. de Queiroz et al.* 15967 (CTES, OXF). **Dist. Fed.**: Lago Paranoá, *Nascimiento et al.* 148 (K); Bacia do Rio São Bartolomeu, *E.P. Heringer et al.* 6693 (IBGE, K, MO); Côrrego Landim, *H.S. Irwin et al.* 14028 (NY). **Espirito Santo**: Santa Teresa, *Wilson Boone* 1116 (MO); *A.P. Duarte* 8834 (RB); Castelo, *R. Goldenberg* 1074 (RB). **Goiás**: Corumbá de Goiás, *E.P. Heringer & A.E.H. Salles* 17024 (IBGE, MO); Serra Geral do Paraná, *W.R. Anderson et al.* 7700 (NY, MO); *H.S. Irwin et al.* 31787 (NY). **Minas Gerais**: *P. Clausen* s.n. (K); *C.W. Mosén* 4521 (S); *A. Arbo et al.* 5288 (CTES, SPF); Viçosa Agricultural College, *Y. Mexia* 4397 (BM, K, MO, NY, S). **Paraná**: Adrianópolis, *G. Hatschbach* 38533 (CTES, NY); Cel. Vivida, *G. Hatschbach* 26373 (CTES, K, MBM, NY, S); *O.S. Ribas* 6203 (MBM). **Rio de Janeiro**: *D. Sucre* 2703 (RB); Petrópolis, *G. Martinelli* 801 (RB). **Rio Grande do Sul**: *P.P.A. Ferreira* 61 (ICN) fide Ferreira and Miotto (2009: 449). **Santa Catarina**: *P. Dusen* 11892 (NY, S). **São Paulo**: *C.W. Mosén* 1499 (S); Mairipora, Beira de Fernão Dias, *J.R. Pirani et al.* 17559 (SPF, K).

#### Note.

In much of its range this species is easily recognised by its creamy-white flowers arranged in subumbellate cymes. However it can only be distinguished from *Ipomoea reticulata* by the larger sepals and corolla and some specimens can be difficult to assign, particularly from the Brasilia area. There is a case, therefore, for treating these two species as subspecies but we are reluctant to make this decision. Although *I. reticulata* is always glabrous or minutely scabridulous-puberulent, the leaves of *I. saopaulista* are commonly densely pubescent or even tomentose, a state never seen in *I. reticulata* and mere size is not, therefore, the only distinguishing feature between the two species.

### 87. Ipomoea reticulata

Taxon classificationPlantaeSolanalesConvolvulaceae

O’Donell, Lilloa 26: 389. 1953. (O’Donell 1953a: 389)

Ipomoea peredoi O’Donell, Lilloa 30: 44. 1960. (O’Donell 1960: 44). Type. BOLIVIA. Santa Cruz, I. Peredos.n. (holotype LIL158045).

#### Type.

COLOMBIA. Norte de Santander, región de Sarare, *J. Cuatrecasas* 13321 (holotype LIL001281, isotypes COL, F).

#### Description.

Weak liana to 3 m, stems woody, glabrous to minutely scabridulous, often dotted with black glands. Leaves petiolate, 4–9 × 3–6 cm, ovate to suborbicular, cordate with rounded auricles, shortly acuminate, usually glabrous but sometimes scabridulous-puberulent, abaxially often minutely black-punctate; petioles 2.5–5 cm, scabridulous. Inflorescence of pedunculate axillary cymes, these often developing into a raceme or panicle-like structure 5–10 cm long; peduncles 1–4.5 cm, sometimes extended into a rhachis up to 3 cm long; secondary peduncles 0.5–1.8 cm long; bracteoles scale-like, caducous; pedicels very variable in length. 5–15 mm long, glabrous; sepals subequal, 5–7 × 3–5 mm, elliptic, obtuse, scarious-margined, inner obovate with very broad scarious margins; corolla 2.3–3.5 cm long, creamy-white with greenish midpetaline bands and (sometimes a dull violet centre), campanulate, glabrous, limb 2.5 cm diam., undulate; stamens held at corolla mouth. Capsules ovoid, 10–12 × 7–8 mm, glabrous; seeds 5 mm long, pilose.

#### Illustration.

Figure 55.

**Figure 55.**
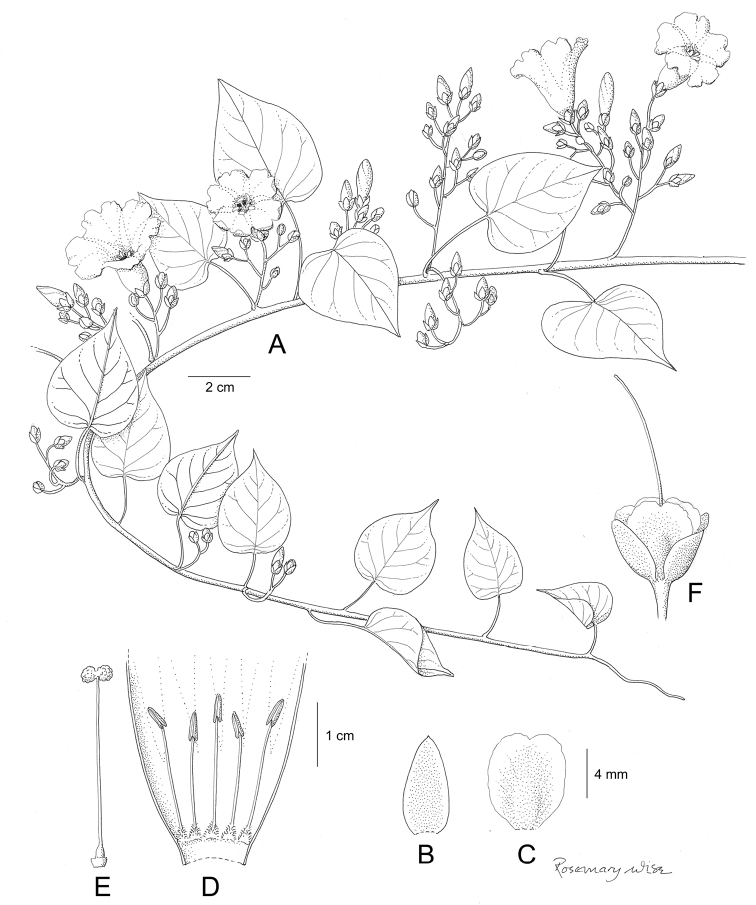
*Ipomoea reticulata***A** habit **B** outer sepal **C** inner sepal **D** corolla opened out to show stamens **E** ovary and style **F** capsule. Drawn by Rosemary Wise **A–E** from *Krapovickas & Schinini* 32452 **F** from *Killeen* 6252.

#### Distribution.

Widely distributed in tropical America from Bolivia north to southern Mexico but becoming less common north of Panama. It is usually found in sub-Andean rainforest or in moister areas of seasonally dry forest in the Amazonian lowlands, rarely above 1000 m.

**BRAZIL. Acre**: *D.C. Daly* 11802 (NY). **Mato Grosso**: *L. Carreira et al.* 895 (INPA, NY). **Pará**: *R.S Secco et al. 201* (MO). Also Goiás and Minas Gerais fide Flora do Brasil (2020).

**BOLIVIA. Beni**: Ballivián, *J. Balderrama* 517 (NY, LPB, SP). **Chuquisaca**: Calvo, *A. Carretero et al.* 867 (HSB, MO, OXF). **Cochabamba**: P.N. Carrasco, Río Ichoa, *O. Colque & L. Mendoza* 472 (MO, NY, OXF, USZ); Chapare, *J.R.I. Wood & B. Williams* 27732 (K, LPB, USZ). **La Paz**: Guanay, *H.H. Rusby* 1995 (NY, MICH). **Pando**: *A. Araujo-M. al.* 5387 (K, USZ). **Santa Cruz**: Ibañez, *M. Nee & L. Bohs* 49612 (CTES, NY, MO, USZ); Ñuflo de Chávez, *J.R.I. Wood* 14767 (K, LPB, USZ); Velasco, *J.R.I. Wood et al.* 28205 (LPB, USZ).

**PERU. Amazonas**: Condorcanqui, *R. Kayap* 628 (MO). **Ayacucho**: La Mar, Villa Union, *J. Roque* 5538 (USM). **Junín**: Chanchamayo, *Sandeman* s.n. (BM). **Loreto**: *R. Vásquez & N. Jaramillo* 9357 (MO). **Madre de Dios**: *R.B. Foster* 6385 (F); Río Acre, *E. Ule* 9706 (K, NY). **Pasco**: Cordillera San Matias, *A.H. Gentry & C. Díaz* 58628 (F, MO); Oxapampa, Palcazú, *R. Vásquez et al.* 38032 (MO). **San Martín**: Juan Jui, Alto Río Huallaga, *G. Klug* 4305 (BM, K, MO S); Mariscal Cáceres, *J. Schunke* 3896 (F, MO).

**ECUADOR. Napo**: Río Aguarico, *J.S. Brandbyge et al.* 36185 (AAU, MO); Yasuni National Park, *R.J. Burnham* 1496 (MICH, QCA). **Pastaza**: Canelos, *H. Lugo* 1545 (K, MO). **Sucumbíos**: Río Cuyabeno, *J.S. Brandbyge et al.* 33820 (AAU, MO). **Zamora-Chinchipe**: Zamora-Romerillos, *T. Croat & M. Menke* 89763 (MO).

**COLOMBIA. Guaviare**: *J. Cuatrecasas* 7433 (COL). **Norte de Santander**: type of *Ipomoea reticulata*. **Putumayo**: Umbria, *G. Klug* 1773 (BM, K, MO, S).

**VENEZUELA. Bolívar**: Sifontes, *G. Aymard* 4712 (MO). **Lara**: Jiménez, P.N. Yacambú, *G. Davidse & A.C. González* 21334 (MO).

**PANAMA.***B.L. Seeman* 4921 (K).

**COSTA RICA.** Puntarenas, *W.A. Haber & E. Bello* 2937 (MO); San José, Aserri, *B.E. Hammel et al.* 22887 (MO)

**MEXICO. Puebla**: *W.G. D’Arcy* 11938 (MO). **Tamaulipas**: Gomez Farias, *S. Rodriquez* 79 (MO). **Veracruz**: *J. Dorantes et al.* 03599 (F, MEXU, MO, XAL); San Andrés Tuxtla, *G. Ibarra Manríquez & S. Sinaca* 2077 (MEXU); Las Tuxtlas, *S. Sinaca* 1021 (MEXU).

**Notes**. Usually easily identified by the small flattish sepals (the inner with rather broad scarious margins) and short, campanulate, cream corolla but sometimes difficult to distinguish herbarium specimens from *Ipomoea batatoides* which also commonly has leaves abaxially gland-dotted. However in *I. batatoides* the corolla is much larger and usually pink, the sepals are coriaceous and convex without broad scarious margins and the axillary inflorescences are usually clearly cymose in form. In southern Brazil *Ipomoea reticulata* is largely replaced by *Ipomoea saopaulista* O’Donell, which differs in its larger corolla. Intermediates between the two are reported from Goiás.

*R. Vásquez* 5044 (FTG, MO) from Peru may represent an undescribed species related to *Ipomoea reticulata*. It is similar in all aspects but all flower parts are much smaller, the sepals 4–4.5 mm long and the corolla c. 1.8 cm in length. It was collected in Ucayali, Prov. Coronel Portillo, about 74°35'S, 8°25'W around km 10 on the Carretera Federico Besadare. More collections are needed to evaluate this plant.

Plants cited from Mexico are similar in inflorescence structure and flower colour but the corolla is rather large and more funnel-shaped and the sepals appear coriaceous. They need investigation and may also belong to a different species.

### 88. Ipomoea tarijensis

Taxon classificationPlantaeSolanalesConvolvulaceae

O’Donell, Lilloa 30: 53. 1960. (O’Donell 1960: 53)

#### Type.

BOLIVIA. Tarija, 1904, *K. Fiebrig* 2655A (holotype BM000758194, isotypes K, P).

#### Description.

Trailing herb, stems up to 2 m long, thinly pubescent. Leaves petiolate, 5–11 × 5–11 cm, ovate to suborbicular, narrowly cordate with rounded, overlapping auricles, apex shortly acuminate, adaxially almost glabrous, abaxially bluish-grey with prominent, raised veins, scurfy-pubescent; petioles 3–6 cm, thinly pubescent. Inflorescence of long-pedunculate, 1–3(–5)-flowered, axillary cymes, peduncles 7–15 cm, straight; bracteoles caducous; secondary peduncles 0.5–1.6 cm; pedicels 0.5–2.5 cm, scurfy-pubescent, slightly widened below calyx, often fracturing at summit; sepals subequal, 7–9 × 4–5 mm, broadly oblong, obtuse, thinly scurfy-puberulent, margins scarious, glabrous, inner c. 1 mm longer and broader with broad scarious margins; corolla 4.5–5 cm long, shortly funnel-shaped being flared from just above basal tube, glabrous, pale pink, limb c. 5 cm in diam., distinctly lobed with rounded lobes, stamens held at corolla mouth. Capsules ovoid, 2 cm long, shortly rostrate, glabrous; seeds 6 mm long, densely lanate.

#### Illustration.

Figures 6G, 56.

**Figure 56.**
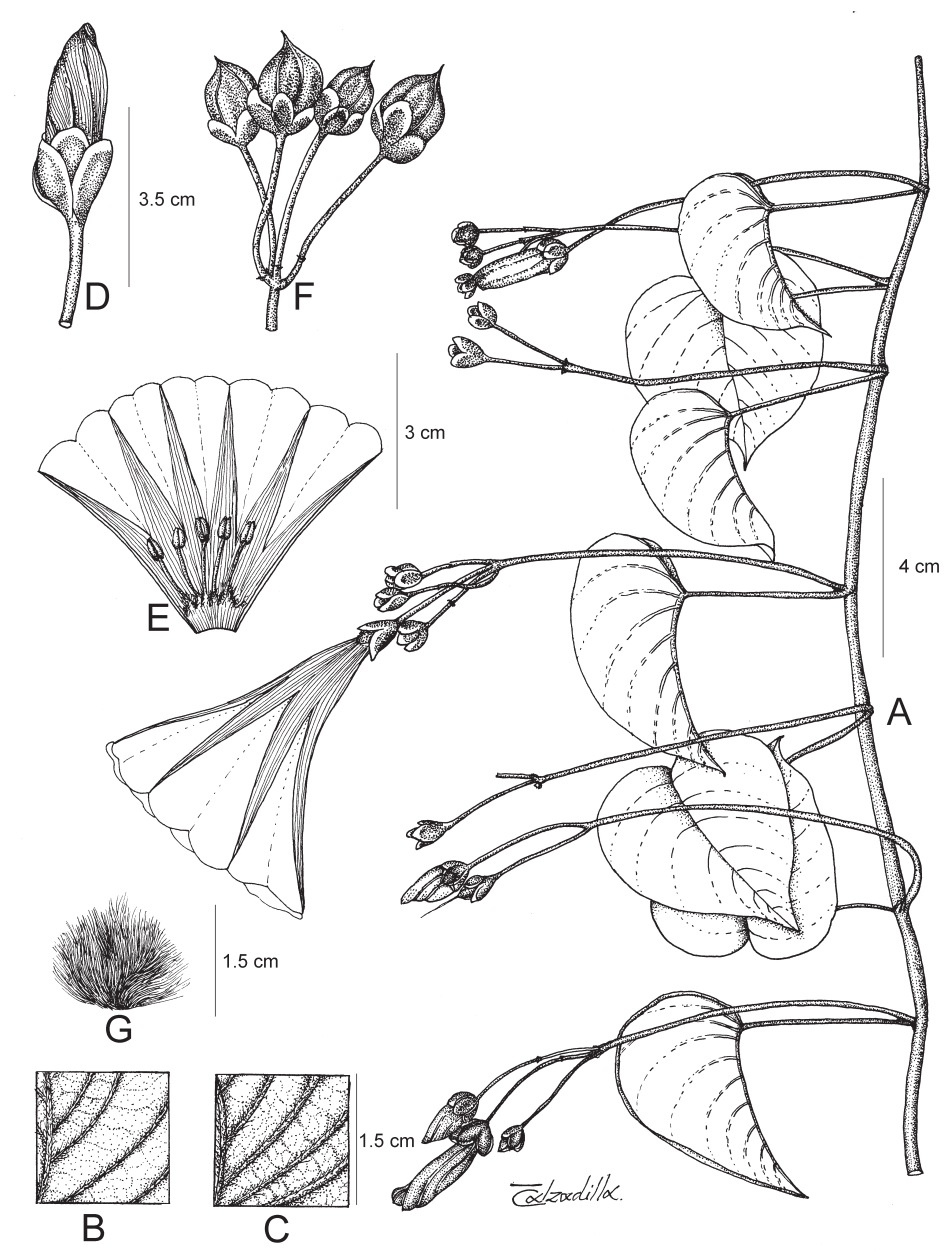
*Ipomoea tarijensis***A** habit from apex of stem **B** adaxial leaf surface **C** abaxial leaf surface **D** bud and sepals **E** corolla opened out to show stamens **F** fruiting inflorescence **G** seed. Drawn by Eliana Calzadilla from *Wood* 27920.

#### Distribution.

Endemic to Tarija Department in Bolivia, where it grows on open stony banks, in abandoned fields and in scrubby gullies around 2500 m, particularly on and around the Cuesta del Condor.

**BOLIVIA. Tarija**: Cercado, *J.R.I. Wood* 15954 (K, LPB); Cuesta del Condor, *J.R.I. Wood* 27920 (OXF, K, LPB, USZ); O’Connor, *S.G. Beck et al.* 22202 (LPB, SI).

#### Note.

O’Donell (1960) compared this species with *Ipomoea jujuyensis* and *I. lilloana*. From the latter it is easily distinguished by the glabrous buds; from the former it is less easily distinguished by the trailing habit, oblong rather than elliptic sepals, the overlapping leaf auricles, the short stamens (2.5 cm, not 4–5 cm long) and the long hairs on the seeds.

The stamens of *Ipomoea tarijensis* are visible at the mouth of the corolla but are unusually short, a character it shares with *I. reticulata*. Molecular studies suggest these two and *I. saopaulista* form a single clade.

### 89. Ipomoea graniticola

Taxon classificationPlantaeSolanalesConvolvulaceae

J.R.I. Wood & Scotland, Kew Bull. 70 (31): 67. 2015. (Wood et al. 2015: 67)

#### Type.

BOLIVIA. Santa Cruz, Ñuflo de Chávez, El Cerrito, *J.R.I. Wood, D. Villarroel & S. Renvoize* 25750 (holotype USZ isotypes K, LPB, UB).

#### Description.

Twining perennial to 2 m, completely glabrous in all vegetative parts; stems slender, trailing or twining; rootstock tuberous. Leaves petiolate, divided into 5 separate leaflets, base ± truncate, leaflets 1.2–3 × 0.2–0.6 cm, attenuate at both ends, apex acute, the basal pair narrowly oblong, the remaining three narrowly oblong-elliptic; petiole 1.2–1.5 cm, commonly straight. Inflorescence of 1–2-flowered, axillary, pedunculate cymes; peduncles slender, 2.5–5 cm; secondary peduncles c. 1.5 cm; bracteoles 1.5 × 0.5 mm, strap-shaped, obtuse, early caducous; pedicels 1.1–2 mm; sepals equal, 7–8 × 3.5 mm, broadly oblong, rounded, margins broad, scarious; corolla 6–7 cm long, pink, glabrous, funnel-shaped, limb 5–6 cm, unlobed, stamens included, stigma obscurely bilobed. Capsules 8 × 6 mm, obovoid, conspicuously 5-lobed, glabrous; seeds 4.5 × 3 mm, ±ovoid, pale brown, with deciduous white marginal hairs c. 3 mm in length.

#### Illustration.

Figure 57.

**Figure 57.**
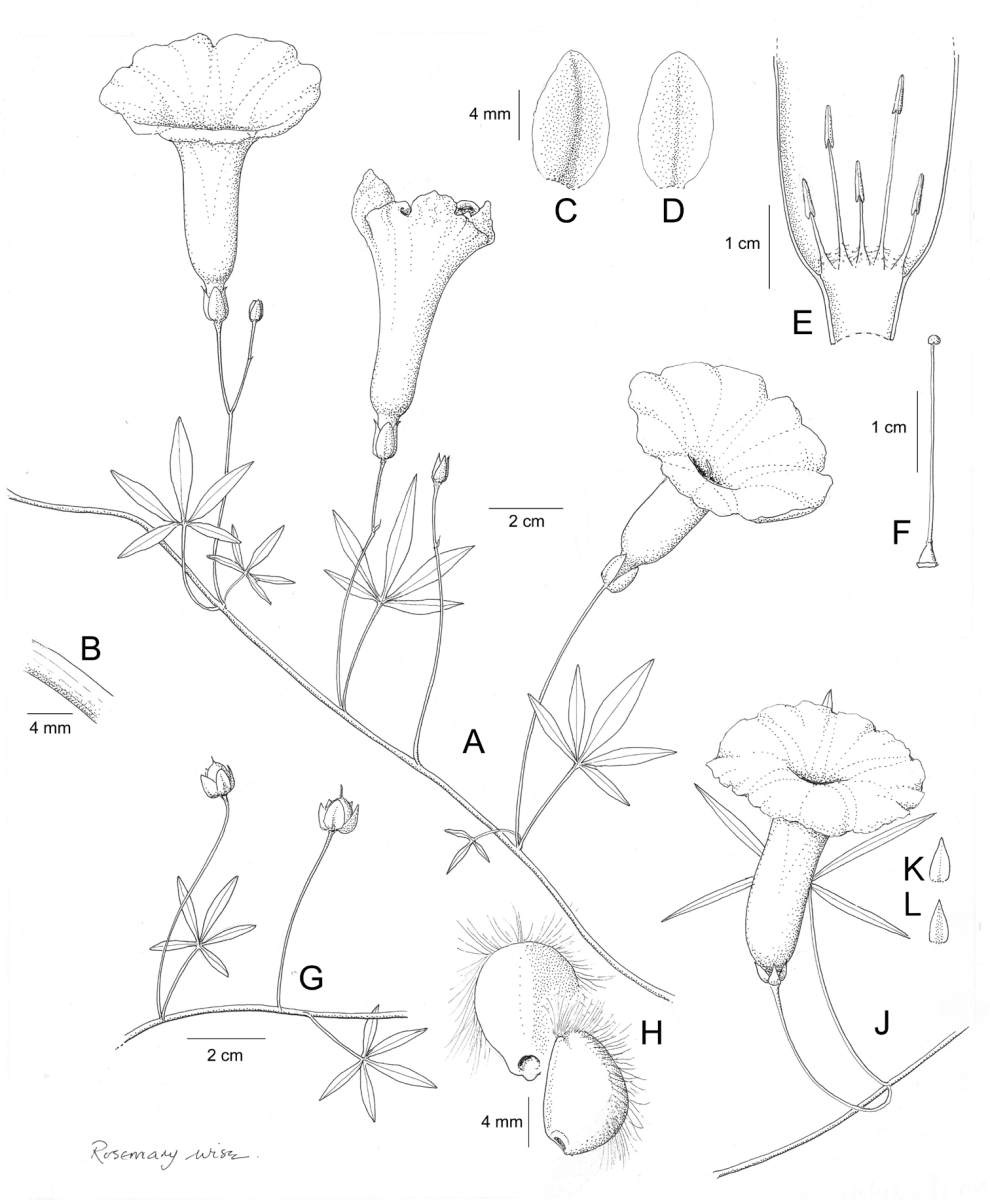
**A–H***Ipomoea graniticola*. **A** habit **B** stem **C** outer sepal **D** inner sepal **E** corolla opened out to show stamens **F** ovary and style **G** shoot with fruiting inflorescence showing capsule **H** seeds. **J–L***Ipomoea subrevoluta*. **J** inflorescence **K** outer sepal **L** inner sepal. Drawn by Rosemary Wise **A–F** from *Wood et al.* 27763; **G, H** from *Wood et al.* 24991; **J–L** from *Wood et al.* 27790.

#### Distribution.

Grows amongst Bromeliads in patches of vegetation on isolated granite inselbergs in eastern Bolivia, northern Paraguay and Brazil.

**PARAGUAY. Alto Paraguay**: Cerro León, *F. Mereles* 6632 (CTES, FCQ).

**BRAZIL. Ceará**: Mun. Granja, Terezinha, São Miguel, *E.B. Souza et al.* 3395 (HUVA, PEUFR). **Mato Grosso**: São João da Barra, *N.A. Rosa & M.R. Santos* 2089 (MG, MO, RB).

**BOLIVIA. Santa Cruz**: Ñuflo de Chávez; El Cerrito *J.R.I. Wood et al.* 24991 (K, LPB, UB, USZ); *J.R.I. Wood et al.* 27763 (OXF, LPB, USZ); Montecristo, *J.R.I. Wood et al.* 27996 (LPB, OXF, USZ).

#### Notes.

This species is related to *Ipomoea rosea* Choisy from NE Brazil differing in the leaves with five narrowly oblong leaflets and in the absence of a tooth-like appendage on the abaxial surface of the sepals. It has been confused with *I. subrevoluta* but differs in the larger obtuse to rounded sepals and grows in a quite different habitat.

The Paraguay specimen resembles *Ipomoea graniticola* in every way except for the presence of an appendage on the abaxial surface of the outer sepals.

### 90. Ipomoea rosea

Taxon classificationPlantaeSolanalesConvolvulaceae

Choisy in A.P. de Candolle, Prodr. 9: 384. 1845. (Choisy 1845: 384)

#### Type.

BRAZIL. Piauí, *Martius* (holotype M0184974).

#### Description.

Glabrous twining herb to 3 m; stems relatively slender. Leaves petiolate, divided into 3 leaflets (reduced 4^th^ or 5^th^ leaflets sometimes present), leaflets 0.1–5.2 × 0.05–1.8 cm, unequal, the terminal usually larger than the laterals, lanceolate to oblong-elliptic, obtuse, basally cuneate; petioles 0.4–3.5 cm. Inflorescence of lax axillary pedunculate cymes; peduncles 1.5–5 cm; bracteoles 1 mm, linear, caducous; secondary peduncles 1.5–2cm; pedicels 2–5 mm; sepals 6–7 × 2–3 mm, unequal, outer oblong-elliptic to obovate, acute, scarious-margined, with subapical often tooth-like acute dorsal protuberance, inner broadly to narrowly oblong, obtuse, scarious with a blunt protuberance; corolla 5–6 cm long, pink, narrowly funnel-shaped, glabrous, limb 3–4 cm diam. Capsules globose, 5–6 mm diam. glabrous; seeds 4 × 3 mm, the angles shortly pilose.

#### Illustration.

Figures 4B, 58.

**Figure 58.**
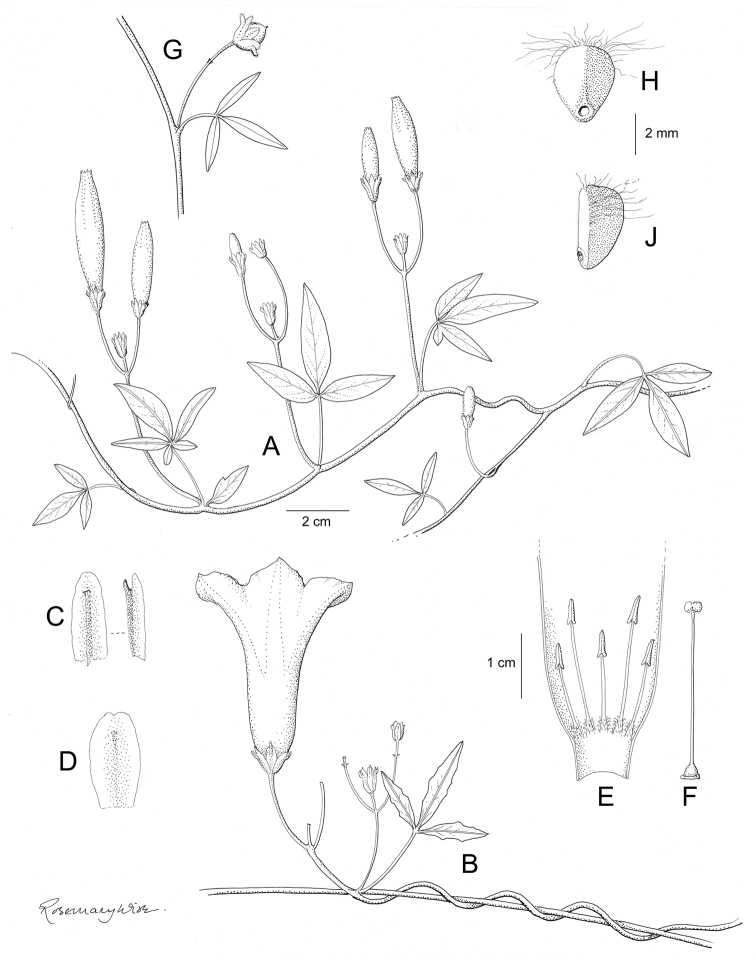
*Ipomoea rosea*. **A** habit with buds **B** habit with corolla **C** outer sepal, abaxial view (left), lateral view (right) **D** inner sepal **E** corolla opened out to show stamens **F** ovary and style **G** calyx and capsule **H** seed **J** seed, lateral view. Drawn by Rosemary Wise **A** from *Figueiredo et al.* 588; **B** from *Pirani et al.* 51360; **C–F** from *Harley et al.* 18947; **G–J** from *Harley et al.* 21217.

#### Distribution.

A characteristic species of caatinga, endemic to NE Brazil.

**BRAZIL. Alagoas**: fide Flora do Brasil (2020). **Bahia**: Serra do Açuruá, NE of Gentio do Ouro, *R.M. Harley et al.* 18947 (K, NY); Serra Geral de Caitité, *R.M. Harley et al.* 21217 (K, NY); Mun. Abaíra, *J.R. Pirani et al.* 51360 (K, MO); Morro de Chapéu, *L. Cardoso* 1639 (RB); Rio de Contas, *R.M. Harley et al.* 54830 (K). **Ceará**: Mun. Quixeramobim, *J. Collares & L. Dutra* 181 (K); Mun. Aiuaba, *M.A. Figueiredo et al.* 588 (K, EAC); *A. Löfgren* 259 (S). **Paraíba**: Mun. Campina Grande, *M.F. Agra* 1132 (K); regiones secas, *J. Coêlho de Moraes* 2105 (K, P, S, W). **Pernambuco**: Mun. Faz. Nova, *W.M. Andrade & L.S. Figueiredo* 149 (K, PEUFR); P.N. do Catimbaú, *G.C. Delgado Junior* (RB); Ibimirim, *A. Gomes* 28 (UFRN); *B. Pickel* 3572 (S). **Piauí**: *G. Gardner* 2245 (K, BM, OXF). **Rio Grande do Norte**: Ceará-Mirim, *J.G. Jardim* 6061 (UFRN); Natal, *V.R.R. Sena* 198 (RB). **Sergipe**: Poço Redondo, *R. Simão-Bianchini* 1746 (ASE).

#### Note.

A slender fragile plant easily fracturing when dry. The leaves are usually with three oblong-elliptic leaflets, much broader than in *Ipomoea graniticola*. The sepals are quite variable and the tooth-like appendage is often reduced to little more than a swelling.

### 91. Ipomoea pterocaulis

Taxon classificationPlantaeSolanalesConvolvulaceae

J.R.I. Wood & L.V. Vasconc., Kew Bull. 72(9): 8. 2017. (Wood et al. 2017a: 8)

#### Type.

BRAZIL. Bahia, Morro do Chapeú, ca. 1 km após Lagoinha na Estrada para Cafarnaum, 11°41'01"S, 41°20'11"W, 902 m, *L.P. de Queiroz, J.R.I. Wood & H. Huaylla* 15957 (holotype HUEFS 209791, isotype OXF, K).

#### Description.

Vigorous twining plant decumbent in open ground or climbing over bushes to several metres, stems stout, prominently winged, glabrous. Leaves petiolate, 2.5–10 × 2–9 cm, ovate-deltoid, acute or obtuse, base shallowly cordate with a broad sinus and rounded to subacute auricles, margin undulate to slightly sinuate, both surfaces glabrous, abaxially paler with prominent veins; petioles 1.3–5.5 cm. Inflorescence of pedunculate axillary cymes; peduncles 2–12 cm; bracteoles 1–2 mm, lanceolate, caducous; secondary peduncles 1–2 cm; pedicels 3–5 mm; sepals subequal, glabrous, 13–15 × 7–10 mm, elliptic, obtuse or rounded; outer often reddish, inner with scarious margins; corolla (6–)8–9 cm long, glabrous funnel-shaped, tube white; limb 7–8 cm diam. Capsules 10 × 8–9 mm, ellipsoid, glabrous, muticous; seeds 6 × 4 mm, dark brown, glabrous except for long white hairs on angles.

#### Illustration.

Figure 59.

**Figure 59.**
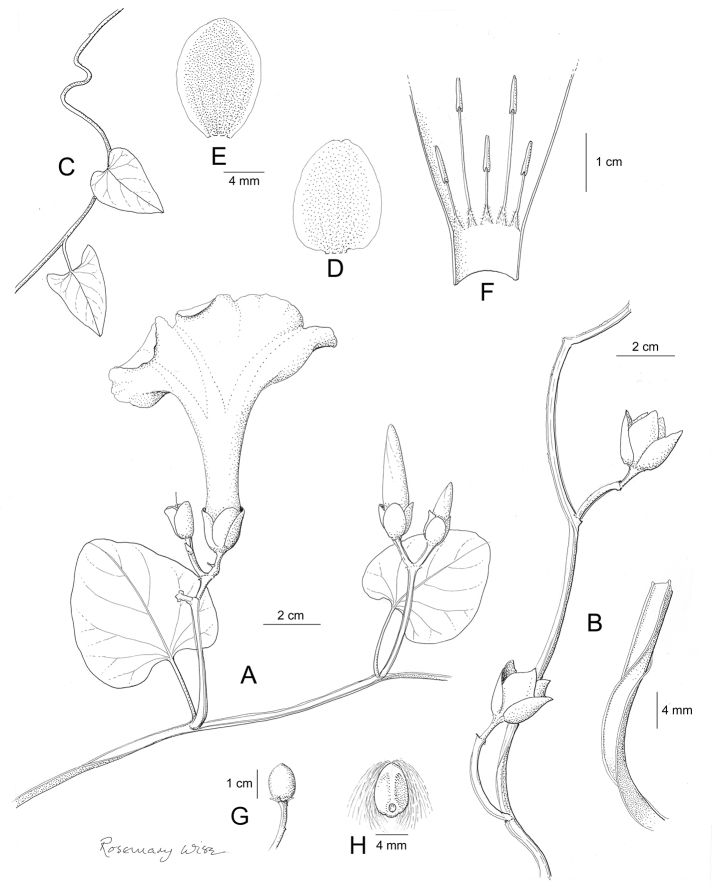
*Ipomoea pterocaulis*. **A** habit **B** mature stem **C** young stem and leaves **D** outer sepal **E** inner sepal **F** corolla opened up to show stamens **G** capsule **H** seed. Drawn by Rosemary Wise **A–E, G, H** from *L. de Queiroz* 15957; **F** from *R. Harley et al.* 53599.

#### Distribution.

An endemic species of the Brazilian Caatinga/Cerrado interface.

**BRAZIL. Bahia**: 14 km SW of Cansanção, *R.M. Harley et al.* 16476 (P, MO, NY); Mun. Abaira, 1.5 km de cidade, *R.M. Harley et al.* 53599 (HUEFS, RB); Mun. Bela Vista, Juremal, *M.V. Moraes* 676 (HUEFS); Mun. Ourolândia, 9 km de Umburanasca, *J.G.A. do Nascimento et al.* 620 (HUEFS). **Pernambuco**: Mun. Afrânio, Serra do Coboclo, *E.P. Heringer et al.* 266 (IPA).

#### Notes.

Superficially distinctive because of its large corolla and winged stem, this species has been identified as *Ipomoea jalapa* (L.) Pursh. However it is easily distinguished by its glabrous stem, sepals and corolla. The seed indumentum is also quite different in the two species.

*R.M. Harley et al.* 16476 is slightly different from the other collections in having shorter sepals about 10 mm long and is included with a degree of uncertainty.

*C. Toleto Rizzini & A. Mattos Filho* 1113 (RB, OXF) from Itaobim, Minas Gerais may also belong to this species. It differs in the compound cymes with up to 15 flowers and in the truncate-based sepals but is otherwise similar. Further collections are needed to clarify its status.

### 92. Ipomoea connata

Taxon classificationPlantaeSolanalesConvolvulaceae

J.R.I. Wood & L.V. Vasconc., Kew Bull. 72(9): 6. 2017. (Wood et al. 2017a: 6)

#### Type.

BRAZIL. Bahia, basin of the upper São Francisco River, 4 km N of Bom Jesus da Lapa on main road to Ibotirama, 43 24W 13 13S, 450 m, 20 April 1980, *R.M. Harley, G.L. Bromley, A.M. De Carvalho, J.L. Hage & H.S. Brito* 21588 (holotype CEPEC, isotype K).

#### Description.

Twining perennial herb to 2 m, stems reddish-brown, glabrous, slightly angled, weakly winged when young. Leaves petiolate, 2.5–6 × 1.5–4 cm (only seen on inflorescence), ovate-deltoid, shallowly cordate with rounded to subacute auricles, acute or obtuse, margin undulate to sinuate, both surfaces glabrous, abaxially paler; petioles 0.6–3 cm, fused with the base of the peduncle for up to 10 mm, slender, glabrous. Inflorescence of pedunculate axillary cymes, sometimes (?usually) compounded into complex branched axillary inflorescences; peduncles 2.5–6.5 cm, glabrous, sometimes extended into a rhachis up to 14 cm long; primary bracteoles petiolate, foliose, ovate-deltoid, 5–20 × 3–11 mm, deciduous and often absent; secondary peduncles 0.7–3 cm long; tertiary peduncles 3–6 mm; ultimate bracteoles c. 1–1.5 mm, ovate, caducous; pedicels 11–21 mm; sepals unequal, glabrous, oblong-ovate, outer 6–8 × 3–4 mm, obtuse, inner 8–9 × 5 mm, rounded, scarious except in the central area; corolla 7–7.5 cm long, narrowly funnel-shaped, pink, glabrous, limb c. 3.5 cm diam. Capsules and seeds not seen.

#### Illustration.

Figure 60.

**Figure 60.**
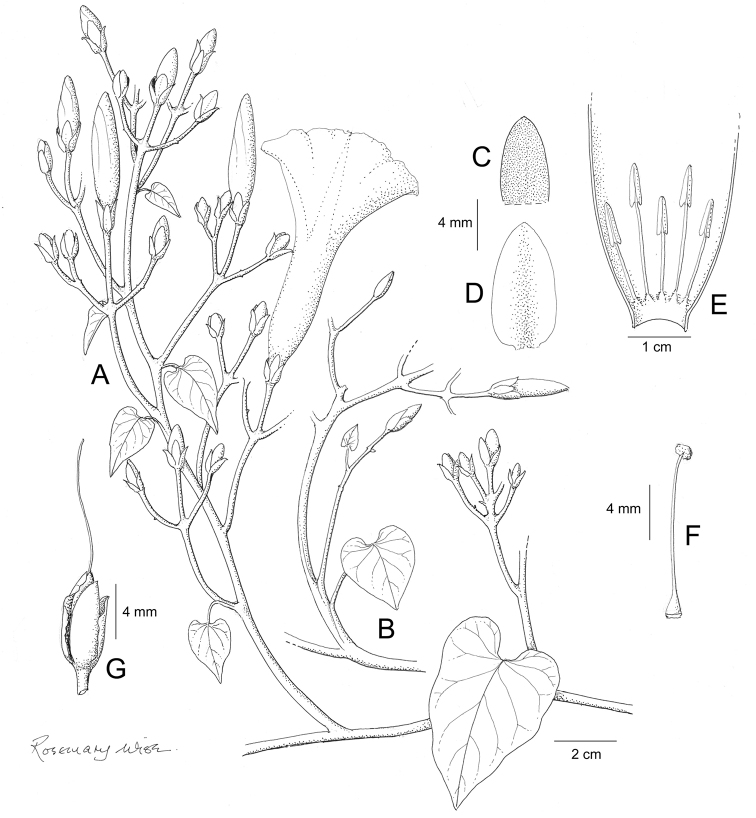
*Ipomoea connata*. **A** habit **B** detail of habit showing partially fused petiole and peduncle **C** outer sepal **D** inner sepal **E** corolla opened out to show stamens **F** ovary and style **G** calyx after corolla has fallen. Drawn by Rosemary Wise from *R.M. Harley et al.* 21588.

#### Distribution.

Endemic to Bahia in Brazil growing in secondary vegetation with caatinga and dry deciduous forest, 450–500 m.

**BRAZIL. Bahia**: upper São Francisco River, Faz. Umbuzeiro da Onça, ca. 8 km from Bom Jesus da Lapa, *R.M. Harley et al.* 21535 (CEPEC, K); 4 km N of Bom Jesus da Lapa on main road to Ibotirama, *R.M. Harley et al.* 21588 (holotype CEPEC, isotype K).

#### Note.

Apparently unique amongst Brazilian species because the petiole is connate with the peduncle for part of its length.

### 93. Ipomoea longifolia

Taxon classificationPlantaeSolanalesConvolvulaceae

Benth., Pl. Hartweg. 16. 1839. (Bentham 1839–57: 16)

Convolvulus queretarensis Sessé & Moçiño, Pl. Nov. Hisp. 1: 24. 1888. (Sessé y Lacasta and Moçiño 1887–90: 24), Type. MEXICO. Querétaro, “Pavón” (isotype BM 000645558).

#### Type.

MEXICO. Zacatecas or Nuevo León, *K.T. Hartweg* 97 (holotype K000612741, isotypes BM, BR, E, GH. LD, NY, OXF, P).

#### Description.

Rhizomatous perennial with a stout woody base; stems decumbent to at least 1 m, herbaceous, glabrous. Leaves shortly petiolate, 8–18 × 0.5–4 cm, lanceolate or oblong-lanceolate, acuminate, base cuneate, glabrous; petioles 0.5–2 cm. Inflorescence of solitary (rarely paired), axillary flowers, peduncles 3–6(–16) cm; bracteoles c. 1 mm, elliptic, scarious, caducous; pedicels 13–21 mm; sepals very unequal, coriaceous, glabrous, margins scarious, outer 10–16 × 6–7 mm, oblong-elliptic, mucronate to retuse, inner 16–23 × 7–8 mm, obovate, rounded; corolla 6.5–11 cm long, funnel-shaped, white with pink throat, glabrous, limb 4–5 cm diam., lobed with apiculate lobes. Capsules 2 × 1.5 cm, ovoid, rostrate, the mucro 3–5 mm, glabrous; seeds 11 × 5 mm, black, glabrous but except for the pilose margins with hairs 3–4 mm long.

#### Illustration.

Carranza (2007: 66).

#### Distribution.

Desert grasslands and dry oak woodland in northern Mexico and the United States Southwest.

**MEXICO. Aguascalientes**: Asientos, al W. del Polvo, *G. García* 4205 (IEB). **Chihuahua**: Urique, Kirare, *P. Tenorio et al.* 9944 (MO). **Durango**: *E. Palmer* 229 (BM, K); Victoria, *F. Shreve* 9169 (ARIZ). **Guanajuato**: Mun. San Miguel Allende, *R.B. Brown* 82-23 (ARIZ); San Felipe, *R. & J.D. Galván* 2260 (IEB), 2674 (IEB). **Jalisco**: Logas de Moreno, *R. Pearce* 2266 (ARIZ). **Querétaro**: 2 km N of El Sauz, *R. Pearce* 2245 (ARIZ); Matancillas, *P. Carillo-Reyes et al.* 509 (IEB). **Sonora**: Mun. Nacozari, *R. Felger* 3653 (ARIZ); Imuris, *S. Doan et al.* 1207 (ASU, DES). **Zacatecas**: NE Zacatecas, *J. Henrickson* 6665 (ARIZ); ibid., *R.G. Engard & H.S. Gentry* 705 (DES).

**UNITED STATES. Arizona**: Nogales, *R.H. Peebles et al.* 4613 (K); Cochise Co., Dragoon Mts, *D & S. Austin* 7582 (ARIZ). **New Mexico**: *C. Wright* 1617 (K).

#### Note.

*Ipomoea longifolia* might be confused with *Ipomoea leptophylla* but the sepals of *I. longifolia* are very unequal and much longer.

• The remaining species in Clade A1 (Species 94–127) include two distinct clades (Species 98–108 and 117–126) inferred from a combination of molecular sequence data and morphology. All species (94–127) have a tendency towards woodiness, most obvious in the Arborescens Clade (Species 117–126). Many, but not all, species have hirsute sepals, strongly discolorous leaves and a tendency to develop inflorescences on leafy axillary shoots.

### 94. Ipomoea sulina

Taxon classificationPlantaeSolanalesConvolvulaceae

P.P.A.Ferreira & Miotto Kew Bull. 66(2): 290. 2011. (Ferreira and Miotto 2011: 290)

#### Type.

BRAZIL. Rio Grande do Sul, Itati, *P.P.A. Ferreira* 287 (holotype ICN; isotypes K, SP, LIL).

#### Description.

Perennial twining plant to 4 m, stems woody, grey-tomentose, somewhat glabrescent. Leaves long-petiolate, 7–23 × 6–22 cm, ovate, acute to acuminate, shortly mucronate, cordate, adaxially tomentellous, green, abaxially grey-tomentose; petioles 5–17 cm, tomentellous. Inflorescence of 1–8-flowered axillary cymes; peduncles 3–16 cm, pubescent; bracteoles 1–3 mm, lanceolate, caducous; secondary peduncles up to 4 cm; pedicels 10–30 mm, puberulent; sepals unequal, glabrous, outer 10–13 × 8–9 mm, broadly ovate, obtuse, inner 14–17 × 12 mm, broadly elliptic, rounded or emarginate, margins scarious; corolla 5–8 cm long, funnel-shaped, glabrous, white with purple throat, limb c. 6.5 cm diam. Capsules subglobose, shortly rostrate, glabrous; seeds glabrous with long marginal hairs.

#### Illustration.

Ferreira and Miotto (2011: 293).

#### Distribution.

Endemic to southern Brazil in Rio Grande do Sul and Santa Catarina Stares growing on the borders of *Araucaria* forest.

**BRAZIL. Rio Grande do Sul**: Taquara, *B. Rambo* 44809 (LIL, PACA); ibid., *B. Rambo* 52115 (LIL, PACA, S). **Santa Catarina**: Itapiranga, *B. Rambo* s.n. (PACA) fide Ferreira and Miotto (2011).

#### Notes.

Resembles *Ipomoea philomega* in the large leaves and in the size and shape of sepals but differs in the hirsute, abaxially grey-tomentose leaves. The strikingly unequal sepals are noteworthy. It was identified as *Ipomoea viridis* Choisy by O’Donell but does not seem to fit the protologue.

Its placement here is uncertain.

### 95. Ipomoea killipiana

Taxon classificationPlantaeSolanalesConvolvulaceae

O’Donell, Lilloa 23: 486. 1950. (O’Donell 1950b: 486)

#### Type.

COLOMBIA. Meta, Villavicencio, road to Restrepo, *H. Schieffer* 833 (holotype US00111409; isotypes GH, LIL, UC).

#### Description.

Twining perennial, the stems glabrous except some pubescence at the nodes. Leaves petiolate, 3–10 × 2.5–11 cm, deeply 5–7–partite, segments oblong, 6–11 mm wide, acuminate and mucronate, scarcely narrowed at base, base shallowly and broadly cordate, adaxially thinly but shortly hispid–pilose, abaxially paler, nerves prominent puberulent; petioles 1.5–3.8 cm, thinly pubescent at base and apex, the abaxial surface pubescent on the veins. Inflorescence of few-flowered, axillary cymes, peduncles 3–3.5 cm, pubescent; bracteoles oblong, 12–15 × 3–4 mm, papery, caducous; secondary peduncles c. 2.5 cm long; pedicels 6–11 mm, pubescent; sepals somewhat unequal, papery, glabrous, the margins narrow and scarious, outer 16–18 × 7–8 mm, oblong-elliptic, subacute, mucronate, inner oblong, 4–5 mm wide; corolla 6 cm long, purple, glabrous, funnel-shaped, limb c. 4 cm diam., entire. Capsules and seeds not seen.

#### Illustration.

Figure 61.

**Figure 61.**
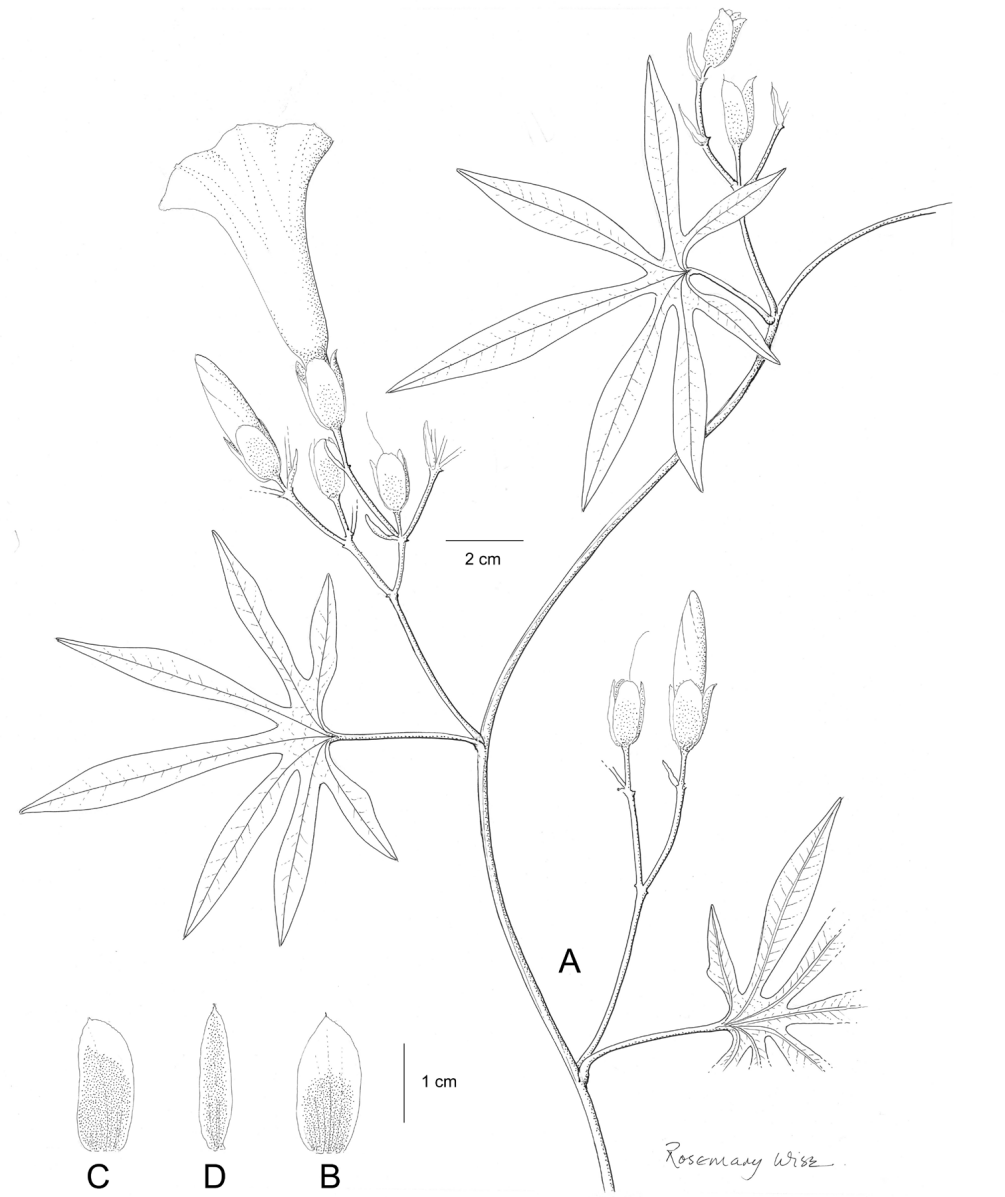
*Ipomoea killipiana*. **A** habit **B** outer sepal **C** middle sepal **D** inner sepal. Drawn by Rosemary Wise from *Schieffer* 833.

#### Distribution.

On cliffs at low altitudes, apparently rare.

**COLOMBIA. Meta**: P.N. Sierra de la Macarena, *R. Callejas* 6484 (MO).

**VENEZUELA. Barinas**: carretera a Pedraza, *L. Aristeguieta* 7993 (MO), fide Austin.

#### Notes.

This species is distinguished by the large foliaceous sepals. Its placement here is uncertain.

There is also a record from French Guiana (Funk et al. 2007: 272) but we have not traced a specimen and its presence there or elsewhere in the Guianas is unconfirmed.

*D. Cardénas et al.* 6498 (COAH, MO) from Serranía La Lindosa, Guaviare, appears to be *Ipomoea killipiana* but all parts are much smaller than in the type described above and the whole appears much more slender; the largest leaves are only 3.7 cm long and the outer sepals 12 × 4 mm. With so little material available it is difficult to be certain which form is most characteristic, if indeed they both belong to the same species.

### 96. Ipomoea cavalcantei

Taxon classificationPlantaeSolanalesConvolvulaceae

D.F. Austin, Acta Amazonica 11(2): 292. 1981. (Austin 1981: 292)

#### Type.

BRAZIL. Pará. Marabá, Serra dos Carajas, 6°00'S, 50°18'W, 700 m, 21 May 1969, *P. Cavalcante* 2086 (holotype MG36666, isotype F).

#### Description.

Scrambling shrub to 1.5 m; stems woody, pubescent when young but glabrescent. Leaves shortly petiolate, 5–10 × 1–2.5 cm, oblong, apex obtuse, shortly mucronate, base broadly cuneate, adaxially shortly pubescent, abaxially paler, the veins highlighted with pale dense pubescence, the intercostal areas nearly glabrous; petioles 0.4–1.5 cm, pubescent. Inflorescence elongate, formed of 1–5-flowered cymes in the leaf axils; peduncles 5–10 mm, pubescent; bracteoles caducous, subulate, c. 2 mm long; secondary peduncles 3–4 mm, often absent; pedicels 5–18 mm, less pubescent than peduncles; sepals subequal, 10–12 × 5 mm, oblong-elliptic, mucronate, outer, densely pubescent esp. towards apex, inner similar but with broad, glabrous margins; corolla vermillion, pubescent esp. on midpetaline bands, hypocrateriform, basal tube 3–3.2 cm long, 3–4 mm wide at base, 6 mm above, limb spreading, c. 3 cm diam., unlobed but midpetaline bands ending in hairy point, stamens exserted, anthers narrowly oblong c. 3.5 mm. Capsules and seeds not seen.

#### Illustration.

Figure 62.

**Figure 62.**
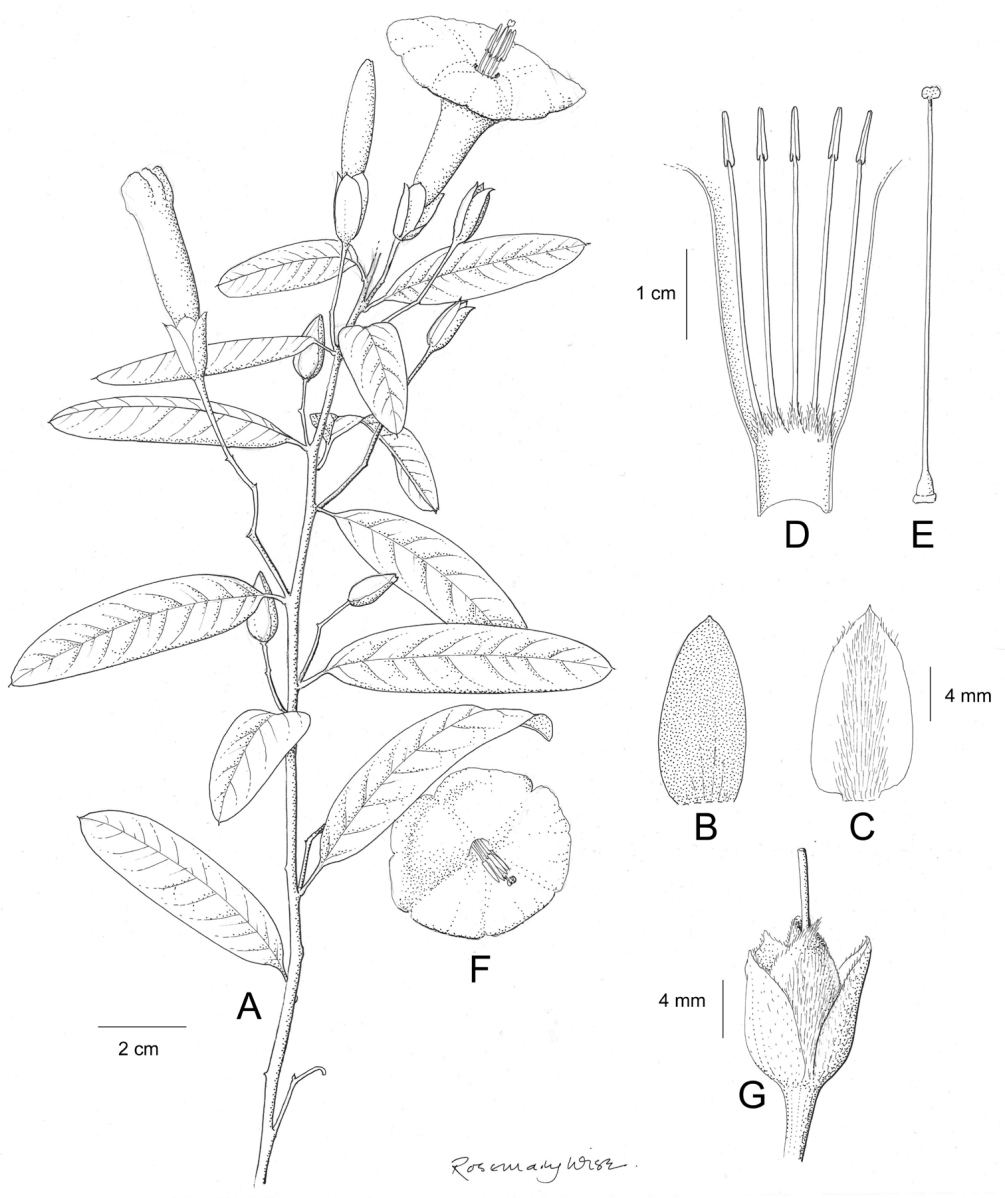
*Ipomoea cavalcantei***A** habit **B** outer sepal **C** inner sepal **D** corolla opened out to show stamens **E** ovary and style **F** corolla **G** calyx in fruit. Drawn by Rosemary Wise **A–C** G from *Sperling et al.* 5584; **D, E** from *dos Santos et al.* 573; **F** from photo.

#### Distribution.

Endemic to NE Brazil, growing in scrub around rock outcrops principally on or near the Serra de Carajás.

**BRAZIL. Pará**: Serra de Carajás, *M.G. Silva & R. Bahia* 2911 MG, FTG, RB); ibid., Serra Norte, *P. Cavalcante & M. Silva* 2651 (MG); ibid., *C.R. Sperling et al.* 5584 (MO); ibid., *H.C. de Lima* 7099 (RB); Mun. Itaituba, estrada Santarém–Cuiabá, BR 163, km 816, Serra do Cachimbo, *I.L. Amaral et al.* 1028, (FTG). **Tocantins**: Mun. Tocantinopolis, Ribeiro do Corrego, along Belem–Brasilia highway, *T. Plowman et al.* 9250 (MG, FTG).

**Notes**. The erect habit, oblong, shortly petiolate leaves combined with the hypocrateriform vermilion corolla make this species very distinct.

A hybrid between this species and *Ipomoea marabaensis* is recorded and illustrated by Simão-Bianchini et al. (2016: 1311).

### 97. Ipomoea marabaensis

Taxon classificationPlantaeSolanalesConvolvulaceae

D.F. Austin & R. Secco, Bol. Mus. Paraense “Emilio Goeldi”, n.s., Bot. 4(2): 188. 1988. (Austin and Secco 1988: 188)

#### Type.

BRAZIL. Pará, Marabá, Carajás, Serra Sul, 16 April 1986, *R.S. Secco et al.* 708 (holotype MG131894).

#### Description.

Erect or clambering shrub, rootstock moderately stout, spreading horizontally with a rhizome or similar structure, stems adpressed pilose or glabrous, woody at least below. Leaves very shortly petiolate, 5–12 × 0.3–2 cm, oblong or lanceolate, apex acuminate, obtuse and mucronate, base cuneate, glabrous (juveniles pubescent) on both surfaces, margins sometimes inrolled, abaxially paler the midrib and side veins highlighted by pubescence. Inflorescence of 1(–3)-flowered axillary cymes from the upper leaf axils, peduncles 2–4 mm, pubescent; bracteoles filiform, 3 mm, caducous; sepals subequal, 12–15 × 5–6 mm, ovate, obtuse, outer thinly pubescent; inner densely white-tomentose, rounded, the margins scarious but hirsute; corolla 6.5–8 cm long, funnel-shaped, deep lilac, densely pubescent on midpetaline bands; limb 3.5 cm diam. Capsules ovoid, glabrous; seeds 5–8 mm, woolly on margins.

#### Illustration.

Figure 63.

**Figure 63.**
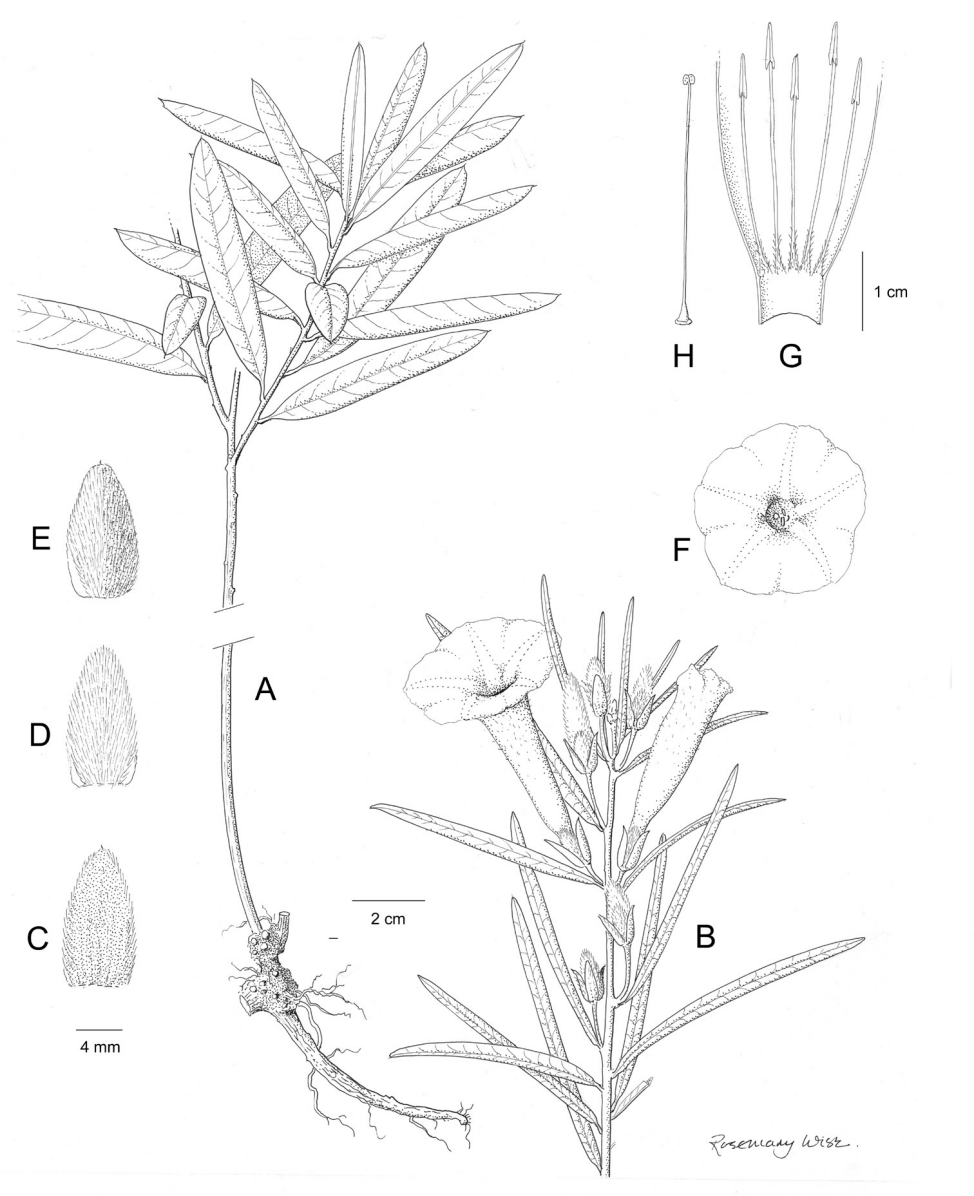
*Ipomoea marabaensis*. **A** habit **B** habit with inflorescence **C** outer sepal **D** middle sepal **E** inner sepal **F** corolla **G** corolla opened out to show stamens **H** ovary and style. Drawn by Rosemary Wise **A–E, G–H** from *Silva et al.* 1773; **F** from photo by R. Harley.

#### Distribution.

Perhaps endemic to the Serra dos Carajás in Brazil, although it is cited for Tocantins by Simão-Bianchini et al. (2016).

**BRAZIL. Pará**: Marabá, Serra dos Carajás, 700–750 m, *A.S.L. da Silva et al. 1773* (MG, MO, NY); *R.S. Secco & R.P. Bahia* 730 (MG); Canaa dos Carajas, *V.T. Giorni et al.* 144 (RB). Mun. Tucuruí, Represa Tucuruí, *T. Plowman et al*. 9610 (FTG); ibid., *T. Plowman et al.* 9771 (FTG).

#### Note.

The correct spelling should be marabaensis. “marabensis” (sic) at the start of the protologue would be an error. It is the only occurrence (out of 11) in the paper where the spelling “marabaensis” is not used.

• Species 98–108 form a clade in the phylogeny inferred from 605 nuclear gene regions.

### 98. Ipomoea calyptrata

Taxon classificationPlantaeSolanalesConvolvulaceae

Dammer, Bot. Jahrb. Syst. 23, Beibl. 57: 40. 1897. (Dammer (1897: 40)

#### Type.

BRAZIL. Minas Gerais, near Arrasnaby, *A.F.M. Glaziou* 15265 (holotype B†, isotypes K000612835, P03878984, R).

#### Description.

Vigorous liana reaching 5 m, stems woody, subtomentose. Leaves petiolate, large, 4–16 × 5–14 cm, ovate to subreniform, apex rounded or retuse, mucronulate, base shallowly cordate to subtruncate, margin slightly undulate. adaxially grey-tomentellous, abaxially white-tomentose with conspicuous reticulate venation; petioles 1.5–5 cm, tomentose. Inflorescence of few-flowered pedunculate, axillary cymes; peduncles 3–5 cm, tomentose; bracteoles 20 × 4 mm, oblanceolate, abaxially grey-tomentose, adpressed to calyx; secondary peduncles 10–15 mm; pedicels 2–7 mm, tomentose; sepals subequal, 14–22 × 8–14 mm, oblong-obovate, rounded, densely white tomentose; corolla 6–7 cm long, pink, funnel-shaped, densely sericeous, limb 7 cm diam., unlobed. Capsules 2 × 1.5 cm, ovoid, glabrous; seeds pilose with long white hairs.

#### Illustration.

Figures 52A, 64.

**Figure 64.**
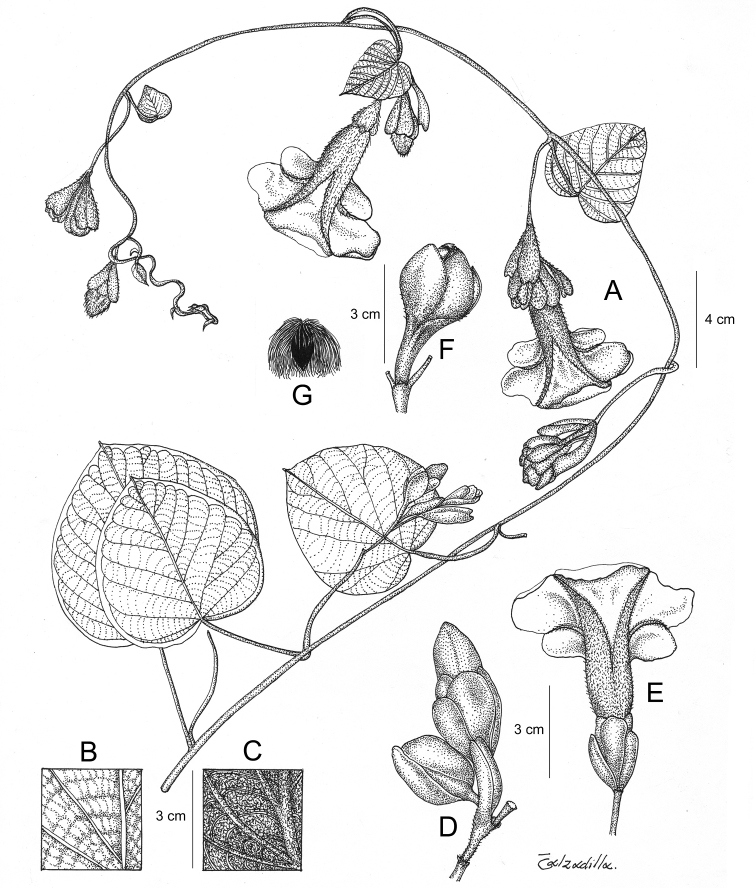
*Ipomoea calyptrata*. **A** habit **B** adaxial leaf surface **C** abaxial leaf surface **D** inflorescence showing bracteoles and calyx **E** flower showing arrangement of bracteoles and sepals **F** calyx in fruit **G** seed. Drawn by Eliana Calzadilla **A–D** from *Wood et al.* 21446; **E** from photo; **F, G** from *Nee* 49257.

#### Distribution.

A rare plant of Brazil and Bolivia. In Bolivia it is characteristic of very dry forest between 1400 and 2000 m on the slopes of the Río Grande valley and its tributaries. Although large and conspicuous, there are few collections and it is clearly rare with a very restricted distribution. The only confirmed record from Brazil is the type collection.

**BRAZIL. Minas Gerais**: Type of *Ipomoea calyptrata*.

**BOLIVIA. Chuquisaca**: Boeto, below Nuevo Mundo, *J.R.I. Wood et al.* 20496 (BOLV, HSB, K, LPB); *J.R.I. Wood et al.* 27659 (OXF, K, LPB, USZ). **Santa Cruz**: Saipina, *J. Balcazar 354* (OXF, LPB, MO, USZ); Alto Mairana, *M. Nee* 49257 (NY, USZ); between Pucara and Santa Rosita, *J.R.I. Wood & M. Mendoza* 21472 (K, LPB, USZ).

#### Notes.

A very distinctive species because of its liana habit, persistent bracteoles appressed to the calyx, large tomentellous sepals and pink flowers. The whole plant is subtomentose with whitish hairs. The very long sepals (14–22 mm in length) serve to distinguish it from other somewhat similar species, such as *Ipomoea brasiliana*.

*A.L. Brochado* 154 (IBGE, OXF) from P.N. Das Emas, Goiás may constitute the first modern collection from Brazil. However, although the inflorescence is similar, the leaves lack the characteristic reticulate venation on the underside.

### 99. Ipomoea veadeirosii

Taxon classificationPlantaeSolanalesConvolvulaceae

J.R.I. Wood & Scotland, Phytokeys 88: 30. 2017. (Wood et al. 2017d: 30)

#### Type.

BRAZIL. Goiás, Chapada de Veadeiros, 42 km N. of Alto do Paraíso, *H.S. Irwin, R.M. Harley & G.L. Smith* 33148 (holotype FTG, isotype ?NY, n.v.).

#### Description.

Twining liana to c. 3 m; stem stout, somewhat woody, densely tomentose. Leaves petiolate, 5–11 × 4–9 cm, ovate, shallowly cordate to subtruncate with rounded auricles, margin undulate, apex obtuse and shortly mucronate, the mucro rather stout, adaxially yellow-green, tomentose, glabrescent when old, abaxially grey-tomentose, the veins highlighted; petioles 0.5–4 cm, tomentose. Inflorescence of flowers borne on axillary bracteate branchlets; bracts 2–2.5 × 1–1.7 cm, ovate, tomentose; cymes 1–2-flowered; peduncles 1–6 cm, tomentose; secondary peduncles pedicel-like, 0.8–1.7 cm, pubescent, more slender than primary peduncles; bracteoles 2–2.3 × 0.8–1.4 cm, narrowly elliptic, obtuse, somewhat boat-shaped, tomentose, persistent and ± clasping the calyx; pedicels 1–4 mm, glabrous; sepals subequal, 11–13 × 5–7 mm, elliptic, obtuse to rounded, outer glabrous, margins scarious; corolla 6–7 cm long, narrowly funnel-shaped, glabrous, deep pink. Capsules and seeds unknown.

#### Illustration.

Figure 65.

**Figure 65.**
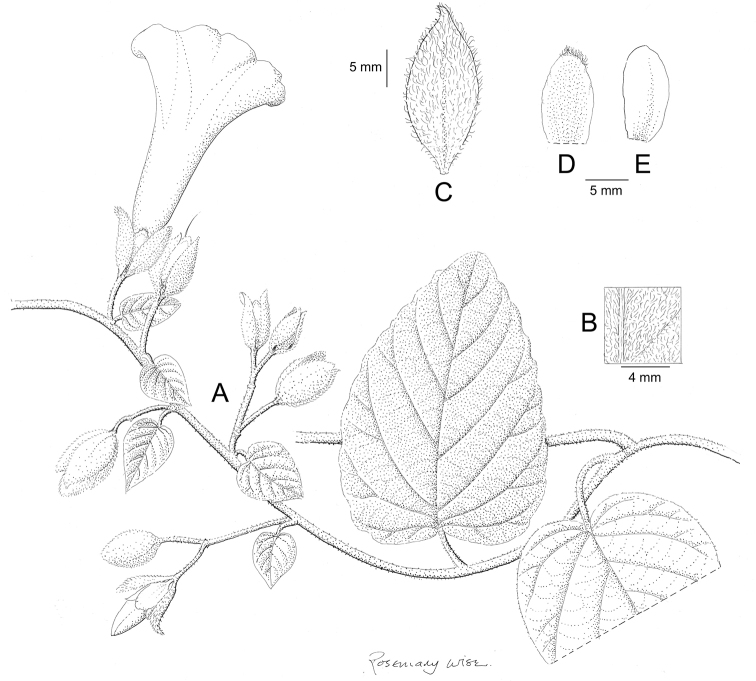
*Ipomoea veadeirosii*. **A** habit **B** abaxial leaf surface **C** bracteole **D** outer sepal **E** inner sepal. Drawn by Rosemary Wise **A, B** from *H.S. Irwin et al.* 33148; **C–E** from *W.R. Anderson et al.* 6691.

#### Distribution.

Endemic to rocky cerrado (campo rupestre?) at 1250–1700 m in the Chapada de Veadeiros in central Brazil.

**BRAZIL. Goiás**: Chapada de Veadeiros, 25 km N of Alto Paraíso, 1700 m, *W.R. Anderson et al. 6691* (FTG, ?NY, n.v.).

#### Note.

Although we have not been able to sequence this species, *Ipomoea veadeirosii* appears to belong to the small clade where *Ipomoea descolei* O’Donell and *I. calyptrata* Dammer belong. All these species are somewhat woody and liana-like and share a densely tomentose indumentum. The inflorescence structure with a tendency for the inflorescence to develop on foliose branchlets is found in a number of woody species, notably the *Arborescens* group. *Ipomoea veadeirosii* appears closest to *I. calyptrata* because of the persistent bracteoles which are appressed to the calyx with the pedicel supressed. It differs most obviously in the glabrous corolla, near glabrous sepals and the roughly tomentose indumentum, which differs from the white tomentellous indumentum of the stem, leaves, bracteoles, sepals and corolla exterior of *Ipomoea calyptrata*.

### 100. Ipomoea descolei

Taxon classificationPlantaeSolanalesConvolvulaceae

O’Donell, Lilloa 23: 440. 1950. (O’Donell 1950a: 440)

Argyreia hirsuta Hook., Bot. Mag. t. 4940. 1856. (Hooker 1856: t. 4940), nom. illeg., non Argyreia hirsuta Wight & Arn. (1837). Type. t. 4940 in Bot. Mag. (lectotype, designated here).
Argyreia choisyana [Hort. [Paris] ex Regel & Körn., Index Seminum (St. Petersburg) 1858: 40. 1859. (Regel and Kornikoff 1859: 40), non Ipomoea choisyana Wight ex C.B. Clarke (1883). Type. Based on Argyreia hirsuta Hook.

#### Type.

ARGENTINA. Misiones, Dept. San Ignacio, *G.J. Schwarz* 3472 (holotype LIL001238).

#### Description.

Perennial herb from a tuberous rootstock, stems stout, decumbent (occasionally twining at tips), densely tomentose with yellowish or whitish hairs. Leaves petiolate, 7–18 × 6–16 cm, ovate, cordate with rounded auricles, apex obtuse and mucronate, margins undulate, dentate or sinuate, adaxially yellow-green, tomentose, abaxially grey-tomentose, the venation highlighted; petioles 2–12 cm, tomentose. Inflorescence of long-pedunculate, few-flowered axillary cymes; peduncles 5–20 cm, tomentose; bracteoles 10–25 × 2–4 mm, linear-lanceolate, attenuate, tomentose, caducous; secondary peduncles, if present 20–27 mm; pedicels 5–20 mm, densely pilose; sepals slightly unequal, outer 12–16 × 7–9 mm, elliptic, acute and mucronate, densely pilose; inner 5–6 mm wide, oblong-elliptic, obtuse, margins broad, glabrous, scarious; central area pilose; corolla 8–9 cm long, funnel-shaped, pink, pilose with yellowish hairs; limb 5 cm diam. Capsules 11–13 mm long, ellipsoid to subglobose, glabrous; seeds 7–8 × 5 mm, densely tomentellous.

#### Illustration.

**Figure 66.**
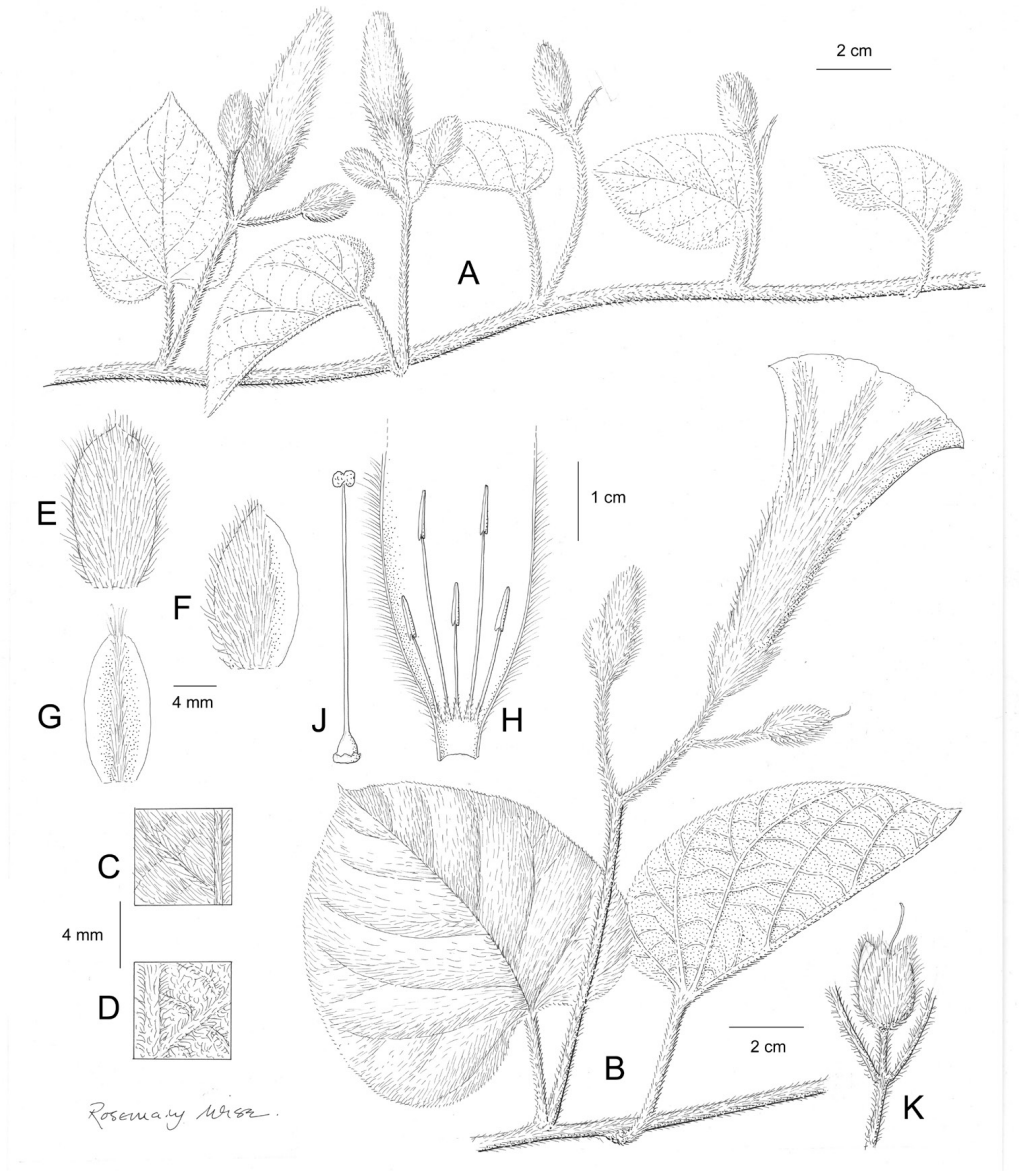
*Ipomoea descolei*. **A** habit from apex of stem **B** habit from near base of stem **C** adaxial leaf surface **D** abaxial leaf surface **E** outer sepal **F** middle sepal **G** inner sepal **H** corolla opened out to show stamens **J** ovary and style **K** calyx in fruit. Drawn by Rosemary Wise from *Pedersen* 5453.

Figures 5A, 66; O’Donell (1959b: 149).

#### Distribution.

A plant of cerrado-like grassland, nearly endemic to Misiones and Corrientes provinces in NE Argentina. Records from Bolivia are errors.

**ARGENTINA. Corrientes**: Santo Tomé, *T.M. Pedersen* 5453 (E, K, S); *T.S. Ibarrola* 1275 (LIL, NY, S). **Misiones**: Candelaria, *M.E. Rodríguez* 01081 (CTES); *G.E. Barboza et al.* 419 (CORD, CTES, SI); *R. Vanni & Radovanovich* 1088 (CTES, K); *E. Ekman 1430* (S); *Medina* 148 (LIL, S)

**PARAGUAY. Itapuá**: Encarnación, Campo Cambyretá, *Pavetti & Rojas* 10896 (LIL).

**BRAZIL. Rio Grande do Sul**: *A. Sehnem* 3583 (SI).

#### Lectotypification.

*Argyreia choisyana* has been correctly identified with *Ipomoea descolei* (Austin et al. 2015). The specimen at St Petersburg (LE00009100) is not a very good match but is cited by Staples and Traiperm (2017: 470) as holotype of *Argyreia choisyana*. However, *Argyreia choisyana* is clearly based on the plate of *Argyreia hirsuta* in the Botanical Magazine (Hooker, WJ 1856: t. 4940), which was painted from a plant grown from seed sent from Paris as “*Argyreia choisyana*”. The plate in the Botanical Magazine looks a better match for *Ipomoea descolei* and is here selected as the lectotype of *Argyreia hirsuta* Hook., as no specimen has been traced at Kew. Seeds were clearly sent from Paris to London and St Petersburg but how they arrived at Paris is unknown. It is not entirely fanciful that the seeds were sent to Paris by Bonpland, who had settled at Corrientes after his release from imprisonment in Paraguay. He may well have seen the horticultural potential of this spectacular *Ipomoea*, which is endemic to the region.

#### Note.

A very distinctive species because of the dense yellowish indumentum that covers all parts, the tendency of the leaves to be undulate or sinuate-lobed and the trailing habit.

### 101. Ipomoea queirozii

Taxon classificationPlantaeSolanalesConvolvulaceae

J.R.I. Wood & L.V. Vasconc., Kew Bull. 72(8): 13. 2017. (Wood et al. 2017a: 13)

#### Type.

BRAZIL. Bahia: Barreiras, ca. 20 km W de Barreiras na estrada para Brasilia, 12°06'42"S, 45°09'47"W, 581 m, 13 April 2005, *L.P. de Queiroz, J.A.Costa, M.N. Stapf & E.B. Souza* 10239 (holotype HUEFS95041, isotype OXF).

#### Description.

Erect subshrub to 1 m from a stout taproot at least 15 cm deep and up to 1.5 cm wide, stems slightly woody, pubescent, glabrescent when old. Leaves very shortly petiolate, 3–18 × 0.3–1.4 cm, but becoming clearly bract-like and much smaller (to 3.5 × 0.3 cm) towards the apex, linear to oblong, finely acuminate to a mucronate apex (rarely obtuse and mucronate), base cuneate to attenuate, margins sometimes inrolled, adaxially almost glabrous apart from a few hairs on the midvein, abaxially grey-green, pubescent, somewhat glabrescent; petioles 0–8 mm, pubescent. Inflorescence terminal, formed of shortly pedunculate 1–3-flowered cymes from the upper leaf (bract)axils, the cymes often reduced to single flowers; peduncles 0.4–1 cm, pubescent; bracteoles 3–11 × 0.5 mm, linear-lanceolate, caducous; pedicels 2–12 mm, often very short upwards, pubescent; sepals subequal, outer 6–10 × 4–8 mm, oblong-elliptic, obtuse to rounded, usually glabrous, margin scarious; inner sepals 1–2 mm longer, obovate-elliptic, truncate or retuse; corolla 4–7 cm long, pink, glabrous, funnel-shaped, limb 3.5–5 cm diam., slightly undulate. Capsules and seed not seen.

#### Illustration.

Figure 67.

**Figure 67.**
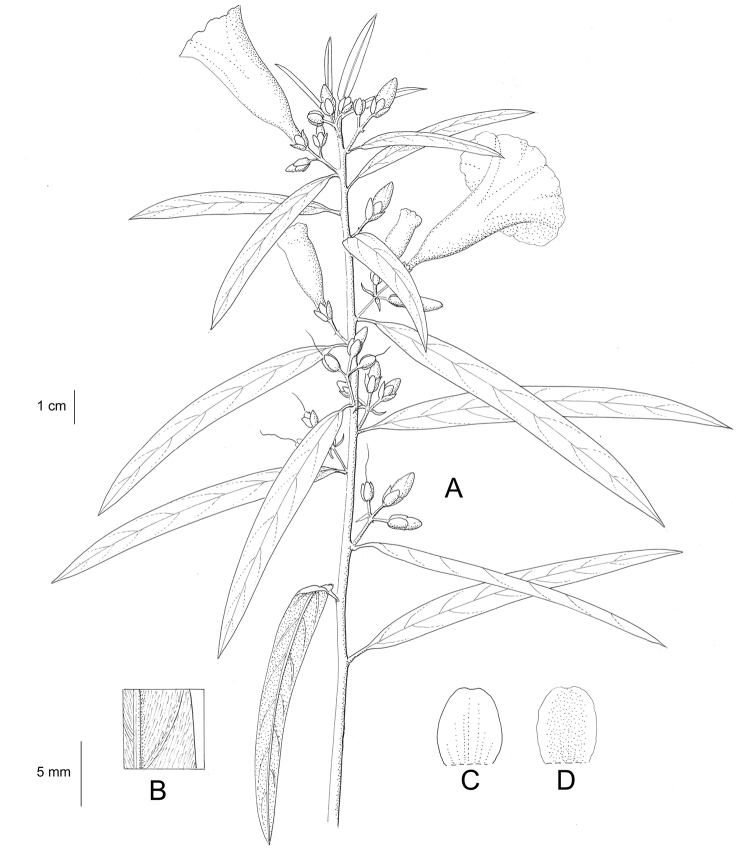
*Ipomoea queirozii*. **A** habit **B** abaxial leaf surface **C** outer sepal **D** inner sepal. Drawn by Rosemary Wise from *L.P. de Queiroz* 10239.

#### Distribution.

A cerrado species from the extreme west of Bahia and neighbouring parts of Tocantins State. It has been found at altitudes of between 500 and 760 m.

**BRAZIL. Bahia**: Valley of the Rio das Ondas, c. 10 km W of Barreiras, *H.S. Irwin et al.* 31335 (FTG); Espigão Mestre, 22 km W of Barreiras, *W.R. Anderson et al.* 36478 (FTG); Formosa do Rio Preto, 40 km da Faz. Estrondo en direção de Mimosa, *L.S. Guedes et al.* 6799 (CEN, HUEFS, RB). **Tocantins**: Dianápolis, distrito de Missões, 2 km de Missões, *R.M. Harley et al.* 56736 (HUEFS)

#### Notes.

This species is similar to most other erect cerrado species in having shortly petiolate, oblong leaves and a subterminal inflorescence in which the reduced leaves clearly function as bracts. It is most likely to be confused with *Ipomoea paludosa*, *I. campestris* or *I. aprica* but is immediately distinguished from all of these and other similar species by the glabrous corolla. Most specimens also have glabrous sepals but *Anderson et al.* 36640 is anomalous for having pubescent sepals. Molecular studies suggest a relationship with *Ipomoea pohlii* Choisy but this also has a pubescent corolla and differs additionally in its solitary flowers which are partially concealed by the relatively large bracts.

As understood here this is a variable species. All cited collections are ±hirsute on the stems, abaxial leaf surfaces and on the peduncles. *De Queiroz et al.* 10239, *Irwin et al.* 31335 and *Anderson et al.* 36478 are outstanding for their branched terminal inflorescence which appears paniculate, whereas in the other collections the flowers are mostly solitary so the inflorescence appears to be a leafy raceme. *Guedes et al.* 6799 is itself somewhat variable with the specimen at CEN having shorter and more obtuse leaves than those at HUEFS and RB.

Two specimens from Minas Gerais are not cited above but may belong to this species. They differ in being completely glabrous and having somewhat granulose stems. Further collections may show that *Ipomoea queirozii* is more variable than described above or may justify recognising the following as a distinct subspecies or even species:

**BRAZIL. Minas Gerais**: Serra do Cipó, c. 145 km N of Belo Horizonte, 1200 m, 15 Feb. 1968, *H.S. Irwin et al.* 20103 (FTG); Canastra, Serra da Babilonia, entre Delfinópolis e São Roque de Minas, 10 Feb. 2012, *J.F.B. Pastore et al.* 3990 (HUEFS).

### 102. Ipomoea neriifolia

Taxon classificationPlantaeSolanalesConvolvulaceae

Gardner, Icon. Pl. t. 471. 1842. (Gardner 1842a: t. 471)

#### Type.

BRAZIL. Goiás, Serra de Natividade, Feb. 1840, *G. Gardner* 3906 (holotype K000612792, isotype BM).

#### Description.

Erect undershrub to 40 cm from a xylopodium, stems distinctly woody, villous when young but eventually glabrescent. Leaves sessile, imbricate, 5.5–12 × 0.3–0.5 cm, linear or narrowly oblong, acute, margins inrolled, thinly pilose, especially below and on veins, thinly punctate on both surfaces. Inflorescence terminal formed of small cymes and individual flowers from the upper leaf axils; peduncles very short, 1–5 mm, villous; bracteoles caducous; pedicels 3–10 mm; sepals subequal, shotly mucronate, but mucro somewhat caducous, outer 7–8 × 4–5 mm, oblong-elliptic, obtuse to subacute, pubescent, inner 8–9 × 5 mm, elliptic, rounded, mucronate, margins scarious, only midrib puberulent; corolla 4–7 cm long, pink, funnel-shaped, glabrous, limb 3–5 cm diam. Capsules and seeds unknown.

#### Illustration.

Figures 8M, 68.

**Figure 68.**
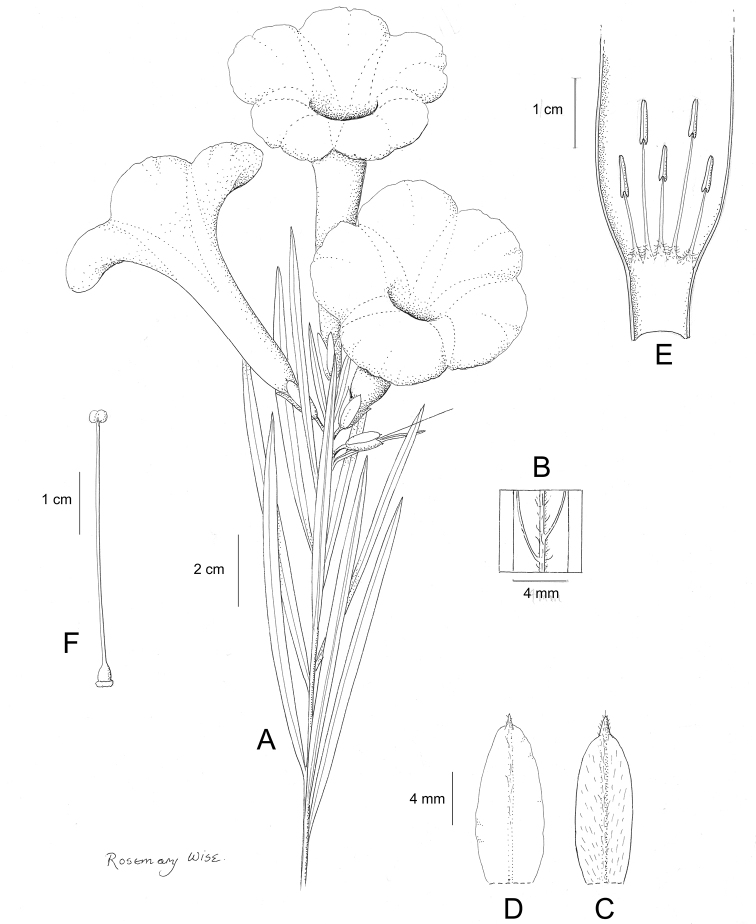
*Ipomoea neriifolia*. **A** habit **B** abaxial leaf surface **C** outer sepal **D** inner sepal **E** corolla opened up to show stamens **F** ovary and style. Drawn by Rosemary Wise from *Rezende et al.* 1011.

#### Distribution.

A rare Brazilian endemic species of cerrado.

**BRAZIL. Bahia**: Espigão Mestre, ca. 100 km WSW of Barreiras, 760 m, *W.R. Anderson et al.* 36640 (FTG). **Goiás**: Type of *Ipomoea neriifolia*. **Tocantins**: Parque Estadual do Jalapão *J.M. Rezende et al.* 1011 (CEN).

#### Note.

This is close to *Ipomoea queirozii* differing in the shorter, broader pubescent sepals.

### 103. Ipomoea pohlii

Taxon classificationPlantaeSolanalesConvolvulaceae

Choisy in A.P. de Candolle, Prodr. 9: 355. 1845. (Choisy 1845: 355)

Ipomoea angustisepala O’Donell, Lilloa 26: 362. 1953. (O’Donell 1953a: 362). Type. BRAZIL. Goiás: Upland and campo near Pose, *G. Gardner* 4292 (holotype K000612839).

#### Type.

BRAZIL. *J.B. Pohl* s.n. (lectotype BR00005307708, designated here; isolectotypes BR, K, M, F (photo of specimen formerly at B).

#### Description.

Erect undershrub, presumably from a xylopodium to at least 1 m, stem tomentose to pubescent, woody below. Leaves subsessile, imbricate, 1.5–6 × 0.5–2.5 cm, oblong, oblong-ovate, acute and mucronate, rounded to truncate at base, variably hirsute from grey-villous to pubescent, paler beneath; petioles 0–3 mm. Inflorescence a short terminal bracteate raceme, flowers solitary in axils of bracts; bracts ±distinct from leaves, typically half the size of the upper leaves; peduncles 1–2 mm; bracteoles linear-lanceolate,15–20 × 3 mm, densely villous; pedicels 1–2 mm, villous; sepals subequal, 15–17 × 2–4 mm, lanceolate with a finely attenuate apex, villous; corolla 5–7 cm long, pink, pubescent, funnel-shaped; limb c. 4 cm diam. Capsules and seeds not seen.

#### Distribution.

Endemic to cerrado in Brazil where it is restricted to Bahia and Goiás.

**BRAZIL. Bahia**: Chapada Occidental de Bahia, 15 km N of Correntina, *R.M. Harley et al.* 21765 (K); 20 km N of Correntina, *R.M. Harley et al.* 21902 (CEPEC, K); 36 km SW of Correntina, *A. Krapovickas* 30171 (CTES, F); Mun. Barreiras, *G. Hatschbach* 42032 (CEPEC, FTG, MBM); San Desidério, *E. Melo et al.* 8179 (HUEFS).**Goiás**: *R.M. Harley et al.* 28588 (SP); Serra Geral de Goiás, *H.S. Irwin et al.* 14381 (NY); São Domingos, *C. Cristóbal & A. Krapovickas* 692 (CTES).

#### Lectotypification.

In choosing a lectotype we have designated the specimen at BR as it is the only syntype from Martius’ herbarium with the location included on the label.

### 104. Ipomoea magna

Taxon classificationPlantaeSolanalesConvolvulaceae

Sim.-Bianch & J.R.I. Wood. Kew Bull. 72 (8): 18. 2017. (Wood et al. 2017a: 18)

#### Type.

BRAZIL. Minas Gerais, 13 km W of Januária on road to Serra das Araras, 575 m, 19 April 1973, *W.R. Anderson*, *P. A. Fryxell, S.R. Hill, R. Reis dos Santos & R. Souza* 9184 (holotype UB, isotypes FTG, NY).

#### Description.

Liana reaching at least 10 m, stems twining, woody, tomentose, latex white. Leaves petiolate, 8–28 × 7–22 cm, ovate, cordate with rounded auricles, apex acute or obtuse and shortly mucronate, margin slightly undulate, adaxially green, roughly tomentellous, abaxially grey-tomentose with highlighted veins; petioles 5–11 cm, tomentose. Inflorescence of axillary cymes wth up to seven flowers; peduncles 2.5–10 cm, tomentose; bracteoles (8–)12–18 × 4–7, oblong or oblong-obovate, obtuse, glabrous, caducous; secondary peduncles 4–23 mm, thinly pubescent; pedicels 10–30 mm, thickened upwards, glabrous; sepals subequal, 12–19 × 9–12 mm, accrescent in fruit to 25 × 14 mm, elliptic to obovate, rounded, glabrous on the exterior but scurfy-pubescent on the interior, inner with narrow scarious margins, slightly larger; corolla 8–12 cm long, funnel-shaped, pale pink on exterior, darker inside tube, glabrous, limb 6–8 cm diam.; anthers and style included. Capsules c. 21 × 12 mm, ellipsoid, glabrous; seeds 12 × 6 mm, pilose on angles with long white hairs up to 20 mm in length.

#### Illustration.

Figure 69.

**Figure 69.**
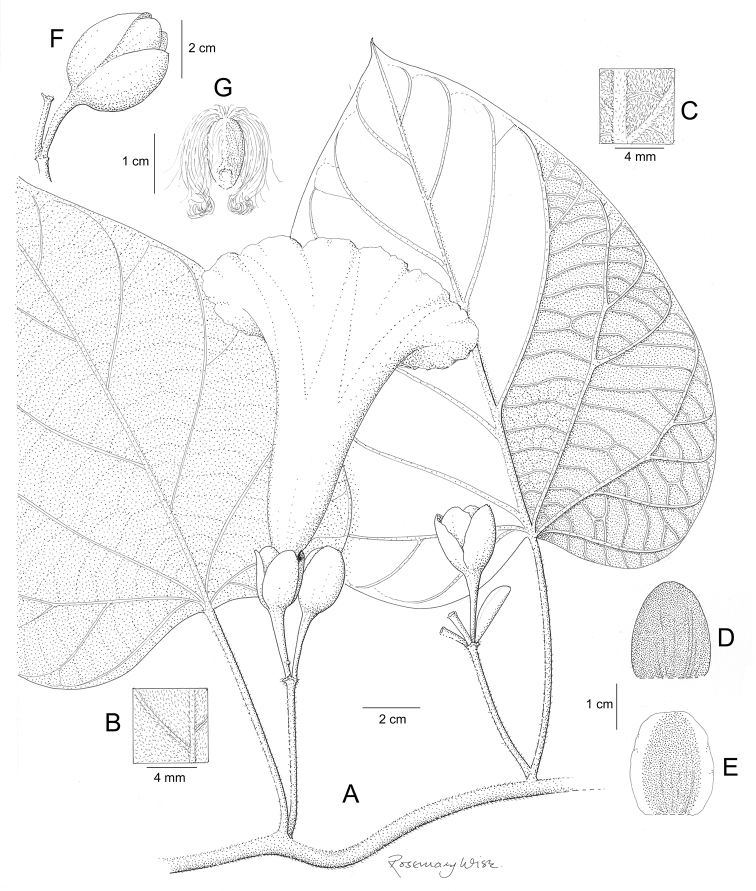
*Ipomoea magna*. **A** habit **B** adaxial leaf surface **C** abaxial leaf surface **D** outer sepal **E** inner sepal **F** calyx in fruit **G** seed. Drawn by Rosemary Wise **A, F, G** from *W.R. Anderson et al.* 10183; **B, C** from *S. A. Mori et al.*; **D, E** from *T. Jost et al.* 508.

#### Distribution.

Centred on Bahia State, Brazil this species is widespread on the borders of scrub and woodland at the transition from the cerrado to caatinga biomes. There is a smaller disjunct population on the borders of Paraguay and Mato Grosso do Sul state.

**PARAGUAY. Amambay**: P.N. Cerro Corá, *J. Fernández Casas & J. Molero* 6141 (MA, G, MO); ibid., *W. Hahn* 1746 (MO, PY); NE of park headquarters, *J.C. Solomon et al.* 7082 (MO, PY); Cerro Sarambí, 20 km from P.N. Cerro Corá, *S. Keel & L. Spinzi* 1833 (FCQ). Concepción: 20 km N of Ybyau, *N. Soria* 5176 (FCQ).

**BRAZIL. Bahia**: Faz. de Cova, *E. Pereira & G. Pabat* 8566 (F); Mun. Maracás, 13–15 km SW of Maracas, *S. A. Mori et al.* 9985 (MO, NY); Reandi, 15–19 km, estrada Urandi-Licinio de Almeida, *T. Jost et al.* 508 (IPA); Mun. Caetité, camino da Faz. Boa Vista para Urânio, *E. Saar et al.* 5254 (ALCB, K). **Ceará**: Serra de Ararifé, *Gardner* 2030 (BM). **Goiás**: Serra Dourada, 6 km NE of Mossamedes, *W.R. Anderson* 10183 (FTG, NY). **Mato Grosso do Sul**: Mun. Bonito, *G. Hatschbach et al.* 74730 (MBM). **Minas Gerais**: 13 km W of Januária on road to Serra das Araras, *W.R. Anderson* 9184 (FTG, NY, UB); Cabeceira Grande, *G. Pereira-Silva et al.* 6398 (CEN).

#### Notes.

Resembling a giant form of *Ipomoea brasiliana* but immediately distinguished by the long hairs on the seeds as well as the larger dimensions of the leaves, sepals and corolla. It appears to be closely related to *I. longibracteolata* but is distinguished by the absence of long white hairs on the inflorescence, the laxer cymes and different-shaped corolla.

The populations from Paraguay and neighbouring Mato Grosso do Sul are poorly known but seem indistinguishable from the larger populations further north in Brazil.

### 105. Ipomoea longibracteolata

Taxon classificationPlantaeSolanalesConvolvulaceae

Sim.-Bianch. & J.R.I. Wood. Kew Bull. 72 (8): 15. 2017. (Wood et al. 2017a: 15)

#### Type.

BRAZIL. Bahia, Mun. Caetité, Faz. Baixa Grande, 14°04'03"S, 42°38'12"W, 820 m, 9 Feb. 1997, *M.L. Guedes, B. Stannard*, *E. Saar & L. Passos* 5276 (holotype HUEFS 28895, isotypes ALCB, CEPEC, HRB, K, SPF).

#### Description.

Liana with white latex reaching 10 m; stems woody, asperous-pilose, bark pale grey. Leaves petiolate, (7–)11–20 × (7–)14–20 cm, ovate, cordate with right-angled sinus and rounded auricles, apex acute, mucronate, sometimes retuse, adaxially thinly pubescent, abaxially paler, densely pubescent, the venation prominent with denser indumentum; petioles (4–)12–13 cm, pilose. Inflorescence of shortly pedunculate, bracteolate, axillary cymes; peduncles 1.5–8 cm, asperous-pilose; bracteoles 2–3 × 0.6–1.3 cm, often, boat-shaped, oblong-elliptic or narrowly obovate, base cuneate, apex obtuse, pilose with long white hairs; secondary peduncles (if present) 1–2 cm; pedicels 0.6–1.5 cm, more densely pilose than peduncles; sepals somewhat variable in size, shape and indumentum but generally unequal, outer 18–24 × (9–)14–16 mm, oblong-elliptic, elliptic, obovate, obtuse to rounded, glabrous or with some long white hairs along midrib on the exterior especially near base but glabrous and glandular on the interior, inner 17–18 × 7 mm, obovate, obtuse to rounded, glabrous; corolla 5–6.5 cm long, glabrous, broadly funnel-shaped to subcampanulate, tube, c. 2 cm wide from just above base pale pink with a dark centre and whitish limb; limb c. 3.5 cm diam. Capsules 2 × 1.5 cm, ellipsoid, glabrous; seeds 7 × 5 mm, densely white-pilose on angles with hairs to 15 mm long.

#### Illustration.

Figure 70.

**Figure 70.**
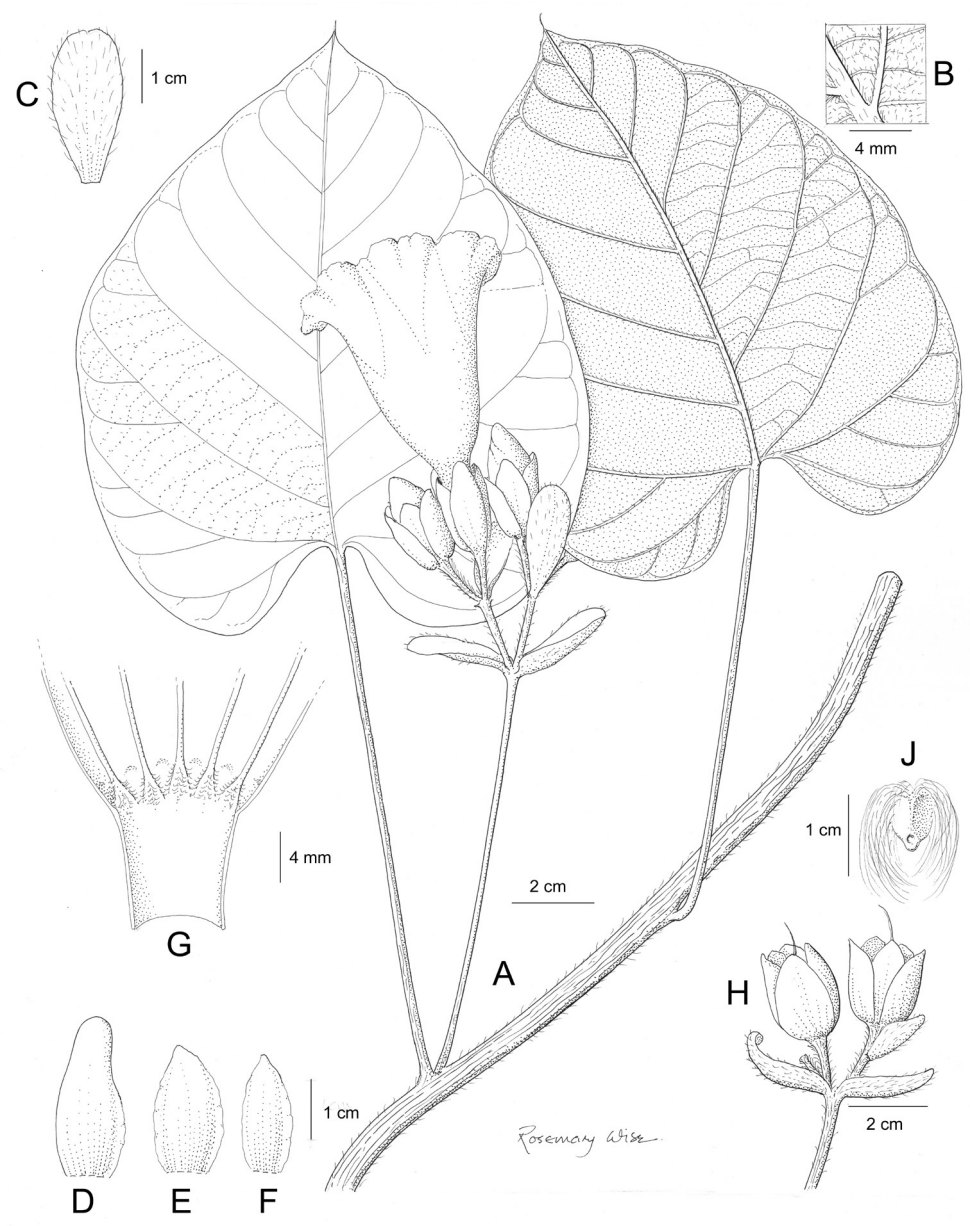
*Ipomoea longibracteolata*. **A** habit **B** abaxial leaf surface **C** bracteole **D** outer sepal **E** middle sepal **F** inner sepal **G** corolla opened up to show stamens **H** fruiting inflorescence showing indumentum and persistent bracteoles **J** seed. Drawn by Rosemary Wise **A, B** from *L.P. de Queiroz et al.* 5963; **C–G** from *L.P. de Queiroz et al.* 2607; **H–J** from *F. França et al.* 59231.

#### Distribution.

Dry scrub with scattered trees in cerrado or caatinga usually on sandy soil in northeastern Brazil.

**BRAZIL. Bahia**: Mun. Abaíra, *L.P. Queiroz et al.* 2607 (HUEFS); Santa Maria da Vitoria, *L.P. Queiroz et al.* 5963 (HUEFS, OXF); Mun. Caetité, *M.L. Guedes et al.* 5276 (ALCB, HUEFS, K); São Desidério, *J.G. de Carvalho-Sobrinho* 471 (HUEFS, OXF). **Goiás**: 15 km N de Alvaorada do Norte, *Hatschbach* 42017 (FTG, MBM); Mun. Nova Roma, *D. Alvarenga et al.* 1303 (IBGE, MO). **Minas Gerais**: Juiz de Fora, *A.F.M. Glaziou* 8821a (P); 1 km E of Rio Pandeiros, near road to Januaria, *W.R. Anderson et al.* 9100 (FTG, NY); Serra do Espinhaço, 5 km NE of Francisco Sá, road to Salinas, *H.S. Irwin et al.* 23210 (FTG, NY).

#### Note.

Distinguished by the relatively long bracteoles, the distinctive white, asperous-pilose indumentum, which is particularly prominent on the inflorescence, and by the characteristically compact inflorescence.

### 106. Ipomoea paradae

Taxon classificationPlantaeSolanalesConvolvulaceae

J.R.I. Wood & Scotland, Kew Bull. 70 (31): 69. 2015. (Wood et al. 2015: 69)

#### Type.

BOLIVIA. Santa Cruz. camino Algodonal a Masicurí, *G.A. Parada, M. Betancur & Y. Inturion* 3151 (holotype USZ, isotypes K, MO).

#### Description.

Liana reaching at least 5 m in height, stems woody, glabrous, obscurely ridged, bark pale brown. Leaves petiolate, 6–12 × 5.5–11 cm, ovate, obtuse and muconate, base cordate with rounded auricles, adaxially green, thinly pubescent, abaxially grey-tomentose with highlighted veins; petioles 3–5 cm, puberulent. Inflorescence of 1–5-flowered, axillary, pedunculate cymes; peduncles 1–3.5 cm, glabrous except for hairs apically; secondary peduncles 1–1.4 cm, pubescent; bracteoles 10–14 × 8–10 mm, oblong-ovate, obtuse, pubescent, deciduous; pedicels 8–12 mm, markedly widened upwards, hirsute below, glabrous upwards; sepals subequal, 15–18 × 10–14 mm, broadly elliptic-obovate, rounded, glabrous, margins scarious; corolla 9–10 cm long, white with pink centre, funnel-shaped with cylindrical basal tube c. 12 mm, glabrous, midpetaline bands ending in a small tooth, limb c. 5–6 cm diam.,very shallowly lobed. Capsules ovoid, 20 × 15 mm, glabrous; seeds 10 × 6 mm, flattened ellipsoid, dark brown, long-pilose, the marginal hairs up to 20 mm.

#### Illustration.

Figure 71.

**Figure 71.**
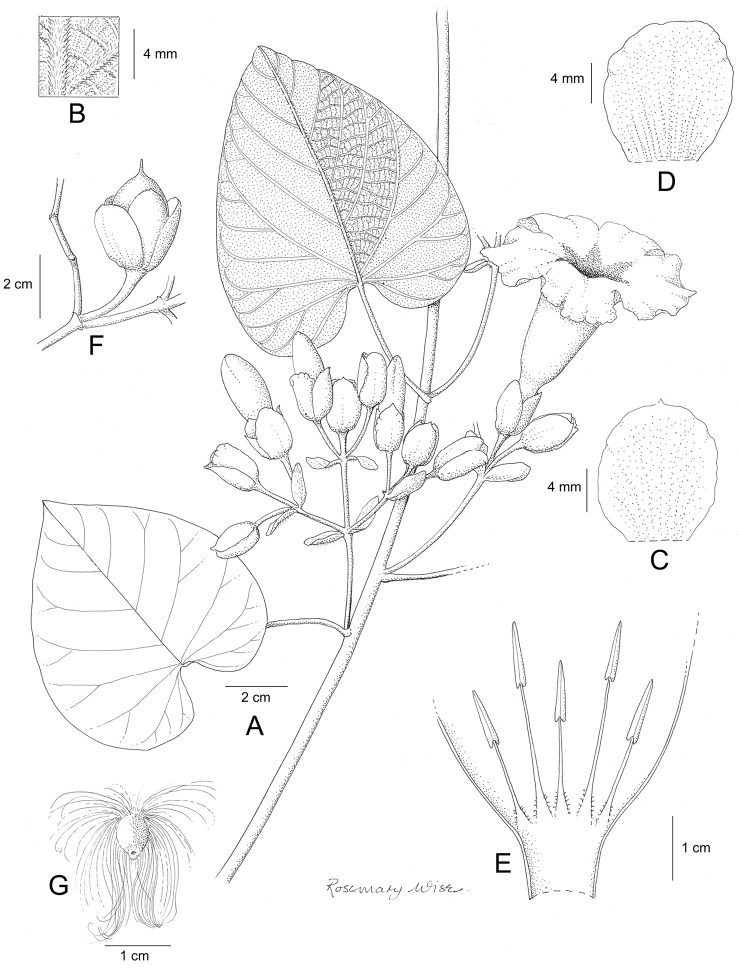
*Ipomoea paradae*. **A** habit **B** abaxial leaf surface **C** outer sepal **D** inner sepal **E** corolla opened out to show stamens **F** fruiting calyx and capsule **G** seed. Drawn by Rosemary Wise **A–E** from *Parada et al.* 3151; **F, G** from *Parada et al.* 162.

#### Distribution.

Endemic to forest and forest relics in areas of the Andean foothills in Santa Cruz Department in Bolivia.

**BOLIVIA. Santa Cruz**: Ibañez, Los Espejillos, *G.A. Parada et al.* 162 (MO, USZ). Ichilo, PN Amboró, ridge between Quebrada Yapojé and Quebrada Caballo, 0.5–1 km above confluence with Río Saquayo, *M. Nee* 40966 (NY, USZ).

#### Notes.

*Parada et al. 162* and *Nee* 40966 are fruiting specimens with glabrous sepals and appear to belong here but in the absence of flowers some doubt about the identity of these collections remains.

*Ipomoea paradae* is somewhat similar to *I. brasiliana* in the indumentum and venation of the leaves and also in the indumentum and size of the sepals but the sepals are always completely glabrous as are the stem and peduncles. The corolla is very distinctive with its white limb and dark red throat, recalling the corolla of *I. juliagutierreziae* and that of *I. longibracteolata*.

### 107. Ipomoea gigantea

Taxon classificationPlantaeSolanalesConvolvulaceae

(Silva Manso) Choisy in A.P. de Candolle, Prodr. 9: 362. 1845. (Choisy 1845: 362)

Convolvulus giganteus Silva Manso, Enum. das Subst. Braz. 18. 1836. (Manso 1836: 18). Type. BRAZIL. Silva Mansos.n. (whereabouts unknown).
Calystegia palmatopinnata Meisn. in Martius et al., Fl. Brasil. 7: 317. 1869 (Meisner 1869: 317). Type. BRAZIL. J.B. Pohl 1759 (isotype K, isotypes M, ?W).
Ipomoea palmatopinnata (Meisn.) Benth. & Hook. f., Gen. Pl. 2 (2): 874. 1876. (Bentham and Hooker 1876: 874).

#### Type.

Based on *Convolvulus giganteus* Silva Manso

#### Description.

Very robust prostrate perennial, stems tomentellous. Leaves petiolate, deeply divided into linear-oblong segments, usually 7–9 in number, the two basal pairs free to an attenuate base, the terminal 3 forming a 3-lobed leaflet, leaflets 5–9 × 1–1.5 cm, apex obtuse and mucronate, softly adpressed-pilose to tomentellous on both surfaces but abaxially paler, the veins highlighted with denser indumentum; petioles 2.5–4.5 cm, tomentose. Inflorescence of solitary (rarely paired), axillary flowers; peduncles 1.5–4.5 cm, pubescent; bracteoles 3–5 × 2.6 –3.5 cm, oblong-obovate, obtuse, mucronate, prominently veined, adpressed pilose, enclosing pedicel and calyx, the margin white-ciliolate; pedicels 5–7 mm, pubescent; sepals slightly unequal, obovate, outer 20–23 × 10–11 mm, obovate, abruptly narrowed to a broad mucronate apex, pilose but with broad glabrous, scarious margins, inner sepals 15 × 6–7 cm, broadly oblong, rounded and mucronate, pubescent centrally but with broad scarious glabrous margins; corolla 9–10 cm long, pink or red, narrowly funnel-shaped, pilose; limb 5–6 cm diam., lobed. Capsules and seeds not seen.

#### Illustration.

Figures 4G, 72.

**Figure 72.**
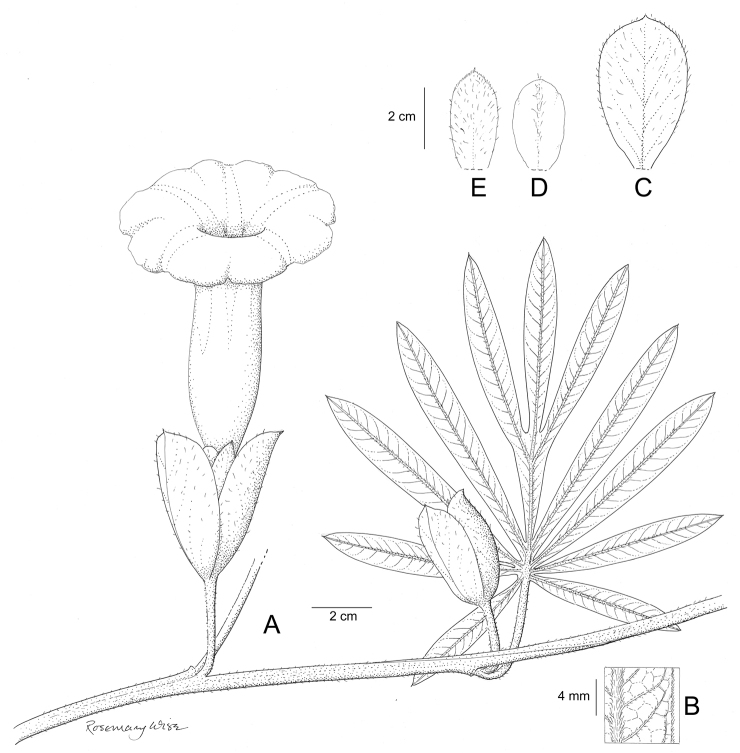
*Ipomoea gigantea*. **A** habit **B** abaxial leaf surface **C** outer sepal **D** middle sepal **E** inner sepal. Drawn by Rosemary Wise **A–E** from *Macedo* 4207 with **A** also from *Brade* 15400.

#### Distribution.

Endemic to the Cerrado region of central Brazil.

**BRAZIL. Goiás**: *H.A. Weddell* (P); Estrada de Goiania a Bela Vista, *A.M. Carvalho & C.F. Delphim* 2252 (CEPEC, K, UB); Serra Dourada, *M.R. Silva & C. Rodrigues* 552 (MO); Serra de Caldas Novas, *E.P. Heringer* 13121 (NY); Anapolis, *E.P. Heringer* 10888 (NY); Goiania, *A.C. Brade* 15400 (HB, RB). **Mato Grosso**: Camino do Barra de Garças al aeropuerto, *A. Krapovickas & C. Cristóbal* 42956 (CTES, SP). **Mato Grosso do Sul**: *F. de Barros* 968 (SP). **Minas Gerais**: *A. Krapovickas et al.* 33047 (MO, CTES); Ituiutaba, *A. Macedo* 4207 (K, NY, S); Amaro Leite, *A. Macedo* 263 (MO, S).

#### Note.

A remarkable plant because of its large sepals and corolla and the deeply divided leaves with up to nine linear-oblong segments.

### 108. Ipomoea brasiliana

Taxon classificationPlantaeSolanalesConvolvulaceae

(Mart. ex Choisy) Meisn. in Martius et al., Fl. Brasil. 7: 261. 1869. (Meisner 1869: 261)

Rivea brasiliana Mart. ex Choisy Prodr. [A.P. de Candolle] 9: 326. 1845. (Choisy 1845: 326). Type. BRAZIL. Provinciae Piau [Piauí]. et prope Joazeiro Prov. Bahia, *Martius* 2478 (holotype M0184899).

#### Type.

Based on *Rivea brasiliana* Mart. ex Choisy

#### Description.

Vigorous twiner or a liana to 5 m high; stems white-tomentose when young but sometimes glabrescent on older parts. Leaves shortly petiolate, 3–10 × 3–9 cm, ovate, obtuse and mucronate, base shallowly to deeply cordate, adaxially dark green, tomentellous, abaxially white-tomentose, the veins often highlighted; petioles 1–4.5 cm, white-tomentose. Inflorescence of shortly pedunculate few-flowered, compact axillary cymes; peduncles 1–5 cm, white-tomentose; bracteoles 1.8–2.2 cm, oblong-boat-shaped, papery, tomentose, caducous; pedicels often less hairy than peduncles, glabrous to (var. subincana) tomentose, 5–8 mm; sepals subequal, 10–13 × 8–9 mm, but strongly accrescent in fruit to 18 × 12 mm, elliptic, obtuse, margins scarious, glabrous to (var. subincana) tomentose, inner more rounded with broader margins; corolla 5–8 cm long, pink, funnel-shaped, nearly glabrous except for a few hairs near the apex of the midpetaline bands to (var. subincana) tomentose, at least in bud; limb 3–4 cm diam. Capsules 12–16 × 12–13 mm, subglobose, glabrous; seeds 10 × 7 mm, minutely tomentellous under a microscope.

#### Illustration.

Figure 52C.

#### Variation.

We formally recognise two varieties that were previously treated as distinct species. Both occupy much the same geographical range and habitat in NE Brazil.

### 108a. Ipomoea brasiliana var. brasiliana

Taxon classificationPlantaeSolanalesConvolvulaceae

#### Diagnosis.

Distinguished by the glabrous or at most thinly pubescent pedicels, sepals and exterior of the corolla

### 108b. brasiliana var. subincana

Taxon classificationPlantaeSolanalesConvolvulaceae

(Choisy) J.R.I. Wood & Scotland, comb. & stat. nov.

urn:lsid:ipni.org:names:77208066-1

Rivea subincana Choisy in A.P. de Candolle, Prodr. 9: 325. 1845. (Choisy 1845: 325). Type. BRAZIL. *Prinz Neuwied*s.n. (lectotype BR000005844524, designated by Delgado Junior et al. 2017).
Ipomoea subincana (Choisy) Meisn. in Martius et al., Fl. Brasil. 7: 259. 1869. (Meisner 1869: 259).

#### Diagnosis.

Distinguished by the tomentose pedicels, sepals and exterior of the corolla.

#### Distribution of species.

A common and characteristic species of the caatinga in NE Brazil.

**BRAZIL. Alagoas**: Piranhas, *R. Simão-Bianchini* 1739 (ASE). **Bahia**: *L.P. de Queiroz et al.* 15963 (HUEFS, OXF)–var. subincana; Remanso, *T. Ribeiro et al.* 59 (ALCB, K); ibid., *E. Ule* 7195 (K); Serra de Açuruá, *R.M. Harley et al.* 18949 (K); ibid., *R.M. Harley et al.* 18928; Tucano, de Carvalho et al. 3936 (CEPEC, K); Senhor de Bonfim–Juazeiro, *R. Harley et al*. 16317 (K, MO); Mun. Uibaí, Serra Azul, *R. Atkinson et al.* 2484 (ALCB, K). Mun. Rio de Contas, Caminho para Lagoa Nova, *R. Harley et al.* 5130 (ALCB, K)–var. subincana; Mun. Abaíra, Engenho dos Vieitas, *R. Harley et al.* 51550, (HUEFS, CEPEC, K)–var. subincana; Olha D’Agua, *E. Pereira & C. Pabst* 9787 (F, HB)–var. subincana; Serra Geral de Caitité, *R.M. Harley* 21156 (K)–var. subincana. **Ceará**: Mun. Aiuaba, *J.R. Lemos* 83 (K); Paçujá, *E.B. Sousa* 2419 (UFRN); Perdicão, *A. Löfgren* 141 (S)–var. subincana; Mun. Quixeré, *M.A. Figueiredo et al*. 632 (IPA, K)–var. subincana. **Dist. Fed**.: Brasilia, *E.P. Heringer* 14763 (NY). **Maranhão**: 35 km N of Carolina, *E.L. Taylor* 1285 (ARIZ, NY). **Pernambuco**: Ibimirim, *M.J.N. Rodal & Tamashiro* 628 (UFRP, K); ibid., *Tschá & Sales* 156 (K); Chapada do Araripe, *R.M. Harley et al.* 54149 (K); Mun. Buíque, *M.J.N. Rodal & A.P.S. Gomes 533* (K); ibid., *K. Andrade et al.* 348 (K, PEUFR)–var. subincana. **Paraíba**: Mun. Campina Grande, *M.F. Agra* 1158 (K). **Piauí**: *Pearson* 64 (K); Mun. Picos, *G. Eiten & L.T. Eiten* 10842 (K, NY); Jurena, *G. Sousa* 660 (HUEFS)–var. subincana; *B.M.T. Walter* 6649 (CEN, RB)–var. subincana; *G. Martinelli* 18061 (RB)–var. subincana. **Rio Grande do Norte**: Natal, *L.A. Cestaro* 97-0020 (UFRN). **Sergipe**: Poço Verde, *G.G. Conceição* 45 (AS).

#### Note.

*Ipomoea brasiliana* is usually treated as distinct from *I. subincana* on the basis of its glabrous sepals. Both taxa occupy the same habitat and geographical range and forms intermediate in indumentum, such as *Oliveira* 723 (HUEFS, K) from Bahia, are sometimes found. Since indumentum alone is unsatisfactory as a character to distinguish species and there is no marked geographical patterning in the variation or molecular evidence to separate these species (Muñoz-Rodríguez et al. 2019), we treat these as a single species under the oldest name *Ipomoea brasiliana*.

### 109. Ipomoea yaracuyensis

Taxon classificationPlantaeSolanalesConvolvulaceae

J.R. Grande & W. Meier, Brittonia, 63(3): 365. 2011. (Grande et al. 2011: 365)

#### Type.

VENEZUELA. Yaracuy: Sierra de Aroa, 1480 m, *L. Aristeguieta & E. Foldats* 1500 (holotype VEN34023).

#### Description.

Twining liana of unknown height; stem thin, cream-coloured, glabrescent. Leaves petiolarte, 7–13 × 5.3–10 cm, ovate-deltoid, apex acuminate, base cordate with rounded auricles, margin often with a distinct angle, adaxially nearly glabrous, abaxially puberulent on the veins, venation prominent; petioles 4.5–7 cm, thinly puberulent. Inflorescence of 1–4-flowered pedunculate, axillary cymes; peduncles 1–4(–8) cm, puberulent; bracteoles 4–8 × 1 mm, linear, deciduous; secondary peduncles c. 10–15 mm; pedicels 10–25 mm; sepals subequal, obovate, mucronate, adpressed puberulous near base, outer 23–31 × 12 mm, inner sepals slightly smaller; corolla c. 7 cm long, creamy-yellow with purplish tube, funnel-shaped, thinly pubescent; limb c. 5–6 cm diam. Capsules 2–2.5 cm, subglobose, rostrate, the persistent style base c. 2 mm long; seeds 10 × 5 mm, tomentose and with long silky marginal hairs up to 14 mm long.

#### Illustration.

Grande et al. (2011: 366).

#### Distribution.

Endemic to the coastal Andes of Venezuela, growing in evergreen forest around 800–1200 m.

**VENEZUELA. Yaracuy**: Sierra de Aroa, Cerro Tigre, *R. Liesner & A.C. González* 9703(MO, VEN); El Amparo, *E. Diederichs* 70 (MO, VEN); Bruzual, arriba de Campo Elías, *E. Rutkis* 460 (VEN).

#### Note.

This species is placed here because of its large pubescent sepals and puberulent corolla but its position is uncertain.

### 110. Ipomoea chrysocalyx

Taxon classificationPlantaeSolanalesConvolvulaceae

D.F. Austin, Flora of Ecuador 15: 45. 1982. (Austin 1982a: 45)

#### Type.

ECUADOR. El Oro, below Zaruma, *Asplund* 15851 (holotype S07–4785, isotype GB).

#### Description.

Twining perennial; stems relatively stout, thinly pilose with pale hairs, latex white. Leaves petiolate, 10–17 × 7–13 cm, ovate, acute to shortly acuminate, cordate, both surfaces appressed pubescent to ±glabrous, the venation spreading at a wide angle, prominent; petioles 4–11 cm. Inflorescence of shortly pedunculate compact axillary cymes with up to 9 flowers; peduncles 2–3.7 cm; bracteoles 7–15 mm, oblong-oblanceolate, relatively persistent; secondary peduncles 8–10 mm; pedicels 3–5 mm, puberulent to pilose; sepals 11–14 × 4–5 mm, subequal, oblong-ovate, obtuse to subacute, densely pubescent; corolla 4–5.5 cm long, funnel-shaped from a very short greenish basal tube, glabrous, white, limb angled but not lobed, 3.5 cm diam. Capsules and seeds unknown.

#### Illustration.

Figure 73.

**Figure 73.**
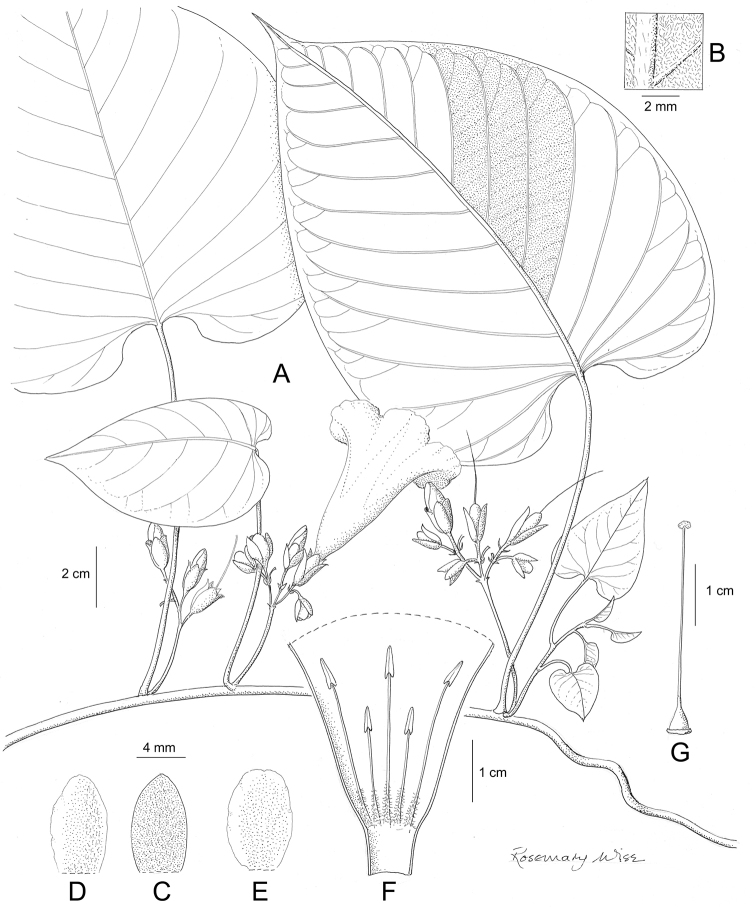
*Ipomoea chrysocalyx*. **A** habit **B** abaxial leaf surface **C** outer sepal **D** middle sepal **E** inner sepal **F** corolla open out to show stamens **G** ovary and style. Drawn by Rosemary Wise **A** from *T. Croat* 58306; **B–G** from *G. Harling & L. Andersson* 14306.

#### Distribution.

A rare species of Ecuador and northern Peru growing in thickets and on rocky slopes between 600 and 1800 m.

**PERU. Amazonas**: Prov. Bongará, 21 km N of Pedro Ruíz, *T. Croat* 58306 (FTG, MO, OXF).

**ECUADOR. El Oro**: Porto Velo-Lourde trail to Salatí, *G. Harling & L. Andersson* 14306 (GB, MO). **Loja**: Chaguarpampa, *F. de la Puente* 1260 (CIP); N. of Macará, *G. Harling & L. Andersson* 18286 (GB); Alamor-Zaderos, *G. Harling & L. Andersson* 17814 (GB).

#### Note.

The placement of this species is provisional. The pubescent corolla and calyx strongly support its placement in Clade A but a final decision cannot be made until this species has been successfully sequenced.

### 111. Ipomoea pochutlensis

Taxon classificationPlantaeSolanalesConvolvulaceae

J.R.I. Wood & Scotland sp. nov.

urn:lsid:ipni.org:names:77208067-1

#### Type.

MEXICO. Oaxaca, Pochutla, Mu. San Miguel del Puerto, copalitilla, cascadas del río, 30 July 1999, *J. Rivera H., S. Salas M. & E. Martínez S.* 1741 (holotype MEXU1234493).

**Diagnosis.** Bears a superficial resemblance to *Ipomoea riparum* in its very shortly pedunculate bracteolate cymes but distinguished by the very unequal, whitish-green, glabrous sepals and the glabrous white corolla.

#### Description.

Robust twining perennial of unknown height; stems glabrous, somewhat sharply angled. Leaves petiolate, 8–12 × 7–10, shallowly 3-lobed, base cordate, apex acute, both surfaces glabrous, minutely white-punctate, abaxially paler, minutely white-punctate and with prominent white veins; petioles 5–8 cm, pseudo stipules arising at their base. Inflorescence of compact, shortly pedunculate, axillary cymes with up to 10 flowers; peduncles 5–6 mm; lower bracteoles c. 10 × 3 mm, broadly lanceolate with petiolar base, acuminate, persistent; secondary peduncles 1–2 mm, upper bracteoles c. 5–6 × 1 mm, linear, acute, persistent; sepals very unequal, very pale whitish-green with darker veins, outer 5–6 × 2–2.5 mm, ovate, apiculate, inner 10–11 × 5 mm, broadly oblong-oblanceolate, rounded or retuse; corolla 4–4.5 cm long, funnel-shaped, white, glabrous, limb c. 2–3 cm wide. Capsules and seeds not seen.

#### Illustration.

Figure 74.

**Figure 74.**
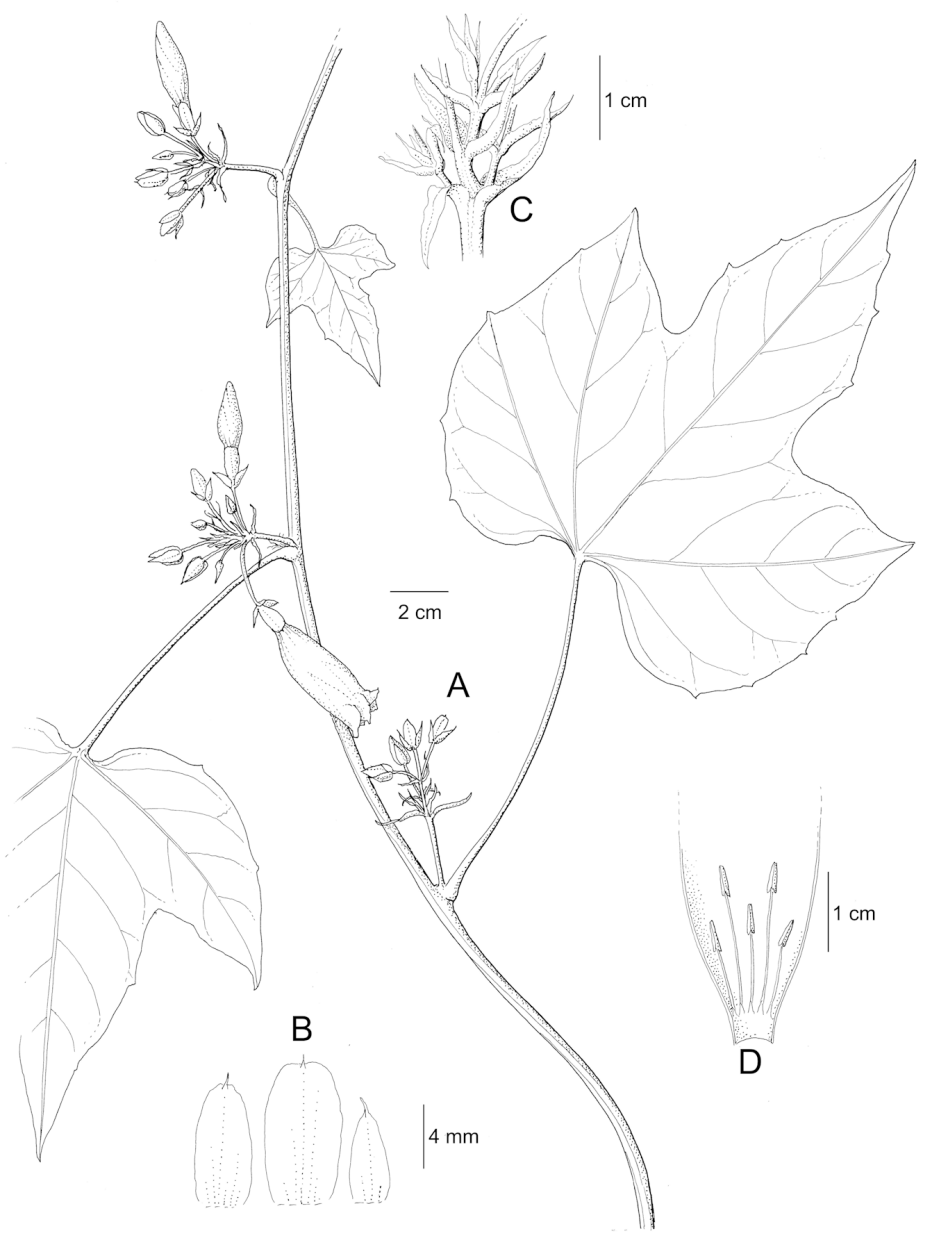
*Ipomoea pochutlensis*. **A** habit **B** sepals (right outermost, left–second, middle inner) **C** inflorescence showing bracteoles and branching **D** corolla opened out. Drawn by Rosemary Wise from *Rivera H., Salas M. & Martinez S.*1741

#### Distribution.

Endemic to Oaxaca in Mexico, where it was found by a stream in semi-evergreen forest at 320 m. Only known from the type collection.

**MEXICO. Oaxaca**: Pochutla, Mun. San Miguel del Puerto, *J. Rivera et al.*1741 (MEXU).

#### Note.

The exact relationships of this species are unclear. Molecular sequencing using *ITS* suggests it is related to *Ipomoea brasiliana* but there is little obvious morphological similarity. In its very shortly pedunculate bracteolate cymes it bears some resemblance to *I. riparum* but the very unequal, whitish-green glabrous sepals and the white glabrous corolla are very distinct.

### 112. Ipomoea riparum

Taxon classificationPlantaeSolanalesConvolvulaceae

Standl. & L. O. Williams, Ceiba 1: 63. 1950. (Standley and Williams 1950: 63)

Ipomoea diriadactylina Hammel, Phytoneuron 2012-27: 1. 2012. (Hammel 2012: 1–6). Type. COSTA RICA. Santa Cruz, rumbo a Vista al Mar por P.N. Diriá, B. Hammel & I. Pérez 25480 (holotype MO6409984, isotypes apparently not distributed).

#### Type.

HONDURAS. Dept. Morazán, Río de la Orilla, *A. Molina* 2528 (holotype EAP, n.v., isotypes GH, F, US).

#### Description.

Perennial liana of unknown height, stems glabrous or with a few dispersed trichomes. Leaves petiolate, 8–20 × 5–15 cm, ovate, acuminate, cordate with rounded auricles, glabrous, abaxially paler, sometimes black-dotted; petioles 4.5–10 cm. Inflorescence of shortly pedunculate, dense, bracteolate axillary cymes; peduncles 0.6–1.5 cm; bracteoles 10–20 × 5–10 mm, elliptic, mucronate, obscurely pustulate, persistent; pedicels 1–5 mm; sepals subequal, somewhat similar in texture to bracteoles, 11–16 × 5–10 mm, oblong-elliptic, obtuse and mucronate, abaxially pustulate, margins paler; corolla 5–7 cm long, funnel-shaped, glabrous, tube greenish, limb 5–6 cm diam., white, undulate; stamens included. Capsules 11–12 × 10 mm, subglobose, shortly rostrate with the basal part of the style persistent, glabrous; seeds 6–7 × 4 mm, with long marginal hairs.

#### Illustration.

Hammel (2012: 2).

#### Distribution.

A rare species of low altitude forest in Central America.

**COSTA RICA**. Type of *I. diriadactylina*.

**HONDURAS.** Morozán, Tegucigalpa-Puente Colorado, *A. & R. Molina* 25845 (BM, F, S); ibid., zona de El Zamorano, *P.C. Standley* 26382 (BM).

#### Note.

Distinctive because of the dense, shortly pedunculate bracteolate cymes and white flowers.

### 113. Ipomoea nivea

Taxon classificationPlantaeSolanalesConvolvulaceae

J.R.I. Wood & Scotland sp. nov.

urn:lsid:ipni.org:names:77208068-1

#### Type.

PERU. Amazonas: Luya, Camporredondo, Ishangas, 6°07'03"S, 78°20'02"W, 1450 m, 30 March 1997, *J. Campos, L. Campos & J. Sembrera* 3748 (holotype MO, isotypes K, OXF).

**Diagnosis.** Resembles *Ipomoea praecana* in the large ovate, cordate leaves, which are abaxially white-floccose to sericeous and in the short peduncles < 12 mm long, but differs in the longer (12 cm, not 6–10 cm), clearly funnel-shaped (not subhypocrateriform), pink (not white) corolla.

#### Description.

Subshrub to 4 m, reported to be succulent; stem densely white-tomentellous. Leaves petiolate, 6–18 × 5–15.5 cm, ovate, base cordate, apex rounded, mucronate, margin undulate, adaxially green, shortly tomentellous, abaxially white-floccose to sericeous, veins more densely hairy; petiole 6–11 cm, sericeous. Inflorescence of shortly pedunculate axillary cymes; peduncles 10–12 mm, sericeous; bracteoles 18 × 8 mm, spathulate, obtuse, sericeous; pedicels 7–8 mm, sericeous; sepals subequal, sericeous, 22 × 15 mm, elliptic-obovate, obtuse, the inner more rounded; corolla pink, funnel-shaped, c. 12 cm long, the exterior densely pubescent, especially on the midpetaline bands. Capsules and seeds not seen.

#### Illustration.

Figure 75.

**Figure 75.**
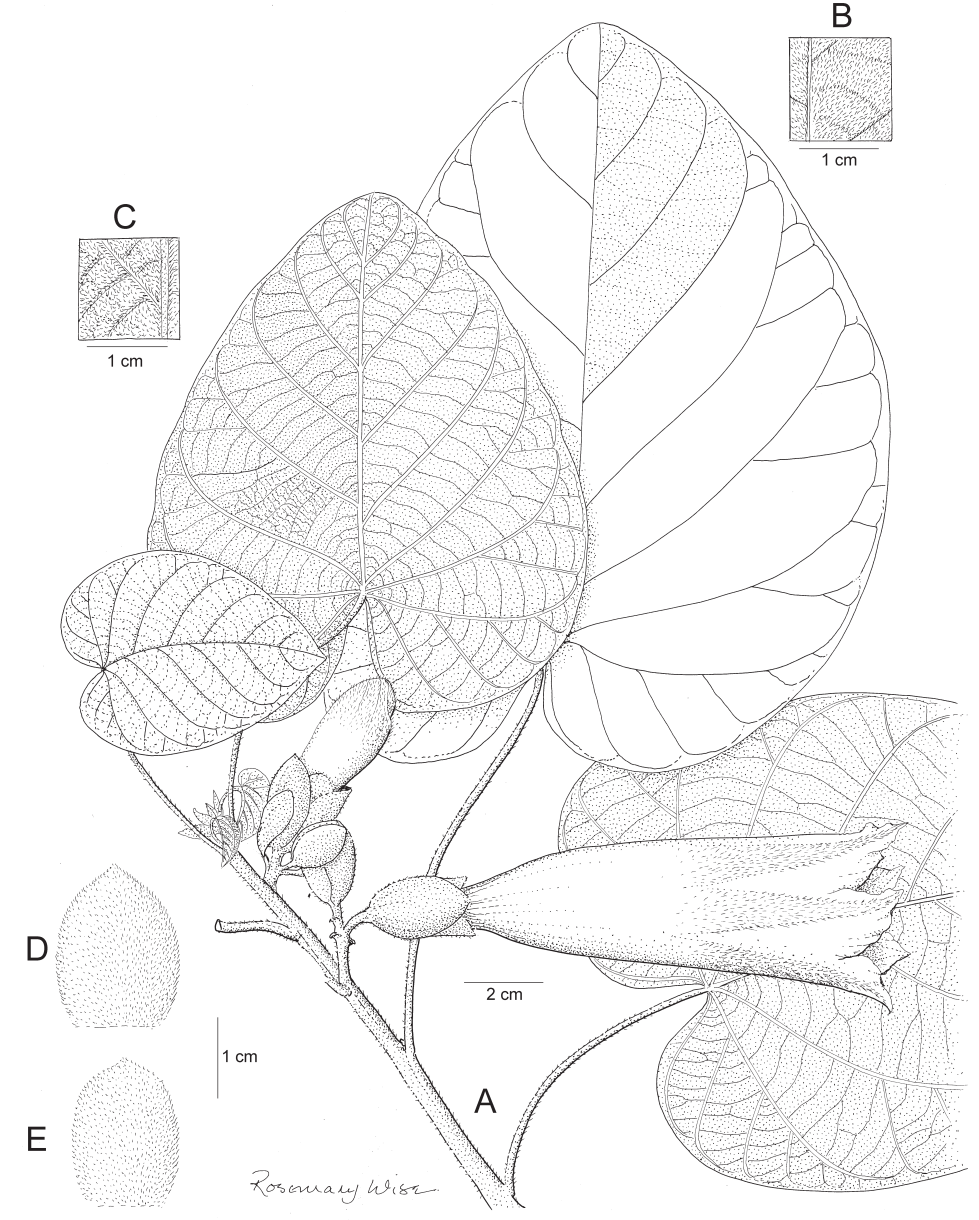
*Ipomoea nivea***A** habit **B** adaxial leaf surface **C** abaxial leaf surface outer sepal **D** outer sepal **E** inner sepal. Drawn by Rosemary Wise from *Campos et al.* 3748.

#### Distribution.

A very rare species endemic to northern Andean Peru only known from the type collection.

**PERU. Amazonas**: The type collection.

#### Note.

Appears to be rather similar vegetatively to *Ipomoea praecana*, especially in the leaf shape, indumentum and short peduncles, but differs in the longer, clearly funnel-shaped, pink corolla.

### 114. Ipomoea mathewsiana

Taxon classificationPlantaeSolanalesConvolvulaceae

Kuntze, Rev. Gen. Pl. 2: 443. 1891. (Kuntze 1991: 443)

#### Type.

PERU. [Junin], Quebrada of Parahuanca, *A. Mathews* 885 (lectotype K000612872, designated here; isolectotypes K, OXF).

#### Description.

Erect shrub to at least 1.25 m; stems stout, woody, all young parts densely white-tomentose. Leaves petiolate, small, 3–6 × 2–5 cm, ovate, cordate with rounded auricles, adaxially glabrous, abaxially white-tomentose, margins highlighted white-tomentose; petioles 0.7–1.8 cm, white-tomentose. Inflorescence subcorymbose, formed of compact cymes borne towards the apex of leafy axillary side shoots; peduncles 3–4.5 cm; bracteoles 11–16 × 2.5–3 mm, linear-oblong, acute, sericeous, papery, deciduous; secondary peduncles 7–10 mm; pedicels 0–10 mm; sepals subequal in size, narrowly elliptic-obovate, outer 14–16 × 4–6 mm, obtuse, tomentose externally, glabrous marginally, middle sepal with a line of hairs along the midrib, inner sepals rounded, truncate or retuse, glabrous; corolla 4.5–5 cm long, pink, funnel-shaped, sericeous in bud and on midpetaline bands. Capsules and seeds unknown.

#### Distribution.

A very rare species endemic to central Andean Peru apparently known from only the type.

**PERU. Junín**: type collection.

**Notes**. Similar in its shrubby habit to *Ipomoea pulcherrima* differing principally in the sericeous corolla and tomentose outer sepals which are scarcely shorter than the inner sepals It is also close to *I. sericosepala* differing in habit and also in the white-felted indumentum and the more corymbose inflorescence with longer bracteoles and sepals.

Its placement here is unconfirmed.

### 115. Ipomoea pulcherrima

Taxon classificationPlantaeSolanalesConvolvulaceae

Ooststr., Recueil. Trav. Bot. Néerl. 30: 206. 1933. (Ooststroom 1933: 206)

#### Type.

PERU. Apurimac, *A. Weberbauer* 5875, holotype B†, isotypes: F, GH, US).

#### Description.

Erect shrub with abundant white latex, stems whitish on young parts, densely pubescent with crisped hairs. Leaves petiolate, 2–9 × 2–8.5 cm, ovate to suborbicular, obtuse, base subcordate to truncate, margins highlighted white, adaxially appressed puberulous, abaxially white-tomentellous; petioles 1.5–3 cm, densely puberulent. Inflorescence subcorymbose, compact, composed of compact reduced cymes borne at the apex of branchlets up to 15 cm long; peduncles 2–4 mm; bracteoles 1.5–2.5 mm, ovate, caducous; pedicels 8–14 mm, sericeous; sepals unequal, outer 5–7 × 4–5 mm, broadly oblong, rounded, glabrous, margins scarious, inner 9–10 × 6–7 mm, suborbicular to obovate; corolla 4–5 cm long, funnel-shaped, glabrous, colour not known, limb c. 2.5 cm diam. Capsules and seeds unknown.

#### Distribution.

Endemic to the Apurimac valley in southern Peru at 1100 m.

**PERU.** Only known from the the type collection.

#### Notes.

Distinguished by the densely pubescent stems, very unequal, glabrous sepals and glabrous corolla.

Although included by McPherson (1981) in the *Arborescens* group, its shrubby habit and unequal sepals suggest otherwise. Its placement here is arbitrary as we have not sequenced any material.

### 116. Ipomoea juliagutierreziae

Taxon classificationPlantaeSolanalesConvolvulaceae

J.R.I. Wood & Scotland, Kew Bull. 70 (31): 68. 2015. (Wood et al. 2015: 68)

#### Type.

BOLIVIA. Chuquisaca, Prov. Zudañez, Joya Charal, ANMI El Palmar, una hora de la comunidad en el sector denominado Almendras, “ladera expuesta al cerro Mojocoya con presencia de Harrisia, Capparis y Caesalpinia, suelo rocoso con musgos secos en el suelo. Especie creciendo sobre ramas de Leguminosa”, 18°35'20"S, 64°50'14"W, 1610 m, *J. Gutiérrez, L. Carrillo, N. Paucar & S. Peres-Cortez* 2588 (holotype HSB, isotype fragment OXF).

#### Description.

Liana with white latex to 6 m, stems glabrous with pale brown bark; young plants multi-stemmed, but non-climbing stems eventually dying off. Leaves not present when plant flowering, petiolate, 4–5.5 × 2.5–4.5 cm, ovate, apex usually acute to shortly acuminate but occasionally rounded, minutely mucronate, base shallowly cordate to subtruncate, glabrous, abaxially paler, with prominent reddish-brown lateral veins; petioles 1–3 cm, very slender, glabrous. Inflorescence on raceme-like side branches towards the branch tips; peduncles short, 3 mm, woody, glabrous; bracteoles resembling very small leaves; secondary pedicels 2 mm; pedicels c. 7 mm, widened upwards, glabrous; sepals subequal, 11–13 × 8–9 mm, broadly elliptic, rounded, glabrous, the margins scarious; corolla 5–6 cm long, glabrous, shortly funnel-shaped, white with dark red throat, limb 5.5–6.5 cm diam., unlobed; longer stamens held at corolla mouth, shorter included, anthers c. 5 mm; stigma biglobose. Capsules (immature) ovoid, c.15 mm long, glabrous; seeds (immature) pilose on the margins.

#### Illustration.

Figure 52E.

#### Distribution.

Endemic to Bolivia where it is known from xerophytic bushland and dry forest in the Río Grande Valley between 1250 and 1600 m.

**BOLIVIA. Chuquisaca**: Zudañez, Joya Charal, ANMI El Palmar, *J. Gutiérrez al.* 2239 (HSB). **Cochabamba**: Campero, Pasorapa, bajada de Buenavista al Río Grande, *C. Antezana* 626 (BOLV, CTES). **Santa Cruz**: Vallegrande, on the ascent from Pampa Negra, *J.R.I. Wood et al.* 28261 (LPP, OXF, USZ).

#### Note.

Resembling species in the Arborescens Clade, molecular studies using *ITS* suggest it is sister to the Arborescens Clade. From *Ipomoea pauciflora*, *I. juliagutierreziae* is distinguished by its liana (not tree-like) habit, obtuse to rounded (not acute) outer sepals and bilobed stigma, each lobe subglobose, 1.25 × 1.25 mm (not ellipsoid to cylindrical, 2 × 1 mm). Additionally the leaves and corolla are notably smaller than in typical *I. pauciflora*.

• The Arborescens Clade (117–126)

Small trees, large shrubs or lianas, copious white latex usually present. Leaves entire, large, the base cordate or truncate, often absent at anthesis. Flowers appearing when plant mostly leafless, few, often clustered on a reduced branchlet forming a subracemose structure; peduncles short, commonly much shorter than the pedicels; bracteoles small, caducous; sepals subequal, large, usually 10–30 mm long, coriaceous, ovate, obtuse, mucronate. Corolla rather large, campanulate to funnel-shaped, white, sometimes with dark purple throat, glabrous or, commonly pubescent on the midpetaline bands; anthers included. Seeds with long white hairs on the angles. Some or all species may be bat pollinated (McDonald 1991).

The species in this clade are not very well-defined but appear to be more easily recognised in the field than in the herbarium. They can be separated by the following key which includes *Ipomoea juliagutierreziae*.

**Table:** 

1	Sepals 14–28 mm long; all vegetative parts densely villous at least when young; cymes usually 1-flowered	**125. *I. murucoides***
–	Sepals 5.5–21 mm long, vegetative parts glabrous to pubescent; cymes mostly 2–5-flowered	**2**
2	Corolla conspicuously tomentose at least in bud, 4–6 cm long; leaves usually tomentose	**124. *I. arborescens***
–	Corolla glabrous or inconspicuously pubescent on the midpetaline bands only, at least 5 cm long; leaves glabrous or pubescent on veins beneath	**3**
3	Leaves linear, mostly < 1 cm wide	**122. *I. chilopsidis***
–	Leaves lanceolate to ovate, > 1 cm wide	**4**
4	Sepals 5.5–13 mm long	**5**
–	Sepals 11–21 mm long	**8**
5	Sepals abaxially glabrous; leaves glabrous; stem glabrous	**6**
–	Sepals abaxially pubescent; leaves pubescent at least abaxially at base of midvein; stem glabrous or, when young, pubescent	**7**
6	Liana; stigmas globose; flowers borne on completely leafless, slender apical branchlets, < 3 mm wide	**116. *I. juliagutierreziae***
–	Tree; stigmas cylindrical; flowers axillary and terminal, borne on stout, leafy stem	**117. *I. pauciflora***
7	Liana; adaxial surface of sepals with bulbous-based hairs; stamens 10–13 mm long	**118. *I. populina***
–	Tree; adaxial surface of sepals with tiny hairs, not bulbous at base; stamens 12–28 mm long (low altitude species)	**119. *I. wolcottiana***
8	Leaves pubescent on both surfaces	**126. *I. teotitlanica***
–	Leaves glabrous or thinly pubescent on veins beneath	**9**
9	Multi-stemmed shrub; leaves rather small, < 6 cm long	**123. *I. seaania***
–	Tree or shrub with a single main trunk; leaves usually > 5.5 cm long	**10**
10	Shrub; sepals pubescent or glabrous externally; stamens 13–28 mm long	**121. *I. rzedowskii***
–	Tree; sepals glabrous externally; stamens 30–40 mm long	**120. *I. intrapilosa***

### 117. Ipomoea pauciflora

Taxon classificationPlantaeSolanalesConvolvulaceae

M. Martens & Galeotti, Bull. Acad. Roy. Sci. Bruxelles 12: 266. 1845. (Martens and Galeotti 1845: 266)

Ipomoea vargasiana O’Donell, Bol. Soc. Peru. Bot. 1: 5. 1948. (O’Donell 1948b: 5). Type. PERU. Cuzco, Anta, *C. Vargas* 1021 (holotype LIL001357, isotypes CUZ, MO).
Ipomoea pauciflora subsp. vargasiana (O’Donell) McPherson, Ann. Missouri Bot. Gard. 68(4): 537. 1981. (McPherson 1981: 537).

#### Type.

MEXICO. Oaxaca, *H. Galeotti* 1403 (holotype BR00006972660, isotype fragments BM, P).

#### Description.

Tree or more commonly shrub to 7 m, variable in habit, with arching branches, often near leafless when flowering, stems glabrous, bark light brown, latex present, white. Leaves petiolate, 4–10 × 2.3–6 cm, ovate, finely acuminate and mucronate, truncate to very shallowly cordate, glabrous; petioles 1.5–5 cm. Inflorescence of shortly pedunculate, 1–3-flowered cymes often borne on small axillary side branches; peduncles 0.2–3 cm; bracteoles 3 mm, oblong, caducous; pedicels 20–32 mm, thickened upwards; sepals subequal, abaxially glabrous, adaxially pubescent, the margins scarious, outer 9–11 × 6–8 mm, oblong-ovate, acute, often mucronate, inner sepals similar but scarious margins broader; corolla 5–7.5 cm long, white, broadly funnel-shaped, glabrous, tube commonly reddish inside, limb 7 cm diam., undulate; stamens 9–12 mm long; stigmas cylindrical, c. 2.5 mm long. Capsules 18–22 × 10–12 mm, ellipsoid glabrous; seeds 10–11 × 5 mm, glabrous apart from the pilose margins, the hairs white c. 9–12 mm long.

#### Illustration.

Figures 3A, 52D.

#### Distribution.

Seasonally dry deciduous woodland mostly between 1000 and 2600 m from southern Peru north to southern Mexico.

**PERU. Ayacucho**: *Weberbauer* 5665 (US), 5667 (US), 5899 (US); La Mar, *J. Roque & C. Arana* 3120 (USM). **Apurimac**: Abancay, *E.K. Balls* 6838 (BM, F, K, US); Grau, *C. Vargas* 5814 (CUZ). **Cusco**: Anta, Limatambo, *H. Galiano* 5723 (MO); ibid., Mollepata, *L. Valenzuela et al.* 9774 (MO, OXF); ibid., *W. Galiano et al.* 5159 (MO). **Huancavelica**: *K.G. Dexter et al*. 6495 (E); Colcabamba, *O. Tovar* 2117 (USM). **Tumbes**: Cerros de Amotape, *A. Gentry et al.* 58318 (MO) fide D. Austin.

**ECUADOR. Loja**: *G. Harling et al.* 15403 (AAH, GB); Catamayo valley, *L. J. Dorr & I. Valdespino* 6643 (QCNE).

**COLOMBIA. Boyacá**: Chicamocha valley, *R. Jaramillo & T. van der Hammen* 4238 (COL, MA).

**NICARAGUA.** Fide Austin et al. (2012).

**HONDURAS.** Fide Austin et al. (2012).

**GUATEMALA.** Fide Austin et al. (2012).

**MEXICO. Chiapas**: Tzimol, *A. Reyes-García & E. Martínez* 203 (BM, MO); *D.E. Breedlove* 22952 (F). **Est. México & Dist. Fed.**: Cult. in Jardín Botánico, *A. García* 4435 (MEXU): Temascaltepec, Luvianos, *G.B. Hinton et al.* 5305 (BM, K), ibid., 8754 (K). **Guerrero**: near Acapulco, *E. Palmer* 619 (BM, K, MICH); Rincón de la Vía, *E. Matuda* 37249 (MEXU); Xalpatlahuac, *C. Toledo & R. Landa* 548 (MEXU). **Jalisco**: *D. Weberbauer* 5322 (MEXU). **Morelos**: Cuenavaca, *E. Bourgeau* 1407 (P, S); Temisco, *M.T. Germán & V. Funk* 595 (MEXU); Yautepec, *R. Quezada* 1915 (MEXU). **Oaxaca**: Cuicatlán, *J.I. Calzado* 24340 (K, MEXU); Santiago Chazumba, *J.I. Calzado* 24479 (K, MEXU); Mount Albán, *C.G. Pringle* 4965 (BM, E, K, MICH, S); Ixtaltepec, *C. Martínez* 1262 (MEXU). **Puebla**: Tehuacán, *J.I. Calzado & A. O. López* 22909 (K, MEXU); Juan N. Méndez, *J.I. Calzada* 24328 (K, MEXU); Ahuehuetitla, *S. Zamudio O. Ocampo* 10981 (IEB, MEXU).

#### Notes.

McPherson (1981) recognised two subspecies but these are poorly defined morphologically and are not recognised here. In any case this species is not always easily separable from *Ipomoea wolcottiana* or *I. populina*, the former differing in the often obscurely pubescent sepals and the latter in the liana habit.

The record of Ipomoea pauciflora M. Martens & Galeotti subsp. vargasiana in Austin and Huáman (1996) from Bolivia is presumably an error as we have been unable to trace any collection or literature reference.

### 118. Ipomoea populina

Taxon classificationPlantaeSolanalesConvolvulaceae

House, Ann. New York Acad. Sci. 18: 226. 1908. (House 1908b: 226)

#### Type.

MEXICO. Guerrero, *E. Palmer* 482 (holotype US00111446; isotypes K, GH, NY, UC, US).

#### Description.

Climbing or trailing liana to at least 4 m, stems glabrous or pubescent. Leaves petiolate, 4.5–13 × 3–9 cm, narrowly ovate, acuminate, base truncate to weakly cordate, usually abaxially pubescent at base of midvein; petioles 2.5–5 cm. Inflorescence of terminal and axillary 1–5-flowered cymes borne on short branchlets; peduncles 0.5–2.5 cm, glabrous or pubescent; bracteoles ovate-deltoid, 2–4 × 1–1.5 mm; pedicels 1.5–3.5 cm, glabrous or pubescent; sepals subequal, 5.5–12 × 6–9 mm, ovate to suborbicular, acute or obtuse, abaxially glabrous or pubescent; adaxially pubescent with bulbous-based hairs; corolla 5.5–8 cm long, funnel-shaped, sparsely pubescent on the midpetaline bands (rarely glabrous), limb 7–10 cm diam.; stamens 10–13 mm, stigmas cylindrical. Capsules ellipsoid, 15–25 mm long; seeds long-pilose on the margins.

#### Distribution.

In scattered localities from southern Mexico south to Nicaragua.

**N**ICARAGUA. Estelí, Mun. Condega, *P. Moreno* 25330 (BM); Madriz, Cerro Quisaca, *W.D. Stevens et al.* 27620 (MO).

**EL SALVADOR.** Santa Ana, P.N. Montecristo, *V.M. Martínez* 500 (BM).

**HONDURAS.** Morazán, *A. Molina* 18464 (BM, NY).

**GUATEMALA.***W. Popenoe* 360a (BM); Zacapa, Río Hondo, *L.O. Williams et al.* 41887 (BM, F, MO, NY); Baja Verapaz, Salamá, *J.M. Christenhusz et al.* 5666 (BM).

**MEXICO. Chiapas**: Cintalapa, *A. Reyes García et al.* 1463 (BM, MO); 30 km from Tuxtla Gutiérrez towards San Cristóbal, *P.J. Stafford et al.* 236 (BM). **Guerrero**: *Langlassé* 612 (F, K, P, US); Montes de Oca, *G.B. Hinton et al.* 11528 (K, MICH, NY, US); Zoyatepec, *E.M. Martínez & B. Morales* 3404 (MEXU). **Oaxaca**: Juchitán, Arroyo Chivela, *E. Pérez García* 1743 (MO); Buenavista, Cerro Guiengola, *L. Torres* 734 (MEXU); Pochutla, *M. Elorsa* 6323 (MEXU). **Puebla**: Caltepec, *P. Tenorio* 7268 (MEXU).

#### Note.

Very similar to *Ipomoea wolcottiana* differing principally in its climbing or prostrate (not tree-like) habit. The pubescent buds are a useful character. Herbarium specimens can be difficult to distinguish from *Ipomoea wolcottiana*.

### 119. Ipomoea wolcottiana

Taxon classificationPlantaeSolanalesConvolvulaceae

Rose, Gard. & Forest 7: 367. 1894. (Rose 1894: 367)

Ipomoea calva House, Bot. Gaz. 43: 410. 1907. (House 1907b: 410). Type. MEXICO. Guerrero, La Junta, *E.W. Nelson* 6992 (holotype US00111373).
Ipomoea calodendron O’Donell, Lilloa 23: 480. 1950. (O’Donell 1950b: 480). Type. PERU. [Piura], valley of Río Quiros, Weberbauer 6396 (holotype US00111371, isotype F, NY).
Ipomoea wolcottiana subsp. calodendron (O’Donell) McPherson, Ann. Missouri Bot. Gard. 68(4): 544. 1981. (McPherson 1981: 544).

#### Type.

MEXICO. Colima, Manzanillo, *E. Palmer 1342* (holotype US00111492, isotypes BM, GH, K, NY).

#### Description.

Tree to 13 m, the trunk up to 30 cm wide and with milky sap, stems shortly puberulent or glabrous. Leaves petiolate, 4–15 × 2.3–9 cm, ovate, acuminate, very shortly mucronate, shallowly cordate to truncate at base, adaxially thinly pubescent to glabrous, abaxially pubescent to obscurely puberulent on veins; petioles 1.5–4.5 cm, slender, glabrous. Inflorescence usually pendent of single flowers or several borne on short branches, sometimes with reduced leaves, peduncles 1–4 mm; bracteoles 2–6 mm, lanceolate, caducous; pedicels 6–24 mm; sepals subequal, 6–12(–15) × 6–7(–8) mm, elliptic, obtuse, abaxially finely puberulent to almost glabrous, adaxially pubescent, margins somewhat scarious; corolla 5–6(–9) cm long, white with dark red throat, glabrous except pubescent tips of the midpetalline bands, limb 5–5.5 cm diam.; stamens 12–30 mm long; stigma globose to elongate. Capsules ellipsoid, 20 × 10 mm, glabrous; seeds 8–10 × 3–4 mm. long-pilose on margins. Reported to be a night flowering species.

#### Illustration.

Figure 9B.

#### Distribution.

Dry, deciduous forest in scattered disjunct locations from Peru through Central America to southern Mexico at relatively low altitudes of 50–900 m,

**PERU. Piura**: Tondopa-Ayabaca, *A. Gentry et al.* 75132 (MO); Paita, *O. Haught* 60a (F, US); Cerro Viento, *O. Haught* 201 (F, US).

**ECUADOR. Loja**: *A. Samaniengo & F. Vivar* 022 (US).

**EL SALVADOR.** Santa Ana, Metapán, *J. Monterrosa* 92 (BM); La Libertad, *K. Sidwell et al.* 512 (BM, MO); *A. Munro et al.* 3676 (BM).

**HONDURAS.***Cox & Guzman* 254 (MO), fide D.F. Austin.

**GUATEMALA.***H. Pittier* 1859 (US), fide D.F. Austin.

**MEXICO. Chiapas**: *A. Reyes García et al.* 1483 (BM, MEXU). **Colima**: Ixtlahuacan, *M. Navarrete de la Paz* 799 (MEXU). **Guerrero**: Papanoa, *E. Langlassé* 736 (GH, K, P, US); Tierra Colorada, *H. Kruse* 2373 (MEXU). **Jalisco**: Chamela, *S. Bullock* 905 (MEXU); La Huerta, *S. Bullock* 1068 (MEXU, MO); ibid., *J. Calónico* 7732 (MEXU). **Michoacán**: Águila, *A. Lozano & M.A. García* 7099 (MEXU); El Camalote, *E. Carranza & I. Silva* 6690 (IEB, MEXU). **Oaxaca**: Tehuantepec, *M. Elorsa* 7781 (MEXU); Santiago Astata, Chacalapa, *C.E. Hughes & M. Elorsa* 1911 (FHO, MEXU). **Puebla**: *C. Rojas-Martínez* 85 (MEXU). **Tabasco**: fide McPherson (1981). **Veracruz**: Cerro Gordo, *J. Dorantes et al.* 01757 (MEXU); Chicuasen, *S. Avendano et al*. 45 (K, MEXU)

#### Note.

McPherson (1981) recognised two subspecies but these are poorly defined morphologically and are not recognised here.

### 120. Ipomoea intrapilosa

Taxon classificationPlantaeSolanalesConvolvulaceae

Rose, Gard. & Forest 7: 367. 1894. (Rose 1894: 367)

Ipomoea murucoides var. glabrata S. Watson, Proc. Amer. Acad. Arts 22: 440. 1887. Type, MEXICO. Jalisco, *E. Palmer* 703 (holotype GH00054521, isotypes BM, K, MEXU, US)

#### Type.

MEXICO. Jalisco, *E. Palmer* 705 (US00111405, lectotype designated by McPherson 1981: 533, isolectotypes BM, GH, K, MEXU).

#### Description.

A small tree to 10 m, stems glabrous. Leaves petiolate, 7–14 × 3–5.5 cm, broadly lanceolate, acuminate, base truncate to shallowly cordate, glabrous or thinly pubescent abaxially near base of midrib; petioles 3–9 cm, glabrous. Inflorescence of axillary or terminal 1–3-flowered cymes often borne on short branchlets; peduncles 0.4–2 cm, glabrous; bracteoles 3–6 × 1–2.5 mm, ovate to elliptic; pedicels 2–5 cm, glabrous; sepals subequal, 13–19 × 7–13 mm, ovate, obtuse, sometimes mucronate, abaxially glabrous, adaxially pubescent; corolla 5–8 cm long, funnel-shaped, glabrous or thinly pubescent on midpetaline bands, white with greenish tube, limb 5–7 cm diam.; stamens 3–4 cm long; style globose to slightly elongate. Capsules 2–2.5 cm long, ellipsoid; seeds with long marginal hairs.

#### Distribution.

Endemic to dry scrub in central Mexico, mostly found in Jalisco but also reported from Zacatecas, Nayarit and Michoacán.

**MEXICO. Jalisco**: *C.G. Pringle* 2443 (BM, GH, K, MICH, MO, UC, US); El Cerrito, Zacoalco de Torres, *J.A. Lomeli* 3140 (MEXU); Tala, *A. Rodríguez & J. Reynosa* 1147 (MEXU); Calvillo-Guadalajara, *J.S. Miller et al.* 363 (MEXU, MO). **Michoacán**: Caula, SW of Morelia, *J.C. Soto Nuñez & L. Cortes* 2376 (MEXU). **Nayarit**: Ixtlan del Rio, *R. Acevedo & J. Sosa* 1247 (MEXU). **Zacatecas**: Juchipila, *J.J. Balleza & M. Adame* 7909 (MEXU); *E.D. Enriquez* 357 (MEXU).

#### Note.

Similar to *Ipomoea wolcottiana* and *I. pauciflora* but distinguished by the larger subequal sepals 13–19 mm long, these sometimes mucronate. The corolla is apparently larger, 7–8 cm long.

### 121. Ipomoea rzedowskii

Taxon classificationPlantaeSolanalesConvolvulaceae

E. Carranza, Zamudio & G. Murghia, Acta Bot. Mex. 45: 32. 1998. (Carranza et al. 1998: 32)

#### Type.

MEXICO. Hidalgo, Mun. Zimapan, *S. Zamudio R. & E. Pérez C*. 9970 (holotype IEB000136313, isotypes ANSM, CAS, CIIDIR, IEB, MEXU, MICH, NY, QMEX, TEX, UAMIZ).

#### Description.

Shrub to 3 m, trunk grey-green to 20 cm thick, glabrous or white-puberulent, much branched at base. Leaves petiolate, 5.5–16.5 × 1.5–5.5 cm, lanceolate to ovate, acuminate, mucronate, base rounded to subcordate, glabrescent; petioles 2–6 cm. Inflorescence of 1–3-flowered cymes from the upper leaf axils; peduncles 0.8–2.6 cm, glabrous or puberulent; bracteoles caducous, not seen; pedicels 10–30 mm, thicker than peduncles; sepals equal, 11–21 × 6–13 mm, ovate, margin scarious, glabrous or puberulent; corolla 4.5–10 cm long, campanulate to broadly funnel-shaped, white, glabrous. Capsules 15–20 × 12–15 mm, ovoid, glabrous; seeds 11–14 mm long, ovoid, brown with long white hairs.

#### Distribution.

Endemic to central Mexico, where it grows in dry scrub on steep limestone rock slopes between 700 and 2000 m.

**MEXICO. Hidalgo**: Baranca Talantango, *F. Miranda* 4022 (MEXU). **Querétaro**: Cadereyta, SE de Mesa de León, *S. Zamudio et al*. 9162 (IEB, MEXU); ibid., La Tinaja, *S. Zamudio & E. Pérez* 9966 (ARIZ, IEB); Vizarrón-San Joaquin, *R. Hernández et al.* 10618 (MEXU).

#### Note.

This species is very close to *Ipomoea intrapilosa*, differing only in the key characters.

### 122. Ipomoea chilopsidis

Taxon classificationPlantaeSolanalesConvolvulaceae

Standl., Publ. Field Mus. Nat. Hist., Bot. Ser. 17: 206. 1937. Standley 1937: 206)

#### Type.

MEXICO. Chihuahua, Quasaremos, *H.S. Gentry* 2391 (holotype F0054835, isotypes A, ARIZ, K, MEXU, MO, S, UC, US).

#### Description.

Shrub 2–5 m high, stems glabrous. Leaves shortly petiolate, 5–20 × 0.7–1.3 cm, elongate, oblong, slightly falcate, acuminate at both ends, glabrous; petioles 8–13 mm. Flowers apparently solitary, axillary; peduncles 6–18 mm; bracteoles not seem; pedicels 1–2.5 cm; sepals subequal, 12–17 × 7–9 mm, abaxially glabrous, adaxially pubescent, outer ovate, acute, mucronulate; inner elliptic, obtuse, with scarious margins; corolla 8–9 cm long, funnel-shaped, white with purple throat, glabrous, limb c. 5 cm diam., entire. Capsules 15–18 × 12 mm, shortly rostrate; seeds pilose on margins with hairs c. 10 mm long.

#### Distribution.

Endemic to the Sierra Madre Occidental in NW Mexico at 1000–1800 m on “high arid crags” in oak and pine forest.

**MEXICO. Chihuahua**: Barranca de Batopilas, *R. Felger & R. Russel* 8078-B (ARIZ); canyon of the Río Batapilas, *V. Siplivinsky et al.* 3999 (DES). **Durango**: *S. González & R.R. Clinebell* 6360 (IEB). **Sonora**: Mesa Atravesada, 1000 m, *P.S. Martin et al.* s.n. (ARIZ); Sierra Sahuaribo, *V.W. Steinmann et al.* 93-284 (ARIZ).

#### Note.

Rather distinctive because of the narrowly oblong, falcate leaves.

### 123. Ipomoea seaania

Taxon classificationPlantaeSolanalesConvolvulaceae

Felger & D.F. Austin, Sida 21: 1296. 2005. (Felger and Austin 2005: 1296)

#### Type.

MEXICO. Sonora, Mun. Guaymas, 1 km N. of Bahía San Carlos, *R. Felger & R.S. Devine* 85-301 (holotype ARIZ-BOT0005024, isotypes ARIZ, CAS, IEB, MEXU, MO, NY, RSA, SD, TEX, US).

#### Description.

Multi-stemmed shrub to 4 m, stems erect, or, upwards, sinuous or spiralling, pubescent, glabrescent, old bark whitish. Leaves shortly petiolate, 1.5–8 × 0.5–2 cm, lanceolate to ovate, apex obtuse to emarginate, base cuneate to truncate, both surfaces glabrous; petioles 2–12 mm. Inflorescence of 1–3 flowers on short shoot-like peduncles 0–5 mm long; bracteoles 5–8 mm, oblong-lanceolate, resembling tiny leaves, caducous; pedicels 8–22 mm, glabrous; sepals slightly unequal, 12–17 × 6–8 mm, abaxially thinly to densely puberulous, adaxially densely puberulous, margins scarious, outer sepals ovate, acute, inner broadly ovate to elliptic, obtuse with broad glabrous, scarious margins; corolla 4–6 cm long, narrowly funnel-shaped with tube 3.5 cm long and c. 1.5 cm wide at mouth, glabrous, white with yellowish midpetaline bands, maroon inside at base of tube, limb c. 6 cm diam. Capsules and seeds unknown.

#### Illustration.

Felger and Austin (2005: 1297).

#### Distribution.

Lower slopes of Sierra El Aguaje in desert scrub on rocky slopes near sea level in NW Mexico.

**MEXICO. Sonora**: San Carlos Bay-Catch-22 airstrip, *T.F. Daniel* 2360 (ASU, CAS).

#### Note.

The holotype was cited as deposited in UA, a non-existent herbarium code. It was apparently intended to refer to the University of Arizona (ARIZ).

### 124. Ipomoea arborescens

Taxon classificationPlantaeSolanalesConvolvulaceae

(Humb. & Bonpl. ex Willd.) G. Don, Gen. Hist. 4: 267. 1838. (Don 1838: 267)

Convolvulus arborescens Humb. & Bonpl. ex Willd., Enum. Pl. 1: 204. 1809. Type. MEXICO. Guerrero, between Acaguisootla and Chilpancingo, Humboldt & Bonpland (holotype B-W 03707-01, isotype P).
Argyreia oblonga Benth., Bot. Voy. Sulphur 133. 1844 [pub.1845]. (Bentham 1845: 133). Type. MEXICO. Nayarit, Tepic, Sinclairs.n. (holotype K000612778).
Ipomoea oblonga (Benth.) Hemsl., Biol. Cent.-Amer., Bot. 2(11): 391. 1882. (Hemsley 1882: 391).
Ipomoea murucoides var. glabrata Rose, Contr. U.S. Natl. Herb. 1: 107. 1891 (Rose 1891: 107), nom. illeg., non Ipomoea murucoides var. glabrata A. Gray. Type. MEXICO. Sonora, *E. Palmer* 316 (holotype US n.v., isotypes GH, K).
Ipomoea cuernavacensis House, Bot. Gaz. 43: 410. 1907. (House 1907b: 410). Type. MEXICO. Morelos, near Cuernavaca, *J.N. Rose & J.H. Painter* 6963 (holotype US00111384, isotype NY).
Ipomoea arborescens var. glabrata Gentry, Carnegie Inst. Wash. Publ.527: 212. 1942. (Gentry 1942: 212). Type. MEXICO. Sonora, San Bernardo, *H.S. Gentry* 1158 (lectotype ARIZ, designated by Austin et al. 2005: 1285, isolectotype MO).
Ipomoea arborescens var. pachylutea Gentry, Carnegie Inst. Wash. Publ.527: 213. 1942. (Gentry 1942: 213). Type. MEXICO. Sonora, Sierra de Alamos, *H.S. Gentry* 3000 (lectotype ARIZ, designated by Austin et al. 2005: 1285, isolectotypes K, MO, UC, US).

#### Type.

Based on *Convolvulus arborescens* Humb. & Bonpl. ex Willd.

#### Description.

Tree 5–15 m high, trunks often 50–70 cm diam., bark pale grey or yellowish (var. pachylutea), latex white, stems tomentellous with matted hairs, especially when young, glabrescent. Leaves 5–27 × 3–10 cm, ovate or lanceolate, cordate, acuminate, adaxially green, abaxially grey-tomentose, ±glabrescent except on veins, glands present at base of midrib; petioles 1.3–9 cm, tomentellous when young. Inflorescence terminal and axillary, composed 1–3-flowered raceme-like cymes borne on short branchlets, peduncles 1–5 mm; bracteoles 4–6 mm, ovate, caducous; pedicels 5–22 mm, widened upwards, tomentose; sepals subequal, 6–14 × 7–8 mm, elliptic, rounded, sometimes mucronate, tomentellous, glabrescent; corolla 4–6 cm long, subcampanulate to funnel-shaped, white with greenish tube and red throat, tomentose, at least in bud. Capsules 1.8–2.3 × 1.2–1.4 cm, ovoid, glabrous, shortly rostrate; seeds 9–16 mm long, the margins white-pilose with hairs c. 12 mm long.

#### Distribution.

Dry forest and scrub, mostly below 1000 m in western and central Mexico.

**MEXICO. Chiapas**: fide Breedlove (1986) (requires confirmation). **Chihuahua**: Barranca de Batopilas, La Bufa-Quirare, *R.A. Bye* 3415 (MEXU). **Colima**: *D.H. Lorence et al.* 3811 (MO); *R. McVaugh & Koelz* 1582 (MICH). **Durango**: Topia, *S. Acevedo & D. Bayona* 411(IEB). **Est. México & Dist. Fed.**: *J.C. Montero Castro et al.* 1255 (MO); Temascaltepec, Tejupilco, *G.B. Hinton* 447 (BM, K). **Guerrero**: Tepecoacuilco de Trujano, *J. Smith & M. Ceuterick* 0626 (ARIZ); *V.W. Steinmann & J.M. Porter* 4861 (ARIZ, IEB). **Jalisco**: *R. McVaugh* 25414 (MICH). **Michoacán**: Zitacuaro-Los Ríos, *G.B. Hinton* 13562 (K, MICH, MO, US); Aguilla, *E. Carranza & I. Silva* 6666 (IEB). **Morelos**: *E.M. Martínez & E.F. Cabrera* 11 (MO); Miacatlán, *J. Ceja & A. Mendoza* 1234 (IEB). **Nayarit**: Ixtlan del Rio, *O. Tellez* 9593 (MEXU). **Oaxaca**: *King* 1766 (MICH). **Puebla**: 5 miles SW of Tehuacan, *R. Barr & C. Mason* 23411 (ARIZ); Amatitlan, *G. Huitron* 2 (MEXU). **Querétaro**: San Joaquin, *S. Zamudio* 3222 (MEXU). **Sinaloa**: Cosala, *A.L. Reina-G et al.* 2006-10 (ARIZ); Concordia, *R. McVaugh* 23583 (MICH); *J. González Ortega* 173 (K); Las Mesas, Cerro Sirotato, *H.S. Gentry* 6144 (ARIZ, DES)–var. pachylutea . **Sonora**: Mun. Hermosillo, *A.L. Reina-G & T.R. Van Devender* 2000-889 (ARIZ); N of Hermosillo, *K.F. Parker* 8222 (ARIZ); *H.S. Gentry* 4888 (ARIZ)–var. pachylutea.

#### Notes.

The Berlin holotype of *Convolvulus arborescens* is a sterile plant cultivated in Berlin. The Paris isotype appears to be of the original collection made by Humboldt and Bonpland.

*Argyreia oblonga* Benth. is cited incorrectly as *Ipomoea oblonga* in IPNI, TROPICOS and Austin and Huáman (1996).

A rather variable species in which a number of varieties have been recognised. Var. pachylutea is often recognised by botanists who know it in the field. It is distinguished by its yellowish bark, larger and more pubescent leaves and larger flower parts, differences that are not readily discernible in the herbarium. It is found on rocky slopes and in low open woodland altitudes of 600–900 m in NW Mexico.

### 125. Ipomoea murucoides

Taxon classificationPlantaeSolanalesConvolvulaceae

Roem. & Schult., Syst. Veg. 4: 248. 1819. (Roemer and Schultes 1819: 248)

Convolvulus macranthus Kunth, Nov. Gen. Sp. 3: 95. 1818 [pub.1819]. (Kunth 1819: 95). Type. MEXICO. Guanajuato, Humboldt & Bonplands.n. (holotype P00670732).
Ipomoea macrantha (Kunth) G. Don, Gen. Hist. 4: 267. 1838. (Don 1838: 267), nom. illeg., non Ipomoea macrantha Roem. & Schult. (1819).
Convolvulus quahutzehuatl Sessé & Moc., Pl. Nov. Hisp. 23. 1887 [pub.1888]. (Sessé y Lacasta and Moçiño 1887-90: 23). Type. MEXICO. *Sessé & Moçiño*s.n. (holotype MA00603845).
Convolvulus arboreus Sessé & Moç., Pl. Nov. Hisp. 23. 1887 [pub. 1888]. (Sessé y Lacasta and Moçiño 1887-90: 23). Type. MEXICO. *Sessé & Moçiño*s.n. (MA00603835, lectotype designated here; isolectotypes BM, F, MA).

#### Type.

A cultivated plant “e horto valentino” (whereabouts unknown).

#### Description.

Tree to 13 m high, trunk to 40 cm diam., white latex abundant, stems floccose with white hairs. Leaves petiolate, 9–20 × 1–7 cm, lanceolate, oblong-lanceolate to ovate, acuminate, base broadly cuneate, usually villous or pubescent when young, somewhat glabrescent; petioles 1–6 cm, tomentose, glabrescent. Inflorescence terminal or from upper leaf axils, laxly corymbose in structure; peduncles 0.3–2 cm, villous; bracteoles ovate, obtuse 10–15 × 5–10 mm, caducous; pedicels 1.5–5 cm, thickened upwards, more densely tomentose than peduncles; sepals slightly unequal, 14–28 × 9–20 mm, oblong-ovate, obtuse to subacute, white-tomentose, the inner slightly shorter but more densely tomentose; corolla 6–9 cm long, funnel-shaped, white with dull red throat, villous; limb c. 5 cm diam., undulate. Capsules 2–2.5 cm long, oblong-ellipsoid, glabrous; seeds 12 × 5 mm, pilose on the margins with hairs 10–15 mm long.

#### Illustration.

**Figure 76.**
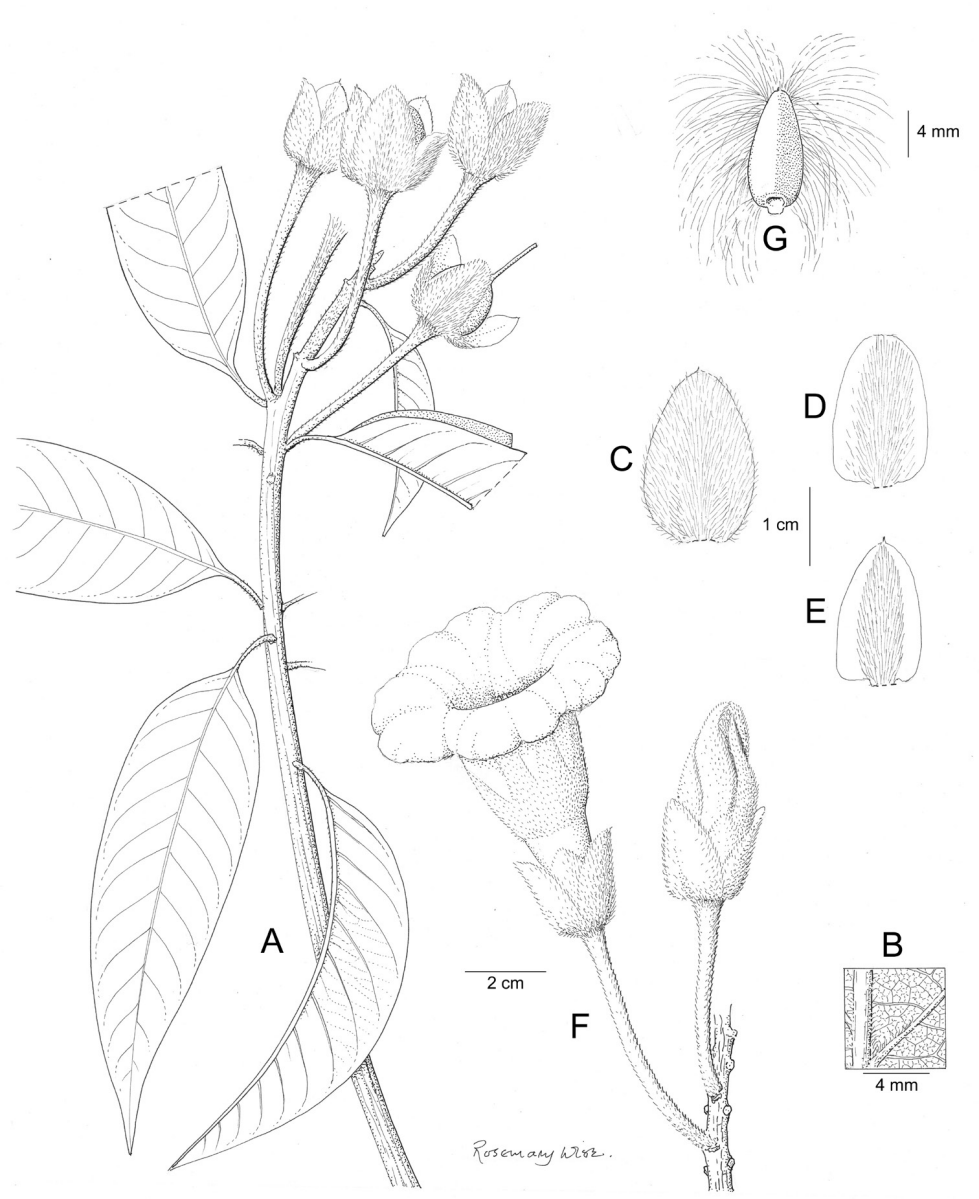
*Ipomoea murucoides*. **A** habit **B** abaxial leaf surface **C** outer sepal **D** middle sepal **E** inner sepal **F** inflorescence **G** seed. Drawn by Rosemary Wise **A, B** from *Soto* 7232; **C–E** from *Skutch* 1938; **F, G** from *Pringle* 6066.

Carranza (2007: 75). Figures 3E, 11B, 76.

#### Distribution.

Dry scrub and open deciduous woodland from 600 to 2400 m from central Mexico south to Guatemala.

**GUATEMALA.***J. Castillo* 1661 (F); *J. Donnell Smith* 1863 (K); Santa Rosa, *Heyde & Lux* 4733 (K); Guatemala City, *O. & I. Degener* 26487 (BM); Huehuetenango, *A.F. Skutch* 1938 (BM); Jalapa, *B.T. Styles* 141 (FHO).

**MEXICO. Aguascalientes**: Aguascalientes-Calvillo, *J.S. Miller et al.* 355 (MO, MEXU). **Chiapas**: *D. E. Breedlove & R.F. Thorne* 21298 (MO). **Durango**: *O.H. Soule* 2076 (MO). **Est. México & Dist. Fed.**: Mont de Guadelupe, *E. Bourgeau* 790 (BM, P, S); Ayotzingo, Chalco, *A. Ventura* 4351 (MEXU). Temascaltepec, *G.B. Hinton* 2786 (BM, K); ibid., San Lucas, *G.B. Hinton* 8755 (K). **Guanajuato**: Cerro Las Tetillas, *G. Ibarra Manríquez & G. Cornejo Tenorio* 6796 (MEXU, MO); Irapuato, *E. Martínez & C.H. Ramos* 39679 (MEXU). **Guerrero**: *J.C. Soto & E.M. Martínez* 3994 (MEXU, MO); San Pedro Atengo, *R. Cruz Duran* 2139 (MEXU). **Hidalgo**: *J.L. Flores* s.n. (MEXU). **Jalisco**: Tapalpa, *E.J. Lott & J.A. Solis Magallanes* 755 (MEXU, MO); ibid., *H.H. Iltis et al.* 816 (K, MEXU). **Michoacán**: Zitácuaro, *J.C. Soto & S. Aureoles* 7232 (BM, MEXU); Coalcomán, *G.B. Hinton* 12692 (K). **Morelos**: Cuernavaca–Cautla, *T. Croat & D.P. Hannon* 65738 (MEXU, MO). **Nayarit**: El Ocote, Sw of Yxtlan, *Y. Mexia* 800 (BM, MO). **Oaxaca**: *C.A. Pringle* 6066 (BM, K, MO, S); Santiago Chazumba, *J.I. Calzada* 24331 (K, MEXU); *M. O. Dillon* 683 (F); Zimatlan, *A. Miranda & O. Hernández* 694 (MEXU). **Puebla**: Tepoxuchil, *F. Nicholas* 622 (K); Cerro Toltepec, *J.L. Contreras* 7537 (MEXU). **Querétaro**: *E. Carranza & A. Amador* 4943 (MEXU); Los Cues, *E. Argüelles* 1933 (FTG, MEXU). **Zacatecas**: *Coulter* 1023 (K); Zapoqui, *T. Croat* 45088 (MEXU, MO); Villanueva, *E.B. Enriquez* 376 (MEXU).

#### Note.

Perhaps the most distinct of the Arborescens Clade because of the dense, white, woolly stem indumentum, large sepals and broadly cuneate leaf bases.

### 126. Ipomoea teotitlanica

Taxon classificationPlantaeSolanalesConvolvulaceae

McPherson, Contr. Univ. Mich. Herb 14: 85. (McPherson 1980: 85)

#### Type.

MEXICO. Oaxaca, Teotitlan Dist., Tambor, 17 miles W of San Antonio, *H.S. Gentry* 22475 (holotype A00054546, isotypes ARIZ, MEXU).

#### Description.

Small tree with grey trunk to 5 m high, stem and branchlets woody, tomentose with white hairs, eventually glabrescent. Leaves rather shortly petiolate, 2–5 × 1.4–5.7 cm, suborbicular, cordate, rounded to retuse, tomentose on both surfaces, adaxially grey-green abaxially white; petioles 5–16 mm, tomentellous. Flowers solitary, axillary; peduncles 0–1 mm; bracteoles 1–1.5 mm, ovate, deciduous; pedicels 4–15 mm, tomentose; sepals subequal, 11–16 × 7–10 mm long, the outer ovate, acute, abaxially tomentose, inner elliptic obtuse, only the midrib tomentose, the margin scarious; corolla 5–6 cm long, funnel-shaped, pale yellow, glabrous. Capsules narrowly ovoid, glabrous; seeds with long, lanate hairs.

#### Illustration.

McPherson (1980: 86).

#### Distribution.

Endemic to Oaxaca and neighbouring Puebla in Mexico, recorded as growing on steep sandstone slopes.

**MEXICO. Oaxaca**: Teotitlan de Flores Magon, *J.I. Calzada* 24325 (MEXU, K), ibid., 24320 (K, MEXU); ibid., El Tambor, *G. Murguía* s.n. [17/1/1991] (IEB). **Puebla**: Tehuacan-Oaxaca, *M. Cházaro & B.L. Mosthul* 7703 (IEB).

### 127. Ipomoea kahloae

Taxon classificationPlantaeSolanalesConvolvulaceae

Gonz.-Martínez, Lozada-Pérez & Ríos-Carrasco, Phytotaxa 356 (1): 50. 2018. (González-Martínez et al. 2018: 50)

#### Type.

MEXICO. Guerrero: Chilpancingo de los Bravo: a 2 km al sur del poblado de Acahuizotla, 807 m, 17°21'17.6"N, 99°27'27.4"W, 27 Aug. 2014 (fl.) *C.A. González-Martínez & S. Rios-Carrasco 390* (holotype FCME; isotypes ENCB, FCME, IEB, MEXU, XAL).

#### Description.

Perennial climber, root woody; stems 2‒5 m long herbaceous, sparsely puberulent, green, 3-winged, the wings 2‒3 mm wide. Leaves petiolate, 11‒17.5(‒21) × 13‒19(‒27) cm, 5(‒7) palmatilobed, the base cordate, the lobes unequal, basal lobes 5.8‒13(‒15) × 2‒6 cm, elliptic, lateral lobes 9.2‒16.7(‒21.5) × 2.2‒7 cm, elliptic, central lobe 9.5‒19.8(‒22) × 3.2‒9 cm, obovate, membranous, margins entire, weakly revolute, the apex acuminate-mucronate, both surfaces puberulent, adaxially green, abaxially light green to whitish, the midvein winged, sparsely puberulent; petioles 5‒13.5 cm × 1‒2.2 mm, sulcate, puberulent, winged, the wings ca. 0.4 mm wide. Inflorescence of pedunculate axillary cymes with (1‒)3‒6 flowers; peduncles 0.8‒1.1 cm, puberulent, weakly winged, not accrescent in fruit; bracteoles 1.5‒2.3 × 0.9‒1.3 cm, coriaceous, obovate, keeled, mucronate, exterior puberulent, pinkish-green; secondary peduncles 3.2‒4.3 mm; pedicels 8 mm, thickened upwards in fruit; sepals subequal, 21‒24 × 8.3‒11 mm, oblong, coriaceous, puberulent, the midvein slightly elevated, base truncate, apex obtuse and mucronate, the central part pinkish-white, the margin whitish-green; corolla c. 6.5 cm long, campanulate above a narrow, cylindrical basal tube, puberulent, white, becoming magenta upwards, the interior with magenta spots and vertical lines, the basal cylindrical tube 1.5‒2 long, the expanded part 3.7‒4 × 3‒3.5 cm, the limb 5.5‒6 cm diam., subentire, weakly 10‒lobed, magenta, glabrous. Capsules 1.4‒1.6 × 0.9‒1 cm, ellipsoid, puberulent, dark brown, the base of the style persistent, ca. 0.5 mm long, 4-seeded; seeds ca. 9.5 × 5 mm ellipsoid, the apex acute, dark brown, minutely reticulate, glabrous except for the up to 8.5 mm long marginal hairs.

#### Illustration.

González-Martínez et al. (2018: 51–53).

#### Distribution.

Endemic to Guerrero at around 800 m in semi-deciduous tropical forest.

**MEXICO. Guerrero**: Only known from a few collections cited by González-Martínez et al. (2018) from around the type locality.

#### Note.

*Ipomoea kahloae* is a very distinctive species with no obvious relatives. It is distinguished by the presence of strongly winged stems and petioles, the subsessile inflorescences with, pinkish-green, obovate keeled bracteoles, the pinkish sepals, and the unusually coloured a campanulate, magenta corolla. Its position here is suggested by molecular data published by González-Martínez et al. (2018).

•• Clade A2 (Species 128–215) is the second major clade within Clade A. It consists of perennial herbs and woody climbers or lianas. Most species are climbing plants but there are a few erect species. The leaves are sometimes absent at anthesis, particularly in the lianas that flower in the dry season. Although leaf shape is often a useful character, many mainly entire-leaved species sometimes present with 3-lobed leaves. The most distinctive feature of the clade lies in the rigid, subequal coriaceous sepals, which are usually glabrous (except in most species in the 128– 143 sequence). The corolla is glabrous (except *Ipomoea discolor* and species 129–131) and may be either hypocrateriform or funnel-shaped. The seeds, where known, are always lanate, with long marginal hairs.

The species in this clade are not always well-defined or easy to distinguish. *ITS* barcode sequences provide little resolution and our 605 nuclear region phylogeny included so few species that few inferences can be drawn, although there is a suggestion that the Caribbean species form a clade. It seems probable that many species have evolved recently often in response to a specific environmental stimulus. Particularly noteworthy is the existence of five species pairs which are vegetatively almost identical but differ markedly in the structure of their corolla. These are *I. oranensis* and *I. exserta*, *I. schulziana* and *I. suburceolata*, *I. pintoi* and *I. ana-mariae*, *I. steudelii* and *I. eggersiana*, *I. proxima* and *I. macdonaldii*, the first in each pair having a funnel-shaped corolla and the second a hypocrateriform corolla, the latter presumably an adaptation for bird pollination. There is also an interesting and problematic group of poorly defined Mexican species (*Ipomoea suaveolens*, *I. proxima*, *I. lottiae*, *I. macdonaldii*, *I. scopulorum*, *I. pseudoracemosa*, *I. pruinosa*), all with white flowers and forming a group in which some species seem to have switched from funnel-shaped corollas to hypocrateriform corollas, more appropriate for night-flowering moth-pollination. Other interesting features of the clade are the presence of species with stellate hairs both in South America and in the Caribbean and the existence of Caribbean species with the leaves arranged on brachyblasts (*I. eggersiana*, *I. steudelii*, *I. microdonta* and *I. tenuifolia*), these last all with unusually small leaves. Several species are also notable for their unusually short peduncles, the flowers thus appearing to be in axillary clusters.

The clade is well represented through most of the Americas but is particularly diverse in the Caribbean, providing all but two of the species endemic to that region. It is less common towards the north of its continental range and is almost absent from the United States.

• Species 128–131 comprise an informal group of erect Mexican species with solitary axillary flowers. They are unusual in the clade for having hirsute sepals and pubescent corollas (except *I. petrophila*).

### 128. Ipomoea petrophila

Taxon classificationPlantaeSolanalesConvolvulaceae

House, Bot. Gaz. 43(6): 408. 1907. (House 1907b: 408)

#### Type.

MEXICO. Chihuahua, *C.G. Pringle* 340 (holotype US00111439, isotypes BM, F, GH, K, LIL, NY, RSA).

#### Description.

Perennial herb to 50 cm, similar in general habit to *I. longifolia*, stem softly pubescent. Leaves shortly petiolate, 4–8 × 1.5–2.5 cm, ovate or ovate-elliptic, acute, base cuneate, softly pubescent on both surfaces; petioles 2–5 mm. Flowers solitary, axillary, long-pedunculate; peduncles 2–3.5 cm, pubescent; bracteoles caducous, not seen; pedicels 9–13 mm, pubescent; sepals slightly unequal, outer 6–8 mm, oblong-ovate, obtuse, pubescent, inner 9–11 mm, oblong-elliptic with scarious glabrous margins; corolla c. 6 cm long, funnel-shaped, glabrous, pink, limb 3.5 cm diam. Capsules 13–15 × 10 mm, ovoid with a persistent style, glabrous; seeds 9–10 × 4–5 mm, blackish, minutely puberulent and with long white marginal hairs, c. 10 mm long.

#### Distribution.

Endemic to northern Mexico where it appears to be rare in rocky grassland.

**MEXICO. Chihuahua**: near Mapula, *F. Shreve* 9060 (ARIZ); Sierra Mapula, Rancho Picacho, *E. Lehto et al.* 21586 (MEXU); Presa Chihuahua, N of El Fresno, *M. Fishbein et al.* 7383 (ARIZ).

#### Note.

This species differs from other erect species with ovate-elliptic leaves from Mexico in its hirsute vegetative parts.

### 129. Ipomoea lenis

Taxon classificationPlantaeSolanalesConvolvulaceae

House, Ann. New York Acad. Sci. 18: 188. 1908. (House 1908b: 188)

#### Type.

MEXICO. Zapatecas, near Berriozabal, *E.W. Nelson* 3889 (holotype NY00319110, isotypes GH, US).

#### Description.

Decumbent or erect perennial herb or subshrub 10–50 cm high, stem densely sericeous-pubescent, base woody, rootstock very stout and woody. Leaves subsessile, 0.6–1 × 0.2–0.4 cm, oblong-elliptic or obovate, acute or obtuse, apiculate, base narrowly cuneate, white-sericeous to tomentose on both surfaces. Inflorescence of solitary axillary flowers, becoming crowded upwards; peduncle 2–3 mm; bracteoles 1 mm, scale-like; pedicels 4–7 mm, thicker than peduncles, pubescent; sepals subequal, 7–10 mm, ovate, obtuse, outer tomentellous, inner tomentellous in central area but with broad scarious glabrous margins; corolla 4.5–6 cm long, funnel-shaped, reddish-purple with a white tube, pubescent in bud at apex, limb 4–5 cm diam. Capsules ovoid, rostrate, glabrous; seeds dark brown with short white marginal hairs.

#### Illustration.

Carranza (2007: 61).

#### Distribution.

Rare and endemic to central Mexico, principally Guanajuato, growing at around 2000–2300 m on stony slopes with low xerophytic scrub and open pine woodland.

**MEXICO. Aguascalientes**: San José de Gracia, *H. Hernández et al.* 234 (MO); Calvillo, Barranca Tortugas, *M. de la Cerda & G. García* 1549 (IEB). **Durango**: N. of Fresnillo Junction, *T. Walker* s.n. (ARIZ). **Guanajuato**: SW of Santa Bárbara, Mun. Ocampo, *E. Pérez & S. Zamudio* 3373 (IEB); Sierra de Jacales, *E. Carranza & J. Becerra* 6094 (IEB); San Pedro Almoloyán, *E. Carranza & J. Becerra* 6071 (IEB). **Jalisco**: Encarnación de Díaz, *S. Zamudio & C.A. Ramírez* 15696 (IEB).

#### Note.

An erect species with white, sericeous to tomentose vegetative parts and very small leaves. Very similar to *Ipomoea durangensis* differing in the obtuse sepals. Specimens from Aguascalientes are somewhat intermediate with *Ipomoea durangensis*.

### 130. Ipomoea durangensis

Taxon classificationPlantaeSolanalesConvolvulaceae

House, Ann. New York Acad. Sci. 18: 187. 1908. (House 1908b: 187)

#### Type.

MEXICO. Durango, *E.W. Nelson* 4639 (holotype US0036705, isotypes GH, K).

#### Description.

Subshrub to 1 m, much branched at base, stems grey-tomentellous with crisped hairs, rootstock a woody xylopodium. Leaves subsessile, 2–3.5 × 0.4–1.5 cm, oblong, base cuneate, apex rounded to obtuse, grey-tomentellous on both surfaces, whitish when young; petioles 2–4 mm. Flowers solitary, axillary; peduncles 0–1 mm; bracteoles linear, filiform, 5–10 mm long, caducous; pedicels 3–10 mm, tomentellous; sepals subequal, 12–16 × 2–3 mm, but accrescent in fruit to 22 × 5.5 mm, lanceolate, acuminate, whitish tomentose; corolla 5.5–6 cm long, funnel-shaped, pale pink with the lower part of the tube cream, sericeous in bud and on mid-petaline bands, limb 4 cm diam. Capsules 12–15 × 8–10 mm, ovoid, glabrous, rostrate; seeds 4 × 4–5 mm, pubescent on margins.

#### Distribution.

Endemic to northern Mexico, principally Durango, in dry, open grassy habitats at altitudes of 1900–2100 m.

**MEXICO. Durango**: *E. Palmer* 366 (BM, K, MO); 6 miles W of Ciudad Durango, *H.S. Gentry & R. Engard* 23614 (ARIZ); 1.7 miles NE of Federico L. Madero, *W.L. Wagner & J. Solomon* 4319 (FTG. MO); 15–20 miles NW of Durango towards La Zarca, *R.H. Hevly et al.* s.n. (ARIZ); Pipasancaro, *E.W. Nelson* 4664 (K); Pánuco de Coronado, *L. López et al.* 25 (IEB); Michilia, *Y. Herrera* 642 (IEB); Suchil, El Mirador, *P. Tenorio et al.* 5967 (MEXU). **Zacatecas**: Oja de Agua near Sombrereta, *R.H. Hevly et al.* s.n (ARIZ).

#### Note.

Very distinctive because of the oblong, subsessile leaves and solitary axillary flowers with suppressed peduncles. The acuminate sepals should be noted.

### 131. Ipomoea ciervensis

Taxon classificationPlantaeSolanalesConvolvulaceae

Painter in House, Bot. Gaz. 43: 408. 1907. (House 1907b: 408)

#### Type.

MEXICO. Querétaro, Hac. Ciervo, *Rose & Painter* 9660 (holotype US00036708, isotypes MEXU00025165, NY00319080).

#### Description.

Erect perennial subshrub to 80 cm from a tuberous rootstock, stem densely tomentose, often much branched from base. Leaves imbricate, shortly petiolate, 4–10 × 2–5 cm, elliptic, apex acute or obtuse, base cuneate, densely white-tomentose on both surfaces but paler beneath; petioles 3–5 mm. Inflorescence of solitary axillary flowers; peduncles 1.5–2 cm, densely pubescent; bracteoles 14–16 mm long, linear spathulate, tomentose; pedicels 4–9 mm; sepals subequal, 15–23 mm, lanceolate, attenuate, white-tomentose; corolla 4.5–6 cm long, funnel-shaped, white, pubescent, limb entire to undulate. Capsules 8–10 × 6–8 mm, conical, glabrous; seeds glabrous except for white marginal hairs c. 3 mm long.

#### Illustration.

Carranza (2007: 69).

#### Distribution.

Dry spiny xerophytic scrub at 2000–2250 m. Endemic to central Mexico.

**MEXICO. Guanajuato**: Mun. Cortazar, SE of El Zapote, *E. Carranza & R.M. García* 5322 (IEB, MEXU, MICH, TEX); cerca de El Zapote, *E. Carranza* 5348 (IEB). **Querétaro**: del Ciervo al Cerro de la Mesa, *F. Altimirano* 1557 (US); SE de La Trinidad, *R. Hernández* 12059 (IEB); W of El Tejocote, *J. Rzedowski* 48839 (IEB).

#### Note.

Resembles *Ipomoea durangensis* but differs in the white corolla, greyer tomentose indumentum, larger, more imbricate leaves and the longer peduncles.

### 132. Ipomoea lozanii

Taxon classificationPlantaeSolanalesConvolvulaceae

Painter in House, Botanical Gazette 43(6): 411. 1907. (House 1907b: 411)

#### Type.

MEXICO. Querétaro, San Juan del Rio, *J.M. Rose & W.H. Painter* 9542 (holotype US00111415, isotypes BM, GH, NY).

#### Description.

Twining perennial herb from a tuberous rootstock, stems somewhat woody, glabrous to thinly pilose. Leaves petiolate, 2–8 × 1.7–2.5 cm, ovate, apex long-caudate, base cordate to subtruncate and shortly cuneate onto the petiole, auricles rounded, both surfaces glabrous; petioles 2–4.5 cm, glabrous. Inflorescence of solitary (very rarely paired), pedunculate flowers, peduncle 0.5–4 cm, pubescent; bracteoles 1 mm, deltoid, caducous; pedicels 20–40 mm, stouter than peduncles and thickened upwards, nearly glabrous; sepals subequal, glabrous 12–14 × 5–7 mm, ovate, shortly mucronate, outer with scattered fleshy teeth on abaxial surface, inner without teeth but with scarious margins; corolla 5–7 cm long, funnel-shaped, deep pink, glabrous, limb c. 5 cm diam. Capsules 8–10 × 5–6 mm, ellipsoid, glabrous; seeds 4–6 mm long, subglobose, brown, puberulent.

#### Illustration.

**Figure 77.**
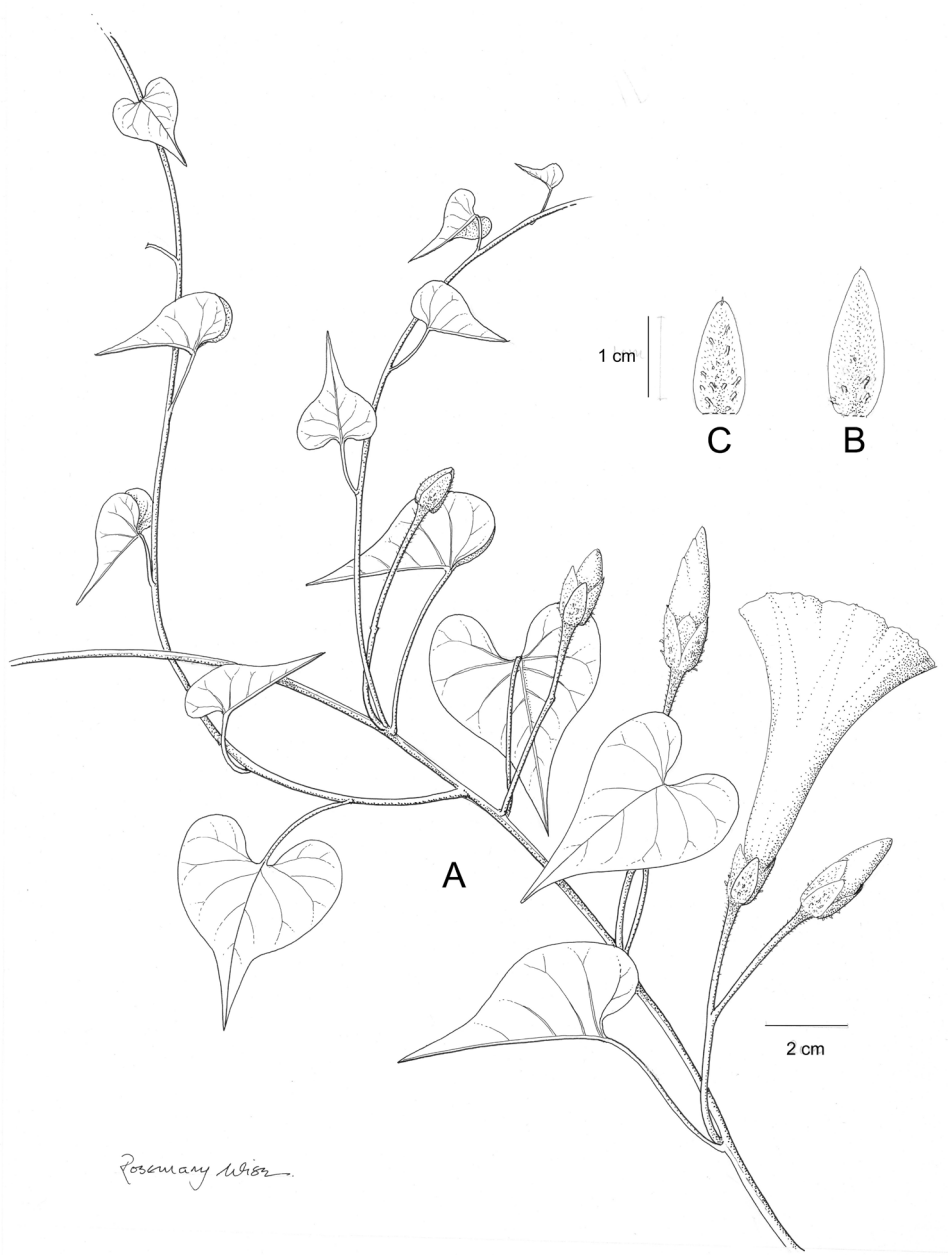
*Ipomoea lozanii*. **A** habit **B** outer sepal **C** inner sepal. Drawn by Rosemary Wise from *Pringle* 10029.

Carranza (2007: 69); Figure 77.

#### Distribution.

Endemic to central Mexico, where it grows in dry pine and oak woodland on rocky hillsides and in rough pasture derived from woodland, mostly between 1000 and 2300 m.

**MEXICO. Guanajuato**: Rincón del Cano, *E. Carranza & E. Pérez* 4995 (IEB, MEXU, TEX); Mun. Victoria, *E. Ventura y E. López* 8485 (IEB). **Hidalgo**: Tecozautla, *S. Rojas* 237 (IEB). **Querétaro**: San Juan del Rio, *C.G. Pringle* 10029 (BM, K, MO, S); Zamorano, *O. Ocampo & E. Pérez* 1221 (IEB). **Sinaloa**: El Saucito, *P. Tenorio et al.* 10292 (MEXU). **Tamaulipas**: 15 km SW of Ciudad Victoria, *G.L. Webster et al.* 11241 (S).

#### Note.

The plate accompanying the protologue is incorrect and shows *Ipomoea collina*. The correct plate is Figure 3 on page 412 of the Botanical Gazette.

### 133. Ipomoea hartwegii

Taxon classificationPlantaeSolanalesConvolvulaceae

Benth., Pl. Hartweg. 15. 1839. (Bentham 1839–57: 15)

Ipomoea albidiflora Matuda, Cact. Suc. Mex 18(3): 78. 1973. (Matuda 1973: 78). Type. MEXICO. Michoacán, *R. Hernández Magaña* 700 (holotype MEXU00204487, isotype MEXU).

#### Type.

MEXICO. *K.T. Hartweg* 96 (holotype K000612756, isotypes BM, E, GH, K, NY, OXF).

#### Description.

Twining perennial herb to c. 2 m, stems woody below, white-pubescent; root tuberous, resembling a small turnip. Leaves petiolate, small, 2–4.5 × 1.8–3.5, ovate-deltoid, pubescent, glabrescent; petioles 0.6–4.3 cm, pubescent. Inflorescence of solitary flowers (rarely in cymes with up to 3 flowers); peduncles 2.5–16 cm, glabrous or pubescent; bracteoles early caducous, not seen; secondary peduncles (if present) 1–3 cm; pedicels 10–30 mm, glabrous; sepals slightly to very unequal, scarious-margined, outer 6–8 × 2. 5–3 mm, oblong to narrowly elliptic, obtuse, abaxially hispid with bulbous-based hairs (rarely glabrous), inner 7–9 × 3–4 mm, oblong-obovate, obtuse, rounded or retuse, with broader scarious margins, glabrous; corolla 4.5–8 cm long, funnel-shaped, white with lavender flush, (sometimes pink), glabrous, limb 4–7 cm diam. Capsules 7–12 × 6–9 mm, ovoid, glabrous; seeds black, 7–8 mm long, shortly pubescent on the angles.

#### Illustration.

Figures 3D, 78.

**Figure 78.**
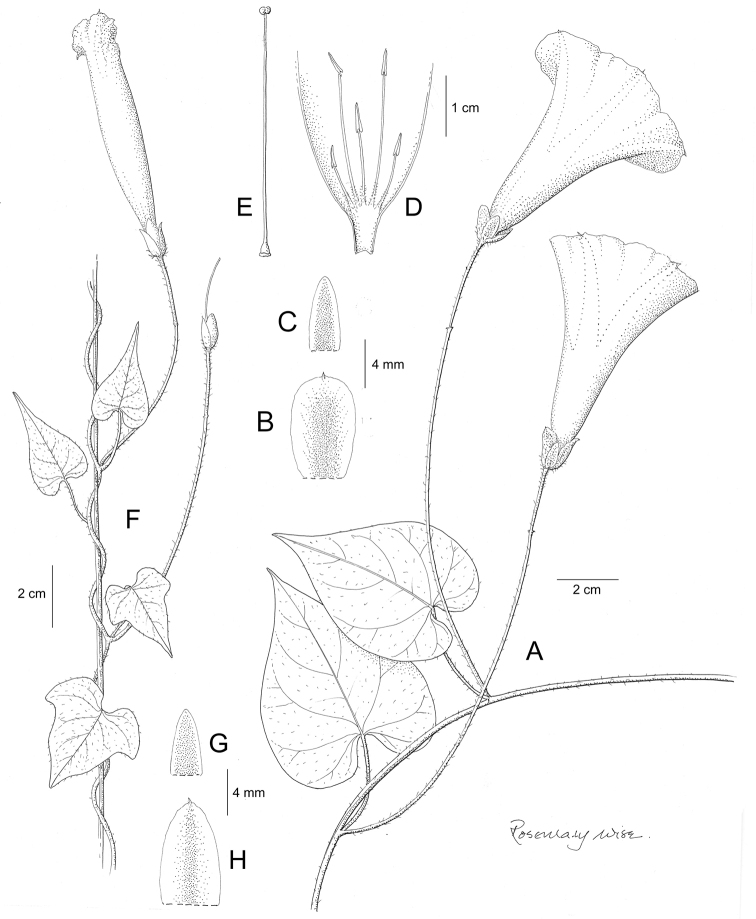
*Ipomoea hartwegii*. **A** habit **B** outer sepal **C** inner sepal **D** corolla opened out to show stamens **E** ovary and style **F** habit **G** habit **H** outer sepal **J** inner sepal. Drawn by Rosemary Wise **A** from *Santos Martínez* 2228; **B–E** from *Hinton* 8083; **F–H** from *Kral* 27530.

#### Distribution.

Endemic to central Mexico, where it grows in scrub and rough grassland at around 2000–2100 m.

**MEXICO. Chihuahua**: Río Mayo, Guasaremos, *H.S. Gentry* 1558 (ARIZ), ibid., 2333 (ARIZ, MO). **Est. México & Dist. Fed.**: Temascaltepec, Cerro Muñeca, *G.B. Hinton* 1382 (BM, K, MO), ibid., Ipericones, *G.B. Hinton* 8083 (K, P, S). **Guanajuato**: M. Doblado, *E. Carranza & E. Pérez* 4938 (IEB, MEXU); La Presa del Chupadero, *E. Ventura & E. López* 9550 (IEB, MEXU); Coroneo, *E. Carranza* 5087 (IEB, MEXU). **Guerrero**: just N. of Arteaga, *D.F. Austin & F. de la Puente* 7691 (FTG). **Jalisco**: 5 miles E of Zapotlanejo, *D. Tuttle* 279 (ARIZ); Tepatitlán-Pegueros, *R. Guzmán et al.* 950 (MEXU). **Michoacán**: Mun. Morelia, *J. Santos Martínez* 2228 (IEB, MEXU, MICH); San Bernardo *E. Carranza* 5546 (IEB, MEXU). **Morelos**: Cuernavaca, *C.G. Pringle* 13779 (ARIZ, S). **Nayarit**: Santa María del Oro, *H.S. Gentry* 11012 (ARIZ); Tepic, *R. Kral* 27530 (MO). **Querétaro**: San Juan del Rio, *C.G. Pringle* 10028 (BM, K, MEXU, MO, S); Humilpan–El Pueblito, *E. Argüelles* 3220 (MEXU). **San Luís de Potosí**: Guadalcázar, *R. Torres Colin* 15218 (MEXU). **Sinaloa**: Villa Unión, *R.L. Oliver et al.* 727 (MO). **Zacatecas**: *Coulter* 1022 (K).

#### Notes.

*Ipomoea hartwegii* is a poorly understood species. It is characterised by the long-pedunculate 1–2-flowered inflorescence and the sepals with conspicuous scarious margins. The leaves are shortly petiolate, especially in contrast to the long-pedunculate flowers and the sepals are usually abaxially hispid with bulbous-based hairs, although in some specimens they are glabrous. It is not unlike a solitary-flowered small-leaved form of *Ipomoea orizabensis*.

*Ipomoea hartwegii* is quite frequently confused with *I. proxima* (as *I. dimorphophylla*) but that species has a shortly pedunculate cymose inflorescence and the leaves are often lobed or at least with undulate margins.

• Species 134–141 are all Mexican species with white flowers and similar morphology although phylogenetic relationships between species have not been determined. Most but not all have hirsute sepals

### 134. Ipomoea cuprinacoma

Taxon classificationPlantaeSolanalesConvolvulaceae

E. Carranza & J.A. McDonald, Lundellia 7: 1. 2004. (Carranza and McDonald 2004: 1)

#### Type.

MEXICO. Michoacán, Mun. Penjamillo, *E. Carranza* 5608 (holotype IEB000187865, isotypes ENCB, IEB, MEXU, TEX).

#### Description.

Robust trailing or twining liana; stems to 14 m, canescent when young but glabrescent. Leaves petiolate, 7–14 × 4–10 cm, base truncate or cordate, apex acuminate, adaxially green, thinly to densely pubescent, abaxially grey-tomentose with some hairs reported to be branched; petioles 2.5–11 cm, pubescent. Inflorescence of pedunculate, axillary 1–3(–5)-flowered cymes, sometimes developing on short branchlets; peduncles (0.5–)1–5.5 cm, densely grey-canescent or subtomentose, somewhat glabrescent; bracteoles lanceolate, 2 mm long, grey-canescent; secondary peduncles c. 1 cm, noticeably less hairy than peduncles; pedicels 0.5–2.5 cm, densely puberulent; sepals somewhat unequal, coriaceous with pale scarious margins, glabrous; outer 5.5–8 × 4–6 mm, obtuse, inner 8–12 × 6–9 mm, truncate; corolla 5.5–8 cm long, funnel-shaped, white with purple throat, glabrous, limb shallowly lobed, c. 4–4.5 cm diam. Capsules 10–17 × 8–12 mm, ellipsoid, glabrous; seeds 7–12 mm long, glabrous apart from the pilose margins with brownish hairs 10–14 mm long.

#### Illustration.

**Figure 79.**
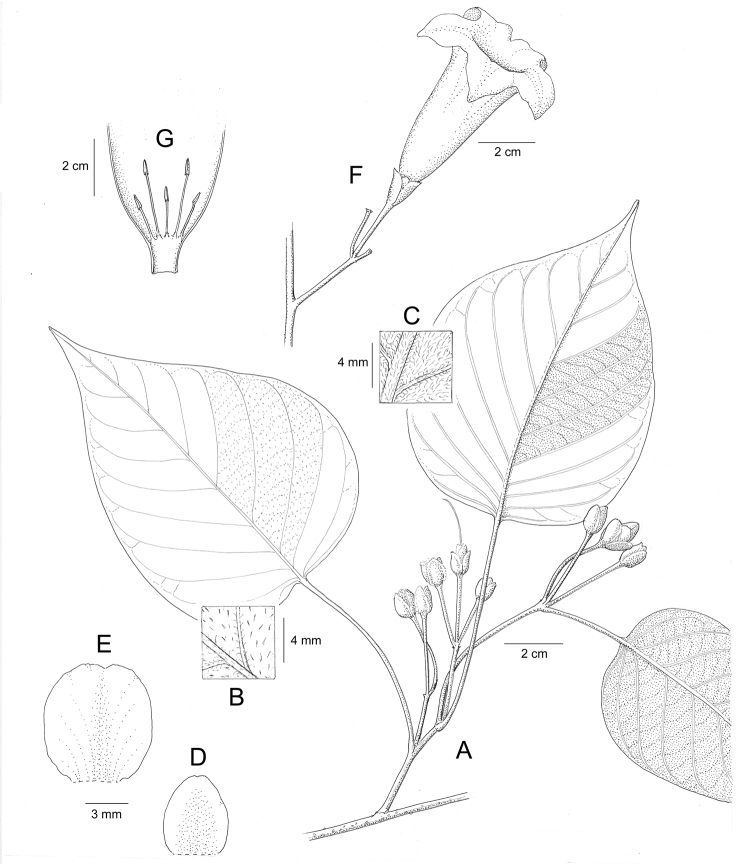
*Ipomoea cuprinacoma*. **A** habit **B** adaxial leaf surface **C** abaxial leaf surface **D** outer sepal **E** inner sepal **F** inflorescence **G** corolla opened out to show stamens. Drawn by Rosemary Wise **A, B, F, G** from *Rothschild* 352; **C, D** from *Macuca* 7220; **E** from *Labat* s.n.

Figure 79; Carranza and McDonald (2004: 2); Carranza (2007: 39).

#### Distribution.

Endemic to central Mexico and apparently uncommon to dry forest mostly between 1000 and 2000 m.

**MEXICO. Colima**: *B.M. Rothschild & T. Upson* 352 (A); *L. Vazquez & B.L. Phillips* 799 (A). **Guerrero**: Vallecito de de Zaragoza, *J.C. Soto Nuñez et al.* 9711 (MEXU); *H. Iltis et al.* 28692 (IEB, TEX). **Jalisco**: Jacotepec, Sierra La Difunta, *J.A. Macuca* 7220 (IEB, MICH); Zapopan, *P. Carrillo-Reyes* 2319 (IEB). **Michoacán**: Tzitzio, *E. Carranza & I. Silva* 6786 (IEB); Churintzio, Sanguijelas, *J.N. Labat* 1834 (IEB, MEXU, P); *C. Feddema* 51 (MICH); Mina, *G.B. Hinton et al.* 10519 (GBH, K); Penjamillo, Cuesta del Platanal, *H. Díaz & E. Pérez* 7242 (IEB). **Sinaloa**: 35 miles E. of Villa Union, *R.L. Oliver et al.* 750 (MO).

#### Note.

Although Carranza and McDonald place this species in the *Arborescens* group and compare it with *Ipomoea populina* House, this is incorrect as it clearly belongs to Clade A2. Neither the purple fruit nor the arborescent habit are obvious in herbarium specimens but specimens are usually easily identified by the large, entire leaves, which are subtomentose abaxially, the pubescent peduncles and young stems, and the few-flowered lax inflorescence. There is some variation in indumentum, some specimens being adaxially (as well as abaxially) hirsute. *Zamudio & Pérez* 10032 (IEB) from Arroyo Toliman, Mun. Zimapan (Hidalgo) looks like a glabrous form of *Ipomoea cuprinacoma*.

### 135. Ipomoea scopulorum

Taxon classificationPlantaeSolanalesConvolvulaceae

Brandegee, Zoë 5: 169. 1903. (Brandegee 1903–05: 169)

Ipomoea rhomboidea House, Ann. New York Acad. Sci. 18: 245. 1908. (House 1908b: 245).

#### Type.

MEXICO. Sinaloa, Tapolobampo, *E. Palmer* 227 (holotype US00111455, isotypes ARIZ, C, MICH, P, RSA, S).

#### Type.

MEXICO. Baja California Sur, Cape region, *T.S. Brandegee* s.n. (holotype UC105176).

#### Description.

Grey prostrate or twining perennial to 2 m, stems subglabrous, pubescent to subtomentose. Leaves petiolate, variable in form, 2–8 × 1.5–7.5 cm, ovate-deltoid, acute, cordate to truncate and cuneate onto the petiole, often shallowly 3-lobed, sometimes deeply 3-lobed with suborbicular to rhomboid lobes that are contracted below, margin somewhat undulate, both surfaces thinly to densely pubescent with simple and branched hairs, especially on the veins; petioles 1–6 cm, nearly glabrous to pubescent. Inflorescence of lax 1–5-flowered cymes; peduncles 1–3.8 cm, pubescent; bracteoles 1–1.5 mm, filiform, caducous; pedicels 15–35 mm, sometimes winged, pubescent; sepals slightly unequal, somewhat coriaceous, outer sepals 5–8 × 3–4 mm, oblong to oblong-elliptic, obtuse, mucronulate, pubescent, the margins scarious, glabrous, inner 9–13 × 6–7 mm, broadly obovate-elliptic, rounded, mucronulate, scarious except for central area; corolla 6–9 cm long, narrowly funnel-shaped, glabrous, white with bluish centre, limb 6–8 cm diam., midpetaline bands ending in a mucro; anthers usually included. Capsules 10 × 10 mm, subglobose, rostrate, glabrous; seeds 7 mm, densely pilose on the margins with hairs to 8 mm.

#### Illustration.

Figure 80.

**Figure 80.**
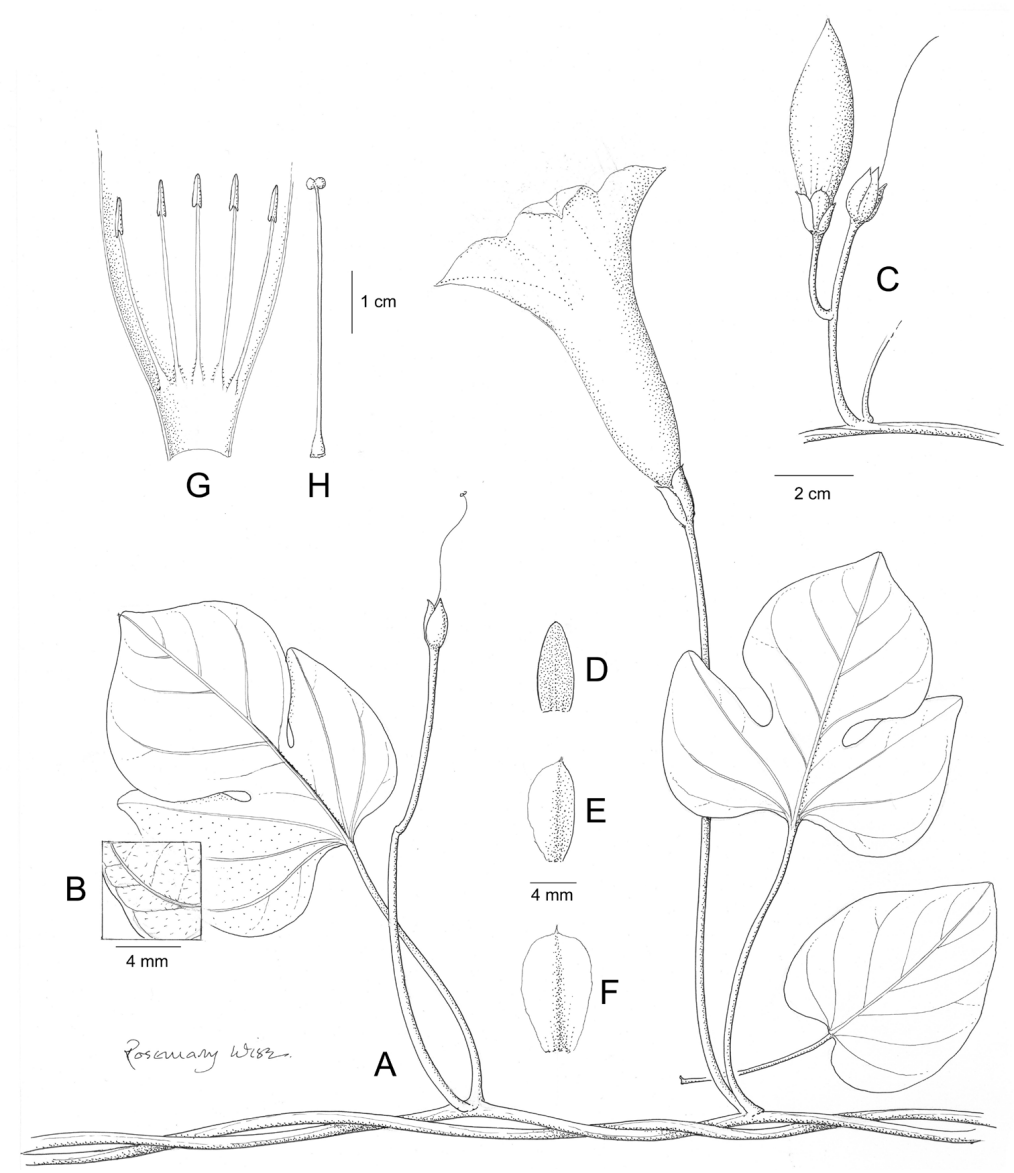
*Ipomoea scopulorum*. **A** habit **B** abaxial leaf surface **C** inflorescence with bud **D** outer sepal **E** middle sepal **F** inner sepal **G** corolla opened out to show stamens **H** ovary and style. Drawn by Rosemary Wise **A, B** from *Gentry* 1574; **C–H** from *T.S. Brandegee* s.n.

#### Distribution.

Growing amongst rocks at low altitudes in northwestern Mexico.

**MEXICO. Baja California Sur**: *T.S. Brandegee* s.n. [11/10/1904] (GH); Rancho La Burrera, *M. Domínguez* 311 (IEB). **Nayarit**: Presa Aguamilpa, *J.I. Calzada et al.* 18610 (MEXU), 18633 (MEXU). **Sinaloa**: Mazatlán, *T.S. Brandegee* s.n. [8/10/1893] (MEXU); ibid., *Ynés Mejia* 48 (MO); Culiacán, Cerro Piedrera, *M. Provance* 9616 (MO, UCR); Presa El Comedero, *R. Vega Aviña et al.* 6098 (MEXU); Sierra de Tacuichamona, *R. Vega Aviña et al.* 6698 (MEXU). **Sonora**: San Bernardo, Río Mayo, *H.S. Gentry* 1574 (ARIZ, F, K, MEXU, MO, S); (ARIZ); Mun. Soyopa, Río Yaqui, *M. Fishbein et al.* 3573 (ARIZ, MO); Mun. Sahuaripa, *A.L. Reina-G et al*. 2003-937 (ARIZ).

#### Typification.

The specimen of *Ipomoea scopulorum* at MEXU (00025258) is a paratype, not an isotype as labelled.

#### Note.

This species is rather variable in leaf size and shape, indumentum and corolla size. Entire leaves are deltoid and basally truncate, but the deeply 3-lobed leaves have the terminal leaflet somewhat rhomboid in form. The indumentum is quite variable in its density and the branched hairs are not easily discerned even with a microscope.

### 136. Ipomoea lottiae

Taxon classificationPlantaeSolanalesConvolvulaceae

McDonald, Biótica 12(3): 219. 1987. (McDonald 1987a: 219)

#### Type.

MEXICO. Jalisco, La Huerta, Est. Biologia, Chamela, *E. Lott, J.A. Solis & S.H. Bullock* 1833 (holotype MEXU00448374, isotypes MO, US, XAL).

#### Description.

Twining perennial, stems woody and wiry, pubescent. Leaves petiolate, 2–6.5 × 2–7 cm, ovate or, more commonly, 3-lobed, acute or obtuse, mucronate, basally cordate to subtruncate and then cuneate onto the petiole, adaxially thinly adpressed pubescent, abaxially silvery, adpressed pilose; petioles 1–4 cm. Inflorescence of few-flowered pedunculate cymes; peduncles 1–2.7 cm, pubescent; bracteoles linear c. 4 × 0.5 mm; pedicels 10–23 mm, pubescent; sepals unequal, outer 3–4 × 2–3 mm, ovate, obtuse and mucronate, thinly pubescent, inner larger, 6–7 × 2–4 mm, obovate-elliptic, retuse, the margins broadly scarious; corolla 4–5.5 cm long, salverform the tube 2–2.5 cm long, glabrous, cream, opening at night; stamens equal, very short; anthers and style included. Capsules 9–11 × 7 mm, glabrous, ovoid, muticous; seeds 5 × 3 mm, long-pilose on the margins with hairs up to 12 mm long.

#### Illustration.

McDonald (1987a: 220).

#### Distribution.

Almost endemic to the Chamela region in dry deciduous forest at low altitudes.

**MEXICO. Guerrero**: *Cortez & Lozano* 2621 (MEXU), fide McDonald (1987). **Jalisco**: Chamela, *A. Gentry & L. Woodruff* 74402 (FTG, MO); *E. Lott* 1207 (MEXU, MO); 11 km S of Guadalajara, *M. Harker & H. Mellowes* 91 (BM); **Michoacán**: Aquila, Barranca de Chila, *J.C. Soto et al.* 2621 (IEB).

#### Note.

This species is distinguished by the white salverform corolla and 3-lobed leaves, but is otherwise very similar to *Ipomoea proxima and I. scopulorum*.

### 137. Ipomoea proxima

Taxon classificationPlantaeSolanalesConvolvulaceae

(M. Martens & Galeotti) Hemsl., Biol. Cent.-Amer., Bot. 2(11): 1882. (Hemsley 1882: 392)

Calonyction proximum M. Martens & Galeotti, Bull. Acad. Roy. Sci. Bruxelles 12: 268. 1845. (Martens and Galeotti 1845: 268). Type. MEXICO. Oaxaca, Yavezia, *H. Galeotti* 1378 (holotype BR0008676993, isotype BR00008676115).
Ipomoea dimorphophylla Greenm., Proc. Amer. Acad. Arts 33(25): 482. 1898. (Greenman 1898: 482). Type. MEXICO. Morelos, near Cuernavaca, C.G. *Pringle* 6658; (lectotype GH n.v., designated (as type) by House (1908b: 257), isolectotypes AC, BKL, BM, BR, CM, E, ENCB, F, K, M, MEXU, MICH, MO, MSC, NY, P, PH, S, US, VT).
Ipomoea oaxacana Greenm., Publ. Field Mus. Nat. Hist., Bot. Ser. 2(8): 336. 1912. (Greenman 1912: 336). Type. MEXICO. Oaxaca, Cerro San Antonio, *C. Conzatti* 2057 (holotype F225829, isotype F).

#### Type.

Based on *Calonyction proximum* M. Martens & Galeotti

#### Description.

Perennial climbing herb with tuberous roots, stem pubescent but somewhat glabrescent, woody. Leaves petiolate, 2.5–4.5 × 1–4.5 cm, ovate, entire or shallowly 3-lobed, acute to acuminate, mucronulate, base truncate to shallowly cordate, adaxially thinly pubescent, glabrescent, abaxially pubescent to grey-tomentose; petioles 3–4.5 cm. Inflorescence of shortly pedunculate 1–6 flowered axillary cymes, sometimes developing on leafy side shoots; peduncles short, 0.3–1 cm puberulent; bracteoles caducous; secondary peduncles 5–10 mm; pedicels 10–20 mm, densely tomentellous, slightly thickened upwards; sepals slightly unequal, ovate to suborbicular, obtuse or rounded, coriaceous, glabrous, margin scarious, outer 5–6 × 4–5 mm, inner 6–8 × 5–6 mm; corolla 5–7 cm long, funnel-shaped, white, glabrous, limb 5–6 cm diam. Capsules subglobose, c. 10 mm, glabrous; seeds 7–9 mm, dark brown, with long white, marginal hairs.

#### Illustration.

**Figure 81.**
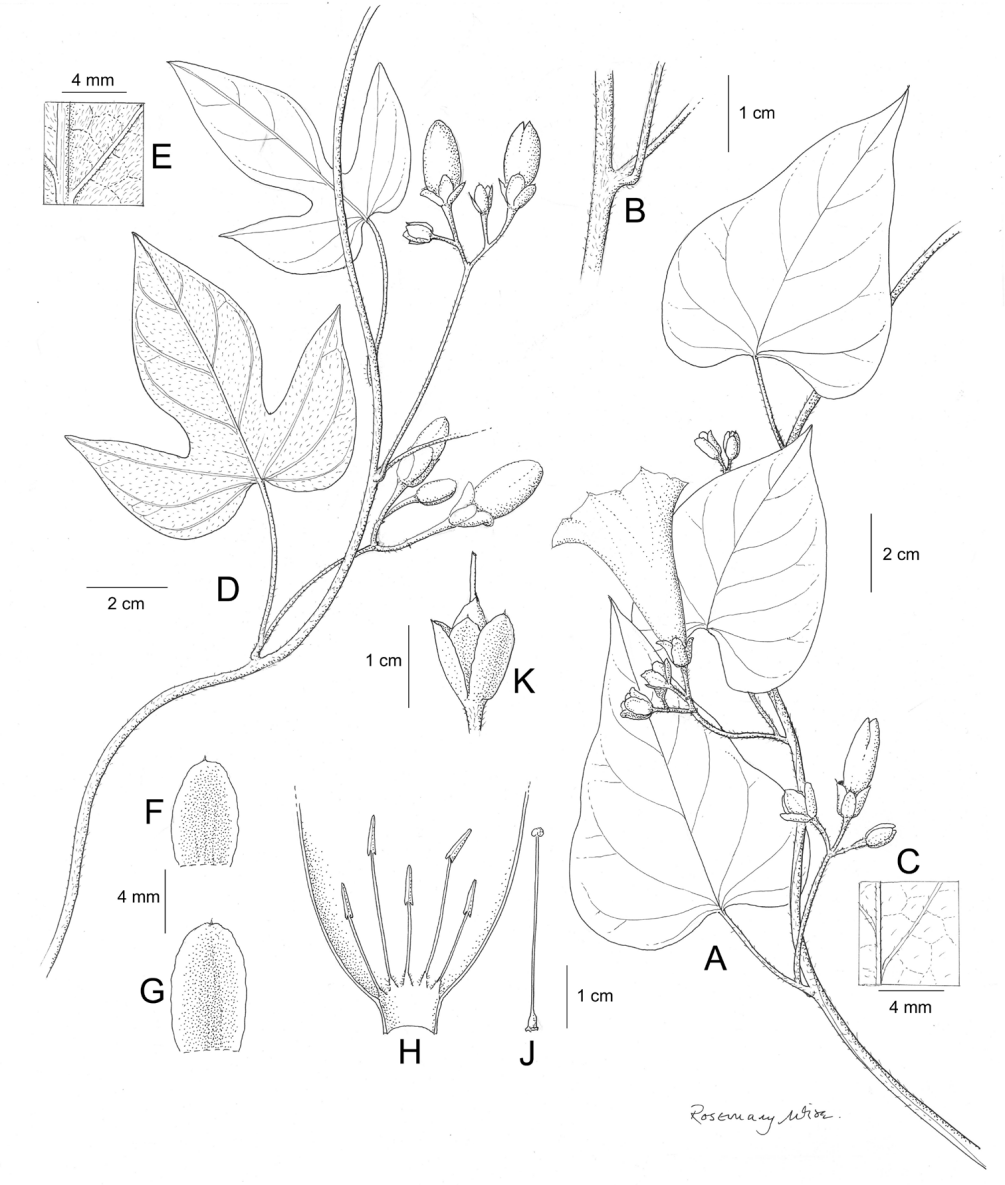
*Ipomoea proxima*. **A** habit with flowers **B** stem **C** adaxial leaf surface **D** habit with buds **E** abaxial leaf surface **F** outer sepal **G** inner sepal **H** corolla opened out to show stamens **J** ovary and style **K** fruit. Drawn by Rosemary Wise **A–C** from *Hinton* 1330; **D–J** from *Hinton* 8262; **K** from *Tenorio* 7147.

Figure 81; Carranza (2007: 97).

#### Distribution.

Oakwoods in the mountains of south-central Mexico at 1800–2500 m.

**MEXICO. Est. México & Dist. Fed.**: Temascaltepec, Telpintla, *G.B. Hinton* 1139 (K); ibid., Tequisquipan, *G.B. Hinton* 1330 (K); ibid., Rincón, *G.B. Hinton* 1547 (K), ibid., 3262 (K). **Guanajuato**: Xichú, *S. Zamudio* 13627 (IEB). **Guerrero**: Vallecitos, Montes de Oca, *G.B. Hinton* 11480 (K). **Michoacán**: *E. Carranza et al.* 7625 (IEB); Aguililla, Apatzingan, *G.B. Hinton et al.* 15188 (GH, MO). **Oaxaca**: *Ghiesbrecht* s.n. (P); Cerro la Culebra, SW de el Enebro, *P. Tenorio et al.* 7147 (MEXU, MO); Santo Domingo Tonalá, *A. Torres Hernández* 505 (IEB); Pochutla, San Miguel del Puerto, *J. Pascual* 550 (ASU); Juchitán, *A. Saynes & A. Sánchez* 3609 (MO). **Puebla**: fide Carranza (2007). **Querétaro**: Tilaco, *E. Carranza & Z. Ortega* 7357 (IEB); Jalpán, *E. Carranza et al.* 7580 (IEB). **San Luís de Potosí**: *C.A. Purpus* 5403 (BM, MO).

#### Notes.

A poorly understood species characterised by the white corolla, truncate, pubescent, usually shallowly lobed leaves and shortly pedunculate cymes often arising on leafy side shoots. There is some variation in indumentum, specimens from Oaxaca having pubescent pedicels and sepals, whereas they are glabrous in the Temascaltepec specimens, *Hinton* 11480 from Guerrero and the lectotype of *Ipomoea dimorphophylla*. *Ipomoea dimorphophylla* was said by Austin et al. (2012) to be conspecific with *Ipomoea batatoides* and the two species were confused by Matuda, but *I. batatoides* is usually pink-flowered with glabrous leaves and is a plant of moist lowland forest.

This species is very close to *Ipomoea scopulorum* from NW Mexico differing in the shorter, more rounded, only slightly unequal sepals. It may intergrade with *Ipomoea suaveolens* but that species has spreading, stiff hairs on the stem and usually also on the pedicels and sepals as well as a narrowly funnel-shaped corolla.

### 138. Ipomoea macdonaldii

Taxon classificationPlantaeSolanalesConvolvulaceae

E. Carranza, Brittonia 63: 66. 2011. (Carranza 2011: 66)

#### Type.

MEXICO. Oaxaca, Pochutla, Mun. Santa Maria Huatulco, *E. Carranza et al.* 7430 (holotype IEB0225154, isotypes IEB, MEXU, NY).

#### Description.

Twining perennial herb 6–10 m high, stems glabrous. Leaves petiolate, 6.5–12 × 4–10 cm, ovate, sometimes 3-lobed to nearly halfway, acuminate, mucronate, base truncate and briefly cuneate onto the petiole, glabrous except for the pilose margin; petioles 2–8 cm. Inflorescence of long-pedunculate compound axillary cymes; peduncles 10–28 cm, glabrous; bracteoles 1.5–4 mm, ovate, caducous; secondary peduncles 1.5–4 cm; tertiary and quaternary peduncles slightly shorter; pedicels 17–30 mm; sepals slightly unequal, glabrous, coriaceous, outer 5.5–6.5 × 2.5–4 mm, oblong-elliptic, convex, obtuse, scarious-margined, inner 7–9 × 4–6 mm, elliptic-obovate, truncate or retuse; corolla c. 5 cm long, hypocrateriform, the tube subcylindrical, 4–5 cm long, white, glabrous, the limb lobed, stamens exserted; Capsules 11–13 × 8 mm, ellipsoid, the style base persistent; seeds 5–7 × 3 mm long, the margins pilose with hairs 10 mm long.

**Illustration**: Carranza (2011: 66).

#### Distribution.

At low altitudes below 200 m near the coast in the coffee zone in Pochutla region of Oaxaca,

**MEXICO. Oaxaca**: Mun. Santa Maria Huatulco, *A. Sánchez Martínez et al.* 1210 (IEB, MEXU); Mun. Santiago Astata, *M. Elorsa* 7526 (MEXU); Mun. San Carlos Yautepec, *N. Velasquez et al.* 453 (MEXU); Mun. San Miguel del Puerto, *J. Riveira et al.* 2003 (MBM); ibid., *S.H. Salas & A. Sánchez* 6133 (IEB).

#### Notes.

This species is very close to *Ipomoea lottiae* differing principally in the nearly glabrous leaves (except pilose margins) and exserted stamens. It also resembles *Ipomoea proxima* but is distinguished by the hypocrateriform corolla.

The following specimens from central Mexico are identical with *I. macdonaldii*, even to the pilose leaf margins, except for the funnel-shaped corolla. They differ from *Ipomoea pseudoracemosa* in the relatively long peduncle 3–13 cm in length as well as the presence of leaves at anthesis. The leaves are petiolate, ovate-deltoid, 4–8 × 4–7 cm, acuminate to a shortly mucronate apex, the base subtruncate and very shortly cuneate onto a petiole 1.5–3.5 cm.

**MEXICO. Est. México & Dist. Fed.**: Temascaltepec, Guayabal, *G.B. Hinton* 8528 (F, GBH, K, MO), ibid., Tejupilco, *G.B. Hinton* 8554 (K). **Michoacán**: Coalcomán, *G.B. Hinton* 12464; Huetamo, *G.B. Hinton* 13324 (K).

### 139. Ipomoea pseudoracemosa

Taxon classificationPlantaeSolanalesConvolvulaceae

McPherson, Contr. Univ. Michigan Herb. 14: 88. 1980. (McPherson 1980: 88)

#### Type.

MEXICO. Jalisco, 6.5 miles NE of Autlán, *R. McVaugh & W.N. Koelz* 1037 (holotype MICH1111340).

#### Description.

Liana, stems up to 5 m long, woody, pubescent, glabrescent. Leaves usually absent at anthesis, not certainly known. Inflorescence of shortly pedunculate axillary clusters of reduced cymes; peduncles 0.2–3 cm, pubescent; bracteoles 2–4 mm, deltoid, caducous; secondary peduncles 0.5–4 mm, glabrous; pedicels 5–17 mm, glabrous; sepals slightly to very unequal, suborbicular, obtuse, rounded or retuse, convex, coriaceous, glabrous or thinly comose at the apex, outer 2.5–4 × 3–4 mm, inner 4–6 × 6 mm, the margins scarious; corolla 5–7 cm long, funnel-shaped, white, glabrous; limb 3.5–6 cm diam.; stamens included. Capsules ovoid, 12–13 × 6–7 mm, glabrous; seeds 6 × 4 mm, long pilose on the margins with hairs 7–9 mm long.

#### Illustration.

McPherson (1980: 90).

#### Distribution.

Endemic to central Mexico on dry scrub-covered hills between 900 and 1500 m.

**MEXICO.** Sine data, *E. Langlassé* 862 (K, P). **Est. México & Dist. Fed.**: Temascaltepec, *G.B. Hinton* 5331 (K). **Guerrero**: Mina, *G.B. Hinton* 9815 (K), 10088 (K); Río de Oro–Zihuatanejo, *C. Rafael Torres et al.* 7741 (MEXU). **Jalisco**: San Cristóbal de la Barranca, *R. McVaugh* 22141 (MICH); Autlán, *E. Carranza & I. Silva* 7175 (IEB). **Michoacán**: Churumuco, *I. Solorio Herrera* 12 (IEB); ibid., *G. Ibarra Manríquez* 6657 (MEXU); La Huacana, *V.W. Steinmann* 3029 (IEB). **Nayarit**: 10 miles SE of Ahuacatlán, *R. McVaugh & W.N. Koelz* 728 (MICH). **Zacatecas**: Moyahua, Cerro La Cantarilla, *E.D. Enriquez* 816 (MEXU).

#### Note.

Distinguished from other similar species by the glabrous, funnel-shaped white corolla, very short peduncles and short, glabrous sepals. The type and all the specimens cited above are leafless so it is very difficult to characterise this species reliably.

### 140. Ipomoea pruinosa

Taxon classificationPlantaeSolanalesConvolvulaceae

McPherson, Contr. Univ. Michigan Herb. 14: 88. 1980. (McPherson 1980: 88)

#### Type.

MEXICO. Guerrero, Casa Verde to Xochipala, *R. McVaugh* 22192 (holotype MICH1000057, isotypes ENCB, MEXU).

#### Description.

Liana to 5 m, stems tomentose, eventually glabrescent. Leaves unknown, absent at anthesis. Inflorescence of compound axillary cymes borne towards the tips of branches; peduncles 0.2–0.6 cm, sericeous; bracteoles caducous, unknown; pedicels 2–9 mm, thickened upwards, sericeous; sepals subequal, ovate or ovate-elliptic, obtuse and sometimes mucronate, coriaceous, sericeous, outer 5–6 × 3–4 mm long, inner 6–7 × 5 mm, the margins broad, scarious; corolla 6.5–9 cm long, funnel-shaped, white with reddish midpetaline bands, sericeous, limb 4.5–7 cm diam., unlobed; stamens included. Capsules 12–15 mm long, oblong-ovoid, shortly rostrate, glabrous; seeds pilose on the margins.

#### Illustration.

McPherson (1980: 89).

#### Distribution.

A little known species, apparently endemic to Guerrero State in Mexico.

**MEXICO. Guerrero**: Casa Verde, *H. Kruse* s.n. [14/2/1970] (MEXU); Eduardo Neri, Venta Vieja, *A. A. Aguilar* 34 (MEXU); Zopilote canyon, Chilpancingo –Río Balsas, *B. Mostul* 1161A (OXF).

#### Note.

The large, nearly white, sericeous corolla, the subequal sericeous sepals and the included anthers distinguish this species, which is leafless when flowering. There is one leaf on *Aguilar* 34. It is 4.5 × 4 cm, suborbicular, abruptly acute, basally cuneate, glabrous, abaxially white, strongly reticulate.

### 141. Ipomoea suaveolens

Taxon classificationPlantaeSolanalesConvolvulaceae

(M. Martens & Galeotti) Hemsl., Biol. Cent.-Amer., Bot. 2: 394. 1882. (Hemsley 1882: 394)

Convolvulus suaveolens M. Martens & Galeotti, Bull. Acad. Roy. Sci. Bruxelles 12: 261. 1845. (Martens and Galeotti 1845: 261). Type. MEXICO. Oaxaca, *H. Galeotti* 1376 (holotype BR000697274; isotypes BR, G, K, P).
Ipomoea rostrata A. Peter, Die Natürlichen Pflanzenfamilien 4 (3a): 30. 1897 [pub. 1891]. (Peter 1891: 30. Type. GUATEMALA. Retaluleu, *Bernouilli & Cario* 1932 (lectotype GOET005708, designated by Staples et al. 2012: 675).
Ipomoea crinita Brandegee, Zoë 5(10): 216. 1905. (Brandegee 1905: 216). Type. MEXICO. Sinaloa, Culiacán, *T.S. Brandegee*s.n. (holotype UC105119, isotypes GH, NY, US).
Ipomoea ursina Brandegee, Univ. Calif. Publ. Bot. 4(19): 382. 1913. (Brandegee 1913: 382). Type. MEXICO. Veracruz: Baños de Carrizal, C.A. Purpus 6240 (holotype UC167862, isotypes BM, F, GH, MO, NY, US).

#### Type.

Based on *Convolvulus suaveolens* M. Martens & Galeotti

#### Description.

Perennial night-flowering liana to 5 m, stems relatively stout, woody below, bristly white-pilose, latex white. Leaves petiolate, 3–12 × 3–8.5 cm, ovate, acute to shortly acuminate, shallowly cordate to truncate, occasionally 3-lobed, thinly hispid-pilose on both surfaces, eventually somewhat glabrescent, abaxially paler; petioles 1.5–5.5 cm, pilose. Inflorescence of long-pedunculate, sometimes leafy, many-flowered, compound cymes; peduncles 5–14 cm, hispid-pilose; lower bracteoles foliose, 10 × 2 mm, lanceolate; upper bracteoles 2 mm, filiform, caducous; secondary peduncles 1–1.5 cm; tertiary peduncles c. 5 mm; pedicels 8–14 mm; sepals slightly unequal, outer 5–8 × 3 mm, oblong-ovate to elliptic, obtuse, convex, densely hispid-pilose, especially near margins, inner 7–8 × 4 mm, obovate, with prominent broad, glabrous, scarious margins; corolla 5–7 cm long, narrowly funnel-shaped above a subcylindrical basal tube, white (night flowering), glabrous, limb c. 4 cm diam., unlobed. Capsules 10–12 mm long, conical, rostrate with persistent style, glabrous; seeds 6–9 mm, glabrous apart from long deciduous marginal hairs.

#### Distribution.

Deciduous dry forest and thorn scrub on mountains of Central America and southwestern Mexico, 0–1900 m.

**EL SALVADOR.** Ahuachapán, *J.M. Rosales* 968 (BM, LAGU); ibid., *T. Croat* 42098 (MO).

**GUATEMALA.** Huehuetenango, *M. Véliz et al.* 99.7619 (MEXU, MO).

**MEXICO. Chiapas**: Berriozábal, *D.E. Breedlove* 20393 (MO); Venustiano Carranza, Soyatitán, *A. Shilom Ton* 3129 (F); Teopisca, *H. Mejia & A. Luna* 754 (IEB); Yautepec, *D. López* 288 (IEB). **Guerrero**: Eduardo Neri, La Yesera, *J.C. Soto* 1092 (MEXU); *O. Tenorio et al*. 1263 (MO); *J.N. Rose et al.* 9339 (US). **Nayarit**: Tuxpan, Microondas Peñitas, *R. Ramírez-Delgadillo et al.* 7404 (IEB). **Oaxaca**: Juchitán, *C. Gallardo-H. & E. Pérez-G* 1515 (MO). **Sinaloa**: *E. Guizar* 3319 (MEXU); Los Labrados, *Y. Mexia* 913 (BM, F, MO). **Veracruz**: Apazapan, Baños del Carrizal, *C.A. Purpus* 6240 (F, MO).

**Notes**. Records from Costa Rica, for example *B.E. Hammel & I. Pérez* 24994 (CR, MO) appear to be all errors for white-flowered forms of *Ipomoea batatoides*.

Distinguished by the stiff, spreading white hairs of the calyx and stems combined with the narrow white corolla, which is funnel-shaped above a long basal cylindrical tube. The peduncles are often long and the sepals very short, often c. 5 mm long.

Some specimens have hirsute stems but glabrous sepals and may be intermediate with *I. pseudoracemosa* or *I. proxima*, such as *J.C. Soto Nuñez* 9877 (MEXU), *S. Valencia Avalos* 1004 (MEXU) and *Monroy de la Rosa* 220 (MEXU) all from Guerrero. *Breedlove* 27392 (MO) lacks corollas but appears to be intermediate with *Ipomoea batatoides*.

### 142. Ipomoea pogonocalyx

Taxon classificationPlantaeSolanalesConvolvulaceae

J.R.I. Wood & Scotland, Kew Bull. 72 (10): 9. 2017. (Wood and Scotland 2017b: 9)

#### Type.

BRAZIL. Maranhão, Mun. Tuntum, Palmerinha, 74 km de Tuntum, *J.U. Santos, E.L. Taylor, G.E. Schotz, N.A. Rosa, C.S. Rosário, T. Rebbeck, J.F. Silva & M.R. Santos* 711 (holotype MG, isotypes FTG, K, NY, US).

#### Description.

Twining perennial herb to 1.5 m, stem and all vegetative parts hirsute with rather stiff, whitish, spreading bulbous-based hairs. Leaves petiolate, 4–13 × 3.5–11 cm, ovate, cordate with rounded auricles, margin entire to slightly undulate, apex abruptly acute, both surfaces hirsute but abaxially paler; petioles 1.7–7.5 cm, hirsute. Inflorescence of axillary pedunculate cymes, usually with 5 flowers; peduncles 2.5–8.5 cm, hirsute, appearing somewhat flexuose; bracteoles 3–4 × 0.5 mm, linear, acuminate, caducous; secondary peduncles 1.3–2.3 cm; pedicels relatively long, 1.6–3.6 cm, slightly more hirsute than peduncles; sepals subequal, 12–15 × 8–9 mm, coriaceous, convex, elliptic-obovate, outer obtuse, abaxially hirsute when young, but hairs somewhat deciduous on the upper part when old, inner sepals rounded, glabrous except for a few hairs near base; corolla 6.5–7 cm long, funnel-shaped, pink, glabrous; limb c. 3.5 cm diam. Capsules and seeds not seen.

#### Illustration.

Figure 82.

**Figure 82.**
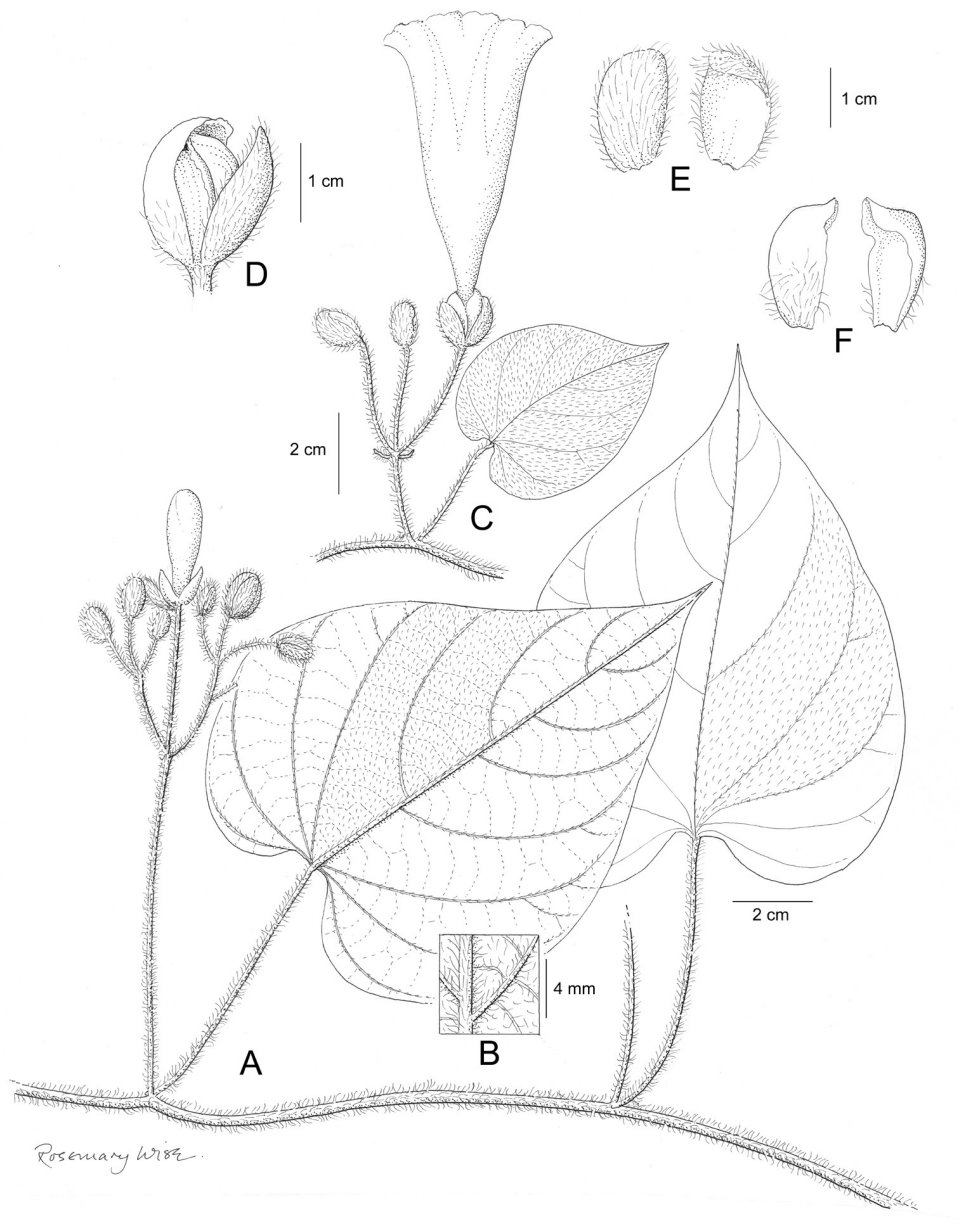
*Ipomoea pogonocalyx*. **A** habit **B** abaxial leaf surface **C** inflorescence with corolla **D** flower bud **E** outer sepal, external (left) and internal (right) surfaces **F** inner sepal, external (left) and internal (right) surfaces. Drawn by Rosemary Wise **A, D–F** from *J.U. Santos et al.* 711; **B–C** from *Pereira-Silva & Moreira* 11328.

#### Distribution.

Rocky ground in seasonally semi-deciduous forest. Endemic to Amazonian Brazil.

**BRAZIL. Maranhão**: Estreito, *G. Pereira-Silva & G.A. Moreira* 11328 (CEN).

#### Note.

Readily recognised by the long, somewhat stiff, spreading bulbous-based hairs that cover all vegetative parts including the outer sepals. The pink flowers and relatively long sepals distinguish it from *Ipomoea suaveolens*. The rather long pedicels suggest an affinity with *I. batatoides* and also perhaps with the next species represented by *Rosa and Santos* 2011.

### 143. Ipomoea

Taxon classificationPlantaeSolanalesConvolvulaceae

sp. C (Rosa & Santos 2011)

#### Description.

Twining perennial of unknown height; stems pilose. Leaves petiolate, 6–13 × 3–7.5 cm, oblong-ovate, base cuneate, margin entire to slightly undulate, apex acuminate and strongly mucronate, adaxially green, pilose with bulbous-based hairs, abaxially grey-tomentose with dense, soft appressed hairs; petioles 2–3.5 cm, pilose. Inflorescence of axillary, pedunculate, often compounded cymes; peduncles 4–7 cm, thinly pilose; bracteoles caducous, not seen; secondary peduncles 1.6–2.8 cm; tertiary peduncles 0.6–1,1 cm; pedicels 0.9–2.5 cm, thinly pilose; sepals subequal, coriaceous, convex, 7–9 × 3–4 mm, outer obtuse to subacute, glabrous but pilose near base, inner glabrous, margins scarious; corolla not seen. Capsules 6–7 × 5 mm subglobose, rostrate with persistent style 3–4 mm, glabrous; seeds 4 × 2.5 mm, blackish, glabrous apart from long marginal hairs c. 6 mm in length.

#### Distribution.

Endemic to Mato Grosso.

**BRAZIL. Mato Grosso**: Rio Juruena, cachceira Santa Iria ponto (SC. 21VB), 25 May 1977, *Rosa & Santos* 2011 (F, FTG, MG, NY).

#### Note.

Distinguished from *Ipomoea batatoides* and related species by the distinctive oblong leaves with the softly appressed pilose indumentum on the abaxial surface. We have not described this species formally as no flowering material is available.

• Species 144–171 form the core of this clade, all with glabrous corollas and sepals, only the first and last somewhat uncertain in their placement.

### 144. Ipomoea mirabilis

Taxon classificationPlantaeSolanalesConvolvulaceae

Ferreira & Sim.-Bianch., Phytotaxa 1355 (1): 30. 2013. (Ferreira et al. 2013: 30)

#### Type.

BRAZIL. Rio Grande do Sul, Tio Hugo, *P.P.A. Ferreira & J. Durigon* 702 (holotype ICN, isotype S).

#### Description.

Twining perennial; stems woody, tomentellous, glabrescent; latex white. Leaves petiolate, 8–15 × 6–10 cm, ovate, cordate with rounded auricles, acute to acuminate and mucronate, tomentose on both surfaces; petioles 5–11 cm. Inflorescence of lax, axillary cymes; peduncles 1–22 cm, tomentose or glabrescent; bracteoles linear or lanceolate, deciduous; secondary and tertiary peduncles 5.5 cm; pedicels 4–22 mm; sepals unequal, glabrous with scarious margins, outermost 6–9 mm, ovate to broadly elliptic, emarginate, inner 9–12 mm, suborbicular to obovate, obtuse, minutely mucronate; corolla c. 6 cm long, funnel-shaped, pink, glabrous, throat dark pink, limb 3–3.5 cm diam. Capsules 10–12 × 9–10 mm, ovoid, shortly rostrate, glabrous; seeds 6–8 mm long, glabrous apart from the long white marginal hairs.

#### Illustration.

Ferreira et al. (2013: 31).

#### Distribution.

Recorded from southern Brazil and neighbouring Argentina growing in deciduous forest margins and scrub.

**ARGENTINA. Misiones**: Iguazú, Wanda, *H.A. Keller & H.F. Romero* 13252 (CTES, OXF); Montecarlo, Col. Guatambo, *H.A. Keller* 4038 (CTES).

**BRAZIL. Rio Grande do Sul**: *P.P.A. Ferreira & J. Durigon* 705 (ICN, INPA); São Francisco de Paula, *A. Seynam* 10020 (MBM). **Santa Catarina**: Descanso. *R.M. Klein* 5119 (HBR) fide Ferreira et al. (2013: 32).

**Notes**. This species is distinguished by its small outer sepal and very lax, branched inflorescence and glabrous corolla.

The placement of this species is uncertain.

### 145. Ipomoea batatoides

Taxon classificationPlantaeSolanalesConvolvulaceae

Choisy, Mém. Soc. Phys. Genève 8(1): 58 [136]. 1838. (Choisy 1838: 58[136])

Ipomoea riedelii Meisn. in Martius et al., Fl. Brasil. 7: 265. 1869. (Meisner 1869: 265). Type. BRAZIL. Bahia, L. Riedels.n. (isotype NY00319218).
Ipomoea microsticta Hallier f., Bull. Herb. Boiss., ser. 1, 7: 411.1899. (Hallier 1899c: 411). Type. GUATEMALA. Escuintla, C. Seler & E. Seler 2427 (holotype B†, isotypes GH, L).
Ipomoea pseudomina K. Schum., Just's Bot. Jahresber. 26(1): 383.1900. (Schumann 1900: 383). Type. BOLIVIA. *O. Kuntze*s.n. (holotype B†, photo F).
Ipomoea glabriuscula House, Bot. Gaz. 43: 409. 1907. (House 1907b: 409). Type. GUATEMALA. *E.T. Heyde*s.n. (holotype US256072).
Ipomoea philipsonii O’Donell, Lilloa 26: 378. 1953. (O’Donell1953a: 378). Type. COLOMBIA. Meta, El Mico airstrip, *W.R. Philipson, J.M. Idrobo & A. Fernández* 1396 (holotype BM000953165, isotype COL).
Ipomoea teruae Ant. Molina & L.O. Williams, Fieldiana, Bot. 32(12): 196. 1970. (Williams 1970a: 196). Type. GUATEMALA Sololá: mountain slopes above Lake Atitlán, *L.O. Williams, A. Molina & T.P Williams* 25331 (holotype F0054901, isotype EAP).

#### Type.

BRAZIL. Bahia, *Blanchet* in Herb. Moric. (holotype G-DC, not seen, fragment F).

#### Description.

Twining perennial to 4 m, stems usually glabrous. Leaves petiolate, 3–11 × 2.5–8 cm, ovate, weakly cordate with rounded auricles, shortly acuminate to an acute apex, occasionally slightly undulate-denticulate or weakly 3-lobed, glabrous or, rarely, pubescent, lower surface paler, often dotted with glands or minute hair bases; petioles 2–7 cm, characteristically slender. Inflorescence of lax pedunculate, axillary cymes; peduncles 3–10 cm; secondary peduncles 1.5–3 cm; bracteoles filiform, 4 mm, caducous; pedicels 5–15 mm long; sepals subequal, coriaceous and somewhat convex, 6–8(–10) × 5 mm, broadly oblong, rounded, usually glabrous, margins narrowly scarious; corolla 4–8 cm long, funnel-shaped, inflated above a narrow basal tube, then gradually widened, pink or, less commonly, white, glabrous, limb 5–6 cm diam., unlobed. Capsules 8 × 6 mm, ellipsoid, glabrous, rostrate; seeds pilose on the margins with long white hairs.

#### Illustration.

Figures 83, 84A.

**Figure 83.**
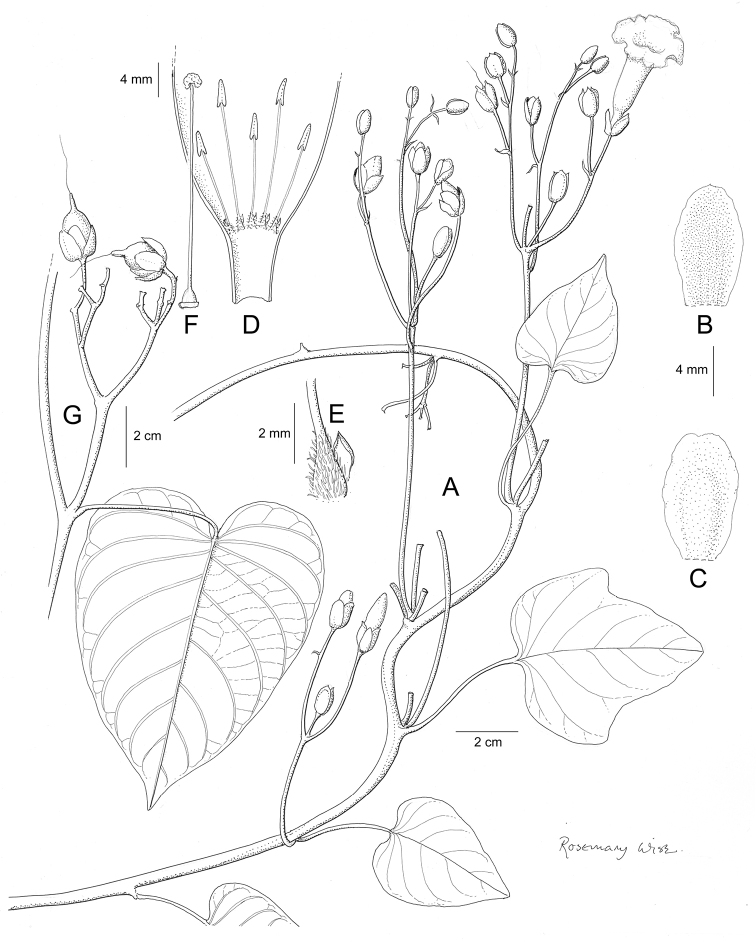
*Ipomoea batatoides*. **A** habit **B** outer sepal **C** inner sepal **D** corolla opened out to show stamens **E** base of stamen **F** ovary and style **G** fruiting inflorescence. Drawn by Rosemary Wise **A–F** from *Colque & Mendoza* 196; **G** from *Santos* 73.

**Figure 84.**
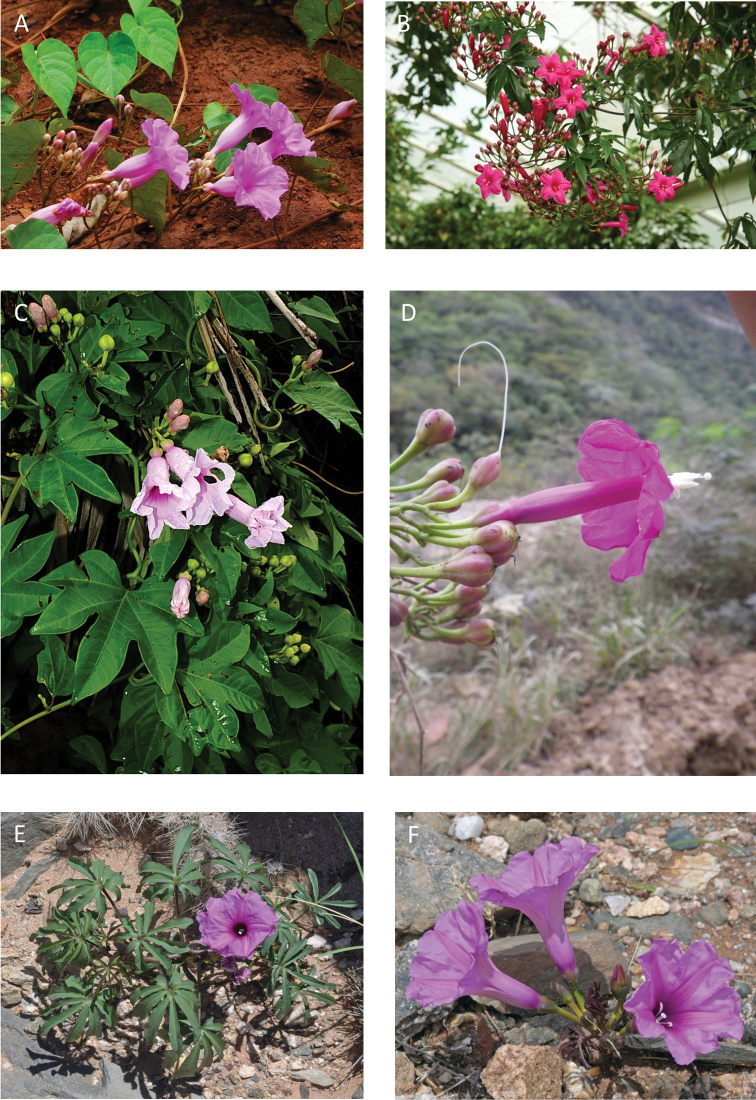
Photographs of *Ipomoea* species. **A***I. batatoides***B***I. horsfalliae***C***I. mauritiana***D***I. exserta***E, F***I. platensis*. **A** Guillaume Leotard **B** Royal Botanic Gardens, Kew **C** S.SANT/Parc Amazonien de Guyane **D** John Wood **E, F** Mario Giorgetti.

#### Distribution.

A widespread species of moist tropical forest at altitudes below 900 m from northern Bolivia and Brazil north to central Mexico but largely absent from the Amazonian lowlands.

**BRAZIL. Alagoas**: Coruripe, Faz. Capiatã, *R.D. Ribeiro et al.* 1022 (RB, OXF). **Amapá**: Macapá, Serra do Navio, *S. Mori et al.* 17687 (NY). **Bahía**: Paulo Afonso, *E.B. Miranda et al.* 853 (HUEFS, OXF); Rio São Francisco, Bom Jesus da Lapa, *R.M. Harley et al.* 21380 (CEPEC, K, NY). **Ceará**: *Schery* 423 (RB). **Goiás**: Campinaçu, Rio Tocantinzinho, *A.A. Santos et al.* 73 (CEN); Niquelândia, *R. Marquete et al.* 2523 (IBGE, MO). **Maranhão**: Monção, Ka’apor Reserve, *W.L. Balée & A. Gely* 879 (K). **Mato Grosso do Sul**: *E.P. Heringer* 853 (NY). **Pará**: Estrada da Fazenda Velha, *Da Silva* 177 (K); Santarém, *R. Spruce* s.n. (K). **Pernambuco**: *J. Falçao* 928 (RB). **Rondônia**: Porto Velho, *W.W. Thomas et al.* 5029 (K, MO, NY), ibid., 4927 (K, NY). **Sergipe**: *L.A. Gomes* 239 (ASE). **Tocantins**: 15 km S of Araguaina, *H.S. Irwin* 21216 (NY). Also Amazonas, Mato Grosso, Paraíba, Piauí, Rio Grande do Norte fide Flora do Brasil (2020).

**FRENCH GUIANA.***Feuillet et al.* 10217 (MO); *J.J. Granville & F. Crozier* 16385 (CAY, P); *G. Cremers* 6169 (P).

**SURINAM.** Lely Mts, *J.C. Lindeman et al.* 512 (K, MO, P); Volyz Mts, *A. Pulle* 284 (K).

**GUYANA.***Stoffers et al.* 458 (MO).

**BOLIVIA. Cochabamba**: Carrasco, Puerto Cotagaita, *O. Colque & L. Mendoza* 196 (OXF, MO, USZ). **La Paz**: Caranavi, *J.R.I. Wood & T. Daniel* 18399 (HSB, K, LPB); Sud Yungas, *J.R.I. Wood, et al.* 20623 (LPB). **Santa Cruz**: Velasco, Cerro Pelao, *T. Killeen & J. Wellens* 6312 (ARIZ, LPB, MO, USZ); Florida-La Mechita, *J.R.I. Wood et al.* 26095 (K, LPB, UB, USZ).

**PERU. Cusco**: La Convención, Echarate, *L. Valenzuela et al.* 9432 (MO, OXF**Loreto**: Río Pastaza, Andoas, *F. Ayala* 2264 (MO, OXF). **Puno**: *P. Nuñez & C. Muñoz* 5152 (MO, USM). **San Martín**: fide McPherson (1993).

**ECUADOR. Napo**: *Lugo* 3434 (QCA); Jatun Sacha, *C.E. Cerón* 6704 (MO). **Orellana**: Chiruisla-Río Tiputini, *J. Jaramillo et al.* 24699 (QCA).

**COLOMBIA. Antioquia**: Urubá, *L. Uribe* 1469 (COL). **Cesar**: Serranía de Perijá, *O. Rivera Díaz* 2919 (COL). **Chocó**: Yuto, *A. Gentry & E. Renteria* 23787 (COL, MO). **Magdalena**: Tucurinca, *R. Romero* 572 (COL); Santa Marta, *H.H. Smith* 1568 (BM, COL, MO, P, S). **Meta**: type of *Ipomoea philipsonii*. **Putumayo**: Mocoa, Vereda Alto campucana, *D. Giraldo* 2018 (COL, MO).

**VENEZUELA. Amazonas**: *R Liesner et al.* 18223 (MO). **Lara**: Santa Rosa, *A.H.G.Alston* 6336 (BM, NY, S). **Yaracuy**: *H. Pittier 13075* (MO, US, VEN). Also Aragua. Carabobo and Falcón fide Austin (1982b).

**PANAMA.** Gorgona-Mamel, *H. Pittier* 2274 (BM, US).

**COSTA RICA.***A. Tonduz* 4803 (BM); San José-Puntarenas, *P. Wilkin* 471 (BM); Limón, Pococí, *F. Araya* 184 (BM, MO); San José, El General, *A.F. Skutch* 2225 (K, S); Puntarenas, Golfito, *M. Segura & F. Quesada* 224 (BM, K, MO); Alajuela, San Ramón, *B. Hammel* 19360 (MO).

**NICARAGUA.** Chinandega, *J.C. Sandino* 3808 (BM, MO); Masaya, *D. Weberbauer* 3068 (BM, MO).

**HONDURAS.** Morazán, Santa Ana, *A. Molina et al.* 31172 (MO).

**EL SALVADOR.** Quezaltepeque, *M. Calderón & W.G. Berendsohn* JBL00559 (MO); Libertad, *R.A. Caballo et al.* 04221 (BM).

**GUATEMALA.***Bernoulli & Cario* 1882 (K); *J.J. Castillo* 1964 (F, S); San Marcos, *J.D. Dwyer* 15307 (MO).

**MEXICO. Chiapas**: Río de la Venta, *D.E. Breedlove* 27392 (MO); *D.E. Breedlove & R.F. Thorne* 30517 (MICH); La Correa, *E. Langlassé* 396 (K). **Guerrero**: Montes de Oca. Vallecitos *G.B. Hinton* 11364 (K). **Jalisco**: La Huerta, *E.J. Lott* 3890 (MO); ibid., *M.G. Ayala* 217 (MEXU). **Michoacán**: Coahuayana, *E. Carranza & I. Silva* 6810 (IEB, MEXU), 7104 (IEB). **Oaxaca**: *K. Velasco-G & G. Juárez* 80 (IEB); Santa Maria Chilchotla, *X. Munn-Estrada et al.* 1311 (MEXU). **Puebla**: Las Margaritas, *B. Gómez* 850 (IEB, K, MEXU). **Querétaro**: Landa de Matamoros, *E. Pérez & E. Carranza* 3759 (IEB); Jalpán, *B. Servín* 408 (IEB). **Veracruz**: San Andrés Tuxtla, *G. Martínez Calderón* 1776 (MEXU); ibid., *S. Sinaca Colín et al.* 978 (IEB); Las Tuxlas, *G. Ibarra* 2079 (MO).

#### Notes.

Plants are usually glabrous and the leaves often dotted beneath with hair bases/glands. Occasional densely pubescent plants occur such as *Ayala* 2264 from Peru and *Breedlove* 27392 from Mexico. White-flowered forms occur in the Yungas of La Paz, Peru, central Mexico and Venezuela and can be easily mistaken for *Ipomoea reticulata* but the calyx and corolla are both larger, the sepals coriaceous with only very narrow scarious margins and the inflorescence clearly of axillary cymes, never subracemose. White-flowered specimens from Mexico are particularly difficult to assign to species, especially when leafless. We have generally treated these as *I. pseudoracemosa* if the peduncles are short and the plant is leafless at anthesis. However, the type of *I. pseudoracemosa* is leafless and it is difficult to characterise that species or distinguish it from *I. batatoides* in the absence of better material and field studies.

Four South American specimens are distinctive because of their large sepals (up to 15 × 10 mm). Three are from Brazil: *R. Ribeiro et al.* 1022 (RB, OXF) from Alagoas, *Miranda et al.* 853 (HUEFS, OXF), from Bahia and *A.A. Santos et al.* 73 (CEN) from Goiás, and one (*J. Schunke* 2590 (F, MO) from Huánuco in Peru. They merit further study and may represent a distinct taxon.

### 146. Ipomoea volcanensis

Taxon classificationPlantaeSolanalesConvolvulaceae

O’Donell, Lilloa 26: 398. 1953. (O’Donell 1953a: 398)

#### Type.

ARGENTINA. Jujuy, [Dept. Tumbaya], Volcán, Toma de la Laguna, 2200 m, *R. Schreiter* 2619 (holotype LIL001290).

#### Description.

Twining perennial herb with tuberous roots; stems glabrous. Leaves petiolate, 6.5–8.5 × 5–7 cm, ovate-deltoid (often shallowly 3-lobed), subtruncate with rounded auricles, long-acuminate, mucronulate, margin somewhat undulate, glabrous on both surfaces; petioles 3–8 cm. Inflorescence of 1–5-flowered, pedunculate axillary cymes; peduncles 4–14 cm, relatively stout; bracteoles fugacious; secondary peduncles 1–2 cm; pedicels 20–35 mm; sepals subequal, convex, coriaceous, obtuse, glabrous, outer 6–8 × 4–6 mm, inner 8–9 × 6 mm, slightly larger, suborbicular; corolla 6–7 cm long, deep pink, glabrous, funnel-shaped, limb 3–3.5 cm diam., shallowly lobed. Capsules and seeds not seen.

#### Illustration.

**Figure 85.**
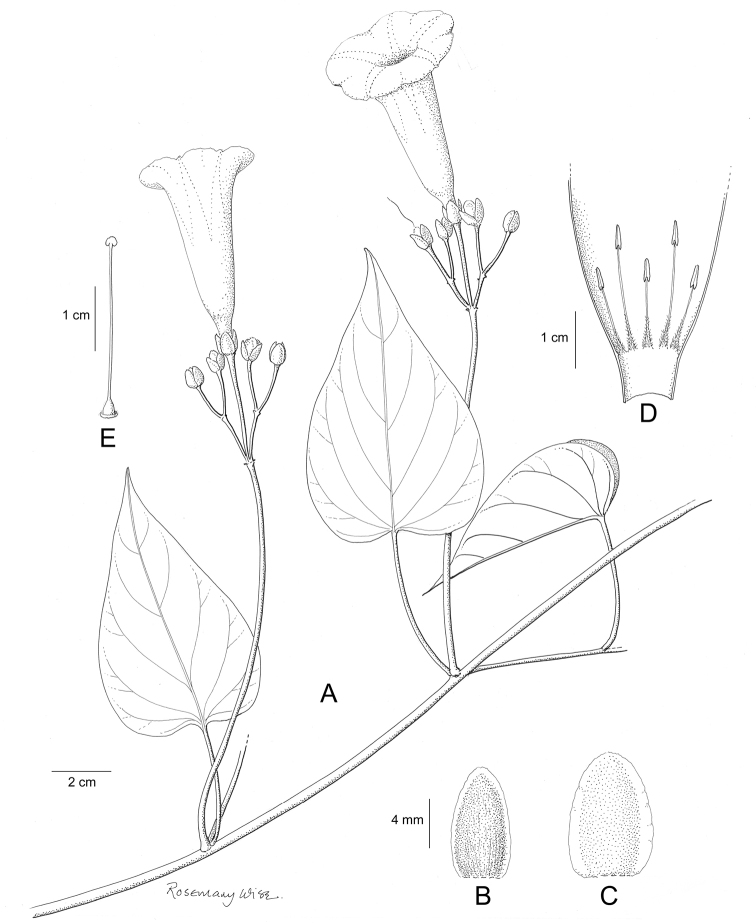
*Ipomoea volcanensis*. **A** habit **B** outer sepal **C** inner sepal **D** corolla opened out to show stamens **E** ovary and style. Drawn by Rosemary Wise from *T. Meyer* 16958.

Figure 85; O’Donell (1959b: 252).

#### Distribution.

In moist Tucuman-Bolivian forest in Andean Argentina and Bolivia at around 1500–2100 m.

**ARGENTINA. Jujuy**: Belgrano, *O. Morrone et al.* 2251 (SI, MO); Yala, *A. Rotman* 1010 (CTES); *T. Meyer* 16958 p.p. (US, LIL); Tumbaya, Volcán, *S. Venturi* 4951 (LIL, MO, US), *Cildella et al.* 517 (CTES); Vallegrande, *Fabris* 3554 (CTES). **Salta**: Rosario de Lerma, *L.J. Novara* 7605 (G, S).

**BOLIVIA. Tarija**: Entre Ríos, on road to Palos Blancos, *J.R.I. Wood et al.* 28059 (LPB, USZ).

#### Note.

Very similar to *Ipomoea austrobrasiliensis* differing in the slightly longer corolla, the subdeltoid, basally subtruncate, often shallowly lobed, consistently smaller leaves. It is also similar to the *I. batatoides*, which differs in the smaller, acuminate, ovate leaves and the fewer-flowered inflorescence. It also lacks the distinctive punctate abaxial leaf surface commonly found in that species.

### 147. Ipomoea austrobrasiliensis

Taxon classificationPlantaeSolanalesConvolvulaceae

J.R.I. Wood & Scotland, Kew Bull. 72(9): 2. 2017. (Wood and Scotland 2017a: 2)

Ipomoea batatoides var. angulata Choisy in A.P. de Candolle, Prodr. 9: 376. 1845. (Choisy 1845: 376). Type. BRAZIL. São Paulo, Martius s.n. (lectotype M0184900).

#### Type.

BRAZIL. Paraná, Mun. Paranagua, Pico Torto, 15 Jan. 1970, *G. Hatschbach 23340* (holotype MBM12820, isotypes F, K, MO).

#### Description.

Vigorous climbing perennial of unknown height, glabrous in all parts. Leaves petiolate, generally large, 10–22 × 9–16 cm, ovate, cordate with rounded auricles, acute to shortly acuminate, margin slightly undulate to subsinuate, sometimes with a distinct tooth above the auricle, glabrous on both surfaces but abaxially paler with prominent venation, the main veins with distinct pale margins; petioles 8–16 cm. Inflorescence of lax, axillary, pedunculate cymes; peduncles 3–10 cm; bracteoles 1–3 mm, linear-oblong, margins scarious, caducous; secondary peduncles 2–3.5 cm; tertiary peduncles (if present) c. 1.5 cm; pedicels 10–18 mm, thickened upwards; sepals subequal, coriaceous, convex, rounded, outer 9–12 × 6–7 mm, obovate; inner slightly wider, broadly elliptic with scarious margins; corolla 4.5–6 cm long, narrowly funnel-shaped, tube pale, limb deep pink, somewhat lobed, c. 4 cm diam. Capsules and seeds not seen.

#### Illustration.

Figure 86.

**Figure 86.**
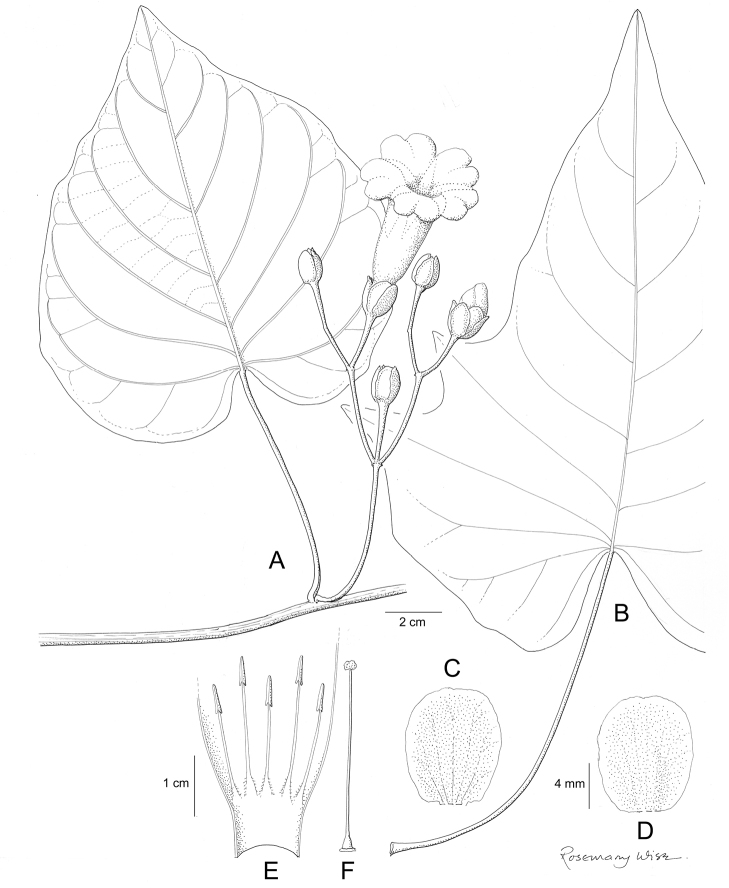
*Ipomoea austrobrasiliensis*. **A** habit **B** leaf **C** outer sepal **D** inner sepal **E** corolla opened out to show stamens **F** ovary and style. Drawn by Rosemary Wise **A, C–F** from *Reitz & Klein* 6615; **B** from *Reitz & Klein* 4102.

#### Distribution.

Endemic to moist Atlantic forest below 300 m in Southern Brazil.

**BRAZIL. Paraná**: Jacarehy, *P. Dusen* 11400 (K, S, MICH, GH); Mun. Guaratuba, Serra do Araraquara, *G. Hatschbach* 12504 (MGM); Mun. Paranaqua, Picadão Cambará–Col. Limeira, *G. Hatschbach* 18597 (MBM). **Santa Catarina**: Pinhal da Companhia, *R. Reitz & Klein* 4102 (US); São Francisco do Sul, *R. Reitz & Klein* 6615 (US). **São Paulo**: type of Ipomoea batatoides var. angulata.

#### Notes.

Distinct from *Ipomoea goyazensis* because of the branched, well-developed cymes with long peduncles, large leaves and somewhat campanulate corolla. From *I. batatoides* it is distinguished by the longer sepals and larger leaves which are undulate and often somewhat angled, almost with a lateral tooth, hence Choisy’s varietal name of *angulata*.

This has been identified as *Ipomoea goyazensis*, perhaps because Choisy treated *I. goyazensis* as a synonym of I. batatoides var. angulata. *Ipomoea goyazensis* is a quite different cerrado species whereas *I. austrobrasiliensis* is a plant of the Atlantic forests of southern Brazil and has not been found in the cerrados of Goiás or further north in Brazil. Plants called *Ipomoea goyazensis* (Simão-Bianchini et al. 2016) are a mixture of *I. austrobrasiliensis* and glabrous forms of *I. goyazensis*.

### 148. Ipomoea goyazensis

Taxon classificationPlantaeSolanalesConvolvulaceae

Gardner, Hooker, Icones 5: t. 479. 1842. (Gardner 1842b: t. 479)

Ipomoea decora Meisn. in Martius et al., Fl. Brasil. 7: 272. 1869. (Meisner 1869: 272). Type. BRAZIL. Goiás, J.B. Pohl 1760 (isotypes K000612854, OXF, W0062252, W0062251, W0062250).

#### Type.

BRAZIL. Goiás, Serra de Santa Brida, *G. Gardner* (lectotype Plate 479 in Hook., Icones 5 (1842b), designated by Wood and Scotland 2017a: 2; epitype *Gardner* s.n., (BM001122231), designated here.

#### Description.

Twining perennial liana to 6 m; stems rather thin but slightly woody, usually completely glabrous but sometimes appressed pilose or pubescent. Leaves petiolate, 4–12×3–10 cm, ovate-deltoid, obtuse and mucronate, both surfaces glabrous or pubescent, adaxially dark green, abaxially very pale with prominent venation; petiole rather short, 1.5–3.5 cm. Inflorescence of subsessile, clustered cymes; peduncles 1–6 mm, glabrous; bracteoles scale-like, caducous; pedicels 0–7 mm, glabrous; sepals subequal, 6–9(–11) mm, elliptic, obtuse to rounded, convex, coriaceous, glabrous, whitish-green when fresh, inner sepals with scarious margins; corolla 5–6 cm long, funnel-shaped, gradually widened from base, glabrous, tube white, limb deep pink, weakly lobed, 2–2.5 cm diam. Capsules (immature), subglobose, glabrous.

#### Illustration.

Figure 87.

**Figure 87.**
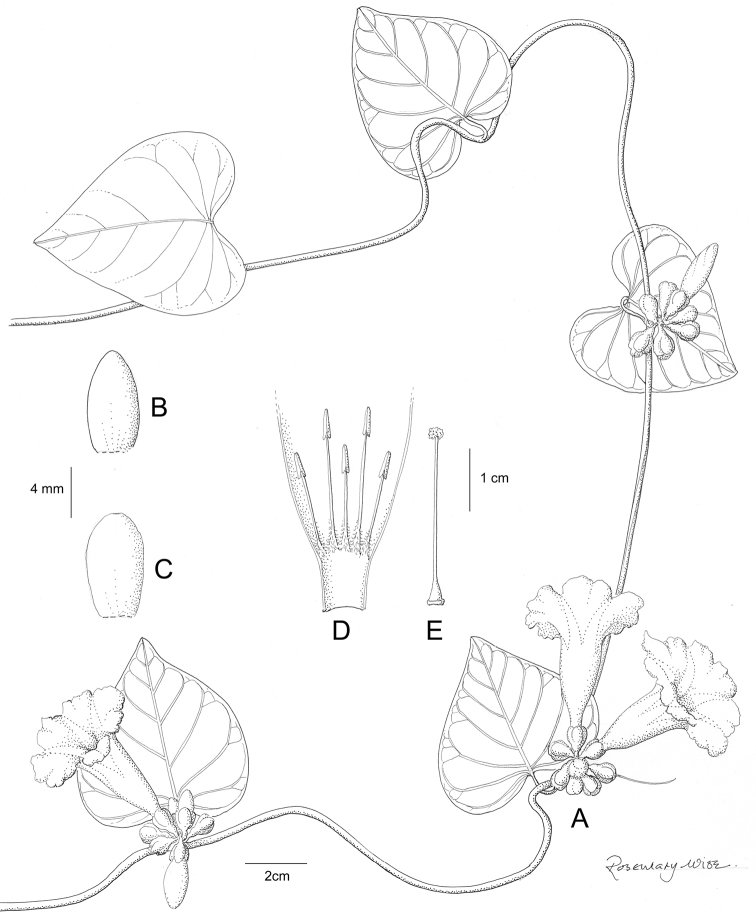
*Ipomoea goyazensis*. **A** habit **B** outer sepal **C** inner sepal **D** corolla opened out to show stamens **E** ovary and style. Drawn by Rosemary Wise from *Wood et al.* 27806.

#### Distribution.

A characteristic species of the cerrado biome in Brazil and Bolivia; apparently not very common and absent from southern Brazil.

**BRAZIL.** Sine loc., *W.J. Burchell* 6656 (K); 6702 (K). **Goiás**: *A. Krapovickas et al*. 33131 (CTES); Colinas do Sul, *D. Alvarenga et al.* 788 (CEN, MO); Hidrolândia, *J.F.B. Pastore* 3078 (HUEFS). **Maranhâo**: Mun. Barra do Corda, *Schatz et al.* 793 (K); *G. Gardner* 6070 (K, BM). **Mato Grosso**: Mun. Novo Mundo, *W. D. Sasaki et al.* 1862 (K). **Minas Gerais**: Ituiutaba, *A. Macedo* 1668 (BM, MO). **Pará**: Tucuruí, *T. Plowman et al.* 9706 (MG, MO); Marabá, *da Silva* 1786 (MG, MO). **Tocantins**: Parque Nacional do Araguaia, *Silva et al*. 3995 (IBGE, MO, RB); Darcinopolis, *G. Pereira-Silva* 12956 (CEN).

**B**OLIVIA. **Santa Cruz**: Velasco, P.N. Noel Kempff Mercado, *T.J. Killeen et al.* 5399 (ARIZ, MO); Santa Rosa de la Roca, *J.R.I. Wood et al.* 27806 (OXF, K, LPB, USZ).

#### Notes.

When Wood and Scotland designated the lectotype for *Ipomoea goyazensis*, no specimen could be found in any of the herbaria where Gardner’s specimens were deposited. Subsequently a sheet of the original material collected by Gardner was found at BM (BM001122231), which is here designated as epitype.

A very distinctive species in the field because of its discolourous leaves, very short peduncles and funnel-shaped corolla with a white tube and pink limb. Herbarium specimens are usually easily identified by the subsessile, clustered flowers with coriaceous, convex sepals.

This species is quite variable in indumentum and glabrous (as in the type of *I. goyazensis*), pubescent (as in the type of *I. decora*) or pilose forms (*Pastore* 3078) occur.

### 149. Ipomoea schulziana

Taxon classificationPlantaeSolanalesConvolvulaceae

O’Donell, Lilloa 14: 186. 1948. (O’Donell 1948a: 186)

#### Type.

ARGENTINA. Salta, Oran, San Pedrito, senda a Astillero, *Schulz* 5483 (holotype LIL107492).

#### Description.

Robust twining liana reaching at least 6 m in height, commonly leafless when flowering, roots tuberous, stems glabrous. Leaves petiolate, mostly 3–8 × 1.5–4 cm, oblong-ovate, acute and mucronate, basally broadly cordate to truncate, margin slightly undulate, glabrous, adaxially green, abaxially somewhat glaucous and with prominent veins; petioles 1–3.5 cm. Inflorescence of axillary, pedunculate simple or compound cymes often developing on axillary branchlets, sometimes very dense and floriferous or panicle-like; peduncles 0.5–5 cm long; bracteoles caducous, not seen; secondary peduncles 0.5–2.5 cm; pedicels 3–8 mm, thickened upwards; sepals slightly unequal, coriaceous, glabrous, outer sepals 5–6 mm, convex, elliptic, obtuse with scarious margins, the inner 7–8 mm, suborbicular, rounded; corolla 5–6.5 cm long, pink, glabrous, funnel-shaped, limb c. 3 cm diam., unlobed. Capsules glabrous; seeds pilose on the angles.

#### Illustration.

**Figure 88.**
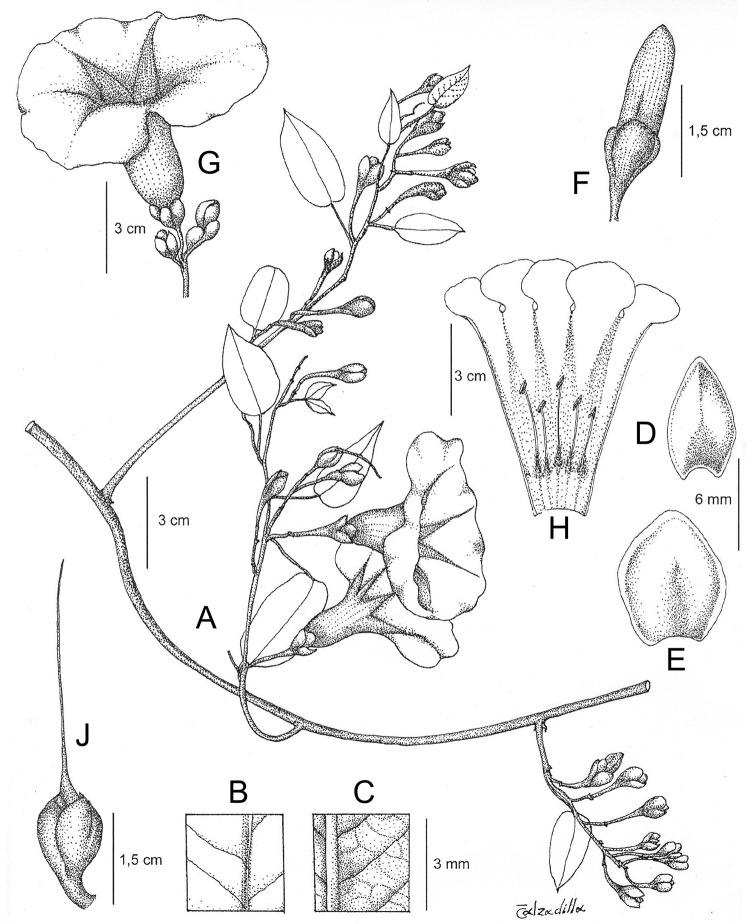
*Ipomoea schulziana*. **A** habit **B** adaxial leaf surface **C** abaxial leaf surface **D** outer sepal **E** inner sepal **F** bud **G** corolla and calyx **H** corolla opened out to show stamens **J** ovary and style. Drawn by Eliana Calzadilla **A–F, H–J** from *Wood et al.* 25053; **G** from *Wood et al.* 27705 and photo.

O’Donell (1959b: 236). Figure 88.

#### Distribution.

A characteristic species of open woodland in the Inter-Andean dry valleys and Bosque Serrano Chaqueño between (200–)850 and 2100 m in southern Bolivia and extreme northern Argentina.

**ARGENTINA. Jujuy**: Laguna de la Brea, *R.E. Fries* 436 (S). **Salta**: type of *Ipomoea schulziana*.

**BOLIVIA. Chuquisaca**: Boeto, below Nuevo Mundo, *M. Kessler* 5198 (LPB); Oropeza, Sucre-Surima, *J.R.I. Wood & J. Gutiérrez* 20232 (BOLV, HSB, K, LPB); Sud Cinti, Las Abras, *R. Lozano et al.* 1375A (MO); Tomina, Llantoj, *J. Gutiérrez et al.* 996 (HSB, NY, MO); Zudañez, ANMI El Palmar, *J. Gutiérrez et al.* 2806 (HSB). **Cochabamba**: Campero, Río Mizque, north of Aiquile, *J.R.I. Wood* 9466 (K, BOLV, LPB). **Santa Cruz**: Caballero, Pulquina-Saipina, *J.R.I. Wood et al.* 27705 (OXF, K, LPB, USZ); Chiquitos, Tucavaca valley, J.R.I. Wood et al. 29394 (LPB, USZ); Cordillera, Camiri, *W.M.A. Brooke* 5546 (BM, NY, F); camino San José to Salinas, *A. Fuentes & G. Navarro* 2012 (USZ); Florida, Mairana, *M. Nee* 49260 (NY, USZ); Vallegrande, Pampa Negra to Naranjos, *G.A. Parada & V. Rojas* 2652 (OXF, MO, USZ). **Tarija**: Arce, *S.G. Beck et al.* 31416 (LPB); Gran Chaco, ANMI Aguaraque, *A. Lliully* 980 (HSB).

#### Note.

Very similar vegetatively to *Ipomoea suburceolata* but easily distinguished by the funnel-shaped corolla with a well-developed entire limb. It has often been misidentified as *I. batatoides* but differs in the leaf shape and inflorescence structure, the flowers often being borne on leafy side shoots.

### 150. Ipomoea suburceolata

Taxon classificationPlantaeSolanalesConvolvulaceae

O’Donell, Lilloa 26: 394. 1953. (O’Donell 1953a: 394)

#### Type.

BOLIVIA. “Caupolican”, fide note on sheet at Kew, *R. Pearce 779* (holotype K).

#### Description.

Liana, glabrous in all parts, stems pale brown, woody. Leaves 4–9 × 4–8 cm, ovate, acute, base cordate to subtruncate, glabrous, abaxially paler, gland-dotted with pale whitish glands. Inflorescence of small cymes, often aggregated into a terminal panicle-like inflorescence; bracts resembling small leaves; peduncles 1.3–2 cm; secondary peduncles 10–15 mm; bracteoles 2–3 mm, oblong-ovate, obtuse, deciduous; pedicels 5–10 mm; sepals reddish, slightly unequal, outer 6–7 mm, ovate, obtuse, inner 8–9 mm, narrowly obovate with scarious margin; corolla 3.5–4 cm long, tubular but somewhat inflated in the middle to 10–12 mm in width, fuchsia-red, limb 5–lobed, 4–5 mm diam., dark red; stamens shortly exserted. Capsules 10–12 × 5 mm, ovoid, style persistent; seeds oblong in outline, c. 5 × 2 mm, long-pilose.

#### Illustration.

Wood et al. (2015: 79).

#### Distribution.

Bolivian endemic restricted to dry forest between 750 and 1200 m in the inter-Andean valleys north of Apolo in the Madidi National Park.

**BOLIVIA. La Paz**: Prov. Tamayo, Río Machariapo, *A. Gentry* 71078 (MO); Hac. Ubitó, *M. Kessler* 4007 (LPB); Asariamas, *L. Cayola* 1746 (LPB); *A. Fuentes* 18492 (LPB, MO).

#### Note.

Very similar to *Ipomoea schulziana* in habit, leaves and tendency of inflorescence to become paniculate but distinguished by the suburceolate corollas of a distinct fuchsia colour, the limb reduced to five very short lobes.

### 151. Ipomoea pintoi

Taxon classificationPlantaeSolanalesConvolvulaceae

O’Donell, Lilloa 26: 380. 1953. (O’Donell 1953a: 380)

#### Type.

BRAZIL. Bahia, Mun. Muritiba, Faz. Velo-Vale, *G.C.P. Pinto* 5-1950 (holotype Herb. Inst. Agron. De Léste, isotype (fragment) LIL452194).

#### Description.

Woody climber to 2 m; stems glabrous, grey, the petiole base persistent and subaculeate, possibly facilitating climbing. Leaves petiolate, 3–5 × 0.7–2.5 cm, oblong-elliptic to oblong-obovate, acute and shortly mucronate, basally cuneate, glabrous, abaxially paler and somewhat glaucous; petioles 5–10 mm, often dark red, glabrous. Inflorescence of many-flowered complex, often compact axillary cymes usually forming a subcorymbose inflorescence on short branchlets; peduncles stiff, woody, often curved, 0.9–4 cm; bracteoles caducous; secondary peduncles 3–9 mm; pedicels 4–13 mm; sepals coriaceous, convex, scarious-margined, glabrous, subequal, outer 5–6 × 3–4 mm, elliptic, obtuse, mucronate, inner 8 × 5 mm, obovate, rounded; corolla 4–6 cm long, funnel-shaped, deep pink, distinctly but shortly lobed with lobes up to 5 mm long; limb 3.5–4 cm diam.; stamens held at mouth. Capsules 12 × 6–7 cm, ovoid, glabrous, shortly rostrate; seeds long-pilose.

#### Illustration.

Figure 5F.

#### Distribution.

Characteristic of Caatinga thorn scrub in NE Brazil.

**BRAZIL. Bahia**: near Morro do Chapaeu, *L. Queiroz et al.* 15956 (HUEFS); Itatim Rio Milagres, *E. Melo et al.* 11190 (HUEFS, OXF); Rodovia Juazeiro-Senhor do Bonfim, km 100, *L Coradin et al.* 5999, (CEN, K). **Pernambuco**: Buíque, Faz. Laranjeiras, *L.S. Figueirêdo & Andrade* 108 (IPA). **Sergipe**: Poço Verde, *G. Viana* 285 (ASE). Also Alagoas fide Flora do Brasil (2020).

#### Notes.

A woody climber with glabrous stems somewhat similar to *Ipomoea schulziana* but differing most obviously in the cuneate-based leaves.

This species has sometimes been confused in herbaria with *Ipomoea ana-mariae*, from which it is distinguished by its funnel-shaped corolla. Fruiting specimens are therefore difficult to determine.

### 152. Ipomoea serrana

Taxon classificationPlantaeSolanalesConvolvulaceae

Sim.-Bianch. & L.V. Vasconc., Brittonia 68: 143 2016. (Vasconcelas et al. 2016: 143)

#### Type.

BRAZIL. Bahia, Andaraí, Serra das Tres Barras, *L.V. Vasconcelas & J.J. Oliveira* 673 (holotype HUEFS, isotypes NY, SP).

#### Description.

Liana with tuberous suborbicular rootstock, all vegetative parts glabrous. Leaves petiolate, 3.5–10.5 × 3–5 cm, obovate, apex rounded to emarginate, base attenuate, venation actinodromous with secondary main veins; petioles 0.7–1.8 cm. Inflorescence of axillary, often compounded, pedunculate cymes; peduncles 1–3 cm; bracteoles ovate, c. 2 mm long, caducous; secondary peduncles 1–2 cm; pedicels 1–2.5 cm; sepals subequal, 7–8 × 5 mm, coriaceous, ovate, rounded, the inner with scarious margins; corolla 4–6.5 cm long, funnel-shaped, deep pink, glabrous, limb weakly lobed. Capsules ellipsoid, 10–12 × 7–10 mm, glabrous; seeds 6 mm long, pilose on the margins.

#### Illustration.

Vasconcelas et al. (2016: 143–144).

#### Distribution.

Endemic to the eastern part of the Chapada Diamantina between 450 and 1200 m in Bahia.

**BRAZIL. Bahia**: Lençois, Serra Barro Branco, *M. I. Cartazo* 03 (ALCB, HUEFS).

#### Note.

Differs from *I. pintoi* in having leaves with actinodromous venation (one main vein and two secondary veins) with 4 or 5 pairs of lateral veins (vs. brochidodromous in *I. pintoi* with 9–12 pairs of veins). The leaves are also rounded to emarginate, not acute to obtuse at the apex.

### 153. Ipomoea ana-mariae

Taxon classificationPlantaeSolanalesConvolvulaceae

L.V. Vasconc. & Sim.-Bianch., Brittonia 68: 142. 2016. (Vasconcelas et al. 2016: 142)

#### Type.

BRAZIL. Bahia, Ibícoara, *L.V. Vasconcelas, E. Melo, F. França & P.H.S*.

*Mercês* 598 (holotype HUEFS, isotypes NY, SP).

#### Description.

Liana with tuberous roots, all vegetative parts glabrous. Leaves petiolate, 3–6 × 1–2.3 cm, lanceolate to ovate, attenuate to a mucronate apex, base cuneate; petioles 0.7–1.8 cm. Inflorescence of compound, axillary cymes; peduncles 1.5–3 cm; bracteoles c. 1 mm, ovate, caucous; seconday peduncles 1–3 cm; pedicels 1–1.5 cm; sepals slightly unequal, 4.5–6 × 3–5 mm, ovate, convex, rounded, the inner slightly larger and with scarious margins; corolla 3–3.5 cm long, hypocrateriform to suburceolate, pink, glabrous, the limb entire, 2–3 mm long. Capsules ovoid, glabrous, 11–12 × 7 mm; seeds 5–6 mm long, pubescent, the hairs up to 12 mm long, more dense on the angles.

#### Illustration.

**Figure 89.**
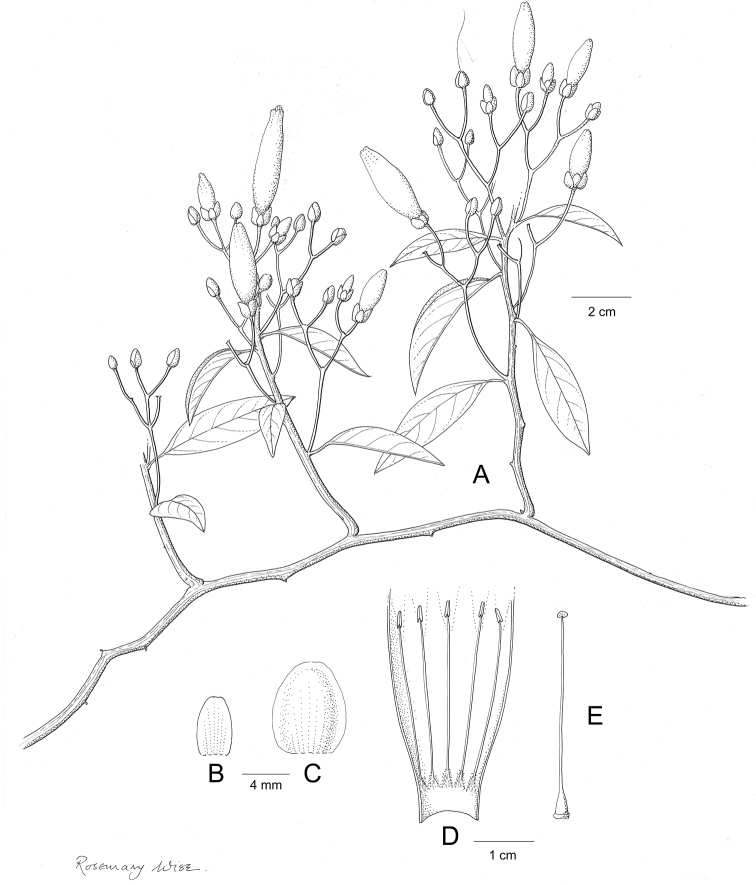
*Ipomoea ana-mariae*. **A** habit **B** outer sepal **C** inner sepal **D** corolla opened out to show stamens **E** ovary and style. Drawn by Rosemary Wise from *W. Ganev 3275*.

Figure 89; Vasconcelas et al. (2016: 143–144).

#### Distribution.

Apparently endemic to Caatinga and Mata Atlântica in Bahia.

**BRAZIL. Bahia**: Jussiape, ca. 14 km antes de Jussiape, na estrada de Capão da Volta, *R.M. Harley & A.M. Giulietti 53949* (HUEFS, SP); Abaíra, *W. Ganev 3275* (HUEFS, HST); Boa Nova, P.N. de Boa Nova, *G.S. Brandão & G.S. Silva* 335 (PEUFR); Poções, Morrinhos, *M.M. Saavedra* 1007 (RB).

#### Note.

Differs from *Ipomoea pintoi* only in the suburceolate corolla with exserted stamens. It is perhaps more widely distributed than is suggested here because of confusion with *Ipomoea pintoi*.

### 154. Ipomoea longistaminea

Taxon classificationPlantaeSolanalesConvolvulaceae

O’Donell, Lilloa 23: 488. 1950. (O’Donell 1950b: 488)

#### Type.

BRAZIL. Bahia, Barrhiña, 7–8 June 1915, *J.N. Rose & P.G. Russell* 19784 (holotype US00111414, isotype NY).

#### Description.

Liana to 3 m; stems woody, white-canescent, peeling off to show glabrous pale brown under-bark. Leaves usually absent at anthesis, petiolate, 2–6 × 1.5–3.5 cm, ovate, base subtruncate with glands, apex often retuse, densely white-canescent on both surfaces; petioles 1.5–2.5 cm, white-canescent. Inflorescence of shortly pedunculate corymbose clusters; peduncles 0.4–4 cm, white-canescent, appearing branchlet-like; secondary peduncles 0.5–1 cm, pubescent; pedicels 6–18 mm, thinly pubescent, glabrescent; sepals subequal, coriaceous, convex, glabrous, outer 7–8 × 4 mm, elliptic, obtuse, inner obovate, 5 mm wide, rounded, margins scarious; corolla 3.7–4 cm long, suburceolate, deep pink, glabrous, limb reduced to short teeth, 3–4 mm long; stamens shortly exserted. Capsules ovoid 15 × 7 mm; seeds long white-pilose.

#### Illustration.

Figures 4H, 8J, 90.

**Figure 90.**
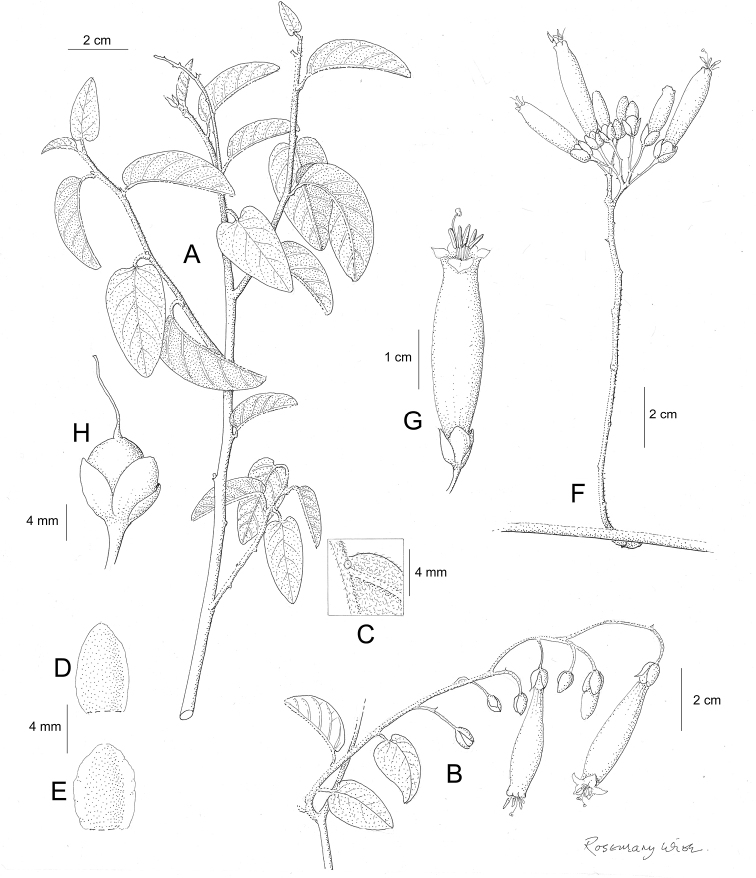
*Ipomoea longistaminea*. **A** habit **B** fertile branch **C** abaxial leaf surface **D** outer sepal **E** inner sepal **F** leafless inflorescence **G** corolla **H** capsule. Drawn by Rosemary Wise **A–C** from *Guilietti* 74866; **D–F** from *Coradin et al.* 5981; **G, H** from *Pastore* 2678.

#### Distribution.

Endemic to NE Brazil, principally Bahia State growing in caatinga.

**BRAZIL. Bahia**: Mucugé, *R.M. Harley & A.M. Guilietti* 54044 (HUEFS, SP); Mun. Abaira, *W. Ganev* 3378 (HUEFS, K); Sobradinho, Rodavia Sobradinho-Santa Fe, *L. Coradin et al.* 5981 (CEN, K, MO); Rio de Contas, *A.M. Guilietti* et al. 2430 (HUEFS, K). **Minas Gerais**: Jaiba, Mocambinho Estrada para Jaiba, km 11, *J.F.B. Pastore* 2678 (HUEFS, OXF). **Pernambuco**: Afrânio, *I.D. Pequeno* 3 (HVASF).

#### Note.

A very distinctive species because of the suburceolate, tubular corolla with exserted stamens combined with the white-tomentose stem and leaf indumentum. Leaves are mostly absent at anthesis.

### 155. Ipomoea franciscana

Taxon classificationPlantaeSolanalesConvolvulaceae

Choisy in A.P. de Candolle, Prodr. 9: 357. 1845. (Choisy 1845: 357)

#### Type.

BRAZIL. [Bahia], Rio São Francisco, *Martius* s.n. (holotype M0184871).

#### Description.

Erect subshrub c. 2 m high, stems stout, woody, reddish, glabrous. Leaves shortly petiolate, 3–5(–7) × 0.7–1.5(–2.5) cm, oblong, oblanceolate, rounded to retuse, cuneate at base, glabrous; petioles 4–7 mm. Inflorescence of few-flowered cymes from the upper leaf axils, often reduced to single flowers; peduncles 4–13 mm; bracteoles caducous, not seen; pedicels 7–15 mm, usually longer than peduncles; sepals slightly unequal, elliptic, convex, rigid, outer 5–8 × 4–5 mm, obtuse, inner 8–11 × 6 mm, obtuse, margin very narrowly scarious; corolla 4.5–5.5 cm long, white to pale lilac, glabrous, narrowly funnel-shaped, limb c. 3 cm diam.; stamens held at mouth. Capsules ellipsoid, 13–14 × 7 mm, glabrous; seeds c. 5 mm long, dark brown with long brownish marginal hairs 10 mm in length.

#### Distribution.

Endemic to Brazil growing around Maracas on granite outcrops at 850–900 m in the Rio São Francisco valley.

**BRAZIL. Bahia**: Mun. Maracás, *S.A. Mori et al*. 10009 (CEEC, NY, MO); *L. Queiroz & Fraga* 3288 (HUEFS); ibid., Faz. Cana Brava, *E.B. dos Santos & S. Mayo* 295 (CEPEC, SP); ibid., *R.M. Harley & A.M. Guilletti* 28237 (K).

• Species 156–161 form a group of similar species with palmately lobed leaves.

### 156. Ipomoea platensis

Taxon classificationPlantaeSolanalesConvolvulaceae

Ker-Gawl., Bot. Reg. 4: 333. 1818. (Ker-Gawler 1818d: 333)

Convolvulus platensis (Ker-Gawl.) Spreng., Syst. Veg., ed. 16, 1: 591. 1825 [pub.1824]. (Sprengel 1824: 591).
Ipomoea digitata var. septempartita Meisn. in Martius et al., Fl. Brasil. 7: 279. 1869. (Meisner 1869: 279) pro major parte, based partially on Ipomoea platensis Ker-Gawl.
Ipomoea lineariloba Peter, Nat. Pflanzenfam IV(3a): 30. 1897 [pub. 1891]. (Peter 1891: 30). Type. Cultivated in Göttingen from seeds thought to have come from Argentina (lectotype GOET005697, designated by Staples et al. 2012: 676.
Ipomoea platensis var. genuina Hassl., Repert. Spec. Nov. Regni Veg. 9: 155. 1911. (Hassler 1911: 155), nom. illeg. Type. PARAGUAY. Gran Chaco, Santa Rita, *Hassler* 2448 (isotype MPU).
Ipomoea platensis var. quinquepartita Hassl., Repert. Spec. Nov. Regni Veg. 9: 154. 1911. (Hassler 1911: 154). Type. PARAGUAY. Gran Chaco, *K. Fiebrig* “1340” [1240] (lectotype G00175089, designated here).
Ipomoea platensis forma subseptempartita [as var. quinquepartita forma subseptempartita] Hassl., Repert. Spec. Nov. Regni Veg. 9: 154. 1911. (Hassler 1911: 154). Type. PARAGUAY. Gran Chaco, Puerto Talavera, *K. Fiebrig* 1402 (lectotype G00175085, designated here; isolectotypes G).
Ipomoea platensis var. subnovempartita Hassl., Repert. Spec. Nov. Regni Veg. 9: 155. 1911. (Hassler 1911: 155). Type. PARAGUAY. [Boquerón/Presidente Hayes], Río Pilcomayo, T. Rojas 77 in Hassler (holotype G00175088).
Ipomoea platensis var. erecta Hassl., Repert. Spec. Nov. Regni Veg. 9: 155. 1911. (Hassler 1911: 155). Type. not cited, PARAGUAY. [Presidente Hayes], west bank of Río Paraguay, Rojas in Hassler 2448a (lectotype G00175091, designated here; isolectotype BM).

#### Type.

Cultivated from seed sent by Cooper from the banks of the Río Plata, apparently not preserved, lectotype t. 433 of the Botanical Register 4 (1818), designated here.

#### Description.

Perennial with stout tuberous roots, stems glabrous, or, less commonly, pubescent, decumbent, erect or twining. Leaves petiolate, 2–8 × 2–9 cm, palmately 5–9-lobed nearly to the base, the lobes 20–55 × 2–10 mm, oblong-oblanceolate, obtuse or acute, margin entire to undulate, base truncate, both surfaces glabrous, abaxially paler; petioles 1–3.5 cm. Inflorescence of 1–7 flowers in pedunculate, axillary cymes; peduncles 1–4 (–13) cm, glabrous; bracteoles 3–4 mm, lanceolate, caducous; pedicels 5–15 mm, thickened upwards, glabrous; sepals 6–10 mm, slightly unequal, obovate to broadly elliptic, obtuse, glabrous, the inner suborbicular and with scarious margins; corolla 4.5–6 cm long, funnel-shaped, pink, glabrous, limb 4–5 cm diam., entire; stamens short. Capsules 7 × 8 mm, subglobose, glabrous, rostrate; seeds tomentose with longer hairs on margins.

#### Illustration.

**Figure 91.**
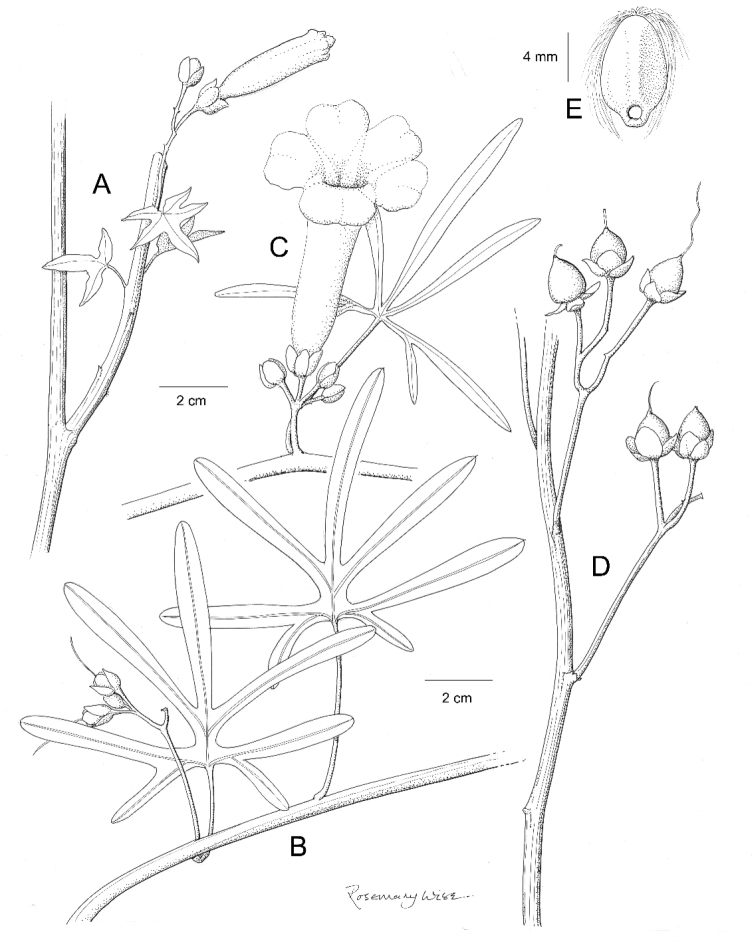
*Ipomoea platensis*. **A** habit **B** habit with leaves **C** inflorescence and corolla **D** fruiting inflorescence with capsules **E** seed. Drawn by Rosemary Wise **A, D, E** from *Spichiger et al.* 2506; **B** from *Spichiger et al.* 2793; **C** from *Fiebrig* 1402.

Figures 83E, F; 91; O’Donell (1959b: 215).

#### Distribution.

Essentially a plant of swampy areas principally along the Paraná-Pilcomayo river systems but also occurring in dry inter-Andean valleys at around 2000 m in Salta. **URUGUAY.***E. Gibert* 176 (K); *P. Favresse* s.n. (P).

**ARGENTINA. Chaco**: Col. Benítez, *A.G. Schulz* 10989 (CTES), 18060 (CTES); La Paz, *H. Keller* 3914 (CTES). **Corrientes**: 12 km E de Colonia Pellegrini, *Tressens et al.* 3744 (CTES, K); Est. Santa María, *T.M. Pedersen* 3303 (C, K, S); Mercedes, *J. Irigoyen & A. Schinini* 159 (CTES, FTG). **Entre Ríos**: Victoria, *E.K.A. Mari* 730 (CTES). **Federal**: *S. Venturi*, 31 (S); *Castellanos* 31-1262 (CTES). **Formosa**: 3 km NW of Pirané, *I. Morel* s195 (S); 324 (K); Laishi Res. Ecol. El Bagual, *A. di Giacpomo* 377 (CTES). **Salta**: Valle de Lerma, *Palaci* 345 (MCNS).

**PARAGUAY. Alto Paraguay**: *K. Fiebrig* 1402 (K); Río Timané, *R. Spichiger et al.* 2506 (FCQ, G), 2793 (FCQ, G). **Guairá**: 15 km N of Tebicuary, *E. Zardini & R, Velázquez* 24088 (MO). **Presidente Hayes**: *E. Zardini & Guerrero* 41637 (FTG); Santa Asunción, *J. de Egea et al.* 192 (BM, FCQ).

**BRAZIL. Paraná**: *Braga* 44 (MBM).

#### Note.

This species is very variable in the number of leaf segments.We have received reports from Mario Giorgetti and seen photographs of this species growing between 2000 and 2500 m in the Calchaqui valley in Salta (Argentina), for example at Cerro Negro, Angastaco at 2060 m. This is at a much higher altitude and more arid habitat than is otherwise known for *Ipomoea platensis* and these populations merit further study. However, *I. platensis* develops very stout tuberous roots, which must be extremely drought-resistant and has long been cultivated successfully as a pot plant.

### 157. Ipomoea mauritiana

Taxon classificationPlantaeSolanalesConvolvulaceae

Jacq., Collectanea 4: 216. 1790 [pub.1791]: 216 (Jacquin 1791: 216)

Ipomoea paniculata var. mauritiana (Jacq.) Kuntze, Rev. Gen. 2: 445 (Kuntze 1891: 445).
Convolvulus paniculatus L., Sp. Pl., ed. 1, 156. 1753. (Linnaeus 1753: 156). Type. Icon in Rheede, Hort. Malab. 11, t. 49 (1692), lectotype designated by Verdcourt (1963: 135).
Ipomoea paniculata (L.) R. Br., Prodr. 486.1810. (Brown, R. 1810: 486), nom. illeg., non Ipomoea paniculata Burm. f. (1768).
Batatas paniculata (L.) Choisy, Mém. Soc. Phys. Genève 6: 436 (54). 1834. (Choisy 1834: 436 [54]).
Modesta paniculata (L.) Raf., Fl. Tellur. 4: 75. 1836 [pub. 1838]. (Rafinesque 1838a: 75).
Ipomoea gossypifolia Willd, Enum. Pl. 208.1809. (Willdenow 1809: 208). Type. Plant of unknown origin (holotype B-W03761).
Convolvulus insignis Andrews, Bot. Reposit 10: t. 636. 1811. (Andrews 1810–15: t. 636). Type. Cultivated plant of unknown origin, lectotype Icon, t. 636 in Bot. Reposit. 10, designated here.
Ipomoea insignis (Andrews) Spreng., Syst. Veg., ed. 16, 1: 592. 1825 [pub.1824]. (Sprengel 1824: 592).
Modesta insignis (Andrews) Raf., Fl. Tellur. 4: 76. 1836 [pub. 1838]. (Rafinesque 1838a: 76).
Ipomoea ennealoba P. Beauv., Flore d'Oware 2: 69. 1819. (Beauvois 1808–20: 69). Type. “Chama” (lectotype Plate 101 in Beauvois (1808–20), designated here).
Ipomoea eriosperma P. Beauv., Flore d'Oware 2: 73. 1819. (Beauvois 1808–20: 73). Type. “le long de la mer depuis Chama jusqu’a la rivière Formose” (lectotype Plate 105 in Beauvois (1808–20), designated here).
Ipomoea bignonioides Sims, Bot. Mag. 53: t. 2645. 1826. (Sims 1826: t. 2645). Type. Icon, t. 2645 in Bot. Mag., epitype, Schomburgk 701 (K000768180), designated by Wood and Scotland 2017a: 6).
Convolvulus bignonioides (Sims) Spreng., Syst. Veg., ed. 16, 4(2, Cur. Post.): 60. 1827. (Sprengel 1827: 60).
Apopleumon bignonioides (Sims) Raf., Fl. Tellur. 4: 72. 1836 [pub. 1838]. (Rafinesque 1838a: 72).
Batatas bignonioides (Sims) G. Don, Gen. Hist. 4: 261. 1838. (Don 1838: 261).
Ipomoea pedata G. Don, Gen. Hist. 4: 281. 1838. (Don 1838: 281). Type. ECUADOR. Guayaquil, *Ruiz & Pavón* (lectotype MA 814670, designated by Wood et al. (2015: 83), isolectotype MA 814671).
Ipomoea pavonii Choisy in A.P. de Candolle, Prodr. 9: 390. 1845. (Choisy 1845: 390). Type. ECUADOR. Guayaquil, *J.A. Pavón* (lectotype G00227879, designated here).
Batatas edulis var. platanifolia Choisy in A.P. de Candolle, Prodr. 9: 339. 1845. (Choisy 1845: 339). Type. GUYANA. R. Schomburgk 701 (isotype BM, BR, K, OXF).
Ipomoea digitata var. quinquefida Meisn. in Martius et al., Fl. Brasil. 7: 278. 1869. (Meisner 1869: 278). Type. GUYANA. R. Schomburgk 701 (lectotype BR0000530737, chosen by Wood and Scotland 2017a: 6).
Ipomoea digitata var. septemfida Meisn. in Martius et al., Fl. Brasil. 7: 279. 1869. (Meisner 1869: 279). Type. INDIA. *N. Wallich* 1350 (lectotype K001112845, designated here).
Ipomoea digitata var. septempartita Meisn. in Martius et al., Fl. Brasil. 7: 279. 1869. (Meisner 1869: 279), p.p. quoad *Burchell* 9924 (BR).
Ipomoea supersticiosa Barb. Rodr., Vellosia 1885-6, 1: 61, t. 17. (Barbosa Rodrigues 1885–6: 61). Type. BRAZIL. Amazonas, Ríos Negro & Yauapery, J. Barbosa Rodrigues in Mus. Bot. Amaz. 634 (whereabouts unknown, lectotype t. 17 in Barbosa Rodrigues 1885–6, designated here).
Ipomoea paniculata var. heterophylla Kuntze, Rev. Gen. 2: 445. 1891. (Kuntze 1891: 445). Type. ECUADOR. Guayas, Guayaquil, Sinclairs.n. (K ex Herb Bentham, lectotype designated here).
Ipomoea digitata auct. mult.

#### Type.

Plant, reputedly from Maurice (Mauritius) cultivated in Vienna, probably not preserved; possible type tab. 200 in Hort. Schoenb. (Jacquin 1797).

#### Description.

Vigorous creeping or climbing perennial, stems somewhat woody, sometimes winged when old, glabrous. Leaves petiolate, 5–14 × 6–16 cm, 5-lobed to about two thirds, base shallowly cordate to truncate and cuneate onto the petiole, lobes elliptic, narrowed at both ends, apex obtuse, both surfaces glabrous, abaxially paler; petioles 2–6 cm, usually glabrous. Inflorescence of pedunculate axillary, occasionally compound cymes; peduncles 3–13 cm, glabrous or puberulent; bracteoles c. 6 mm long, linear, caducous; secondary peduncles (if present) 5–15 mm; pedicels 5–22 mm, puberulent; calyx subglobose in outline, the sepals slightly unequal, elliptic, convex, coriaceous with a very narrow scarious margin, glabrous or puberulent near base, 7–10 × 5–6 mm, the outer obtuse, the inner rounded; corolla 5–6 cm long, inflated above a narrow basal tube, pink, glabrous, limb c. 3 cm diam. Capsules 10–15 × 6–10 mm, ovoid, glabrous; seeds 6 mm long, lanate.

#### Illustration.

Figure 84C; Austin (1998: 403); Bosser and Heine (2000: 37); Deroin (2001: 215).

#### Distribution.

Pantropical in distribution but preferring equatorial regions. Scattered and rather uncommon in the neotropics, perhaps more common in the Guianas than elsewhere in the New World.

**BRAZIL. Amazonas**: *B.A. Krukoff* 6510 (K, S). **Maranhão**: *Froes in Krukoff* 11650 (S). **Rio de Janeiro**: *A. Glaziou* 11262 (P). **Rio Grande do Norte**: *M.T. Dawe* (K). **Pará**: *G.M. Pies & G.A. Black* 1620 (P). **Pernambuco**: Fernando do Noronha, *Ridley et al*. 91 (BM, P). Also reported from Amapá in Flora do Brasil 2020 under construction.

**GUYANA.***A.S. Hitchcock* 17513 (NY, S); *Jansen-Jacobs et al*. 4327 (K, U), 4759 (K, U); *P. Maas et al.* 7390 (K, U); *N. Sandwith* 158 (K).

**SURINAM.***Sterringa* 12432 (K); *Berthoud-Coulon* 508 (BM).

**FRENCH GUIANA**: *Rothery* 177 (K); *O. Tostain* 232(P); *M.F. Prevost* 697 (P).

**BOLIVIA. Beni**: Iténez, *D. Ibañez* 295 (LPB). **Pando**: Manuripi, Conquista, *E. de la Sota* 976 (LIL); *F. Fernández Casas & A. Susana* 8598 (LPB, NY, MO). **Santa Cruz**: Germán Busch, Puerto Suárez, *M. Mendoza et al.* 2548 (USZ, K).

**PERU. Loreto**: *Asplund* 14512 (S); *J. Ruiz* 1168 (K); *R. Vásquez et al.* 3693 (K, MO, USM). **Madre de Dios**: Tambopata, Pampas del Heath, *M. Aguilar & D. Castro* 453 (MO, OXF).

**ECUADOR. Guayas**: *Sinclair* s.n. (K); *K.T. Hartweg* s.n. (K); *L. Fraser* s.n. (BM). **Manabí**: 50 km N of Pedernales, *D. Weberbauer et al.* 11712A (MO, OXF, QCNE).

**COLOMBIA. Amazonas**: *J.M. Duque* 2439 (COL). **Antioquia**: *E. Forero* 1999 (COL). **Casanare**: *C. Camargo* 033 (COL).

**VENEZUELA. Amazonas**: *Wessels Boer* 1928 (K); *Chaffanjon* s.n. (P)

**PANAMA.***H. Pittier* 4392 (US); *Duchassaing* s.n. (P)

**COSTA RICA.** Cahuita National Park, *P. Wilkin & S.B. Jennings* 119 (BM, MO); Limon, Puerto Viejo, *A. Cascante & M. Zamora* 388 (K).

**NICARAGUA.***P. Moreno* 12360 (MO).

**HONDURAS.** La Mosquitia, *C. Ashe* 56 (BM); *J. Hjalmarson* 16/7/1852 (S).

**BELIZE.** Sittee River, *W.A.Schipp* 636 (BM, K, MO, S).

**JAMAICA.***G.R. Proctor* 29322 (BM).

**DOMINICAN REPUBLIC.***E. Ekman* H15635 (S), H12303 (S).

**PUERTO RICO.** Sine data (P)

**LESSER ANTILLES. Guadeloupe**: *Stehlés*.n. (P). **Martinique**: *M. Belanger* s.n. (P).

**St. Vincent**: *L. Guilding* s.n. (K). **Barbados**: *E.G.B. Gooding* 680 (BM).

**TRINIDAD.***B.O. Williams* 12038 (K).

#### Typification.

*Ipomoea pavonii* Choisy is based on *I. pedata* G. Don as exemplified by the specimen at Geneva (G00227879). Hallier based *Calonyction pavonii* on the Tafalla specimen at G (00016027), which is *Ipomoea setosa*.

*Ipomoea paniculata* var. “*eriocarpa* (Beauv.) Kuntze” appears to be a mistake for var. eriosperma.

There are several syntypes of *Ipomoea paniculata* in the Wallich Herbarium, which could be selected as a lectotype of *Ipomoea digitata var. septemfida*. As there seems no suitable specimen in Martius’ herbarium, we have rather arbitrarly selected one of the Wallich specimens with 7-fid leaves as lectotype.

#### Note.

*Ipomoea mauritana* is a very variable plant, particularly in the Old World where forms with unlobed leaves are reported. It is somewhat unsatisfactorily distinguished from *Ipomoea cheirophylla* and similar species, and records in floras, checklists and data bases are often unreliable. It is a plant of humid tropical lowlands and the leaves are larger than in related species and the inflorescence is commonly compound. *Sinclair* s.n. from Guayaquil illustrates this well; some leaves are entire, some have prominent lateral teeth, some are 3-lobed to over half way and some are 5-lobed almost to base, the lobes oblong to ovate.

### 158. Ipomoea maranyonensis

Taxon classificationPlantaeSolanalesConvolvulaceae

J.R.I. Wood & Scotland, Kew Bull. 72: 6. 2017. (Wood and Scotland 2017b: 6)

#### Type.

PERU. Amazonas, Bagua Province, Imaza District, Com. Yamayakat, *R. Vásquez, A. Peña & E. Chávez* 23929 (holotype FTG115761, isotypes MO, USM).

#### Description.

Liana of unknown height, apparently glabrous in all parts; stems glabrous. Leaves petiolate, 5.5–10 × 7–8 cm, 3–5-lobed to near the base, the 4^th^ and 5^th^ lobes often only partially developed, lobes oblong-elliptic or lanceolate, 0.5–2.5 cm wide, acuminate; base truncate, abaxially paler; petioles 4–6 cm. Inflorescence of compounded axillary cymes 20–30 cm long; peduncles 9–12 cm long; 2–6^th^ degree peduncles 1–4 cm; bracteoles 1 mm, oblong, scale-like, caduous; pedicels 7–11 mm; sepals subequal, 5–7 × 3–4 mm, elliptic, coriaceous, convex, outer rounded, minutely mucronate, inner ±scarious, rounded; corolla c. 4 cm long, pink, funnel-shaped, glabrous; limb c. 2.5 cm diam., the midpetaline bands ending in teeth. Capsules 6 × 4 mm, ovoid with a slender persistent style, glabrous; seeds (possibly immature) 3 × 1.5 mm, pilose with long white hairs on the margins.

#### Illustration.

Figure 92.

**Figure 92.**
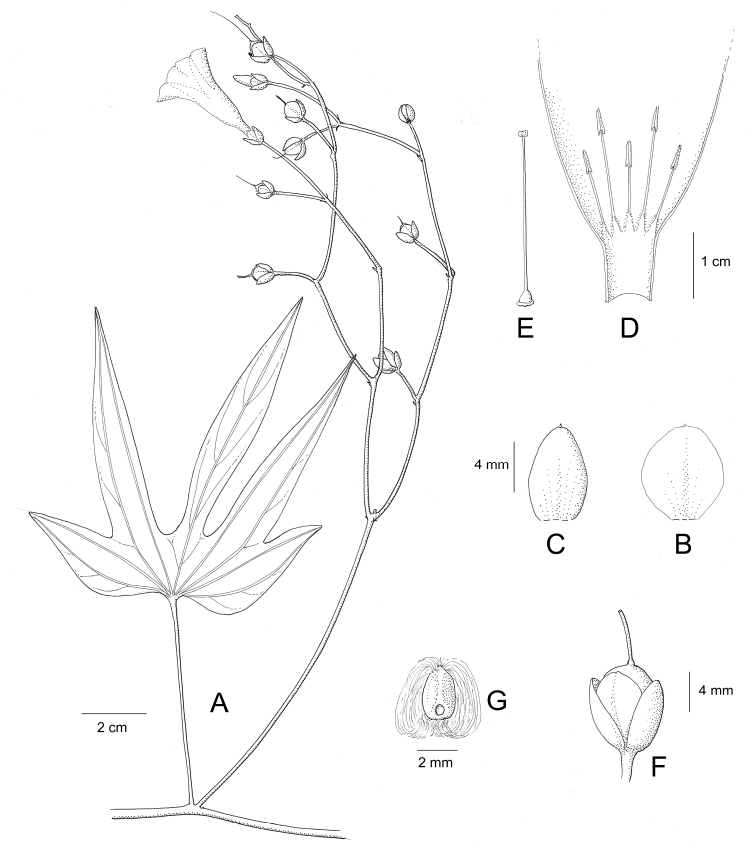
*Ipomoea maranyonensis*. **A** habit **B** outer sepal **C** inner sepal **D** corolla opened out to show stamens **E** ovary and style **F** calyx and capsule **G** seed. Drawn by Rosemary Wise **A, F, G** from *R. Vazquez et al.* 18569; **B–E** from *R. Vazquez* 23929.

#### Distribution.

“Transitional Primary Forest” in the Marañon Valley in northern Peru.

**PERU. Amazonas**: Prov. Bagua, *R. Vásquez et al.* 18569 (FTG, MO).

#### Note.

This species has been identified as *Ipomoea mauritiana* and is clearly related to that very variable species. However, it is immediately distinguished by the compound axillary inflorescences which reach 30 cm in length and are divided up to six times. Additionally, the sepals, corolla and capsules are all much smaller than in *I. mauritiana*.

### 159. Ipomoea cheirophylla

Taxon classificationPlantaeSolanalesConvolvulaceae

O’Donell, Lilloa 29. 141. 1959. (O’Donell 1959b: 141)

#### Type.

ARGENTINA. Salta, Dept. Rosario de la Frontera, Las Termas, *C. O’Donell* 5360 (holotype LIL, not seen).

#### Description.

Twining perennial 2–5 m in height, stems wiry, glabrous or pubescent. Leaves petiolate, 4–6(–10) × 5–7(–10) cm, 5–7-lobed to just above the base, base truncate or broadly cordate and cuneate onto the petiole, lobes oblong-elliptic, narrowed at both ends, apex obtuse and mucronate, usually glabrous but pubescent in the Tarija area; petioles 1–4(–8) cm. Inflorescence of usually compound, axillary cymes of 1–5(–7) flowers; peduncles 2–8 cm, pubescent; bracteoles 2 mm, oblanceolate, caducous; secondary peduncles 1.3–2.2 cm; pedicels 7–18 mm, pubescent; sepals slightly unequal, elliptic, convex, coriaceous with a very narrow scarious margin, glabrous or puberulent near base, the outer 6–10 × 3–5 mm, obtuse, inner 5–7 mm wide, rounded; corolla 4–5 cm long, funnel-shaped, pink, glabrous, limb 4 cm diam., shallowly lobed. Capsules 10 × 8 mm, ellipsoid to subglobose, glabrous; seeds 5–6 mm long, dark brown, woolly.

#### Illustration.

O’Donell (1959b: 143).

#### Distribution.

Scattered in occurrence in northern Argentina, western Paraguay, southern Bolivia and the extreme west of Brazil. Essentially a species of the western and northern Chaco fringes. It is usually a species of the dry inter-Andean valleys and chaco lowlands but sometimes grows in seasonally swampy areas suggesting its ecological requirements are not as distinct from those of *Ipomoea mauritiana* as suggested by O’Donell (1959b: 146).

**ARGENTINA. Catamarca**: Yacatula, *C. Spegazzini* s.n. [3/1897] (LP). **Cordoba**: *C. Spegazzini* s.n. [11/1902-3/1903] (LP). **Formosa**: Matacos, *Solis Neffa et al.* 588 (CTES). **Jujuy**: *Zuloaga et al.* 10244 (CTES). **Salta**: Anta, *E. Saravia* 1267 (CTES); Capital, Sierra de Vélez, *L.J. Novara* 5889 (G). **Tucumán**: *Legname & Cuezzo* 4515 (CTES, LIL); Yerba Buena, *S. Venturi* 1328 (LIL, LP, SI).

**PARAGUAY. Alto Paraguay**: Puerto Casado, *T. Rojas* 3031 (LIL, SI); 4 de Mayo–Lagarenza *F. Mereles* 2631(FCQ); P.N. Defensores del Chaco, *M. Vavrek & E. Enciso* 36 (PY). **Boquerón**: 10 km NW of Nueva Asunción, *A. Krapovickas et al.* 45436 (CTES)

**BRAZIL. Mato Grosso do Sul**: Corumbá, *S. Moore* 972 (BM); *A. Pott et al.* 4837 (CPAP).

**BOLIVIA. Beni**: Ballivián, *J. Balderrama* 366 (LPB, CTES); Cercado, Ibiato, *M.T. Martinez & M. Adler* 75 (K, LPB, USZ). **Chuquisaca**: Luis Calvo, *E. Saravia & Nelson* 10474 (CTES); Tomina, *J.R.I. Wood* 8003 (K, LPB); Zudañez, *J.R.I. Wood & H. Huaylla* 21548 (HSB, K, LPB). **Cochabamba**: Campero, *J.R.I. Wood & H. Huaylla* 20256 (K, LPB). **La Paz**: Iturralde, Luisita, *S.G. Beck & R. Haase* 10005 (BOLV, LPB). **Santa Cruz**: Caballero, *M. Nee* 46682 (USZ, MO, NY); Chiquitos, Quimome, *J.R.I. Wood & B. Williams* 27906 (USZ); 30 km SE of Pailón, *G. Navarro* 2155 (CTES); Cordillera, *M. Mendoza* 2731 (USZ); Vallegrande, Pucará, *J.R.I. Wood & M. Mendoza* 21488 (K, LPB, USZ); Velasco, El Refugio, *J.R.I. Wood & H. Huaylla* 20753 (K, LPB, USZ). **Tarija**: Arce, *T. Meyer* 21810 (LIL); Gran Chaco, Palos Blancos, *J.R.I. Wood et al.* 27616 (K, LPB, USZ); O’Connor, *M. Mendoza* 2877 (K, USZ).

#### Note.

Similar to *Ipomoea mauritiana* but more slender (leaves mostly < 7 cm long) and inflorescence fewer flowered with longer secondary and tertiary peduncles so the inflorescence appears more lax. The two species are not always easily separated but were drscussed in detail by O’Donell (1959b).

### 160. Ipomoea blanchetii

Taxon classificationPlantaeSolanalesConvolvulaceae

Choisy in A.P. de Candolle, Prodr. 9: 387. 1845. (Choisy 1845: 387)

#### Type.

BRAZIL. [Bahia], Serra de Açurua, Rio São Francisco, *Blanchet* 2906 (holotype G, not found, isotypes BM, K, NY, P).

#### Description.

Twining perennial herb, stem bifariously pubescent, glabrescent. Leaves petiolate, 3–6 × 4–7 cm, 3-lobed to slightly more than halfway (the lateral lobes sometimes shallowly lobed), base broadly cordate, apex obtuse and mucronate, glabrous; petioles 3–7 cm, glabrous. Inflorescence of shortly pedunculate axillary cymes; peduncles 5–20 mm; bracteoles not seen; secondary peduncles, if present, 2–4 mm; pedicels 6–18 mm; sepals subequal, coriaceous, convex, elliptic, obtuse, glabrous, 7–8 × 4–5 mm, inner similar but with scarious margins; corolla 3.5–5 cm long, red, funnel-shaped, glabrous, limb 2.5 cm diam. Capsules and seeds not seen.

#### Distribution.

Mostly dry forest or caatinga in northeastern Brazil.

**BRAZIL. Bahia**: Remanso, *L.P. de Queiroz et al.* 10060 (HUEFS). **Ceará**: Serra de Araripe, *G. Gardner* 2424 (BM, K). **Goiás**: Serra Dourada, *H.S. Irwin et al.* 11882 (MO, NY). **Paraíba**: *M. de F. Agra* 1728 (NY). **Pernambuco**: Chapada do Araripe, *M.E. Saraiva* 135 (RB); Morro do Quixaba, *A.M. Miranda* 3214 (RB). **Piauí**: Mun. Floriando, *A.M. Miranda et al*. 5054 (UB, PEUFR); Teresina-Picos, *Rizzini* s.n. (RB); Caracol, *E. Melo et al.* 9243 (HUEFS). **Rio de Janiero**: *A.F.M. Glaziou* 11263 (K, P). **Rondônia**: 24 km NNW of Ariquemes, *D. Frame et al.*112 (NY).

#### Notes.

Part of the *Ipomoea mauritiana* complex, this species differs from *I. cheirophylla* in the generally 3-lobed leaves, with usually obtuse segments, but may prove conspecific although molecular sequencing suggests it may be distinct. Records from Brazil in Flora do Brasil 2020 under construction almost certainly include collections of *I. cheirophylla* (from Paraná), *I. caloneura* (from Mato Grosso and perhaps Minas Gerais) and *I. mauritiana* from the Amazon basin.

A specimen at P (*Mocquerys* s.n.) from Carabobo in Venezuela is atypical in having 5-lobed leaves and might be a dwarf form of *Ipomoea mauritiana* or even *I. cheirophylla*.

### 161. Ipomoea caloneura

Taxon classificationPlantaeSolanalesConvolvulaceae

Meisn. in Martius et al., Fl. Brasil. 7: 281. 1869. (Meisner 1869: 281)

Ipomoea blanchetii var. pubescens Meisn. in Martius et al., Fl. Brasil. 7: 280. 1869. (Meisner 1869: 280). No type cited, presumably BRAZIL. Mato Grosso, Cuiabá, L. Riedel [818] (lectotype NY00319160, designated here; isolectotype LE01025974).
Ipomoea tapirapoanensis Hoehne, Arq. Bot. Estado São Paulo, new ser. 1: 38. 1938. (Hoehne 1938: 38). Type. BRAZIL. Mato Grosso, Tapirapoa[n], *F.C. Hoehne* 1668 (isotype R!).

#### Type.

BRAZIL. Goiás, *W.J. Burchell 6582* (holotype BR0000006972875, isotype K).

#### Description.

Trailing or climbing perennial, stems thinly pilose. Leaves petiolate, 2–6 × 2.5–8 cm, base cordate with rounded auricles, margin somewhat undulate, 3-lobed to about half way, lobes acute to shortly acuminate, central lobe elliptic, narrowed at base, laterals broadly ovate, both surfaces pilose, abaxially paler; petioles 1.5–3 cm, pilose. Inflorescence of mostly 3–5-flowered axillary cymes; peduncles 3(–11) cm, glabrous or thinly pilose; bracteoles linear-lanceolate, 2–4 mm; secondary peduncles 1–4.5 cm; pedicels 6–20 mm, glabrous or, rarely, thinly pilose; sepals unequal, coriaceous, convex, glabrous, margins scarious, when fresh pale green, shiny; outer sepals 6–7 × 4 mm, elliptic, obtuse, inner 10–11 mm, obovate, obtuse; corolla 6–7 cm long, funnel-shaped, pale pink with dark centre, glabrous, limb c. 5 cm diam., lobed. Capsules 7–8 × 6 mm, ovoid, glabrous, rostrate; seeds 4–5 × 2.5 mm pale brown, glabrous apart from lanate angles with hairs c. 10 mm long.

#### Illustration.

Figure 93.

**Figure 93.**
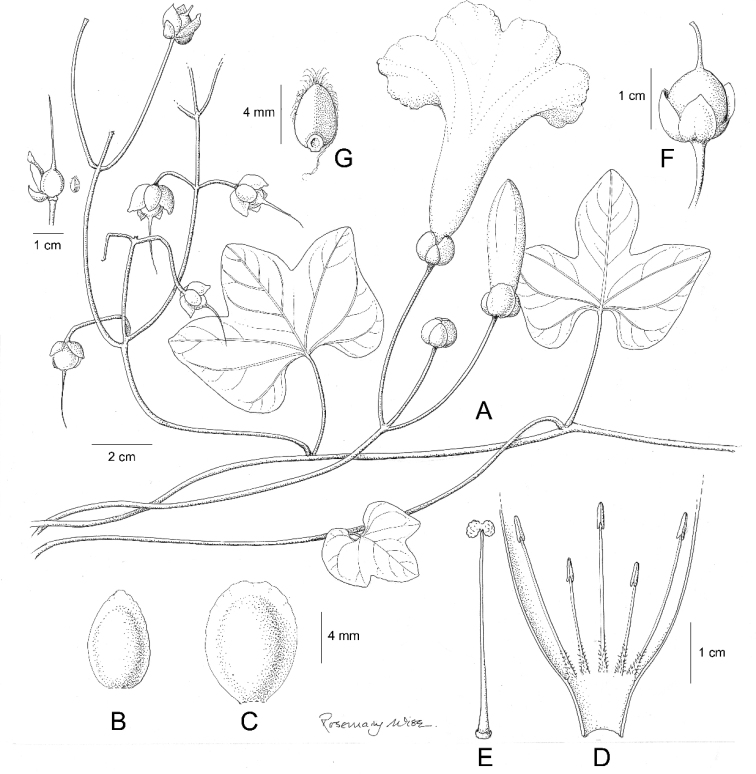
*Ipomoea caloneura*. **A** habit with (left) capsule and seed **B** outer sepal **C** inner sepal **D** corolla opened up to show stamens **E** ovary and style **F** capsule **G** seed. Drawn by Rosemary Wise **A–E** from *Wood & Soto* 27916; **F, G** from *Wood & Soto* 27430.

#### Distribution.

An uncommon plant of the Cerrado biome in Bolivia and Brazil, growing in campo cerrado and on granite rock outcrops.

**BRAZIL. Goiás**: the type. **Mato Grosso**: Espinheiros, near Cuiabá, *C.A.M. Lindman* 3013 (S); Chapada dos Guimares, Cuiabá, *F. Mereles* 2493 (FCQ), 2524 (FCQ). **Tocantins**: Palmeiropolis, *J.B. Pereira & G.A. Moreira* 65 (CEN).

**BOLIVIA. Santa Cruz**: Velasco: Las Mechitas, *R. Guillén et al.* 311 (FTG, LPB, USZ); km 69, Santa Rosa-Piso Firme, *J.R.I. Wood & D. Soto* 27430 (K, LPB, USZ); Cerro Pelao, *J.R.I. Wood & D. Soto* 27916 (OXF, K, LPB, USZ).

#### Note.

Very similar to *Ipomoea blanchetii and I. cheirophylla* differing in the pilose stem and leaves. The leaves are always 3-lobed with much broader segments than in *I. cheirophylla*.

In designating a lectotype of Ipomoea blanchetii var. pubescens, we have chosen the NY specimen as it appears to have a label in Meisner’s handwriting annotating it as “β pubescens nob. (6./1./68.)”. Both this specimen and the one at LE are so poor that they are difficult to identify certainly but the location and pubescent indumentum suggest strongly that it is *Ipomoea caloneura*.

• Species 162–165 are characterised by the presence of stellate hairs. These are completely absent in a few forms of *Ipomoea bonariensis* and are hard to find in Species 164–165.

### 162. Ipomoea bonariensis

Taxon classificationPlantaeSolanalesConvolvulaceae

Hook., Bot. Mag. 65: t. 3665. 1839. (Hooker 1839: t. 3665)

Convolvulus hookerianus D. Dietr., Syn. Pl. 1: 666. 1839. (Dietrich 1839: 666). Type. Based on Ipomoea bonariensis Hook.
Ipomoea heterotricha Meisn. in Martius et al., Fl. Brasil. 7: 280. 1869. (Meisner 1869: 280), nom. illeg., non Ipomoea heterotrichaDidrichsen (1856). Type. BRAZIL. *F. Sello(w)* (holotype ?B†, n.v.).
Ipomoea obtusiloba Meisn. in Martius et al., Fl. Brasil. 7: 283. 1869. (Meisner 1869: 283). Type. BRAZIL. *F. Sello* 3605 (holotype B†, photo F; possible type BR0005951314 sine col.).
Ipomoea obtusiloba var. tridens Meisn. in Martius et al., Fl. Brasil. 7: 283. 1869. (Meisner 1869: 283). Type. “Uruguay and Brazil, Parana”, *J. Tweedie* 7 (whereabouts unknown).
Ipomoea astrotrichota Dammer, Bot. Jahrb. Syst. 23(5), Beibl. 57): 40. 1897. (Dammer 1897: 40). Type. BRAZIL. “environs de Rio de Janeiro”, *A.F.M. Glaziou* 13008 (holotype B†; isotypes K, P).
Ipomoea bonariensis var. calvescens Hallier f., Jahrb. Hamburg. Wiss. Anst. 16: 51. 1899. (Hallier 1899a: 51). Type. Based on I. astrotricota Dammer
Ipomoea bonariensis subvar. triloba Hallier f. [as var. calvescens subvar. triloba], Jahrb. Hamburg. Wiss. Anst. 16: 51. 1899. (Hallier 1899a: 51). Type. Based on I. astrotricota Dammer
Ipomoea bonariensis subvar. integrifolia Hallier f. [as var. calvescens subvar. integrifolia], Jahrb. Hamburg. Wiss. Anst. 16: 52. 1899. (Hallier 1899a: 52). Type. BRAZIL. Rio de Janeiro, Lagoa de Freitas, *E. Ule* 3852 (holotype B†, isotype ?HBG, n.v.).
Ipomoea bonariensis var. genuina Chodat & Hassl., Bull. Herb. Boiss., ser. 2: 5: 695.1905. (Chodat and Hassler 1905: 695), nom. illeg.
Ipomoea bonariensis forma villicaulis Chodat & Hassl. [as var. genuina forma villicaulis], Bull. Herb. Boiss., sér. 2, 5: 695. 1905. (Chodat and Hassler 1905: 695). Type. PARAGUAY. Cordillera de Los Altos, *E. Hassler* 3798 (lectototype G00174714, designated here; isolectotypes G).
Ipomoea bonariensis var. grandiflora Chodat & Hassl., Bull. Herb. Boiss., ser. 2: 5: 695. 1905. (Chodat and Hassler 1905: 695). Type. PARAGUAY. *E. Hassler* 2984 (lectotype P0355032, designated here; isolectotypes BM, K000612836).
Ipomoea bonariensis var. cordifolia Chodat & Hassl., Bull. Herb. Boiss., ser. 2: 5: 695. 1905. (Chodat and Hassler 1905: 695). Type. PARAGUAY. Río Apa, *E. Hassler* 8384a (G, not found).
Ipomoea bonariensis var. rupestris Chodat & Hassl., Bull. Herb. Boiss., ser. 2: 5: 695. 1905. (Chodat and Hassler 1905: 695). Type. PARAGUAY. Tobatí, *E. Hassler* 6163 (lectotype G00174719, designated here; isolectotypes BM, G).
Ipomoea bonariensis subsp. mollis Hassl., Fedde, Repert. Spec. Nov. Regni Veg.9: 153. 1911 (Hassler 1911: 153). Type. PARAGUAY. Concepción, Río Paraguay, *E. Hassler* 7378 (lectotype NY00319150, designated here; isolectotype BM).
Ipomoea bonariensis forma cordata [as var. grandiflora forma cordata] Hassl., Repert. Spec. Nov. Regni Veg. 9: 153. 1911. (Hassler 1911: 153). Type. PARAGUAY. *E. Hassler* 8284a (lectotype NY00621757, designated here).
Ipomoea bonariensis forma glabrata [as var. grandiflora forma glabrata] Hassl., Repert. Spec. Nov. Regni Veg. 9: 153. 1911. (Hassler 1911: 153). Type. PARAGUAY. *E. Hassler* 8284 (holotype G, isotype BM).
Ipomoea bonariensis forma intermedia [as var. grandiflora forma intermedia] Hassl., Repert. Spec. Nov. Regni Veg. 9: 153. 1911. (Hassler 1911: 153). Type. PARAGUAY. *E. Hassler* 8378, 8378a, 8378b. (syntypes G?).
Ipomoea bonariensis var. pubisepala [as subsp. mollis var. pubisepala] Hassl., Repert. Spec. Nov. Regni Veg. 9: 153. 1911. (Hassler 1911: 153). Type. PARAGUAY. *E. Hassler* 1513 (BM, K), 1681 (?G), 2984 (BM, K00612836), 3798 (G00174713, G00175936, P03550327), all syntypes.
Ipomoea biglandulosa Arechav., An. Mus. Nac. Montevideo 7: 204. 1911. (Arechavaleta y Balpardo 1911: 204). Type. URUGUAY, no type cited, lectotype, specimen labelled “Ipomoea biglandulosa n. sp. Orillas del Uruguay, florece en verano,” (MVM), designated here.
Ipomoea florentiana Hoehne, Anexos Mem. Inst. Butantan, Secc. Bot. 1, Fasc. 6: 73. 1922. (Hoehne 1922: 73). Type. BRAZIL. São Paulo, 2 April 1918, J. Florêncio Gomez 1742 (holotype SP).
Ipomoea corumbaensis Hoehne, Anexos Mem. Inst. Butantan, Secc. Bot. 1, Fasc. 6: 74. 1922. (Hoehne 1922: 74). Type. BRAZIL. Mato Grosso do Sul, *Hoehne* 2741 (holotype SP).
Ipomoea bonariensis var. chacoensis O’Donell, Lilloa 29: 125. 1959. (O’Donell 1959b: 125). Type. ARGENTINA. Chaco, Dept. Tapenagá, *C.L. Schulz* 1500 (holotype LIL001226).
Ipomoea bonariensis var. erecta J.R.I. Wood & Scotland, Kew Bull. 70(31): 80. 2015. (Wood et al. 2015: 80). Type. PARAGUAY. Nueva Asunción, cerca del cuartel de Gral. Eugenio A. Garay, *A. Charpin & L. Ramella* 21528 (holotype G).

#### Type.

Cultivated plant grown from seed collected by Tweedie at Buenos Aires (lectotype K00612912, designated by Wood et al. 2015: 79).

#### Description.

A very variable trailing or, more commonly, twining perennial to at least 3 m in height, roots stout, tuberous, stems becoming woody when old, sparsely or densely roughly hirsute with stellate hairs. Leaves petiolate, usually 3–9 × 3–9 cm, ovate, obtuse and mucronate, entire or commonly 3–5-lobed to about half way or margin sinuate, adaxially dark-green, asperous, stellate-pubescent, abaxially grey, stellate-tomentose; petioles 1–6 cm. Inflorescence of pedunculate, 1–10-flowered, axillary cymes, sometimes becoming racemose in form; peduncles 2–8 cm, stout, asperous-stellate-hirsute; bracteoles scale-like, caducous; secondary peduncles 1–3.5 cm; pedicels 2–10 mm (very rarely more); calyx globose in outline, sepals slightly unequal, coriaceous, convex, obtuse, usually glabrous, the margins scarious, outer 5–8 × 4–5 mm, elliptic, inner slightly broader and longer; corolla 4–7 cm long, funnel-shaped, pink, glabrous, limb c. 4 cm diam., shallowly lobed. Capsules narrowly ovoid, 11–12 × 7 mm, glabrous; seeds 5–7 mm long, pilose with hairs 10 mm long.

#### Illustration.

**Figure 94.**
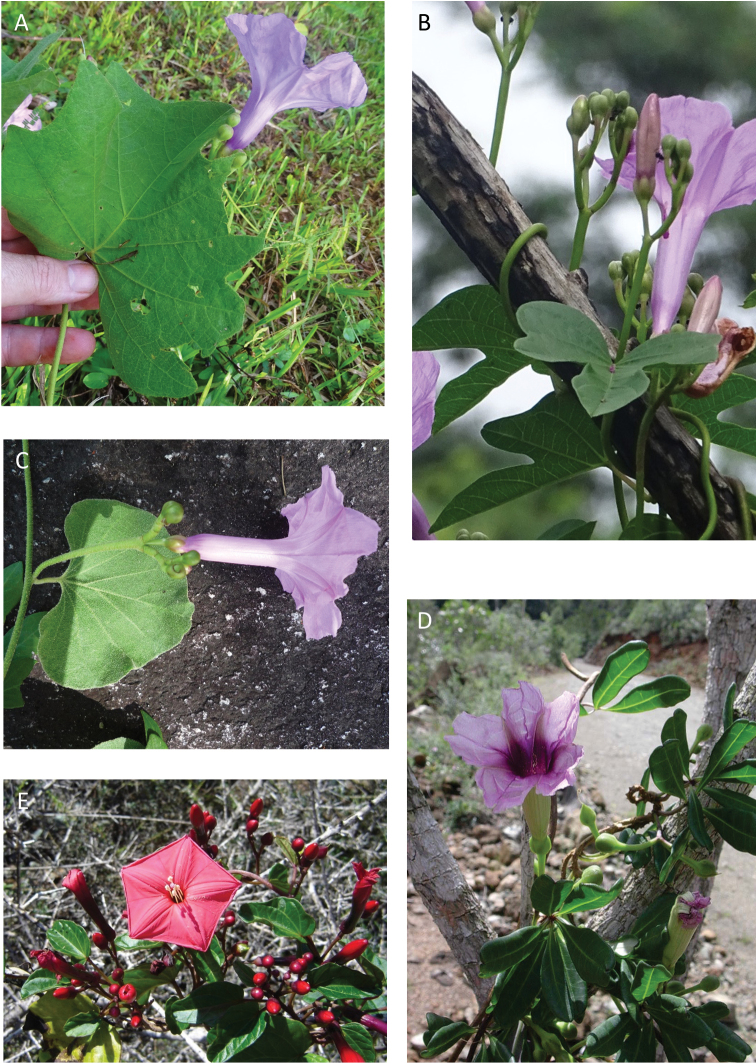
Photographs of *Ipomoea* species. **A–C** Variation in *I. bonariensis***A** form with laciniate leaves **B** form with digitately lobed leaves **C** form wth ovate, cordate leaves **D***I. carolina***E***I. microdactyla*. **A, B** Hector Keller **C** Beth Williams **D, E** Ramona Oviedo.

Figures 9C, 94A–C; O’Donell (1959b: 121).

#### Distribution.

Abundant in much of Paraguay, the Chiquitania of Bolivia, parts of southern Brazil and Misiones in Argentina but scattered and less common elsewhere in Argentina, Bolivia, Uruguay and Brazil. We have not traced the record from Peru (Austin and Huáman (1996), which seems improbable. It is often found in scrub and on forest margins around rock platforms although it is not restricted to this habitat. Its absence from many lowland areas is surprising.

**URUGUAY.** Sine data, *J. Tweedie* s.n. (K, LIL, SI); Artigas, Cuereim, *M.B. Berro* 2568 (K, LIL); Concepción, *P. Lorentz* s.n. [2/1877] (BM).

**ARGENTINA. Buenos Aires**: *C.M. Hicken* 13701 (K, SI), 14476 (K, SI). **Chaco**: *A.G. Schulz* 2076 (CTES) – var. chacoensis. **Corrientes**: *S.G. Tressens et al.* 3595 (CTES, K). **Entre Ríos**: *Lorentz* 927 (BM, CORD). Formosa: Patiño, *A. Krapovickas & C. Cristóbal* 46543 (CTES). **Jujuy**: Ledesma, *L. Galetto* 296 (CORD). **Misiones**: *G.J. Schwarz* 5148 (LIL, S); *H. Keller & G. Prance* 3377 (K); Iguazú, *H. Keller et al.* 2713 (CTES) – forma glabrata; Candelaria, *H. Keller & Franco* 13281 (CTES, OXF) – f. glabrata. Also Catamarca, Salta, Santa Fe and Santiago del Estero, fide O’Donell (1959b) and Austin and Costea (2008). **PARAGUAY. Alto Paraguay**: 30 km SE of Lagerenza, *R. Spichiger et al.* 2581 (G) – var. chacoensis; Cerro León, *F. Mereles* 6639 (FCQ); Nueva Asunción, *F. Mereles & R. Degen* 4900 (FCQ) – var. chacoensis. **Alto Paraná**: *K. Fiebrig* 5381 (BM, K), 6113 (BM, K). **Boquerón**: Destacamento de General Garay, *R. Degen & F. Mereles* 2974 (FCQ) – var. erecta; *F. Mereles & R. Degen* 5139 (FCQ) – var. chacoensis. **Caazapá**: Reserva Tapyta, *I. Vera* 1821 (FCQ). **Cordillera**: Cerro Tobatí, *E. Zardini* 6739 (FCQ, MO); ibid., *E. Zardini & R. Degen* 3647 (FCQ, MO). **Guairá**: Villa Rica: *Balansa* 1176 K), *E. Zardini & R. Velázquez* 8930 (FCQ). **Itapúa**: Isla Yacyreta. *M. Quintana et al.* 141 (PY); ibid., *A. Pin et al.* 70 (PY) – both forma glabrata. **Paraguarí**: *Balansa* 1051 (K); P.N. Ybycuí, *E. Zardini & T. Tilleria* 35051 (PY). **Presidente Hayes**: O. *Aquino & G. Polini* 493 (FCQ) – var. chacoensis. **San Pedro**: *N. Soria* 5513 (FCQ).

**BRAZIL. Bahia**: *Fiaschi et al.* 2758 (CEPEC, NY). **Espirito Santo**: *A.L. Peixoto et al.* 3302 (MO). **Goiás**: Minacu, *G. Periera-Silva* 4806 (CEN). **Mato Grosso do Sul**: *A. Pott et al.* 9917 (CPAP). **Paraná**: Jaguaraíva *P. Dusen* s.n. [13/3/1904] (S). **Rio de Janeiro**: Canteiro, *J.R. Mattos* 306 (RB). **Rio Grande do Sul**: *E. Leite* 2296 (GH); *J. Tweedie* s.n. (K). **São Paulo**: *M.R. Silva* 754 (MO); *C.W. Mosén* 3441 (S). Also Mato Grosso, Minas Gerais and Santa Catarina, all fide Flora do Brazil 2020.

**BOLIVIA. Beni**: Cercado, T.C.O. Ibiato, *M.T. Martinez et al.* 9 (USZ). **Santa Cruz**: Germán Busch, Puerto Suárez–Cerro Mutún, *J.R.I. Wood & D. Villarroel* 25904 (K, LPB, UB, USZ); Chiquitos, Santiago de Chiquitos, *J.R.I. Wood & D. Soto* 27186 (K, LPB, USZ); Cordillera, P.N. Kaa-Iya, *A. Fuentes & G. Navarro* 2338 (BOLV, LPB, MO, USZ); Guarayos, *A. Krapovickas & A. Schinini* 31877 (CTES); Ichilo, Buenavista, *J. Steinbach* 7098 (BM, LIL, K, MO); Ñuflo de Chávez, Piedra de Calama, *J.R.I. Wood* 27458 (K, LPB, USZ); Velasco, *M. Dematteis.et al.* 3564 (CTES, K); San Ignacio-Vilabela, *J.R.I. Wood et al.* 27858 (OXF, K, LPB, USZ). **Tarija**: Gran Chaco, *P. Zuñiga et al.* 20 (HSB) – var. erecta.

#### Lectotypification.

The synonomy of the infraspecific taxa of the very variable *Ipomoea bonariensis* is immensely complicated with some collections being cited by Hassler under several names. Not all collections have been located at Geneva but we have lectotypified names wherever possible in the hope of achieving a degree of nomenclatural stability.

#### Note.

*Ipomoea bonariensis* is usually easily recognised by the stellate hairs that cover all vegetative parts. However, it is extremely variable in habit, leaf form and indumentum and numerous infraspecific taxa have been described. Stems are usually trailing or climbing but plants with erect stems and subterminal inflorescences (I. bonariensis var. erecta) occur in the western Chaco on the borders of Bolivia and Paraguay. Most plants are distinctly stellate hairy, although the indumentum varies from sparse to dense. However, occasional specimens which are entirely glabrous occur (I. bonariensis forma glabrata). These are reported from Paraguay and Brazil but are a particular feature of Misiones Province in Argentina. Leaves are typically entire but they are commonly lobed. Plants with 3–5-lobed leaves were treated as I. bonariensis var. chacoensis by O’Donell but as I. bonariensis subsp. mollis by Hassler, and have been reported from the Argentinian Chaco and neighbouring parts of Paraguay. Some plants have prominently laciniate leaves (*Keller & Franco* 13257) and resemble some forms of *Ipomoea homotrichoidea* in their leaf shape.

### 163. Ipomoea homotrichoidea

Taxon classificationPlantaeSolanalesConvolvulaceae

O’Donell, Lilloa 14: 173. 1948. (O’Donell 1948a: 173)

Ipomoea heterotricha var. homotricha Chodat & Hassl., Bull. Herb. Boiss., ser. 2: 5: 694. 1905. (Chodat and Hassler 1905: 694). Type. PARAGUAY. San Estanislao, Hassler *Hassler* 4168 (lectotype G00174930, designated here).
Ipomoea heterotricha forma suborbiculata Chodat & Hassl. [as var. homotricha Chodat & Hassl. forma suborbiculata], Bull. Herb. Boiss., ser. 2: 5: 694. 1905. (Chodat and Hassler 1905: 694). Type. PARAGUAY. [San Pedro], San Estanislao, *Hassler* 6005 (lectotype G00174755, designated here; isolectotypes BM, G).
Ipomoea heterotricha forma dentata Chodat & Hassl. [as var. homotricha Chodat & Hassl. forma dentata], Bull. Herb. Boiss., ser. 2: 5: 694. 1905. (Chodat and Hassler 1905: 694). Type. PARAGUAY. [Cordillera], Piribebuy, *Hassler* 6708 (lectotype G00174756, designated here).
Ipomoea heterotricha forma subtriloba Chodat & Hassl. [as var. homotricha Chodat & Hassl. forma subtriloba], Bull. Herb. Boiss., ser. 2: 5: 694. 1905. (Chodat and Hassler 1905: 694). Type. PARAGUAY. [Canendiyú], Ipé Hú, *Hassler* 5073 (lectotype G00174927, designated here; isolectotypes BM, G, MPU, NY, P, UC).
Ipomoea heterotricha forma cordifolia Chodat & Hassl. [as var. homotricha Chodat & Hassl. forma cordifolia], Bull. Herb. Boiss., ser. 2: 5: 694. 1905. (Chodat and Hassler 1905: 694). Type. PARAGUAY. [San Pedro], Río Corrientes, *E. Hassler* 5872 (isotype BM).
Ipomoea bonariensis subsp. aspera Hassl., Repert. Spec. Nov. Regni Veg. 9: 153. 1911. (Hassler 1911: 153). Type. PARAGUAY. *E. Hassler* 6708 (lectotype G00174756, designated here).
Ipomoea bonariensis var. pubescens [as subsp. aspera Hassl. var. pubescens] Hassl., Repert. Spec. Nov. Regni Veg. 9: 154. 1911. (Hassler 1911: 154), nom. illeg. superfl. for I. heterotricha var. homotricha.
Ipomoea bonariensis forma trichosepala [as subsp. aspera Hassl. var. pubescens forma trichosepala] Hassl., Repert. Spec. Nov. Regni Veg. 9: 154. 1911. (Hassler 1911: 154), nom. illeg., based in part on Ipomoea heterotricha forma subtriloba Chodat & Hassl. and in part on Ipomoea bonariensis forma glabrior [as var. grandiflora forma glabrior] Chodat & Hassl. Type. PARAGUAY. *E. Hassler* 4800 (lectotype G00174931, designated here).
Ipomoea bonariensis var. tomentosa [as subsp. aspera Hassl. var. tomentosa] Hassl., Repert. Spec. Nov. Regni Veg. 9: 154. 1911. (Hassler 1911: 154). Type. PARAGUAY. San Estanislao, *E. Hassler* 4168 (lectotype G00174930, designated here).
Ipomoea bonariensis var. hispida [as subsp. aspera Hassl. var. hispida ] Hassl., Repert. Spec. Nov. Regni Veg. 9: 154. 1911. (Hassler 1911: 154). Type. PARAGUAY. *E. Hassler* 5073 lectotype G00174927, designated here; isolectotypes BM, G, MPU, NY, P, UC).
Ipomoea bonariensis forma subintegra [as subsp. aspera Hassl. var. hispida forma subintegra], Hassl., Repert. Spec. Nov. Regni Veg. 9: 154. 1911. (Hassler 1911: 154), nom. illeg. superfl. Type. Based partially on I. heterotricha forma suborbiculata.
Ipomoea bonariensis forma lobata [as subsp. aspera Hassl. var. hispida forma lobata Hassl.], Repert. Spec. Nov. Regni Veg. 9: 154. 1911 (1911: 154). Type. PARAGUAY. Caaguazú, Río Yhú, *E. Hassler* 9535 (lectotype G00174938, designated here; isolectotypes BM, G, K, P).

#### Type.

PARAGUAY. Amambay, *T. Rojas* 4051 (holotype LIL001245).

#### Description.

Decumbent or twining perennial, stems hispid with prominent, relatively long stellate hairs. Leaves petiolate, polymorphic, 3–11 × 2.5–10 cm, ovate to subreniform, entire, obscurely to deeply 3-lobed or lyrate-dentate with acute teeth, apex obtuse, base truncate to narrowly cordate, densely and roughly stellate hairy on both surfaces; petioles 1–5 cm, hispid-stellate-pilose. Inflorescence of 1–3-flowered, pedunculate axillary cymes; peduncles 3–12 cm, hispid-pilose; bracteoles 4 × 1 mm, lanceolate, obtuse, caducous; secondary peduncles 3–17 mm; pedicels short, 3–12 mm, glabrous or nearly so; sepals coriaceous, glabrous or thinly stellate hairy, 8–11 mm long, outer sepals elliptic, obtuse and mucronate, inner sepals obovate, obtuse with scarious margins; corolla 5–9 cm long, pink, funnel-shaped, glabrous, limb 4–5 cm diam., unlobed; ovary glabrous. Capsules and seeds not seen.

#### Distribution.

Nearly endemic to eastern Paraguay with a single record from a neighbouring area of Brazil.

**PARAGUAY. Amambay**: Pedro Juan Caballero, *Krapovickas et al.* 45909 (K, CTES); ibid., *A. Schinini et al.* 36021 (PY). **Caaguazú**: Yhú, *Jorgensen* 4858 (S, SI, US). **Canindeyú**: Yerbales de Maracayú, Río Carimbatay, *Hassler* 4539 (P). **Paraguarí**: P.N. Ybicu’í, *E. Zardini and R. Velazquez* 15895 (MO, PY). **San Pedro**: 5 km SW de San Estanislao, camino a Rosario, *Krapovickas et al.* 45822 (K, CTES).

**BRAZIL. Paraná**: *Cordeiro et al. 4701* (MBM).

#### Note.

Clearly related to *Ipomoea bonariensis*, this species was treated as a synonym of that species by Austin et al. (2015) but is readily distinguished by the relatively long (0.5–1.5 mm) arms of the branched hairs, Even more than *I. bonariensis*, it shows an extraordinary range of leaf shape, entire, merely dentate to laciniate or 3-lobed. It occurs in scattered locations in eastern Paraguay and neighbouring parts of Paraná State in Brazil.

### 164. Ipomoea oranensis

Taxon classificationPlantaeSolanalesConvolvulaceae

O’Donell, Lilloa 14: 185. 1948. (O’Donell 1948a: 185)

Ipomoea santacruzensis O’Donell, Arq. Mus. Paranaense, Curitiba 9: 237. 1952. (O’Donell 1952: 237). Type. BOLIVIA. Santa Cruz, Camiri, *M. Cardenas* 4258 (holotype LIL001282; isotype US).

#### Type.

ARGENTINA. Salta, Dept. Orán, San Andrés, *Pierotti* 280 (lectotype LIL001265, designated here).

#### Description.

Vigorous twining perennial, stems thinly to densely pubescent with stellate and simple hairs. Leaves petiolate, 4–15 × 2–9 cm, ovate-deltoid (rarely 3-lobed to half way), shortly acuminate and mucronate, base shallowly cordate to truncate and broadly cuneate onto the petiole, adaxially thinly pubescent, abaxially grey-tomentose; petioles 1–7 cm, tomentellous. Inflorescence of lax, axillary, often compound, pedunculate cymes; peduncles 3–11.5 cm, tomentose; bracteoles 5–10 mm, filiform, tomentose, caducous; secondary peduncles 1.5–2 cm; tertiary peduncles slightly shorter; pedicels 0.5–2 cm, pubescent to tomentose; sepals coriaceous, somewhat unequal, outer sepals 6–11 × 5–7 mm, elliptic, obtuse, convex, entirely glabrous or pubescent in the lower half only, margins scarious; inner sepals 7.5–12 mm, slightly broader and rounded; corolla 4.5–7 cm long, funnel-shaped, pink, glabrous, limb 4–5 cm diam., shallowly lobed; stamens and style included. Capsules 14–15 × 12–13 mm. suborbicular, glabrous; seeds 7 × 4 mm, long-pilose.

#### Illustration.

Figure 95.

**Figure 95.**
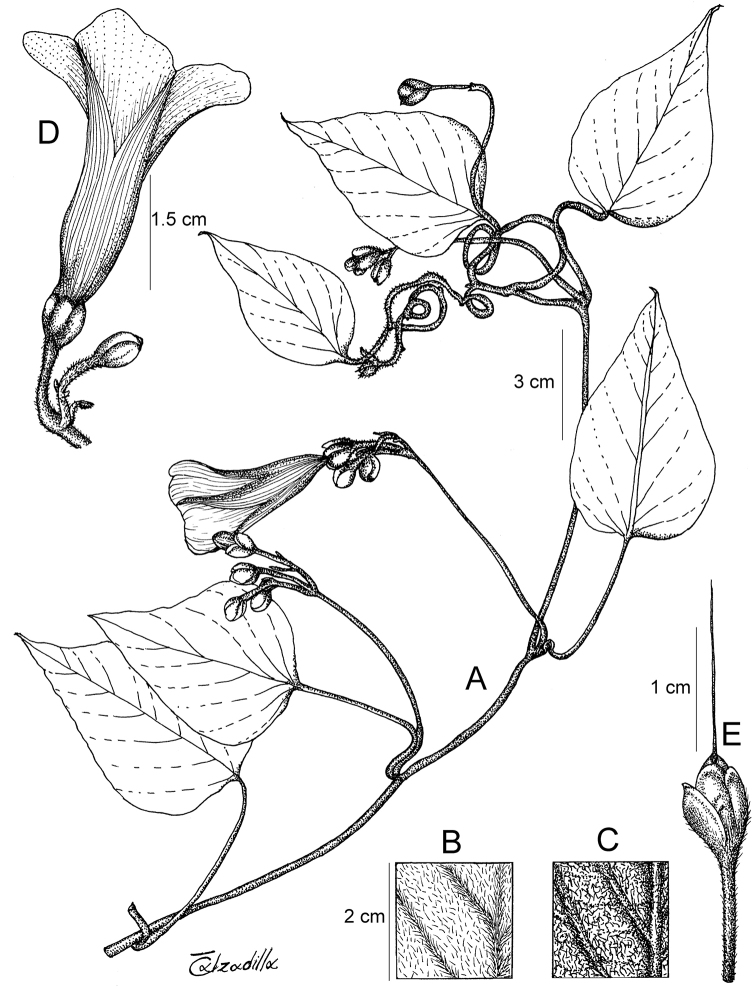
*Ipomoea oranensis*. **A** habit **B** adaxial leaf surface **C** abaxial leaf surface **D** corolla and calyx **E** calyx in fruit. Drawn by Eliana Calzadilla from *Wood et al.* 27639.

#### Distribution.

Serrano Chaqueño and Tucuman-Bolivian woodland between about 650 m and 2300 m extending along the eastern Andean slopes from just south of Santa Cruz through the Departments of Chuquisaca and Tarija to Orán in Argentina.

**ARGENTINA. Salta**: Only known from the type.

**BOLIVIA. Chuquisaca**: Azurduy, Tarvita, *M. Jiménez & J. Villalobos* 755 (MO, OXF); Boeto, below Nuevo Mundo, *J.R.I. Wood et al.* 20493 (BOLV, HSB. K. LPB); Siles, Serrania Los Milagros, *M. Serrano et al.* 6853 (OXF, MO); Tomina, Sopachuy, *J.R.I. Wood* 20458 (HSB, K, LPB); Zudañez, Mojocoya-Sacha Pampa, *J.R.I. Wood & M. Serrano* 13356 (HSB, K, LPB). **Santa Cruz**: Cordillera, Charagua, *I. Vargas* 474 (NY, USZ); Ibañez, Los Espejillos, *G.A. Parada et al.* 124 (OXF, MO); Vallegrande, G. *A. Parada et al.* 3191 (USZ). **Tarija**: O’Connor, Entre Ríos, *M. Mendoza et al.* 2860 (K, USZ).

#### Note.

Both indumentum and leaf shape vary considerably in this species as in the related *Ipomoea bonariensis*. Leaves are usually entire but 3-lobed forms occur occasionally.

*I. oranensis* has stellate hairs but they are never obvious as in *I. bonariensis* and are often difficult to find, most hairs being simple. *Ipomoea oranensis* is very similar to *I. exserta* and the two species are almost indistinguishable when not in flower. However, the funnel-shaped corolla of *I. oranensis* is totally different from the hypocrateriform corolla of *I. exserta* with its exserted stamens.

### 165. Ipomoea exserta

Taxon classificationPlantaeSolanalesConvolvulaceae

J.R.I. Wood & Scotland, Kew. Bull. 50 (31): 82. 2015. (Wood et al. 2015: 82)

#### Type.

BOLIVIA. Chuquisaca, Prov. Luis Calvo, 14 km E of Monteagudo, on pass before descent to Timboy Pampa *J.R.I. Wood* 9693 (holotype HSB, isotypes K, LPB).

#### Description.

Liana to 6 m; stems sometimes pendulous from overhanging branches, woody below, somewhat wiry above, thinly pilose, glabrescent when old, sometimes leafless when flowering. Leaves petiolate, 6–14 × 5–10 cm, mostly ovate, sometimes suborbicular, occasionally shallowly 3-lobed or with a single lateral tooth, base cordate to subtruncate with rounded auricles, apex acute, margin entire to obscurely undulate, adaxially dark green, appressed pubescent with long hairs, abaxially grey-matted-tomentose with some stellate hairs mixed with unbranched hairs; petiole relatively short, 3–4 cm, pilose. Inflorescence of compact many-flowered, axillary cymes, often subracemose in form; peduncle 5–11 cm, commonly twisted, shortly pilose; bracteoles oblong, caducous; secondary peduncles 6–10 mm, glabrous; pedicels 2–10 mm, glabrous; sepals subequal, 9–11 × 4–6 mm obovate-elliptic, coriaceous, convex, glabrous, margins narrow, scarious; outer sepals minutely mucronate; inner sepals slightly wider than the outer, rounded; corolla 5–5.8 cm long, completely glabrous, hypocrateriform, the basal subcylindrical tube 3.5–3.8 × 0.5–0.9 cm, brownish, the limb 1.5–2 cm long, spreading, lobed, dark pink; stamens and style exserted 5–8 mm. Capsules and seeds not seen.

#### Illustration.

Figure 96.

**Figure 96.**
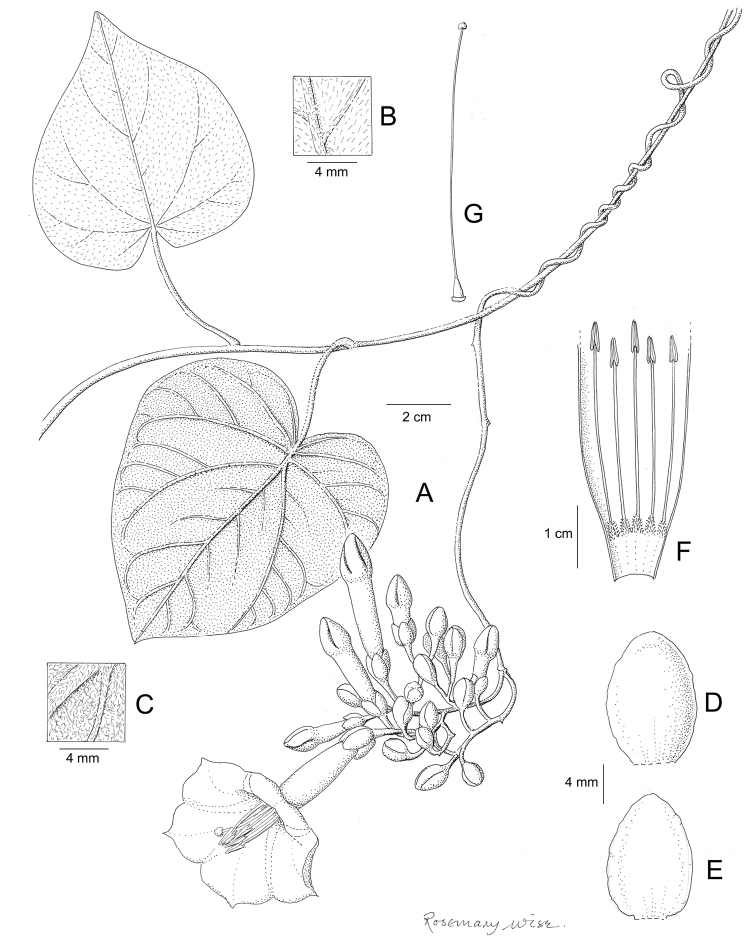
*Ipomoea exserta*. **A** habit **B** adaxial leaf surface **C** abaxial leaf surface **D** outer sepal **E** inner sepal **F** corolla opened out to show stamens **G** ovary and style. Drawn by Rosemary Wise from *Wood* 16093.

#### Distribution.

Endemic to Bolivia in Bosque Serrano Chaqueño between 400 and 1400 m. from the Santa Cruz area south to the Villamontes area.

**BOLIVIA. Chuquisaca**: Boeto, Pampa del Tigre, *J.R.I. Wood et al.* 20543 (K, LPB); Luis Calvo, Monteagudo-Timboy Pampa, *J. Gutiérrez et al.* 320 (MO, USZ); Incahuasi, *J.R.I. Wood et al.* 27643 (K, LPB, USZ); Siles, Río Azero, *J.R.I. Wood* 13308 (K, LPB). **Santa Cruz**: Cordillera, Tatarenda, *J.R.I. Wood et al.* 16093 (K, LPB, USZ); Florida, Bella Vista, *L. Arroyo et al.* 2868 (USZ); Ibáñez, La Angostura, *M. Mendoza & Eduardo* 987 (K, USZ). **Tarija**: Gran Chaco, Río Pilcomayo gorge, *J.R.I. Wood et al.* 27595 (K, LPB, USZ); O’Connor, Palos Blancos towards Entre Ríos, *J.R.I. Wood et al.* 28042 (LPB, OXF, USZ).

#### Notes.

Similar to *Ipomoea oranensis* but immediately distinguished by the hypocratiform corolla with a cylindrical tube and exserted genitalia.

Both *Ipomoea exserta* and *I. oranensis* have some stellate hairs mixed with unbranched hairs but these are often difficult to see. The two species may sometimes grow together but *I. oranensis* is found most commonly at higher altitudes, from 1600 to 2200 m, although there are a few records from as low as 650 m.

### 166. Ipomoea asplundii

Taxon classificationPlantaeSolanalesConvolvulaceae

O’Donell, Arq. Mus. Paranaense 9: 211. 1952. (O’Donell 1952: 211)

#### Type.

BRAZIL. Mato Grosso, Santa Cruz da Barra, banks of Río Paraguay, *C.A.M. Lindman* 3197 (holotype S07-43711, isotype S09-37459).

#### Description.

Vigorous twining perennial of unknown height but reaching at least 50 cm, stems pubescent. Leaves petiolate, 2–7 × 1.3–5.5 cm, ovate-cordate (sometimes shallowly 3-lobed or repand), obtuse and strongly mucronate, base subcordate or truncate and briefly cuneate onto the petiole, adaxially green-tomentose with long hairs, abaxially densely silvery-sericeous-tomentose with dense long whitish hairs; petioles 6–22 mm, pubescent, the base often with filiform pseudo-stipules. Inflorescence of lax, axillary, pedunculate cymes, these sometimes paired; peduncles 0.7–8 cm, pubescent; bracteoles 3–11 mm, linear-filiform, pubescent, tardily deciduous; secondary and tertiary peduncles (if present) 7–23 mm; pedicels 0.3–1.2 cm, pubescent; sepals subequal, coriaceous, convex, outer 6–8 × 3–4 mm, elliptic, acute or obtuse, thinly pilose; inner sepals c. 1 mm longer and wider, obovate-elliptic, broader and rounded, glabrous; corolla 4–6 cm long, funnel-shaped, pink, glabrous, limb 2.5–4 cm diam., unlobed. Capsules 7 × 5 mm, ellipsoid, glabrous, slender, persistent style 2–2.5 mm; seeds (immature) oblong, 5 mm, angles long pilose with dirty white hairs.

#### Illustration.

Figure 97.

**Figure 97.**
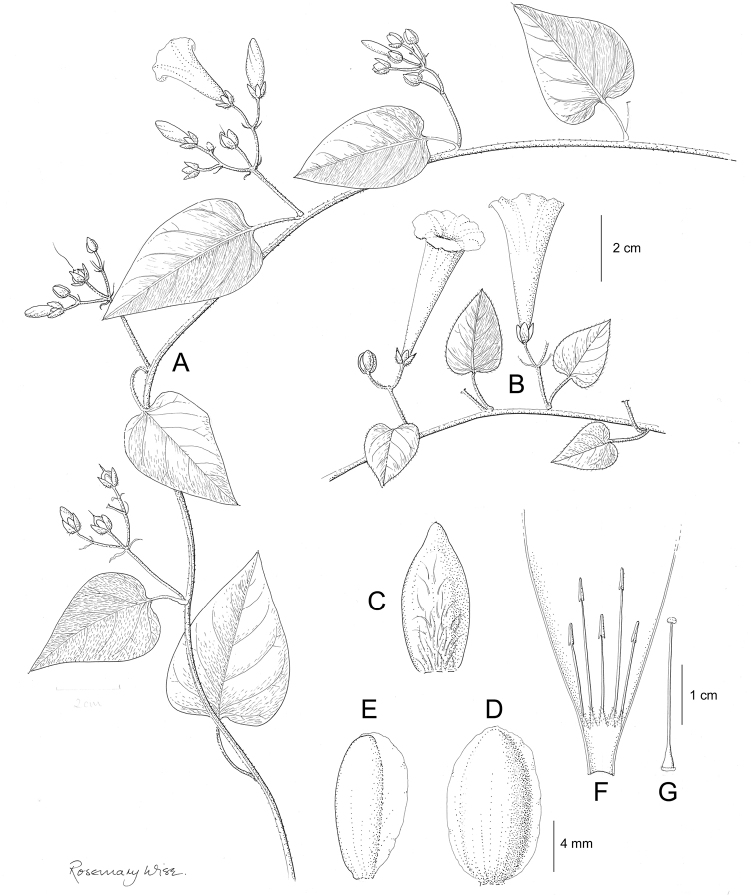
*Ipomoea asplundii*. **A** habit **B** habit **C** outer sepal **D** middle sepal **E** inner sepal **F** corolla opened out to show stamens **G** ovary and style. Drawn by Rosemary Wise **A** from *Plowman et al.* 8128; **B–G** from *Secco et al*. 442.

#### Distribution.

Endemic to Amazonian Brazil, growing in cerrado and on rocks in forest: **BRAZIL.** Sine loc., *W.J. Burchell* 858A (K). **Mato Grosso**: Novo Mundo, P. Est. do Cristalino, *G. Henicka et al.* 25 (K); Alta Floresta, P. Est. do Cristolino, *D. Sasaki et al.* 1355 (K); Santa Cruz do Xingu, *D.C. Zappi et al.* 3062 (K, RB). **Tocantins**: Mun. Pres. Kennedy, Faz. Primavera, *T. Plowman et al.* 8128 (FTG, MG); P.N. do Araguaia, Ilha da Bananal, *R.C. Mendonça et al.* 3951 (IBGE, OXF, US). **Pará**: Araguaia, 20 km W of Redenção, *T. Plowman et al*. 8625 (FTG, MG); Marabá, Serra Norte de Carajas, *R.S. Secco et al*. 442 (MG, MO). Also recorded for Goiás in Flora do Brasil 2020 under construction.

#### Note.

Rather distinct because of its densely pubescent indumentum with long weakly appressed hairs, persistent linear bracteoles and generally compact inflorescence. The type is atypical in the sense that it has a relatively long, compound inflorescence.

### 167. Ipomoea argentea

Taxon classificationPlantaeSolanalesConvolvulaceae

Meisn. in Martius et al., Fl. Brasil. 7: 247. 1869. (Meisner 1869: 247)

Ipomoea villosa var. argentea (Meisn.) Hallier f., Jahrb. Hamb. Wiss. Anst. 16: 53. 1899. (Hallier 1899a: 53). Type. Based on Ipomoea argentea Meisn.
Batatas villosa Choisy in A.P. de Candolle, Prodr. 9: 337. 1845. (Choisy 1845: 337). Type. BRAZIL. São Paulo, Ytu, *Martius* 609 (lectotype M0184921, designated here).
Ipomoea villosa (Choisy) Meisn. in Martius et al., Fl. Brasil. 7: 244. 1869. (Meisner 1869: 244), nom. illeg., non Ipomoea villosa Ruiz & Pav. (1799).
Exogonium villosum (Choisy) Peter, Die Natürlichen Pflanzenfamilien 4 (3a): 28. 1897 [pub. 1891]. (Peter 1891: 28).
Ipomoea comosa House, Ann. New York Acad. Sci. 18: 201. 1908. (House 1908b: 201). Type. Based on Ipomoea villosa (Choisy) Meisn. (House 1908b: 201).
Ipomoea stachyoides Meisn. in Martius et al., Fl. Brasil. 7: 240. 1869. (Meisner 1869: 240). Type BRAZIL. Goiás, *W.J. Burchell* 6586 (holotype BR00005792245, isotypes K, P).
Ipomoea hypoleuca Taub., Bot. Jahrb. 21: 449.1895. (Taubert 1895: 449). Type. BRAZIL. Goiás, Serra Dourada, *E. Ule* 3013 (holotype B†, isotype HBG506563).
Ipomoea argentea var. hypoleuca (Taub.) Hallier f., Jahrb. Hamburg. Wiss. Anst. 16(3): 19. 1899. (Hallier 1899a: 53).

#### Type.

BRAZIL. Goyas et Piauhy: *G. Gardner* 3356 (lectotype BR0000005837519, designated by Wood et al. 2015: 74, isolectotype K).

#### Description.

Erect perennial, stem stout and somewhat woody, often simple, white-tomentose, 0.3–1 m high. Leaves subsessile, 2.5–10(–14) × 2–3.5(–5.5) cm. broadly oblong to oblong-elliptic, acute and sometimes mucronate, cuneate to rounded at base, densely sericeous or tomentose on both surfaces, adaxially greenish, abaxially grey; petioles 0–5 mm. Inflorescence terminal, formed of sessile or shortly pedunculate compact cymes from the upper leaf axils, cymes commonly single-flowered but sometimes with 2–10 flowers; peduncles 0–2.5 cm, tomentose; bracteoles linear-lanceolate to ovate, up to 10 × 5 mm, hirsute, somewhat persistent; pedicels 1–3 mm; sepals subequal, 7–9 mm, elliptic, obtuse, coriaceous, convex, brown when dry, the outer villous but glabrescent, inner glabrous; corolla 5–6 cm long, funnel-shaped, pink, glabrous, limb c. 4 cm diam., distinctly lobed. Capsules glabrous, ellipsoid, 8 × 6 mm, very shortly rostrate; seeds c. 3 mm, long-pilose.

#### Illustration.

**Figure 98.**
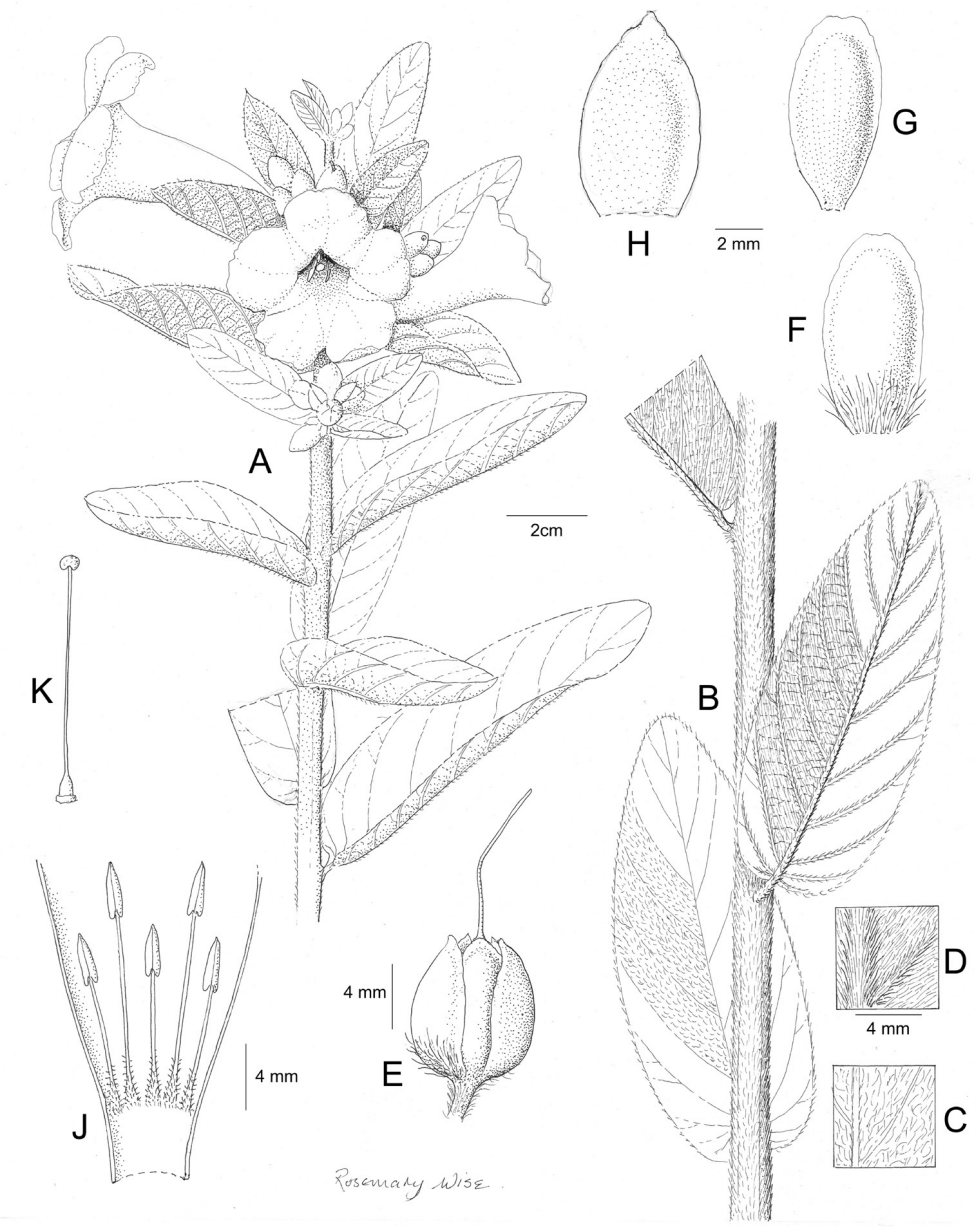
*Ipomoea argentea*. **A** habit **B** stem and leaves **C** adaxial leaf surface **D** abaxial leaf surface **E** calyx **F** outer sepal **G** middle sepal **H** inner sepal **J** corolla opened out to show stamens **K** ovary and style. Drawn by Rosemary Wise **A, E–K** from *Wood et al.* 25639; **B–D** from *Ratter et al.* 2830.

Austin (1998: 401); Figures 6C, 8A, 98.

#### Distribution.

A characteristic species of cerrado in Brazil extending to Paraguay, Brazil and to the llanos of Venezuela and Colombia.

**PARAGUAY. Amambay**: *Rojas in Hassler* 10055 (BM, K).

**BRAZIL. Dist. Fed.**: Rio São Bartolomeu, *E.P. Heringer et al.* 6100 (IBGE, K); Burração, *G. Kirkbride* 3967 (K). **Goiás**: *E.P. Heringer* 10859 (UB); Chapada da Veadeiros, *J.R. Pirani et al.* 1827 (MO, USP, K); *G. Gardner* 3908 (K); summit of Cerro Dourada, 20 km SE of Goias Velho, *H.S. Irwin et al.* 11728 (FTG, NY) – var. hypoleuca; Rio dos Couros, *A.F.M. Glaziou* 21786a (P) – var. hypoleuca. **Mato Grosso**: Novo Mundo, Parque Est. Cristalino, *D. Sasaki et al.* 2125 (K); Rio Brilhante, *G. Hatschbach* 26119 (MBM, RB). **Minas Gerais**: Campina Verde, *A. Macedo* 249 (BM); Patrocínio, Morro da Pedras, *H.S. Irwin et al.* 25462 (NY), Belo Horizonte, *F.C. Hoehne* 3064 (F, SP); Caldas, *J.F.Widgren* in *A.F. Regnell* 225 (K, S); *A.F. Regnell* III 193 (S). **Paraná**: *P. Dusen* 14932 (S), 16383 (S); Jaguariaiva, *G. Hatschbach* 52795 (MBM). **São Paulo**: *Rawitscher* s.n. [1/3/1945] (SPF, K); *A.C. Brade* 5567(S). Mato Grosso do Sul fide Flora do Brasil (2020).

**BOLIVIA. Santa Cruz**: Ángel Sandoval, Santo Corazón, Cerro Pelón, *J.R.I. Wood et al.* 25639 (USZ). Velasco, P.N. Noel Kempff Mercado, *R. Guillén & T. Centurion* 845 (ARIZ, BOLV, F, FTG, MO, NY, USZ); *J.R.I. Wood et al.* 25244 (K, LPB, UB, USZ).

**COLOMBIA. Caquetá**: *J.C. Betancour* 1928 (COL). **Casanare**: . *Saravia* 2705 (COL). **Meta**: *L. Uribe* 1346 (COL). **Vaupés**: *J.M. Idrobo* 9460 (COL).

**VENEZUELA. Amazonas**: *J. Steyermark et al*. 131513 (MO); *Wessels Boer* 1925 (NY); **Bolívar**: *J. Steyermark et al*. 131713 (MO). **Orinoco**: Maypures, *Spruce* 3605 (BM, K).

#### Note.

A very distinct species because of its erect habit, subsessile silvery leaves and lobed corolla.

#### Variation.

Despite its distinctiveness this species is quite variable and this is reflected in our molecular results which suggest that it is not monophyletic although it is difficult in the present state of our knowledge to reconcile molecular results with morphology. The majority of the specimens including the lectotype have leaves dull green adaxially and grey abaxially (Wood et al; 2015: 73 (Figure 22B). However, some specimens have more lustrous sericeous leaves similar to the original syntype *Spruce* 3605 (Wood et al. 2015: 73, figure 22A). Further investigation is required to assess whether these forms are of taxonomic significance. Var. hypoleuca is especially distinct and is often recognised, sometimes as a distinct species. It is an erect herb with leaves 3–5 × 1.2–3 cm, ovate, adaxially green, shortly tomentose but abaxially densely white-tomentellous. It is restricted to Goiás in the Brazilian planalto.

### 168. Ipomoea paulistana

Taxon classificationPlantaeSolanalesConvolvulaceae

(Silva Manso) Stellfeld, Tribuna Farm., Curitiba 13: 86. 1945. (Stellfeld 1945: 86)

Convolvulus paulistanus Silva Manso, Enum. Subst. Braz. 17. 1836. (Manso 1836: 17).
Ipomoea echioides Choisy, Mém. Soc. Phys. Genève 8(1): 54 [132]. 1838. (Choisy 1838: 54 [132]). Type. BRAZIL. Mato Grosso, Serra-Nov., Silva Manso & Lhotsky 32 (syntype G 00135526).
Ipomoea echioides var. villosula Meisn. in Martius et al., Fl. Brasil. 7: 244. 1869. (Meisner 1869: 244). Type. BRAZIL. Mato Grosso, Cuaibá, L. Riedel (NY00319184, lectotype, designated here).
Ipomoea rondoniae Hoehne, Anexos Mem. Inst. Butantan, Bot. 1, fasc. 6: 68, pl. 14. 1922. (Hoehne 1922: 68). Type. BRAZIL. Mato Grosso (extreme NE), nas márgenes do Cautária Grande, Pouso Primeiro de Fevereiro na Rondônia, Kuhlmann 2265 (holotype SP000579?, isotype R).
Ipomoea rondoniae var. breviracemosa Hoehne, Anexos Mem. Inst. Butantan, Bot. 1, fasc. 6: 69, pl. 15. 1922.(Hoehne 1922: 69). Type. BRAZIL. Mato Grosso, Estrada ao Diamantina, perto de Cuaibá, Kuhlmann 2269 (holotype SP?, isotype R).

#### Type.

BRAZIL. Mato Grosso, Cuyaba, *Silva Manso & Lhotsky* 32 (G00135526, lectotype, designated by Wood et al. 2015: 74).

#### Description.

Erect herb to c. 0.75 cm, usually unbranched, stems pubescent, often leafless below, rootstock an elongate tuber. Leaves sessile, numerous, imbricate, 0.5–6(–13) × 0.1–0.5(–1.2) cm, diminishing in size upwards, linear-oblong, base narrowly cuneate, apex acute, mucronate, margins commonly inrolled, both surfaces densely softly pilose or pubescent. Inflorescence terminal, ±racemose in appearance, up to 30 cm long, formed of cymes, which are often reduced to solitary flowers arising in the axils of the leaf-like bracts; peduncles 0–3 cm (often absent), erect; bracteoles 3–10 mm, linear, pilose, deciduous; pedicels 2–6 mm; sepals 5–7 mm, subequal, elliptic, usually truncate, rigid, convex, the outer pubescent, acute, the inner subglabrous; corolla 5–6 cm long, pink, funnel-shaped, glabrous, limb 3–4 cm diam., weakly lobed. Capsules globose, 4–5 mm diam., glabrous, apex shortly rostrate, the dead style remaining till the fruit matures; seeds c. 3 mm, long-pilose.

#### Distribution.

A characteristic cerrado species of Bolivia and Brazil found from around 200 to 900 m.

**BRAZIL Goiás**: Mun. Corumbá, *A. Macedo 4476* (K); Colinas, *A.A. Arbo et al. 3679* (K, CTES); Chapada de Veadeiros, *J.R. Pirani* et al. 1828 (SPF, K). **Maranhão**: Carolina, *M.F. da Silva* 1090 (NY). **Mato Grosso**: 270 km N. of Xavantina, *D.R. Gifford* 98 (K); *Philcox & Ferreira* 4403 (K), 4530, (K), *J. Ratter et al.* 857 (K); Cuiabá, *Malme* 1288 (S); Río Yocuara, *C.A.M. Lindman* 3113 (S). **Minas Gerais**: Morro do Cachorro, *A. Krapovickas & C. Cristóbal* 33460 (CTES). **Tocantins**: *R.D. Reeves* 2890 (CEN); Guaraí, *H. S, Irwin* 21518 (NY). Also Mato Grosso do Sul, fide Flora do Brasil (2020).

**BOLIVIA. Beni**: Vaca Díaz, Pampas de San Lorenzo, *P. Maas et al.* 8722 (NY, MO). **Santa Cruz**: Serranía de Santiago de Chiquitos, *J.R.I. Wood & D. J. Goyder* 16968 (K, LPB, USZ); *J.R.I. Wood* 18824 (K, LPB); Velasco, P.N. Noel Kempff Mercado, *L. Sánchez et al.* 253 (ARIZ, FTG, MO); *J.R.I. Wood et al.* 18213 (K, LPB); Hac. Acuario, *R. Guillén et al.* 309 (ARIZ, FTG, OXF, MO).

#### Typification.

In designating a lectotype of Ipomoea echioides var. villosula, we have chosen the NY specimen as it appears to have a label in Meisner’s handwriting annotated as “β villosula nob.”.

#### Note.

Very distinctive because of its imbricate leaves which diminish in size upwards. It is occasionally confused with *Ipomoea argentea* but the leaves are never silvery-tomentose as in that species.

### 169. Ipomoea schomburgkii

Taxon classificationPlantaeSolanalesConvolvulaceae

Choisy in A.P. de Candolle, Prodr. 9: 354. 1845. (Choisy 1845: 354)

Ipomoea graminiformis Meisn. in Martius et al., Fl. Brasil. 7: 250. 1869. (Meisner 1869: 250). Type. BRAZIL. Goiás, *W.J. Burchell* 8556 (holotype BR00005305780, isotype K).

#### Type.

GUYANA. *R. Schomburgk* 692 (holotype K000612791, isotype BM).

#### Description.

Glabrous perennial herb with xylopodium and erect, somewhat succulent stems to 50 cm. Leaves sessile, 4–16 × 0.1–0.3 cm, linear, somewhat glaucous, tapering at both ends, acute. Inflorescence ± terminal, up to 30 cm long, but usually much less, formed of pedunculate cymes from the uppermost leaf axils; peduncles 0–5 cm, diminishing in length upwards; bracteoles filiform, up to 10 mm long, caducous; pedicels 5–13 mm; sepals subequal, coriaceous, somewhat convex, 5–7 × 3–4 mm, elliptic, outer acute to obtuse, c. 1 mm shorter than inner, inner rounded, slightly scarious on margins; corolla c. 4.5 cm long, pink with a darker centre, glabrous, limb weakly lobed, c. 4 cm diam. Capsules and seeds not seen.

#### Illustration.

**Figure 99.**
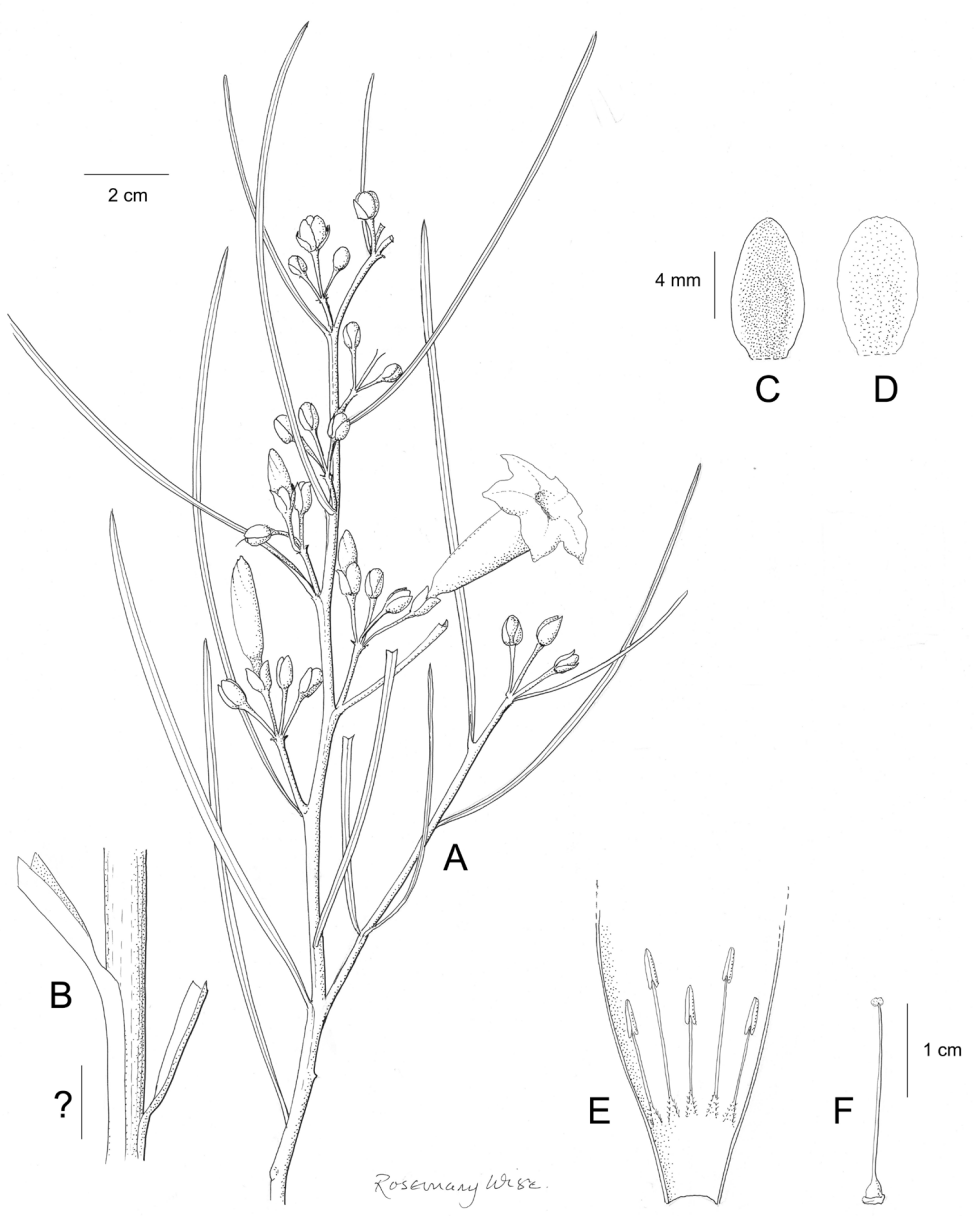
*Ipomoea schomburgkii*. **A** habit **B** portion of stem **C** outer sepal **D** inner sepal **E** corolla opened out to show stamens **F** ovary and style. Drawn by Rosemary Wise from *Wood & Soto* 27914.

Figure 99; Austin (1998: 401).

#### Distribution.

Scattered on seasonally flooded plain at low altitudes in South America from the Guianas, Venezuela and Colombia south to Bolivia. Most common in the llanos region of eastern Colombia, southern Venezuela and the Guianas. Rare elsewhere. Records from Paraguay are errors.

**BRAZIL. Goiás.** Type of *Ipomoea graminiformis*. **Mato Grosso**: *C.A.M. Lindman* 3115 (S); São José do Xingu, *D.C. Zappi et al.* 3229 (K, RB); Parque Estadual Cristalino, *J.H. Piva* 999 (K). **Pará**: Parque Indigena do Tumucumaque, *P. Cavalcante* 2520 (K). **Rondônia**: Vilhena, *M.G. da Silva* 4654 (INPA). Also Mato Grosso do Sul and Tocantins fide Flora do Brasil (2020).

**GUYANA.***Jansen-Jacobs et al.* 4608 (K).

**SURINAM.***Rombouts* 386 (K).

**BOLIVIA. La Paz**: Iturralde, Luisita, *S.G. Beck & R. Haase* 10100 (LPB); *R. Haase* 801 (LPB), 840 (LPB). **Santa Cruz**: Velasco, El Refugio, *R. Guillén & V. Roca* 2992 (ARIZ, LPB, MO, OXF, USZ); *J.R.I. Wood & D. Soto* 27914 (OXF, K, LPB, USZ).

**COLOMBIA. Arauca**: Laguna La Venera, *J.P. Jørgensen* 62 (COL). **Guainía**: La serranía, Manacasias, *J. Cuatrecasas* 7821 (COL). **Guaviare**: Barrancon, *N.C. Garzón* 0026 (COL). **Meta**: *Lehmann* 8796 (K). **Vichada**: San José de Ocune, *O. Haught* 2799 (US); Maypures, *Spruce* 3810 (K).

**VENEZUELA. Amazonas**: Puerto Ayacucho, *G. Davidse & O. Huber* 14953 (MO). **Apure**: *G. Aymard* 5649 (MO). **Bolívar**: *J. Steyermark et al.* 131362 (MO). **Guárico**: *K.R. Robertson & D.F. Austin* 178 (MO). **Monagas**: *L. Aristeguieti* 3912 (MO).

#### Note.

Readily distinguished by the herbaceous, slightly fleshy stems, linear leaves, subequal sepals, glabrous corolla and distinctive habit and habitat.

### 170. Ipomoea densibracteata

Taxon classificationPlantaeSolanalesConvolvulaceae

O’Donell, Lilloa 23: 438. 1950. (O’Donell 1950a: 438)

#### Type.

BOLIVIA. Santa Cruz, Prov. Cordillera, Cabezas, *I. Paredo* 453 (holotype LIL001236).

#### Description.

Vigorous climber or liana to 4 m; stems stout, densely pubescent. Leaves petiolate, 3–9 × 2–8 cm, ovate, obtuse and mucronate, margin undulate, cordate and cuneate onto the petiole, adaxially green, densely pubescent, abaxially grey-subtomentose; petioles 1–3 cm, subtomentose. Inflorescence of solitary bracteate flowers aggregated into dense cymes or racemes; bracts resembling small leaves; peduncles 1–3.5 cm, densely pilose to tomentose; bracteoles foliose, 1.2–2.5 × 0.5–0.8 cm, oblong-elliptic, obtuse, narrowed to a cuneate base, persistent; pedicels 0–5 mm; sepals hidden by bracteoles, subequal, 8–9 × 4–6 mm, elliptic, obtuse, coriaceous, convex, somewhat pubescent when young, glabrescent and completely glabrous when in fruit; corolla 5–8 cm long, funnel-shaped, pink with a darker centre, glabrous, limb 2.5–4 cm diam., undulate. Capsules enclosed by persistent bracts, 7–8 × 5 mm, glabrous, ovoid, rostrate; seeds 5 mm, oblong, long-pilose.

#### Illustration.

Figure 100.

**Figure 100.**
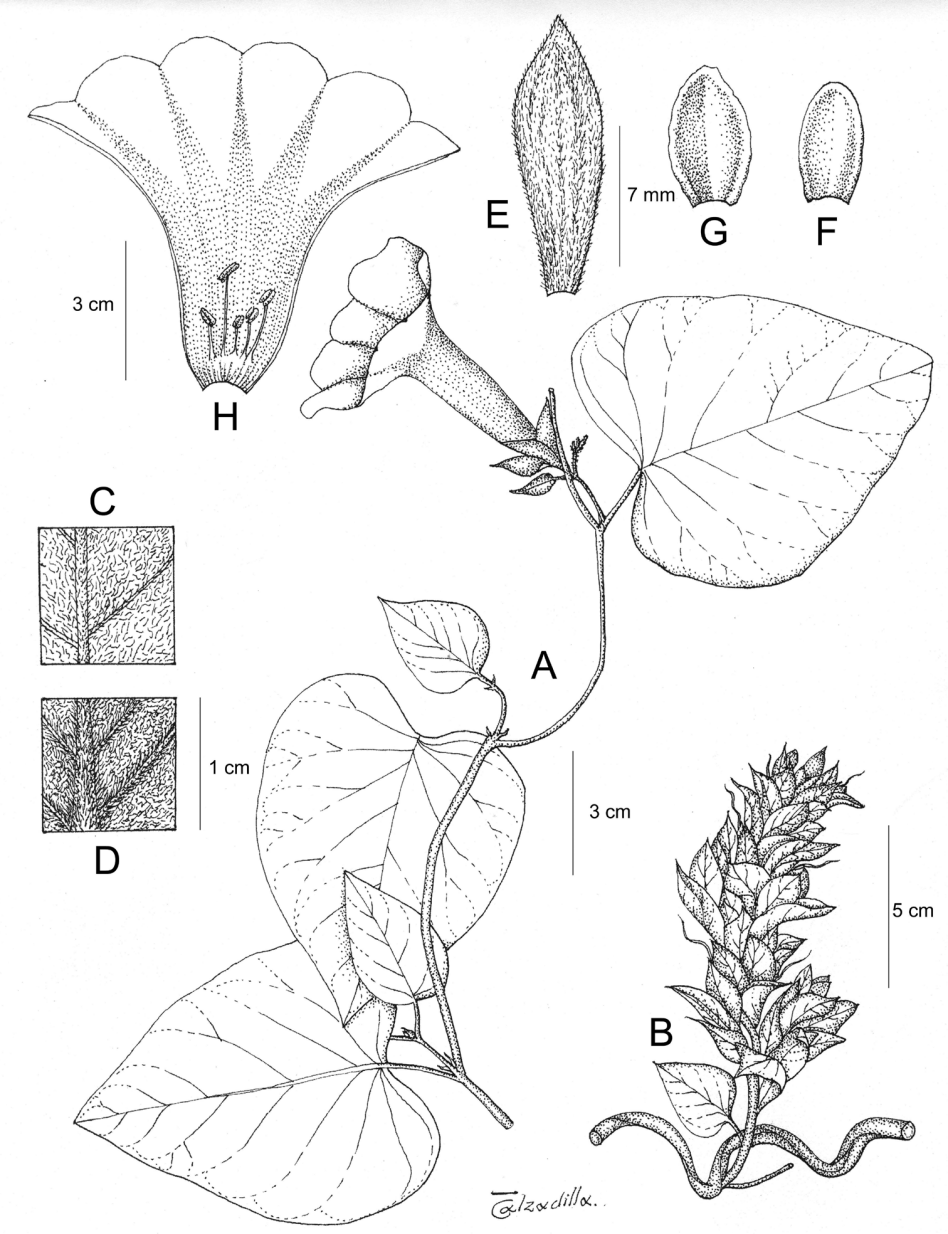
*Ipomoea densibracteata*. **A** habit with poorly developed inflorescence **B** well developed inflorescence **C** adaxial leaf surface **D** abaxial leaf surface **E** bracteole **F** outer sepal **G** inner sepal **H** corolla opened up to show stamens. Drawn by Eliana Calzadilla from *Wood et al.* 28016.

#### Distribution.

An uncommon Bolivian endemic growing in scattered populations below 500 m in scrub and on forest margins around the northern and western fringes of the Chaco.

**BOLIVIA. Santa Cruz**: Chiquitos, El Tinto–Laguna Concepción, *J.R.I. Wood & D. Soto* 27118 (K, LPB, USZ); Cordillera, San José to Tucavaca, *Solis Neffa et al.* 1846 (CTES, LPB); *A. Fuentes* 1421 (ARIZ, CPAP, USZ); Ñuflo de Chávez, Concepción, *J.R.I. Wood & D. Soto* 27919 (OXF, K, LPB, USZ); Velasco, San Ignacio to San Miguel, *J.R.I. Wood et al.* 13134 (K, LPB, USZ).

#### Note.

A very distinctive species because of its persistent foliose bracteoles combined with the coriaceous, convex sepals.

### 171. Ipomoea verruculosa

Taxon classificationPlantaeSolanalesConvolvulaceae

(Pittier) O’Donell, Lilloa 26: 397. 1953. (O’Donell 1953a: 397)

Exogonium verruculosum Pittier, J. Washington Acad. Sci. 21: 142. 1931. (Pittier 1931: 142). Type. VENEZUELA. Aragua, zona xerofítica de Chuau, *H. Pittier* 12118 (holotype VEN, not seen, isotypes G, LIL, NY, US).
Ipomoea avicola D.F. Austin, Fl. Venezuela 8: 143. 1982. (Austin 1982b: 143), nom. superfl. based on Exogonium verruculosum Pittier

#### Type.

Based on *Exogonium verruculosum* Pittier

#### Description.

Twining subshrub to 1.5 m; stems glabrous, woody when old, strongly warted. Leaves petiolate, dimorphic, 2.5–4 × 2–3 cm, ovate cordate or, more commonly deeply 3–5-lobed with apical lobe elliptic, much larger than the laterals, apex obtuse, lobes contracted at base, both surfaces glabrous; petioles 1–2.2 cm. Inflorescence of axillary pedunculate cymes; peduncles 0.5–2.5 cm, glabrous, slightly warted; bracteoles 1.5–2 mm, ovate, obtuse, caducous; secondary peduncles 5–7 mm; pedicels relatively slender, 7–10 mm; sepals subequal, 5–7 mm long, oblong-elliptic, obtuse, glabrous, the inner ones with a rounded scarious apex; corolla 3–4.5 cm long, glabrous, hypocrateriform, the tube 2.5–3.3 cm, pale, limb 0.7–0.9 cm in diam., lobed, lobes deep red, 4–6 mm long, ovate, often reflexed; stamens exserted. Capsules ovoid, glabrous, c. 10 mm long; seeds 5 × 2.5 mm, pilose with long marginal hairs to 8 mm.

#### Distribution.

Endemic to NW Venezuela where it grows in xerophitic scrub up to 400 m, but usually near the coast.

**VENEZUELA. Aragua**: Distrito Giradot, Carretera Cata–Cuyagua, *V.M. Badillo* 4809 (FTG). **Carabobo**: Puerto Cabello, *E. André* s.n. (K); 5 km E of Puerto Cabello, *W.J. Hahn et al.* 5089 (FTG, MO, US); cumbre Gañango–Patanemo, *B. Trujillo* 8817 (FTG). **Dist. Fed.**: Mun. Vargas, Catia La Mar, *N. Ramírez* 2665 (FTG); Libertador, *J. Steyermark* 112744 (FTG, MO); Arrecife, *L. Aristeguieta* 4533 (VEN).

#### Note.

The approximate position of this species is inferred from its morphology.

### 172. Ipomoea discolor

Taxon classificationPlantaeSolanalesConvolvulaceae

(Kunth) G. Don, Gen. Hist. 4: 270. 1838. (Don 1838: 270)

Convolvulus discolor Kunth, Nov. Gen. Sp. 3: 105 (1818 [pub. 1819]. (Kunth 1819: 105), *nom. cons*. Type. VENEZUELA. Amazonas, Carichana, Humboldt & Bonpland 1045 (holotype P00670759).
Ipomoea irengana N.E. Br., Trans. Linn. Soc. London, Bot. ser. 2, 6: 51. 1901. (Brown, NE 1901: 51). Type. GUYANA. Ireng Valley, *McConnell & Quelch, 265* (lectotype K000612822, designated here).

#### Type.

Based on *Convolvulus discolor* Kunth

#### Description.

Slender twining herb, stems pubescent. Leaves petiolate, very small, 1.2–4 × 1.2–2.3 cm, ovate, obtuse and mucronate, shallowly cordate with rounded auricles, adaxially green, tomentellous, abaxially white-canescent. Flowers solitary, axillary; peduncles 4–25 mm, pubescent; bracteoles filiform, c. 1 mm, caducous; pedicels 6–15 mm, pubescent; sepals subequal, 7–11 mm long, oblong-lanceolate, acute or obtuse, outer pubescent, inner glabrous, scarious, more rounded; corolla 3.5–5.5 cm long, pale pink, funnel–shaped, thinly pubescent, limb undulate, 3.5–4 cm diam.

#### Illustration.

Austin (1998: 399).

#### Distribution.

Dry rocky hills at low altitudes in Venezuela and Guyana; apparently uncommon.

**GUYANA.** Type of *Ipomoea irengana*.

**VENEZUELA. Bolívar**: Cedeño District, *B. Boom & Grillo* 6399 (FTG, NY); Cerro Gavilan, *J. Wurdack & J.V. Monachino* 40901 (FTG, NY); Agua Amena, *J. Steyermark et al.* 131440 (FTG, MO); 1 km S of Quebrada la Flore, *J. Steyermark et al.* 131637 (FTG). **Lara**: between Quibor and Cubiro, *D.F. & S. Austin* 6019 (FTG).

#### Note.

This species is anomalous in this clade because of the thinly pubescent corolla.

### 173. Ipomoea aurantiaca

Taxon classificationPlantaeSolanalesConvolvulaceae

L.O. Williams, Fieldiana Bot. 32: 187. 1970. (Williams 1970a: 187)

#### Type.

MEXICO. Chiapas, Mun. Tuxtla Gutiérrez, *D.E. Breedlove & P. Raven* 13362 (holotype F0054825, isotype DS).

#### Description.

Twining perennial, stems somewhat woody below, glabrous, often slightly winged. Leaves petiolate, 4–10 × 2–6 cm, ovate, finely acuminate, distinctly truncate to very broadly cuneate, glabrous, abaxially paler with prominent veins; petioles 1.5–2.5 cm. Inflorescence of 1–5-flowered axillary cymes; peduncles 2–7 cm, stout; bracteoles squamose, c. 1 mm; secondary peduncles sometimes present, 1–1.5 cm; pedicels 3–13 mm, thickened upwards; sepals unequal, glabrous, ovate to suborbicular, outer 4–6 mm, obtuse with narrow scarious margin, inner 7–12 mm rounded, mostly scarious; corolla 5–6 cm long, funnel-shaped, orange or yellow, glabrous, limb c. 3 cm diam., shallowly lobed with oblong-ovate lobes. Capsules 15 × 6–9 mm, conical, glabrous, rostrate; seeds 10–12 × 4 mm, black with long white marginal hairs to 10 mm.

#### Distribution.

Low altitude forest from southern Mexico south to Costa Rica.

**COSTA RICA.** Punta Arenas, *W.A. Haber & E. Bello* 5984 (MO, FTG).

**NICARAGUA.** Boaco, San Lorenzo, *P. Moreno* 18523 (FTG, MO).

**GUATEMALA.** Huehuetenango, *J. Steyermark* 51015 (F).

**MEXICO. Chiapas**: *D.E. Breedlove* 28080 (MICH); Barranca el Chorreadero, *H. & C. Cabrera* 5914 (ARIZ, MEXU); Chiapa de Corzo, El Chorreadero, *D.E. Breedlove & Thorne* 20461 (MO); Tuxtla Gutiérrez, *D.E. Breedlove & P.H. Raven* 1332 (MO).

#### Note.

Distinct because of its yellowish corolla and small truncate leaves.

### 174. Ipomoea robinsonii

Taxon classificationPlantaeSolanalesConvolvulaceae

House, Ann. New York Acad. Sci. 18(6): 257. 1908. (House 1908b: 257)

#### Type.

MEXICO. Morelos, Cuernavaca, *C.G. Pringle* 7338 (holotype GH00054536, isotypes ARIZ, CAS, MICH, NY, US).

#### Description.

Liana; stems all woody, glabrous. Leaves petiolate, 6–10 × 2.3–3.8 cm, oblong-elliptic, obtuse or acute, mucronulate, base broadly cuneate to subtruncate, glabrous, abaxially paler; petioles 1–1.8 cm. Inflorescence of solitary flowers arising on short axillary shoots; peduncles 2–8 mm; bracteoles 15–26 mm, oblong or oblong-elliptic, acute, foliose, shortly petiolate (to 2 mm), persistent; pedicels 1–2 mm; sepals strongly coriaceous, glabrous, slightly unequal, outer 8 × 6 mm, elliptic, acute, convex, inner similar but 9–10 mm long; corolla 7–8 cm long, funnel-shaped, cream (?), glabrous, limb c. 3.5 cm. Capsules 15 × 7 mm, narrowly ovoid, glabrous, rostrate with mucro c. 7 mm; seeds 8 × 2–3 mm, pilose with long marginal hairs 10–12 mm in length.

#### Distribution.

Endemic to southern Mexico, where it grows in deciduous tropical forest up to 1000 m.

**MEXICO. Colima**: Ixtlahuacan, *E.J. Lott et al.* 1929 (MEXU, MO). **Est. México & Dist. Fed.**: Temascaltepec, *G.B. Hinton* 6543 (K, MO). **Guerrero**: Mun. Coahuayutla, *Y. Ramírez-A et al.* 766 (ARIZ, IEB, MEXU); Cerro El Cigarillo, *J.C. Soto Nuñez* 16295 (MEXU). **Jalisco**: La Manzanilla, *R. McVaugh* 20959 (MICH). **Michoacán**: Mun. Churumuco, *V. W Steinmann & Y. Ramírez-A* 5881 (ARIZ, IEB); Aguila, *E. Carranza & I. Silva* 6806 (IEB); Lázaro Cárdenas, Alta de la Barranca, *E. Carranza & I. Silva* 6816 (IEB, MEXU). **Oaxaca**: Santa María Huatulco, *A. Sánchez Martínez & A. Ruíz* 1071 (IEB, MEXU). **Puebla**: Pollatzin, *F. Miranda* 2945 (MEXU).

• Species 175–177. These three species form a group diagnosed by their woolly seeds and strongly discolorous leaves. *ITS* sequence data suggests Species 178–179 are also members of this clade although the seeds differ.

### 175. Ipomoea isthmica

Taxon classificationPlantaeSolanalesConvolvulaceae

J.R.I. Wood & Buril, Kew Bull. 72 (44): 2. 2017. (Wood et al. 2017b: 2)

#### Type.

PANAMA. Prov. Panama, Cerro Jefe, 22 Sept. 1972, *A. Gentry* 6135 (holotype MO).

#### Description.

Perennial liana to 8 m in height; stems woody, thinly pubescent, purplish-brown. Leaves petiolate, 7–15 × 6–11 cm, ovate, ovate-deltoid or suborbicular, truncate to very broadly cuneate with rounded auricles, apex very shortly acuminate and mucronulate, acute or retuse, margin entire to obscurely denticulate, adaxially green, glabrous or sparsely and softly strigose, abaxially densely silvery-sericeous, punctate, the venation prominent; petioles 3.5–8 cm, terete, pubescent. Inflorescence of compact axillary cymes with c. 3–10 flowers; primary peduncles 0.8–2 cm, grey-sericeous; bracteoles 2–3 × 0.5 mm, linear, obtuse, somewhat scarious, puberulent, caducous; secondary peduncles 3–5 mm, puberulent; pedicels 5–15 mm, puberulent below, becoming glabrous and thickened upwards; sepals unequal, glabrous, outer 5–7 × 4–6 mm, ovate to obovate, rounded, margins narrow, scarious, inner sepals 8–11 mm long and wide. suborbicular, rounded to retuse, the margins broad, scarious; corolla 4.5–5.5 cm long, funnel-shaped, glabrous on the exterior limb pale magenta, tube greenish with a purple-black base; limb c. 3.5 cm diam., apparently weakly lobed. Capsules 18–20 × 12–15 mm, ovoid, very shortly apiculate with persistent style base, glabrous, 4-seeded; seeds 5 × 2.5 mm, dark brown, broadly oblong in outline, densely lanate with matted brownish cottony hairs up to 15 mm long.

#### Illustration.

**Figure 101.**
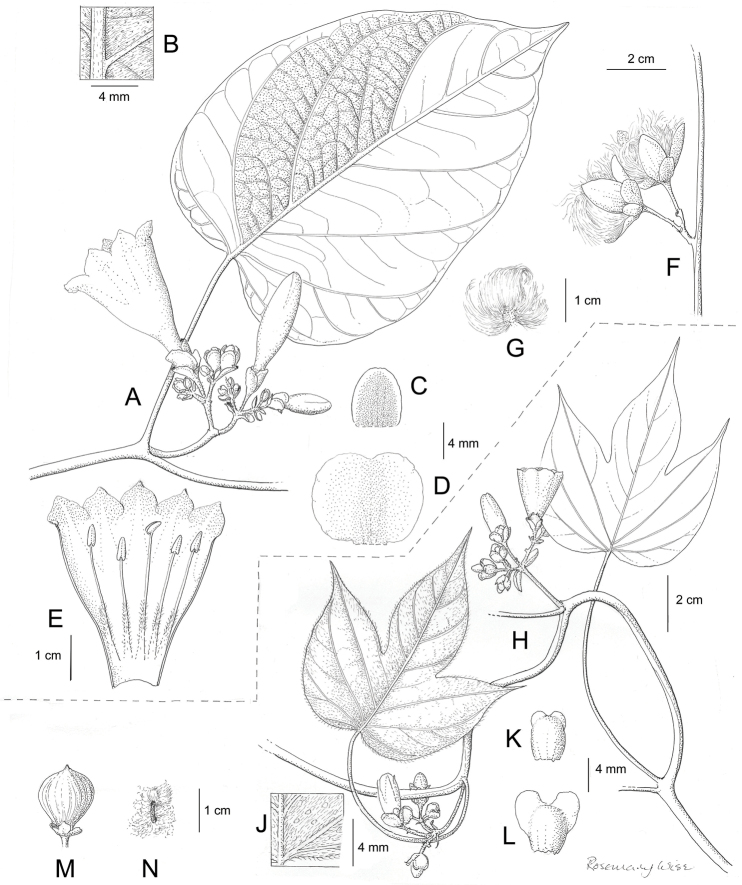
**A–G***Ipomoea isthmica*. **A** habit showing leaf and inflorescence **B** abaxial leaf surface **C** outer sepal **D** inner sepal **E** corolla opened up to show stamens **F** fruiting cyme **G** seed. **H–N***Ipomoea eremnobrocha*. **H** habit showing leaves and inflorescence **J** abaxial leaf surface **K** outer sepal **L** inner sepal **M** capsule **N** seed. Drawn by Rosemary Wise **A–E** from *Nee* 7912; **F, G** from *McPherson & Merello* 8202; **H–J** from *Polo* 39; **K, L** from *D’Arcy* 9551; **M, N** from *Correa et al*. 11312.

Figure 101A–G; Wood et al. (2017b: 4).

#### Distribution.

Endemic to eastern Panama in low altitude cloud forest, 300–1000 m.

**PANAMA.** Chagres National Park, Cerro Jefe, *A. Gentry* 6135 (MO); ibid., *E.L. Tyson et al.* 4292 (MO); ibid., *K.J. Sytsma* 2018 (MO); Llano-Carti road, *M. Nee* 7912 (MO); ibid., *G. McPherson & M. Merello* 8202 (MO, PMA); ibid., *T. Antonio* 3731 (FTG, MO).

#### Note.

Readily distinguished by the large ovate-suborbicular leaves, magenta corolla with a blackish throat and unequal sepals.

### 176. Ipomoea eremnobrocha

Taxon classificationPlantaeSolanalesConvolvulaceae

D.F. Austin, J. Torrey Bot. Soc. 12: 145. 1997. (Austin 1997: 145), emend. J.R.I. Wood & Buril (Wood et al. 2017b: 5)

#### Type.

PANAMA. Cerro Campana, *A. Gentry* 5759 (holotype cited from MO and isotype cited from FAU, but both missing).

#### Description.

Perennial climber or liana of unknown height but reaching at least several metres high, stems twining, somewhat woody below, herbaceous above, thinly pubescent when young, glabrescent, pale brown. Leaves petiolate, 5–12 × 7–12 cm, ovate in outline, 3-lobed to about half way, base ± truncate or subcordate and shortly cuneate onto the petiole, apex finely acuminate and shortly mucronate, central lobe oblong-elliptic (rarely ovate), 2–5 × 2–4 cm, laterals broadly ovate, margins entire or undulate, adaxially green, pubescent, abaxially densely silvery-sericeous with appressed hairs and scattered white glands; petioles 4–11 cm, thinly pubescent. Inflorescence of compact axillary cymes; primary peduncles 0.5–2.5 cm, stout, pubescent; bracteoles 2–7 × 0.5–1 mm, filiform to lanceolate, acuminate, pubescent, tardily deciduous; secondary peduncles 3–5 mm; pedicels 3–8 mm, pubescent; sepals somewhat unequal, outer 4–5 × 2–3 mm, broadly oblong, truncate or slightly retuse, glabrous or with a few hairs at the base, inner 5–6 × 3–4 mm, obovate, usually strongly retuse with a broad sinus so appearing winged, margins scarious; corolla ±campanulate, white, glabrous on the exterior, 2–2.5 cm long; limb 2.2–2. 5 cm diam. Capsules 12–13 × 10–11 mm, ellipsoid to subglobose, very shortly apiculate with persistent style base, glabrous, 4-seeded; seeds 6 × 1.5–2 mm, brown, broadly oblong in outline, densely lanate with matted cottony hairs up to 10 mm long.

#### Illustration.

Figure 101H–N.

**Figure 102.**
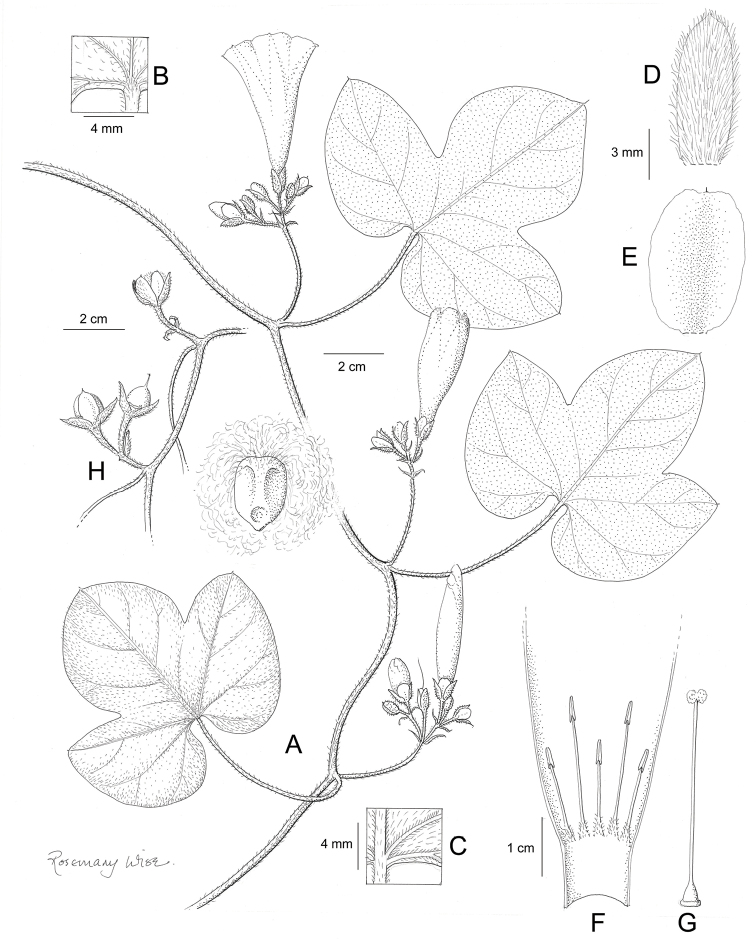
*Ipomoea peteri*. **A** habit **B** adaxial leaf surface **C** abaxial leaf surface **D** outer sepal **E** inner sepal **F** corolla opened out to show stamens **G** ovary and style **H** fruiting inflorescence with capsules **J** seed. Drawn by Rosemary Wise **A–C** from *Morgensen* 1106; **D–G** from *Whitefoord* 8288; **H–J** from *Wallnöfer & Tut-Tesucun* 9662.

#### Distribution.

A species with a remarkable disjunct distribution with one population in NE Brazil and the other in Panama, extending with some doubt to Costa Rica, from where fertile material has not been seen. It is a species of forest from around 100 to 1150 m.

**BRAZIL. Bahia**: Litoral Sul, Itagibá, Mata da Botinha, *M.L. Guedes et al.* 16520 (ALCB, US); Muritiba, *E.C. Schmidt et al.* 313 (HUEFS). **Paraíba**: Mun. Areia, Mata do Pau Ferro, *Andrade-Lima et al.* 01 (IPA, OXF). **Sergipe**: São Cristóvao, *M. Landim et al.* 1316 (ASE7882).

**PANAMA.** Restricted to Altos de Campana: *C.E. Polo* 39 (F, MO, PMA); *M.D. Correa et al.* 8074 (F), 11312 (MO); *W.G. D’Arcy* 9551 (MO), *9592* (MO), *B. Hammel* 5519 (MO); *R. Méndez* 57 (MO).

**COSTA RICA.** Cuenca del San Carlos, *B. Hammel* 20340 (MO); Cuenca del Sarapiquí, *B. Hammel* 20688 (MO).

#### Notes.

This species differs from *Ipomoea isthmica* by the 3-lobed leaves, shorter, pubescent, subequal sepals and shorter campanulate corolla. It can be distinguished from *Ipomoea peteri* by the pink corolla, finely acuminate leaf lobes and the obovate, merely pubescent (not oblong-lanceolate, tomentose) sepals.

For a discussion about confusion with *Ipomoea isthmica*, see Wood et al. (2017d).

### 177. Ipomoea peteri

Taxon classificationPlantaeSolanalesConvolvulaceae

(Kuntze) Staples & Govaerts, Phytologia 97(3): 220. 2015. (Staples et al. 2015: 220)

Ipomoea sericophylla Peter, Nat. Pflanzenfam. IV (3a): 31. 1897 [pub. 1891). (Peter 1891: 31), nom. illeg., non Ipomoea sericophylla Meisn. (1869). Type. GUATEMALA. *Bernoulli & Cario* 1892 (lectotype GOET005709, designated by Staples et al. [2012: 676]).
Mouroucoa peteri Kuntze, Revis. Gen. Pl. 3(2): 218. 1898. (Kuntze 1898: 218). Type. Based on Ipomoea sericophylla Peter
Ipomoea tuxtlensis House, Ann. New York Acad. Sci. 18: 256. 1908. (House 1908b: 256). Type. MEXICO. Chiapas, *E.W. Nelson* 3094 (holotype US00111481).
Pharbitis lindenii M. Martens & Galeotti, Bull. Acad. Roy. Soc. Bruxelles 12(2): 272. 1845. (Martens and Galeotti 1845: 272), non Ipomoea lindenii M. Martens & Galeotti (1845). Type. MEXICO. Tabasco, Linden 296 (holotype BR00006973063, isotypes K, MICH).
Ipomoea silvestris Brandegee, Univ. Calif. Publ. Bot. 6(8): 190. 1915. (Brandegee 1915: 190). Type. MEXICO. Veracruz, C.A. Purpus 7309 (holotype UC174944, isotype US).

#### Type.

Based on *Ipomoea sericophylla* Peter

#### Description.

Perennial climber apparently with tuberous roots; stem pubescent. Leaves petiolate, 3.5–9 × 3.5–9, sometimes ovate but usually 3-lobed to about halfway or slightly less, lobes elliptic, narrowed at both ends, occasionally ovate or somewhat repand, acute or shortly acuminate, mucronate, base truncate to cordate, often cuneate onto the petiole, adaxially appressed pilose, abaxially softly adpressed silvery-grey pilose; petioles 2–8.5 cm, pubescent. Inflorescence of few-flowered, pedunculate axillary cymes; peduncles usually short, 1–5 cm, pubescent; bracteoles 4–12 mm, filiform; secondary peduncles c. 5 mm; pedicels 5–12 mm, thickened upwards, pubescent; sepals unequal, outer 8–11 × 3 mm, oblong-lanceolate, acute, often mucronate, tomentose, inner 11–14 × 4–5 mm, broadly oblong-elliptic, rounded to retuse, glabrous or pubescent in the centre but with scarious, glabrous margins; corolla 4–6 cm long, funnel-shaped, deep pink, glabrous, limb c. 3 cm diam. Capsules 7–10 mm, globose, shortly rostrate, glabrous; seeds 4 × 3 mm, brown, lanate.

#### Illustration.

Figures 11A, 102.

#### Distribution.

Endemic to Central America from Nicaragua north to Southern Mexico, growing at low altitudes, in various kinds of woodland including pine forest, secondary woodland and flooded forest.

**NICARAGUA.** Atlántico Norte, Cerro Waylawás, *J.J. Pipoly* 4490 (MO).

**BELIZE.** Orange Walk District, *C. Whitefoord* 8035; Whitehills, *C. Whitefoord* 8288 (BM); Stann Creek, *W.A. Schipp* 421 (BM, K); Northern River, *P.H. Gentle* 1038 (K); Stann Creek, *P.H. Gentle* 3073 (K); Toledo, Deep River, *Z. Goodwin & G. López* 1709 (MO).

**GUATEMALA.** Petén, Lago Petén Itza, *B. Wallnöfer & Tut-Tesucun* 9662 (NY, MO, W); P.N. Tikal, *R. Tun Ortíz* 241(BM, F, MO).

**MEXICO. Campeche**: Calakmul, *E. Martínez et al.* 29268 (BM, MEXU). **Chiapas**: *E. Martínez* 14747 (ARIZ, MEXU); Tzimol, *A. Reyes-García & G. Urquijo* 791 (BM, MEXU); Ocosingo, *Aguilar* 2622 (BM, MEXU, MO). **Quintana Roo**: Benito Juárez, *E.F. & H. Cabrera* 3463 (MO); Nuevo Xcan, *O. Téllez* 2870 (BM, MEXU). **Tabasco**: Balancán, *A. Novelo et al.* 58 (BM, MEXU, MO); ibid., Naranjito, *F. Menendez et al.* 263 (K, MEXU, MO). **Veracruz**: Zacuapan, *C.A. Purpus* 7309 (BM); ibid., 10672 (K); Hidalgotitlan, *B. Vazquez* 1239 (BM). **Yucatán**: *E. Martínez* 31466 (BM, MEXU); Valladolid, Xuilub, *B. Morgensen* 1106 (AAU, K, NY); Buena Vista, *G.F. Gaumer* 769 (K, S).

#### Notes.

Somewhat variable in the density of the indumentum. It differs from *Ipomoea eremnobrocha* in the narrower, acute, mucronate outer sepals. It is generally more densely hirsute.

*Hinton* 8120 (K) from Acatitlan, Temascaltepec is correctly identified as this species but the location would appear to be wrong. There was perhaps an error in the labelling.

### 178. Ipomoea heterodoxa

Taxon classificationPlantaeSolanalesConvolvulaceae

Standl. & Steyerm., Publ. Field Mus. Nat. Hist., Bot. Ser. 23: 82. 1944. (Standley and Steyermark 1944: 82)

#### Type.

BELIZE. Maskall, *P. Gentle* 871 (holotype F0054844, isotype S).

#### Description.

Twining perennial or small liana to c. 4 m, stems glabrous, somewhat woody. Leaves petiolate, palmately divided into 5–7 lobes, the lateral 4 lobes sessile to shortly petiolate, the terminal lobe pedately trilobed, lobes 2–7 × 0.5–1.8 cm, oblong-oblanceolate, obtuse to subacute, glabrous, abaxially paler; petioles 3–5 cm. Inflorescence of shortly pedunculate, cluster-like cymes, the cymes often paired; peduncles 0–12 mm; secondary peduncles 2–4 mm; bracteoles early caducous, not seen; pedicels 6–9 mm; sepals subequal, rigid and somewhat convex, glabrous, the margins scarious, outer 4–5 × 4 mm, obovate-elliptic, obtuse, inner sepals 5 × 5 mm, rounded to retuse; corolla 3–3.5 cm, funnel-shaped, white with pink-flushed limb, glabrous, gradually widened from base, tube whitish-green; limb c. 2 cm diam. Capsules 7 × 4–5 mm, ovoid, rostrate, glabrous, the mucro 2–3 mm long; seeds 5–7 mm long, pilose with white marginal hairs.

#### Illustration.

Figure 7D.

#### Distribution.

Endemic to Central America in dry forest at very low altitudes.

**GUATEMALA.** Petén, P.N. Tikal, *R. Tun Ortíz* 364; ibid., 440 (BM, MO); ibid., *E. Contreras* 367 (F, MO, XAL); Lago Petén Itzá, *B. Wallnöfer* 9506 (K, MO, W).

**BELIZE.** Stann Creek, *G.R. Proctor* 35804 (BM); ibid., *W.A. Schipp* 846 (BM, K, S); Cayo, Chaa Creek Trails, *M.J. Balick et al.* 3171 (NY, OXF).

**MEXICO. Campeche**: Calakmul, Puente El Papagayo, *E. Martínez et al.* 31814 (BM, MEXU); ibid., Narciso Mendoza, *D. Álvarez* 544 (BM, MEXU). **Chiapas**: Ocosingo, *E. Martínez* 15974 (MO). **Quintana Roo**: Laguna Ocum, *E. Cabrera* 293 (BM, MEXU): Felipe Carillo Puerto, *E.F. Cabrera & L. Cortez* 396 (BM, MEXU). **Tabasco**: Balancan, *A. Novelo et al.* 115 (K, MEXU). **Yucatán**: Mérida, *A. Schott* 589 (BM); Izamal, *G.F. Gaumer* 989 (BM, K, S); Valadolid, Xuilub, *B. Morgensen* 1182 (AAU, K, MO).

#### Note.

The palmately lobed leaves and shortly pedunculate cymes distinguish this species. The distribution in Fl. Mesoamericana 4(2): 332 is completely wrong.

### 179. Ipomoea steerei

Taxon classificationPlantaeSolanalesConvolvulaceae

(Standl.) L.O. Williams, Fieldiana, Bot. 32(12): 195. 1970. (Williams 1970a: 195)

Exogonium steerei Standl., Publ. Carnegie Inst. Wash. 461(4): 83. 1935. (Standley 1935: 83). Type. MEXICO. Yucatán, Chichen Itza, *W.C. Steere* 1545 (holotype F668631, isotypes G, LL, MICH, MO, NY).
Ipomoea clewellii C. Nelson, Phytologia 72(6): 401. 1992. (Nelson 1992: 401). Type. HONDURAS. Gracias a Dios, Ahuas, *A. Clewell* 3679 (TEFH).

#### Type.

Based on *Exogonium steerei* Standl.

#### Description.

Perennial twining plant with wiry, pubescent stems up to 4 m high. Leaves shortly petiolate, 2.5–6 × 2–3.5 cm, oblong-oblanceolate or oblong-elliptic, cuneate, apex acute and strongly mucronate, adaxially thinly pilose, green, abaxially densely adpressed silvery-pilose; petioles 3–11 mm, pubescent. Inflorescence of solitary or few-flowered axillary cymes; peduncles 1 –3 cm, densely silvery-pilose; bracteoles 10–18 × 2–3.5 mm, oblanceolate, acute, tapering to a petiole-like base, caducous; pedicels 8–15 mm, glabrous to thinly pubescent, especially below; sepals unequal, coriaceous, glabrous with scarious margins, outer 5–7 × 3–4 mm, elliptic to suborbicular, rounded to obtuse, inner 9–10 × 5–6 mm, elliptic to obovate, rounded to retuse; corolla 4.5–5.5 cm long, pink, glabrous, funnel-shaped, limb 3.5 cm diam., unlobed. Capsules 12–15 × 6–7 mm, ovoid, acute, glabrous; seeds 8–10 mm, ovoid, pilose.

#### Distribution.

Deciduous woodland, flooded forest and mangroves at low altitudes in Central America; rather uncommon.

**HONDURAS.** Type of *Ipomoea clewellii*.

**GUATEMALA.** Petén, P.N. Tikal, Bajo de Santa Fe, *C.L. Lundell* 16492 (MO).

**MEXICO. Campeche**: Hecelchakán, *E. & H. Cabrera* 13968 (F, MO), 13943 (BM, IEB, MEXU); Calakmul, *E. Martínez et al*. 35970 (IEB). **Quintana Roo**: camino a Vigía Chica, *E. Cabrera & H. Cabrera* 3529 (BM, MEXU, MO, K); ibid., *O. Téllez & E. Cabrera* 3186 (BM, MEXU, MO). **Yucatán**: Valladolid, Xuilub, *B. Morgensen* 1064 (AAU, K).

#### Note.

The oblong-elliptic leaves with long, silvery appressed hairs abaxially are distinct as is the habitat.

• Species 180–182 form a group of three Mexican species with a hypocrateriform corolla and a tendency to be leafless at anthesis.

### 180. Ipomoea conzattii

Taxon classificationPlantaeSolanalesConvolvulaceae

Greenm., Publ. Field Columb. Mus., Bot. Ser. 2(6): 258. 1907. (Greenman 1907: 258)

Exogonium conzattii (Grenm.) House, Bull. Torrey Bot. Club 35: 102. 1908. (House 1908a: 102).

#### Type.

MEXICO. Oaxaca, Almoloyas, *C. Conzatti* 1666 (holotype F0054836, isotypes MEXU, VT).

#### Description.

Twining liana; stems woody with grey bark, white canescent when young. Leaves absent at anthesis, petiolate, 1.5–10 × 1–6.5 cm, ovate-deltoid, subrhomboid, panduriform or shallowly 3 –lobed, margin undulate, apex acute, obtuse or retuse, mucronate, base truncate and cuneate onto the petiole, pubescent on both surfaces, abaxially paler; petioles 0.5–6 cm, pubescent. Inflorescence of very shortly pedunculate compact corymbs; peduncle 0.3–2.8 cm, sericeous; bracteoles 2–3 mm, ovate-deltoid, sericeous, caducous; secondary peduncles 1–5 mm; pedicels 12–15 mm, thickened upwards, canescent; sepals slightly unequal, outer 5–7 × 3–6 mm, elliptic, obtuse, densely pubescent to canescent, greenish, inner sepals obovate, rounded, pubescent in the centre but with broad glabrous scarious margins; corolla 3–4.5 cm long, cylindrical-hypocrateriform, deep pink, glabrous or with a few hairs, somewhat rugose, limb 2–3 cm diam., lobed, stamens exserted. Capsules ellipsoid, 9 × 6–8 mm, glabrous: seeds 6 × 3 mm, pilose on margins with long white hairs c. 7 mm long.

#### Distribution.

Endemic to central Mexico, growing around 1200 to 2200 m in dry deciduous woodland and scrub; apparently uncommon.

**MEXICO. Est. México & Dist. Fed.**: El Zapote, *S. Zamudio* 10995 (IEB, MEXU); Villa Guerrero, *E. Matuda et al.* 28029 (MEXU). **Guerrero**: Cerro Xilotzin, *E. Moreno-G* 871 (MEXU). **Morelos**: Xochicalco, *Hahn* s.n. (P); Cuautla, Sierra de Topotzlán, *D.H. Lorence* 5021 (MEXU, MO); Tezoyuca-E. Zapata, *J. Vásquez* 252 (MEXU); Cuernavaca, *E. Fournier* s.n. [1866] (P); *E. Lyonnet* 550400015 (IEB, MEXU). **Oaxaca**: Almolayas, *C. Conzatti* 1950 (F); Cuicatlan, *J.I. Calzada* 23880 (K, MEXU). **Puebla**: Tehuacán, *C.A. Purpus* 5833 (BM, MO); ibid., *H.S. Gentry* 23385 (ARIZ, DES, MEXU); Cuicatlán, *G.A. Salazar et al*. 9383 (MEXU); Caltepec, *P. Tenorio & C. Romero* 5100 (MEXU); *R. Razo & R. García* III-39 (IEB). **Veracruz**: *Long & Burch* 3278 (F); Acutzingo, *J.E. Rivera* 5451 (MEXU).

#### Note.

Distinguished from related species by the relatively broad corolla limb, near glabrous corolla but canescent sepals and pedicels.

### 181. Ipomoea concolor

Taxon classificationPlantaeSolanalesConvolvulaceae

(Matuda) D. Austin, Ann. Missouri Bot. Gard. 64: 335. 1977 [pub. 1978]. (Austin 1978: 335)

Exogonium concolorum Matuda, Anales Inst. Biol. Univ. Nac. México 36: 116. 1965. (Matuda 1965: 116). Type. MEXICO. Guerrero, Rincón de la Vía, H. Kruse 844 (holotype MEXU00092233; isotypes IEB, MEXU).
Ipomoea praecox McPherson & Meacham, Contr. Univ. Michigan Herb. 14: 85. 1980. (McPherson 1980: 85), nom. illeg., non Ipomoea praecox C. Wright (1870). Type. MEXICO. Oaxaca, limestone hillside SW of Sola de Vega, *R. Moran* 10095 (holotype UC1235383).
Ipomoea mcphersonii D.F. Austin, Taxon 45: 12. 1996. (Austin and Huáman 1996: 12). Type. Based on Ipomoea praecox McPherson & Meacham

#### Type.

Based on *Exogonium concolorum* Matuda

#### Description.

Climbing perennial; stems woody to 2 m, pubescent. Leaves petiolate, 8–12 × 7–9 cm, broadly ovate, apex abruptly and shortly acuminate, base truncate and shortly cuneate onto the petiole, adaxially glabrous to thinly pubescent, abaxially tomentose, paler; petioles 5–7 cm, pubescent. Inflorescence a dense, many-flowered pedunculate cyme; primary peduncles 1–2.5 cm, tomentose; bracteoles 3–5 mm, lanceolate, caducous; secondary peduncles 1–4 mm; pedicels 4–12 mm, sericeous; sepals subequal, 7–8.5 × 3.5–4.5 mm, ovate-elliptic, obtuse, coriaceous, reddish, pubescent, the inner sepals more densely pubescent but with glabrous, scarious margins; corolla 3–4 cm long, tubular-hypocrateriform, dark pinkish-red, sericeous, limb lobed, the lobes 3–6 mm long, ovate, obtuse; stamens exserted. Capsules 22 mm long (fide Matuda), conical, glabrous; seeds 7–8 × 5 mm, densely pilose on the margins with white hairs c. 8 mm long.

#### Illustration.

McPherson (1980: 87).

#### Distribution.

Limestone rocks in scrub at c. 750 m. Endemic to southern Mexico.

**MEXICO. Guerrero**: Chilpancingo, *E. Matuda & E. Halvenger* s.n. (MEXU). **Oaxaca**: *Ghiesbrecht* s.n. (P); Pochutla, San Miguel del Puerto, *A. Nava Zafra et al.* 618 (IEB, MEXU).

#### Note.

Somewhat similar to *Ipomoea tehuantepecensis* in habit, corolla shape and in the reddish sepals but immediately distinguished by the sericeous exterior of the corolla and sepals. Also resembles *Ipomoea conzattii* but differs in the much shorter corolla lobes and the sericeous corolla.

### 182. Ipomoea tehuantepecensis

Taxon classificationPlantaeSolanalesConvolvulaceae

L. Torres, R. Torres, M.P. Ramírez & J.A. McDonald, J. Bot. Res. Inst. Texas 2(2): 793 2008. (Torres et al. 2008: 793)

#### Type.

MEXICO. Oaxaca, Tehuantepec, camino al Arroyo de Las Minas, *R. Torres C. & C. Martínez R.*11255 (holotype MEXU01240513; isotype MO).

#### Description.

Twining liana of unknown height; stems stout, woody, glabrous. Leaves deciduous before anthesis, petiolate, 5–11 × 3.5–8.5 cm, broadly ovate, acute, base truncate or subcordate and cuneate onto petiole, both surfaces glabrous, abaxially paler; petioles 3–8.5 cm. Inflorescence a many-flowered, compact, complex cymose structure; primary peduncles 1–3 mm, glabrous; bracteoles caducous, not known; secondary peduncles 6–6.5 mm; pedicels 4–6 mm, glabrous; sepals subequal, 4–4.5 × 2–3 mm, elliptic, obtuse, mucronate, reddish, glabrous, the inner slightly larger and with scarious margins; corolla 2.5–3 cm long, cylindrical-hypocrateriform, red, glabrous, limb 5-lobed, lobes 3–6 mm long and wide, recurved, stamens exserted. Capsules 9–13 × 6–7 mm, ellipsoid, glabrous; seeds 7 × 4.5 mm, pilose on angles with hairs c. 7 mm long.

#### Illustration.

Torres et al. (2008: 794).

#### Distribution.

Endemic to the area around Tehuanteptec where it prefers steep slopes in low deciduous forest up to 750 m.

**MEXICO. Oaxaca**: Cerro Guien Gola, *P.J. Stafford et al.* 8 (BM, MEXU, MO).

#### Note.

Distinguished from *Ipomoea conzattii* and *I. concolor* by the shorter, glabrous sepals and corolla.

• Species 183–215 are endemic to the Caribbean region. Our 605 nuclear regions sequence data suggests they form a distinct clade but our sampling is too limited to confirm this with confidence. They form the nearest thing to an island radiation within *Ipomoea*.

### 183. Ipomoea alterniflora

Taxon classificationPlantaeSolanalesConvolvulaceae

Griseb., Cat. Pl. Cub. 202. 1866. (Grisebach 1866: 202)

Ipomoea obtusata Griseb., Cat. Pl. Cub. 202. 1866. (Grisebach 1866: 202). Type. CUBA. *C. Wright* 3092 (holotype GOET000343, isotypes GH, K, YU).
Ipomoea obtusata var. latifolia Griseb., Cat. Pl. Cub. 202. 1866. (Grisebach 1866: 202). Type. CUBA. *C. Wright* 3099 (holotype GOET?, not seen, isotypes G, GH, K, NY).
Ipomoea excisa Urb., Symb. Antill. 9: 246. 1924. (Urban 1924b: 246). Type. CUBA. Prov. Pinar del Río [La Habana?], Sierra de Anafe, Loma San Gabriel, 21 March 1920, *E.L. Ekman* 10558 (holotype S07-4426).
Ipomoea cubensis sensu Urban (1925) et auct. mult.

#### Type.

CUBA occ., *C. Wright* s.n. (holotype GOET000347, possible isotypes GH, HAC ex Herb. Sauvalle 1635, NY).

#### Description.

Perennial twining herb, stem glabrous, pale brown. Leaves shortly petiolate, 2–7 × 1.5–3.5 cm, ovate to ovate-elliptic, sometimes shallowly lobed, apex shortly acuminate, acute, obtuse or, sometimes, retuse, base cordate to broadly cuneate, margin undulate, glabrous, abaxially paler; petioles 1–3 cm. Inflorescence of 1–5-flowered, axillary cymes; peduncles 1.5–6 cm; bracteoles caducous; secondary peduncles 0.3–1.7 cm; pedicels 6–20 mm; sepals slightly unequal, glabrous with scarious margins, coriaceous, outer 10 × 5–6 mm, ovate to suborbicular, rounded, inner 9–11 × 6–8 mm; corolla 4–5.5 cm long, funnel-shaped, greenish-yellow, greenish-yellow with pink throat or pink, glabrous. Capsules 10–14 × 7–10 mm, ovoid, rostrate, glabrous; seeds 5–7 × 3–4 mm, blackish with very long white, marginal hairs 6–10 mm long.

#### Distribution.

*Ipomoea alterniflora* is endemic to western Cuba from where all collections come. It appears to be a plant of forest relics.

**CUBA. Isla de Juventud (Pinos)**: *E.L. Ekman* 12563 (S); *A. Alvarez et al.* (HAJB 455570). **Pinar del Río**: Mantua, Camarones, cima de Los Cabreros. *A. Alvarez al.* (HAJB 51183); Baños San Vicente, *N.L. Britten et al.* 7481 (NY); El Sapapo, Pinar de Sabanalamar, *A. Areces et al.* 28396 (HAC); Cabo Corrientes, Jaimanilas, *R.A. Quintana et al.* (HAJB 34218)–a good match with *Wright* 3092; Guanahacabibes, *J. Bisse et al.* (HAC, HAJB33208); Pinares de Cajálbana, La Palma, *Bro. Alain & J. Acuña* 1167, 1168 (HAC); Pinar del Rangel; Mogote de la Bandera, *Roig* 8358 (HAC). **La Habana**: Loma de la La Pita, San Miguel de Casanova, *Bro. León* 8388 (HAC); Sierra de Anafe, *P. Wilson* 11417 (NY); ibid., *E. Ekman* 13494 (BM, S); Caimito, *J. Bisse et al.* (HAJB 51278)–good match with *Ekman* 10558; Tetas de Managua, *H.A. Van Hermann* 318 (HAC). **Matanzas**: San Miguel de los Baños, *J. Bisse & Rojas* (HAJB 4522)–red-flowered. **Villa Clara**: Sierra Alta de Agabama, *R. Berazaín et al.* (HAJB 58044).

#### Notes.

*Ipomoea alterniflora* is a variable species characterised by its glabrous stem and leaves. The leaves are usually ovate, cordate and shortly acuminate to an obtuse apex but are sometimes lobed as in the possible isotypes in HAC and NY. The corolla colour in the holotype is whitish-green and this is clearly the same in the GH and HAC isotypes but the NY isotype is more darkly coloured and could be red.

The most variable aspects of this species lie in the leaf shape. In the type of *Ipomoea obtusata* the leaves are ovate-elliptic with a rounded to cuneate base and obtuse apex. In the type of *I. excisa* the leaves are ovate but the apex is retuse. Although the extreme forms are rather distinct, there are many specimens that connect these with more common forms typified by *Wright* 3099 and the type of *I. alterniflora*.

### 184. Ipomoea cubensis

Taxon classificationPlantaeSolanalesConvolvulaceae

(House) Urb., Symb. Antill. 9: 427. 1925. (Urban 1925: 427)

Exogonium cubense House, Bull. Torrey Bot. Club. 35(3): 105. 1908. (House 1908a: 105). Type. CUBA. Matanzas: gorge of the Río Yamuri, N.L. Britton & J.A. Shafer 495 (holotype NY00111062).

#### Type.

Based on *Exogonium cubense* House

#### Description.

Slender twining perennial to several metres; stems glabrous, somewhat woody. Leaves petiolate, 4.5–8 × 3.5–8 cm, ovate, truncate to subcordate at base, entire or sinuately 3–5-lobed, glabrous, somewhat reticulate-veined. Inflorescence of few-flowered axillary cymes; peduncles 2–9 cm; bracteoles caducous, not seen; secondary peduncles 0.8–1.5 cm; pedicels 10–35 mm; sepals unequal, ovate, obtuse, margin scarious, outer 5–6 mm, inner 8–10 mm; corolla c. 5 cm long, white, tube narrow for 2–2.5 cm, then funnel-shaped, midpetaline bands ending in mucros, limb 4–5 diam., 5-lobed; stamens weakly exserted. Capsules 13 × 8 mm, ovoid, rostrate, glabrous; seeds long pilose with hairs to 10 mm.

#### Distribution.

Endemic to woodland in western Cuba.

**CUBA. Pinar del Río**: Candelaria, Soroa cerca del Orquideario, *H. Manitz* (HAJB51284); ibid., *J. Bisse & F. Meyer* (HAJB36292); Soroa, Río San Cristóbal, *J. Bisse et al.* (HAJB37868). **Matanzos**: Peninsular Hicacos, Rincón Francés *J. Bisse & G. Klotz* (HAJB26142) – with doubt.

#### Note.

This is a puzzling and misunderstood species. It is essentially the same as *Ipomoea alterniflora* but the basal half of the corolla tube is cylindrical, the stamens are exserted and the leaves sinuate-margined. However, none of the specimens cited above is quite as distinct as the type and careful field observations are needed to confirm that this species really is distinct from *Ipomoea alterniflora*. Most specimens called *Ipomoea cubensis* are correctly *Ipomoea alterniflora*.

### 185. Ipomoea merremioides

Taxon classificationPlantaeSolanalesConvolvulaceae

Alain, Rev. Soc. Cub. Bot. 13: 8. 1956. (Liogier 1956: 8)

#### Type.

CUBA. Prov. Oriente [Holguín], Río Lebisa, Sierra de Cristal, 30 Dec. 1955, *Bro. Alain & M. López Figueiras* 4834 (holotype HAC, isotype US).

#### Description.

Twining perennial liana, stems stout, woody, glabrous, but sometimes with lenticels. Leaves petiolate, 6–13 × 5.5–10 cm, ovate-deltoid, weakly cordate to subtruncate, finely acuminate, mucronate, margin entire, glabrous; petioles 2–6 cm. Inflorescence of pedunculate axillary, many-flowered compound cymes; peduncles 2–5 cm; bracteoles caducous; secondary peduncles 1–1.5 cm; pedicels 10–20 mm; sepals subequal, 4–5 mm, suborbicular, coriaceous, rounded; corolla 1.5–1.7 cm long, white, glabrous. Capsules ovoid, 11–12 mm long, glabrous; seeds ovoid, 6 × 4 mm long, densely pilose over the whole surface with hairs to 12 mm.

#### Distribution.

Endemic to eastern Cuba and perhaps restricted to the Sierra de Cristal. **CUBA. Holguín**: Sierra de Cristal, Montes de la Nicaro, subida a La Loma de los Mulos, *Bro. Alain et al.* 5339 (HAC, HAJB); ibid., *Br. Alain et al.* 9653 (HAC); Charrascos, km 5 de Sabanilla a Cajobabo, Baracoa, *Bro. Alain et al.* 7718 (HAC, HAJB).

#### Note.

This species is clearly related to *Ipomoea alterniflora* but is distinguished by the small corolla, short sepals, many-flowered inflorescence, stout woody stems and glabrous leaves.

### 186. Ipomoea erosa

Taxon classificationPlantaeSolanalesConvolvulaceae

Urb., Symb. Antill. 9: 425. 1925. (Urban 1925: 425)

#### Type.

CUBA. Prov. Oriente [Holguín-Guantánamo], Sierra de Nipe, Río Canapú, *E.L. Ekman* 15127 (holotype S07-4425).

#### Description.

Twining perennial; stems pubescent with spreading white hairs. Leaves petiolate, 5–8 × 2.5–5 cm, ovate-deltoid to almost elliptic, base cordate, apex obtuse to rounded, apiculate, margin denticulate, both surfaces grey pubescent to tomentellous, abaxial veins prominent; petioles 1–4.5 cm, tomentellous. Inflorescence of axillary pedunculate cymes borne on short leafy branchlets with up to 7 flowers; peduncles 0.9–1.5 cm, tomentellous; pedicels 5–15 mm; sepals suborbicular, rounded, convex, outer 7–8 × 6.5 mm, inner 8–10 × 8 mm; corolla c. 8 cm long, white, glabrous, funnel-shaped, tube widened to 1.3 cm at mouth, limb 3 cm diam.; anthers unequal, included. Capsules and seeds unknown.

#### Distribution.

Endemic to Eastern Cuba and only known from the type collection.

#### Note.

This species is distinguished by the pubescent, denticulate, leaves, the short peduncles and the white flowers.

### 187. Ipomoea balioclada

Taxon classificationPlantaeSolanalesConvolvulaceae

Urb., Symb. Antill. 9: 245. 1924. (Urban 1924b: 245)

#### Type.

CUBA. Prov. Oriente [Guantánamo], Sierra Maestra supra Daiquiri, c. 800 m, 28 Oct. 1916, *E.L. Ekman* 8080 (holotype S07-4401, isotypes BM, G, NY).

#### Description.

Twining perennial, stems somewhat woody, glabrous but covered in numerous flat black glands. Leaves petiolate, 5–9 × 3–6 cm, deltoid, acuminate, base truncate with rounded auricles, margin slightly sinuate, both surfaces glabrous; petioles 1.5–4 cm, glandular. Inflorescence of pedunculate axillary cymes; peduncles 2.5–5 cm, glandular, glabrous; bracteoles lanceolate, 2.5–3 mm long, caducous; secondary peduncles 6–10 mm; pedicels 16–18(–25) mm; sepals unequal, rather rigid, glabrous with prominent scarious margins, elliptic, obtuse to rounded, outer 6–9 × 4–6 mm, inner 10–12 × 6–7 mm; corolla 5–5.5 cm long, funnel-shaped, glabrous, pink, stamens shortly exserted. Capsules ovoid, 12 × 9 mm, rostrate, glabrous; seeds 5 × 3 mm, long-pilose on the margins with hairs up to 12 mm.

#### Distribution.

Endemic to eastern Cuba in the Sierra Maestra. We have not seen any collections other those by Ekman.

**CUBA. [Guantánamo**?]: Sierra Maestra, Arroyo Jiménez, *E.L. Ekman* 14805 (HAC, S).

#### Note.

Although O’Donell annotated specimens of this species as *Ipomoea alterniflora*, it is very distinct because of the black glands on the stem, peduncles and, sometimes, the petioles. The corolla is pink.

### 188. Ipomoea passifloroides

Taxon classificationPlantaeSolanalesConvolvulaceae

House, Ann. New York Acad. Sci. 18(6): 230. 1908. (House 1908b: 230)

#### Type.

CUBA. [Guantánamo], Sierra Maestra, Jiquarito Mountain, 2400 ft, 18 Sept. 1906, *N. Taylor* 504 (holotype NY00111090).

#### Description.

Villous twining perennial. Leaves petiolate, 3–7 × 3–7.5 cm, broadly ovate, cordate, mostly 3-lobed (except leaves at extremities), auricles rounded, apex obtuse and mucronate, velvety-tomentose on both surfaces, abaxially paler and brownish; petioles 8–25 mm, tomentose. Inflorescence of compact axillary cymes; peduncles short, 0.7–1.7 cm, tomentose; bracteoles 6–8 mm, oblong-ovate, obtuse, tomentose; secondary peduncles 2–3 mm; pedicels 3–4 mm, densely hirsute; sepals subequal, 7–9 × 3–4 mm, elliptic to suborbicular, coriaceous, scarious-margined, pubescent at base, glabrous apically; corolla 4–5 cm long, campanulate to funnel-shaped, strongly ventricose above base, glabrous, deep pink, the limb 2–2.5 cm diam., weakly lobed. Capsules ovoid, 11–12 × 9–10 mm, dark brown, glabrous; seeds 3 × 2.5 mm; pubescent, the angles long-pilose with hairs c. 7–8 mm long.

#### Distribution.

Restricted to the Sierra Maestra in the east of Cuba and the island of Grand Cayman.

**CUBA.** Sierra Maestra, Alcarraza River, *Bro. Clemente* 5075 (HAC, NY); *A. Gentry & Lewis* 50981 (MO, FTG); *J. Acuña* 7731 (HAC); *Moncada & Machado* 1766 (HAC); *M. López Figueiras* 40380 (HAC), 2316 (HAC), 380 (HAJB), 388 (HAJB). **Holguin**: El Uvero, *J. Bisse & H. Lippold* (HAJB 14474).

**GRAND CAYMAN.** NW of East End Village, *G.R. Proctor & J. Lane* 47346 (FTG).

#### Note.

This species is distinguished by its 3-lobed leaves, which are abaxially velvety pubescent. The corolla is pink.

### 189. Ipomoea hypargyreia

Taxon classificationPlantaeSolanalesConvolvulaceae

Griseb., Cat. Pl. Cub. 204: 1866. (Grisebach 1866: 204)

Ipomoea hypargyreia var. baracoensis Urb., Symb. Antill. 9: 245. 1924. (Urban 1924b: 245). Type. CUBA. Prov. Oriente [Guantánamo], Baracoa, Loma de Cuaba near Pinales, *E.L. Ekman* 3589 (holotype S07-4474).
Ipomoea platyclada Urb., Symb. Antill. 9: 245. 1924. (Urban 1924b: 245). Type. CUBA. Prov. Oriente [Holguín-Guantánamo], Sierra de Nipe, Río Piloto, *E.L. Ekman* 3342 (holotype S07-4475, isotype NY).

#### Type.

CUBA. *C. Wright* 1/69 (holotype GOET000345, isotypes GH, ?HAC).

#### Description.

Perennial herb; stems adpressed pilose, becoming glabrescent. Leaves petiolate, often large, 5–12 × 2–6 cm, ovate, acute or acuminate, mucronate, base cordate with rounded auricles, adaxially green, pubescent, abaxially silver-sericeous; petioles 1–1.8 cm, subsericeous. Inflorescence of leafy, few-flowered axillary cymes; peduncles 1–1.8 cm, grey-canescent; bracteoles leaf-like, petiolate, 20–25 mm, narrowly ovate, acuminate, grey-canescent, deciduous; pedicels 4–6 mm, less canescent than peduncles; sepals slightly unequal, suborbicular, obtuse, mucronulate convex, coriaceous, glabrous, outer 6 × 5 mm, inner 8–9 mm; corolla c. 4 cm long, funnel-shaped, pink, glabrous, limb c. 2.5 cm diam. Capsules glabrous; seeds 5 mm, subglobose, pilose with hairs up to 10 mm long.

#### Distribution.

Apparently endemic to Eastern Cuba.

**CUBA. Guantánamo**: Carretera de Quibiján, Baracoa, *Bro. Alain & M. López* 7119 (HAC, HAJB); Río del Padre, *Bro. B. Hioram* 4243 (HAC); Monte Libano, *E.L. Ekman* 10301 (S); Sierra de Imias, *J. Bisse et al.* HAJB52454); Río Duaba, *J. Bisse et al.* (HAJB39654). **Holguín**: Sierra del Cristal, *E.L. Ekman* 15916 (S); Montes de Gran Tierra, Moa, *J. Acuña* 3320 (HAC); Moa hacia La Melba, *J. Bisse & H. Lippold* (HAJB11379).

#### Note.

This species is characterised by its large, ovate, abaxially sericeous leaves, glabrous sepals and pink corolla.

### 190. Ipomoea clarensis

Taxon classificationPlantaeSolanalesConvolvulaceae

Alain, Mem. Soc. Cuba Nat. Hist. Felipe Poey 22: 121. 1955. (Liogier 1955: 121)

#### Type.

CUBA. [Villa Clara], Santa Clara, Loma de la Gloria, Banao Mts, 30 July 1918, Bro. *León & Roca* 7959 (holotype HAC, isotype NY).

#### Description.

Twining perennial, stems pilose, eventually glabrescent. Leaves petiolate, large, 4–16 × 2.5–8.5 cm, deltoid, sometimes 3-lobed, acuminate to an acute or obtuse, mucronate apex, base weakly cordate, appressed pilose on both surfaces, paler beneath; petioles 1.5–5 cm, pubescent. Inflorescence of usually 3-flowered axillary cymes; peduncles 3–7 cm, glabrous; secondary peduncles 1–2 cm; bracteoles linear, 2–3 mm, caducous; pedicels 8–14 mm, thickened upwards; sepals subequal, 9 × 6 mm, elliptic, obtuse to rounded, convex, reddish with white scarious margins, glabrous; corolla 3.5–4.5 cm long, subhypocrateriform, the tube cylindrical, expanded into a limb c. 1 cm long and 2–3 cm diam. at apex, dark red, glabrous. Capsules 9 × 5 mm, ellipsoid, rostrate, glabrous; seeds long-pilose, c. 8 mm in length.

#### Distribution.

Endemic to mountains in central Cuba.

**CUBA. Villa Clara**: Trinidad Mountains, *R.A. Howard* 6465 (A, BM, NY, S); Loma de Ponciano, Sancti-Spiritus, *Bro. León* 6704 (NY); Pico Potrerillo, *Bro. Alain* 6360 (HAC); Topes de Collantes, Trinidad [Sancti Spiritus], *Bro. León & M. Victorin* 19065 (HAC, NY); Sierra de Escambray, 5 km al S de topes de Collantes, *J. Bisse & H. Lippold* (HAJB9732). **Cienfuegos**: Complejo San Juan, Cumanayagua, *R. Oviedo et al*. s.n. [2/11/1986] (HAC), s.n. [3/11/1986] (HAC); ibid., *L. González et al*. (HAJB60249).

#### Note.

This species is distinguished by the pubescent leaves and glabrous, red corolla.

### s191. Ipomoea incerta

Taxon classificationPlantaeSolanalesConvolvulaceae

(Britton) Urb., Symb. Antill. 9: 247. 1924. (Urban 1924b: 247)

Exogonium incertum Britton, Mem. Torrey Bot. Club. 16: 94. 1920. (Britton 1920: 94). Type. CUBA. Holguín, J.A. Shafer 1235 (holotype NY00111064, isotype NY).

#### Description.

Twining perennial, largely leafless when flowering; stems wiry, grey, glabrescent. Leaves shortly petiolate,1.5–2 × 0.6–0.6 cm, oblong, obtuse, cuneate at base, glabrous, gland-dotted on both surfaces; petioles 3 mm. Inflorescence borne on short lateral woody shoots, ± racemose in structure, rhachis 1–2 cm, glabrous; bracteoles not seen; pedicels 5–8 mm; sepals subequal, 5–6 mm long, glabrous, convex, coriaceous, outer elliptic, obtuse, inner suborbicular, rounded with broader scarious margins; corolla 3–3.5 cm long, ± cylindrical, the limb only c. 10 mm diam., dark red, glabrous. Capsules ovoid, glabrous, much exceeding calyx; seeds with long woolly hairs.

#### Distribution.

Endemic to the hills surrounding Holguin in eastern Cuba. Apparently very rare and known from very few collections.

**CUBA. Holguin**: Lomas que rodean Holguín, *M. López Figueiras* 934 (HAJB).

#### Note.

A little-known species characterised by its glabrous oblong leaves. The plant is leafless when flowering and the corolla is subhypocrateriform.

### 192. Ipomoea argentifolia

Taxon classificationPlantaeSolanalesConvolvulaceae

A. Rich. ex Sagra, Hist. Fis. Cuba, Bot. 3: 131. 1850. (Sagra 1850: 131)

Exogonium argentifolium (A.Rich. ex Sagra) House, Bull. Torrey Bot. Club. 35(3): 102. 1908. (House 1908a: 102).

#### Type.

CUBA. Isla de Pinos [Isla de la Juventud], *M.R. de la Sagra* 1689 (holotype P00622212).

#### Description.

Perennial liana, stems woody, floccose. Leaves often absent at anthesis, shortly petiolate, 3.5–11 × 1–4 cm, thick in texture, oblong or obovate-oblanceolate, apex cuneate and with prominent stout mucro, base narrowly cuneate, densely tomentose on both surfaces, grey adaxially, white abaxially; petioles 8–20 mm, white-tomentose. Inflorescence on short leafy axillary branchlets 2–8 cm long; bracteoles 5–6 mm, linear-oblanceolate, tomentose, caducous; pedicels 5–14 mm, pilose to tomentose; sepals subequal, 10–11 × 6–7 mm, elliptic, obtuse, densely tomentose; corolla 3.5–4.5 cm long, hypocrateriform, basal cylindrical tube 3–3.5 × 0.5–0.6 cm, dark red, limb, 1.5–2 cm diam., red, glabrous, stamens exserted.

#### Illustration.

Wood and Scotland (2017c: 6).

#### Distribution.

Endemic to Cuba, growing in woodland in the extreme east and extreme west of the island.

**CUBA. Granma**: Media Luna, Niquero, *R. Alonso* 13598 (HAC), 20502 (HAC). **Holguín**: Sierra de Nipe, *E.L. Ekman* 10136 (NY, S); 9554 (S); 3080 (S); *J. Bisse et al.* (HAJB36052); *M. López Figuieras* 1744 (HAC, HAJB). **Isla de la Juventud [I. de Los Pinos**]: *C. Wright* 449 (HAC, K); *N.L. Britton et al.* 14353 (NY); *E.L. Ekman* 12222 (S), 12116 (S); *Bro. Alain & E.P. Killip* 2078 (HAC); *A.H. Curtiss* 489 (NY); *E.P. Killip* 45793 (US). **Santiago de Cuba**: Rente, Bahia de Santiago, *Bro. Clemente* 2570 (HAC); Sierra Santa María del Loreto, *M. López Figuieras 317*, 3021 (HAJB); Ocujal, *J. Bisse & H. Lippold* (HAJB14078); Entre el Cuero y Nima-Nima, *M. López Figuieras* 970 (HAC, HAJB).

#### Note.

The sepals are obtuse, noticeably longer than broad and the inflorescence is borne on leafy side branches. The corolla limb is broader (2–2.5 cm) than in *Ipomoea praecox*. Also noteworthy are the cuneate leaf base, white-tomentose leaves and white-tomentose sepals.

It was recorded in error from Mexico (Villaseñor 2016: 702).

### 193. Ipomoea praecox

Taxon classificationPlantaeSolanalesConvolvulaceae

Wright, Anales Acad. Cien. Med., Habana 7: 46. 1870. (Sauvalle 1870: 46)

#### Type.

CUBA. [Pinar del Río], Lomas de Rangel, *C. Wright* 3646 [No. 1653 in Sauvalle 1870] (lectotype HAC, designated by Wood and Scotland (2017: 4), possible isolectotypes GH, K, NY).

#### Description.

Twining perennial of unknown size; stems densely white-villous, somewhat glabrescent. Leaves absent at flowering, petiolate, 2.5–5.3 × 1.6–2.4 cm, ovate, cordate, apex rounded to retuse, mucronate, both surfaces tomentellous but abaxially grey; petioles 8–10 mm. Inflorescence of very shortly pedunculate, rather dense, up to 6-flowered cymes, often racemose in form, the peduncle forming the rhachis of the raceme; peduncles 4–20 mm (but < 7 mm to first bracteole), villous; bracteoles 7 × 1.5 mm, narrowly oblong, acute; secondary peduncles 2–3 mm; pedicels 7–10 mm; sepals subequal, suborbicular to broadly obovate, 7–8 × 5–7 mm, slightly enlarging in fruit, reddish, lanate below, glabrous above; corolla 3–3.5 cm long, red, glabrous, hypocrateriform with cylindrical tube; limb 1.5–2 cm. Capsules glabrous, ovoid; seeds 5–6 × 3–4 mm with long white marginal hairs.

#### Illustration.

Wood and Scotland (2017c: 5).

#### Distribution.

Endemic to western Cuba, where it is characteristic of limestone mogotes.

**CUBA. Pinar del Río**: Santa Cruz de los Pinos, *Bro. León* 22872 (HAC); ibid., *Bro. Alain* 466 (HAC, HAJB); La Palma, Loma Peluda de Cajalbana, *J. Bisse & H. Lippold* s.n. (HAC); Bahia Honda, Finca Toscano, *J. Bisse & H. Lippold* (HAJB18678); Candelaria, Sierra del Rosario, Loma Pelada de Cayajabos (del Mulo), *J. Bisse et al.* (HAJB48979); Las Villas, Soledad, *A. Gonzáles* 554 (BM).

#### Note.

The type of *Ipomoea praecox* is leafless. It also differs from *Ipomoea argentifolia* in the smaller suborbicular, upwardly glabrous sepals.

### 194. Ipomoea calophylla

Taxon classificationPlantaeSolanalesConvolvulaceae

C. Wright ex Griseb., Cat. Pl. Cub. 204. 1866. (Grisebach 1866: 204)

Ipomoea lacteola House, Ann. New York. Acad. Sci. 18(6): 229. 1908. (House 1908b: 229), nom. superfl. Type based on I. calophylla C. Wright ex Griseb.

#### Type.

CUBA. *C. Wright* 3098 [1651] (holotype GOET000348, isotypes BM, G, GH, HAC, K, S, US, YU).

#### Description.

Climbing perennial; stems tomentose, twining when young, eventually woody. Leaves petiolate, 0.8–4.2 × 1–1.8 cm, oblong or oblong-ovate, obtuse to retuse, mucronate, base truncate to cordate, adaxially green, tomentellous, abaxially white-tomentose; petioles 0.6–1.8 cm, tomentose. Inflorescence of solitary flowers, usually developing on short dense bracteate lateral branches, the bracts resembling small leaves; peduncles up to 1–1.3 cm, tomentose; bracteoles 4–9 × 1–2 mm; filiform, tomentose; pedicels 2–4 mm; sepals 10–16 × 7–9 mm, broadly oblong-elliptic, obtuse, tomentose; corolla 5–5.5 cm long, funnel-shaped, glabrous, pale pink, limb shallowly lobed, 4 cm diam.; stamens unequal, included. Capsules c. 11 × 6 mm, ovoid, glabrous; seeds 5–6 × 4 mm, long-pilose with hairs to 10 mm long.

#### Distribution.

Endemic to Cuba and restricted to woodland in the west.

**CUBA. Pinar del Río**: *Bro. Alain & J. Acuña* 2296 (HAC); *Bro. León* 13206 (HAC, HAJB); *Van Hermann* 15536 (HAC).

#### Notes.

The corolla is larger and more funnel-shaped than in *Ipomoea argentifolia* and *I. fuchsioides* and the stamens are included. It is also similar to *Ipomoea jalapoides* but the stamens are included and the sepals are also larger. The short dense lateral flowering branchlets are very characteristic.

For discussion about the use of the name *Ipomoea calophylla*, see Wood and Scotland (2017c).

### 195. Ipomoea jalapoides

Taxon classificationPlantaeSolanalesConvolvulaceae

Griseb., Cat. Pl. Cuba 202. 1866. (Grisebach 1866: 202)

Exogonium jalapoides (Griseb.) House, Bull. Torrey Bot. Club. 35(3): 101. 1908. (House 1908a: 101).

#### Type.

CUBA. “Occ.”, Wright 3097[1636] (holotype GOET000344, isotypes GH, HAC, K, NY, S, US, YU).

#### Description.

Perennial, probably twining herb; stems herbaceous, white-tomentose. Leaves petiolate, 2–4.3 × 0.7–1.8 cm, narrowly ovate, sometimes 3-lobed with long central lobe, apex acute to shortly acuminate and strongly mucronate, base cordate with rounded auricles, adaxially grey canescent, abaxially white-tomentose; petioles 7–14 mm, tomentose. Inflorescence of few-flowered, leafy axillary cymes; peduncles 1–2.8 cm, tomentose; bracteoles 4–5 mm, linear-lanceolate, tomentose; pedicels 6–9 mm, densely tomentose; sepals subequal, outer 8–10 × 5 mm, elliptic, obtuse, tomentose, inner, glabrous except for tomentose central area, the margins scarious; corolla 5–5.5 cm long, narrowly funnel-shaped, basal tube only slightly widened upwards c. 1 cm, dark red, limb broad, 2–3 cm diam., red, glabrous. Capsules ovoid, 10 × 7 mm, glabrous; seeds 5 × 3 mm, pilose with long marginal hairs.

#### Distribution.

Endemic to CUBA, apparently only known from the type collection.

#### Note.

The leaf base is cordate to truncate and the leaves are sometimes 3-lobed. The corolla is longer than in *Ipomoea argentifolia* and *I. fuchsioides*.

### 196. Ipomoea montecristina

Taxon classificationPlantaeSolanalesConvolvulaceae

Hadač, Folia Geobot. Phytotax. 5: 430. 1970. (Hadač 1970: 430)

#### Type.

CUBA. provincia Oriente, “montibus Montecristo dictis alt. circ. 800 m s. m., solo “laterit” dicto, legi 27.1.68”, *Hadač* 1279, (holotype PR).

#### Description.

Twining perennial; stems sericeous, somewhat woody, and wiry. Leaves shortly petiolate, 2.3–6.5 × 0.8–3.2 cm, oblong-ovate, base cuneate to weakly cordate, apex acute and shortly mucronate, adaxially dark green, densely pubescent, abaxially densely grey-velutinous, shiny; petioles 3–8 mm, sericeous. Inflorescence of pedunculate axillary cymes with up to 12 flowers; peduncles 1.4–3 cm, grey-tomentose; bracteoles linear, 3–6 × 1 mm, densely tomentose; secondary peduncles 3–12 mm, tomentose; pedicels 4–7 mm, thickened upwards and becoming less tomentose; sepals subequal, outer 5–6 × 3–4 mm, pubescent towards base, glabrescent, inner 6–8 × 4 mm, ovate, obtuse to rounded, reddish-brown, coriaceous, glabrous, margin narrow, palid; corolla 3–3.5 cm long, pink, glabrous, narrowly funnel-shaped; limb c. 1.5 cm diam.; stamens included. Capsules 10–11 × 5–6 mm, ovoid, glabrous, muticous; seeds 5 × 3 mm, blackish, with long marginal hairs up to 10 mm long.

#### Illustration.

Wood and Scotland (2017c: 8).

#### Distribution.

Endemic to Eastern Cuba, perhaps limited to Guantánamo, where it grows on limestone mountains.

**CUBA. Holguín**: Región (Pinares) de Moa, Baracoa, *Bro. León* 21291 (HAC); **Guantánamo**: San Antonio del Sur, *J. Bisse et al.* (HAJB29883, HAC); *A. Álvarez et al.* (HAJB43089); ibid., *J. Bisse et al.* (HAJB48105); subida hacia la zona de Monte Libano, J. *Bisse & E. Köhler* (HAJB7924); Felicidad de Yateras, pinar de la zona de Monte Cristi, *J. Bisse* (HAJB 20234); ibid., *J. Bisse & A. Alvarez* (HAJB43272); ibid., *J. Bisse et al.* (HAJB49387); Jamaica, Monte Cristi, *J. Bisse et al.* (HAJB39180).

#### Note.

The combination of red corolla, near glabrous sepals and the shiny-silvery sericeous indumentum render this species relatively distinct.

### 197. Ipomoea fuchsioides

Taxon classificationPlantaeSolanalesConvolvulaceae

Griseb., Cat. Pl. Cub. 205. 1866. (Grisebach 1866: 205)

Exogonium fuchsioides (Griseb.) House, Bull. Torrey Bot. Club. 35: 101. 1908. (House 1908a: 101).
Ipomoea fuchsioides var. parvifolia Griseb., Cat. Pl. Cub. 205. 1866. (Grisebach 1866: 205). Type. CUBA. *C. Wright* 3095 (holotype GOET005700, isotypes BM, G, GH, HAC, K, MA, MO, YU).
Ipomoea arnoldsonii Urb., Symb. Antill. 9: 424. 1925. (Urban 1925: 424). Type. CUBA. Pinar del Río, Viñales, *E.L. Ekman* 18029 (holotype S07-4319).

#### Type.

CUBA. “Occ.”, *C. Wright* [655] (lectotype GOET002513, designated here; isolectotypes, GH, GOET, YU).

#### Description.

Slender twining herb, stems scabrous to pilose. Leaves petiolate, 1.2–5.5 × 0.4–2.2 cm, narrowly to broadly ovate-deltoid, acute, mucronate, base truncate to shallowly cordate, pubescent on both surfaces, abaxially much paler; petioles 0.3–0.5 mm, pubescent. Inflorescence of shortly pedunculate, few-flowered axillary cymes, sometimes aggregated into small panicles on short branchlets; peduncles 0.3–0.6 cm; bracteoles caducous; pedicels 5–8 mm; sepals subequal, coriaceous, glabrous, reddish-brown with scarious margins, outer 4–5 × 2.5 mm, elliptic to suborbicular, obtuse to rounded, inner similar but 5–6 mm long; corolla 2.5–4 cm long, salver-shaped, dark red, glabrous, the cylindrical tube slightly widened below limb, limb 2 cm diam. Capsules suborbicular, 5–6 × 4 mm, glabrous; seeds (immature) densely pilose with long hairs.

#### Distribution.

Endemic to western Cuba and apparently characteristic of limestome mogotes.

**CUBA. Isla de la Juventud [Pinos**]: *E.L. Ekman* 12354 (S), 11822 (S). **Pinar del Río**: Tumidero, *J.A. Shafer & Bro. León* 3423 (HAC); Guanajay Mountain, *P. Wilson* 1789 (HAC); La Cajálbana, La Palma, *Bro. Alain & J. Acuña* 1224 (HAC); ibid., *J. Bisse & H. Lipold* (HAJB18301); *A. Alvarez al.* (HAJB51236); Bahía Honda, *Bro. León* 12554 (HAC); ibid., *A. Alvarez et al.* (HAJB51223); ibid., al norte del Pan de Guajaibón, *J. Bisse* (HAJB9619); Mogote del Queque, Viñales, *Bro. Alain* 3522 (HAC).

#### Note.

Plants treated as Ipomoea fuchsioides var. glabra with glabrous leaves are, in our opinion, *Ipomoea microdactyla*.

### 198. Ipomoea microdactyla

Taxon classificationPlantaeSolanalesConvolvulaceae

Griseb., Cat. Pl. Cub. 204. 1866. (Grisebach 1866: 204)

Exogonium microdactylum (Griseb.) House, Bull. Torrey Bot. Club. 35(3): 102. 1908. (House 1908a: 102).
Ipomoea repanda var. microdactyla (Griseb.) D. Powell, J. Arnold Arbor. 60(2): 259. 1979. (Powell 1979: 259).
Ipomoea fuchsioides Griseb. var. glabra Griseb., Cat. Pl. Cub. 205. 1866. (Grisebach 1866: 205). Type. CUBA. *C. Wright* [134] 1865 (holotype GOET 005701).
Ipomoea repanda var. pratensis C. Wright ex Griseb., Cat. Pl. Cub. 204. 1866. (Grisebach 1866: 204). Type. CUBA. *C. Wright*s.n. (holotype GOET 005699, isotypes GH, K, YU).
Ipomoea repanda var. undulata C. Wright ex Griseb., Cat. Pl. Cub. 204. 1866. (Grisebach 1866: 204). Type. CUBA. *C. Wright*s.n. (holotype GOET 005698).
Exogonium microdactylum var. integrifolium House, Bull. Torrey Bot. Club. 35(3): 103. 1908. (House 1908a: 103). Type. CUBA. *C. Wright* 3102[1654] (possible holotype NY00111066, isotype K).
Ipomoea beyeriana Urb., Symb. Antill. 9: 425. 1925. (Urban 1925: 425). Type. CUBA. Pinar del Río, km 13 on highway to Coloma, *E.L. Ekman* 18234 (holotype S07-4420).

#### Type.

CUBA. *C. Wright* 3094[1655] (holotype GOET 002497, isotypes BM, GH, HAC, K, MO, NY, S, US, YU).

#### Description.

Twining perennial herb with tuberous rootstock, apparently lacking white latex, stems glabrous, pale brown, somewhat woody. Leaves petiolate, polymorphic, 1–4 × 0.5–2.5 cm, usually broadly to narrowly deltoid, shortly acuminate, mucronate, basally truncate to subcordate, sometimes 3–5 lobed with lobes ±oblong, margin often undulate and sublobed, glabrous, abaxially paler and often with prominent veins; petioles 1–2.7 cm. Inflorescence of solitary or paired (rarely twice paired to 4 or more and becoming subracemose) pedunculate flowers; peduncles 1–2 (– 4.5) cm; bracteoles caducous, not seen; secondary peduncles 5–10 mm; pedicels 5–15 mm; sepals slightly unequal, glabrous with broad scarious margins, coriaceous, outer 5 mm, obovate-suborbicular, rounded, inner 6 mm, broadly oblong-obovate; corolla 3.5–4 cm long, red, glabrous, tube subcylindrical but slightly widened upwards, often curved, limb c. 3.5 cm, diam., shallowly lobed; stamens exserted. Capsules ovoid, 5–12 × 5–10 mm, shortly rostrate, glabrous; seeds 6 × 3–4 mm, pilose with long hairs up to 10 mm.

#### Illustration.

Figure 94E; Acevedo-Rodríguez (2005: 172).

#### Distribution.

Common in dry woodland and secondary scrub in the Bahamas and Cuba and with isolated populations in Florida and on Mona Island, Puerto Rico.

**UNITED STATES. Florida**: *Rugel* s.n. (BM); *J.K. Small et al.* 6452 (S), 7943 (S); Dade Co, *L.J. Brass* s.n. (ARCH).

**BAHAMAS.** Acklins Island, *H.F.A. von Eggers* 3965 (BM, K); Andros: *J.I. & A.R. Northrop* 394 (K, NY); ibid., *J.K. Small & J.J. Carter* 8752 (K, NY). Grand Bahama: *W.H. Lewis* 7176 (MO). Berry Island: *N.L. Britton & C.F. Millspaugh* 2336 (NY). Governor Harbour: *N.L. Britton & C.F. Millspaugh* 5504 (NY). New Providence: *A.H. Curtiss* 211 (K, MO, NY). San Salvador: *D.S. Correll & D.C. Wasshausen* 46862 (NY). Watlings Island: *P. Wilson* 7212 (K, NY).

**TURKS & CAICOS ISLANDS.** Middle Caicos, *B.J. Pollard et al.* 1354 (K); Salt Cay, *B.N. Manco et al.* 419 (K); North Caicos, *D.S. Correll* 49469 (NY); *P. Wilson* 7716 (K, NY); South Caicos: *D.S. Correll* 49280 (MO, NY).

**CUBA. Camagüey**: *N.L. Britton et al.* 13253 (NY). **Cienfuegos**: Castillo de Jagua, *R. Combs* 609 (K). **Guantánamo**: Fisherman’s Point, *N.L. Britton et al.* 2107 (NY). **Isla de Juventud (Pinos)**: *N.L. Britton et al.* 14288 (NY). **La Habana**: Mayabeque, *Bro. Alain* 1963 (NY). **Matanzos**: Conabi, *Bro. León* 13126 (NY). **Pinar del Río**: *J.A. Shafer* 11777 (MO). **Santiago de Cuba**: *M. López Figueiras* 909 (HAJB). **Villa Clara**: *Bro. León* 11350 (NY); *A. Luna* 802 (NY).

**PUERTO RICO.** Mona Island: *R.O. Woodbury et al.* M81 (NY).

#### Notes.

*Wright* 3094, *Shafer* 2607 from Camaguey and sine data from Bahamas (K) have 3–5 lobed leaves. Other specimens have entire leaves.

*Ipomoea beyeriana* is only known from the type. We believe it is an entire-leaved form of *I. microdactyla* Griseb., based on the leaf shape and the reddish sepals. Urban (1925: 425) suggested an affinity with *Ipomoea fuchsioides* and it is not unlike the type of I. fuchsioides var. glabra and is an even better match for *Wright* 3102, the type of Exogonium microdactylum var. integrifolium House. (Wood and Scotland 2017c).

### 199. Ipomoea repanda

Taxon classificationPlantaeSolanalesConvolvulaceae

Jacq., Enum. Syst. Pl. 13. 1760. (Jacquin 1760: 13)

Convolvulus repandus (Jacq.) Desr., Encycl. 3: 555. 1789 [pub. 1792]. (Desrousseaux 1792: 555).
Exogonium repandum (Jacq.) Choisy, Mém. Soc. Phys. Genève 8: 128[50]. 1838. (Choisy 1838: 128[50]).
Quamoclit repanda (Jacq.) Roberty, Candollea 14: 41. 1952. (Roberty 1952: 41).
Ipomoea eriosperma Berthel. ex Spreng., Syst. Veg. 1: 598. 1825 [pub. 1824]. (Sprengel 1824: 598), nom. illeg., non Ipomoea eriosperma P. Beauv. (1819). Type. GUADELOUPE. *C.L.G. Bertero*s.n. (lectotype TO, isolectotype M0184975).

#### Type.

Icon, t. 20 in Jacquin, Sel. Stirp. Amer. (1763), designated by Austin (1978a: 336).

#### Description.

Twining liana to several metres; stems woody, glabrous, pale, reported to have abundant white latex. Leaves petiolate, 5–13 × 3–10 cm, deltoid, base rounded, truncate or cordate, apex acuminate, margin undulate, both surfaces glabrous; petioles 2–9.5 cm. Inflorescence of pedunculate axillary cymes; peduncles 2.5–7 cm; bracteoles caducous; secondary peduncles 12–15 mm; pedicels 5–15 mm; sepals subequal, 6–7(–8) × 4 mm, ovate to suborbicular, obtuse (outer) to rounded (inner), reddish, glabrous, the margins scarious; corolla 4–4.5 cm long, subcylindrical but slightly widened upwards, curved, red, glabrous, limb deeply divided with oblong, apiculate lobes c. 3–4 × 1.5 cm; stamens shortly exserted. Capsules ovoid, 14 × 8 mm, shortly rostrate, glabrous; seeds 8 × 5 mm, shortly pilose on the margins.

#### Illustration.

**Figure 103.**
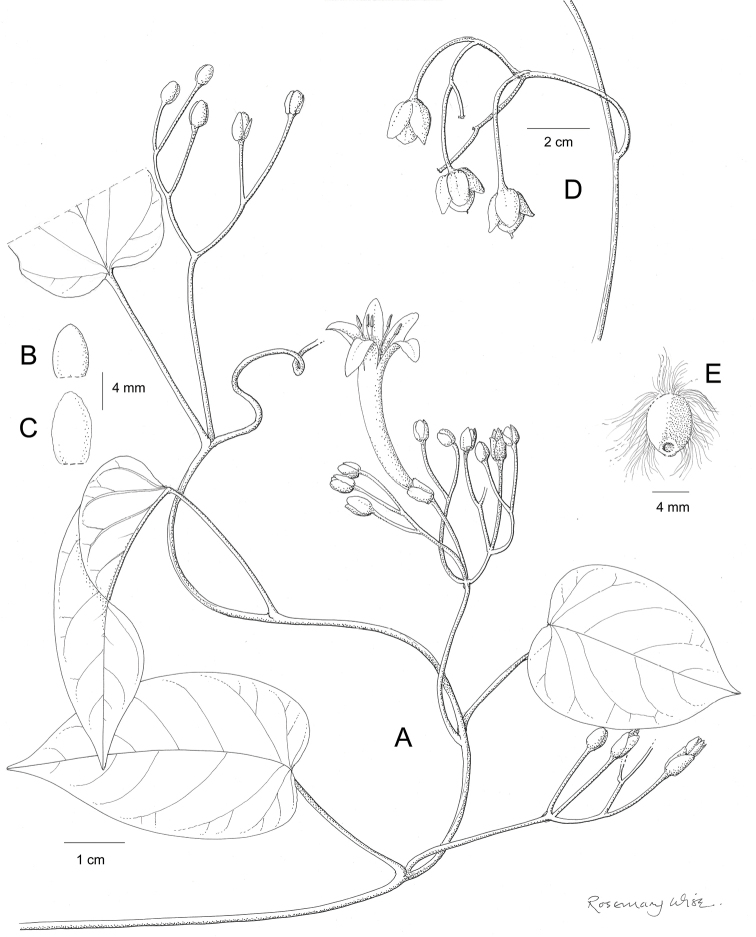
*Ipomoea repanda*. **A** habit **B** outer sepal **C** inner sepal **D** fruiting inflorescence **E** seed. Drawn by Rosemary Wise from *Whitefoord* 5244.

Figures 8B, 103; Acevedo-Rodríguez (2005: 176).

#### Distribution.

Widely distributed from the eastern part of Hispaniola through Puerto Rico and south through the Windward Islands to Tobago. It grows in moist forest and around mogotes. Absent from Barbados and Trinidad.

**DOMINICAN REPUBLIC.** Peninsula Samaná, Pan de Azúcar, *E.L. Ekman* H15176 (S); *A.H. Liogier* 5 (P).

**PUERTO RICO.***F. Axelrod & L. Pérez* 8375 (K); Maricao, *P. Sintenis* 289 (K, BM, P, S); Sierra de Luquillo, *R.A. Howard* 16812 (A, BM, MO, P, S); *T.G. Hartley* 13328 (MO, P).

**LESSER ANTILLES. U.S. Virgin Islands**: St John, *G. Prance et al.* 29343 (BM, NY); ibid., *P. Acevedo-Rodríguez* 3123 (MO, NY), 2839 (NY); St Thomas, *H.F.A. von Eggers* 253 (K); ibid., *C.H. Ostenfeld* 150 (C, P). **U.K. Virgin Islands**: Tortola, *C. Clubbe* 15 (K); *Fishlock* 339 (K). **Barbuda**: fide Powell (1979). **Antigua**: *Wullschlägel* s.n. (S); Monks Head Hill, *H.E. Box* 1280 (BM, MO). **Montserrat**: Jubilee Mountain, *R.A. Howard* 19665 (BM, NY); *M.A. Hamilton et al.* 403 (K). **Guadeloupe**: *A. Duss* 2478 (MO, NY); *C. Sastre et al.* 4249 (P). **Dominica**: Glasham, *D.H. Nicolson* 2089 (BM, US); *C. Whitefoord* 5244 (BM); *S.R. Hill et al.* 25544 (NY). **Martinique**: *L. Hahn* 536 (BM, K, P); *A. Duss* 1890 (NY); *C. Sastre* 6570 (P). **St Lucia**: *P. Beard* 1068 (MO, S); Morne Tabac, *G.R. Proctor* 21573 (BM); *R.A. Howard* 19915 (NY). **St Vincent**: *P. Beard* 1354 (MO, S); *H.H. & G.W. Smith* 1301 (K, NY); Bequia fide Powell (1979). **Grenada**: *K. Barbour et al.* 93425 (BM); Mt. St. Catherine, *G.R. Proctor* 17257 (BM).

**TRINIDAD. Tobago**: *J. Greg* (BM).

#### Note.

Very similar to *Ipomoea microdactyla* but sepals 6–7(–8) mm, corolla narrowly tubular, curved. limb < 1 cm long, deeply lobed with oblong-ovate, acute lobes.

### 200. Ipomoea sphenophylla

Taxon classificationPlantaeSolanalesConvolvulaceae

Urb., Symb. Ant. 5: 474 (1908). (Urban 1908: 474)

#### Type.

NETHERLANDS ANTILLES. St. Eustatius. Signal Hill, no collection cited; neotype. East boundary of Statia Terminals N.V., on the northwest side of Mary’s Glory, Oct. 27, 1994, *Jan Faber* s.n. (A), designated by Howard & McDonald 1995).

#### Description.

Robust liana to 8 m from a napiform rootstock with pendent glabrous stems. Leaves petiolate, 3–7 × 1–2 cm, oblanceolate to obovate, obtuse or truncate and mucronate, basally cuneate and attenuate onto the petiole, coriaceous, glabrous; petioles 1–1.5 cm. Inflorescence a simple or compound cyme with up to 5 flowers; peduncles 1–1.8 cm; bracteoles not known; secondary peduncles more slender, 5–20 mm; pedicels 20–30 m; sepals glabrous, pink, unequal, outer 5–7 mm, elliptic, inner 6–8 mm, ovate; corolla 2.2–2.5 cm long, funnel-shaped, glabrous, lavender, limb 2–2.5 cm diam., rotate, 10-lobate; stamens held at mouth. Capsules globose, 6–7 mm diam., glabrous; seeds 4 mm long, dark brown-pilose with hairs 7–8 mm long.

#### Distribution.

Endemic to the islands of St. Eustatius and (fide Axelrod 2017) Saint Barthélemy.

**NETHERLANDS ANTILLES.** St Eustatius. *I. Boldingh* 1038 (K, NY); *B.M. Boom* 11296 (NY).

#### Note.

Resembles *Ipomoea repanda* but the leaves are of a distinctive obcuneate shape and the stamens not fully exserted. Further details of this species are provided by Bush and Madden (2012).

• Species 201–204. These four species are characterised by their small leaves which develop on brachyblasts.

### 201. Ipomoea microdonta

Taxon classificationPlantaeSolanalesConvolvulaceae

J.R.I. Wood & Scotland, Kew Bull. 72(45): 8. 2017. (Wood and Scotland 2017c: 8)

Ipomoea cavanillesii sensu Sauvage (1870) and Sauget and Liogier (1957).

#### Type.

CUBA. Camargüey, 2–7 April 1912, *N.L. Britton, E.G. Britton & J.F. Cowell* 13178 (holotype NY, isotype MO).

#### Description.

Slender twining perennial; stems thin, wiry, woody, minutely asperous. Leaves petiolate, borne on small brachyblasts, 3-foliate, leaflets 3–10 × 1–5 mm, obovate-oblanceolate, apex obtuse to retuse, base cuneate, margin undulate, adaxially thinly hirsute, abaxially glabrous; petioles 2–8 mm. Inflorescence of solitary, axillary, pedunculate flowers; peduncles short, 1–2 mm; bracteoles caducous; pedicels 4–6 mm, glabrous; sepals unequal, outer 4–5 × 3 mm, elliptic-obovate, obtuse, smooth, glabrous, margins scarious, inner 6–7 × 4 mm, elliptic, rounded; corolla pink, funnel-shaped, glabrous, c. 3 cm long; limb 1–1.5 cm diam., stamens and style included. Capsules c. 9 × 6 mm, ovoid, rostrate, glabrous; seeds 4 × 2.5 mm, blackish, glabrous but with dense long marginal hairs 5–10 mm in length.

#### Illustration.

Figure 104.

**Figure 104.**
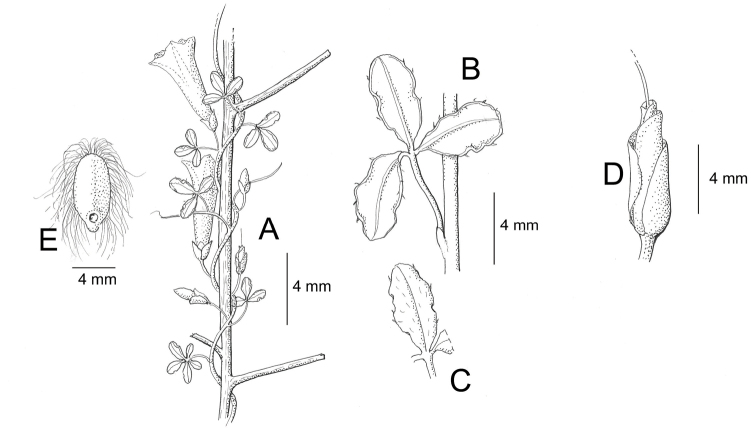
*Ipomoea microdonta*. **A** habit **B** leaf **C** terminal leaflet **D** calyx **E** seed. Drawn by Rosemary Wise from *N.L. Britton et al.* 13178.

#### Distribution.

Endemic to Cuba growing in sandy plain near Camagüey.

**CUBA.** Sine loc., *C. Wright* 3086 (K). **Camargüey**: La Ciega, Caobillas, *J. Acuña* 1540 (HAC); Sabana de Croms, *Bro. León & M. Victorin* 17641 (HAC); Guaímaro, al norte de Monte Grande, *R. Berzaín et al.* (HAJB31501).

#### Note.

This species has been identified as *Ipomoea cavanillesii*, a synonym of *I. cairica*. It has nothing to do with *I. cairica* and from the structure of the sepals and the leaf shape, it is probably closest to *Ipomoea eggersiana*.

### 202. Ipomoea eggersiana

Taxon classificationPlantaeSolanalesConvolvulaceae

A. Peter Die Natürlichen Pflanzenfamilien 4 (3a): 30. 1897 [pub. 1891]. (Peter 1891: 30)

Exogonium eggersii House, Bull. Torrey Bot. Club 35: 104. 1908. (House 1908a: 104). Type. U.S. VIRGIN ISLANDS, St Thomas, Feb. 1887, H.F.A. von Eggerss.n. (holotype NY00111063, isotypes G, L).
Ipomoea eggersii (House) D.F. Austin, Ann. Missouri Bot. Gard. 64: 335. 1978. (Austin 1978a: 335)

#### Type.

U.S. VIRGIN ISLANDS, St Thomas, *H.F.A. von Eggers* 252 (lectotype GOET005714, designated by Staples et al. 2012: 674, isolectotypes G, K, NY, P).

#### Description.

Twining herb; stems somewhat woody, glabrous, roots tuberous, turnip-like, white latex abundant. Leaves clustered on brachyblasts, petiolate, very small, 0.5–0.9 × 0.3–0.7 cm, reniform, bilobed or digitately 3-lobed with the apical lobe bilobed, base truncate, lobes obtuse, glabrous, abaxially paler; petioles 0.3–09 cm. Inflorescence of solitary flowers or several in a raceme-like inflorescence up to 2.5 cm long; peduncles 2–3 mm; bracteoles minute, caducous; pedicels 5–7 mm; sepals glabrous, slightly unequal, outer 4–5 mm, oblong-ovate, rounded, scarious-margined, inner similar but 5–6 mm; corolla 3.5–4 cm long, broadly funnel-shaped, glabrous, tube greenish, limb lilac or pink, 2.5–4 cm diam. Capsules 11–13 × 6–7 mm, ellipsoid, the style persistent as a mucro, glabrous; seeds 5 × 2.5 mm, pilose with long marginal hairs up to 10 mm.

#### Illustration.

**Figure 105.**
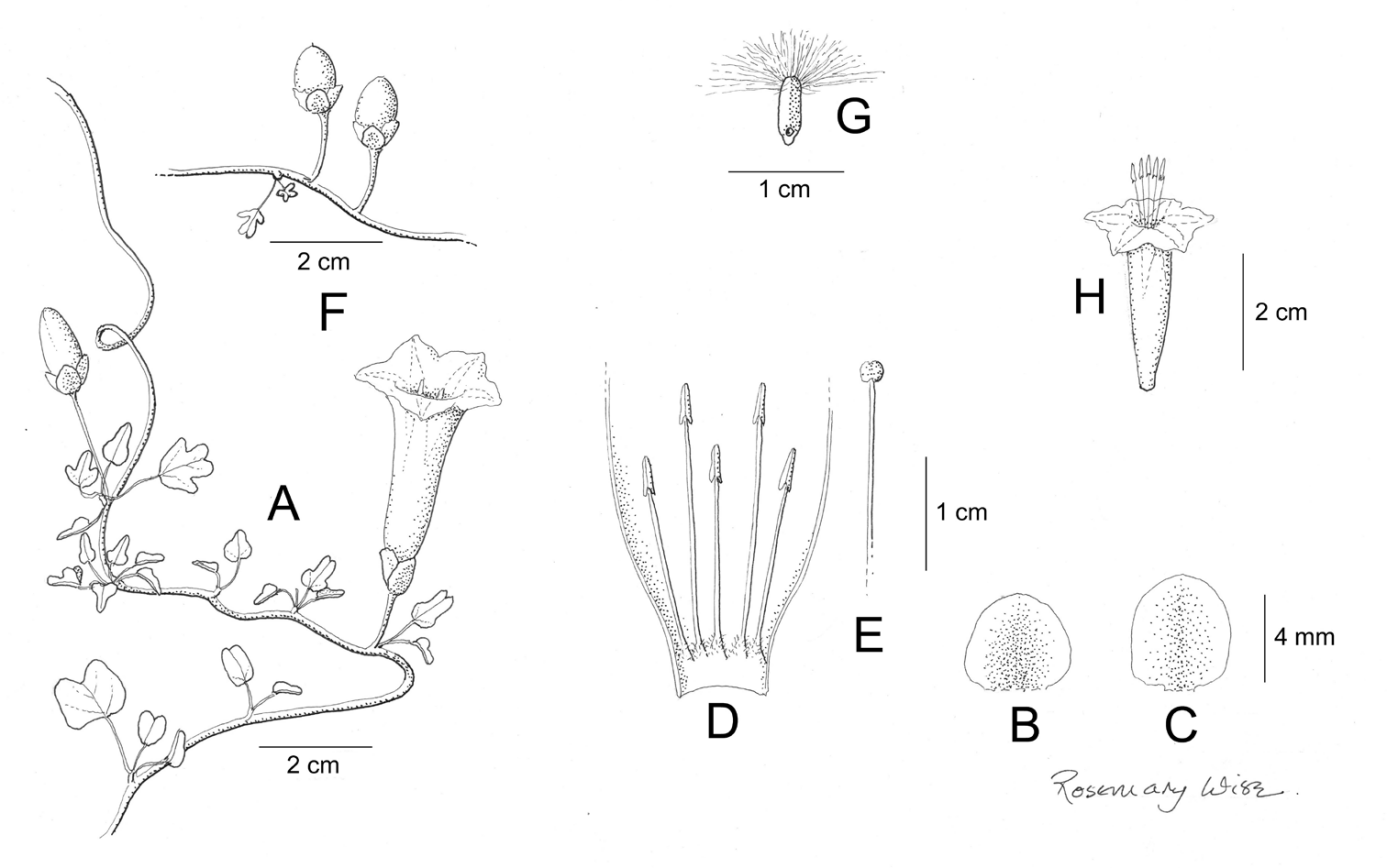
**A–G***Ipomoea eggersiana*. **A** habit **B** outer sepal **C** inner sepal **D** corolla opened out to show stamens **E** style **F** fruiting inflorescence with capsules **G** seed. *Ipomoea steudelii*. **H** corolla. Drawn by Rosemary Wise A from *von Rohr* s.n.; **B–G** from *Urote* 35; **H** from *Drucker* 138.

Acevedo-Rodríguez (2005: 168) (as *Ipomoea eggersii*); Figures 11D, 105A–G.

#### Distribution.

Virgin Islands south east to Barbuda, in scrub near the shore.

**LESSER ANTILLES. U.S. Virgin Islands**: St Croix: Belleview Estate, *R.A. Howard* 15278 (BM); *Ogdon & Wilson* s.n. [18 Jan 1980] (BM); Lang’s Peak, *F.R. Fosberg* 60856 (MO); St Thomas: *Lehmann* 210 (K); *N.L. Britton et al.* 50 (K); Water Island, *R.A. Woodbury* WI-81 (MO, NY), *H.F.A. von Eggers* 529 (P). **U.K. Virgin Islands**: Norman Island: *D.S. & H.B. Correll* 50480 (NY). **Netherlands Antilles**: St Martin: *R.A. Howard* 18373 (A, NY). **Anguilla**: *W. Urote* 35 (BM); *G.R. Proctor* 18542 (BM). **Barbuda**: *Gregory* 1899 (BM).

#### Note.

*Ipomoea eggersiana* forms a species pair with *I. steudelii*, the two species differing only in their corolla shape, colour (crimson in *I. steudelii*, pink in *I. eggersiana*) and distribution. The corolla of *I. eggersiana* is funnel-shaped, whereas that of *I. steudelii* is hypocrateriform with exserted stamens.

### 203. Ipomoea steudelii

Taxon classificationPlantaeSolanalesConvolvulaceae

Millsp., Publ. Field. Columb. Mus., Bot. Ser. 2(1): 86. 1900. (Millspaugh 1900: 86)

Exogonium arenarium Choisy, Mém, Soc. Phys. Genève 8: 129 [51]. 1838. (Choisy 1838: 129[51]). Type. PUERTO RICO. *C.L.G. Bertero*s.n. (lectotype G00135397, designated by Austin 1977: 337).
Ipomoea arenaria (Choisy) Steud., Nomencl. Bot. 1: 815. 1841. (Steudel 1840: 815), non Ipomoea arenaria Roem. & Schult. (1819).
Ipomoea arenaria var. integerrima Kuntze, Rev. Gen. 2: 442. 1891. (Kuntze 1891: 442), nom. illeg., type var.
Ipomoea arenaria var. palmatifida Kuntze, Rev. Gen. 2: 442. 1891. (Kuntze 1891: 442), Type. PUERTO RICO. Guayama (no type specified).

#### Type.

Based on *Exogonium arenarium* Choisy

**Diagnosis.** Almost identical to *Ipomoea eggersiana* in habit, leaves and fruit but corolla crimson, subcylindrical, the limb hypocrateriform, c. 2.5 cm wide, distinctly lobed, the lobes 6–7 mm long, stamens exserted.

#### Illustration.

Acevedo-Rodríguez (2005: 178); Figure 105H.

#### Distribution.

Almost endemic to Puerto Rico but also present on a few small nearby islands and apparently in Haiti, although this is based on an old record that requires confirmation.

**HAITI.***P.A. Poiteau* s.n. (fide Liogier 1994: 92).

**PUERTO RICO.** Santana, *P. Sintenis* 3226 (BM, K, S), 5540 (S); *W. Drucker* 138 (BM); *A.P. Garber* 126 (K); Bayamon, *A.H. Liogier* 10693 (NY), 10699 (NY); Susúa, *A. H. Liogier et al.* 29636 (NY); Caja de Muertos Islands, *R.O. Woodbury et al.* MB202 (MO, NY). Also on adjacent islands of Culebra, Culebrita, Vieques Islands, fide Acevedo-Rodríquez (2005).

### 204. Ipomoea tenuifolia

Taxon classificationPlantaeSolanalesConvolvulaceae

(Vahl) Urb., Symb. Antill. 5: 472. 1908. (Urban 1908: 472)

Convolvulus tenuifolius Vahl, Symb. Bot. 3: 33. 1794. (Vahl 1794: 33). Type. JAMAICA. Sine data (holotype C10009690, possible isotype BM).
Ipomoea fawcettii Urb. ex House in Ann. New York Acad. Sci. 18: 216. 1908. (House 1908b: 216). Type. JAMAICA. Long Mountain, south side, 9 Nov. 1907, *W. Harris* 10010 (holotype NY00111099, isotype BM).

#### Type.

Based on *Convolvulus tenuifolius* Vahl

#### Description.

Twining perennial liana to 3 m; stems woody, grey, glabrous. Leaves borne on brachyblasts, sometimes clustered, petiolate, palmately divided into 5–7 leaflets, leaflets 1–2.5 × 0.05–0.7 cm, linear, oblong, oblanceolate to obovate, obtuse or retuse, tapered at base into petiole, paler and punctate beneath, glabrous; petioles 1.5–3.5 cm. Flowers solitary or paired,± terminal from the brachyblasts; peduncle very short, 1–2 mm; bracteoles caducous; pedicels 6–14 mm; sepals unequal, glabrous with broad scarious margins, outer 4–5 × 3–4 mm, elliptic to suborbicular, inner c. 7 × 4–5 mm, elliptic, obtuse; corolla 3–3.5 cm long, narrowly funnel-shaped, glabrous, tube greenish, limb pale pink, 2–2.5 cm diam.; stamens held at corolla mouth. Capsules ovoid, rostrate, glabrous; seeds long pilose.

#### Distribution.

A Jamaican endemic.

**JAMAICA.** St Catherine, Hellshire Hills, *C.D. Adams* 10775 (BM); Long Mt., *W. Harris* 11944 (BM, K, NY); St Andrew, *G.R. Proctor* 17412 (BM); St Thomas, *G.R. Proctor* 36516 (BM); *McFadyen* s.n. (K).

### 205. Ipomoea lachnaea

Taxon classificationPlantaeSolanalesConvolvulaceae

Spreng., Neue Entdeck. Planzenk. 3: 29. 1822. (Sprengel 1822: 29)

#### Type.

DOMINICAN REPUBLIC. *C.L.G. Bertero* s.n. (isotypes M, MO, MPU012116, MPU 011719).

#### Description.

Climbing herb, stems grey-tomentose. Leaves unequal, borne in fascicles, shortly petiolate, 2–6 × 1–2.5 cm, oblong-elliptic, acute to emarginate, base cuneate to weakly cordate, both surfaces densely appressed canescent/tomentose, abaxially silvery; petioles c. 1 cm. Flowers in subsessile axillary clusters; peduncles 0–1.5 cm; bracteoles 8–20 mm, oblanceolate, obovate to elliptic, acute, resembling diminutive leaves; pedicels 0–3 mm; sepals 10–15 mm, linear-lanceolate, acuminate, silvery pilose on both surfaces; corolla 3–3.5 cm long, subcylindrical, suburceolate, limb no wider than tube, 2–3 mm, long, toothed, purple, tomentose; stamens included.

#### Distribution.

Endemic to semi-dry forest in the Dominican Republic, apparently rare. **DOMINICAN REPUBLIC.** Loma Tibisi, *A.H. Liogier* 11779 (NY); La Romana. *A. H. Liogier* 20762 (NY); *M.M. Mejía P. & T. Zanoni* 9163 (NY); Azua, *M.D. Fuertes L.* 1891 (NY).

#### Note.

Distinguished by the tomentose, purple suburceolate corolla, the relatively large sepals and the tomentose leaves.

### 206. Ipomoea luteoviridis

Taxon classificationPlantaeSolanalesConvolvulaceae

Ekman & Leonard, Repert. Spec. Nov. Regni Veg. 24: 11. 1927. (Urban 1927: 11)

#### Type.

HAITI. Massif du Nord, Gros-Morne, Morne Chabre, *E.L. Ekman* 5025 (S07-4662, lectotype, designated here).

#### Description.

Twining perennial, stems somewhat woody, hirsute. Leaves petiolate, 1.5–5 × 0.7–3.5 cm, deltoid, ovate to broadly oblong, repand, sinuate or very shallowly 3-lobed, apex retuse and sometimes apiculate, base broadly cuneate to truncate, densely stellate-hairy on both surfaces, adaxially grey-green, abaxially grey; petioles 1–3(–7) cm, hirsute. Inflorescence of dense cymes, axillary and on leafy branchlets; peduncles 0.2–1.5 cm, tomentose; bracteoles 3–4 × 1 mm; oblong to lanceolate, deciduous; pedicels 6–8 mm, grey stellate-tomentose; sepals subequal, outer 5–6 mm, suborbicular, rounded, tomentose, inner c. 6 mm, pubescent, shiny; corolla 1.2–1.7 cm long, glabrous, greenish-yellow, campanulate, limb deeply lobed with lanceolate-elliptic lobes; anthers strongly exserted, the glandular base easily visible in the corolla mouth. Capsules 7–8 × 6 mm, subglobose, glabrous; seeds 4–5 × 2 mm, pilose on the angles with long white hairs reaching c. 8 mm.

#### Distribution.

Endemic to the island of Hispaniola where it is frequent, often growing on serpentine deposits.

**HAITI.***E.L. Ekman* H4559 (S), H6170 (S), H9279 (S); St Michel de L’Atalaye, *E.C. Leonard* 7385 (NY); Montagnes Noires, *T.A. Zanoni et al.* 23991 (NY).

**DOMINICAN REPUBLIC.***E.L. Ekman* H16227 (S), 12688 (S); Santiago Rodríguez, *A. H. Liogier* 13243 (NY); Monseñor Noel, *A. H. Liogier* 17589; Cordillera Central, *T.A. Zanoni et al.* 25400 (NY).

#### Typification.

There are two sheets of *Ekman* H5025 at S. We have selected the sheet with open corollas as the lectotype.

#### Note.

Very distinct because of the stellate hairs on vegetative parts. The inflorescence has very short hairy peduncles and short pedicels so inflorescence in axillary clusters. The corolla is yellow-green, broadly campanulate and with strongly exserted anthers.

### 207. Ipomoea nematoloba

Taxon classificationPlantaeSolanalesConvolvulaceae

Urb., Symb. Antill. 3 (2): 349. 1902. (Urban 1902–3: 349)

#### Type.

HAITI. Monte Bienac, *W. Buch* 587 (isotypes GH00054570, NY00111088).

#### Description.

Climbing perennial; stems glabrous, wiry, woody. Leaves petiolate, divided digitately into 5–7 lobes, the laterals sometimes pedate, lobes linear, 2.5–6.5 × 0.1–0.25 cm, often incurved, obtuse and mucronate; petioles 1.5–3 cm. Inflorescence of axillary and terminal leafy racemes 3–6 cm long; rhachis 1–6 cm, relatively stout; bracteoles caducous; pedicels 3–5 mm; sepals subequal, glabrous, coriaceous, 4–5 × 2 mm, elliptic to suborbicular, rounded, somewhat scarious, especially on the margins; corolla 1.5–2 cm long, greenish-yellow with pinkish lobes, glabrous, the tube 7–9 mm, the limb deeply lobed, the lobes oblong, up to 4 × 10 mm, stamens exserted. Capsules 10–11 × 5–6 mm, narrowly obovoid, style usually persistent, glabrous; seeds c. 3 mm long, long-pilose with hairs up to 8 mm long.

#### Distribution.

Endemic to Hispaniola, where it is common in dry forest.

**HAITI.***E.L. Ekman* H2164 (S), H3066 (S), H6697 (S); Massif des Matheux, *E.L. Ekman* H5156 (K, NY, S).

**DOMINICAN REPUBLIC.** Azua, *A. Liogier* 14947 (NY); Santiago. *A. Liogier* 15278 (NY); *A. Liogier* 16915 (NY).

#### Note.

This species is characterised by the deeply lobed corolla and obovoid capsules. The leaves are digitately lobed with linear lobes.

### 208. Ipomoea carolina

Taxon classificationPlantaeSolanalesConvolvulaceae

L., Sp. Pl. 1: 160. 1753. (Linnaeus 1753: 160)

Ipomoea umbellata L., Syst. Nat., ed. 10, 2: 924. 1759. (Linnaeus 1759a: 924). Type. Icon in Plumier in Burman, Pl. Amer: t. 92, f. 2 (1756), designated by Staples and Austin in Staples and Jarvis (2006: 1023).
Ipomoea caroliniana Lam., Tabl. Encycl. 1(2): 464. 1793 [11 Feb 1793], nom. superfl. Type. Based on Catesby 2: t.91 [erroneously 19] (1743).
Ipomoea heptaphylla Griseb., Pl. Wright. 2: 527. 1862. (Grisebach 1862a: 527), nom. illeg., non Ipomoea heptaphyllaSweet (1830). Type. CUBA. *C. Wright* 1371[1649] (lectotype GOET002514, designated here; isolectotypes B, GH, GOET, HAC, K, MO, NY, PH, S, YU).
Quamoclit heptaphylla (Griseb.) M. Gómez, Fl. Habana 346. 1899 [pub.1897]. (Gómez de la Maza y Jiménez 1897: 346).
Ipomoea yamuriensis Urb., Symb. Antill. 9: 247. 1924. (Urban 1924b: 247). Type. CUBA. Prov. Oriente, [Matanzas], Río Yamuri, 600–700 ft., 7 Dec. 1910, J.A. Shafer 7819 (isotypes NY, GH).

#### Type.

Icon in Catesby, Nat. Hist. Carolina 2: 91, t. 91 (1743), designated by Dandy (1958; 112).

#### Description.

Scrambling liana; stems woody, glabrous, bark pale brown. Leaves petiolate, digitately divided into 3–5 often very unequal, shortly petiolate leaflets, leaflets 2–6.5 × 0.7–2.2 cm, oblanceolate to obovate, acute, obtuse or retuse, tapering into a petiolar base, margin often undulate, both surfaces glabrous, somewhat coriaceous in texture; petioles 1.7–4.7 cm. Inflorescence of few-flowered axillary cymes; peduncles 0.3–4 cm, often stout and woody and becoming brachyblast-like; bracteoles early caducous, not seen; secondary peduncles 7–13 mm, mostly spreading at right angles to peduncle; pedicels 7–20 mm; sepals glabrous, coriaceous, margins scarious, slightly unequal, outer 6–8 mm, ovate, obtuse, inner 9–10 × 8 mm, elliptic to suborbicular, rounded; corolla 4–5 cm long, funnel-shaped, pale violet with a dark centre, glabrous, tube pale on the exterior, limb c. 3 cm diam., weakly lobed; stamens included. Capsules ovoid to ellipsoid, 10–14 × 8 mm, glabrous; seeds 5–6 mm, long-pilose, the hairs up to 15 mm, principally marginal.

#### Illustration.

**Figure 106.**
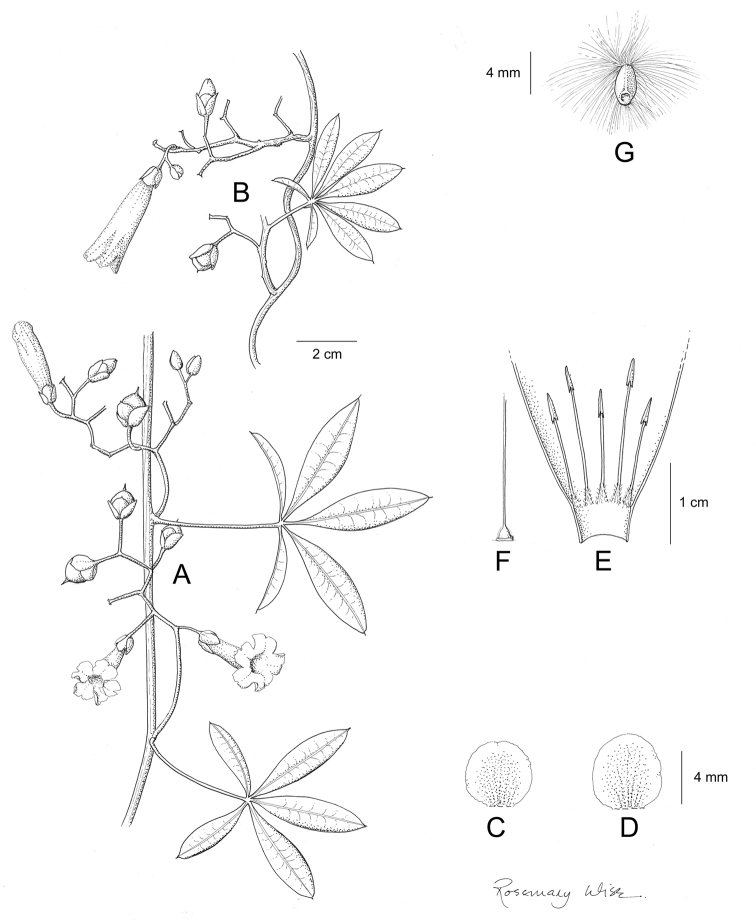
*Ipomoea carolina*. **A** habit **B** habit **C** outer sepal **D** inner sepal **E** corolla opened out to show stamens **F** ovary and style **G** seed. Drawn by Rosemary Wise **A, C–G** from *Correll* 50233; **B** from *Morton & Acuña* 2919.

Figures 11C, 94D, 106.

#### Distribution.

Growing in dry forest in the Bahamas and Cuba, probably most common in the latter.

**BAHAMAS.***C. Mathews* 79 (K). North Andros, *D.S. Correll et al.* 49373 (MO). New Providence: *N.L. Britton & L. Brace* 180 (NY); ibid., *P. Wilson* 8396 (K, MO, NY); ibid., *D.S. Correll* 50233 (BM).

**CUBA.***M. López Figuieras* 1273 (HAJB), 1631 (HAJB), 2023 (HAJB), 2287 (HAJB). **Camaguey**: *J.A, Shafer* 2866 (NY). **Cienfuegos**: *R. Combs* 509 (K, NY). **Guantánamo**: Loma Santa Teresa, El Yunque, *J.A. Shafer* 7742 (K, NY); Baracoa, *F. Michelangeli et al.* 1461 (NY); Río Yara, Sierra Maestre, *E.L. Ekman* 16412 (BM, S). **Holguín**: Sierra Nipe, *C.V. Morton & J. Acuna* 2919 (BM, US). **Isla de Juventud (Pinos)**: *N.L. Britton et al. 15530* (NY). **La Habana**: Madruga, *Bro. León* 8941 (NY). **Matanzos**: *N.L. Britton & P. Wilson* 41 (K, NY). **Pinar del Río**: *J. A. Shafer* 11115 (MO). **Santiago de Cuba**: *E.L. Ekman* 7992 (NY, S), 14848 (NY, S). **Villa Clara**: *Bro. León* 4108 (NY).

#### Note.

This species is very distinct because of its digitately divided leaves with oblanceolate or obovate leaflets combined with a funnel-shaped corolla, which is pale violet with a dark centre.

### 209. Ipomoea furcyensis

Taxon classificationPlantaeSolanalesConvolvulaceae

Urb., Symb. Antill. 3(2): 351 1902. (Urban 1902–3: 351)

Convolvulus macrorhizos L., Syst. Nat., ed. 10, 2: 923. 1759. Type. Icon in Plumier in Burman, Pl. Amer. T, 90, f. 1 (1756), designated by Staples and Jarvis (2006: 1021).
Ipomoea macrorhiza (L.) Roem. & Schult., Syst. Veg. 4: 211. 1819. (Roemer and Schultes 1819: 211), nom. illeg., non Ipomoea macrorhizaMichaux (1803).
Ipomoea plumieriana House, Bot. Gaz. 43(6): 413. 1907. (House 1907b: 413). Type based on Convolvulus macrorhizos L.

#### Type.

HAITI. Furcy Mountains, *L. Picarda* 1501 (?B† wherabouts uncertain).

#### Description.

Liana; stems woody, glabrous. Leaves petiolate, digitately divided into 5–7 leaflets, leaflets 1.5–11 × 0.5–3.5 cm, variable in size in the same leaf, oblong-elliptic or oblanceolate, acuminate to an obtuse, mucronate apex, base attenuate to a short petiole, glabrous; petioles 1.5–6.5 cm. Inflorescence of lax, much-branched axillary cymes; peduncles 2.5–8 cm; bracteoles caducous; secondary and tertiary peduncles 1–5 cm; pedicels 11–17 mm; sepals 7–10 mm, obovate-elliptic, rounded, coriaceous, reddish, margins scarious, inner slightly exceeding outer; corolla 4–5 cm long, glabrous, pinkish-purple, funnel-shaped, the tube abruptly widened just above the base, limb very broad, 3–4 cm diam. Capsules 12–14 × 7 mm; narrowly ovoid to subconical, acute, the style somewhat persistent; seeds pilose.

#### Distribution.

Endemic to and common in moist mountain forests in Hispaniola.

**HAITI.** Jacmel, *Fr. Xavier* 1896 (BM); *E.L. Ekman* H1230 (S), 2253 (S); Massif de la Selle, *E.L. Ekman* H10880 (K, NY, S); Massif de la Hotte, *T.A. Zanoni et al.* 24080 (MO, NY). **DOMINICAN REPUBLIC.** San Juan, Piedra del Aguacate, *R.A. Howard* 9428 (BM); Barahona, *M.D. Fuertes* 1397 (BM, K, NY); ibid., *E.L. Ekman* H11011 (S); Santiago, *A.H. Liogier* 17238 (NY); ibid., La Hotte, *R.A. Howard* 12248 (BM); San José de Occoa, *A.H. Liogier* 24961 (NY); La Vega, *T.A. Zanoni et al.* 27545 (MO, NY).

#### Note.

This is the Hispaniola counterpart of *Ipomoea lineolata* and *I. carolina*. It is distinguished by its relatively long, usually oblanceolate leaflets and the relatively long peduncles and pedicels.

### 210. Ipomoea lineolata

Taxon classificationPlantaeSolanalesConvolvulaceae

Urb., Symb. Antill. 3 (3): 355. 1903. (Urban 1902–1903: 355)

Ipomoea grisebachii Urb. (1903: 353), nom. illeg., non Ipomoea grisebachiiPrain (1894). Type. JAMAICA. Guy’s Hill, Moneague, *Alexander*s.n. (lectotype K000612811, designated by Wood and Scotland 2017c: 14).
Ipomoea rubella House, Bot. Gaz. 43: 414. 1907. (House 1907b: 414). Type. Based on I. grisebachii Urb.
Ipomoea carmesina Proctor, J. Arnold Arbor. 63(3): 292. 1982. (Proctor 1982: 292). Type. JAMAICA. [Trelawny], near Crown Lands road extension 4.5–5 miles NW of Troy, 7 Sept. 1974, *G.R. Proctor* 34169 (holotype IJ!).

#### Type.

JAMAICA. *Wilson* “1126 aut 1155” (probably destroyed at B in 1943, no duplicate found at NY, neotype *G.R. Proctor* 10429 (BM001122860), from Dolphin Head, Jamaica, designated by Wood and Scotland 2017c: 14).

#### Description.

Liana climbing over scrub to 5 m; stems woody, glabrous, reddish-brown. Leaves petiolate, palmately divided into 3–5(–7) petiolate leaflets, the terminal leaflet larger, leaflets 2.3–12 × 1.5–5 cm, lanceolate to oblanceolate, obovate or elliptic, acuminate-caudate, mucronate, narrowed at base into a petiole 5–10 mm long, glabrous, abaxially paler with numerous lateral veins; petioles 1.6–6 cm. Inflorescence of pedunculate, axillary cymes with 3 to many flowers, primary peduncles 2–11 cm, stout, sometimes forming a rachis of a raceme; bracteoles caducous, not seen; pedicels 0.7–3.5 cm, thickened upwards; sepals subequal coriaceous, glabrous, suborbicular-elliptic, acute, obtuse or rounded, outer 5–10 mm, inner 10–12 mm; corolla 5–6 cm long, funnel-shaped, glabrous, pink; limb 3–4.5 cm diam. Capsules ovoid, shortly rostrate, glabrous; seeds long-pilose with hairs to 12 mm.

#### Illustration.

Figure 107B–E.

**Figure 107.**
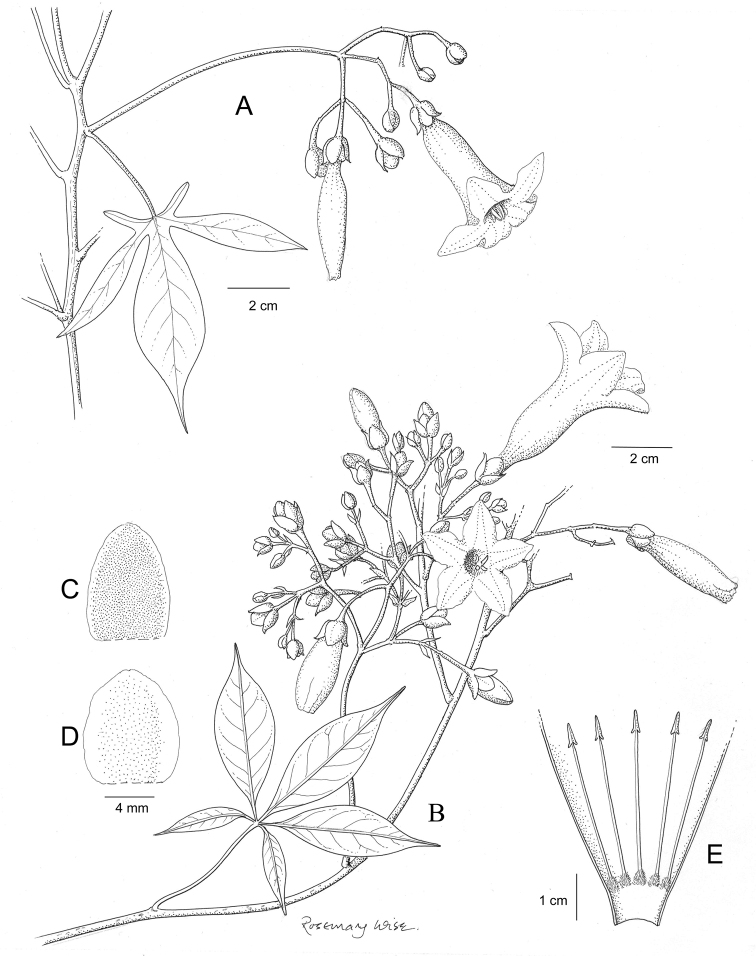
*Ipomoea horsfalliae***A** habit showing lobed leaves found in some cultivated forms. **B–E***I. lineolata***B** habit C outer sepal **D** inner sepal **E** corolla opened out to show stamens. Drawn by Rosemary Wise **A** from *Jack* 4278; **B–E** from *Fosberg* 42712.

#### Distribution.

Endemic to Jamaica where it gows in mountain woodland.

**JAMAICA.** Clarendon, *G.L. Webster & G.R. Proctor* 5413 (BM); Hanover, *G.R. Proctor* 10429 (BM); Manchester, *Purdie* s.n. (K); Portland, *H.A. Osmaston* 5101 (BM); St Andrew, *T.G. Yuncker* 17184 (BM); St Ann, *G.L. Webster & G.R. Proctor* 5639 (A, BM, MICH); St Catherine, *G.R. Proctor* 34186 (BM); St James, *W. Stearn* 31 (BM); St Thomas, *C.D. Adams* 7262 (BM); Trelawny, *G.R. Proctor* 21374 (BM).

#### Notes.

This is the Jamaica counterpart of *Ipomoea furcyensis* and *I. carolina*. It is distinguished by its (usually) broadly elliptic, ovate to obovate leaflets and slightly larger corolla.

*Ipomoea lineolata* is quite variable, particularly in the number of leaflets (3–7) and in their shape (lanceolate to oblanceolate, obovate or elliptic), the leaflets narrowed to a petiolar base. The corolla varies somewhat in length but is usually 5–6 cm long and the anthers are held at the mouth of the corolla and are not clearly exserted. The inflorescence is much branched in most plants (and also in the type of *I. carmesina*) but specimens with 3–5-flowered cymes are not uncommon. It should be noted that all these populations have leaves divided into distinct leaflets with a petiolar base including the oldest specimen of wild provenance we have seen (*Purdie* s.n.) collected in November 1843.

### 211. Ipomoea horsfalliae

Taxon classificationPlantaeSolanalesConvolvulaceae

Hook., Bot. Mag. 61, t. 3315. 1834. (Hooker 1834b: t. 3315)

#### Type.

Plate 3315 In Bot. Mag., epitype. K000612699, designated by Wood and Scotland 2017c: 11.

#### Description.

Liana climbing to 10 m over scrub; stems woody, glabrous, often muricate with blunt warts. Leaves palmately divided into 5–7 leaflets, leaflets 4–14 × 0.8–3 cm, sessile or basally fused, oblong-elliptic, obovate or oblanceolate, acuminate or obtuse, narrowly cuneate at base, glabrous, abaxially paler; petioles 3–7 cm. Inflorescence of axillary pedunculate cymes, which are often aggregated to form a many-flowered terminal panicle; peduncles and panicle rhachis 2.5–15 cm long; secondary peduncles, if present, 1.5–5 cm; bracteoles 3–4 mm, lanceolate with scarious margins, caducous; pedicels 10–15 mm; sepals coriaceous, slightly unequal, outer 7–8 × 5 mm, ovate, convex, obtuse with narrow scarious margin, inner 9–10 × 7 mm, elliptic, rounded with broad scarious margin; corolla 4.5–6 cm long, glabrous, usually dark red with paler tube, narrowly funnel-shaped, limb distinctly lobed, 3–4 cm diam., stamens held at mouth or slightly exserted. Capsules rostrate, glabrous; seeds with long brown marginal hairs.

#### Illustration.

Figures 84B, 107A; Acevedo-Rodríguez (2005: 170); Wood and Scotland (2017c: 12–13).

#### Distribution.

Cultivated throughout the tropics. The following records are all of cultivated plants.

**BRAZIL. Minas Gerais**: *Y. Mexia* 5744a (NY). **Rio de Janeiro**: *C.G. Pinto* 222 (RB); *J.R. Mattos* 380 (RB). **São Paulo**: *G.D. Passerini* s.n. [20/4/2003] (RB).

**SURINAM.** Fide Austin and Huáman (1996).

**GUYANA.** Fide Austin and Huáman (1996).

**VENEZUELA.** Fide Austin and Huáman (1996).

**BERMUDA.***S. Brown et al.* 1952 (NY)

CUBA. Cienfuegos, *J.G. Jack* 4278 (A); La Habana, *Bro. León* 8499 (NY).

**PUERTO RICO.***Sintenis* 4655 (BM, S); *N.L & E.O. Britton* 7419 (NY), 9178 (NY)

**LESSER ANTILLES. U.S. Virgin Islands**: St Croix: *J.B. Thompson* 1055 (NY). **Guadeloupe**: *A. Duss* 3086 (NY). **Martinique**: *A. Duss* 1882 (NY). **Barbados**: *L.M. Andrews* 646 (NY).

**TRINIDAD.** Fide Baksh-Comeau et al. (2016).

**HAWAII.** Fide http://www.starrenvironmental.com

#### Note.

Our understanding of *Ipomoea horsfalliae* has been set out elsewhere (Wood and Scotland 2017c) and is essentially that this is a cultivated plant distinct from all known wild populations but probably derived from *Ipomoea lineolata*, a Jamaican endemic. As understood here, *I. horsfalliae* is a variable ornamental species whose leaves can be divided into (3–) 5 (–7) sessile leaflets or are, less commonly, 3–5-lobed. The inflorescence is of compound cymes, the anthers shortly exserted or held at the corolla mouth. Most plants are sterile and capsules are rarely found. Reported by Powell (1979) and Acevedo-Rodríquez (2005) to hybridise with *Ipomoea repanda* producing 5-lobed leaves with flowers resembling those of *I. repanda*. These supposed hybrids and similar forms with 5-lobed leaves are here treated as *I. horsfalliae* but require investigation to confirm their status.

### 212. Ipomoea ternata

Taxon classificationPlantaeSolanalesConvolvulaceae

Jacq., Pl. Hort. Schoenbr. 1: 16, t. 37. 1797. (Jacquin 1797a: 16)

Ipomoea thomsoniana Mast, Gardener’s Chronicle new series 20: 818. 1883. (Masters 1883: 818). Type. “East Indies” [error for JAMAICA], cult. *Masters*s.n. (holotype K000612816).
Ipomoea saxicola Proctor, J. Arnold Arbor. 63(3): 292. 1982. (Proctor 1982: 292). Type. JAMAICA. Clarendon Parish, Glenwood Springs, along road between Balcarres and Sunbury, 27 Sept. 1974, *G.R. Proctor* 34185 (holotype GH00054580, isotypes BM, NY).
Ipomoea ternata var. saxicola (Proctor) J.R.I. Wood & Scotland, Kew Bull. 14. 2017. (Wood and Scotland 2017c: 14).

#### Type.

Cultivated from material collected in Jamaica, *Jacquin* s.n. (holotype W0042716).

#### Description.

Robust liana to 16 m from a large root tuber; stem woody, glabrous, sometimes warted. Leaves petiolate, digitately divided into three leaflets, leaflets 7.5–14 × 3.5–6.5 cm, obovate, abruptly narrowed to an acute, obtuse or retuse, mucronate apex, base cuneate with a distinct petiole 2–5 mm long, very coriaceous, fleshy and glossy, glabrous; petioles 2.3–7.2 cm. Inflorescence of several pedunculate flowers from the leaf axils, arising on stubby brachyblasts, sometimes cauliflorous on old plants; peduncles 5–12 mm; bracteoles caducous, not seen; pedicels 30–38 mm; sepals glabrous, very unequal, elliptic, margins slightly scarious, outer 7–10 × 7–9 mm, rounded, inner 17–20 × 12–14 mm, obtuse; corolla c. 5 cm long, funnel-shaped, white, the tube tinged red. Capsules ellipsoid, 15–18 × 12 mm glabrous; seeds pilose with long silky marginal hairs, 10–12 mm in length.

#### Distribution.

Endemic to Jamaica, growing on wooded limestone hills.

**JAMAICA.** St Ann, *W.T. Stearn* 593 (BM), ibid., Union Hill, *G.R. Proctor* 26486 (BM); ibid., Clydesdale, *W.R. Philipson* 1134 (BM); Manchester, *W. Stearn* 377 (BM, K), ibid., Old England, *W. Harris* 6598 (BM); St Andrew, *I. Maxwell* s.n. [1/1927] (BM), ibid., Cinchona, *W. Harris* 7410 (BM); St Catherine, *Perkins* 9005 (BM, K); Portland, *G.R. Proctor* 8574 (BM); Trelawny, *Grady Webster et al.* 5374 (BM, S), 5634 (BM); ibid., West & Arnold 280 (BM); Clarendon, Bird Cave Rock, *B.D. Morley et al.* 939 (BM) – var. saxicola; Glenwood Springs, *G.R. Procter* 33630 (BM) – var. saxicola.

**Notes**. Although most specimens of *Ipomoea ternata* are glabrous, a very distinct roughly pilose variety has been found near Glenwood Springs in Clarendon Parish. Originally described as a distinct species this can be recognised as var. saxicola.

### 213. Ipomoea desrousseauxii

Taxon classificationPlantaeSolanalesConvolvulaceae

Steud., Nomencl. Bot. 1: 816. 1840. (Steudel 1840: 816)

Convolvulus eriospermus Desr., Encycl. 3: 567. 1789 [pub. 1792]. (Desrousseaux 1792: 567). Type. Sine data, P-LAM (?P00666142).
Exogonium eriospermum (Desr.) Choisy, Mém. Soc. Phys. Genève 8(1): 52[130]. 1838. (Choisy 1838: 52 [130]).
Ipomoea eriosperma (Desr.) Urb., Symb. Antill. 3(2): 351. 1902. (Urban 1902–3: 351), nom. illeg., non Ipomoea eriosperma P. Beauv. (1819).
Ipomoea leuconeura Urb., Symb. Antill. 3: 350. 1902. (Urban 1902–3: 350). Type. HAITI. *L Picarda* 16 (NY), *C. Ehrenberg* 134 (GH), *W. Buch* 5 (GH), syntypes.
Exogonium leuconeurum (Urb.) House, Bull. Torrey Bot. Club 35: 106. 1908. (House 1908a: 106).

#### Type.

Based *Convolvulus eriospermus* Desr.

#### Description.

Climbing perennial; stems glabrous, up to 4 m long. Leaves petiolate, usually small, digitately divided to or almost to the base into (3–)7 lobes, the lateral lobes often pedate, base truncate and broadly cuneate onto the petiole, lobes 0.7–6 × 0.2–1.5 cm, oblong-oblanceolate, obtuse or rounded, glabrous, abaxially gland-dotted; petioles 0.7–5 cm. Inflorescence of (1–)2–5(–10) flowers borne in short axillary cymes; peduncles 5–20 mm, glabrous; bracteoles 1 mm, scale-like, caducous; secondary peduncles c. 5 mm; pedicels 6–20 mm, straight, glabrous; sepals subequal, 4–6 mm, obovate-suborbicular, rounded, glabrous with scarious margins, the inner perhaps 1 mm longer than the outer; corolla 2.5–4 cm long, narrowly funnel-shaped to subcylindrical, c. 7 mm wide, the tube only slightly widening upwards, red, glabrous, the limb 2.5 cm diam. Capsules 9–10 × 5–6 mm, oblong-ovoid, glabrous, much exceeding the calyx; seeds 4–5 × 2 mm, pilose with long white hairs.

#### Distribution.

Widespread in dry forests in Hispaniola, often on limestone, where it is endemic.

**HAITI.** Montagnes du Trau d’Eau, *E.L. Ekman* H2126 (K, S), 3043 (S), 9761 (S); Port de Paix, *E.L. Ekman* H3546 (K, MO, NY, S); ibid., *E.C. & G.M. Leonard* 14602 (K, US).

**DOMINICAN REPUBLIC.***E.L. Ekman* H14595 (S); *Emanuelsson* 2731 (S); El Plátano, Bayaguana, *A.H. Liogier* 18717 (K, NY); Azua, *A.H. Liogier* 24873 (NY); Cabo Rojo-Las Mercedes, *A.H. Liogier* 13815 (P); Beata Island, *D. Fairchild* 2611 (P).

#### Note.

Some specimens from Haiti, for example *Ekman* H9548 (S) and *Ekman* H9624(S), have stouter corollas, the tube c. 10 mm wide.

### 214. Ipomoea digitata

Taxon classificationPlantaeSolanalesConvolvulaceae

L., Syst. Nat., ed. 10, 2: 924. 1759. (Linnaeus 1759a: 924)

Quamoclit digitata (L.) G. Don, Gen. Hist. 4: 260. 1838. (Don 1838: 260).
Ipomoea paniculata var. digitata (L.) Kuntze, Revis. Gen. Pl. 2: 445. 1891. (Kuntze 1891: 445).
Ipomoea rubrocincta Urb. Symb. Antill 3(2): 347. 1902. (Urban 1902–3: 347). Type. HAITI. Inter La Coupe et Pintade, *W. Buch* 482 (holotype B?†, fragment and photo of holotype US).
Ipomoea rubrocincta var. brachyloba Urb., Symb. Antill 7: 341: 1912. (Urban 1912: 341). Type. HAITI. Poste Coudon, *W. Buch* 1015 (holotype B, isotype GH00054578).

#### Type.

Icon in Plumier in Burman, Pl. Amer. 81, t. 92, f. 1 (1756), uncertainly designated by Stearn 1961: 17), redesignated as lectotype here).

#### Description.

Stout climbing perennial; stems woody below, usually glabrous. Leaves petiolate, rather small, palmately 5–7-lobed generally to about two thirds, base truncate, lobes 1–2.5 × 0.2–0.7 cm, oblong to narrowly lanceolate, obtuse to retuse, sometimes muricate, the laterals smaller and often pedate, margin entire, undulate or crenate, usually glabrous. Inflorescence of pedunculate 2–5-flowered axillary cymes; primary peduncles 2–6.5 cm; bracteoles 1 mm, deltoid, acute, deciduous; secondary and tertiary peduncles 1.5–2.5 cm; pedicels 5–11 mm; sepals subequal, 3–5 mm, suborbicular, obtuse or rounded, coriaceous, glabrous, margins reddish, the inner scarious; corolla 3–4 cm long, funnel-shaped from a very narrow base, pink, glabrous, limb c. 2 cm diam., shallowly lobed. Capsules ellipsoid, 8–9 × 5 mm, glabrous; seeds 5–6 × 2 mm, long-pilose with white hairs c. 7 mm long.

#### Illustration.

Liogier (1994: 81).

#### Distribution.

Endemic to coastal forest on limestone hills in Hispaniola.

**HAITI.** Massif de la Hotte, *E.L. Ekman* H2433 (S); H6048 (S); Massif des Cahos, Gonaïves, *E.L. Ekman* H9065 (NY, S).

**DOMINICAN REPUBLIC.** Santo Domingo, *E.L. Ekman* H13512 (K, S); Río Arriba del Norte, *R.A. & E.S. Howard* 8923 (BM); Monte Cristi, *A.H. Liogier* 16417 (NY).

### 215. Ipomoea clausa

Taxon classificationPlantaeSolanalesConvolvulaceae

Rudolph. ex Ledeb. & Adlerstam, Diss. Bot. Pl. Doming.14. 1805. (Ledebour and Adlerstam 1805: 14)

Exogonium pedatum Choisy, Mém. Soc. Phys. Genève 8: 130 [52]. 1838. (Choisy 1838: 130 [52]). Type. DOMINICAN REPUBLIC. Santo Domingo, P.A. Poiteaus.n. (holotype G00418215, isotype P00666136).
Ipomoea viridiflora Urb., Symb. Antill. 3: 348. 1902. (Urban 1902–3: 348). Type. HAITI. *C. Ehrenberg* 345 (holotype ?B†, isotype US00111489).
Exogonium viridiflorum (Urb.) House, Bull. Torrey Bot. Club 35: 106. 1908. (House 1908a: 106).
Ipomoea buchii Urb., Symb. Antill 3(3): 356. 1903. (Urban 1902–03: 356). Type. HAITI. Near Petit Coupe, *W. Buch* 817 (holotype B†, isotype US00111368).
Ipomoea samanensis Urb., Repert. Spec. Nov. Regni Veg. 20: 343. 1924. (Urban 1924a: 343). Type. DOMINICAN REPUBLIC. On south side of Samana Bay, *W.L. Abbott* 1282 (isotype GH00054579).
Ipomoea pitoniana Urb., Repert. Spec. Nov. Regni Veg. 24: 10. 1927. (Urban 1927: 10). Type. HAITI. Massif du Nord, Port de Paix, Haut Piton, *E.L. Ekman* H4603 (S07-4774, lectotype designated here; isolectotypes K, S).
Ipomoea selleana Urb., Repert. Spec. Nov. Regni Veg. 24: 11 1927. (Urban 1927: 11). Type. HAITI. Massif de la Selle. Nouvelle Touraine, Chapelle Faure, 1000 m, *E.L. Ekman* H1532b (holotype S07-4779).
Ipomoea hospitalis Urb., Ark Bot. 23A(5): 102. 1930. (Urban 1930: 102). Type. HAITI. Port-au-Prince, Massif de la Selle, 2 Oct. 1927, *E.L. Ekman* H9111 (holotype S07-4467).
Ipomoea hotteana Urb. & Ekman, Ark. Bot. 23A (5): 103. 1930. (Urban 1930: 103). Type. HAITI. Massif de la Hotte, group Morne-Rochelois, Miragoane, limestone cliffs near Etang-Miragoane, *E.L. Ekman* H7227 (lectotype S07-4472, designated here; isolectotype S).

#### Type.

DOMINICAN REPUBLIC. *P. A. Poiteau* (possible fragment US, possible isotypes G, P).

#### Description.

Slender woody twiner; stems pale brown, usually glabrous. Leaves petiolate, 2–7 × 1.5–6 cm, polymorphic, ovate-deltoid, entire or 3-lobed or palmately divided into 5 pedate, ovate to oblanceolate lobes, apex acute, obtuse or emarginate and mucronate, base truncate or weakly cordate and broadly cuneate onto petiole, abaxially paler, both surfaces glabrous; petioles 1.3–4.2 cm. Inflorescence of pedunculate axillary cymes; peduncles strikingly variable in length from 1–10 cm; bracteoles 1–2 mm, linear-lanceolate, scarious, caducous; secondary peduncles 5–13 mm; pedicels 3–15 mm; sepals subequal, glabrous, coriaceous, margins scarious, outer 8–10 mm, elliptic, rounded or obtuse, inner similar but 9–11 mm; corolla 3–5 cm long, abruptly widened above the short basal tube but not at limb, greenish-white, glabrous, limb weakly lobed, c. 2 cm diam.; stamens included. Capsules globose, glabrous; seeds with long woolly hairs.

#### Distribution.

Endemic to Hispaniola growing in scrub at low altitudes.

**HAITI.** Isla Tortue *E.L. Ekman* H4085 (S); ibid., *E.L. Ekman* H9744 (K, S); ibid., *E.C. Leonard* 13901 (K, MO, NY); Massif de Cahos, Gonaïves, *E.L. Ekman* H9064 (S); Massif des Matheux, *E.L. Ekman* 9162 (K, S).

**DOMINICAN REPUBLIC.** Sine loc., *R. Schomburgk* 1857 (K); Sierra Martín García, Barahona, *M. Mejía et al.* 1282 (NY); Sierra Prieta, *A.H. Liogier* 24108 (NY); La Romana, *A.H. Liogier* 24231 (NY); Peravia, *T.A. Zononi et al.* 18081 (NY).

**Notes**. The location of the original material used for the description of *Ipomoea clausa* is uncertain. There is a fragment at US, which may belong but it was probably based on a duplicate of the same Poiteau collection which is at G and P.

The type of each name represents a form with distinct leaves: *Ipomoea clausa* has 3-lobed leaves; *I. hospitalis* has small deltoid leaves c. 2 cm long; *I. hotteana* is a form with digitate leaves, the terminal lobe oblanceolate c. 4.5 cm long; *I. pitoniana* has deltoid leaves which are commonly shallowly lobed and c. 4 cm long; *I. selleana* is similar in leaf form but the leaves are less lobed and the margins strongly undulate. All forms have obscure hairs on the stem and leaf veins but these are more obvious abaxially in *I. selleana*.

This variable species is in many ways a Hispaniola counterpart of the Cuban *Ipomoea alterniflora*.

• Species 216–217 are sisters to each other and sisters to the rest of Clades A1–2. They are very different in their calyx structure.

### 216. Ipomoea setosa

Taxon classificationPlantaeSolanalesConvolvulaceae

Ker-Gawl., Bot. Reg. 4: t. 335. 1818. (Ker Gawler 1818e: t. 335)

#### Type.

Icon, Ker Gawler, Bot. Reg. 4: t. 335, lectotype, designated by J.A. McDonald (1994: 110).

#### Description.

Scrambling perennial herb, stems with soft fleshy trichomes and bluish-green bloom but otherwise glabrous. Leaves petiolate, 10–32 × 10–32 cm, mostly 3–7(–9)-lobed to about halfway but sometimes ovate-orbicular, apex shortly acuminate, obtuse and mucronate, base cordate with rounded auricles, margin irregularly dentate with scattered teeth, both surfaces glabrous; petioles 5–14 cm, armed with soft fleshy trichomes. Inflorescence of long-pedunculate axillary cymes; peduncles 5–15 cm, usually armed with soft fleshy trichomes; bracteoles 5–10 × 2 mm, oblong, mucronate, caducous; secondary peduncles 1.5–3 cm; pedicels 1–4 cm, markedly thickened upwards, glabrous or armed with soft fleshy spines, often purplish-brown; sepals subequal, 8–10 mm at anthesis (accrescent to 16 mm in fruit), ovate, acute, convex, glabrous or with soft fleshy trichomes, purplish-brown, the margins scarious; corolla 4–10 cm long, funnel-shaped, pink, glabrous, limb c. 2.5 cm diam. Capsules subglobose, 15 mm long, glabrous; seeds 7 × 5 mm, woolly, nearly black.

#### Distribution.

Widely distributed but scattered and never common throughout tropical America north to Mexico but apparently absent from Colombia and the Guianas and rare in Brazil.

#### Variation.

*Ipomoea setosa* is an isolated species, and as here delimited very variable. All specimens of *Ipomoea setosa* we have seen from South America except *Eggers* 15768 from Ecuador differ from the type in having sepals that lack fleshy trichomes. They always have 3-lobed leaves and the corolla is relatively small, being 5–6.5 cm long. Specimens from Mexico have 5–9-lobed leaves, a large corolla up to 10 cm in length and sepals densely covered in soft spines. *Eggers* 15768 from Ecuador and most plants from Central America are intermediate between these extremes and accord with the type, having 3-lobed leaves and sepals armed with fleshy trichomes. Plants mostly from Belize generally treated as *I. sepacuitensis* seem to be part of the same species differing only in the large corolla (similar to Mexican examples) and the absence of trichomes except on the stem. These four taxa are here treated as geographical subspecies which can be separated by the following key:

**Table:** 

1	Leaves 3-lobed, rarely entire; sepals devoid of fleshy trichomes or almost so	**2**
–	Leaves 3–7-lobed; Sepals armed with fleshy trichomes	**3**
2	Corolla short, 5–6.5 cm; pedicel strongly swollen below calyx; peduncles and pedicels with fleshy trichomes	**subsp. pavonii**
–	Corolla 6–8 cm; pedicel only slightly widened below calyx; peduncles and pedicels without fleshy trichomes	**subsp. sepacuitensis**
3	Leaves 3-lobed	**subsp. setosa**
–	Leaves 5–7-lobed	**subsp. melanotricha**

### 216a. Ipomoea setosa subsp. setosa

Taxon classificationPlantaeSolanalesConvolvulaceae

Convolvulus setosus (Ker-Gawl) Spreng., Syst. Veg. 1: 594. 1825 [pub. 1824]. (Sprengel 1824: 594).
Modesta setosa (Ker-Gawl.) Raf., Fl. Tellurica 4: 76. 1836 [pub. 1838]. (Rafinesque 1838a: 76).
Batatas setosa (Ker-Gawl) Lindl., Sketch Veg. Swan R. append. 1: 15. 1839. (Lindley 1839a: 15).
Calonyction setosum (Ker-Gawl.) Hallier f., Bull. Herb. Boiss. 5: 1048. 1897. (Hallier 1897b: 1048).
Ipomoea macrantha Peter, Die Natürlichen Pflanzenfamilien 4 (3a): 31. 1897 [pub. 1891]. (Peter 1891: 31), nom. illeg., non Ipomoea macrantha Roem. & Schult. (1819). Type. GUATEMALA. Retalulëu, *Bernoulli & Cario 1888* (lectotype GOET005711, designated by Staples et al. 2012: 676).
Calonyction campanulatum Hallier f., Bull. Herb. Boiss. 5: 1050. 1897. (Hallier 1897b: 1050). Type. Based on Ipomoea macrantha Peter
Ipomoea campaniflora Hallier f., Meded. Rijks-Herb. 46: 20. 1922. (Hallier 1922: 20). Type. Based on Calonyction campanulatum Hallier f.
Ipomoea setosa var. campanulata (Hallier f.) House, Ann. New York. Acad. Sci. 18: 219. 1908. (House 1908b: 219).

#### Diagnosis.

Leaves 3-lobed. Sepals covered in fleshy trichomes. Corolla 6–9 cm long.

#### Distribution.

Essentially restricted to Central America where it occurs sporadically in bushy places and on forest margins.

**ECUADOR. Guayas**: *H.F.A. von Eggers* 15768 (K).

**PANAMA.** Los Santos, Tonosi, *E.L. Tyson et al.* 2950 (MO).

**COSTA RICA.** Puntarenas, Buenos Aires, *M. Grayum* 9565 (F, MO); Puntarenas, Res. Carara, *R. Zuñiga* 558 (K, MO); Alajuela, *G. Carballo* 566 (K, MO).

**NICARAGUA.** Rivas, N. de San Juan del Sur, *W.D. Stevens* 30429 (MO); ibid., along road to Cárdenas, *W.D. Stevens* 34370 (MO).

**HONDURAS.** Res. Tawahka Asangni, *P. House* s.n. (BM); Olancho, Río Juticalpa, *A. Molina* 13252 (F).

**EL SALVADOR.** Ahuachapán, A.P. Santa Rita, *J.M. Rosales* 2078 (MO).

**BELIZE.***N.C. Goldstein et al.* 27 (MO); Belize Foundation for Research and Environmental Education, *S.W. Brewer & G. Stott* 6647 (BM, MO).

**GUATEMALA.***J.A. Pozuelos* 8087 (MO); *E. de Pöll* 7719 (MO); Petén, San Luis, *R. Tun Ortíz* 2174 (BM, F).

### 216b. Ipomoea setosa subsp. pavonii

Taxon classificationPlantaeSolanalesConvolvulaceae

(Hallier f.) J.R.I. Wood & Scotland, comb. & stat. nov.

urn:lsid:ipni.org:names:77208069-1

Calonyction pavonii Hallier f., Bull. Herb. Boiss. 5: 1048. 1897. (Hallier 1897b: 1048). Type. ECUADOR. Guayaquil, *R. Spruce* 6498 ex Herb. De Candolle (lectotype G00418182, designated here).
Ipomoea setosa var. pavonii (Hallier f.) House, Ann. New York. Acad. Sci. 18: 220. 1908. (House 1908b: 220).
Ipomoea chaetophora Hallier f., Meded. Rijks-Herb. 46: 20. 1922. (Hallier 1922: 20). Type. Based on Calonyction pavonii Hallier f.
Ipomoea pickelii Hoehne, Boletin de Agricutura (São Paulo), 35(1): 477. 1934. (Hoehne 1934: 477). Type. BRAZIL. Pernambuco, *D. Pickel* 386 (whereabouts uncertain, SP?).
Ipomoea horrida Huber ex Ducke, Anais. Acad. Brasil. Cienc. 31: 304. 1959. (Ducke 1959: 304). Type. BRAZIL. Ceará, *A. Ducke* 1151 (holotype MG, isotype F).

#### Diagnosis.

Leaves 3-lobed. Sepals glabrous, lacking fleshy trichomes. Corolla relatively small, 5–6.5 cm long.

#### Illustration.

Figure 15A, B.

#### Distribution.

Essentially restricted to South America, but occurring occasionally elsewhere (Jamaica, United States) and in the Old World. It is sporadic and uncommon everywhere. It usually grows in disturbed bushy areas and appears to be most common in the Andean foothills on the border between Argentina and Bolivia.

**ARGENTINA. Salta**: *T. Meyer* 8493 (S); *Legname & Cuezzo* 8007 (CTES, LIL); San Martin, *Legname et al.* 10148 (K, LIL). **Jujuy**: *O. Ahumada* 4245 (CTES); *O. Ahumada & Castellon* 7259 (CTES).

**BRAZIL; Bahia**: Est. Embasa Cachoeira, *Pedro do Cavalho et al.* 341 (NY); Feira de Santana, *F. França & E. Melo* 1886 (K, UEFS). **Ceará**: Maracanaúm *A. Ducke* 2544 (K).

**GUYANA.** Cultivated, sine data (K).

**BOLIVIA. Chuquisaca**: Tomina, Río Azero, *J.R.I. Wood* 8283 (K, LPB). **Santa Cruz**: Cordillera, Lagunillas, *A. Krapovickas & A. Schinini* 31364 (CTES, LIL); Florida, Mairana, *M. Nee* 47760 (LPB, NY, USZ); Ñuflo de Chávez, Lomerío, *F. Mamani* 774A (USZ); Vallegrande, camino a Masicuri, *G.A. Parada et al.* 3149 (MO, USZ). **Tarija**: Gran Chaco, Villamontes, *Pflanz* 4145 (US).

**PERU. Tumbes**: Puerto Pizarro-Estero El Bendito, *R. Ferreyra* 16227 (MO, USM); *A. Gentry & C. Díaz* 58219 (USM). **Piura**: Chulucanas Panecillo, *E. Laure* 5343 (P).

**ECUADOR. Guayas**: *E. Asplund* 16012 (K, S). *R. Spruce 6498* (K, P).

**VENEZUELA. Guárico**: *L. Aristeguieta et al.* 6449 (K, VEN).

**NICARAGUA.** Matagalpa, *P.P. Moreno* 25076 (BM).

**UNITED STATES. Mississippi**: Pearl River, *F.H. Sargent* 10494 (MISS).

**JAMAICA.***Marsh* 1133 (K) – leaves only.

#### Typification.

In designating a lectotype for *Calonyction pavonii* we have selected the Spruce collection from De Candolle’s herbarium in preference to the specimen from Boissier’s herbarium, even though this last specimen is the only one actually annotated *Calonyction pavonii* by Hallier. This is because the Boissier collection appears to contain an extraneous element (spiny sepals) pasted to the attachment at the bottom left of the sheet, which is not in accord with the protologue (“sepala glaberrima”). The De Candolle specimen is thus the only extant syntype fully in accord with the protologue, the Marsh collection from Jamaica having been destroyed in Berlin in 1943.

#### Note.

The plants from northern Peru conform to subsp. pavonii in their small corolla and glabrous sepals but are remarkable for having unlobed, suborbicular, coarsely dentate leaves.

### 216c. Ipomoea setosa subsp. melanotricha

Taxon classificationPlantaeSolanalesConvolvulaceae

(Brandegee) J.R.I. Wood & Scotland, comb. & stat. nov.

urn:lsid:ipni.org:names:77208070-1

Ipomoea melanotricha Brandegee, Univ. Cal. Publ. Bot. 4: 381. 1913. (Brandegee 1913: 381).

#### Type.

MEXICO. [Veracruz], Zacuapan, *C.A. Purpus* 5747 (holotype UC163009, isotypes BM, F, GH, MO, NY, US).

**Diagnosis**. Leaves 5–7(–9)-lobed. Sepals densely covered in fleshy spines. Corolla large, 6.5–10 cm long.

#### Distribution.

Restricted to Mexico, where it occurs sporadically at low altitudes below 700 m in forest and on forest margins.

**MEXICO. Chiapas**: *D.E. Breedlove* 28568 (MO); Arriaga, *J.C. Soto et al.* 13202 (BM); Tonala, *C.A. Purpus 6905* (BM). **Durango**: Montes de Oca, *G.B. Hinton* 9896 (K). **Guerrero**: La Unión, *J.C. Soto et al.* 6018 (IEB, MEXU). **Jalisco**: Santa Cruz de Vallarta, *Y. Mejia* 1246 (BM). **Michoacán**: *G.B. Hinton* 12613 (K); Timalcota, *E. Langlassé 680* (K). **Oaxaca**: Pochutla, *A. Sánchez Martínez et al.* 1079 (IEB, MEXU); Tehuantepec, *M. Elorsa* 1243 (MEXU). **Sinaloa**: *J.M. & E. Aguilar* 1264 (MEXU). **Tamaulipas**: *J.A. McDonald* 604 (IEB). **Veracruz**: Salto de Eyipantla, San Andrés Tuxtla, *M. Nee* 23606 (BM); Zacuapan, *C.A. Purpus* 5747(BM); *F. Ventura* 2580 (MICH); *P. Zamora & J. López* 3521 (IEB).

### 216d. Ipomoea setosa subsp. sepacuitensis

Taxon classificationPlantaeSolanalesConvolvulaceae

(Donn. Sm.) J.R.I. Wood & Scotland, comb. & stat. nov.

urn:lsid:ipni.org:names:77208071-1

Ipomoea sepacuitensis Donn. Sm., Bot. Gaz. 56: 59. 1913. (Donnell Smith 1913: 59). **Type.** GUATEMALA. Alta Verapaz, *O.F. Cook & R.F. Griggs* 590 (holotype US408299, isotype US).

#### Diagnosis.

Stem and petioles pilose with fleshy trichomes. Leaves 3-lobed. Peduncles, pedicels and sepals devoid of fleshy trichomes. Corolla 6–7 cm long.

#### Distribution.

Disturbed lowland forest in the extreme south of Mexico and neighbouring Guatemala and Belize.

**BELIZE.** Cayo District, Arenal road, *M.J. Balick* et al. 3322 (FTG, NY); Gales Point, *S.W. Brewer & G. Stott* 6649 (BM, MO); Cayo District, *W.A. Schipp* 878 (BM, K, MO, S); ibid., Ceibo Camp, *M. Peña-Chocarro et al.* 1020 (BM, MEXU, MO); ibid., Chiquibul, *A.K. Munro et al.* 1114 (BM); ibid., *C. Whitefoord* 10247 (BM).

**GUATEMALA.***F. de la Puente* 3796 (CIP); Santa Elena, *R. Tun Ortíz* 2242 (BM, F); Alta Verapaz, *F.M. Barton* s.n. (K); Petén, P.N. Tikal, *E. Contreras* 502 (F).

**MEXICO. Chiapas**: Mun. Ocosingo, *E. Martínez & R. Lombera* 26176 (K); La Libertad, Chancala, *D.E. Breedlove & F. Almeda* 57818 (MO). **Quintana Roo**: Mun. Othón P. Blanco, desvio a Mérida, *J.L. Tapia-Muñoz* 1378 (MO).

#### Note.

The BM specimen of *Schipp* 878 is abnormal in having 5-lobed leaves.

### 217. Ipomoea peruviana

Taxon classificationPlantaeSolanalesConvolvulaceae

O’Donell, Bol. Soc. Peruana Bot. 1: 4 (O’Donell 1948b: 4)

Ipomoea acrensis J.R.I. Wood & Scotland, Kew Bull. 72(10): 2 (Wood and Scotland 2017b: 2). **Type.** BRAZIL. Acre, Mun. de Río Branco, Apa do Ireneu Derra, 12 July 2007, C. S. Pessoa, E. Consuelo, I.E.S. Moll, P. Palhares, Adriana, F. Obermüller, M. Silveira, I.M. Saar & W. Castro 302 (holotype RB501233).

#### Type.

PERU. Loreto, Balsapuerto, *G. Klug* 3089 (holotype S07-4771, isotypes BM, F, GH, K, MO, NY, US).

#### Description.

Twining perennial liana of unknown height; stems glabrous, somewhat woody. Leaves petiolate, 6–16 × 5–12 cm, ovate, shortly acuminate to a fine point, cordate, the auricles rounded or acute, margin undulate, sometimes 3-lobed to half way, often irregularly dentate, glabrous, paler beneath, thin in texture, main veins abaxially prominent; petioles 9.5–11 cm, glabrous. Inflorescence of up to 7-flowered, axillary, pedunculate compound cymes, glabrous; peduncles 12–15 cm, stout, woody; bracteoles not seen, caducous; secondary and tertiary peduncles c. 2.5 cm; pedicels 2.3–6.5 cm, conspicuously thickened upwards; sepals slightly unequal, outer 18–22 × 10–12 mm, narrowly oblong-elliptic, acute or obtuse, mucronate, inner sepals very slightly shorter, pale green; corolla c. 10–11 cm long, glabrous, pale blue, narrowly funnel-shaped, the tube 2–2.5 cm wide for 5–7 cm; limb 5–6 cm diam., apparently lobed. Capsules and seeds not seen.

#### Illustration.

Figures 108, 190E.

**Figure 108.**
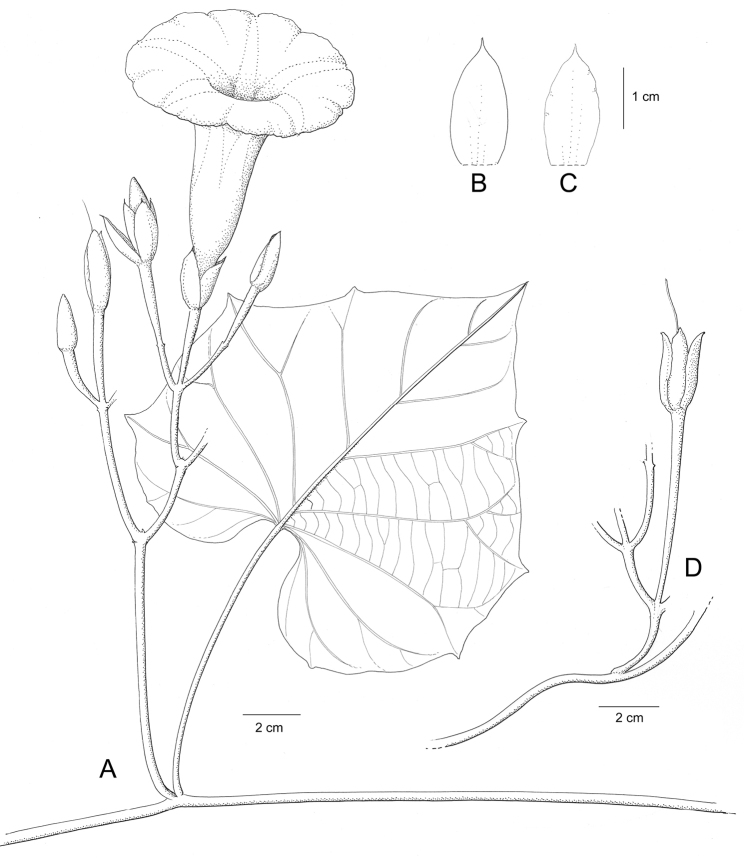
*Ipomoea peruviana*. **A** habit **B** outer sepal **C** inner sepal **D** fruiting calyx. Drawn by Rosemary Wise **A** from *Pessoa et al.* 302; **B, C** from *M. Alexiades & A. Byrne* 865; **D** from *A. Gentry et al.* 37636.

#### Distribution.

Amazonian Peru and Bolivia and neighbouring Acre in Brazil. It appears to be scattered in disturbed tropical rainforest over a wide area but uncommon.

**BRAZIL. Acre**: type of *Ipomoea acrensis*.

**BOLIVIA. Beni**: Marbán, Puente San Pablo, *M.T. Martinez & M. Adler* 83 (K, LPB, USZ). **Cochabamba**: Carrasco, Valle de Sajta, *J.R.I. Wood et al.* 28915 (K, LPB, USZ).

**PERU. Huánuco**: Huallaga valley, *A. Gentry et al.* 37636 (FTG, MO, OXF, USM); *J. Díaz in De La Puente* 4290 (CIP, FTG). **Loreto**: type of *Ipomoea peruviana*. **Madre de Dios**: Tambopata, *M. Alexiades & A. Byrne* 865 (NY, OXF, USM). **San Martín**: *G. Klug* 4326 (LIL, S).

#### Notes.

All parts of this species are glabrous, the inflorescence long-pedunculate and up to 7-flowered. The leaves may be entire or 3-lobed and the corolla is a characteristic pale blue.

Wood et al. (2017d: 11) discussed how *Ipomoea acrenis* had been confused with material of *I. cuscoensis* and as the type of *I. acrensis* belongs to *I. peruviana* it is here included as a synonym of *I. peruviana*.

•• Clade A3 (Species 218–233) comprises the Batatas Clade and a single sister species, *Ipomoea cryptica*. Unlike Clades A1 and A2, about half the species are annuals and none are woody. The pollen is also somewhat different (Figure 10 A, B) with relatively more echinulae.

• The Batatas Clade (Species 218–232) is an economically important clade containing the sweet potato and its crop wild relatives and is well supported in our 605 nuclear regions and chloroplast whole genome sequence data.

Annual or perennial herbs; stems trailing and rooting or twining, never woody. Leaves ovate, entire or 3–5-lobed but never divided into segments. Flowers in pedunculate cymes (only solitary by reduction), the pedicels commonly relatively short compared to the peduncles; bracteoles small, usually caducous; sepals equal or somewhat unequal, membranous, often chaffy in fruit, lanceolate or oblong to ovate or obovate, margins glabrous or ciliate, hyaline, the central vein prominent, laterals sometimes present; apex mucronate to caudate. Corolla relatively small (< 5 cm long), campanulate or funnel-shaped, glabrous, white, pink or pale pink with a dark pink throat, the midpetaline bands often terminating in small teeth; stamens often rather short; filaments with basal hairs sometimes extending upwards; anthers included. Ovary and capsule glabrous or hirsute, 2-locular, 4-seeded; seeds glabrous or sparsely pubescent.

Based on their morphology several species including *Ipomoea amnicola* and *I. cryptica* might be interpreted as belonging to this clade but both differ in their pilose seeds, while the latter also has very unequal sepals, the outermost very short. As *Ipomoea cryptica* is, in fact, sister to the Batatas Clade it is included it in the following key.

Most species are poorly defined morphologically, although our extensive nuclear data retrieves most taxa as monophyletic. Plants intermediate morphologically are not uncommon and are difficult to assign to species so specimens misidentified even by experienced *Ipomoea* specialists are commonly found in most herbaria. *Ipomoea cynanchifolia* appears to be morphologically intermediate between *I. ramosissima* and *I. grandifolia*, occurring only within the range of the latter. *Ipomoea grandifolia* itself resembles a large-flowered form of *I. triloba* and appears to be intermediate between *I. triloba* and perhaps *I. australis*. *Ipomoea leucantha* appears to be an intermediate between *I. cordatotriloba* and *I. lacunosa*. *Ipomoea tiliacea* and *I. littoralis* are difficult to separate except on molecular or geographical grounds and *I. tiliacea* has frequently been recorded from the Old World, probably always erroneously. Records of *I. littoralis* from Mexico have been shown to be errors for *I. batatas* (McDonald and Austin 1990). Cultivated *I. batatas* is usually easily identified by its perennial habit, trailing stem which roots at the nodes, ciliate sepals and subumbellate cymes but wild populations can be very difficult to separate from *I. tiliacea* on the one hand or *I. trifida* on the other. Forms of *Ipomoea batatas* are fairly commonly misidentified as *I. trifida* (Austin 1982a: 41; Deroin 2001) and some “species”, *I. confertiflora*, for example, have been treated as belonging both to *I. trifida* and *I. batatas*. This is not entirely surprising as there is strong evidence that *I. batatas* has evolved from *I. trifida* (Muñoz-Rodríguez et al. 2018).

Several species are more common near the sea or on islands, although not strictly maritime (*Ipomoea tiliacea*, *I. triloba* and possibly *I. tenuissima*). *Ipomoea littoralis* is the only truly maritime species although some forms of *I. batatas* (var. apiculata) occur on coastal sand dunes.

The species can be separated using the following key:

**Table:** 

1	Corolla < 2.5 cm long; plants mostly annual, always slender	**2**
–	Corolla > 2.5 cm long; plants perennial or annual, usually relatively robust	**7**
2	Outer sepals elliptic-obovate, 0–1 veined	**3**
–	Outer sepals lanceolate or oblong-lanceolate, 3–5 veined	**4**
3	Capsules ovoid, usually pilose; leaves usually thinly pubescent	**231. *I. cynanchifolia***
–	Capsules compressed-globose, glabrous; leaves usually glabrous, occasionally very thinly pubescent	**230. *I. ramosissima***
4	Sepals oblong, 5–6 mm long	**229. *I. triloba***
–	Sepals lanceolate, 8–13 mm long	**5**
5	Corolla white (very rarely pink); Capsules 10–15 mm diam	**224. *I. lacunosa***
–	Corolla pink (very rarely white); Capsules 6–9 mm diam	**6**
6	Sepals mostly 8–11 mm long (Brazil and neighbours)	**228. *I. grandifolia***
–	Sepals mostly 10–14 mm long (widespread, uncommon)	**225. *I. leucantha***
7	Sepals broadly obovate to suborbicular, usually white and papery; corolla 4.5–5.5 cm long (Central America)	**218. *I. splendor-sylvae***
–	Sepals oblong, lanceolate or obovate, always longer than broad; corolla < 4.5 cm long	**8**
8	Outermost sepal very short, 1–3 mm long; corolla pink; seeds long-pilose on margins......	**233. *I. cryptica***
–	Outermost sepal > 5 mm long; corolla pink or white; seeds glabrous or very shortly tomentellous	**9**
9	Slender, 1–2-flowered herb with pubescent strap-shaped sagittate leaves (Cuba, Florida, Hispaniola, Mona Island)	**232. *I. tenuissima***
–	Slender or robust herbs, 1–many-flowered; leaves not strap-shaped, rarely sagittate, but, if so, completely glabrous	**10**
10	Cymes 1–3-flowered; leaves somewhat fleshy, variable in shape, but characteristically with an obtuse to rounded mucronate apex and a very narrow basal sinus (coasts of the Indian and Pacific Oceans but absent from continental Africa and America)	**222. *I. littoralis***
–	Cymes 1–many flowered; leaves not fleshy, usually lacking the characteristic shape described above; new world species unless cultivated	**11**
11	Sepals glabrous; perennial twining plant with clearly cymose inflorescence	**12**
–	Sepals variously hirsute, but if glabrous, plant an annual weed or flowers clustered in a subumbellate inflorescence	**13**
12	Outer sepals 6–10 mm long, ovate to oblong-ovate or oblong-elliptic, strongly mucronate, margins scarious; corolla pink; filaments pubescent almost to apex	**221. *I. tiliacea***
–	Outer sepals 5–6.5 mm long, oblong-obovate, rounded, mucronulate, not scarious; corolla white or pale pink; filaments pubescent at base only	**223. *I. lactifera***
13	Sepals oblong-lanceolate	**14**
–	Sepals obovate, ovate or elliptic	**15**
14	Sepals chartaceous even at anthesis, unequal, the outer shorter than the inner, obscurely 1-veined	**219. *I. trifida***
–	Sepals not chartaceous at anthesis, equal in length or nearly so, obscurely 3-veined	**220. *I. batatas*** forms
15	Annual herb, not rooting at nodes; cymes always lax and few-flowered, never umbellate in form	**16**
–	Perennial herb, often decumbent and rooting at the nodes; cymes compact, umbellate or subcapitate in form	**220. *I. batatas***
16	Leaves entire to 5-lobed but usually 3-lobed, the central lobe contracted at the base; pedicels muricate; seeds with short hairs on angles	**226. *I. cordatotriloba***
–	Leaves entire (rarely 3-lobed, but if so, never with the lobe contracted at base); pedicels almost always smooth; seeds completely glabrous	**227. *I. australis***

### 218. Ipomoea splendor-sylvae

Taxon classificationPlantaeSolanalesConvolvulaceae

House, Muhlenbergia 3: 43. 1907. (House 1907b: 43)

Ipomoea umbraticola House, Ann. New York Acad. Sci. 18(6): 259. 1908. (House 1908b: 259). Type. COSTA RICA. Nicoya, A. Tonduz 13677 (holotype NY00547073, isotypes BM. K, US).

#### Type.

HONDURAS. Puerto Sierra, *P. Wilson* 286 (holotype NY00380475).

#### Description.

Twining herb to 3 m, probably a short-lived perennial; stems glabrous, often winged. Leaves petiolate, 2–13 × 2.5–10.5 cm, ovate, occasionally undulate to shallowly 3-lobed, cordate with rounded auricles, shortly acuminate, usually glabrous; petioles 1.5–4.5 cm. Inflorescence of axillary pedunculate cymes; peduncles 3.5–15 cm, usually straight; bracteoles c. 1 mm, deltoid, scarious, caducous; secondary peduncles 1–2.2 cm; tertiary peduncles c. 0.5 mm; pedicels 5–11 mm; sepals unequal, scarious, glabrous, outer 4–6 mm, orbicular, mucronulate, inner 7–10 mm, obovate, rounded usually minutely mucronate; corolla 4.5–6 cm long, funnel-shaped, pink, the tube dark purple inside, limb 4–4.5 cm diam.; filaments thinly covered in short glandular hairs. Capsules 7–9 × 5 mm, ovate, glabrous; seeds 4–5 × 2.5 mm, glabrous apart from relatively woolly deciduous marginal hairs 3–4 mm long.

#### Illustration.

Figures 7F, 109.

**Figure 109.**
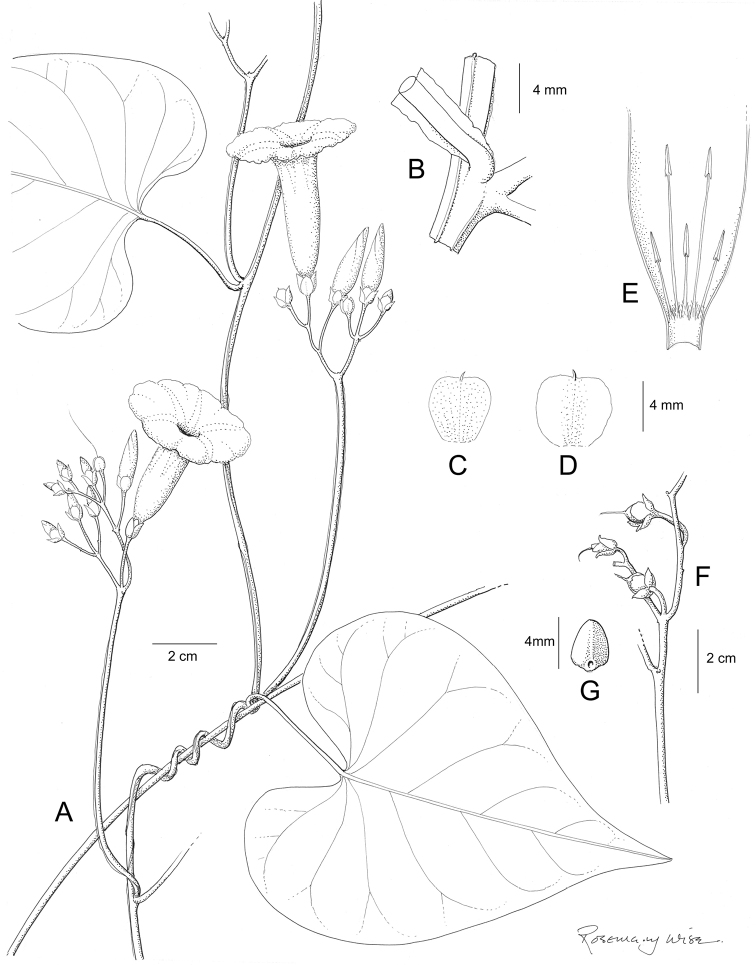
*Ipomoea splendor-sylvae*. **A** habit **B** winged stem **C** outer sepal **D** inner sepal **E** corolla opened out to show stamens **F** fruiting inflorescence **G** seed. Drawn by Rosemary Wise **A–D** from *Wilkin* 488; **E, F** from *Stevens & Montiel* 26745; **G** from *Khan et al.* 862.

#### Distribution.

Scattered in forest areas of Central America from southern Mexico to Costa Rica.

**COSTA RICA.** Guanacaste, Samara-Playa Carillo, *P. Wilkin* 465 (BM); ibid., Samara-Nicoya, *P. Wilkin* 472 (BM); ibid., Santa Cruz –Nicoya, *P. Wilkin* 488 (BM); Puntarenas, Isla Chira, *Khan et al.* 862 (BM); Guanacaste, Bagaces, *U. Chavarría* 1369 (K, MO); *B. Hammel et al.* 18688 (CR, MO).

**NICARAGUA.** Masaya, P.N. Volcán Masaya, *W.D. Stevens* 5233 (B, MO); Madriz, Somoto, *W.D. Stevens & O.M. Montiel* 26745 (BM, MO); Santa Rosa, Canyon of Río Sinecapa, *L.O. Williams & A. Molina* 42451 (BM); Chinandega, Volcán San Cristóbal. *P.P. Moreno* 25003 (BM).

**EL SALVADOR.** Ahuachapán, San Francisco Menéndez, *J.M. Rosales* (BM, MO); Libertad, Plan de la Laguna, *R. Villacorta* 499 (K); ibid., Mun. Antiguo Cuscatlan, *P. Lemus* s.n. [7/12/1988] (K).

**HONDURAS.** Colón, *J. Saunders* 1044 (FTG).

**BELIZE.** Chiquibul National Forest, *L. Urban* 90 (E); El Cayo, *P. H. Gentle* 2422 (K).

**GUATEMALA.***F. de la Puente* 3755 (FTG); Petén, camino Saepuy, *R. Tun Ortíz* 664 (BM, F, MO).

**MEXICO. Campeche**: *K.J. Virgo* 189 (K); *P. Alvaro* 653 (MBM, MEXU, MO); Calakmul, *E. Martínez et al.* 31649 (BM, MEXU, MO). **Chiapas**: *D.E. Breedlove* 40609 (MO); Pijijiapan–Arriaga, *A. Bourg* 159 (IEB). **Oaxaca**: Pochutla, *A. Sánchez Martínez et al.* 1187 (IEB). Quintana Roo: *C. & H. Cabrera* 4290 (MEXU). **Yucatán**: *G.F. Gaumer 23163* (MO); *E.F. & H. Cabrera* 10708 (MO).

#### Note.

*Ipomoea splendor-sylvae* is one of the most distinct species in the Batatas Clade because of its large pink flowers with a corolla usually around 5–6 cm long. The subspherical, white, chaffy calyx with broadly obovate to suborbicular glabrous sepals is also distinct.

### 219. Ipomoea trifida

Taxon classificationPlantaeSolanalesConvolvulaceae

(Kunth) G. Don, Gen. Hist. 4: 280. 1838. (Don 1838: 280)

Convolvulus trifidus Kunth, Nov. Gen. Sp. 3: 107. 1818 [pub.1819]. (Kunth 1819: 107). Type. VENEZUELA. Amazonas, Humboldt & Bonpland 1136 (holotype P00670762).
Ipomoea batatas forma trifida (Kunth) Nishiyama, Bot. Mag. Tokyo 84: 385. 1971. (Nishiyama 1971: 385).
Convolvulus hepaticifolius Willd. in Roem. & Schult., Syst. Veg. 4: 303. 1819. (Roemer and Schultes 1819: 303). Type. VENEZUELA. Carichana, Humboldt & Bonplands.n. (B-W-03709).
Ipomoea ramonii (“*ramoni*”) Choisy in A.P. de Candolle, Prodr. 9: 380. 1845. (Choisy 1845: 380). Type. CUBA. La Habana, *Ramón de La Sagra*s.n. (holotype G-DC00135860).
Ipomoea triloba forma ramonii (Choisy) Nishiyama, Bot. Mag. Tokyo 84: 385. 1971.
Ipomoea roseana House, Muhlenbergia 3: 43. 1907. (House 1907b: 43). Type. MEXICO. Colima, *E. Palmer* 978 (holotype US00111458).

#### Type.

Based on *Convolvulus trifidus* Kunth

#### Description.

Perennial twining herb, uniformly finely pubescent. Leaves petiolate, 2–11 × 2–10 cm, ovate or, more commonly, 3–(5)-lobed, acute to acuminate, apiculate, base cordate, pubescent on both surfaces, occasionally glabrous, abaxially paler; petioles 1.5–12.5 cm, thinly pubescent. Inflorescence of usually long-pedunculate axillary cymes; peduncles 3–26.5 cm, glabrous or, especially above, thinly pilose; bracteoles 1.5–2 × 1 mm, ovate, acute, scarious; secondary peduncles 0.5–4 cm; pedicels 3–7 mm, thinly pilose; sepals scarious, thinly pilose with only the central vein prominent, slightly unequal, outer 4–10 × 3 mm, elliptic or ovate, obtuse and mucronate, inner slightly longer; corolla 2.5–4 cm long, pink, glabrous, shortly funnel-shaped; limb 2.5–3.5 cm; nectary yellow. Capsules subglobose, 5–7 mm, glabrous or hairy; seeds 3–3.5 mm long, glabrous or nearly so.

#### Illustration.

**Figure 110.**
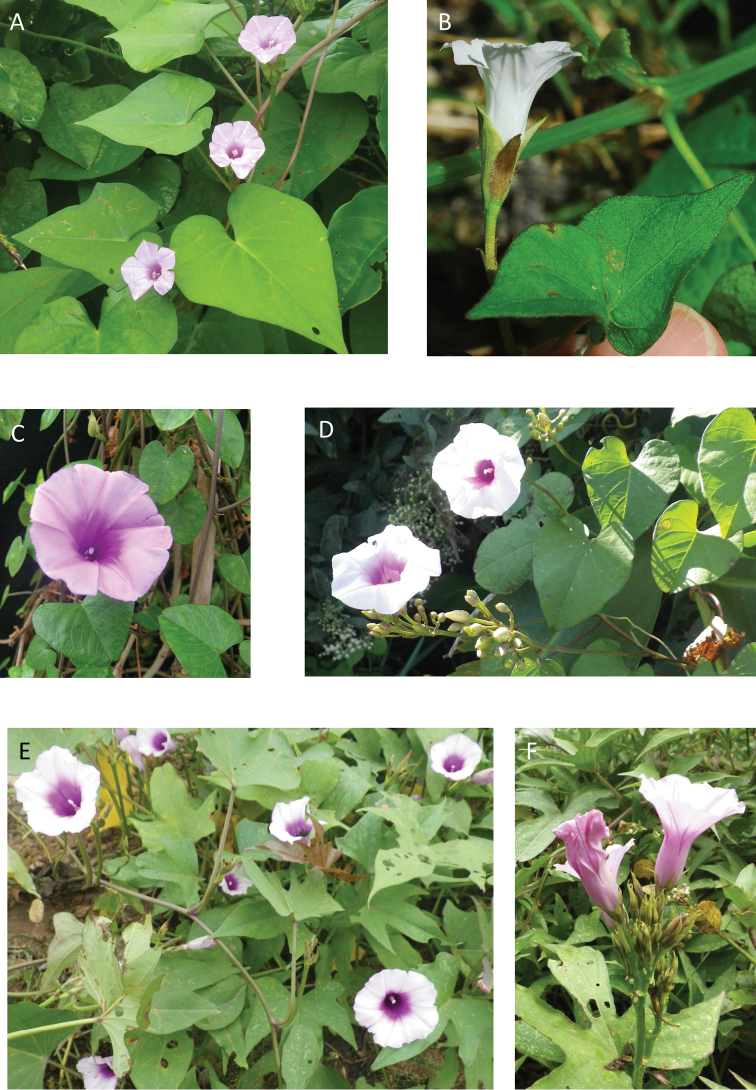
Photographs of *Ipomoea* species. **A***I. grandifolia***B***I. lacunosa***C***I. littoralis***D***I. cryptica***E, F***I. batatas*. **A**, **D–F** John Wood **B** Steven Turner **C** Rick Miller.

**Figure 111.**
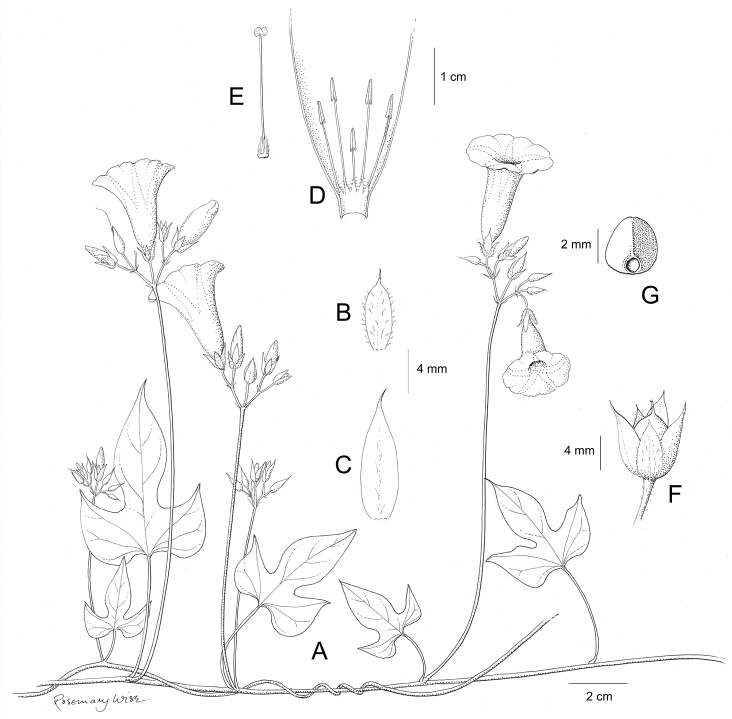
*Ipomoea trifida*. **A** habit **B** outer sepal **C** inner sepal **D** corolla opened out to show stamens **E** ovary and style **F** capsule **G** seed. Drawn by Rosemary Wise **A** from *Tun Ortíz* 321; **B–E** from *Miller* 77; **F, G** from *Seymour* 5436.

Figures 7H, 110D, 111.

#### Distribution.

Essentially Central American, mostly near the Caribbean coast, but absent from the Caribbean islands except Cuba and Trinidad. Records from Ecuador (Jørgensen 1999) and other places distant from Central America require confirmation and are probably errors for *Ipomoea batatas* (Austin 1982a: 41).

**COLOMBIA. Atlántico**: Palmar de la Verela-Pontedera, *A. Dugand* 3471 (COL). **Bolívar**: Cartagena, *J.A. Molina & F.A. Barklay* 19B024 (MO). **Magdalena**: *O. Haught* 3875 (COL, K, US), 4477 (S, US). **Santa Marta**: *H.H. Smith* 1569 (BM, COL, E, K, MO); ibid., *H.H.Smith* 1570 (BM, K, MO, S).

**VENEZUELA. Anzoategui**: *J. Steyermark* 115407 (P). **Aragua**: Tovar, *A. Fendler* 2074 (K); **Miranda**: 8 km beyond El Palmar on road to San José de Las Altos, *C. Jeffrey & B. Trujillo* 2351 (K). **Nueva Esparta**: Margarita Island, *O.O. Miller & J.R. Johnston* 77 (BM, K).

**PANAMA.***Sinclair* s.n. (K); canal area, Las Cruces trail, *A.A. Hunter & P.H. Allen* 723 (K, MO); Frijoles, *H. Pittier* 2677 (BM); Santiago, *B.L. Seeman* s.n. (BM, K).

**COSTA RICA.***A.F. Skutch* 2570 (S); Playa Maranjo, *P. Wilkin* 416 (BM); Guanacaste, *P. Wilkin & S.B. Jennings* 109 (BM); Guanacaste, *E. López* 98 (MO, BM); Alajuela, *B. Hammel* 19715 (MO, BM); Puntarenas, *M. Chavarría* 601 (K); Bagaces-Libería *M. Chavarría* 1051 (K, MO); Heredia, Sarapiqui, *I. Chacón* 439 (MO); Guanacaste, Monteverde, *D.F. Austin* 7848 (FTG, MO).

**NICARAGUA.***A. Molina* 20101 (F); Managua, *F.C. Seymour* 5436 (BM); ibid., *W.D. Stevens* 4772 (BM, MO), Matagalpa, Cerro El Pilon, *W.D. Stevens* 9428 (BM, MO); Bealego, *Sinclair s.n.* (K).

**HONDURAS.** Colonia Miramonte, *M.G. Pineda* 97 (BM, TEFH); *J. Hjalmarsan* (S).

**EL SALVADOR.** La Libertad, *K. Sidwell et al.* 510 (BM, LAGU, MO); *P.C. Standley* 21292 (S); Morazán, Montecristo, *J.M. Tucker* 444 (K, UC).

**BELIZE.** Stann Creek, *D. Dwyer et al.* 579 (MO).

**GUATEMALA.***H. Bartlett* 315 (S); Petén, P.N. Tikal, *R. Tun Ortíz* 321 (BM, MO); ibid., 388 (BM, F, MO).

**MEXICO. Campeche**: *E.F. Cabrera* 12504 (XAL) fide Austin. **Chiapas**: Escuintla, *E. Matuda* 2154 (K). **Colima**: *J. Maillet* s.n. [1985] (IEB). **Est. México & Dist. Fed.**: Nanchititla, Temascaltepec, *G.B. Hinton* 3434 (K). **Guerrero**: Petatlán, *E. Langlassé* 630 (K); Temisco, *Y. Mexia* 8865 (K, S); Acapulco, *W. Hancock* 31 (K); Santa Bárbara, Coyuca, *G.B. Hinton* 8504 (K); Pino, Mina *G.B. Hinton* 9784 (K); Acapulco, *E. Palmer* 123 (K). **Jalisco**: *P. Carillo-Reyes et al.* 3622 (IEB). **Michoacán**: Coalcomán, *G.B. Hinton et al.* 12513 (K); Coahuayana, *J.C. Soto Nuñez et al.* 11169 (MEXU). **Oaxaca**: *S. Salas et al.* 4780 (ARIZ, MO); *H. Hernández* 876 (IEB). **Tabasco**: Paraíso, *E.F. & H. Cabrera* 14740 (MO) fide Austin. **Quintana Roo**: José María Morelos, Chichancanab, *G.F. Gaumer* 2117 (MO) fide Austin. **Veracruz**: Córdoba-Veracruz, *D.F. & S. Austin* 5060 (FTG); 9 km S of Tampico, *E. Palmer* 543 (BM, K). **Yucatán**: *J.S. Flores* 9262 (XAL) fide Austin.

**CUBA.***H. Manitz* 51339 (HAJB); *F. de la Puente* 5341 (CIP, FTG). **Artemisa**: Guanajay, *A.H. Curtiss* 632 (BM, K, NY). **Guantánamo**: Bayate, *E.L. Ekman* 10120 (BM, S). **La Habana**: *H. Van Hermann* 231 (BM, NY). **Matanzas**: Herradura, *F.S. Earle* s.n. [2/11/1906] (NY). **Pinar del Río**: Sierra de Anafé, *N.L. Britton et al*. 9593 (K, NY). **Santiago de Cuba**: *M. López Figueiras* 307 (HAC, HAJB, NY).

**TRINIDAD.***A. Fendler* 583 (K).

**Notes**. Although common in Central America and on Cuba, this species is frequently misidentified and records from Africa, Madagascar, Paraguay, Peru, Ecuador, Brazil, northern Mexico, most Caribbean islands and the United States are probably erroneous. Even within its area of occurrence, many specimens may be wrongly named.

*Ipomoea trifida* has unequal, narrow, oblong-lanceolate, characteristically chaffy sepals. The only other species with distinctly chaffy sepals are *I. splendor-sylvae* with obovate to suborbicular sepals and some unassigned forms discussed below under *I. batatas*, which itself has arisen from *I. trifida* (Muñoz-Rodríguez et al. 2018). Some specimens are difficult to separate but the shape and texture of the sepals is usually decisive. *Ipomoea trifida* is reported (Austin 1978a) to have filaments hirsute for most of their length but it is not certain how constant this character is.

### 220. Ipomoea batatas

Taxon classificationPlantaeSolanalesConvolvulaceae

(L.) Lam., Tabl. Encycl. 1: 465. 1793. (Lamarck 1793: 465)

Convolvulus batatas L., Sp. Pl. 1: 154. 1753. (Linnaeus 1753: 154). Type. INDIA. Herb. Linn. No.77.5 (S, lectotype designated by Biju, 2003 755).
Convolvulus esculentus Salisb., Prodr. Stirp. Chap. Allerton 123. 1796. (Salisbury 1796: 123), nom. illeg. superfl. Type. Based on Convolvulus batatas L.
Convolvulus edulis Thunb. ex Murray, Syst. Veg., ed. 14: 203. 1784. (Murray 1784: 203). Type. JAPAN. *Thunberg* (holotype UPS).
Batatas edulis (Thunb. ex Murray) Choisy, Mem. Soc. Phys. Genève 6: 435 [53]. 1834. (Choisy 1834: 435 [53]).
Ipomoea edulis (Thunb. ex Murray) Niederl., Bol. Mens. Mus. Prod. Argent. 3 (29): 190. 1890. (Niederlein 1890: 190).
Ipomoea batatas var. edulis (Thunb. ex Murray) Makino, Fl. Japan 476. 1925. (Makino 1925: 476).
Convolvulus platanifolius Vahl, Symb. Bot. 3: 26. 1794. (Vahl 1794: 26). Type. Illustration in L. Plukenet (1692: t.167, f. 3), lectotype designated here.
Ipomoea platanifolia (Vahl) Roem. & Schult., Syst. Veg. 4: 220. 1819. (Roemer and Schultes 1819: 220).
Ipomoea fastigiata var. platanifolia (Vahl) Griseb., Fl. Brit. W.I. 468. 1864 [pub. 1862]. (Grisebach 1862b: 468).
Ipomoea villosa Ruiz & Pav., Fl. Peruv. 2: 12, t. 121. 1799. (Ruiz and Pavón 1799: 12). Type. ECUADOR. Guayaquil, *Ruiz, Pavón & Dombey* (lectotype MA814679, designated here; isolectotypes BM, OXF).
Ipomoea catesbaei G. Mey., Prim. Fl. Esseq. 103. 1818. (Meyer 1818: 103). Type. Based on Catesby 2: 60, t. 60, lectotype designated here.
Convolvulus fastigiatus Roxb., Fl. Indica, ed. 2, 2: 48. 1824. (Roxburgh 1824: 48) Type. INDIA. Bengal, (lectotype, icon Roxburgh 1355 (K), designated here).
Ipomoea fastigiata (Roxb.) Sweet, Hort. Brit., ed. 1: 188. 1826. (Sweet 1826: 188).
Ipomoea batatas var. fastigiata (Sweet) Kuntze, Rev. Gen. Pl. 2: 442. 1891. (Kuntze 1891: 442).
Convolvulus edulis Vell. Fl. Flumin. 72. 1825 [pub. 1829]. (Vellozo 1829: 72), nom. illeg., superfl. based on Convolvulus batatas L.
Convolvulus tuberosus Vell. Fl. Flumin. 72. 1825 [pub. 1829]. (Vellozo 1829: 72), nom. illeg., non Convolvulus tuberosus Spreng. (1824). Type. BRAZIL. Not specified, (lectotype, original parchment plate of Flora Fluminensis in the manuscript section of the Biblioteca Nacional, Rio de Janeiro [cat. no.: mss1198651-057], designated here; later published in Vellozo, Fl. Flum. Icon. 2: t. 57. 1827 [pub. 1831]).
Convolvulus esculentus Vell., Fl. Flumin. 73. 1825 [pub. 1829]. (Vellozo 1829: 73), nom. illeg., non Convolvulus esculentus Salisb. (1796). Type. BRAZIL. Not specified, (lectotype, original parchment plate of Flora Fluminensis in the manuscript section of the Biblioteca Nacional, Rio de Janeiro [cat. no.: mss1198651-058], designated here; later published in Vellozo, Fl. Flum. Icon. 2: t. 58 1827. [pub. 1831]).
Convolvulus batata Vell., Fl. Flumin. 73. 1825 [pub. 1829]. (Vellozo 1829: 73). Type. BRAZIL. Not specified, (lectotype, original parchment plate of Flora Fluminensis in the manuscript section of the Biblioteca Nacional, Rio de Janeiro [cat. no.: mss1198651-059], designated here; later published in Vellozo, Fl. Flum. Icon. 2: t. 59 1827. [pub. 1831]).
Convolvulus cordatifolius Vell., Fl. Flumin. 73. 1825 [pub. 1829]. (Vellozo 1829: 73). Type. BRAZIL. Not specified, (lectotype, original parchment plate of Flora Fluminensis in the manuscript section of the Biblioteca Nacional, Rio de Janeiro [cat. no.: mss1198651-060], designated here; later published in Vellozo, Fl. Flum. Icon. 2: t. 60 1827. [pub. 1831]).
Convolvulus varius Vell., Fl. Flumin. 73. 1825 [pub. 1829]. (Vellozo 1829: 73). Type. BRAZIL. Not specified, (lectotype, original parchment plate of Flora Fluminensis in the manuscript section of the Biblioteca Nacional, Rio de Janeiro [cat. no.: mss1198651-061], designated here; later published in Vellozo, Fl. Flum. Icon. 2: t. 61 1827. [pub. 1831]).
Convolvulus variabilis Schltdl. & Cham., Linnaea 5: 116. 1830. (Schlechtendal and Chamisso 1830: 116). Type. MEXICO. Veracruz, Hacienda de la Laguna, Schiede & Deppes.n. (holotype HAL0037741, isotype LE, n.v.).
Ipomoea variabilis (Schltdl. & Cham.) Choisy in A.P. de Can.dolle, Prodr. 9: 383. 1845. (Choisy 1845: 383).
Ipomoea indica var. variabilis (Schltdl. & Cham.) L.O. Williams, Fieldiana, Bot. 32 (12): 191. 1970. (Williams 1970a: 191).
Batatas xanthorhiza Bojer, Hort. Maurit. 225. 1837. (Bojer 1837: 225). Type. MAURITIUS. “Cult. Danes les habitations”. No specimen cited.
Batatas edulis var. xanthorhiza (Bojer) Choisy in A.P. de Candolle, Prodr. 9: 338. 1845. (Choisy 1845: 338).
Batatas betacea Lindl., Bot. Reg. (Edwards) 25: 93. 1839. (Lindley 1839c: 93). Type. No specimen preserved, lectotype t. 56 in Bot. Reg. (Edwards) 26 (1839), designated here.
Ipomoea apiculata M. Martens & Galeotti, Bull. Acad. Roy. Sci. Bruxelles 12(2): 262. 1845. (Martens and Galeotti 1845: 262). Type. MEXICO. Veracruz, *H. Galeotti* 1381 (lectotype BR00006972851, designated here; isolectotypes BR, P).
Ipomoea batatas var. apiculata (M. Martens & Galeotti) J.A. Mcdonald & D.F. Austin, Brittonia 42 (2): 118. 1990. (McDonald and Austin 1990: 118).
Convolvulus attenuatus M. Martens & Galeotti, Bull. Acad. Roy. Sci. Bruxelles 22: 265. 1845. (Martens and Galeotti 1845: 265). Type. MEXICO. Oaxaca, *H.G. Galeotti* 1399 (syntypes BR, P, G, MEXU).
Batatas wallii Morren, Ann. Soc. Roy. Agric. Gand. 2: 285–286, t. 74. 1846. (Morren 1846: 285). Type. GUATEMALA. *Père Wall de Poperingue*s.n. (whereabouts uncertain).
Ipomoea wallii (Morren) Hemsl., Biol. Cent.-Amer., Bot. 2 (11): 396. 1882 (Hemsley 1882: 396).
Ipomoea batatas var. leucorrhiza Griseb., Fl. Brit. W. Ind. 468. 1864 [pub. 1862]. (Grisebach 1862b: 468). Type. ANTIGUA. Wullschlagels.n. (whereabouts unknown).
Ipomoea batatas var. porphyrorhiza Griseb., Fl. Brit. W. Ind. 468. 1864 [pub. 1862]. (Grisebach 1862b: 468). Type. JAMAICA. collector and whereabouts unspecified.
Batatas edulis var. porphyrorhiza (Griseb.) Ram. Goyena, Fl. Nicarag. 2: 649. 1911. (Ramírez Goyena 1911: 649).
Ipomoea batatas var. dissoluta Kuntze, Rev. Gen. Pl. 2: 442. 1891. (Kuntze 1891: 442). Type. Not specified.
Ipomoea batatas var. subscandens Kuntze, Rev. Gen. Pl. 2: 442. 1891. (Kuntze 1891: 442). Type. INDIA. Deccan, not specified.
Ipomoea fastigiata var. ciliata Huber, Bol. Mus. Paraense Hist. Nat. Ethnogr. 2: 512. 1898. (Huber 1898: 512). Type. BRAZIL. Para, Rio Anauerá-pucú, *M. Guedes* 582 (holotype MG).
Ipomoea vulsa House, Muhlenbergia 3 (3): 45. 1907. (House 1907b: 45). Type. MEXICO. Veracruz, Orizaba, *F. Mueller*s.n. (holotype US00111491, isotypes NY, US).
Ipomoea purpusii House, Ann. New York Acad. Sci. 18: 248. 1908. (House 1908b: 248). Type. MEXICO. Veracruz, near Zacuapan, C.A. Purpus 2113 (holotype NY00319135, isotypes F, US).
Ipomoea batatas var. lobata Gagnep. & Courchet, Fl. Indochine 4: 241.1915. (Gagnepain and Courchet 1915: 241). Type. VIETNAM. Tonkin, Ninh-binh, Bon s.n. & Long-Tcheou, Beauvais (syntypes P?, n.v.).
Ipomoea confertiflora Standl., Publ. Carnegie Inst. Wash. 461: 83. 1935. (Standley 1935: 83). Type. BELIZE. Río Grande, W.A. Schipp 1236 (holotype F0054833, isotypes A, BM, GH, K, MICH, MO, NY, S).
Ipomoea davidsoniae Standl., Publ. Field Nus. Nat. Hist., Bot. Ser. 22: 98. 1940. (Standley 1940c: 98). Type. PANAMA. Chiriqui, Bajo Mono, *M.E. Davidson* 595 (holotype F0O54838, isotype MO).
Ipomoea mucronata Schery, Ann. Missouri Bot. Gard. 28: 463. 1941. (Woodson and Schery 1941: 463). Type. PANAMA. Chiriqui, near Peña Blanca, *R.E. Woodson & R.W. Schery* 323 (holotype MO00340730).
Ipomoea batatas forma trifida Moldenke, Phytologia 2: 224. 1947. (Moldenke 1947: 224). Type. ECUADOR. Loja, La Toma, R. Espinosa 492 (holotype NY00319162).
Ipomoea tiliacea var. merremioides Fosberg, Smithsonian Contrib. Bot. 21: 15. 1975. (Fosberg and Sachet 1975: 14). Type. FRENCH POLYNESIA. Hiva Oa Island, *M.H. Sachet* 1300 (holotype US00111475, isotype P).
Ipomoea tiliacea var. smithii Fosberg, Smithsonian Contrib. Bot. 21: 15. 1975. (Fosberg and Sachet 1975: 15). Type. FIJI. Viti Levu, *A.C. Smith* 4468 (holotype US00111476, isotype BISH).
Ipomoea tabascana J.A. McDonald & D.F. Austin, Brittonia 42: 116. 1990. (McDonald and Austin 1990: 116). Type. MEXICO. Tabasco, S. limit of Ejido López Zamora, *D.F.Austin & F. de la Puente* 7505 (holotype not at US, isotypes CIP [Lima], FTG, XAL, n.v.).

#### Type.

Based on *Convolvulus batatas* L.

#### Description.

Creeping (rarely climbing) perennial herb rooting from the stem and developing storage roots; stems extending to cover several metres, glabrous to coarsely pilose, often stiut in cultivated and feral forms. Leaves petiolate, very variable in form but usually rather large, 3–15 × 5–12 cm, ovate or shallowly to deeply 3–5-lobed, cordate, shortly acuminate, both surfaces glabrous to coarsely pilose, abaxially somewhat glaucous and with prominent veins; petioles usually rather long, 4–15 cm. Inflorescence of long-pedunculate, axillary, dense umbellate cymes; peduncles 5–30 cm long, stout; bracteoles filiform, c. 2 mm long, caducous; secondary peduncles 5–15 mm; pedicels very short, 5–10 mm long; sepals 7–11 mm, unequal, margins often but not always ciliate, outer slightly shorter than inner, oblong-elliptic to oblong-oblanceolate, abruptly mucronate with a hair point c. 2 mm long, prominently 1–5-veined, the inner sepals broadly elliptic, rounded and mucronate; corolla 4–4.5 cm long, pink, often with a dark centre, glabrous; ovary pubescent, rarely fertile so capsules and seeds usually absent.

#### Illustration.

Figure 110E, F; Acevedo-Rodríguez (2005: 165); Bosser and Heine (2000: 35); Deroin (2001: 173, 247) (as *Ipomoea trifida*).

#### Distribution.

The sweet potato is of American origin but is now cultivated throughout tropical and subtropical regions of the world with greatest production reported from China. We have seen examples of cultivated plants from all parts of the Americas including Easter Island [*F. Fuentes* 3 (K), 4 (K)] and Hawaii [*J. Stokes* s.n. 1/1912 (K); Oahu, *Christopherson et al.* 1594 (K)] with the exception of the extreme south and Canada. Outside cultivation, plants are usually found in derelict fields and on roadsides near settlements Most cultivated plants are sterile but we have seen occasional specimens of apparently wild, fertile plants from various countries in tropical America including Colombia, Ecuador, Mexico, Panama and Venezuela. No apparently naturally occurring populations are reported from the Caribbean islands, Brazil or the Guianas. Obviously cultivated plants are not cited below but many of the records are of escapes from cultivation although some may be of wild populations.

**FRENCH GUIANA.***Berthoud-Coulon* 505 (BM).

**SURINAM.***M. Berthoud-Coulon* 507 (BM)

**BOLIVIA.** (escapes from cultivation). **La Paz**: Murillo, Valle de Zongo, Cahua, 1300 m, 14 June 1980, *S.G. Beck* 3688 (CTES, CUSCO, FTG, LPB, MO, USZ). **Santa Cruz**: Ñuflo de Chávez, Concepción *J.R.I. Wood & D. Soto* 27939 (OXF, K, LPB, USZ); *J.R.I. Wood et al.* 28090 (LPB, OXF, USZ).

**PERU. Huánuco**: *J. Schunke* 2013 (G). **Lambayeque**: *T. Torres* s.n. (USM). **Loreto**: Chanintía, *A. Montalvo* s.n. (USM); Boquerón, *R. Ferreyra* 1185 (USM); Iquitos, *H. Murphy* 301 (MO, OXF). **Madre de Dios**: Tambopata, Puerto Maldonado, *I. Huamantupa & A. Montero* 3671 (MO, OXF). **Pasco**: Oxapampa, Huancabamba, camino a Pozuzo, *R. Rojas et al.* 2513 (MO, OXF). **Piura**: *E. Laure* 5326 (P), 5370 (P).

**ECUADOR. Cotapaxi**: *B. Sparre* 17329 (S). **El Oro**: Arenillas, *E. Asplund* 15676 (K, S). **Guayas**: San Ignacio, *I. Holmgren* 88 (S); Guayaquil, *L. Fraser* (BM). **Los Ríos**: *B. Sparre* 17916 (S); C. *Játiva & C. Epling* 182 (S). Manabí: *J. Brandbyge* 42773 (AAU, ARIZ); *Eggers* 15105 (P). **Pinchincha**: *B. Sparrre* 14820 (S).

**COLOMBIA. Antioquia**: Angelópolis, *G. Gutiérrez & F. Barklay* 17C654 (BM). **Boyacá**: *A.E. Lawrance* 544 (BM). **Cauca**: La Paila, *I.F. Holton* s.n. [1853] (K). **Cundinamarca**: La Mesa, *J. Triana* 3807 (BM). **Magdalena**: Santa Marta, *H.H. Smith* 1912 (E). **Meta**: Villavicencio, *J. Triana* 3803 (BM). **Putumayo**: *J. Ewan* 16705 (BM). **Valle**: *A. Gentry et al.* 59527 (FTG).

**VENEZUELA. Dist. Fed.**: Caracas-Guayra, A.H.G. Alston 5500 (BM). **Zulia**: *A. Fernández* 20591 (MA).

**PANAMA.***B.L. Seeman* 488 (BM), 1604 (BM), 6453 (BM, MO); *E.L. Tyson* 6994 (BM, PMA); *C. Whitefoord & A. Eddy* 71 (BM); *C. Hamilton et al.* 1300 (FTG, MO); *A. Ibañez et* al. 1804 (MA).

**COSTA RICA.***A.F. Skutch* 2570 (K), 3672 (K); *H. Pittier* 13675 (K); Santa Elena-San Rafael, *P. Wilkin* 436 (BM); Puntarenas, Cordillera de Talamanca, *F. Quesada* et al. 1147 (BM); Limon, *B. Hammel et al.* 19673 (BM).

**NICARAGUA.***M. Araquistain & J.C. Sandino* 1384 (FTG); Zelaya, *W.D. Stevens et al.* 6453 (BM, MO).

**EL SALVADOR.***G. Davidse et al*. 37459 (MO).

**HONDURAS.** Gracias a Dios, *P. House* 37 (BM); *J. Saunders* 709 (FTG).

**BELIZE.** Georgeville-Augustine, *G.R. Proctor* 29630 (BM); *D.R. Hunt* 150 (BM).

**GUATEMALA.** Alta Verapaz, *H. von Türckheim* 1437 (K); *Bernoulli & Cario* 1906 (K).

**MEXICO. Campeche**: *E. & H. Cabrera* 13444 (BM, MEXU, MO). **Chiapas**: *A. Reyes-García & E. Martínez* 132 (BM, MEXU); *J.C. Soto et al.* 13219 (BM, MEXU). **Est. México & Dist. Fed.**: Temascaltepec: *G.B. Hinton* 2009 (K). **Guerrero**: *G.B. Hinton* 8501 (K), 9510 (K); Mina, 9699 (K). **Oaxaca**: *D.F. Austin & F. de la Puente* 7672 (FTG). **Quintana Roo**: Isla de Cozumel, *E. & H. Cabrera* 10541 (BM, MEXU). **Veracruz**: *E. Kerber* 37 (BM); *M. Botteri* 560 (BM, K, OXF); *J. Linden* 257 (K); *H. Galeotti* 1351 (K); Bandaril, Jalapa, *E.K. Balls & W.B. Gourlay* 5483 (E, BM); *Gouin* s.n. [1867] (P).

#### Typification.

Although often claimed to be an illegitimate name, *Ipomoea fastigiata* (Roxb.) Sweet appears to have been validly published. Sweet refers to Flora Indica, not Hortus Benghalensis but incorrectly gives the date as 1816, which is, in fact, incorrect for both these publications.

#### Notes.

*Ipomoea batatas* appears to have arisen naturally in pre-human times in Tropical America and is most closely related to *I. trifida*. Its origins are discussed by Muñoz-Rodríguez et al. (2018). It is widely cultivated throughout the tropics and the orange-fleshed variety is of particular importance as it is rich in a precursor of Vitamin A.

*Ipomoea batatas* is usually readily identified in the field because of its root tubers and perennial creeping habit, the stems rooting at the nodes. Herbarium specimens are distinguished by the strongly and usually abruptly mucronate sepals with a distinct mucro and a pronounced central vein with 2–4 less prominent lateral veins. The sepals are usually ciliate and the flowers characteristically clustered in a subumbelliform structure at the apex of a long peduncle. The leaves are commonly 3-lobed.

#### Variation.

Various apparently wild forms of *Ipomoea batatas* are relatively distinct morphologically and have been recognized over the years. The plant treated as Ipomoea batatas var. apiculata (*I. apiculata*, *I. vulsa*) represents a form from coastal sand dunes near Veracruz but is also found in Campeche and Oaxaca. It is a slender plant, rooting at the nodes or twining, with deeply 3–5(–7)-lobed glabrous leaves, cymes of 1–3 flowers, very unequal sepals (the outer oblong, mucronate much narrower and shorter than the inner obovate sepals) and a distinctly campanulate corolla c. 3 cm long but with a tube c. 1.5 cm wide. We have seen the following additional specimens:

**MEXICO. Campeche.***E. & H. Cabrera* 12504 (IEB). **Oaxaca**: *Ghiesbrecht* s.n. (P03548796)

**Veracruz**: *D.F. Austin & F. de la Puente* 7480 (FTG), *Vislet* 1856 (P), *G. Castillo-Campos et al.* 1438 (IEB), *E. Matuda* 17095 (MEXU).

The plants described as *Ipomoea tabascana* are very slender glabrous plants, rooting at the nodes and with strap-shaped, strongly sagittate leaves and few-flowered cymes. They are only known from marshy ground near the type locality in Tabasco (Austin et al. 1991). Molecular studies show *I. tabascana* to be nested within *I. batatas*, probably representing a hybrid between *I. batatas* and *I. trifida*. (Srisuwan et al. 2006; Muñoz-Rodríguez et al. 2018).

*Ipomoea confertiflora* also appears somewhat distinct and has sometimes been treated as belonging to *I. trifida* (Austin and Huáman 1996) because of the somewhat scarious sepals. It has oblong, somewhat twisted outer sepals. As well as the type, *M. Araquistain & J.C. Sandino* (FTG) from Nicaragua, *N. Garwood et al*. 819 (BM) from Costa Rica, *I.F. Holton* 540 (K) from Cauca, Colombia and *C. H. Dodson* 3811 (F) from Ecuador fit this form.

Two distinct forms come from Ecuador. One of these is represented by the type of *Ipomoea villosa* Ruiz & Pav. from Guayaquil, which has been treated as *I. leucantha*. This has trilobed leaves and long, lanceolate, acuminate sepals 13–14 mm in length. Very similar is *Asplund* 15966 (S) from Manta, Manabí Province and *U. Chavarria* 1343 (BM, MO) from Costa Rica. Somewhat similar plants with entire leaves and slightly shorter sepals come from Pinchincha in Ecuador (*B. Sparre* 14810 (S) and Piura in Peru (*E. Laure* 5370 (P)). All of these plants have a large corolla 4.5–5 cm in length. They integrade with more typical forms of *I. batatas* in western Ecuador.

Another distinct form comes from around Esmeraldas in Ecuador. This is a glabrous or sparsely pubescent twining herb with unequal, chartaceous, obovate to obrhomboid sepals with a single prominent central nerve extended as a mucro, the outer sepals 5–6 × 3 mm, the inner 7–8 × 4 mm. This was identified as a tetraploid form of *Ipomoea batatas* by Austin et al. (1992) although it had sometimes been previously identified as *I. triloba* or *I. trifida*. Examples include *H. Balslev & W.C. Steere* 3131 (GB), *J. Hudson* 730 (MO, US), *L. Holm-Nielsen et al.* 25318 (AAU, ARIZ) and *B. Sparre* 15286 (S), 15308 (S), 15341 (S) and 15517 (S).

### 221. Ipomoea tiliacea

Taxon classificationPlantaeSolanalesConvolvulaceae

(Willd.) Choisy in A.P. de Candolle, Prodr. 9: 375. 1845. (Choisy 1845: 375)

Convolvulus tiliaceus Willd., Enum. Pl. 1: 203. 1809. (Willdenow 1809: 203). Type. BRAZIL. Hoffmanseggs.n. (holotype B-W03691-01).
Convolvulus indicus Miller, Gard. Dict., ed. 8: 5. 1768 (Miller 1768: 5), nom. illeg., non Convolvulus indicus Burm. (1755). Type. JAMAICA W. Houston (holotype BM000953181).
Ipomoea cymosa G. Mey., Prim. Fl. Esseq. 99. 1818. (Meyer 1818: 99). Type. SURINAM. *E.K. Rodschied* 89 (GOET 002526).
Ipomoea surinamensis Miq., Linnaea 18: 600. 1845. (Miquel 1845: 600). Type. SURINAM. *H.C. Focke* 816 (holotype U0001416).
Ipomoea alba Garcke, Linnaea 22: 66. 1849. (Garcke 1849: 66), nom. illeg., non. I. alba L. (1753). Type. SURINAM. *H. Kegel* 960 (GOET002527).
Ipomoea stenocolpa Garcke, Linnaea 22: 67. 1849. (Garcke 1849: 67). Type. SURINAM. Paramaribo, *H. Kegel* 987 (holotype GOET002528).
Ipomoea fastigiata var. vulgaris Meisn. in Martius et al., Fl. Brasil. 7: 267. 1869. (Meisner 1869: 267). Type. BRAZIL. Salzman 360 (lectotype P03538439, designated here).
Convolvulus umbellatus Sessé & Moçiño, Pl. Nov. Hisp. 22 1887 [pub. 1888]. (Sessé and Moçiño 1887–1890: 220), nom. illeg., non Convolvulus umbellatus L. (1753). Type. MEXICO. *Sessé & Moçiño*s.n. (MA5017).
Convolvulus biflorus Sessé & Moçiño, Fl. Mex. 35. 1893. (Sessé y Lacasta and Moçiño 1893: 35), nom. illeg., non Convolvulus biflorus L. (1763). Type. MEXICO. *Sessé & Moçiño* 5048 (probable holotype MA603862).
Ipomoea fastigiata var. pauciflora Meisn., Meisn. in Martius et al., Fl. Brasil. 7: 267. 1869. (Meisner 1869: 267). Type. BRAZIL. Raben 283 (lectotype BR00005306763, designated here).

#### Type.

Based on *Convolvulus tiliaceus* Willd.

#### Description.

Twining perennial herb to several metres in height, usually glabrous in all vegetative parts; stems woody below, herbaceous above. Leaves petiolate, 4–16 × 2.2–11 cm, ovate, shortly acuminate and mucronate (rarely retuse), base cordate with rounded (rarely acute or dentate) auricles, margin entire or (rarely) somewhat dentate, abaxially paler; petioles 1–13 cm. Inflorescence of axillary pedunculate cymes; peduncles 1.5–8 cm; bracteoles ovate, c. 1 mm, caducous; secondary peduncles 0.2–1.5 cm; pedicels 5–15 mm; sepals slightly unequal, glabrous, outer 6–10 × 3–4 mm, ovate to oblong-ovate or oblong-elliptic, strongly mucronate, margins scarious, inner 9–11 × 4–7 mm, elliptic to obovate, obtuse and mucronate, scarious; corolla 3.5–6 cm long, pink often with a dark centre, glabrous, funnel-shaped, limb 4.5–5 cm, undulate but midpetaline bands ending in small teeth; filaments thinly pubescent for half their length. Capsules c. 8 × 9 mm, depressed globose, glabrous; seeds c. 4 × 3 mm, black, glabrous or shortly pubescent on the angles.

#### Illustration.

**Figure 112.**
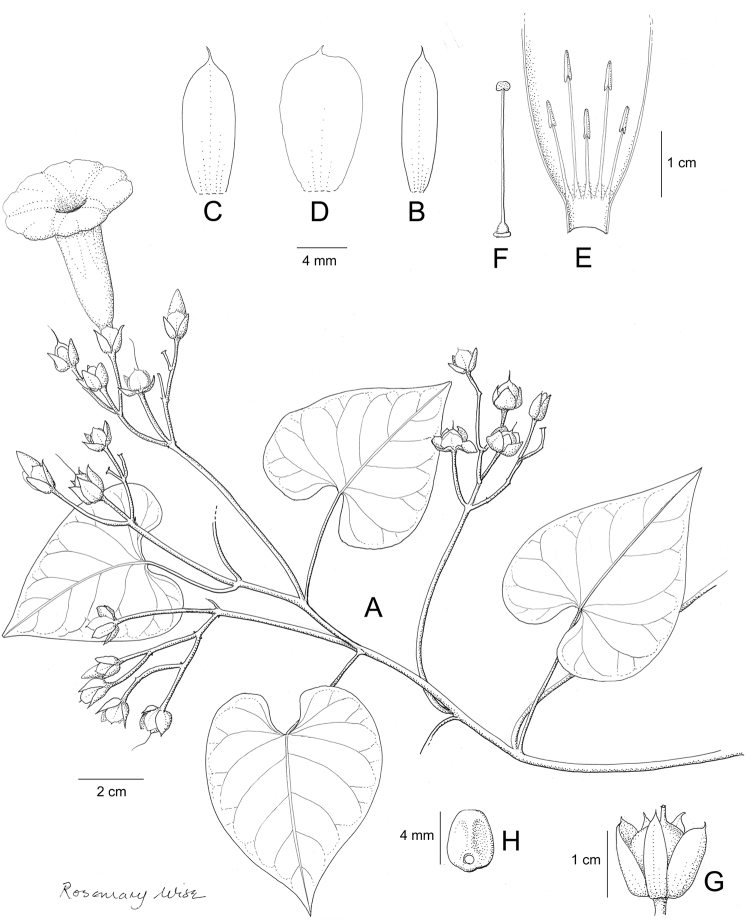
*Ipomoea tiliacea*. **A** habit **B** outer sepal **C** middle sepal **D** inner sepal **E** corolla opened out to show stamens **F** ovary and style **G** capsule **H** seed. Drawn by Rosemary Wise **A–F** from *Curtiss* 1293; **G** from *Stearn* 38; **H** from *Proctor* 18508.

Figure 112; Acevedo-Rodríguez (2005: 180).

#### Distribution.

Secondary forest and disturbed bushland, usually within a few kilometres of the coast. In South American along the Caribbean and Atlantic coasts south to Rio Grande do Sul in southern Brazil. On the Pacific only confirmed from the Choco in Colombia northwards. Widespread and frequent on the Caribbean Islands and on the Caribbean coasts of Central America north to Veracruz but less common on the Pacific side. Reported as naturalised in the Old World but most, probably all, of these records are errors for *Ipomoea littoralis* or *I. batatas*.

**BRAZIL. Amazonas**: Dermini River, *P. Acevedo-Rodríguez et al.* 8166 (NY). **Bahia**: *Blanchet* 1016 (BM); *Glocker* 330 (BM). **Pará**: Breves, Amazon estuary, *E.P. Killip & A.C. Smith* 30211 (NY). **Paraná**: Balneario de Canoas, Pontal de Paraná, *E.L. Siquiera et al.* 525 (MBM); Ilha dos Ihres, São Francisco do Sul, *F. Vieira* 974 (MBM). **Pernambuco**: Tapera, *B. Pickel* 128 (BM). **Rio de Janeiro**: *J.F.Widgren* 331 (S). **Roraima**: *J.A. Ratter et al*. 5900 (E). **Rio Grande do Sul**: *P.P.A. Ferreira* 126 (ICN), fide Ferreira and Miotto (2009: 449). **São Paulo**: Bertioga, 30 km E of Santos, *A. Krapovickas & C. Cristóbal* 33565 (CTES, MBM); Cananéia, *M.G. Caxambu et al.* 4124 (MBM).

**FRENCH GUIANA.***P. Sagot* 371 (BM, S), 372 (BM); *F. Billiet & B. Jadin* 1609 (BM, BR).

**SURINAM.***W.R. Hostman* 330 (BM, OXF); *J. Lanjouw* 1086 (S).

**GUYANA.***Jenman* 4200 (BM); *A.S. Hitchcock* 16664 (S).

**COLOMBIA.** sine data, *Linden* 1591 (BM, OXF). **Magdalena**: *H.H. Smith* 1912 (BM), 1567 (K); *E.P. Killip & A.C. Smith* 20917 (COL); *J. Cuatrecasas* 13354 (COL, US).

**VENEZUELA.***Moritz* 41 (BM). **Delta Amacuro**: *J. Steyermark* 87685 (K). **Monagas**: Paloma, *H.H. Rusby & Squires* 15 (BM, NY).

**PANAMA.***C. Hamilton et al.* 1300 (FTG, MO)

**COSTA RICA.** Vera Blanca de Sarapiquí, *A.F. Skutch* 3672 (K, S); San José, *A. Tonduz* 7089 (K), 8622 (BM); Limon, Cahinta, *P. Wilkin & S.B. Jennings* 117 (BM).

**NICARAGUA.***L.O. Williams* 42321 (BM, F); Jinotega, Mun. Wiwili, *I. Coronado et al.* 3120 (BM, MO).

**HONDURAS.** La Mosquitia, *C. Ashe* 57 (BM); *J. Saunders* 709 (FTG); Ceiba, *T.G. Yuncker* 8561 (BM, K, MO).

**BELIZE.** Hector Creek, Sibun River, *P. H. Gentle* 1409 (K, S); *M.E. Peck* 664 (K); Stann Creek, *W.A. Schipp* 283 (BM, K).

**GUATEMALA.** Esquintla, *J. Donnell Smith* 1999 (K); ibid., 2222 (K)

**MEXICO. Campeche**: *E. & H. de Cabrera* 14469 (IEB). **Chiapas**: Esquintla, *E. Matuda* 2133 (K); San Pedro Nolasco, *C. Jurgensen* 592 (K). **Guerrero**: Atoyac, Galeana, *G.B. Hinton* 10900 (K, S). **Yucatán**: Izamal, *G.F. Gaumer* 915 (BM, K, S).

**BAHAMAS.** Great Bahama, *L. Brace* 3600 (NY).

**CUBA.***C. Wright* 1648 (BM, S); Bayate, *E.L. Ekman* 10122 (NY, S); 6636 (BM, S); *A.H. Curtiss* 249 (HAC); *J. Shafer* s. n. [4/1903] (HAC). **Camaguey**: *J.A Shafer* 1846 (NY). **Granma**: La Anita, *M. López Figueiras* 781 (NY). **La Habana**: Santiago de las Vegas, *H. Van Hermann* 231 (BM). **Pinar del Río**: *N.L. Britton et al.* 9666 (NY). **Villa Clara**: Manicaragua, *F. de la Puente* 5324 (FTG); Camajuani, *F. de la Puente* 5347 (FTG).

**CAYMAN ISLANDS.***D.R. Stoddart* 5057 (BM); *M. Brunt* 1716 (BM); *W. Kings* 299 (BM).

**JAMAICA.***G.R. Proctor* 8308 (BM), 21911 (BM); *A.B. Rendle* 152 (BM); *A.D. Skelding* 3534 (BM); *W. Stearn* 38 (BM, S); *Maxon* 10504 (S).

**HAITI.***E.L. Ekman* H9156 (S); Etang Saumatre, *E.C. Leonard* 3544 (NY).

**DOMINICAN REPUBLIC.** Santo Domingo, *E.L. Ekman* H11152 (NY, S); La Vega, *A.H. Liogier* 24738 (NY); *H.A. Allard* 13192 (S); *P. Fuertes* 425 (E), 1156 (E).

**PUERTO RICO.***R.J. Wagner* 457 (BM); Lajas, *A.H. Liogier* 31128 (NY); San Juan, *F.S. Axelrod* 3396 (NY).

**LESSER ANTILLES. US Virgin Islands**: fide Acevedo-Rodríquez (2005). **Netherlands Antilles**: St Eustatius: *B.M. Boom et al.* 11202 (NY). St Marten: *I. Boldingh* 2913 (NY). St Kitts: *G.R. Proctor* 18508 (BM). **Antigua**: *H.E. Box* 1049 (BM), 1362 (BM). **Montserrat**: *G.R. Proctor* 19015 (BM). **Guadeloupe**: *R.P. Quentin* 631 (P); Marie Galante, *G.R. Proctor* 20265 (BM). **Dominica**: *C. Whitefoord* 3961 (BM), 43411 (BM). **Martinique**: *W. Hahn* 80 (BM); *C. Sastre* 7691 (P). **St Lucia**: *G.R. Proctor* 18007 (BM). **St Vincent**: *H.H. & G.W. Smith* 1293 (BM); Bequia fide Powell (1979). **Grenada**: *G.R. Proctor* 17155 (BM); *G.C. Druce* s.n. (OXF). **Barbados**: fide Gooding et al. (1965).

**TRINIDAD.***A. Fendler* 585 (BM). **Tobago**: *Clement & Ryves* 93/230 (BM); *W.E. Broadway* 4395 (S).

#### Typification.

Tropicos (www.tropicos.org) states that Nelson (1997: 393) designated Sessé and Moçiño 5048 as lectotype of *Ipomoea biflora* but this is doubtful as he merely cited it as the type and it may in any case be the de facto holotype in the absence of other possible types.

#### Notes.

*Ipomoea tiliacea* is quite variable in sepal and to a lesser extent corolla size. It is a perennial, which is nearly always completely glabrous and with unlobed leaves. In the neotropics it is only likely to be confused with rare forms of *Ipomoea batatas* combining glabrous sepals with entire leaves. From these it is best distinguished by the lax, clearly cymose inflorescence. Records of *I. tiliacea* from the Old World are all, or mostly, errors for the superficially similar *I. littoralis*, which is best separated by its rounded or obtuse, somewhat succulent leaves and 1–3-flowered cymes. The range of the two species appears not to overlap and molecular studies support their distinctiveness.

Records from Peru (McPherson 1993) are probably errors for *Ipomoea batatas* and require confirmation.

### 222. Ipomoea littoralis

Taxon classificationPlantaeSolanalesConvolvulaceae

Blume, Bijdr. Fl. Ned. Ind. 13: 713. 1825. (Blume 1825–26: 713)

Ipomoea batatas var. littoralis (Blume) Nishiyama, Bot. Mag. 84: 385. 1971 (Nishiyama 1971: 385).
Convolvulus denticulatus Lam., Encycl. 3(2): 540. 1789 [pub. 1792]. (Lamarck 1792: 540). Type. “Isles Mahé, Sechelles et des Trois Frères”. *Commerson*s.n. (holotype MPU009875, isotype P-JUSS-6810).
Ipomoea denticulata (Lam.) Choisy, Mém. Soc. Phys. Genève 6: 467 [85]. 1834. (Choisy 1834: 467 [85]), comb. illeg., non Ipomoea denticulata R. Br. (1810).
Ipomoea nicobarica Kurz, J. Asiat. Soc. Bengal, 2 (Nat. Hist.) 45(3): 141. 1876. (Kurz 1876: 141). Type. INDIA. Nicobar Islands, Kamorta, *S. Kurz*s.n. (lectotype K001081746, designated here; specimen with Kurz’s annotation and type locality on label).
Ipomoea choisiana Wight ex Safford, Contr. U.S. Natl. Herb. 9: 298. 1905. (Safford 1905: 298). Type. Based on Convolvulus denticulatus Desr.
Ipomoea gracilis sensu auct. mult., non R. Brown (1810).

#### Type.

INDONESIA. Java, *Blume* 1710 (lectotype L0004194, designated here; isolectotypes L, P).

#### Description.

Perennial trailing or (less commonly) twining herb, stems often rooting at the nodes, glabrous or with a few hairs. Leaves petiolate, 1–7 × 2–7 cm, somewhat coriaceous, usually ovate, cordate with rounded auricles, less commonly deltoid or sagittate with acute auricles, entire but sometimes angled or lobed, apex subacute, obtuse, rounded, or retuse, mucronulate, both surfaces glabrous, veins prominent abaxially; petioles 2.5–5 cm. Inflorescence of few-flowered axillary cymes, often reduced to a single flower; peduncles sometimes paired in the leaf axils, 1–5 cm, usually much shorter than pedicels, glabrous; bracteoles 1.5 mm long, filiform, caducous; pedicels 10–25 mm, glabrous; sepals unequal, glabrous, outer 6–10 × 3–4 mm, oblong-elliptic, acute or obtuse, mucronate, inner 8–12 × 7–10 mm, elliptic to suborbicular, mucronate, the margins thin and membranous; corolla 3–5 cm long, funnel-shaped, glabrous, pale pink with a dark throat; stamens short. Capsules globose or depressed globose, 6–7 mm long, glabrous; seeds 3.5–4 mm, glabrous.

#### Illustration.

**Figure 113.**
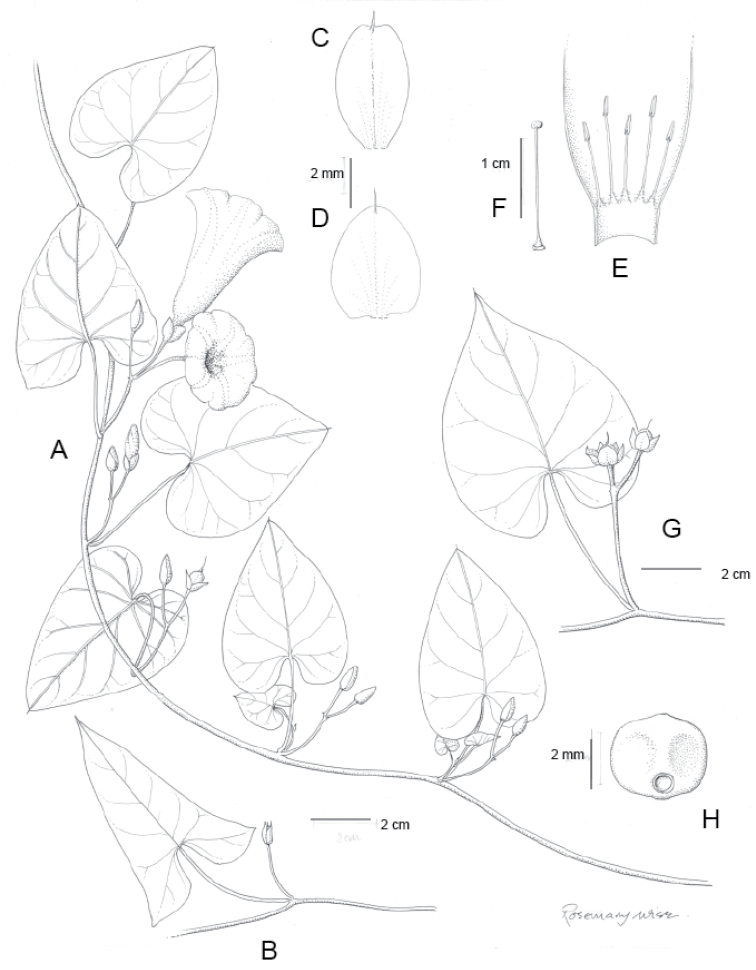
*Ipomoea littoralis*. **A** habit **B** variant leaf shape **C** outer sepal **D** inner sepal **E** corolla opened out to show stamens **F** ovary and style **G** fruiting inflorescence **H** seed. Drawn by Rosemary Wise **A** from *Lister* s.n.; **B** from *Setchell* 461; **C–H** from *Brass* 13946.

Figures 110C, 113; Bosser and Heine (2000: 47); Deroin (2001: 209).

#### Distribution.

Widely distributed on tropical sea shores through most of the Pacific and Indian oceans (Austin 1991c) but absent from the American and African continents, although present in Madagascar. It is especially characteristic of oceanic islands, where it is often found growing on the seashore but sometimes inland in scrub near the sea. In the Americas it is only known from the Hawaii archipelago.

**HAWAII.***Hillebrand* 393 (K); s.n. (BM). Apparently rare fide A. Whistler (pers. com.).

#### Note.

The leaves of this species are very variable in shape but are characteristically succulent, the apex is usually obtuse to rounded and the base cordate with a very narrow sinus so the auricles almost touch each other. The cymes consist of only 1–3 flowers unlike the somewhat similar *Ipomoea tiliacea*. The mucros on the sepals are caducous like the bracteoles.

It is reported as being used as a vegetable in Polynesia (Austin 1991c).

### 223. Ipomoea lactifera

Taxon classificationPlantaeSolanalesConvolvulaceae

J.R.I. Wood & Scotland, Kew Bull. 70 (31): 91. 2015. (Wood et al. 2015: 91)

#### Type.

BOLIVIA. Santa Cruz, Prov. Ichilo, 2–20 km from Buenavista along road to El Huaytú, *J.R.I. Wood & D. Soto* 27954 (holotype USZ, isotypes OXF, K, LPB).

#### Description.

Perennial twining herb of unknown height, latex white, stem glabrous. Leaves petiolate, 5–9 × 3.3–7 cm, ovate, base cordate and very broadly cuneate onto the petioles, auricles rounded, apex acuminate to a shortly mucronate apex, margin entire, glabrous except for an area of puberulence on veins and margin at base around point of insertion of petiole; petiole 2.2–6.8 cm, glabrous but thinly puberulent upwards. Inflorescence of long pedunculate, many-flowered, axillary cymes; peduncles 5–10 cm, glabrous, secondary peduncles 1.5–3 cm; bracteoles 1 × 1 mm, suborbicular, early caducous leaving a prominent basal scar; pedicels 8–14 mm, glabrous to slightly farinose; sepals glabrous or somewhat farinose, unequal, somewhat papery in texture, the margins slightly scarious but not conspicuously pale, outer 5–6.5 × 2.5–3 mm, oblong-obovate, rounded, the central vein prominent, slightly raised and terminating in a mucro, inner 7–8.5 × 5 mm, elliptic, rounded, minutely mucronulate with the mucro deciduous; corolla 3–4 cm long, broadly funnel-shaped and gradually widened from base, limb 2–2.5 cm diam., white or very pale pink with darker centre, glabrous; ovary glabrous. Capsules and seeds not seen.

#### Illustration.

Figure 114.

**Figure 114.**
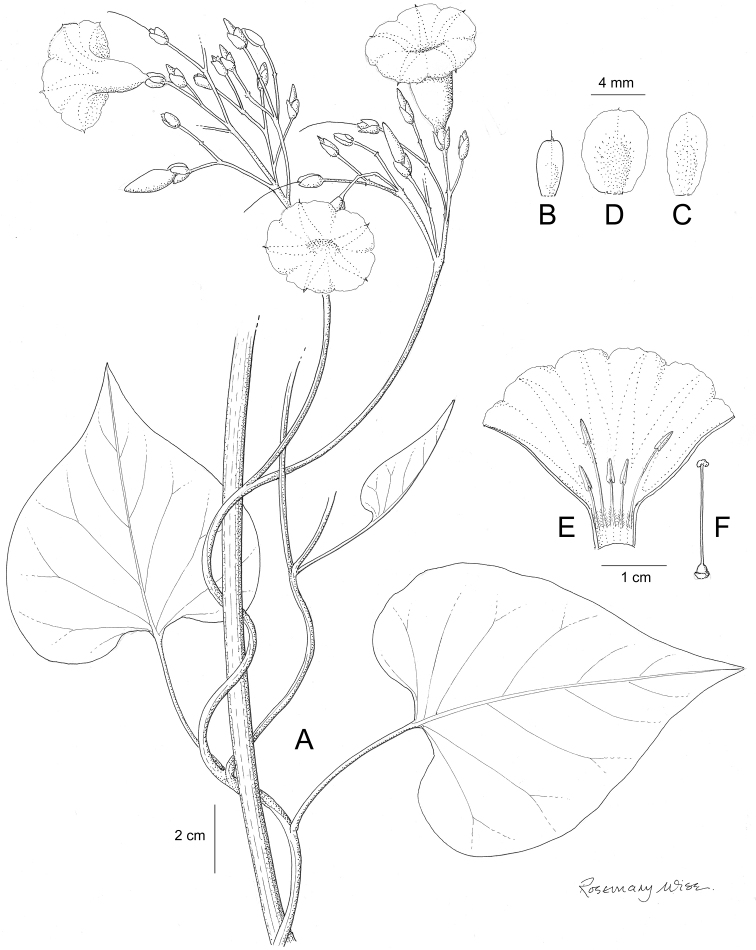
*Ipomoea lactifera*. **A** habit **B** outer sepal **C** middle sepal **D** inner sepal **E** corolla opened out to show stamens **F** ovary and style. Drawn by Rosemary Wise from *Solomon* 14004.

#### Distribution.

Endemic to humid forest or forest relics in the Andean foothills of Bolivia and Ecuador between 200 and 1000 m.

**BOLIVIA. Beni**: Ballivián, upstream from Rurrenabague, *D.C. Daly et al.* 6639 (FTG); Est. Biologica del Beni, *G. Caity* 149 (K, LPB, OXF); Cercado, *F. de la Puente* 3593 (CIP). **Cochabamba**: Chapare, El Choclotal, *J.R.I. Wood* 23411 (K, LPB, USZ); P.N. Carrasco, Yanamayo, *M. Zarate* 6455 (BOLV, LPB). **Santa Cruz**: Ichilo, P.N. Amboró, opposite El Huaytú, *J.C. Solomon* 14004 (K, LPB, MO).

**ECUADOR. Morona-Santiago**: Centro Shuar Yukatais, Chacras, *B. Bennett & P. Gómez A* 3783 (OXF, ?NY, QCNE).

#### Note.

The discovery of *Ipomoea lactifera* is of exceptional interest as it is an additional crop wild relative of the sweet potato. Apart from *I. batatas* itself it is the only perennial species of this group growing in Bolivia and the first with an exclusively Andean distribution. From other species in this clade it can be distinguished by its large white or pale pink corolla and relatively broad obovate to elliptic sepals.

### 224. Ipomoea lacunosa

Taxon classificationPlantaeSolanalesConvolvulaceae

L. Sp. Pl. 1: 161. 1753. (Linnaeus 1753: 161)

Ipomoea triloba forma lacunosa (L.) Nishyama, Bot.Mag. Tokyo 84: 385. 1971. (Nishiyama 1971: 385).
Convolvulus ciliolatus Michx., Fl. Bor.-Amer. 1: 137. 1803. (Michaux 1803: 183). Type. UNITED STATES. Tennessee, Nashville, collector and whereabouts unknown.
Ipomoea ciliolata (Michx.) Pers., Syn. Pl. 1: 180. 1805. (Persoon 1805: 180).
Ipomoea ciliosa Pursh, Fl. Amer. Sept. 1: 146. 1813. (Pursh 1813: 146). Type. Based on Convolvulus ciliolatus Michx.
Ipomoea verrucipes Ten. ex C.A. Mey., Index Seminum (St Petersburg)1843: 76. 1843. (Meyer 1843: 76). Type. Not cited, but reported by Choisy to have been grown from seeds from Mexico.

#### Type.

UNITED STATES. Carolina, lectotype, Dillenius, Hort. Eltham. 1: 103. t. 87, f. 102 [103] designated by Staples in Staples and Jarvis in Taxon 55: 1022. 2006.

#### Description.

Slender twining annual herb, stems glabrous to thinly pilose. Leaves petiolate, 3–8 × 2–7 cm. usually ovate, acuminate and base cordate with rounded auricles but sometimes 3- or 5-lobed with shortly acuminate lateral lobes, subglabrous or more commonly with scattered long hairs; petioles 1–9 cm. Inflorescence of shortly pedunculate 1–3-flowered cymes; peduncles 0.6–6.5 cm, very variable in length, usually pubescent; bracteoles 2–4 mm long, filiform; pedicels 2–8 mm; sepals subequal, 10–14 × 2–4 mm, somewhat accrescent in fruit, narrowly to broadly ovate, acuminate to a long fine aristate tip, ciliate on margins and often also pilose; corolla 1.8–2 cm long, funnel-shaped, white or pale pink, glabrous, limb c. 1 cm diam., shortly lobed, the lobes mucronate. Capsules subglobose, 10–15 mm long and wide, pilose; seeds 5–6 mm long, dark brown, ellipsoid, glabrous.

#### Illustration.

**Figure 115.**
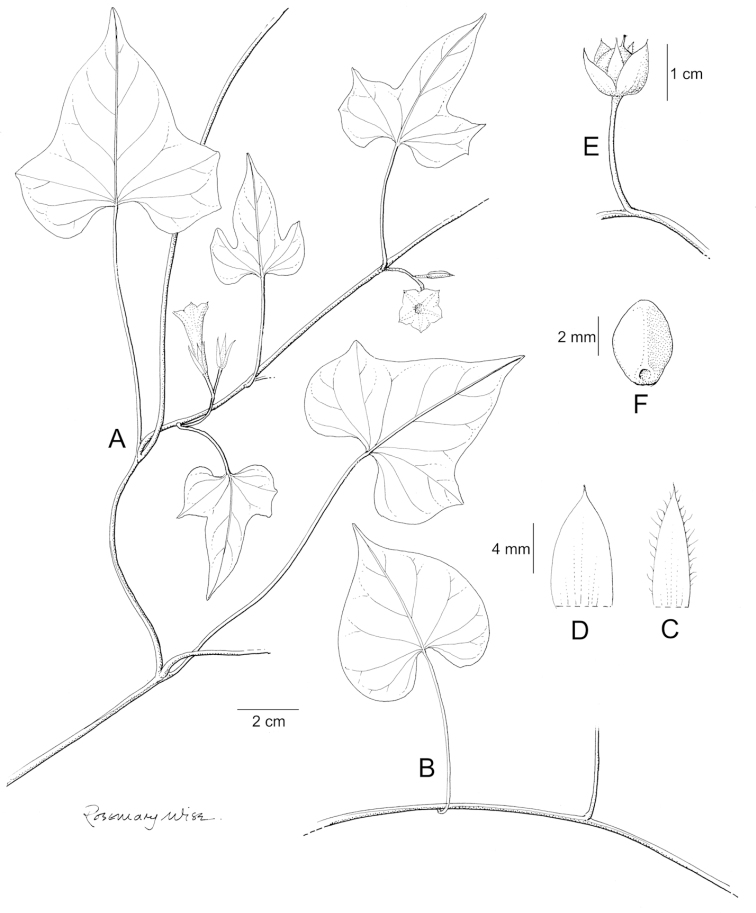
*Ipomoea lacunosa*. **A** habit **B** simple leaf **C** outer sepal **D** inner sepal **E** capsule with calyx **F** seed. Drawn by Rosemary Wise **A** from *McCarthy* s.n.; **B** from *Mackenzie* s.n.; **C–F** from *Harper* 520.

Figures 110B, 115; Haddock et al. (2015: 234).

#### Distribution.

A weedy species of the south eastern United States extending north to Pennsylvania, Illinois and Indiana and west to Texas and Missouri. Perhaps occurring as an ephemeral weed outside the United States, for example in Jamaica (Adams 1972), but all records in the New World from outside the eastern United States require confirmation. Outside the Americas it is reported as an adventive in Europe (Sell and Murrell 2009: 348) and East Asia (Fang and Staples 1997: 301).

**UNITED STATES. Alabama**: *S.B. Buckley* s.n. (OXF); *C.T. Bryson & K. Reddy* 20432 (ARIZ). **Arkansas**: *R.A. Thompson et al.* 1004 (K); *T. Nuttall* s.n. (OXF). **Delaware**: *W.D. Longbottom* 16023 (NY). **Florida**: Jacksonville, *Drummond* (K); *D.H. Williams* 2635 (SEL). **Georgia**: *W.S.B. Jones et al.* 1554 (BM); *R.M. Harper* 520 (BM, E, K); *T. Nuttall* (OXF). **Illinois**: *F.E. McDonald* s.n. [9/1913] (S). **Indiana**: *R.F. Schulenberg* 75-1363 (MOR). **Kansas**: *R.L. McGregor* 266 (S). **Kentucky**: *C.W. Short* s.n. (K). **Louisiana**: Monroe, *Dale Thomas* 21174 (BM); *E.J. Palmer* 8823 (K). **Maryland & D.C.**: *E.S. Steele* s.n. (E). **Missouri**: *J. Steyermark* 76626 (BM); *R.T. Ovrebo & C.M. Sladewski* 1003 (K). **Mississippi**: *C. T. Bryson & K. Reddy* 20353 (ARIZ). **North Carolina**: *Biltmore* 2575a (S); *J.H. Horton* 321 (BM). **Ohio**: *J.F. James* 2158 (BM); 2163 (K). **Pennsylvania**: *J. Ebert* s.n. [8/10/2006] (MOAR). **South Carolina**: *T. Nuttall* s.n. (OXF); *R.D. Porcher* 2132 (CLEMS). **Tennessee**: *A. Ruth* s.n. [9/1895] (S); *Rugel* s.n. (OXF). **Texas**: *D.S. & H.B. Correll* 32046 (LL). **Virginia**: Bedford Co., *A.H. Curtiss* s.n. (E); *K.K. Mackenzie* 1778 (E); *E.K. Balls* 7792 p.p. (BM). **West Virginia**: *R. Hall et al.* 52 (MUHW).

#### Note.

Distinguished by the small white corolla and relatively large capsule (> 10 mm wide, not less than 9 mm).

### 225. Ipomoea leucantha

Taxon classificationPlantaeSolanalesConvolvulaceae

Jacq., Icon. Pl. Rar. 2: t. 318. 1788. (Jacquin 1786–1793: t. 318)

Ipomoea batatas var. leucantha (Jacq.) Nishiyama, Bot. Mag. Tokyo 84: 385. 1971. (Nishiyama 1971: 385).
Euryloma leucantha (Jacq.) Raf., Fl. Tellur. 4: 75. 1836 [pub. 1838]. (Rafinesque 1838a: 75)
Quamoclit leucantha (Jacq.) G. Don, Gen. Hist. 4: 258. 1838. (Don 1838: 258).
Convolvulus dentatus Blanco, Fl. Filip., ed. 1: 89. 1837. Type. Plate 31 (of Ipomoea commutata) in Fl. Filip., ed. 3. 1877, lectotype designated by Austin 1978b: 121.
Ipomoea blancoi Choisy in A.P. de Candolle, Prodr. 9: 349. 1845. (Choisy1845: 349). Type. Based on Convolvulus dentatus Blanco
?Ipomoea hirta M. Martens & Galeotti, Bull. Acad. Bruxelles 12 (2): 264. 1845. (Martens and Galeotti 1845: 264). Type. MEXICO. Oaxaca, *H. Galeotti* 1374 (BR000006972639, BR0000006973315 syntypes).
Ipomoea trifida var. ymalensis House, Ann. New York Acad. Sci.18: 254. 1908. (House 1908b: 254). Type. MEXICO. [Sinaloa], Imala, *E. Palmer* 1746 (holotype NY, not found, isotypes F, S, US).
Ipomoea lacunosa forma purpurea Fernald, Rhodora 40: 454. 1938. (Fernald 1938: 454). Type. UNITED STATES. Virginia, *Fernald & Long* 7580 (lectotype GH, ? designated by Austin 1978b: 121).
Ipomoea trichocarpa forma albiflora Ahles, J. Elisha Mitchell Soc. 75: 129. 1959. Type. UNITED STATES. South Carolina, Colleton Co. *H.E. Ahles* 17956 (holotype UNC).

#### Type.

Jacquin, Icon. Pl. Rar. 2: t. 318, 1788, lectotype designated by Austin (1978b: 120).

#### Description.

Twining annual herb similar to *I. cordatotriloba* and other annual species of the Batatas Clade. Leaves petiolate, 3–5 × 1.5–4 cm, ovate, entire or shallowly 3-lobed, cordate, the auricles sometimes with a large tooth, apex shortly acuminate, abaxially paler, glabrous; petioles 2.5–3.5 cm. Inflorescence of dense cymes comprising about 5 clustered flowers; peduncles 8–12 mm, glabrous; pedicels 4–7 mm; sepals subequal, 10–14 mm long, lanceolate, acuminate and apiculate, pilose or glabrous; corolla 1.5–2 (–3.5) cm long. Capsules subglobose, 6–8 × 5–6 mm diam., pilose; seeds c. 3.5 × 2 mm long, glabrous.

#### Distribution.

Occurs sporadically, principally in the eastern United States and in Central America south to Ecuador and Brazil. It is also reported from the Old World, principally in Asia, but these records are of uncertain status and have not often been accepted in recent publications on Asian *Ipomoea*. The following records should be treated as provisional.

**BRAZIL. Bahia**: *R.M. Harley et al.* 21816 (K).

**ECUADOR. Guayas**: Guayaquil, *E. Asplund* 15643 (S), 15652 (S). **Napo**: Yasuni, Rio Tiputini, *R. Burnham* 1439 (MICH, QCA). **Sucumbios**: Gonzalo Pizarro, Rio Aguarico, *A. P. Yañez et al.* 1067 (QCA).

**COLOMBIA.** Sine loc., *E. André* 1833 (K); 1839 (K).

**COSTA RICA.** Nicoya, *A.H. Tonduz* 13680 (BM); *U. Chavarria & F. Rizo-Patrón* 2244 (MA).

**MEXICO. Jalisco**: *C. & J.G. Cortes* 608 (MEXU). **Michoacán**: Morelia, *J.M. Escobedo* 2181 (IEB, MEXU); San Antonio Labrador, *J.C. Soto Nuñez* 10914 (MEXU). *Querétaro*: Jalpan, *E. Carranza & E. Pérez* 5209 (IEB, MEXU). **Sonora**: Mori, Yaqui country, *H.S. Gentry* 4743 (MEXU); San Luis, Río Colorado, *R. Felger* 85-1032 (MEXU). **Tamaulipas**: *M.E. González* 28 (MEXU).

**UNITED STATES. Florida**: *A.H. Curtiss* 5575 (K). **Mississippi**: *T.C. Lockley* s.n. [18/8/1997) (FTG). **Missouri**: Boonville, *G. Yatskievych* 96-78 (MO).

**JAMAICA.***G.R. Proctor* 16096 (BM); *R.D. Henry & C.D. Adams* 12893 (BM).

#### Note.

A poorly understood entity considered by Austin (1978b passim) to have arisen as a natural hybrid between *I. lacunosa* and *I. cordatotriloba* in the United States and occurring sporadically elsewhere as a weed or casual, being spread as a contaminant of rice seeds. Molecular sequencing shows this species to be polyphyletic (Muñoz-Rodríguez et al. 2018) and further studies are needed before the characteristics and distribution of this taxon can be confirmed.

### 226. Ipomoea cordatotriloba

Taxon classificationPlantaeSolanalesConvolvulaceae

Dennst., Nomencl. Bot. 1: 246. 1810. (Dennstedt 1810: 246)

Convolvulus carolinus L., Sp. Pl. 1: 154. 1753. (Linnaeus, 1753: 154), non Ipomoea carolina L. (1753). Type. Icon. in Dillenius, Hortus Elthamensis 1: 100. t. 84 f. 98 (1732), designated by Staples in Staples and Jarvis (2006: 1020).
Ipomoea trichocarpa Elliot, Sketch Bot. S.C. 7 Ga. 1: 258. 1817. (Elliot 1817: 258). Type. Based on Convolvulus carolinus L.
Ipomoea triloba forma trichocarpa (Elliot) Nishiyama, Bot. Mag. Tokyo 84: 385. 1971. (Nishiyama 1971: 385).
Ipomoea commutata Roem. & Schult., Syst. Veg. 4: 228. 1819. (Roemer and Schultes 1819: 228), nom. illeg. superfl. Type. Based on Convolvulus carolinus L.
Convolvulus scrobiculatus Lindl., Bot. Reg. 13; t 1076, 1827. (Lindley 1827b: t. 1076). Type. A cultivated plant of American origin (lectotype t. 1076 in Botanical Register (Lindley1827b), designated here).
Ipomoea scrobiculata (Lindl.) Sweet, Hort. Brit., ed. 2: 372. 1830. (Sweet 1830: 372).
Ipomoea trifida var. berlandieri A. Gray, Syn. Fl. N. Amer. 2: 212. 1878. (Gray 1878: 212). Type. UNITED STATES. Texas, J. Berlandier 546 [1931] (holotype GH0054470, isotypes BM, K, MO, NY, PH).
Ipomoea trifida var. torreyana A. Gray, Syn. Fl. N. Amer. 2: 212. 1878. Type. UNITED STATES. Texas, *C. Wright*s.n. (lectotype GH00054469, designated by Austin (1978b: 126).
Ipomoea trichocarpa var. torreyana (A. Gray) Shinners, Field & Lab.21: 164. 1953. (Shinners 1953: 164).
Ipomoea cordatotriloba var. torreyana (A. Gray) D.F.Austin, Taxon 37: 185. 1988. (Austin 1988: 185).
Ipomoea trichocarpa forma pubescens Ahles, J. Elisha Mitchell Soc. 75: 129. 1959. (Ahles 1959: 129). Type. UNITED STATES. South Carolina, Calhoun Co, *H.E. Ahles* 35245 (holotype UNC).

#### Type.

Based on *Convolvulus carolinus* L.

#### Description.

Slender twining (occasionally trailing) annual herb, stems to 3 m, glabrous, thinly pilose with long white hairs or densely pubescent. Leaves petiolate, 2.5–8 × 1.5–6 cm, 3– 5-lobed, the central lobe narrowed at base (very rarely unlobed), narrowly cordate with rounded, entire or dentate auricles, apex shortly acuminate, mucronate, glabrous or thinly pilose on veins and margins or pubescent; petioles 0.5–5 cm, muricate. Inflorescence of axillary, pedunculate, umbelliform cymes, usually with 1–5(–9) flowers, and more lax than in *Ipomoea batatas*; peduncles 2–9 cm; bracteoles 5–7 mm, filiform, pilose, relatively persistent; pedicels 4–9 mm; sepals subequal, usually ciliate with stiff spreading hairs, occasionally glabrous, outer sepals 8–11 mm, ovate, gradually narrowed to an outwardly curved fine point, the central vein usually distinct, inner sepals 10–12 mm, obovate, abruptly or gradually narrowed to a mucronate apex, less hairy; corolla (2.5–)3.5–4.5 cm long, gradually widened from base, pink with a dark centre, glabrous, limb c. 2.5 cm diam., unlobed. Capsules subglobose, 7–8 mm, pilose; seeds brown, hemispherical, 3.5 mm long, shortly pubescent on the angles.

#### Illustration.

Diggs et al. (1999: 557)

#### Distribution.

This species is apparently restricted to the United States and Mexico. In the United States it is more strictly southern than *I. lacunosa*. Records from elsewhere, for example from Venezuela (Hokche et al. 2008) require confirmation. It is a lowland species not usually found above 1000 m.

**MEXICO. Chihuahua**: *C.G. Pringle* 781 (K, S). **Tamaulipas**: *G.S. Hinton* 20526 (GBH)

**UNITED STATES. Alabama**: *C.T. Bryson* 20420 (MMNS). **Arkansas**: *Leavenworth* s.n. (K). **Florida**: St John’s River, *A.H. Curtiss* 2161 (K), 5280 (E); *Rügel* 506 (BM); Gainesville, *W. Judd & T. Lucansky* 2751 (BM). **Georgia**: *R. Carter* 9217, Louisiana: *Tracy & Lloyd* 125 (ARIZ); *P.E. Hyatt* 11166 (LSU). **Louisiana**: *Tracy & Lloyd* 125 (BM). **Mississippi**: *C. T. Bryson & K. Reddy* 20350 (ARIZ), 20355 (ARIZ). **New Mexico**: *J. Skehan* 80 (RM). **North Carolina**: Wilmington, *Bradley & Sears* 3575(K, S). **South Carolina**: *Drummond* s.n. (K). **Texas**: *Lindheimer* 1033 (BM, K, S); *B.F. Bush* 275 (K), 1405 (K); *Drummond* 215 (K); *C.T. Bryson* 22361 (VSC).

#### Note.

As interpreted here this is an entirely Northern Hemisphere species that is almost restricted to the United States, where it is a common in the south east. The leaves are nearly always 3–5-lobed and the corolla deep pink with a dark centre. Plants named var. torreyana are a glabrous form of this species.

### 227. Ipomoea australis

Taxon classificationPlantaeSolanalesConvolvulaceae

(O’Donell) J.R.I. Wood & P. Muñoz, comb. & stat. nov.

urn:lsid:ipni.org:names:77208072-1

Ipomoea trichocarpa var. australis O’Donell, Bol. Bot. Soc. Argent. 4: 260. 1953. (O’Donell 1953b: 260). Type. ARGENTINA. Tucumán, Lillo12909 (holotype LIL n.v., isotype NY00319232).
Ipomoea cordatotriloba var. australis (A. Gray) D.F. Austin, Taxon 37: 185. 1988. (Austin 1988: 185).

#### Type.

Based on Ipomoea trichocarpa var. australis O’Donell

#### Description.

Slender twining (occasionally trailing) annual herb, stems to 3 m, glabrous, thinly pilose with long white hairs or densely pubescent. Leaves petiolate, 2.5–8 × 1.5–6 cm, entire, ovate-deltoid or (very rarely) shallowly 3-lobed, narrowly cordate with rounded, entire or dentate auricles, apex shortly acuminate, mucronate, glabrous or thinly pilose on veins and margins or pubescent; petioles 0.5–5 cm, smooth. Inflorescence of axillary, pedunculate, umbelliform cymes, usually with 1–5(–9) flowers; peduncles 2–9 cm; bracteoles 5–7 mm, filiform, pilose, relatively persistent; pedicels 4–9 mm; sepals subequal, usually ciliate with stiff spreading hairs, occasionally glabrous, outer sepals 8–11 mm, ovate, gradually narrowed to an outwardly curved fine point, the central vein usually distinct, inner sepals 10–12 mm, obovate, abruptly or gradually narrowed to a mucronate apex; corolla (2.5–) 3.5–4.5 cm long, gradually widened from base, pink with a dark centre, glabrous, limb c. 2.5 cm diam., unlobed. Capsules subglobose, 7–8 mm, pilose; seeds brown, hemispherical, 3.5 mm long, glabrous.

#### Illustration.

Figures 5C, 116.

**Figure 116.**
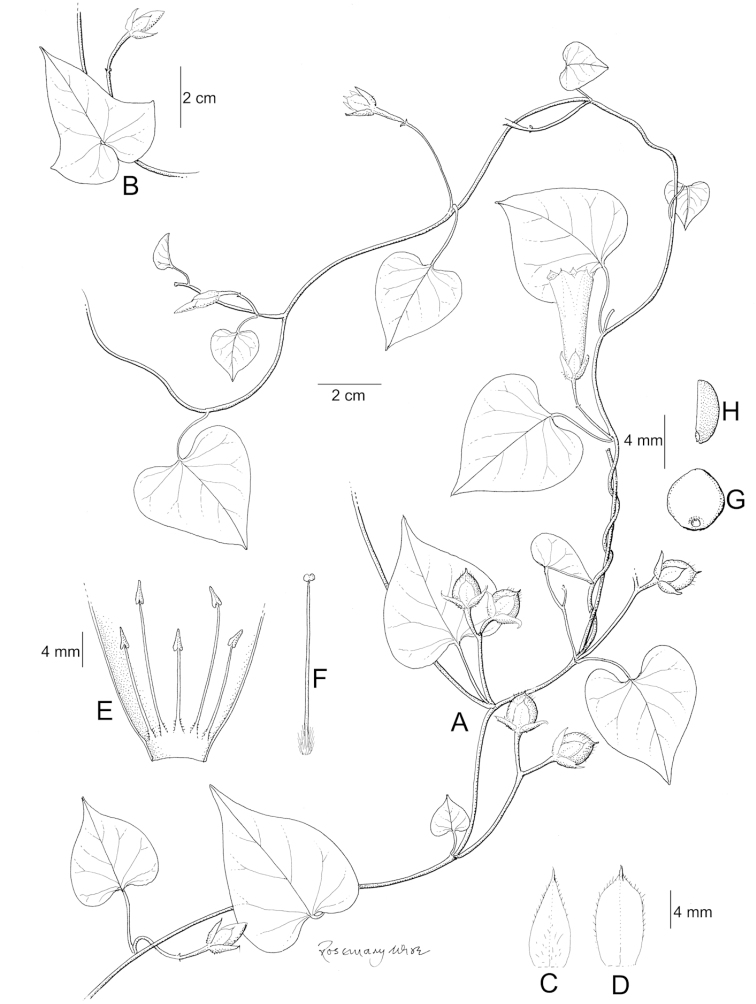
*Ipomoea australis*. **A** habit **B** leaf and peduncle **C** outer sepal **D** inner sepal **E** corolla opened out to show stamens **F** ovary and style **G** seed, front view **H** seed, side view. Drawn by Rosemary Wise **A, C–H** from *Wood & Soto* 27929; **B** from *Pedersen* 2593.

#### Distribution.

Restricted to the Southern hemisphere where it is found in Argentina, Paraguay, Bolivia and Brazil, in the last of which it is apparently rare. Records from elsewhere require confirmation. It is a lowland species not usually found above 1000 m. See also O’Donell 1953b for numerous citations under Ipomoea trichocarpa var. australis.

**ARGENTINA. Catamarca**: *Brizuela* 124 (LIL). **Chaco**: *A.G. Schulz* 15973 (CTES). **Corrientes**: *T.M. Pedersen* 2593 (C, E, S); *M. M. Arbo* 718 (CTES). **Formosa**: *I. Morel* 7831 (LIL). **Jujuy**: *A. Schinini and Vanni* 22343 (CTES). **La Rioja**: *Biurron* 3309 (CTES). **Salta**: Oran, *A. Krapovickas & G. Seijo* 47683 (CTES), *A. Krapovickas & A. Schinini* 30456 (CTES). **Tucumán**: *S. Venturi* 4216 (LIL, LP, US).

**PARAGUAY. Amambay**: *A. Krapovickas & A. Schinini* 32661 (CTES). **Caaguazú**: *A. Krapovickas & C. Cristóbal* 44869 (CTES). **Caazapá**: Abai, Com. Aché de Ypetimi, *P. da Motta* 98 (FCQ). **Central**: Asunción, *I. Basualdo* 98 (FCQ); Patino, *G.W. Teague* 571 (BM); *T. Morong* 103 (E); Ñemby, *L. Pérez et al.* 13 (PY). **Concepción**: Loreto, *M. Dematteis et al.* 3149 (CTES, FCQ). **Cordillera**: Caacupé, *E. Lurvey* 274 (CTES, PY); Río Salado hacia Limpio, *J.R.I. Wood et al.* 28142 (FCQ). **Guairá**: Cordillera de Ybytyruzú, *E. Zardini et al.* 4237 (FCQ); Col. Independencia, *J.R.I. Wood et al.* 28155 (FCQ). **Ñeembucú**: Estancia Redondo, *R. García et al.* 01 (FCQ). **Paraguarí**: Paraguarí town, *J.R.I. Wood et al.* 28151 (FCQ). **Presidente Hayes**: Est. Fortín Salazar, Laguna Carpa Cue, *C. Vogt* 372 (FCQ); *M.M. Arbo et al.* 1600 (CTES). **San Pedro**: *N. Soria* 5416 (FCQ).

**BRAZIL. Mato Grosso do Sul**: Fazenda Nhumirim, Corumba, *A. Pott et al.* 2929 (MBM).

**BOLIVIA. Beni**: Cercado, Casarave, *M.T. Martinez & M. Adler* 66 (K, LPB, USZ). **Chuquisaca**: *E. Saravia* 10851 (CTES). **La Paz**: Sud Yungas, bajada de Chulumani a Asunta, *J.R.I. Wood et al.* 20610 (BOLV, K, LPB, USZ). **Santa Cruz**: Germán Busch, 28 km al sur del Rincón del Tigre, *J.R.I. Wood et al.* 24581 (K, LPB, UB, USZ); Chiquitos, Valle de Tucuvaca, *J.R.I. Wood et al*. 23473 (K, LPB, UB, USZ); Cordillera, carretera entre Boyuibe y Camiri, *J.R.I. Wood et al.* 27629 (OXF, LPB, USZ); Florida, Los Negros *J.R.I. Wood et al.* 22769 (K, LPB, USZ); Ibañez, *M. Nee* 49030 (NY, USZ); Ichilo, Buenavista, *J. Steinbach* 7042 (GH, K, S). Ñuflo de Chávez, San Antonio de Lomerío, *J.R.I. Wood* 27756 (K. LPB, USZ). Sara, La Bélgica *J.R.I. Wood* 22120 (K, LPB). Velasco, Carmen Ruiz, *J.R.I. Wood & D. Soto* 27413 (K, LPB, USZ). **Tarija**: Arce, *M. Coro* 1174 (LIL). Gran Chaco, 19 km N. of Camatindi, *M. Dematteis et al.* 1949 (CTES, GH).

#### Notes.

*Ipomoea australis* usually has entire leaves (rarely 3-lobed), but never with the lobe contracted at base, pedicels almost always smooth, seeds completely glabrous. Our molecular studies give support for the recognition of *I. australis* as a distinct species.

*Wood et al. 27611* (K, LPB, USZ) from Villamontes, Gran Chaco, appears to be *I. australis* but the ovary and capsules are glabrous.

### 228. Ipomoea grandifolia

Taxon classificationPlantaeSolanalesConvolvulaceae

(Dammer) O’Donell, Arq. Mus. Paranaense 9: 222. 1952. (O’Donell 1952: 222)

Jacquemontia grandifolia Dammer, Bot. Jahrb. Syst. 23 (Beibl. 57): 41. 1897. (Dammer 1897: 41). Type. BRAZIL. Rio de Janiero, *A.F.M. Glaziou* 11257 (holotype B†, isotypes C, K).
Ipomoea setifera var. orbicularis Chodat & Hassl., Bull. Herb. Boiss., ser. 2, 5: 687. 1905. (Chodat and Hassler 1905: 687). Type. PARAGUAY. [Concepción], Río Apa, *E. Hassler* 7961a (holotype G00175007).
Ipomoea coccinea var. luteola Arechav. Anales Mus. Nac. Montevideo 4: 191. 1911. (Arechavaleta y Balpardo 1911: 191). Type. URUGUAY. Not specified. (?MVM, n.v.).

#### Diagnosis.

This is distinguished from *Ipomoea australis* by the shorter corolla (1.5–2.5 cm long), which is uniformly pink. The sepals are usually narrowly (not broadly) ovate, but this character is not constant. It is essentially a large-flowered form of *I. triloba* and has the appearance of being an intermediate with *I. australis*.

#### Illustration.

Figure 110A.

#### Distribution.

*Ipomoea grandifolia* is apparently frequent in NE Argentina, Paraguay, eastern Bolivia and much of southern Brazil whereas *I. australis* is mostly found in the Andean foothills of Argentina and Bolivia but extends into Paraguay. There are few certain records of *I. grandifolia* from Bolivia, all from the eastern lowlands where it grows on disturbed grassy roadsides at low altitudes. The record from Peru appears correctly named but requires confirmation.

**URUGUAY.***E.J. Gibert* 240 (K).

**ARGENTINA. Chaco**: *A.G. Schulz* 10440 (CTES), 6349 (CTES). **Corrientes**: *J. Paula-Souza et al.* 7131 (CTES); *M. Dematteis et al.* 941 (CTES); Cáceres, *Zamudio* 298 (CTES). **Entre Ríos**: *A. Schinini* 12993 (CTES); *A. Burkart & N.S.Troncoso* 27875 (CTES). **Misiones**: *H. Keller* 8726 (CTES), 8738(CTES); *M. Dematteis & A. Krapovickas* 1920 (CTES).

**PARAGUAY. Amambay**: Pedro Juan Caballero, *A. Krapovickas et al*. 45906 (CTES, K). **Caazapá**: Tavai, *I. Basualdo* 002204 (FCQ); Abai, Com. Aché de Ypetimi, *P. da Motta* 93 (FCQ). **Canindeyú**: Ñandurokai, *B. Jiménez et al.* 1857 (BM, PY). **Concepción**: *K. Fiebrig* 5301 (BM, K). **Cordillera**: Pirareta, *E. Lurvey* 427 (PY); Eusebio Ayala, *E. Lurvey* 429 (PY). **Guiará**: Villarrica, *E. Hassler* 8710 (BM); Villarica–Paraguarí, *J. de Egea et al*. 1323 (FCQ); Yurai near Col. Independencia, *J.R.I. Wood et al.* 28156 (FCQ). **Misiones**: *E. Lurvey* 386 (PY); **Itapúa**: Triumfo, *E. Lurvey* 76 (PY). **Pres. Hayes**: *A. Krapovickas & C. Cristóbal* 43241 (CTES). **Misiones**: San Miguel, *F. Mereles & J. de Egea* 10140 (FCQ); San Juan Bautista, *E. Lurvey* 386 (PY). **San Pedro**: Est. Alegria, *F. González* 854 (FCQ).

**BRAZIL. Acre**: Rio Branco, *E. Ule* 8285 (K). **Amazonas**: Manaos, *J.W.H. Traill* 548 (K); *E. Ule* 5409 (K). **Bahia**: Correntina, *R.M. Harley* 21816 (K). **Mato Grosso**: north of Xavantina, *J.A. Ratter et al.* 1404 (E, MO) – intermediate with *I. cordatotriloba*. **Minas Gerais**: *A.F.M. Glaziou* 14128 (BM); *Trinta and Fromm* 1802 (CTES). **Paraná**: *A. Krapovickas & C. Cristóbal* 40921 (CTES); *G. Hatschbach* 47573 (HB, K); Jacarahy, *G. Jansson* s.n. [24/3/1914] (K). **Rio Grande do Sul**: *G.E. Barboza al.* 896 (CTES); *E. Pereira* 8628 (HB, K). **Rio de Janeiro**: *A.F.M. Glaziou* 13012 (K). **Santa Catarina**: *A. Krapovickas & C. Cristóbal* 43979 (CTES), 44000 (CTES, K).

**BOLIVIA. Cochabamba**: Carrasco: al lado del retén de Ivirgazama, *J.R.I. Wood & B. Williams* 27733 (K, LPB, USZ). **Chuquisaca**: Luis Calvo, La Pista, *E. Saravia* 10851 (HSB). **Santa Cruz**: Chiquitos: Santiago, *J.R.I. Wood* 28136 (LPB, OXF, USZ); Cordillera, Camiri, *J.R.I. Wood et al.* 28486 (LPB, USZ); Florida, Bermejo, *J.R.I. Wood* 28107 (LPB, OXF, USZ); Ibañez, salida a Abapó, *J.R.I. Wood et al.* 28474 (K, LPB, USZ); Ñuflo de Chávez, c. 1 km from centre of San Javier along road towards Concepción, *J.R.I. Wood & D. Soto* 27943 (OXF, K, LPB, USZ).

**PERU. Cusco**: La Convención, Huayapata, *G. Calatayud* 3261 (MO, OXF).

#### Notes.

*Ipomoea grandifolia* is relatively easy to distinguish in the field by the small entirely pink corollas which look distinct from the larger corollas of *I. australis* with their darker throat and pale limb.

Specimens from Formosa e.g. *Schinini et al.* 32696 (CTES) are intermediate with *Ipomoea australis. J.A. Ratter et al.* 1404 (E, MO) from north of Xavantina, Mato Grosso is problematic; the corolla is too large for *I. grandifolia* and *I. cynachifolia* (to which molecular data suggests it belongs) but it is out of the geographical range of *I. australis*.

*Ipomoea grandifolia* was a forgotten species misplaced in *Jacquemontia* until it was transferred into *Ipomoea* and rediagnosed by O’Donell (1952: 226–228). Comparing his summary of its characteristics in 1952 with that in his posthumous account of *Ipomoea* in Argentina (O’Donell 1959b) O’Donell had clearly come to depend on flower size alone to distinguish *I. grandifolia*, rather than any of the secondary characters discussed in 1952. Examination of the surviving isotype of *Ipomoea grandifolia* at Kew shows a plant with a corolla 2–2.2 cm long and narrowly ovate outer sepals which taper to a mucronate apex. This is a near perfect match for *Wood & Williams* 27733 from Ivirgazama in Cochabamba Department. Unfortunately the narrower sepals are no more convincing as a character than the corolla size as many specimens of *I. australis* have similar sepals, rather than the more usual ovate, more abruptly mucronate sepals often found in that species.

*Ipomoea grandifolia* is also very close to the widespread *I. triloba* L, which is absent from South America according to Austin (1978b) and Austin and Huáman (1996), although widely distributed as a weed in the Old World. Austin (1978b: 120) claims *I. grandifolia* is a hybrid but only suggests *Ipomoea australis* as one parent. Perhaps it has arisen as a result of hybridisation with an introduced *I. triloba* resulting in offspring showing a range of corolla sizes, sepal shape and indumentum that bridges the two species, but there is no molecular evidence for this.

### 229. Ipomoea triloba

Taxon classificationPlantaeSolanalesConvolvulaceae

L., Sp. Pl. 1: 161. 1753. (Linnaeus 1753: 161)

Convolvulus trilobus (L.) Desr. in Lam., Encycl. 3: 564. 1789 [pub. 1792]. (Desrousseaux 1792: 564).
Quamoclit triloba (L.) G. Don, Gen. Hist. 4: 259. 1838. (Don 1838: 259).
Amphione lobata Raf., Fl. Tellur. 4: 79. 1836 [1838], nom. illeg. superf. Type. Based on Ipomoea triloba L.
Ipomoea eustachiana Jacq., Obs. 2: 12, t. 36. 1767. (Jacquin 1767: 12). Type. Icon, t. 36 in Jacquin (1767), lectotype designated by Austin (1978b: 127).
Quamoclit eustachiana (Jacq.) G. Don, Gen. Hist. 4: 259. 1838. (Don 1838: 259).
Ipomoea triloba var. eustachiana (Jacq.) Griseb., Fl. Brit. W.I. 470. 1864 [pub. 1862]. (Grisebach 1862b: 470).
Ipomoea parviflora Vahl, Symb. Bot. 3; 34. 1794. (Vahl 1794: 34). Type. U.S. VIRGIN ISLANDS. St Croix, *H. West*s.n. (C100009694, lectotype, designated here).
Ipomoea galapagensis Anderss., Kongl. Vetensk. Acad. Handl. 1853: 313. 1855. (Andersson 1855: 313). Type. ECUADOR. Galapagos Islands, Chatham Island, *N. Andersson* 120 (holotype S07-4429, isotype K).
Ipomoea hirta M. Martens & Galeotti, Bull. Acad. Roy. Soc. Bruxelles 12: 264. 1845. (Martens and Galeotti 1845: 264). MEXICO. Oaxaca, *H. Galeotti* 1374 (lectotype BR000006973315, isolectotypes BR, G, K, P).
Ipomoea triloba var. genuina Meisn. in Martius et al., Fl. Brasil. 7: 277. 1869, (Meisner 1869: 277), nom. illeg. autonymic var.
Ipomoea triloba var. quinqueloba Kuntze, Rev. Gen. Sp. 2: 446. 1891. (Kuntze 1891: 446). Type. U.S. VIRGIN ISLANDS. St Thomas, *O. Kuntze* 26 (lectotype NY000111084, designated here).
Convolvulus heterophyllus Sessé & Moçiño, Fl. Mex. 36. 1893. (Sessé y Lacasta and Moçiño 1893: 36), nom. illeg., non Convolvulus heterophyllus Willd. (1809). Type. MEXICO. Sessé and Moçiño 1655 (holotype MA603868).
Ipomoea krugii Urb., Symb. Antill. 5: 472. 1908. (Urban 1908: 472). Type. PUERTO RICO. Mayagüez, Krug 776 (holotype B†, photo F).
Ipomoea laxiflora H.J.Chowdhery & Debta, Indian J. Forest. 32(1): 120. 2009 (Chowdhery and Debta 2009: 120). Type. INDIA. Uttarakhand, Dehra Dun district, Botanical Survey of India Campus, *H.J. Chowdhery* 108601 (holotype BSD, isotype CAL0000018586).

#### Type.

Icon in Sloane, Voy. Jamaica 1: t. 97, f. 1 (1707), lectotype, designated by Austin (1978b: 127).

#### Description.

Annual herb, stems twining, thinly pilose to glabrescent. Leaves petiolate, 1.5–8 × 1.5–4 cm ovate or, more commonly shallowly to deeply 3-(5)-lobed, acute to acuminate, apiculate, base cordate, adaxially thinly pilose, abaxially glabrous, paler, occasionally both surfaces glabrous; petioles 1.2–6 cm. Inflorescence of pedunculate axillary cymes; peduncles 3–5 cm, glabrous or thinly pilose; bracteoles 2–3 × 0.25 mm, filiform; secondary peduncles 0.2–0.5 cm; pedicels 3–7 mm, thinly pilose, sometimes muricate; sepals scarious-margined, ciliate on midrib and margins, subequal, 5–6 (–10) mm long, oblong-mucronate or oblong-caudate; corolla 1.5–2 (–2.5) cm long, campanulate, glabrous, pink; limb 1.3–1.6 cm diam. Capsules 5–6 mm diam., subglobose, bristly pilose (rarely glabrous); seeds 2.8–3 × 2 mm, brown glabrous.

#### Illustration.

Figure 10A; Acevedo-Rodríguez (2005: 183); Proctor (2012: 548).

#### Distribution.

Common on the Galapagos Islands and in the Caribbean, but rare elsewhere except as an introduced weed. It is apparently more frequent on islands than on the continent. This species is quite commonly reported as a casual or a weed in the Old World and its near complete absence from continental South America is, therefore, puzzling. It is possible that it has sometimes been confused with *Ipomoea grandifolia*.

**ECUADOR. Galápagos**: *F.R. Fosberg* 45049 (K, US); *Snow* 560 (K); *T.W.J. Taylor* 133 (K); *G. Harling* 5227 (S); *Fagerlind & Wibom* 2935 (S); *U. & I. Eliasson* 2167 (S); Santa Cruz, *P.S. Bentley* 221 (K, US). **Loja**: Garza Real-Paletillas Malvas, *J. Jaramillo et al.* 31949 (QCA). **Napo**: Yasuní, *V. Persson et al.* 4614a (BM).

**COSTA RICA.** Puntarenas, Golfito, *M. Chavarría* 673 (K, MO).

**HONDURAS.** Copán-San Pedro Sula, *S. Blackmore & M. Chorley* 3776 (BM)

**BELIZE.** Honey Camp, *C.L. Lundell* 656 (S); Caye Caulker, *C. Whitefoord* 8231 (BM).

**GUATEMALA.***R. Tun Ortíz* 258 (S)

**MEXICO. Campeche**: Kalkiní-El Remate, *M. Peña-Chocarro et al.* 591 (BM). **Chiapas**: Berriozabal, *A. Reyes-García et al.* 431 (BM, MEXU). **Chihuahua**: *E. Palmer* 213 (K). **Est. México & Dist. Fed.**: Tejupilco, Temascaltepec, *G.B. Hinton* 8416 (K), ibid., Nanchititla, *G.B. Hinton* 8557 (K). **Guerrero**: Vallecitos, Montes de Oca, *G.B. Hinton* 10915 (K); Acapulco, *E. Palmer* 141 (K). **Nayarit**: Tepic, *G. Flores-Franco et al.* 4229 (MEXU). **Oaxaca**: *M. Elorsa* 2356 (MEXU). **Sinaloa**: Concordia, *M. Ruiz et al.* 2009-336 (ARIZ). **Sonora**: Río Mayo, *H.S. Gentry* 1681 (E, K); Guaymas, *E. Palmer* 306 (BM, E); Pitihaya, *R.S. Felger & F.W. Reichenbacher* 85-1296 (ARIZ). **Tabasco**: *E. & H. de Cabrera* 14993 (MEXU). **Tamaulipas**: Tampico, *E. Palmer* 472 (BM, K). **Veracruz**: *P. Pedraza* 236 (F). **Yucatán**: Izamal, *F. Gaumer* 981 (BM, E, K); Chichancanab, *F. Gaumer* 2117 (BM, S); Cozumel Island, *F. Gaumer* s.n. (K).

**UNITED STATES. Arizona**: fide Austin (1991a). **California**: Riverside, A.C. Sanders 8743 (ARIZ, DES). **Florida**: *A.H. Curtiss* 5575 (E); *J.K. Small* 8729 (NY, S); *R.T. Clausen & W.M. Buswell* 6224 (K); *J.H. Simpson* 397 (K); *F. Rugel* 506 (BM). **Texas**: *E. Hall* 484 (BM)

**BAHAMAS.** Watlings Island, *P. Wilson* 7296 (K, NY); Grand Bahama, *D.S. Correll* 40474 (NY); *Webster & Williams* 10767 (S).

**TURKS & CAICOS ISLANDS.***M.R. Corcoran* 41 (K); *D.S. Correll* 43295 (NY).

**CUBA.***C.F. Baker* s.n. [5/11/ 1904] (HAJB); *C. Wright* 3085 (BM, NY, S). **La Habana**: *H.A. Van Hermann* 159 (NY). **Santiago de Cuba**: *Chrysogone* 4890 (NY); *R.A. Howard* 5783 (S, NY).

**CAYMAN ISLANDS.***W. Kings* GC330 (BM); *G.R. Proctor* 35184 (BM); *M. Brunt* 1941 (BM)

**JAMAICA.***Asprey* 372 (K); *C.D. Adams* 6193 (BM); *G.R. Proctor* 16094 (BM), 34309 (BM); *W. Harris* 10163 (BM).

**DOMINICAN REPUBLIC.***L.C. Richard* s.n. (P).

**PUERTO RICO.***P. Sintenis* 827 (K, S); *M. Del Llano* s.n. [7/9/1979] (NY).

**LESSER ANTILLES. U.S. Virgin Islands**: St Thomas, *H.F.A. von Eggers* 254 (K); St Croix, *F.R. Fosberg* 54140 (K, NY). **U.K. Virgin Islands**: Tortola, *W.G. D’Arcy* 317 (BM, FLAS). **Netherlands Antilles**: St Eustatius: *B.M. Boom et al.* 11185 (NY). **Anguilla**: *G.R. Proctor* 18520 (BM). **St Kitts**: *G.R. Proctor* 18484 (BM). **Martinique**: *Hahn* 83 (BM, K); *C. Sastre* 9910 (P). **Guadeloupe**: H. Stehlé79 (NY). **Barbados**: *A. Macintosh* 349 (K). St Barts, Antigua fide Powell (1979).

**TRINIDAD.** fide Hill and Sandwith (1953). **Tobago**: *N. Sandwith* 1818 (K, NY).

**NETHERLANDS ANTILLES.** Aruba, Bonaire, Curaçao fide Proosdij (2012)

**HAWAII.** Oahu, *O. Degener* 24355 (K), *C. Pemberton & J.P. Martin* s.n. [18/3/1943] (BM).

#### Notes.

There are two specimens of Ipomoea triloba var. quinqueloba in the Kuntze herbarium at New York, both collected on St Thomas in the U.S. Virgin Islands. Neither is a very good specimen, but that labelled no. 26 is here selected as a lectotype, rather than no. 149.

We have included *Ipomoea laxiflora* as a synonym of *I. triloba*, even though we have seen no specimens. It differs only in the glabrous ovary and capsule, a variation which is not likely to be significant at species level. Molecular studies of a range of specimens would be desirable to confirm our decision here.

### 230. Ipomoea ramosissima

Taxon classificationPlantaeSolanalesConvolvulaceae

(Poir.) Choisy in A.P. de Candolle, Prodr. 9: 377. 1845. (Choisy 1845: 377)

Convolvulus cymosus Ruiz & Pav., Fl. Peruv. 2: 9. 1799 (Ruiz and Pavón 1799: 9), non Ipomoea cymosus Desr. (1792). Type. PERU. Huánuco, *Ruiz & Pavón*s.n. (lectotype MA 814677, designated by Wood et al. (2015; isolectotypes F, MA, OXF).
Convolvulus ramosissimus Poir., Encycl., Suppl. 3: 468. 1813 [pub. 1814]. (Poiret 1814–17: 468). Type. Based on C. cymosus Ruiz & Pav.
Ipomoea dichotoma Choisy in A.P. de Candolle, Prodr. 9: 383. 1845. (Choisy 1845: 383), nom. illeg., non Ipomoea dichotomaKunth (1819). Type. BRAZIL. Lund 319 (holotype G00135826!).
Ipomoea dichotoma var. longiflora Choisy Prodr. [A.P. de Candolle] 9: 383. 1845. (Choisy 1845: 383). Type. BRAZIL. Moritiba, J.S. Blanchet 3482 (holotype G00227888, isotypes F, NY, P).
Ipomoea dichotoma var. integrifolia Meisn. in Martius et al., Fl. Brasil. 7: 281. 1869 (Meisner 1869: 281). Type. BRAZIL. *Martius*s.n. (lectotype M0184976, designated here) .
Ipomoea dichotoma var. trilobata Meisn. in Martius et al., Fl. Brasil. 7: 281. 1869. (Meisner 1869: 281). Type. BRAZIL. *W.J. Burchell* 1066 (lectotype BR00005793983, designated here).
Ipomoea ramosissima var. rosea Hallier f., Jahrb. Hamburg Wissens. Anst. 16: 45. 1899. (Hallier 1899a: 45). Type. BRAZIL. Santa Catarina, Blumenau, *E. Ule* 770 (holotype B†).
Ipomoea ramosissima forma rosea (Hallier f.) O’Donell, Arq. Mus.Parana 9: 231. 1952. (O’Donell 1952: 231).
Ipomoea dichotoma subvar. hirsuta Hallier f., Jahrb. Hamburg Wissens. Anst. 16: 45. 1899. (Hallier 1899a: 45). Type. BRAZIL. Rio de Janeiro, Serra dos Orgãos, *E. Ule* 2412 (holotype HBG, n.v.).
Ipomoea perplexa L.O. Williams, Fieldiana Bot. 32: 193. 1970. (Williams 1970a: 193). Type. BELIZE. *H.H. Bartlett* 12868 (holotype MICH1111342, isotype F).
Ipomoea quesadana Standl., Publ. Field Mus. Nat. Hist., Bot. Ser. 22: 99. 1940. (Standley 1940c: 99). Type. COSTA RICA. Alajuela, Villa Quesada, *A.C. Smith* 1609 (holotype F0054892, isotype EAP).

#### Type.

Based on *Convolvulus cymosus* Ruiz & Pav.

#### Description.

Slender twining annual or possibly short-lived perennial herb, usually nearly glabrous in all parts but occasionally stems thinly pilose. Leaves petiolate, mostly 3–5.5 × 2–4.5 cm, ovate or shallowly 3-lobed, cordate with rounded to obtuse auricles, apex shortly acuminate, mucronate, glabrous or adaxially with a few hairs; petioles 1.5–5 cm, glabrous or thinly pubescent. Inflorescence of long pedunculate axillary umbelliform cymes with 2–5 flowers; peduncles 2–10 cm; bracteoles tiny, triangular, caducous; pedicels 5–15 mm; sepals subequal, oblong-obovate with broad scarious margins, rounded and mucronate, glabrous or with a few marginal cilia, outer sepals 3.5–6 mm,; inner sepals c. 1 mm longer; corolla 1.5–2.5 cm long, subcampanulate to shortly funnel-shaped, pink with a dark centre, glabrous, limb 1.5–1.75 cm diam., unlobed or shallowly lobed, sometimes dentate. Capsules 2–3 × 4 mm, depressed-subglobose, enclosed by sepals, glabrous, the slender style somewhat persistent; seeds 3 × 2.5 mm, ellipsoid, dark brown, glabrous or pilose on the angles.

#### Illustration.

**Figure 117.**
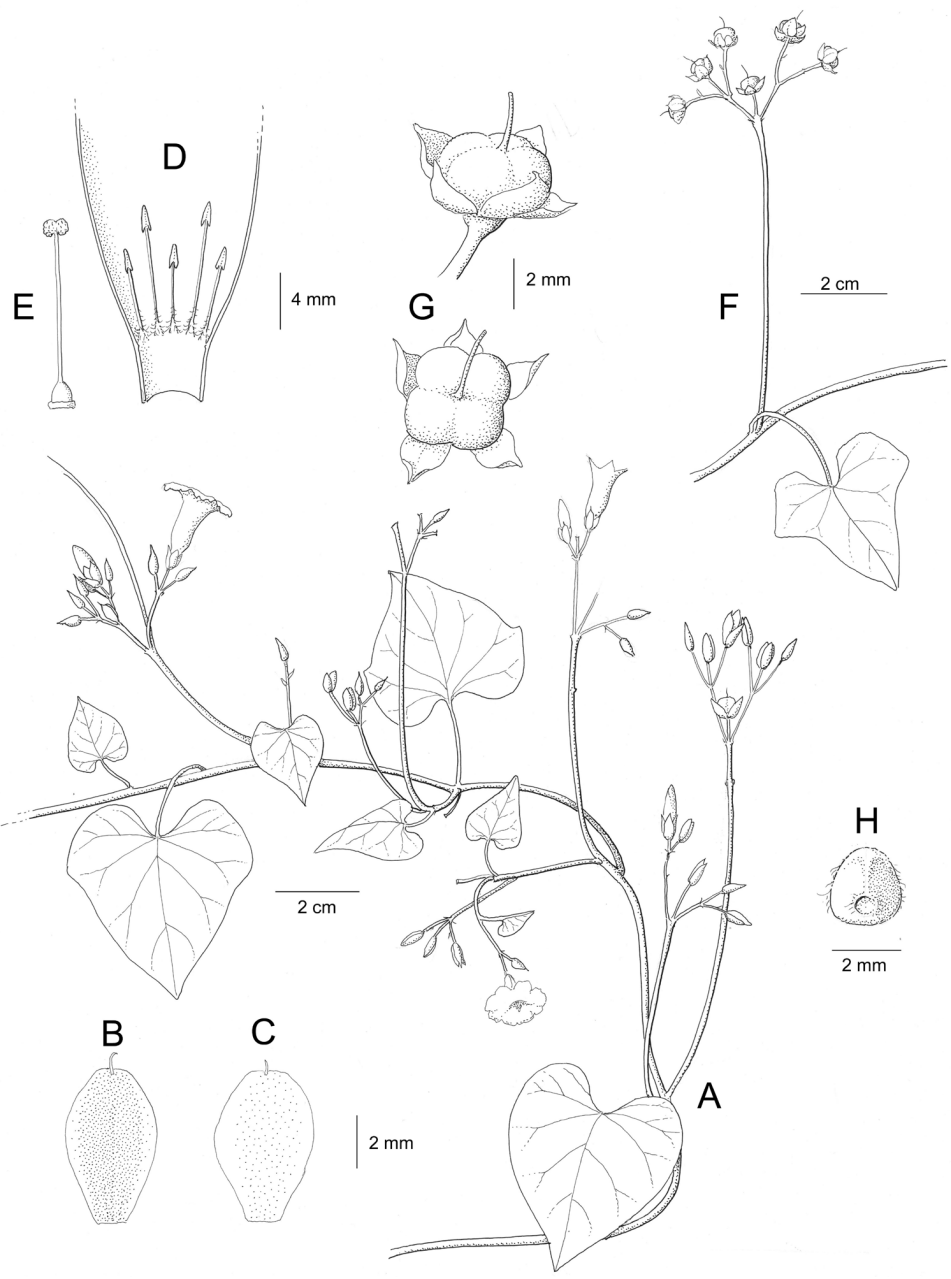
*Ipomoea ramosissima*. **A** habit **B** outer sepal **C** inner sepal **D** corolla opened out to show stamens **E** ovary and style **F** fruiting inflorescence **G** capsules **H** seed. Drawn by Rosemary Wise **A** from *Wood & Soto* 27957; **B–F** from *Wood & Soto* 27942; **G** from photo.

Figures 8P, 117; O’Donell (1959b: 229).

#### Distribution.

Widely distributed in tropical America south to Argentina growing in the tropical lowlands and perhaps favouring areas with good rainfall but with a distinct dry season. Like *Ipomoea dumetorum* it is noticeably more common south of the Equator, being rare in Mesoamerica and not recorded at all from Guatemala and Mexico. It grows on forest margins, in forest relics and in disturbed places around settlements, often appearing on wire fences. It is usually found below 500 m but reaches at least 1600 m in the Andes.

**ARGENTINA. Salta**: Oran, *S. Venturi* 5590 (BM, SI); *Legname & Cuezzo* 7483 (LIL, CTES). **Jujuy**: Ledesma, *J.H. Hunziker et al.* 12233 (MO).

**BRAZIL. Amazonas**: *I.L. do Amaral* 492 (NY). **Bahia**: Ilheus, *Mattos Silva et al.* 3487 (CTES, UESC); ibid., *J.L. Hage & E.B. dos Santos* 1085 (K). **Dist. Fed.**: *H.S. Irwin et al.* 15321 (CTES, NY). **Espirito Santo**: Santa Bárbara de Caparaó, *Y. Mexia* 4103 (BM, S). **Goiás**: Caiaponia-Aragarças, *D.R. Hunt* 6104 (K): Niquelândia, *F.C.A. Oliveira* 329 (RB). **Mato Grosso**: Novo Mundo, *D. Zappi et al.* 1317 (K). **Minas Gerais**: *A. Macedo* 1788 (S); *D.R. Hunt* 5436 (K). **Paraná**: *Jansoun* 64 a (K, S); *G. Hatschbach* 47573 (HB, K). **Pernambuco**: *A.M. Miranda* 3466 (RB). **Rio de Janeiro**: *G. Gardner* 5558 (BM); *Widgen* 520 (S); *J. Miers* s.n. (BM). **Rondônia**: *L. Texeira* 435 (NY, RB).

**BOLIVIA.***M. Bang* 2246 (E, NY, GH, F, K, MO). **Beni**: Est. Biológica del Beni, *T. Killeen & Palacios* 3441 (ARIZ, BOLV, MO, USZ); Marbán, Puente San Pablo, *M. T. Martinez & M. Adler* 84 (K, LPB, USZ). **La Paz**: Sud Yungas, Río Bopi, *B.A. Krukoff* 10692 (GH, F, K, MO); A.N.M.I. Madidi, Asariamas, *L. Cayola* 1743 (LPB, MO). **Santa Cruz**: Guarayos, Ascención, *J.R.I. Wood & D. Soto* 27933 (OXF, K, LPB, USZ); Ibañez: Los Espejillos, *G.A. Parada et al.* 195 (MO, OXF, USZ); Ichilo, Buenavista, *J. Steinbach* 7165 (BM, E, F, K, NY); Santiesteban, Río Grande, *J.R.I. Wood & D. Soto* 27950 (LPB, OXF, USZ); Ñuflo de Chávez, Concepción, *J.R.I. Wood & D. Soto* 27937 (OXF, K, LPB, USZ).

**PERU. Amazonas**: Condorcanqui, *J.A. Leveau* 14 (MO). **Cusco**: Quispicanchis, Río Araza, *P. Nuñez* 14107 (MO); La Convención, Vilcabamba, *G. Calatayud et al.* 2554 (CUZ, MO); *C. Vargas* 4579 (CUZ). **Huánuco**: *E. Asplund* 12638 (S). **Junín**: *A. Lourteig* 3095 (P, USM); Montayaco, near San Ramón, *A. Gentry & G.T. Prance* 16427 (MO). **Loreto**: Aguaytia, *F. Woytowski* 34457 (MO). **Madre de Dios**: Manu, Atalaya, *C. Sobrevila et al.* 1781 (F); Tambopata, *P. Nuñez et al*. 11116 (MO). **Pasco**: Oxapampa, Palcazu, *R. Vásquez & A. Monteagudo* 27727 (MO, USM). **San Martín**: *D. Melin* 214 (S); *R. Ferreyra* 4753 (USM); Taropoto, *R. Spruce* s.n. (K); Lamas, *J. Schunke* 9751 (MO, USM).

**ECUADOR. Galápagos**: *G. Harling* 5120 (S); *U. & I. Eliasson* 939 (S). **Napo**: Río Napo, *H. Lugo* 2269 (K, MO); *J. Korning & K. Thomsen* 47027 (AAU). **Pastaza**: Montalva, *B. Ljtnant & U. Molau* 13458 (AAU). **Pichincha**: Cerro Antisana, *P. Grubb et al.* 36 (K). **Zamora-Chinchipe**: *T. Croat* 91949 (MO, QCNE).

**COLOMBIA. Amazonas**: Araracuara, *L. Aguirre* 1003 (COL). **Putumayo**: Puerto Asís, *J. Cuatrecasas* 11250 (COL).

**VENEZUELA. Amazonas**: Atabapo, *E. Marín* 1687 (MO). **Portuguesa**: Las Cruces, *B. Stergios* 6629 (MO).

**PANAMA.***R.L. Liesner* 95 (RB); Darién, *J.A. Duke* 10217 (MO).

**COSTA RICA.** Guanacaste, Cordillera de Tilarán, *G. Rivera* 3016 (CR, K); *B. Hammel et al.* 18888 (MO); Alajuela, Artezalea, *A. Molina* 17239 (F).

**NICARAGUA.** Matagalpa, *W.D. Stevens* 11945 (CTES; MO); Río San Juan, El Catillo, R. Loredo 2366 (BM, MO); Chontales, *R. Tate* 248 (BM, K).

**EL SALVADOR.** Ahuachapán, Río Paz, *A. Munro et al.* 3614 (BM).

**BELIZE.** Chiquibul Forest Reserve, *C. Whitefoord* 10516 (BM), 10033 (BM, MO); ibid., *A. Munro et al.* 1123 (BM, MO).

#### Notes.

*Ipomoea ramosissima* can generally recognised by its small flowers and obovate sepals. It is usually glabrous but can only be safely separated from *I. cynanchifolia* when in fruit. The ripe capsules are always glabrous and distinctly depressed. There remains a considerable residue of non-fruiting specimens in herbaria, principally from Bolivia which could be either *I. cynanchifolia* or *I. ramosissima*.

Records from Paraguay (Austin and Costea 2008) are errors for *Ipomoea amnicola*. The record from the Galapagos Islands is unexpected but seems correct.

### 231. Ipomoea cynanchifolia

Taxon classificationPlantaeSolanalesConvolvulaceae

Meisn. in Martius et al., Fl. Brasil. 7: 274. 1869. (Meisner 1869: 274)

#### Type.

BRAZIL. Minas Gerais, Lagoa Santa, *E. Warming* (lectotype BR000005951567, flowering portion on sheet, designated by O’Donell (1952: 218), isolectotype P).

#### Description.

Slender twining annual herb, nearly glabrous in all parts. Leaves petiolate, mostly 3–5.5 × 2–4.5 cm, ovate or shallowly 3-lobed, cordate with rounded to obtuse auricles, apex shortly acuminate, mucronate, adaxially thinly pubescent or glabrous; petioles 1.5–5 cm. Inflorescence of long pedunculate axillary umbelliform cymes with 2–5 flowers; peduncles 2–10 cm; bracteoles tiny, triangular, caducous; pedicels 5–15 mm; sepals subequal, oblong-obovate with broad scarious margins, rounded and mucronate, usually glabrous but occasionally ciliate; outer sepals 3.5–6 mm; inner sepals c. 1 mm longer; corolla 1.5–2.5 cm long, funnel-shaped, pink with a dark centre, glabrous, limb 1.5–1.75 cm diam., unlobed, sometimes dentate. Capsules 3–4 × 4 mm, ovoid, exceeding sepals, glabrous or thinly pilose, the slender style somewhat persistent; seeds 3–3.5 × 2.5 mm, ellipsoid, dark brown, glabrous.

#### Illustration.

Figure 118.

**Figure 118.**
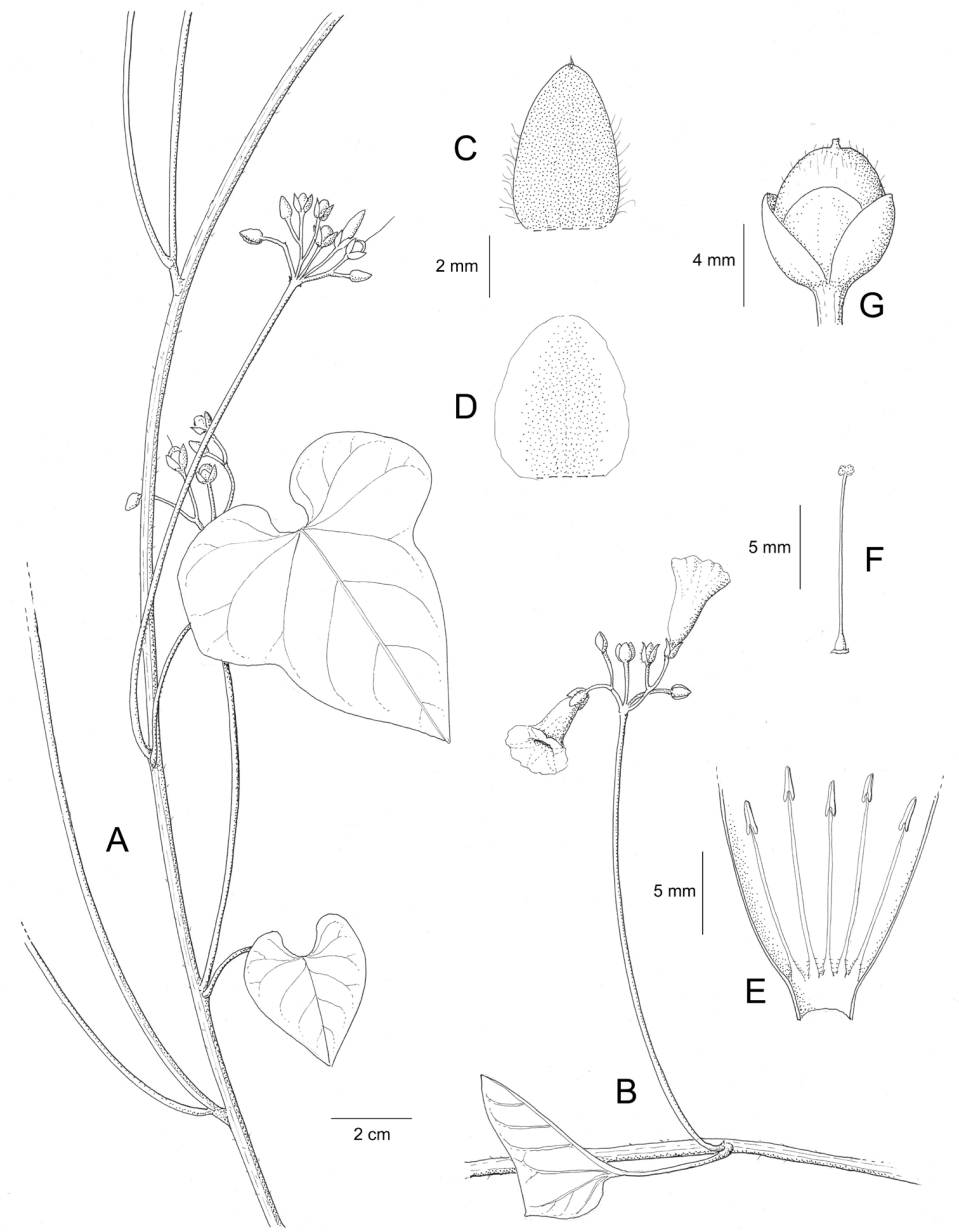
*Ipomoea cynanchifolia*. **A** habit **B** inflorescence **C** outer sepal **D** inner sepal **E** corolla opened out to show stamens **F** ovary and style **G** capsule. Drawn by Rosemary Wise from *Y. Mexia* 4497.

#### Distribution.

This species is known from scattered locations in Brazil and Bolivia but may be under-recorded. It is a plant of the Cerrado biome, usually below 700 m in disturbed places usually near settlements or around rock outcrops.

**BRAZIL. Bahia**: 4 km N. of Bom Jesus da Lapa, *R.M. Harley et al.* 21572 (K). **Dist. Fed.**: *Ramalho et al.* 43 (UB); *D. Alvarenga* 701 (IBGE, OXF). **Goiás**: Niquelândia, *F.C.A. Oliveira et al.* (IBGE, OXF). **Rio de Janeiro**: *A. Glaziou* 14128 (K). **Minas Gerais**: *Y. Mexia* 4497 (BM, S). **São Paulo**: Mun. Eldorado, Est. Jacupiranga, *Braidotti et al.* 1 (SP, CTES).

BOLIVIA. Santa Cruz: Ángel Sandoval, 51 km S of Las Petas sobre el camino a Candelaria, *J.R.I. Wood et al.* 24871 (K, LPB, UB, USZ); Ascención de Guarayos, en camino a San Ramón, *M. Mendoza et al.* 2146 (K, USZ). Ibañez, Angostura, *R. Steinbach* 328 (NY, MICH). Ichilo, Reserva El Choré, *G.A. Parada et al.* 22 (OXF, MO, USZ); Ñuflo de Chávez, salida de Concepción, *J.R.I. Wood et al.* 24117 (K, LPB, UB, USZ); c. 25 km from Concepción along road to San Javier, *J.R.I. Wood & D. Soto* 27941 (USZ); Velasco, Reserva Forestal Bajo Paraguá, Cerro Diamentina, *T. Killeen & J. Wellens* 6343 (ARIZ, LPB, USZ, MO); 5 km N de San Miguel en camino a San Ignacio, *J.R.I. Wood et al.* 24284 (K, LPB, UB, USZ); Warnes, Las Barreras, *F.E. Tollervey* 2519 (K).

#### Notes.

*Ipomoea cynanchifolia* is very close to *I. ramosissima* and is only safely separable when good fruit is available. It is distinguished by the thinly pilose (rarely glabrous) ovoid capsules which are clearly visible above the fruiting calyx. The shape of the fruiting capsule is the decisive character as the capsule indumentum is not constant in the Batatas Clade. No secondary characters are reliable but it is noteworthy that most specimens cited above and by O’Donell (1952: 218) flower in the March–May period, much earlier than *Ipomoea ramosissima*.

*Ipomoea cynanchifolia* has the appearance of a hybrid between *Ipomoea ramosissima* and *I. grandifolia* but there is no molecular evidence to support this suggestion. It combines the characters of the two species and is more or less sympatric with the latter.

### 232. Ipomoea tenuissima

Taxon classificationPlantaeSolanalesConvolvulaceae

Choisy in A.P. de Candolle, Prodr. 9: 376. 1845. (Choisy1845: 376)

#### Type.

HISPANIOLA. *Desportes* s.n. (lectotype P-JUSS-6797 [P00666123], designated here).

#### Description.

Slender herb, stems glabrous to bristly pilose. Leaves petiolate, small, 2–4.5 × 0.5–1 cm, strap-shaped, strongly sagittate, sometimes with a smaller side lobe, apex obtuse, apiculate, usually both surfaces evenly hirsute; petioles 0.7–1.5 cm. Flowers solitary or paired from the leaf axils; peduncles 1.3–3.5 cm; bracteoles 2 mm, filiform, tardily deciduous; pedicels 2–7 mm; sepals quite variable in indumentum and shape, outer sepals 5–7 mm, oblong-oblanceolate, acuminate and apiculate, pubescent, often ciliate, often spreading at maturity; inner sepals narrowly elliptic-obovate, obtuse, mucronate; corolla 2.5–3 cm long, narrowly funnel-shaped, deep pink, glabrous, limb c. 1–1.2 cm diam. Capsules subglobose, 4–6 mm, the style somewhat persistent, pilose or glabrous; seeds 2–2.5 × 1.5–2 mm, glabrous.

#### Illustration.

**Figure 119.**
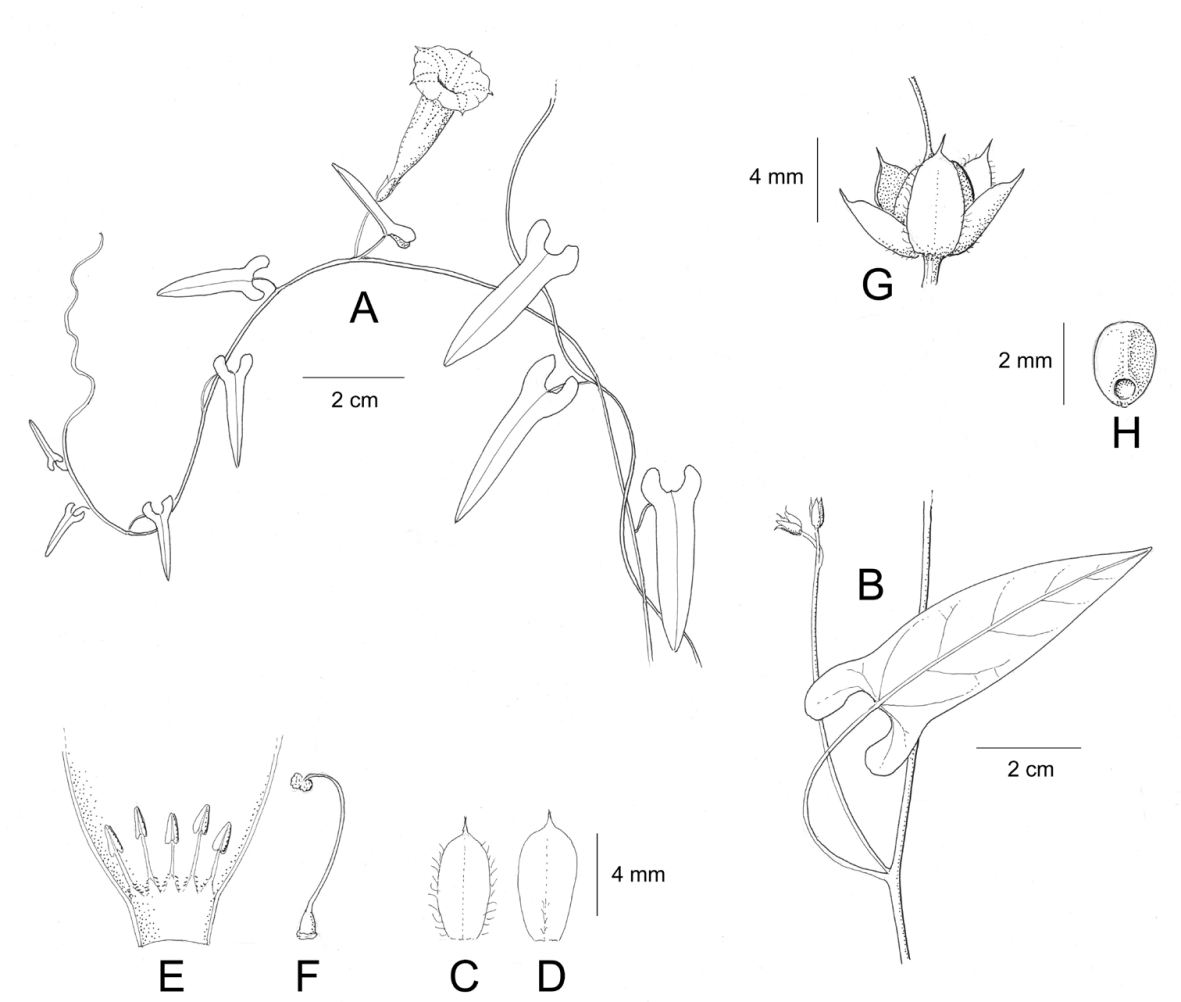
*Ipomoea tenuissima*. **A** habit **B** habit with larger leaf **C** outer sepal **D** inner sepal **E** corolla opened out to show stamens **F** ovary, style and stigma **G** fruiting calyx and capsule **H** seed. Drawn by Rosemary Wise **A, C–H** from *Wright* 1651; **B** from *Curtiss* 495.

Figure 119; Acevedo-Rodríguez (2005: 180).

#### Distribution.

Centred on Cuba and extending north to Florida and east to the Island of Hispaniola. Recorded as growing in pinelands in Florida.

**UNITED STATES. Florida**: *J.K. Small et al.* 6557 (S), 6581 (K, S); *J.K. Small & Carter* 1903 (K); Dade County, *L.J. Brass* 2421(ARCH).

**CUBA.***C. Wright* 1651 (BM, NY, P, S); *Earle & Wilson* 2412 (HAC); *Bro. León & Dahlgren* 23399 (HAC); *J. Bisse & F. Meyer* (HAJB28182). **Camagüey**: *J. A. Shafer* 1139 (NY). **Cienfuegos**: *R. Combs* 238 (K, MO, P). **Holguin**: Mir, *E.L. Ekman* 7528 (BM, S). **Isla de Juventud (Pinos)**: *E.P. Killip* 43943 (NY, P, S); *A.H. Curtiss* 495 (BM, E, HAC, K, MO, NY, P). **La Habana**: *N.L. Britton et al.* 680 (HAC, NY); Vibora, *E.L, Ekman* 1262 (NY). **Matanzas**: *Bro. León* 12493 (NY). **Pinar del Río**: *N.L. Britton et al.* 6336 (NY). **Villa Clara**: Manacas, *Bro. León* 5854 (NY).

**HAITI.** Massif du Nord, *E.L. Ekman* H6092 (NY, S), H8395 (S).

**DOMINICAN REPUBLIC.** La Vega, Jarabacoa, *E.L. Ekman* H14163 (S); Santiago, San José de las Matas. Leonor, *E.J. Valeur* 504 (K, MO, NY, S); San Juan, *A. Liogier* 12462 (NY); ibid., *R.A. & E.S. Howard* 8738 (BM, NY).

**PUERTO RICO.** Mona Island, Cabo Rojo, *N.L. Britton et al.* 2397 (NY).

#### Typification.

The lectotype in P-JUSS is not annotated by Choisy but is the only possible specimen that could be chosen as the type.

#### Note.

Easily distinguished by the strap-shaped, strongly sagittate leaves which are hirsute on both surfaces. The flowers are always solitary or paired. For a relatively slender plant the corolla is quite long reaching 3 cm. *Ekman* H8395 is an unusually robust example.

### 233. Ipomoea cryptica

Taxon classificationPlantaeSolanalesConvolvulaceae

J.R.I. Wood & Scotland, Kew Bull. 70 (31): 92. 2015. (Wood et al. 2015: 92)

Ipomoea peckoltii var. major Meisn. in Martius et al., Fl. Brasil. 7: 268. 1869. (Meisner 1869: 268). Type. BRAZIL. [Amazonas], ad oram meriodionalem flum. Amazonum, ad ostium flum. Solimoes, *R. Spruce* 1702 (holotype B?†, isotypes BM, K000612858, P).

#### Type.

BOLIVIA. Santa Cruz, Prov. Ichilo, 2–10 km from Buenavista along road to Huaytu, *J.R.I. Wood & D. Soto* 27955 (holotype USZ, isotypes K, LPB, OXF).

#### Description.

Twining perennial or liana to 5 m, stems glabrous. Leaves petiolate, ovate-deltoid, mostly 4–10 × 2–7.5 cm, base broadly cordate to subhastate, the auricles usually acute, sometimes rounded, apex acuminate to an obtuse and mucronate apex, both surfaces glabrous; petiole 1–5 cm, glabrous. Inflorescence of rather dense, 3–15-flowered, axillary, pedunculate cymes; peduncles 5–12 cm, glabrous; bracteoles ovate, acute, c. 2 mm long, caducous; secondary peduncles and pedicels short, 5–8(–13) mm, glabrous; sepals very unequal, glabrous, outer 1–3 mm long, suborbicular to elliptic, the margins scarious, inner 7–8 mm, broadly elliptic, rounded, margins broad, scarious; corolla 3.5–6 cm long, glabrous, funnel-shaped, tube lilac, limb unlobed, 3.5–4 cm diam., pink. Capsules 10–11 × 6–8 mm, ellipsoid, glabrous, the style persistent as a long awn about as long as the capsule; seeds 6 × 3 mm, blackish with long white marginal hairs c. 6 mm long.

#### Illustration.

**Figure 120.**
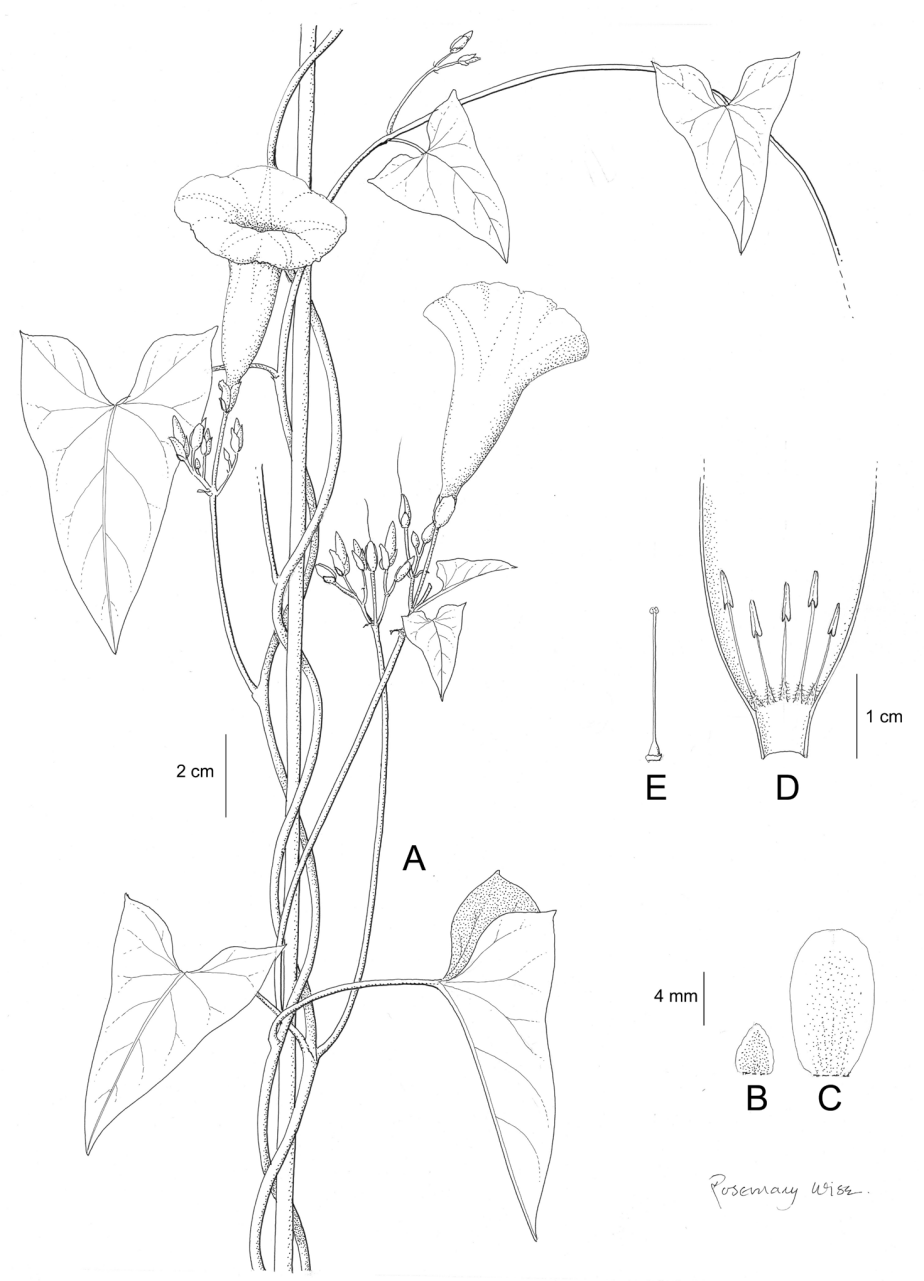
*Ipomoea cryptica*. **A** habit **B** outer sepal **C** inner sepal **D** corolla opened out to show stamens **E** ovary and style. Drawn by Rosemary Wise from *Parada et al.* 1537.

Figures 7C, 10B, 110D, 120.

#### Distribution.

Locally common in NE Bolivia and present also in Colombia, Peru and Brazil, possibly frequent in the SW Amazonian region. In Bolivia it is a species of lowland forest, forest relics and drainage dykes, growing usually in seasonally flooded places.

**BRAZIL. Amazonas**: Rio Juruá [Yarúa] near Independencia, *B.A. Krukoff 4582* (BM, NY, S).

**BOLIVIA. Beni**: Yacuma/Ballivián, Est. Biológica de Beni, *E. Rivero* 152 (CTES, LPB, SP, USZ); Cercado, Laguna Limonsin, *D. Soto et al.* 1331 (OXF, USZ); Marbán, Casarabe, *F. de la Puente* 3572 (CIP, FTG); Moxos, *G.A. Parada et al.* 1537 (OXF, MO, USZ); Laguna Mauso, *D. Soto et al.* 1487 (USZ). **La Paz**: Larecaja, Guanay, *H. Rusby* 1987 (BM, K, NY, MICH, P, US). **Santa Cruz**: Germán Busch, Rincón del Tigre-La Gaiba, *J.R.I. Wood et al.* 28721 (K, LPB, UZ); Ichilo, Río Surutú, *J. Steinbach* 6311 (A, K); Buenavista to Huaytu, *J.R.I. Wood & D. Soto* 27955 (OXF, K, LPB, USZ); San Carlos, *M. Martinez* 2 (OXF, USZ); Santiesteban, between Montero and Okinawa, *J.R.I. Wood & D. Soto* 27952 (OXF, K, LPB, USZ); Sara, Buenavista to Portachuelo, *J.R.I. Wood & D. Soto* 27961 (OXF, K, LPB, USZ).

**PERU. Loreto**: Florida, Río Putumayo, at mouth of Río Zubineta, *G. Klug* 2049 (BM, S); left bank of Río Marañon above Rancho Indiana, *Y. Mexia* 6408 (BM, S).

**COLOMBIA. Guainía**: Colombian side of Río Orinoco, near Río Atabapo, *J.J. Wurdack & L.S. Adderley* 42789 (P).

#### Notes.

This species is very similar morphologically to glabrous-leaved forms of *Ipomoea squamosa* and *I. anisomeres* because of the short outer sepals but molecular studies indicate there is no close relationship. However, like both these species *I. cryptica* has a congested, many-flowered inflorescence and sepals with distinct scarious margins. From *Ipomoea squamosa* it is best separated by the very short outer sepal (2–3 mm long) and the completely glabrous stem, petioles and leaves; *I. squamosa* is usually at least thinly pubescent at the base or near the margins of the leaves in South American specimens. From *I. anisomeres* it is best distinguished by the much shorter pink corollas and the seeds with long white marginal hairs.

According to our molecular studies (Muñoz-Rodríguez et al. 2019) this species is sister to the whole Batatas Clade.

••• Clade B (species 234–338) comprises species mostly from Mexico and surrounding countries although it includes quite a few South American species. Like Clade A, there is no obvious morphological feature common to the whole clade. Species may be perennial or annual but there are no truly woody plants. Clade B divides into two large but morphologically ill-defined clades, Clade B1 (species 234–289) and Clade B2 (species 290–337). Within both B1 and B2, there are several small clades which are well defined morphologically. These are indicated in the text.

• Species 234–253 comprise the Pharbitis Clade but we have no molecular sequence data for *I. spruceana*, *I. calcicola*, *I. zacatecana*, *I. mairetii*, *I. invicta*, *I. lambii* and *I. laeta* so their inclusion must be regarded as provisional.

Annual or perennial herbs, stems twining, often robust. Leaves entire or 3–5-lobed, commonly variable within the same species. Flowers in pedunculate axillary cymes (occasionally solitary), often reduced to bracteolate heads; pedicels characteristically shorter than peduncles and sometimes very short; bracteoles usually prominent, persistent and occasionally (*I. neurocephala*) forming an involucre; sepals herbaceous, often elongate and accrescent in fruit, in some species prominently hirsute with stiff spreading hairs; corolla often large and showy, blue, pink or purple, rarely white; anthers included (except *I. jamaicensis* and *I. ampullacea*); stigma typically 3-lobed. The seeds are minutely pubescent or tomentellous but never pilose or lanate. The decisive character in the traditional circumscription of this group lies in the trilocular ovary and capsule, which is 6-seeded. However, this character is not present in all species included in this clade, such as *I. neurocephala* and *I. magnifolia* although molecular sequencing shows that they belong.

Species that probably belong to this clade can be separated by the following key.

**Table:** 

1	Corolla glabrous on th e exterior, even in bud	**2**
–	Corolla pubescent or pilose on the exterior, at least in bud	**10**
2	Corolla hypocrateriform,; stamens exserted (Jamaica)	**235. *I. jamaicensis***
–	Corolla funnel-shaped; stamens included in corolla tube	**3**
3	Flowers in compact bracteolate heads, the pedicels very short; sepals and bracteoles glabrous, puberulent or pubescent	**4**
–	Flowers in a lax inflorescence, the pedicels > 10 mm long; sepals and bracteoles pilose with long, patent hairs	**5**
4	Bracteoles up to 3.7 cm cm long; sepals 20–23 mm long; peduncles up to 17 cm long (Mexico)	**251. *I. invicta***
–	Bracteoles usually < 10 mm long, rarely more; sepals 11–20 mm; peduncles < 9 cm long (widespread)....	**234. *I. indica***
5	Corolla 7–9 cm long; leaves 5-lobed; flowers solitary (United States)	**243. *I. lindheimeri***
–	Corolla < 5 cm long; leaves entire or 3(–5)-lobed; flowers solitary, paired or in cymes	**6**
6	Stem and leaves glabrous; sepals finely acuminate to a mucronate apex (Brazil)	**240. *I. spruceana***
–	Stem and leaves hirsute; sepals varied but never finely acuminate to a mucronate apex.	**7**
7	Sepals ovate, cordate, c. twice as long as broad; perennial with napiform root; cymes 1(–2)-flowered	**242. *I. pubescens***
–	Sepals lanceolate or ovate, three or more times longer than broad, cuneate at base; annuals with fibrous rootstock; cymes usually with several flowers	**8**
8	Corolla pink (rarely white or blue); sepals oblong-lanceolate, obtuse or acute; leaves entire or 3–5-lobed	**238. *I. purpurea***
–	Corolla blue with a white tube (drying pink): sepals ovate with an elongate apex, notably accrescent in fruit; leaves usually 3-lobed	**9**
9	Corolla < 3.5 cm long; sepals < 2 cm long at anthesis, the tips recurving; peduncle very short	**237. *I. hederacea***
–	Corolla 4–4.5 cm long; sepals c. 3 cm long at anthesis, the tips erect; peduncles long or short	**236. *I. nil***
10	Corolla very large, 10–12 cm in length; leaves commonly lobed, discolorous	**253. *I. laeta***
–	Corolla < 9 cm long; leaves entire or lobed, not usually strongly discolorous	**11**
11	Bracteoles linear-filiform, < 6 mm long; leaves small, < 4 cm long	**12**
–	Bracteoles varied in shape, > 10 mm long, but if narrowly linear, leaves large, exceeding 5 cm long	**13**
12	Leaves entire, often with a lateral tooth; sepals green, bristly white-pilose	**239. *I. zacatecana***
–	Leaves 3(–5)-lobed without lateral teeth; sepals green with white margins, pubescent.......	**241. *I. calcicola***
13	Corolla, stem and sepals pilose with long spreading hairs	**14**
–	Corolla, stem and sepals glabrescent, puberulent or pubescent with appressed hairs	**16**
14	Pedicels very short or absent; flowers in bracteolate heads, the bracteoles persistent, conspicuous	**15**
–	Pedicels 3–10 mm long; bracteoles distant from flowers, deciduous and not very conspicuous; flowers solitary or up to 3 (Ecuador)	**245. *I. harlingii***
15	Outer bracteoles ovate to suborbicular, 7–20 × 7–24 mm, pale green with darker veins	**244. *I. neurocephala***
–	Outer bracteoles lanceolate to ovate, 20–25 × 5 mm, uniformly green	**246. *I. villifera***
16	Corolla shortly pubescent to sericeous in bud, ±glabrescent at anthesis; bracteoles linear; corolla large, 7–9 cm long (Bolivia and Peru)	**247. *I. magnifolia***
–	Corolla pubescent at anthesis; corolla < 8 cm long; bracteoles expanded, ovate to elliptic (Mexico and Central America)	**17**
17	Corolla white; stamens exserted	**248. *I. ampullacea***
–	Corolla pink; stamens included in the corolla tube	**18**
18	Sepals glabrous; stem and leaves pubescent; slender plant with wiry stems and corolla 7–8 cm long	**252. *I. lambii***
–	Sepals, stem and leaves retrorse-pilose or tomentose; stout perennial or liana with corolla 4.5–8 cm long	**19**
19	Bracteoles caducous; corolla up to 8 cm long, indumentum retrose-pilose	**249. *I. temascaltepecensis***
–	Bracteoles persistent; corolla 4–4.5 cm long, indumentum tomentose at least on sepals and abaxial leaf surface	**250. *I. mairetii***

### 234. Ipomoea indica

Taxon classificationPlantaeSolanalesConvolvulaceae

(Burm.) Merrill, Interpr. Herb. Amboin.445. 1917. (Merrill 1917: 445)

Convolvulus indicus Burm., Herb. Amboin. Actuar. [6]. 1755. (Burman 1755: 6). Type. Icon. Besler, Hort. Eyst. Aest. Ord. 8: t. 2 (1613), lectotype designated by Fosberg (1976).
Pharbitis indica (Burm.) R.C. Fang, Fl. Reipubl. Popularis Sin. 64: 105. 1979. (Fang 1979 and Huang: 105).
Convolvulus roseus Miller, Gard. Dict. ed. 8: Convolvulus n.18. 1768. (Miller 1768: Convolvulus n. 18). Type. JAMAICA. Houstons.n. (holotype BM000953173).
Convolvulus acuminatus Vahl, Symb. Bot. 3: 26. 1794. (Vahl 1794: 26). Type. [U.S. VIRGIN ISLANDS]. St Croix, *West*s.n. (?holotype C10009677).
Ipomoea acuminata (Vahl) Roem. & Schult., Syst. Veg. 4: 228. 1819. (Roemer and Schultes 1819: 228), nom. illeg., non Ipomoea acuminata Ruiz & Pav. (1799).
Pharbitis acuminata (Vahl) Choisy in A.P. de Candolle, Prodr. 9: 342. 1845. (Choisy 1845: 342).
Ipomoea vahliana House, Ann. New York. Acad. Sci. 18: 204. 1908. (House 1908b: 204). Type. Based on Convolvulus acuminatus Vahl
Ipomoea indica var. acuminata (Vahl) Fosberg, Bot. Not. 129: 38. 1976. (Fosberg 1976: 38).
Ipomoea congesta R. Br. (Brown 1810: 485). Type. AUSTRALIA. Queensland, Cape York Penisular, *Banks & Solander* (holotype BM001040638).
Convolvulus congestus (R. Br.) Spreng., Syst. Veg. 11: 601. 1825 [pub. 1824]. (Sprengel 1824: 601), nom. illeg., non Convolvulus congestus R. Br. (1814).
Pharbitis acuminata var. congesta (R.Br.) Choisy in A.P. de Candolle, Prodr. 9: 343. 1845. (Choisy 1845: 343).
Ipomoea mutabilis Ker-Gawl., Bot. Reg. 1: t. 39. 1815. (Ker-Gawler 1815: t. 39). Type. Cultivated from seed from Veracruz, Mexico (lectotype t. 39 in Bot. Reg. 1 (1815), designated here).
Convolvulus mutabilis (Ker Gawl.) Spreng., Syst. Veg. (ed. 15 bis) 1: 593. 1825 [pub. 1824]. (Sprengel 1824: 593).
Modesta mutabilis (Ker-Gawl.) Raf., Fl. Tellur. 4: 76. 1836 [pub. 1838]. (Rafinesque 1838a: 76).
Ipomoea cathartica Poir. in Lam., Encycl. Meth. Suppl. 4: 633. 1816. (Poiret 1814–17: 633). Type. [DOMINICAN REPUBLIC]. Santo Domingo, P. A. Poiteau (holotype FI?, isotype P-JUSS-6829).
Pharbitis cathartica (Poir.) Choisy in A.P. de Candolle, Prodr. 9: 342. 1845. (Choisy 1845: 342).
Convolvulus mollis Kunth, Nov. Gen. Sp. Pl. 3: 104. 1818 [pub.1819]. (Kunth 1819: 104), nom. illeg., non Convolvulus mollis Burm. f. (1768). Type. VENEZUELA. Sucre, Cumaná, Humboldt & Bonpland 233 (holotype P00670757).
Ipomoea mollis (Kunth) G. Don, Gen. Hist. 4: 275. 1838. (Don 1838: 275).
Pharbitis mollis (Kunth) Choisy in A.P. de Candolle, Prodr. 9: 342.1845. (Choisy 1845: 342).
Convolvulus bogotensis Kunth, Nov. Gen. Sp. Pl. 3: 104. 1818 [pub.1819]. (Kunth 1819: 104). Type. COLOMBIA. Santa Fe de Bogota, Humboldt & Bonplands.n. (holotype P00670756).
Ipomoea bogotensis (Kunth) G. Don, Gen. Hist. 4: 273. 1838. (Don 1838: 273).
Pharbitis bogotensis (Kunth) Choisy in A.P. de Candolle, Prodr. 9: 341. 1845. (Choisy 1845: 341).
Ipomoea lilacina Schrank, Denkschr. Bot. Ges. Regensb. 2: 31. 1822. (Schrank 1822: 31). Type. BRAZIL. [Minas Merais, sabaria], *C.F. P. Martius*s.n. (lectotype M0184854, designated here).
Convolvulus portoricensis Spreng., Syst. Veg. 1: 595. 1825 [pub. 1824]. (Sprengel 1824: 595. Type. PUERTO RICO. No type cited.
Ipomoea portoricensis G. Don, Gen. Hist. 4: 278. 1838. (Don 1838: 278).
Ipomoea amoena Blume, Bijdr. Ned. Ind. 718. 1825. (Blume 1825–26: 718). Type. INDONESIA. “In Moluccanis insulis, nunc etiam in horto Bot. Buitenzorgii culta”. (Wherabouts unknown, ?BOG).
Convolvulus pudibundus Lindl., Bot. Reg. 12: t. 999. 1826. (Lindley 1826: t. 999). Type. Cultivated plant from St. Vincent (lectotype t. 999 in Bot. Reg. 12 (1826), designated here).
Ipomoea pudibunda (Lindley) G. Don, Gen. Hist. 4: 276. 1838. (Don 1838: 276).
Ipomoea punctata Macfad., Bot. Misc. 2: 116. 1830 (Macfadyen 1830: 116). Type. JAMAICA. Aylmer’s Estate Estate. (type not found at K).
Ipomoea cataractae Endl., Prodr. Fl. Norfolk. 53. 1833. (Endlicher 1833: 53). Type. NORFOLK ISLAND. Caskade Bay, *F.L. Bauer*s.n. (lectotype W0050656, designated here; isolectotypes W0050657, K000830895).
Pharbitis insularis Choisy, Mém. Soc. Phys., Genève 6: 57 [439]. 1834. (Choisy 1834: 57 [439]. Type. POLYNESIA. Friendly Islands, *D. Nelson* (lectotype BM001209580, designated here).
Ipomoea insularis (Choisy) Steud., Nomencl. Bot. 1: 817. 1840. (Steudel 1840: 817).
Ipomoea learii Knight ex Paxton, Paxton’s Mag. Bot. 6: 267. 1839. (Paxton 1839: 267). Type. Cultivated plant from Sri Lanka sent by Lear (lectotype K000612914, designated here).
Pharbitis learii (Knight ex Paxton) Lindl., Edwards’s Bot. Reg. 27: t. 56. 1841. (Lindley 1841: t. 56).
Pharbitis medians Choisy in A.P. de Candolle, Prodr. 9: 343.1845. (Choisy 1845: 343). Type. REUNION [Bourbon], *Bory St Vincent*s.n. (lectotype G00134749, designated here).
Pharbitis rosea Choisy in A.P. de Candolle, Prodr. 9: 342. 1845. (Choisy 1845: 342). Type. BRAZIL. Minas Gerais, Congonhas de Sabará, *Martius* 1228 (probable holotype M0184855).
Pharbitis dealbata M. Martens & Galeotti, Bull. Acad. Roy. Sci. Bruxelles 12: 272. 1845. (Martens and Galeotti 1845: 272). Type. MEXICO. [Veracruz], Dans les bois de Mirador et de Zacupan”, *H. Galeotti* 1352 (holotype BR, not found, isotypes G00227843, K000612766).
Ipomoea dealbata (M. Martens & Galeotti) Hemsl., Biol. Cent.-Amer., Bot. 2(11): 386. 1882. (Hemsley 1882: 386).
Pharbitis calycosa A. Rich., Hist. Fis. Cuba 3: 128. 1850. (Sagra 1850: 128). Type. CUBA. (P, not found).
Pharbitis hispida var. imberbis Beurl., Kongl. Vetensk. Acad. Handl. 40: 138. 1854 [pub. 1856]. (Beurling 1856: 138). Type. PANAMA. Colón, Porto Bello, J.I. Bilbergs.n. (isotype S-07-4787).
Pharbitis grandiflora Beurl., Kongl. Vetensk. Acad. Handl. 40: 139. 1854 [pub. 1856]. (Beurling 1856: 139). Type. PANAMA. Colón, Porto Bello, J.I. Bilbergs.n. (isotype S-R-7905).
Ipomoea jamaicensis var. glabrata Griseb. Fl. Brit. W.I. 474. 1864 [pub. 1862]. (Grisebach 1862b: 474). Type. JAMAICA. Mcfaddens.n. (lectotype K000612713 with Grisebach’s annotation, designated here).
Ipomoea jamaicensis var. glabrata Meisn. in Martius et al., Fl. Brasil. 7: 226. 1869. (Meisner 1869: 226), nom. illeg., non Ipomoea jamaicensis var. glabrataGrisebach (1862b). Type. BRAZIL. MEXICO, Tampico, Berlandier 29 (lectotype BM001209638, designated here).
Ipomoea jamaicensis var. intermedia Meisn. in Martius et al., Fl. Brasil. 7: 226. 1869. (Meisner 1869: 226). Type. BRAZIL. São Paulo, Ypanema, *C.F.P. Martius*s.n. (lectotype M0184878, designated here).
Ipomoea jamaicensis var. sericea Meisn. in Martius et al., Fl. Brasil. 7: 226. 1869. (Meisner 1869: 226). Type. BRAZIL. Minas Gerais, Congonhas de Sabará, *C.F.P. Martius* 1228 (lectotype M0184855, designated here).
Ipomoea jamaicensis forma triloba Arechav., An. Mus. Nac. Montevideo 7: 194. 1909. (Arechaveleta y Balpardo 1909: 194). Type. URUGUAY. Not specified, (?MVM, n.v.).
Ipomoea halierca I.M. Johnst., Proc. Calif. Acad. Sci. ser. 4, 20: 85. 1921. (Johnston 1921: 85). Type. MEXICO. Clarion Island [Revillagigedo], *H.L. Mason* 1553 (holotype CAS0003013, isotypes CAS, F, GH, K, MICH, US).
Ipomoea congesta var. brevipedunculata Hochr., Candollea 5: 185. 1934. (Hochreutiner 1934: 185). Type. INDONESIA. Java, Tjinjiroean, *Hochreutiner* 1635 (G, n.v.).
Ipomoea indica forma albiflora Stone, Micronesica 2: 139. 1966. (Stone 1966: 139). Type. GUAM. Harmon Village, *Stone* 4729 (GUAM, n.v.).

#### Type.

Based on *Convolvulus indicus* Burm.

#### Description.

Twining perennial herb, stems pubescent. Leaves petiolate, 5–15 × 5–15 cm, ovate or, commonly, shallowly 3-lobed, both forms sometimes on the same plant, apex acuminate and shortly mucronate, base cordate with rounded auricles, adaxially pubescent, abaxially paler, pubescent to grey-tomentose; petioles 2.5–10 cm. Inflorescence of axillary, pedunculate clusters, sometimes reduced to single flowers; peduncles 5–9 cm, pubescent; bracteoles ± persistent, pubescent, usually narrowly linear-lanceolate, acuminate, 4–10 × 0.5–1 mm but sometimes oblong-elliptic, foliose and shortly petiolate, c. 15–30 × 4–15 mm; secondary peduncles 2–3 mm; pedicels very short, 2–7 mm; sepals subequal, 13–20 × 3–5 mm, narrowly ovate, finely acuminate, pubescent, often somewhat spreading at muturity; corolla 5–6 cm long, funnel-shaped, deep blue with violet midpetaline bands, drying pink, glabrous, limb unlobed, 4–5 cm diam. Capsules subglobose, 8–10 mm diam., glabrous, 6-seeded; seeds black, 4–5 mm long, appearing glabrous but minutely tomentellous.

#### Illustration.

**Figure 121.**
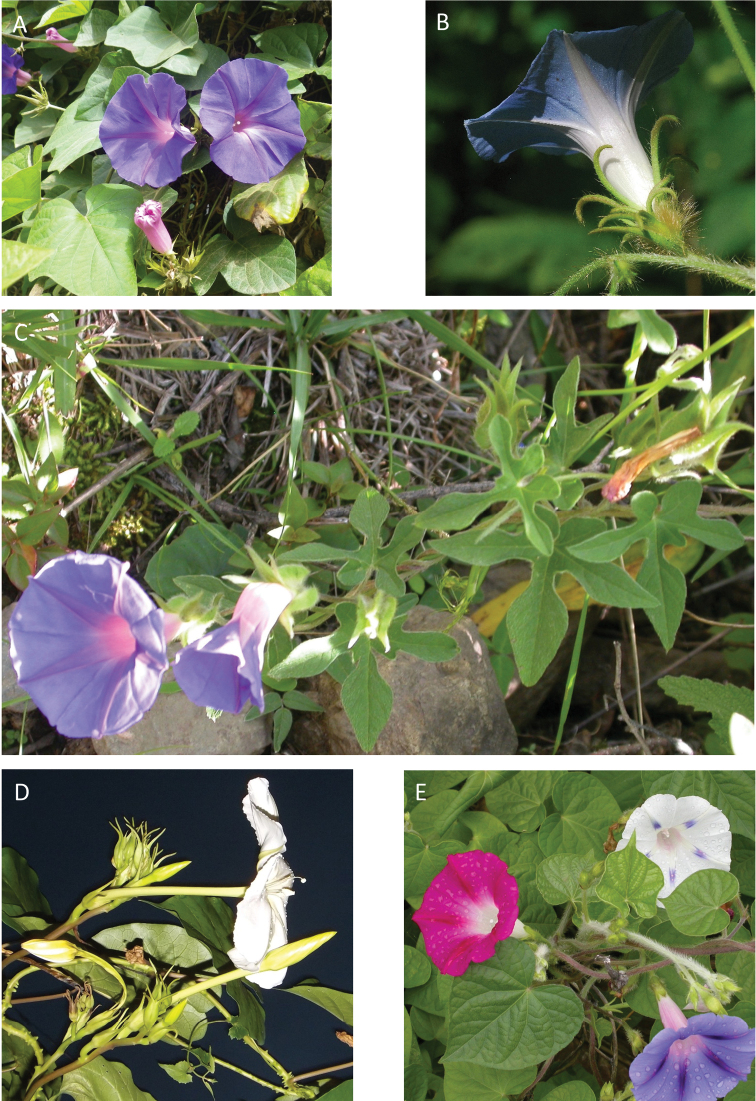
Photographs of *Ipomoea* species. **A***I. indica***B***I. hederacea***C***I. pubescens***D***I. alba***E***I. purpurea* (colour variants). **A** John Pink **B** Steven Turner **C** Darwin Initiative Project 162/11/010 **D** Tom Carruthers **E** Darwin Initiative Project 162/11/010.

Figure 121A; O’Donell (1959b: 135); Acevedo-Rodríguez (2005: 170); Austin (1998: 404); Bosser and Heine (2000: 46); Deroin (2001: 203).

#### Distribution.

Widely cultivated as an ornamental and frequently naturalised throughout tropical and subtropical countries of the world. Of New World, perhaps Mexican, origin, it is not native in India despite its name. Most of the records cited below are of naturalised, wild populations, but some records may have been of cultivated plants. In some areas it is very common, the Caribbean islands, and Misiones Department in Argentina, for example, whereas in others it is surprisingly uncommon as in Bolivia and Ecuador. There is some evidence that it is more common on islands and is certainly on most islands in tropical America. It probably spreads in humid areas by cuttings and broken off shoots as a result of cattle grazing as seeds are not commonly formed.

**URUGUAY.***W.G. Herter* 272 (LIL, MO, S, SI); *M. Berro* 5009 (K).

**ARGENTINA. Buenos Aires**: *C.M. Hicken* 14477 (K, SI). **Chaco**: *T. Meyer* 519 (LIL). **Corrientes**: *T.M. Petersen* 2652 (C, K, S); Corrientes, *A. Krapovickas* 41921 (CTES, FCQ, K). **Entre Ríos**: *T. Meyer* 10213 (LIL). **Misiones**: *E.L. Ekman* 1438 (S); Iguazu, *R. Vanni et al.* 2872 (CTES, K). **Tucumán**: *A. Lourteig* 990 (LIL).

**PARAGUAY.***T. Rojas* 13279 (LIL, S). **Alto Paraná**: Est. San Pedro, *E. Zardini & L. Guerrero* 42736 (PY). **Central**: San Lorenzo, *N. Soria* 727 (FCQ); **Guairá**: Reserva Ybytyrusu, *M. Vera et al.* 2804 (FCQ); Villarica, *P. Jörgensen* 4296 (MO, S). **Itapúa**: Cordillera San Rafael, *F. González* 312 (FCQ); Yacyretá, *J. de Egea & T. Hostettler* 228 (BM, FCQ). **Misiones**: Isla Yacyretá, *S. Keel et al.* 1374 (FCQ). **Ñeembucú**: Pilar, *C. Vogt & A. Contreras* 718 (FCQ). **Paraguarí**: 1 km NW of Sapacai, *J.R.I. Wood et al.* 28153 (FCQ); Macizo Acahay, *E. Zardini & A. Aguayo* 8397 (PY). **San Pedro**: *A.L. Woolston* 1228 (K, S).

**BRAZIL. Bahia**: *Blanchet* 694 (BM); *J.L. Hage et al.* 1369 (CEPEC, K); *L.P. de Queiroz et al.* 15916 (HUEFS, OXF). **Dist. Fed.**: *E.P. Heringer* 13063 (NY). **Goiás**: *W.R. Anderson et al.* 7777 (NY). **Minas Gerais**: *C.W. Mosén* 1912 (S), *Widgen* s.n. (K, S). **Paraná**: *P. Dusen* 3945 (NY, S). **Rio de Janeiro**: *L. Torres & M. Vianna* 110 (NY). **Rio Grande do Sul**: *Malme* 488 (S). **Santa Catarina**: *L.B. Smith et al.* 7252 (K, S, US). **São Paulo**: *M. Sakane* 683 (NY, SP).

**GUYANA.***Mazarini Forest Station* 425 (K).

**CHILE.***Pahlman* s.n. 2/2/1912 (S).

**BOLIVIA. Cochabamba**: Cercado, *R. Steinbach* 69 (F, LPB, NY, MICH, MO, S, US). **Santa Cruz**: Chiquitos: Santiago, *J.R.I. Wood* 28138 (LPB, OXF, USZ); Florida, Samaipata, *J.R.I. Wood* 28108 (LPB, OXF, USZ)

**PERU. Amazonas**: *R. Ferreyra* 7096 (K). **Cajamarca**: San Ignacio, Chulalapa, *J. Campos & M. Vásquez* 2514 (MO, OXF). **Lima**: sea cliffs, *F.R. Fosberg et al.* 28235 (K); Canta, *G. Vilcapoma* 7649 (USM). **Pasco**: *D.N. Smith* 2746 (USM). **San Martín**: Alto Río Huallaga, *Llewelyn Williams* 6804 (F).

**ECUADOR. Galápagos**: *A.M. Stewart* 323 (MO).

**COLOMBIA. Bolívar**: *E.P. Killip & A.C. Smith* 14132 (GH). **Boyacá**: *A.E. Lawrance* 193 (BM, K, MO, S). **Cundinamarca**: *J. Triana* 3803 (BM). **Norte de Santander**: Ocaña, *L. Schlim* 210 (K). **Quindío**: *E. André* 2058 (K). **Valle**: *F.W. Pennell & E.P. Killip* 8497 (K).

**VENEZUELA.***J. Steyermark* 87777 (S). **Miranda**: *A. Carmona* 14 (MO). **Sucre**: *J. Steyermark & R. Liesner* 120713 (MO).

**PANAMA.***B.C. Seemann* 172 (BM, K); Chagres, *A. Fendler* 244 (K)

**COSTA RICA.** Guacimo, *A. Tonduz* 14738 (BM, K); Alajuela, Valverde Vega, *M. Chavarria* 592 (K, MO); San José, *M. Chavarria* 669 (K, MO); Limón Prov., Cahuita, *P. Wilkin* 473 (BM); S. of Limón, *P. Wilkin* 481 (BM).

**NICARAGUA.** Estelí, El Portillo, *L.O. Williams & A. Molina* 42320 (BM, F).

**HONDURAS.** Siguatepeque, *T.C. Yuncker et al.*6041 (K); Colón, *J. Saunders* 944 (BM); Copán-Sta Rita, *A. & A.R. Molina* 24671 (BM, F)

**BELIZE.***P. Gentle* 148 (S); Honey Camp, *C.L. Lundell* 660 (K); New Town, *W.A. Schipp* 830 (BM, K); Orange Walk, *C. Whitefoord* 8149 (BM).

**GUATEMALA.** Petén, P.N. Tikal, *R. Tun Ortíz* 167 (BM, F), 257 (F, S), 259 (BM, F).

**MEXICO. Campeche**: E. de Constitución, *E.F. & H. Cabrera* 13521 (BM, MEXU). **Chiapas**: Ocosingo, *E. Martínez et al.* 25219 (K). **Durango**: El Mezquital, *A. García* 1053 (IEB). **Guanajuato**: Santa Catarina, *E. Carranza & E. Pérez* 5108 (IEB). **Guerrero**: *G.B. Hinton* 10432 (K). **Hidalgo**: *S. Montes et al.* 47 (IEB). **Michoacán**: Nuevo Urecho, *E. Vargas* 37 (IEB); Coalcomán, *E. Carranza & I. Silva* 6809 (IEB). **Querétaro**: Pinal de Amoles, *E. Pérez* 4466 (IEB). **Quintana Roo**: Tulum, *O. Téllez* & *E. Cabrera* 3197 (BM); Chumpon, *E. Cabrera* 601 (BM, MEXU). **Sinaloa**: Clarion Island, *A.W. Anthony* 403 (E, K, S). **Tabasco**: Tenosique, *S. Zamudio* 755 (IEB). **Tamaulipas**: *E. Palmer* 201 (K); *Berlandier* 29 (BM). **Veracruz**: Valle de Córdoba, *E. Bourgeau* 1737 (K); ibid., *E. Kerber* 32 (BM, K); Misantla, *C.A. Purpus* 5955 (BM). **Yucatán**: Cozumel Island, *G.F. Gaumer* 119 (K); Kantunilkin, *E.F. & H. Cabrera* 14287 (IEB).

**UNITED STATES. California**: *R. Moran* 13134 (BM); *R. Spjut* 16034 (BM). **Florida**: *A.H. Curtiss* 2168 (BM, K), 5843 (K); *H. Moldenke* 388 (K, S), 772 (K); *Rugel* 197 (BM).

**BERMUDA.***O. Degener* 1234 (NY); *A.B. Rendle* 790 (BM).

**BAHAMAS.** Hog Island. *H.F.A. von Eggers* 4149 (BM, K); New Providence, Nassau, *A.E. Wright* 146 (K); Anguilla Isles, *P. Wilson* 8019 (K); Eleuthera, *W.H. Lewis* 7243 (NY); Grand Bahama, *W.H. Lewis* 7106 (NY).

**CUBA.***C. Wright* 3088 (BM, K, NY); *M. López Figuieras* 804 (HAC, HAJB). **Guantánamo**: Baracoa, *E.L. Ekman* 3984 (NY, S). **Cienfuegos**: *J.G. Jack* 631) (A). **La Habana**: *Bro. León* 20619 (NY). **Las Tunas**: Manatí, *Bro. León* 16782 (HAC, NY). **Matanzos**: *A.H. Curtis* 575 (BM, K). **Pinar del Río**: *N.L. Britton* 7565 (NY). **Santiago de Cuba**: *F. Millspaugh* 1079 (NY). **Villa Clara**: *Bro. Fernando*. 357 (NY).

**CAYMAN ISLANDS.***D.R. Stoddart* 7040 (BM); *G.R. Proctor* 35112 (BM); *W. Fawcett* [5/1888] (K).

**JAMAICA.***C.D. Adams* 8575 (BM); *Sangster* 537 (BM); *W. Stearn* 152 (BM), 262 (BM); *W. Purdie* s.n. (K); *W. Harris & N.L. Britton* 10785 (K, NY).

**HAITI.***L.R. Holdridge* 1924 (BM); *E.L. Ekman* H9255 (S); *T.A. Zanoni et al.* 34717 (NY).

**DOMINICAN REPUBLIC.***R.A. & E.S. Howard* 9703 (BM); *E.L. Ekman* H16116 (S); *M.M. Mejía & T. Zanoni* 6032 (NY).

**PUERTO RICO.***H.F.A. von Eggers* 618 (K); *P. Sintenis* 963 (K); *C. Taylor* 10065 (NY).

**LESSER ANTILLES. U.S. Virgin Islands**: St Thomas: *H.F.A. von Eggers* 1138 (K); St John: *P. Acevedo-Rodríguez & A. Siacca* 4666 (NY). **U.K. Virgin Islands**: Tortula: *Fistlock* 151 (K). **St Kitts**: fide Powell (1979). **Montserrat**: *J.A. Shafer* 526 (NY). **Guadeloupe**: *A. Duss* 4419 (NY). **Dominica**: *C.A. Shillingford 159* (BM). **St. Lucia**: *I. Vélez* 3309 (K). **St Vincent**: *H.H. & G.W. Smith* 1169 (K, NY). **Barbados**: *C.C. Skeete* 6 (K).

**TRINIDAD.***W. Johnson* 1079 (BM).

**HAWAII.***C.R. Annable & D. Atha* 3091 (NY); *G.W. Barclay* 1333 (BM); *Faurie* 1039 (BM).

#### Typification.

The type of *Ipomoea punctata* has not been found. There are two McFadyen specimens at Kew but neither are labelled *Ipomoea punctata* nor is Aylmer Estate cited on the labels.

Meisner cited various syntypes of Ipomoea jamaicensis var. glabrata but many of these have not been traced and may have been destroyed in Berlin in 1943. We have selected *Berlandier* 29 (BM) as lectotype as it conforms with the protologue.

#### Note.

This species might be confused with *Ipomoea purpurea* and *I. magnifolia* but it is usually easily distinguished by the leaves grey-pubescent or tomentose beneath and the clustered flowers with very persistent bracteoles. However, it is extremely variable so leaves are sometimes glabrous, lobed or entire, bracteoles may be reduced and flowers are occasionally solitary varying in colour from blue to deep violet with prominent darker midpetaline bands. Molecular studies suggest it is not monophyletic so more than one species may eventually be recognised.

### 235. Ipomoea jamaicensis

Taxon classificationPlantaeSolanalesConvolvulaceae

G. Don, Gen. Hist. 4: 278. 1838. (Don 1838: 278)

Convolvulus jamaicensis Spreng., Syst. Veg. 1: 595. 1825 [pub. 1824]. (Sprengel 1824: 595), nom. illeg., Convolvulus jamaicensis Jacq. (1768). Type. JAMAICA. No type cited, neotype icon in Sloane, Voy. Jamaica 1: t. 98, f. 2 (1707), designated here.
Pharbitis jamaicensis (G. Don) Gibert, Enum. Pl. 28. 1873 (Gibert 1873: 28).
Convolvulus tomentosus L., Sp. Pl. 1: 156. 1753. Type. Icon in Sloane, Voy. Jamaica 1: t. 98, f. 2 (1707), designated by Staples and Jarvis (2006: 1022).
Pharbitis tomentosa (L.) Choisy in A.P. de Candolle, Prodr. 9: 342.1845. (Choisy 1845: 342).
Ipomoea tomentosa (L.) Urb., Symb. Antill. 3 (2): 344. 1902. (Urban 1902–3: 344), nom. illeg., non Ipomoea tomentosaChoisy (1838).

#### Type.

Based on *Convolvulus jamaicensis* Spreng.

#### Description.

Liana to 6 m, stems woody, pubescent when young. Leaves petiolate, 3.5–10 × 3.5–11 cm, ovate in outline, 3-lobed to about half way, shortly acuminate, base cordate with rounded auricles, adaxially pubescent, abaxially grey-tomentose, rarely glabrous on both surfaces; petioles 2.5–5 cm, very thinly to densely pubescent. Inflorescence of few-flowered pedunculate cymes; peduncles 1.5–8.5 cm; bracteoles 3–12 mm, ovate, acuminate, pale green, caducous; pedicels 3–12 mm, noticeably more slender and more pubescent than peduncles; sepals subequal but inner slightly narrower, 10–14 × 2–4 mm at anthesis, oblong-lanceolate, shortly acuminate to caudate, pubescent, herbaceous, somewhat accrescent in fruit reaching 15 × 8 mm; corolla 5–7 cm long, glabrous, salver-shaped, the tube 4–5 cm long, subcylindrical and only slightly widened, dark purple, limb 4–5 cm diam., pentagonal, crimson to magenta, stamens exserted; stigma 3-lobed, exserted. Capsules 10 × 9 mm, subglobose, glabrous; seeds 5 × 4 mm, blackish, densely covered in short erect hairs.

#### Illustration.

Figure 122.

**Figure 122.**
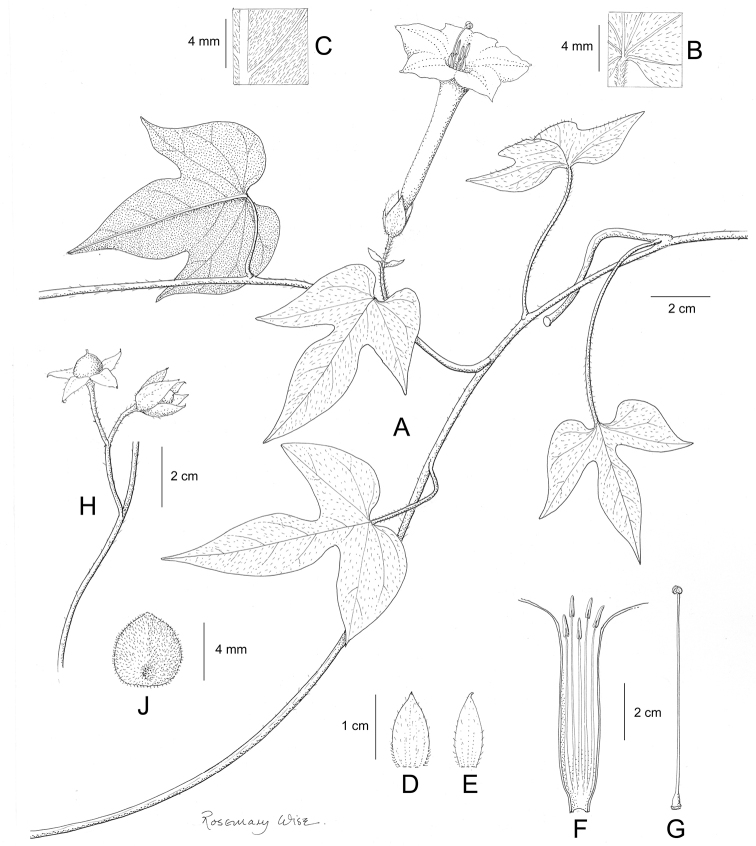
*Ipomoea jamaicensis*. **A** habit **B** adaxial leaf surface **C** abaxial leaf surface **D** outer sepal **E** inner sepal **F** corolla opened out to show stamens **G** ovary and style **H** fruiting inflorescence with capsule **J** seed. Drawn by Rosemary Wise **A–C** from *Proctor* 17443; **D–J** from *Stearn* 208.

#### Distribution.

Endemic to Jamaica where it appears to be common in thickets, scrub and in forest relics on limestone hills.

**JAMAICA.***March* s.n. (K); Manchester, *C.D. Adams* 8516 (BM); St Catherine, *G.R. Proctor* 7253, ibid., 8298, ibid., 17443 (BM); Great Goat Island, *W. Harris* 9212 (BM); St Andrew, *G.R. Proctor* 18336 (BM); Clarendon, *W. Stearn* 208 (BM).

#### Note.

This species appears to have evolved as an adaptation of *Ipomoea indica* for humming bird pollination. It differs in the exserted stamens from a salver-shaped corolla as well as in the leaves, which are always three lobed.

### 236. Ipomoea nil

Taxon classificationPlantaeSolanalesConvolvulaceae

(L.) Roth, Catal. Bot. 1: 36. 1797. (Roth 1797: 36)

Convolvulus nil L., Sp. Pl., ed. 2: 219. 1762. (Linnaeus 1762: 219). Type. Icon in Dillenius, Hort. Eltham. 1: 96, t. 80, f. 91 (1732), designated by Verdcourt (1957b: 232–233).
Pharbitis nil (L.) Choisy, Mém. Soc. Phys., Genève 6: 439 [57]. 1834. (Choisy 1834: 439[57]).
Convolvulus hederaceus L., Sp. Pl. 1: 154. 1753. (Linnaeus 1753: 154), non Ipomoea hederacea Jacq. (1787). Type. Herb. Burser XVII: 6 (UPS), designated by Staples and Jarvis (2006: 1020).
Convolvulus hederaceus var. zeta L. Sp. Pl. 154. 1753. Type. Icon in Dillenius, Hort. Eltham. 1: 96, t. 80., f. 91 (lectotype designated by Shinners 1965).
Ipomoea scabra Forssk., Fl. Aegypt-Arab. 44. 1775. (Forsskal 1775: 44). Type. YEMEN. Hadie, [Hadiyah], *P. Forsskal*s.n. (holotype C10002425).
Pharbitis forskoelii G. Don, Gen. Hist. 4: 263. 1838. (Don 1838: 263). Type. Based on Ipomoea scabra Forssk.
Convolvulus dillenii Desr., Encycl. 3: 544. 1789 [pub. 1792]. (Desrousseaux 1792 544). Type. Based on icon in Dillenius, Hort. Eltham. 1: 97, t. 81, f. 93 (1732).
Ipomoea dillenii (Desr.) Roem. & Schult., Syst. Veg. 4: 227. 1819. (Roemer and Schultes 1819: 227).
Pharbitis nil var. abbreviata Choisy in A.P. de Candolle, Prodr. 9: 343. 1845. (Choisy 1845: 343). Type. Based on Convolvulus dillenii Desr.
Ipomoea bicolor Lam., Tabl. Encycl. 1: 465. 1793. (Lamarck 1793: 465). Type. SOUTH AFRICA. Cape of Good Hope, *P. Sonnerat* (holotype P-LAM00357501).
Ipomoea cuspidata Ruiz & Pav., Fl. Peruv. 2: 11. 1799. (Ruiz and Pavón 1799: 11). Type. PERU. Ruiz & Pavón, sine data (lectotype MA817964, designated here).
Convolvulus peruvianus Syst. Veg. 1: 593. 1824 [pub. 1825]. (Sprengel 1824: 593). Type. Based on Ipomoea cuspidata Ruiz & Pav.
Pharbitis cuspidata (Ruiz & Pav.), D. Don, Gen. Hist. 4: 270. 1838. (Don 1838: 270).
Ipomoea longicuspis Meisn. in Martius et al., Fl. Brasil. 7: 227, (Meisner 1869: 227), nom. illeg. superfl. for I. cuspidata.
Convolvuloides triloba Moench, Methodus 452. 1794 (Moench 1794: 452), nom. rej.
Ipomoea caerulea Ker-Gawl., Bot. Reg. 4: t. 276. 1818. (Ker-Gawler 1818b 4: t. 276). Type. Icon, t. 276 in Bot. Reg. (1818), lectotype, designated here.
Ipomoea caerulescens Roxb., Fl. Ind., ed. 2, 2: 90. 1824. (Roxburgh 1824: 90). Type. INDIA. Lectotype *Roxburgh* Icon no. 1951 (K), designated here.
Ipomoea hederacea var. integrifolia C.B. Clarke, Fl. Brit. India 4: 200. 1883. (Clarke 1883: 200). Type. Based on Ipomoea caerulescens Roxb.
Pharbitis nil var. integrifolia Choisy in A.P. de Candolle, Prodr. 9: 343. 1845. (Choisy 1845: 343). Type. Based on Ipomoea caerulescens Roxb.
Ipomoea caerulea J. Koenig ex Roxb., Fl. Ind., ed. 2, 2: 91. 1824. Type. INDIA. (lectotype Koenigs.n. BM001209637, designated here).
Pharbitis caerulea (J. Koenig ex Roxb.) Wall. ex O’Shaugnessy, Beng. Disp. 505. 1842. (O’Shaungnessy 1842: 505)
Ipomoea setosa Blume, Bijdr. Fl. Ind. Ned. 714. 1825. (Blume 1825–26: 714), nom. illeg., non Ipomoea setosa Ker-Gawl. (1818e). Type. INDONESIA. Java, in scrub in the mountains, (possible type L0931182).
Ipomoea nil var. setosa (Blume) Boerl., Handl. Fl. Ned. Ind. 2: 511.1899. (Boerlage 1899: 511).
Ipomoea trichocalyx Steud., Nomencl. Bot. 1: 819. 1840. (Steudel 1840: 819), nom. illeg., non Ipomoea trichocalyx G. Don (1838). Type. Based on Ipomoea setosa Blume
Convolvulus tomentosus Vellozo, Fl. Flumin. 74. 1825 [pub. 1827]. (Vellozo 1829: 74). Type. BRAZIL. (lectotype, original parchment plate of Flora Fluminensis in the manuscript section of the Biblioteca Nacional, Rio de Janeiro [cat. no.: mss1198651-065], designated here; later published in Vellozo, Fl. Flum. Icon. 2: t. 65 1827. [pub. 1831], the published plate designated as lectotype by Austin 1986: 356).
Pharbitis purshii G. Don, Gen. Hist. 4: 263. 1838. (Don 1838: 263). Type. Various types cited.
Pharbitis speciosa Choisy in A.P. de Candolle, Prodr. 9: 343. 1845. (Choisy 1845: 343).Type. MEXICO (G, not found).
Ipomoea longicuspis var. brevipes Meisn. in Martius et al., Fl. Brasil. 7: 227. 1869. (Meisner 1869: 227). Type. Based on Pharbitis speciosa Choisy
Pharbitis limbata Lindl., J. Hort. Soc. 5: 33. (Lindley 1850a: 33). Type. Cultivated plant grown with seed from Java.
Pharbitis nil var. limbata (Lindl.) Hook. f., Bot. Mag. t. 5720. 1868. (Hooker, JD 1868: t.5720). Type. Based on Pharbitis limbata Lindl., but wrongly referred to as Ipomoea albomarginata in the text.
Ipomoea githaginea A. Rich., Tent. Fl. Abyss. 2: 65. 1851. (Richard 1851: 65). Type. ETHIOPIA. R. Takkaze, *Schimper* 784 (isotype K).
Ipomoea hederacea var. himalaica C.B. Clarke, Fl. Brit. India 4: 200. 1883. (Clarke 1883: 200). Type. INDIA. Sikkim, Lingcham, *C.B. Clarke* 25486 (syntype K001081728).
Ipomoea vaniotiana H. Lévl., Repert. Spec. Nov. Regni Veg. 9: 453. 1911. (Léveillé 1911: 453). Type. CHINA. Hong Kong, bois des Douglas, *E. Bodinier* 1465 (holotype E, not found).
Ipomoea hederacea auct. mult., non Jacq.

#### Type.

Based on *Convolvulus nil* L.

#### Description.

Trailing or twining herb, stems roughly pilose. Leaves petiolate, 3–12 × 3–14 cm, 3-lobed, the lobes typically ovate, abruptly narrowed to an acute or very shortly acuminate apex, base cordate, thinly to densely pubescent on both surfaces, paler beneath; petioles 1.5–7 cm. Inflorescence of pedunculate axillary compact cymes, sometimes reduced to 1–2 flowers; peduncles 0.5–18 cm, usually pilose; bracteoles 3–7 mm, filiform, relatively persistent; secondary peduncles 3–8 mm; pedicels 3–10 mm; sepals 15–32 mm, lanceolate tapering into a long linear point, densely pilose with bulbous-based hairs, especially near the base; corolla 3.5–4.5 cm long, funnel-shaped, glabrous, tube white, limb blue, drying pink, 3–4 cm diam., unlobed. Capsules 7–10 × 6 mm, subglobose, glabrous, style slender, persistent; seeds puberulent.

#### Illustration.

Figure 5D; O’Donell (1959b: 199); Acevedo-Rodríguez (2005: 172); Bosser and Heine (2000: 49); Deroin (2001: 221).

#### Distribution.

A pantropical weed of disturbed bushy places often near settlements. It is essentially a lowland species usually found below about 1000 m, although it is occasionally found growing to at least 2000 m. Although generally common, records are sparse from some areas, the northern Andes for example, and its presence is by no means universal.

**ARGENTINA. Catamarca**: *A. Hunzinker* 18763 (CORD). **Chaco**: *A.G. Schulz* 7485 (MO). **Córdoba**: *A. Krapovickas & R. Vanni* 41269 (CTES, K); *P. Lorentz* 63 (BM). **Corrientes**: *T.M. Petersen* 9670 (C, NY, S). **Formosa**: *P. Jorgensen* 3368 (MO). **La Rioja**: *A.T. Hunziker & J. A. Caro* 13510 (CORD). **Misiones**: *M. Múlgura de Romero* 2933 (CTES, MO, SI). **Santiago del Estero**: *S. Venturi* 10281 (BM, LIL, MO). **Tucumán**: *S. Venturi* 10437 (BM, LIL, MO).

**PARAGUAY. Alto Paraná**: Tatí Yupí, *Itaipu Binacional* 56 (PY). **Caazapá**: Tapyta, *M. Vera et al.* 251 (BM, CTES, FCQ, MO). **Boquerón**: Pirizal, *L. Britos* 15 (FCQ). **Canindeyú**: Ygatimi, *M. Vera et al.* 964 (FCQ). **Central**: Luque, *L. Pérez et al.* 64 (MO, PY). **Cordillera**: Caacupé, *E. Lurvey* 334 (PY). **Guairá**: Villarica-Paraguarí, *J. de Egea et al.* 1318 (FCQ). **Itapúa**: Alto Vera, *F. Mereles* 9809 (FCQ). **Paraguarí**: Cerro Palacios, *E. Zardini* 4612 (FCQ, MO). **Presidente Hayes**: Est. 11 de junio, *J. de Egea et al.* 946 (BM, FCQ). **San Pedro**: *A.L. Woolston* 656 (K, S); *Jorgensen* 4036 (S); Cruce Liberación, *J. de Egea et al*. 1260 (FCQ).

**BRAZIL. Alagoas**: *M.N. Rodrigues* 1163 (RB). **Amazonas**: *E. Ule* 8283 (NY). **Bahia**: *R.M. Harley et al.* 21812 (K, NY). **Ceará**: *J.P. Souza et al.* 11124 (RB). **Dist. Fed.**: *H.S. Irwin* 12184 (NY). **Espirito Santo**: *R.C. Forzza* 5550 (RB). **Mato Grosso**: *Malme* s.n. [22 April 1903] (S); *D. Philcox & A. Ferreira* 4201 (K, NY). **Mato Grosso do Sul**: *S. Moore* 848 (BM, NY). **Minas Gerais**: *G. Davidse et al.* 11423 (MO, NY). **Pará**: *R. Spruce* s.n. (BM, NY). **Paraíba**: *M.F. Agra* 748 (RB). Paraná: *A. Moreira* s.n. (RB). **Pernambuco**: *A.M. Miranda* 3449 (RB). **Rio de Janeiro**: *G. Gardner* 79 (BM). **Rondônia**: *L. Texeira* 517 (MO, NY, RB). **Santa Catarina**: *P. João* 183 (RB). **São Paulo**: *H. Filho* 65 (RB).

**FRENCH GUIANA.***R. Benoist* fide Lemée (1952).

**SURINAM.***Berthould-Coulon* 510 (BM); *O. Poncy* 1188 (P).

**GUYANA.***K.R. Robertson & D.F. Austin* 304 (MO).

**BOLIVIA. Beni**: Ballivián, Reyes, *M. Cardenas* 5389 (LIL). **Chuquisaca**: Oropeza, Río Chico, *J.R.I. Wood* 9604 (K, LPB); Zudañez, Pasorapa-Mojocollo, *J.R.I. Wood et al*. 27725 (K, LPB, USZ). **La Paz**: Larecaja, Tipuani, *O. Buchtien* 605 (BM, GH, K, LIL, NY, MO, US); Murillo, *M. Bang* 534 (BM, F, GH, K, MICH, MO, NY, US); Sud Yungas, *S.G. Beck* 32840 (K, LPB). **Santa Cruz**: Germán Busch, Candelaria, *J.R.I. Wood et al.* 27833 (K, LPB, OXF, USZ); Santiesteban, *M. Nee* 47090 (MO, NY, USZ); Vallegrande, *L. Arroyo et al.* 5165 (MO, USZ). **Tarija**: O’Connor, Entre Ríos, *M. Dematteis et al.* 3434 (CTES, K).

**PERU. Cajamarca**: *A. Gentry et al.* 22701 (USM). **Cusco**: *G. Calatayud et al.* 1455 (CUS, MO); Convención, *C. Vargas* 3304 (CUZ). **Huánuco**: *R. Ferreyra* 6731 (USM). **Ica**: *A. Cano et al.* 5853 (USM). **Lambayeque**: *R. Ferreyra* 12399 (MO). **Lima**: *T.H. Goodspeed* 11336 (UC, K); Chancay, *R. Ferreyra* 14138 (USM). **Loreto**: *M. Rimachi* 11631 (IBE, MO). **Piura**: *R. Ferreyra* 5921 (MO, USM). **San Martín**: *G. Klug* 4229 (BM, K, S). **Tumbes**: *R. Ferreyra* 5667 (MO).

**ECUADOR. Galápagos**: *H. Schimpff* 21 (BM, MO); *Fagerlind & Wibom* 2806 (S). **Guayas**: *J.E. Madsen* 63008 (AAU, MO). **Loja**: *G. Harling & L. Andersson* 13335 (MO). **Los Ríos**: *C. Játiva & C. Epling* 183 (MO, NY, S, US). **Manabí**: *C.E. Cerón* 18743 (ARIZ, MO). Pichincha: Alluriquin, *G. Harling & L. Andersson* (QCA).

**COLOMBIA. Antioquia**: *L. Uribe* 2537 (COL). **Bolívar**: Cartagena, *E.P. Killip & A.C. Smith* 14034 (BM, NY, S). **Cesar**: Poponte, *C. Allen* 838 (MO). **Magdalena**: Santa Marta, *H.H. Smith* 1572 (BM, K, NY, S). **Nariño**: Pasto, *J. Triana* 3808 (BM). **Norte de Santander**: Ocaña, *J. Linden* 210 (BM); **Valle**: *W.A. Barklay* 17C926 (COL).

**VENEZUELA. Aragua**: Tovar, *A. Fendler* 936 (K). **Bolívar**: *F. Delascio & R. Liesner* 6857 (MO). **Dist. Fed**.: Caracas, *A.H.G. Alston* 5442 (BM, S). **Falcón**: *R. Liesner et al.* 7608 (MO). **Guárico**: *G. Davidse* 4219 (MO). **Maracaibo**: *Moritz* 1238 (BM, K). **Miranda**: *P.E. Berry* 1121 (MO); *J. Steyermark* 104042 (MO, S). **Portuguesa**: *J.A. Steyermark* 126916 (MO). **Sucre**: *J. Steyermark* 96083 (MO).

**PANAMA.** Río La Olla (Cabra), *R. Cambra* 46 (BM, UNAP).

**COSTA RICA.** San José, El General, *A. Skutch* 2876 (K, S), ibid., 4003 (K, S); Puntarenas, *M. Grayum & B, Hammel* 9563 (BM); Nicoya, *A. Tonduz* 13687 (BM); Río Ceibo, *H. Pittier* 6628 (BM).

**NICARAGUA.** Carretera a Matagalpa, *A. Molina* 22862 (BM, F); Managua, *W.D. Stevens et al.* 3943 (BM, MO).

**EL SALVADOR.***R. Aparicio & R. Hernández* 74 (MO, LAGU).

**HONDURAS**. Río Yeguare, *A. Molina* 720 (BM).

**BELIZE.***P.H. Gentle* 841 (MO).

**GUATEMALA.** Petén, P.N. Tikal, *R. Tun Ortíz* 352 (BM, S); Sacatepéquez, *M. Véliz* 99.7358 (BM).

**MEXICO. Baja California Sur**: *J.L. León* 2199 (MEXU). **Campeche**: Kalkini-El Remate, *M. Peña-Chocarro et al.* 589 (BM). **Chiapas**: *D. Breedlove* 28567 (MEXU, MO). **Chihuahua**: Guasaremos, Río Mayo, *H.S. Gentry* 2409 (S). **Colima**: *A.C. Sanders et al.* 11356 (MEXU). **Est. México & Dist. Fed.**: Temascaltepec, *G.B. Hinton* 2004 (BM, K). **Guerrero**: Temisco, *Y. Mexia* 8713 (S); Pungarabato, Coyuca, *G.B. Hinton et al.* 6641 (BM). **Jalisco**: *S.H. Bullock* 1451 (MO). **Michoacán**: Tacupa, Huetamo, *G.B. Hinton* 5512 (BM, K). **Nayarit**: *G. Flores* 1052 (MEXU, MO). **Nuevo León**: *C. Pringle* 13276 (MO). **Oaxaca**: Pinotepa, *H. Galeotti* (BR, BM). **Querétaro**: *E. Carranza & H. Diáz* 4728 (IEB, MEXU). **Quintana Roo**: *F.C. Cabrera* 16962 (MEXU). **Sinaloa**: El potrerillo, *J.G. Ortega* 5923 (MEXU). **Sonora**: *H.S. Gentry* 1683 (MO, MEXU). **Tamaulipas**: *R. Wunderlin et al.* 1178 (MO); *M.C. Johnston* 5814 (MEXU). **Veracruz**: *T. Croat* 44027 (MEXU, MO). **Yucatán**: *G.F. Gaumer* 1380 (S), Chichankanab, *G.F. Gaumer* 2055 (BM, K).

**UNITED STATES. Florida**: *A.H. Curtiss 5281, G.V. Nash 2482 (K).***Missouri**: *P. Raven* 27688 (BM). **North Carolina**: *L. Kitching* [1906] (BM). **Texas**: *J. Reverchon* 653 (P).

**BAHAMAS.***A.E. Wright* 130 (K, NY); *D.S. Correll* 48293 (NY).

**CUBA.***C. Wright* 1647 (K, MO). **Cienfuegos**: Soledad, *A. Gonzáles* 441 (BM). **Pínar del Río**: *E.L. Ekman* 18028 (MO, S). **Santiago de Cuba**: Bayate, *E.L. Ekman* 6652 (BM, K, S). **Villa Clara**: *A. Luna* 805 (NY).

**JAMAICA.***G.R. Proctor* 27695 (BM), 18429 (BM); *W. Stearn* 36 (BM); *W. Harris* 9155 (K, NY); *E.T. Robertson* 754 (K); *T.G. Yuncker* 17768 (NY).

**HAITI.***E.L. Ekman* H2003 (K, NY, S); *E.C. Leonard* 7732 (NY).

**DOMINICAN REPUBLIC.***R. Schomburgk* 101 (BM). *E.L. Ekman* H5787 (S); *A.H. Liogier* 24383 (NY); *T.A. Zanoni et al.* 18180 (NY).

**PUERTO RICO.***P. Sintenis* 2912 (K); 3216 (BM, S); *F. & A. Axelrod* 1769 (NY), 8649 (K).

**LESSER ANTILLES. U.S. Virgin Islands**: St Croix: *F.R. Fosberg* 59167 (BM, US). **U.K. Virgin Islands**: Tortola: *D’Arcy* 315 (BM). **Antigua**: *H.E. Box* 1139 (BM). **Montserrat**: *R.A. & E.S. Howard 19642* (BM); *G.R. Proctor* 19010 (BM). **Guadeloupe**: *A. Duss* 2475 (NY). **Dominica**: *C.A. Shillingford* 361 (MO). **Martinique**: *A. Duss* 1231 (NY). **St Lucia**: fide Powell (1979). **St Vincent**: *H.H. & G.W. Smith* 1168 (K, NY). **Grenada**: *W. Hawthorne & D. Jules* 947 (FHO). **Barbados**: *E.G.B. Gooding* 186 (BM).

**TRINIDAD.***D. Vesey-Fitzgerald* 4515 (BM); *A. Fendler* 588 (BM); *N.W. Simmonds* 196 (K).

**NETHERLANDS ANTILLES. Curaçao**: *M. Arnold-Broeders* 3651 (BM, NY), *A.S.J. van Proosdij et al*. 574 (K, U).

#### Notes.

The blue flowers, 3-lobed leaves and long, pilose sepals which taper from near the base make this an easily identified species except in North America, where it has commonly been misnamed *I. hederacea* Jacq., which is distinguished by its smaller corolla and the recurved tips of the sepals – these reported as fleshy but this is not apparent on herbarium specimens.

*R. Ferreyra* 14138 is a rather remarkable specimen of what appears to be an erect plant. It is one of a number of somewhat aberrant plants from the coastal Lomas of Peru.

### 237. Ipomoea hederacea

Taxon classificationPlantaeSolanalesConvolvulaceae

Jacq., Collectanea 1: 124. 1787. (Jacquin 1787: 124)

Convolvulus hederifolius Salisb., Prodr. Stirp. Chap. Allerton 123. 1796. (Salisbury 1796: 123). Type. Based on Ipomoea hederacea Jacq.
Cleiemera hederacea (Jacq.) Raf., Fl. Tellur. 4: 77. 1836 [pub. 1838]. (Rafinesque 1838a: 77).
Convolvulus hederaceus var. beta L., Sp. Pl. 154. 1753. (Linnaeus 1753: 154). Type. Icon in Dillenius, Hort. Eltham. 1: 98, t. 82, f. 94, designated by Shinners (1965).
Convolvulus hederaceus var. eta L., Sp. Pl. 154. 1753. (Linnaeus 1753: 154). Type. Icon in Dillenius, Hort. Eltham. 1: 96, t. 80, f. 92, designated by Shinners (1965).
Cleiemera hirsuta Raf., Fl. Tellur. 4: 78. 1836 [pub. 1838] (Rafinesque 1838a: 78). Type. Based on Icon in Dillenius, Hort. Eltham. 1: 96, t. 80, f. 92.
Ipomoea barbata Roth, Catalecta Bot. 1: 37.1797. (Roth 1797: 37). Type. Grown from seed of unspecified origin (whereabouts unknown).
Pharbitis barbata (Roth) G. Don, Gen. Hist. 4: 263. 1838 (Don 1838: 263).
Ipomoea barbigera Sweet, Brit. Flow. Gard. 1: t. 86. 1823. (Sweet 1823–25: t. 86). Type. Icon, t. 86 in Sweet, Brit. Flow. Gard. 1, lectotype, designated here).
Pharbitis barbigera (Sweet) G. Don, Gen. Hist. 4: 262. 1838. (Don 1838: 262).
?Ipomoea avicularis Raf., Fl. Ludov. 47. 1817. (Rafinesque 1817: 47). Type. Not specified.
?Ipomoea phymatodes Spreng., Nov. Prov. 24. 1818. (Sprengel 1818: 24). Type. Not specified.
Ipomoea hederacea var. integriuscula A. Gray. Syn. Fl. N. Amer., ed. 2: 2: 433. 1886. (Gray 1886: 433). Type. USA. Florida, St John’s River, *A.H. Curtiss 2158* (holotype GH00054459, isotypes MO, NY, VT).
Ipomoea hederacea var. integrifolia Hallier f., Jahrb. Hamburg. Wiss. Anst. Beih. 16: 42. 1899. (Hallier 1899a: 42), nom. illeg., non Ipomoea hederacea var. integrifolia C.B. Clarke (1883). Type. Based on Dillenius, Hort. Eltham. 1: 98, t. 82, f. 94.
Ipomoea desertorum House, Ann. New York Acad. Sci. 18: 203. 1908. (House 1908b: 203). Type. UNITED STATES. Arizona, Tucson, Thornber 29 (holotype NY00319061).

#### Type.

Plant cultivated in Vienna *Jacquin* s.n. (lectotype W, designated by Austin et al. (2014: 167ff.).

#### Description.

Annual herb; stems twining, sparsely to densely pubescent. Leaves petiolate, 5–12 cm long and wide, usually 3(–5)-lobed, rarely entire, base cordate, apex acute to acuminate, both surfaces pubescent; petioles 3–12 cm, pubescent. Inflorescence of 1–3-flowered axillary cymes; peduncles 5–10 cm; bracteoles lanceolate to elliptic, 5–8 × 2–3 mm, persistent; pedicels 3–7 mm; sepals 12–18 × 4–5 mm, lanceolate, abruptly narrowed from a broad base, apex long acuminate, often recurved, densely pilose, especially near base; corolla 2–3.7 cm long, funnel-shaped, light blue with a whitish tube, limb 1.5–5.5 cm diam., shallowly lobed. Capsules depressed-globose, 8–12 mm, glabrous, enclosed by accrescent sepals; seeds up to 4, 4–4.5 mm, pyriform, dark brown, densely puberulent.

#### Illustration.

Figures 3G, 121B.

#### Distribution.

A common species of disturbed bushy places in temperate regions of the USA and Canada, which extends uncommonly into northern Mexico. There are many records from elsewhere in the Americas in different databases and in the literature (Austin and Huáman 1996, Nelson 2008, Austin et al. 2012, for example) but the only one we have traced is from Cuba. Most are errors for *Ipomoea nil* and others may be adventives but confirmation is required in each case. It is reported reliably as an adventive in Europe, for example Sell and Murrell 2009: 348.

**MEXICO. Baja California Sur**: Mesa del Potrero de San Javier, *A. Carter* 4985 (BM, MEXU, UC). **Chiapas**: Chicoasén, *A. Reyes García* 887 (BM, MEXU). **Sonora**: *T.R. Van Devender et al.* 90-468B (ARIZ); *H.S. Gentry* 4733 (MEXU); *A. Burquez & V.W. Steinmann* 96-1366 (MEXU). **Michoacán**: Cerro El Águila, *G. Cornejo Tenorio* 3025 (K, MEXU). **Oaxaca**: San Juan Bautista Cuicatlán, *J.I. Calzada* 24613 (K, MEXU). **Tamaulipas**: Miquihuana, *L.R. Standford et al.* 793 (ARIZ).

**UNITED STATES. Alabama**: Mobile, *M.G. Lelong* 8176.2 USAM). **Arizona**: *R. Felger et al.* 02-318 (ARIZ); *J. Tedford* 06-253 (ARIZ). **Arkansas**: *M. Stewart* 88-144 (UARK). **Delaware**: *R.C. Bauman* 313 (K). **Florida**: *A.H. Curtiss* 2158 (MO, NY); Drummond (K). **Georgia**: *L.E. Foote* s.n. (GA). **Illinois**: *G.H. French* 2158 (K). **Indiana**: Posey Co., *C. Deam* 37698 (ALBC). **Kansas**: *Bodin* 1884 (S). **Kentucky**: *G.W. Libby* OB-563 (EKY). **Louisiana**: Assumption, *E. Ewan* 18902 (BM). **Maryland**: *W.D. Longbottom* 18334 (NY). **Mississippi**: Oktibbeha, *M. Kirkpatrick* 16 (MISSA). **Missouri**: *Trusik et al.* 9A (S), *G. Yatskievich* 14-43 (MO); Ozarks, Jefferson Co., *P. Raven* 27296 (BM, MO); *G. Davidse* 38553 (MO). **New Mexico**: *R.D. Worthington* 19948 (DES). **New York**: *D.E. Atha* 14192 (NY). **North Carolina**: Warsaw, *D.L. Martin* 185 (UNCC). **Oklahoma**: *K.C. Bennett* 2689 (KHD). **South Carolina**: Piedmont, *Bio* 453 (FMUH). **Tennessee**: Benton Co., *T. Walker* 16069 (TENN). **Texas**: Texar, *W.R. Carr* 21275 (TEX). **Virginia**: *A.H. Curtiss* s.n. [14/9/1872] (S).

**CANADA. Ontario**: fide Scoggan (1979: 1257).

**CUBA. La Habana**: *S.A. Morales* s.n. (HAC).

#### Notes.

Very similar to *Ipomoea nil* differing in the smaller corolla 2–3.7 (not 3.5–4.5) cm long and particularly the shorter sepals (12–18 (not 15–32) mm long) with abruptly narrowed, somewhat fleshy, obtuse tips which are usually recurved. As in *Ipomoea nil* the sepal base is accrescent and becomes even more strikingly ovate in fruit. The two species may intergrade.

*Ipomoea phymatodes* is cited in synonomy with doubt. It was compared with *I. hederacea “carolina*”, the flowers are said to be solitary and the root tuberous, which is wrong, but the exterior sepals described as “revolutis” seem correct. Likewise, *I. avicularis* is also cited in synonomy with doubt. Again no type was preserved and the protologue is inadequate to be certain of the species identity.

Some Specimens from Baja California Sur are intermediate with *Ipomoea nil* (*E. Martinez & A. Ibarra* 40654 (MEXU); *A. Carter* 4985, 5195 (MEXU) with short corolla but erect sepals.

### 238. Ipomoea purpurea

Taxon classificationPlantaeSolanalesConvolvulaceae

(L.) Roth, Bot. Abh. Beobacht. 27. 1787. (Roth 1787: 27)

Convolvulus purpureus L., Sp. Pl. (ed.2): 219. 1762. (Linnaeus 1762: 219). Type. Icon in Dillenius, Hort. Eltham. 1: 100, t. 84, f. 97 (1732), designated by Verdcourt (1957b: 233).
Convolvulus mutabilis Salisb., Prodr. Stirp. Chap. Allerton 123. 1796. (Salisbury 1796: 123). Type. Based on Convolvulus purpureus L.
Pharbitis purpurea (L.) Voigt., Hort. Suburb. Calcutt. 354. 1845. (Voigt 1845: 354).
Quamoclit purpurea (L.) M. Gómez, Fl. Habana 347. 1899 [pub.1897]. (Gómez de la Maza y Jiménez 1897: 347).
Convolvulus hederaceus var. gamma L. Sp. Pl. 154. 1753. Type. Icon in Dillenius, Hort. Eltham. 1: 99, t. 83, f. 96 (lectotype designated by Shinners 1965).
Ipomoea punctata Pers., Syn. Pl.: 1: 184. 1805. (Persoon 1805: 184). Type. Based on Dillenius, Hort. Eltam. 99 t. 83 f. 96.
Pharbitis punctata (Pers.) G. Don, Gen. Hist. 4: 263. 1838. (Don 1838: 263).
Convolvulus hederaceus var. epsilon L. Sp. Pl. 154. 1753. Type. Icon in Dillenius, Hort. Eltham. 1: 100, t. 84, figure 97 (lectotype designated by Shinners 1965).
Ipomoea discolor Jacq. Pl. Hort. Schoenbr. 3: 6. 1798. (Jacquin 1798: 6), nom. rej. Type. Plant cultivated at Vienna (possible type M0184978).
Ipomoea intermedia Schult., Observ. Bot. 37. 1809. (Schultes 1809: 37). Type. Based on Ipomoea discolor Jacq.
Ipomoea glandulifera Ruiz & Pav., Fl. Peruv. 2: 12, t. 121a. 1799. (Ruiz and Pavón 1799: 12). Type. PERU. t. 121a in Ruiz and Pavón 1799, lectotype, designated here).
Ipomoea villosa Ruiz & Pav., F. Peruv. 2: 12. 1799. (Ruiz and Pavón 1799: 12). Type. PERU. Pozuzo and Muña, (lectotype t. 121b in Ruiz and Pavón (1799), designated here).
Ipomoea hispida Zuccagni, Collectanea 127. 1806. (Roemer 1806–09: 127). Type. Not cited.
Pharbitis hispida (Zuccagni) Choisy, Mém. Soc. Phys., Genève 6: 438 [56]. 1834. (Choisy 1834: 438 [56]).
Ipomoea zuccagnii Roem. & Schult., Syst. Veg. 4: 230. 1819, nom. superfl., based on Ipomoea hispida Zuccagni
Ipomoea hirsutula J. Jacq., Ecl. Pl. Rar. 1: 65. 1811 [pub. 1813]. (Jacquin 1813: 65). Type. t. 44 in J.F. Jacquin (1813), lectotype designated by Austin (1990).
Cleiemera cuspidata Raf., Fl. Tellur. 4: 78. 1836 [pub. 1838]. Type. Based on icon in Dillenius, Hort. Eltham. 1: 96, t. 80, f. 96.
*Pharbitis diversifolia Lindl., Edwards's Botanical Register 23: t. 1988. 1837 (Lindley 1837: t. 1988). Type. PERU. *A. Mathews* 2050, portion at top right of sheet in Herb Lindley (CGE 06401 p.p., lectotype, designated Wood et al. (2015: 101), isolectotypes BM, OXF).
*Pharbitis nil var. diversifolia (Lindl.) Choisy in A.P. de Candolle, Prodr. 9: 343. 1845. (Choisy 1845: 343).
*Ipomoea purpurea var. diversifolia (Lindl.) O’Donell, Lilloa 26: 385. 1953 (O’Donell1953a: 385).
*Ipomoea affinis M. Martens & Galeotti, Bull. Acad. Roy. Sci. Bruxelles 12 (2): 263. 1845. (Martens and Galeotti 1845: 263). Type. MEXICO. Oaxaca, *H. Galeotti* 1377 (lectotype BR 00006973353, designated here; isolectotypes G, MO, P, W).
*Ipomoea pilosissima M. Martens & Galeotti, Bull. Acad. Roy. Sci. Bruxelles 12 (2): 264. 1845. (Martens and Galeotti 1845: 264). Type. MEXICO. Oaxaca, *H. Galeotti* 1364 (lectotype BR0006972738, designated here).
*Ipomoea purpurea forma triloba Meisn. in Martius et al., Fl. Brasil. 7: 223. 1869. (Meisner 1869: 223). Type. Not specified.
*Ipomoea mexicana A. Gray, Syn. Fl. N. Amer. 2: 210. 1878. (Gray 1878: 210). Type. UNITED STATES. New Mexico, *C. Wright* 1612 (lectotype GH00054732, designated here; isolectotypes K, NY).
Ipomoea wattii C.B. Clarke, J. Linn. Soc., Bot. 25: 49. 1889. (Clarke 1889: 49). Type. INDIA. [Nagaland], Kohima, *C.B. Clarke* 41307 (holotype K000830827).
*Ipomoea diehlii M.E. Jones, Contr. W. Bot. 12: 53.1908. (Jones 1908: 53). Type. MEXICO. Chihuahua, San Diego Canyon, I.E. Diehls.n. (holotype RSA0002420).
Ipomoea chanetii H. Lévl., Repert. Spec. Nov. Regni. Veg. 9: 42. 1911. (Léveillé 1911: 452). Type. CHINA. Tché-Ly, Tchen Ting Fou, *L. Chanet* 124 (holotype E00284512). Plants with lobed leaves are indicated with an asterisk*.

#### Type.

Based on *Convolvulus purpureus* L.

#### Description.

Twining annual herb, stems pilose. Leaves petiolate, 3–8(–15) × 3.5–8(–14) cm, ovate (rarely 3-lobed to half way), shortly acuminate, cordate with rounded auricles, both surfaces thinly to densely hispid-pilose; petioles 3–15 cm, pilose. Inflorescence of 2–5-flowered, pedunculate, axillary cymes, often umbellate in form; peduncles 1.5–7 cm, pubescent; bracteoles 2–8 mm, filiform, relatively persistent; pedicels 0.5–1.8 cm, pubescent but pilose apically; sepals subequal, 11–17 × 2–3 mm, lanceolate to oblong-lanceolate, acute to subobtuse, hispid-pilose, more densely so in lower half, inner sepals with scarious margins; corolla 4–5 cm long, funnel-shaped, tube white, limb usually pink, sometimes cream or bluish, glabrous, 4 cm diam., unlobed. Capsules subglobose, 9–11 mm, glabrous, 6-seeded; seeds 5 mm long, appearing glabrous but minutely tomentellous under a microscope.

#### Illustration.

Figures 5D, 10C, 121E; Acevedo-Rodríguez (2005: 174); Bosser and Heine (2000: 51); Deroin (2001: 237); Carranza (2007: 102).

#### Distribution.

Widely distributed throughout the tropics as an escape from cultivation or as a weed. It is abundant in the dry inter-Andean valleys of northern Argentina, Bolivia and Peru between around 1000 m and 2800 m and is similarly abundant in upland areas of Mexico. It is much less common in more humid lowland areas, there are few records from the Cerrado or Chaco biomes, and it is absent from much of Central America, the Guianas and the Caribbean. There is perhaps a scattering of records of cultivated plants amongst the following.

**URUGUAY.***W.G. Herter* 1372 (S).

**ARGENTINA. Catamarca**: *C. Saravia Toledo et al.* 12925 (CTES, S), 13037 (CTES, K); *S. Venturi* 7052 (BM). **Córdoba**: Dique San Roque, *Stuckert* 14826 (LIL); Achiras, *D.O. King* 727 (BM). **Entre Ríos**: *A. Burkart et al*. 29583 (K, MO, SI). **Jujuy**: *C. O’Donell* 2803 (LIL). **La Rioja**: Aimogasta, *A.T. Hunziker* 4995 (LIL). **Mendoza**: *Cuezzo* 2655 (LIL). **Misiones**: *Montes* 15484 (LIL, S). **Salta**: *L.J. Novara* 7406 (G), 8848 (S); *M. Dematteis & A. Schinini* 2688 (CTES, K). **Tucumán**: San Pedro de Colalao, *S. Venturi* 4396 (LP, NY, LIL).

**PARAGUAY. Alto Paraná**: Est. Río Bonito, *E. Zardini & Guerrero* 42621 (MO, PY). **San Pedro**: *A.L. Woolston* 1254 (K, S).

**BRAZIL. Minas Gerais**: *Lindberg* 162 (S); Ituiutaba, *A. Macedo* 4153 (K, S). **São Paulo**: *Heiner* 282 (S); *W. Hoehne* s.n. [29/3/1955] (K).

**FRENCH GUIANA.** Cultivated fide Lemée (1952).

**CHILE**: Santiago, *Barbosa* 5653 (MO)

**BOLIVIA. Chuquisaca**: Oropeza, Sucre-Yotala, *J.R.I. Wood* 19287 (HSB, K, LPB); Boeto, Villa Serrano-Nuevo Mundo, *J.R.I. Wood* 28126 (LPB, OXF, USZ); Tomina, Padilla, *J.R.I. Wood et al.* 27656 (K, LPB, USZ). **Cochabamba**: Carrasco, Hoyadas, *J.R.I. Wood et al.* 19429 (BOLV, HSB, K, LPB, USZ); Cercado, *M. Atahuachi* 716 (BOLV). **La Paz**: Nor Yungas, Coripata, *M. Bang* 2113 (BM, F, GH, K, LPB, NY, MO, US). **Potosí**: Sud Chichas, *F. Zenteno et al.* 11569 (K, LPB). **Santa Cruz**: Caballero, *J. Balcazar & Franco* 471 (LPB, USZ); Florida, Pampa Grande, *P. Acevedo-Rodríguez et al.* 4549 (ARIZ, NY, US, USZ). **Tarija**: Arce, Padcaya-Chaguaya, *S.G. Beck et al*. 26134 (ARIZ, LPB, MO); Cercado, Pampa Redonda, *F. Zenteno et al.* 3492 (LPB) – var. diversifolia.

**PERU. Amazonas**: *D.N. Smith & J. Cabanillas* 7114 (MO). **Ancash**: *P. Francia* 152 (MO). **Arequipa**: *D. Montesinos* 2799 (USM). **Cajamarca**: Huarango, *E. Rodríguez* 1254 (MO, OXF). **Cusco**: *C. Vargas* 537 (CUZ, MO); Anta, *C. Vargas* 20560 (CUZ) – var. diversifolia. **Huancavelica**: *O. Tovar* 5018 (USM) – var. diversifolia.**Huánuco**: *C. Vargas* 5270 (CUZ). **Junín**: *F. Woytkowski* 35389 (MO). **La Libertad**: *R. Ferreyra* 7661 (MO). **Lima**: *R. Ferreyra* 6147 (MO). **Moquegua**: *P. Cáceres* 2929 (USM). **Pasco**: *D.N. Smith* 4130 (MO, USM). **Piura**: *R. Ferreyra* 10762 (USM) – var. diversifolia. **San Martín**: *D. Melin* 297 (S). **Tumbes**: *C. Díaz et al.* 6060 (MO).

**ECUADOR. Galápagos**: *G. Harling* 5176 (S). **Azuay**: *C.H. Dodson* 11641 (MO). **Chimbarazo**: *H. Lugo* 1842 (MO). **Imbabura**: Ibarra, *W. Jameson* 408 (BM). **Pichincha**: *X. Cornejo & S. Laegaard* 1961 (AAU); *E. Asplund* 20326 (K, NY, S).

**COLOMBIA. Antioquia**: *J.J. Triana* s.n. [6/1857] (COL); *L. Uribe* 1935 (COL) – var. diversifolia. **Boyacá**: Tungurahua: *M. Acosta-Solis* 9064 (F); La Uvita, *J. Cuatrecasas* 1849 (COL). **Cauca**: *A. Pérez* 6074 (COL). **Cundinamarca**: Santandercito, *L. Uribe* 584 (COL) – var. diversifolia. **Magdalena**: Santa Marta, *H.H. Smith* 1575 (MO). **Nariño**: *A. Fernández* 1145 (COL). **Valle**: *P.A. Silverstone-Sopkin* 2458 (MO).

**VENEZUELA.***Moritz* 1065 (BM) – var. diversifolia. **Dist. Fed.**: Caracas, *A.H.G. Alston* 5441 (BM, S). **Miranda**: *B. Trujillo* 18800 (MO). **Táchira**: *J. Steyermark & R. Liesner* 118507 (MO).

**PANAMA.** Chiriquí, *E.L. Tyson* 5662 (MO).

**COSTA RICA.***M. Chavarria* 670 (K, MO); Alajuela, *P. Wilkin* 501 (BM) – var. diversifolia; ibid., *P. Wilkin & S. Jennings* 120 (BM);.

**NICARAGUA.***P.P. Moreno* 17845 (MO).

**EL SALVADOR.** Santa Ana, Chalchuapa, *D. Rodríguez et al.* 1416 (BM).

**GUATEMALA.** Barcenas Experimental Station, *A & A.R. Molina* 26902 (BM, F).

**MEXICO. Chiapas**: *D.E. Breedlove* 20366 (MO). **Chihuahua**: *E.W. Nelson* 6252 (K); Colonia García, *C.H.T. Townsend & C.M. Barber* 232 (BM, K) – var. diversifolia; Seven Stars Mine, *C.H.T. Townsend & C.M. Barber* 413 (BM) – var. diversifolia. **Coahuila**: *J. Gregg* 653 (MO); *E. Palmer* 905 (K) – var. diversifolia. **Durango**: *E. Palmer* 639 (BM); *E. Palmer* 591 (K) – var. diversifolia, 639 (K). **Est. México & Dist. Fed.**: Temascaltepec, *G.B. Hinton* 1820 (B, K) – var. diversifolia, 4606 (K), 5414 (BM, K), 6511 (BM, K) – var. diversifolia, 8413 (K), 8455 (S) –var. diversifolia; *E. Bourgeau* 624 (K), 727 (K), 1062 (K); *C.G. Pringle* 6607 (BM, K, S) – var. diversifolia; *H. Iltis et al.* 28621 (K) – var. diversifolia. **Guanajuato**: *Haage* 944 (K). **Guerrero**: *E. Matuda* 96 (MO); *G.B. Hinton* 9694 (K) – var. diversifolia. **Jalisco**: *E. Palmer* 583 (BM, K) – var. diversifolia. **Michoacán**: *G. Arsène* 1950 (K) – var. diversifolia; Morelia, *G. Arsène* 5473 (BM) – var. diversifolia. **Nuevo León**: *M. Taylor* 105 (S). **Oaxaca**: type of Ipomoea affinis – var. diversifolia. **Puebla**: *Bro. Nicholas* s.n. (K); Tehuacan, *C.A. Purpus* 5732 (BM). **Sonora**: *A.L. Reina & T.R. Van Devender* 2005-1651 (MO); Río Mayo, *H.S. Gentry* 1709 (K) – var. diversifolia. **Tamaulipas**: *L.R. Stanford et al.* 2309 (MO). **Veracruz**: Valle de Córdoba, *E. Bourgeau* 1728 (K, P, S); Orizaba, *M. Botteri* 565 (BM, K, OXF) – var. diversifolia. **Zacatecas**: *J.E, Kirkwood* 74 (MO).

**UNITED STATES. Alabama**: *G. Een* s.n. 26/7/1950 (S). **Arizona**: *W.W. Jones* s.n. (K); *J. C. Blumer* 1807 (K) – var. diversifolia. **California**: *P.H. Raven* 7963 (K, S). **Florida**: fide Wunderlin and Hansen 2011: 392. **Georgia**: *N.C. Craft Coile & C. Dunn* 1236 (BM). **Kansas**: *R.L. McGregor* 320 (S). **Kentucky**: *G. Een* s.n. 23/9/1950 (S). **Michigan**: *M Fallass* s.n. [15/10/1897] (ALBC). **Mississippi**: *D.R. Morgan* 1447 (MISS, MO). **Missouri**: *G. Yatskievych* 96-77 (MO). **New Mexico**: *F.A. Barkley* 14710 (S) – var. diversifolia; *Earle & Earle* 332 (BM, K) – var. diversifolia. **New York**: *D. Atha & D. McClelland* 6873 (NY). **North Carolina**: *Horton* 346A (S). **South Carolina**: *Nelson & Boyle* 17404 (NY). **Tennessee**: *R. Kral* 74450a (BM). **Texas**: *Biltmore* 14909 (S). **Virginia**: *E.K. Balls* 7705 (BM, K).

**CANADA**. **Ontario**: *Macoun* s.n. (K).

**CUBA. Pinar del Río**: Viñales, *Britton & Britton* 7530 (NY).

**PUERTO RICO**. *A. Stahl* 791 (NY).

#### Typification.

The plate (t.121b) in Ruiz and Pavón (1799) is chosen as lectotype of *Ipomoea villosa* in preference to the collection at Madrid (MA814679) as this was collected in 1800 after the publication of *Ipomoea villosa* and, in any case, represents a mixed gathering, apparently from Ecuador.

#### Notes.

*Ipomoea purpurea* is quite variable. It usually has entire leaves but sometimes lobed-leaved specimens occur, apparently more commonly in Mexico than in South America. Specimens with lobed leaves can be named Ipomoea purpurea var. diversifolia (Lindl.) O’Donell (1953a: 385).

Specimens with lobed leaves can be confused with *Ipomoea nil* but can usually be distinguished by the shorter oblong-lanceolate sepals and pink flowers. However, flower colour is variable and occasional specimens are difficult to assign to species. The sepals are usually subobtuse but specimens are found with very acute sepals, such as *Rodríguez* 1254 (MO, OXF) from Peru. *R. Ferreyra* 10762 is a short erect specimen from the Peruvian coastal desert region.

### 239. Ipomoea zacatecana

Taxon classificationPlantaeSolanalesConvolvulaceae

J.R.I. Wood & Scotland sp. nov.

urn:lsid:ipni.org:names:77208074-1

#### Type.

MEXICO. Zacatecas, Mun. Villanueva, Carr. 54 on Zacatecas-Guadalajara highway, c. 1 km S del desvio a Laguna del Carretero, 23 Aug. 1995, *E. D. Enriquez E*. 568 (MEXU964013).

**Diagnosis**. Superficially resembles *Ipomoea purpurea* but differs in the prominent lateral tooth and sagittate base of the leaves, the finely acuminate sepals and mostly solitary flowers with a pubescent corolla.

#### Description.

Perennial herb from woody, xylopodium-like rootstock, much-branched at the base; stems prostrate, up to 1 m long, thinly pilose, reddish when young but woody, glabrous and muricate when old. Leaves petiolate, 1.5–3.5 × 0.7–2.4 cm, rather small, ovate, acute, base sagittate with acute auricles, the margin sometimes with a large tooth towards the base, both surfaces green, thinly pilose; petioles 0.4–1.9 cm, pubescent. Inflorescence of 1–3-flowered pedunculate, axillary cymes, the flowers mostly solitary; peduncles 1–4.5 cm, thinly pilose; bracteoles 5–7 mm, linear, relatively persistent; pedicels 3–6 mm, thinly pilose; sepals subequal, 12–13 × 5–6 mm, ovate, finely acuminate, the base rounded to cuneate, bristly white-pilose; corolla 4.5–5.5 cm long, funnel–shaped, deep pink with whitish tube, pubescent towards the apex. Capsules and seeds unknown.

#### Illustration.

Figure 123.

**Figure 123.**
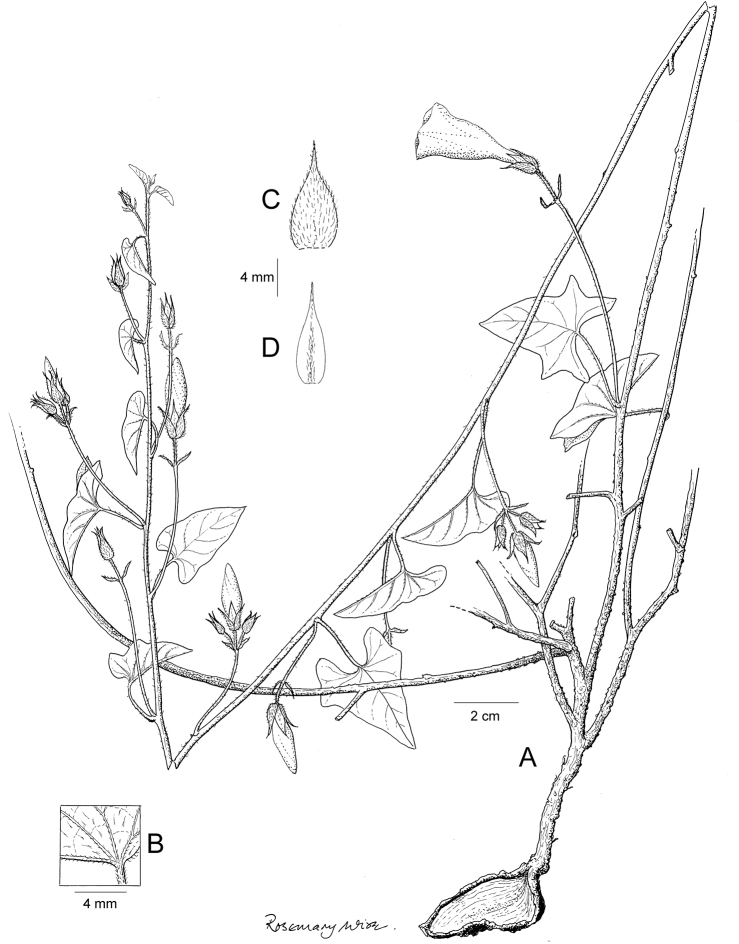
*Ipomoea zacatecana*. **A** habit **B** abaxial leaf surface **C** outer sepal **D** inner sepal. Drawn by Rosemary Wise from *Enriquez* 568.

#### Distribution.

Endemic to Zacatecas in Mexico, growing in dry grassland in open oak woodland with *Bouteloua*, *Chloris* and *Muhlenbegia*.

**MEXICO. Zacatecas**: type collection.

#### Note.

The placement of this species is uncertain but it is provisionally placed here because it bears a superficial resemblance to *Ipomoea purpurea*, although it differs in the prominent lateral tooth and sagittate base of the leaves, the finely acuminate sepals and mostly solitary flowers with a pubescent corolla. It is also possible that its correct placement is near *I. rupicola* as it has similar small leaves often with a lateral tooth and a pubescent corolla. However, the sepals are quite different.

### 240. Ipomoea spruceana

Taxon classificationPlantaeSolanalesConvolvulaceae

Benth. ex Meisn. in Martius et al., Fl. Brasil. 7: 223. 1869. (Meisner 1869: 223)

#### Type.

BRAZIL. Pará, Santarém, May 1850, *R. Spruce* 703 (holotype M0184963, isotypes BM, FI, M. MG, K, TCD).

#### Description.

High twining perennial herb, stems glabrous, reddish. Leaves petiolate, 6–11 × 3–9, deeply 3-lobed (to about 3/4^th^s), shallowly cordate, the central lobes lanceolate 3–4 × 0.5–1 cm, acuminate to a fine point, the laterals slightly smaller, often shallowly lobed, glabrous, the lower surface paler; petioles 1.3–1.7 cm, glabrous. Flowers somewhat densely clustered at apex of a long peduncle; peduncles 2–11 cm, sparsely hispid-pilose with bulbous based hairs; bracteoles 6–15 × 2–3 mm, narrowly ovate, boat-shaped, finely acuminate, relatively persistent, hirsute; pedicels 5–15 mm, variable in length in the same cluster, hispid-pilose; sepals slightly unequal, outer 14–17 mm, ovate, finely acuminate, densely pilose, inner narrowly ovate, pilose with scarious, glabrous margins; corolla 3.5–5 cm long, dark pink, glabrous, funnel-shaped, limb c. 2.5 cm diam., unlobed. Capsules broadly ovoid, 6–9 × 5 mm, glabrous; seeds 4.5 mm, shortly and densely pilose with hairs c. 1 mm long.

#### Illustration.

Figure 124.

**Figure 124.**
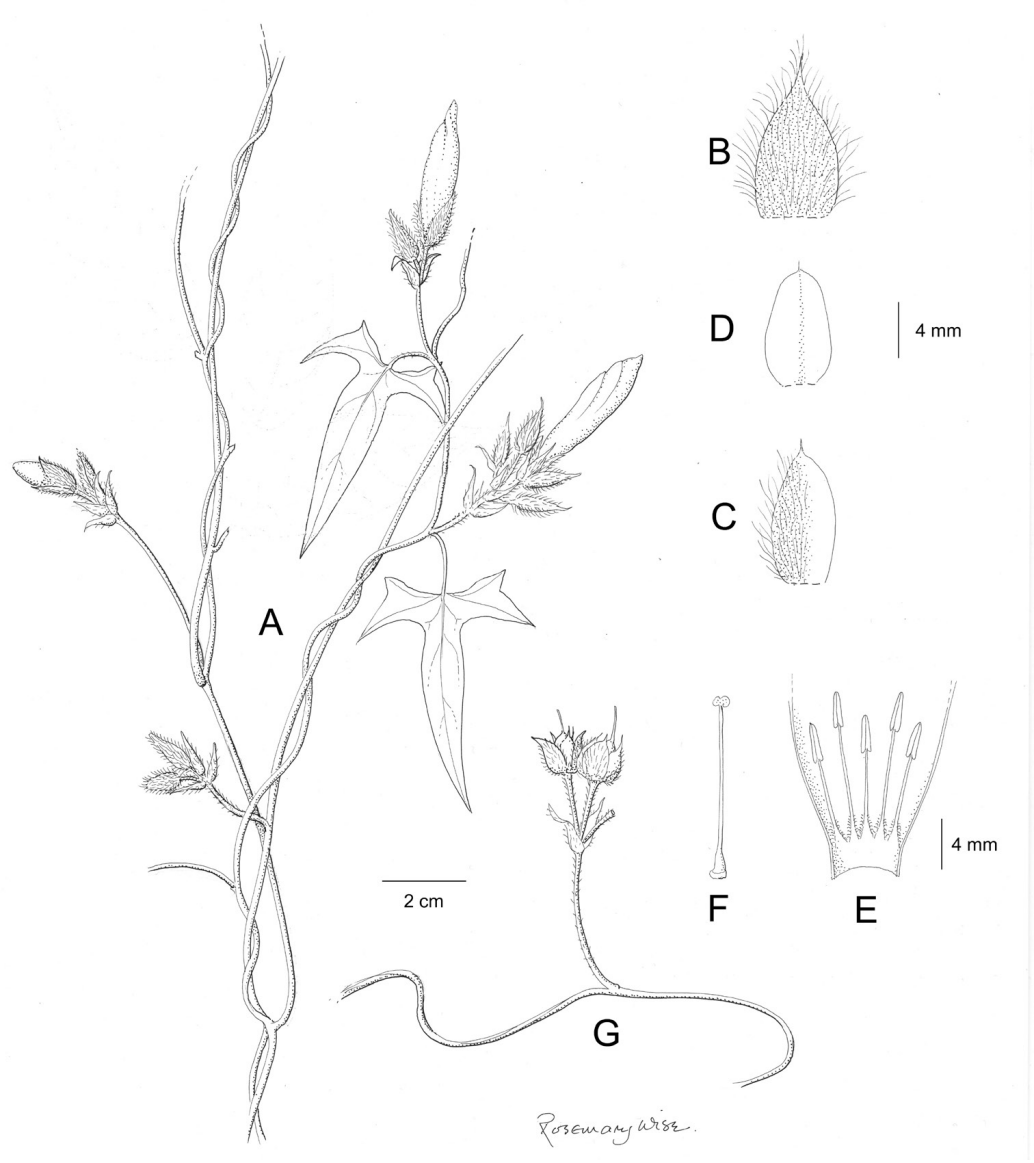
*Ipomoea spruceana*. **A** habit **B** outer sepal **C** middle sepal **D** inner sepal **E** corolla opened out to show stamens **F** ovary and style **G** fruiting inflorescence. Drawn by Rosemary Wise from *Spruce* 703.

#### Distribution.

Endemic to Amazonian Brazil, apparently very rare although several, apparently unsupported records from different states are included in Flora do Brasil 2020 under construction.

**BRAZIL. Maranhão**: Loreto, Ilha de Balsas, *G. & L.T. Eiten* 4131 (NY).

#### Note.

This species has a bilobed stigma (Figure 124) and, as we have not been able to sequence a specimen, its placement in this clade is provisional.

### 241. Ipomoea calcicola

Taxon classificationPlantaeSolanalesConvolvulaceae

J.R.I. Wood & Scotland sp. nov.

urn:lsid:ipni.org:names:77208075-1

#### Type.

MEXICO. Querétaro, Cadereyta, Cerros calizos E. de Vizarrón, 13 Sept. 1994. *J. Orozco H., R. Hernandez M. & C. Orozco L*. 10806 (MEXU).

**Diagnosis.** Very distinct because of the 3-lobed, discolorous leaves, very long peduncles, acuminate, aristate sepals with prominent scarious margins and the reddish-purple pubescent corolla. Superficially it resembles *Ipomoea spruceana* but the shortly pubescent indumentum is very different from the pilose inflorescence of that species.

#### Description.

Twining perennial herb; stems dark reddish-brown, pubescent. Leaves petiolate, 2.5–4 × 3–5 cm, 3-lobed (sometimes 5-lobed, fide field notes), central lobe oblanceolate, laterals with rounded auricles and forward-pointing tips, base broadly cordate, apex finely acute, shortly mucronate, margin undulate to obscurely dentate, adaxially dark green, glabrous, abaxially pale green, pubescent; petioles 2.5–5 cm, pubescent. Inflorescence of compact, long-pedunculate, axillary cymes; peduncles 8–30 cm, pubescent; bracteoles 2–3 mm long, filiform; secondary peduncles very short, < 1 cm; pedicels 5–13 mm, pubescent; sepals unequal, oblong-lanceolate, acuminate, shortly aristate, outer 10–11 × 2 mm, pubescent, dark green with white margins, inner 13–14 × 3 mm, glabrous, entirely scarious apart from a broad central green midrib; corolla c. 3–4 cm long, probably funnel-shaped, reddish-purple, pubescent. Capsules and seeds unknown.

#### Illustration.

Figure 125.

**Figure 125.**
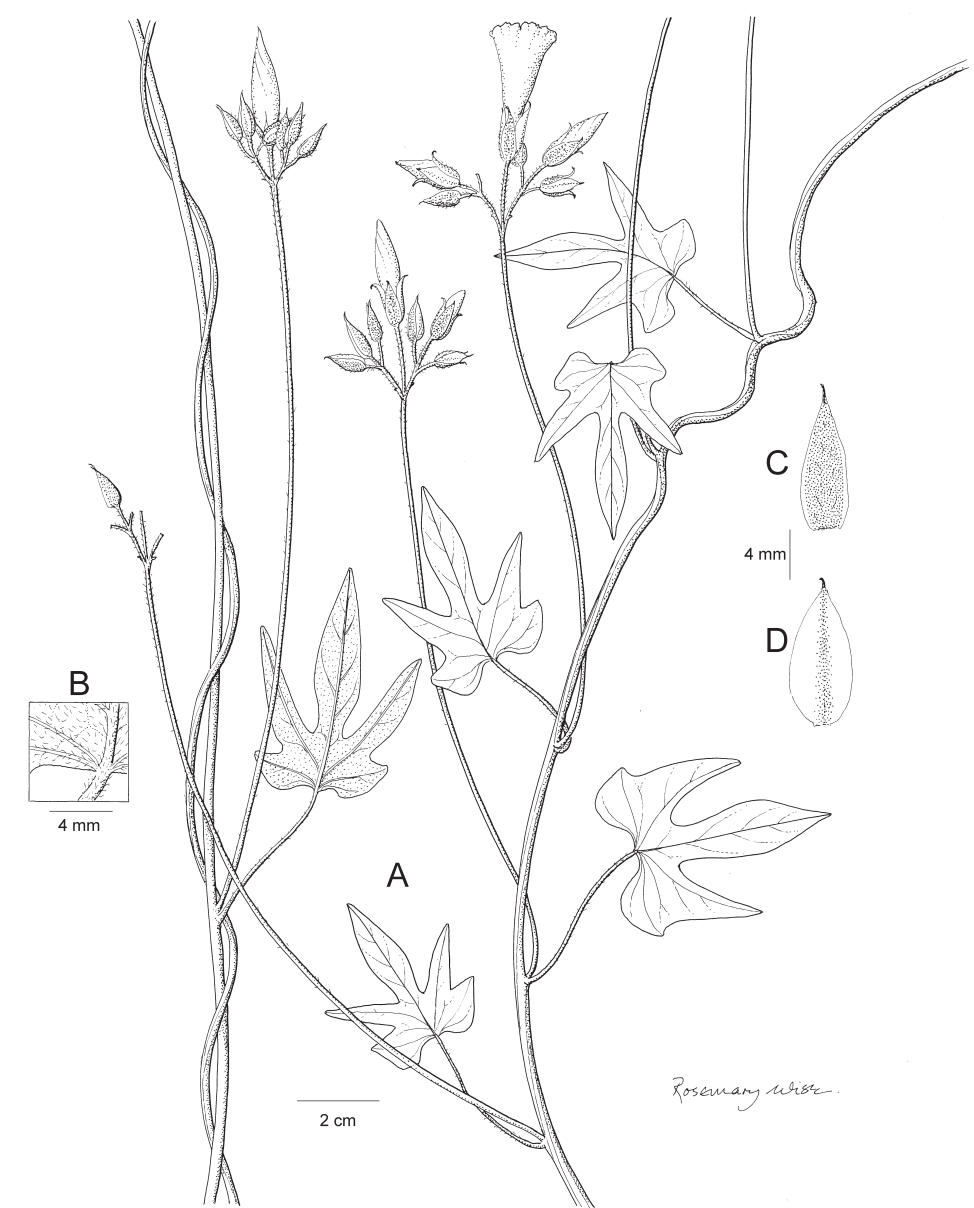
*Ipomoea calcicola*. **A** habit **B** abaxial leaf surface **C** outer sepal **D** inner sepal. Drawn by Rosemary Wise from *Orozco* 10806.

#### Distribution.

Endemic to Querétaro in Mexico, where it grows in dry pine and oak woodland at 2200 m on rocky limestone soil.

**MEXICO. Querétaro**: type collection.

#### Note.

The distinct 3-lobed, discolorous leaves, very long peduncles, acuminate, aristate sepals with prominent scarious margins and the reddish-purple pubescent corolla mark out this species. There is a strong, probably superficial, resemblance to *Ipomoea spruceana* in the leaf shape and colouring, inflorescence structure and sepal shape but the shortly pubescent indumentum is very different from the pilose inflorescence of *I. spruceana*. The placement of *I. calcicola* is uncertain and its inclusion here is based on its similarity with *I. spruceana*.

### 242. Ipomoea pubescens

Taxon classificationPlantaeSolanalesConvolvulaceae

Lam., Tabl. Encycl. 1(2): 465. 1791 [pub. 1793]. (Lamarck 1793: 465)

Convolvulus pubescens (Lam.) Willd., Hort. Berol. 1: 203. 1809. (Willdenow 1809: 203).
Pharbitis pubescens (Lam.) Choisy in A.P. de Candolle, Prodr. 9: 344. 1845. (Choisy 1845: 344).
Ipomoea heterophylla Ortega, Nov. Rar. Pl. Descr. Dec. 1: 9. 1797. (Ortega 1797–1800: 9). Type. MEXICO. Horto Regio. *C.G. Ortega*s.n., lectotype (MA222592) designated by Austin in Sida 14: 452 (1991).
Batatas heterophylla (Ortega) G. Don, Gen. Hist. 4: 261. 1838. (Don 1838: 261).
Pharbitis heterophylla (Ortega) Choisy in A.P. de Candolle, Prodr. 9: 344. 1845. (Choisy 1845: 344).
Aniseia heterophylla (Ortega) Meisn. in Martius et al., Fl. Brasil. 7: 321. 1869. (Meisner 1869: 321).
Ipomoea ortegae Poir., Encycl. Suppl. 4: 633. 1816. (Poiret 1814–17: 633), nom. illeg. superfl. Type. Based on Ipomoea heterophylla Ortega
Ipomoea papiru Ruiz & Pav., Fl. Peruv. 2: 11. 1799. Type. PERU. Tarma, Icon, t. 120, f. a in Ruiz and Pavón (1799) (lectotype designated by McDonald, Fl. Veracruz 77: 96 (1996), cited in error as t. 12).
Convolvulus papiru (Ruiz & Pav.) Spreng., Syst. Veg. 1: 592. 1825 [pub. 1824]. (Sprengel 1824: 592).
Batatas papiru (Ruiz & Pav.) G. Don, Gen. Hist. 4: 261. 1838. (Don 1838: 261).
Ipomoea subtriloba Ruiz & Pav., Fl. Peruv. 2: 12. 1799. (Ruiz and Pavón 1799: 12). Type. PERU. Huasa Huasi, *Ruiz, Pavón & Dombey*s.n. (lectotype MA814673, designated here).
Ipomoea papiru var. subtriloba (Ruiz & Pav.) Pers., Syn. Pl. 1: 185. 1805. (Persoon 1805: 185).
Batatas subtriloba (Ruiz & Pav.) G. Don, Gen. Hist. 4: 261. 1838. (Don 1838: 261).
?Ipomoea varia Roth, Catal. Bot. fasc. ii. 17. 1798 [dated1800]. (Roth 1798: 17). Type. Not cited.
Convolvulus heterophyllus Willd., Enum. 207. 1809. (Willdenow 1809: 207), nom. illeg., non Ipomoea heterophylla Ortega (1797). Type. Plant cultivated at Berlin (holotype B-W 03766).
Ipomoea willdenowii Roem. & Schult., Syst. Veg. 4: 211. 1819. (Roemer and Schultes 1819: 211). Type. Based on Convolvulus heterophyllus Willd.
Batatas willdenowii (Roem. & Schult.) G. Don, Gen. Hist. 4: 261. 1838. (Don 1838: 261).
Ipomoea hirsuta Schrank, Denkschr. Bot. Ges. Regensb. ii. 30. 1822. (Schrank 1822: 30), nom. illeg., non Ipomoea hirsuta R. Br. (1810). Type. sine data, (probable type, M0184987, labelled ‘Ipomoea hirsuta’).
Ipomoea martiusiana Steud., Nomencl. Bot. 1: 817. 1840. (Steudel 1840: 817). Type. Based on Ipomoea hirsuta Schrank
Ipomoea lindheimeri var. subintegra House, Ann. New York Acad. Sci. 18(6): 196. 1908. (House 1908b: 196). Type. UNITED STATES. Arizona (south), near Fort Huachuca, *J.G. Lemmon* 2835 (holotype GH00054463, isotype K).
Ipomoea heterophylla var. subcomosa House, Ann. New York Acad. Sci. 18: 196. 1908. (House 1908b: 196). Type. MEXICO. Durango, Ciudad Durango, *E. Palmer* 590 (holotype NY00319095, isotypes BM, F, MO, US).

#### Type.

AMERICA. Sine data (holotype P-LAM00357477).

#### Description.

Low trailing or twining herb with slender stems, pubescent in all parts, rootstock a carrot-shaped tuber. Leaves petiolate, 2–6 (–8) × 2–6(–9) cm, ovate, with sinuate margins or, usually 3–5-lobed to near base, lobes oblong-elliptic, narrowed at both ends, acute, shortly mucronate, laterals sometimes shallowly lobed near base, base cordate with rounded auricles, both surfaces densely pubescent; petioles 1–2(–5) cm, pubescent. Inflorescence of solitary or, occasionally paired, axillary flowers; peduncles 1–4 cm, pubescent; bracteoles 4–8 mm long, linear, persistent, pubescent; pedicels 2–10 mm, pubescent; sepals unequal, grey-pubescent or pilose, outer 12–21 × 6–10 mm, ovate, acuminate, base cordate, inner lanceolate, 2–4 mm wide; corolla 4–5 cm long, funnel-shaped, glabrous, tube flushed reddish, limb purplish, c. 2 cm diam., unlobed but midpetaline bands terminating in a tooth. Capsules subglobose, 8–12 mm long, glabrous, enclosed by sepals, 3-locular, up to 6-seeded; seeds 4–6 mm long, minutely tomentellous.

#### Illustration.

Figures 5H, 121C; O’Donell (1959b: 219).

#### Distribution.

Amphitropical in its distribution occurring in the United States and Mexico and along the Andes from Peru south to northern Argentina with an isolated station in central Colombia. It is locally common in dry stony grassland between 2300 and 3900 m, reaching higher altitudes than by any other *Ipomoea* species except *I. plummerae*.

**ARGENTINA. Catamarca**: Belén, *G.E. Barboza et al.* 606 (CORD). **Jujuy**: Tumbaya, *R. Kiesling* 5072 (SI); *C. O’Donell* 5447 (LIL). **Salta**: *T. Meyer* 5015 (LIL); Santa Victoria, *E. Zardini et al.* 1667 (FCQ, PY). **Tucumán**: *R. Schreiter* 10467 (LIL).

**BOLIVIA. Chuquisaca**: Oropeza, Yotala-Sucre, *J.R.I. Wood & J. Gutiérrez* 20195 (HSB, K, LPB); Tomina, 14 km S of Padilla, *S.G. Beck* 6269 (FTG, LPB); Yamparaez, Lamboyo, *J.R.I. Wood* 17844 (HSB, K, LPB). **Cochabamba**: Campero, Pasorapa-Buenavista, *J.R.I. Wood et al.* 19449 (BOLV, HSB, K, LPB, USZ); Capinota, *E. Thomas* 307 (BOLV, LPB); Punata, Cerro Tuti, *A. Fuentes* 2657 (MO, USZ); Quillacollo, *N. Ritter* 671 (NY). **La Paz**: Murillo, Mecapaca, *J. Solomon* 7406 (FTG, LPB, NY, MO). **Potosí**: Charcas, Torotoro, *J.R.I. Wood et al.* 19215 (BOLV, K, LPB). **Santa Cruz**: Vallegrande, *J.R.I. Wood et al.* 27675 (OXF, K, LPB, USZ). Tarija: Arce, Padcaya, *M. Serrano et al.* 5938 (ARIZ, MO); Cercado, Cuesta del Condor, *M. Mendoza* 2850 (USZ).

**PERU. Ancash**: *R. Ferreyra* 7374 (K). **Apurimac**: Aymareas, Challhuanca, *P. Nuñez* 7176 (MO); Grau, *C. Vargas* 5729 (CUZ). **Cajamarca**: *H. Müller & P. Gutte* 8086 (USM). **Cusco**: Calca, *C. Vargas* 938 (MO); Urubamba, Pumawanca, *P. Nuñez* 7467 (CUZ). **Huancavelica**: *O. Tovar* 184 (USM). **Lima**: San Buenaventura, *G. Vilcapoma* 8012 (USM).

**COLOMBIA. Cundinamarca**: Mosquera, Laguna de la Herrera, *R. Torres* 472 (COL); ibid., *Z. Espina* 409 (COL); ibid., Zanjón de las Cátedras, *A. Lourteig & Hernandez* 3068 (P, S).

**MEXICO. Chihuahua**: *E.W. Nelson* 6159 (K); Guasaremos, Río Mayo, *H.S. Gentry* 2458 (K, S); near Colonia García, *C.H.T. Townsend & C.M. Barber* 220 (ASU, BM, K, MO, P); San Buenaventura, *M.H. Mayfield et al.* 269 (MEXY, TEX). **Coahuila**: *J. Gregg* 389 (MO); Sierra de San Marcos, *W.L. Minckley* s.n. (ASU). **Durango**: *E. Palmer* 590 (K); *R.L. Oliver et al*. 650 (MO); Cerro Prieto-La Providencia, *E.W. Nelson* 4962 (K). **Est. México & Dist. Fed.**: *F. Cesar et al.* 185 (MEXU); Encinillas, *T. Croat* 44130 (MEXU, MO); *M. Bourgeau* 625 (P). **Guanajuato**: *J. Rzedowski* 49770 (MO); San Nicholas, *E. Ventura & E. López* 7201 (IEB, MEXU). **Hidalgo**: *C.A. Purpus* 1756 (MO); Tepeapulco, *F. Ventura* 23 (ASU, MEXU). **Michoacán**: El Fresno, *J. Rzedowski* 44030 (IEB). **Oaxaca**: Nochixtlán, *A. Ibarra* 236 (MEXU). **Puebla**: *E.M. Lira Charco et al.* 1580 (MEXU). **Querétaro**: San Joaquín a Vizarron, *E. Carranza & S. Zamudio* 6223 (IEB, MEXU). **San Luís de Potosí**: *J. G. Schaffner* 426 (P), 619 (K); *E. Reeves* R-6308 (ASU). **Sonora**: fide Felger et al. (2012). **Veracruz**: Cerro al sur de El Limón, *C.H. Ramos* 212 (MEXU). **Zacatecas**: Sierra del Astillero, *J. Henrickson* 13334 (MEXU).

**UNITED STATES. Arizona**: Santa Cruz County, *D.F. & S.K. Austin* 7605 (ASU). **New Mexico**: Luna Co, Baldy Peak, *R.D. Worthington* 18897 (L).

#### Typification.

The lectotype of *Ipomoea papiru* was wrongly cited and is, therefore, corrected above.

#### Notes.

A usually very distinct species on account of its deeply-lobed, hirsute leaves and ovate, basally cordate, outer sepals. Rare entire-leaved forms occur, for example *Wood* 17697 from Bolivia. Some specimens from the Chihuahua desert are intermediate with *I. lindheimeri*.

The root is eaten fide Gutiérrez-R (2016).

### 243. Ipomoea lindheimeri

Taxon classificationPlantaeSolanalesConvolvulaceae

Gray, Syn. Fl. N. Amer. 2(1): 210. 1878. (Gray 1878: 210)

Ipomoea heterophylla sensu Torrey, Rep U.S. Mex. Bound. Bot. 2(1): 149. 1859. (Torrey 1859: 149).
Ipomoea heterophylla var. aemula House, Ann. New York. Acad. Sci. 18: 196. 1908. (House 1908b: 196). Type. MEXICO. Chihuahua, *C.G. Pringle* 1339 (holotype GH; not seen, isotypes F, K, NDG, MEXU, NY, US).

#### Type.

UNITED STATES. Texas, New Braunfels, *Lindheimer* 622 (lectotype GH00054462, designated here).

#### Description.

Trailing or twining herb, stem adpressed pilose from a tuberous rootstock. Leaves petiolate, 2–3.5 × 2–3.5 cm, palmately 3–5(–7)-lobed to just over half way, base cordate, lobes elliptic, acute or obtuse, narrowed at both ends, thinly adpressed pilose on both surfaces; petioles 1–3.2 cm, pilose. Inflorescence of solitary, axillary flowers; peduncles 1.5–8 cm, thinly pubescent; bracteoles 3–8 mm, linear, relatively persistent; pedicels 2–15 mm, densely pubescent to densely pilose; sepals subequal, 17–23(–32) × 4–6 mm, broadly lanceolate, finely acuminate, outer pubescent, inner scarious and glabrous except pubescent midvein and ciliate margin; corolla 7–9 mm long, narrowly funnel-shaped, pink with white tube, glabrous, midpetaline bands terminating in distinct teeth. Capsule and seeds not seen.

#### Illustration.

Figure 126.

**Figure 126.**
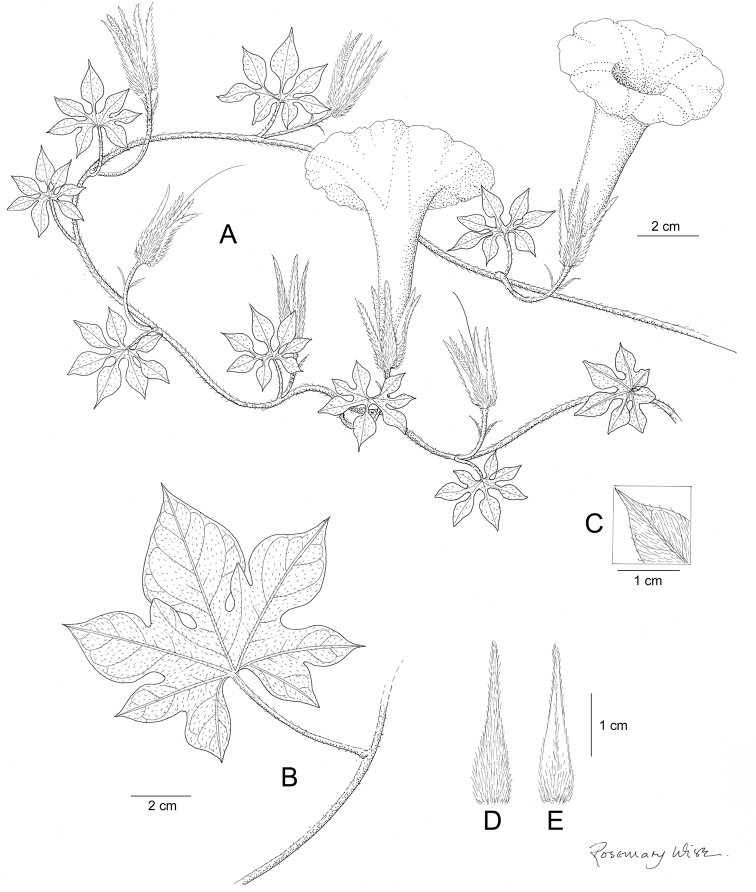
*Ipomoea lindheimeri*. **A** habit **B** leaf **C** leaf apex, abaxial surface **D** outer sepal **E** inner sepal. Drawn by Rosemary Wise from *Wright* 1613.

#### Distribution.

Uncommon in semi-desert areas of the eastern United States–Mexico border areas.

**MEXICO. Chihuahua**: 5 km N of San Miguel, *M.C. Johnston et al.* 8968 (MEXU). **Coahuila**: Puerto Santa Ana, *F.L. Lyle Wynd & C.H. Mueller* 238 (MEXU, S); Cañon de la Barrica, *T. Wendt & E. Lott* 1383 (ARIZ, ASU). **Nuevo León**: Parque de Chepinque, *D. Seigler et al.* 13395 (MEXU).

**UNITED STATES. Arizona**: fide Austin (1991a). **New Mexico**: *C. Wright* 1613 (BM, K, P, US); Florida Mts., *R.D. Worthington* 19766 (DES). Texas-New Mexico borders: *C. Wright* 508 (BM, K, OXF, US); *D.E. Atha & M. Greener* 11731 (NY); *J. Reverchon* 654 (P). **Texas**: *G.L. Fisher* 50066 (S); Mount Emory Mts., *B. H. Warnock* 158 (K); Brewster Co., *W. Hodgson & A.D. Zimmerman* 3658 (DES).

#### Note.

This species is characterised by its long-pilose, linear sepals and palmately lobed leaves combined with the solitary, narrowly funnel-shaped flowers. Var. aemula has rather broader based sepals and approaches those of Ipomoea lindheimeri var. subintegra. It is possible that this variety represents a hybrid between *I. pubescens* and *I. lindheimeri*.

### 244. Ipomoea neurocephala

Taxon classificationPlantaeSolanalesConvolvulaceae

Hallier f., Jahrb. Hamburg. Wiss. Anst. 16 (Beiheft 3): 40. 1899. (Hallier 1899b: 40)

Ipomoea igualensis Weath., Proc. Amer. Acad. Arts 45: 427. 1910. (Weatherby 1910: 427). Type. MEXICO. Guerrero, Iguala Cañon, *C.G. Pringle* 10054 (lectotype GH00054505, designated here; isolectotypes ARIZ, ASU, BRIT, CAS, COLO, CTES, DUKE, ENCB, F, GH00054505, LL, MEXU, MICH, MSC, NY, OKLA, RSA, SD, UC, US, VT, WIS).
Ipomoea federalis K. Afzelius, Svensk. Bot. Tidsk. 60: 483. 1966. (Afzelius 1966: 483). Type. BRAZIL. Distrito Federal, *J.M. Pires et al.* 9487 (holotype S07-4427 (fragment), epitype UB, designated here).
Ipomoea sawyeri D.F. Austin, Brittonia 43: 93. 1991. (Austin 1991d: 93). Type. PERU. Puno, *F. de la Puente* 3271 (holotype not received at US, isotypes FAU, now Fairchild (FTG), CIP).

#### Type.

BOLIVIA. [La Paz], Larecaja, Sorata, *G. Mandon* 1489 (holotype B†, isotypes K000612865, P03547986).

#### Description.

Twining, probably annual herb, stems hispid-pilose. Leaves petiolate, 2.5–7.5 × 2–8 cm, ovate, shallowly cordate and broadly cuneate onto the petiole, auricles rounded, apex shortly acuminate, both surfaces appressed pilose, abaxially paler; petioles 2–8 cm, hispid-pilose. Inflorescence of dense pedunculate axillary heads with 1–5 flowers; peduncles 2–12 cm, hispid-pilose; bracteoles 7–20 × 7–24 mm (but smaller inside head), ovate, acuminate, pale green with prominent dark green veins, persistent, forming an involucre round the flowers; pedicels 3 mm; sepals long-pilose, dissimilar, outer 13–14 × 4–5 mm, ovate, acuminate to an obtuse apex, inner linear-lanceolate, 9 × 2 mm; corolla 2–3.5(–5) cm long, pilose with very long hairs, narrowly funnel-shaped, tube pale with dark midpetaline bands, limb mauve, weakly lobed, c. 1.5 cm in diam. Capsules ovoid, glabrous, 4-seeded; seeds minutely puberulent.

#### Illustration.

Figure 127.

**Figure 127.**
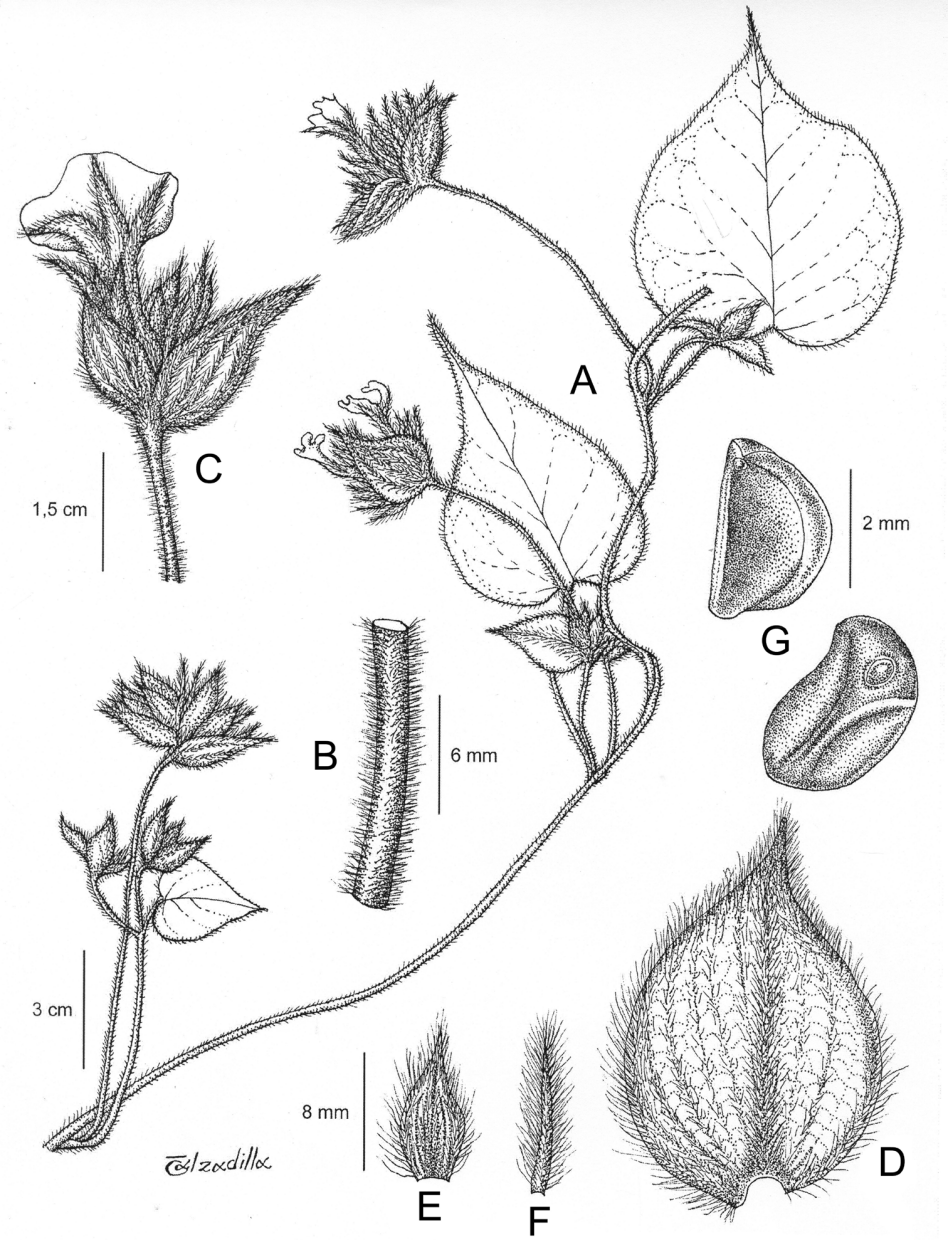
*Ipomoea neurocephala*. **A** habit **B** stem **C** flower head with involucre **D** bracteoles **E** outer sepal **F** inner sepal **G** seeds. Drawn by Eliana Calzadilla from *Parada & Rojas* 2609.

#### Distribution.

This species is of very scattered occurrence in Andean Bolivia and Peru, the planalto of Brazil and central Mexico between 800 and 2400 m in areas of dry forest.

**BRAZIL. Dist. Fed.**: Universidade de Brasilia, *H.S. Irwin et al.* 9562 (FTG, MO, NY). **Minas Gerais**: Rio Arrependido, *G. Pereira-Silva et al.* 6303 (CEN).

**BOLIVIA. La Paz**: Muñecas, Río Charazani, *A.F. Fuentes & R. Cuevas* 7966 (LPB, MO, SP); Sud Yungas *S.G. Beck et al.* 29796 (K, LPB, MO, SP). **Santa Cruz**: Vallegrande, Pucarillo, *G.A. Parada & V. Rojas* 2609 (OXF, MO, USZ).

**PERU. Cusco**: La Convención, Choquellohuanca, *Marin* 2112 (F, CUZ); ibid., Potrero, *C. Vargas* 12735 (CUZ); ibid., Amaiba, *C. Vargas* 4189 (CUZ); ibid., Santa Ana, *G. Calatayud* et al. 1575 (MO, OXF); Urubamba, Macchu Pichu, *L. Valenzuela et al.* 1629 (MO, OXF).

**MEXICO. Colima**: Ixtlahuacán, *E.J. Lott et al.* 1928 (MEXU, MO). **Guanajuato**: *J.C. Soto & G. Silva* 4543 (MO). **Est. México & Dist. Fed.**: Temascaltepec, Nanchititla, *G.B. Hinton* 8480 (GBH); *J.F. Doebley* 518 (FTG). **Guerrero**: Mina, Manchón, *G.B. Hinton* 9588 (GBH, GH, MO); El Cuindancito, *J. Soto Nuñez & G. Silva 4543* (MEXU). **Jalisco**: *J.F. Doebley* 452 (FTG); El Limón, *A. Flores* 3684 (MEXU). **Michoacán**: Aguililla, *E.M. Martínez et al.* 5380 (MEXU, MO); Chinicuila, *E. Sahagún et al.* 1197 (IEB). **Nayarit**: Amatlafán de Canas, *P. Carilllo-R & J.A. Lomelí* 3459 (IEB, MO).

#### Typification.

The holotype of *Ipomoea federalis* at S is a small fragment, so we have designated the isotype at UB as an epitype as this species can only be adequately interpreted through this second specimen. The synonymy of this species has been discussed extensively by Austin and Bianchini (1998).

#### Note.

Very distinct because of the inflorescence of bracteolate heads, the strongly veined bracteoles forming an involucre around the flowers. However it is quite variable with bracteoles not always as well developed, so sometimes merely lanceolate, and the corolla sometimes up to 5.5 cm as in *Marin* 2112, which is exceptionally robust.

### 245. Ipomoea harlingii

Taxon classificationPlantaeSolanalesConvolvulaceae

D.F. Austin, Fl. Ecuador 15: 49. 1982. (Austin 1982a: 49)

#### Type.

ECUADOR. El Oro, Zaruma-Portovelo, *Harling & Andersson* 14154 (holotype GB).

#### Description.

Twining perennial of unknown height, stems with spreading yellowish trichomes. Leaves petiolate, 4.5–17 × 3.5–14 cm, ovate, acute with a distinct acumen c. 1 cm long, cordate, appressed pilose with long hairs on both surfaces; petiole 1.5–10 cm, pilose. Inflorescence of pedunculate, axillary, few-flowered compact cymes, sometimes reduced to single flowers; peduncles 2.5–6 cm, bearded; bracteoles 1–1.7 × 0.2–0.3 cm, linear-lanceolate, mucronate, pilose, deciduous; pedicels 5–10 mm, pilose; sepals very unequal, pilose with golden hairs externally; outer bract-like, 13–20 × 8–10 mm, ovate, cordate, acute, mucronate, middle sepal lanceolate, 11–13 × 4–5 mm, inner linear, c. 9 × 2 mm; corolla 4–4.5 cm long, narrowly funnel-shaped, blue-violet, pilose with stiff spreading hairs, limb apparently lobed. Capsules and seeds unknown.

#### Illustration.

Figure 128.

**Figure 128.**
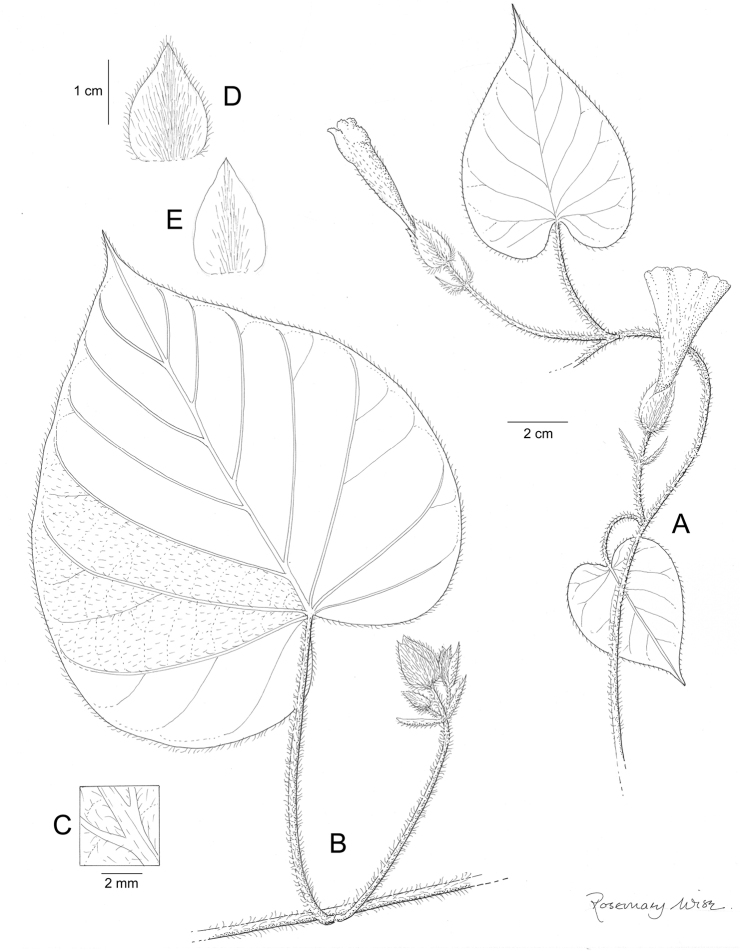
*Ipomoea harlingii*. **A** habit **B** leaf **C** abaxial leaf surface **D** outer sepal **E** inner sepal. Drawn by Rosemary Wise from *Harling & Anderson* 14154.

#### Distribution.

Endemic to Ecuador, where it grows in low altitude cloud forest at 1000–1300 m.

**ECUADOR. El Oro**: the type collection. **Loja**: Hac. Banderones, 5 km from El Limo-Casadeos road, *B. B. Klitgaard et al.* 530 (AAH, GB, LOJA, QCNE).

#### Note.

Molecular evidence does not support the distinction of this species from *Ipomoea neurocephala*. However, morphologically it is easily distinguished by the linear-lanceolate bracteoles, which are positioned 5–10 mm below the flower so not forming an involucre. Further collections may demonstrate that the two species should be merged but we keep them apart for the time being.

### 246. Ipomoea villifera

Taxon classificationPlantaeSolanalesConvolvulaceae

House, Muhlenbergia 5: 70. 1909. (House 1909a: 70)

#### Type.

GUATEMALA. Huehetenango, near Jacaltenango, *E.W. Nelson* 3579 (holotype US00111486).

#### Description.

Climbing perennial to 4 m, stem muriculate, densely pilose with brownish hairs. Leaves petiolate, rather large, 7–12 × 6–12 cm, orbicular to ovate, entire or weakly 3-lobed, acute to acuminate, base cordate, adaxially pubescent, abaxially whitish, densely pubescent; petioles 5–7 cm. Inflorescence subcapitate, formed of pedunculate, bracteate heads; peduncles 11–20 cm, villous; bracteoles 20–25 × 5 mm, narrowly oblong-ovate, acuminate, ±persistent; pedicels very short, 0–5 mm; sepals subequal, 16–22 mm, broadly lanceolate, acuminate, villous; corolla c. 5 cm long, funnel-shaped, purple, villous on tube and lobes. Capsules and seeds not known.

#### Distribution.

A plant of forest and scrubby swamp below 1300 m over a limited area of Mesoamerica; apparently uncommon.

**HONDURAS.** Ocotepeque, Sinuapa, *A. Molina et al.* 31434 (MO).

**GUATEMALA.** Huehuetenango, Río Seligua, *L.O. Williams et al.* 41319 (BM, F, MO); Chiquimula, Esquipulas, *A Molina & A.R. Molina* 25159 (BM, F, MO).

**MEXICO. Chiapas**: Ixtapa, *R.M. Laughlin* 2157 (F); Pinola las Rosas, Teopisca, *D.E. Breedlove* 41154 (MO). **Oaxaca**: San Miguel Chimalapa. Río Portamonedas, *S. Maya* 2441 (MEXU).

#### Note.

Probably closely related to *Ipomoea neurocephala* but the corolla much larger and bracteoles narrowly oblong-ovate.

### 247. Ipomoea magnifolia

Taxon classificationPlantaeSolanalesConvolvulaceae

Rusby, Mem. Torrey Bot. Club 6: 84. 1896. (Rusby 1896: 84)

#### Type.

BOLIVIA. Cochabamba, Espirito Santo, *M. Bang* 1277 (lectotype NY 319197, designated by Wood et al. 2015: 97, isolectotypes NY, MO, K, US barcode 0111417).

#### Description.

Vigorous liana to 7 m, stems pubescent. Leaves petiolate, very large. 11–20 × 7–20 cm, ovate (rarely shallowly 3-lobed), acuminate to a fine point, cordate with rounded auricles, thinly to densely adpressed pubescent on both surfaces; petioles 5–15 cm, pubescent. Inflorescence of long-pedunculate, axillary, rather compact cymes; peduncles 8–30 cm, pubescent; bracteoles 10–11 mm, linear or filiform, finely acuminate, caducous; secondary peduncles 1–1.5(–10) cm; pedicels 3–14 mm, pubescent; sepals very unequal, somewhat variable in shape and size, outer sepals 12–17 × 4–5 mm, broadly lanceolate, acuminate, the tips usually recurved, pilose to glabrous, inner sepals 7–10 × 3–4 mm, oblong, obtuse or acute, sometimes mucronate, pilose to merely ciliate, margin scarious; corolla 7–9 cm long, mauve, funnel-shaped with broad tube, in bud pubescent but glabrescent later, limb 5–6 cm diam; stigma biglobose. Capsules and seeds not seen.

#### Illustration.

Figure 129.

**Figure 129.**
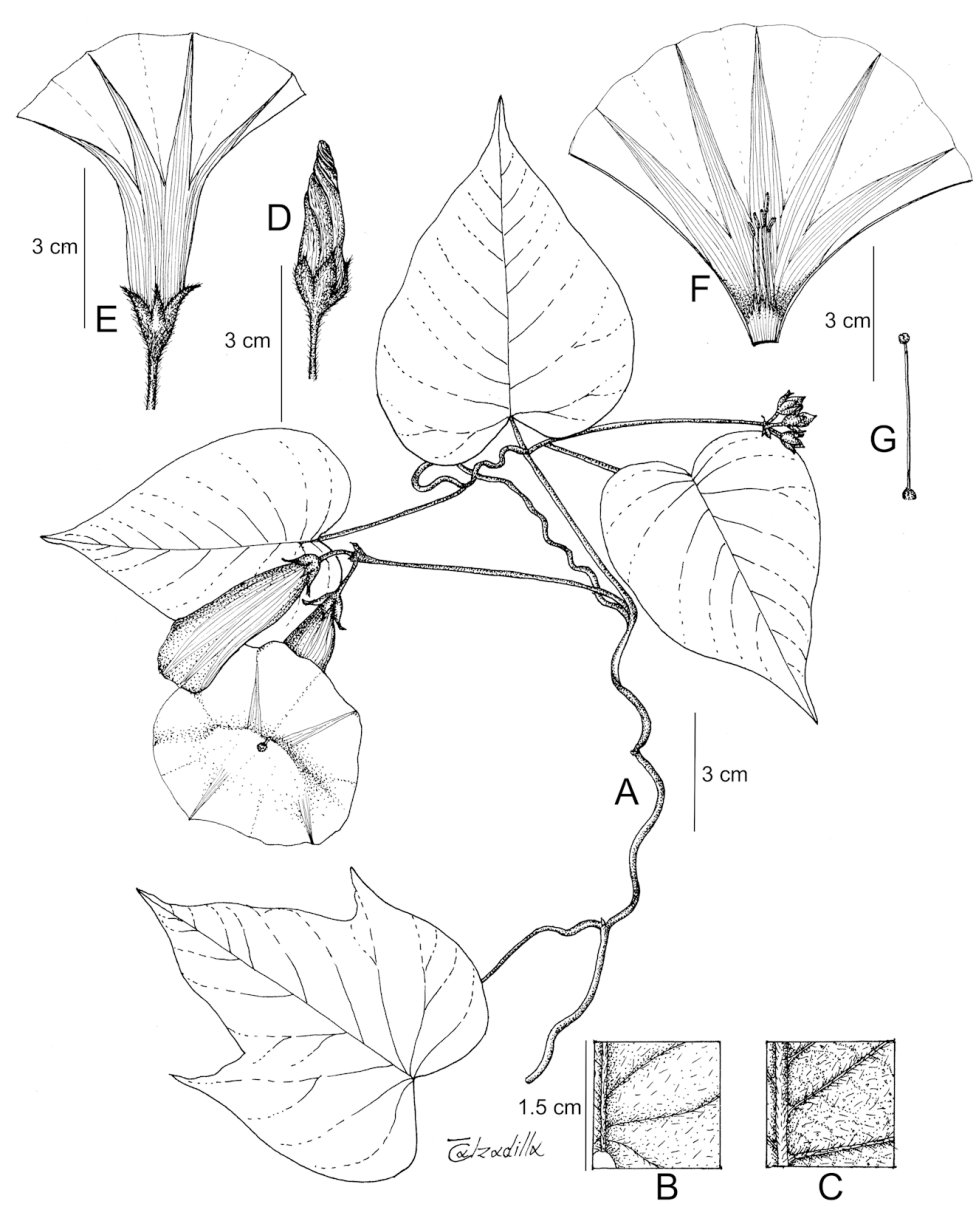
*Ipomoea magnifolia*. **A** habit **B** adaxial leaf surface **C** abaxial leaf surface **D** calyx and bud **E** calyx and corolla **F** corolla opened up to show stamens **G** ovary and style. Drawn by Eliana Calzadilla **A** from *Wood & Daniel* 18416; **B, C, F, G** from *Beck* 32903; **D, E** from *Solomon* 18838.

#### Distribution.

Endemic to moist Andean forest in northern Bolivia and southern Peru where it grows from 750 to 1900 m in the lower cloud forest region.

**BOLIVIA. Cochabamba**: Chapare, Locotol, 1800 m, April 1950, *M. Cardenas* 458 (LIL). **La Paz**: 5–10 km E of Caranavi on road to Alto Beni *J.R.I. Wood & T. Daniel* 18384 (K, LPB); Murillo, Valle de Zongo, *J. Solomon* 18838 (FTG, LPB, MO); Nor Yungas, 8 km from Coroico towards Coripata, *J.R.I. Wood & T. Daniel* 18416 (K, LPB); Saavedra, ANMI Apolobamba, *A. Fuentes et al.* 7073 (ARIZ, MO); Sud Yungas, Puente Villa, *S.G. Beck* 32903 (K, LPB); Tamayo, P.N. Madidi, *A. Fuentes et al.* 9300 (LPB, MO).

**PERU. Cusco**: La Convención, Echarate, Papelpata, *G. Calatayud et al.* 2972 (MO, OXF), 3769 (MO, OXF); Vilcabamba, Espiritopampa, *G. Calatayud et al.* 2590 (MO, OXF).

#### Notes.

Somewhat resembling a large-leaved *Ipomoea indica* but leaves never grey-tomentose beneath, bracteoles caducous, sepals very unequal, the inner oblong, much shorter than the outer and the inflorescence not usually compact.

*Ipomoea magnifolia* is very variable with respect to the indumentum of the sepals and the corolla, varying from subglabrous to pilose, although there is always the tendency for hairs to fall with age. *Fuentes et al.* 9300 (MO) is a very unusual specimen, the inflorescence is exceptionally long-pedunculate and very dense, the outer sepals are broadly oblong not lanceolate, obtuse and mucronate, not acuminate and a mere 10 mm long.

### 248. Ipomoea ampullacea

Taxon classificationPlantaeSolanalesConvolvulaceae

Fernald, Proc. Amer. Acad. Arts 33(5): 89. 1897. (Fernald 1897: 89)

#### Type.

MEXICO. Guerrero, Acapulco, *E. Palmer* 483 (holotype GH00054482, isotypes: K, US).

#### Description.

Liana with white latex, stem thinly pilose. Leaves petiolate, 9–14 × 8–14 cm, broadly ovate, shortly acuminate, cordate (often shallowly 3 –lobed), thinly hispid-pilose on both surfaces, abaxially paler; petioles 6–12 cm, thinly pilose. Inflorescence of long-pedunculate, axillary cymes; peduncles 11–20 cm, stout, straight; bracteoles narrowly ovate, acuminate, pubescent, caducous; secondary peduncles 1–2.5 cm; pedicels 6–22 mm, puberulent; sepals dissimilar, pubescent, 26–40 × 8–10 mm, outer ovate with an elongated obtuse apex, inner sepals narrower, slightly longer, with an elongate spathulate apex; corolla 6–8 cm long, subhypocrateriform with broad basal tube and spreading limb, white, opening at night, thinly pilose on midpetaline bands in bud, anthers exserted, filaments red, pubescent; stamens shortly exserted; stigma 3-lobed. Capsules broadly ovate, c. 2 cm long, glabrous; seeds not seen.

#### Illustration.

Figure 4F.

#### Distribution.

Endemic to Mexico growing in humid hill forest 650–2000 m.

**MEXICO. Colima**: lower slopes of Vulcan de Colima, *A.C. Sanders et al.* 10730 (MO).

**Guerrero**: Montes de Oca, Vallecitos, *G.B. Hinton* 11730 (ARIZ, GH, K, MO); Mun. Azueta, *J.C. Soto Nuñez* 11632 (MEXU). **Jalisco**: Mun. Puerto Vallarta, *E. Carranza et al*. 6130 (ARIZ); Mun. La Huerta, Chamela, *S.H. Bullock* 2060 (K, MO); Arroyo Colorado, Chamela, *E. Lott & T. Wendt* 2192 (K); Chamela, *A. Megallanes* 4151 (F). **Michoacán**: Mun. Lázaro Cárdenas, *E. Carranza & I. Silva* 6707 (IEB), 7277 (IEB). **Sonora**: fide Felger et al. (2012). **Sinaloa**: Africa, Sierra Tacuichamona, *H.S. Gentry* 5658 (ARIZ, MO); San Ignacio, *J.G. Ortega* 5022 (K).

#### Note.

Unique in the Pharbitis Clade for having white, night-flowering, presumably moth-pollinated flowers.

### 249. Ipomoea temascaltepecensis

Taxon classificationPlantaeSolanalesConvolvulaceae

P. Wilkin, Kew Bull. 50(1): 95. 1995. (Wilkin 1995: 95)

#### Type.

MEXICO. Est. México, Temascaltepec district, *G.B. Hinton et al.* 5316 (holotype K000612716, isotype GH).

#### Description.

Liana resembling *Ipomoea ampullacea* in habit, white latex and thinly retrose pilose indumentum. Leaves petiolate, 6.5–16 × 6–18 cm, broadly ovate, shortly acuminate, cordate (sometimes very shallowly 3-lobed), occasionally with marginal teeth, adaxially sparsely adpressed hispid-pilose, abaxially paler, more densely hirsute; petioles 4.5–8 cm, thinly pilose. Inflorescence of long-pedunculate, few-flowered axillary cymes; peduncles 7–28 cm, pubescent; bracteoles resembling small leaves, caducous; pedicels 5–22 mm, puberulent; sepals somewhat unequal, pubescent, outer 15–28 × 7–12 mm, ovate and gradually tapered to an acuminate apex, inner similar but lanceolate and 2–4 mm shorter; corolla 4.5–8 cm long, funnel-shaped, pink, pubescent; stamens included; stigma 3-lobed. Capsules globose. 10–15 mm, glabrous, shortly rostrate; seeds up to 6, 5.5–6 mm long, whitish-puberulent.

#### Illustration.

Figure 130.

**Figure 130.**
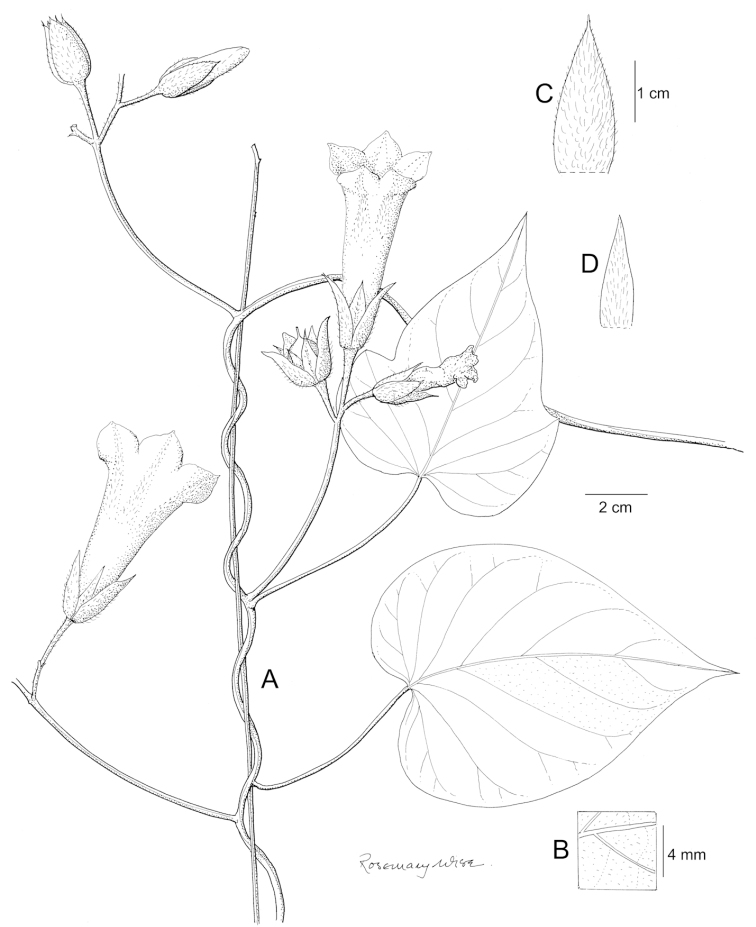
*Ipomoea temascaltepecensis*. **A** habit **B** abaxial leaf surface **C** outer sepal **D** inner sepal. Drawn by Rosemary Wise from *Hinton* 2928.

#### Distribution.

Endemic to the Temascaltepec region of Mexico State at around 1200 m.

**MEXICO. Est. México**: Temascaltepec, *G.B. Hinton et al.* 8258 (F, K, MO); ibid., Yperricones, *G.B. Hinton et al.* 341 (K); ibid., Pungarancho, *G.B. Hinton et al.* 4786 (K, BM, GH); ibid., Platanal, *G.B. Hinton et al.* 8590 (K, GH); ibid., Rincón del Carmen, *G.B. Hinton et al.* 8610 (K, GH).

#### Notes.

Essentially a locally evolved species related to *Ipomoea ampullacea* but with pink flowers adapted for insect pollination.

There is an unexpected record from Sonora (*T.R. Van Devender & A.L. Reina-G*. 99-548 (MO), which we have not seen.

### 250. Ipomoea mairetii

Taxon classificationPlantaeSolanalesConvolvulaceae

Choisy in A.P. de Candolle, Prodr. 9: 374. 1845. (Choisy 1845: 374)

Calonyction venustum M. Martens & Galeotti, Bull. Acad. Roy. Sci, Bruxelles 12 (2): 270. 1845. (Martens and Galeotti 1845: 270). Type. MEXICO. Tabasco, J.J. Linden 306 (holotype GENT n.v., isotype BR000006973339).
Ipomoea venusta (M. Martens & Galeotti) Hemsl. ex Godman & Salvin, Biol. Cent.-Amer., Bot. 2(11): 395. 1882. (Hemsley 1882: 395).

#### Type.

MEXICO. *Mairet* s.n. (holotype G-DC, not found).

#### Description.

Climbing or trailing liana to 7 m, stems stout, densely hirsute. Leaves petiolate, 8–20 × 7.5–16 cm, large, ovate-suborbicular, shortly acuminate, cordate with rounded auricles, adaxially thinly pubescent to strigose, abaxially densely grey-tomentose; petioles 2.5–4 cm densely pubescent. Inflorescence of few-flowered long-pedunculate axillary cymes; peduncles 1.5–20 cm, tomentose; bracteoles 2–3 × 0.5–1.5 cm, ovate to narrowly elliptic, obtuse, pubescent, persistent; secondary peduncles 0.5–3 cm; pedicels 5–15 mm, tomentose; sepals equal, 16–22 × 7–11 mm, oblong-ovate, tomentellous, obtuse, somewhat accrescent in fruit; corolla 4–5.5 cm long, pubescent, narrowly funnel-shaped, tube white, limb reddish purple, 5–6 cm diam.; stigma 3-lobed. Capsules subglobose, 1.2–1.5 cm, glabrous, six-seeded; seeds 5–7 mm, minutely puberulent.

#### Illustration.

McDonald (1994: 71).

#### Distribution.

Dry oak woodland below 1600 m from central Mexico south to Honduras.

**HONDURAS.** Comayagua, *A. & A.R. Molina* 34235 (MO).

**GUATEMALA.** Chimaltenango, *P.C. Standley* 80879 (F).

**MEXICO. Chiapas**: *Matuda* 18471 (MEXU); Santa Rosa, *Heyde & Lux* 4350 (K). **Durango**: *Mairet* 695 (MO). **Guerrero**: Mun. San Luis Acatlán, *E. M. Martínez & B. Morales* 3470 (MO); Mochitlán, Agua de Obispo, *H. Kruse* 963 (IEB). **Michoacán**: Chinicuila, *I.G. Hernández* s.n. [7/3/2009] (IEB). **Nayarit**: Mesa del Nayar, *O. Téllez et al.* 12138 (MO). **Oaxaca**: Putla de Guerrero, *T. Croat* 45854 (MO); Sierra San Pedro *C. Jürgensen* 551 (BM, K, OXF); Cafetal Concordia, *Morton & Makrinius* 2507 (US, MICH); San Miguel del Puerto, Rancho Oreeja de León, *J. Pascual* 2022 (IEB). **Sinaloa**: Ocarahui, Sierra Surutato, *H.S. Gentry* 6250 (ARIZ, MO). **Veracruz**: *Hahn* s.n. (P); Valle de Córdoba, *Bourgeau* 1738 (BM, K, P, S); Mirador, *J. Linden* 1119 (K); Orizaba, *J. Ball* s.n. (K); Zacualpan, *C.A. Purpus* 2391 (BM, MO).

#### Note.

Not unlike *Ipomoea temascaltepecensis* but more hirsute generally, the leaves tomentose beneath and sepals subequal.

### 251. Ipomoea invicta

Taxon classificationPlantaeSolanalesConvolvulaceae

House, Ann. New York Acad. Sci. 18(6): 193. 1908. (House 1908b: 193)

#### Type.

MEXICO. Jalisco, San Sebastián, *E.W. Nelson* 4087 (holotype US00111404, isotypes K, GH).

#### Description.

Liana climbing to 8 m, stems brown, strigose. Leaves petiolate, 6–14 × 4.5–11 cm, ovate, cordate, apex acuminate, mucronate, adaxially glabrous or nearly so, abaxially paler, thinly pubescent; petioles 4–6.5 cm, subglabrous to pubescent. Inflorescence of long-pedunculate dense, few-flowered, axillary cymes; peduncles 2.5–17 cm, subglabrous to pubescent; bracteoles 23–37 × 12–18 mm, ovate-elliptic, acuminate, cuneate at base, whitish-green with prominent veins, persistent; secondary peduncles 1.8 cm, stout; pedicels 5–10 mm, widened upwards; sepals unequal, outer 20–22 × 8–10 mm, narrowly elliptic, acute and mucronate, veins prominent, glabrous, inner sepals 13–20 × 4–5 mm, oblong-elliptic, noticeably smaller; corolla 6–7 cm long, glabrous, funnel-shaped, widened abruptly above a broad whitish basal tube, limb 7 cm diam., somewhat lobed, deep pinkish-purple; stigma 3-lobed. Capsules subglobose, 10 mm wide, enclosed by persistent sepals; seeds not seen.

#### Distribution.

A forest species endemic to central Mexico at 1100–1250 m.

**MEXICO. Guerrero**: NE del valle de Zaragoza, *E.M. Martínez & J.C. Soto* 3715 (MO); Montes de Oca, Vallecitos, *G.B. Hinton* 11766 (K). **Jalisco**: 22 km S of Talpa de Allende, *R. McVaugh* 23331 (MICH), foothills of Sierra de Manantlán, *R. McVaugh* 23246 (MICH).

### 252. Ipomoea lambii

Taxon classificationPlantaeSolanalesConvolvulaceae

Fernald, Bot. Gaz. 20: 535. 1895. (Fernald 1895: 535)

#### Type.

MEXICO. Nayarit, Tepic, *F.H. Lamb* 556 (holotype GH00054509, isotypes CAS, NY, US).

#### Description.

Perennial herb climbing to 4 m; stems thin, wiry, pubescent. Leaves petiolate, 6–17 × 4–15 cm, ovate, often shallowly 3-lobed, base cordate with rounded to acute auricles and a narrow sinus, apex acuminate, abaxially paler, thinly pubescent; petioles 1–8 cm. Inflorescence of compact 2–4-flowered pedunculate, axillary cymes; peduncles 1.7–15 cm; bracteoles 2–3.5 × 0. 5–1.2 cm, oblong-elliptic, boat-shaped, chartaceous; pedicels 5–15 mm, glabrous; sepals slightly unequal 15–20 × 10 mm, ovate, obtuse, mucronate, glabrous, the inner slightly shoerter and narrower; corolla 7–8 cm long, deep pink, funnel-shaped, thinly pubescent on midpetaline bands, limb c. 5 cm diam. Capsules and seed unknown.

#### Distribution.

A rare species of oak woodland in central Mexico between 1100 and 1300 m.

**MEXICO.** “Sierra Madre”, 1100 m, *Langlassé* 909 (P). **Guerrero**: *V.W. Steinmann & J.M. Porter* 4942 (IEB). **Michoacán**: Cerro Cumbitinda, Mun. Tingambato, *H. Díaz Barriga* 5176 (IEB).

#### Note.

Very similar to *Ipomoea invicta* but more pubescent, the flower buds noticeably hairy.

### 253. Ipomoea laeta

Taxon classificationPlantaeSolanalesConvolvulaceae

A. Gray, Proc. Amer. Acad. Arts 22: 439. 1887. (Gray 1887: 439)

#### Type.

MEXICO. Jalisco, Río Blanco, *E. Palmer* 341 (holotype GH00054508, isotypes BM, NY, MO, NDG, P, US, YU).

#### Description.

Climbing perennial, stems, leaves and other vegetative parts pubescent. Leaves petiolate, 2.5–4.5 × 3–6 cm, palmately lobed to near the base, lobes broadly to narrowly ovate-elliptic, acuminate to an acute apex, narrowed at base, leaf base cordate, abaxially whitish, sometimes sericeous; petioles 2–3.5 cm. Inflorescence of solitary axillary flowers; peduncles 5–8 cm; bracteoles 12–14 mm, oblong-lanceolate, finely apiculate, deciduous; pedicels 4–7 mm, densely pilose; sepals unequal, outer 17–20 × 7–10 mm, broadly ovate with rounded to truncate base, apiculate, pilose, inner 15–16 mm, obtuse to retuse, pilose only along midrib, margins broad, scarious; corolla 10–12 cm long, funnel-shaped, pink, pilose, limb entire, c. 9 cm diam.; stigma biglobose. Capsules and seeds not seen.

#### Illustration.

Figure 131.

**Figure 131.**
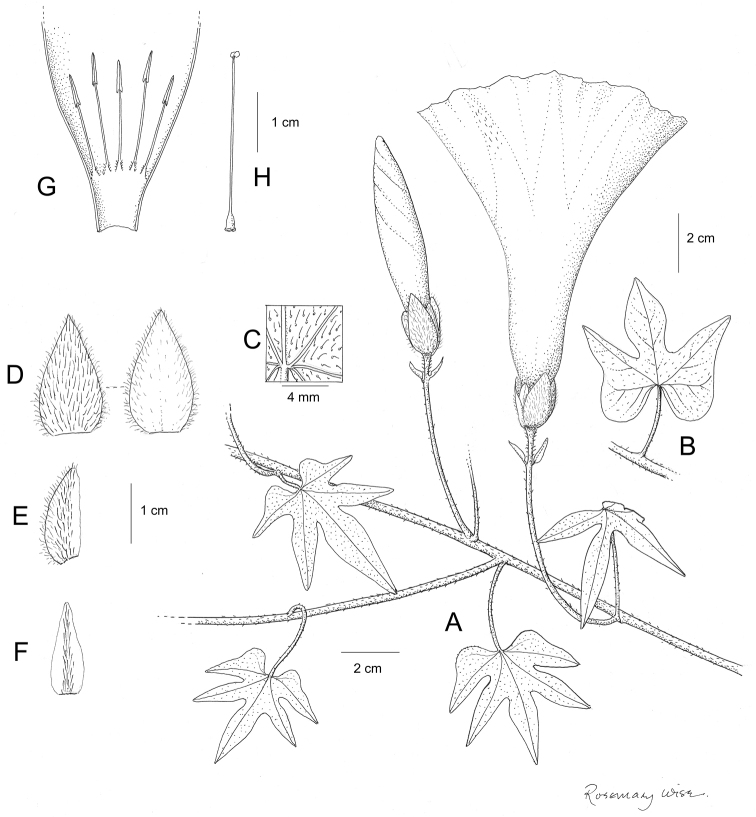
*Ipomoea laeta*. **A** habit **B** variation in leaf shape **C** abaxial leaf surface **D** outer sepal, abaxial surface (left), adaxial surface (right) **E** middle sepal **F** inner sepal **G** corolla open out to show stamens **H** ovary and style. Drawn by Rosemary Wise **A, B** from *Pringle* 10620; **C–H** from *Solbrig & Orduff* 4442.

#### Distribution.

Endemic to north western Mexico, growing in *Quercus* and *Pinus* woodland between 1000 and 1700 m.

**MEXICO. Chihuahua**: Río Mayo, Sierra Charuco, *H.S. Gentry* 1788 (F, S). **Coahuila**: East of 5 de Mayo, Viesca, *G.B. Hinton et al.* 28506 (GBH). **Jalisco**: Guadalajara, *C.G. Pringle* 4456 (BM, E, F, MO, P, S); Lago de Chapala, *O.T. Solbrig & R. Ornduff* 4442 (NY, UC); Zapotitan de Hidalgo, *D.P. Gregory & G. Eiten* 214 (MO, P); Zapopan, La Primavera, *A. Bourg* 139 (IEB); Ixtlahuacan del Rio, *Y. Hernandez Magaña et al.* 9441 (MEXU); La Huerta, Rancho Cuixmala, *E. J Lott* 2867 (UCR). **Nayarit**: Xalisco, *G. Flores et al.* 4025 (MO, n.v.); Cerro de San Juan, Tepic, *Y. Mexia* 684 (BM). **Sinaloa**: Com. La Guásima, Concordia, *M. Ruiz et al.* 2009-278 (ARIZ). **Sonora**: fide Felger et al. (2012).

#### Notes.

This species is usually easily recognised by its large carolla and the palmately-lobed discolorous leaves. However, as with many species in the Pharbitis Clade, the leaves may be entire or lobed. *Gentry* 1788 and *Ruiz et al.* 2009-278 differ from other specimens in having entire, strongly abaxially sericeous leaves.

Placement of this species in the Pharbitis Clade is provisional.

### 254. Ipomoea thurberi

Taxon classificationPlantaeSolanalesConvolvulaceae

A. Gray, Syn. Fl. N. Amer. 2: 212. 1878. (Gray 1878: 212)

Ipomoea gentryi Standl., Publ. Field Mus. Nat. Hist., Bot. Ser. 22: 46. 1940. (Standley 1940b: 46). Type. MEXICO. Chihuahua, Río Mayo, *H.S. Gentry* 2497 (holotype F0054842, isotypes ARIZ, MO).
Ipomoea sessilis L.O. Williams, Fieldiana, Bot. 32(12): 195. 1970. (Williams 1970a: 195). Type. GUATEMALA. Huehuetenango, *J. Steyermark* 51566 (holotype F0054897).

#### Type.

UNITED STATES. Arizona, *G. Thurber* 966 (holotype GH00054547).

#### Description.

Twining or trailing perennial from a thickened woody tuberous rootstock like a xylopodium; stems glabrous. Leaves petiolate; at least sometimes held at right angles to petiole, 1–5 × 2.5–6 cm, deltoid, finely acuminate and mucronate, margin undulate, base sagittate with basal auricles acute, sometimes bifurcate and leaves becoming ±5-lobed, thinly pilose on both surfaces; petioles 0.6–2.4 cm. Inflorescence of solitary, axillary flowers; peduncles 3–5 (–7) mm, sometimes muricate or with a few stipitate glands; bracteoles 1–2 mm, deltoid; pedicels 4–12 mm, thicker than peduncle and widened upwards; sepals equal, glabrous, 14–25 × 3–4 mm, narrowly lanceolate, acute to acuminate, mucronate, outer sometimes verrucose near base; corolla 5–9 cm long, flared, funnel-shaped, very gradually widened from a narrow basal tube, pale pink, glabrous, limb 5–6 cm diam.; ovary 3-locular. Capsules subglobose to ovoid, 6–7 mm, strongly rostrate with mucro 4–6 mm long, glabrous; seeds up to 6, c. 4 mm long, ovoid, dark brown, tomentellous.

#### Distribution.

A species with a strikingly disjunct distribution between Central America and the Sonora desert region that is very unusual and merits investigation. It is mostly found between 1100 and 1900 m in dry rocky areas in open oak woodland.

**NICARAGUA.** Hac. Corpus, Chontales, *W.D. Stevens* 22449 (MO).

**GUATEMALA.** Type of *Ipomoea sessilis*.

**MEXICO. Chihuahua**: *H.S. Gentry* 2612 (F, K); Nabogame, *J.E. Leferrière* 1612 (ARIZ, ASU, MEXU). **Durango**: Buenos Aires, Tepehuanes, *P. Tenorio & S. Romero* 1193 (MEXU). **Est. México & Dist. Fed.**: Temascaltepec, Chorrera, *G.B. Hinton* 4746 (K); ibid., *G.B. Hinton* 6502 (K). **Nayarit**: *G. Flores-Franco et al.* 2751 (MEXU). **San Luis Potosí**: *C.C. Parry & E. Palmer* 665 (P). **Sonora**: between Ures and Moctezuma, *N. Snow & T.P. Prinzie* 6594 (MO); Los Pilares, 23 km E de Yécora, *T. Van Devender et al.* 98-911 (ARIZ, ASU); Yécora, *A.L. Reina-G et al.*97-717 (MEXU).

**UNITED STATES. Arizona**: Huachuca Mts, *J.G. Lemmon* 2833 (BM, GH, K, P); Cochise Co., Canelo Hills, *G. Yatskievich* 80-347 (MO); Santa Cruz Co., Pena Blanca Lake-Sycamore Canyon, *D.F & S. Austin* 7603 (ARIZ, ASU).

#### Note.

Very characteristic are the solitary, very shortly pedunculate flowers, the gradually widened flared corolla and the long, narrow sepals.

• Species 255–257 (and more distantly 258) form a small clade of closely related species.

### 255. Ipomoea marginisepala

Taxon classificationPlantaeSolanalesConvolvulaceae

O’Donell, Lilloa 23: 490. 1950. (O’Donell 1950b: 490)

#### Type.

ARGENTINA. Tucumán, Dept. Tafí, Cerro Aconquija, *J.B. Sotelo* 415 (holotype LIL001262).

#### Description.

Relatively weak, probably annual, twining herb, glabrous in all parts. Leaves petiolate, 3–9 2.5–7 cm, ovate, cordate with rounded auricles, acuminate to a fine point, margins undulate; petioles 3–8(–12) cm, somewhat warted. Inflorescence of pedunculate, axillary cymes, often with only 2 fully developed flowers; peduncle relatively stout, 2–15 cm; bracteoles 1–3 mm, deltoid, fugacious; secondary peduncles 0.8–1.5 cm; pedicels mostly 20–30 mm, slightly swollen upwards; sepals subequal, 5–6 ×3 mm, oblong-lanceolate, acute, dark green with white margin; corolla 2.5–4 cm long, funnel-shaped, glabrous, tube white, yellowish inside, limb blue, c. 3 cm diam., unlobed. Capsules ovoid, 7 mm wide, 8 mm long, rostrate with a beak 3–5 mm long, glabrous; seeds 6–7 mm long, appearing glabrous but minutely tomentellous under a microscope.

#### Illustration.

O’Donell (1959b: 186).

#### Distribution.

Dry inter-Andean valleys of northern Argentina and southern Bolivia but scattered in occurrence and uncommon in both countries, growing between about 700 and 2000 m.

**ARGENTINA. Jujuy**: San Pedro, *A.L. Cabrera et al.* 30247 (SI); Candelaria, *S. Venturi* 3859 (LIL, SI); El Carmen, *L.J. Novara & S. Bruno* 9846 (G, S). **Salta**: Capital, Atocha, *L.J. Novara* 9668 (G, S); Rosario de la Frontera, *M. Lillo* s.n. (LIL, SI). **Santiago del Estero**: Guasayán, *S. Pierotti* s.n. [6/4/1944] (CORD, LIL). **Tucumán**: type collection.

**BOLIVIA. Chuquisaca**: Oropeza, Chuquichuqui, *J.R.I. Wood* 10904 (HSB, NY, K). **Potosí**: Charcas, Río Caine, *L. Rico & Windsor-Shaw* 1634 (K, MO, NY). **Santa Cruz**: Caballero, Saipina, *J. Balcazar* 367 (MO); Pulquina, *N. Biggs & D. Zappi* 70 (K, USZ); Cordillera, pie de la Muela del Diablo, *J.R.I. Wood et al.* 27631 (K, LPB, USZ); Vallegrande, Moro Moro, *J.R.I. Wood et al.* 27692 (K, LPB, USZ). **Tarija**: Gran Chaco, Villamontes-Palos Blancos, *J.R.I. Wood et al.* 27612 (K, LPB, USZ); O’Connor, Entre Ríos–Cañadas, *M. Coro* 1119 (LIL).

#### Notes.

Very similar to and possibly conspecific with *Ipomoea cardiophylla* A. Gray but molecular studies using *ITS* suggest the two species are distinct. Further sampling is needed to resolve these issues.

*Kessler et al.* 6119 (LPB) from Loma Larga towards Masicuri in Vallegrande Province (Bolivia) may belong here but differs in the presence of stiff trichomes on the calyx and in having somewhat toothed leaves. It requires further investigation and might represent a distinct species.

### 256. Ipomoea cardiophylla

Taxon classificationPlantaeSolanalesConvolvulaceae

A. Gray, Syn. Fl. N. Amer., ed. 2, 2: 213. 1886. (Gray 1886: 213)

#### Type.

UNITED STATES. Texas, near El Paso, *C. Wright* 511 (holotype GH, isotype K).

#### Description.

Twining annual herb, stems glabrous. Leaves petiolate, 2–6 ×1.3–3.8 cm, ovate, cordate with rounded auricles, narrowed to an obtuse, mucronate apex, margin entire, both surfaces glabrous and green; petioles 1.5–6.5 cm. Inflorescence of 1–5-flowered, axillary cymes; peduncles 1–3 mm on new shoots, up to 8 cm on older shoots, stout; bracteoles caducous; pedicels 12–14 mm, becoming reflexed in fruit; sepals subequal, 4–6 ×2–4 mm, ovate-deltoid, very acute, glabrous, margins scarious, white; corolla 2.5–2.7 cm long, funnel-shaped, blue drying pink with pale tube, glabrous, limb 3–3.5 cm diam. Capsules very large, ovoid, 10–12 ×8–12 mm, rostrate, glabrous; seeds 5–6 ×3 mm, shortly and finely puberulent.

#### Distribution.

In semi-desert in the United States southwest and northern and central Mexico.

**MEXICO. Chihuahua**: *C.G. Pringle* 617 (BM, K, P). Coahuila, 25 miles SW of Monclava, *E. Palmer* 904 (K, P); near Rancho Cerro de la Madera, *T. Wendt* 1780 (ASU). **Durango**: Mapimí, *A. Herrera* 1 (IEB). **Guanajuato**: Xichú, *S. Zamudio & J. Becerra* 11623 (IEB); ibid., Ca. De Huamuchil, *J. Rzedowski* 52929 (IEB). **Hidalgo**: Tecozautla, *S. Rojas* 378 (IEB). **Michoacán**: Cuitzeo, *E. Carranza & I. Silva* 7255 (IEB). **Nuevo León**: *G.B. Hinton* 21674 (GBH). **Oaxaca**: *V. González & G. Conzatti* 898 (GH). **Querétaro**: Salida a San Luis de Potosí, *E. Argüelles* 276 (MEXU, NY); Mun. Corregiodora, *L. Hernández* 6536 (IEB). **San Luís de Potosí**: Villa Juárez, *S. Zamudio* 3817 (IEB). **Sonora**: Sierra Anibácachi, SW of Agua Prieta, *T.R. Van Devender et al.* 2004-117 (ARIZ). **Tamaulipas**: San Nicholás, *M. Martínez* 5057 (IEB). **Veracruz**: Zacuapan, *C.A. Purpus* 4320 (BM, F, GH, US).

**UNITED STATES. Arizona**: Cochise Co., Tombstone, *D.F. & S. Austin* 7608 (ASU); *S. Walker* s.n. (UTC); Santa Cruz, *W. Hodgson* 3913 (DES). **New Mexico**: Grant, Silver City, *A.D. Zimmerman* 2006 (DES). **Texas**: Trans Pecos Mountains region fide Correll and Johnston (1970).

#### Notes.

Very similar to *Ipomoea marginisepala* in all characteristics and difficult to separate except geographically, although molecular studies suggest the two species are distinct. In the type only, the peduncles are suppressed.

This species is often confused with and sometimes treated as a synonym of *Ipomoea aristolochiifolia* (Austin 1982a: 38) but is readily distinguished by the lanceolate to ovate, acute unwarted sepals and by the peduncle which does not pass through the leaf sinus.

### 257. Ipomoea tricolor

Taxon classificationPlantaeSolanalesConvolvulaceae

Cav., Icon 3: 5, t. 208. 1795. (Cavanilles 1795–96: 5)

Convolvulus venustus Spreng., Syst. Veg. 1: 600. 1825 [pub. 1824]. (Sprengel 1824: 600). Type. Based on Ipomoea tricolor Cav.
Ipomoea hookeri G. Don, Gen. Hist. 4: 274. (Don 1838: 274), nom. illeg. superfl. for Ipomoea tricolor Cav.
Ipomoea rubrocaerulea Hook., Bot. Mag. 8: t. 3297. 1834. (Hooker WJ 1834a: t. 3297). Type. Cultivated plant from Guanajuato, MEXICO. Richardson s.n., not preserved, lectotype t. 3297 in Bot. Mag., designated by McDonald (1994: 121).
Convolvulus rubrocaeruleus (Hook.) D. Dietr., Syn. Pl. 1: 670. 1839. (Dietrich, D 1839: 670).
Pharbitis rubrocaerulea (Hook.) Planch., Fl. Serres Jard. Eur. 9: 281, t. 966. 1854. (Planchon 1854: 281).
Ipomoea schiedeana Ham., Edwards’s Bot. Reg. 24: Misc. 19. 1838. (Lindley 1838a: 19), nom. illeg., non Ipomoea schiedeana Zucc. (1831). Type. Cultivated plant from MEXICO. Schiedes.n., not preserved, lectotype drawing by Nairn (OXF), designated by McDonald (1994: 121).
Ipomoea violacea auct. mult. (non L.)

#### Type.

[cultivated plant from Mexico], *Cavanilles* s.n. (lectotype MA475860, designated here).

#### Description.

Twining annual herb, glabrous in all parts, stems robust and often thick (4–5 mm broad). Leaves petiolate, 3–12 × 2–10 cm, ovate, cordate with rather angular, nearly rounded auricles, apex acuminate, both surfaces glabrous; petioles 1.5–11 cm. Inflorescence of pedunculate, few-flowered axillary cymes; peduncles 3–20 cm; bracteoles 1–2 mm, oblanceolate, early caducous; secondary peduncles 0.5–2.5 cm; pedicels 1.5–3 cm, spreading at a wide angle; sepals subequal, 5–7 × 3 mm, oblong-lanceolate, acute, dark green with white margin, inner slightly longer than the outer; corolla 5–7.5 cm long, funnel-shaped, glabrous, tube white, yellowish inside, limb blue, 4 cm diam. Capsules 10 × 6 mm, ovoid, glabrous, rostrate; seeds 7 × 3 mm, blackish, appearing glabrous but minutely tomentellous under a microscope.

#### Illustration.

Figure 6B; Acevedo-Rodríguez (2005: 180).

#### Distribution.

Usually presumed to be of Mexican origin, but widely cultivated as an ornamental plant, even in temperate countries, and the following citations mix cultivated plants with garden escapes, adventives on roadsides and weeds of disturbed areas. It rarely appears truly native even in central Mexico.

**BRAZIL. Minas Gerais**: *H. Mello Barreto* 5170 (F, SP). **São Paulo**: *J. Santoro* 589 (LIL, SP).

**BOLIVIA. La Paz**: Calacota, *J. Solomon* 18363 (LPB, MO); Inquisivi, Licoma, *J.R.I. Wood et al.*29179 (LPB, USZ). **Santa Cruz**: Florida, Pampa Grande, *M. Nee & M. Mendoza* 52929 (MO, NY); Ichilo, Buenavista, *J.R.I. Wood & D. Soto* 27960 (USZ).

**PERU. Ayacucho**: *C. Vargas* 15676 (CUZ).

**ECUADOR. Loja**: *G. Harling* 6006 (MO, S); Catamayo valley, *C. Huttel* 1980 (QCA, QCNE).

**COLOMBIA. Antioquia**: Medellín, *J. Triana* s.n. (BM, P). **Cundinamarca**: Fusagasugá, *E. André* 1601 (K).

**VENEZUELA. Aragua**: *A. Fendler* 2087 (K, MO). **Dist. Fed.**: Caracas, *Moritz* 491 (BM); Lara: Barquismeto, *F. de la Puente* 784 (OXF). **Mérida**: *J. de Bruijn* 1345 (K, MO, S, WAG).

**COSTA RICA.** Alajuela, *M. Chavarría* 726 (K, MO).

**NICARAGUA.** Río Grande, *J.T. Atwood & P. Mena* 2484 (BM, GH, MO, NY); Estelí, Pueblo Nuevo, *L.O. Williams & A. Molina* 42399 (BM, F); *W.D. Stevens* 26630 (MO).

**GUATEMALA.** Casillas, Santa Rosa, *Heyde & Lux* 4352 (BM); *J. Donnell Smith* 4352 (K).

**MEXICO. Campeche**: *E.F. & H. Cabrera* 10862 (MEXU). **Chiapas**: Motozintla, *D.E. Breedlove* 40546 (MO). **Guanajuato**: León, *E. Carranza & I. Silva* 6276 (IEB). **Guerrero**: Adama Temisco, Cerro de Otote, *Y. Mexia* 8863 (MO, S); Teloloapan, *J.C. Soto Nuñez* 19892 (MEXU); Zihugio, Mina, *G.B. Hinton* 9723 (K). **Hidalgo**: Tasquillo, *R. Hernández & D. Rodríguez* 4982 (MO). **Jalisco**: Chapala, *E. Palmer* 702 (BM, K); ibid., *W.B. Gourlay* 62 (K); La Unión, *J.C. Soto Nuñez et al.* 11275 (MEXU); ibid., 12515 (K). **Michoacán**: Morelia, *G. Arsène* s.n. [18/8/1910] (K); Coalcomán, *G.B. Hinton* 12496 (GBH, K, MO). **Morelos**: *Fröderström & Hultén* 483 (S), 406 (S); Cuernavaca, *E. Bourgeau* 1409 (K, P). Miacatlan, *G. Flores & E. Cabrera* 648 (MEXU). **Oaxaca**: *J. Tournon* 564 (P). **Puebla**: Coxcatlán. *J.I. Calzada* 24297 (K). **Querétaro**: *E. Argüelles* 2797 (IEB). **Veracruz**: *C.M. Rosas* 745 (BM). **Yucatán**: Izamal, *G.F. Gaumer* 329 (BM, K); Silam, *G.F. Gaumer* 1661 (BM, K, S).

**UNITED STATES. Colorado**: *S. Peck* 193 (KHD). **Missouri**: *J. Sheets* 104 (SEMO). **Texas**: *L.H. Shinners* 9445 (FSU).

**CUBA. Pinar del Río**: *J. Bissé & C. Schez* (HAJB51411).

**DOMINICAN REPUBLIC.***E.J. Valeur* 272 (K, NY, S); *A.H. Liogier* 13861 (NY), 17790 (NY).

**PUERTO RICO.***N.L. & E.G. Britton* 9117 (NY).

**LESSER ANTILLES. U.S. Virgin Islands**: St Croix: fide Acevedo-Rodríquez (2005); St John: *P. Acevedo-Rodríguez* 3119 (MO, NY). **Antigua**: *H.E. Box* 1341 (BM). **Guadeloupe**: *A. Duss* 3591 (NY).

### 258. Ipomoea barbatisepala

Taxon classificationPlantaeSolanalesConvolvulaceae

A. Gray, Syn. Fl. N. Amer., ed. 2, 1: 212. 1886. (Gray 1886: 212)

#### Type.

USA, Texas, *C. Wright* 507 (holotype GH00054451, isotypes BM, GH, K, US).

#### Description.

Slender twining annual herb, stems glabrous. Leaves petiolate, 3–9 × 3–9 cm in outline but usually small, palmately divided into 3 lobes, shallowly cordate to truncate and briefly cuneate onto petiole, the terminal lobe lanceolate acuminate, narrowed at base, the 2 lateral lobes forked or trifurcate, glabrous, both surfaces green. Inflorescence of few-flowered pedunculate cymes, flowers often solitary; peduncles 1.5–5 cm, recurving in fruit; bracteoles 2 mm, linear-lanceolate, scarious with green midrib; pedicels 6–15 mm, lateral flowers often developing tardily; sepals subequal, 9–12(–15) × 2–3 mm, linear-lanceolate, acuminate, densely covered in stiff bristles c. 3 mm long; corolla 1.6–2.3 cm long, the tube white, glabrous, the limb bluish-purple, c. 2 cm diam., unlobed but midpetaline bands terminating in a tooth. Capsules subglobose 9 × 10 mm, glabrous; seeds up to 6.5 × 2.5 mm, appressed pubescent often appearing glabrous, brown.

#### Variation.

We formally recognise two varieties that were previously treated as distinct species.

### 258a. Ipomoea barbatisepala var. barbatisepala

Taxon classificationPlantaeSolanalesConvolvulaceae

#### Diagnosis.

Distinguished by the lanceolate sepals with stiff spreading bristles.

#### Illustration.

Figure 4E.

### 258b. Ipomoea barbatisepala var. angustata

Taxon classificationPlantaeSolanalesConvolvulaceae

(Choisy) J.R.I. Wood & Scotland, comb. & stat. nov.

urn:lsid:ipni.org:names:77208076-1

Ipomoea angustata Brandegee, Univ. Calif. Publ. Bot. 4(19): 383. 1913. (Brandegee 1913: 383). Type. MEXICO. Sinaloa, Culiacan, *T.S. Brandegee*s.n. (holotype UC105148).

#### Diagnosis.

Distinguished by the narrow linear-lanceolate, glabrous sepals.

#### Distribution of species.

Locally common between 200 and 2400 m in the Sonora Desert of Southern Arizona, but uncommon and scattered in other semi desert areas of northern Mexico and the United States southwest.

**MEXICO. Baja California Sur**: Comondú, *A.M. Narvaez* 2012-209 (HCIB). **Guerrero**: *J. Calónico Soto* 17769 (MEXU). **Jalisco**: *Montes & Salazar* 874 (FTG). **Michoacán**: *J. Soto Nuñez* 10918 (MEXU). **Oaxaca**: Santa Maria de Tule, *W.G. D’Arcy* 11973 (FTG, MO). **Sinaloa**: El Potrerillos, *J.G. Ortega* 874 (K). **Sonora**: Yécora, *T.R. Van Devender* 97-1016 (ARIZ, MEXU). **UNITED STATES. Arizona**: Apache Pass, *J.G. Lemmon* 439 (BM, P); Pima County, *J. Tedford* 06-255, (ARIZ); *W. Hodgson* 23418 (DES); Santa Cruz county, *W. Hodgson et al*. 15772 (DES). **New Mexico**: Loma County, Tres Hermanas Mts., *R.D. Worthington* 19947 (DES, FTG); Florida Mountains, *R.D. Worthington* 18612 (L). **Texas**: El Paso, Franklin Mountains, *R.D. Worthington* 14686 (DES).

#### Note.

*Ipomoea barbatisepala* appears superficially to be a relative of *Ipomoea nil* but the capsule is 4-seeded and molecular studies place it close to *I. tricolor*. The linear-lanceolate sepals with stiff spreading hairs are distinct but these are absent in the type of *Ipomoea angustata*. This has never been recollected but is superficially very distinct and is recognised as var. angustata.

• Species 259–267 form a small clade but lack any clear common morphological character. The presence of two species with an unusual ovary structure is noteworthy.

### 259. Ipomoea chiriquensis

Taxon classificationPlantaeSolanalesConvolvulaceae

Standl., Ann. Missouri Bot. Gard. 27: 334. 1940. (Standley 1940a: 334)

#### Type.

PANAMA. Upper valley of Río Chiriquí, *P.H. Allen* 1512 (holotype MO152718, isotypes GH, L, US).

#### Description.

Liana 3–6 m high, stems glabrous, latex present, white. Leaves petiolate, 10–19 × 9–11 cm, ovate, abruptly shortly acuminate, cordate, glabrous; petioles 7–14 cm. Inflorescence of pedunculate, axillary cymes of 2–6 flowers; peduncles 8–10 cm; bracteoles caducous, not seen; secondary peduncles 2 cm; pedicels 30–50 mm; sepals unequal, glabrous, outer 7–10 × 5 mm, oblong-ovate, acuminate, inner 12–15 × 7–8 mm, ovate to broadly oblong, rounded, mucronate, margins broad, scarious; corolla 6–9 cm long, midpetaline bands terminating in a tooth, white, glabrous, limb 7–8 cm diam.; stamens at mouth. Capsules 1.5 cm long, ovoid, glabrous; seeds glabrous.

#### Illustration.

Austin (1975b: 206).

#### Distribution.

Apparently rare localised to western Panama and Costa Rica in moist hill forest around 1800–2000 m.

**PANAMA.** Chiriqui: Nueva Suissa, *T.B. Croat* 13504 (MO).

**COSTA RICA.***A. Tonduz* 11701 (MO). San José, Cordillera de Talamanca, Copey de Dota, *M.M. Chavarria* 1069 (K, MO).

#### Note.

This species is characterised by the long-peduncled, few-flowered cymes of white flowers with broad oblong-ovate, mucronate, mostly scarious inner sepals.

### 260. Ipomoea decasperma

Taxon classificationPlantaeSolanalesConvolvulaceae

Hallier f., Bull. Herb. Boiss. 5: 386. 1897. (Hallier 1897a: 386)

Ipomoea oreophila House, Ann. New York Acad. Sci. 18: 195. 1908. (House 1908b: 195). Type. MEXICO. Hidalgo, Lena Station, *C. G. Pringle* 10034 (holotype GH00054522, isotypes BM, CM, F, K, M, MEXU, NY, S, US).
Ipomoea emetica auct.

#### Type.

MEXICO. [Jalisco], Zacoalco, Valley of Mexico, *E. Bourgeau* 797 (lectotype G00342886 ex Herb. DC, designated here; isolectotypes P, S).

#### Description.

Twining perennial to 1 m from a large root tuber, stems pubescent. Leaves petiolate, 2–8.5 × 1–5.3 cm, ovate-panduriform to subreniform, base cordate to subsagittate, auricles rounded to subacute, somewhat spreading, apex acute to obtuse, mucronate, margin undulate or with 1–2 large lateral teeth or 3–5-lobed, sparsely pubescent on both surfaces, abaxially paler; petioles 1.2–5 cm, pubescent. Inflorescence of solitary axillary flowers; peduncles 2–5.2 cm, pubescent; bracteoles 2–4 mm, linear, tardily deciduous; pedicels 5–14 mm, pubescent; sepals slightly unequal, pubescent, strongly accrescent in fruit; outer 7–10 × 4–7 mm, ovate-deltoid with a broad truncate to subcordate base, acuminate, inner c. 1 mm longer, narrower and basally cuneate; corolla 3.5–4 cm long, funnel-shaped, nearly glabrous but with a few hairs towards the apex of the midpetaline bands, tube whitish, limb deep pink, limb c. 3 cm diam. Capsules 10–12 mm, globose, 5-locular with up to ten seeds; seeds lentil-shaped, 4 mm, densely pubescent with short stiff hairs.

#### Distribution.

Endemic to central Mexico growing in secondary *Quercus* woodland at 1900–2500 m.

**MEXICO. Durango**: *E. Palmer* 592 (BM, K, S); Súchil, *S. González & Y. Herrera* 1341(MEXU). **Est. México y Dist. Fed.**: Valley of Mexico, *A. Schmitz* 108 (BM, W); Mun. Huehuetoca, *J. Rzedowski* 34330 (FTG, MEXU); Temascaltepec, *G.B. Hinton* 6525 (F), 8442 (K), 8454 (K). **Guanajuato**: Pénjamo, La Loma, *E. Pérez & J. Becerra* 4009 (IEB, MO); San Felipe, Los Altos de Ibarra, *R. & J.D. Galvín* 2298 (IEB); Coroneo, *E. Carranza* 5345 (IEB, MEXU). **Hidalgo**: type of *Ipomoea oreophila*. **Jalisco**: Zacoalco, Vale of Mexico, *E. Bourgeau* 497 (P, G), 728 (K, P, S), 792 (G, P); **Michoacán**: Morelia, *G. Arsène* 3486 (G, MO), 5972 (G, MO); ibid., Cerro del Aguila, *G.C. Tenorio et al.* 2247 (IEB, K, MEXU). **Querétaro**: Amealco de Bonfil, *E. Carranza & I. Silva* 6180 (IEB, MEXU); Huimilpan, *E. Argüelles* 2613 (IEB, MEXU).

**Notes**. Very distinct because of the 10-seeded capsule, leaf shape and truncate-based outer sepals. A record from Sonora: fide Felger et al. (2012) seems unlikely.

O’Donell annotated specimens of this species as *Ipomoea emetica* Choisy and was followed in this by Austin and Huáman (1996). The case for rejecting the name, *I. emetica* in favour of *I. decasperma* was made by Wood and McDonald (2018).

### 261. Ipomoea orizabensis

Taxon classificationPlantaeSolanalesConvolvulaceae

(G. Pelletan) Ledeb. ex Steud., Nomencl. Bot. 1: 818. 1840. (Steudel 1840: 818)

Convolvulus orizabensis G. Pelletan, J. Chim. Méd. 10: 11. 1834. (Pelletan 1834: 11). Type. MEXICO. Veracruz (lectotype, icon. in J. Chim. Méd. 10: 11. t. 2, designated by McDonald (1994: 85).
Convolvulus serotinus DC., Cat. Pl. Horti Monsp. 97. 1813. (Candolle 1813: 97), non Ipomoea serotina Roem. & Schult. (1819). Type. MEXICO. Sine data. (holotype MPU013593).
Ornithosperma serotina (DC.) Raf., Fl. Ludov.: 149 (1817). (Rafinesque 1817: 149).
Quamoclit serotina (DC.) G. Don, Gen. Hist. 4: 259. 1838. (Don 1838: 259).
Pharbitis serotina (DC.) Choisy in A.P. de Candolle, Prodr. 9: 341. 1845. (Choisy 1845: 341).
Ipomoea tyrianthina forma serotina (DC.) Voss, Vilmorins Blumengärtn. 711. 1894. (Voss 1894–96: 711).
Convolvulus superbus Kunth, Nov. Gen. Sp. 3: 103. 1818 [pub. 1819]. (Kunth 1819: 103). Type. MEXICO. entre Aguscarco & montañas de Jorullo, Humboldt & Bonplands.n. (holotype P00670754).
Ipomoea superba (Kunth) G. Don, Gen. Hist. 4: 275. 1838, (Don 1838: 275), nom. illeg, non Ipomoea superba Ledeb. (1822).
Convolvulus sanguineus Willd. ex Roem. & Schult. Syst. Veg. 3: 302. 1819, (Roemer and Schultes 1819: 302), non Ipomoea sanguineaVahl (1794). Type. MEXICO. Jonello & Toluca, Humboldt & Bonplands.n. (holotype B-W03704).
Ipomoea tyrianthina Lindl., Edwards’s Bot. Reg. 24: 87. (Misc. 162). 1838. (Lindley 1838c: 87). Type. Cultivated from seeds collected by Dickson (holotype CGE00071, isotype K).
Pharbitis tyrianthina Hook., Bot. Mag. 69: t. 4024. 1843. (Hooker 1843: t. 4024)
Pharbitis longipedunculata Martens & Galetti, Bull. Acad. Roy. Sci. Bruxelles 12(2): 271. 1845. (Martens and Galeotti 1845: 271). Type. MEXICO. Hidalgo, dans les bois de Sabino, *H. Galeotti* 1387 (holotype BR00006972677, isotypes K, P).
Ipomoea longipedunculata (Martens & Galeotti) Hemsl., Biol. Cent.-Amer., Bot. 2(11): 389. 1882. (Hemsley 1882: 389).
Pharbitis lilacina Schltdl. ex Kunze, Linnaea 20: 31. 1847. (Kunze 1847: 31), non Ipomoea lilacinaBlume (1825–26) . Type. A plant sent by Ehrenberg from Mexico and cultivated at Halle (not found).

#### Type.

Based on *Convolvulus orizabensis* G. Pelletan

#### Description.

Twining perennial, stems pilose to glabrous, becoming muricate to spinulose when old. Leaves petiolate, 3–13 × 2.5–11 cm, ovate, entire or 3–5-lobed, cordate with rounded auricles and very narrow sinus, shortly acuminate or cuspidate, mucronate, usually pubescent or hirsute at least on the margins and abaxial veins, occasionally glabrous abaxially paler; petioles 2.5–7 cm, usually pubescent. Inflorescence of 1–5-flowered, axillary, pedunculate cymes; peduncles 2–15 cm; bracteoles 2–10 mm, filiform; pedicels 10–35 mm, commonly reflexed in fruit; sepals slightly unequal, lanceolate to ovate or elliptic, finely acuminate, shortly mucronate, glabrous, pubescent or villous, the margins white, scarious, outer (4–)11–18 × 4–5 mm, the inner usually slightly shorter, the scarious margins broader; corolla 5.5–7.5 cm long, funnel-shaped, glabrous, the tube pale, midpetaline bands ending in a mucro, the limb purple, 5–6 cm diam. Capsules ovate, 10–13 × 6–8 mm, glabrous; seeds 4–5 mm long, rounded, puberulent.

#### Variation.

Very variable in indumentum from glabrous to pubescent or hirsute in varying degrees. The sepals too vary from being subequal or the outer or inner slightly longer, the apex usually acuminate but sometimes obtuse. The leaves may be ovate or 3–7-lobed. *Pringle* 8737 has unusually finely acuminate sepals. This species was divided into four varieties by McDonald (2001) and molecular studies (Keith et al. 2017, Muñoz-Rodríguez et al. 2019) suggest *I. orizabensis* consists of more than one taxa. However it is unclear to date whether or not the current infraspecific classification is congruent with the molecular evidence. In any case we believe these varieties merit the status of geographical subspecies and have accordingly changed their status. The four subspecies can be distinguished by the following key:

**Table:** 

1	Leaves 3–7-lobed	**subsp. collina**
–	Leaves entire	**2**
2	Leaves hirsute	**subsp. orizabensis**
–	Leaves glabrous	**3**
3	Leaves with a distinctive cuspidate apex; inner sepals almos entirely scarious	**subsp. novogaliciana**
–	Leaves shortly acuminate; inner sepals with narrow scarious margins	**subsp. austromexicana**

### 261a. Ipomoea orizabensis subsp. orizabensis

Taxon classificationPlantaeSolanalesConvolvulaceae

#### Diagnosis.

Stems, leaves and sepals hirsute. Sepals mostly > 10 mm long, with broad scarious margins, the outermost somewhat foliose. Leaves entire, cordate.

#### Illustration.

Figure 132.

**Figure 132.**
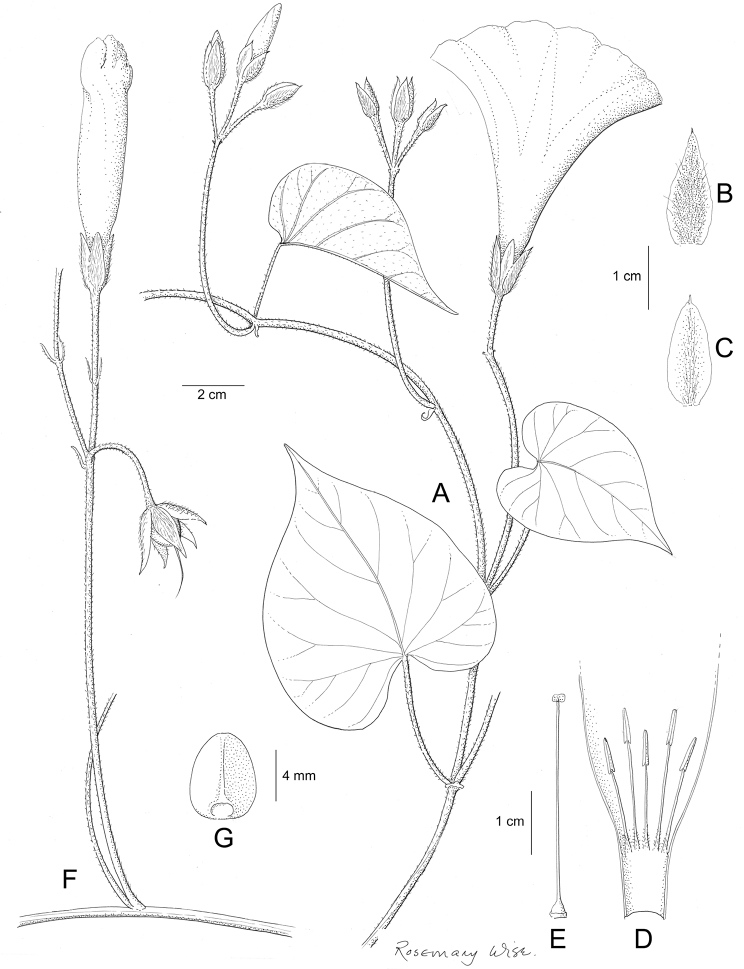
*Ipomoea orizabensis***A** habit **B** outer sepal **C** inner sepal **D** corolla opened out to show stamens **E** ovary and style **F** fruiting inflorescence with capsules **G** seed. Drawn by Rosemary Wise **A** from *Heyde & Lux* 3189; **B–G** from *Meyer & Rogers* 3027.

#### Distribution.

The common subspecies of scrubby hillslopes mostly between 1900 and 2500 m extending from central Mexico south to Honduras.

**HONDURAS.** Morazán, Laperterique, *A. & A.R. Molina* 25855 (MO).

**GUATEMALA.** Quiché, San Miguel Uspantan, *Heyde & Lux* 3189 (F, GH, K); *P.C. Standley* 82406 (F).

**MEXICO. Aguascalientes**: *R. McVaugh* 16635 (MICH). **Chiapas**: Motozintla, *P. J. Stafford et al.* 249 (BM, MEXU, MO); San Cristóbal, *A. Méndez* 8341 (MEXU). **Coahuila**: Saltillo, *J. Gregg* 321 (MO); Melchior Múzquiz, *J.A. Villarreal et al.* 8710 (MEXU). **Colima**: Rancho El Jabali, *A.C. Sanders et al.* 8516 (MO). **Durango**: El Indio, *P. Tenorio et al.* 9732 (MEXU, MO). **Est. México & Dist. Fed.**: Temascaltepec, *G.B. Hinton et al.* 8007 (K); Pedregal, *C.G. Pringle* 6452 (BM, K, MEXU, MO, S); Toluca, *C.G. Pringle* 8432 (BM, K, MO); Valle de México, *E. Bourgeau* 495 (K, P); Amecameca, *C.A. Purpus* 1755 (BM); San Andrés, *E. Lyonnet* 474 (BM, MEXU, MO). **Guanajuato**: Victoria, *J. Rzedowski* 44744 (IEB, MEXU, MO); San José Iturbide, *J. Gutiérrez* 194 (MEXU). **Guerrero**: Mina, Tierras Blancas, *G.B. Hinton* 9728 (K, MO). **Hidalgo**: Puerto Ignacio Isidro Díaz, *D.L. Spellman et al.* 1059 (MO). **Jalisco**: Guadalajara, *C.G. Pringle* 4448 (BM, K); Río Blanco, *E. Palmer* 335 (BM, K). **Michoacán**: Zitacuaro, *G.B. Hinton* 11922 (K); Sierra Torricillas, *G.B. Hinton* 12339 (K); Uruapan, *G.B. Hinton* 15461 (K); Morelia, *G. Arsène* 521 (K). **Morelos**: Cuernavaca, *E. Halbinger* s.n. [3/9/1977] (MEXU); ibid., *G.B. Hinton* 17457 (K). **Nayarit**: *R. McVaugh* 18713 (MICH); Nayar, *G. Flores et al.* 1718 (MEXU). **Nuevo León**: Zaragoza, Cerro El Viejo, *F. Meyer & D.J. Rogers* 3027 (BM, MO); Monterey, *C.G. Pringle* 8737 (BM, K, MEXU, S). **Oaxaca**: Cerro San Felipe, *C. Conzatti* 1608 (F); Mitla, *R. Torres et al.* 6980 (MEXU). **Puebla**: Puerto del Aire, *T.S. Elias et al.* 1144 (MO); Azumbilla, *P. Tenorio* 17521 (MEXU). **Querétaro**: Landa de Matamoros, El Madroño, *E. Carranza & E. Pérez* 5410 (IEB, MEXU). **San Luís Potosí**: Álvarez, *E. Palmer* 2045 (MEXU, MO); km 87, El Milagro, *S.M. Mertz* 126 (MEXU). **Sinaloa**: Sierra Surotato, *H.S. Gentry* 6220 (GH, MO, NY). **Tabasco**: Macuspana, *R.J. & C. Taylor* 12569 (MO). **Tamaulipas**: Tlaxcala, *E.K. Balls* 4837 (BM, CAS, K). **Veracruz**: Orizaba, *Seaton* 256 (F, GH, NY).

### 261b. Ipomoea orizabensis subsp. collina

Taxon classificationPlantaeSolanalesConvolvulaceae

(House) J.R.I. Wood & Scotland stat. nov.

urn:lsid:ipni.org:names:77208077-1

Ipomoea collina House, Bot. Gaz. 43(6): 412. 1907. (House 1907b: 412). Type. MEXICO. Coahuila, *E. Palmer* 396 (holotype US471266, isotypes CAS, F, GH, K, MO, NY, UC).
Ipomoea orizabensis var. collina (House) J.A. McDonald, Lundellia 4: 87. 2001. (McDonald 2001: 87).
Ipomoea batatoides Benth., Pl. Hartw. 46. 1840. (Bentham 1839–57: 46), nom. illeg., non Ipomoea batatoidesChoisy (1838). Type. MEXICO. [Hidalgo], Mestitlán, *K.T. Hartweg* (K000612737).
Ipomoea mestitlanica Choisy in A.P. de Candolle, Prodr. 9: 389. 1845. (Choisy 1845: 389). Type. Based on I. batatoides Benth.

#### Type.

Based on *Ipomoea collina* House

**Diagnosis**. Leaves 3–7-lobed, the segments narrowly oblong in outline, narrowed at both ends.

#### Illustration.

Carranza (2007: 89) (includes variations in leaf shape).

#### Distribution.

Principally in the drier areas of northern Mexico, especially the Sonora desert.

**MEXICO. Coahuila**: Cuatrociénagas, Sierra de San Marcos, *E. Carranza et al.* 1667 (IEB); Ramos de Arizbe, Sierra de la Paila, *J.A. Villarreal* 3923 (IEB). **Guanajuato**: Jaral del Progreso, *Schumann* 941 (P); sine loc., *Schnee* s.n. (P). **Hidalgo**: Type of *Ipomoea batatoides* Benth. **Sonora**: Sierra de Parras, *C.G. Purpus* 4975 (BM, F, GH, MO). Also Chihuahua, Nuevo León, Tamaulipas and Zacatecas fide McDonald (2001: 87).

### 261c. Ipomoea orizabensis subsp. austromexicana

Taxon classificationPlantaeSolanalesConvolvulaceae

(J.A. McDonald) J.R.I. Wood & Scotland stat. nov.

urn:lsid:ipni.org:names:77208078-1

Ipomoea orizabensis var. austromexicana J.A. McDonald, Lundellia 4: 86. 2001. (McDonald 2001: 86). Type. MEXICO. Chiapas, San Andrés Larrainzar, Summit of Chuchil Ton, *D.E. Breedlove* 29283 (holotype MEXU00252964).

#### Type.

Based on Ipomoea orizabensis var. austromexicana J.A. McDonald

**Diagnosis.** Distinguished by the glabrous leaves and sepals, which are relatively short (< 8 mm long), broadly elliptic to deltoid, the scarious margins very narrow.

#### Distribution.

Extreme western Guatemala to the Mayan highlands of central Chiapas, growing mostly between 1500 and 2000 m.

**GUATEMALA.** Sacatepequez, *P.C. Standley* 64686 (F).

**MEXICO. Chiapas**: Pinabeto, *E. Matuda* 15477 (F); La Independencia, *D. E. Breedlove* 33479 (MEXU).

### 261d. Ipomoea orizabensis subsp. novogaliciana

Taxon classificationPlantaeSolanalesConvolvulaceae

(J.A. McDonald) J.R.I. Wood & Scotland stat. nov.

urn:lsid:ipni.org:names:77208079-1

Ipomoea orizabensis var. novogaliciana J.A. McDonald, Lundellia 4: 87. 2001. (McDonald 2001: 87). Type. MEXICO. Michoácan, carretera de Periban a Buenavista, N. Soto 2451 (holotype MEXU00363973, isotype ENCB).

#### Type.

Based on Ipomoea orizabensis var. novogaliciana J.A. McDonald

**Diagnosis.** Distinguished by the small glabrous leaves, 3–5 × 2–3.5 cm, truncate or very shallowly cordate at base and the apex subcuspidate with an elongate prominent acuminate tip. The sepals are relatively short (4–6 mm), the inner sepal broadly elliptic, entirely scarious except for the green midrib. The corolla is relatively short, 3–6 cm long.

#### Illustration.

Figure 133.

**Figure 133.**
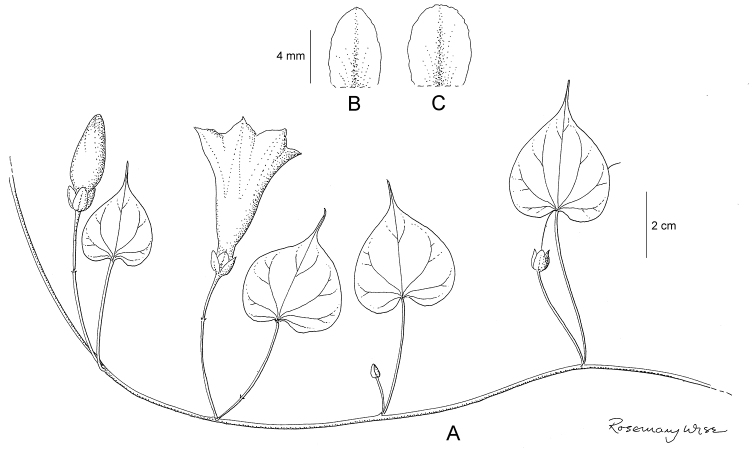
Ipomoea orizabensis subsp. novogaliciana**A** habit **B** outer sepal **C** inner sepal. Drawn by Rosemary Wise from *Fuentes* 612.

#### Distribution.

Uncommon in central Mexico.

**MEXICO.**). **Jalisco**: Tecalitlán, *M. Fuentes* 612 (MICH). **Michoacán**: near Rincón, *G. Arsène* 5489 (MEXU, MO); near Morelia, *G. Arsène* 5946 (GH, MEXU, NY).

### 262. Ipomoea gilana

Taxon classificationPlantaeSolanalesConvolvulaceae

K. Keith & J.A. McDonald, Syst. Bot. 42: 974. 2017. (Keith et al. 2017: 974)

#### Type.

UNITED STATES. New Mexico, 9 km N. of junction of State Highway 152 and Forest Service Road 157, *K. Keith* 12 (Holotype TEX, isotype UNM).

#### Description.

Perennial twining herb with stems up to 2 m long from a tap root 1–6 cm long. Leaves petiolate, 3–8 × 3–7 cm, entire or 5–7-lobed, lobes elliptic 1–6 × 0.5–2 cm, base cordate, apex acuminate, glabrous apart from the pubescent veins; petioles 3–9 cm, thinly pilose. Inflorescence of solitary axillary flowers, opening at night; peduncles 0–7 cm; bracteoles linear, 5 × 1 mm, linear, persistent; pedicels 15–25 mm, becoming recurved in fruit; sepals subequal, 11–14 × 3–5 mm, somewhat accrescent by 2 mm in fruit, ovate, acute or acuminate, outer adpressed pilose with scarious margins, inner glabrous, scarious; corolla narrowly funnel-shaped, 6–7 cm long, tube white, limb pale blue, limb 6–7.5 cm diam.; stamens shortly exserted or at mouth. Capsules ovoid, c. 15 × 15 mm, glabrous, trilocular; seeds (4–)6, black, 4–6 mm long, glabrous.

#### Distribution.

Endemic to open forest of *Pinus* and *Quercus* spp. in the Black Range in Gila National Park at 2045 m.

**UNITED STATES. New Mexico**: *K. Keith & C. Hunter* 2 (UNM).

#### Note.

A night-flowering species with pale blue flowers and shortly exserted stamens.

### 263. Ipomoea leucotricha

Taxon classificationPlantaeSolanalesConvolvulaceae

Donn.-Sm., Bot. Gaz. 23: 10. 1897. (Donnell Smith 1897: 10)

#### Type.

GUATEMALA. *E.W. Nelson* 3512 (holotype US00111412, isotype F).

#### Description.

Twining perennial to 4 m, stems silvery-canescent when young, somewhat glabrescent. Leaves petiolate, 6.5–12 × 5.5–10 cm, ovate-orbicular, cordate with rounded auricles, apex obtuse but terminating in a mucro up to 5 mm long, margin undulate, sometimes lobed, adaxially thinly adpressed pilose, abaxially silvery-canescent; petioles 2.5–4 cm, grey-pubescent. Inflorescence of pedunculate, often dense, axillary cymes; peduncles 4–5(–10) cm, sericeous; bracteoles linear, 7–14 × 1–2 mm, sericeous, deciduous; secondary and tertiary peduncles 5–12 mm; pedicels 5–11 mm, grey-sericeous; sepals unequal, outer ovate 8–10 × 3 mm long, including a fine recurving mucro 3–4 mm long, densely tomentose, inner 10–13 × 4 mm, ovate, acuminate, the apex usually erect, margins scarious but thinly tomentose; corolla 5–7 cm long, funnel-shaped, purple, sericeous, limb 5 cm diam., weakly lobed. Capsules unknown.

#### Illustration.

Figure 134.

**Figure 134.**
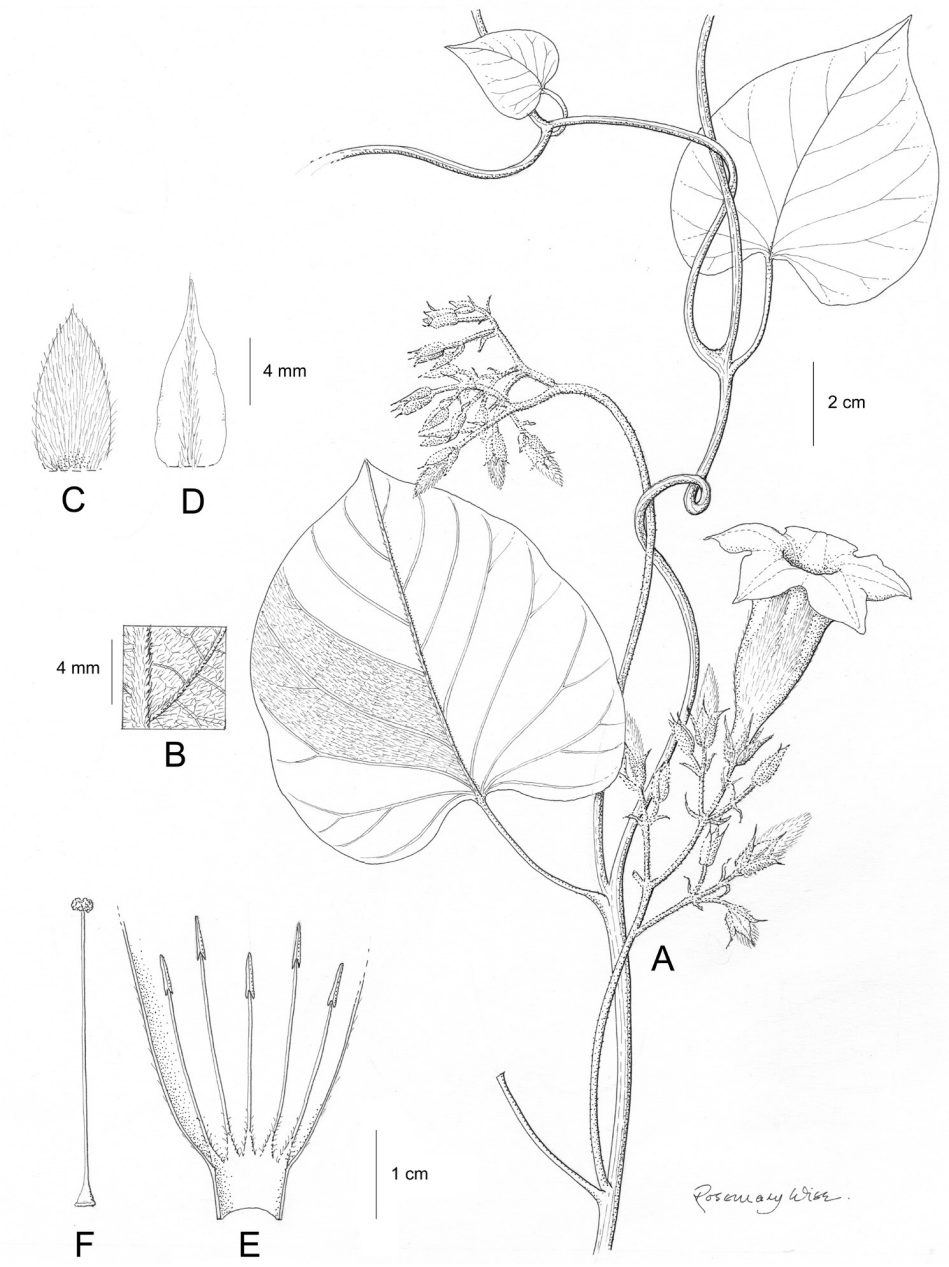
*Ipomoea leucotricha*. **A** habit **B** abaxial leaf surface **C** outer sepal **D** inner sepal **E** corolla opened out to show stamens **F** ovary, style and stigma. Drawn by Rosemary Wise **A** from *Moore* 2107; **B–F** from *Wilkin* 434.

#### Distribution.

Disturbed deciduous forest, 800–1200 m, in Central America, apparently uncommon.

**COSTA RICA.** Puntarenas, Monte Verde, *W.A. Haber* 4050 (FTG); Monteverde-San Luis, *P. Wilkin* 434 (BM).

**NICARAGUA.** Jinotega, *A.D. Moore* 2107 (BM, FTG, MO); Llano el Pozo, Estelí, *P.P. Moreno* 19329 (MO).

**GUATEMALA.** Type collection.

**MEXICO. Chiapas**: Mun. San Fernando, Tuxtla-Gutierrez-Chicoasen Dam, *D.E. Breedlove* 41474 (ARIZ, MO).

### 264. Ipomoea tuboides

Taxon classificationPlantaeSolanalesConvolvulaceae

O. Deg. & Ooststr., Fl. Hawaii, fam. 307. 1940. (Degener 1932–1940: fam. 307)

Ipomoea tuboides var. pubescens O. Deg. & Ooststr. in O.Deg., Fl. Hawaiiensis, fam. 307. 1940. (Degener 1932–1940: fam. 307). Type. HAWAII. Big Island, between Pu’awa’awa’a and Hu’ehe’e, *O. Degener* 6006 (holotype BISH1006750).
Ipomoea tuboides forma irregularis O. Deg. & Ooststr. [as var. pubescens forma irregularis] in O. Deg., Fl. Hawaiiensis, fam. 307. 1940. (Degener 1932–1940: fam. 307). Type. HAWAII. Big Island, between Pu’awa’awa’a and Hu’ehe’e, *O. Degener* 6020 (holotype BISH1006751, isotype BISH).
Ipomoea tuboides forma digitata O. Deg. & Ooststr. [as var. pubescens forma digitata] in O. Deg., Fl. Hawaiiensis, fam. 307. 1940. (Degener 1932–1940: fam. 307). Type. HAWAII. Big Island, between Wai’ohinu and Ka’alu’alu, *O. Degener* 5988 (holotype BISH1006753, isotype BISH).

#### Type.

HAWAII. Oahu, *O. Degener & Y. Nitta* 5981 (holotype BISH1006749; isotypes BISH, F, GH, MASS, MO, NY, S, US, WIS).

#### Description.

Prostrate or twining perennial, stems slender, woody below, glabrous except for small green protuberances. Leaves petiolate, 4–7 × 4–6 cm, ovate, obtuse or acute and mucronulate, cordate, margin entire, toothed, sinuate or 3-lobed, green and glabrous on both surfaces; petioles 2.3–4.7 cm, false stipules sometimes present. Inflorescence of solitary pedunculate, axillary flowers; peduncles 1–1.5 cm; bracteoles 5–6 mm, caducous; pedicels 1.5–2.5 cm; sepals slightly unequal, outer 7–8(–15) × 3–4 mm, oblong-elliptic, glabrous, margin scarious, becoming reflexed in fruit, inner 9–10(–22) mm, obtuse to mucronate with broad scarious margins; corolla white with lilac tinge, weakly salverform, the basal cylindrical tube narrow, 2.5 cm long and 0.75 cm wide, the limb 4.5–5 cm wide, glabrous, stamens included. Capsules ovoid-conical, glabrous; seeds trigonous, pubescent with woolly marginal hairs.

#### Illustration.

http://www.starrenvironmental.com/images/search/?q=Ipomoeatuboides

#### Distribution.

Endemic to the Hawaiian Islands where it grows on lava flows.

**UNITED STATES. Hawaii**: *J. Lau & C. Cory* 2498 (BISH, FTG); Moloka’i, Kamakou Reserve, *L.W. Cuddihy* 1218 (BISH). Lanai, *G. Munro* 945 (BM, K); Maui, *F.R. Fosberg* 48346 (K, US); *O. Degener* 25100 (BISH, K); Oahu, *O. Degener* 5978 (K), 27905 (E).

#### Note.

This Hawaian endemic is of considerable interest as molecular studies (Muñoz-Rodríguez et al. 2019) show its nearest relatives are *Ipomoea retropilosa*, *I. chenopodiifolia* and *I. leucotricha*, and, more distantly, *I. decasperma* and *I. orizabensis*. *Ipomoea tuboides* presumably arrived in Hawaii as a result of long-distance dispersal and subsequently evolved into a distinct species.

The floral dimensions in the protologue are much larger than in the specimens we have examined.

This is one of a number of species in which extrafloral nectaries have been reported (Keeler 1985) but this is something of an evolutionary curiosity in this case as there are no native ants in Hawaii.

### 265. Ipomoea retropilosa

Taxon classificationPlantaeSolanalesConvolvulaceae

(Pittier) D.F. Austin, Ann. Missouri Bot. Garden 64(2): 337. 1977 [pub. 1978]. (Austin 1978a: 337)

Exogonium retropilosum Pittier, J. Wash. Acad. Sci. 21: 143. 1931. (Pittier 1931: 143). Type. VENEZUELA. Mérida, Timotes, *H. Pittier* 12698 (holotype VEN12090, isotypes F, G, MO, US).
Ipomoea chenopodiifolia *sensu*Austin and Huáman (1996).

#### Type.

Based on *Exogonium retropilosum* Pittier

#### Description.

Trailing or scrambling liana of unknown size, stems woody, glabrous to scabrid-pilose, sometimes postulate. Leaves petiolate, 3–9 × 2– 7 cm, ovate, abruptly narrowed to an acuminate, mucronate apex, base shallowly cordate, both surfaces adpressed pilose with whitish hairs to glabrous; petioles 4–6.5 cm, pubescent. Inflorescence of few-flowered, pedunculate axillary cymes, primary peduncle stout, slightly woody, 1.3–6 cm, roughly pubescent, secondary peduncles 0.3–1 cm; bracteoles 6 × 1 mm, linear, pubescent, caducous; pedicels 15–32 mm, pubescent or glabrous, slightly thickened upwards; sepals subequal, outer 6–8 × 5–7 mm, broadly ovate, acute and shortly mucronate, glabrous to pilose; inner sepals c. 1 mm longer, glabrous or a broad line of hairs along the middle, margins scarious; corolla tubular, ± hypocrateriform, glabrous, the tube 3.5–4.5 cm long, c. 7–8 mm wide, dark, limb 3.5–4.5 cm wide, unlobed, magenta, stamens shortly exserted. Capsules 7 × 8 mm, subglobose, glabrous, rostrate; seeds not seen.

#### Variation.

We recognise two subspecies:

### 265a. Ipomoea retropilosa subsp. retropilosa

Taxon classificationPlantaeSolanalesConvolvulaceae

#### Diagnosis.

Sepals thinly to densely pilose on the abaxial surface. Young stems and abaxial leaf surface thinly to densely pubescent.

#### Illustration.

Figure 135.

**Figure 135.**
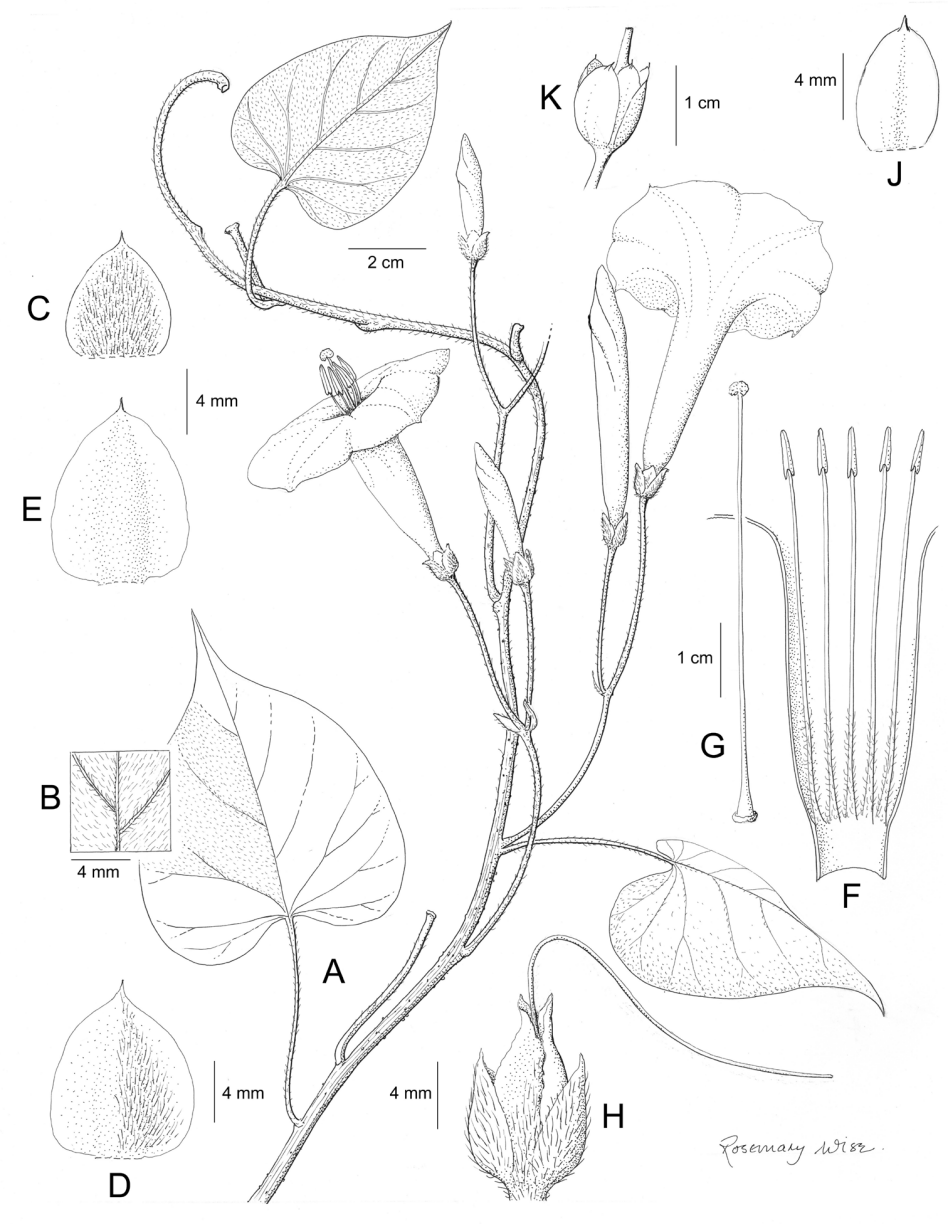
**A–H**Ipomoea retropilosa subsp. retropilosa. **A** habit **B** abaxial leaf surface **C** outer sepal **D** middle sepal **E** inner sepal **F** corolla opened out to show stamens **G** ovary, style and stigma **H** young fruiting calyx. **J, K**I. retropilosa subsp. cundinamarcana. **J** outer sepal **K** calyx with rostrate apex to capsule. Drawn by Rosemary Wise **A–H** from *J. B. Simmons* 281; **J, K** from *André* s.n.

#### Distribution.

In cloud forest near streams in the coastal sierra of Venezuela between 1500 and 1800 m approximately.

**VENEZUELA. Aragua**: Colonia Tovar, *Moritz* 1686 (BM, K); Ricaurte, 3 km E of Colonia Tovar, *J.A. Steyermark & R.L. Liesner* 121997 (MO). **Mérida**: south of Timote[s], 1976, *J.B. Simmons* 281 (K); **Trujillo**: Varela, 1500 m, *L. Aristeguieta* 4884 (MO).

### 265b. Ipomoea retropilosa subsp. cundinamarcana

Taxon classificationPlantaeSolanalesConvolvulaceae

J.R.I. Wood & Scotland, Kew Bull. 72 (10): 16. 2017. (Wood and Scotland 2017b: 16)

#### Type.

COLOMBIA. Cundinamarca, Quebrada el Chico, al norte de Bogotá, 2700–2800 m, 30 Nov. 1952, *H. Humbert, J. Idrobo & R. Jaramillo* 27532 (holotype P03538230).

**Diagnosis.** Sepals completely glabrous. The young stems and abaxial surface of the leaves are also glabrous.

#### Illustration.

**Figure 136.**
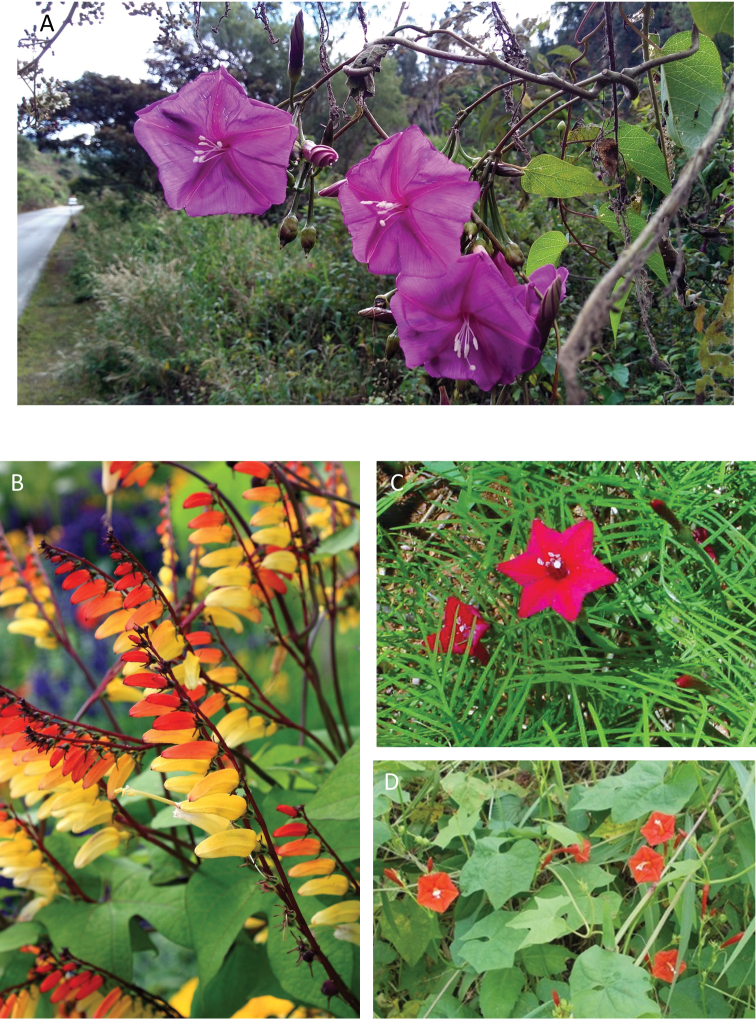
Photographs of *Ipomoea* species **A**I. retropilosa subsp. cundinamarcana**B***I. lobata***C***I. quamoclit***D***I. hederifolia*. **A** Jhon Infante-Betancourt **B** Alamy Ltd. **C** Ramona Oviedo **D** Amed Pupo.

Figures 135J–K, 136A.

#### Distribution.

Cloud forest in the Eastern Cordillera of Colombia.

**COLOMBIA. Boyacá**: Tunja-Ramiriqui, *J. Infante-Betancour* s.n. (COL). **Cundinamarca**: type of subsp. cundinamarcana. **Meta**: “Villavicencio,” [1875-6], *E. André* 137 (K).

#### Note.

This species has been confused with the rather similar *Ipomoea chenopodiifolia* of Mexico and Guatemala but differs in the shape and size of the sepals, which are subequal, broadly ovate, never more than 9 mm long, rather than distinctly unequal, lanceolate to narrowly ovate with the inner sepals up to 13 mm long. Austin (1982b: 187) added to the confusion by distinguishing *Ipomoea retropilosa* on the grounds that it had a funnel-shaped corolla, although in fact it has a hypocrateriform corolla as can be easily seen on the Geneva isotype, which appears to be the only duplicate of the type with a corolla.

### 266. Ipomoea chenopodiifolia

Taxon classificationPlantaeSolanalesConvolvulaceae

(M. Martens & Galeotti) Hemsl., Biol. Cent.-Amer., Bot., 2: 385. 1882 (Hemsley 1882: 385)

Calonyction chenopodiifolium M. Martens & Galeotti, Bull. Acad. Roy. Sci. Bruxelles 12: 269. 1845. (Martens and Galeotti 1845: 269). Type. MEXICO. Oaxaca, Yavezia, *H. Galeotti* 1375 (holotype BR00006972929).

#### Type.

Based on *Calonyction chenopodiifolium* M. Martens & Galeotti

#### Description.

Trailing or scrambling liana, 2–4 m high, stems woody, glabrous or thinly pubescent. Leaves petiolate, 3–12 × 2–10.5 cm, ovate, acute to shortly acuminate, shallowly cordate, thinly pubescent to glabrous on both surfaces, abaxially prominently veined; petioles 3–5 cm, pubescent or glabrous. Inflorescence of few-flowered, pedunculate, axillary cymes; peduncles stout, woody, 6–18 cm long, bifariously pubescent; bracteoles caducous, not seen; secondary peduncles 1–1.7 cm; pedicels 15–25 mm, slightly thickened upwards, glabrous to pubescent; sepals unequal, outer 7–9 × 2–3 mm, lanceolate, acute, glabrous, inner 10–12 × 4–5 mm, oblong-ovate, obtuse to rounded, the margins broad, scarious; corolla variable in shape from hypocrateriform to funnel-shaped, the tube 3.5–4.5 cm long, c. 7–8 mm wide, limb 3.5–4.5 cm wide, unlobed, deep pink or magenta, stamens included to shortly exserted. Capsules 10–13 mm, conical, glabrous, rostrate; seeds 7–8 mm, shortly pubescent.

#### Illustration.

Figure 137.

**Figure 137.**
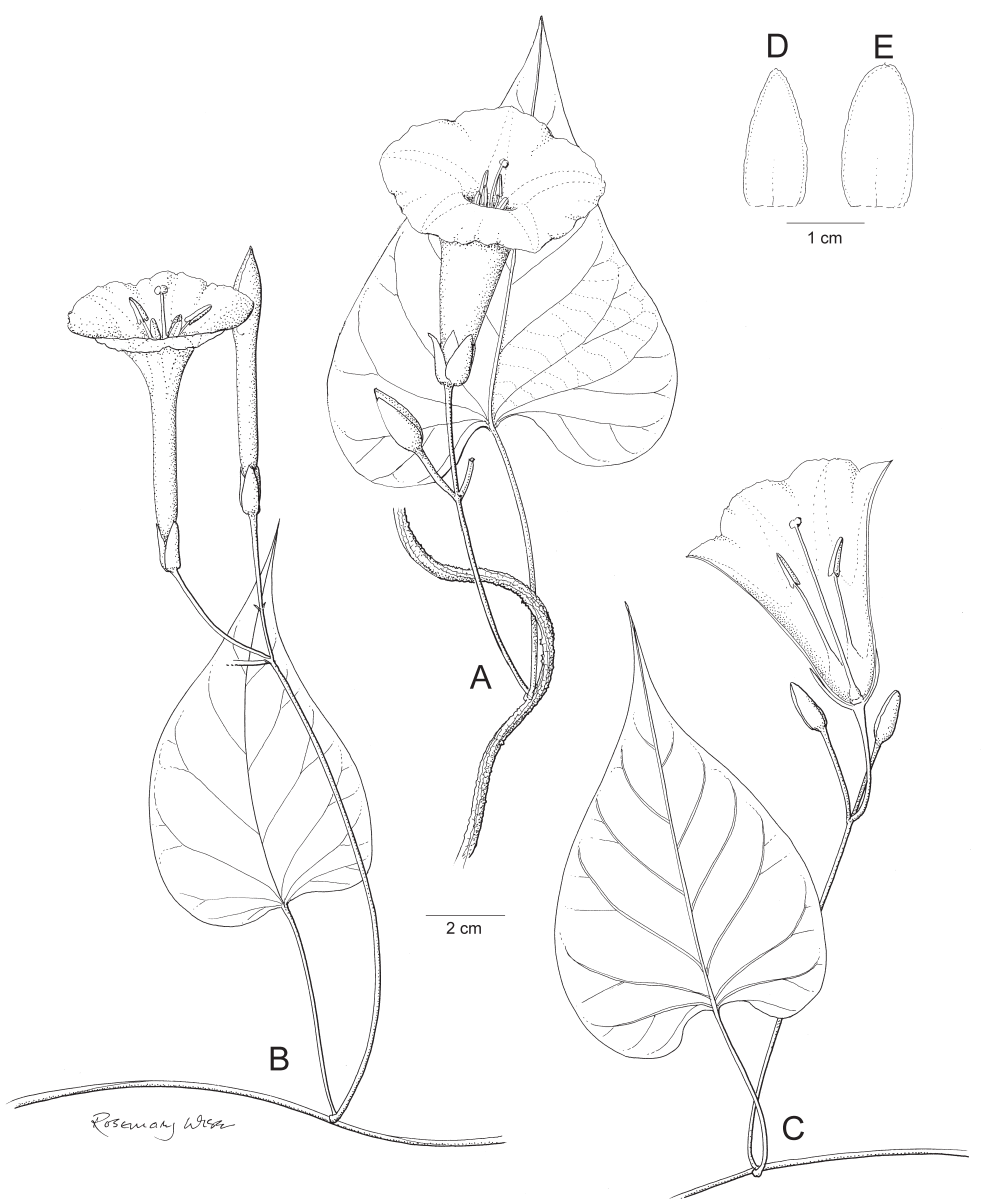
Ipomoea chenopodiifolia subsp. chenopodiifolia**A** habit showing corolla shape and exsertion of stamens and style. Subsp. signata**B** habit showing corolla shape and exsertion of stamens and style. Subsp. bellator**C** habit showing included stamens and style **D** outer sepal **E** inner sepal. Drawn by Rosemary Wise **A** from *Olazo* 1132; **B** from from *Martínez & García* 22197; **C** from *Nuñez* 11826; **D, E** from *de Avila* 143.

#### Variation.

This species can be divided into three subspecies based on corolla shape, exsertion of stamens and geographical distribution:

**Table:** 

1	Corolla tube cylindrical, scarcely widened upwards, < 7 mm wide at summit	**subsp. signata**
–	Corolla tube widened upwards, > 10 mm wide at summit	**2**
2	Stamens shortly exserted from corolla tube, corolla hypocrateriform, c. 15 mm wide at summit	**subsp. chenopodiifolia**
–	Stamens included in corolla tube; corolla funnel-shaped, > 2 cm wide at summit	**subsp. bellator**

### 266a. Ipomoea chenopodiifolia subsp. chenopodiifolia

Taxon classificationPlantaeSolanalesConvolvulaceae

#### Diagnosis.

Corolla tube gradually narrowed upwards, the anthers and style weakly exserted from the corolla mouth. This is the type subspecies which is somewhat intermediate between the other two subspecies.

#### Illustration.

Figure 137A.

#### Distribution.

Hill forest between 1750 and 1900 m. Endemic to Oaxaca State in Mexico.

**MEXICO. Oaxaca**: Miahuatlan, *T. Croat* 46030 (MO); ibid., San J. Coatlan, *A. Campos* 3421 (MEXU); Mun. Santiago Textitlán, Paraje Río Aguacate, *I. Trujillo* 1131 (MEXU); ibid., *I. Trujillo* 1132 (IEB, MEXU); ibid., Paraje arriba de Río Tronco Rambo, *I. Trujillo* 847 (MEXU); Sola de Vega, *M.E. Jacob Salinas* 320 (MEXU).

### 266b. Ipomoea chenopodiifolia subsp. signata

Taxon classificationPlantaeSolanalesConvolvulaceae

(House) J.R.I. Wood & Scotland, comb. & stat. nov.

urn:lsid:ipni.org:names:77209932-1

Ipomoea signata House, Muhlenbergia 3: 46.1907. (House 1907a: 46). Type. GUATEMALA. Huehuetenanggo, between Jacaltenanto and San Martín, *E.W. Nelson* 3595 (holotype US00111469).

#### Type.

Based on *Ipomoea signata* House

**Diagnosis**. Corolla tube subcylindrical for almost all its length, slightly widening just below the limb; stamens and style exserted.

#### Illustration.

Figure 137B.

#### Distribution.

Hill forest in Guatemala and neighbouring Chiapas State in southern Mexico from (1000–)1700 to 2100 m.

**GUATEMALA.** Valle de Fuego, *Salvin* s.n. (K); ibid., *A.F. Skutch* 548 (F);

Sacatepéuquez, *A. Molina & A.R. Molina* 124834 (F); ibid., *A. Molina & A.R. Molina* 24834 (F); ibid., San Lucas Sacatepéuquez, *M. Véliz* 94.3489 (MEXU).

**MEXICO. Chiapas**: Mun. Unión Juárez, entre Talquián y Toniná, *E.M, Martínez & A. García* 22197 (MEXU); Mun. Motozintla de Mendoza, Cerro Moxotal, *D.E. Breedlove* 41722 (MEXU); ibid., *D.E. Breedlove* 40467 (MEXU); ibid., *D.E. Breedlove* 22832 (MO); Chiapa de Corzo, *D.E. Breedlove* 22912 (MO); Pueblo Nuevo Solistahuacán, *D.E. Breedlove* 23203 (MO).

### 266c. Ipomoea chenopodiifolia subsp. bellator

Taxon classificationPlantaeSolanalesConvolvulaceae

J.R.I. Wood & Scotland subsp. nov.

urn:lsid:ipni.org:names:77209933-1

#### Type.

MEXICO. Guerrero, Mun. Chichihualco 2550 m, 16 Aug. 1985, *J.C. Soto Nuñez & S. Román* 9974 (holotype MEXU 1354154).

**Diagnosis**. Differs from Ipomoea chenopodiifolia subsp. chenopodiifolia by the clearly funnel-shaped corolla tube with the stamens and style included below the mouth of the corolla.

#### Illustration.

Figure 137C.

#### Distribution.

Endemic to hillforest between 2450 and 2700 m in Guerrero and neighbouring parts of Oaxaca in western Mexico, growing at higher altitudes than the other subspecies.

**MEXICO. Guerrero**: Mun. Tlacotepec, Puerto Jilhuero, *J.C. Soto Nuñez* 11826 (MEXU); Mun. Chilpancingo, 6 km NW de Omiltemi, *P. Tenorio et al.* 2613 (MEXU); ibid., 11.3 km al S de Carrizal, *R. Torres Colín* 7710 (MEXU); Mun. Metlatonoc, *A. de Avila* 143 (MEXU). **Oaxaca**: Mun. Putla Villa de Guerrero, San Andrés Chicahuaxtla, Cerro Zarzamora, *T.MacDougall* s.n. (MEXU37282); San Martín Peras, Juxtlahuaca, *J.I. Calzada* 22200 (MEXU)

#### Note.

Records of *Ipomoea chenopodiifolia* from Venezuela are errors for *I. retropilosa*.

### 267. Ipomoea noctulifolia

Taxon classificationPlantaeSolanalesConvolvulaceae

McPherson, Contrib. Univ. Michigan Herb. 14: 91. 1980. (McPherson 1980: 91)

#### Type.

MEXICO. Jalisco, 5 miles SW of Santa Cruz de las Flores, *R. McVaugh* 16308 (holotype MICH1111344).

#### Description.

Prostrate trailing herb, stems coarsely pubescent with stiff, bulbous-based hairs. Leaves shortly petiolate, 0.8–3 cm long; rounded in outline, the lobes acute, basally cordate, margin dentate, adaxially glabrous or thinly pubescent, abaxially pubescent at least on the veins; petioles 0.6–1.8 cm. Flowers solitary, axillary, pedunculate; peduncles 1–22 mm, pubescent; bracteoles 1 mm, ovate; pedicels 2–15 mm, thickened upwards, pubescent; sepals unequal, outer 2–4 × 1–3 mm, inner 5.5–8.5 mm, ovate to elliptic, obtuse and sometimes mucronate, glabrous; corolla 6–7 cm, funnel-shaped, reddish-purple, glabrous, limb 3–4 cm wide. Capsules ovoid, 5–7 mm long, glabrous; seeds softly pubescent.

#### Illustration.

McPherson (1980: 92); McDonald (1987c: 86).

#### Distribution.

Endemic to Jalisco in central Mexico and recorded as growing in degraded woodland.

**MEXICO. Jalisco**: Zapopan: *J.A. Lomeli* 3378 (IEB); La Peña, Ejutla, *P. Carillo-Reyes* 2244 (IEB); Colotitlán, *M. & H de Cházaro* 4817 (IEB, MEXU).

#### Note.

The inflorescence takes the form of a long leafy raceme. The position of this species here requires confirmation.

### 268. Ipomoea mcvaughii

Taxon classificationPlantaeSolanalesConvolvulaceae

McPherson, Contrib. Univ. Michigan Herb. 14: 94. 1980. (McPherson 1980: 94)

#### Type.

MEXICO. Oaxaca, NE of Putla, *R. McVaugh* 22268 (holotype MICH1111345, isotype ENCB).

#### Description.

Climbing perennial, stems glabrous. Leaves petiolate, mostly 5–13 × 3–8 cm, ovate, cordate, apex acuminate, glabrous but sometimes ciliate on margins with stiff hairs; petioles 0.5–7 cm. Inflorescence of compact, leafy sessile, axillary cymes, bracts resembling small leaves; bracteoles 1.5–3 × 0.75 cm, ovate; pedicels 3–4 mm, glabrous; sepals unequal, elliptic or obovate, acute or obtuse and mucronate, pubescent on margins, outer 6.5–9 × 2–2.5 mm, inner 11–13 × 5 mm; Corolla 5–7.5 cm, funnel-shaped, pink with whitish basal tube, glabrous, limb 3–4 cm diam. Capsules and seeds unknown.

#### Illustration.

McPherson (1980: 93); McDonald (1987c: 83).

#### Distribution.

Endemic to Oaxaca in southern Mexico.

**MEXICO. Oaxaca**: Pinotepa Nacional, N of Putla de Guerrero, *T. Croat* 45865 (MO).

#### Note.

Resembles *Ipomoea dumosa* by having prominent bracts enclosing the inflorescence but differing in the terminal inflorescence. The position of this species here requires confirmation.

### 269. Ipomoea mirandina

Taxon classificationPlantaeSolanalesConvolvulaceae

(Pittier) O’ Donell, Lilloa 26: 370.1953. (O’Donell 1953a: 370)

Exogonium mirandinum Pittier, J. Wash. Acad. Sci. 21: 143. 1931. Type. VENEZUELA. Miranda, *H. Pittier* 12217 (holotype US00111498, isotype NY).

#### Type.

Based on *Exogonium mirandina* Pittier

#### Description.

Liana to 5 m, stems woody subglabrous, striate. Leaves petiolate, 8–14 × 6.5–12.5 cm, ovate, shortly acuminate, broadly cordate, glabrous, abaxially paler; petioles sulcate, 4.5–8 cm. Inflorescence of 2–5-flowered axillary cymes; peduncles 6–8 (–19)cm, stout, glabrous; bracteoles 2 mm, filiform, caducous; secondary peduncles (if present) 3–5 cm, arching; pedicels 1–2.5 cm; sepals dissimilar, outer 18–25 × 12–16 mm, broadly obovate, convex, obtuse to rounded, glabrous, purple-brown, inner similar in size but truncate with broad scarious margins, all drying dark brown; corolla glabrous, hypocrateriform, tube 5–6 cm long, somewhat widened in middle to 12 mm, then narrowed to 6 mm, brown, limb 6–7 cm diam., deep pink, stamens exserted; pink. Capsules glabrous, 15 mm ovoid; seeds 8 × 5 mm, densely pilose with brownish hairs c. 5 mm long.

#### Illustration.

Figure 138.

**Figure 138.**
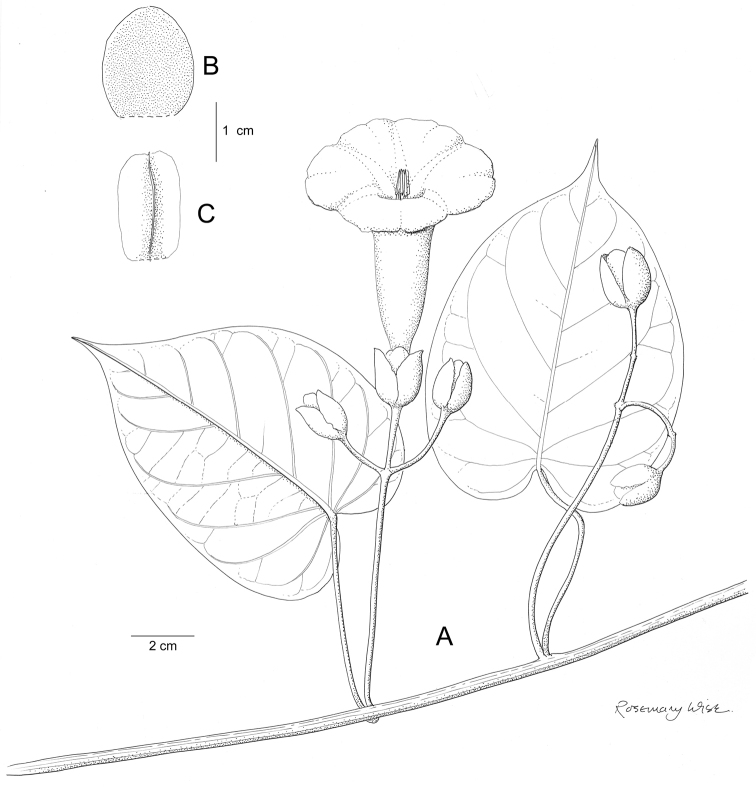
*Ipomoea mirandina*. **A** habit **B** outer sepal **C** inner sepal. Drawn by Rosemary Wise from *Fendler* 942.

#### Distribution.

Hill forests at c. 700–1200 m in Venezuela and Panama. Its occurrence in Colombia is to be expected.

**VENEZUELA. Aragua**: Dist. Ricaurte, carretera Tejerias-Tiara, cerca de Caguita, *Bunting* 4301 (FTG); Tovar, *A. Fendler* 942 (K, MO). **Carabobo**: *H. Pittier* 8034 (US). **Dist. Fed.**: Avila, Quebada Chacaito, *B. Manara* s.n. (FTG). **Miranda**: Colinas de Carrizal, *G. & B. Morillo* 4563 (FTG); Cerro Naiguatá, *J. Steyermark* 91853 (MO). **Sucre**: Peninsula de Paria, camino de Manical a Los Pocitos de Sta. Isabel, NW de Irapa, *K. Dumont et al.* VE-7514, (FTG); Manical, *J. Steyermark & R. Liesner* 120821 (MO). **Yaracuy**: *A. Gentry & L. Puig* 14381 (MO).

**PANAMA.** Altos de Campana, *A. Gentry* 5768 (MO, FTG); *W.H. Lewis et al.* 3155(FTG, MO); *M.D. Correa & E. Montenegro* 10149 (FTG, PMA); *W. D’Arcy* 9556 (CTES, MO); above Río Primero Brazo, 5 miles NW Santa Fe, *T. Croat* 23132 (FTG, MO).

#### Note.

The very large sepals combined with the hypocrateriform corolla with exserted stamens make this species distinctive. Its placement here is provisional.

### 270. Ipomoea parasitica

Taxon classificationPlantaeSolanalesConvolvulaceae

(Kunth) G. Don, Gen. Hist. 4: 275. 1838. (Don 1838: 275)

Convolvulus parasiticus Kunth, Nov. Gen. Sp. 3: 103. 1818 [pub. 1819]. (Kunth 1819: 103). Type. VENEZUELA. near Caracas, Humboldt & Bonplands.n. (holotype P00670753!).
Convolvulus circinnatus Willd. ex Roem. & Schult., Syst. Veg. 4: 302. 1819. Type. VENEZUELA. Caracas, Bonpland & Humboldt 660 (holotype BW03703010).
Ipomoea perlonga B.L. Rob., Proc. Amer. Acad. Arts 29: 319. 1894. (Robinson 1894: 319). Type. MEXICO. Jalisco, Tequila, *C.G. Pringle* 4519 (holotype GH00054527, isotypes BKL, BM, COLO, E, F, GOET, K, M, MA, MEXU, MIN, MSC, MU, NDG, NY, P, PH, S, UC, VT).

#### Type.

Based on *Convolvulus parasiticus*.

#### Description.

Annual or short-lived perennial twining herb to 7 m; stem rather stout and with scattered soft spiny projections, branches rigid. Leaves petiolate, 3–10 × 2–9 cm, ovate, cordate with rounded auricles, apex finely acuminate, abaxially paler, usually adaxially thinly pubescent, sometimes glabrous; petioles 3–5 cm, puberulent. Inflorescence of pedunculate, axillary cymes; peduncles stout, 3–5 cm; bracteoles 3 mm, linear-lanceolate, caducous; secondary peduncles 4–6 mm; pedicels 15–22 mm, stout, thinly puberulent, spreading at a wide angle and often reflexed in fruit; sepals slightly unequal, broadly elliptic with wide scarious margins, outer 6–7 × 5 mm, obtuse and mucronate, abaxially with a few hairs, inner sepals similar but rounded and minutely mucronulate, glabrous; corolla sericeous in bud, 2.5–4 cm long, funnel-shaped, tube white outside, yellow inside, limb blue (drying pink), c. 3 cm diam., deeply lobed. Capsules 7–12 × 5 mm, glabrous, ovoid, acute above a small apical corona; seeds 6–7 mm, brown, glabrous.

#### Distribution.

Widely distributed in America from Mexico south to Bolivia but scattered in occurrence, generally uncommon and often of uncertain status. It is usually found on fences, field border and similar disturbed bushy places at altitudes below 1000 m.

**BRAZIL.** Principally in the north east: **Ceará**: *J.P. Souza et al.* 11034 (RB). **Dist. Fed.**: *H.S. Irwin et al.* 15862 (MO, NY, RB, W). **Goiás**: *H.S. Irwin et al.* 14959 (FTG, MO, NY). **Minas Gerais**: *A. Macedo* 676 (MO), *G. Pereira-Silva et al.* 6364 (CEN). **Paraíba**: *J. Coelho de Moraes* 1841 (RB). **Pernambuco**: *Miranda et al.* 483 (PEUFR); *E.P. Heringer et al.* 720 (IPA, NY). **Rio Grande do Norte**: *J. Freitas* 10100 (UFRN). **Serjipe**: *D.G. Oliveira* 300 (ASE). Also Bahia and Maranhão fide Flora do Brasil (2020).

**BOLIVIA. Santa Cruz**: Ichilo, Buenavista, *J. Dorantes et al.* 1710 (CTES); Ñuflo de Chávez, San Javier, *M. Mendoza & Rivadineira* 2431 (USZ, K); *J.R.I. Wood & D. Soto 27944* (OXF, K, LPB, USZ).

**PERU. Lambayeque**: *T. Plowman et al.* 14300 (F, MO). **Tumbes**: *A. Gentry & C. Díaz* 58297 (MO).

**VENEZUELA. Aragua**: *A. Fendler* 930 (K, MO); *Moritz* 44 (BM). **Dist. Fed.**: Caracas, La Florida, *A.H.G. Alston* 5446 (BM, F, S). Also Lara and Miranda fide Austin (1982b).

**COSTA RICA.** Nicoya, *A. Tonduz* 13679 (BM, K, P); San José, San Ignacio de Acosta, *Khan et al.* 257 (BM); *B. Hammel* 18682 (F, MO).

**NICARAGUA.** Matagalpa, *P.P. Moreno* 25101 (BM), 25082 (BM); Esteli, Pueblo Nuevo, *L.O. Williams & A. Molina* 42410 (BM, F).

**HONDURAS.***A. Molina & A.R. Molina* 34212 (MO).

**EL SALVADOR.***G. Davidse et al.* 37458 (BM, LAGU, MO).

**GUATEMALA.** Santa Rosa, *Heyde & Lux* 4024 (BM, K).

**MEXICO. Baja California Sur**: La Paz, *J.I. Calzada* 25226 (K, MEXU). **Chiapas**: *D.E. Breedlove* 40603 (MO). **Chihuahua**: *P. Tenorio et al.* 10070 (MO). **Est. México & Dist. Fed.**: Valle de México, *E. Bourgeau* 1265 p.p. (K); Temascaltepec, G.B. Hinton 8591 (K). **Guanajuato**: NE de Gavia, *J. Rzedowski* 40931a (IEB). **Guerrero**: Atoyac, *G.B. Hinton* 10898 (K); Montes de Oca, Vallecitos, *G.B. Hinton* 11716 (GBH, K); Petatlán, *E. Langlassé* 629 (K, P). **Jalisco**: *R. McVaugh* 24625 (MICH). **Michoacán**: Arteteaga, *E. Carranza & V.W. Steinmann* 6291 (IEB). **Oaxaca**: Santa María Chimalapa, *S. Maya* 2195 (MEXU). **Querétaro**: Pinal de Amoles, Escanelilla, *S. Zamudio* 5847 (IEB). **Sinaloa**: San Ignacio, Ajoya, *J. González Ortega* 51 (K). **Sonora**: Algodones, Río Mayo, *H.S. Gentry* 1682 (IEB, K, MO); *A.L. Reina-G et al.* 2001-656 (ARIZ). **Veracruz**: Catemaco, La Victoria, *A. Bourg* 182 (IEB); Emiliana Zapata, Ranchito Nuevo, *R.A. Pedraza & H. Perales* 322 (IEB).

**LESSER ANTILLES. Guadeloupe**: *Stehlé* 202 (P) – recorded as introduced.

#### Notes.

A rather fleshy plant with a blue, lobed corolla limb, white tube and, usually, soft spines on the stem. The ripe fruit is held on a recurved peduncle. Dried specimens are superficially very similar to *Ipomoea tricolor* with a blue corolla, white-margined sepals and similar divaricating pedicels but can be distinguished by the sericeous corolla buds and different sepals.

*NGS* sequencing of nuclear genes places this species in the Calonyction Clade, something suggested by the fleshy spines on the stem. *ITS*, however, places it in a clade closer to *Ipomoea mirandina* and its allies.

• Species 271–274 form the small but distinctive Calonyction Clade which consists of night flowering species with white or pale lilac hypocrateriform corollas and often awned sepals. The pollen is also distinct in having blunt gemmiform spines (Figure 10D) although similar spines occur rarely elsewhere in *Ipomoea*.

### 271. Ipomoea muricata

Taxon classificationPlantaeSolanalesConvolvulaceae

(L.) Jacq., Hort. Schoenb. 3(2): 40. 1798. (Jacquin 1798: 40)

Convolvulus muricatus L., Mant. Pl. 1: 44. 1767. (Linnaeus 1767: 44). Type. INDIA. Braads.n. (LINN218.18, lectotype, designated by Verdcourt in Hubbard and Milne-Redhead, Fl. Trop. East Africa, Convolvulaceae 130, 1963).
Calonyction muricatum (L.) G. Don, Gen. Hist. 4: 264. 1838. (Don 1838: 264).
Calonyction speciosum var. muricatum (L.) Choisy in A.P. de Candolle, Prodr. 9: 345. 1845. (Choisy1845: 390).
Ipomoea turbinata Lag., Gen. Sp. Pl. 10. 1816. (Lagasca y Segura 1816: Pl. 10), nom. illeg., superfluous. Type. Based on I. muricata (L.) Jacq.
Convolvulus petiolaris Kunth, Nov. Gen. Sp. Pl. 3: 105. 1818 [pub. 1819]. (Kunth 1819: 105). Type. MEXICO. Volcán de Jorullo, Humboldt & Bonplands.n. (holotype P00670758).
Ipomoea petiolaris (Kunth) G. Don, Gen. Hist. 4: 275. 1838. (Don 1838: 275), nom. illeg., non Ipomoea petiolarisSprengel (1824).
Ipomoea bona-nox var. purpurascens Ker-Gawl., Bot. Reg. 4: t. 290. 1818. (Ker-Gawler 1818c: t. 290). Type. Plate 290 in Botanical Register (lectotype, designated here).
Bonanox muricata Raf., Fl. Tell. 4: 77. 1836 [pub. 1838]. (Rafinesque 1838a: 77). Type. Based on Bot. Reg. t. 290 (1818).
Convolvulus colubrinus Blanco, Fl. Filip., ed. 2, 66. 1845. (Blanco 1845: 66). Type. Lectotype t. 315 in Blanco. Fl. Filipinas, designated here.
Ipomoea tubiflora Hook. f., Trans. Linn. Soc. 20: 204. 1847. (Hooker, J.D. 1847: 204). Type. ECUADOR. Galapagos, James Island, *C. Darwin*s.n. (holotype CGE00308).
Calonyction longiflorum Hassk., Pl. Java Rar. 523. 1848. (Hasskarl 1848: 523). Type. Not cited but reference made to Brown (1810: 340), which appears to be an erroneous reference.
Ipomoea shirensis Baker, Bull. Misc. Inf., Kew 46: 74. 1894. (Baker 1894: 74), nom. illeg., non Ipomoea shirensis Oliver (1884). Type. [MALAWI]. Shire Highlands, *Kirk*s.n. (lectotype K000097188, designated by M.L. Gonçalves (1987: 112).
Ipomoea kirkiana Britten, J. Bot. 32: 85. 1894. (Britten 1894: 85). Type. Based on I. shirensis Baker
Ipomoea spinulosa Brandegee, Zoe 5: 169. 1903. (Brandegee 1903–5: 169). Type. MEXICO. Baja California Sur, *Brandegee*s.n. (isotype US).
Ipomoea calderonii Standl., J. Wash. Acad. Sci. 14: 242. 1924. (Standley 1924: 242). Type. El Salvador, *S. Calderón* 883 (holotype US 00111369).

#### Type.

Based on *Convolvulus muricatus* L.

#### Description.

Vigorous annual climbing or trailing plant; stems stout, armed with soft herbaceous spiny projections. Leaves petiolate, 7–18 × 6–17 cm, ovate or, rarely, 3-lobed, cordate with rounded auricles, apex shortly acuminate, glabrous; petioles 3–15 cm. Inflorescence of 1–2(–5)-flowered, pedunculate cymes; peduncles 2.5–20 cm, usually long, but, if short, commonly with soft spines; bracteoles caducous; pedicels 1.5–4.5 cm, stout and strongly swollen upwards, becoming reflexed in fruit; sepals unequal, accrescent in fruit, glabrous, white with green midrib, outer 10–14 mm, narrowly ovate, attenuate into a point up to 7 mm long, inner 7–12 mm, broadly ovate, abruptly narrowed to an awn 3–4 mm long; corolla dark lilac, 5–6 cm long, glabrous, tube narrowly cylindrical below but widened to 10 mm below limb, limb c. 4 cm diam., spreading, unlobed. Capsules ovoid, 1.5–2 cm long and wide, glabrous, rostrate, the persistent style c. 3 mm long, the pedicel commonly reflexed; seeds 8–10 mm long, glabrous.

#### Illustration.

O’Donell (1959b: 195).

#### Distribution.

Scattered throughout the tropics but rarely abundant. It is usually found growing in disturbed bushy places at low altitudes.

**ARGENTINA. Salta**: Campo Santo, *C. O’Donell* 2669 (CTES, LIL).

**BRAZIL. Ceará**: Caucaia, *A.S.F. Castro* 1810 (EAC); Aquiraz, *A.S.F. Castro* 2493 (EAC). **Minas Gerais**: Ituiutaba, *A. Macedo* 749 (MO). **Pernambuco**: Fernando de Noronha, *A.M. Miranda* 4086 (RB).

**BOLIVIA. Chuquisaca**: Com. Orotote, *R. Lozano et al.* 1183 (MO). **Santa Cruz**: Chiquitos, Santiago, *J.R.I. Wood & B. Williams* 27904 (K, LPB, USZ); Cordillera, Alto Parapeti, *R. Chávez de Michel* 261 (LPB); Velasco, Carmen Ruiz–San José Campamento, *J.R.I. Wood et al.* 27838 (K, LPB, USZ). **Tarija**: Gran Chaco, near Villamontes, *A. Krapovickas & A. Schinini* 31182 (CTES, F, MO); O’Connor, 5 km N of Entre Ríos, *M. Atahuachi et al.* 1519 (BOLV).

**PERU. Lambayeque**: *M. Weigend et al.* 8529 (USM).

**ECUADOR. Galápagos**: Santa Cruz: *F. Fagerlind & G.Wibom* 3228 (S); *P.S. Bentley* 235 (NY, MO, QCNE, US). **Guayas**: *E. Asplund* 15917 (S). **Loja**: Zapotepampa, *F. Vivar* 1358 (LOJA). **Manabí**: Jipijapa, *M. Montesdeoca et al.* 976 (QAP).

**VENEZUELA. Bolívar**: *L. Aristeguieta* 5817 (US, VEN). **Guárico**: Valle de Guanape-Altagracia de Orituco, *L. Aristeguieta* 6454 (MO, VEN).

**COSTA RICA.** Guanacaste, *B. Hammel & I. Pérez* 25849 (MO).

**NICARAGUA.** Rivas, San Juan del Sur, *W.D. Stevens & O.M. Montiel* 30403 (HULE, MO); Carazo, La Palma, Chacocente, *M. Aranda* 121 (MO).

**EL SALVADOR.** Ahuachapan, Área Protegida Santa Rita, *J.M. Rosales* 1940 (BM, MO).

**HONDURAS.** Comayagua, La Libertad, *C.H. Nelson et al.* 7579 (MO).

**MEXICO. Baja California Sur**: type of *Ipomoea spinulosa*. **Est. México & Dist. Fed.**: Temascaltepec, Naranjo, *G.B. Hinton* 5011 (K); ibid., Tejupilco, *G.B. Hinton* 8414 (GBH, K, MO). **Guerrero**: Punarabato, Coyuca, *G.B. Hinton* 6932 (BM, GBH, K, MO). **Sinaloa**: Fuerte, *J.N. Rose et al.* 13566 (K); Imala, *H.S. Gentry* 5464 (MEXU). **Sonora**: Yécora, *A.L. Reina-G et al.* 98-1515 (MEXU). **Yucatán**: Izamal, *G.F. Gaumer* 987 (BM, K, MO, P); Mérida, *Schott* 684 (BM).

**UNITED STATES. Florida**: fide Wunderlin and Hansen 2011: 392. Kentucky: *M.J. McWhirter* s.n. [7/2002] (EKY). **Mississippi**: Washington Co., *C.T. Bryson* 21209 (ARIZ).

**NETHERLANDS ANTILLES. St Eustatius**: fide Powell (1979). **Curaçao**: *Proosdij et al.* 654 (K, NY, U). **Bonaire**: fide Proosdij (2012).

#### Notes.

Commonly confused with *Ipomoea alba* but when flowering easily identified by the shorter lilac corolla which is widened below the limb. In fruit it is more difficult to separate but the aristate tip of the inner sepals is only 2–3 mm long.

*Ipomoea tubiflora* Hook. f. from James Island (Santiago) in the Galapagos represents a plant with slender stems devoid of fleshy spines. A more recent specimen (*P.S. Bentley* 235 (NY, MO, US) from Santa Cruz Island) is somewhat similar but with some fleshy stem spines so providing a link to more typical *Ipomoea muricata*. It is interesting that the Galapagos Islands also have extreme forms of *Ipomoea incarnata*, suggesting that isolation and the arid climate is allowing the evolution of distinct forms.

### 272. Ipomoea alba

Taxon classificationPlantaeSolanalesConvolvulaceae

L., Sp. Pl. 1: 161. 1753. (Linnaeus 1753: 161)

Calonyction album (L.) House, Bull. Torrey Bot. Club 31: 591. 1904. (House 1904: 591).
Ipomoea bona-nox L., Sp. Pl., ed. 2: 228. 1762. (Linnaeus 1762: 228), nom. illeg., superfluous name for I. alba L.
Convolvulus bona-nox (L.) Spreng., Syst. Veg. 1: 600. 1825 [pub. 1824]. (Sprengel 1824: 600).
Calonyction speciosum Choisy, Mém. Soc. Phys. Genève 6: 441[59]. 1834. (Choisy 1834: 441[59]). Type. Based on Ipomoea bona-nox L.
Calonyction bona-nox (L.) Bojer, Hort. Maurit. 227. 1837. (Bojer 1837: 227).
Calonyction speciosum var. vulgare Choisy in A.P. de Candolle, Prodr. 9: 345. 1845. (Choisy1845: 390), var. illeg., autonymic var.
Ipomoea aculeata forma bona-nox (L.) Voss, Vilmorins Blumengärtn. 708. 1894. (Voss 1894–96: 708).
Ipomoea aculeata var. bona-nox (L.) Kuntze, Rev. Gen. Pl. 2; 442. 1891. (Kuntze 1891: 442).
Quamoclit bona-nox (L.) M. Gómez, Fl. Habana 345. 1897 [dated 1899]. (Gómez de la Maza y Jiménez 1897: 345).
Convolvulus aculeatus var. bona-nox (L.) Kuntze, Rev. Gen. Pl. 3: 212. 1898. (Kuntze 1898: 212).
Bonanox indica Raf., Fl. Tell. 4: 77. 1836 [pub. 1838]. (Rafinesque 1838a: 77). Type. Based on Ipomoea bona-nox L.
Bonanox riparia Raf., Fl. Tell. 4: 77. 1836 [pub. 1838]. (Rafinesque 1838a: 77). Type. Based on Ipomoea bona-nox in Bot. Mag. t. 752 (1804).
Convolvulus aculeatus L., Sp. Pl. 155. 1753. Type. Icon in Plukenet, Phytographia t. 276, f. 3 (1694), designated by Gunn (1972: 153).
Calonyction aculeatum (L.) House, Bull. Torrey Bot. Club 31: 590. 1904. (House 1904: 590).
Convolvulus latiflorus Desr., Encycl. Meth. 3: 561. 1789 [pub. 1792]. (Desrousseaux 1792: 561). Type. Santo Domingo and Martinique (syntype P-JUSS, not found).
Ipomoea latiflora (Desr.) Roem. & Schult., Syst. Veg. 4: 240. 1819. (Roemer and Schultes 1819: 240).
Euryloma latiflora (Desr.) Raf., Fl. Tellur. 4: 75. 1836 [pub. 1838]. (Rafinesque 1838a: 75)
Ipomoea longiflora Humb. & Bonpl. ex Willd., Enum. Pl. 1: 207. 1809. (Willdenow 1809: 207). Type; CUBA. La Habana, Humboldt & Bonplands.n. (possible holotype B-W 03759-01, isotype P00670772).
Quamoclit longiflora (Humb. & Bonpl. ex Willd.) G. Don, Gen. Hist. 4: 259. 1838. (Don 1838: 259).
Ipomoea grandiflora Roxb., Hort. Bengal. 14. 1814. (Roxburgh 1814: 14)., nom. illeg., non Ipomoea grandiflora (L.f.) Lam. (1791). Type. Rheede, Hort. Malab. 11: t. 50 (1692), lectotype designated by Turner (2013: 157).
Ipomoea roxburghii Steud., Nomencl. Bot. 1: 819. 1840, (Steudel 1840: 819), non Ipomoea roxburghiiSweet (1826). Type. Based on Ipomoea grandiflora Roxb.
Ipomoea bona-nox var. grandiflora (Roxb.) C.B. Clarke, Fl. Brit. India 4: 197. 1883. (Clarke 1883: 197).
Ipomoea tubulosa Willd. ex Roem. & Schult., Syst. Veg. 4: 789. 1819. (Roemer and Schultes 1819: 789). Type. America meridionalis, Humboldt & Bonplands.n. (holotype B-W03755-01).
Convolvulus pulcherrimus Vell., Fl. Flumin. 72. 1829 [dated 1825]. (Vellozo 1829: 72). Type. BRAZIL. (lectotype, original parchment plate of Flora Fluminensis in the manuscript section of the Biblioteca Nacional, Rio de Janeiro [cat. no.: mss1198651-054], designated here; later published in Vellozo, Fl. Flum. Icon. 2: t. 54 1827. [pub. 1831]).
Ipomoea noctiluca Herb., Bot. Reg. 11: t. 917. 1825. (Herbert 1825: t. 917). Type. Lectotype t. 889 in Bot. Reg. 11 under Ipomoea latiflora, designated here. No specimen found at CGE.
Calonyction noctilucum (Herb.) Sweet, Hort. Brit., ed. 3: 482. 1839. (Sweet 1839: 482).
Ipomoea ambigua Endl., Prod. Fl. Norf. 53, 1833. (Endlicher 1833: 53). Type. NORFOLK ISLAND, *F.L. Bauer*s.n. (lectotype W0050659, designated here).
Calonyction macrantholeucon Colla, Mem. Nov. Sp. Calon. 15. 1840. (Colla 1840: 15). Type. A cultivated plant (lectotype TO, sheet numbered 5077, designated here).
Calonyction speciosum var. macrantholeucon (Colla) Choisy in A.P. de Candolle, Prodr. 9: 345. 1845. (Choisy1845: 390).
Calonyction megalocarpum A. Rich. ex Sagra, Hist. Fis. Cuba, Bot. 3: 129. 1850. (Sagra 1850: 129). Type. CUBA. Canasi (lectotype P04039238, designated here).
Ipomoea noctiflora Griff., Not. Pl. Asiat. 4: 286. 1854. (Griffith 1854: 286). Type. MYANMAR (BURMA). Mergui, Mrs Hutton’s Garden, no specimen found.
Calonyction pulcherrimum D. Parodi, Contr. Fl. Paraguay 12. 1877. (Parodi 1877: 12). Type. [PARAGUAY], “in ripis Paraná”, no specimen cited or known.
Ipomoea aculeata var. heterophylla Kuntze, Rev. Gen. Pl. 2; 442. 1891. (Kuntze 1891: 442). Type. PUERTO RICO. No type specified.
Calonyction bona-nox var. lobatum Hallier f., Bull. Herb. Boiss. 5: 1037. 1897. (Hallier 1897b: 1037). Type. JAMAICA. *H. Sloane*s.n. (lectotype BM000589513, designated here).
Calonyction bona-nox subvar. calvum Hallier f., [as var. lobatum subvar. calvum] Bull. Herb. Boiss. 5: 1037. 1897. (Hallier 1897b: 1037). Type. As for var. lobatum Hallier f.
Ipomoea aculeata auct. mult. Amer., non Blume

#### Type.

Icon in Rheede, Hort. Ind. Malabar 11: t. 50 (1692), designated by Verdcourt (1963: 130) in Hubbard and Milne-Redhead (Eds) Fl. Trop. East Africa, Convolvulaceae.

#### Description.

Vigorous scrambling or trailing plant, stems to 10 m, glabrous, sometimes armed with soft spiny projections, sometimes subtomentose. Leaves petiolate, 5–15 × 4–14 cm, ovate, sometimes-lobed to about one third, acuminate to a fine hair point, cordate at the base, auricles sometimes with broad teeth, both surfaces glabrous; petioles 3–18 cm. Inflorescence of 1–3-flowered, pedunculate, axillary cymes; peduncles 2–9(–20) cm, stout; bracteoles caducous, not seen; pedicels 5–15 mm, swollen below flower; sepals unequal, glabrous, outer sepals 15–25 × 4–6 mm, lanceolate with a long awn 5–12 mm in length, green with white margins inner sepals 12–20 mm including a 2–5 mm long awn, ovate, whitish with green midrib; corolla hypocrateriform, with a narrow cylindrical whitish-green tube 5–12 cm long and a spreading, white limb 4–5 cm in diam., glabrous. Capsules ovoid, c. 3 cm long, glabrous; seeds 11–13 mm long, glabrous.

#### Illustration.

Figures 4C, 10D, 121D; O’Donell (1959b: 105); Acevedo-Rodríguez (2005: 165); Austin (1998: 400); Bosser and Heine (2000: 44); Deroin (2001: 167).

#### Distribution.

A pantropical weedy species, not certainly known as a native anywhere but clearly of neotropical origin. Widely distributed in disturbed damp bushy places, particularly along shaded tropical streams, mostly below about 1600 m and probably native in this habitat in the Neotropics. It is also cultivated growing in gardens as high as Sucre (2800 m) in Bolivia as well as in gardens and conservatories in cool temperate countries.

**URUGUAY.***W.G. Herter* 73 (MO, S).

**ARGENTINA. Catamarca**: *P. Jörgensen* 1421 (MO). **Córdoba**: *A.T. Hunziker* 17369 (CORD). **Corrientes**: *M.M. Arbo et al.* 6607 (CTES, S). **Formosa**: *P. Jörgensen* 3065 (MO). **Misiones**: *E.L. Ekman* 1439 (S).

**PARAGUAY. Alto Paraguay**: Estancia Miranda, *F. Mereles 6848* (FCQ). **Alto Paraná**: *E. Zardini & E. Florentin* 40048 (MO). **Caazapá**: P.N.Caaguazú, *L. Molas* 762 (PY). **Canindeyú**: *Simonis et al.* 236 (PY, U). **Central**: *T. Morong* 269 (BM); Ypacaraí, *E. Zardini et al.* 2402 (FCQ, MO). **Cordillera**: Tobatí, *E. Zardini & R. Velázquez* 27362 (FCQ, MO). **Guairá**: Villarica, *G.W. Teague* 532 (BM); La Colmena–San José, *F. Mereles & F. González* 7907 (FCQ). **Itapúa**: Isla Yacyretá, *J. de Egea et al.* 337 (BM, FCQ). **Paraguarí**: Macizo Acahay, *E. Zardini* 6160 (MO, PY); Cerro Acahay, *L.R. Landrum et al.* 8625 (ARIZ, FCQ). **San Pedro**: *A.L. Woolston* 1548 (K).

**BRAZIL. Acre**: *G.T. Prance et al.* 12008 (K, MO, NY). **Amazonas**: *B.A. Krukoff* 4509 (MO, NY, S); Rio Solimões, *R. Spruce* 1626 (K); Manãos, *J. Loew* 182 (K). **Bahia**: *J.L. Hage & E.B. dos Santos* 1174 (K). **Ceará**: Villa de Orato, *G. Gardner* 1771 (BM, K). **Dist. Fed.**: *E.P. Heringer et al.* 1932 (NY). **Espirito Santo**: *H. Boudet-Fernandes* 1588 (MO). **Mato Grosso**: *B. Dubs* 1268 (K, Z). **Minas Gerais**: Ituiutaba, *A. Macedo* 1922 (BM). **Pará**: *E.P. Killip* 30648 (NY). **Paraná**: *G. Hatschbach* 24141 (MBM, MO). **Pernambuco**: Fernando do Noronha, *Ridley et al.* s.n. [1887] (BM, P). **Rio de Janeiro**: *M.R. Barbosa et al.* 18855 (K). **Rio Grande do Sul**: *A. Kegler* 180 (MO). **Rondônia**: *G.T. Prance et al.* 6544 (NY). **Santa Catarina**: *A. Gavieski* 81 (K). **São Paulo**: *N.A. Rosa & J.M. Pires* 3838 (NY).

**FRENCH GUIANA.***Courbon* s.n. (P); *Sastre* 4697 (P).

**SURINAM.***J. Langouw & J.C. Lindman* 1551 (MO).

**GUYANA.** Fide Austin and Huáman (1996).

**BOLIVIA. Beni**: *S.G. Beck* 5561 (LPB). **Chuquisaca**: *M. Cárdenas* 5733 (US). **Cochabamba**: Chapare, *J. Steinbach* 9355 (BM, NY, GH, F, MO, S). **La Paz**: *J. Solomon* 13710 (LPB, MO). **Pando**: *S.G. Beck et al.* 19555 (COL, LPB). **Santa Cruz**: *J.R.I. Wood et al.* 19653 (BOLV, K, LPB, USZ). **Tarija**: *M. Serrano et al.* 7608 (LPB).

**PERU. Amazonas**: Chachapoyas, *R.W. Bussmann et al.* 16839 (MO). **Cajamarca**: *P.C. Hutchison & J.K. Wright* 3607 (K, P, UC). **Cusco**: *G. Calatyud et al.* 1942 (CUZ, MO); Convención, *C. Vargas* 3482 (CUZ). **Huánuco**: Huallaga valley, *A. Gentry et al.* 37627 (MO). **Ica**: San Juan Baptista, *O. Whaley et al.* 213 (K). **Lima**: Canta, *P. Gonzáles* 135 (USM). **Loreto**: *A. Gentry et al.* 32130 (MO). **Madre de Dios**: *P. Nuñez* 12308 (CUZ, MO). **Pasco**: *L. Valenzuela et al.* 12618 (MO, USM). **Puno**: Sandia, *C. Vargas* 16409 (CUZ). **San Martín**: *J. Schunke* 4022 (F). **Ucayali**: *K.R. Young & G. Sullivan* 658 (NY).

**ECUADOR. Galápagos**: *G. Harling* 5615 (S). *C. Crossland* 455 (K). **Guayas**: *R. Spruce* 6493 (BM, K). **Imbabura**: *G. Harling* 4320 (MO, S). **Loja**: *R. Espinosa* 215a (NY). **Manabí**: *Eggers* 15461 (P). **Napo**: *H. Lugo* 2747 (MO). **Pastaza**: *B. Løjtnant & U. Molau* 13283 (AAU, GB).

**COLOMBIA. Amazonas**: *J. Duque* 2415 (COL). **Antioquia**: Naranjo: *E. André* 372 (K). **Cauca**: *K. von Sneidern* s.n. [18/11/1941] (S). **Chocó**: Bahía Solano, *E. P. Killip* 33586 (COL, NY). **Cundinamarca**: *G. Dugand* 3805 (COL). **Magdalena**: Santa Marta, *H.H. Smith* 1581 (NY, S). **Valle**: *F.C. Lehmann* 7906 (K).

**VENEZUELA. Aragua**: Tovar, *A. Fendler* 929 (K). **Bolívar**: *J. Steyermark et al.* 104063 (MO). **Miranda**: *K. Robertson & D.F. Austin* 222 (MO). **Sucre**: *J. Steyermark & R. Liesner* 121014 (MO). **Zulia**: *G.S. Bunting et al.* 12569 (MO).

**PANAMA.***A. Gentry* 4428 (BM, MO); *H. Pittier* 2244 (BM); *Duchassaing* s.n. [1851] (P).

**COSTA RICA.** El General, *A.F. Skutch* 4265 (K, S); Puntarenas, Coto Brus, *M.M. Chavarría* 691 (K, MO); Tucurrique, *A. Tonduz* 12942 (BM, K).

**NICARAGUA.***D. Weberbauer* 7340 (BM); *L.O. Williams & A. Molina* 42471 (BM, F); *W.D. Stevens* 3942 (BM, MO).

**HONDURAS.***S. Blackmore & G.L.A. Heath* 1763 (BM).

**EL SALVADOR.***Hartman* 59 (S); Lago Illopango, *K. Sidwell et al.* 529 (BM).

**BELIZE.***M.E. Peck* 762 (K); Orange Walk, *C. Whitefoord* 8174 (BM).

**GUATEMALA.** Escuintla, *J. Donnell Smith* 2017 (K); *R. Tun Ortiz* 665 (BM, F).

**MEXICO. Campeche**: Calakmul, *D. Álvarez & J.C. Soto Nuñez* 1251 (IEB). **Chiapas**: Ocosingo, *E. Martínez et al.* 25431 (K); *P. J. Stafford et al.* 150 (BM). **Est. México & Dist. Fed.**: Valle de México, *E. Bourgeau* 1382 (K, P, S). **Guerrero**: Galeana, *G.B. Hinton* 11195 (K). **Guanajuato**: Valle de Santiago, *M. González* 70 (IEB). **Jalisco**: *E. Palmer* 727 (BM); La Huerta, Rancho Cuixmala, *A.C. Sanders et al.* 10510 (K). **Michoacán**: Coalcomán, *G.B. Hinton* 12698 (K). **Morelos**: Tlayacapan, *Hernández et al.* 648 (IEB). **Nayarit**: Nuevo Vallarta *R. Barraza* s.n. (IEB). **Oaxaca**: *W.H. Camp 2423* (K, NY). **Puebla**: Ajalpan, *J.I. Calzada* 23614 (K, MEXU). **Querétaro**: Landa de Matamoros, *A. Herrera* 49 (IEB). **Quintana Roo**: *O. Téllez* 2006 (BM, MEXU). **Sonora**: fide Felger et al. (2012). **Tabasco**: Nacajuca, *H. Cálix* 416 (IEB). **Veracruz**: *E. O. Darlet* s.n. (K); *E. Kerber* 47 (BM, P). **Yucatán**: Cozumel Island, *G.F. Gaumer* 75 (K), 330 (BM, P).

**UNITED STATES. Florida**: *A.H. Curtiss* 2166 (BM, K, P, S); *H. Moldenke* 758 (K, S); *L. Kitching* s.n. [1905] (BM). **Louisiana**: *C. Reid & T. Baker* 5860 (LSU).

**BERMUDA.***F.S. Collins* 250 (K, NY).

**BAHAMAS.** New Providence, *D.S. Correll* 48400 (NY); Ackling Island: *L. Brace* 4287 (NY).

**CUBA.***López Figuieras* 783 (HAJB); *C. Wright* 450 (BM); *N.L. Britton* 6660 (NY); *R. Combs* 716 (NY); *J.A. Shafer* 1524 (NY).

**HAITI.***L. R. Holdridge* 2013 (BM, NY); *E.L. Ekman* H9194 (S).

**DOMINICAN REPUBLIC**. *E.L. Ekman* H16197 (S); *M.M. Mejia Pimentel & T. Zanoni* 9216 (NY); *Poiteau* s.n. (P).

**PUERTO RICO.***P. Sintenis* 446 (S); *A.A. Heller* 375 (NY).

**JAMAICA.***W. Harris* 8458 (BM); *W. Stearn* 398 (BM); *G.R. Proctor* 20703 (NY).

**LESSER ANTILLES. Guadeloupe**: *A. Duss* 3499 (NY); *H. & M. Stehlé* 8166 (P). **Dominica**: *C. Whitefoord* 6017 (BM); *W.H. Hodge* 811 (BM). **Martinique**: *A. Duss* 428 (NY); *Belanger* 219 (P). **St Vincent**: fide Powell (1979).

**TRINIDAD.***A. Fendler* 589 (BM, P).

**HAWAII.***Faurie* 1037 (BM); *F.R. Fosberg* 57423 (K); *R. Kuykendall* 137 (BM); *L.H. MacDaniels* 149 (BM); *T.G. Lammers* 8045 (BM, F); *O. Degener* 24511 (BM); Honolulu, *C.R. Annabale & D.E. Atha* 3097 (NY).

#### Typifications.

McDonald, (1994: 13) designated Velloso (1829 69, t. 25) as the lectotype of *Convolvulus pulcherrimus* Vell. but there is no plate on this page and t. 25 does not correspond to an *Ipomoea*. Hence a new lectotype is designated above.

Herbert’s description of *Ipomoea noctiluca* in the Botanical Register 11: t. 917 (Herbert 1825: t. 917) is included after the description of *Hibiscus racemosus* Lindl. No type is cited and there is no specimen at CGE but there is a reference to the illustration of *Ipomoea latiflora* (t. 889 in Bot. Reg. 11), which was, according to Herbert, drawn from one of his cultivated plants which he believed to be a distinct species. This is described after *Hibiscus racemosus*. We have, therefore, designated t. 889 in the Botanical Register as the lectotype of *Ipomoea noctiluca*.

#### Notes.

Unmistakeable when in flower but fruiting material can be difficult to distinguish from *Ipomoea muricata* except by the longer aristate points of the sepals. Plants with tomentose stems are known from Ecuador: *F. Vilar* 517 (LOJA) from the Galapogos Islands and *F. Vilar* 190 (LOJA) from Catamayo, Loja. They may occur elsewhere.

### 273. Ipomoea santillanii

Taxon classificationPlantaeSolanalesConvolvulaceae

O’Donell, An. Inst. Biol. Mex. 12: 93. 1941. (O’Donell 1941: 93)

Calonyction ventricosum Hallier f., Bot. Jahrb. Syst. 16: 556. 1894 [pub.1893]. (Hallier 1893a: 556), non Ipomoea ventricosa (Bertero) G. Don (1838). Type. MEXICO. Veracruz, Valle de Cordoba, *E. Bourgeau* 1993 (holotype G00227297, isotype P).

#### Type.

Based on *Caloncytion ventricosum* Hallier f.

#### Description.

Perennial climber reaching 15 m, stems stout, glabrous, sometimes with occasional fleshy teeth. Leaves petiolate, very large, 15–20 × 12–18 cm, ovate, shortly acuminate, cordate, glabrous to thinly pubescent on the abaxial veins towards the base; petioles 10–20 cm. Inflorescence of long-pedunculate axillary cymes with up to 12 flowers, usually in a cymose cluster but sometimes (as in the type) forked into two branches, appearing lax and racemose; peduncles 6–30 cm; bracteoles caducous, suborbicular, convex, with fine terminal mucro c. 2–6 mm long, c. 2–3 cm × 1.5–2.5 cm, membranous with prominent venation; secondary peduncles up to 6.5 cm; pedicels 1–2.5 cm, thickened upwards; sepals similar, 9–14 × 4–7 mm, ovate-elliptic, obtuse, glabrous; corolla white, glabrous, funnel-shaped, basal cylindrical tube 1.5–5 cm long, 0.3–06 cm wide, then abruptly swollen before gradually being widened to mouth, the whole tube 4–6 cm long, the limb 2–3 cm long and 4–5 cm wide,; stamens shortly exserted. Capsules 2.5–3 × 1.5 cm, conical, rostrate, glabrous; seeds glabrous, 10 × 6 mm.

#### Illustration.

Figure 8F.

#### Distribution.

Southern Mexico to Costa Rica. Disturbed woodland on stony ground, 800–1300 m.

**COSTA RICA.** San José, *P. Döbbeler* 934 (BM); Río María, *A. Tonduz* 8439 (BM), 13051 (K).

**EL SALVADOR.** La Libertas, Antigua Cuscatlan, *P. Lemus* s.n. [13/1/1989] (K, LAGU).

**GUATEMALA.** Chapadero, Santa Rosa, *Heyde & Lux* 4354 (K).

**MEXICO. Chiapas**: Mun. San Fernando, *J. Carmen Soto et al.* 13324 (BM, MEXU, ARIZ); Esquintla, *E. Matuda* 17292 (K). **Colima**: Rancho El Jabali, *E. Lott et al.* 3009 (MICH). **Est. México & Dist. Fed.**: Temascaltepec, Tejupilco, *G.B. Hinton* 2296 (BM, K); ibid., Luvianos, *G.B. Hinton* 7169 (K); Piedras Negras, *E. Matuda* 29702 (IEB). **Guerrero**: El Balsamo, *J.C. Soto Nuñez* 11469 (MEXU). **Jalisco**: San Sebastián del Oeste, *T.S. Cochrane et al.* 12045 (IEB). **Michoacán**: Coalcomán, Sierra Naranjillo, *G.B. Hinton* 12680 (K); Zirimicuaro, *S. Zamudio* 11264 (IEB). **Oaxaca**: Santa Cruz Tepitotula, P. *Osorio* 265 (IEB). **Veracruz**: Orizaba, *E. Bourgeau* 3024 (P, K); Moyapan, Orizaba, *M. Rosas* 709 (BM. GH, MEXU).

#### Note.

The variations in inflorescence structure and corolla size are so great that it is difficult to believe only one species is involved.

### 274. Ipomoea magniflora

Taxon classificationPlantaeSolanalesConvolvulaceae

O’Donell, Lilloa 26: 369. 1953. (O’Donell 1953a: 369)

Ipomoea skutchii J.A. McDonald, Harvard Pap. Bot. 4; 55. 1993. (McDonald 1993a: 55). Type. COSTA RICA. A.F. Skutch 2982 (holotype NY00547068, isotypes K, GH, S, US).

#### Type.

COSTA RICA. San José, *A. F. Skutch* 2982 (holotype S12-2092, isotypes K, GH, NY, US).

#### Description.

Climbing perennial; stem glabrous but strongly muricate. Leaves long petiolate, large, 14–21 × 11–16 cm, ovate, shortly acuminate, cordate with narrow sinus and rounded auricles, adaxially with scattered hairs, abaxially paler, pubescent on the veins; petioles 16–24 cm, glabrous. Inflorescence with up to 4 flowers, somewhat racemose in structure; peduncle c. 30 cm, glabrous; bracteoles lanceolate, c. 11 × 2 mm, caducous; pedicels 1.5–2 cm, thickened upwards; sepals unequal, long-aristate, glabrous with scarious margins, outer 10–14 mm with 5–6 mm long mucro, oblong-elliptic, inner 14–16 mm with 2–3 mm long mucro, ovate; corolla c. 13 mm long, white, funnel-shaped, the basal cylindrical tube c. 2 cm long, but limb c. 6 cm wide, buds and midpetaline bands pubescent. Capsules and seeds unknown.

#### Distribution.

Endemic to Costa Rica and only known from the type collection.

**COSTA RICA.** Type collection.

• Species 275–279 form another distinct small clade characterised by having pinnatifid leaves and a relatively large corolla. This clade is almost restricted to Mexico.

### 275. Ipomoea jacalana

Taxon classificationPlantaeSolanalesConvolvulaceae

Matuda, Anales Inst. Biol. Univ. Nac. México 35: 58. 1965. (Matuda 1965: 58)

#### Type.

MEXICO. Hidalgo, Cumbre de Jacala, *E. Matuda* 37288 (holotype MEXU00050769).

#### Description.

Trailing or climbing plant, stems finely pilose, glabrescent. Leaves petiolate, 4–9 × 2.5–7 cm, ovate-deltoid, obtuse, base cordate and then cuneate onto the petiole with distinct rounded auricles, margin irregularly dentate, both surfaces green, abaxially paler, strongly reticulate, veins pubescent; petioles 2–5 cm. Inflorescence of 1–3 flowered, axillary cymes; peduncles 10–15 cm; bracteoles 1–4.5 × 0.5–3.5 cm, ovate, petiolate, dentate, resembling small leaves; pedicels 14–22 mm, tickened upwards, pubescent; sepals subequal, 15–17 × 6 mm, narrowly oblong-elliptic, acuminate, margin scarious, outer sepals pubescent, glabrescent; corolla 5.5–6.5 cm long, funnel-shaped, pinkish-purple with paler tube, glabrous, limb c. 3 cm diam. Capsules and seed unknown.

#### Distribution.

Endemic to Hidalgo in Mexico and only known from the type.

**MEXICO. Hidalgo**: type collection.

#### Note.

The leaf shape suggests this species is intermediate between *Ipomoea stans* and another species, perhaps *I. orizabensis*. The foliose bracteoles and dentate leaves are distinctive characters.

### 276. Ipomoea stans

Taxon classificationPlantaeSolanalesConvolvulaceae

Cav., Icon 3: 26. 1795. (Cavanilles 1795–96: 26)

Convolvulus stans (Cav.) Kunth, Nov. Gen. Sp. 3: 96. 1818 [pub. 1819]. (Kunth 1819: 96).
Convolvulus firmus Spreng., Syst. Veg. 1: 613. 1825 [pub. 1824]. (Sprengel 1824: 613), nom. illeg. superfl., based on C. stans (Cav.) Kunth
Convolvulus sinuatus Sessé & Moçiño, Pl. Nov. Hisp. 1: 24. 1888. (Sessé y Lacasta and Moçiño 1887–90: 24), nom. illeg. non Convolvulus sinuatus Petagna ex Steudel (1840). Type. MEXICO. Guanajuato, Ixtla, *Sessé & Moçiño* 117 [5023], lectotype MA603854, designated by McDonald 2001: 89).
Ipomoea stans var. hirsuta B.L. Rob., Proc. Amer. Acad. Arts 29: 319. 1894. (Robinson 1894: 319). Type. MEXICO. Jalisco, *E. Palmer* 324 (lectotype GH n.v., designated by House (1908b: 187), isolectotypes MO, NDG, US, YU).
Ipomoea jaliscana House, Ann. New York Acad. Sci. 18: 187. 1908. (House 1908b: 187). Type. Based on Ipomoea stans var. hirsuta B.L. Rob.

#### Type.

Plant cultivated in Madrid, presumably of Mexican origin, lectotype MA475857, designated by McDonald (1994: 133) and re-designated here (see below).

#### Description.

Perennial herb (reported sometimes to be annual), branched at base with many ascending stems to 1 m forming a small bush, stems woody below, crisped pubescent, rootstock tuberous, reputed to be poisonous. Leaves shortly petiolate 2.3–5.5 × 1.5–2.5 cm, oblong to oblong-ovate, obtuse to truncate, base truncate and then broadly cuneate onto petiole, margin lyrate-dentate, both surfaces glabrous with scabrous veins and margins to pubescent with densely pubescent veins, abaxially with prominent venation; petiole 3–5 mm. Flowers solitary (rarely paired), axillary; peduncles 0.5–5 cm, pubescent; bracteoles variable sometimes linear-oblanceolate c. 5 mm long, sometimes foliose with lyrate margins and reaching 15 mm; pedicels 3–10 mm, thickened upwards, scabrous to pubescent; sepals unequal, glabrous to scabrous, margins scarious, outermost 7–12 × 6 mm, broadly to narrowly oblong-elliptic, obtuse, inner 10–16 × 8 mm; corolla 6–7.5 cm long, flared to funnel-shaped, purple, glabrous, limb c. 4 cm diam. Capsules 14–16 × 11 mm, ovoid, shortly rostrate, glabrous, ±enclosed by sepals; seeds 6–8 × 4–5 mm, minutely puberulent.

#### Illustration.

**Figure 139.**
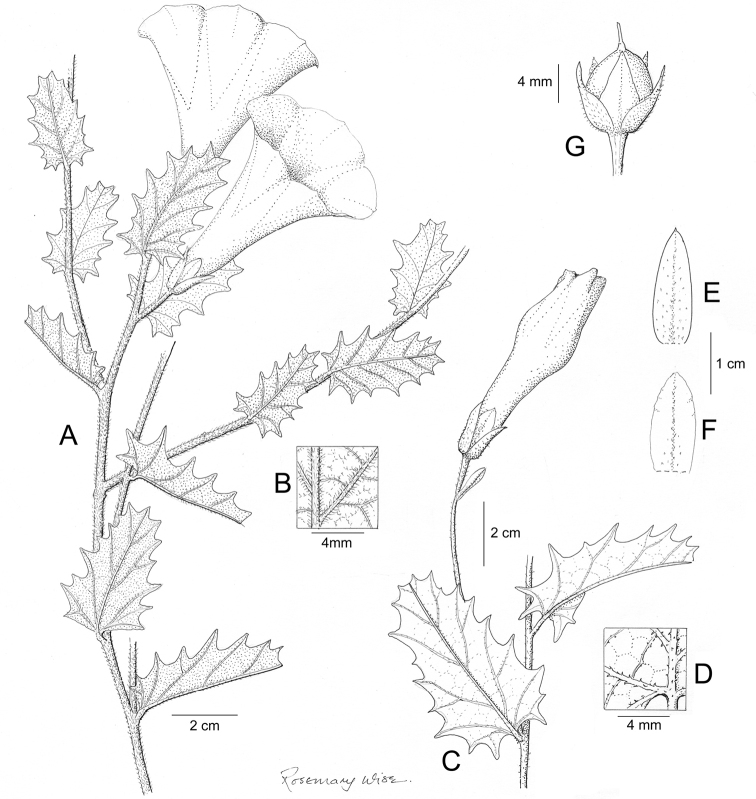
*Ipomoea stans*. **A** habit (1) **B** abaxial leaf surface **C** habit (2) **D** abaxial leaf surface **E** outer sepal **F** inner sepal **G** calyx and capsule. Drawn by Rosemary Wise **A, B** from *Pringle* 4488; **C–G** from *Bourgeau* 46.

Carranza (2007: 61); Figures 10G, 139.

#### Distribution.

Locally common in open pine forest, dry scrub and secondary vegetation, 1300–2700 m. Endemic to central Mexico.

**MEXICO. Est. México & Dist. Fed.**: *Y. Mexia* 2751 (BM, P); Sierra de Guadelupe, *E. Bourgeau* 496 (BM, MO, P, S); ibid., *E.K. Balls & W.B. Gourlay* 4948 (K). **Guanajuato**: *E. Ventura & E. López* 6964 (MEXU). **Guerrero**: *P. Tenorio et al.* 9566 (MO). **Hidalgo**: Pachuca, *C.G. Pringle* 6915 (BM, K, P, S); *H. Piug* 4797 (P). **Jalisco**: Guadalajara, *C.G. Pringle* 4488 (BM) – var. hirsuta; Río Blanco, *E. Palmer* 324 (BM, K) – var. hirsuta; Zapopan, *A. Rodríguez & P. Montiel-Moncayo* 6340(IEB) – var. hirsuta. **Michoacán**: Morelia, Punguato, *G. Arsène* 5967 (BM, S, US); Tiripetio, Morelia, *G. Cornejo Tenorio* 3475 (K). **Oaxaca**: *Ghiesbreght* s.n. (K, P); Tihuacan, *C. Conzatti* 164 (K). **Puebla**: *P. Tenorio & C. Romero* 6828 (MO); San Antonio, *E.K. Balls & W.B. Gourlay* 4510 (BM, K); Juan N. Méndez, *J.I. Calzada* 24405 (K, MEXU); San Luis Tultitlanapa, *C.A. Purpus* 3367 (BM). **Querétaro**: Colón, Galerías, *E. Carranza & E. Pérez* 4907 (IEB, MEXU). **San Luís Potosí**: *Parry & Palmer* 627 (BM, MO). **Veracruz**: Totalco, *M. Vásquez* 2100 (BM, K, MEXU). **Zacatecas**: *Dressler* 71 (MO); *Hartweg* s.n. (K).

#### Typification.

McDonald (1994: 133) designated the lectotype from MA but did not annotate or specify the sheet. Hence we have redesignated MA475857 as the lectotype.

#### Note.

Very variable in indumentum and size and shape of sepals and bracteoles. Very hairy plants with relatively short but broad sepals from Jalisco (*Pringle* 4488, *E. Palmer* 324, *A. Rodríguez & P. Montiel-Moncayo* 6340) may be recognised as **var. hirsuta**.

### 277. Ipomoea tacambarensis

Taxon classificationPlantaeSolanalesConvolvulaceae

Carranza, Sida 20: 1351. 2003. (Carranza 2003: 1351)

#### Type.

MEXICO. Michoacán, Tacámbaro, *E. Carranza & V.W. Steinmann* 6393 (holotype IEB000164902, isotypes CAS, IEB, MEXU, MICH, TEX, XAL).

#### Description.

Clearly resembling *Ipomoea stans* in the distinct leaves and erect habit but differing as follows. Perennial herb with stout, erect stems to 2.2 m. Leaves distinctly petiolate, mostly 16–30 × 12–24 cm; petioles mostly 2–4.5 cm. Inflorescence of axillary and terminal cymes with 5–15 flowers, appearing a many-flowered panicle; peduncles 9–18 cm; bracteoles 12–17 mm long, oblong-lanceolate, caducous; pedicels 12–30 mm long; sepals subequal, scarious, 15–17 mm, oblong-ovate, usually glabrous; corolla 7–9 cm long, deep red., presumably glabrous.

**Ilustration**. Carranza 2003: 1352.

#### Distribution.

Endemic to the Balsas depression area in Michoacán State in central Mexico.

**MEXICO. Michoacán**: Tacámboro, Punta de la Loma-Paso de Morelos, *Carranza & V.W. Steinmann* 6397 (IEB).

#### Note.

Resembles a large vigorous form of *Ipomoea stans*.

### 278. Ipomoea ancisa

Taxon classificationPlantaeSolanalesConvolvulaceae

House, Ann. New York Acad. Sci. 18(6): 187. 1908. (House 1908b: 187)

#### Type.

MEXICO. Chihuahua, below Pacheco, *E.W. Nelson* 6276 (holotype US059993, isotypes GH, K, NY).

#### Description.

Erect perennial herb or subshrub to 1.5 m, often much branched, glabrous in all parts. Leaves shortly petiolate, up to 11 cm long, ±pinnately divided into filiform segments 1.5–8 × 0.05–0.1 cm; petioles 0.5–0.8 cm. Inflorescence of long-pedunculate, solitary or paired axillary flowers; peduncles 5–11 cm long, usually straight and rather stout; bracteoles ovate-deltoid, 1–2 mm long, deciduous; pedicels 1–2 cm, in fruit widening upwards and becoming recurved; sepals slightly unequal, outermost 5–7 × 4.5–6 mm, broadly ovate to suborbuicular, rounded, with broad scarious margins; inner conspicuously larger 8–10 × 7–8 mm, broadly elliptic, rounded, margins broad, scarious but the midvein extending to apex; corolla 9–10 cm long. funnel-shaped, glabrous, white to pale pink, limb entire, 8 cm diam. Capsules subglobose, 15–16 mm, glabrous; seeds 7–8 × 6 mm, glabrous.

#### Distribution.

Locally common in northern Mexico in the Chihuahua-Sonora border areas at around 1400–2000 m, where it grows in very dry oak woodland on rocky slopes. Endemic to Mexico.

**MEXICO. Chihuahua**: Mun. De Madera, *R. Spellenberg* 13835 (ARIZ, NMC); Río Mayo, *H.S. Gentry* 2648 (K, MEXU); Pacheco, Bowman Ranch, *J. Spencer & N.D. Atwood* 644 (K). **Sonora**: Mun. Yécora, *Van Devender & Reina G.* 2000-663 (ARIZ); Cañon de Huépari, *S.S. White* 2692 (MEXU); 10.3 miles E of Yécora, *M. Fishbein et al.* 2546 (ARIZ, MEXU).

#### Note.

Sometimes treated as a form of *Ipomoea sescossiana* but distinguished by the much longer leaves with filiform leaf segments < 1 mm in width. Molecular studies raise doubts about the distinctness of these two species, although they are easily separated morphologically.

### 279. Ipomoea sescossiana

Taxon classificationPlantaeSolanalesConvolvulaceae

Baillon, Bull. Mens. Soc. Linn. Paris 1: 385. 1883. (Baillon 1883: 385)

Ipomoea pringlei A. Gray, Proc. Amer. Acad. Arts 22: 307. 1887. (Gray 1887: 307). Type. MEXICO. Chihuahua, *C.G. Pringle* 782 (holotype GH00054532, isotypes AC, BM, COLO, F, IBUG, K, MA, MEXU, MIN, MO, MSC, NDG, NY, P, RSA, S, TEX).

#### Type.

MEXICO. San Luis de Potosí, *Sescosse* s.n. (lectotype P03560164, designated here; isolectotypes P, US).

#### Description.

Bushy perennial herb to c. 1 m, plant entirely glabrous. Leaves shortly petiolate, short, 1.5–3.5 cm long, ±pinnately divided into linear segments 0.5–1.5 × 0.1 cm; petioles 0.2–0.7 cm. Inflorescence of solitary, long-pedunculate flowers; peduncles stout, 1–5 cm; bracteoles 1–2 mm, lanceolate, caducous; pedicels 0.6–1.6 cm, widened upwards; sepals unequal, outer 5–7 × 3–4 mm, broadly or narrowly ovate-elliptic, obtuse, scarious-margined, inner 9–10 × 7 mm, obovate or broadly elliptic, rounded with broad, scarious margins and green centre; corolla 7.5–9 cm long, deep bluish-pink with a pale tube, glabrous, funnel-shaped, limb unlobed, 4–7 cm diam. Capsules 10–18 × 8–10 mm, ovoid, rostrate, mucro c. 3 mm long, glabrous; seeds 7 × 4 mm (possibly immature), pubescent.

#### Illustration.

Figure 140.

**Figure 140.**
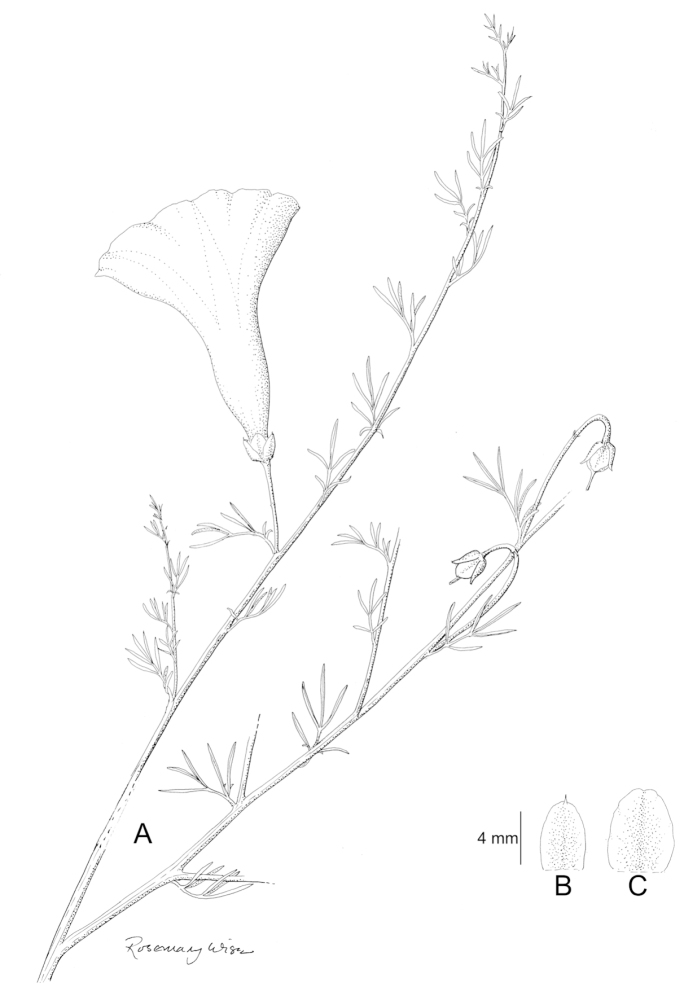
*Ipomoea sescossiana*. **A** habit **B** outer sepal **C** inner sepal. Drawn by Rosemary Wise from *Pringle* 782.

#### Distribution.

Endemic to northern Mexico growing from 1600 to 2350 m on dry rocky mountains.

**MEXICO. Aguas Calientes**: Tepezalá, *De La Cerda-García* 1416 (IEB). **Chihuahua**: Mun. Guerrero, *R. Spellenberg* 13842 (ARIZ, NMC); 41 km S. of Villa Ahumada, *J. Henrickson* 5867 (MEXU); Los Encinillos, *H.S. Gentry* 8218 (MEXU). **Coahuila**: 8.4 miles S of Moctezuma, *R. Worthington* 7284 (ARIZ, TEX, UTEP). km 15 W of Concepción del Oro, *L.R. Stanford et al.* 534 (ARIZ, NY). **Durango**: El Oro to Guanacivi, *E.W. Nelson* 4729 (K); *L. McGill* 9305 (ASU). **San Luis de Potosí**: *C.L. Lundell* 5149 (TEX); Venado, *F. Sánchez* 412 (ASU, DES, MEXU). **Sonora**: fide Felger et al. (2012). **Zacatecas**: Junction of Rutas 54 and 45, *W.L. Wagner & J.C. Solomon* 4212 (MO); NW of Fresnillo, *G.L. Webster & G.J. Breckon* 15505 (MEXU).

• Molecular sequence data suggests species 280–288 (and possibly 289) form a natural group but no obvious morphological feature seems to unite the whole clade although individual species clusters are readily discernible.

### 280. Ipomoea pittieri

Taxon classificationPlantaeSolanalesConvolvulaceae

O’Donell, Lilloa 23: 499. 1950. (O’Donell 1950b: 499)

#### Type.

VENEZUELA. Guárico, Laguna de la Mesa de El Sombrero, *H. Pittier* 12470 (holotype NY00319208, isotypes G, M, NY, VEN, US).

#### Description.

Prostrate herb, stems somewhat succulent, rooting at the nodes, glabrous. Leaves long–petiolate, palmately divided into 5–7 lobes, appearing to be formed of 3 leaflets, the central leaflet elliptic to lanceolate, 2–3.5 × 0.3–0.8 cm, basally attenuate, the lateral segments bipartite, sometimes with an additional simple lobe; petioles 3.5–7.5 cm. Flowers solitary, axillary; peduncles 0–2 mm; bracteoles 2–3 mm, obovate acute, scarious; pedicels very long, 3–15 cm; sepals similar, 3–3.5 mm long, oblong–elliptic, obtuse to emarginate, somewhat scarious, glabrous; corolla 2–3 cm long, funnel–shaped, pink with dark throat, glabrous, limb unlobed, c. 1.5 cm diam.

#### Distribution.

Seasonally flooded plain in the Llanos of Venezuela and the lower Magdalena in Colombia.

**COLOMBIA. Atlántico**: *A. Dugand* 4828 (US).

**VENEZUELA. Anzoátegui**: Río Caní, entre Guanipa y Cantaura, *H. Pittier* 15111 (VEN). **Apure**: just south of Mantecal, *G. Davidse et al.* 3886 (MO, FTG); Montecal, *B. Sergios* (MO, PORT); Sabana de la Candelaria, *H. Guyon* 131 (P). **Falcón**: Carretera Marsillal–Geritu, *B. Trujillo* 8804, (ARIZ). **Guárico**: Calabozo, Palmar de las Barbitas, *R.A. Montes* 933 (MO).

#### Note.

A very distinctive prostrate rooting herb with palmately divided leaves.

### 281. Ipomoea dumetorum

Taxon classificationPlantaeSolanalesConvolvulaceae

Willd. ex Roem. & Schult., Syst. Veg. 4: 789. 1819. (Roemer and Schultes 1819: 789)

Ipomoea serotina Roem. & Schult., Syst. Veg. 4: 215. 1819. (Roemer and Schultes 1819: 215). Type. Origin unknown, Balbiss.n. (holotype TO).
Convolvulus pauciflorus Willd. ex Roem. & Schult., Syst. Veg. 4: 789. 1819. (Roemer and Schultes 1819: 789). Type. PERU. Humboldt & Bonplands.n. (B–W 03695–01).
Convolvulus dumetorum Kunth, Nov. Gen. Sp. Pl. 3: 101. 1818 [pub. 1819]. (Kunth 1819: 101), non Ipomoea dumetorum Willd ex Roem. & Schult. (1819). Type. COLOMBIA. “in temeratis Andinum Quinduensium”, Bonplands.n. (holotype P00670749).
Convolvulus pulchellus Kunth, Nov. Gen. Sp. 3: 101. 1818 [pub. 1819]. (Kunth 1819: 101). Type. PERU. Bonplands.n. (holotype P00670748!).
Ipomoea pulchella (Kunth) G. Don, Gen. Hist. 4: 276. 1838. (Don 1838: 276), nom. illeg., non Ipomoea pulchella Roth (1821).
Convolvulus glaucescens Kunth, Nov. Gen. Sp. Nov. Gen. Sp. 3: 101. 1818. [pub. 1819]. (Kunth 1819: 101). Type. ECUADOR. Quito, Bonpland & Humboldt (P00670750!).
Ipomoea glaucescens (Kunth) G. Don, Gen. Hist. 4: 275. 1838. (Don 1838: 275).
Ipomoea dumetorum var. glaucescens (Kunth) Choisy in A.P. de Candolle, Prodr. 9: 378. 1845. (Choisy 1845: 378).
Quamoclit mutica Choisy in A.P. de Candolle, Prodr. 9: 335. 1845. (Choisy 1845: 335). Type. PERU. Lima, *Dombey*s.n. (Probable holotype P00666112).
Ipomoea oligantha Choisy in A.P. de Candolle, Prodr. 9: 380. 1845. (Choisy 1845: 380). Type. Based on Convolvulus pauciflorus Willd. ex Roem. & Schult. and C. pulchellus Kunth
Ipomoea chilensis A. Braun & C.D. Bouché, Index Sem. [Berlin], append. 1: 1. 1857 [pub. 1858]. (Braun and Bouché 1858: 1). Type. CHILE. Plant from Chile cultivated at B from seed sent by R.A. Philippi (?B†, n.v.).
Convolvulus pauciflorus var. chilensis (A. Braun & C.D. Bouché) Kuntze, Revis. Gen. Pl. 3: 214. 1898. (Kuntze 1898: 214).
Ipomoea paposana Phil., Viage Des. Atacama 299. 1860. (Philippi 1860: 210). Type. CHILE. Antofagusta, Paposo, R.A. Philippi s.n. (holotype SGO00003907).
Ipomoea dumetorum forma alba Moldenke, Phytologia 2: 224. 1947. (Moldenke 1947: 224). Type. ECUADOR. Loja, La Argelia, Espinosa 215a (holotype NY00319183).

#### Type.

Sine loc, probably Colombia or Ecuador, *Humboldt & Bonpland* (holotype B–W 03750-01 0), photo F).

#### Description.

Twining annual herb, stems glabrous, sometimes muricate. Leaves petiolate, mostly 4–10 × 3–7 cm, ovate–deltoid (rarely 3–lobed), hastate to broadly cordate, auricles rounded or acute, apex acute and finely mucronate, margin entire or with a large marginal tooth, both surfaces usually glabrous but sometimes abaxially pubescent on veins near base; petioles 2.5–4(–8) cm. Inflorescence of pedunculate axillary cymes; peduncles 2–8 cm, sometimes paired, glabrous or hirsute at base; bracteoles 2–3 mm, narrowly linear–lanceolate, acuminate, ±persistent; secondary peduncles short, 0.5–1 cm; pedicels mostly 1–1.5 cm, often recurved in bud; sepals slightly unequal, the inner slightly shorter than the outer, 5–6 mm, broadly ovate to elliptic, obtuse, mucronulate, pale green with prominent dark spots and pale margins, outer sepals sometimes muricate, usually glabrous, rarely pubescent; corolla 2–2.8 cm long, broadly funnel–shaped, glabrous, tube pale pink or white, limb pink (sometimes reported to be bluish), c. 2 cm diam. Capsules glabrous, ovoid, rostrate, the persistent style c. 2 mm long; seeds 5–6 × 2–2.5 cm, black, microscopically tomentellous.

#### Illustration.

Figure 2B; O’Donell (1959b: 153).

#### Distribution.

This is a common species extending from Argentina north along the Andes through Central America to reach the southern United States. It is noticeably more common south of the Equator than further north. It is mostly found in disturbed bushy places and on woodland borders between 2000 and 3000 m but reaches at least 3500 m in Bolivia and Peru and is reported from low altitudes in the coastal deserts of Chile around Antofagasta and commonly from the coastal Lomas in Peru, suggesting that the presence of damp cloud and mist are significant in its distribution.

**CHILE.** Antofagusta: type of *Ipomoea paposana*.

**ARGENTINA. Catamarca**: Andalgalá, *P. Jorgensen* 1213 (LIL, MO, US). **Jujuy**: Tumbaya, *A. Krapovickas et al.* 46658 (CTES), 47893 (CTES. MO). **Salta**: Los Toldos, *L.J. Novara* 5274 (G); R. de Lerma, *L.J. Novara* 6580 (G), La Caldera, *L.J. Novara* 6642 (G); Orán, *Pierotti* 1302 (LIL). **Tucumán**: *T. Meyer* 13978 (LIL); Burruyacu, *Monetti* 2032 (LIL, P).

**BOLIVIA. Chuquisaca**: Boeto, Nuevo Mundo, *J. Gutiérrez et al.* 603 (ARIZ, HSB, LPB, MO); Zudañez, A.N.M.I. El Palmar, *J.R.I. Wood et al.* 23298 (K, LPB). **Cochabamba**: Campero, Pasorapa–Bellavista, *J.R.I. Wood et al.* 19450 (BOLV, HSB, K, LPB, USZ); Capinota, Apillapampa, *E. Thomas* 371 (BOLV, LPB); Cercado: *E.K. Balls* 6214 (BM, BOLV, K, US). **La Paz**: Murillo, Valle de Zongo, *S.G. Beck* 3668 (MO, LPB); Saavedra, Charazani, *A. Fuentes et al.* 6840 (ARIZ, LPB, MO); Sud Yungas, Puente Chiltuayo, below Lambate, *J.R.I. Wood et al.29194* (LPB, USZ). **Santa Cruz**: Vallegrande, *G.A. Parada & V. Rojas* 2610 (OXF, MO, USZ). **Tarija**: Arce: Cerro Pabellón, *S.G. Beck et al.* 26076 (ARIZ, LPB); O’Connor, Narvaez, *J. Solomon* 10389 (LPB, MO).

**PERU. Amazonas**: Chachapoyas, *A. Mathews* s.n. (K). **Ancash**: *E. Cerrate* 550 (USM); *R. Ferreyra* 8668 (USM). **Apurimac**: *C. Vargas* 8760 (CUZ). **Arequipa**: Condesuyos, Chuquibamba, *D. Stafford* 1181 (BM, K); Mollendo, *D. Stafford* 832 (BM, K). **Cajamarca**: *C. Díaz & M. Severo Baldeón* 2873 (MO); *A. Sagástegui* 15377 (F). **Cusco**: Macchu–Picchu, *T.G. Tutin* 1274 (BM); Urubamba, *H.H. Iltis et al*. s.n. [19/12/1962] (K), Paucaratambo, *E.K. Balls* 6787 (BM, K). **Huánuco**: *Proaño* 175 (USM). **Ica**: Santiago, Lomas de Amara, *O. Whaley et al.* 1744 (K). **Junín**: Huancayo, *J. Soukup* 3152 (MO, P, S). **Lima**: Lomas de Atocongo, *T.W. Böcher et al.* 377 (K); Lomas de Chancayllo, *R. Ferreyra* 16599 (MO USM); *M. La Torre et al.* 973 (USM). **Moquegua**: *R. Ferreyra* 11603 (USM). **Piura**: *R. Ferreyra* 13757 (USM). **Tacna**: *R. Ferreyra* 12647 (USM).

**ECUADOR. Azuay**: *C.W.T. Penland & R.H. Summers* 1045 (F, US). **Cañar**: *T. Croat & M. Menke* 89025A (MO). **Chimbarazo**: *L. A. Mille* (US). **El Oro**: *G. Harling & Andersson* 18798 (GB). **Loja**: *B. MacBryde* 309 (MO); *Sparre* 16674 (S). **Manabí**: *E. Asplund* 15946 (S). **Pinchincha**: *C.E. Cerón* 15212 (MO). **Tungarahua**: *M. Acosta–Solis* 9313 (F); Ambato, *E. Bravo* 625 (QCA).

**COLOMBIA. Cundinamarca**: Bogotá, *J. Triana* 3801 (BM); *Goudot* (K). **Magdalena**: Sierra Nevada de Santa Marta, *J. Cuatrecasas* 24782A (COL, US). **Nariño**: *H.H. Martines* 29 (COL). **Quindío**: type of *Convolvulus dumetorum* Kunth

**VENEZUELA. Mérida/Táchira**: San José to Mucutuy, *C. Jeffrey et al.* 2120 (K).

**COSTA RICA.***M. Chavarría* 625 (INB).

**MEXICO. Est. México & Dist. Fed.**: Ciudad Universitaria, *R. Bye* 27004 (MEXU); Tepotzotlán, *J. Rzedowski* 36567 (MO); Polotitlán, *E. Matuda et al.* 26545 (MEXU); Pedregal, *E. Lyonnet* 108 (BM). **Guanajuato**: San Felipe, W of Altos de Ibarra, *G. Arias* 2297 (IEB); *R. & J.D. Galván* 2297 (MEXU). **Hidalgo**: 6 km al N de Tlalnalapa, *J. Rzedowski* 35931 (ASU, IEB, MEXU). **Jalisco**: *E. Bourgeau* 796 (P, S, US). **Michoacán**: Zacapu, W of La Angostura, *A. Grimaldo* 534 (IEB, MEXU); Pedregal de Arocutín, *M.E. Molino & S. Zamudio* 296 (IEB). **Morelos**: Huitzilac, *I. Diáz* 1145 (MEXU). **Oaxaca**: *C. Conzatti* 2274 (US) fide McDonald. **San Luis Potosí**: *J.G. Schaffner* 620 (GH, K).

**UNITED STATES. New Mexico**: White Mountains, *E.O. Wooton* 630 (NY, P); Organ Mountains, *A. McDonald* 140 (TEX). **Texas**: Mount Livermore, *Hinckley* 322 (NY).

#### Notes.

Although commonly misidentified, this species is readily identified in the field by its small pink (reported as pale blue in the northern hemisphere) flowers and pale green sepals with distinct dark spots. The sepals are usually completely glabrous but some specimens from Peru (*G. Calatuyud* 2382, 3261 (both MO, OXF) have pubescent sepals. It is sometimes confused with *Ipomoea aristolochiifolia* but usually grows at a higher altitude and the peduncle never passes through the sinus of the leaf base.

*Quamoclit mutica* was identified as *Ipomoea tricolor* by McPherson (1993) but the specimen at Paris is clearly *I. dumetorum* as suggested by Choisy’s inclusion of *Ipomoea serotina* under this species.

### 282. Ipomoea simulans

Taxon classificationPlantaeSolanalesConvolvulaceae

D. Hanb., J. Linn. Soc. Bot. 11: 281. 1871. (Hanbury 1871: 281)

#### Type.

Cultivated plant from MEXICO, Guanajuato, Sierra Gorda near San Luis de la Paz, *Finck* s.n. (lectotype K000612720, designated by McDonald 1987c: 62, isolectotype BM).

#### Description.

Slender twining perennial herb, stems glabrous, roots reported to be tuberous. Leaves petiolate, 4.5–11 × 1.5–6 cm, ovate, cordate, the auricles sometimes incurved and almost touching, apex acuminate, both surfaces glabrous; petioles 2–6.5 cm. Inflorescence of solitary (or paired) pedunculate, axillary flowers; peduncles 2–6 cm, sometimes arising through the leaf sinus, commonly flexuose; bracteoles 2 mm, oblong–lanceolate; pedicels 7–15 mm, distinctly thicker than the peduncles; sepals slightly unequal, ovate to ovate–oblong, obtuse to rounded, glabrous, the margins scarious, abaxial surface with dark spots, outer 4–6 × 5 mm, inner 6–8 × 5–6 mm; corolla 2.5–4.5 cm long, shortly funnel–shaped and flared from the base, blue (?) drying purple, glabrous, limb 2–4 cm diam. Capsules 9 × 6–7 mm, ovoid, rostrate, glabrous; seeds up to 4, 5 × 3–4 mm, glabrous.

#### Illustration.

McDonald (1987c: 85).

#### Distribution.

Endemic to central Mexico at altitudes of 1500–2500 m.

**MEXICO. Est. México & Dist. Fed.**: Temascaltepec, *G.B. Hinton* 8348 (GBH, K). **Hidalgo**: *A. Villa Kamel* 91 (IEB). **Michoacán**: Morelia, N of Zapote, *G. Arsène* s.n. [4/8/1910] (P). **Morelos**: Cuernavaca, *C.G. Pringle* 6565 (BM, GH, K, MO, S, US); Huitzilac, *J. Vásquez* 2300 (MEXU). **Oaxaca**: Cerro San Felipe, *C. Conzatti* 4174 (US); Santiago Textitlán, *A. Zarate Marcos* 683 (MEXU). **Querétaro**: Pinal de Amoles, *E. Carranza & E. Pérez* 5415 (IEB, MEXU); ibid., *S. Zamudio & E. Carranza* 6850 (IEB); Landa de Matamoros, *B. Servín* 1291 (IEB). **San Luis Potosí**: Xilitla, *E. Carranza & S. Zamudio* 5933 (IEB).

#### Notes.

Rather similar to *Ipomoea dumetorum* but perennial with tuberous roots and a larger corolla reaching 4.5 cm in length. The leaves are also more strongly sagittate.

This is the source of “Tampico Jalap”.

### 283. Ipomoea miquihuanensis

Taxon classificationPlantaeSolanalesConvolvulaceae

J.A. McDonald, Brittonia 39: 110. 1987. (McDonald 1987b: 110)

#### Type.

MEXICO. Tamaulipas, 7 km SW of Miquihuana, *Stanford, Retherford & Northcraft* 705 (holotype GH00054520, isotype MO).

#### Description.

Slender twining herb, stems glabrous, reddish. Leaves petiolate, ovate–deltoid, base cordate to sagittate with narrow acute to obtuse auricles, apex finely acuminate and mucronate, both surfaces glabrous; petioles 0.5–3 cm. Inflorescence of solitary axillary flowers; peduncles 1.2–3 cm; bracteoles minute, aristate; pedicels 3–11 mm; sepals unequal, ovate–oblong, obtuse to rounded, sometimes mucronulate, glabrous, dotted with dark glands, margins narrow, scarious, outer 3–4 × 2 mm, inner 4.5–6 × 3 mm; corolla 4–6 cm long, funnel–shaped, reddish–purple with paler tube, glabrous, limb c. 4 cm diam., subentire. Capsules and seeds unknown.

#### Illustration.

McDonald (1987c: 85).

#### Distribution.

Apparently rare in pine forest at 2000–3200 m in NE Mexico.

**MEXICO. Tamaulipas**: type of *Ipomoea miquihuanensis*. **San Luis de Potosí**: *M. Virlet d’Aoust* 1852 (P). **Nuevo León**: *J.C. Hinton* 19261 (GBH, n.v.).

#### Note.

The dark glands on the sepals and the high altitude habitat confirm the affinity with *Ipomoea dumetorum*, but it is easily distinguished by its much larger corolla.

### 284. Ipomoea caudata

Taxon classificationPlantaeSolanalesConvolvulaceae

Fernald, Proc. Amer. Acad. Arts 36: 498. 1901. (Fernald 1901: 498)

Ipomoea hintonii L. O. Williams, Econ Bot. 24: 400. 1970. (Williams 1970b: 400). Type. MEXICO. Est. México, Nanchititla, G.B. Hinton et al. 8474 (holotype F0054847, isotypes LL, MO, NY, US).

#### Type.

MEXICO. Morelos, Sierra de Tepoxtlán, *C.G. Pringle* 8448 (holotype GH00054487, isotypes AC, BM, CM, DAO, E, ENCB, F, GOET, ISC, K, M, MEXU, MICH, MIN, MSC, MO, NDG, NY, P, PH, RM, RSA, S, UC, US, VT).

#### Description.

Slender, probably twining perennial herb, stems glabrous, reaching 3 m. Leaves petiolate, 4–11 × 1–4.5 cm, narrowly ovate, long acuminate to a fine mucronulate point, base sagittate with acute, apiculate auricles, both surfaces glabrous, abaxially somewhat reticulate and somewhat glaucous; petioles 1.5–6.5 cm. Inflorescence of solitary (rarely in 2–3-flowered cymes) pedunculate flowers; peduncles 8–12 cm; bracteoles 1 mm, squamose, caducous; pedicels 18–30 mm; sepals very unequal, outer 3–6 × 3–4 mm, ovate, obtuse, scarious-margined, dotted with conspicuous dark glands, inner 8–11 mm, broadly oblong, retuse, mostly scarious except at base; corolla 3.5–5 cm long, salverform with a basal tube c. 4 cm long, pink, glabrous, limb short, c. 2 cm diam., stamens exserted. Capsules and seeds unknown.

#### Illustration.

McDonald (1987c: 85).

#### Distribution.

Endemic to seasonally upland pine and oak woodland in central Mexico.

**MEXICO. Est. México & Dist. Fed.**: type of *Ipomoea hintonii*. **Morelos**: Sierra de Tepoxtlán, *C.G. Pringle* 13590 (GH, S); Tlayacapan, Barranca Tepecapa, *R. Hernández-Cárdenas et al*. 522 (IEB); Tepozteco, *E. Lyonnet* 540800007 (IEB); *J. Espinosa* 79 (MEXU).

#### Note.

Close to *Ipomoea simulans* and *I. miquihuanensis* differing in the narrow corolla tube and exserted stamens.

### 285. Ipomoea tenuiloba

Taxon classificationPlantaeSolanalesConvolvulaceae

Torr., Rep. U.S. Mex. Bound. 2(1): 148–149. 1859. (Torrey 1859: 148)

Ipomoea lemmonii A. Gray, Proc. Amer. Acad. Arts 19: 91. 1884 [pub. 1883]. (Gray 1883: 91). Type. UNITED STATES. Arizona, mountains near Fort Huachuca, *J.G. Lemmon* 2840 (holotype GH00054461, isotypes CAS, P, US).
Ipomoea tenuiloba var. lemmonii (A. Gray) Yatsk. & C.T. Mason, Madroño 31(2): 106. 1984. (Yatskievych and Mason 1984: 106).
Ipomoea leptosiphon S. Watson, Proc. Amer. Acad. Arts 23: 280. 1888. (Watson 188: 280). Type. MEXICO. Chihuahua, *C.G. Pringle* 1337 (holotype GH00054514, isotypes E, F, K, NDG, NY, PH, TEX, US).

#### Type.

UNITED STATES. Texas, near Puerto de Paysano, *J. M. Bigelow et al.* s.n. (holotype NY00319068, isotype US).

#### Description.

Perennial herb from a thickened tuberous rootstock (like an elongated bulb), scrambling or twining, completely glabrous. Leaves petiolate, digitate with 5–9 (usually 8) linear, acute leaflets 1–6 × 0.05–0.25(–0.6) cm; petioles 5–35 mm. Flowers axillary, usually solitary, pedunculate; peduncles 1–5 cm, often bent at apex ; bracteoles 1–2 mm, filiform, tardily deciduous; pedicels 2–8 mm, thickened upwards, recurving in fruit; sepals unequal, glabrous with scarious margins, broader in fruit, outer 5–9 × 2–3 mm, lanceolate, acute, mucronate, sometimes muricate abaxially, inner 7–14 × 3–4 mm, oblanceolate, rounded, shortly mucronate; corolla 3.5–10 cm long, with a long trumpet-shaped tube gradually widened in upper half to c. 1.5 cm, white, pale pink or purplish, glabrous. midpetaline bands terminating in a mucro, limb c. 2 cm diam.; stamens held at mouth of corolla. Capsules held on a recurved pedicel, compressed-globose, 6–9 mm diam., glabrous, rostrate with mucro up to 5 mm long; seeds 2.5–5 × 2–4 mm, ellipsoid, black.

#### Distribution.

Semi-desert areas of the United States Southwest and NW Mexico, mostly growing at altitudes of 1700–2200 m, but rather local and infrequently collected.

**MEXICO. Chihuahua**: S of Guadelupe, *E. H. Nelson* 4822 (K, US); Temosachi, *J. Laferrière* 1727 (ARIZ, MEXU); Sierra Canelo, Río Mayo, *H.S. Gentry* 2529 (ARIZ, F, GH, K, MEXU, US); Colonia García, *C.H.T. Townsend & C.M. Barber* 271 (BM, F, K, MO, NY, P, US). **Durango**: Durango-Mazatlan, *G. Yatskievych* 85-236 (INDIANA, ARIZ); Tepehuanes *O. Bravo Bolañsa* 150 (MEXU). **Sonora**: Río Bavispe Region, Sierra de el Tigre, *S.S. White* 3474 (ARIZ, GH); Yécora, *T.R. Van Devender & A.L. Reina-G* 2001-844 (MEXU).

**UNITED STATES. Arizona**: Pima Co, Santa Catalina Mountains, *J. Tedford* 06-218 (ARIZ); Cochise Co., Mule Pass, *F.W. Reichenbacher* 811 (ARIZ); ibid., Chiricaha Nat. Mon., *D.G. Doramus* s.n. (ARIZ); ibid., Coronado Nat. Forest, *K. Stieve* 49 (ASU). **New Mexico**: Hidalgo Co, Peloncillo Mts., *E. Makings & C.D. Littlefield* 3054b (DES, UCR). **Texas**: type collection.

#### Note.

This species can be recognised by its distinctive subhypocrateriform corolla, the tube only expanding just below the limb. Yatskievich and Mason (1984) and McDonald (1995) recognised two varieties but these overlap morphologically and geographically. The type has a pale pink or white corolla mostly 5–10 cm long with the inner sepals 11–14 mm in length. **Var. lemmonii** is more western in its distribution and has a darker, smaller corolla 3.3–5.2 cm long with shorter inner sepals <10 mm long.

### 286. Ipomoea madrensis

Taxon classificationPlantaeSolanalesConvolvulaceae

S. Watson, Proc. Amer. Acad. Arts 23: 281. 1888. (Watson 1b888: 281)

#### Type.

MEXICO. Chihuahua, *C.G. Pringle* 1338 (holotype GH00054517, isotypes: E, F, GH, K, MEXU, MIN, MO, NDG, NY, PH, US).

#### Description.

Perennial herb to 50 cm from a bulb-like tuber, stems ascending or decumbent, glabrous. Leaves shortly petiolate, 1.5–5 × 0.3–4 cm, rhombic, oblong to oblong-lanceolate, acute and mucronate, cuneate at base, entire or with 2–4 small linear-oblong lobes from the base of the main lobe or ± palmately divided into 3 leaflets, glabrous; petioles 0.5–1.5 cm. Flowers solitary, rarely in pairs, axillary; peduncles 0.3–3.3 cm, usually glabrous; bracteoles 1–3 × 2 mm, filiform, moderately persistent; pedicels 4–11 mm, muricate; sepals subequal, narrowly ovate, acuminate, outer 6–10 × 4–6 mm, abaxially muricate, inner slightly larger, the midrib muricate, the margins glabrous, scarious; corolla 5–5.5 cm long, funnel-shaped, glabrous, tube white, the limb purplish, 2.5–3 cm diam. Capsules 3-locular, depressed-subglobose, 5–6 mm wide, glabrous; seeds c. 2 mm wide, densely puberulent.

#### Illustration.

Carranza (2007: 71).

#### Distribution.

Endemic to northern and central Mexico, growing in pine and oak woodland, 1600–2700 m.

**MEXICO. Aguascalientes**: *J. Rzedowski* 14159 (MEXU); Sierra del Laurel. *R. McVaugh* 18383 (MICH). **Chihuahua**: La mesa de Urucán, *P. Tenorio & C. Romero* 6158 (MO); Caborachi, *R. Hernández* 8527 (MEXU). **Durango**: *González & Acevedo* 1805 (MEXU). **Est. Mexico & Dist. Fed.**: Temascaltepec, Timbres, *G.B. Hinton* 1234 (F, GH, K, NY). **Guanajuato**: Sierra Santa Rosa, *E. Carranza & H. Zepeda* 5022 (IEB). **Michoacán**: Cerro El Aguila, *G. Cornejo Tenorio* 2810 (IEB). **Nayarit**: *J.N. Rose* 2109 (US). **Querétaro**: Amealco-San Juan del Río, J. *Rzedowski* 48571 (IEB). **Sonora**: Yécora, *A.L. Reina-G* 2000-541 (ARIZ). **Zacatecas**: *J.N. Rose* 2780 (US).

#### Note.

The leaves are somewhat polymorphic varying from entire to palmately lobed, a feature that together with the muricate sepals suggests a relationship with *Ipomoea plummerae*, which is supported by molecular results.

### 287. Ipomoea plummerae

Taxon classificationPlantaeSolanalesConvolvulaceae

A. Gray, Syn. Fl. N. Amer. Ed. 2, 1: 434. 1886. (Gray 1886: 434)

Ipomoea plummerae var. typica Ooststr., Recueil. Trav. Bot. Neerl. 30: 210. 1933. (Ooststroom 1933: 210), nom. illeg. superfl.
Quamoclit pedata M. Martens & Galeoti, Bull. Acad. Roy. Sci. Bruxelles 12: 270. 1845. (Martens and Galeotti 1845: 270), non Ipomoea pedata G. Don (1838). Type. MEXICO. [Jalisco], Guadalajara, *H. Galeotti* 1392 (lectotype BR00006972714, designated here).
Ipomoea capillacea var. patens A. Gray, Syn. Fl. N. Amer., ed. 2: 434. 1886. (Gray 1886: 434). Type. MEXICO. Nuevo Leon, *E. Palmer* 910 (lectotype GH00054486 (portion on left side of sheet), designated by McDonald 1995: 111).
Ipomoea patens (A. Gray) House Ann, New York Acad. Sci. 18: 237. 1908. (House 1908b: 237).
Ipomoea armata var. patens (A. Gray) M.E. Jones, Contr. W. Bot. 12: 53. 1908 (Jones 1908: 53).
Ipomoea minuta R.E. Fries, Nova Acta Regiae Soc. Sci. Upsal. 4: 113. 1905 (Fries (1905: 113). Type. ARGENTINA. Jujuy, Santa Catalina, Kurtz 11437 (lectotype S, designated by McDonald (1995: 111) portion with barcode S07-4678 redesignated as lectotype here).
Ipomoea cuneifolia A. Gray, Proc. Amer. Acad. Arts 19: 90. 1884 [pub. 1883]. (Gray 1883: 434), nom. illeg., non Ipomoea cuneifolia Meisn. (1869). Type. UNITED STATES., Arizona, *J.G. Lemmon* 2837 (holotype GH00054458, isotypes BM, CAS, F, MO, NY!, US!).
Ipomoea egregia House, Torreya 6: 124. 1906. (House 1906: 124). Type. based on Ipomoea cuneifolia A. Gray
Ipomoea plummerae var. cuneifolia (A. Gray) J.F. Macbr., Publ. Field Mus. Nat. Hist., Bot. Ser. 11: 4. 1931. (Macbride 1931: 4).
Ipomoea plummerae forma adiantifolia Ooststr., Recueil. Trav. Bot. Neerl. 30: 210. 1933. (Ooststroom 1933: 210). Type. PERU. Arequipa, A. Weberbauer 1561 (holotype B?†.).
Ipomoea minuta forma adiantifolia (Ooststr.) O’Donell, Lilloa 29: 193. 1959. (O’Donell 1959b: 193).
Ipomoea plummerae forma rhombifolia Ooststr., Recueil. Trav. Bot. Neerl. 30: 221. 1936. (Ooststroom 1936: 221). Type. BOLIVIA. Potosi, Lagunillas, *M. Cardenas* 430 (lectotype US00390637, designated by McDonald (1995: 115).
Ipomoea plummerae var. cupulata J.A. McDonald, Harvard Pap. Bot. 6: 115. 1995. (McDonald 1995: 115). Type. MEXICO. Chihuahua, Río Mayo, *H.S. Gentry* 2541 (holotype GH00054529, isotypes ARIZ, CAS, F, K).

#### Type.

UNITED STATES. South Arizona, *Wright, Loew, Mr and Mrs J.G. Lemmon* 2839 (holotype GH00054464 (portion on top right of sheet), isotypes UC).

#### Description.

Completely glabrous perennial herb with subterranean bulb-like root tuber; stems usually several, branched near base, decumbent or ascending, up to 30 cm long but often very short. Leaves petiolate, small, digitately divided into 5–7 segments, segments 3–30 × 1–3 mm, linear to linear-oblanceolate, obtuse and mucronate or (less commonly) simple, rhomboidal, basally cuneate but apically acute or 3-fid with acute lobes; petioles 3–15 mm. Flowers solitary, axillary; peduncles 5–12(–40) mm; bracteoles 1–2 mm, filiform; pedicels 1–4 mm; sepals slightly unequal outer 5.5.–7 mm, oblong, acute to obtuse, muricate, inner similar but 7–8 mm and with broad scarious margins and green, central, sometimes muricate midrib; corolla 2–3 cm long, glabrous, funnel-shaped; tube dirty white, limb dark pink, c. 2 cm diam., unlobed. Capsules 6–7 mm long, subglobose, glabrous, the slender style persistent, up to 6-seeded; seeds 3–4 mm, dark brown, minutely tomentellous.

#### Illustration.

**Figure 141.**
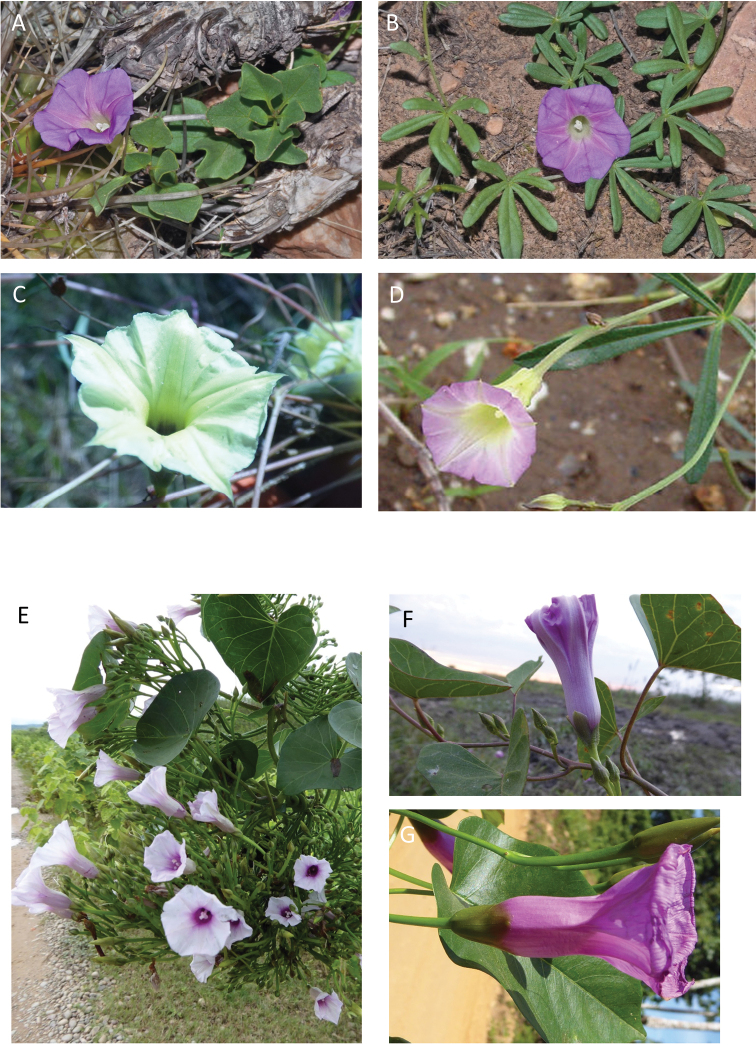
Photographs of *Ipomoea* species **A***I. plummerae*, form with entire leaves **B***I. plummerae*, form with lobed leaves **C***I. longeramosa***D***I. costellata***E***I. amnicola***F***I. paludicola***G***I. chondrosepala*. **A, B** Mario Giorgetti **C** Rosemary Clegg **D** Firefly Forest **E–G** Maira Martinez.

Figures 2E, 141A, B; O’Donell (1959b: 191).

#### Distribution.

A species with a disjunct distribution closely paralleling that of *Ipomoea pubescens*, being found in Colombia, Peru, Bolivia and Argentina in South America and the United States and Mexico in North America. It is characteristic of open stony hillsides with subpuna vegetation between 2400 and 4000 m, the lower altitudes recorded from the extreme south and the extreme north of its range. It usually grows in small populations, often only a single plant being found.

**ARGENTINA. Catamarca**: El Candado, *P. Jorgensen* 1043 (LIL, MO); Ambat, *A.T. Hunziker & T di Fulvio* 19823 (CORD); *R. Schreiter* 10559 (LIL). **Córdoba**: *A. Burkart* 7447 (SI). Jujuy: *F.O. Zuloaga et al.* 6039 (MO). **La Rioja**: *Kurtz* 15482 (CORD). **Salta**: *L.J. Novara* 1601 (S), *E. Zardini et al.* 1880 (MO). **San Luis**: *Vignati* 169 (LP). **Tucumán**: *C. Olrog* s.n. [1/1951] (S); Tafi, *Schickendantz* 1892 (LIL).

**BOLIVIA. Chuquisaca**: Oropeza: *H. Huaylla* 989 (MO); Tomina, El Villar, *Carretero et al.* 1153 (ARIZ, HSB, MO); Zudañez, A.N.M.I. El Palmar, *J.R.I. Wood* 17843 (K, LPB). **Cochabamba**: Arque, *P. Ibisch & Rojas* 745 (BOLV, LPB); Carrasco, López Mendoza, *J.R.I. Wood* 8959 (K, LPB). Cercado, *M. Cardenas* 2261 (GH, LIL); Quillacollo, *M. Zarate et al.* 2180 (BOLV, LPB, MO). **La Paz**: Larecaja, *G. Mandon* 1490 (BM, K, P, NY, S); Murillo, Aranjuez, *S.G. Beck* 24966 (LPB). **Potosí**: Bustillos, *S.G. Beck* 6172 (LPB); Charcas, Torotoro, *J.R.I. Wood et al.* 19214 (BOLV, K, LPB); Frías, La Palca-Cayara, *J.R.I. Wood* 9021 (K, LPB); Sud Chichas, *F. Zenteno* 11610 (LPB). **Santa Cruz**: Vallegrande, *L. Arroyo et al.* 5466 (USZ). **Tarija**: Arce, Cerro Pabellón, *S.G. Beck et al.* 26106 (LPB); Cercado, Tucumilla, *K. Fiebrig* 2446 (BM, K, GH, P).

**PERU. Apurimac**: Grau, *C. Vargas* 12413 (CUZ); Abancay, *C. Vargas* 9055 (CUZ). **Arequipa**: Quequena, *D. Stafford* 1293 (BM). **Ayacucho**: *J. Barrientos* 78 (USM). **Cusco**: *D. Stafford* 221 (BM); Urubamba, *P. Nuñez* 7444 (MO, USM); Calca, *C. Vargas* 6364 (CUZ). **Lima**: *E. Asplund* 13822 (S); Surco, *R. Ferreyra* 9654 (USM). **Junín**: Tarma, *P.C. Hutchison & O. Tovar* 4206 (MO). **Moquequa**: Carumas, *A. Weberbauer* 7275 (BM, S); *D. Montesinos* 751 (USM). **Puno**: *J. Soukup* 485 (P); *C. Vargas* 12500 (CUZ).

**COLOMBIA. Cundinamarca**: *M. Schneider* 1126 (S); Mosquera, *R. Jaramillo* (COL).

**MEXICO. Chihuahua**: Juárez, *E.W. Nelson* 6085 (GH, K, US); Colonia García, *C.H.T. Townsend & C.M. Barber* 228 (BM, F, GH, P, US); Arroyo Hondo, Sierra Charuco, *H.S. Gentry* 1787 (CAS, F, K, US).**Coahuila**: fide McDonald (1995). **Durango**: El Salto, *P. Tenorio & J. Ignacio* 9712 (MEXU, MO); Mezquital. Santa María de Ocotán, *S. Acevedo* 295 (IEB). **Est. México & Dist. Fed.**: *J.G. Schaffner* s.n. (P); Texcoco, *A. Ventura* 4225 (IEB). **Guerrero**: Mina, Manchon-Aguazarca, *G.B. Hinton* 9651 (BM, CAS, F, GH, K, NY, US). **Jalisco**: Balaños, *L.M. Villareal* 1924 (IEB). **Michoacán**: Morelia, *G. Arsène* 5204 (GH, US); Pátzcuaro, Cerro Blanco, *E. Pérez* 4005 (IEB); Uruapan, *G.B. Hinton* 15439 (K). **Puebla**: Cerro de Gavilán, *C.A. Purpus* 3906 (BM, CAS, E, NY, US). **Sinaloa**: Concordia, Cañon Santa María, *A.C. Sanders et al.* 21084 (UCR). **Sonora**: Navjoa, *T.R. Van Devender et al.* 93-1245 (ARIZ). **Veracruz**: *Nevling & Gómez-Pompa* 1820 (F, MEXU). **Zacatecas**: fide McDonald (1995).

**UNITED STATES. Arizona**: Coconino Co., *E. Lehto* 3454 (ARIZ, BM); Cochise Co., Chiricahua Mts., *S. Walker* s.n. [11/8/1963] (UTC); ibid., *W. Hodgson* 2600 (DES). **New Mexico**: White Mountains, *E. O. Wooton* 627 (CAS, GH, K, NMC, P, US); *C. Wright* 1616 (K); Mogollon Mts., *O.B. Metcalfe* 271 (K). **Texas**: Glass Mountains, *Warnock* 160 (GH).

#### Typifications.

There are several problems with the typification of the names listed above. The sheet with barcode GH00054464 (*Ipomoea plummerae*) consists of two collections of which only the portion towards the top and on the right of the sheet is the lectotype (*Wright, Loew, Mr and Mrs J.G. Lemmon* 2839), the other collection on the left (mounted on whiter paper) is *Wright* 1616, which is not part of the lectotype. Similarly, GH00054486 (Ipomoea capillacea var. patens) consists of two collections, of which only the plant on the left (whiter) side of the sheet is *Palmer* 910, constituting the lectotype. In the case of *Quamoclit pedata* there are three syntypes and we have designated the sheet annotated “holotype” by McDonald as the lectotype. McDonald chose *Kurtz* 11437 as the lectotype of *Ipomoea minuta* but it is actually a mixed gathering consisting of a typical plant (S07-4678) and forma adiantifolia (S12-7294), as annotated by O’Donell. In order to clarify the ambiguity we have redesignated the portion on the left of the sheet with barcode S07-4678 as the lectotype.

#### Notes.

*Ipomoea plummerae* is exceptionally variable in its leaf form and various infraspecific taxa have been recognised. The typical plant has leaves digitately divided into 5–7 linear leaflets. However, plants with rhomboidal leaves occur sporadically, the leaves basally cuneate but apically acute, the margin crenate, deeply 3–5-toothed or variously lobed. These are found usually in the presence of typical plants and can be recognised as forma adiantifolia if so desired. Ooststroom (1933: 208) illustrates the range of variation found in the leaf shape of this species in Peru. Forma adiantifolia appears to be restricted to the United States in the northern hemisphere but is common in Peru, Bolivia and Argentina.

In NW Mexico there occurs a relatively distinct variety with a nearly salverform corolla and a cylindrical basal tube 10–14 mm long. This can be recognised as var. cupulata.

The root is eaten in some Andean communities (Gutiérrez-R, 2016).

### 288. Ipomoea capillacea

Taxon classificationPlantaeSolanalesConvolvulaceae

(Kunth) G. Don, Gen. Hist. 4: 267. 1838. (Don 1838: 267)

Convolvulus capillaceus Kunth, Nov. Gen. Sp. 3: 97. 1818 [pub. 1819]. Kunth 1819: 97). Type. COLOMBIA. Humboldt & Bonpland 2046 (holotype P00670737).
Ipomoea muricata Cav., Icones 5: 52, pl. 478, f.2. 1794 [pub. 1799], nom. illeg., non Ipomoea muricata (L.) Jacq. (1798). Type. MEXICO. Guanajuato, *L. Née*s.n. (lectotype MA 475850, designated here).
Ipomoea armata Roem. & Schult., Syst. Veg. 4: 214. 1819. (Roemer and Schultes 1819: 214). Type. Based on Ipomoea muricata Cav.
Leptocallis armata (Roem. & Schult.) G. Don in Sweet, Hort. Brit., ed. 3: 482. 1839. (Sweet 1839: 482).
Ipomoea muricatisepala Matuda, Ann. Inst. Biol. Mex. 34: 124. 1964. (Matuda 1964: 124), nom. superfl. Type. Based on Ipomoea muricata Cav.
Ipomoea pseudo-linum Pittier, J. Wash. Acad. Sci. 17: 287. 1927. (Pittier 1927: 287). Type. VENEZUELA. Dist. Fed.: sobre Caracas, *H. Pittier* 7279 (holotype VEN, isotypes GH00054611, US00111450).
Ipomoea muricata forma alba Woodson & Seibert, Ann. Missouri Bot. Gard. 24: 201. 1937. (Woodson and Seibert 1937: 201). Type. PANAMA. Chiriquí: Llanos del Volcán, *R.J. Seibert* 341a (holotype MO152735).

#### Type.

Based on *Convolvulus capillaceus* Kunth

#### Description.

Perennial herb with a subterranean elongate, bulb-like rootstock, similar to *Ipomoea plummerae*; stems to 30 cm, usually erect, glabrous. Leaves imbricate, subsessile, digitately divided into 5 segments, the segments 6–7 mm long, filiform, acute, apiculate; petioles 0–1.5 mm. Inflorescence of solitary axillary flowers; peduncles 1–2 mm; bracteoles 1–2 mm long, scarious, lanceolate to ovate; pedicels 2–6 mm; sepals unequal, outer 4–5 × 2 mm, ovate, acute, muricate or warty except on broad scarious margins; inner 5–6 × 4 mm, broadly ovate, acute to obtuse, muricate, scarious except for midrib, mostly smooth but warted near base; corolla 2.5–3 cm long, funnel-shaped, pink, glabrous, limb entire, 1.7 cm diam. Capsules subglobose, 3–5 mm, glabrous, the delicate style persistent; seeds 3 × 2.5 mm, brown, tomentellous.

#### Illustration.

Carranza (2007: 61).

#### Distribution.

Seasonally dry mountainous regions from the United States Southwest through Mexico and Central America to Peru, occurring mostly from 500–2000 m often at somewhat lower altitudes than *Ipomoea plummerae*. It is very sporadic in South America.

**PERU. Cusco**: Convención, Potrero, *C. Vargas* 1855 (CUZ).

**ECUADOR. Imbabura**: Near Carchi, *L.B. Holm-Nielsen & J.L. Jaramillo* 28931 (MO).

**COLOMBIA. Cauca**: *F.C. Lehmann* 602 (K), 7907 (F, K, US); *K. von Sneidern* 280 (S), 2539 (S, US). **Magdalena/Cesar**: Sierra Nevada de Santa Marta, *Purdie* s.n. (K); *L. Schlim* 760 (BR, K).

**VENEZUELA. Aragua**: Col. Tovar, *Moritz* 782 (BM), *A. Fendler* 952 (GH, K). **Carabobo**: *H. Pittier* 9025 (GH); **Dist. Fed.**: *J. Steyermark* 56982 (F). **Mérida**: *J. Steyermark* 57048 (F). **Miranda**: *G. & B. de Morillo* 3718 (MO); *R.W.G. Dennis* 2253 (K).

**PANAMA. Chiriquí**: Llanos del Volcán, *R.J. Seibert* 341 (GH, K, MO, NY): *P.H. Allen* 4847 (F, K, MO).

**COSTA RICA.** Puntarenas, Buenas Aires, *M. Valerio* 122 (K, MO).

**NICARAGUA.** Nuevo Segovia, *W.D. Stevens* 3308 (MO); ibid., Santa Maria de Los Pinos, *P.P. Moreno* 24527 (BM, MO).

**HONDURAS.***A. Molina* 7510 (F); Siguatepeque, *T.G. Yuncker et al.* 5712 (K).

**EL SALVADOR.** Chalchuapa, *Calderon* 6969 (US).

**GUATEMALA.***J. Steyermark* 48220 (F, NY): *G. Bernoulli* 331 (K).

**MEXICO. Aguascalientes**: *K.T. Hartweg* 94 (BM). **Baja California Sur**: Sierra San Francisquito, *T.S. Brandegee* s.n. (CAS, NY, US). **Coahuila**: *E. Palmer* 9100 (K). **Chiapas**: Tonalá, *E.W. Nelson* 2879 (US). **Chihuahua**: *C.G. Pringle* 1340 (F, K); Río Mayo, Guasaremos, *H.S. Gentry* 2334 (CAS, F, GH, K, S). **Durango**: *E. Palmer* 302 (BM, K); Mun. Santiago Papasquiaro, *P. Tenorio & C. Romero* 1034 (MO), 4179 (MEXU); *E.W. Nelson* 4640 (K, US). **Est. México & Dist. Fed**.: Tacubaya, *M. St. Pierre* 2595 (K, P); Tepotzotlán, *D.G. Saucedo* s.n. [7/8/1966] (F); Texcoco, *A. Ventura* 4252 (BM, NY); Temascaltepec, Mina de Agua, *G.B. Hinton* 1407 (K), ibid., Nanchititla, *G.B. Hinton* 6523 (BM, K), ibid., *G.B. Hinton* 8456 (K, NY, US). **Guanajuato**: San Felipe, *J. Rzedowski* 47296 (IEB). **Guerrero**: Manchón, Mina, *G.B. Hinton* 9211 (GH, K, NY, US). **Jalisco**: Guadalajara, *C.G. Pringle* 11048 (K). **Michoacán**: Morelia, *G. Arsène* 6701 (MO, US); Cerro del Águila, Morelia, *E. Sánchez et al.* 86 (K, MEXU); Pátzcuaro, Cerro Blanco, *E. Pérez* 4006 (IEB). **Morelos**: W of Cuernavaca, *J. Flores Crespo* 327 (ASU). **Nayarit**: fide McDonald (1995). **Oaxaca**: *D.H. Lorence et al.* 3543 (MO). **Puebla**: Cerro de Paxtle, *C.A. Purpus* 3368 (BM, CAS, F, MO, US). **Querétaro**: Colón, La Esperanza, *S. Zamudio* 8004 (IEB). **San Luis de Potosí**: *S.E. Verhoek-Williams et al.* 506 (MO); Cerro de San Miguelito, *J.A. Nova et al.* 417 (K). **Sinaloa**: San Ignacio, *J. G. Ortega* 494 (K). **Sonora**: Mesa Las Cabañas, *A.L. Reina-G et al.* 2009-1334 (ARIZ). **Veracruz**: Veracruz-Orizaba, *Müller* 1605 (K); *H. Galeotti* 1353 (BR, K). **Zacatecas**: *K.T. Hartweg* s.n. (K); Valparaíso, San Pedro de la Sierra, *P. Carillo-Reyes & F. Puig* 3241 (IEB).

**UNITED STATES. Arizona**: *J.G. Lemmon* 2836 (BM, K, US); Cochise Co., Chirocahua Mts., *J.C. Blumer* 1643 (ARIZ, F, K, NMC, RM, US). **New Mexico**: Catron Co., Gila Cliff Mont., *E. Bennet* 156 (ARIZ); ibid., *J. Kramer* 4 (RM). **Texas**: Trans-Pecos Mountains region fide Correll and Johnston (1970).

#### Note.

*Ipomoea capillacea* and *I. plummerae* are very close and often confused. Indeed molecular evidence appears to give little support for their distinction. Morphologically *I. capillacea* is distinguished by its erect habit and imbricate leaves with filiform leaflets. The sepals are slightly shorter reaching only 5 mm.

### 289. Ipomoea jujuyensis

Taxon classificationPlantaeSolanalesConvolvulaceae

O’Donell, Lilloa 14: 174. 1948. (O’Donell 1948a: 174)

#### Type.

ARGENTINA. Jujuy, Dept. Capital, Lagunas de Yala, *O’Donell* 4835 (holotype LIL 182934, isotype P).

#### Description.

Twining perennial to 6 m from a tuberous rootstock, stems pubescent to subhispid. Leaves petiolate, ovate, shortly acuminate, cordate with rounded auricles, thinly adpressed pubescent; petioles 2–10 cm, pubescent. Inflorescence of pedunculate axillary cymes with up to five flowers; peduncle 5–15 cm, pubescent, stout; bracteoles 2–3 mm long, broadly lanceolate, caducous; pedicels 1–2.5 cm, thickened upwards, stout, pubescent, often deflexed at maturity; sepals slightly unequal, rounded and emarginate, usually mucronulate, the margins scarious, outer 6–8 × 5–6 mm, elliptic, obtuse, thinly pubescent, inner 7–8 × 8–9 mm, suborbicular, glabrous; corolla 6.5–9 cm long, funnel–shaped from a short basal tube, violet, glabrous or minutely puberulent on the midpetaline bands, limb 4.5–6 cm diam., undulate. Capsules 14–16 × 8–10 mm, ovoid, rostrate, the apex c. 4 mm long; glabrous; seeds 7 × 5–6 mm, blackish, tomentellous.

#### Illustration.

Figure 11L; O’Donell (1959b: 169).

#### Distribution.

Scattered along the Andes from northern Argentina to Peru and southern Ecuador, mostly 1800 to 2500 m, but apparently absent from Bolivia.

**ARGENTINA. Catamarca**: Yacutula, *F. Schickendantz* 70 (CORD); Belén, *G.E. Barboza et al.* 604 (CORD, MA); ibid., 1959 (CTES); *H. & O. Brücher* s.n. [21/2/1949] (S). **Jujuy**: Laguna Yala, *O’Donell* 4871 (LIL, P), 5554 (LIL, P); ibid., *M.A. Negritto et al.* 295 (CORD, CTES); *T. Meyer* 16958 (LIL). **Salta**: Rosario de Lermo, *A.M. Ciadella* 354 (SI). **Tucumán**: Tafi, *L. Castillon* 355 (LIL); *S. Venturi* 2917 (US).

**PERU. Cusco**: Paruro, Mayhura, *C. Vargas* 855 (LIL).

**ECUADOR. Loja**: *C.W.T. Penland & R.H. Summers* 1134 (GH, US); *M. Rivet* 950 (P); Loja–Zamora road, *G. Harling & L. Andersson* 14075 (AAU, MO). **Pichincha**: *B. Sparre* 14627 (S); *F. de la Puente* 1299 (CIP).

#### Notes.

Molecular studies indicate this species is an isolated species in Clade B. It is somewhat arbitrarily placed near *Ipomoea dumetorum* with which it shares a strongly rostrate capsule, scarious-margined sepals and minutely tomentellous seeds. It is easily distinguished, however, by the perennial habit, pubescent leaves, larger corolla and the absence of dark spots on the sepals.

The record from Bolivia (Wood et al. 2015) was an error for *Ipomoea squamosa* and there is doubt about the correct identification of the plants from Ecuador and Peru.

•• Clade B2 is composed of species 290–338. Although this clade is well supported by all our sequence data, no obvious morphological feature characterises the clade.

• Species 290–311 form a clade within B2. Although there seems to be no character uniting this clade, there are obvious species clusters such as species 290–294.

### 290. Ipomoea purga

Taxon classificationPlantaeSolanalesConvolvulaceae

(Wender.) Hayne, Getreue Darstell. Gew. 12: 5. 1833. (Hayne 1833: 5)

Convolvulus purga Wender., Pharm. Central-Blatt 1: 457. 1830. (Wenderoth 1830: 457). Type. MEXICO. Veracruz, Chiconquiaco, Schiedes.n. (lectotype NY00318915, designated by McDonald 1987c: 55, isolectotypes BM, GH, K, P).
Exogonium purga (Wender.) Benth., Pl. Hartw. 46. 1840. (Bentham 1839–57: 46).
Batatas purga (Wender) Peterm., Pflanzenreich, ed. 1: 497, t. 132, fig. 750. 1838–1845. (Petermann 1838–1845: 497).
Ipomoea jalapa Nutt. in Coxe, Journ. Am. Med. Sci. 5: 305. 1829 [pub.1830]. (Coxe 1830: 305), nom. illeg. non Ipomoea jalapa (L.) Pursh (1814). Type. Plant from Xalapa [Veracruz], cultivated in the United States. (lectotype t. 1 (p. 306A) in Coxe (1830), designated here).
Ipomoea schiedeana Zucc., Flora 14 (2): 801. 1831. (Zuccarini 1831: 801). Type. MEXICO. Veracruz, Chiconquiaco, Schiedes.n. (BM, GH, K, M?.NY, P).
Ipomoea jalapa Schiede & Deppe ex G. Don, Gen. Hist. 4: 271. 1838. (Don 1838: 271), nom. suerfl. et illeg. non Ipomoea jalapa (L.) Pursh (1814). Based in part on I. purga (Wender.) Hayne and in part on I. schiedeana Zucc.
Convolvulus officinalis Pelletan, J. Chim. Méd. 10: 6. 1834. (Pelletan 1834: 6). Type. MEXICO. Veracruz, Orizaba, *Le Danois*s.n. (holotype P00607314).

#### Type.

Based on *Convolvulus purga* Wender.

#### Description.

Perennial twining or trailing herb to 7 m, roots tuberous, stems often dark-red pigmented, glabrous. Leaves petiolate, 4–12 × 3–8 cm, ovate, cordate to sagittate, the auricles rounded or acute, apex narrowly acuminate, mucronulate, both surfaces glabrous; petioles 2.5–6 cm. Flowers solitary or paired from the leaf axils; peduncles 4–8.5 cm long; bracteoles 2 mm long, lanceolate-deltoid; pedicels 10–20 mm, thickened upwards; sepals subequal, glabrous, ovate, acute, obtuse or emarginate and mucronulate, margins scarious, outer 3–8 × 3–4 mm, inner slightly larger, up to 10 × 7 mm; corolla hypocrateriform, 4–6 cm long, widened from the cylindrical base at about half way, glabrous, limb c. 5.5 cm diam., deep pink; stamens and style exserted up to 1 cm. Capsules conical, 7–9 mm long and wide, glabrous; seeds up to 4, 5–6 mm long, puberulent.

#### Illustration.

McDonald (1987c: 81).

#### Distribution.

A local Mexican endemic centred on where Hidalgo, Puebla and Veracruz meet. It grows in montane pine and oak forest around 2000 m.

**MEXICO. Hidalgo**: Trinidad Iron Works, *C. G. Pringle* 8889 (BM, F, K, MEXU, NY, S, US); Zacualtipan, *K.T. Hartweg* s.n. (K); *H. Puig* 3094 (P); Tenango de Doria, *O. Alcantara Ayala & E. Ortiz* 1183 (MEXU). **Puebla**: Texiutlán, *W. Orcutt* 4003 (F); El Mirador, Ocpaco, *J.L. Contreras* 9105 (MEXU). **Veracruz**: *E.K. Balls* 5475 (US); *R.V. Ortega* 1520 (F).

#### Notes.

Similar to *Ipomoea dumosa* with which it is often confused differing in the subequal sepals 6–10 mm long, the apex obtuse or emarginate, the inner sometimes mucronate, and the longer peduncles 4–8.5 cm in length so leaves not enveloping the base of the corolla.

The tuberous roots were much valued in the past as a “safe” purgative. Still sometimes cultivated (McDonald 1994: 100, Don 1838: 271).

### 291. Ipomoea dumosa

Taxon classificationPlantaeSolanalesConvolvulaceae

(Benth.) L.O. Williams, Fieldiana, Bot. 32: 190. 1970. (Williams 1970a: 190)

Exogonium dumosum Benth., Pl. Hartw. 46. 1840. (Bentham 1839–57: 46). Type. MEXICO. Hidalgo, San Cornelia, *K.T. Hartweg*s.n. (lectotype K000612761, designated by Williams (1970b: 190), isolectotypes K, LD).
Calonyction galeottii M. Martens, Bull. Acad. Roy. Sci. Bruxelles 12: 268. 1845. (Martens and Galeotti 1845: 268). Type. MEXICO. Veracruz, *H. Galeotti 1355* (holotype BR00006972615, isotypes BR, G, K, P).
Ipomoea purga auct.

#### Type.

Based on *Exogonium dumosum* Benth.

#### Description.

Climbing perennial herb to 5 m with fibrous roots, stems glabrous, relatively slender, wiry. Leaves usually very shortly petiolate, 4–10 × 2–6 cm, ovate, acuminate to an obtuse mucronate apex, base cordate with rounded auricles and narrow sinus, thin in texture, glabrous, abaxial veins prominent, usually glabrous, occasionally puberulent; petioles 2–6(–50) mm long, puberulent or glabrous. Inflorescence of very shortly pedunculate, 1–5-flowered axillary cymes, the base often enveloped by the leaves; peduncles 0.2–4 cm, often briefly fused to the petiole and penetrating the leaf sinus, shortly pilose or glabrous; bracteoles 1–2 mm, ovate, caducous; pedicels 3–15 mm, glabrous or thinly and very shortly pilose; sepals unequal, glabrous with white scarious margins, outer 3–5 × 3 mm, oblong-ovate, obtuse and mucronate, inner 8–12 mm, oblong-lanceolate, mucronate; corolla 5–7 cm long, glabrous, hypocrateriform with subcylindrical tube 4.5–6 cm long, slightly widening upwards, limb 3.5–4.5 cm diam., unlobed, deep reddish-purple to red, stamens exserted. Capsules 12–14 × 7–8 mm, conical, glabrous; seeds 4–5 × 4 mm, puberulent.

#### Illustration.

**Figure 142.**
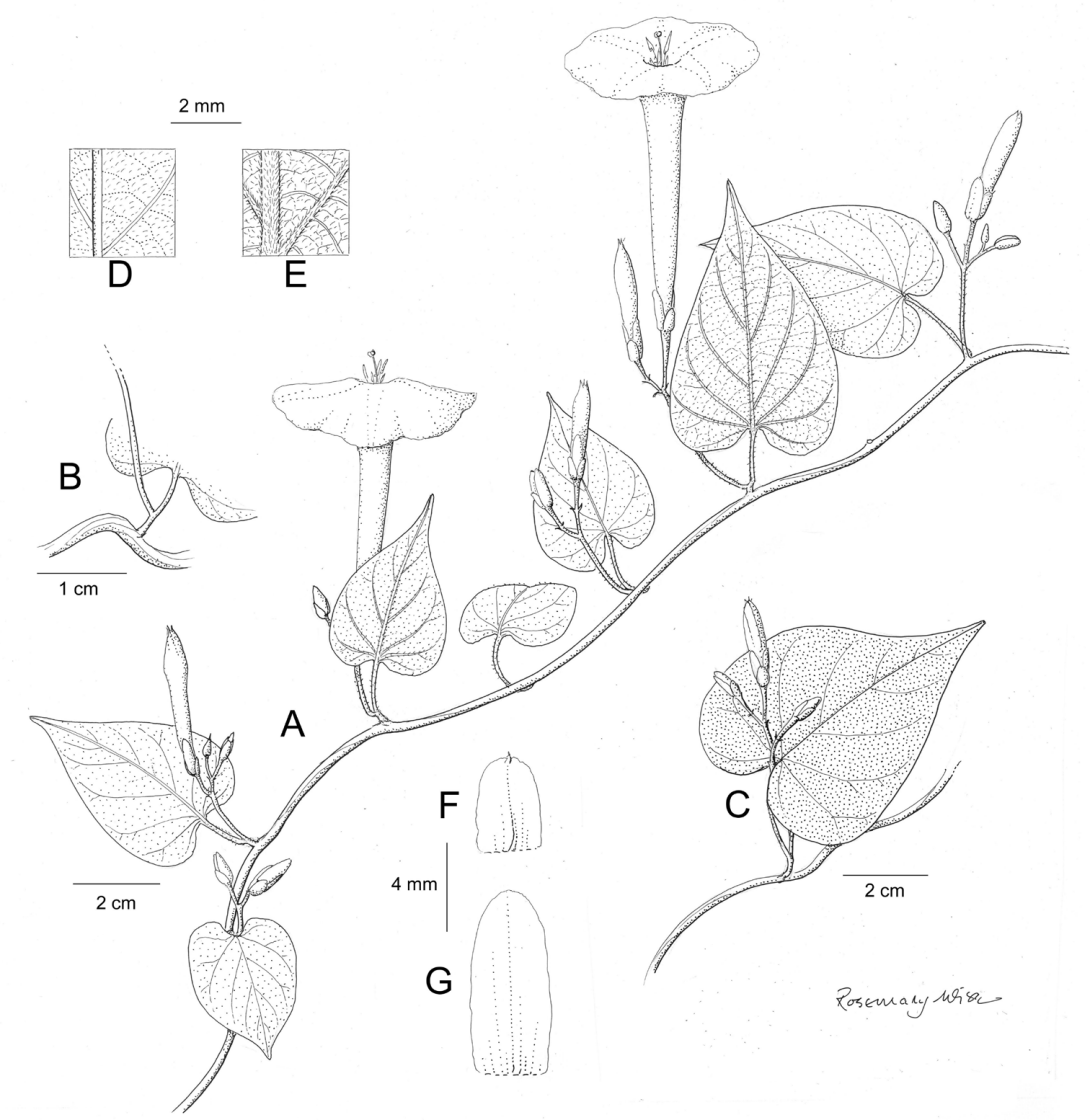
*Ipomoea dumosa*. **A** habit **B** habit showing fused petiole and peduncle **C** habit showing peduncle penetrating leaf sinus **D** adaxial leaf surface **E** abaxial leaf surface **F** outer sepal **G** inner sepal. Drawn by Rosemary Wise **A, C–G** from *Hinton* 9479; B from *Hinton* 11207.

McDonald (1994: 41); Figures 10E, 142.

#### Distribution.

Widely distributed from Panama through Central America north to central Mexico. It is found at altitudes below about 1300 m in various kinds of disturbed and natural woodland but often in rather moist areas of otherwise dry woodland. The two records from Brazil are anomalous but appear correctly named.

**BRAZIL. Goiás**: *A. St. Hilaire* 778 (P). **Paraná**: Sete Quedas/Guaíra, *Buttura* s.n. (MBM74804).

**PANAMA.** Chiriqui, *W.H. Lewis et al.* 729 (MO).

**COSTA RICA.** San José, El General, *A.F. Skutch* 2270 (K, NY, MO, US); Tucurrique, *A. Tonduz* 12854 (BM); *Wall* 31 (S); Puntarenas, Coto Brus, *M.M. Chavarria* 688 (K, MO).

**NICARAGUA.***W.D. Stevens et al.* 29321 (MO)

**HONDURAS.** Copán, *L.O. Williams et al.* 43009 (BM, F); *A. Molina & A.R. Molina* 24606 (F, MO, US).

**EL SALVADOR.***Hartman* 98 (S)

**GUATEMALA.** Esquintla, San Luis, *J. Donnell Smith* 2014 (K); *A. Molina & A.R. Molina* 25372 (F, MO); *Kellermam* 5140 (MEXU, US); Santa Rosa, *Heyde & Lux* 4353 (BM, K); *L. Rodríguez* 1439 (P).

**MEXICO. Chiapas**: *D.E. Breedlove & R.F. Thorne* 20949 (MO). **Colima**: foothills of Vulcan de Colima, *A.C. Sanders et al.* 10418 (MEXU). **Est. México & Dist. Fed.**: Temascaltepec, *G.B. Hinton* 479 (BM, GBH); ibid., *G.B. Hinton* 2220 (BM, K, NY US); ibid., *G.B. Hinton* 4810 (K); ibid., San Lucas, *G.B. Hinton* 8594 (K); ibid., *G.B. Hinton* 11207 (K). **Guerrero**: *G.B. Hinton* 9479 (K); Mina, *G.B. Hinton* 9637 (K, NY, US); Montes de Oca, *G.B. Hinton* 11770 (K), Zitacuaro, *G.B. Hinton* 13427 (K). **Jalisco**: *E. Palmer* 373 (BM, MO); Jalpa, *E.W. Nelson* 4022 (K, US). **Michoacán**: *L. Rowntree* 246 (ARIZ); Zitácuaro, El Tizate, *Y. Ramírez & V.W. Steinmann* 490 (ARIZ, IEB); Charo, *E. Carranza & I. Silva* 6780 (IEB). **Oaxaca**: Choapam, *Y. Mexia* 9173 (K, MO, S); San Juan Bautista Tuxtepec, *A. Flores* 1019 (IEB). **Puebla**: *Fröderström & Hultén* 870 (S); Hueytamalco, Las Margaritas, *G. Cornejo Tenorio* 2764 (IEB); ibid., *B. & G. Gómez* 374 (K, MEXU, MO). **Puebla**: Hueytamalco, *B. & G. Gómez* 374 (MO). **Querétaro**: Landa de Matamoros, *J. Rzedowski* 54119 (IEB). **San Luis Potosí**: Tamazunchale, *D.B. Dunn et al.* 17534 (MO). **Sonora**: San Pedro Nolasco Island, *C. Jurgensen* 553 (BM). **Veracruz**: Valle de Córdoba, *E. Bourgeau* 1730 (K, P); ibid., *E. Kerber* 40 (BM, K); Orizaba, *M. Botteri* 561 (BM, K); *C. Hernández et al*. 222 (F).

#### Notes.

*Ipomoea dumosa* is usually recognised easily by the short peduncle which is enclosed in the folded leaf combined with the hypocrateriform corolla and exserted stamens.

The two records from Brazil are anomalous but the specimens appear correctly named. There is no evidence that *Ipomoea dumosa* is cultivated and it is unlikely that the labels were wrongly attached, especially in the case of the collection from Sete Quedas. Unfortunately this site has been flooded as a result of the construction of the Itaipú Dam so this species is presumably extinct in this site.

*Ipomoea dumosa* has rather distinct pollen (Figure 10E), the spines being blunt and genmmiform as in species from the Calonyction Clade.

*Ipomoea dumosa* is the best known species in a complex of partially intergrading species. *Ipomoea seducta* is only distinguished by its funnel-shaped corolla and some specimens from Guerrero, Michoacán and Estado Mexico, are rather arbitrarily placed in one or other of these species. *Ipomoea tubulata* is only separated by the distinctly lobed corolla with short, ovate-deltoid lobes but some specimens from Michoacán are intermediate in character.

### 292. Ipomoea seducta

Taxon classificationPlantaeSolanalesConvolvulaceae

House Ann. New York Acad. Sci. 18: 241. 1908. (House 1908b: 241)

#### Type.

GUATEMALA. Altaverapaz, *H. von Tűrckheim7926* (holotype NY00547070, isotypes GH, K, MICH, US).

#### Description.

Perennial twining herb to 5 m, stems glabrous, somewhat wiry. Leaves petiolate, 3–11 × 2.5–9 cm, ovate with long acuminate apex, cordate, glabrous, frequently enclosing the inflorescence; petioles 0.3–5 cm, glabrous. Inflorescence of solitary (rarely in cymes of 2–3) axillary flowers; peduncles 0.3–5 cm, often penetrating the leaf sinus; bracteoles scale-like, c. 1 mm; pedicels 6–8 mm; sepals unequal, the outer 3–4 × 2–3 mm, ovate, acute, the inner 7–9 × 3–4 mm, elliptic; corolla 5–6 cm long, funnel-shaped, flaring from near the base, lilac-purple, glabrous, limb 4–5 cm diam. Capsules c. 12 × 10 mm, conical, rostrate; seeds 6–7 × 3–4 mm, puberulent and minutely ciliolate on margins.

#### Distribution.

Deciduous forest up to 2200 m from central Mexico south to Honduras.

**EL SALVADOR.** La Libertad, hacia Túneles, *A. Molina* 21447 (F).

**HONDURAS.** Comayagua, *C. Nelson* 7454 (MO).

**GUATEMALA.** Cobán. Alta Verapaz, *H. von Türckheim* 101 (K), Cubilquitz, 7926 (K); Cañon del Río Chixoy, *L.O. Williams et al*. 40563 (MO).

**MEXICO. Chiapas**: *E.W. Nelson* 3403 (US); *D.E. Breedlove* 10069 (F). **Colima**: Comala, Rancho El Jabali, *A.C. Sanders et al.* 10647 (K, MO); *L. Vásquez* 370 (MEXU). **Guerrero**: Galeana, Tecpán, *G.B. & J.C. Hinton* 10813 (GBH, K). **Jalisco**: *R. McVaugh* 26396 (MICH); San Sebastián, *Y. Mexia* 1643 (BM, US). **Michoacán**: Coalcomán, *G.B. Hinton et al.* 12700 (F, K, MO, US), ibid., 12332 (K); ibid., *E. Carranza & I. Silva* 6926 (IEB, MEXU). **Nayarit**: Tepic-Miramar, *S. Aguilar* 89 (MEXU); ibid., *E. Carranza et al.* 6124 (IEB, MEXU). **Oaxaca**: Santa María Chimalapa, *H. Hernández* 2121 (MO). **Sinaloa**: Concordia, *A.C. Sanders et al.* 4542 (UCR).

#### Note.

Identical to *Ipomoea dumosa* apart from the funnel-shaped corolla with included stamens. Some specimens, especially from Guerrero, Michoacán and Est. Mexico, are rather arbitrarily placed here or in *I. dumosa*.

### 293. Ipomoea tubulata

Taxon classificationPlantaeSolanalesConvolvulaceae

Sessé & Moçiño, Flora Mexicana 42 (Naturaleza (Mexico City) ser. 2, 2, append.: 42. 1893 (Sessé y Lacasta and Moçiño 1891–97: 39)

Quamoclit tubulosa M. Martens & Galeotti, Bull. Acad. Roy. Sci. Bruxelles 12: 270. 1845. (Martens and Galeotti 1845: 270). Type. MEXICO. Michoacán, Uruapan, *H. Galeotti* 1393 (holotype BR, isotypes BR, G, P, W).
Ipomoea tubulosa (M. Martens & Galeotti) Hemsl., Biol. Cent.-Amer., Bot. 2: 395. 1882. (Hemsley 1882: 395), non Ipomoea tubulosa Willd. ex Roem. & Schult. (1819).
Exogonium uhdeanum Fenzl. ex Hallier f., Bot. Jahrb. Syst. 16: 559. 1894 [pub.1893]. (Hallier 1893a: 559), nom. nud.
Ipomoea uhdeana (Hallier f.) D.F. Austin, Ann. Missouri Bot. Gard. 64: 332. 1977 [pub. 1978]. (Austin 1978a: 332), basionym illeg.
Ipomoea urbinei House, Muhlenbergia 3(3): 41. 1907. (House 1907a: 41). Type. MEXICO. Jalisco, Vulcan de Colima, *Barcéna* 214 (holotype MEXU†, lectotype, icon of *Barcéna* 214 in House 1907a: t.2, f.2, designated by McDonald 1987c: 51).
Exogonium woronovii Standl., Publ. Field Mus. Nat. Hist., Bot. Ser. 11: 171. 1932. (Standley 1932: 171). Type. MEXICO. Michoacán, Rodillo del Diablo near Uruapan, *G. Woronow* 2906 (holotype F641479).
Ipomoea shinnersii var. woronovii (Standl.) D.F. Austin, Ann. Missouri Bot. Gard. 64: 337. 1977 [pub. 1978]. (Austin 1978a: 337).
Ipomoea woronovii (Standl.) D.F. Austin, Taxon 32: 626. 1983. (Austin 1983: 626).

#### Type.

MEXICO. [Michoacán], Uruapan, *Sessé & Moçiño* 463 (lectotype MA603821, designated here).

#### Description.

Perennial herb to 2 m, stems glabrous or pubescent, often reddish. Leaves petiolate, 3.5–7 × 3–5.5 cm, ovate, finely acuminate and mucronate, base cordate with rounded auricles, sometimes concealing petiole, adaxially glabrous to thinly pubescent, abaxially paler, minutely pubescent; petioles 1–3 cm, glabrous or pubescent. Inflorescence of usually 1–3-flowered, pedunculate axillary cymes; peduncles 1.4–3.5 cm, glabrous or puberulent; bracteoles 4–5 × 2 mm, oblong-elliptic; secondary peduncles, if present, much shorter than pedicels; pedicels 5–9 mm, glabrous or puberulent; sepals unequal, glabrous with scarious margins, outer 3–4 × 2 mm, ovate-deltoid, acute to obtuse, minutely mucronate, inner 7–9 × 3–4 mm, obtuse to emarginate and mucronate; corolla 3–4.5 cm long, hypocrateriform, red, glabrous, limb c. 1.5 cm diam., deeply lobed, the lobes deltoid, acute, 5–7 mm long, stamens weakly exserted. Capsules 11–12 mm, conical; seeds 6–10 mm, dark brown, puberulent.

#### Illustration.

McDonald (1987c: 80).

#### Distribution.

Mexico. Moist hill forest around 1600–2000 m, many records are from around Uruapan.

**MEXICO.** Sine loc., *Schiede* s.n. (K). **Jalisco**: Tuxpan, *J. Villa & J. Chávez* 572 (IEB, MICH). **Michoacán**: Coalcomán, Zarzamora, *G.B. Hinton* 12254 (K, NY, TEX, US); 2.5 km N. de Zirimicuaro, Mun. Ziracuaretiro, *S. Zamudio* 11263 (FTG, IEB); Tepelcatepec to Coalcomán, *V.W. Steinmann et al*. 5603 (ARIZ); Puerto de Las Cruces, *J. C. Soto Nuñez* 10987 (MEXU).

#### Typification.

Although McVaugh (2000: 200) indicated that *Ipomoea tubulata* Sessé & Moçiño was the oldest available name for this species, it has been ignored until now. We have designated MA603821 as lectotype as it is a good specimen with the original name annotated on the sheet.

#### Note.

A little-known species somewhat similar to *Ipomoea dumosa* in habit, corolla shape and colour. It differs in the relatively long pedicels and the lobed corolla with short triangular lobes, a character which also serves to separate it from *I. electrina*. The stamens are weakly exserted and the cymes 1–3-flowered.

### 294. Ipomoea electrina

Taxon classificationPlantaeSolanalesConvolvulaceae

D.F. Austin & J.A. McDonald, Novon 12: 29. 2002. (Austin and McDonald 2002: 29)

Exogonium luteum House, Bull. Torrey Bot. Club 35: 103. 1908. (House 1908a: 103), non Ipomoea lutea Hemsl. (1879). Type. MEXICO. Oaxaca, Cuesta de Chiquihuetlan, *C. Conzatti & J. González* 668 (holotype GH00054448, isotype NY).
Ipomoea woronovii var. lutea (House) D.F.Austin, Taxon 32: 626. 1983. (Austin 1983: 626).
Ipomoea shinnersii D.F. Austin, Ann. Missouri Bot. Gard. 64: 337. 1977 [pub. 1978]. (Austin 1978a: 337). Type. Based on Exogonium luteum House, nom. illeg. “woronowii” should have been used.
Ipomoea crocea McPherson ex Breedlove, Listados Floríst. México 4: 75. 1986. (Breedlove 1986: 75), nom. nud.

#### Type.

Based on *Exogonium luteum* House

#### Description.

Perennial herb to 3 m, stems woody below, pubescent. Leaves petiolate, 4–10 × 1.5–7 cm, ovate, finely acuminate, adaxially pubescent, abaxially tomentellous; petioles 1.5–3.5 cm. Inflorescence of axillary cymes of 3–18 flowers; peduncles 1.5–5 cm, pubescent; bracteoles linear-lanceolate, 5–10 × 1 mm, somewhat persistent; secondary peduncles 1–1.5 cm; pedicels 1.5–3 cm, pubescent ; sepals unequal, coriaceous, often verrucose basally, glabrous or pubescent, outer 4–6 × 3–4 mm, ovate-deltoid, acute, inner 6.5–9.5 × 4–5 mm, oblong-elliptic, obtuse to rounded, scarious marginally; corolla 5–6.5 cm long, hypocrateriform, yellow or orange, glabrous except apically, the cylindrical tube 4–6 mm wide, the limb deeply lobed, the lobes linear-oblong 15–23 × 2 mm, being more deeply lobed and spreading when mature, the apex comose; stamens exserted 5–10 mm. Capsules conical; seeds dark brown, long-pubescent.

#### Illustration.

Figures 8D, 143.

**Figure 143.**
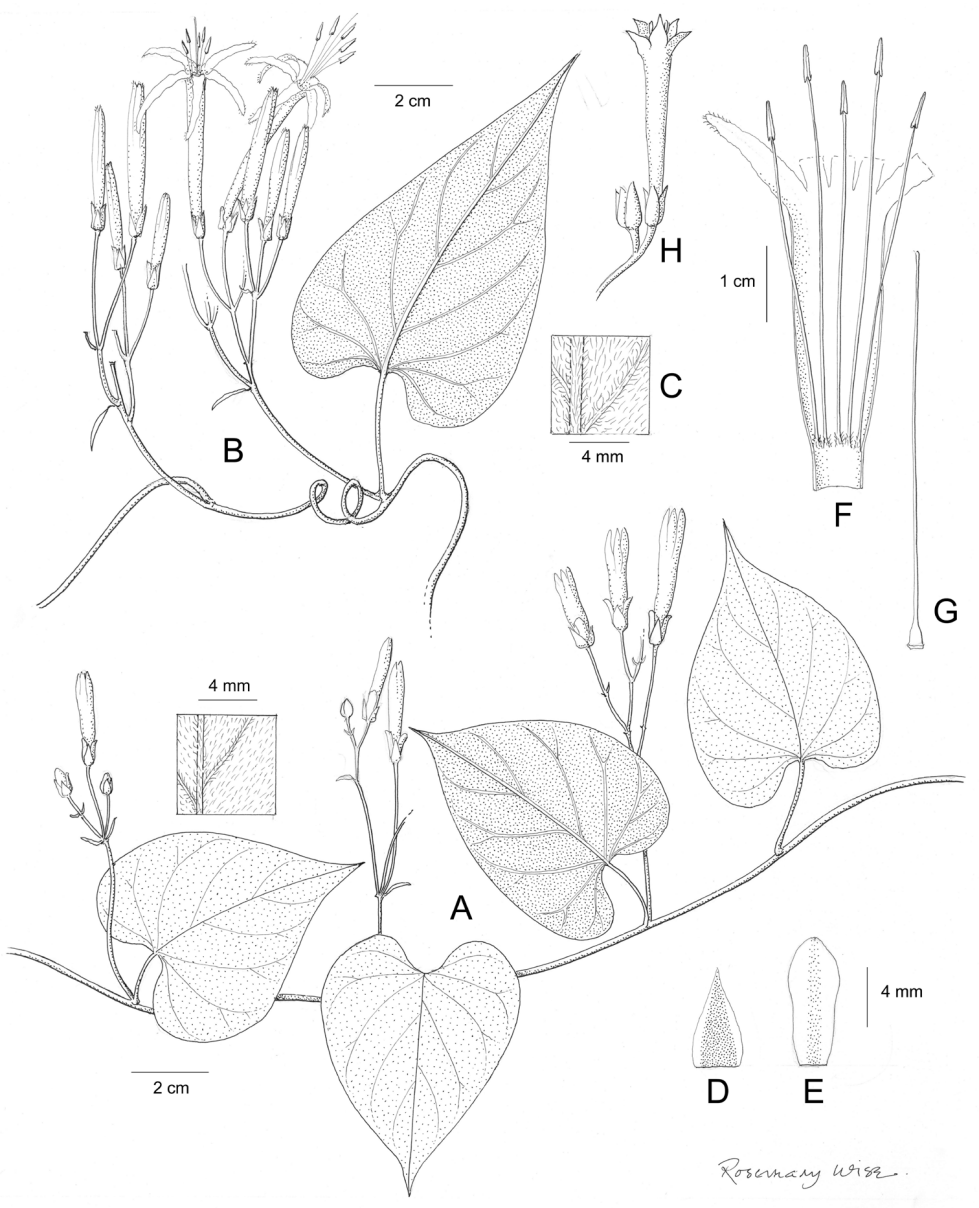
*Ipomoea electrina*. **A** habit **B** habit **C** abaxial leaf surface **D** outer sepal **E** inner sepal **F** corolla opened out to show stamens **G** ovary and style **H** flower showing less divided limb. Drawn by Rosemary Wise **A–G** from *Breedlove* 27626; **H** from *Purpus* 9189.

#### Distribution.

Endemic to southern Mexico, where it grows in dry deciduous oak forest between 700 and 2100 m.

**MEXICO. Chiapas**: *D.E. Breedlove* 27626 (MICH, MO). **Oaxaca**: Hac. Monserrate, *C.A. Purpus* 9189 (MO, US); Nejapa de Medero, *E. Martínez Luis* 332 (IEB); San Miguel Suchixtepec, *P. Tenorio et al.* 18410 (MEXU); Cerro Marimba, Tehuantepec, *C. Martínez* 1035 (MEXU).

#### Note.

In Flora Mesoamericana, Austin et al. (2012) treated *Exogonium luteum* as a synonym of *Ipomoea urbinei* without reference to *I. electrina* but this appears to have been an error. The two species are somewhat similar and have been confused, *Ipomoea electrina* sometimes being treated as a variety of *I. urbinei* (Austin 1983). *Ipomoea electrina* is distinguished by the orange or yellow corolla with long linear-oblong, spreading lobes which are comose at the apex.

### 295. Ipomoea bernoulliana

Taxon classificationPlantaeSolanalesConvolvulaceae

Peter, Nat. Pflanzenfam. 4 (3a): 30. 1897 [1891]. (Peter 1891: 30

Rivea bernoulliana (Peter) Hallier f., Bot. Jahrb. Syst. 18: 158. 1894 [pub.1893]. (Hallier 1893b: 158).
Ipomoea santae-rosae Standl. & Steyerm., Publ. Field Mus. Nat. Hist., Bot. Ser. 23(2): 81. 1944. Type. GUATEMALA. Santa Rosa, vic. Chiquimulilla, *P.C. Standley* 79287 (holotype F0054894).

#### Type.

GUATEMALA. *Bernoulli & Cario* 1902 (lectotype GOET002541, designated by Staples and Austin 2010: 467).

#### Description.

Slender liana to c. 5 m, stems woody, pubescent when young, glabrescent. Leaves petiolate, 4–10 × 3–7 cm, ovate, cordate with rounded auricles, apex finely acuminate and mucronate, margin undulate to slightly denticulate, adaxially glabrous, abaxially pubescent to subglabrous with hairs only at intersection with petiole; petioles 1.5–7 cm, glabrous. Inflorescence of solitary, long-pedicellate, axillary, flowers, often arising on axillary branchlets; peduncles 2–5 mm, pubescent or glabrous; bracteoles 2 mm, deltoid, scarious, caducous; pedicels 2.5–3.3 cm, relatively slender, glabrous; sepals unequal, acute or ±oblong, obtuse mucronate, chartaceous with narrow, scarious margins and prominent longitudinal veining, glabrous, outer 18–21 × 4 mm, strictly oblong, inner 22–30 × 6–7 mm, oblong-oblanceolate; corolla 6–8 cm long, pinkish-purple, glabrous, funnel-shaped, limb c. 4 cm wide, shallowly lobed. Capsules 8–12 mm, globose, glabrous; seeds 7–10 mm, puberulent.

#### Illustration.

Figures 3B, 144.

**Figure 144.**
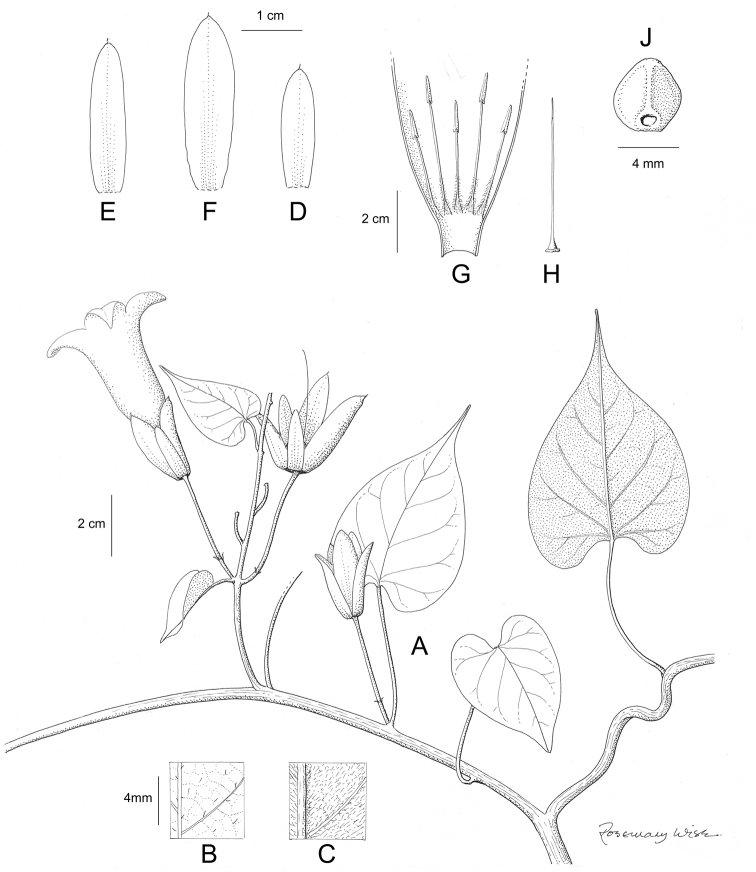
*Ipomoea bernoulliana***A** habit **B** adaxial leaf surface **C** abaxial leaf surface **D** outer sepal **E** middle sepal **F** inner sepal **G** corolla opened out to show stamens **H** ovary and style **J** seed. Drawn by Rosemary Wise **A–H** from *Standley* 27496; **J** from *Nelson* 3925.

#### Distribution.

An infrequently collected species of Central America growing in disturbed forest, mostly at altitudes below 1000 m.

**COSTA RICA.** San José, Mora, Ciudad Colón, *M.H. Grayum & N. Zamora* 9667 (MO); ibid., El Rodeo, *A. Cascante* 1381 (CR, K).

**NICARAGUA.** Estelí, Condega, *P.P. Moreno* 23480 (MO); Madriz, Las Sabanas, *W. D. Stevens et al.* 26942 (HULE, MO).

**HONDURAS.** Morazán, San Antonio de Oriente, *P.C. Standley* 27496 (BM, F); ibid., Tegucigalpa, *C. Nelson* 3925 (BM); San Joséde Comayagua *A. Molina et al.* 31459 (MO).

**EL SALVADOR.** Usulután, Laguna de Alegría, *D. Williams* 145 (MO); La Libertad, *A. K. Munro et al.* 3737 (BM, MO).

**GUATEMALA.** Sacatepéquez, Alotenango, *J.J. Mont & J.M. Vargas* 2725 (MO, NY).

**MEXICO. Chiapas**: Berriozábal, *D. Breedlove* 23051 (MO).

#### Note.

Very distinct because of the finely acuminate leaves, short peduncles combined with long pedicels, solitary flowers and long oblong, chartaceous, veined sepals.

### 296. Ipomoea jicama

Taxon classificationPlantaeSolanalesConvolvulaceae

Brandegee, Proc. Calif. Acad. Sci., ser. 2, 2: 188. 1889. (Brandegee 1889: 188)

Ipomoea odorata Eastw., Leafl. W. Bot. 3: 257. 1943. (Eastwood 1943: 257). Type. MEXICO. Baja California Sur, 5 miles N. of Comondu, *B.J. Hammerly* 172 (holotype CAS0003018, isotype CAS).

#### Type.

MEXICO. Baja California Sur, Magdalena Island, *T.S. Brandegee* s.n. 1889 (holotype UC105236; isotype US, fragment GH).

#### Description.

Perennial twining herb with tuberous roots to 2 m, stem glabrous. Leaves petiolate, 4–5.5 × 2–3 cm, broadly ovate, cordate, acute, margin weakly to strongly sinuate-dentate with broad irregular teeth, glabrous; petioles 1–3.5 cm. Flowers solitary or in few-flowered axillary cymes; peduncles 1–1.5 cm; bracteoles c. 1 mm, scale-like, caducous; pedicels 22–35 mm, thickened upwards; sepals unequal, glabrous, outer 10–12 mm, lanceolate, acute, inner 16–22 mm, narrowly ovate, acute and apiculate; corolla 5–6 cm long, funnel-shaped above a basal tube 1–1.5 cm long, glabrous, pale pink with a white throat, limb 4–4 cm diam., weakly lobed; stamens included. Capsules ovoid, 8–9 × 6 mm, glabrous; seeds up to 4, 5–7 mm long, shortly puberulent.

#### Illustration.

McDonald (1987c: 87).

#### Distribution.

Endemic to the southern part of the Baja California peninsula, where it grows on rocky slopes in dry deciduous forest around 500–600 m.

**MEXICO. Baja California Sur**: Sierra de la Giganta, Valle de Arroyo Hondo, *A. Carter* 5007 (BM), 5620 (BM, MICH, MO, UC); sine data, *M.L. Diguet* (P).

#### Note.

Somewhat similar to *Ipomoea tastensis* but the corolla much smaller and the stamens included.

### 297. Ipomoea tastensis

Taxon classificationPlantaeSolanalesConvolvulaceae

Brandegee, Zoë 5: 169. 1903. (Brandegee 1903–5: 169)

Calonyction tastense (Brandegee) House, Bull. Torrey Bot. Club 33: 318. 1906. (House 1906: 318).

#### Type.

MEXICO. Baja California Sur, Sierra El Taste, *T.S. Brandegee* s.n. [11/1902] (lectotype UC105180, designated by McDonald (1987c: 70).

#### Description.

Liana to 10 m, stems woody, glabrous, twining; rootstock tuberous. Leaves petiolate, 4–10 × 3–7 cm, ovate, long-acuminate, cordate to sagittate, the auricles with deltoid teeth, margin usually with several large teeth, glabrous; petioles 2–5.5 cm, slender. Flowers solitary, axillary; peduncles 1–3 cm; bracteoles caducous, not seen; pedicels 20–45 mm, thickened upwards; sepals unequal, lanceolate, acuminate, glabrous but basally muricate, outer 16–30 × 3–5 mm, inner 26–37(–50) mm; corolla 9–12 cm long, white, glabrous, subhypocrateriform, the basal tube long, c. 6 cm in length, limb 5–8 cm diam., lobes mucronate; stamens inserted high in tube and shortly exserted. Capsules subglobose, 1.5–2 × 1.5 cm; seeds 9–12 mm long, puberulent.

#### Illustration.

McDonald (1987c: 87).

#### Distribution.

Endemic to the southern part of the Baja California peninsular, where it grows in low deciduous forest at around 400 m.

**MEXICO. Baja California Sur**: one mile W of San Antonio, *B.J. Hammerly* 416 (CAS, US); Sierra San Francisquito, *T.S. Brandegee* Oct 1 1899 (US); El Palmiar Canyon, *R.M. Turner & C.H. Lowe* 59-138 (ARIZ); 3 km al N. del Poblado La Huerta, *M. Domínguez-L*. 3526 (ARIZ, HCIB).

#### Note.

The stamens are reported to be exserted but this is only visible in one specimen.

### 298. Ipomoea aristolochiifolia

Taxon classificationPlantaeSolanalesConvolvulaceae

G. Don, Gen. Hist. 4: 277. 1838. (Don 1838: 277)

Convolvulus aristolochiifolius Kunth, Nov. Gen. Sp. 3: 102. 1818 [pub.1819]. (Kunth 1819: 102), nom. illeg., non Convolvulus aristolochiifolius Mill. (1768). Type. VENEZUELA. Humboldt & Bonpland (holotype P 00670751).
Ipomoea oocarpa Benth., Bot. Voy. Sulphur 136. 1844 [pub. 1845]. (Bentham 1845: 136). Type. ECUADOR. Guayaquil, Sinclairs.n. (holotype K000612732).
Ipomoea peckoltii Meisn. in Martius et al., Fl. Brasil. 7: 269. 1869. (Meisner 1869: 268). Type. BRAZIL. Rio de Janeiro, *T. Peckolt* 234 (lectotype BR0000006973520, designated by McDonald 1994: 82).
Ipomoea tuerckheimii Vatke ex Donn.-Sm., Bot. Gaz. 40: 8. 1905. (Donnell Smith 1905: 8). Type. GUATEMALA. Alta Verapaz, *H. von Tuerckheim* 386 (holotype US00111480, isotypes BM, K, US, GH, P, PH).
Ipomoea peninsularis Brandegee, Zoë 5: 168. 1903. (Brandegee 1903–5: 168). Type. MEXICO. Baja California Sur, Cape Region, *T.S. Brandegee*s.n. (isotype UC105173).
Ipomoea austin-smithii Standl., Publ. Field Mus. Nat. Hist., Bot. Ser.18: 1566. 1938. (Standley 1938: 1566). Type. COSTA RICA. San Ramón, *A.M. Brenes* 16899 (holotype F0054827).
Ipomoea concinna House, Muhlenbergia 3: 42, 1907. (House 1907a: 42). Type. MEXICO. Jalisco, *Bárcena* 553 (holotype MEXU, n.v.).
Ipomoea cordata L.B. Smith & B.G. Schub., Contr. Gray Herb. 77: 31. 1939 (Smith and Schubert1939: 31). Type. MEXICO. Guerrero, G.B. Hinton 6984 (holotype GH00054494, isotypes K, MO, US).
Ipomoea viscosa Wiggins, Contr. Dudley Herb. 4: 21. 1950 (Wiggins 1950: 21). Type. MEXICO. Sonora, I.L. Wiggins 7505 (holotype DS, now CAS0003021, isotype US).
Ipomoea tweediei auct., non Hook., Bot. Mag. 69, t. 3978. 1842. (W.J. Hooker 1842).

#### Type.

Based *Convolvulus aristolochiifolius* Kunth

#### Description.

Slender twining annual herb, stems shortly pilose or glabrous. Leaves petiolate, 1.5–6 × 1–3.5 cm, ovate-deltoid, narrowed to an acuminate and mucronate apex, base very narrowly cordate often with overlapping rounded auricles, margin often with a few teeth, ciliolate, adaxially glabrous, abaxially paler, the veins puberulent; petioles 0.5–5.5 cm, glabrous to sparsely pilose. Inflorescence of 1–3(–6)-flowered, axillary, pedunculate cymes; peduncle 2.5–6 cm, puberulent, very slender, often curved, arising through the sinus of the leaf base; bracteoles 1–1.5 mm, triangular-lanceolate; pedicels mostly 5–10 mm, very slender, glabrous; calyx lanceolate in outline; sepals subequal, often warted on exterior near base but otherwise glabrous, 3–5 × 1.5–2 mm, oblong-lanceolate, obtuse, mucronate, dark green with pale scarious margin; corolla 1.5–2.5 cm long, campanulate, tube white, limb blue (drying pink), very shallowly lobed, 1.2–1.8 cm diam. Capsules glabrous, ovoid, the style often persistent as a rostrate tip; seeds puberulent.

#### Illustration.

**Figure 145.**
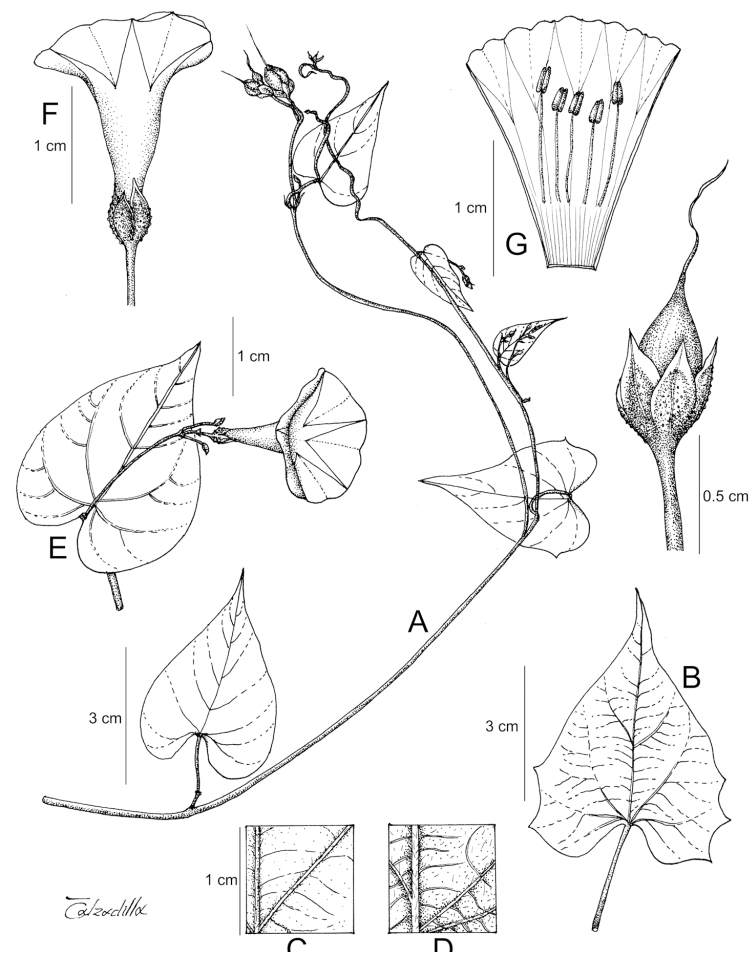
*Ipomoea aristolochiifolia*. **A** habit **B** leaf **C** adaxial leaf surface **D** abaxial leaf surface **E** inflorescence showing position of peduncle **F** calyx and corolla **G** corolla opened up to show stamens **H** calyx and capsule. Drawn by Eliana Calzadilla **A, C–H** from *Wood et al.* 27680; **B** from *Wood et al.* 27651.

Figures 2C, 145; O’Donell (1959b: 113).

#### Distribution.

Widely distributed through the Americas up to 2300 m, principally in the mountains of Central America (especially Nicaragua and Costa Rica) and the Andes but absent from both the very wet and the drier regions and never very abundant. It is a plant of shrubberies such as coffee plantations and disturbed scrubby places.

**ARGENTINA. Jujuy**: *A. Krapovickas et al.* 47387 (CTES, MO). **Salta**: Antillas, Cerro Negro, *S. Venturi* 10403 (BM, LIL, MO); Cerrillos, *L.J. Novara* 7704 (G, S). **Tucumán**: *S. Venturi* 315 (LIL, SI, US).

**BRAZIL. Minas Gerais**: *Y. Mexia* 4624 (BM, MO, NY, S). **Paraíba**: *J. Vasconcellos* 371 (RB). **Paraná**: *Y.S. Kuniyoshi & A.C. Svolenski* (MBM). **Rio de Janeiro**: *O.C. Góes* 630 (RB). **São Paulo**: *K. Mizoguchi* 1548 (NY). According Flora do Brasil 2020 under construction it is more common with records additionally from Santa Catarina, Mato Grosso do Sul, Distrito Federal, Bahia, Alagoas and Pernambuco.

**BOLIVIA. Chuquisaca**: Boeto, below Nuevo Mundo, *J.R.I. Wood et al.* 27660 (K, LPB, USZ); Luis Calvo, Serrania de Iñao, *J.A. Peñaranda & J. Tudela* 924 (MO, OXF); Tomina, Thiumayo, *J.R.I. Wood et al.* 27651 (OXF, K, LPB, USZ). **La Paz**: Muñecas, Río Charazani, *A. Fuentes & R. Cuevas* 7969 (BOLV, LPB, MO); Inquisivi, couth of Licoma, *J.R.I. Wood et al.* 29178 (LPB, USZ); Murillo, Zongo Valley, *J. Solomon* 13130 (FTG, LPB, MO); Nor Yungas, Coroico, *O. Buchtien* 3879 (E, NY, US). **Potosí**: Charcas, Torotoro, *J.R.I. Wood et al.* 21968 (K, LPB). **Santa Cruz**: Florida, Achira Camping, *M. Nee et al.* 49024 (NY); Vallegrande, Piraimiri, *J.R.I. Wood et al.* 21764 (K, LPB, USZ). **Tarija**: O’Connor, Chuquiaca, *K. Fiebrig* 2753 (BM, E, GH, K, P, S, US).

**PERU. Amazonas**: Chachapoyas, *A. Mathews* s.n. (BM). **Ancash**: Chiquian, *K. Young & M. Eisenberg* (MO). **Cajamarca**: *Llatas Quiroz* 2916 (F). **Cusco**: *G. Calatayud et al.* 2283 (MO). **Pasco**: Oxapampa, *D.N. Smith* 4129 (MO, USM). **Piura**: *E. Laure* 5492 (P). **Tumbes**: Zarumilla, *C. Díaz et al.* 4834 (MO, USM).

**ECUADOR. Guayas**: *A.S. Hitchcock* 20027 (F, NY, US). **Loja**: *G. Harling & L. Andersson* 13587 (MO). **Pichincha**: *R. Benoist* 2157bis (P). **Tungurahua**: *E. Asplund* 7644 (S).

**COLOMBIA. Cauca**: *K. von Sneidern* 24 (S). **Cesar/Magdalena**: “Ocaña” (Sierra Nevada de Santa Marta?), *L. Schlim* 256 (BM, P). **Cundinamarca**: Pacho, *L. Rosero* 382 (COL). **Norte de Santander**: *J. Cuatrecasas* 13451 (COL).

**VENEZUELA. Dist. Fed.**: *Funck* 175 (C, P); *L. Aristeguieta* 7771 (VEN). **Mérida**: *Moritz* 1289 (BM). **Táchira**: Saisayal, Río Negro valley, *L. Bernardi* 11012 (G). Also Lara, Miranda, and Yaracuy fide Austin (1982b).

**PANAMA.** Los Santos, Tonosi, *J.A. Duke* 12483 (MO).

**COSTA RICA.** El General, *A.F. Skutch* 3823 (K, S); Alajuela, San Ramón, *Khan et al.* 715 (BM); *W.D. Stevens & O.M. Montiel* 26719 (BM, MO); Puntarenas, Coto Brus, *M.M. Chavarría* 700 (K, MO).

**NICARAGUA.** Estelí, *L. Williams & A. Molina* 42472 (BM, F); Cerro El Coyolito, *P. Moreno* 25266 (BM) ; *I. Coronado et al*. 471 (P).

**HONDURAS.***S. Lagos-Witte et al.* 54 (MO) fide Tropicos.

**EL SALVADOR.** Morazán, Montes deb Cacaguatique, *J.M. Tucker* 670 (K, UC).

**BELIZE.***C.M. Brown* 14 (E); Chiquibul Forest Reserve, *C. Whitefoord* 10029 (BM).

**GUATEMALA.** Chiquimula, *A. Molina & A.R. Molina* 25390 (BM, DUKE, MO).

**MEXICO. Baja California Sur**: Type of *Ipomoea peninsularis*. **Chiapas**: Berriozábal, *D.E. Breedlove* 20404 (MO). **Est. México & Dist. Fed.**: Temascaltepec, *G.B. Hinton* 5173 (K), 8555 (K, MO); Tejupilco, *G.B. Hinton* 8555 (K, MO, NY). **Guerrero**: Mina, El Mono, *G.B. Hinton* 9675 (GBH, K, MO). **Michoacán**: Coalcomán, *G.B. Hinton* 12258 (GBH, K, MO). **Nayarit**: La Bahada, *E. Lehto* 24226 (ASU). **Querétaro**: Tanchanaquito, *E. Carranza* 4294 (IEB). **Sinaloa**: Sierra Surotato, *H.S. Gentry* 6477 (MO). **Sonora**: Agua Prieta, Rancho La Calera *A.L. Reina-G & T.R. Van Devender* 2006-705 (MO, NMC, USON). **Veracruz**: Valle de Córdoba, *E. Bourgeau* 1733 (K, P).

**UNITED STATES. Texas**: Cameron Co., *W.R. Carr* 14104 (TEX) – not seen.

#### Note.

Readily recognised by the delicate habit, small blue flowers, warted sepals with white margins and, particularly, by the peduncle which passes through the sinus of the leaf base. It is commonly confused with *Ipomoea dumetorum* but in that species the sepals have dark blotches and the peduncle does not pass through the sinus at the base of the leaf.

### 299. Ipomoea odontophylla

Taxon classificationPlantaeSolanalesConvolvulaceae

J.R.I. Wood & Scotland, Kew Bull. 70 (31): 108. 2015. (Wood et al. 2015: 108)

#### Type.

BOLIVIA. Santa Cruz, Prov. Florida, bajando c. 3 km de La Yunga de Mairana, hacia el puesto de los guardeparques, *J.R.I. Wood, M. Mendoza & C. Antezana* 21431 (holotype USZ, isotypes K, LPB).

#### Description.

Twining perennial reaching 5 m in height, stems glabrous, pale brown. Leaves petiolate, 6–13 × 4–10 cm, ovate, deeply cordate with rounded auricles, apex acute to shortly acuminate, mucronate, margin denticulate with acute teeth, adaxially pubescent on the veins with scattered hairs on the intercostal areas, abaxially pubescent, veins prominent; petioles 3–8 cm, sparsely pubescent but the widened base strongly pubescent. Inflorescence of 1–5-flowered, pedunculate axillary cymes; peduncles 2.5–6 cm long, glabrous; bracteoles 1–2 × 0.5 mm, very narrowly lanceolate; pedicels 10–25 mm, notably thickened upwards and differing somewhat in texture from the peduncles, glabrous or with a few hairs at base of calyx; sepals subequal, outer 7 × 3–4 mm, lanceolate to ovate, acute or subacute, glabrous, margin scarious, inner sepals slightly larger, 8 × 4–5 mm, ovate to suborbicular, rounded, scarious except near base; corolla 4–5.5 cm long, funnel-shaped, glabrous, tube white, limb blue, drying pink, c. 3.5 cm diam., shallowly lobed; stamens included. Capsules 16 × 13 mm, ovoid, glabrous, rostrate, the mucro c. 3 mm long; seeds 9 × 4 mm, oblong in outline, brown, glabrous.

#### Illustration.

Figure 146.

**Figure 146.**
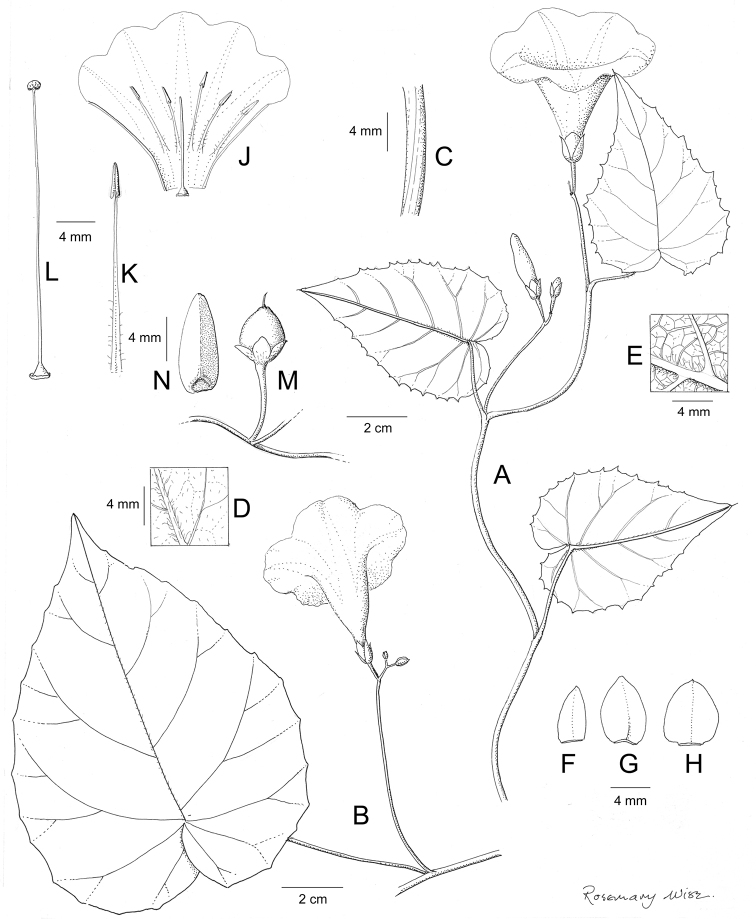
*Ipomoea odontophylla*. **A** habit **B** flowering shoot **C** stem **D** adaxial leaf surface **E** abaxial leaf surface **F** outer sepal **G** middle sepal **H** inner sepal **J** corolla opened out to show stamens **K** stamen **L** ovary and style **M** capsule **N** seed. Drawn by Rosemary Wise **A–L** from *Wood et al.* 21431; **M, N** from *Nee et al.* 52029.

#### Distribution.

A narrowly endemic species known only from the Yunga de Mairana in the Parque Nacional Amboró near Santa Cruz, Bolivia, where it grows in somewhat disturbed cloud forest around 2200–2300 m.

**BOLIVIA. Santa Cruz**: Prov. Florida, La Yunga de Mairana, *M. Nee et al.* 52029 (K, NY, USZ); ibid., *J.R.I. Wood et al.* 19636 (K, LPB, USZ); ibid., *J.R.I. Wood* 28111 (LPB, OXF, USZ)

#### Note.

Readily distinguished from *Ipomoea aristolochiifolia* by its relatively large denticulate leaves 4–5 cm in length, larger corolla and by the peduncle that does not pass through the leaf sinus.

### 300. Ipomoea huayllae

Taxon classificationPlantaeSolanalesConvolvulaceae

J.R.I. Wood & Scotland, Kew Bull. 70 (31): 108. 2015. (Wood et al. 2015: 108)

#### Type.

BOLIVIA. La Paz, Prov. Tamayo, ANMI Apolobamba, camino Pelechuco-Apolo, entre Puente Coronara y Hac. Corapara, *A. Fuentes & H. Huaylla* 12939 (holotype LPB; isotypes MO, OXF, K).

#### Description.

Twining herb, possibly annual, stems thinly pubescent with spreading hairs when young, glabrescent when older. Leaves petiolate, 6–10 × 3–7 cm, ovate, acute and finely mucronate, base cordate with rounded auricles, margin entire to slightly undulate; petioles 1–7 cm, pubescent. Inflorescence of solitary axillary flowers (rarely a second, non-developing flower present); peduncle, 3–5.5 cm, pubescent, penetrating leaf sinus; bracteoles 1 × 0.25 mm, deltoid, obtuse, green with white margins; pedicels 6–12 mm, thickened upwards, pubescent; sepals slightly unequal, outer 6 × 2.5 mm, acute, green, pubescent, the hairs with swollen bases, margins scarious, glabrous, inner sepals 7–9 × 4 mm, broadly oblong-elliptic, minutely mucronate, only the middle green and pilose, the margins and apex scarious, glabrous; corolla glabrous, c. 4 cm long, funnel-shaped with the rim of the limb recurved, pale blue with whitish tube and midpetaline bands, limb c. 4 cm diam., unlobed, midpetaline bands ending in a point. Capsules glabrous, ovoid, 14–15 × 11 mm; seeds 7 × 6 mm, flattened-ovoid, dark brown, superficially glabrous but minutely pilosellous under a microscope.

#### Illustration.

Figure 147.

**Figure 147.**
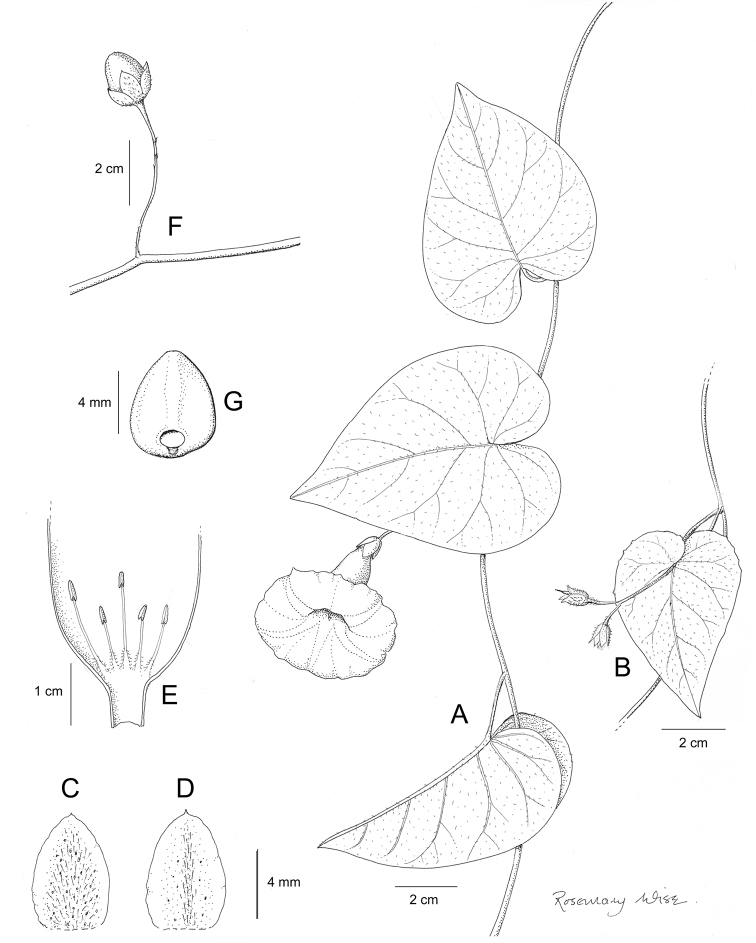
*Ipomoea huayllae*. **A** habit **B** inflorescence showing position of peduncle **C** outer sepal **D** inner sepal **E** corolla opened out to show stamens **F** capsule **G** seed. Drawn by Rosemary Wise **A, G–F** from *Fuentes et al.* 12939; **B–E** from *Huaylla et al.* 2754.

#### Distribution.

Endemic to Bolivia and only known from Yungas cloud forest with secondary vegetation between 2100–2300 m in the ANMI Apolobamba.

**BOLIVIA. La Paz**: Prov. Tamayo, ANMI Apolobamba, camino Pelechuco-Apolo, *H. Huaylla et al.* 2754 (MO, OXF).

#### Note.

Closely related to *Ipomoea aristolochiifolia* as apparent from the peculiar placement of the peduncle in the leaf sinus but immediately distinguished by the larger corolla c. 4 cm long (not 1.5–2.5 cm), the pubescent sepals and the larger leaves, pubescent beneath.

### 301. Ipomoea elongata

Taxon classificationPlantaeSolanalesConvolvulaceae

Choisy in A.P. de Candolle, Prodr. 9: 355. 1845. (Choisy 1845: 355)

Calonyction dubium M. Martens & Galeotti, Bull. Acad. Roy. Sci. Bruxelles 12: 268. 1845. (Martens and Galeotti 1845: 268). Type. MEXICO. Oaxaca, Misteca-Alta et Yavezia, *H. Galeotti* 1362 (lectotype BR00006972943, designated here; isolectotypes BR, G).
Ipomoea dubia (M.Martens & Galeotti) Hemsley, Biol. Centr.-Amer., Bot. 2: 286. 1882. (Hemsley 1882: 286), nom. illeg., non Ipomoea dubia Roem. & Schult. (1819).
Ipomoea mestecensis House, Bot. Gaz. 43: 411. 1907. (House 1907b: 411). Type. Based on Calonyction dubium M. Martens & Galeotti

#### Type.

MEXICO. Oaxaca, *Andrieux* 212 (holotype G00135527, isotype K).

#### Description.

Trailing or twining perennial, stems slender, nearly glabrous. Leaves petiolate, 2.3–5.5 × 1–2 cm, deltoid, acuminate to an aristate point, base sagittate with elongate, lanceolate or ovate, acute, sometimes bifurcate auricles, margin entire, undulate or with a few broad teeth, both surfaces glabrous to thinly pubescent, abaxially paler; petioles 1–3 cm, thinly pubescent. Inflorescence of solitary (rarely paired) pedunculate, axillary flowers; peduncles 5–48 mm, sometimes penetrating leaf sinus, thinly pubescent bracteoles 1–2 mm, deltoid; pedicels 3–19 mm, commonly bent at right angles to peduncle, thinly pubescent; sepals unequal, outer (5–) 8–9 × 2–2.5 mm, lanceolate, acuminate, glabrous but often muricate on dorsal surface, inner (8–) 9–11 × 3 mm, oblong, obtuse, mucronate, margins broadly scarious; corolla 6–10 cm long, narrowly funnel-shaped with a long gradually widening paler tube, glabrous, midpetaline bands terminating in a distinct tooth, limb pink 3.5–4 cm diam. Capsules subglobose, glabrous; seeds 4, 6–7 × 4–5 mm, puberulent.

#### Illustration.

**Figure 148.**
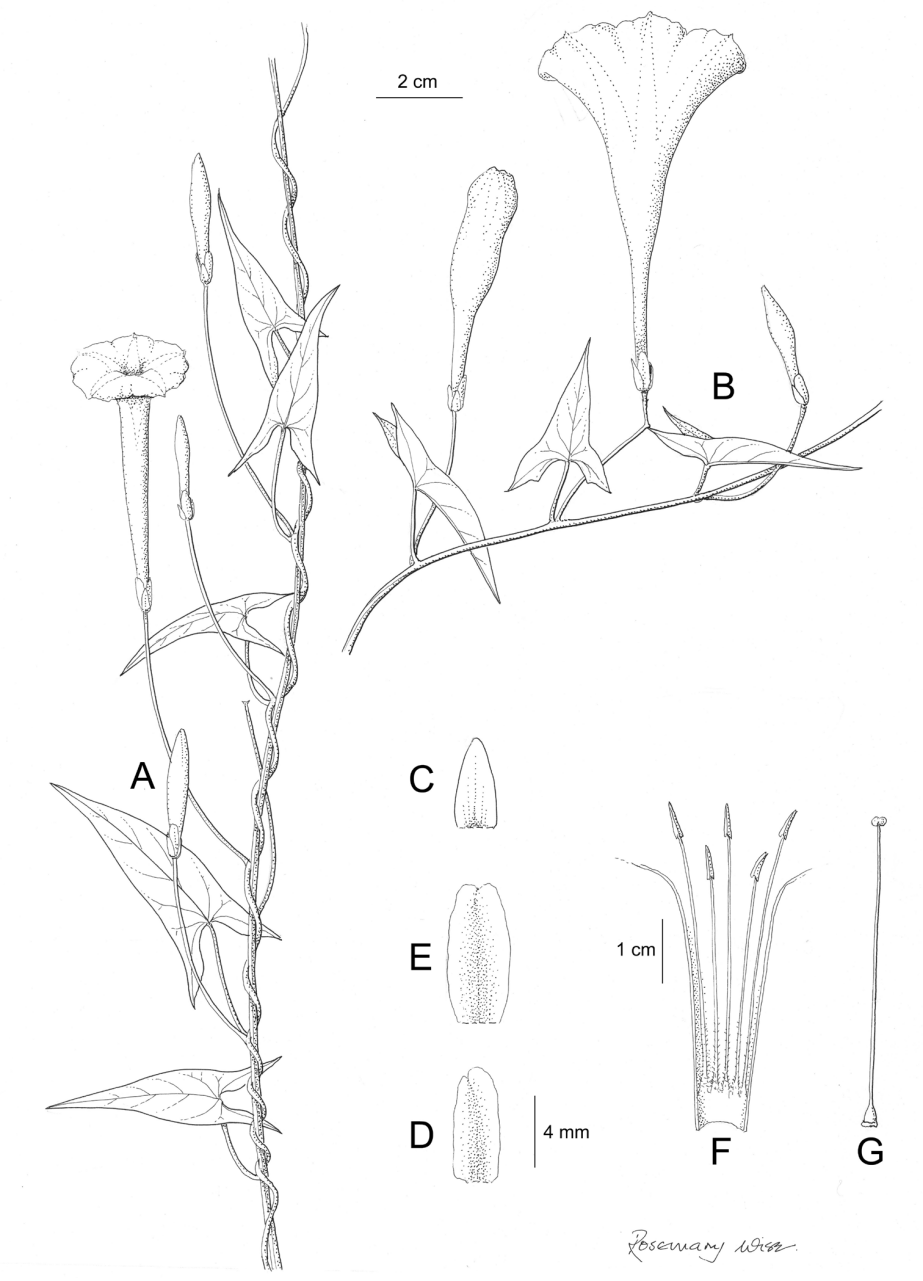
*Ipomoea elongata*. **A** habit **B** habit **C** outer sepal **D** middle sepal **E** inner sepal **F** corolla opened up to show stamens **G** ovary and style. Drawn by Rosemary Wise **A, B–G** from *Hinton* 8474; **B** from *Purpus* 3904.

McDonald (1987c: 84); Figures 8Q, 148.

#### Distribution.

Low oak woodland and scrub, mostly between 1500 and 2500 m in southern Mexico and Guatemala.

**GUATEMALA.***Huehuetenango, A. & A.R. Molina* 26497 (F).

**MEXICO. Est. México & Dist. Fed.**: Temascaltepec, *G.B. Hinton et al.* 4987 (K); ibid., Nanchitla *G.B. Hinton et al.* 8474 (K); Valle de Bravo, *E. Matuda et al.* 31768 (MO). **Guerrero**: Mina, *G.B. Hinton* 9703 (GBH, K), Manchón, *G.B. Hinton* 9646 (GBH, K, MO), Montes de Oca, *G.B. Hinton* 11542 (GBH, K). **Michoacán**: Charo, Pie de la Mesa, *E. Carranza & M.E. Molina* 7143 (IEB). **Oaxaca**: *C.G. Pringle* 4693 (BM, CM, K, MO); Cerro San Felipe, *C. Conzatti* 4975 (MO); El Cerezal, Ixtlan, *D.H. Lorence et al.* 3542 (MO); Cerro Verde, San Luis Tultitlanapa, *C.A. Purpus* 3535 (BM); San Felipe Tejalapam, *M. Cruz* 570 (IEB); Juxtlahuaca, *A. García Mendoza* 5093(MEXU). **Puebla**: Lomas de San Alfonso, *J.L. Contreras* 7749 (MEXU); Cerro del Pavilan, *C.A. Purpus* 3904 (BM, MO). **Querétaro**: *E. Pérez & E. Carranza* 3766 (IEB); Jalpan de Serra, *E. Carranza et al.* 5872 (IEB).

#### Note.

The muricate outer sepals are rather distinctive. Some Querétaro specimens have large lateral teeth.

### 302. Ipomoea schaffneri

Taxon classificationPlantaeSolanalesConvolvulaceae

S. Watson, Proc. Amer. Acad. Arts 18: 123. 1883. (Watson 1883: 123)

#### Type.

MEXICO. San Luis de Potosí, *J.W. Schaffner* 621 (holotype GH00054541, isotypes K, MEXU).

#### Description.

Trailing or climbing herb, stems thinly pilose. Leaves shortly petiolate, 2.5–4.5 × 2–4.5 cm, broadly ovate to suborbicular, base broadly cordate and cuneate onto the petiole, apex shortly acuminate, mucronulate, margin irregularly sinuate to coarsely dentate, thinly adpressed pilose on both surfaces, especially on the veins; petioles 1–3 cm. Inflorescence of solitary, pedunculate flowers; peduncles 1.5 –3 cm; bracteoles 3–7 mm, lanceolate, semi-persistent; pedicels 5–18 mm, noticeably stouter than peduncles; sepals slightly unequal, outer 8–12 × 3–4 mm, ovate, acute, thinly pilose, the margins scarious, inner similar but 1–2 mm longer, mucronate and with fewer hairs; corolla 5–6 cm long, narrowly funnel-shaped, pale pink, glabrous; stamens held at corolla mouth. Capsules conical, c. 10 mm long; seeds 6 × 5 mm, brown, puberulent.

#### Illustration.

McDonald (1987c: 86).

#### Distribution.

Endemic to north east Mexico, apparently only known from the type.

**MEXICO. San Luis de Potosí**: type collection.

### 303. Ipomoea ignava

Taxon classificationPlantaeSolanalesConvolvulaceae

House, Ann. New York. Acad. Sci. 18: 214. 1908. (House 1908b: 214)

Ipomoea maltratana Standl., Publ. Field Mus. Nat. Hist., Bot. Ser. 22: 46. 1940. (Standley 1940b: 46). Type. MEXICO. Veracruz, Maltrata, *E. Matuda* S106 (holotype F0054852, isotype F).

#### Type.

MEXICO. Oaxaca, Las Sedas a La Carbonera, *C. Conzatti & G. Gonzáles* 261 (holotype GH00054504, isotype NY).

#### Description.

A slender trailing or twining herb, stems glabrous. Leaves petiolate, 1–5.5 × 1–6 cm, ovate, acuminate, mucronulate, base hastate, auricles rounded or angled, margin dentate with large teeth, strigose on both surfaces or glabrous adaxially; petioles 0.5–4 cm. Inflorescence of solitary or paired flowers from the leaf axils; peduncles 1.2–4.8 cm, erect; bracteoles 1–2 mm, lanceolate; pedicels 4–13 mm, thicker than peduncles, becoming reflexed; sepals unequal, glabrous, strongly muricate, outer 3–4 × 3–4 mm, ovate, acute or obtuse, often mucronate, inner 5–8 × 3 mm, oblong-lanceolate, mucronate; corolla 4–5.5 cm long, funnel-shaped, pink or purplish, glabrous, limb 4–5 cm diam. Capsules 8 × 6 mm, conical, glabrous; seeds 5 × 4 mm, puberulent.

#### Illustration.

McDonald (1987c: 86).

#### Distribution.

A rare Mexican endemic of dry deciduous forest at altitudes of 2000–2400 m.

**MEXICO. Est. México & Dist. Fed.**: Temascaltepec, Nanchititla, *G.B. Hinton* 8563 (GBH, MO, n.v.). **Guerrero**: Chilpancingo, *H. Kruse* 2097 (IEB, MEXU). **Oaxaca**: La Carbonera, *C. Conzatti* 804 (GH); Santiago Naranjas, *S. Zamudio et al.* 12817 (IEB); Tlaxiaco, *R. Torres et al.* 7145 (MEXU). **Querétaro**: Cadereyta, *S. & E. Zamudio* 10296 (IEB). **Veracruz**: type of *Ipomoea maltratana*.

#### Note.

Differs from *Ipomoea schaffneri* in the shorter sepals and the clearly funnel-shaped corolla.

### 304. Ipomoea eximia

Taxon classificationPlantaeSolanalesConvolvulaceae

House, Muhlenbergia 3: 44. 1907. (House 1907a: 44)

#### Type.

MEXICO. [Veracruz], Orizaba, *F. Műller* s.n. (holotype NY00319087).

#### Description.

Trailing perennial herb, stems slender, glabrous. Leaves shortly petiolate, 1–2 × 1–2 cm, ovate to deltate or reniform, apex obtuse, mucronulate, base cordate, margins strigose; petioles 4–14 mm. Flowers solitary, axillary; peduncles 0.4–1.1 cm, glabrous or thinly strigose; bracteoles caducous, not seen; pedicels 4 mm, muricate; sepals unequal, oblong, outer 3–4 × 2 mm, acute, muricate, central vein prominent, inner 4–5 mm, acute or obtuse, smooth; corolla 5–7 cm long, narrowly funnel-shaped, purple with white tube, apparently glabrous, limb c. 4 cm diam. Capsules and seeds unknown.

#### Illustration.

McDonald (1987c: 86).

#### Distribution.

A rare species endemic to central Mexico, where it is recorded from Pine Forest at around 1800 m.

**MEXICO. Hidalgo**: Los Reyes, *E. Matuda* 37451 (MEXU), 37452 (IEB). **Veracruz**: type collection.

#### Note.

Somewhat similar to *Ipomoea ignava*, but leaves entire, deltoid in shape and with smaller, muricate sepals. The corolla of the Matuda specimen is rather small but otherwise fits well.

### 305. Ipomoea meyeri

Taxon classificationPlantaeSolanalesConvolvulaceae

(Spreng.) G. Don, Gen. Hist. 4: 275. 1838. (Don 1838: 275)

Convolvulus meyeri Spreng., Syst. Veg. 1: 597 1824 [pub.1825]. (Sprengel 1824: 597). Type. Plant of unknown origin, *T. Meyer* in Herb. Willd (holotype B-W03633 as Convolvulus cuspidatus).
Convolvulus hederaceus Mill., Gard. Dict., ed. 8. 1768. (Miller 1768Convolvulus No. 17), nom. illeg., non Convolvulus hederaceus L. (1753). Type. JAMAICA. Sloan s.n. (BM000589539).
Ipomoea brachypoda Benth., Bot. Voy. Sulphur 135 1844 [pub. 1845]. (Bentham 1845: 135). Type. PANAMA. “Colombia”, Sinclairs.n. (lectotype K000612870, designated here).
Ipomoea caerulea Bello , Anales Soc. Esp. Hist. Nat. 10: 296. 1881. (Bello y Espinosa 1881: 296), *nom. illeg.*,non Roxb. (1818). Type. PUERTO RICO. Bello, (not found, probably B†).
Ipomoea iostemma House, Ann. New York Acad. Sci. 18: 207. 1908. (House 1908b: 207). Type. COSTA RICA. Nicoya, A. Tonduz 13680 (NY00319099, isotype K).
Ipomoea iodantha Brandegee, Univ. California Publications in Botany 4(19): 383. 1913. Type. MEXICO. Baja Caifornia Sur, Cape region, La Mesa, *T.S. Brandegee*s.n. (UC105204).
Ipomoea chiapensis Brandegee, Univ. Cal. Publ. Bot. 6; 60. 1914 (Brandegee 1914: 60). Type. MEXICO. Chiapas, Tonala, C.A. Purpus 6907 (holotype UC172963, isotypes BM, F, MO, NY).

#### Type.

Based on *Convolvulus meyeri* Spreng.

#### Description.

Twining herb, possibly annual; stems slender, glabrous to thinly pubescent or pilose. Leaves petiolate, 2–9 × 1.7–7.5 cm, ovate-deltoid, cordate with rounded auricles, apex acuminate, shortly mucronate; margin slightly undulate or with a broad triangular lateral tooth on either side above the rounded auricles, rarely shallowly 3 lobed, usually glabrous, sometimes adaxially thinly pilose; petioles 0.5–10 cm, thinly pilose. Inflorescence of dense pedunculate axillary clusters, noticeably shorter than the leaves; peduncles 0.3 to 4(–8) cm, glabrous; bracteoles 7–25 mm, linear, thinly pilose, persistent; secondary peduncles 0–17 mm; pedicels 2–7 mm, glabrous; sepals subequal, herbaceous, linear-lanceolate, long-acuminate with a partially recurved apical mucro, outer (12–)15–17 × 2 mm, accrescent to 20 × 4 mm, glabrous, thinly or densely long-pilose especially towards the base, inner sepals with thin scarious margins, c. 2 mm shorter; corolla 2.3–3 cm long, subcampanulate, the tube white but lobes blue, glabrous, limb 2 cm diam., entire. Capsules 6–8 × 5–6 mm, ovoid, rostrate with 4 mm long persistent style, scurfy puberulent; seeds 4 × 3 mm, tomentose.

#### Illustration.

**Figure 149.**
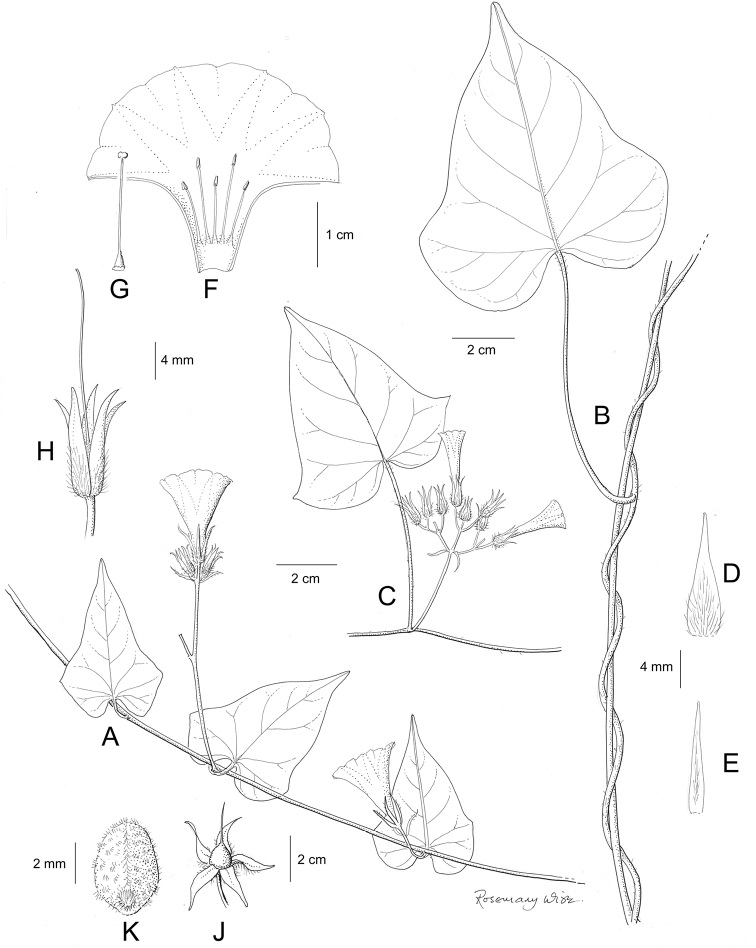
*Ipomoea meyeri*. **A** habit **B** stem and leaves **C** inflorescence **D** outer sepal **E** inner sepal **F** corolla opened out to show stamens **G** ovary and style **H** calyx in fruit **J** capsule **K** seed. Drawn by Rosemary Wise **A, C–H** from *Anderson* 1895 B from Araya 3, **J, K** from *H.H. Smith* 1573.

Acevedo-Rodríguez (2005: 172); Austin (1998: 403); Figures 7A, 11K, 149.

#### Distribution.

Moist scrubby forest and disturbed bushy habitats, usually below 500 m, from northern Brazil and Peru to NW Mexico and the larger Caribbean Islands; apparently absent or rare in some areas including smaller islands, southern Colombia, Guatemala and Belize and very scattered in occurrence towards the limits of its range, especially in South America.

**BRAZIL. Pará**: Santarém, *Zerny* s.n. (W).

**PERU. Cusco**: La Convención, Echarate, Papelpata, *G. Calatayud et al.* 4137 (MO, OXF).

**ECUADOR. Guayas**: *J.E. Madsen* 63753 (AAU); *C.H. Dodson & A. Gentry* 13872 (MO). **El Oro**: *J. Steyermark* 54079 (F). **Esmeraldas**: *J. Hudson* 731 (MO).

**COLOMBIA. Atlántico**: Barranquila, *H. Elias* s.n. (COL). **Bolívar**: Turbaco, *E.P. Killip & A.C. Smith* 14232 (F, US); *J. Espina* 818 (COL). **Cesar**: *C. Allen* 835 (K, MO). **Córdoba**: *B. Anderson* 1895 (COL, K). **Magdalena**: Santa Marta, *H.H. Smith* 1573 (BM, E, K, MO, NY, P, S).

**VENEZUELA. Anzoátegui**: Libertad, *G. Davidse & A.C. González* 19736 (MO). **Aragua**: Tovar, *A. Fendler* 934 (K, MO). **Bolívar**: *E. Holt & W. Gehriger* 181 (NY, US, VEN). **Carabobo**: Barbula, *Ll. Williams & A.H.G. Alston* 342 (P, BM, S). **Dist. Fed.**: *T. Croat* 21596 (MO). **Miranda**: *K.R. Robertson & D.F. Austin* 219 (MO).

**PANAMA.** Herrera, *P. H. Allen* 4073 (MO); Chiriqui, *T.B. Croat* 22542 (MO).

**COSTA RICA.** Guanacaste, *U. Chavarría* 1370 (K, MO); Puntarenas, *B.E. Hammel* 18633 (CR, MO); San José, *D. Santamaria* 377 (CR, MO).

**NICARAGUA.** Nueva Segovia, *P.P. Moreno* 13354 (MO); Rivas, *W.D. Stevens & O.M. Montiel* 30424 (MO).

**HONDURAS.** Comayagua, *C.H. Nelson et al.* 6265 (MO)

**EL SALVADOR.** Morazán, *J.M. Tucker* 482 (K); Santa Ana, Metapán, *J. Monterrosa* 2108 (LAGU, MO, W).

**GUATEMALA.** Fide Standley and Williams 1970: 41.

**MEXICO. Baja California Sur**: type of *Ipomoea iodantha*. **Chiapas**: type of *Ipomoea chiapensis*. **Guerrero**: Petatlan. *E. Langlassé* 641 (K); Coyuca, *G.B. Hinton* 6908 (K); Mina, *G.B. Hinton* 9808 (BM, K); ibid., *G.B. Hinton*. 9674 (K). **Michoácan**: Lázaro Cárdenas, near La Mira, *E. Carranza & I. Silva* 6882 (IEB). **Oaxaca**: Tehuantepec, *S.H. Salas et al.* 3377 (MEXU, MO). **Querétaro**: Jalpán de Serra, Tancanaquito, *E. Carranza & I. Silva* 6000 (IEB). **Quintana Roo**: José María Morelos, *F. Gaumer* et al. 2125 (F, MO, S). **Sonora**: Mun. Alamos, Río Mayo, *A.C. Sanders et al.* 12560 (ARIZ); Rancho Mezquite Cuate, Arroyo de Alamos, *C.D. Bertelsen & C. Smith* 92-134 (ARIZ). **Vera Cruz**: *J.A. McDonald* 1954 (XAL). **Yucatán**: Izamal, *F. Gaumer et al.* 991 (K, MO, S).

**CUBA.***Bro. Clemente* 5694 (HAC), 5732 (HAC); *C. Wright* 451 (K); Guantanamo, Bayate, *E.L. Ekman* 6555 (BM, S).

**JAMAICA.***McFadyen* s.n. (K); *E.T. Robertson* 768 (K); St. Andrew, *G.R. Proctor* 8280 BM); ibid., *C.D. Adams* 8509 (BM), St Thomas, *G.R. Proctor* 2421 (BM).

**HAITI.***E.L. Ekman* H7221 (K, S); St. Raphael, *E.C. Leonard* 9102 (S, US).

**DOMINICAN REPUBLIC.***E.L. Ekman* H10916 (S); *H. A. Allard* 13880 (MO, S); *A. Liogier* 9065-21 (MO).

**PUERTO RICO.***P. Sintenis* 828 (MO, S), 5533 (BM, K).

**TRINIDAD.***A. Fendler* 587 (BM, K).

### 306. Ipomoea mitchelliae

Taxon classificationPlantaeSolanalesConvolvulaceae

Standl., Publ. Field Mus. Nat. Hist., Bot. Ser. 8: 39. 1930. (Standley 1930: 39)

Ipomoea variabilis auct. sensu Austin et al. 2012: 341.

#### Type.

HONDURAS. Atlántica, La Fragua, *P. C. Standley* 52658 (holotype F0054855, isotype US).

#### Description.

Twining or trailing perennial; stems slender, rugose, glabrous or pilose. Leaves petiolate; 6–15 × 4–12 cm, ±ovate-deltoid, sometimes shallowly 3-lobed, margin sometimes with a single lateral tooth, base subhastate, apex obtuse and mucronate; petioles 6–12 cm. Inflorescence of 1–4-flowered axillary cymes; peduncles 3–5 cm; bracteoles c. 6–8 × 2 mm, linear-oblong, relatively persistent; pedicels 5–8 mm; sepals subequal, 12–14 × 3–4 mm, lanceolate to ovate, acuminate to apiculate, herbaceous, pilose with spreading hairs near base but glabrous towards the apex, the inner sepals narrower; corolla 6–7 cm long, funnel-shaped, glabrous, the tube whitish, the limb blue. Capsules subconical, 2.5–3 cm long and wide, glabrous; seeds 3–4 mm long, rounded, puberulent, black.

#### Distribution.

Primary and secondary woodland at low altitudes in southern Mexico and Central America, locally common, for example in Veracruz, but perhaps overlooked, particularly in Central America.

**COSTA RICA.***P. Wilkin* 474 (BM).

**HONDURAS.***F. de La Puente* 4569 (CIP), 4455 (CIP); La Mosquitia, *C. Ashe* 159 (BM).

**MEXICO. Campeche**: fide Austin et al. (2012). **Chiapas**: *Aguilar* 996 (MO); Ocosingo, *S. Sinaca & R. Lombera* 2472 (IEB); Motozintla, *A. Bourg* 150 (IEB). **Oaxaca**: *R. López Luna* 355 (NY); Sierra San Pedro Nolasco, *Jurgensen* 857 (K); Temazcal, *M. Sousa* 1007 (MEXU). **Puebla**: *F. Ventura* 21703 (F). **Quintana Roo**: fide Austin et al. (2012). **San Luis Potosí**: *R.M. King* 4389 (F, NY). **Veracruz**: *F. Müller* 119 (NY); *Gouin* s.n. (P); *R. Pedraza* 269 (F); *M.A. García et al.* 589 (IEB); *M. Rosas* 1390 (MEXU). **Yucatán**: *E. Ucán Ek* 3139 (XAL).

#### Note.

Resembles *Ipomoea meyeri* but differs in the larger corolla. It is also confused with and is superficially similar to *Ipomoea indica* but differs in the bilocular capsule with 4 seeds (v. trilocular with 6 seeds) and the distinctive sepals with long, often yellowish hairs.

For a discussion about the application of the name *Ipomoea variabilis*, See Austin and MacDonald (2014b).

### 307. Ipomoea expansa

Taxon classificationPlantaeSolanalesConvolvulaceae

McDonald, Brittonia 34: 336. 1982. (McDonald 1982: 336)

#### Type.

MEXICO. Guerrero, 3.6 miles N of turnoff to San Vicente de Benitezon road from Atoyac to El Paraíso, *J.A. McDonald* 185 (holotype TEX00372564, isotypes MEXU, TEX).

#### Description.

A slender trailing or twining perennial to 8 m, stems becoming woody. Leaves petiolate, or subsessile on fertile branches, 2.5–10 × 1–7 cm, often somewhat dimorphic, ovate to broadly lanceolate, cordate, hastate or sagittate with rounded or acute auricles, sometimes with dentate lobes, apex acuminate, glabrous; petioles 1–6 cm. Flowers solitary or paired, axillary; peduncle 0.5–2 cm, often penetrating the leaf sinus; bracteoles minute, c. 1 mm long; pedicels 8–30 mm, often stouter than peduncle; sepals slightly unequal, glabrous, oblong-elliptic, obtuse, margins white, scarious, outer 3–5 × 2.5–3 mm, inner 6 × 3 mm; corolla 4–6 cm long, pale blue, glabrous, subsalverform, flaring upwards, the basal cylindrical tube 1–1.5 cm long, limb 3–3.5 cm diam. Capsules conical, 11 × 7 mm, glabrous; seeds up to 4, 4–5 × 3–4 mm, minutely puberulent.

#### Illustration.

McDonald (1987c: 82).

#### Distribution.

A rare species of disturbed areas on the southern slopes of the Sierra Occidental growing in moister areas than *Ipomoea puncticulata*.

**MEXICO.** “Sierra Madre”, *E. Langlassé* 903 (K, P). **Guerrero**: Atoyac, El Ranchito, *J.C. Soto Nuñez & E.M. Martínez* 5109 (MEXU). **Oaxaca**: Pochutla Dist., Concordia, *E. Makrinius* 841 (US); Tlaxiaco, Cerro Yucuntusu, *M. Mendoza* 134 (IEB); Etla, San Felipe Tejalapa, *C. Cervantes-M* 149 (MEXU).

#### Note.

Very close to *Ipomoea puncticulata* differing in the larger, blue-coloured corolla with a distinct cylindrical basal tube up to 1.5 cm long. The leaves are often dimorphic, differing in appearance on trailing or twining stems.

### 308. Ipomoea puncticulata

Taxon classificationPlantaeSolanalesConvolvulaceae

Benth., Bot, Voy. Sulphur 136. 1844 [pub. 1845]. (Bentham 1845: 136)

Ipomoea sagittula House, Ann, New York. Acad. Sci. 18: 244. 1908. (House 1908b: 244). Type. MEXICO. Jalisco, San Sebastián–Las Palmas, *E.W. Nelson* 4129 (holotype US00111463, isotype GH).

#### Type.

MEXICO. Guerrero, circa Acapulco, *Sinclair* s.n. (holotype K000612722).

#### Description.

Slender twining perennial with wiry whitish stems with flaky bark, reaching several metres in length, sometimes rooting at the nodes. Leaves petiolate, 2–8 × 1–6 cm, lanceolate, acute and mucronate, base sagittate, cordate or subtruncate, the auricles up to 1 cm long, adaxially glabrous, abaxially paler, white-punctate, glabrous or puberulent; petioles 3–20 mm. Inflorescence of 1–5-flowered axillary cymes; peduncles 1.5–2 cm, often penetrating leaf sinus as in *I. aristolochiifolia*; bracteoles scale-like, c. 1 mm long; pedicels 5–10 mm; sepals very unequal, oblong-elliptic to lanceolate, outer 2–5 × 2 mm, acute, inner 5–7 mm, rounded; corolla 3.5–4 cm long, funnel-shaped, gently flared from a slender base, white, glabrous, limb 3.5–4 cm diam., unlobed. Capsules ovoid, 8–9 × 6–7 mm; seeds 4, 4 × 3 mm, dark brown, puberulent.

#### Illustration.

McDonald (1987c: 82).

#### Distribution.

A rare species of central Mexico, growing in disturbed deciduous forest between 400 and 1800 m.

**MEXICO. Guerrero**: Vallecitos, Montes de Oca, *G.B. Hinton* 11612 (GH, K, MO, US); La Unión, *V.W. Steinmann & J.M. Porter* 9496 (MEXU). **Jalisco**: NW of San Sebastián, *Y. Mejia* 1896 (BM, F, GH, MO, US); 7 miles S of El Tuito, *R. Spellenberg* 6438 (MICH). **Michoacán**: Coalcomán, *G.B. Hinton* 12594 (F, GH, K, MO, US); ibid., Aguila *G.B. Hinton* 12615 (K); San Juan de Lima, *R. McVaugh* 22991 (MICH); Lazáro Cárdenas, *E. Carranza & I. Silva* 7279 (MEXU). **Nayarit**: *G. Flores et al.* 943 (MO); San Blas & Tepic, *G.W. Barclay* s.n. (BM).

#### Note.

In the type the leaves are densely white-punctate on the lower surface. These dots only occur obscurely in *Hinton* 11612 and 12594.

### 309. Ipomoea nationis

Taxon classificationPlantaeSolanalesConvolvulaceae

(Hook.) G. Nicholson, Ill. Dict. Gard. 2: 191. 1885. (Nicholson 1885: 191)

Quamoclit nationis Hook., Bot. Mag. 90: t. 5432. 1864. (Hooker, WJ1864: 191). Type. PERU. *A. Mathews* 721 (lectotype K000612866, designated here; isolectotypes K, OXF).

#### Type.

Based on *Quamoclit nationis* Hook.

#### Description.

Perennial climbing herb with root tubers, stems glabrous or pilose. Leaves petiolate, 2–8.5 × 1.3–8 cm, ovate-deltoid to suborbicular, acute, base truncate to cordate, the auricles rounded to subacute, margin entire or undulate, abaxially paler, glabrous except at apex of petiole and on main veins beneath; petioles 1–7 cm, usually glabrous below but pubescent upwards. Inflorescence of long pedunculate, 1–3-flowered axillary cymes; peduncles 6–20 cm, thinly retrorse-pilose, occasionally glabrous; bracteoles 1–3 mm, lanceolate, tardily caducous; pedicels 7–15 mm, retrorse-pilose; sepals unequal, outer 8–10 × 2–3 mm, oblong-lanceolate, acute to mucronulate, pubescent, green, inner 8–10 × 4 mm, ovate, obtuse and strongly mucronate, scarious apart from a green central area; corolla scarlet, glabrous, hypocrateriform with cylindrical tube 3–3.5 cm long and c. 0.5 cm wide, limb 3–5 cm diam.; stamens exserted. Capsules 6 × 5 mm, ellipsoid, enclosed by persistent sepals, glabrous; seeds 4 × 1.5 mm, glabrous.

#### Illustration.

Figures 8G, 150.

**Figure 150.**
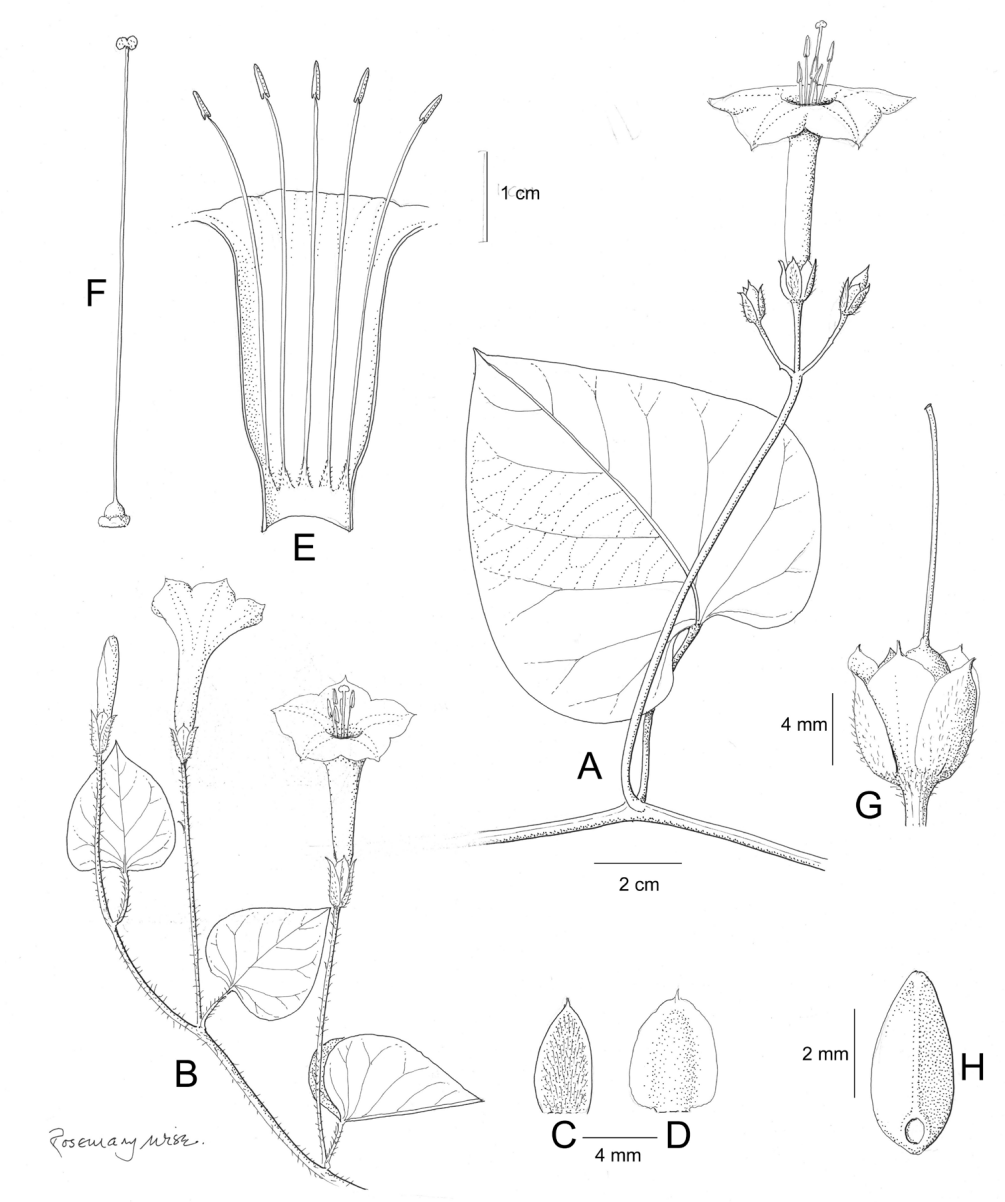
*Ipomoea nationis*. **A** habit **B** habit with single flower **C** outer sepal **D** inner sepal **E** corolla opened out to show stamens **F** ovary and style **G** Capsule and calyx in fruit **H** seed. Drawn by Rosemary Wise **A** from *Stork & Horton* 9261 B from *Matthews* 778, **C–G** from *Saunders* 987 H from *Sandeman* 4339.

#### Distribution.

Endemic to Peru, occurring principally in the coastal lomas near Lima, but ascending to at least 2700 m in the Canta district.

**PERU. Cajamarca**: San Pedro, *A. Sagástagui et al.* 15609 (F, MO). **Junín**: Satipo-Junín, *F. de la Puente* 680 (CIP). **Lima**: *D. Stafford* 36 (K); Matucana, *J.F. Macbride & Featherstone* 144 (F); Canta, *R. Ferreyra* 8992 (USM); Lomas region, *C. Sandeman* 4339 (OXF), *R. Ferreyra* 6917 (F, USM); Puruchuca, *A. Matthews* 778 (K).

#### Typification.

In choosing a lectotype we have selected the Mathews collection at Kew. This sheet has a copy of the Botanical Magazine plate pinned to it and it is obvious that the illustration and much of the protologue must have been based on this collection rather than the piece sent by W. Nation (K000612867) which lacks an open corolla.

### 310. Ipomoea alexandrae

Taxon classificationPlantaeSolanalesConvolvulaceae

D.F. Austin, Fl. Ecuador 15: 36. 1982. (Austin 1982a: 36)

#### Type.

ECUADOR. Loja, SW slope of Cerro Villonaco, 2100 m, *B. Sparre* 16212 (holotype S07-4318).

#### Description.

Twining perennial with stems pale brown, wiry, probably woody below, glabrous. Leaves petiolate, 2.5–4.5 × 2–3 cm, ovate-deltoid, acute to acuminate, base subtruncate to very shallowly cordate, glabrous with prominent veins; petioles 0.5–2 cm. Inflorescence of very compact, shortly pedunculate, axillary cymes of up to 6 flowers, sometimes borne on short branchlets; peduncles 2–4 mm; bracteoles 1–2 mm, deltoid, subscarious, caducous; pedicels 8–10 mm; sepals very unequal, outer 3–4 × 2–3 mm, ovate, usually emarginate and mucronate, inner 7–8 × 4 mm, oblong to oblong–ovate, emarginate, margins broad, scarious; corolla scarlet, glabrous, salverform with cylindrical tube 2.5–2.8 × 0.6–0.8 cm, the limb 3.5 cm diam., slightly lobed. Capsules and seeds unknown.

#### Illustration.

Figure 151.

**Figure 151.**
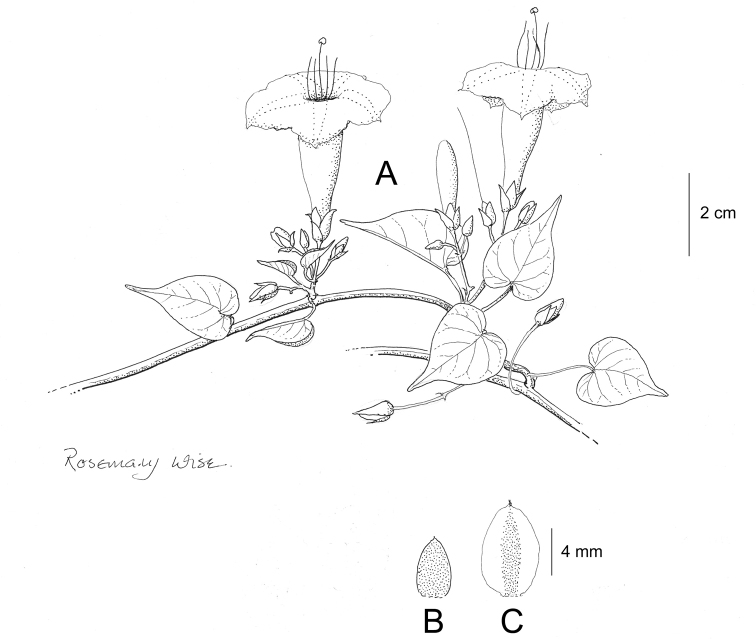
*Ipomoea alexandrae***A** habit **B** outer sepal **C** inner sepal. Drawn by Rosemary Wise from *Sagástagui & Téllez* 12708.

#### Distribution.

Southern Ecuador and northern Peru, on mountain slopes between 1500 and 2000 m; known from one collection in each country and only one collection apart from the type.

**PERU. Cajamarca**: Choropampa–Magdalena, *A. Sagástagui & O. Tellez* 12708 (MO, HUT, FTG, NY).

**ECUADOR. Loja**: type collection.

#### Note.

Appears to be related to *Ipomoea nationis* but sepals very unequal in size, the corolla tube shorter and all parts glabrous. The placement of this species is provisional.

### 311. Ipomoea velardei

Taxon classificationPlantaeSolanalesConvolvulaceae

O’Donell, Bol. Soc. Peruana de Bot. 1: 6. 1948. (O’Donell 1948b: 6)

Ipomoea velardei var. aequatoriana O’Donell, Lilloa 26: 395, t. 17. 1953. (O’Donell 1953a: 395). Type. ECUADOR. Chimborazo: 1200 m, *A.S. Hitchcock* 20301 (holotype NY00319239; isotypes GH, US).

#### Type.

PERU. Lima, Tornamesa, *Velarde Nuñez* 1633 (holotype LIL001297).

#### Description.

Probably perennial twining herb with pilose to glabrescent stems. Leaves petiolate, 4–12 × 3–10 cm, ovate to suborbicular, acuminate to an acute or obtuse apex, mucronulate, base cordate with rounded auricles, sometimes with a lateral tooth, both surfaces nearly glabrous to pubescent; petioles 2.5–6 cm, pilose. Inflorescence of long-pedunculate cymes, often subumbellate in form; peduncles 8–20 cm, pilose; bracteoles 3–5 mm, linear; pedicels 5–32 mm, glabrous to pilose, widened upwards; sepals 6–7 mm, lanceolate to oblong, finely obtuse to acute and submucronate, margin white scarious, glabrous to pilose; corolla 2.5–4 cm long, funnel-form, bluish-purple with white tube, sericeous to pilose in bud or glabrous (var. aequatoriana), limb 2.5 cm diam. Capsules 10–11 × 3 mm, ovoid, rostrate (but soon deciduous), glabrous; seeds 5–5 × 2.5–3 mm, blackish, tomentellous.

#### Illustration.

Figure 152.

**Figure 152.**
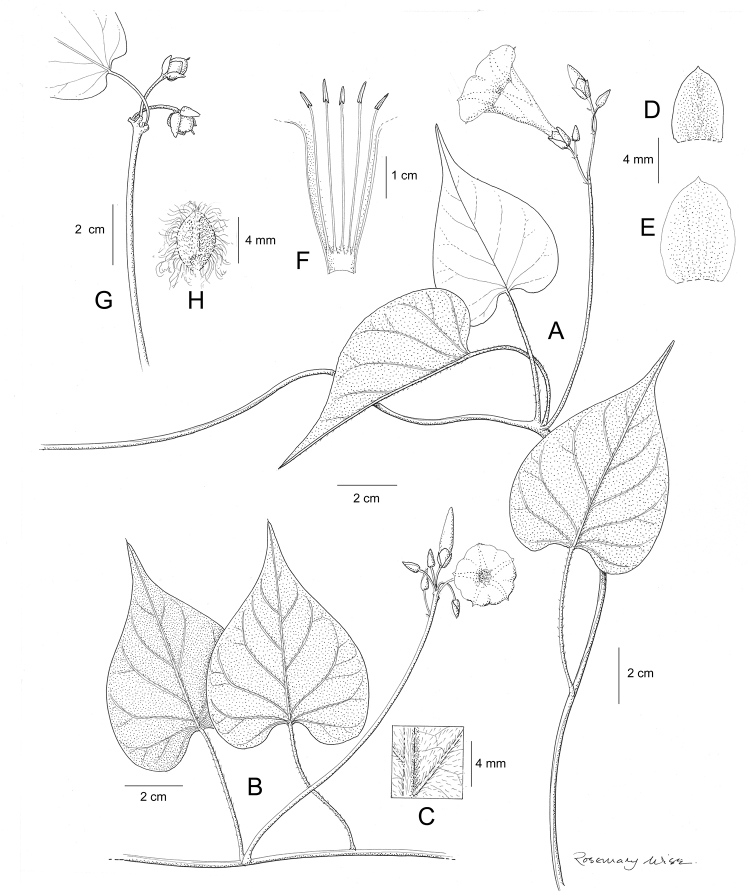
*Ipomoea velardei*. **A** habit **B** habit **C** abaxial leaf surface **D** outer sepal **E** inner sepal **F** corolla opened out to show stamens **G** fruiting inflorescence **H** seed. Drawn by Rosemary Wise **A–E, G, H** from *Benoist* 4708; **F** from *Castelnau* s.n.

#### Distribution.

A rarely collected plant of Peru and Ecuador, apparently growing in dry areas of the Andes below 2200 m.

**PERU.** Sine loc., *Castelnau* s.n. [6/1847] (P). **Ancash**: Santa Arriba de Lampanin, *J. Mostacero et al.* 1824 (FTG, MO). **Cajamarca**: Prov. Contumazá, Yetón, *A. Sagástegui et al*. 9716 (FTG, MO, OXF).

**ECUADOR. Chimbarazo**: Huigra, *Rose & Rose* 23818; *F. de la Puente* 1465 (CIP). **Loja**: between Catamayo and Loja, *F. de la Puente* 1255 (FTG, CIP); between Catamayo and Catacocha, *P.M. Jorgensen et al.* 1459 (LOJA, QCA); Sabanilla, *B. Merino et al*. 4895 (LOJA).

#### Note.

A poorly known and rather variable species distinguished (in the type variety) by its hirsute corolla and by the pilose sepals, stem peduncles and pedicels. In Ecuador the glabrous var. aequatoriana is most likely to be confused with *Ipomoea dumetorum* but lacks the distinctive dark glands on the sepals which are characteristic of that species. Molecular studies suggest var. aequatoriana is sister to *Ipomoea meyeri*. It is possible that var. aequatoriana and the typical variety represent different species but the lack of material renders it impossible to make an informed decision.

• Species 312–327. The Quamoclit Clade. Annual or perennial twining herbs, usually rather slender in habit; stem and leaves glabrous or thinly pubescent. Leaves variable in form, ovate, entire, 3-lobed, palmately divided, or pinnate. Flowers in pedunculate cymes (never solitary), bird-pollinated; bracteoles very small; sepals usually unequal, with a prominent subterminal abaxial awn (absent in *I. quamoclit*); corolla always glabrous, hypocrateriform or suburceolate, always with a relatively long, subcylindrical tube and a spreading limb which may be entire, deeply lobed or much reduced; anthers usually exserted but sometimes (*I. rubriflora*) held at the mouth of the corolla. Ovary and capsule 4-locular and 4-seeded; seeds tomentellous, the hairs equal or unequal in length but long marginal hairs are always absent.

Species 321 to 327 are superficially very similar and have often been treated in the past as *Ipomoea coccinea* L. All are slender annual twining herbs with hypocrateriform red flowers and exserted or near exserted stamens. Several species are very similar, differing only in one or two characters (*I. cholulensis* and *I. indivisa*, for example), so diagnostis descriptions only are provided in several cases so that species can be distinguished as clearly as possible.

This clade has long been accepted as a distinct group. Its most distinct features are the presence of an awn on the abaxial surface of the sepals and the 4-locular ovary.

**Table:** 

1	Leaves pinnatidifid, usually bearing pseudostipules	**312. *I. quamoclit***
–	Leaves entire, bifid or palmately lobed; pseudostipules absent	**2**
2	Corolla suburceolate, the limb reduced to five short teeth	**3**
–	Corolla not as above, the limb prominent, entire or lobed, not reduced to five short teeth	**4**
3	Corolla red, yellow or orange; sepal awns 3–4 mm long; secondary peduncles c. 12 mm long	**319. *I. lobata***
–	Corolla maroon; sepal awns 5–6 mm long; secondary peduncles to 25 mm long	**320. *I. gloverae***
4	Corolla limb deeply lobed	**5**
–	Corolla limb unlobed, at most undulate	**8**
5	Leaves palmately lobed to the base into 5–9 lobes	**313. *I. fissifolia***
–	Leaves entire or shallowly lobed into 3(–5) lobes	**6**
6	Leaves 3-lobed; corolla yellow, red or yellow with purple markings	**7**
–	Leaves entire, sagittate; corolla red	**318. *I. spectata***
7	Corolla yellow with purple markings, lobes 10–15 mm long	**314. *I. neei***
–	Corolla yellow or red, lobes 5–6 mm long	**317. *I. lutea***
8	Inflorescence corymbose, long-pedunculate, peduncles at least 10 cm long, usually much more	**9**
–	Inflorescence cymose, the peduncles usually < 10 cm long but occasionally to 20 cm	**10**
9	Corolla limb 3–4 cm diam.	**315. *I. funis***
–	Corolla limb < 2.5 cm diam	**316. *I. hastigera***
10	Leaves entire	**11**
–	Leaves 3–5-lobed	**18**
11	Inner sepals very short, usually < 3 mm; Capsule always muticous	**321. *I. hederifolia***
–	Inner sepals 4–6 mm long; Capsule rostrate, the style persistent	**12**
12	Ovary and Capsule usually pubescent; awns on sepals 4–8 mm long; fruiting pedicels usually erect (Peru and Ecuador)	**325. *I. dubia***
–	Ovary and capsule glabrous; awns on sepals 2–6 mm long; fruiting pedicels deflexed or (in *I. rubriflora*) erect	**13**
13	Fruiting pedicels erect (Andean Argentina and Bolivia)	**323. *I. rubriflora***
–	Fruiting pedicels deflexed	**14**
14	Desert species of Mexico and United States Southwest; slender herb	**322. *I. cristulata***
–	Plants of other areas and habitat; plant relatively robust	**15**
15	Leaves glabrous adaxially; sepal awns 2.5–6 mm long (United States)	**327. *I. coccinea***
–	Leaves glabrous to hirsute adaxially; sepal awns 2–3.5 mm long	**16**
16	Seeds with hairs of different lengths, distributed unevenly over the seed; leaves ovate, usually glabrous (southern South America, usually below1000 m)	**324. *I. indivisa***
–	Seeds uniformly tomentellous; leaves ovate to lanceolate usually pubescent (Ecuador and Venezuela north to Mexico, above 700 m)	**325. *I. cholulensis***
17	Style persistent on capsule; inner sepals 4–6 mm long	**19**
–	Capsule muticous; inner sepals 2–3(–4) mm long	**321. *I. hederifolia***
18	Fruiting pedicel erect (Andean Argentina and Bolivia)	**323. *I. rubriflora***
–	Fruiting pedicels reflexed (Mexico and United States)	**322. *I. cristulata***

### 312. Ipomoea quamoclit

Taxon classificationPlantaeSolanalesConvolvulaceae

L., Sp. Pl., 1: 159. 1753. (Linnaeus 1753: 159)

Convolvulus pennatifolius Salisb., Prodr. Stirp. Chap. Allerton 124. 1796. (Salisbury 1796: 124), nom. illeg. superfl. Type. Based on Ipomoea quamoclit L.
Convolvulus quamoclit (L.) Spreng., Syst. Veg. 1: 591. 1825 [pub. 1824]. (Sprengel 1824: 591).
Quamoclit vulgaris Choisy, Mem. Soc. Phys. Genève 6: 52 [434]. 1834. (Choisy 1834: 52 [434]). Type. Based on Ipomoea quamoclit L.
Ipomoea cyamoclita St.-Lag., Ann. Soc. Bot. Lyon 7(1): 128. 1880, (Saint-Lager 1880: 128), nom. illeg. superfl. Type. Based on Ipomoea quamoclit L.
Quamoclit quamoclit (L.) Britton, Ill. Fl. N. U.S. 3: 22. 1898. (Britton and Brown 1898: 22). (Saint-Lager 1880: 128).
Convolvulus pinnatus Desr. in Lamarck, Encycl., 3: 567. 1789 [pub.1792]. (Desrousseaux 1792 567). Type. Cultivated plant from East Indies (lectotype P00357495, designated here).
Quamoclit pinnata (Desr.) Bojer, Hort. Maurit. 224.1837. (Bojer 1837: 224).
Ipomoea erecta Michx, J. Hist. Nat. 1: 410. 1792. (Michaux 1792: 410). Type. UNITED STATES. Florida. Sine col. (whereabouts unknown).
Quamoclit vulgaris var. albiflora G. Don, Gen. Hist. 4: 260. 1838. (Don 1838: 260). Type. No type specified.

#### Type.

INDIA. Herb. Clifford 66, Ipomoea 1 (BM000558077), designated by Biju 2003: 755).

#### Description.

Twining annual herb, plant completely glabrous. Leaves shortly petiolate, often bearing pseudo-stipules, 1–7(–9) × 0.8–7 cm, ovate-elliptic in outline, deeply pinnatifid to the main vein, the segments linear, acute, mostly 8–15 pairs; petioles 0.5–3(–4.5) cm. Inflorescence of 1(–5)-flowered axillary, pedunculate cymes; peduncles 1–5 (–14) cm; bracteoles elliptic, c. 1 mm long; pedicels 8–20 mm, swollen upwards; sepals slightly unequal, oblong-elliptic, obtuse and very shortly mucronate, the mucro < 1 mm long, margins scarious, the outer 4–6 × 2–3 mm, the inner c. 1 mm longer; corolla usually metallic red, hypocrateriform, the tube 2–3 cm long, widened upwards, the limb c. 2 cm diam., deeply lobed with acute lobes; stamens exserted. Capsules ovoid, 7–9 mm long, rostrate, glabrous; seeds c. 5 mm, hirsute with hairs in patches.

#### Illustration.

Figure 136C; Acevedo-Rodríguez (2005: 176); Austin (1998: 404); Bosser and Heine (2000: 29); Deroin (2001: 239).

#### Distribution.

Widely cultivated and sometimes naturalised throughout the tropics. Most records cited below are of cultivated plants, but it is occasionally naturalised around villages particularly in the humid lowlands. It seems to be most common in the Amazon region, especially in Loreto (Peru) and Amazonas, Para and Mato Grosso in Brazil. It is of an uncertain New World origin but might come from the Amazon region given the existence of apparently natural populations in this region.

**URUGUAY.** fide O’Donell (1959a: 365).

**ARGENTINA.***T.M. Pedersen* 5335 (S). **Misiones**: *G.J. Schwarz* 2200 (LIL). **Salta**: *C. O’Donell* 2592 (LIL). **Tucumán**: *S. Venturi* 322 (LIL).

**PARAGUAY. Amambay**: *J. Solomon et al.* 6989 (MO).

**BRAZIL. Acre**: *C.A. Cid Ferreira & A. Souza* 3010 (NY). **Amazonas**: Lago Tefé, *T.C. Plowman et al.* 12571 (MO, NY). **Bahia**: *Blanchet* s.n. [1831] (BM, NY). **Goiás**: *D. Philcox & Ferreira* 4447 (K, MO, S). **Mato Grosso**: *B. Dubs* 2024 (E, NY, S, Z). **Mato Grosso do Sul**: *E.P. Heringer et al.* 944 (MO, NY). **Minas Gerais**: *G. Prance et al.* 14367 (MO, NY, S). **Pará**: Conceição do Araguaia, *T.C. Plowman* 8762 (MO, NY). **Paraíba**: *L.A. Pereira & E. Chagas* 241 (NY). **Paraná**: *P. Dusen* 11430 (GH, S). **Rio de Janeiro**: *Gardner* s.n. [1837] (BM). **Rondônia**: *M.G. da Silva* 450 (NY). **Santa Catarina**: *A.C. Cervi* 6120 (NY). **Tocantins**: *M.G. da Silva* 3594 (NY).

**FRENCH GUIANA.***P. Sagot* 369 (BM, S).

**SURINAM.***W.R. Hostman* 645 (MO, S).

**GUYANA.***A.S. Hitchcock* 17367 (NY, S).

**BOLIVIA. Beni**: *J. Balderrama* 361 (NY, LPB, USZ). **Cochabamba**: *T.J. Killeen et al*. 3498 (ARIZ, BOLV, LPB, MO, USZ). La Paz: *O. Buchtien* 1478 (US). **Pando**: Suárez, Porvenir, *F. Fernández Casas & A. Susanna* 8352 (LPB, MA, MO, NY). **Santa Cruz**: Santa Rosa de la Roca, *J.R.I. Wood et al*. 27793 (K, LPB, USZ).

**PERU. Cajamarca**: *J. Campos et al. 4111* (MO, OXF, USM). **Cusco**: *C. Vargas* 2481 (CUZ, LIL, MO). **Junín**: *J. Soukup* 2843 (F). **Loreto**: Balsapuerto, *G. Klug* 3115 (BM, F, S); *R. Ferreyra 3351* (LIL, MO); Maynas, Iquitos, *R. Vásquez & N. Jaramillo* 16704 (MO, OXF). **Madre de Dios**: *P. Nuñez & P. Monice* 5364 (MO). **Ucayali**: *J. Schunke & J. Graham* 15099 (MO, USM).

**ECUADOR. Guayas**: *I. Holmgren* 115 (S). **Los Ríos**: *C. Dodson et al.* 13754 (MO); *B. Ståhl & J. Knusen* 1289 (GB). **Napo**: *L.B. Holm-Nielsen et al.* 19773 (AAH, MO). **Sucumbíos**: *E. Freire et al.* 2879 (MO).

**COLOMBIA. Amazonas**: *J. Duque* 2466 (COL). **Antioquia**: *F.J. Roldán et al.* 571 (MO). **Chocó**: *H. León* 551 (COL, MO). **Magdalena**: *H.H. Smith* 1586 (COL, MO). **Meta**: *R. Jaramillo* 310 (COL); *H. Humbert* 27177 (COL, P). **Putumayo**: *G. Klug* 1646 (F, MO, S). **Valle**: Gorgona Island, *J. Aguirre et al.* 300 (BM, MA).

**VENEZUELA. Amazonas**: *A. Gentry & P.E. Berry* 14614 (MO). **Anzoátegui**: *A. Fernández* 13709 (USM). **Bolívar**: *L. Williams* 11225 (VEN). **Carabobo**: *H. Pittier* 8179 (VEN); **Guárico**: *R. Rondeau* 358 (MO). **Nueva Esparta**: Margarita Island, *O.O. Miller & J.R. Johnston* 76 (BM, F, NY).

**PANAMA.***R.E. Woodson & R.W. Scherry* 825 (MO).

**COSTA RICA.** Puntarenas, Puerto Quepos, *Khan et al.* 426 (BM); Puntarenas, *A. Molina* 27407 (BM).

**NICARAGUA.** P.N. Volcán Masaya, *D. Weberbauer* 2899 (BM, MO).

**HONDURAS.** Olancho, Catacamas, *M. Chorley* 221 (BM, MO).

**EL SALVADOR.***J.M. Tucker* 510 (K).

**BELIZE.** Forest Home, *W.A. Schipp****1055*** (BM, K, S), *P.H. Gentle* 3017 (K).

**GUATEMALA.** Petén, *R. Tun Ortíz* 1487 (BM); *P.C. Standley* 23960 (S), 64033 (F).

**MEXICO. Campeche**: *E.F. & H. Cabrera* 14537 (MEXU). **Chiapas**: *E. Martínez & R. Lombera* 26193 (K); *A. Reyes García & M. Sousa* 2059 (BM). **Chihuahua**: *H.S. Gentry* 2434 (K, S). **Est. México & Dist. Fed.**: Temascaltepec, Luvianos, *G.B. Hinton* 5022 (BM, K). **Guerrero**: *G.B. Hinton* 10848 (K). **Jalisco**: *E.J. Lott* 734 (MO). **Michoacán**: *F.R. Barrie & M. Luckow* 1528 (NY). **Narayit**: *Y. Mexia* 972 (BM). **Oaxaca**: *Ghiesbrecht* s.n. (K). **Sinaloa**: Maztlan, *A. Carter & L. Kellogg* 3646 (BM, UC). **Sonora**: *H.S. Gentry* 1059 (S). **Tabasco**: *N. del Rivero* 7 (MO). **Veracruz**: Comaltepec, *G. Martínez Calderón* 1174 (BM). **Yucatán**: *G.F. Gaumer* 1263 (F).

**UNITED STATES. Alabama**: *J.R. MacDonald* 10868 (IBE). **Florida**: *A.H. Curtiss* 2155 (BM, K, S). **Georgia**: *J.B. Walker & C.R. Annable* 1066 (NY), 6009 (K). **Mississippi**: *K. Rogers* 3868 (IBE). **Missouri**: *B. Summers* 9961 (MO). **New Jersey**: *H. Moldenke* 2656 (FSU). **South Carolina**: *Leonard & Radford* 1942 (S).

**BAHAMAS.***J.E. Eckenwalder* 1625 (NY).

**CUBA.***López Figuieras* 684 (HAJB). **Cienfuegos**: Soledad, *A. González* 101 (BM, NY). **Isla de Juventud** [Pinos]: *E.L. Ekman* 11963 (S). **La Habana**: *H. van Hermann* 1125 (NY).

**CAYMAN ISLANDS.***G.R. Proctor* 29367 (BM).

**JAMAICA.***W. Harris* 6985 (BM); *G.R. Proctor* 27666 (BM); *T.G. Yuncker* 18138 (NY)

**HAITI.***E.L. Ekman* H9155 (S)

**DOMINICAN REPUBLIC.***E.J. Valeur* 719 (BM, C, F, K, S); Samaná, *E.L. Ekman* H15367 (K, NY, S); *H.A. Allard* 13215 (MO).

**PUERTO RICO**. *P. Sintenis* 4946 (K); *F.S. Axelrod & A. Comas* 7490 (MO, NY).

**LESSER ANTILLES. US Virgin Islands**: St Croix, *A.E. Ricksecker* 26 (MO, NY). **Netherlands Antilles**: St Eustatius fide Axelrod (2017). **St Kitts**: fide Powell (1979). **Antigua**: *H.E. Box* 1046 (BM). **Montserrat**: *J.A. Shafer* s.n. [7/1/1907] (NY). **Martinique**: *Williams* 32 (BM); *A. Duss* 1887 (MO, NY). **Dominica**: *C. Whitefoord* 4035 (BM). **Guadeloupe**: *A. Duss* 2473 (NY). **St Lucia**: fide Powell (1979). **St Vincent**: *L. Guilding* s.n. (K). **Grenada**: fide Powell (1979). **Barbados**: *E.G.B. Gooding* 188.

**TRINIDAD.***Dale* s.n. (K). **Tobago**: *G.S. Meyer* 32 (K).

**HAWAII.** Maui, *H. St John* 24741 (K).

#### Note.

A unique species because of its pinnate leaves. The pseudo-stipules and vermilion flowers are also unusual.

#### Typification.

In designating a lectotype for *Convolvulus pinnatus* we have chosen the most complete of the two specimens in the Lamarck herbarium.

### 312 × 327. Ipomoea × multifida

Taxon classificationPlantaeSolanalesConvolvulaceae

(Raf.) Shinners, Sida 2: 265. 1966. (Shinners 1966: 265)

Quamoclita [Quamoctita] multifida Raf., New Fl. 4: 57. 1838 (Rafinesque 1838b: 57).
Quamoclit sloteri House in Bailey, Gentes Herbarum; Occasional Papers on the Kinds of Plants 1(3): 128, f. 60. 1923. (Bailey 1923: 128). Type. Cultivated plant, Thorburn seed 67440 (holotype consists of two sheets BH000128400 and BH000128401).
Ipomoea × sloteri (House) Ooststr., Fl. Males., Ser. 1, Spermat. 4: 483. 1953. (Ooststroom 1953: 483).

#### Type.

A cultivated plant ex Herb. Collins (not found).

**Diagnosis.** This is the garden hybrid Ipomoea quamoclit × coccinea, which was originally grown as long ago as the 1830s (Rafinesque 1838b). It is known as Cardinal Climber and can be recognised by its deeply palmately-pinnatifid leaves and red flowers resembling *Ipomoea coccinea* more than *I. quamoclit*. It is more vigorous than either of the two parent species.

#### Distribution.

Although Rafinesque suggested it sometimes grew spontaneously, there are no other reports that this hybrid grows outside gardens. The following are records of cultivated plants:

**UNITED STATES. Missouri**: *G. Engelmann* (K). **New York**: Ithaca, *W.J. Dress* 1199 (BM).

#### Note.

The name *Ipomoea × sloteri* is generally used for this hybrid but there seems no reason why the older Ipomoea × multifida should not be adopted.

### 313. Ipomoea fissifolia

Taxon classificationPlantaeSolanalesConvolvulaceae

(McPherson) Eckenw., Brittonia 41: 79. 1989. (Eckenwalder 1989: 79)

Quamoclit fissifolia McPherson, Contr. Univ. Mich. Herb. 14: 97. 1980 (McPherson 1980: 97). Type. MEXICO. Michoacán, west of Aguililla, *R. McVaugh* 24694 (holotype MICH1163198).

#### Type.

Based on *Quamoclit fissifolia* McPherson

#### Description.

Woody liana, 4–6 m long, stems glabrous. Leaves petiolate, 2–14 × 2–14 cm, deeply palmately and subpedately lobed nearly to the base, the lobes usually 5–9, 2–9 × 0.1–1.4 cm, linear to lanceolate, narrowed at both ends, entire, glabrous; petioles 2.8–10 cm. Inflorescence of long-pedunculate, many-flowered axillary cymes; peduncles 20–50 cm long; bracteoles 1–1.5 mm, deltoid, mucronate; secondary and tertiary peduncles 1–1.5 cm; pedicels 10–40 mm; sepals unequal, ovate to suborbicular, obtuse or retuse, carinate, glabrous, outer 2.5–3.5 mm with 1–3 mm long awn, inner 4.5–6 mm with 2–5 mm long awn; corolla, greenish-red, glabrous, hypocrateriform with a curved tube 2.5–3 cm long, the limb c. 3 cm diam., lobed with lobes ovate, 9–12 mm long; stamens and style exserted. Capsules ovoid, 8–10 mm long, glabrous, muticous; seeds shortly pubescent.

#### Illustration.

McPherson (1980: 96).

#### Distribution.

On limestone rocks at 1400–1450 m in central Mexico. Only known from the type.

**MEXICO. Michoacán**: the type collection.

#### Note.

The deeply palmately divided leaves with up to 7 leaflets are very distinct.

### 314. Ipomoea neei

Taxon classificationPlantaeSolanalesConvolvulaceae

(Spreng.) O’Donell, Lilloa 29: 69. 1959 (O’Donell 1959a: 69)

Calboa vitifolia Cav. Ic. 5: 51, tab. 476. 1794 [pub.1799]. (Cavanilles 1799: 51), non Ipomoea vitifoliaBlume (1825–26). Type. MEXICO. Nayarit, San Blas, *Nee*s.n. (lectotyoe MA222539, designated here; isolectotypes MA).
Macrostemma vitifolia (Cav.) Pers., Syn. Pl. 1: 185. 1805. (Persoon 1805: 185).
Quamoclit vitifolia (Cav.) G. Don, Gen. Hist. 4: 259. 1838. (Don 1838: 259).
Convolvulus neei Spreng. Syst. Veg. 1: 593–4. 1825 [pub.1824]. (Sprengel 1824: 593–4). Type. Based on Calboa vitifolia Cav.
Ipomoea peduncularis Bertol., Novi Comment. Acad. Sci. Inst. Bononiensis 4: 408–9. 1840. (Bertoloni 1840: 408–9). Type. GUATEMALA. Escuintla, *J. Velazquez*s.n. (whereabouts not traced).
Ipomoea hartwegii Meisn. in Martius et al., Fl. Brasil. 7: 220. 1869. (Meisner 1869: 220), nom. illeg., non Benth. (1839). Type. GUATEMALA. *K.T. Hartweg* 603 (lectotype K000612755, designated here; isolectotypes BM, K).
Ipomoea acaponetensis M.E. Jones, Contr. W. Bot. 18: 65. 1933. (Jones 1933: 65). Type. MEXICO. Nayarit, Acaponeta, *M.E. Jones* 23247 (holotype POM, now RSA0002385).

#### Type.

Based on *Calboa vitifolia* Cav.

#### Description.

Somewhat woody climber (less commonly trailing), stems glabrous. Leaves petiolate, 4–14 × 3.5–13, palmately 3-lobed, lobes acute to acuminate, base deeply cordate with right-angled sinus, lateral lobes often with some marginal teeth, moth surfaces nearly glabrous but veins pubescent especially near the base beneath; petioles 2.5–11 cm. Inflorescence usually very long-pedunculate, corymbose, many-branched with 10–70 flowers; peduncles 0.5–40 cm; bracteoles 1–2 mm, ovate, caducous; secondary peduncles 1–1.5 cm, tertiary to ultimate peduncles1–2.5 cm ; pedicels 8–20 mm, slender; sepals, unequal, outer 2.5–4 mm, ovate, obtuse, the mucro 2–5 mm, often spreading, inner 4–4.5 mm, the mucro up to 5 mm long; corolla 2.5–3.5 cm long, tube 1–2.4 cm, widened above a cylindrical base, the limb deeply lobed 1–1.5 cm, the lobes oblong-lanceolate, yellow or yellow with purple markings in throat, glabrous, anthers and style strongly exserted. Capsules ovoid, c. 9 × 8 mm, glabrous, erect, muticous; seeds irregularly hirsute.

#### Illustration.

Figures 8C, 153.

**Figure 153.**
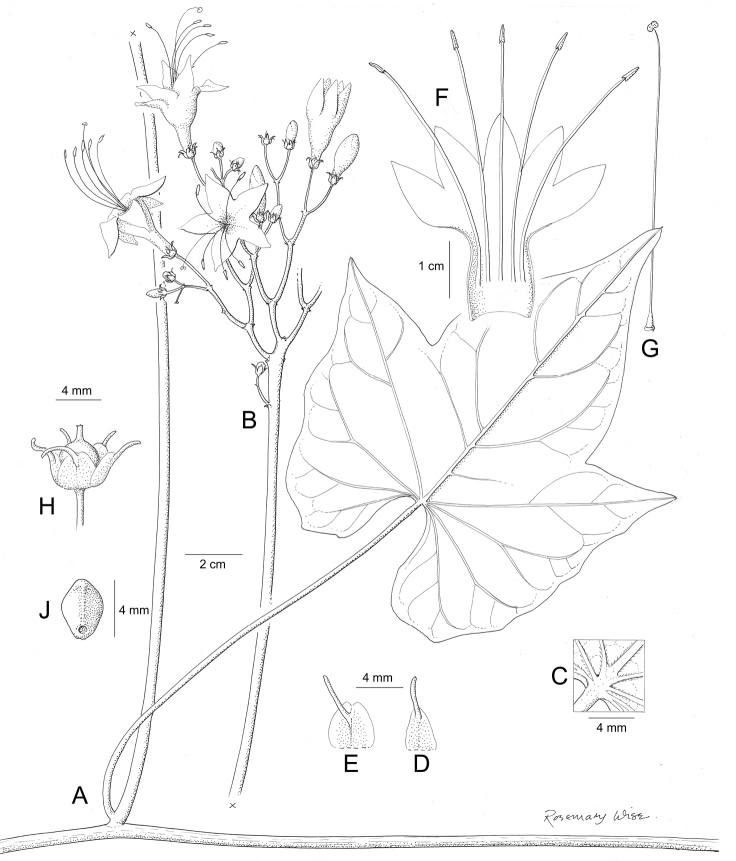
*Ipomoea neei*. **A** habit **B** inflorescence from same habit **C** abaxial leaf surface **D** outer sepal **E** inner sepal **F** corolla opened out to show stamens **G** ovary and style **H** capsule and calyx **J** seed. Drawn by Rosemary Wise **A–C** from *Skutch* 2043; **D–H** from *Burnham* 132; **J** from *Tripp et al*. 5757.

#### Distribution.

River margins, swampy areas and similar moist scrub at low altitudes from Panama north to central Mexico.

**PANAMA.***B.C. Seemann* (BM); Chiriqui, *W.H. Lewis et al.* 410 (MO).

**COSTA RICA.** Tucurriques, *A. Tonduz* 12972 (BM, K), 2237 (BM); San José, Mora, El Rodeo, *A. Cascante* 1242 (CR, K).

**NICARAGUA.** Madriz, Cerro El Fraile, *P. Moreno* 23511 (BM, MO); Chontales, Hac. San Martín, *W.D. Stevens* 22865 (BM, MO).

**HONDURAS.***J. Hjalmarson* (S); Copán Ruinas, *A. Molina & A.R. Molina* 34252 (MO).

**EL SALVADOR.***V. Hartman* (S); Lago Ilopango, *K. Sidwell et al.* 570 (BM, MO).

**GUATEMALA.** Santa Rosa, *Heyde & Lux* 4349 (BM, K); Quezaltenango, *A.F. Skutch* 2043 (BM); Aceituna, *J. Donnell Smith* 1874 (K).

**MEXICO. Chiapas**: El Chichon, *Burnham* 132 (BM); Ocosingo, *E. Martínez & R. Lombera* 26191 (K); *E. Tripp et al*. 5757 (COLO, OXF). **Colima**: *E. Palmer* 1104 (BM, K); Rancho el Jabalí, *L. Rico & E. Martínez* 990 (K). **Durango**: San Dimas, *M. González* 2404 (IEB). **Est. México & Dist. Fed.**: Temascaltepec, Luvianos, *G.B. Hinton* 3199 (BM, K), ibid., Tenaya, *G.B. Hinton* 3320 (BM, K). **Guerrero**: Montes de Oca, Vallecitos, *G.B. Hinton* 11723 (K); Río de Santiago, Galeana, *G.B. Hinton* 11196 (K); Arcelia, *V.W. Steinmann & J.M. Porter* 839 (IEB). **Jalisco**: San Sebastián, *Y. Mexia* 1790 (BM, MO); Zacoalco de Torres, *J.A. Lomelí* (IEB); Lago La María, *A.C. Sanders et al.* 10694 (K, MO). **Michoacán**: Coalcomán, Aguila, *G.B. Hinton* 15846 (K); Aguililla, *E. Carranza et al. 6679* (IEB). **Nayarit**: Tepic-Puerto Vallarta, *R. Ramírez & G. Flores* 863 (MEXU, MO). **Oaxaca**: Santa María Chimalapa, *H. Hernández* 958 (MO). **Sinaloa**: Sierra Tacuicamona, *H.S. Gentry* 5578a (MEXU, MO). **Tabasco**: Teapa, *M.A. Margaña et al*. 1016 (MO). **Veracruz**: Sanborn, *C.R. Orcutt* 3034 (BM, MO); Catemaco, *G. Martínez Calderón* 1836 (BM, MEXU, MO).

#### Typification.

In choosing a lectotype of *Ipomoea hartwegii* Meisn., we have selected the Kew specimen as the most complete of the existing syntypes. It is not clear where the specimen Meisner used to prepare the protologue was housed.

### 315. Ipomoea funis

Taxon classificationPlantaeSolanalesConvolvulaceae

Schldt. & Cham., Linnea 5: 118. 1830. (Schlechtendal and Chamisso 1830: 118)

Morenoa grandiflora La Llave in La Llave & Lex., Nov. Veg. Descr. Fasc. 1: 17. 1824. (La Llave and Lexarza 1824: 17), non Ipomoea grandiflora (L.f.) Lam. (1791). Type. MEXICO. San José del Corral, sine col. (wherabouts unknown).
Quamoclit grandiflora (La Llave) G. Don, Gen. Hist. 4: 259. 1838. (Don 1838: 259).
Ipomoea llaveana Meisn. in Martius et al., Fl. Brasil. 7: 219. 1869. (Meisner 1869: 219). Type. Based on Morenoa grandiflora La Llave
Quamoclit langlassei House, Bull. Torrey Bot. Club. 36; 597. 1909. (House 1909b; 597). Type. MEXICO. probably Guerrero, *E. Langlassé* 875 (holotype US00111497, isotypes GH, K, P).
Ipomoea funis var. langlassei (House) O’Donell, Lilloa 29: 41. 1959 (O’Donell 1959a: 41).

#### Type.

MEXICO. Veracruz, San Andrés, *Schiede & Deppe* 556 (lectotype HAL98219, designated here; isolectotype NY).

#### Description.

Climbing herb, stems glabrous or pubescent. Leaves ovate, petiolate, 7–12(–17) × 3–8(–13) cm, entire (var. langlassei), undulate, irregularly dentate or 3-lobed, base cordate sometimes with a square sinus, apex finely acuminate, both surfaces thinly pubescent to glabrous; petioles 2.5–15 cm. Inflorescence of long-pedunculate few-flowered cymes; peduncles 10–35 cm; bracteoles 0.5–3 mm, ovate, caducous; secondary to ultimate peduncles 1–1.5 cm; pedicels 7–35 mm; sepals with scarious margins, outer 4–5 × 4 mm, ovate, obtuse or truncate, strongly mucronate, awn 3–9 mm, inner elliptic, slightly longer and broader with a similar awn; corolla 5–6 cm long, red, glabrous, funnel-shaped, basal cylindrical tube 2.5–3 cm, then strongly widened, limb broad, c. 5 cm diam., shallowly lobed, stamens weakly exserted. Capsules globose, glabrous; seeds tomentose with scattered tufts of longer hairs.

#### Illustration.

Figure 3H.

#### Distribution.

Endemic to central Mexico, where it grows in disturbed bushy places, especially in damp gullies and along streams between 1600 and 2300 m.

**MEXICO. Dist. Fed.**: *A. García-M* 4368 (MEXU) – possibly introduced. **Guanajuato**: fide O’Donell (1959a: 40). **Guerrero**: Mina, *G.B. Hinton* 9866 (K); ibid., Fresnos, 9752 (K); Leonardo Bravo, *M. Castro* 153 (IEB); *J.C. Soto* 7496 (MEXU). **Michoacán**: Aguililla, *Y. Ramírez-Amezcua & V. Steinmann* 1222 (ARIZ, IEB); Nuevo San Juan Parangaricutiro, *V.W. Steinmann & J.M. Porter* 3982 (IEB, MEXU); San Miguel, *Leavenworth & Hoogstrahl* 1075 (MO); Cerro Tancítaro, Apo, *R. McVaugh* 24874 (MICH). **Oaxaca**: *H. Galeotti* 1358 (K); Miahuatlán, *T. Croat* 46031 (MO); Santa Cruz Itundujia, *K. Velasco-G. et al.* 2823 (IEB); Sierra de Mihuatlán, *F. Miranda* 8836 (MEXU). **Puebla**: Jardín del Calvario, *G. Arsène* 2339 (BM, K, MO, NY, US). **San Luis de Potosí**: *Verles d’Aoust* 1882 (P) – location seems improbable. **Veracruz**: *M. Botteri* 465 (BM); Orizaba, *E. Bourgeau* 2985 (K, P); *A. Barrera et al.* 331 (MEXU); *L.A. Castillo-Hernández et al.* 342 (MEXU).

#### Typification.

We have designated the specimen at Halle as lectotype of *Ipomoea funis* as it has Schiede’s original label attached to the sheet.

#### Notes.

O’Donell recognised Ipomoea funis var. langlassei for plants recorded from Guerrero which differ from the type in their entire, adaxially nearly glabrous leaves. However, there is much variation overall in this species in leaf shape, lobing and dentation as well as in indumentum, and we do not think this variety merits recognition.

A specimen from Chinicuila (Michoacán) *J.C. Soto Nuñez et al*. 11115 (MEXU) appears to be intermediate with *Ipomoea hastigera*.

### 316. Ipomoea hastigera

Taxon classificationPlantaeSolanalesConvolvulaceae

Kunth, Nov. Gen. Sp. 3: 111. 1818 [pub. 1819]. (Kunth 1819: 111)

Convolvulus hastiger (Kunth) Spreng., Syst. Veg. 1: 605 1825 [pub. 1824]. (Sprengel 1824: 605).
Quamoclit hastigera (Kunth) G. Don, Gen. Hist. 4: 259. 1838 (Don 1838: 259).
Ipomoea humboldtiana Roem. & Schult., Syst. Veg. 4: 789. 1819. (Roemer and Schultes 1819: 789). Type. “CENTRAL AMERICA”, presumably, MEXICO. Humboldt & Bonpland in Herb. Willd. (B-W03765-01), treated as “Ipomoea angularis” in Humboldt mss.
Morenoa globosa La Llave in La Llave & Lex., Nov. Veg. Descr. Fasc. 1: 5. 1824. (La Llave amd Lexarza 1824: 5). Type. MEXICO. [Veracruz], San José del Corral (wherabouts unknown).
Quamoclit globosa (La Llave) G. Don, Gen. Hist. 4: 259. 1838. (Don 1838: 259).
Calboa globosa (La Llave) Lindl., J. Hort. Soc. 5: 82 1850. (Lindley 1850b: 82).
Ipomoea globosa (La Llave) Meisn. in Martius et al., Fl. Brasil. 7: 220. 1869. (Meisner 1869: 220).
Quamoclit lindleyi House, Bull. Torrey Bot. Club. 36; 597. 1909. (House 1909b: 597). Type. Based on Morenoa globosa La Llave
Quamoclit russeliiflora M. Martens & Galeotti, Bull. Acad. Brux. 12, 2: 271. 1845. (Martens and Galeotti 1845: 271). Type. MEXICO. Veracruz, Mirador *H.G. Galeotti* 1354 (holotype BR000006972905, isotype G).
Quamoclit kerberi E. Fourn., Bull. Soc. Bot. France 30: 187. 1883. (Fournier 1883: 187). Type. MEXICO. Veracruz, Cordoba, *E. Kerber* 50 (lectotype P00625548, designated here; islectotypes BM, BR, C, K, MPU, P).
Ipomoea kerberi (E. Fourn.) C. Sprenger, Bull. Soc. Tosc. Ortic. 19: 116. 1894. (Sprenger 1894: 116).

#### Type.

MEXICO. Near Mexico City, *Humboldt & Bonpland* 3993 (P00670771).

#### Description.

Twining, presumably annual herb, stems glabrous or pubescent. Leaves petiolate, 8–10 × 7–12 cm, entire, ovate, or, more commonly, 3-lobed, base shallowly cordate with a broad sinus, lobes acuminate, mucronate, the central lobe narrowed at base, glabrous to pubescent; petioles 5–10 cm. Inflorescence a long-pedunculate corymb, with flowers clustered; peduncles mostly 10–25 cm long, corymbose branches short, mostly < 1 cm; bracteoles 1–2 mm, ovate-deltoid; pedicels 7–20 mm; sepals oblong-ovate, obtuse or truncate, glabrous or pilose, margins scarious, outer 2–3.5 × 2 mm, mucro 4–7 mm, inner slightly larger, 3–4 mm long, the mucro 4.5–7 mm; corolla red, 3–3.5 cm long, tube 2.2–2.5 cm long, subcylindrical and gradually widened from base, limb, 5-angled but not lobed, c. 10–12 mm wide, stamens exserted. Capsules globose, c. 7 mm diam., glabrous, style not persistent; seeds 3.5–5 mm long. tomentellous.

#### Illustration.

Figure 154.

**Figure 154.**
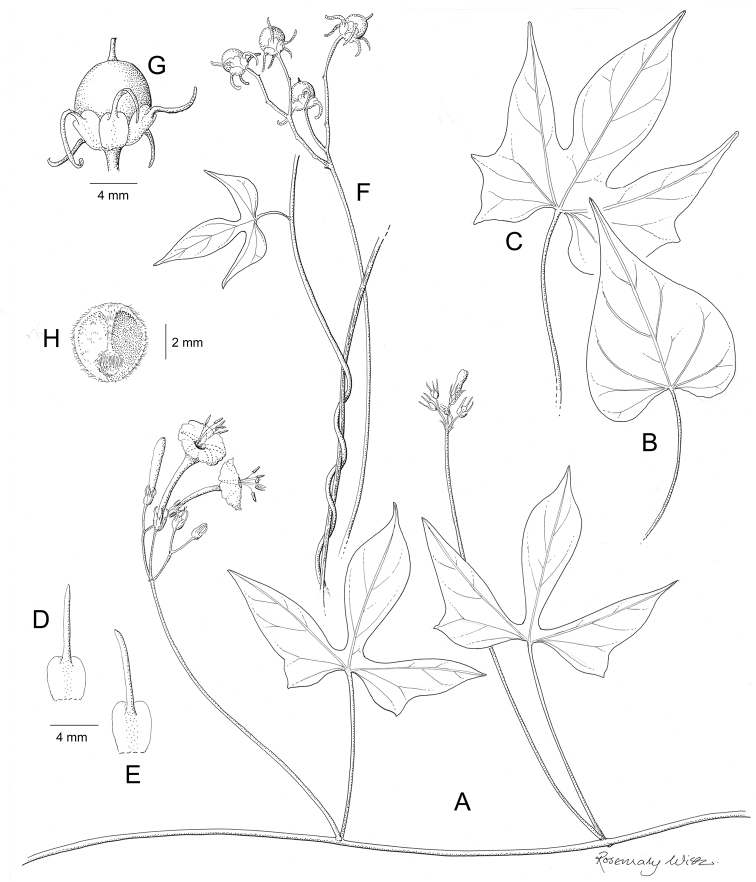
*Ipomoea hastigera*. **A** habit with flowers **B** entire leaf **C** lobed leaf **D** outer sepal **E** inner sepal **F** fruiting inflorescence **G** capsule **H** seed. Drawn by Rosemary Wise **A, C–G** from *Calonico et al.* 21293; **B** from *Rosas* 939.

#### Distribution.

Endemic to south-central Mexico mostly between 700 and 1800 m, so often at lower altitudes than *Ipomoea funis*. It grows in disturbed deciduous woodland, often near streams.

**MEXICO. Chiapas**: Cintalapa, *A. Reyes García et al.* 1499 (BM, MEXU); Tzimol, *A. Reyes García* 1041 (BM, MEXU); Mapastepec, Reserva El Triunfo, *R.J. Hampshire et al.* 606 (BM). **Guerrero**: Galeano, Pie de la Cuesta, *G.B. & J.C. Hinton* 11061 (K, MO); Cruz de Ocote, *E. Martínez & F. Barrie* 5697 (MEXU); Mun. Azueta, *J. C. Soto Nuñez* 11544 (MEXU). **Jalisco**: Rincón de Mananatlán, *M. Cházaro & J.A. Vásquez* 8665 (MEXU). **Michoacán**: Coalcomán, *G.B. & J.C. Hinton* 12704 (MO). **Oaxaca**: Choapan, *Y. Mexia* 9253 (K, MO, S); *M. Ghiesbreght* s.n. (K, P). **Puebla**: Tuxamapan de Galeana, *S. Hernández & J.L. Contreras* 657 (MEXU). **Veracruz**: Zacuapan, *C.A. Purpus* 6317 (BM, MO); Córdoba, *E. Bourgeau* 1727 (K, P, S); Atoyac, *E. Kerber* 159 (BM, K); Puente de San Miguel, *M. Rosas* 939 (BM); Ixtaczoquiatlán, *M. Nee* 23866 (BM, F).

### 317. Ipomoea lutea

Taxon classificationPlantaeSolanalesConvolvulaceae

Hemsley, Diagn. Pl. Nov. 34. 1879. (Hemsley 1878–80: 34)

Quamoclit lutea (Hemsley) Hallier f., Bot. Jahrb. 16: 537. 1894 [pub.1893]. (Hallier 1893a: 537).
Ipomoea lutea forma rubra O’Donell, Lilloa 29: 67. 1959. (O’Donell 1959a: 67). Type. MEXICO. Chiapas, Chicharras, *E.W. Nelson* 3768 (holotype GH, isotype US).

#### Type.

GUATEMALA. *O. Salvin & C. Godman* s.n. (holotype K000612779).

**Diagnosis.** Very similar to *I. hastigera* but reaching 5 m, the corolla yellow, orange or red, 4–5 cm long, the tube 3.5–4 cm, the limb distinctly lobed to 4–6 mm.

#### Distribution.

Endemic to woodland between 700–1500 m in the extreme south of Mexico and Guatemala.

**GUATEMALA.** Quezaltenango, *T. Croat & Hannon* 63459 (MO, FTG); Sololá, *H. Förther* 10241 (BM); Sacatepeguez, *Hunnewell* 14879 (GH).

**MEXICO. Chiapas**: Unión Juárez, Córdoba, *E. Ventura & E. López* 1283 (BM); Kulaktik, Tenejapa, *A. Méndez Ton* 5560 (IEB, MO). **Oaxaca**: Ixtlán de Juárez, *J.C. Flores et al.* 054 (IEB, MO). **Tabasco**: Huimanguillo, *J. Calónico Soto* 21293 (BM, IEB).

### 318. Ipomoea spectata

Taxon classificationPlantaeSolanalesConvolvulaceae

McDonald, Harvard Pap. Bot. 4: 49. 1993. (McDonald 1993b: 49)

Quamoclit coccinea var. jaliscana House, Bull. Torrey Bot. Club 36. 601. 1909. (House 1909b: 601). Type. MEXICO. Jalisco, between San Sebastián and summit of Mount Bufa de Mascota, *E.W. Nelson* 4094 (holotype US00111496).
Ipomoea hastigera var. jaliscana (House) O’Donell, Lilloa 29: 45, 1959. (O’Donell 1959a: 45).

#### Type.

Based on Quamoclit coccinea var. jaliscana House

**Diagnosis.** Distinguished from *I. hastigera* by the sagittate or hastate leaves, extended inflorescence with secondary peduncles 2–12 mm long, outer sepals 5–6 × 3–5 mm, the corolla tube 2.7–3 cm, the limb deeply lobed, 2.5–3 cm diam. Capsules at least sometimes with a persistent style.

#### Distribution.

Endemic to central Mexico occurring in and near the Sierra Manantlán at around 1500–2000 m where it grows seasonally moist pine and oak woodland.

**MEXICO. Jalisco**: San Sebastián, *Y. Mexia* 1439 (BM, F, MO, US); *H.H. Iltis et al.* 1291 (WIS); *J. Villa et al.* 116 (MICH); *O. Téllez et al.* 13763 (MEXU). **Michoacán**: Coalcomán, *G.B. Hinton* 12729 (K); Villa Victoria, *E. Carranza & I. Silva* 7087 (IEB). **Nayarit**: Tepic, Cerro San Juan, *O. Téllez et al.* 12380 (IEB, MO); ibid., *G. Flórez & O. Ramírez* 2357 (MO); ibid., *G. Flórez & R. Ruenes* 1943 (IEB).

### 319. Ipomoea lobata

Taxon classificationPlantaeSolanalesConvolvulaceae

(Cerv.) Thell., Viertel, Nat. Ges. Zurich 64: 775. 1919. (Thellung 1919: 775)

Mina lobata Cerv., Nov. Veg. Descrip. 3. 1824. (Cervantes 1824: 3). Type. Cultivated plant from Mexico (holotype G, n.v.).
Quamoclit lobata (Cerv.) House, Bull. Torrey Bot. Club 36: 602. 1909. (House 1909b: 602).
Quamoclit mina G. Don, Gen. Hist. 4: 259, 1838. (Don 1838: 259). Type. Based on Mina lobata Cerv.
Ipomoea mina (G. Don) Voss., Vilm. Blumengärtn., ed. 3: 710. 1895. (Voss 1894–6: 710)
Ipomoea versicolor Meisn. in Martius et al., Fl. Brasil. 7: 220. 1869, (Meisner 1869: 220), nom. superfl. Type. Based on Mina lobata Cerv.
Convolvulus mina (G. Don) Kuntze, Revis. Gen. Pl. 3: 215. 1898. (Kuntze 1898: 215).

#### Type.

Based on *Mina lobata* Cerv.

#### Description.

Annual twining herb, stem usually glabrous. Leaves petiolate, 3–12 × 2.5–10, ovate or, more commonly 3-lobed to about half way, base cordate with rounded auricles, apex shortly acuminate, obtuse and mucronate, near glabrous but sometimes puberulent on the veins beneath, abaxially paler; petioles 2–5 cm. Inflorescence of long-pedunculate axillary cymes appearing to form an elongate bifurcate secund raceme; peduncles (5–)10–16(–30)cm; rhachis above branching point, (2–)8–12 cm; bracteoles 1–2 mm, linear-lanceolate, moderately persistent; pedicels slender, 2–6 mm, longer below; sepals dissimilar, glabrous or, occasionally, thinly pilose, outer oblong-ovate, 2–3 × 1.5 mm with terminal awn 2–4 mm long, inner sepals with broader base, elliptic, 3–3.5 × 2 mm and awn 2–4 mm long; corolla tubular, curved, suburceolate,1.8–2.5 cm long, yellow, red or orange, limb formed of 5 small tooth-like lobes; stamens strongly exserted; style exserted. Capsules subglobose, 7 mm diam., glabrous; seeds 4 mm long, pubescent with hairs in patches.

#### Illustration.

Figure 136B; Deroin (2001: 161) as *Mina lobata*.

#### Distribution.

This species is probably of Mexican origin but is widely cultivated and occasionally naturalised in the Americas. It is perhaps native in deciduous forest in south-central Mexico in and near the state of Guerrero. The following citations mostly represent cultivated plants–it is rarely naturalised.

**BRAZIL.** Reported from São Paulo, Rio de Janeiro, Minas Gerais and Pará in Flora do Brasil 2020.

**BOLIVIA. La Paz**: Prov. Nor Yungas, Coroico, pie de Uchumachi, *S.G. Beck* 29599 (LPB).

**PERU. La Libertad**: Pacasmayo, *H.O. Forbes* (BM).

**COLOMBIA. Cundinamarca**: *H. Garcia* 10978 (COL).

**VENEZUELA. Mérida**: *L.E. Ruiz-Terán* 1115 (MO).

**HONDURAS.***P.C. Standley* 13347 (F).

**GUATEMALA.***J. Steyermark* 52164 (F).

**MEXICO. Chiapas**: Esquintla, Monte Ovando, *T. Croat* 47530 (MO). **Est. México & Dist. Fed.**: Temascaltepec, *G.B. Hinton* 5070 (K, S); ibid., Ixtapan, ibid., *G.B. Hinton* 2248 (K); ibid., Platanal, *G.B. Hinton* 7092 (BM, K); ibid., Ixtapan, *G.B. Hinton* 2248 (BM, K); Tejupilco, *V.W. Steinmann et al.* 4136 (IEB). **Guerrero**: W of Suriana, *Y. Mexia* 8807 (F, K, MO, S, US); Mun. de Iguala y Buenavista. Cañón de La Mano, entre Los Amates y El Naranjo, *C. Catalán et al*. 439 (MO); Amatitlán, *R. Cruz Duran & M.E. García* 459. **Michoacán**: Tacupa, Huetamo, *G.B. Hinton* 5631 (BM, K); Zitacuaro, *G.B. Hinton* 13258 (K); Morelia, *G. Arsène* 3277 (K, P); Huacana, *V.W. Steinmann & E. Carranza* 3150 (IEB). **Oaxaca**: *C.L. Smith* 900 (MO). **Puebla**: *Father Nicolas* s.n. (P). **San Luis Potosí**: *J.G. Schaffner* 111 (K), 355 (BM, NY). **Veracruz**: Orizabi, *M. Botteri* 954 (K).

**UNITED STATES. North Carolina**: *J.W. Hardin & A. Russell* s.n. (NCSC). **Utah**: *M.B. Piep* 13087 (UTC).

#### Note.

Quite unlike other species of *Ipomoea* except *I. gloverae* on account of its raceme-like inflorescence combined with aristate sepals and tubular corolla, the limb replaced with five small teeth.

### 320. Ipomoea gloverae

Taxon classificationPlantaeSolanalesConvolvulaceae

J.A. McDonald, Harvard Pap. Bot. 4: 51. 1993. (McDonald 1993b: 51)

#### Type.

MEXICO. Michoacán, 12 km W. of Aguililla on road to Dos Aguas, *F. Barrie, T.P. Ramamoorthy & E. Martínez* 568 (holotype TEX00372567, isotype MEXU).

#### Description.

Twining herb 5–6 m high, stems sparsely pilose. Leaves petiolate, 4–12.5 × 4–11.5 cm, ovate or shallowly 3-lobed, acuminate, base cordate with rounded auricles, glabrous or pilose on the veins; petioles 4–9 cm, pilose. Inflorescence a compound, long-pedunculate axillary raceme with 5–10 flowers; peduncles 21–28 cm, pilose; bracteoles 2–2.5 × 1.5 mm, ovate, apparently caducous; secondary peduncles 0.5–2.5 cm, diminishing in length upwards; pedicels 3–6 mm, glabrous except for a few hairs at base; sepals subequal, 2–2.5 × 1.5 mm, ovate or elliptic, obtuse, but with an awn 5–6 mm long; corolla 2–2.5 cm long, maroon, glabrous, curved above a short basal cylindrical tube, suburceolate, limb reduced to 5 small teeth c. 1 mm long; stamens and style strongly exserted. Capsules and seeds unknown.

#### Illustration.

McDonald (1993b: 50).

#### Distribution.

On roadsides around 1200 m in Michoacán in the same general area as *Ipomoea fissifolia*.

**MEXICO. Michoacán**: Aguililla, *J. González et al.* 412 (IEB); ibid., *E. Carranza & I. Silva* 6665 (IEB)

#### Note.

The corolla is suburceolate resembling that of *Ipomoea lobata* but is maroon in colour and the inflorescence is secund, not cymose as stated in the protologue. It is distinguished by the corolla colour, much longer secondary peduncles to 25 mm (not 12 mm) and longer sepal awns.

### 321. Ipomoea hederifolia

Taxon classificationPlantaeSolanalesConvolvulaceae

L., Syst. Nat., ed. 10, 2: 925. 1759. (Linnaeus 1759: 925)

Quamoclit hederifolia (L.) G. Don, Gen. Syst. 4: 259. 1838. (Don 1838: 259).
Ipomoea coccinea var. hederifolia (L.) A. Gray, Syn. Fl. N. Amer. 2: 209. 1878. (Gray 1878: 209).
Mina hederifolia (L.) Bello, Apuntes Fl. Puerto Rico 1: 294. 1881. (Bello y Espinoza 1881: 294).
Convolvulus coccineus var. hederifolius (L.) Kuntze, Revis. Gen. Pl. 3: 213. 1898. (Kuntze 1898: 213).
Quamoclit coccinea var. hederifolia (L.) House, Bull. Torrey Bot. Club 36: 599. 1909. (House 1909b: 599).
Ipomoea luteola Jacq., Collectanea 2: 266. 1789 [dated 1788]. (Jacquin 1789: 266). Type. Icon 35 in Jacquin, Icones plantarum rariorum 1, drawn from cultivated plant grown from seed from Guatemala, lectotype, designated here (Jacquin 1781–1786).
Convolvulus luteolus (Jacq.) Spreng., Syst. Veg. (Sprengel) 1: 599. 1825 [pub. 1824). (Sprengel 1824: 599).
Quamoclit luteola (Jacq.) G. Don, Gen. Hist. 4: 258. 1838. (Don 1838: 258).
Quamoclit coccinea var. luteola (Jacq.) Choisy in A.P. de Candolle, Prodr. 9: 335. 1845. (Choisy 1845: 335).
Ipomoea coccinea var. luteola (Jacq.) Meisn. in Martius et al., Fl. Brasil. 7: 218. 1869. (Meisner 1869: 218).
Ipomoea coccinea forma luteola (Jacq.) Voss, Vilm. Blumengärtn., ed. 3, 1: 709. 1894. (Voss 1894–6: 709).
Ipomoea angulata Lam., Encycl. 1: 464. 1793 [dated 1791]. (Lamarck 1793 464). Type. MAURITIUS [Ins. Franciae], *Sonnerat* (lectotype P-LAM00357492, designated here).
Convolvulus angulatus (Lam.) Spreng., Syst. Veg. (Sprengel): 1: 594. 1825 [pub. 1824 [[(Sprengel 1824: 594).
Quamoclit angulata (Lam.) Bojer, Hortus Maurit. 224. 1837. (Bojer 1837: 224).
Ipomoea acutangula Ruiz & Pav., Fl. Peruv. 2: 10, t.119. 1799. (Ruiz and Pavón 1799: 10). Type. PERU. *Ruiz, Pavón & Dombey*s.n. (lectotype MA814698, designated here; isolectotypes MA).
Convolulus acutangulus (Ruiz & Pav.) Spreng., Syst. Veg. (Sprengel): 1: 605. 1825 [pub. 1824). (Sprengel 1824: 605).
Quamoclit acutangula (Ruiz & Pav.) Choisy in A.P. de Candolle, Prodr. 9: 335. 1845. (Choisy 1845: 335).
Ipomoea sanguinea Vahl, Symb. Bot. 3: 33. 1794. (Vahl 1794: 33). Type. U.S. VIRGIN ISLANDS. St. Croix, R. West s.n. (lectotype C10009670, designated here; isolectotypes BM, C, MA).
Convolulus sanguineus (Vahl) Spreng., Syst. Veg. (Sprengel): 1: 595. 1824 [pub. 1825). (Sprengel 1824: 595).
Doxema sanguinea (Vahl) Raf., Fl. Tellur. 4: 75. 1836 [pub. 1838]. (Rafinesque 1838a: 75).
Quamoclit sanguinea (Vahl) G. Don, Gen. Hist. 4: 259. 1838. (Don 1838: 259).
Ipomoea angularis Willd., Ges. Naturf. Freunde Berlin Neue Schriften 4: 197. 1803. Type. INDIA. Rottler s.n. (holotype B-W03747-01).
Ipomoea dichotoma Kunth, Nov. Gen. Sp. 3: 112. 1818 [pub. 1819]. (Kunth 1819: 112). Type. [COLOMBIA], Regno Novae Granatae, ad ostia fluminis Sinu, locis humidis, Humboldt & Bonpland 1372 (holotype P00670775).
Quamoclit dichotoma (Kunth) G. Don, Gen. Hist. 4: 259. 1838. (Don 1838: 259).
Ipomoea phoenicea Roxb., Fl. Indica 2: 92. 1824. (Roxburgh 1824: 92). Type. INDIA. Plant cultivated at Calcutta, *Roxburgh* in *Wallich* 1372 (lectotype K-W001112944, designated here).
Convolvulus phoeniceus (Roxb.) Spreng., Syst. Veg. (Sprengel): 1: 596. 1825 [pub. 1824). (Sprengel 1824: 596).
Quamoclit phoenicea (Roxb.) Choisy, Mem. Soc. Phys. Geneve 6: 433 [51]. 1834. (Choisy 1834: 433[51]).
Ipomoea coccinea var. curviflora Griseb., Fl. Brit. W. I. 472. 1864 [pub. 1862). (Grisebach 1862b: 472). Type. JAMAICA. *March*s.n. (?? GOET, not at K).
Ipomoea nephrophylla Meisn. in Martius et al., Fl. Brasil. 7: 219. 1869. (Meisner 1869: 219). Type. ECUADOR. Guayas, Cerrito near Guayaquil, Jameson 395 (lectotype BM001209581, designated here).
Quamoclit brevipedicellata Hallier f., Bull. Herb. Boiss.7: 416. 1899. (Hallier 1899c: 416). Type. GUATEMALA. Grenada, Friedrichstahl 929 (W) & Huehuehtenango, Seler 3204 (?B), syntypes.
Ipomoea brevipedicellata (Hallier f.) Hallier f., Meded. Rijks-Herb. 46: 20. 1922 (Hallier 1922: 20).
Ipomoea praematura Eckenwalder, Brittonia 41(1): 75. 1989. (Eckenwalder 1989: 75). Type. Cultivated plant grown at Toronto from seed collected in Grenada, *J.E. Eckenwalder* 2525 (holotype TRT, isotypes GH, K, MO, NY, US).
Ipomoea coccinea auct. mult., non L.

#### Type.

Icon in Plumier in Burman, Pl. Amer. 4: 82, t. 93, f. 2 (1756), lectotype designated by O’Donell (1959a: 48).

#### Description.

Twining annual, stems glabrous or thinly pilose. Leaves petiolate, 2–12 × 2–11 cm, variable in shape, most commonly 3-lobed to about half way, sometimes very shallowly lobed so leaf coarsely 3–5-dentate, sometimes simply ovate, apex acute or obtuse, mucronate, base cordate with obtuse auricles, glabrous to thinly pubescent; petioles mostly 1–6 cm. Inflorescence of pedunculate, axillary cymes; peduncles 5–15 cm long; bracteoles ovate, c. 1 mm, caducous; secondary peduncles 1–2.5 cm; pedicels 3–12 mm, remaining erect in fruit; sepals slightly unequal, oblong-elliptic, obtuse to rounded with a prominent awn, margins scarious, glabrous, outer sepals 1.5–3 mm with mucro mostly 2–5 mm long, inner slightly larger with broader scarious margins; corolla red, hypocrateriform, usually curved, glabrous, the tube 2–4 cm long, slightly widened upwards, limb 1.8–2.5 cm diam., very shallowly lobed to entire, weakly spreading, acute; stamens exserted. Capsules 5–7 mm, subglobose, lacking an apical mucro, glabrous; seeds 3–4 mm long, shortly tomentose.

#### Illustration.

Figure 10F; 136D; Proctor (2012: 546); Acevedo-Rodríguez (2005: 168); Bosser and Heine (2000: 53); Deroin (2001: 199).

#### Distribution.

Common in tropical America from the southern United States to northernmost Argentina; introduced but widespread and frequent in the Old World tropics. It is usually found in disturbed bushy places and secondary scrub below 1000 m (rarely reaching 1500 m). It is more strictly tropical than many widespread species being absent from the three southern states of Brazil, Uruguay and most of Paraguay as well as most of northern Mexico. It is also rare in the Venezuelan Llanos, the Guianas and the Amazon region and there are no records from Pando in Bolivia, Amazonas in Colombia or Acre and Amapá in Brazil, indicating that it tends to avoid the Amazon basin.

**ARGENTINA. Salta**: Oran, *T. Meyer* 8372 (LIL), fide O’Donell (1959a).

**PARAGUAY. Amambay**: *Rojas in Hassler* 10544 (BM, S); *Fernández Casas & J. Molero* 6196 (MO, NY).

**BRAZIL. Bahia**: *C. von Glocker* 597 (BM, NY, US); Espigão Mestre, *W.R. Anderson et al.* 36954 (MO, NY); Feira de Santana, *L.P. de Queiroz* 15975 (HUEFS, OXF). **Ceará**: *A. Löfgren* 522 (S). **Dist. Fed.**: *V.F. Paiva* 576 (RB). **Espirito Santo**: *A.C. Brade* 18439 (HB, RB). **Goiás**: *B. Walter* 1408 (CEN, RB); *H.S. Irwin* 15077 (NY). **Maranhão**: *G. Eiten* 4046 (NY, RB). **Mato Grosso**: *C.A.M. Lindman* 3411 (S); *L.M. Carreira* 814 (NY). **Mato Grosso do Sul**: *D. Smith* 49 (K). **Minas Gerais**: Viçosa, *Y. Mexia* 4690 (BMS); *A. Macedo* 330 (RB). **Pará**: *R. Spruce* 695 (NY). **Paraíba**: *J. Coelho de Moraes* 917 (RB). **Pernambuco**: Igarassu, *H.C. Silva* 43 (MO). **Piauí**: *Gardner* s.n. (BM). **Rio de Janeiro**: *J.F.Widgren* 148 (S). **Rio Grande do Norte**: *S. Tsugaru* B1218 (NY, MO). **Rondônia**: *W. Thomas et al.* 5023 (MO, NY). **Roraima**: *G.H. Tate* 107 (NY). **São Paulo**: *A. Macedo* 693 (S); *C.W. Mosén* 22 (S).

**FRENCH GUIANA.** Fide G. Léotard (pers. com.).

**GUYANA.***R. Schomburgk* 511 (BM).

**BOLIVIA. Beni**: Est. Biológica del Beni, *E. Villanueva et al.* 859 (F, LPB). **Chuquisaca**: Calvo, Sierra Mandiyapecua, *E. Saravia et al.* 11740 (LPB). **La Paz**: Inquisivi, Cahuata-Miguillas, *T. Ortuño et al.* 346 (K, LPB); Nor Yungas, *J. Solomon et al.* 18973 (MO, K, LPB, USZ). **Santa Cruz**: Germán Busch, Serranía de Mutún, *D. Villarroel et al.* 2053 (USZ); Ibañez, *A. Fuentes* 393 (BOLV, LPB, MO, NY, USZ); Velasco, Bajo Paraguá, *T.J. Killeen & J. Wellens* 6274 (ARIZ, BOLV, LPB, NY, MO, USZ). **Tarija**: Gran Chaco, west of Villamontes, *A. Krapovickas & A. Schinini* 39175 (CTES, LPB).

**PERU. Amazonas**: *R. Ferreyra* 13337 (MO). **Cajamarca**: *F. Woytkowski* 6863 (MO). **Cusco**: La Convención, *Y. Mexia* 8041 (BM). **Junín**: *A. Gentry & G.T. Prance* 16405 (MO). **Lambayeque**: *A. Gentry et al.* 22600 (MO). **Loreto**: *M. Rimachi* 10498 (MO, USM). **Pasco**: Oxapampa, Chontabamba, *R. Rojas et al.* 2337 (MO). **San Martín**: Chazuta, Río Huallaga, *Klug* 4021 (BM); *F. Woytkowski* 35037 (MO, S); *G. Klug* 3442 (MO, S).

**ECUADOR. El Oro**: *G. Harling & L. Andersson* 13416 (MO). **Guayas**: Guayaquil, *Pavón* s.n. (BM); *C. E. Cerón et al.* 19963 (MO). **Imbabura**: *L.B. Holm-Nielsen & J. Jaramillo* 28916 (MO). **Loja**: *B. Klitgaard et al.* 466 (AAU). **Manabí**: *C. Cerón et al.* 6742 (MO).

**COLOMBIA. Atlántico**: *A. Dugand* 4032 (COL, US). **Antioquia**: *R. Callejas & A. Echeverri* 11461 (MO). **Bolívar**: A. *Dugand & R. Jaramillo* 2846 (COL, US); *R. Romero C*. 9256 (COL). **Boyacá**: *A.E. Lawrance* 223 (BM, MO). **Caldas**: *G. Lozano* 5967 (COL). **Cesar**: Chimichagua, *O. Rivera-Díaz* 3307 (COL). **La Guajira**: *T. Saravia* 2308 (COL). **Magdalena**: Santa Marta, *H.H. Smith* 1587 (BM, COL, F, GH, MICH, MO, S). **Norte de Santander**: *J. Cuatrecasas* 16264 (COL). **Quindío**: *M.C. Vélez et al.* 551 (COL). **Santander**: *J.L. Fernández* 20863 (COL). **Sucre**: *L.H. Soto & H. Giraldo* 64 (MO). **Valle**: *I. Cabrera* 7023 (MO).

**VENEZUELA. Bolívar**: *B.K. Holst & H. Van de Werff* 2521 (MO). **Dist. Fed.**: Caracas, La Florida, *A.H.G. Alston* 5445 (BM, S). **Guárico**: *G. Davidse* 4192 (MO). **Nueva Esparta**: Margarita Island: *O.O. Miller & J. Johnston* 75 (BM). **Portuguesa**: *F.J. Ortega* 539 (MO). **Sucre**: Peninsula de Paria, *J. Steyermark & M. Rabe* 96440 (MO).

**PANAMA.***T. Croat* 12911 (MO); *Hunter & Allen* 9 (S); Alhajuela, *H. Pittier* 2341 (BM); *J. A. Duke 6063* (E, MO).

**COSTA RICA.** Guanacaste, *U. Chavarria* 1098 (BM, MO); Puntarenas, *M.H. Grayum & B. Hammel* 9564 (BM, MO); *H. Pittier* 13670 (K); Nicoya, *A. Tonduz* 13670 (BM).

**NICARAGUA.** Managua, *W.D. Stevens* 5354 (BM, MO); peninsula de Coseguina, *S. Marshall* 6621 (BM, F).

**EL SALVADOR.** Ahuachapán, *J.M. Rosales* 1764 (BM, LAGU); *P.C. Standley* 19569 (US).

**HONDURAS**. Tiger Island, *G.W. Barclay* 2560 (BM); *A. Molina* 718 (F).

**BELIZE.** Cayo, *C. Whitefoord* 2201 (BM); *H.H. Bartlett* 369 (US, MO, MEXU, F); Corozal, *P. H. Gentle* 839 (MO).

**GUATEMALA.** Petén, *R. Tun Ortiz* 662 (BM, F), ibid., 526 (BM, F); Lago Petén Itzá, *B. Wallnöfer* 9536 (K, W).

**MEXICO. Baja California Sur**: Rancho Palmilla, *A. Carter & F. Chisaki* 3598 (BM, UC); La Junta, *I.L. Wiggins* 15386 (CAS, K). **Campeche**: Kalkiní-El Remate, *M. Peña-Chocarro et al*. 590 (BM); Calakmul, *E. Martínez et al.* 31473 (BM, MEXU). **Chiapas**: Acacoyagua, *E. Matuda* 17398 (K). **Est. México & Dist. Fed.**: Temascaltepec, *G.B. Hinton* 1759 (BM, K). **Guerrero**: Coyuca, *G.B. Hinton* 6875 (BM, K); Acapulco, *W. Hancock* 32 (K). **Jalisco**: *M.G. Ayala* 983 (K, MEXU); Ajijic, *Harker & Mellowes* 1 (BM). **Michoacán**: Huetamo, *G.B. Hinton* 7114 (BM, K); Coalcomán, *G.B. Hinton*12333 (K); Churumuco, *K.B. Hernández & L. Sánchez* 74 (K). **Nayarit**: Yxtlan del Rio, *Y. Mejia* 748 (BM, MO). **Oaxaca**: *C. Conzatti* 4433 (US). **Querétaro**: Landa de Matamoros, *L.J. Ramos* 1401 (K). **Quintana Roo**: Pucté, *O. Téllez & E. Cabrera* 1258 (BM, MEXU). **San Luís Potosí**: *M.T. Edwards* 484 (F). **Sinaloa**: *W.G. Wright* 1269 (US). **Sonora**: *E. Palmer* 310 (US). **Tamaulipas**: *R.M. King* 3810 (NY). **Veracruz**: San Miguel, *L. Monroy et al.* 93 (BM). **Yucatán**: Chichankanab, *G.F. Gaumer* 2124 (BM).

**UNITED STATES. Florida**: *J.R. Buckhalter* 12850 (UWFP). **Georgia**: *L.C. Anderson* 3786 (FSU). **Louisiana**: *C.B. Coryell* 21 (LSU). **Mississippi**: *B. Parajuli* 5 (NKU). **North Carolina**: *Kitching* s,n. [1/10/1906] (BM). **Texas**: *S.M. Tracy* 7718 (BM).

**BAHAMAS.***A.R. Northrup* 120 (K); *D.S. Correll et al.* 49560 (NY).

**CUBA.***Bro. Clemente* 6303 (HAJB); *López Figueras* 439 (HAJB), 686 (HAJB). **Cienfuegos**: Soledad, *J.G. Jack* 6571 (A, K, P). **Holguín**: Sierra de Nipe, *E.L. Ekman* 10705 (BM, K, S).

**CAYMAN ISLANDS.***M. Brunt* 1688 (BM).

**JAMAICA.***G.R. Proctor* 20563 (BM), 15885 (BM); *T.G. Yuncker* 17210 (S); *L. Wynter* 747 (K); *C.R. Orcutt* 3428 (K, UC, US).

**HAITI.***E.L. Ekman* H2093 (S), 9091 (S); *L.R. Holdridge* 1814 (NY).

**DOMINICAN REPUBLIC.***M. Fuertes* 1360 (BM, K, S); *W. Greuter & R. Rankin* 24912 (B, K, S); *E.L. Ekman* H11155 (K, S).

**PUERTO RICO.***F. Axelrod & P. Chávez* 7315 (K); *D.E. Atha & T. Zanoni* 794 (NY).

**LESSER ANTILLES. U.S. Virgin Islands**: St Croix, *F.R. Fosberg* 59368 (BM, US). **St Kitts**: *G.R. Proctor* 18483 (BM). **Antigua**: *H.E. Box* 1266 (BM, US). **Montserrat**: *J.A. Shafer* 132 (NY, US). **Guadeloupe**: *A. Duss* 2477 (NY, US). **Dominica**: fide Powell (1979). **Martinique**: fide Powell (1979). **St Lucia**: *R.A. Howard et al.* 19984 (BM). **St Vincent**: *H.H. & G.W. Smith* 172 (K, NY); Cannuoan Island, *R.A. Howard* 11117 (A, BM, NY). **Grenada**: fide Powell (1979). **Barbados**: fide Powell (1979).

**TRINIDAD.***Baker & Simmonds* 14838 (K); Gasparee Island, *N.L. Britton* 2792 (NY); Pinte Gourde, *N.L. Britton & W.E. Broadway* 2651 (NY).

**HAWAII.** Cultivated fide St John (1973).

#### Typifications.

There appears to be no specimen at W of *Ipomoea luteola*, so we have designated the corresponding plate as the type. This is a yellow-flowered form of this normally red-flowered species.

In designating a lectotype for *Convolvulus angulatus* Lam. we have chosen the most complete of the three specimens in the Lamarck herbarium, the specimen being attributed to Sonnerat.

We have designated the BM specimen of *Jameson* 395 as the lectotype of *Ipomoea nephrophylla* as the specimen at K is *Ipomoea abutiloides*. At some stage labels must have been mixed and this may have happened with other duplicates of this number, which we have not traced.

#### Notes.

A lowland species that can be recognised by the very short sepals and, in fruit, by the erect peduncle and muticous capsule. It has commonly been misidentified as *I. coccinea* L., a species which is endemic to the south east of the United States.

*Ipomoea praematura* was based on a cultivated plant grown in Toronto from seeds collected in Grenada. This seems at best a form of the widespread *I. hederifolia* and is not recognised here. It is distinguished with difficulty from a widespread and variable *I. hederifolia* by the greenish-pink corolla tube, the limb alternating pink and orange and the ovoid, acute capsule with persistent valves but these differences do not seem significant.

### 322. Ipomoea cristulata

Taxon classificationPlantaeSolanalesConvolvulaceae

Hallier f., Meded. Rijks-Herb. 46: 20. 1922. (Hallier 1922: 20)

Quamoclit gracilis Hallier f., Bull. Herb. Boiss. 7: 416. 1899. (Hallier 1899c: 416), nom. illeg., non Ipomoea gracilis R.Brown (1810). Type. MEXICO. *E. Bourgeau* 1061 (lectotype G00418183, designated here; isolectotypes K, P, S).

#### Type.

Based on *Quamoclit gracilis* Hallier f.

#### Distribution.

Slender annual twining herb; stems glabrous or pilose at the nodes. Leaves petiolate, 1.5–10 × 1–7 cm, ovate, 3–5-lobed or, less commonly, entire, base cordate to subtruncate with rounded auricles, apex acute to acuminate, margin irregularly dentate, abaxially glabrous or pubescent; petioles 2–9 cm. Flowers 3–7 in axillary pedunculate cymes; peduncles 3–10 cm; bracteoles 1–2 mm, lanceolate; pedicels 5–14 mm, becoming reflexed in fruit; sepals unequal, oblong, rounded or truncate, outer c. 3 × 2 mm, often adaxially muricate, the subterminal arista 3–5 mm long, inner sepals 4–6 × 3–3.5 mm, the arista c. 3 mm long; corolla hypocrateriform, 2–2.6 cm long, red or orange-red, glabrous; the limb 1–1.5 cm diam.; stamens exserted. Capsules globose, 7–8 mm long; seeds 3.5–5 mm long, ovoid, blackish, tomentellous.

#### Illustration.

Figure 155.

**Figure 155.**
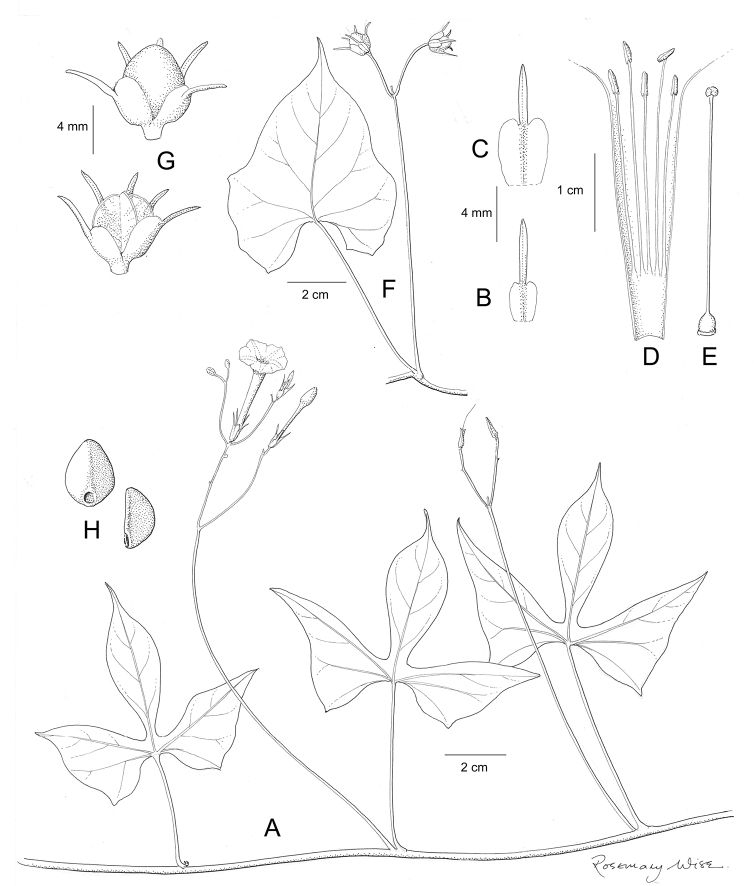
*Ipomoea cristulata*. **A** habit **B** outer sepal **C** inner sepal **D** corolla opened out to show stamens **E** ovary and style **F** fruiting inflorescence **G** capsules **H** seeds. Drawn by Rosemary Wise **A–F** from *Palmer* 103; **G** from *Blumer* 1808; **H** from *Lyonet* 108.

#### Distribution.

Dry, often semi-desert regions of the United States Southwest and northern and central Mexico. It grows in disturbed bushland and similar habitats up to about 2300 m, but appears to be rare below 1500 m.

**MEXICO. Baja California Sur**: Sierra de la Giganta, *A. Carter* 4986 (BM, CAS, MEXU). **Chihuahua**: *E.W. Nelson* 6739 (K); Sierra Canelo, Río Mayo, *H.S. Gentry* 2505 (K, S); Seven Star Mine, *C.H.T. Townsend & C.M. Barber* 382 (BM, K). **Coahuila**: *D. Flyr* 1164b (MO). **Durango**: *C.W. Bollwinkel & R.P. Wunderlin* 155 (MO); Vicente Guerrero, *S. González* 1490 (IEB). **Est. México & Dist. Fed.**: Churubusco, *C.R. Orcutt* 4306 (BM); Pedregal, *E. Lyonnet* 684 (K). **Guanajuato**: *J.N. & J.S. Rose* 11512 (US); Laguna de Yuriria, *S. Zamudio & H. Diáz* 4624 (IEB). **Jalisco**: Tuxpan, *Barnes & Land* 320 (K). **Michoacán**: Morelia, *G. Arsène* 3477 (MO); ibid., *G. Cornejo Tenorio* 2340 (IEB); Morelia-Quiroga, *J.I. Calzada* 8153 (IEB, MEXU). **Querétaro**: Colón, Santa María del Mexicano, *R. Hernández* 11783 (IEB). **San Luis de Potosí**: *C.C. Parry & E. Palmer* 625 (K). **Sinaloa**: La Palmito-El Carrizo, *J.L. Reveal & N.D. Atwood* 3626 (K). **Sonora**: *S.S. White* 2670 (S); Yécora, Río Maycoba, *A.L. Reina-G* 95-456 (ARIZ); Alamos, *Eggli et al.* 1997 (MEXU).

**UNITED STATES. Arizona**: Santa Rita Mts., *Kearney & Peebles* 10563 (K, US): *J. Tedford* 06-504 (ARIZ); Pima, Rincon Peak, *M.A. Baker* 16352 (ARIZ); Chiricahua Mts, *J.C. Blumer* 1808 (K). **New Mexico**: Lovelace Ranch, *F.A. & M.M. Iwen* 151 (BM); Organ Mts., *G.R. Vasey* 344 (BM), *E.O. Wooton* 629 (K); Grant, Mangus Valley, *S. Beckworth* 150 (DES). **Texas**: *C. Wright* 506 (BM); Presidio, Shafter, *A.C. Sanders* 4179 (UCR); Trans Pecos Mountains fide Correll and Johnston (1970).

#### Typifications.

Hallier cited various syntypes following his description of *Quamoclit gracilis*, all those from Berlin apparently destroyed in 1943 so the Bourgeau specimen at G is here selected as lectotype. It is duplicated at K, P and S.

#### Notes.

This species is similar to *Ipomoea hederifolia* and *I. rubriflora* in its morphology but is generally more slender. From *I. hederifolia* it is distinguished by the often muricate outer sepals, the inner sepals 4–6 mm long, the corolla tube generally straight, the narrower limb < 1.5 cm diam. and the style persistent on the capsule; from *I. rubriflora* it can be distinguished by the often reflexed fruiting pedicel.

*Ipomoea cristulata* favours desert conditions and sometimes has stiff, virgate branches as in *Eggli et al.* 1997 (MEXU).

### 323. Ipomoea rubriflora

Taxon classificationPlantaeSolanalesConvolvulaceae

O’Donell, Lilloa 29: 79. 1959. (O’Donell 1959a: 79)

#### Type.

ARGENTINA. Cordoba, Dept. San Alberto, entre Mina Clavero y Nono, *O’Donell & Rodríguez* 708 (holotype LIL, n.v., isotype NY00319220).

#### Description.

Similar to *I. hederifolia* in habit, variability of leaf shape and general features of the inflorescence but more robust, the stems distinctly angled, glabrous except at nodes. Leaves usually glabrous or nearly so; sepals unequal, glabrous or pubescent, outer sepals oblong-obovate, 3–4 × 2–2.5 mm; inner sepals 5–6 × 3–4 mm; corolla limb 2–3 cm diam., stamens very shortly exserted. Capsules strongly rostrate terminating in a persistent mucro 3–5 mm long, the fruiting pedicel erect; seeds tomentose with hairs unequal in length those bordering the central groove longer.

#### Illustration.

**Figure 156.**
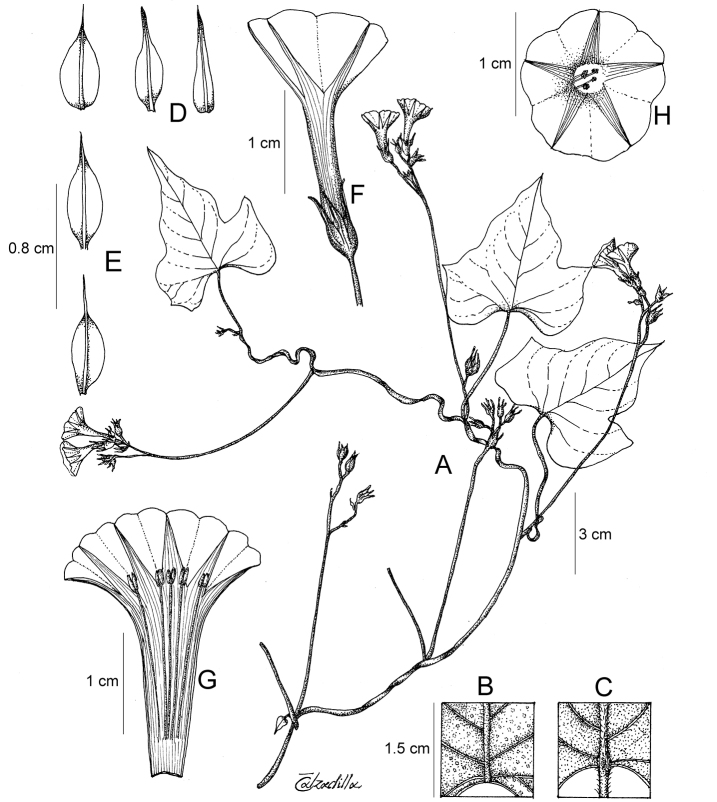
*Ipomoea rubriflora*. **A** habit **B** adaxial leaf surface **C** abaxial leaf surface **D** outer sepals **E** inner sepals, middle at bottom **F** calyx and corolla **G** corolla opened up to show stamens **H** corolla mouth showing position of anthers. Drawn by Eliana Calzadilla **A–C, G, H** from *Wood et al.* 27678; **D–F** from *Wood et al.* 27657.

Figures 8H, 156; O’Donell (1959b: 234).

#### Distribution.

Endemic to scrubby banks in the dry inter-Andean valleys of Bolivia and northern Argentina, mostly growing between 1500 and 2700 m.

**ARGENTINA. Córdoba**: *P.G. Lorentz* 61 (BM. P). **Catamarca**: La Puntilla, *M. Villafane* 1240 (LIL, RB); Ancasti, *J. Brizuela 1048* (LIL, P). **La Rioja**: *Castellamos* s.n. [4/2/140] (LIL). **Jujuy**: Capital, *S. Venturi* 8702 (BM, L, NY, S, SI); Cochinoca, Abra Pampa, *S. Venturi* 9372 (BM, LIL, SI). **Salta**: Cachi, *L.J. Novara* 6066 (G); R. de Lerma, *L.J. Novara* 6296 (G), 7883 (G, S), 9675 (S). **Santiago del Estero**: *Cuezzo* 2441 (LIL). **San Luis**: *A. Vignati* 494 (LIL, LP, NY). **Tucumán**: Cerro del Campo, *S. Venturi* 10438 (S); Tafí, *R. Rocha* 808 (CTES, LIL).

**BOLIVIA. Chuquisaca**: Azurduy, Com. San Pedro, *R. Lozano et al.* 3173 (MO, OXF); Boeto, Nuevo Mundo, *J.R.I. Wood et al.* 27657 (OXF, K, LPB); Oropeza, *M. Cardenas* 575 (NY); Tomina, Padilla, *J.R.I. Wood* 8306 (K, LPB). **Cochabamba**: Capinota, *M. Mercado & A. Haigh* s.n. (K); Mizque, *W.J. Eyerdam* 25205 (F, K). **La Paz**: Sud Yungas, Plazuela-Lambate, *J.R.I. Wood et al.* 29191 (LPB, USZ). **Santa Cruz**: Caballero, Pulquina, Com. Anamal, *M. Garvizu & Muñoz* 1089 (USZ, K); Vallegrande, *L. Arroyo et al.* 5214 (MO, USZ). **Tarija**: Arce, Padcaya, *S.G. Beck et al.* 26166 (LPB); Cercado, *E. Bastian* 827 (LPB, USZ); O’Connor, Serranía Nogal, *M. Serrano & J. Villalobos* 7436 (LPB).

#### Note.

*Ipomoea rubriflora* is most easily distinguished from similar species by the erect, rostrate fruiting capsule combined with the lobed leaves and slightly longer sepals. However, some specimens such as *Hieronymus s.n.*[10/1879] (BM); *D.O. King* 731 (BM) have erect muticous capsules although the sepals are too large for *Ipomoea hederifolia* so presumably belong to *Ipomoea rubriflora*.

### 324. Ipomoea indivisa

Taxon classificationPlantaeSolanalesConvolvulaceae

(Vell.) Hallier f., Meded. Rijks. Herb., Leiden 46: 20. 1922. (Hallier 1922: 20)

Convolvulus indivisus Vell. (Vellozo 1825 [1829]: 71). Type. BRAZIL (lectotype, original parchment plate of Flora Fluminensis in the manuscript section of the Biblioteca Nacional, Rio de Janeiro [cat. no.: mss1198651-050], redesignated here; later published in Vellozo, Fl. Flum. Icon. 2: t. 50. 1827. [pub. 1831], the published plate designated as lectotype by Wood et al. 2015: 115.
Quamoclit indivisa (Vell.) Hallier f., Bot. Jahrb. Syst. 25: 732. 1898. (Hallier 1898d: 732).

#### Type.

Based on *Convolvulus indivisus* Vell.

**Diagnosis.** Very similar to *Ipomoea hederifolia* and *I. rubriflora*, differing from both in always having unlobed leaves which may be either entire or dentate. In habit, indumentum, sepal dimensions and rostrate capsule it is similar to *Ipomoea rubriflora* but in fruit it is easily distinguished by the reflexed fruiting pedicels. Flowering specimens can sometimes be impossible to separate but *Ipomoea rubriflora* usually has 3-lobed leaves, whereas in *I. indivisa* the leaves are always unlobed.

#### Distribution.

Common in southern Brazil and adjacent parts of Argentina and Uruguay but almost absent from the Andean region, being essentially a lowland species. It has possibly been confused with *Ipomoea rubriflora* in some areas of South America.

**URUGUAY.***Gibert* 231 (K); *W.G. Herter* 1835 (MO); *Berro* 1166 (LIL).

**ARGENTINA. Buenos Aires**: La Plata, *Gomez* 65 (CTES). **Entre Ríos**: *T.M. Pedersen* 8205 (K, S), *A. Burkart et al.* 25392 (CTES). **Misiones**: Iguazo, *H. Keller et al.* 1731 (CTES); San Pedro, *Mulgura de Romero et al.* 3144 (CTES).

**BRAZIL. Dist. Fed.**: *E.P. Heringer et al.* 3781 (K). **Espirito Santo**: *A. Stival-Santos* 557 (RB). **Minas Gerais**: *A.F. Regnell* I, 301 (S); *J.F.Widgren* 298 (K, S). **Paraná**: *G. Hatschbach* 26338 (MBM, G), 42763 (CTES, MO); Curitiba, *P. Dusen* 3260 (S), 11441 (S). **Rio de Janeiro**: *O.C. Goés* 260 (RB). **Rio Grande do Sul**: *Palacios-Cuezzo* 2039 (LIL, S); *C.A.M. Lindman* 1263 (S); *A. Krapovickas et al.* 22984 (CTES); *M. Ritter* 395 (F). **Santa Catarina**: *A. Krapovickas & C. Cristóbal* 41976 (ARIZ, CTES); *A. Korte* 6713 (FURB); *L.B. Smith* 11896 (NY). **São Paulo**: *C.L. Mosén* 1496 (P, S); *K. Mizoguchi* 1549 (MO).

**BOLIVIA. La Paz**: Yungas, 1890, *M. Bang* 587 (F, K, NY, GH, RB); *O. Buchtien* 5525 (F, GH, MO, S, US); Guaybillas, *T. Herzog* 162 (S).

#### Notes.

The Bolivian population of this species is disjunct from the main population in southern Brazil and Uruguay, and grows at a higher altitude (to 1400 m). It has not been recollected for almost a hundred years.

*Ipomoea indivisa* is very close to *I. coccinea* and *I. cholulensis* Kunth, both of which also have unlobed leaves and deflexed fruiting pedicels. It is distinguished from both with difficulty by the crests on its seeds which have longer hairs, different from the short tomentose hairs covering most of the rest the seed. *Ipomoea cholulensis* differs additionally in the narrower, usually pubescent leaves. Preliminary molecular studies tend to support *I. coccinea* as a distinct species but do not confirm that *I. indivisa* is distinct from *I. cholulensis*.

### 325. Ipomoea cholulensis

Taxon classificationPlantaeSolanalesConvolvulaceae

Kunth, Nov. Gen. Sp. 3: 112. 1818 [pub. 1819]. (Kunth 1819: 112)

Convolvulus cholulensis (Kunth) Spreng., Syst. Veg. 1: 599. 1825 [pub.1824]. (Sprengel 1824: 599).
Quamoclit cholulensis (Kunth) G. Don, Gen. Hist. 4: 259. 1838. (Don 1838: 259).
Ipomoea coccinea var. pubescens Schltdl. & Cham., Linnaea 5: 118. 1830. (Schlechtendal and Chamisso 1830: 118). Type. MEXICO. Near Jalapa, Hac. de la Laguna, Schiede & Deppes.n. (?HAL, n.v.).
Quamoclit coccinea var. pubescens (Schltdl. & Cham.) G. Don, Gen. Hist. 4: 258. 1838. (Don 1838: 258).
Quamoclit indivisa var. pubescens (Schltdl. & Cham.) Hallier f., Bull. Herb. Boiss. 7: 414. 1899. (Hallier 1899c: 414).
Ipomoea parviflora Sessé & Moc, Fl. Mexic., ed. 1: 42. 1893 (Sessé y Lacasta and Moçiño 1893: 42), nom. illeg., non Ipomoea parvifloraVahl (1794). Type. MEXICO. *Sessé & Moçino* 462 (1630) (lectotype MA00603909, designated here).

#### Type.

MEXICO. Puebla, *Humboldt & Bonpland* s.n. (holotype P00670774).

#### Description.

Twining annual herb, stems glabrous to tomentose. Leaves petiolate, 3–8 × 1.5–3.5 cm, lanceolate, or ovate, acuminate, mucronate, base cordate to sagittate, strongly auriculate, the auricles rounded to acute, sometimes very shallowly bilobed, the margin entire or undulate, abaxially usually pubescent, but sometimes glabrous; petioles 7–35 mm. Inflorescence of lax. few-flowered axillary cymes; peduncles usually long, 6–19 cm, angled, pubescent or glabrous; bracteoles 1–2 mm, ovate, persistent; pedicels 5–13 mm becoming reflexed in fruit; sepals unequal, outer c. 3 mm, oblong or oblong-ovate, rounded with a mucro 1–3 mm long, glabrous but veins muricate on dorsal surface, inner sepals 4–5 mm, elliptic, the mucro 1–2 mm long; corolla 2–2.5 cm long, narrowly hypocrateriform, red, limb 8–10 mm diam., shallowly lobed, stamens exserted. Capsules 6 × 6 mm, compressed-globose, rostrate with 3 mm long persistent style, glabrous; seeds 3 × 2 mm, minutely puberulent, appearing glabrous under a hand lens.

#### Illustration.

Figure 11M.

#### Distribution.

Frequent on mountains from 700 to 2700 m from Ecuador north to southern Mexico.

**ECUADOR. Imbabura**: Cotacachi, *E. Freire et al.* 809 (QAP, QCNE). **Loja**: Jera, 10 km N. of Saraguro, *L. Ellemann* 66985 (AAU); **Pichincha**: Reserva Pululahua, *H. Gavilanes et al.* 167 (QCNE).

**COLOMBIA. Antioquia**: Bello, *W.A. Archer* 125 (MEDEL, MO, US). **Cauca**: El Tambo, *K. von Sneidern* 282 (S); ibid., *J.M. Idrobo* 261 (COL); Popayan, *F.C. Lehmann* 5860 (K). **Cundinamarca**: Ubalá near Bogotá, *J. Triana* 3806 (BM, COL); Sumapaz, *Tracey* 352 (K). **Huila**: San Agustín, *T. Sprague* 306 (K, US); *R. E. Schultes & M. Villarreal* 5294 (MO); *R. Romero* 6645 (COL). **Nariño**: *D. Diáz et al*. 881 (COL). **Norte de Santander**: *J. Cuatrecasas et al.* 12376 (US, F). **Santander**: *L. Uribe-Uribe* 1990 (LIL). **Valle**: La Calera, *J.E. Ramos* 512 (MO).

**VENEZUELA.** Sine data, *Moritz* 46 (BM). **Aragua**: Tovar, *A. Fendler* 933 (K, MO). **Mérida**: *Funcke & Schlim* 112 (BM).

**COSTA RICA.** San José, *Khan, Tebbs & Vickery* 38 (BM); *A. Tonduz* 1571 (F, US).

**NICARAGUA.***P.P. Moreno* 18442 (MO); *W.D. Stevens & A. Grijalva* 15658 (MO).

**HONDURAS.***J. Valerio* 1741 (F, LIL).

**EL SALVADOR.** San Salvador, *M.A. & H. Renderos* 71 (LAGU, MO).

**GUATEMALA.** Cobán, Alta Veracruz, *H. von Türckheim* 304 (BM, K, US): *R.A. Montes* 350 (S); Santa Rosa, *Heyde & Lux* 4025 (BM).

**MEXICO. Baja California Sur**: Sierra de La Giganta, *J.L. León de la Luz* 9842 (IEB). **Chiapas**: Chuchil Ton, Bochil, *D.E. Breedlove* 29305 (MO). **Colima**: *Vazquez & Phillips* 63 (K). **Est. México & Dist. Fed.**: Temascaltepec, Calera, *G.B. Hinton* 2550 (BM, K), ibid., Pungarancho, *G.B. Hinton* 5132 (BM, K, US), ibid., Ypercones, *G.B. Hinton* 5163 (BM, K, US). **Guerrero**: *G.B. Hinton* 11166 (K, LIL, US). **Hidalgo**: Tlanchinol, *I. Luna et al.* 732 (MEXU). **Jalisco**: San Sebastián, *E.W. Nelson* s.n. (K, US). **Michoácan**: Tingambato, *A. Martínez* 482 (IEB). **Morelos**: *H. Fröderström & E. Hultén* 571 (S); *E. & H. de Cabrera* 12242 (MEXU). **Nayarit**: *A. Bourg* 135 (IEB). **Oaxaca**: *R. Torrez & C. Martínez* 12704 (ARIZ, MEXU); Zimatlán, *A. Miranda & O.L. Hernández* 558 (MEXU). **Puebla**: *H. Fröderström & E. Hultén* 1184 (S). **Querétaro**: Jalpan de Serra, *B. Servín* 581 (IEB). **Veracruz**: Orizaba, *M. Botteri* 463 (BM), 558 (K); *E.K. Balls & Gourlay* 5484 (K, US); *Ortiz* 1421 (F); Ojo de Agua, Orizaba, *M. Rosas* 75 (A, K); Coacoatzintla, *R. Arriaga* 2 (MEXU).

#### Typification.

In designating a lectotype for *Ipomoea parviflora* Sessé & Moçiño, we have chosen MA00603909, to which is pasted Sessé and Moçiño’s draft description, in preference to MA603910 or MA603911. This last is *Ipomoea costellata*. All three specimens are incorrectly labelled in Madrid.

#### Notes.

Apparently most common in Colombia and Mexico, this species grows at higher altitudes than its close relative *Ipomoea indivisa* and is rarely found below 1000 m.

*A.L. Gentry* 22600 (MO) from 26 km E of Olmos in Lambeyeque (Peru) appears to be *Ipomoea cholulensis* but the material is very poor and further collections are needed to confirm the presence of *I. cholulensis* in Peru.

### 326. Ipomoea dubia

Taxon classificationPlantaeSolanalesConvolvulaceae

Roem. & Schult., Syst. Veg. 4: 216. 1819. (Roemer and Schultes 1819: 216)

Ipomoea angulata Ortega, Nov. Rar. Pl. Dec. 7–8: 83 1797. (Ortega 1797 –1800: 83), nom. illeg., non Ipomoea angulata Lam. (1793). Type. Plants grown at Madrid from seed sent by Ruiz and Pavón (lectotype OXF00006441, designated here; isolectotype P).
Quamoclit ruiziana G. Don, Gen. Hist. 4: 258. 1838. (Don 1838: 258), nom. illeg., superfl., Type. Based on Ipomoea angulata Ortega

#### Type.

Based on *Ipomoea angulata* Ortega

#### Description.

Prostrate, ascending or erect annual herb; stems glabrous or pubescent. Leaves petiolate, 2–7 × 1.5–5.5 cm, entire (rarely shallowly 3-lobed), ovate, cordate with rounded or angled auricles, apex finely acuminate, mucronate, margin entire or (rarely) undulate, adaxially glabrous, abaxially glabrous or pubescent, especially on the veins; petioles 2–7 cm, glabrous or pubescent. Inflorescence of long-pedunculate, few-flowered axillary cymes; peduncles (1.4–)3–7.5 cm, remaining erect in fruit, glabrous or pubescent; bracteoles 1–3 mm, lanceolate, acuminate; secondary peduncles 5–7 mm; pedicels 2–7 mm, often angled, glabrous or pubescent; sepals unequal, outer ovate with scarious margins, midvein sometimes extended to form a wing, glabrous or puberulent, 4–5 × 2–3 mm, terminating in a mucro 4–8 mm long, inner sepals 5–6 × 3 mm with a mucro 4–8 mm long; corolla 2–2.5 cm long, hypocrateriform, scarlet, glabrous, limb c. 1.5 cm diam., unlobed. Capsules 5–6 × 6–7 mm, compressed globose, rostrate, the persistent style c. 3 mm long, pubescent or glabrous; seeds 4 × 2 mm, distinctly tomentose.

#### Distribution.

Endemic to Peru and Ecuador between 400 and 2700 m, most records from coastal and lower western semi-desert slopes of the Andes in the Lima area.

**PERU. Ancash**: *E. Cerrate et al.* 5180 (MO, USM). **Cajamarca**: Contumaza, *A. Sagástegui & López* 9166 (FTG, MO), 10529 (FTG, MO), 1573 (F). **Ica**: Mun. Yauca del Rosario, *O. Whaley et al.* 460 (K). **La Libertad**: *P. Nuñez et al.* 6265 (CUZ); Trujillo, *A. Sagástegui & Cabanillas* 8743 (TYG, HUT); ibid., *A. Sagástegui & J. Mostacero* 10447 (MO); ibid., Contumaza, El Balconcito, *A. Sagástegui & S. Leiva* 16404 (OXF). **Lambayeque**: *E. Cerrate et al.* 5242 (USM). **Lima**: *S.G.E. Saunders* 855 (K); *C.A. Weatherby* 11320 (K); *A. Gentry et al.* 19912 (FTG, MO); entre Chosica y Surco, *R. Ferreyra* 6938 (MO, USM). **Piurá**: Chililique, Bajo Naranjo, *E. Laure* 5477 (P); Huancabamba, La Beatita, *Llatas Quiroz* 2455 (F). **San Martín**: *Chrostowski* 69-197 (S). **Tumbes**: Zarumilla, Lechugal, *R. Ferreyra* 10659 (MO, USM).

**ECUADOR. Chimbarazo**: Cañon del Río Chanchan, Huigra, *W.H. Camp* 2970 (FTG, S); Huigra, *J.N. & G. Rose* 22298 (NY, US). **Loja**: Sabanilla, *C. Quintana et al.* 2887 (QCA). **Pichincha**: Reserva Pululahua, Canton Quito, *C. E. Cerón* 2258 (MO). **Tungurahua**: *J.E. Madsen* 36442 (AAH).

#### Typification.

There appears to be no syntype at Madrid so we have selected the specimen at OXF as the lectotype as this is a more complete specimen than that at Paris.

#### Note.

Distinct because of the relatively large sepals with long erect awn-like mucros. The capsule is very unusual, being often pubescent. The short, angled or winged pedicels are also noteworthy.

### 327. Ipomoea coccinea

Taxon classificationPlantaeSolanalesConvolvulaceae

L., Sp. Pl. 1: 160. 1753. (Linnaeus 1753: 160)

Quamoclit coccinea (L.) Moench, Methodus 493. 1794. (Moench 1794: 493).
Convolvulus coccineus (L.) Salisb., Prodr. Stirp. Chap. Allerton 126. 1796. (Salisbury 1796: 126).
Neorthosis coccinea (L.) Raf., Fl. Tellur. 4: 75. 1836 [pub. 1838]. (Rafinesque 1838a: 75).
Mina coccinea (L.) Bello, Apuntes fl. Puerto Rico 1: 294. 1881. (Bello y Espinosa 1881: 294).
Convolvulus coccineus var. typicus Kuntze, Rev. Gen. 3(2): 213. 1898. (Kuntze 1898: 213), nom. illeg., superfl.

#### Type.

Herb. Linn. No. 219.3 (LINN), designated by Wijnands (1983: 88).

#### Description.

Annual herb, stems glabrous except on nodes. Leaves petiolate, entire, 5–8 × 3–5.5 cm, ovate to coarsely dentate, acute and mucronate, cordate, usually sagittate with dentate auricles, glabrous except on the veins beneath; petioles 2.5–5.5 cm. Inflorescence of lax, few-flowered cymes; peduncles 1–13 cm; bracteoles 1–3 mm, broadly lanceolate; pedicels 5–15 mm, eventually becoming reflexed in fruit; sepals unequal, outer 3 mm, oblong to elliptic, rounded to obtuse, smooth, the mucro 2–6 mm, the inner c. 5 mm long, oblong, the mucro 2–5 mm; corolla tube 2–2.5 cm long, lobes 0.5–1 cm, virtually undivided, red or red or variegated with yellow, glabrous, stamens exserted. Capsules broadly ovate, muticous or shortly rostrate, c. 7 mm, glabrous; seeds uniformly tomentose.

#### Distribution.

Endemic to southeastern USA, where it grows on waste ground, roadsides, stream sides and in ditches, apparently with a preference for seasonally moist habitats.

**UNITED STATES. Arkansas**: *V. Board* s.n. [2/8/1967] (UARK). **Florida**: *Buckley* s.n. (K). **Georgia**: *C. Dorby* 110 (GA). **Illinois**: *G.H. French* 2154 (K). **Kansas**: *W.H. Horr & R.L. McGregor* E424 (S). **Kentucky**: *R. Peter* s.n. (K); *D.R. & B.K. Windler* 2836 (VSC). **Louisiana**: *Drummond* s.n. (K). **Maryland & Dist. Col.**: *L.C. Wheeler* 5148 (BM, RSA). **Missouri**: *Mackenzie* 1055 (S). **New Jersey**: *W.M. Benner* 9773 (LSU). **North Carolina**: Sandy Creek, N of Gillburg, *H.E. Ahles & R. Leisner* 20404 (UNC, BM); *Rügel* 436 (BM); *R.K. Brummitt* 21959 (E, K). **South Carolina**: *G. Newberry* 16055 (UCSC). **Tennessee**: *A. Armstrong* 594 (KHD). **Texas**: *C. Wright* 511 (K). **Virginia**: *G.W. Ramsey* 493 (BM); *E.K. Balls* 7704 (BM, US). **West Virginia**: *E.L. Morris* 1209 (K).

#### Notes.

The name *Ipomoea coccinea* is still commonly but erroneously used for many different species in this clade.

Some specimens from outside the eastern United States may be correctly named *Ipomoea coccinea*, for example *Martínez* 31473 (BM) from Campeche, Mexico. These merit further investigation.

• Species 328–334 form another well-defined small clade characterised by their palmately (sometimes pedately) lobed leaves and mucronate sepals. Most species are annuals. It is centred on Mexico and, following House (1908b) can be referred to as the Pedatisecta Clade

### 328. Ipomoea costellata

Taxon classificationPlantaeSolanalesConvolvulaceae

Torr., Bot. Mex. Bound. 149. 1859. (Torrey 1859: 149)

Convolvulus digitatus Sessé & Moc., Pl. Nov. Hisp. 24. 1888. (Sessé y Lacasta and Moçiño 1887–90: 24). Type. MEXICO. *Sessé & Moçino* 887 (holotype MA00603823).
Convolvulus pedatus Sessé & Moc., Pl. Nov. Hisp. 24. 1888. (Sessé y Lacasta and Moçiño 1887–90: 24), nom. illeg., non Convolvulus pedatusRoxburgh (1824). Type. MEXICO. *Sessé & Moçiño* 107 (lectotype MA606866, designated here).
Ipomoea painteri House, Muhlenbergia 3: 41. 1907. (House 1907a: 41). Type. MEXICO. Dist. Fed., near Guadelupe, *J.N. Rose & Painter* 6825 (holotype US US00390631).
Ipomoea pusilla Brandegee, Univ. Cal. Publ. Bot. 4: 382. 1913. (Brandegee 1913: 382). Type. MEXICO. Veracruz, C.A. Purpus 6152 (holotype UC149878, isotypes GH, F, P, US).
Ipomoea futilis A. Nelson, Univ. Wyoming Publ. Sci. Bot, 1(3): 65. 1924. (Nelson, A 1924: 65). Type. UNITED STATES. Arizona, Hanson 1016 (holotype RM0002262).
Ipomoea costellata var. edwardsensis O’Kennon & G.L. Nesom, Sida 20: 39. 2002. (O’Kennon and Nesom 2002: 39). Type. UNITED STATES. Texas, Travis County, *B.C. Tharp*s.n. (holotype TEX00026687).

#### Type.

UNITED STATES. Texas, *C. Wright* 505 (lectotype GH00054454, designated by House (1908b: 234), isolectotypes BM, F, GH, K, MO, NY, US).

#### Description.

Slender annual herb, usually branched at base with decumbent or ascending branches, glabrous or with a few scattered hairs; stems to 2 m long but usually much less. Leaves petiolate, small, 0.7–2.5 × 2–3 cm, variably palmatisect with 5–7 separate leaflets, the laterals pedate, leaflets 1–2.7 × 0.1–0.2 cm, linear-oblong, apiculate, glabrous or with a few scattered hairs; petioles 0.2–3 cm. Flowers solitary (rarely paired), axillary, pedunculate; peduncles slender, 1.8– 5 cm, straight; bracteoles 1–2 × 0.25 mm, filiform, scarious-margined; pedicels 7–25 mm; sepals subequal, lanceolate to ovate, acute, mucronate, the mucro c. 1 mm long, glabrous or nearly so, margins scarious, outer 3–5 × 1–2 mm, often muricate along prominent midrib, inner up to 6 × 3 mm; corolla 1–1.2 cm long, funnel-shaped, purplish, glabrous. Capsules 4–5 × 4 mm, globose to ovoid, glabrous, the slender style somewhat persistent; seeds 3–3.5 × 2 mm, black, minutely tomentellous.

#### Illustration.

Figure 141D, 157.

**Figure 157.**
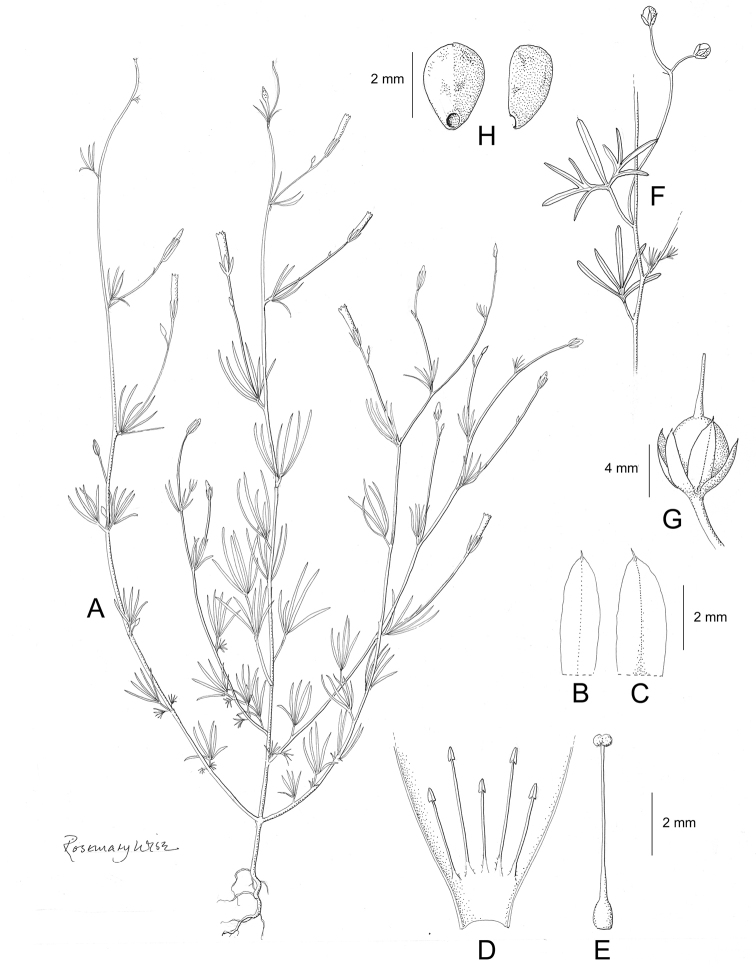
*Ipomoea costellata***A** habit **B** outer sepal **C** inner sepal **D** corolla opened out to show stamens **E** ovary and style **F** fruiting inflorescence **G** capsule **H** seeds. Drawn by Rosemary Wise **A** from *Wooton* 625; **B–E** from *Kearney & Peebles* 10143; **F–H** from *Palmer* 649.

#### Distribution.

Dry scrub and deserts, principally in the United States Southwest and Mexico but of unknown status in Guatemala and apparently naturalised in Venezuela. It is found from low altitudes up to at least 2300 m.

**VENEZUELA. Dist. Fed.**: *H. Pittier* 15137 (VEN) n.v.

**GUATEMALA.***J. Steyermark* 29498 (F), 50738 (F, US).

**MEXICO. Aguascalientes**: Calvillo, *M.C. Provance et al.* 1436 (UCR). **Baja California Sur**: Sierra de La Giganta, Puerto Escondido, *A.M. Carter & R. Moran* 5536 (MO); ibid., Mesa de San Gerónimo, *A. Carter* 5019 (BM, UC). **Chiapas**: Tuxtla Gutiérrez, *D.E. Breedlove* 13875 (F); Mun. Comitan, *A.R. Garcia* 1101 (BM). **Chihuahua**: Sawakoa, Río Mayo, *H.S. Gentry* 2456 (K, S); Seven Star Mine, *C.H.T. Townsend & C.M. Barber* 383 (BM, K, MO, P). **Coahuila**: *E. Palmer* 2095 (K); Ramos Arispe, Sierra de la Paila, *J.A. Villarreal* 4690 (ASU). **Durango**: *E. Palmer* 649 (BM, E, K, MO); Nombre de Dios, *R. Jiménez & S. Acevedo* 111 (IEB). **Est. México & Dist. Fed.**: *J. Rzedowski* 37551 (IEB). **Guanajuato**: Romita, San Francisco de Gavia, *J. Rezedowski* 52424 (IEB). **Hidalgo**: Alfajayucan, *R. Hernández* 6482 (MO). **Jalisco**: Guadalajara, *C.R. Barnes & W.J.G. Land* 124 (K). **Michoacán**: Morelia, *G. Cornejo Tenorio* 2350 (IEB). **Morelos**: Cuernavaca, *Berlandier* 974 (BM). **Oaxaca**: Cerro Juárez, *C. Conzatti* 1957 (MO); Cañon de Tomellin, *C. Conzatti* 2055 (K). **Puebla**: Coxcatlán, *C.A. Purpus* 4215 (BM, MO). **Querétaro**: Ezequiel Montes, Las Rosas, *J. Rzedowski* 53658 (IEB); 3 km W de Las Rosas, *E. Argüelles* 2664 (IEB). **San Luis de Potosí**: fide McDonald (1995). **Sinaloa**: La Noria, *Y. Mexia* 3601/2 (MO). **Sonora**: Cajón de los Guerrijos, *Lumholtz* 430 (K); Sahuipa, *T.R. Van Devender et al*. 2009-800 (ARIZ), El Guayabo, *T.R. Van Devender et al*. 93-1230 (ASU), Mesa Mesiaca, *T.R. Van Devender et al*. 62-1124 (ARIZ, ASU). **Tamaulipas**: Ciudad Victoria-Jaumave, *V.W. Steinmann et al.* 3707 (IEB). **Veracruz**: Baños de Carrizal, *C.A. Purpus* 6152 (BM, CAS, F, GH, MO, US). **Yucatán**: Ruinas de Xtampú, *J.L. Tapia & G. Carnevali* 1120 (ASU). **Zacatecas**: San Juan Capistrano, *J.N. Rose* 2434 (K, US).

**UNITED STATES. Arizona**: *Parker* 8421 (S); Patagonia Mts., *T.H. Kearney & R.H. Peebles* 10143 (K, US); Pima Co., *McManus & McLaughlin* 439 (ARIZ); Gila Co., *J. Ward* 881 (DES); Chiricahua Mts, *J.C. Blumer* 1663 (K). **New Mexico**: Florida Mts., *A.I. Mulford* 1111 (K); Mogollon Mts., *O.B. Metcalfe* 766 (BM, K); Apache Pass, Chiricahua, *J.G. Lemmon* 442 (BM); Organ Mts., *E.O. Wooton* 625 (K). **Texas**: Franklin Mountains, *R.D. Worthington* 17077 (L); Presidio Co., *W.R. Carr* 31818 (NY).

#### Typification.

In selecting a lectotype for *Convolvulus pedatus* we have chosen MA606866 in preference to MA603824 (cited by Nelson, 1997; 393) because it has Sessé and Moçiño’s original manuscript notes attached and these correspond to the protologue in Flora Mexicana.

#### Note.

Var. edwardensis differs in the short peduncle (<2.2 cm) and pure white corolla with ovate, apiculate (not rounded) lobes.

### 329. Ipomoea chamelana

Taxon classificationPlantaeSolanalesConvolvulaceae

J.A. McDonald, Biótica 12(3): 217. 1987. (McDonald 1987a: 217)

#### Type.

MEXICO. Jalisco, La Huerta, Arroyo Colorado, cerca de los Pozos, Est. Biologica Chamela, *E.J. Lott* 729 (holotype MEXU00448375, isotypes MO, US, XAL).

#### Description.

Annual herb with slender twining glabrous stems. Leaves small, palmately divided into 6–10 linear, acute segments, apparently one central lobe and various secondary lobes arising on the two lateral lobes, glabrous. Inflorescence of solitary, axillary, pedunculate flowers; peduncles slender 0.7–2.5 cm; bracteoles 1 mm, deltoid, sessile, scarious; pedicels 6–12 mm, distinctly thicker than the peduncles; sepals slightly unequal, outer 4–5 × 1 mm, lanceolate, finally acuminate and apiculate, muricate along the midrib, glabrous, margins scarious, inner 6 mm long, abaxially smooth, the apex obtuse and apiculate; corolla 1.7–2.5 cm long, subcampanulate, yellow, glabrous, the midpetaline bands ending in a point. Capsules subglobose, glabrous; seeds black, glabrous or minutely puberulent.

#### Illustration.

McDonald (1987a: 218).

#### Distribution.

Endemic to Mexico and apparently restricted to the area around La Huerta in Jalisco at low altitudes.

**MEXICO. Jalisco**: *G. Ayala* 985 (K, MEXU), *Rothschild & Phillips* 058 (K); Los Conejos-Llano Grande, *T.S. Cochrane et al.* 11996 (IEB).

### 330. Ipomoea ramulosa

Taxon classificationPlantaeSolanalesConvolvulaceae

J.R.I. Wood & Scotland sp. nov.

urn:lsid:ipni.org:names:77208080-1

#### Type.

MEXICO. Guerrero. Agua de Obispo, 745 m, 31 Dec. 1965, *Kruse* 964 (Holotype MEXU74987).

**Diagnosis**. Probably related to *Ipomoea costellata* and its allies because of its lobed leaves and aristate sepals but very distinct because of the winged stem, deeply 3-lobed leaves, the much-branched, almost paniculate inflorescence, the white corolla with stamens held at the corolla mouth.

#### Description.

Completely glabrous climbing perennial; stems 5–6 m long, stout, slightly winged, reddish Brown. Leaves petiolate, 3.5–7 × 5–10 cm, 3-lobed, the central lobe broadly to narrowly oblong-elliptic, narrowed at both ends, acuminate, mucronate, the mucro 2 mm long, lateral lobes shallowly lobed or, near base, bilobed, the upper lobe forward-pointing, the lower lobe spreading, base broadly cordate, margin entire, abaxially paler; petioles 4–5.5 cm. Inflorescence of lax, compound, long-pedunculate, axillary cymes; peduncles 11–14 cm; bracteoles 1 mm, deltoid; secondary and subsequent peduncles 3–4.5 cm; pedicels 1–2.3 cm; sepals unequal, oblong-elliptic, terminating in a fine aristate point, the arista 2–3 mm long; outer 13–15 × 6 mm, the inner slightly longer and broader with broad scarious margins; corolla 6–6.5 cm long, funnel-shaped, white with tube yellowish-green inside, glabrous, the stamens held at the corolla mouth; stigma biglobose. Capsules and seeds unknown.

#### Illustration.

Figure 158.

**Figure 158.**
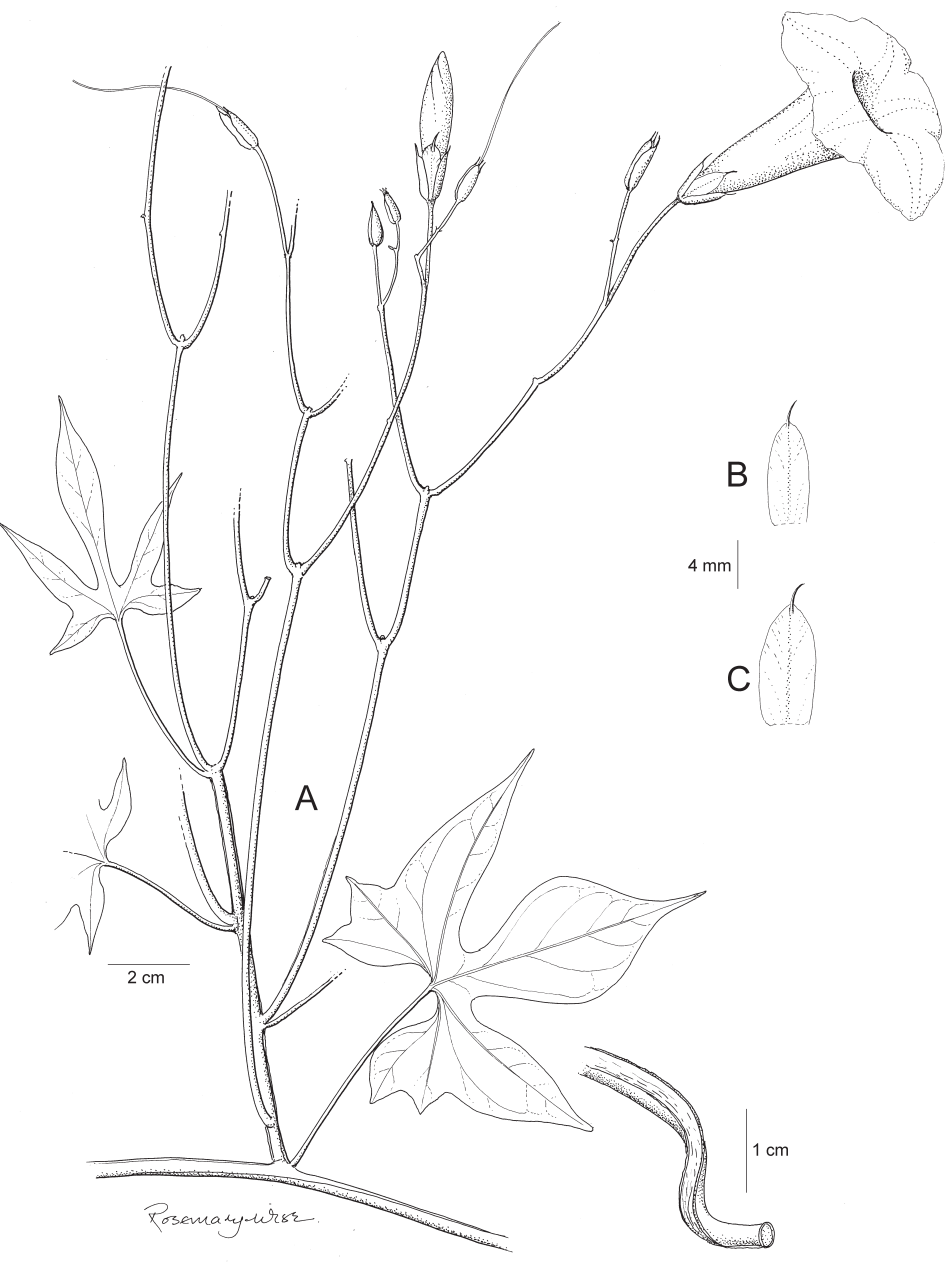
*Ipomoea ramulosa***A** habit **B** outer sepal **C** inner sepal. Drawn by Rosemary Wise from *Kruse* 964.

#### Distribution.

Endemic to Guerrero in Mexico, growing in a damp gully on alluvial soil at 745 m.

**MEXICO. Guerrero.** Type collection.

#### Note.

A very distinctive species because of the winged stem, deeply 3-lobed leaves, much-branched, almost paniculate inflorescence and the aristate sepals. The white flowers are reported to be aromatic. Molecular sequencing using *ITS* suggests a relationship with *Ipomoea costellata* and its allies but there is little superficial morphological similiarity apart from the aristate sepals and trilobed leaves.

### 331. Ipomoea perpartita

Taxon classificationPlantaeSolanalesConvolvulaceae

McPherson, Contr. Univ. Michigan Herb. 14: 94. 1980. (McPherson 1980: 94)

#### Type.

MEXICO. Jalisco, Cabo Corrientes, *R. McVaugh* 26371 (holotype MICH1111343).

#### Description.

Twining herb to 3 m, stems glabrous, woody. Leaves shortly petiolate, 0.5–2 × 0.7–2.5 cm, palmately divided into 5–7 segments, the segments with 1–3 pinnately arranged lateral lobes, all linear, acute, glabrous; petioles short, 2–12 mm, pseudo-stipules present at base. Inflorescence of 1–2-flowered, pedunculate, axillary cymes; peduncles 2–5.5 cm; bracteoles 1–3 mm, lanceolate; pedicels 8–12 mm, noticeably thicker than peduncle; sepals unequal, oblong-ovate, mucronate, glabrous, margins whitish, outermost 3.5–5 × 2.5 mm, inner 6–7.5 mm; corolla 3–4 cm long, funnel-shaped, white with purple tube, glabrous, limb 2.5 cm diam. Capsules glabrous, 6–7 mm long; seeds 3 × 1.5 mm, minutely pubescent.

#### Illustration.

McPherson (1980: 95).

#### Distribution.

Endemic to Jalisco in central Mexico.

**MEXICO. Jalisco**: 18 km W of San Sebastián, *Cochrane et al.* 12053 (A, IEB, WIS); Cabo Corrientes, *E. Carranza et al.* 6133 (IEB).

#### Note.

Like *Ipomoea cairica* and *I. quamoclit*, this species has pseudo-stipules at the base of the petiole. It is also distinguished by the bipinnatisect leaf segments.

### 332. Ipomoea diegoae

Taxon classificationPlantaeSolanalesConvolvulaceae

M.C. Lara, Acta Bot. Mex. 67: 68. 2004. (Lara 2004: 68)

#### Type.

MEXICO. Guerrero, Mun. Iguala de la Independencia, *M. Castro* 40 (holotype FCME, isotypes ENCB, IEB, MEXU).

#### Description.

Climbing or prostrate annual herb to 1 m, from a fibrous root, stem glabrous. Leaves petiolate, deeply palmatisect into 14–16 segments, segments 10–60 × 0.3–2.8 mm, diminishing in size outwards, linear or ensiform, acute and mucronate, glabrous; petioles 0.3–3.6 cm, often with pseudo-stipules arising from the base. Inflorescence of few-flowered, long pedunculate axillary cymes, sometimes reduced to solitary flowers; peduncles 5–13 cm; bracteoles 1–2 mm, lanceolate, persistent; secondary peduncles (if present) 1–1.5 cm; pedicels 13–20 mm; sepals unequal, broadly lanceolate, mucronulate, green with scarious margins glabrous, outer 8–8.5 × 2 mm, inner 9–14 × 3–4 mm, the scarious margins wide; corolla 4.5–7 cm long, funnel-shaped, pink, glabrous, limb 4–6.5 cm diam. Capsules ovoid, 10 × 6 m, glabrous; seeds 4, 5.5 × 2.5 mm, puberulous.

#### Distribution.

Endemic to the Valle de Iguala in Guerrero (Mexico) growing in disturbed areas derived from dry forest between 500 and 1000 m.

**MEXICO. Guerrero**: Iguala, *M. Castro* 179 (IEB, MEXU).

#### Note.

Distinguished from similar species by the relatively long leaf segments and sepals (> 8 mm long). The pink corolla is also distinctive and the division of the leaves into 14–16 segments also serves to distinguish this species.

### 333. Ipomoea sororia

Taxon classificationPlantaeSolanalesConvolvulaceae

D.F. Austin & J.L. Tapia, Sida 19: 807. 2001. (Austin and Tapia-M 2001: 807)

#### Type.

MEXICO. Yucatán, *J.L. Tapia-M & Carnevali* 1120 (holotype CICY047694, isotypes: F, FTG K, MEXU, MO, NY, UCAM, XAL).

#### Description.

Twining annual herb to 2 m, stems glabrous. Leaves petiolate, 2.5–5 × 3–7 cm, pedately 5–7-lobed, lobes entire or basal lobes 1–3 lobed, lobes oblong-lanceolate, acute, glabrous; petioles 2–3 cm. Inflorescence in 1–2-flowered axillary cymes; peduncles 5–3.5 cm; bracteoles 1–2 mm, lanceolate; pedicels 4–5 mm; sepals slightly unequal, outer 5–8 mm, ovate, cordate, with a reflexed lanceolate terminal mucro, abaxially with 3 prominent papillae (soft spines), inner 6–8 mm, ovate, with a 3–4 mm mucro, the margins scarious but abaxial papillae absent; corolla 2–2.5 cm long, white with a lavender throat, funnel-shaped, glabrous, stamens included. Capsules 5 × 4 mm, ovoid, glabrous; seeds unknown.

#### Illustration.

Austin and Tapia-M (2001: 808).

#### Distribution.

Endemic to dry forest bordering mangrove swamp near sea level in southern Mexico.

**MEXICO. Campeche**: 2 km NE of Chiná, *C. Gutiérrez* 6056 (UCAM); Punta Arenas, Tankuche, *F. & H. Cabrera* 15304 (MEXU). **Yucatán**: Hunucmá, *A. Espejo et al.* 1281 (MO).

#### Note.

Similar to *Ipomoea costellata* and *I. ternifolia* but with distinct papillose outer sepals, and also occupying a distinct habitat.

### 334. Ipomoea ternifolia

Taxon classificationPlantaeSolanalesConvolvulaceae

Cav., Icon. 5: 52, tab. 478. 1799. (Cavanilles 1799: 52)

#### Type.

MEXICO. Guerrero, Acapulco, *L. Née* s.n. (holotype MA654733).

#### Description.

Trailing or climbing annual or perennial herb, stems and vegetative parts glabrous or thinly pilose with scattered hairs. Leaves petiolate, 1–8 × 1–6 cm, palmately divided to the base into 5–11 leaflets, the principal leaflets variable in shape, usually oblong-elliptic, acute, narrowed at both ends, the two basal lobes 3-lobed to near base with two lobes smaller, having 7–11 segments in total; petioles 1–3 cm. Inflorescence of 1–3-flowered (often solitary) axillary cymes; peduncles 1–6 cm; bracteoles 1 mm, narrowly deltoid, caducous; pedicels 6–18 mm; sepals subequal, 6–11(–14) × 2–3 mm lanceolate to narrowly elliptic, acuminate to a fine point, bristly-pilose to subglabrous, margins white, scarious; corolla 1.5–4.5 cm long, funnel-shaped, pink, glabrous, limb 3–4 cm diam. Capsules depressed globose, 5–7 mm diam., glabrous, rostrate; seeds dark brown, 3-angled, 2–3 mm, puberulent.

#### Variation.

*Ipomoea ternifolia* is a variable species in habit and in the size and shape of the leaves, sepals and corolla. It is here divided into two geographical subspecies:

### 334a. Ipomoea ternifolia subsp. ternifolia

Taxon classificationPlantaeSolanalesConvolvulaceae

Ipomoea muricata Cav. var. villosa Choisy in A.P. de Candolle, Prodr. 9: 353. 1845. (Choisy 1845: 353). Type. MEXICO. Cuernavaca, Berlandier 974 (lectotype G00135571, designated by Staples and Govaerts in Staples et al. (2015: 221).
Ipomoea ternifolia var. villosa (Choisy) Staples & Govaerts, Phytologia 97: 221. 2015. (Staples et al. 2015: 221).
Convolvulus tenuifolius M. Martens & Galeotti, Bull. Acad. Roy. Sci. Bruxelles 12: 260. 1845. (Martens and Galeotti 1845: 260), nom. illeg., non Convolvulus tenuifoliusVahl (1794). Type. MEXICO. Oaxaca, *H. Galeotti* 1373 (lectotype BR00006992521, designated here; isolectotypes BR, G).
Ipomoea delphiniifolia M. Martens & Galeotti, Bull. Acad. Roy. Sci. Bruxelles 12: 265. 1845. (Martens and Galeotti 1845: 265). Type. MEXICO. [Puebla], *H. Galeotti* 1366 (lectotype BR000006991883, designated here; isolectotypes BR, G, K).
Ipomoea pedatisecta M. Martens & Galeotti, Bull. Acad. Roy. Sci. Bruxelles 12: 265. (Martens and Galeotti 1845: 265). Type. MEXICO. [Oaxaca], *H. Galeotti* 1370 (lectotype BR00006992545, designated here; isolectotypes BR, G, K).
Ipomoea valida House, Muhlenbergia 3: 40. 1907. (House 1907a: 40). Type. MEXICO. [Colima], Manzanillo, *E. Palmer* 1031 (holotype US00390639, isotypes BM, CAS, GH, US).
Ipomoea ternifolia var. valida (House) J.A. McDonald, Harvard Pap. Bot. 6: 122.1995. (McDonald 1995: 122). Type. MEXICO. Colima, Manzanillo, *E. Palmer* 1031 (holotype US00390639, isotypes GH, NY).

#### Diagnosis.

Plants always twining. Longest leaf segments on mature branches < 4 cm long; sepals narrowly elliptic 5–9 mm; corolla 1.5–2.8 cm long. The basal cylindrical part of the corolla tube is usually < 5 mm long but in var. valida, which is only known from the type locality in Colima, the basal cylindrical tube is 16–18 mm long.

#### Illustration.

Figure 7B.

#### Distribution.

Open dry forest in central Mexico extending in scattered locations into Central America. It is found at different altitudes up to 2300 m.

**COSTA RICA.** Guanacaste, P.N. Santa Rosa, *B.E. Hammel & C. Cano* 19575 (CR, MO).

**EL SALVADOR.** Fonseca, *G.W. Barclay* 2602 (BM, NY, US).

**MEXICO. Est. México & Dist. Fed.**: Puerto de Santa Isabel, Sierra de Guadelupe, *E.K. Balls & W.B. Gowlay* 4934 (K); Zacoalco, *E. Bourgeau* 726 (K, P). **Guanajuato**: *E. Hernández et al.* X-2308 (MEXU). **Guerrero**: Acapulco, *E. Palmer* 234 (BM, K); Ajuchitlán del Progreso, *P. Chamu Alonso* 246 (IEB); Copalillo, *Monroy de la Rosa* 164 (IEB). **Jalisco**: Barranca de Tequila, *C.G. Pringle* 4439 (BM, F, GH, K, MO, NY, S, US). **Michoacán**: Huetamo, *P. Tenorio et al*. 1546 (ENCB, MO); Zitácuaro, *G.B. Hinton* 13215 (IEB, K), Tiquicheo, *G.B. Hinton* 13327 (F, GH, K, MO, NY, US); Churumuco, *G. Ibarra* 6619 (K). **Morelos**: *Fröderström & Hultén* 484 (S); Mayotepec-Las Estancas, *J.F. Doebley* 486 (ARIZ). **Nayarit**: SE of Acaponeta, *R. McVaugh* 21753 (NY). **Oaxaca**: Tehuantepec, Puente Zimatán, *S.H. Salas et al.* 3539 (ARIZ), ibid., 4745 (MO); *J.I. Calzada* 24271 (K, MEXU). **Puebla**: *C.A. Purpus* 1281 (F). **Sinaloa**: Sierra Surotato, *H.S. Gentry* 6215 (MEXU, MO). **Querétaro**: Cadereyta de Montes, Las Moras, *H. Diáz & E. Carranza* 7486 (IEB). **Zacatecas**: San Juan Capistrano, *J.N. Rose* 2454 (F, GH).

#### Lectotypification.

Two specimens each of *Convolvulus tenuifolius*, *Ipomoea pedatisecta* and *I. delphiniifolia* are held at BR. The lectotypes chosen are each based on the specimens annotated as holotypes by McDonald as these have corollas.

### 334b. Ipomoea ternifolia subsp. leptotoma

Taxon classificationPlantaeSolanalesConvolvulaceae

(Torr.) J.R.I. Wood & Scotland, comb. & stat. nov.

urn:lsid:ipni.org:names:77208081-1

Ipomoea leptotoma Torr., in Emory, Rep. U.S. Mex. Bound. 2(1): 150. 1859. (Torrey 1859: 150). Type. MEXICO. Sonora, Thurber 977 (lectotype GH00267279, designated by House (1908b: 235), isolectotype NY).
Pharbitis leptotoma (Torr.) Peter Nat. Pflanzenfam. IV (3a): 31. 1891 [dated 1897]. (Peter 1891: 31).
Ipomoea ternifolia var. leptotoma (Torrey) J.A. McDonald, Harvard Pap. Bot. 6: 120. 1995. (McDonald 1995: 120).
Ipomoea radiatifolia Kellogg, Proc. Calif. Acad. Sci. 7: 163. 1876 [pub. 1877]. (Kellogg 1877: 163). Type. [MEXICO. Sonoro], Gulf of California, Ajiabampo, *W.J. Fisher*s.n. (whereabouts unknown).
Ipomoea leptotoma var. wootonii E.H. Kelso, Rhodora 39: 151. 1937. (Kelso 1937: 151). Type. UNITED STATES. Arizona, Santa Rita Mountains, E.O. Wootons.n. (holotype US00390627).
Ipomoea leptotoma forma wootonii (E.H. Kelso) Wiggins, Contr. Dudley Herb. 4: 21. 1950. (Wiggins 1950: 21).
Ipomoea divergens House, Muhlenbergia 3: 40. 1907. (House 1907a: 40). MEXICO. Sonora, Guaymas, *E. Palmer* 231 (holotype US00390633, isotypes C, GH, K, NDG, NY YU).

#### Type.

Based on *Ipomoea leptotoma* Torr.

**Diagnosis**. Plants relatively robust, initially suberect, eventually decumbent or twining. Longest leaf segments on mature branches 4–7 cm long; sepals lanceolate usually 9–14 mm long, rarely less; corolla 2.5–4.5 cm long.

#### Distribution.

Essentially a plant of the Sonora Desert region of Arizona and NW Mexico with a few records from outside this area.

**MEXICO. Baja California Sur**: Bahía de Concepción, *I.L. Wiggins* 11421 (US); *A. Carter* 4969 (MEXU); Cerro de la Giganta, *A. Carter & L. Kellogg* 3136 (BM); Arroyo del Rancho de la Presa, *J.L. León de la Paz* 9807 (IEB). **Chihuahua**: Batopilillas, 2624 (CAS, F, GH, K, MEXU, S, US). **Est. México & Dist. Fed.**: Temascaltepec, Chorrera, *G.B. Hinton* 1822 (K), ibid., Plaza de Gallos 5176 (BM, K), ibid., 8499 (GH, NY); **Guerrero**: Mezcala, *A.A. Monroy* 685 (MEXU); Coyuca, *G.B. Hinton* 5459 (BM, K), ibid., 6471 (BM, K). **Puebla**: Jolalpan, SW of San Pedro Las Palmas, *R. Razo & R. García* 8 (IEB); Jolalpán, *E. Guizar* 1410 (MEXU). **Sinaloa**: Imala, *E. Palmer* 1705 (F, GH, NY, S, US); San Ignacio, *R. Vega & J.A. Gutiérrez* 9415 (MEXU); ibid., *J. González Ortega* 583 (K), *H.S. Gentry* 9483 (CAS, GH, NY); Choix, El Potrerilos, *J. González Ortega* 872 (K). **Sonora**: *I.L. Wiggins & Rollins* 280 (CAS, GH, MO, NY, US); Tecolote road, *F.W. Reichenbacher* 1038 (ARIZ); W of El Sabino, *P. Tenorio et al.* 4609 (IEB, MEXU); Mun. Cucurpe, *T.R. Van Devender* 90-477 (ARIZ); Cajón de los Guerrijos, *C. E. Lloyd* 431 (GH, K); Oputo, *C.V. Hartman* 195 (CAS, GH, K, US).

**UNITED STATES. Arizona**: *J.G. Lemmon* 3039 (BM, CAS, F, US); *Gooding* 2436 (CAS, GH, S); Cochise County (probably), *C. Wright* 1614 (BM, GH, K, MO, NY, US); ibid., *E. Makings* 868 (ASU); Pima Co., *D.F. & S. Austin* 7595 (ARIZ). New Mexico fide Austin (1991b).

### 335. Ipomoea microsepala

Taxon classificationPlantaeSolanalesConvolvulaceae

Benth., Bot. Voy. Sulph. 136. 1844 [pub.1845]. (Bentham 1845: 136)

Ipomoea nelsonii Rose, Contr. U.S. Natl. Herb. 1(9): 343. 1895. (Rose 1895: 343). Type. MEXICO. Colima, Manzanillo, *E. Palmer* 1363 (lectotype US00111426, designated here; isolectotypes BM, GH, K, NY, US).
Ipomoea amplexicaulis Fernald, Bot. Gaz. 20(12): 535. 1895. (Fernald 1895: 535). Type. MEXICO. [Nayarit], Tepic, *F.H. Lamb* 576 (holotype GH00054481, isotypes F, MSC, NY, US).
Ipomoea equitans M.E. Jones, Contr. W. Bot. 15: 149. 1929. (Jones 1929: 149). Type. MEXICO. Nayarit, Tigre Mine near Acaponeta, *M.E. Jones* 23139 (holotype POM, now RSA0002421; isotypes CAS, GH, NY, UC, WIS).

#### Type.

MEXICO. Guerrero, Acapulco, *Sinclair* s.n. (lectotype K000612734, designated here; isolectotypes K).

#### Description.

Climbing herb to 4 m, stems glabrous or thinly pilose, slender or stout and woody. Leaves shortly petiolate, 3–6 × 0.7–3.5 cm, small, ovate, cordate with rounded auricles, obtuse to acuminate, mucronate, usually glabrous; petioles 0.2–1.5 cm. Inflorescence of simple or compound axillary cymes from the leaves and/or in the axils of bracts (resembling reduced leaves) in a many-flowered raceme-like axillary inflorescence up to 20 cm long; peduncles 3–5 cm, usually passing through the sinus of the leaf blade; bracteoles 1–2 mm, linear-lanceolate, deciduous; secondary peduncles 4–6 mm; pedicels 5–8 mm; sepals subequal, 2–2.5 × 1 mm, oblong-lanceolate, acute or obtuse, margins white, glabrous; corolla 2.5–3 cm long, yellow, glabrous, funnel-shaped, limb prominently flared and deeply lobed. Capsules 4–6 mm, globose, glabrous; seeds 2–3 mm, puberulent.

#### Distribution.

Scrub at low altitudes below 1000 m in central and southern Mexico and neighbouring parts of Guatemala.

**GUATEMALA.***Bernoulli & Cario* 1923 (K).

**MEXICO. Chiapas**: Cacahoatán, *D.E. Breedlove* 42584 (CAS, MO). **Colima**: type of *Ipomoea nelsonii*. **Durango**: Chacala, *E.A. Goldman* 339 (BM, US). **Guerrero**: Montes de Oca, *G.B. Hinton* 11658 (GBH, K); Atoyac de Alvarez, *G.B. Hinton* 11002 (GBH, K, MO); Petatlán, *J.C. Soto Nuñez et al.* 12091 (MEXU). **Michoacán**: Huetamo, *G.B. Hinton* 5510 (K); Lázaro Cárdenas, *J.C. Soto Nuñez et al.* 2745 (MEXU). **Oaxaca**: Tuxtepec, *E.W. Nelson* 318 (US); Pochutla, *S.H. Salas et al.* 3638 (MEXU); Tehuantepec, *M. Elorsa* 4285 (MEXU). **Sinaloa**: Sierra Tacuichamona, *H.S. Gentry* 5561 (MEXU, MO); Concordia, *T.R. Van Devender et al.* 2006-192 (MEXU). **Veracruz**: La Lima, *M. Nee* 23777 (BM); Cosamaloapan, *Martínez Calderon* 1323 (BM, F, GH, K, MEXU, MO).

#### Typification.

There are three sheets of the type collection of *Ipomoea microsepala* at K, of which the sheet with bar code 000612734 was annotated as holotype by McDonald. Since there seems no particular reason why this sheet was identified as the holotype in preference to the others, we are here lectotypifying it to remove any uncertainty.

### 336. Ipomoea minutiflora

Taxon classificationPlantaeSolanalesConvolvulaceae

(M. Martens & Galeotti) House, Muhlenbergia 5: 71. 1909. (House 1909a: 71)

Convolvulus minutiflorus M. Martens & Galeotti, Bull. Acad. Roy. Sci. Bruxelles 12: 262. 1845. (Martens and Galeotti 1845: 262). Type. MEXICO. Oaxaca, *H. Galeotti* 1372 (holotype BR0006973018, isotypes BR, G, K, P).
Ipomoea filipes Benth. ex Meisn. in Martius et al., Fl. Brasil. 7: 274. 1869. (Meisner 1869: 274).Type. BRAZIL. Pará, Santarém, *R. Spruce*s.n. (holotype M0185021, probable isotypes K, NY, OXF, TCD).
Ipomoea gracillima Peter, Nat. Pflanzenfam. 4 (3a): 30. 1897 [pub. 1891], (Peter 1891: 30). Type. VENEZUELA. *A. Fendler* 2089 (lectotype GOET005720, designated by Staples et al. 2012: 675, isolectotypes K, MO).

#### Type.

Based on *Convolvulus minutiflorus* M. Martens & Galeotti

#### Description.

Annual herb; stems trailing, spreading from a central rootstock, pilose with white bulbous based whitish hairs, sometimes glabrescent. Leaves petiolate, 0.5–2.7 × 2.5, broadly ovate to subreniform, abruptly and shortly acuminate, base cordate with rounded auricles, margins ciliate, both surfaces thinly pilose to glabrous; petioles 2–17 mm, pilose. Inflorescence of solitary axillary flowers or few-flowered cymes often appearing to form apparently terminal panicles at the branch tips; peduncles 1–3 cm, filiform, usually straight, often arising through the sinus at the base of the leaf, thinly pilose; bracteoles c. 1 × 0.25 mm, linear-lanceolate, glabrous; secondary peduncles, if present, 2–2.5 cm, glabrous; pedicels 1.5–6 mm, often recurved or bent at an angle to the peduncle, glabrous; sepals subequal, 2–3 × 1 mm, accrescent to 3.5–4 mm in fruit, lanceolate, acuminate, pilose, margins narrow, white; corolla 4.5–5 mm long, pale yellow, campanulate, glabrous. Capsules globose, muticous, c. 3 mm. glabrous; seeds 2 × 1.5 mm, rounded-trigonous, minutely puberulent.

#### Illustration.

**Figure 159.**
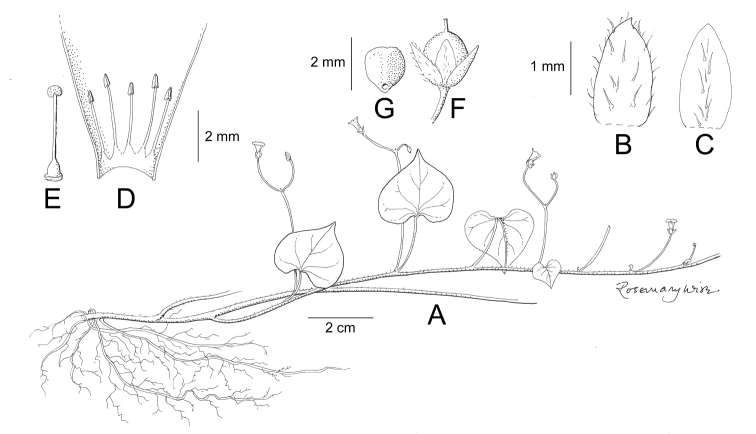
*Ipomoea minutiflora*. **A** habit **B** outer sepal **C** inner sepal **D** corolla opened out to show stamens **E** ovary and style **F** calyx and capsule **G** seed. Drawn by Rosemary Wise **A** from *Weberbauer* 2812; **B–G** from *Tonduz* 13669.

Austin (1975b: 208); Figures 11N, 159.

#### Distribution.

Deciduous tropical forest in northern South America to northern Mexico, usually at low altitudes except in Mexico where it reaches 1600 m. Widespread but very scattered and rather uncommon.

**BRAZIL. Dist. Fed.**: *E.P. Heringer* 3810 (MO). **Pará**: Carajás, *F.D. Gontijo* 183 (RB).

**COLOMBIA. La Guajira**: *T. Saravia* 2899 (COL). **Magdalena**: Santa Marta, *H.H. Smith* 1589 (BM, COL, K, MO, P, S); Tucarinca, *C. Romero* 577 (COL).

**VENEZUELA. Aragua**: *H. Pittier* 15639 (US, VEN). **Bolívar**: *T. Sprague* s.n. (K). **Carabobo**: Barbula, Valencia-La Entrada, *A.H.G. Alston* 5628 (BM). **Guárico**: Est. Biol. Calabozo, *L. Aristeguieta & F. Tamayo* 4394 (MO, VEN). **Monagas**: Ezequiel Zamora, *A. Fernández* 10184 (COL). Also Barinas and Yaracuy fide Austin (1982b).

**PANAMA.***W.H. Lewis et al.* 3004 (MO, RB).

**COSTA RICA.** Nicoya, *A. Tonduz* 13669 (BM, K); Puntoarenas, P.N. Corcovado, *R. Aguilar* 3777 (BM, INB, K, MO); ibid., *B. Hammel* 18761 (CR, F, MO); ibid., Aguirre, *A. Estrada et al.* 2641 (CR, K).

**NICARAGUA.** P.N. Volcán Masaya, *D. Weberbauer* 2812 (BM, MO); Chontales, *W.D. Stevens & O.M. Montiel* 26592 (BM, MO).

**EL SALVADOR.** Morazán, Montecristo, *J.M. Tucker* 438 (K); Sonsonate, *D. Rodríguez & J. Trejo* 00139 (B, BM, LAGU, MO, W).

**HONDURAS.***A. Molina* 18392 (F); Comayagua, *C.H. Nelson et al.* 6074 (MO).

**BELIZE.** Stann Creek, *W.A. Schipp* 740 (BM, K, MO, S).

**GUATEMALA.** Santa Rosa, Chupadero, *W.C. Shannon* 4026 (K).

**MEXICO. Baja California Sur**: Los Cabos, *R. Domínguez Cadena* 2455 (MEXU). **Campeche**: *Chan* 6156 (CICY). **Chiapas**: Tonala, *C.A. Purpus* 6909 (BM, MO, NY). **Chihuahua**: Río Moris junction with Río Agua Caliente, *P.D. Jenkins* 91-79-A (ARIZ). **Colima**: *C.R. Orcutt* 4536 (BM, MEXU, MO). **Est. México & Dist. Fed.**: Temascaltepec, *G.B. Hinton* 5002 (GBH, K). **Guerrero**: Galeana, San Luis, *G.B. & J.C. Hinton* 10864 (GBH, K, MO); Acapulco, *E. Palmer* 109 (K). **Jalisco**: La Huerta, *M.G. Ayala* 1023 (K). **Michoacán**: Huetamo, Tacupa, *G.B. Hinton* 7119 (K). **Morelos**: Ixtla, *R. Ramírez et al.* 3924 (MEXU). **Nayarit**: San Blas, *G. Flores & R. Ramírez* 2479 (MEXU). **Oaxaca**: Tehuantepec, *E. Martínez & M. Elorsa* 32727 (MEXU); Pochutla, *A. Sánchez Martínez & A. Nava* 357 (MEXU). **Puebla**: Acatlan, *R. Miranda* 2461 (MEXU). **Sinaloa**: Imala, *E. Palmer* 1674 (S, US); Las Mesas, Sierra Surotato, *H.S. Gentry* 6665 (DES); **Sonora**: Pinal, Río Mayo, *H.S. Gentry* 1687 (K, MEXU, S); Mun. Alamos, Arroyo Huirotal, *T.R. van Devender* 94-935 (ARIZ). **Veracruz**: Palmilla, *F. Ventura* 2690 (ASU, MEXU).

#### Note.

Very distinctive because of the small yellow corolla and tiny sepals.

### 337. Ipomoea suffulta

Taxon classificationPlantaeSolanalesConvolvulaceae

(Kunth) G. Don, Gen. Hist. 4: 276. 1838. (Don 1838: 276)

Convolvulus suffultus Kunth, Nov. Gen. Sp. 3: 102. 1818 [pub. 1819]. (Kunth 1819: 102). Type. MEXICO. Michoacán, Vulcan de Jorullo, Humboldt & Bonplands.n. (holotype P00670752).

#### Type.

Based on *Convolvulus suffultus* Kunth

#### Description.

Procumbent perennial herb with woody rootstock, stems glabrous or hispid-hirsute, up to 4 m long. Leaves petiolate, 1–6 × 1–5 cm, suborbicular to reniform, shortly acuminate, mucronate, margin entire to slightly dentate, glabrous to thinly hispid-pilose; petiole 0.5–1.6(–5.8) cm. Flowers usually solitary, bracteate, the fertile leaves (bracts) folded so forming a spathe around the flower; peduncle not differentiated from the petiole; bracteoles 1 mm, ovate to suborbicular, mucronulate, apparently persistent; pedicels 2–3 mm; sepals somewhat unequal, glabrous to thinly pubescent, outer 3 × 1.25–1.5 mm, lanceolate, obtuse, minutely mucronate, inner 4–5 × 1.5 mm, oblong-lanceolate, obtuse, margins scarious; corolla 4.5–6 cm long, gradually widened (flared) from a short narrowly cylindrical basal tube, reddish-purple, pink or white, glabrous, limb 3.5–4 cm wide. Capsules subglobose, 8–10 mm, glabrous, enclosed by bracts; seeds 6–7 mm long, rounded, blackish, puberulent.

#### Illustration.

Figure 10H; McDonald JA (1987c: 84).

#### Distribution.

Rock outcrops in open deciduous oak or pine forest, mostly between 1000 and 1700 m in central Mexico south to Guatemala.

**G**UATEMALA. *J. Steyermark* 51602 (US).

**M**EXICO. **Chiapas**: Valley of Jiguipilas, *E.W. Nelson* 2920 (GH, K, US); Ocozocoautla de Espinosa, *D.E. Breedlove* 27543 (MO); Tzimol, *A. Reyes García & G. Urquijo* 825 (BM, MEXU). **Est. México & Dist. Fed**.: Temascaltepec, Ixtapan, *G.B. Hinton* 1631(BM, K, MEXU); Tejupilco, *G.B. Hinton* 1606 (BM, K); Tejupilco, *H. Vibrans* 5449 (MEXU). **Guerrero**: Mina, *G.B. Hinton* 9271 (GH, K, MO); Montes de Oca, *G.B. Hinton* 11363 (K); Cajeles, *H. Kruse* 1975 (IEB). **Jalisco**: Tecalitlán, *E. Carranza al.* 6793 (IEB, MO); Tuxpan, C.R. *Barnes & W. Land* 323(K). **Michoacán**: Zitacuaro, *G.B. Hinton* 13188 (K, MO); Cerro Cobrero, *V.W. Steinmann* 5453 (ARIZ, IEB); Carácuaro, *E. Carranza & P. Carrillo* 6388 (IEB). **Nayarit**: Ixtlán to Cerro Juanacata, *Y. Mexia* 869 (MO), 896 (BM); Ahuacatlan, *O. Téllez* 9313 (MEXU). **Oaxaca**: *C.G. Pringle* 4755 (BM, E, K, MO, P, S); Santo Domingo Tonalá, *A. Torres Hernández* 191 (IEB); San Juan Mixtepec, *E. Hunn* 1854 (MEXU).

#### Note.

Very distinct because of the leaf-like bracts folded to form a spathe around the very small calyx and small subglobose Capsules and the suppression of the peduncle by fusion with the petiole. The flared funnel-shaped corolla is also distinct.

### 338. Ipomoea bracteata

Taxon classificationPlantaeSolanalesConvolvulaceae

Cav., Icon 5: 51. 1794 [pub. 1799]. (Cavanilles 1799: 51)

Exogonium bracteatum (Cav.) Choisy ex G. Don, Gen. Hist. 4: 264. 1838. (Don 1838: 264).
Quamoclit bracteata (Cav.) Roberty, Candollea 14: 41. 1952. (Roberty 1952: 41).
Ipomoea cincta Roem. & Schult., Syst. Veg. 4: 254. 1819. (Roemer and Schultes 1819: 254), nom. illeg. superfl. for I. bracteata Cav.
Convolvulus obvallatus Spreng., Syst. Veg. 1: 595. 1825. [pub. 1824]. (Sprengel 1824: 595). Type. Based on Ipomoea bracteata Cav.
Ipomoea spicata Kunth, Nov. Gen. Sp. 3: 112. 1818 [pub.1819]. (Kunth 1819: 112). Type. MEXICO. Guerrero, La Venta de Acaguisotla, Humboldt & Bonplands.n. (holotype P00670776).
Exogonium spicatum (Kunth) Choisy, Mém. Soc. Phys. Genève 8: 50 [128]. 1837 [pub. 1838]. (Choisy 1838: 50[128]).
Exogonium olivae Bárcena, Viaje Cav. Cachuam. 29. 1844. (Bárcena 1844: 29). Type. MEXICO. Cuernavaca, *Bárcena*s.n. (holotype MEXU†, lectotype plate in Bárcena (1844: 29), designated by McDonald 1987c: 59).
Convolvulus bractiflorus Sessé & Moçiño, Pl. Nov. Hisp. 23. 1888). (Sessé y Lacasta and Moçiño 1887– 90: 23). Type. MEXICO. Cuernavaca, Sessé & Moçiño “1629” (lectotype MA00603817, designated by McDonald 1987c: 59).
Ipomoea bracteata var. pubescens B.L. Rob. & Greenm., Amer. J. Sci. ser. 3. 50: 160. 1895. Type. MEXICO. Jalisco, Guadalajara, *C.G. Pringle* 4734 (holotype GH00054485, isotypes BM, BKL, BR, CM, G, GOET, ISC, K, MEXU, MIN, MO, MSC, NDG, NY, S, US, UC, VT).
Exogonium bracteatum var. pubescens (Rob. & Greenm.) House, Bull. Torrey Bot. Club 35: 101. 1908. (House 1908a: 101).
Ipomoea bracteata var. viridibracta McDonald, Brenesia 28: 60. 1987. (McDonald 1987c: 60). Type. MEXICO. Est. Mexico, Temascaltepec, G.B. Hinton 7526 (holotype NY00319073).

#### Type.

MEXICO. Guerrero, Dos Caminos near Acapulco, *Née* s.n. (lectotype MA475867, designated by McDonald 1987c: 58, isolectotype MA).

#### Description.

Vigorous liana; stems climbing or trailing to 7 m, glabrous, plant often leafless when flowering. Leaves petiolate, 2–8 × 2–7 cm, ovate to deltoid, acute or acuminate, mucronate, base shallowly cordate, glabrous or (var. pubescens) pubescent; petioles 0.1–4 cm. Inflorescence of bracteolate axillary raceme-like cymes, rhachis 2–23 mm long, slightly zigzag; peduncles fused to petioles, 3–7 mm; bracteoles showy, 2–4.5 cm long and wide, ovate, acuminate, folded, pink or, rarely (var. viridibracta), greenish, glabrous; pedicels 2–5 mm, usually recurved; sepals subequal, 5–9 × 2–3 mm, oblong-ovate with white margins, the outer obtuse, mucronate, innerslightly larger; corolla 2.2–4 cm long, hypocrateriform, the tube 4–7 mm wide, pink or (rarely) greenish, glabrous, limb reduced to 5 lobes 2–3 mm long and wide, stamens exserted. Capsules 6–10 × 4–8 mm, conical, glabrous; seeds 4–5 × 2–3 mm, ellipsoid, puberulent.

#### Illustration.

Figure 160.

**Figure 160.**
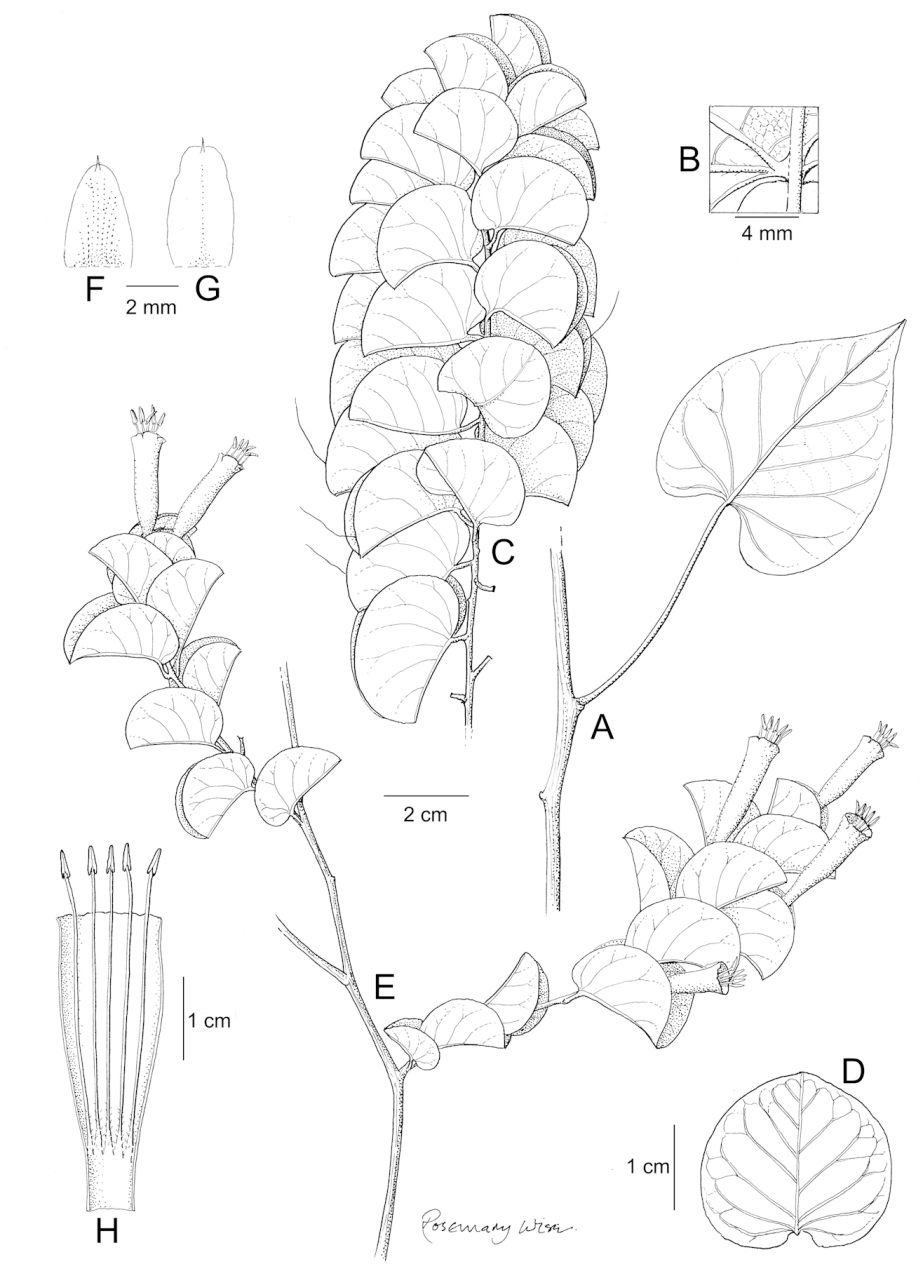
*Ipomoea bracteata*. **A** leaf **B** abaxial leaf surface **C** Stem and bracts **D** bract **E** flowering habit **F** outer sepal **G** inner sepal **H** corolla opened out to show stamens. Drawn by Rosemary Wise **A–D** from *Pringle* 8012; **E** from *Hinton et al.* 7529; **H** from *Y. Mexia* 1917.

#### Distribution.

Endemic to Mexico but locally common in deciduous tropical forest below about 1600 m.

**MEXICO. Baja California Sur**: Sierra Laguna, *H.S. Gentry* 4437 (K, MEXU); Miraflores, *A. Carter* 2659 (K, UC). **Chihuahua**: Río Batapilas, *M. Kimnach & Brandt* 905 (MEXU). **Colima**: Colima-Manzanillo, *E. Carranza & I. Silva* 6036 (IEB, MEXU). **Durango**: Topia, *S. Acevedo & D. Bayona* 346 (IEB, MEXU). **Est. México & Dist. Fed.**: Temascaltepec, Plaza de Gallos, *G.B. Hinton* 1749 (BM, K), ibid., Ixtapan, *G.B. Hinton* 3019 (BM, K), ibid., Calera, 7529 (BM, K, NY). **Guerrero**: Coyuca, *G.B. Hinton* 5560 (K); Placeres, Mina, *G.B. Hinton* 9974 (BM, K); Achotla, *Y. Mexia* 8743 (K); Coyuco de Catalán, *J.C. Soto Nuñez* 11436 (E, MEXU). **Hidalgo**: Chapalhuacan, *R.M. Saucedo & O.A. Ayala* 855 (MEXU). **Jalisco**: Talpa de Allende-Tomatlán, *K.M. Peterson & C.R. Broome* 442 (K); Patalarga, *P. Carillo-Reyes et al*. 7248 (IEB); San Sebastián to Los Reyes, *Y. Mexia* 1917 (BM). **Michoacán**: *G.B. Hinton* 6974 (K); S. of Taretan, *E. Carranza & V. Steinmann* 6316 (IEB). **Morelos**: Cuernavaca, *C.G. Pringle* 8012 (GH, K, MO, US); ibid., *Bourgeau* 1246 (K, P). **Nayarit**: Acaponeta, *R. Ramírez & G. Flores* 816 (IEB). **Oaxaca**: Laguna el Portrerón, *M. Elorsa* 5731(IEB). **Puebla**: Teotalca, *A.G. Miranda & C. García* 898 (MEXU). **Sinaloa**: Rosario, *F.H. Lamb* 450 (GH, K); Mazatlan, *J.G. Ortega* 5590 (K); Culiacán, *Rito Vega* 2646 (MEXU). **Sonora**: Mun. Alamos, *T.R. Van Devender* 94-166 (ASU); Mun. Huatobampo, *S.L. Friedman* 32-94 (ASU); San Bernardo, Río Mayo, *H.S. Gentry* 1293 (K). **Veracruz**: Remulatero, *C.A. Purpus* 8644 (BM), 16377 (K).

#### Notes.

A very distinctive species with woody stems and a subcylindrical hypocrateriform corolla which is enclosed by a pair of showy bracteoles. As in *Ipomoea dumosa* the petiole and peduncle are partially fused. Although very distinct *I. bracteata* is also very variable. The leaves are usually glabrous but a pubescent form (var. pubescens) occasionally occurs; the bracteoles and corolla are usually pink but plants with greenish bracteoles (var. viridibracta) and green corollas are occasionally found.

*Ipomoea bracteata* flowers in the dry season, fide Chemás-Jaramillo and Bullock (2005).

••• Clade C (Species 339–378) comprise a morphologically heterogeneous group of American and Australasian species, which contains a number of small, well-supported and morphologically distinct clades, which are indicated in the following sequence.

### 339. Ipomoea pes-caprae

Taxon classificationPlantaeSolanalesConvolvulaceae

(L.) R. Br. in Tuckey, Narr. Exped. Zaire 477. 1818. (Tuckey 1818: 477)

Convolvulus pes-caprae L., Sp. Pl. 159. 1753. (Linnaeus 1753: 159). Type. Linn. No. 218.59 (lectotype LINN, designated by St John [1957: 65]).
Plesiagopus sovana Raf., Fl. Tellur. 4: 78. 1838. (Rafinesque 1838a: 78). Type. Based on Convolvulus pes-caprae L.
Ipomoea aegopoda St. Lag., Soc. Ann. Bot. Lyon. 7: 70. 1880. (Saint-Lager 1880: 70), nom. illeg. superfl. Type. Based on Ipomoea pes-caprae (L.) R. Br.
Quamoclit pes-caprae (L.) M. Gómez, Fl. Habana 346. 1899 [pub.1897]. (Gómez de la Maza y Jiménez 1897: 346).
Convolvulus brasiliensis L., Sp. Pl. 159. 1753. (Linnaeus 1753: 159). Type. Icon in Plumier, Descr. Pl. Amer. 89, t. 104 (1693), lectotype designated by St John (1957: 66).
Ipomoea brasiliensis (L.) Sweet, Hort. Suburb. Lond. 35. 1818. (Sweet 1818: 35).
Latrienda brasiliensis (L.) Raf., Fl. Tellur. 4: 81. 1836 [pub.1838]. (Rafinesque 1838a: 81).
Ipomoea pes-caprae subsp. brasiliensis (L.) Ooststr., Blumea 3: 533. 1940. (Ooststroom 1940: 533).
Ipomoea pes-caprae var. emarginata Hallier f., Bull. Soc. Roy. Bot. Belg. 37: 98.1898. (Hallier 1898a: 98). Type. Based on Convolvulus brasiliensis L.
Ipomoea bilobata var. emarginata (Hallier f.) F.N. Williams, Bull. Herb. Boiss., ser. 2, 5: 438. 1905. (Williams, FN 1905: 438).
Ipomoea brasilianus , L., Fl. Jam. 14. 1759 (lapsus?). (Linnaeus 1759b: 14).
Ipomoea biloba Forssk., Fl. Aegypt-Arab. 44. 1775. (Forsskal 1775: 44). Type. YEMEN. Zabid, *Forsskal*s.n. (lectotype BM001014578, designated by Verdcourt [1963: 121]).
Ipomoea pes-caprae var. biloba (Forssk.) Hallier f., Annuario Reale Ist. Bot. Roma 7: 231. 1898. (Hallier 1898c: 231).
Convolvulus maritimus Desr. Encycl. Meth. 3(2): 550. 1792 [dated 1789). (Desrousseaux 1792: 550), nom. illeg., non Convolvulus maritimusLamarck (1779). Type. Various syntypes cited.
Convolvulus bauhiniarefolius Salisb., Prodr. Stirp. Hort. Chapel Allerton 125. 1796. (Salisbury 1796: 125), nom. illeg. superfl. Type. Based on Convolvulus pes-caprae L.
Ipomoea maritima R.Br., Prodr. 486. 1810. (Brown, R 1810: 486). Type. Based on Convolvulus maritimus Desr.
Batatas maritimus (R.Br.) Bojer, Hort. Maurit. 225. 1837 (Bojer 1837: 225).
Convolvulus biglandulosus Stokes, Bot. Mat. Med. 1: 326. 1812. (Stokes 1812: 326), nom. illeg. superfl. Type based on Convolvulus brasiliensis L.
Convolvulus capripes Stokes, Bot. Mat. Med. 1: 327. 1812. (Stokes 1812: 327), nom. illeg. superfl. Type based on Convolvulus pes-caprae L.
Ipomoea orbicularis Elliot, Sketch Bot. S. Carolina 1(3): 257. 1817. (Elliot 1817: 157). Type. UNITED STATES. Georgia, Cumberland Island, *W. Bernard* (syntypes PH00016071 & CHARL-BY2408).
Bonanox orbicularis (Elliot) Raf., Fl. Tellur. 4: 77. 1836 [pub. 1838]. (Rafinesque 1838a: 77).
Convolvulus bilobatus Roxb., Fl. Ind., ed. 2: 73. 1824. (Roxburgh 1824: 73). Type. Plant cultivated at Calcutta with roots from the Moluccas, (lectotype *Wallich* 1359 (K0011128888, portion on right of sheet, designated here).
Ipomoea bilobata (Roxb.) G. Don in Sweet, Hort. Brit., ed. 3, 489. 1839. (Sweet 1839: 489).
Convolvulus retusus Colla, Hort. Ripul. append. 3: 144 [31]. 1826. (Colla 1826b: 144), nom. nud. Type. GUADELOUPE. *Bertero*s.n. (TO).
Convolvulus rotundifolius Schumach. & Thonn., Beskr. Guin. Pl. 102. 1827. (Schumacher and Thonning 1827: 102). Type. GHANA. *Thonning*s.n. (syntype C100003635).
Ipomoea brevipes Sessé & Moçiño ex Choisy in A.P. de Candolle, Prodr. 9: 349. 1845. (Choisy 1845: 349). Type. Fl. Mexicana, unpublished image (whereabouts unknown, not found in the Torner Collection of Sessé and Moçiño Biological Illustrations).
Ipomoea pes-caprae forma arenaria Dammer, Pflanzenw. Ost-Afrikas 332. 1895. (Engler 1895: 332). Type. TANZANIA. *C. Holst* 3040 (holotype B†, isotype HBG505562).
Ipomoea pes-caprae forma albiflora Domin, Biblioth. Bot. 89: 1090. 1928. (Domin 1928: 536), nom. nud. Based on a collection by Domin from Yarraba, Queensland, Australia.
Ipomoea pes-caprae var. perunkulamensis P. Umam. & P. Daniel, J. Econ. Taxon. Bot. 23: 691. 1999. (Umamaheswari and Daniel 1999: 691). Type. INDIA. [Tamil Nadu], Ramanathapurum Distr., Perunkulam, *P. Daniel* 101473 (holotype CAL, isotype MH).

#### Type.

Based on *Convolvulus pes-caprae* L.

#### Description.

Vigorous trailing perennial; stems stout, glabrous, rooting at the nodes, up to 30 m in length; latex present. Leaves petiolate, 3.5–9 × 3–10 cm, coriaceous and somewhat succulent, ovate to reniform or suborbicular, apex emarginate to shallowly bilobed (rarely rounded), base truncate to weakly cordate, abaxially paler, prominently veined and with glands near base of midrib; petioles 2–10 cm. Inflorescence of shortly pedunculate axillary cymes; peduncles 1.5–14 cm; bracteoles 2–3.5 mm, ovate-deltoid, acuminate, caducous; pedicels 1.5–2.7 cm, thickened upwards; sepals slightly unequal, pale green, coriaceous, suborbicular or broadly ovate, outer 5–12 × 6 mm, elliptic, mucronate, inner 7–11 × 7–9 mm, slightly larger, suborbicular with scarious margins; corolla 4–5 cm long, funnel-shaded, pink, glabrous, limb 4–5 cm diam. Capsules 1.5–2.2 cm, subglobose, glabrous, the slender style somewhat persistent; seeds 6–8 mm, “pea”-shaped, black, shortly tomentose; pedicel often persistent on fallen capsule so aiding dispersal in the sea.

**Figure 161.**
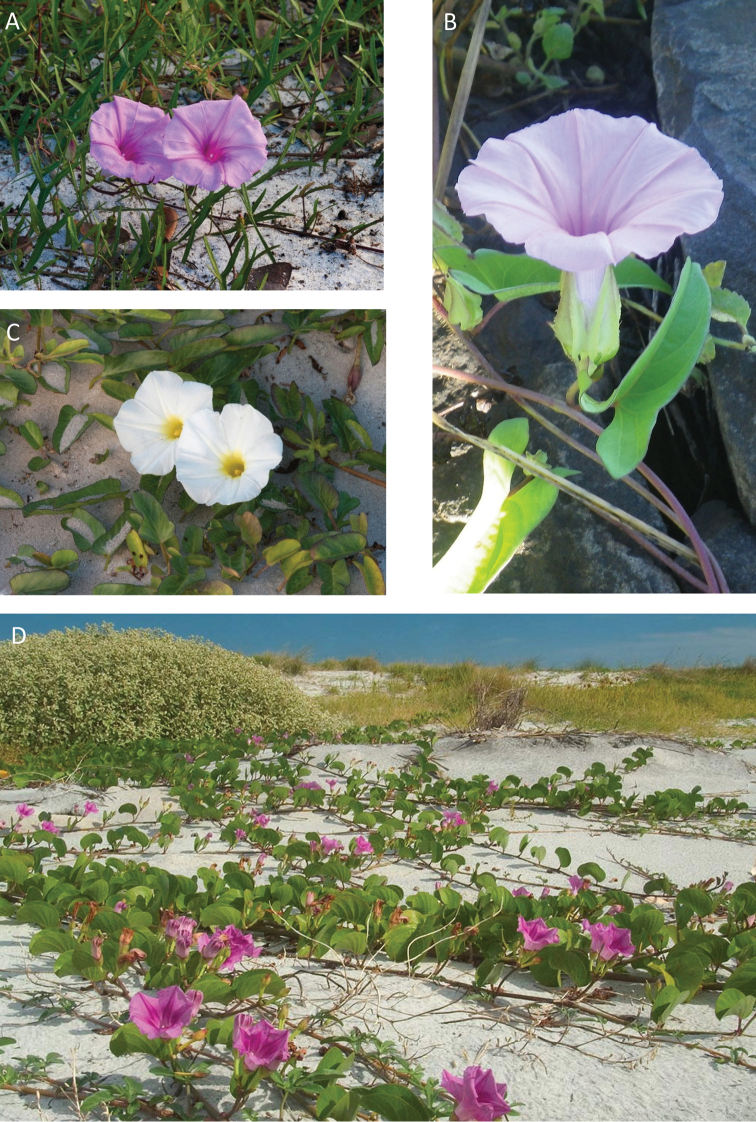
Photographs of *Ipomoea* species. **A***I. sagittata***B***I. fimbriosepala***C***I. imperati***D***I. pes-caprae*. **A** Alamy Ltd. **B** John Wood **C** Alamy Ltd. **D**http://plantworld2.blogspot.com.

**Illustrations**. Figure 161D; Proctor (2012: 551); Acevedo-Rodríguez (2005: 174); Bosser and Heine (2000: 31); Deroin (2001: 229).

#### Distribution.

Pantropical on sand near the sea; a characteristic seashore plant, also occurring rarely in saline conditions inland.

**BRAZIL. Alagoas**: *S. Tsugaru* B1452 (NY, MO). **Bahia**: *Blanchet* 336 (BM); *R.M. Harley* 17098 (K, MO, RB), 18056 (K, RB). **Ceará**: *A. Löfgren* 1 (S); *A.S.F. Castro* 1371 (EAC). **Espirito Santo**: *Z.A. Trinta & E. Fromm* 2147 (K). **Pará**: *M.N. Bastos* 1362 (RB). **Paraíba**: *J.C. de Moraes* 2276 (NY); *M.F. Agra* 1440 (K). **Paraná**: *G. Hatschbach1208* (S). **Pernambuco**: *G. Gardner* s.n. [12/1837] (BM); Fernando de Noronho, *G. Prance* 26336 (NY). **Piauí**: *M.L. Montes* 12 (CEPEC). **Rio de Janeiro**: *J. Fontella* 2997 (RB); *Hemmendorf* 410 (S). **Rio Grande do Norte**: *M.B. de Sousa* 154 (RB). **Santa Catarina**: *A. Krapovickas & C. Cristóbal* 42118 (K, CTES); Guanabara, *A.P. Duarte* 6251 (K, RB). **São Paulo**: *C.W. Mosén* 3442 (S); *K. Mizoguchi* 974 (MO, NY). **Sergipe**: *C. Farney* 2746 (RB).

**FRENCH GUIANA.***B. Bordenave* 112 (P)

**SURINAM.** Fide Austin and Huáman (1996).

**GUYANA.***A. Leechman* s.n. [5/4/1917] (K); *A.S. Hitchcock* 16571 (NY, S); *A.C. Persaud* 140 (F).

**PERU. Lambayeque**: *E. Cerrate et al.* 5279A (MO). **Tumbes**: *R. Ferreyra* 12282 (MO).

**ECUADOR. Galapagos**: *T. Taylor* 94 (K), *H. Van der Werff* 1851 (S), *G. Harling* 5574 (S). Esmeraldas: *B. Sparre* 15336 (S). **Guayas**: *L. Holm-Nielsen* 2504 (AAU, MO, NY, S). **Manabí**: *L. Holm-Nielsen* 21793 (AAU, K, MO).

**COLOMBIA. Antioquia**: *F.J. Rodán et al.* 512 (MO). **Cauca**: *K. von Sneidern* 4862 (S). **Chocó**: *A. Gentry & M.E. Fallen* 17504 (COL, MO); *P. Pinto* 142 (COL). **Magdalena**: Santa Marta, *H.H. Smith* 1582 (K, NY, MO, S); *T. Plowman* 3538 (K). **San Andrés Island**: *Romero* 9021 (COL); *A. Fernández* 5178 (COL).

**VENEZUELA.***E. Asplund* 15015 (S). **Anzoátegui**: *F. & J.F. Delascio* 12885 (MO). **Carabobo**: El Palito, *A.H.G. Alston* 6094 (BM, S). **Delta Amacuro**: *J.A. Steyermark et al*. 114915 (MO). **Dist. Fed.**: *J. Luteyn* 8347 (F). **Sucre**: *J. Steyermark* 108325 (MO).

**PANAMA.***A. Fendler* 239 (K); *W.H. Lewis* 2852 (MO, RB).

**COSTA RICA.***A.A. Beetle* 26212 (K, UC); *J. Solano* 140 (K, MO); *M. Chavarría* 707 (K, MO).

**NICARAGUA.***R. Tate* 344 (K); *W.D. Stevens* 27205 (MO).

**HONDURAS.***T.G. Yuncker et al.* 8250 (K, US); *A. Molina* 23284 (MO).

**EL SALVADOR.***K.J. Sidwell et al.* 639 (BM, MO).

**BELIZE.***C.L. Lundell* 1931 (MICH, S); *W.A. Schipp* 624 (K); *P.H. Gentle* 7836 (MO).

**GUATEMALA.***R. Escobar* s.n. [20/9/2003] (MO).

**CLIPPERTON ISLAND.***M.H. Sachet* 320 (K).

**MEXICO. Baja California Sur**: *J.I. Calzada* 25244 (K); Las Cruces, *I.L. Wiggins* 15672 (K). **Chiapas**: *D.E. Breedlove & R.F. Thorne* 20851 (MO). **Colima**: Clarion Island: *H.J. Mason* 1559 (K, UC). **Jalisco**: *R. Acevedo* 1015 (UCR). **Michoacán**: *J.C. Soto* 3738 (MO). **Nayarit**: *O. Téllez & G. Flores* 11768 (MO). **Oaxaca**: *C. Martínez* 828 (MO). **Quintana Roo**: *O. Telléz & E.F. Cabrera* 1870 (MO). **Sinaloa**: *M. Ruíz et al.* 2006-481 (ARIZ). **Sonora**: *S.L. Friedman* 43-96 (ASU). **Tabasco**: *F. Ventura* 20544 (MO). **Tamaulipas**: *G.L. Fisher* 46180 (S); *E. Palmer* 257 (K, MO). **Veracruz**: *C.R. Orcutt* 3463 (K). **Yucatán**: *G.F. Gaumer* 662 (K, S).

**UNITED STATES. Florida**: *A.H. Curtiss* 2160 (BM, K, S), 5533 (K); *H. Moldenke* 258a (K). **Louisiana**: *S. Javed & C. Reid* 8 (LSU). **Mississippi**: *D. Damaree* 33337 (S), 33688 (S). **Texas**: *Gust & Stone* 308 (MO); *H. Aguilar et al.* 1058 (K).

**BERMUDA.***A.B. Rendle* 800 (BM); *F.S. Collins* 252 (K).

**BAHAMAS.***F. Dale* (BM); *R.A. & E.S. Howard* 10196 (NY, S); *P. Wilson* 7973 (K, NY)

**TURKS & CAICOS ISLANDS.***P. Raven* 28245 (BM, MO)

**CUBA.***H. Manitz* s.n. [6/11/1983] (HAJB), (HAJB29817); *C. Wright* 452 (K); *W. Palmer* 1146 (NY); *R. Combs* 614 (NY).

**CAYMAN ISLANDS.***M. Brunt* 1743 (BM).

**JAMAICA.***Morley & Whitefoord* 978 (BM); *W. Stearn* 179 (BM); *G.R. Proctor* 11504 (BM); *T.G. Yuncker 17126* (NY).

**HAITI.***E.L. Ekman* H9960 (K, NY, S); *E.C. Leonard* 14211 (NY)

**DOMINICAN REPUBLIC.***M. Fuertes* 1159 (BM, K); *H.A. Allard* 14392 (S); *T.A. Zanoni & M. Mejia* 17119 (MO, NY).

**PUERTO RICO.***G.P. De Wolf* 1910 (A, BM, MO, S); *P. Sintenis* 86 (K).

**LESSER ANTILLES. U.S. Virgin Islands**: St Croix: *Thompson 983* (S); St John: *P. Acevedo-Rodríguez et al.* 2052 (NY). **U.K. Virgin Islands**: Tortola: *W.G. D’Arcy* 4759 (BM, MO). **Netherlands Antilles**: St Eustatius: *B.M. Boom et al.* 11266 (NY). **St Kitts**: *A.L. Britton & J.F. Cowell* 434 (NY). **Barbuda**: *Gregory* s.n. (BM). **Antigua**: *Wheeler* 5 (BM). **Montserrat**: *D. Potter* 5557 (GH). **Guadeloupe**: *Duchassaing* (K, P); *A. Duss* 3501 (NY). **Dominica**: *Wilbur et al.* 7984 (BM). **Martinique**: *Hahn* 1200 (BM). **St Lucia**: *G.R. Proctor* 18117 (BM). **St Vincent**: *H.H. & G.W. Smith* 490 (K). **Grenada**: *G.R. Proctor* 17206 (BM); *P. Beard* 1266 (K, NY, S). **Barbados**: *E.G.B. Gooding* 189 (BM).

**TRINIDAD.***W.E. Broadway* 9401 (K). **Tobago**: *H.F.A. von Eggers* 5624 (K).

**NETHERLANDS ANTILLES. Aruba**, **Bonaire**, **Curaçao** fide Proosdij (2012).

**HAWAII.***Phillips & Johnson* 716 (MO); *A.A. Heller* 2097 (BM); *G.W. Barklay* 1328 (BM).

#### Notes.

*Ipomoea pes-caprae* is commonly divided into two subspecies or varieties. Only subsp. brasiliensis (or var. emarginata, if recognised at varietal level) occurs in the New World. It is recognised by its emarginate leaves, whereas the type from the northern Indian Ocean area has deeply bilobed leaves, the lobes somewhat divergent. Opinions about the status of these two forms have varied over the years. Recent molecular studies (Miryeganeh et al. 2014) suggest the two forms are genetically separate and rarely hybridise but some intermediates occur and the issue is not yet fully resolved.

*Ipomoea pes-caprae* is sometimes confused with *I. asarifolia* but the latter has subreniform leaves and very unequal, often muricate sepals.

Molecular data suggests this species is most closely related to a small clade of Australian species. It is widespread on tropical sea shores. Its total world distribution is given in detail by St John (1970).

We have been unable to trace any publication data for the combination. Ipomoea pes-caprae var. brasiliensis (L.) A. St.-Hil.

### 340. Ipomoea amnicola

Taxon classificationPlantaeSolanalesConvolvulaceae

Morong in Morong & Britton, Ann. New York Acad. Sc. 7: 170. 1892. (Morong and Britton 1892: 170)

#### Type.

PARAGUAY. Banks of the Pilcomayo, *T. Morong* 974 (lectotype NY00319140, designated here, isolectotypes MO, NY, R).

#### Description.

Somewhat succulent twining or trailing perennial, completely glabrous in all parts. Leaves petiolate, 2–8(–12) × 2–8-(10) cm, ovate, sometimes broadly so, usually constricted in the middle to form a tapering acuminate apical portion, base cordate with rounded auricles, abaxially slightly glaucous; petioles 1–10 cm. Inflorescence of lax to rather dense, many-flowered, pedunculate, simple or compound cymes; peduncles 1–5 cm; bracteoles 1–3 mm, lanceolate to ovate, caducous; secondary peduncles 5–20 mm; pedicels 0.8–2.5 cm; sepals slightly unequal, coriaceous, glabrous, outer 4–6 × 3–4 mm long, ovate-elliptic, convex, obtuse and shortly mucronate, inner 5–7 × 4–5 mm long, broadly oblong-elliptic to obovate, rounded, with broad scarious margins; corolla 2–5.5 cm long, pale lilac to pink with dark centre, glabrous, funnel-shaped, the limb 2.5–3.5 cm diam., unlobed. Capsules 7–12 × 6 mm, conical, shortly rostrate, glabrous; seeds 5–7 × 2.5–4 mm, reddish brown, the surface minutely tomentellous, the angles densely pilose.

We recognise two subspecies, which intergrade in the region around the Pantanal and perhaps elsewhere.

### 340a. Ipomoea amnicola subsp. amnicola

Taxon classificationPlantaeSolanalesConvolvulaceae

Ipomoea nuda N.E. Br. *Trans. & Proc. Bot. Soc. Edinb.* 20: 63. 1894, nom. illeg., non Ipomoea nudaPeter 1891. (Brown, NE: 1894: 63). Type. PARAGUAY. RVo Pilcomayo. *J.G. Kerr* 12 (not found at K).

#### Diagnosis.

Inflorescence of usually rather dense axillary cymes; peduncles 1–5 cm; outer sepals 4–5 mm long, inner sepals 5–5.5 mm long; corolla 2–3 cm long, pale lilac with dark centre, the limb 2.5–3 cm diam; seeds 5 × 2.5 mm.

#### Illustration.

**Figure 162.**
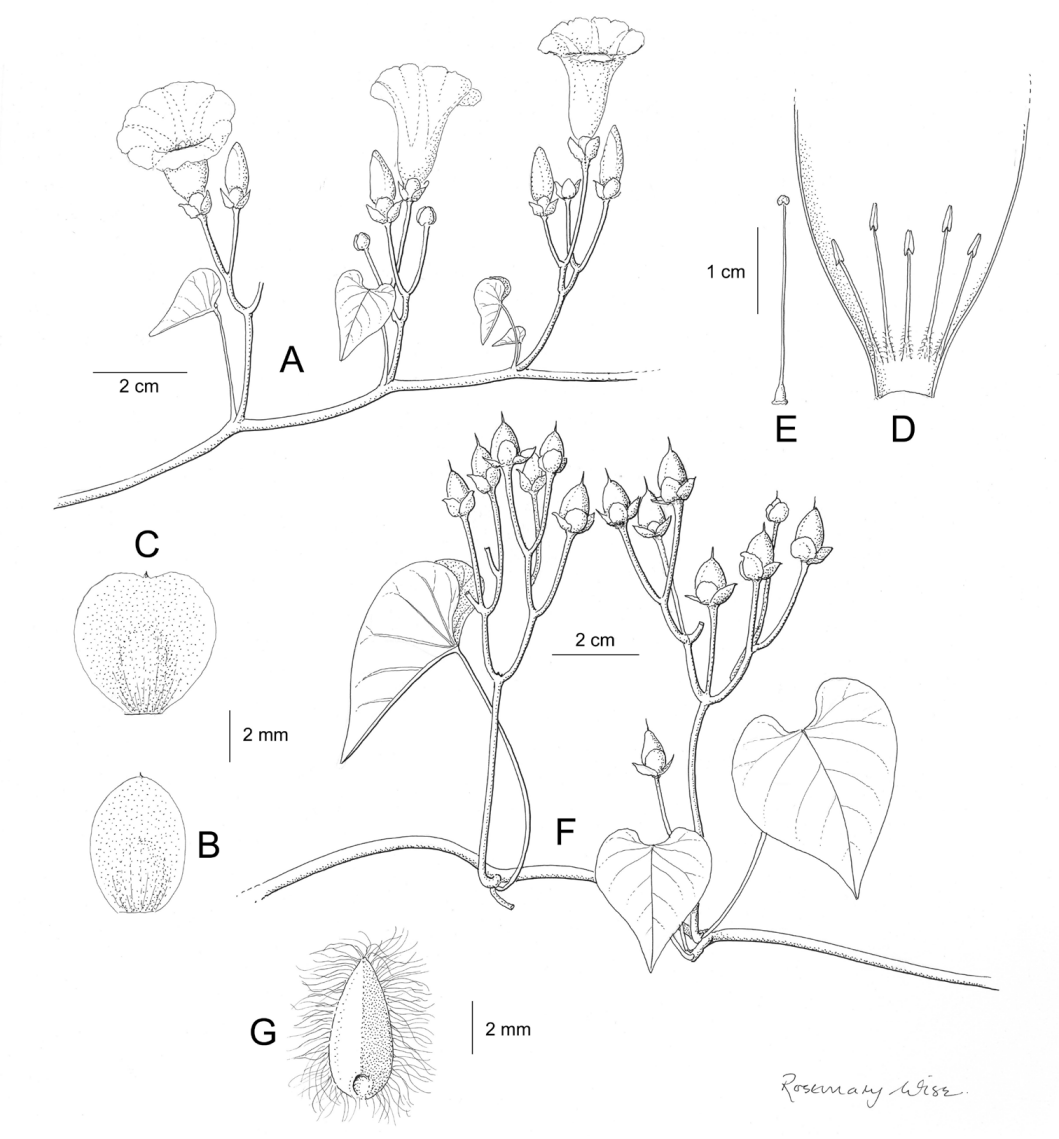
*Ipomoea amnicola subsp. amnicola*. **A** habit **B** outer sepal **C** inner sepal **D** corolla opened out to show stamens **E** ovary and style **F** habit with capsules **G** seed. Drawn by Rosemary Wise **A–E** from *Wood & Mamani* 27484; **F–G** from *Wood & D. Soto* 27947.

Figures 2G, 141E, 162.

#### Distribution.

This subspecies has an amphitropical distribution being found in the southern United States and South America. In South America it is most common as a species of dry Chaco scrub near the Andes in western Argentina, western Paraguay and southern Bolivia but penetrates the Andean cordillera along dry river valleys. It also occurs in dry areas of NW Peru and neighbouring parts of Ecuador and in the upper Magdalena valley in Colombia. In the United States it is perhaps introduced and is most common in the Rio Grande region of Texas. No records from Mexico have been traced.

**ARGENTINA. Catamarca**: *Brizuela* 626 (LIL); Pomán, *P.D. Cantino* 807 (CORD, GH). **Chaco**: *C. O’Donell* 5563 (LIL). **Córdoba**: *Cuezzo* 903 (LIL); Pocho, *A.T. Hunziker & J.A, Caro* 13477 (CORD). **Corrientes**: *T.M. Pedersen* 3866 (C, P, S); *A. Schinini* 4470 (ASU, CTES). **Formosa**: *S. Pierotti* 4175 (LIL, P). **Jujuy**: *A.L. Cabrera* 34061 (MO). **La Rioja**: *Stucker* 17135 (LIL); General Ángel Peñalosa, *A.T. Hunziker et al.* 15117 (CORD, MO). **Salta**: *L.J. Novara et al.* 8901 (S). **Santa Fe**: *S. Venturi* 297 (LIL). **Santiago del Estero**: *T. Meyer* 17076 (LIL).

**PARAGUAY.** Chaco región. **Alto Paraguay**: *F. Mereles* 6728 (FCQ). **Boquerón**: *F. Mereles & R. Degen* 5150 (FCQ), 5680 (FCQ), 5948 (CTES, FCQ). **Central**: *E. Zardini* 2674 (FCQ, MO). **Paraguarí**: Carpegua, *T. Rojas* 3371 (S). **Presidente Hayes**: Maroma, *M. Peña-Chocarro et al.* 1918 (BM, 2556 (BM); *F. Mereles & R. Degen* 6425 (FCQ).

**BRAZIL. Mato Grosso do Sul**: Faz. Uberaba, *J. Almeida de Jesus* 1735 (RB); Estrada Pantaneira, *E.P. Heringer* 831 (NY).

**BOLIVIA.** Inter-andean dry valleys and chaco. **Chuquisaca**: 100 km E of Boyuibe, *B. Mostacedo & T.J. Killeen* 354 (NY, LPB, USZ); Zudañez, Puente Inca, *J.R.I. Wood et al.* 2724 (K, LPB, USZ). **Cochabamba**: Campero, Puente Arce, *J.R.I. Wood* 28119 (K, OXF, USZ). **La Paz**: Sud Yungas: *S.G. Beck* 22444 (K, LPB); Tamayo, ANMI Madidi, *A. Araujo-M et al.* 2869 (LPB, MO). **Potosí**: Charcas, Río Caine bridge, *J.R.I. Wood et al.* 23244 (K, LPB). **Santa Cruz**: Ángel Sandoval, Candelaria, *J.R.I. Wood et al.* 24870 (K, LPB, UB, USZ). Chiquitos, Taperas: *J.R.I. Wood et al. 27873* (K, LPB, USZ). Caballero: La Palisada, *J.R.I. Wood & A. Haigh* 21839 (K, LPB, P); Cordillera, Abapó, *J.R.I. Wood & F. Mamani* 27484 (K, LPB, USZ). Ibañez, *M. Nee* 49480 (LPB, MO, NY, USZ); Ñuflo de Chávez, San Julián, *J.R.I. Wood & D. Soto* 27947 (K, LPB, OXF, USZ); Vallegrande, Río Grande, *G.A. Parada et al.* 4387 (MO, USZ). **Tarija**: Gran Chaco, Palos Blancos, *J.R.I. Wood et al*. 28028 (LPB, OXF, USZ).

**PERU. Amazonas**: Río Chamaya, Bagua-Olmos, *T. Croat* 58302 (MO). **Cajamarca**: *T. Croat* 58367A (MO); *P.C. Hutchison & J.K. Wright* 6734 (F, UC). **Lambayeque**: *Llatas Quiroz* 2402 (F).

**ECUADOR. Loja**: La Toma-El Tambo, *J.E. Madsen et al.* 7772 (AAU).

**COLOMBIA.** Upper Magdalena Valley. **Huila**: *F.R. Fosberg* 19610 (US). **Tolima**: Honda, *E. André* 561 (K).

**UNITED STATES. Georgia**: Spalding County, *W. Hardcastle* s.n. (GA); **Missouri**: Jackson, *B.F. Bush* 9691 (BM, MO). **Texas**: Cameron County, *R. Runyon* 2916 (BM), 2904 (S); Hidalgo County, *E.U. Clover* 301 (MEXU); Kleberg County, *W.R. Carr* 25097 (MEXU).

#### Typification.

There are two sheets of *Morong* 974 at NY. We have selected the best of these as lectotype, rather than the sheet labelled as holotype in an unknown hand as this lacks most diagnostic details.

#### Note.

In the field *Ipomoea amnicola* (especially subsp. amnicola) is usually easily recognised by the relatively small corolla which is pale pink with a dark centre. It often blankets shrubs and small trees where it occurs. The leaves are quite glabrous, usually somewhat glaucous and slightly fleshy. It is not a very easy plant to dry successfully so leaves are often deciduous on herbarium specimens. It can be confused rather easily with species from the Batatas Clade.

### 340b. Ipomoea amnicola subsp. chiliantha

Taxon classificationPlantaeSolanalesConvolvulaceae

(Hallier f.) J.R.I. Wood & Scotland, comb. & stat. nov.

urn:lsid:ipni.org:names:77208082-1

Ipomoea chiliantha Hallier f., Bull. Herb. Boiss. 7 (5), append. 1: 50. 1899. (Hallier 1899c: 50). Type. PARAGUAY. “Villa occidental”, *Lorentz*s.n. (holotype B†, lectotype GOET, designated by Wood et al. 2015: 29).

#### Type.

Based on *Ipomoea chiliantha Hallier f*.

**Diagnosis.** Inflorescence of usually long pedunculate, axillary cymes, sometimes compounded; peduncles 5–13 cm; outer sepals c. 6 × 4 mm long, inner c. 7 × 5 mm; corolla 4–5.5 cm long, pink, darker in the centre, limb 3–3.5 cm diam.; seeds 7 × 4 mm.

#### Illustration.

**Figure 163.**
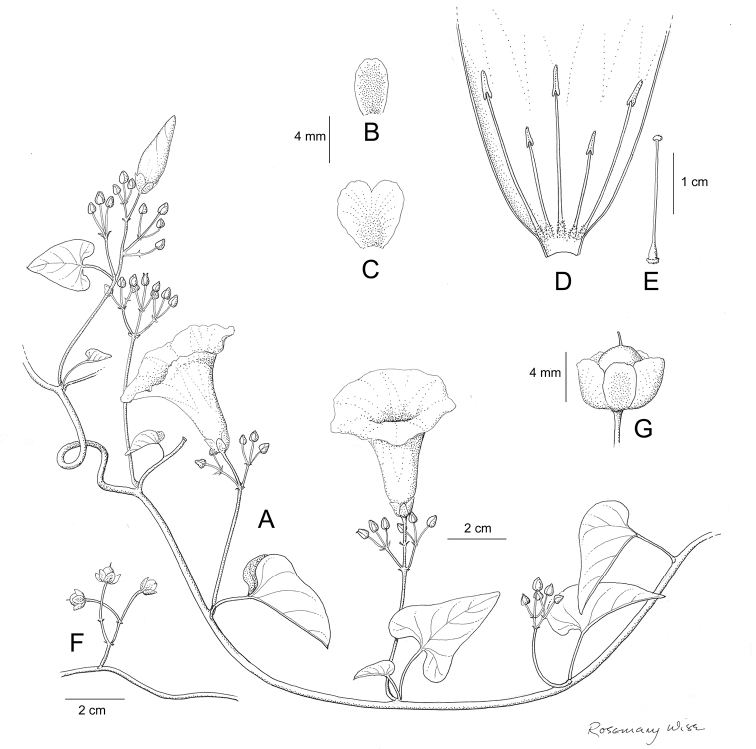
*Ipomoea amnicola subsp. chiliantha*. **A** habit **B** outer sepal **C** inner sepal **D** corolla opened out to show stamens **E** ovary and style **F** fruiting cyme **G** fruiting calyx and capsule. Drawn by Rosemary Wise **A** E from *Mereles* 162; **B–D** from *Petersen* 5497; **F, G** from *Mereles* 2550.

Figure 163; O’Donell (1959b: 147) as *Ipomoea chiliantha*.

#### Distribution.

This subspecies seems to prefer seasonally flooded swampy ground both in Bolivia, Paraguay and the Brazilian Pantanal.

**ARGENTINA. Misiones**: *T.M. Pedersen* 5497 (C, S). **Chaco**: Isla Anequera, *A. Krapovickas & C. Cristóbal* 12733 (CTES), *A. G. Schulz* 2059 (CTES, LIL). **Formosa**: Dept. Pilcomayo, *C. Cristóbal et al.* 2146 (CTES), **Santa Fe**: *Pensiero & Tivano* 3212 (CTES). **Corrientes**: Dept. Capital, *S.G. Tressens et al.* 769 (CTES, MO).

**PARAGUAY. Alto Paraguay**: Est. Cerrito, *F. Mereles* 7006 (FCQ). **Central**: Ypacaraí, *E. Hassler* 11582 (BM, K), 12532 (BM). **Concepción**: San Luis: *K. Fiebrig* 4485A (BM, K, MO). **Cordillera**: *E. Hassler* 1856 (K); *E. Zardini & U. Velázquez* (MO). **Presidente Hayes**: Est. Santa Maria del 12, *M. Peña-Chocarro et al.* 2565 (BM, FCQ); km 130, Ruta Transchaco, *F. Mereles* 2244 (CTES), Puente Remanso, *F. Mereles* 4460 (FCQ); km 58, Ruta Transchaco, *A. Krapovickas & C. Cristóbal* 43220 (CTES, F, FCQ, K). **San Pedro**: abundant in plain west of Com. 25 de Diciembre, *J.R.I. Wood & G. González* 28473 (FCQ); between Río Apa y Río Aquidaban, *K. Fiebrig* 4483 (BM).

**BRAZIL. Mato Grosso do Sul**: Puerto Murtinho, *Robert* 885 (K).

**BOLIVIA. Beni**: Cercado, Puerto Varodor, *Maldonado et al.* 58 (LPB). **Santa Cruz**: Chiquitos, El Tinto–Quimome, *J.R.I. Wood & B. Williams* (OXF, K, LPB, USZ); Germán Busch: Yacuses, *J.R.I. Wood & D. Villarroel* 25541 (K, LPB, UB, USZ); Ñuflo de Chávez *J.R. Abbott* 16966 (BOLV, HSB, LPB, MO, USZ); Puente San Miguelito, *J.R.I. Wood et al.* 27743 (OXF, K, LPB, USZ).

#### Note.

Subsp. chiliantha has the appearance of a large-flowered subsp. amnicola and intermediates occur particularly in the Corumbá region. *Parada et al.* 947 (USZ, MO, OXF) from Carmen Rivero Tórrez and *Wood & Pozo* 26078 (K, LPB, UB, USZ) from San José de Chiquitos are examples from Bolivia but intermediates appear more commonly in the Brazilian Pantanal.

• Species 341–343 comprise a small clade with distinctive ribbed sepals

### 341. Ipomoea fimbriosepala

Taxon classificationPlantaeSolanalesConvolvulaceae

Choisy in A.P. de Candolle, Prodr. 9: 359. 1845. (Choisy 1845: 359)

Ipomoea setifera var. fimbriosepala (Choisy) Fosberg, Smithsonian Contr. Bot. 36: 24. 1977. (Fosberg and Sachet 1977: 24).
Ipomoea choisyi Montrouz., Mém. Acad. Sci. Lyon 10: 237. 1860. (Montrouzier 1860 237). Type. NEW CALEDONIA. *R.P. Montrouzier*s.n. (?holotype P00198442).
Aniseia hastata Meisn. in Martius et al., Fl. Brasil. 7: 319. 1869, non Ipomoea hastata L. (1771). Type. BRAZIL. São Paulo, *W.J. Burchell* 4752 (lecotype BR000005837595, designated here; isolectotype K000612829).
Ipomoea phylloneura Baker, J. Linn. Soc. 21: 426. 1885. (Baker 1885: 426). Type. Based on Aneseia hastata Meisn.
Ipomoea assumptionis Morong, Ann. New York. Acad. Sci. 7: 170. 1892. (Morong and Britton 1892: 170). Type. PARAGUAY. Cerca de Asunción, *T. Morong* 584 (holotype NY00319146, isotypes MO, NY, WIS).
Ipomoea rubra var. palustris Urb., Symb. Antill. 3: 345. 1902. (Urban 1902–3: 345). Type. PUERTO RICO. *P. Sintenis* 962 (isotypes BM, K).
Ipomoea palustris (Urb.) Urb., Symb. Antill. 9: 423. 1925. (Urban 1925: 423).
Ipomoea gilletii De Wild. & T. Durand, Bull. Herb. Boiss. Ser. 2, 1: 36. 1901 [pub. 1900]. (Wilderman and Durand 1900: 36). Type. CONGO D.R. Kisantu, *J. Gillet* 419 (holotype BR00008886279).
Ipomoea pinosia Alain, Revista Soc. Cub. Bot. 13: 60. 1957 (Liogier 1957: 60). Type. CUBA. Isla de Juventud [Isle of Pines], road to San Ignacio de las Piedras, *E.P. Killip* 45247 (holotype US00111440, isotypes B, HAC).
Ipomoea indica var. hosakae Fosberg, Bot. Notis. 129: 38. (Fosberg 1976: 38). Type. CAROLINE ISLANDS. Truk, Moen Island, Hosaka 2713 (holotype US00111403, isotype BISH).
Ipomoea stenantha Dunn, Kew Bull. Add. Ser. 10: 180. 1912. (Dunn and Tutcher 1912: 180). Type. CHINA. Guangdong, Lan-fa Shan, sine coll. (holotype K000830829).
Aniseia stenantha (Dunn) Ling ex R.C. Fang & S.H. Huang, Fl. Reipubl. Popularis Sin. 64(1): 42. 1979. (Fang and Huang 1979: 42).
Aniseia stenantha var. macrostephana Y.H. Zhang, Acta Phytotax. Sin. 24(2): 155. 1986. (Zhang 1986: 155). Type. CHINA. Zhejiang, Longquan, *P.L. Chiu* 1078 (holotype HHBG).
Ipomoea calidicola Standley & L.O. Williams Ceiba 3: 127.1952. (Standley and Williams 1952b: 127). Type. NICARAGUA. *P.C. Standley* 20094 (holotype EAP, Panama, isotype US00111370).

#### Type.

Mauritius. Culta in Hort. Bot. Pamplemousse, 1839, *L. Bouton* s.n. (lectotype G00135515, designated by Delgado Junior et al. (2017).

#### Description.

Twining annual herb, young stems glabrous, older stems setose. Leaves petiolate, 4–9 × 3–5 cm, narrowly (to broadly) deltoid, base sagittate to hastate, auricles acute to obtuse, glabrous, abaxially paler; petioles 2–7.5 cm. Inflorescence of 1(–2)-flowered axillary, pedunculate cyme; peduncles 0.5–3.5 cm; bracteoles 8–15 × 3–5 mm, ovate, acuminate to apiculate, membranous, pale green, moderately persistent; pedicels 1–2.5 cm; outer sepals 13–20 × 7–10 mm, ovate, apex finely mucronate, base truncate, abaxially 3-winged, the wings smooth or (especially below) dentate, inner sepals c. 5 mm shorter, unwinged; corolla 2.5–3.5 cm long, funnel-shaped, pink, glabrous, limb c.5 cm diam., shallowly lobed, the lobes acute. Capsules 12–15 × 12–14 mm, ovoid, glabrous, enclosed by the sepals; seeds 5–6 mm long, minutely tomentellous.

**Illustrations**. Figure 161B; O’Donell (1959b: 156); Deroin (2001: 193).

#### Distribution.

Pantropical in distribution but scattered in occurrence, the populations usually small and impermanent, growing in lowland areas besides lakes, ponds and similar disturbed moist habitats; perhaps most common in the New World around the fringes of the Chaco and in the Llanos of Colombia and noticeably less common in Central America and Mexico.

**ARGENTINA. Chaco**: *A.G. Schulz* 8126 (CTES); *C. Cristóbal et al.* 1534 (CTES). **Corrientes**: *M.M. Arbo et al.* 6591 (CTES, MO, S); *S.G. Tressens et al.* 5027 (CTES). **Misiones**: *M.E. Rodriquez* 01111 (CTES); *H. Keller and Paredes* 9580 (CTES).

**PARAGUAY. Alto Paraná**: *G. Caballero Marmori* 301 (CTES). **Amambay**: *E. Hassler* 7961 (K, S), 10780 (BM, K); 26 km S. de Bella Vista, *M. Dematteis et al.* 3377 (CTES, FCQ). **Central**: near Asunción, *B. Balansa* 1060 (K). **Canindeyú**: 23 km E of Ygatimi, *B. Jimenez & G. Marin* s.n. (PY). **Presidente Hayes**: *A. Krapovickas & C. Cristóbal* 45113 (CTES).

**BRAZIL. Acre**: *Gwynne Vaughan* 47 (K). **Amazonas**: Río Jurua, *Ule* 5196 (K); *L. Teixera* 1333 (NY). **Mato Grosso**: Río Turvo, N of Xavantina, *H.S, Irwin et al.* 16080 (NY). **Minas Gerais**: *A. Macedo* 1675 (S), 1802 (BM, MO, S). **Paraná**: sine col. 257 (RB). **Rio Grande do Sul**: *P.P.A. Ferreira* 233 (ICN) fide Ferreira and Miotto (2009: 445).

**GUYANA.***Appun* 2458 (K)

**BOLIVIA. Beni**: Ballivián, Est. Biológica del Beni, *J. Balderrama* 370 (LPB, MO); **La Paz**: Luisita, *R. Haase* 666 (LPB). **Santa Cruz**: Velasco, c. 35 km N of Santa Rosa de la Roca, *J.R.I. Wood et al.* 27081 (K, LPB, USZ); Chiquitos, Robore, *A. Krapovickas & A. Schinini* 36379 (CTES).

**PERU. Loreto**: *F. Ayala* 808 (MO). **San Martín**: *M. Rimachi* 10265 (F, MO, NY); *R. Ferreyra* 7879 (USM).

**COLOMBIA. Casanare**: Tauramena, *L. Uribe-Uribe* 3587 (COL). **Guainía**: Río Iníridi, *J. Espina* 361 (COL). **Guaviare**: *R. López & O. Rodríguez* 1818 (COL). **Meta**: *R. Cortes et al.* 1106 (COL).

**VENEZUELA. Delta Amacuro**: *J.A. Steyermark et al.* 114849 (MO). **Monagas**: *G. Davidse* et al. 4590 (MO).

**GUATEMALA.***Bernoulli & Cario* 1899 (K).

**MEXICO. Tabasco**: *E. Matuda* 3252 (MEXU). **Veracruz**: *Orozco* 252 (F, MEXU, XAL).

**CUBA.***A. Alvarez et al.* (HAJB50908). **Pinar del Río**: *E.L. Ekman* 17908 (NY, S).

**PUERTO RICO.** Type of Ipomoea rubra var. palustris.

#### Typification.

Deroin suggested that Bosser and Heine had lectotypified *Ipomoea fimbriosepala* with the Lindley collection at CGE. However, they merely cited the collection under the acronym CAM.

The type material of *I. assumptionis* may be a mixed collection with *I. setifera* but the holotype at NY looks unmistakeably to be *I. fimbriosepala*.

#### Note.

This species is similar to *Ipomoea setifera* in having sepals in which the veins are extended into wings, these commonly dentate; also in the relatively persistent, pale green membranous sepals but differing in being annual, the bracteoles narrower, the sepals only 3-winged and the corolla much shorter. Fruiting specimens can be difficult to distinguish and the two species are commonly misidentified.

### 342. Ipomoea setifera

Taxon classificationPlantaeSolanalesConvolvulaceae

Poir., Encycl. 6: 17. 1804. (Poiret 1804: 17)

Convolvulus setifer (Poir.) Spreng., Syst. Veg.1; 597. 1825 [pub.1824]. (Sprengel 1824: 597).
Calystegia setifera (Poir.) Meisn. in Martius et al., Fl. Brasil. 7: 316. 1869. (Meisner 1869: 316).
Convolvulus ruber Vahl, Eclog. Amer.2: 12. 1798. (Vahl 1798: 12). Type. AMERICA. *J.P.B. von Rohr*s.n. (holotype C10009689, isotype BM).
Ipomoea rubra (Vahl) Millsp., Publ. Field Colomb. Mus., Bot. ser. 2: 86. 1900. (Millspaugh 1900: 86), nom. illeg., non Ipomoea rubra Murray (1791).
Ipomoea breviflora G. Mey., Prim. Fl. Esseq. 100. 1818. (Meyer 1818: 100). Type. SURINAM. Río Essequibo, *E.K. Rotschied* 306 (probable type GOET002523).
Calystegia setifera var. poeppigii Meisn. in Martius et al., Fl. Brasil. 7: 317. 1869. (Meisner 1869: 317). Type. BRAZIL. Amazon River, Serpa, *E.F. Poeppig* (lectotype W0062141, designated here).
Ipomoea setifera var. poeppigii (Meisn.) Hoehne, Anexos Mem. Inst. Butantan, Secc. Bot. 1, Fasc. 6: 63. 1922. (Hoehne 1922: 63).
Ipomoea pandurata var. cuspidata O. Kuntze, Rev. Gen. 1(2): 445. 1891. (Kuntze 1891: 445). Type. Cultivated plant from U.S. Virgin Islands, St Thomas, *Kuntze*s.n. (isotypes NY01429999, K000830889).
Ipomoea lesteri Baker, Bull. Misc. Inform. Kew 1892: 83. 1892. (Baker 1892: 83). Type. GAMBIA. *J.Brown-Lester*s.n. (K000097035, lectotype, designated here).
Ipomoea rubra var. alboflavida Urb., Symb. Antill. 3: 345. 1902. (Urban 1902–3: 345). Type. PUERTO RICO. Stahl 791 (whereabouts unknown, ?B†).
Ipomoea serrulifera Stand & Williams Ceiba 3: 128. 1952. (Standley and Williams 1952b: 128). Type. NICARAGUA. San Juan del Norte, *C.L. Smith* 84 (holotype EAP, isotypes F0054896, US00111468).

#### Type.

GUYANA. *Brocheton* s.n. (holotype P-LAM00357506, isotype P-JUSS-6811).

#### Description.

Trailing or twining herb, stems often roughly hirsute with stiff hairs. Leaves petiolate, 4–14 × 3–11 cm, ovate-deltoid or subreniform with wide-spreading obtuse or rounded auricles, base broadly cordate, apex obtuse, emarginate and mucronate, less commonly acute or acuminate, glabrous, lower surface paler, reticulate-veined; petioles 1–8 cm, glabrous but often with scattered tubercles. Inflorescence of pedunculate, 1–3(–5)-flowered, axillary cymes peduncles (0.3–)3–5(–8) cm, sometimes tuberculed; bracteoles 1.2–2 × 0.6–1.5 cm, ovate, long-mucronate, persistent, pale green, convex, concealing the pedicel bases; pedicels 8–28 mm; sepals unequal, glabrous, outer sepals 15–22 × 10–15 cm, elliptic, acute, finely aristate, abaxially 5-winged, wings smooth or, often, softly tubercled, inner sepals c. 15 × 6 mm shorter, ovate, pale, unwinged; corolla 5.5–8 cm long, funnel-shaped, pink, glabrous, limb c. 4 cm diam., unlobed. Capsules ovoid, 10–12 mm long and wide, often enclosed in slightly accrescent sepals; seeds 7–8 mm, minutely pubescent.

**Illustrations**. Figure 2A; O’Donell (1959b: 239); Acevedo-Rodríguez (2005: 178).

#### Distribution.

Widely distributed in tropical America and Africa and apparently more permanent everywhere than *Ipomoea fimbriosepala*. It occurs in many different habitats but prefers stream sides and is occasionally abundant in flooded forest as along the Río Guapore on the Brazil-Bolivia frontier. It seems most common in the Americas in the Amazon basin, the Guianas, Puerto Rico and the Dominican Republic. We have seen no specimens from Mexico, Peru, Ecuador or Haiti.

**ARGENTINA. Corrientes**: San Ignacio, *A. Krapovickas et al.* 44141 (CTES, MO); Ituzaingó, *T. Meyer* 6036 (LIL). **Misiones**: Eldorado, *R. Vanni et al.* 4060 (CTES); Iguazú, *F.O. Zuloaga* 5655 (MO, SI); San Ignacio, *G.J. Schwarz* 7735 (LIL, RB).

**PARAGUAY. Alto Paraná**: *K. Fiebrig* 6097 (LIL, SI). **Central**: *E. Zardini & C. Velázquez* 27531 (MO). **Concepción**: *M. Dematteis et al.* 2922 (CTES, MA). **Itapúa**: Yacyreta, *J. De Egea et al.* 347 (BM, FCQ); Cerro Ybycui, *M. Quintana et al.* 226 (FCQ, PY). **Misiones**: Isla Yvyku’i, *F. González Parini & M.J. López* 602 (FCQ).

**BRAZIL. Acre**: *R.C. Forzza* 6174 (RB). **Amapá**: *D.F. Austin et al.* 6959 (MBG, NY). **Amazonas**: *D.G. Campbell et al.* P22077 (K, MO, NY, S); *D.F. Austin* 6959 (MBG, MO). **Dist. Fed.**: *G. Davidse et al.* 12160 (MO). **Goiás**: *N.T. Silva* 4823 (RB, MBG, MO, NY); Serra Dourada, *E.P. Heringer* 10861 (NY); Alto Paraíso, *H.S. Irwin et al.* 12685 (NY). **Mato Grosso**: P. Estadual Cristalino, *D. Sasaki et al.* 1694 (K); Santa Ana, *S. Moore* 488 (BM). **Mato Grosso do Sul**: Rio Paraná, *L. Bernardi* 18211 (NY). **Minas Gerais**: Ituiutaba, *A. Macedo* 1076 (BM, MO, RB, S); *V.C. Souza et al.* 5194 (K, SPF). **Pará**: *D.F. Austin* 4023 (MO), 4044 (MO); *F. Drouet* (F); Itaituba, *I.L. do Amaral et al*. 1224 (NY); Santarém, *R. Spruce* s.n. (BM, K). **Paraná**: Gueira, *G. Hatschbach et al.* 13324 (K, MBM, NY); *A. Duarte* 1829 (RB). **Roraima**: *G.T. Prance et al.* 4090 (NY, K).

**FRENCH GUIANA.***Von Rohr* 110 (BM); *F. Billiet et al*. 6240 (K); *Sagot* 1308 (BM, P).

**SURINAM.***J. & P.A. Florschütz* 551 (F, K); *B. Hammel & S. Koemar* 21200 (MO).

**GUYANA.***J. G. Myers* 5495 (K); *S.A. Harris* EC22 (K).

**BOLIVIA. Beni**: Vaca Díaz, Guayamerin, *Anderson* 12084 (NY). **Pando**: Río Manuripi, *A. Paniagua & P.F. Foster* 739 (LPB). **Santa Cruz**: Germán Busch, Río Paraguay, *S.G. Beck* 27559 (K, LPB); Guarayos, Com. Momene, *J.R.I. Wood & D. Soto* 27934 (OXF, K, LPB, USZ); Ñuflo de Chávez, Concepción–Lomerío, *J.R.I. Wood et al.* 24971 (K, LPN, UB, USZ); Velasco, Bajo Paraguá, *T. Killeen* 6250 (ARIZ, MO, SP, USZ).

**COLOMBIA.***C. Feddema* 2023 (MICH, S). **Antioquia**: Turbo, *A. Gentry* 9460 (COL). **Chocó**: Bahía Solano, *E.P. Killip & H. García* 33596 (COL, US). **Norte de Santander**: Ocaña, *Kalbreyer* 1273 (K).

**VENEZUELA. Delta Amacaru**: *J. Steyermark et al.* 115154 (MO).

**PANAMA.***A. Fendler* 243 (K).

**COSTA RICA.** Puertarenas, Golfito, *F. Quesado* 825 (BM, K, MO); *R. Schlising* 2860 (F).

**NICARAGUA.** Greytown, *R. Tate* 346 (K); Puerto Isabel, *E. Narvaez & J.T. Atwood* 2888 (BM, F, MO, NY).

**BELIZE.** Stann Creek, *W.A. Schipp* 495 (BM, K, MO, NY, S); *J.D. Dwyer et al.* 647 (MO).

**GUATEMALA.***Friedrichsthal* s.n. (K).

**BAHAMAS.***D.S. & H.B. Correll* 47982 (BM, NY).

**CUBA. [Guantánamo**]: Baracoa, *E.L. Ekman* 4014 (NY, S).

**JAMAICA.***W. Harris* 12468 (K); *W.T. Stearn* 227 (BM, K); *C.D. Adams* 12297 (BM); *C.R. Orcutt* 4149 (BM).

**DOMINICAN REPUBIC.***E.L. Ekman* H11053 (K, S), 11235 (S); *Higgins & Higgins* 59 (K, NY); *A.H. Liogier* 14415 (NY); *H. von Türckheim* 3741 (NY).

**PUERTO RICO.***A. Heller* 376 (K, NY); *R.J. Wagner* 432 (BM, S); *P. Sintensis* 963 (K, NY, S); *B.M. Boom* 10071 (NY).

**LESSER ANTILLES. U.S. Virgin Islands**: St John: *P. Acevedo-Rodríquez* 3094 (NY). **Guadeloupe**: *Hammarlund* 20 (S), 42 (S); *A. Duss* 2474 (NY). **Dominica**: *C. Whitefoord* 5296 (BM). **Martinique**; fide Powell (1979). **St Lucia**: fide Powell (1979). **St Vincent**: *H.H. Smith & G. Smith* 1164 (K, NY).

**TRINIDAD.***W.E.Broadway* s.n. [8/12/1932] (BM, K); *R.E.D. Baker* 14570 (K).

#### Note.

Distinguished from *Ipomoea fimbriosepala* by the much larger corolla, perennial habit, broader bracteoles which enclose the pedicel bases and the 5-winged sepals. The two species are commonly confused.

### 343. Ipomoea parvibracteolata

Taxon classificationPlantaeSolanalesConvolvulaceae

J.R.I. Wood & L.V. Vasconc., Kew Bull. 72 (8): 5. 2017. (Wood et al. 2017a: 5)

#### Type.

BRAZIL. Bahia, Casa Nova, estrada para a Fazenda Santarém, *L.P. de Queiroz et al.* 9615 (holotype HUEFS88992, isotype MBM).

#### Description.

Twining perennial herb reaching 3.5 m; stems slightly woody, glabrous. Leaves petiolate, 1–4 × 1–4 cm, ovate to suborbicular, abruptly narrowed to an acute or shortly acuminate apex, base cordate with rounded auricles, margin slightly undulate; abaxially paler; petioles 0.6–2 cm. Inflorescence of 1–3-flowered, axillary cymes; peduncles 2.2–8.5 cm, noticeably thicker than the secondary peduncles and pedicels; bracteoles 3 × 0.5 mm, somewhat scarious, caducous; secondary peduncles 0.8–2.5 cm; pedicels 1–3 cm long; sepals unequal, ovate or ovate elliptic, acute and mucronate, glabrous, outer 15–27 × 8–11 mm, dark green, prominently 5-ribbed, the ribs sometimes muricate; inner 12–18 × 6–8, pale green with scarious margins, the longitudinal veins many, the midvein terminating in a fine, fragile mucro; corolla 10–10.5 cm long. funnel-shaped, pink, glabrous; limb c. 9 cm diam., entire. Capsules enclosed by the persistent sepals, 13 × 7 mm, narrowly ovoid, muticous, glabrous; seeds 7 × 4 mm, blackish, minutely scabridulous.

#### Illustration.

Figures 11J, 164.

**Figure 164.**
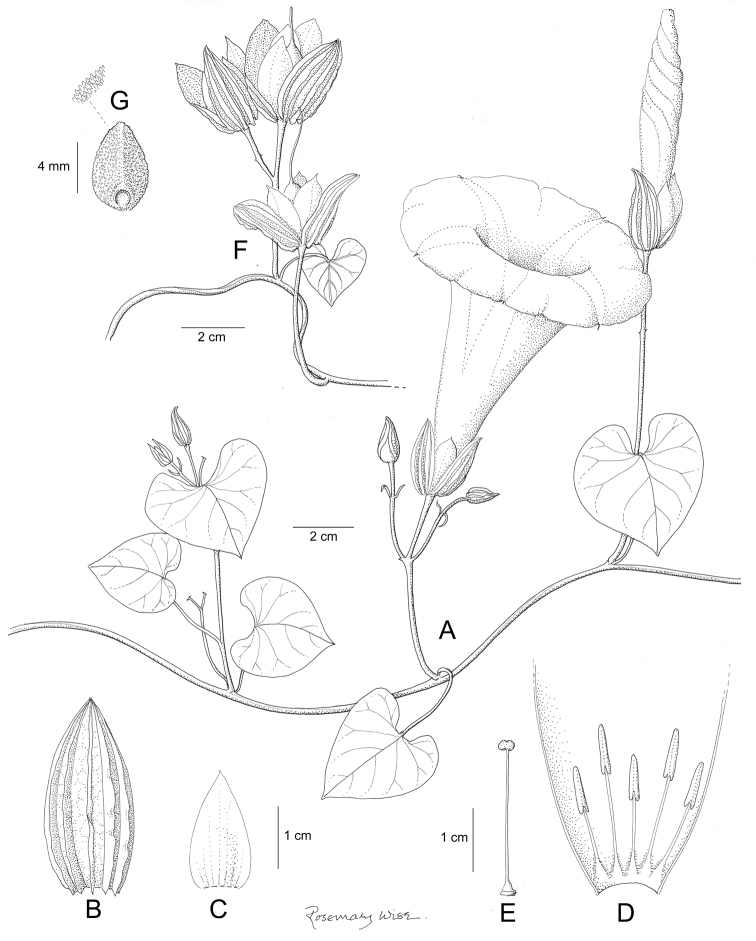
*Ipomoea parvibracteolata*. **A** habit **B** outer sepal **C** inner sepal **D** corolla opened up to show stamens **E** ovary and style **F** fruiting inflorescence with capsule **G** seed. Drawn by Rosemary Wise **A** from *L.P. de Queiroz* 4888; **B–E**, from *L. P. de Queiroz et al.* 9675; **F, G** from *da Silva et al.* 18.

#### Distribution.

Endemic to Brazil and apparently restricted to the area round Petrolina on the borders of Bahia and Pernambuco States in locations under the influence of the Rio São Francisco.

**BRAZIL. Bahia**: Barra, Ibiraba, *L.P. de Queiroz* 4888 (HUEFS); 40 km E de Ramanso, *L.P. de Queiroz et al.* 9675 (HUEFS); Pilão Arcado, *L.P. de Queiroz et al.* 14713 (HUEFS). **Pernambuco**: Arredores de Petrolina, *E.P. Heringer et al.* 80 (IPA, OXF); *M.M. da Silva et al.* 18 (HUEFS, K).

#### Note.

Obviously related to *Ipomoea setifera*, under which name it is usually identified, this species is distinguished by its very small leaves, very large corolla (and other flower parts) and the tiny, linear bracteoles.

• Species 344–347 comprise a distinct clade characterised by the very unequal sepals, which are transversally muricate.

### 344. Ipomoea coriacea

Taxon classificationPlantaeSolanalesConvolvulaceae

Choisy in A.P. de Candolle, Prodr. 9: 358. 1845. (Choisy 1845: 358)

#### Type.

BRAZIL. *J.B. Pohl s.n.* (holotype BR00006972585, probable isotype W).

#### Description.

Undershrub to 2 m, stems ascending or arching stems (rarely (?never) climbing), stout, glabrous, woody. Leaves petiolate, large, coriaceous, 6–14 × 4–9, oblong-elliptic, elliptic or suborbicular, retuse and mucronulate, base broadly cuneate, both surfaces usually glabrous, rarely abaxially tomentellous; petioles 0.4–1 cm. Inflorescence often somewhat “wizened” and scarred, formed of small, pedunculate, somewhat umbellate cymes from the uppermost leaf axils or arising on short lateral branches; peduncle 1–5 cm, stout, often woody; bracteoles triangular, acuminate, 2(–5) mm, moderately persistent; secondary peduncles often present, short, < 10 mm long, woody; pedicels 10–22 mm, relatively slender but widened below calyx; sepals very unequal, glabrous, outer 6–8 × 7 mm, ovate, rounded, muricate, inner 13–16 mm, obovate-elliptic, rounded, somewhat scarious, nearly smooth; corolla 6–7 cm long, funnel-shaped, gradually widened from base, pink, glabrous, limb 3.5–7 cm diam., weakly lobed. Capsules 13 × 8 mm, ovoid, glabrous; seeds not seen.

#### Distribution.

Endemic to the Cerrado biome in the Planalto of Brazil at about 1000 m and almost restricted to Goiás.

**BRAZIL. Goiás**: *G. Gardner* 3710 (K), 3910 (K); *W.J. Burchell* 7944 (K, BR). Niquelândia. *H.S. Irwin* 34669 (MO, NY); ibid., *Cavalcanti et al.* 1580 (CEN, SP); ibid., *B.M.T. Walter* 1210 (CEN, RB); Campinaçu, Faz. Praia Grande, *T. Cavalcanti et al.* 1841 (CEN, NY); Cavalcante. *H.S. Irwin et al.* 24241 (MO, NY); Teresina de Goiás, *W.R. Anderson* 7491 (NY); *M.A. Da Silva et al.* 3359 (IBGE, K). **Tocantins**: Arraias, *A.M. Amorim* 9398 (RB).

#### Note.

A vigorous woody subshrub which differs from *Ipomoea procurrens* in its erect or arching woody stems but the two species are not well-defined.

### 345. Ipomoea procurrens

Taxon classificationPlantaeSolanalesConvolvulaceae

Meisn. in Martius et al., Fl. Brasil. 7: 254. 1869. (Meisner 1869: 254)

Ipomoea procurrens var. pilosula Chodat & Hassl., Bull. Herb. Boiss., ser. 2, 5: 692. 1905. (Chodat and Hassler 1905: 692). Type. PARAGUAY. [San Pedro]. *E. Hassler* 5873 (lectotype G00175045, designated here; isolectotypes BM, G, K, MO, MPU, P, S, UC).

#### Type.

BRAZIL. Minas Gerais, 1845, *J.F.Widgren* 302 (lectotype BR000005307715, designated here; isolectotypes K, M, R, S).

#### Description.

Decumbent, ascending or erect plant with xylopodium, stems somewhat woody, glabrous or, especially on young stems, shortly pubescent. Leaves very shortly petiolate, 2.3–6.5(–14) × 0.6–5.5 cm, very variable in size and shape fom plant to plant, lanceolate, ovate, narrowly or broadly oblong, apex retuse, obtuse or rounded and mucronulate, base broadly cuneate to rounded, slightly asymmetric, both surfaces usually glabrous, veins prominent abaxially; petioles 3–8 mm. Inflorescence of pedunculate, 1–3-flowered cymes from upper leaf axils; peduncles 2–30 mm, glabrous to densely pubescent; bracteoles 4–5 mm, narrowly deltoid; pedicels 5–20 mm, longer than peduncles, sometimes muricate; sepals unequal, outer sepals 7–13 mm, lanceolate to ovate, acuminate to obtuse and mucronate, muricate; inner sepals 11–18 × 4–6 mm, lanceolate to ovate, obtuse and mucronate; corolla 6–7 cm long, funnel-shaped, pink, glabrous, limb c. 5 cm diam., unlobed. Capsules and seeds not seen.

#### Illustration.

Figure 6F.

#### Distribution.

A characteristic cerrado species of the planalto of central Brazil extending to Bolivia and Paraguay.

**PARAGUAY. Amambay**: *E. Hassler* 9760 (BM, K, S); *A. Krapovickas et al.* 45908 (CTES, K); *N. Soria* 7683 (FCQ); *A. Schinini et al.*36061 (CTES). **San Pedro**: *E. Zardini & S. Zavala* 46794 (MO).

**BRAZIL. Dist. Fed.**: *Ferreira* 174 (IBGE, K); *H.S. Irwin* 26635 (MO, NY, RB, W). **Goiás**: Cristalina, *J.R. Pirani* 1528, 1614 (K, SPU); Serra da Ortiga, *G. Hatschbach* 33324 (MO, RB); Niquelândia, *B. Walter* 1382 (CEN, RB); Ituiutaba, *A. Macedo* 31 (MO, NY, R); Alto Paraíso, *W.R. Anderson* 6235 (NY). **Mato Grosso**: 27 km N of Xavantina, *D.R. Gifford* 82 (K); 4.5 km S of Xavantina, *D. Philcox & A. Ferreira* 3737 (K); 60 km N of Xavantina, *H.S. Irwin et al.* 15983 (MO, NY); Pedro Gomes, *G. Hatschbach* 37422 (RB). **Mato Grosso do Sul**: *A. Krapovickas & C. Cristóbal* 34305 (CTES, G); *E.P. Heringer et al.* 934 (IBGE, FTG). **Minas Gerais**: *A.A. Arbo et al.* 3188 (CTES, K); Morro das Pedras, *H.S, Irwin et al*. 25559 (MO, NY); Serra do Rio Preto, *H.S. Irwin* 10298 (NY); Formoso, *M.A. da Silva* 3680 (RB). **São Paulo**: *C.W. Mosén* 4284 (S).

**BOLIVIA. Santa Cruz**: Velasco, P.N. Noel Kempff Mercado, *W.W. Thomas et al.* 5595 (FTG, NY); *J.R.I. Wood et al.* 25231 (K, LPB, UB, USZ).

#### Notes.

Although rather variable in habit and in leaf and sepal shape this species is usually recognised easily by the shortly petiolate, oblong to ovate leaves with a cuneate base and the distinctive muricate outer sepals.

The type of var. pilosula is very atypical with large flowers and scarcely muricate sepals. It is one of a number of atypical specimens found by Hassler in Paraguay but never recollected.

### 346. Ipomoea paludicola

Taxon classificationPlantaeSolanalesConvolvulaceae

J.R.I. Wood & Scotland, Kew Bull. 70 (31): 24. 2015. (Wood et al. 2015: 24)

Ipomoea serpens Meisn. in Martius et al., Fl. Brasil. 7: 275. 1869. (Meisner 1869: 275), nom. illeg., non Ipomoea serpens L. (1759). Type. BRAZIL. Minas Gerais, Rio das Velhas, J.B. Pohl 3173 (lectotype W0052417, designated by Wood et al. 2015: 24, isolectotype W).

#### Type.

Based on *Ipomoea serpens* Meisn.

#### Description.

Erect, twining or trailing herb, glabrous in all vegetative parts; rootstock stout and somewhat tuberous; stems slightly succulent, often rooting at nodes. Leaves petiolate, sagittate, often strongly so, the auricles linear to lanceolate, acuminate or less commonly, rounded, 2–4 × 0.2–6 cm, the blade (excluding auricles) 2.5–7.5 × (0.1–) 1.7–1.9 cm, lanceolate, narrowly to broadly oblong or oblong-elliptic, apex obtuse and mucronulate, green on both surfaces but somewhat darker adaxially; petioles 2–5 cm. Inflorescence of shortly pedunculate, axillary cymes, often reduced to a single flower; peduncles 0.5 –3.5 cm; bracteoles 1–1.5 × 0.2 mm, deltoid, caducous; pedicels 8–15 mm; sepals very unequal, outer sepals 4–7 × 3–3.5 mm, oblong, obtuse to rounded, mucronate, the mucro deciduous, dark green, often transversely muricate, margin scarious, inner sepals much larger, 8–14 × 5 mm, broadly oblong-obovate, rounded or retuse and mucronulate, the mucro deciduous, conspicuously pallid and subscarious; corolla 7–8.5 cm long, pink, glabrous, funnel-shaped, limb 4–5 cm diam., unlobed. Capsules 8 × 8 mm, ovoid, glabrous; seeds 4.5 × 3 mm, blackish, minutely puberulent.

#### Illustration.

Figures 141F, 165.

**Figure 165.**
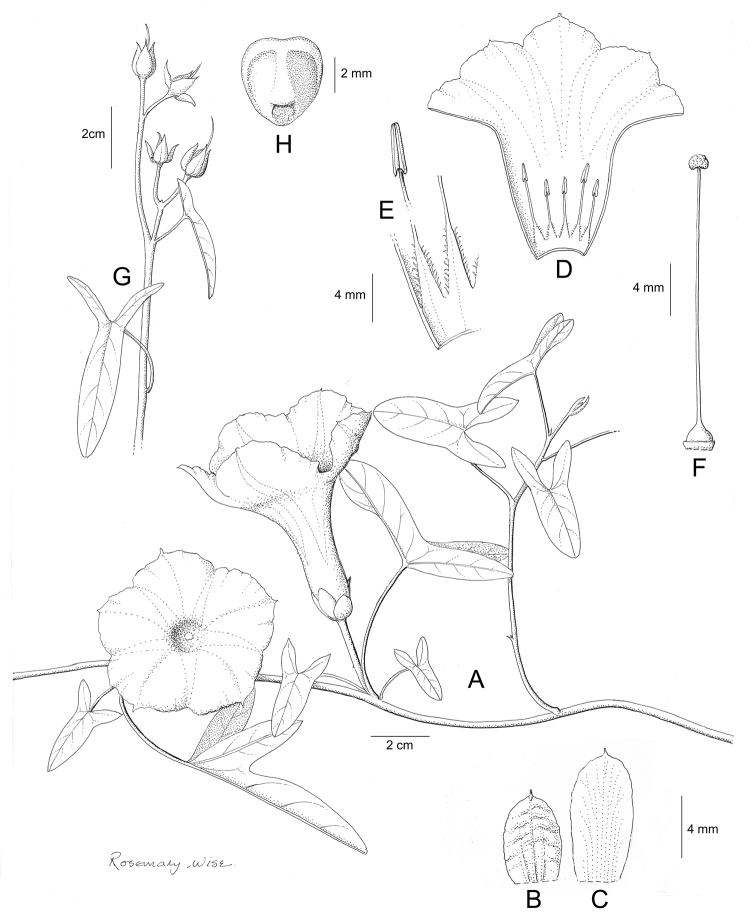
*Ipomoea paludicola*. **A** habit **B** outer sepal **C** inner sepal **D** corolla opened out to show stamens **E** detail of anther and filament base **F** ovary and style **G** shoot with fruiting inflorescence **H** seed. Drawn by Rosemary Wise **A–F** from *Wood & Huaylla* 20763; **G, H** from *Guillén & Choré* 1446.

#### Distribution.

Common in Bolivia on seasonally flooded lowland plains in parts of the Beni, the Río Paraguá basin around the Noel Kempff Park and in Brazil in the Pantanal. It is also extends along the Río Paraguay into Paraguay and occurs in Minas Gerais and Bahia states in Brazil as well as in Venezuela and north to Costa Rica. It may be more widespread that the following records suggest.

**PARAGUAY. Cordillera**: 47 km W de Caacupé, *F. de la Puente* 3599 (CIP-Lima). **Paraguarí**: 1901/2, *E. Hassler* 7680 (BM). **San Pedro**: Distr. Lima, Estancia Carumbe, *T.M. Pedersen* 9460 (MBM); Rosario, *E. Zardini & L. Guerrero* (ARIZ, MO).

**BRAZIL. Amazonas**: Manaos, *E. Ule* 8955 (K). **Bahia**: 3 km de Campo Alegre de Lourdes, *Nunes et al.* 420 (ARIZ, HUEFS). **Mato Grosso**: Mun. Cáceres, Pantanal, 1976, *Dobreimer & Tokarnia* 1255 (R). **Mato Grosso do Sul**: Corumbá, *A. Pott et al.* 2436 (MBM); *A. & V.J. Pott* 7678 (CPAP); ibid., *V. J. Pott et al.* 1353 (CPAP); Mun. Poconé, *A. Pott et al.* 4808 (MBM); *A. Pott* 5036 (CPAP, MBM); *V.J. Pott et al.* 1716 (CPAP). **Minas Gerais**: Rio Das Velas, *J.B. Pohl* 2173 (W); 2 km de Januaria, *Merdes Maghalães* 6087 (RB); Itacarambi, *O.S. Ribas & J.M. Silva* 7772 (MBM);

**GUYANA.** Rapununi River, Dadanawa, *M.L. Jansen-Jacobs et al.* 5612 (ARIZ, MO, P).

**BOLIVIA. Beni**: Cercado, Ibiato, *M.T. Martinez et al.* 81 (K, LPB, USZ); Yacuma, Santa Ana de Yacuma, *M. Atahuachi et al.* 985 (BOLV, LPB). **Santa Cruz**: Velasco, El Toledo, *J.R.I. Wood & H. Huaylla* 20763 (HSB, K, LPB, USZ); Pampas de San Ramón, *S.R.P. Halloy et al.* 4307 (NY).

**VENEZUELA. Apure**: Est. Biológica “El Frio”, *S. Castroviejo & Ginés López* 142 (MA); Muños, 63 km W of Mantecal, *G. Aymard et al.* 5051 (MO); Mantecal, *B. Stergios* 2380 (MO). **Bolívar**: El Palmar, Hac. Costa Rica, *C. Sastre et al.* 8558 (P).

**COSTA RICA.** Guanacaste, *L. D. Gómez* 18943 (COL, MO); ibid.; Cantón de la Cruz, de Bahia Salinas a Santa Cecilia, *E. López & M. Segura* 92 (MO, K).

**MEXICO. Tabasco**: Huimanguillo, *E. Lott et al.* 1352 (IEB, MEXU, MO).

#### Note.

This species has usually been included within *Ipomoea asarifolia* and is clearly closely related but is easily distinguished by the sagittate rather than suborbicular, reniform leaves. Molecular data (Muñoz-Rodríguez et al. 2019) suggests that *Ipomoea paludicola* is sister to *Ipomoea procurrens*, being more closely related to that species than to *I. asarifolia*.

Various forms of *I. paludicola* can be encountered. Where it is growing among bushes it occurs as a climbing plant. On open flood plain it is usually trailing and rooting at the nodes, but erect flowering specimens occur during the dry season.

### 347. Ipomoea asarifolia

Taxon classificationPlantaeSolanalesConvolvulaceae

(Desr.) Roem. & Schult., Syst. Veg. 4: 251. 1819. (Roemer and Schultes 1819: 251)

Convolvulus asarifolius Desr. in Lam., Encycl. 3: 562. 1792 [dated 1789]. (Desrousseaux 1792: 562). Type. SENEGAL. Roussillons.n. (holotype P-LAM00357544, isotype P-JUSS-6798).
Amphione asarifolia Raf., Fl. Tellur. 4: 79. 1836[1838]. (Rafinesque 1838a: 79).
Convolvulus rugosus Rottler, Ges. Naturf. Freunde Berlin Neue Schriften 4: 196. 1803. (Rottler 1803: 196). Type. INDIA. Marmelon, *J.P. Rottler s*.n. (holotype B-W03683-01).
Ipomoea rugosa (Rottler) Choisy, Mém. Soc. Phys. Genève 6: 446 [64]. 1834. (Choisy 1834: 446[64]).
Ipomoea crassifolia Cav., Descr. Pl. 100. 1802. (Cavanilles 1801–1802: 100). Type. Plant grown at Madrid from seeds sent by Ruiz and Pavón (lectotype MA475846, designated here).
Ipomoea beladamboe Roem. & Schult., Syst. Veg. 4: 233. 1819. (Roemer and Schultes 1819: 233). Type. Icon of Beladamboe in Rheede Malabar 11: 119 t. 58, lectotype, designated here.
Convolvulus beladambu (Roem. & Schult.) Spreng., Syst. Veg. 1: 608. 1825 [pub. 1824]. (Sprengel 1824: 608).
Ipomoea latifolia M. Martens & Galeotti, Bull. Acad. Roy. Sci. Bruxelles 12: 266. 1845. (Martens and Galeotti 1845: 266). Type. MEXICO. [Veracruz] Cordoba, *H. Galeotti* 1401 (holotype BR00006973186, isotypes BR, G).
Ipomoea nymphaeifolia Griseb., Cat. Pl. Cub. 203. 1866. (Grisebach 1866: 203), nom. illeg., non Ipomoea nymphaeifolia Blume (1826). Type. CUBA. *C. Wright* 3089 (holotype GOET002499, isotypes GH, K, MO, MPU, NY, S, US).
Ipomoea grisebachii Prain, J. Asiat. Soc. Bengal, part 2, Nat. Hist. 63(2): 107. 1894. (Prain 1894: 107). Type. Based on Ipomoea nymphaeifolia Griseb.
Ipomoea urbica Salzm. ex Choisy in A.P. de Candolle, Prodr. 9: 349. 1845. (Choisy 1845: 349). Type. BRAZIL. Bahia, *Martius* 2020 (lectotype M0184909, desigated here).
Ipomoea urbica var. muricata Choisy in A.P. de Candolle, Prodr. 9: 350. 1845. (Choisy 1845: 350). Type. BRAZIL. Illheos, Blanchet 3046 (P?, not found).
Ipomoea pes-caprae var. heterosepala Chodat & Hassl., Bull. Herb. Boiss., ser. 2, 5: 692. 1905. (Chodat and Hassler 1905: 692). Type. PARAGUAY. [Concepción] Y-cua-pona, *E. Hassler* 7680 (isotypes S12-1297, UC).

#### Type.

Based on *Convolvulus asarifolius* Desr.

#### Description.

Trailing glabrous perennial rooting at the nodes, stems much branched, stout, angled, fleshy. Leaves petiolate, 2.5–8(–9) × 3–9(–11) cm, reniform, suborbicular, obtuse to rounded, base truncate to shallowly cordate with rounded auricles, usually folded when pressed, veins radiating from base, glabrous; petioles 2.5–9 cm. Inflorescence of pedunculate axillary cymes with up to 10 flowers, flowers often solitary, but sometimes umbellate from apex of peduncle; peduncles 0.5–7 cm, angular; bracteoles 1–2 mm, deltoid; pedicels often rather short, 5–25 mm; sepals unequal, elliptic, obtuse to emarginate and mucronate, outer 5–9 × 4 mm, often somewhat muricate, inner 9–15 × 6–7 mm, elliptic, ±scarious; corolla 5–6 cm long, funnel-shaped, white, yellow-green or pink with darker centre, glabrous, limb 3.5–4 cm diam., unlobed. Capsules glabrous, suborbicular, 10–12 × 8–10 mm, the slender style somewhat persistent; seeds 5–7 × 4 mm; minutely tomentellous (appearing glabrous under a hand lens).

#### Illustration.

Austin (1998: 402).

#### Distribution.

Widespread in the Americas, West Africa and Asia, but apparently absent from east and South Africa, Madagascar and China and many areas of the Americas. It grows in disturbed wet places, often near the coast or inland near large rivers; it is sporadic in occurrence.

**PARAGUAY.** North: Villa Socna, between Río Apa and Río Aquidaban, 1908–9, *K. Fiebrig* 5008 (K, P). **Concepción**: *I. Basualdo* 3782 (FCQ). **San Pedro**: Com. 25 de Diciembre, *J.R.I. Wood & G. González* 28472 (FCQ); Puerto Rosario, *A.F. Woolston* 1166 (K, NY, S)

**BRAZIL. Amapá**: *D.F. Austin et al*. 6965 (MG, MO, NY). **Amazonas**: *Pabst* 9432 (K); *A. Lasseign* P21174 (MO, NY, S); *P. & H. Maas* 367 (K, MO); Manãus, *E.P. Killip* 30046 (NY). **Bahia**: *Blanchet* s.n. (BM, NY); *M.M. Arbo et al.* 7366 (CTES, NY). **Ceará**: *F.E. Drouet* 2491 (F, K, MO, NY, S). **Maranhão**: *G & L.T. Eiten* 4570 (K). **Pará**: *B.A. Krukoff* 5863 (K, NY); *T. Croat* 62098 (MO); *S. Tsugaru & Y. Sano* B-510 (MO, NY). **Paraíba**: *M.F. Agra* 1168 (K). **Pernambuco**: *G. Gardner* 1072 (BM, K, P); *B. Pickel* 3709 (NY). **Piauí**: *L. Coradin et al*. 5859 (CEN, K); Teresina, *F. Chagas & Silva* 57 (IBGE, K, MO). **Rio Grande de Norte**: *M.T. Dawe* 6 (K). **Rondônia**: *G.T. Prance et al.* 5896 (K, NY, S).

**FRENCH GUIANA.** Oyapock River, *G. Léotard* 1240 (CAY).

**PERU. Ancash**: *P. Francia* 144 (MO). **Cajamarca**: *R. Ferreyra* 7057 (K); *A. Sagástegui* 14479 (MO); *C. Vargas* 10397 (CUZ). **Huánuco**: *R. Bird* 1517 (MO). **Ica**: Mun. Ocucaje, *O. Whaley et al.* 571 (K). **La Libertad**: *A. Sagástegui* 14911 (MO); *I. Sánchez Vega* 4337 (F). **Lambayeque**: *P.C. Hutchison & Wright* 3365 (K, P, S, UC, USM); *J. Hudson* 948 (CTES, MO); *R. Ferreyra* 7609 (USM). **Lima**: *H. Cuming* 975 (BM). **Piura**: *R. Ferreyra* 10760 (MO); *O. Haught* F-177 (F); *O. Haught* 210 (BM, US); *M.S. Chrostowski* 5/1 (K); *C.R. Worth et al.* 9007 (K, UC). **Tumbes**: *R. Ferreyra* 12330 (MO, USM); *A. Gentry & C. Diáz* 58179 (NY, MO).

**ECUADOR. Chimbarazo**: Pallatanga-Panza Gorda, *J. Jaramillo et al.* 26869 (QCA). **Guayas**: Guayaquil, *R. Spruce* 6319 (BM, K); *K.T. Hartweg* 674 (BM, K, NY. P); *Pavón* s.n. (BM); *E. Asplund* 15609 (K, S); Cañaveral, *J.E. Madsen* 7401 (AAU). **Loja**: *G. Harling & L. Andersson* 22533 (MO); *J.E. Madsen et al.* 7401 (AAU). **Manabí**: *G. Harling et al.* 9496 (MO).

**COLOMBIA. Montería**: *B. Anderson* 1849 (K).

**VENEZUELA. Bolívar**: *J. Steyermark* 88866 (NY, K).

**PANAMA.***B.L. Seeman* 173 (K); *J.F. MacBride* 2674 (F); *A.A. Hunter & P.H. Allen* 469 (P).

**NICARAGUA.***W.D. Stevens* 27852 (MO).

**MEXICO. Chiapas**: Tonalá, *R.E. Gereau & G.J. Marin* 1845 (MO). **Quintana Roo**: Isla de Cozumal, *E.F. & H. de Cabrera* 6817 (MEXU, MO).

**UNITED STATES. Florida**: *K. Craddock Burks et al.* 1159 (FSU, FTG), 1074 (FSU).

**CUBA.***P. Wilson* s.n. [22/8/1904] (HAJB); **Isla de Juventud [Pinos**]: *A.H. Curtiss* 219 (BM, K, MO, P). **Pinar del Río**: *Bro. Alain* 2805 (NY). **La Habana**: *H. Van Hermann* 384 (BM); **Camagüey**: *N.L. Britton et al.* 13084 (NY). **Guantánamo**: Bayate, *E.L. Ekman* 10027 (NY), 15316 (S).

**JAMAICA.***W. Stearn* 280 (BM), 982 (BM); *G.R. Proctor & Mullings* 21824 (BM); *W.H. Harris* 11830 (MO, NY); *T.G. Yuncker* 18030 (NY).

**LESSER ANTILLES. Martinique**: *C. Sastre* 9868 (P). **St Lucia**: *G.R. Proctor* 17693 (A, BM); *R.A. Howard et al.* 20004 (NY).

#### Typification.

In designating a lectotype of *Ipomoea crassifolia* we have chosen the specimen cultivated in Madrid (MA475846) and annotated “Ipomoea crassifolia” as the description was based on this, rather than the original collection by Ruiz and Pavón from Guayaquil, which is also kept at Madrid (MA814663).

#### Notes.

The folded reniform leaves are very characteristic.

Records from Bolivia (Wood et al. 2014) are errors for *Ipomoea paludicola*.

### 348. Ipomoea leptophylla

Taxon classificationPlantaeSolanalesConvolvulaceae

Torr. in J.C. Frémont, Rep. Exped. Rocky Mts. 94. 1845. (Torrey 1845: 94)

Convolvulus caddoensis Buckley, Proc. Acad. Nat. Sc. Philadelphia 165: 6. 1862 [pub. 1863]. (Buckley1863: 6). Type. UNITED STATES. [Texas], Addo peak in NW Texas, *Durand*s.n. sine data (probable holotype PH00006612).

#### Type.

*J.C. Frémont* s.n. (holotype NY00319064, isotypes K, NY).

#### Description.

Erect branched undershrub, stems glabrous, yellowish, rootstock massive, spindle-shaped, woody. Leaves subsessile, 3.5–10 × 0.2–0.6 cm, narrowly oblong, obtuse and mucronate, base cuneate, glabrous; petioles 2–6 mm. Inflorescence of few-flowered axillary cymes; peduncles 0.5–3.5 cm, rather stout; bracteoles 1–3 mm, deltoid, caducous; pedicels 7–15 mm, thickened upwards and of different texture to peduncle; sepals unequal, outer 5–8 mm, ovate, obtuse with scarious margins, inner similar but 10–12 mm, broadly elliptic and more rounded; corolla 5.5–7 cm long, funnel-shaped, glabrous, pink, limb entire, 4.5–7 cm diam. Capsules 14 × 14 mm, subglobose, rostrate with 6 mm long mucro, glabrous, much larger than calyx; seeds 10 × 4 mm, brown, tomentellous.

#### Illustration.

**Figure 166.**
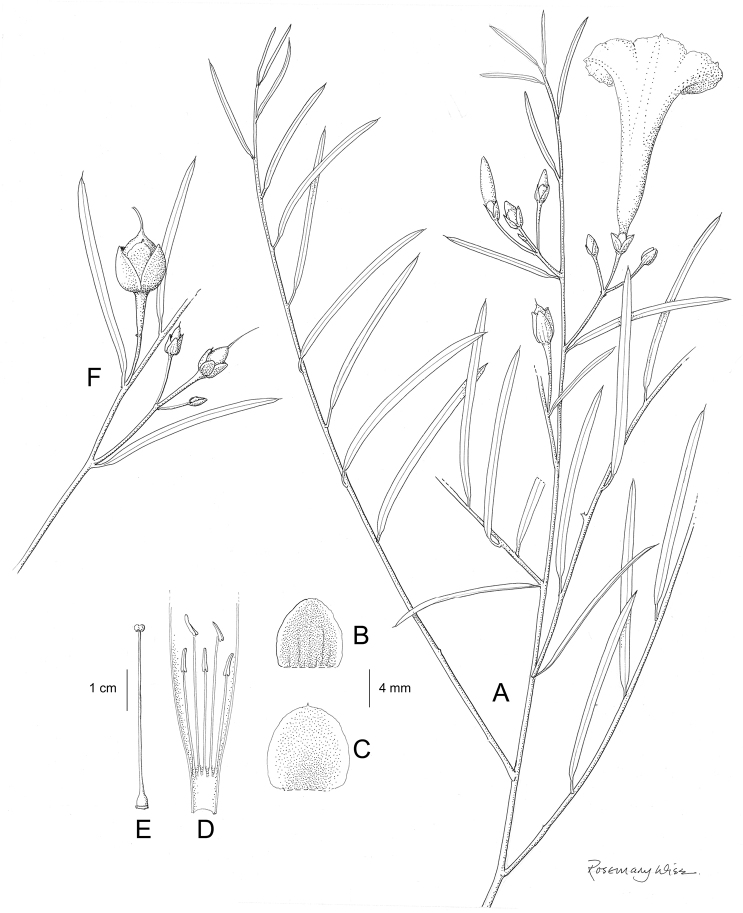
*Ipomoea leptophylla*. **A** habit **B** outer sepal **C** inner sepal **D** corolla opened out to show stamens **E** ovary and style **F** fruiting calyx and capsule. Drawn by Rosemary Wise **A–C** E from *Fendler* 660; **D** from *Eastwood* s.n.; **F** from *Lindheimer* fasc. 4: 66.

Figure 166; Haddock et al. (2015: 235).

#### Distribution.

Prairie region of Midwest United States extending into northern Mexico. It is usually found in short grassland on sandy or gravelly soil mostly between 1000 and 1900 m.

**MEXICO. Chihuahua**: 10 km W of Chihuahua, *L. McGill* 8280 (ASU).

**UNITED STATES. Colorado**: Denver, *J.L. Wingate* 8527 (KHD); Adams Co., *D. Demaree* 29534 (BM); North Denver, *Eastwood* 39 (K). **Kansas**: *B. Kuhn* 7375 (RM). **Nebraska**: Sheridan, *S.M. Clarke* 15-17 (BRY). **New Mexico**: Kiowa Nat. Grassland, *Van Devender* 84-377 (ARIZ); *Fendler* 660 (BM). **Oklahoma**: Glass Mts., *M. Fishbein* 6913 (ARIZ). **South Dakota**: hot springs at Mammoth site, *M. Nee* 21515 (NY, FTG); Black Hills, *P.A. Rydberg* 903 (K). **Texas**: *Lindheimer* fasc. 4: 661 (BM, OXF, P); Gaines Co, *H.S. Gentry* 20616 (ARIZ). **Wyoming**: Goshen, Fort Laramie, *B.C. Buffum* s.n, 5/9/1892 (RM); *E.W. Nelson* 2575 (BM, US).

#### Note.

Differs from *Ipomoea longifolia* in the linear-oblong leaves and shorter sepals. The rootstock is reported to be “massive” with the “diam. of a telephone pole” (Weber and Wittmann 2012: 166).

### 349. Ipomoea shumardiana

Taxon classificationPlantaeSolanalesConvolvulaceae

(Torr.) Shinners, S.W. Naturalist 6(2): 101. 1961. (Shinners 1961: 10)

Convolvulus shumardianus Torr. in R.B. Marcy, Explor, Red River Louisiana 291–2. 1853. (Marcy 1853: 291). Type. UNITED STATES. Marcy’s expedition 17 July 1852, sine col. (NY00318908).
Ipomoea shumardii Torr. in R.B. Marcy, Explor. Red River Louisiana 191. 1854, nom. nud., printing error.
Ipomoea carletonii Holz., Contr. U.S. Natl. Herb 1: 211. 1892. (Holzinger 1892: 211). Type. UNITED STATES. Oklahoma, Logan Co., Guthrie, *M.A. Carleton* 472 (holotype US00111374, isotypes K, US).

#### Type.

Based on *Convolvulus shumardianus* Torr.

#### Description.

Glabrous twining perennial. Leaves petiolate, 2–6 × 0.7–2.5 cm, broadly to narrowly ovate-deltoid to rhombic, truncate, rounded to cuneate at base, widest near base, acuminate, mucronate; petioles 0.8–3 cm. Inflorescence of few-flowered, pedunculate cymes; peduncles 0.5–6 cm; bracteoles 2–3 mm, ovate-deltoid, acuminate, somewhat persistent; pedicels 5–12 mm; sepals unequal, outer ovate, obtuse and mucronate, ribbed, 8–9 mm, inner 11–12 mm, oblong-ovate, pale and ± scarious, acute to apiculate; corolla 5–8 cm long, broadly trumpet-shaped, gradually widened from base, pink, glabrous, the tube 3.5–4.5 cm, limb undulate c. 6–7 cm diam. Capsules and seeds not seen.

#### Illustration.

Diggs et al. (1999: 559).

#### Distribution.

A local endemic found on the borders of Oklahoma and Texas.

**UNITED STATES. Oklahoma**: Logan County, 1.25 miles S of Mulhall, *R. Pearce* 1799 (ARIZ); Sandy Loam, *K.C. Bennett* s.n. (KH); Payne County, 3 miles S. of Mulhall, *J.C. Semple & K. Shea* 675 (MO). **Texas**: Cooke County, half mile N of Dexter, *R. Pearce* 2081 (ARIZ).

#### Note.

*Ipomoea shumardiana* differs from *I. leptophylla* in the distinct leaf base. The two species intergrade and some specimens, e.g. *Semple & Shea* 675 are somewhat intermediate. *Ipomoea shumardiana* may prove to be only a form of *I. leptophylla*.

### 350. Ipomoea pandurata

Taxon classificationPlantaeSolanalesConvolvulaceae

(L.) G. Mey., Prim. Fl. Esseq. 100. 1818. (Meyer 1818: 100)

Convolvulus panduratus L., Sp. Pl., ed. 1: 153. 1753. (Linnaeus 1753: 153). Type. UNITED STATES. Virginia, Clayton 641 (lectotype BM000051711, selected by Staples & Austin in Staples and Jarvis 2006: 1021).
Convolvulus ciliolatus Michx., Fl. Bor.-Amer. 1: 137. 1803. (Michaux 1803: 137). Type. UNITED STATES. Tenessee, Knoxville, *Michaux*s.n. (lectotype P00320303, designated here).
Ipomoea ciliolata (Michx.) Pers., Syn. Pl. 1: 183. 1805. (Persoon 1805: 183).
Ipomoea ciliosa Pursh, Fl. Amer. Sept. 1: 146. 1813 (Pursh 1813: 146), nom. illeg., superfl. Type. Based on Convolvulus ciliolatus Michx.
Convolvulus candicans Solander ex Sims, Bot. Mag. 39, pl. 1603. 1813. (Sims 1813: pl. 1603). Type. Specimen grown at Kew in 1776 (Herb Banks BM, not found).
Ipomoea candicans (Solander ex Sims) Sweet, Hort. Brit. 289 (1826). (Sweet 1826: 289).
Ipomoea pandurata var. candicans (Solander ex Sims) Choisy Prodr. [A.P. de Candolle] 9: 381. 1845. (Choisy 1845: 381).
Ipomoea pandurata var. rubescens Choisy in A.P. de Candolle, Prodr. 9: 381. 1845. (Choisy 1845: 381). Type. UNITED STATES. Kentucky, Boonsborough, *R. Peter*s.n. (holotype G00135789).
Ipomoea karwinskiana Regel, Index Seminum [St. Petersburg] 46. 1857. (Regel 1857: 46). Type. Plant cultivated at St Petersburg grown from seed sent by Karwinsky from Mexico (lectotype LE01025977, designated here).
Ipomoea pandurata forma leviuscula Fernald, Rhodora 51: 75. 1949. (Fernald 1949: 75). Type. Based on Ipomoea pandurata var. rubescens Choisy
Ipomoea pandurata var. hastata Chapm., Fl. South. U.S. 343. 1860 (Chapman 1860: 343). Type. UNITED STATES. Florida to Mississippi, *Chapman*s.n. (whereabouts uncertain).
Ipomoea schrenkiana A. Peter, Nat. Pflanzenfam. 4 (3a): 30. 1897 [pub. 1891]. (Peter 1891: 30). Type. UNITED STATES. New York, Flushing, Long Island, *J. Schrenk*s.n. (lectotype GOET005717, designated by Staples et al. 2012: 675).

#### Type.

Based on *Convolvulus panduratus* L.

#### Description.

Trailing or twining perennial herb; stems glabrous to puberulent, rootstock an enlarged, woody tuber. Leaves petiolate, 2–14 × 1.8–9.5 cm, ovate-deltoid, sometimes weakly to strongly 3-lobed, cordate with rounded auricles, shortly acuminate, both surfaces glabrous or puberulent, especially on the veins, abaxially paler often with reddish veins; petioles 1–6 cm, glabrous. Inflorescence of usually short, few-flowered axillary cymes; peduncles 0.6–5(–14) cm, glabrous; bracteoles 2–11 × 0.5–6 mm, linear, oblong or oblong-obovate, papery, deciduous; secondary peduncles c. 10 mm; pedicels 5–11 mm; sepals unequal to almost equal, outer sepals 10–15(–20) × 4–7(–9) mm, oblong–ovate, obtuse, abaxially usually with prominent raised vertical veins, the base subtruncate, inner 15–18(–22) × 5–7 mm, oblong-ovate, rounded; corolla 4–7 cm long, white with dark pink centre, glabrous, campanulate to funnel-shaped, limb 5–6 cm diam., undulate or lobed, the midpetaline bands terminating in small teeth. Capsules 10–15 × 6–10, narrowly ovoid, glabrous; seeds 5 × 3 mm, pilose with brownish hairs c. 5 mm long.

#### Illustration.

**Figure 167.**
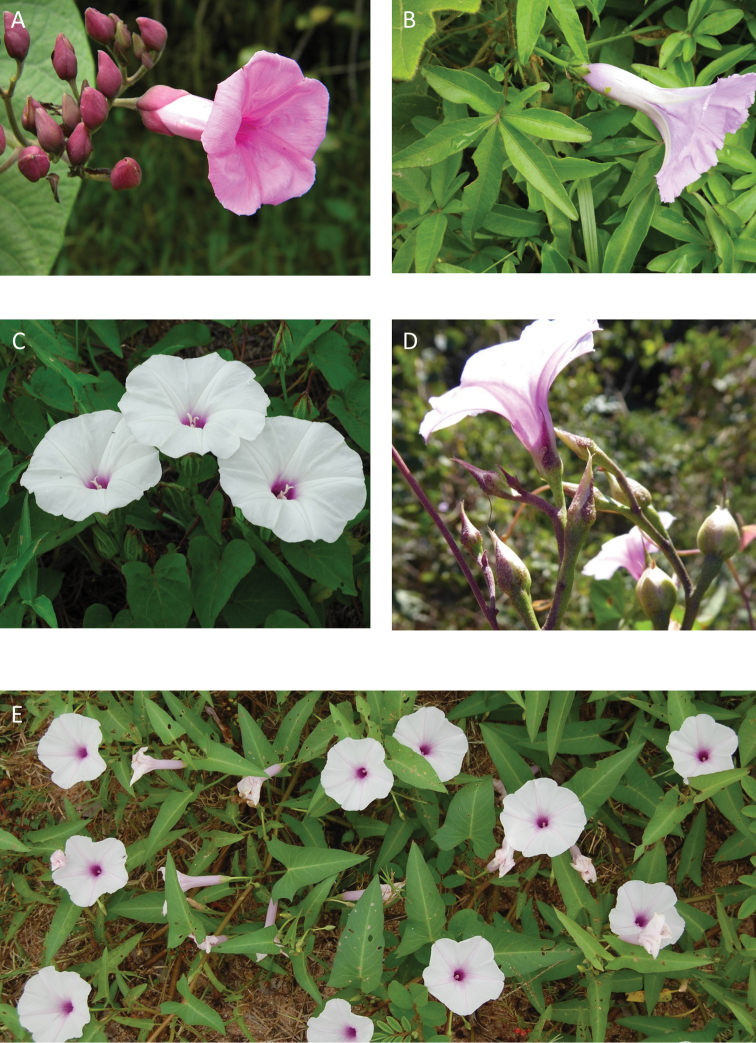
Photographs of *Ipomoea* species. **A***I. philomega***B***I. cairica***C***I. pandurata***D***I. acanthocarpa***E***I. aquatica*. **A**http://faunaandfloraofvietnam.blogspot.com**B** John Wood **C** Steve Turner **D** Maira Martinez **E** Wikipedia Commons.

Figure 167C; Haddock et al. (2015: 235).

#### Distribution.

Widespread in the eastern United States extending west to Texas, Kansas and Illinois and just entering Canada (Ontario). It is a plant of open grassy places, roadsides, woodland margins and remains of prairie grassland at low altitudes.

**UNITED STATES. Alabama**: *S.T. McDaniel* 9039 (IBE, MO). **Arkansas**: *D.E. Atha* 12341 (NY). **Florida**: Manatee River, *F. Rugel* [1845] (BM); *G.V. Nash* 777 (K). **Georgia**: *R. Ware* 112 (GA). **Illinois**: Tazewell Co., *V.H. Chase* 10069 (BM). **Indiana**: *Friesner* 22758 (S). **Kansas**: *B. Rohrer* 60 (S). **Kentucky**: *Biltmore*1279b (S); *E.M. Browne* 72H31.5 (EKY); Boonsborough, *R. Peter* s.n. [8/1834] (K). **Louisiana**: Covington, *Drummond* s.n. [1832] (BM); *L. Chance* 930 (MISSA). **Maryland**: *T. Holm* s.n. [8/7/1921] (S); *Petrak* 1950 (S). **Mississippi**: *Seymour* 172 (S). **Missouri**: Busiek State Forest, *K. Sykes & J. Stone* 10 (BM, MO); Ozarks, Jefferson Co., *P.H. Raven* 27200 (BM, MO). **New Jersey**: Princeton, *Moldenke* 8673 (BM). **New York**: *Whitford* 166 (NY). **North Carolina**: Swift Creek, *H. Ahles & B. Carswell* 58689 (BM, UNC); Biltmore, *Gadeceau* 1279 (BM). **Ohio**: *R.M. Lowden* 4226 (LSU). **Oklahoma**: *Handler* 375 (S); *Stevens* 1356 (K). **Pennsylvania**: *Moldenke* 20495 (S). **South Carolina**: *Meyer & Townesmith* 1038 (PH, MO); *J. Nelson* 28619 (USCH). **Texas**: Bowie, along Red River, *D.S. Correll* 31242 (MO). **Virginia**: *A.H. Curtiss* s.n. 3/8/1871 (K). **West Virginia**: *J. Donnell Smith* s.n. [12/9/1879] (S).

**CANADA. Ontario**: Lewiston, *R.B. Thompson* 1924 (BM); Lake Erie, *Burgess* 1594 (BM).

#### Lectotypification.

We have selected the Michaux collection from Knoxville, Tennessee at P as lectotype of *Convolvulus ciliolatus* as it appears to be the only extant specimen that fits the protologue.

#### Notes.

Very variable particularly in the leaf shape (entire to deeply lobed) and in the relative and absolute sizes of the sepals as also in the size of the corolla and leaves, although the leaves are usually small (c. 5 cm long).

Distinguished by the white corolla with a pink throat and the prominently veined sepals. *Ipomoea candicans* is a form in which the abaxial leaf surface is white-tomentellous.

### 351. Ipomoea sagittata

Taxon classificationPlantaeSolanalesConvolvulaceae

Poir., Voy. Barbarie 2: 122. 1789. (Poiret 1789: 122)

Convolvulus speciosus Walter, Fl. Carol. 93. 1788, (Walter 1788: 93), nom. illeg., non Convolvulus speciosus L.f. (1782). Type. UNITED STATES, not specified.
Convolvulus sagittifolius Michx., Fl. Bor.-Amer. 1: 138. 1803. Type. Based on Convolvulus speciosus Walter
Ipomoea sagittifolia (Michx.) Ker-Gawl., Bot. Reg. 6: t. 437. 1820. (Ker-Gawler 1820: t.437). Type. Based on Convolvulus speciosus Walter
Convolvulus wheleri Vahl, Symb. Bot. 2: 36. 1791. (Vahl 1791: 36). Type. SPAIN. Valencia, La Albufera, Barnadess.n. (lectotype C10009688, designated by Austin & McDonald 2014 (44): 1).

#### Type.

ALGERIA. Souk, *Desfontaines* s.n. (holotype P00680360).

#### Description.

Glabrous, perennial trailing or twining herb, stems slender. Leaves petiolate, deltoid-sagittate, the central lobe lanceolate, acuminate to a mucronate point, 2–6.5 × 0.2–1.5 cm (excluding auricles), the two auricles similar in shape but slightly shorter than the main part of blade, narrowly oblanceolate, acute, apex finely acuminate; petioles 1–4.2 cm. Inflorescence of solitary (very rarely paired), axillary flowers; peduncles 5–12(–26) mm; bracteoles 1–2 mm, ovate, acute, caducous; pedicels 13–16(–20) mm, thickened upwards; sepals unequal, outer 7–8 × 3–5 mm, oblong-elliptic, rounded, mucronate, margins narrow, scarious, inner 9–12 × 5–7 mm, oblong-elliptic, rounded, mucronulate, subscarious; corolla 4–7 cm long, funnel-shaped, pink, glabrous, limb 4.5–6 cm diam., entire. Capsules and seeds not seen.

#### Illustration.

**Figure 168.**
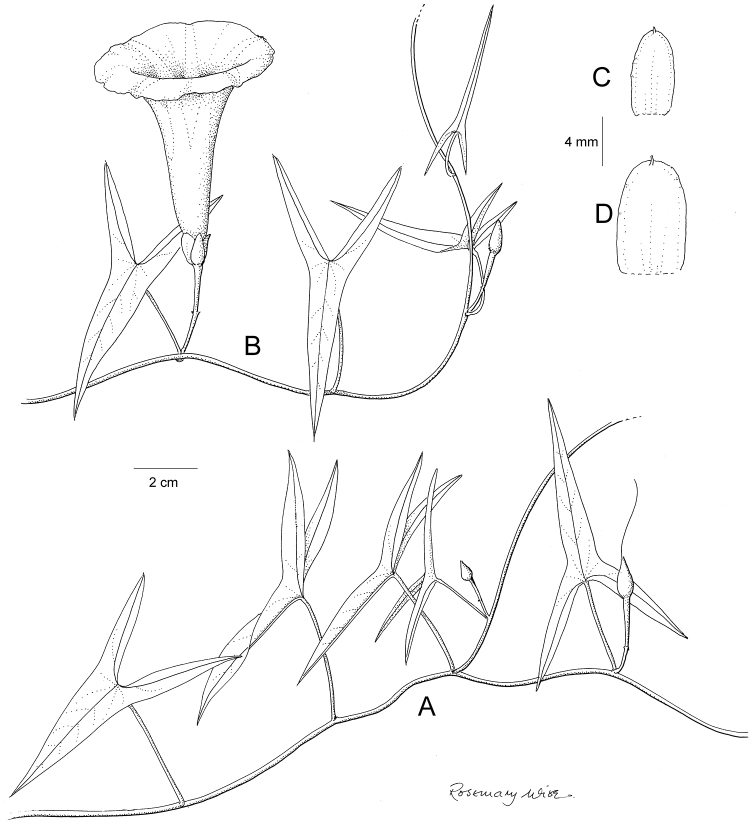
*Ipomoea sagittata*. **A** leaves and stem **B** habit with flower **C** outer sepal **D** inner sepal. Drawn by Rosemary Wise from *Curtiss* 5047.

McDonald (1994: 41); Figures 161A, 168.

#### Distribution.

Salt marsh and coastal grasslands. Coastal USA, from North Carolina south to Florida and along the Gulf coast to Mexico and thence south to Belize and Guatemala; also on Jamaica, Cuba, Bahamas and Bermuda: apparently absent from the Caribbean proper. It also occurs rarely inland, by saline lakes and streams as in Coahuila. It is apparently native or an ancient introduction on coasts in the Old World around the Mediterranean Sea.

**BELIZE.***C. Whitefoord* 2541 (BM); *G.R. Proctor* 35786 (MO); *J.A. Ratter et al.* 6569 (K).

**GUATEMALA.***J. Steyermark* 51539 (F).

**MEXICO. Campeche**: *G. Carnevali* 5851 (ARIZ). **Chiapas**: *E. Matuda* 3253 (MEXU). **Coahuila**: *F. Shreve* 8488 (ARIZ). **Quintana Roo**: *E.F. & H. Cabrera* 13939 (MEXU). **San Luis de Potosí**: *C.A. Purpus* 5446 (BM, MO). **Tabasco**: *A. Novello & A. Guerra* 4429 (MO). **Veracruz**: fide McDonald (1994). **Yucatán**: *D.F. Austin* 5025 (FTG).

**UNITED STATES. Alabama**: *Deramus* D149 (IBE). **Florida**: Jacksonville, *A.H. Curtiss* 2167 (BM, K), 5047 (K); St. Mark’s, *Rugel* s.n. [1843] (BM, K, OXF, S); 12 miles N of Naples, *W.J. Dress & R. Moran* 2492 (BM); *T.G. Lammers* 5884 (MA). **Georgia**: St Catharine’s Island, *S.B. Jones et al.* 24506 (BM); *R. Thorne & R. Norris* 6258 (GA). **Louisiana**: New Orleans, *Drummond* 1832 (K). **Mississippi**: *A.B. Seymour* 103 (S); *J. Wooten* s.n. (USMS). **North Carolina**: *Stevenson & Bradley* 3318 (E); Roanoke Island, *P.O. Schallert* 22877 (FTU). **South Carolina**: *J. Nelson* 28758 (USCH). **Texas**: *Lindheimer* Fasc.1 128 (BM, K, OXF); Aransas, *P. Fryxell* 5138 (IEB).

**BERMUDA.***S. Brown & N.L. Britton* 299 (BM, K); *F.S. Collins* 253 (BM, K).

**BAHAMAS.***N.L. Britton & L.J.K. Brace 393* (K, MO).

**CUBA.***C. Wright 3087* (HAC, K, MO); *Bro. León* 14162 (HAJB); *J. Bisse et al.* (HAJB34960); *E.L. Ekman* 895 (S), 18326 (S).

**JAMAICA.***G.R. Proctor* 37176 (MO); *R.A. Howard & G.R. Proctor* 14529 (BM).

#### Note.

Distinguished by the strongly sagittate leaves, the auricles nearly equalling the blade, the oblong-elliptic unequal sepals and solitary flowers.

### 352. Ipomoea philomega

Taxon classificationPlantaeSolanalesConvolvulaceae

(Vell.) House, Ann. New York Acad. Sci. 18: 246. 1908. (House 1908b: 246)

Convolvulus philomega Vell., Fl. Flumen.74, t.63. 1825 [pub. 1829]. (Vellozo, 1829: 74). Type. BRAZIL (lectotype, original parchment plate of Flora Fluminensis in the manuscript section of the Biblioteca Nacional, Rio de Janeiro [cat. no.: mss1198651-063], redesignated here; later published in Vellozo, Fl. Flum. Icon. 2: t. 63. 1827. [pub. 1831], the published plate (Vellozo 1831) designated as lectotype by Austin, 1982b: 74)
Ipomoea demerariana Choisy in A.P. de Candolle, Prodr. 9: 361. 1845. (Choisy 1845: 361). Type. GUYANA. *Parker*s.n. (holotype K000899614).
Ipomoea capparoides Choisy in A.P. de Candolle, Prodr. 9: 376. 1845. (Choisy 1845: 376). Type. BRAZIL. Bahia, Blanchet 861 (isotype BM).
Ipomoea macrophylla Choisy in A.P. de Candolle, Prodr. 9: 374. 1845. (Choisy 1845: 374). Type. BRAZIL. Pará, sine col. (P, n.v.).
Ipomoea cardiosepala Meisn. in Martius et al., Fl. Brasil. 7: 265. 1869. (Meisner 1869: 265). Type. BRAZIL. Rio de Janiero, *W.J. Burchell* 1865 (holotype BR, isotype K).
Ipomoea macrophylla var. selloana Meisn. in Martius et al., Fl. Brasil.7: 264. 1869. (Meisner 1869: 264). Type. BRAZIL. Rio de Janeiro, *F. Sello*s.n. (B?†).
Ipomoea costaricensis Kuntze, Revis. Gen. Pl. 2: 443. 1891. (Kuntze 1891: 443). Type. COSTA RICA. Zwischen Bagua und Angostura, Kuntze s.n. (isotype NY00319084).
Aniseia syringifolia Dammer, Bot. Jahrb. Syst. 23(Beibl. 57): 38. 1897. Type. BRAZIL. “Rio de Janeiro”, *A.F.M. Glaziou* 8191 (K, RB).
Ipomoea paraensis Peter, Nat. Pflanzenfam. 4 (3a): 30. 1897 [pub. 1891]. (Peter 1891: 30). Type. BRAZIL. Pará, Rio Capim, ad Lac. Putirytá, herb. Schwacke III, 160 (lectotype GOET005710, designated by Staples et al. 2012: 675).
Ipomoea philomega var. marowynensis Ooststr., Recueil Trav. Bot. Néerl. 33: 221. 1936. (Ooststroom 1936: 221). Type. FRENCH GUIANA. St. Jean, *R. Benoist* 892 (holotype P03539779).

#### Type.

Based on *Convolvulus philomega* Vell.

#### Description.

Liana to 10 m; stems thick, woody, glabrous. Leaves petiolate, 7–13 × 7–12 cm, broadly ovate, shallowly cordate with rounded auricles, apex acute or shortly acuminate, shortly mucronate, adaxially glabrous, abaxially pubescent (var. marowynensis) or glabrous; petioles 6–10 cm. Inflorescence of many-flowered, pedunculate axillary cymes, these often appearing paniculate or racemose with peduncle extended to form a central rhachis; peduncle 3–20 cm long, stout, glabrous; bracteoles 17–19 × 3–8 mm, oblong to narrowly obovate, acute, deciduous; secondary peduncles 1–3 cm; sepals subequal, glabrous or (rarely) pubescent, 12–17 × 10–14 mm, outer oblong-elliptic, rounded, often reddish, inner obovate with scarious margins; corolla 5–6 cm long, deep pink, glabrous, narrowly funnel-shaped with a narrow, tube which is slightly constricted below limb, limb c. 4 cm diam., unlobed. Capsules ovoid, 13 × 10 mm, glabrous; seeds 6 × 3–4 mm, woolly.

#### Illustration.

**Figure 169.**
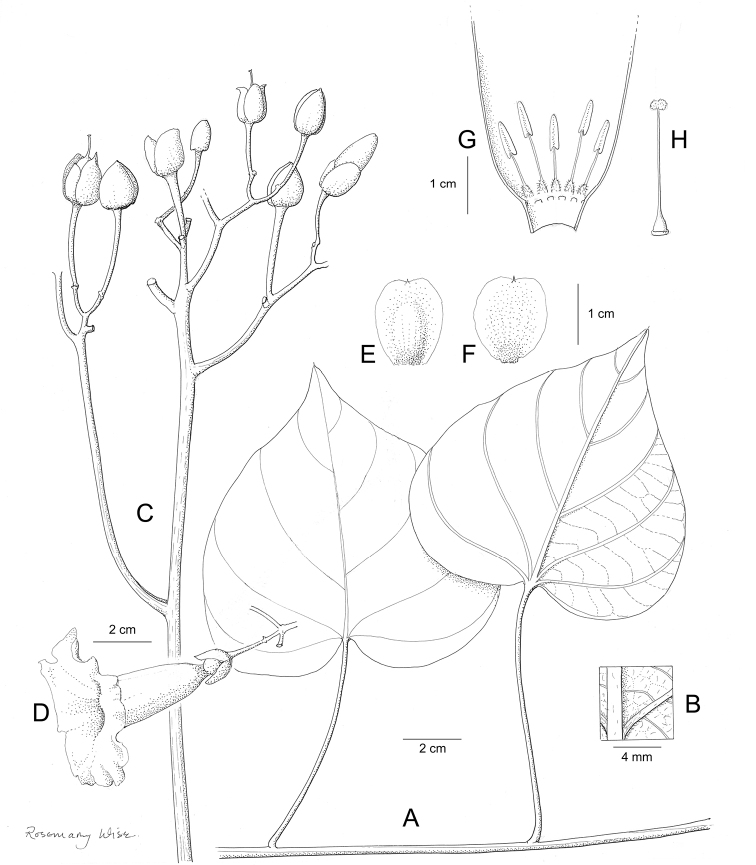
*Ipomoea philomega*. **A** leaves and stem **B** abaxial leaf surface **C** inflorescence D flower **E** outer sepal **F** inner sepal **G** corolla opened out to show stamens **H** ovary and style. Drawn by Rosemary Wise **A–C** from *Bianchetti* 548 **D** from photo; **E–H** from *Buchtien* 1219.

Austin (1998: 404). Figure 167A, 169.

#### Distribution.

*Ipomoea philomega* is a characteristic and common plant of moist rainforest throughout the neotropics and is probably the best indicator species of *Ipomoea* for this habitat. It is rare above 1000 m and is not present in seasonally dry tropical forests so absent from most of Mexico, coastal Colombia and Venezuela, most of the Cerrado and all the Chaco.

**BRAZIL. Acre**: Rio Branco, *J.U. Santos et al.* 59 (RB, MG, FTG). **Alagoas**: *M. Oliveira* 891 (RB). **Amapá**: *E. de Oliveira* 4396 (NY). **Amazonas**: *D.G. Campbell et al.* P20904 (K). **Ceará**: *J. Paula-Souza* 11128 (ESA). **Mato Grosso**: P. Estadual Cristalina, *D. Sasaki et al.* 2182 (K). **Pará**: *D. G. Campbell et al*. P22496 (NY, K, S); *W.R. Anderson* 10851 (MO). **Paraná**: Guaíra, *G. Hatschbach* 13327 (RB). **Pernambuco**: *A. Melo* 358 (CEPEC). **Rio de Janeiro**: *E. Pereira* 9894 (K, HB, RB), 36920 (K); *R. Marquete* 1520 (K). **Rondônia**: *W.R. Anderson* 12157 (MO). **Roraima**: *M. Nadruz* 2626 (RB). **Tocantins**: *L.B. Bianchetti et al.* 548 (CEN).

**FRENCH GUIANA.***Oldeman* 578 (K, P); *F. Crozier* 1672 (K, P); *Cremers & Hoff* 10620 (G).

**SURINAM.***J.C. Lindman et al.* 605 (K); *R.J. Evans et al.* 2689 (K, MO).

**GUYANA.***D.H. Davis 233* (K), *N. Sandwith* 248 (K); Essequibo River, *B. Maguire & D.B. Fanshawe* 22891 (BM, NY).

**BOLIVIA. Beni**: Est. Biológica del Beni, *E. Rivero* 256 (K, LPB, SP). **Cochabamba**: Carrasco, Puerto Villarroel, *F. Fernández Casas* 7914 (NY, MO); Chapare, *J.R.I. Wood* 21400 (BOLV, K, LPB). **Pando**: Manuripi, *de la Sota* 925 (LIL); Suárez, Cobija *F. Fernández Casas & A. Susanna* 8086 (NY, MO, G); *E.Ule* 9703 (K). **Santa Cruz**: Ichilo, Río Ichilo bridge, *M. Nee* 46450 (LPB, MO, NY, USZ).

**PERU. Amazonas**: *P.C. Hutchison & J.K. Wright* 3647 (K, UC). **Cusco**: *C. Sandeman* 3712 (K, OXF). **Loreto**: *T. Croat* 19851 (K, MO, P); Maynas, Iquitos, *S. McDaniel & Rimachi* 25337 (F, MO, USM); *A. Gentry et al.* 61993 (USM). **Madre de Dios**: Río Malinowski con Tambopata, *C. Evrard* 9776 (BM, BR); *S.F. Smith et al.* 1642 (F, K, P); 1988 (F). **Pasco**: *A. Gentry & D.N. Smith* 36044 (MO, USM). **San Martín**: *J. Schunke* 4726 (F, K, MO). **Ucayali**: *R. Vásquez & N. Jaramillo* 1521 (MO).

**ECUADOR. Carchi**: Tulcán, *D. Rubio et al.* 1635 (MO). **Esmeraldas**: *C. Játiva & C. Epling* 2158 (MO, NY, S, US). **Imbabura**: *G. Harling* 4301 (MO). **Morona-Santiago**: valle alto de Río Quimi, Cordillera del Condor, *W. Quizhbe* 2283 (MO, LOJA). **Napo**: *M. Lugo* 3116 (K, MO, S); Yasuni Forest Reserve, *P. Acevedo-Rodríguez et al.* 7570 (K, NY, P); *E. Asplund* 16475 (S). **Orellana**: *M.J. Macía et al.* 2981 (MO). **Pichincha**: Res. Forest. Endesa, *J. Jaramillo* 6730 (GB). **Sucumbíos**: *H. Balslev et al.* 84729 (AAU, MO). **Zamora-Chinchipe**: Cordillera del Condor, *W. Quizhbe* 2260 (LOJA, MO).

**COLOMBIA. Amazonas**: P.N. Amacayaca, *J.M. Cardie & M.L. Vidal* 190A (BM); *T. Plowman et al.* 2288 (K). **Antioquia**: Río Negro, *J. Cuatrecasas* 26192 (COL). **Boyacá**: *A.E. Lawrance* 366 (BM), 468 (BM, K), 755 (K, MO, RB, S); **Chocó**: *J.W.L. Robinson* 314 (K); *E. Forrero* 2321 (COL, RB). **Meta**: *J. Espina* 416 (COL). **Nariño**: Tumaco, *R. Romero 3385* (COL). **Putumayo**: Umbria, *G. Klug* 1799 (BM, K, MO, S). **Santander**: *A. Gentry & E. Rentería* 20054 (MO). **Valle**: *R.E. Schultes & Villarroel* 7393 (K). **Vaupés**: Río Apaporis, *R.E. Schultes & I. Cabrera* 112639 (BM).

**VENEZUELA. Amazonas**: *B. Stannard* 421 (K). **Apure**: *G. Davidse & A.C. González* 21903 (MO). **Barinas**: *J. Steyermark & M. Rabe* 96516 (MO, NY, VEN). **Bolívar**: *G. Aymard et al.* 4169 (MO). **Delta Amacuro**: *C.A. Blanco* 420 (MO). **Lara**: P.N. Yacambú, *G. Davidse & A.C. González* 20986 (MO, OXF). **Mérida**: *de Bruijn* 967 (MO, VEN). **Miranda**: Paéz, *G. González* 1167 (MO). **Sucre**: *J. Steyermark & G. Agostini* 91391(K, VEN), 99588 (S, VEN). **Táchira**: *J. Steyermark et al*. 119717 (MO). **Zulia**: *de Bruijn* 1215 (K, MO, VEN); Also Monagas and Yaracuy fide Austin (1982b).

**PANAMA.** Comarca de San Blas, *J.F. McDonagh et al.* 108 (BM, MO); Arenoso, *R. J. Seibert* 607 (K); *W. Lewis* 3423 (F).

**COSTA RICA.** Puntarenas, P.N. Isla del Coco, *F. Quesada* 1056 (K, MO); Limon, *P. Wilkin et al.* 114 (BM); Alajuela, *K. Flores & K. Martínez* 117 (BM, MO).

**NICARAGUA.** Jinotega, *I. Coronado et al.* 2450 (BM, MO); Zelaya, Cano Costa Riquita, *W.D. Stevens* 5017 (BM, MO).

**HONDURAS.** Puerto Lempira, *C.H. Nelson* & *E. Romero* 4183 (MO).

**BELIZE.** Cayo, Smokey Branch River, *C. Whitefoord* 9067 (BM); Stann Creek, *P.H. Gentle* 2761 (K, MO); ibid., *W.A. Schipp* 288 (K).

**GUATEMALA.** Izabal, *T. Croat* 41816 (MO); ibid., El Estor, *E. Contreras* 11152 (S).

**MEXICO. Veracruz**: San Andrés Tuxtla, *G. Ibarra Manríquez* 479 (IEB, MO).

**JAMAICA.***G.R. Proctor* 33468 (BM); St Ann’s, *Purdie* (K).

**HAITI.***E.L. Ekman* H10319 (S).

**DOMINICAN REPUBLIC.***E.L. Ekman* H15891 (S).

**LESSER ANTILLES. U.S. Virgin Islands**: St Thomas, *M. Finlay* s.n. [1841] (P). **St Kitts**: fide Powell (1979). **Guadeloupe**: fide Powell (1979). **Dominica**: *Ramage* 175 (BM, K); *C. Whitefoord* 3633 (BM); *Ernst* 1815 (BM, US). **Martinique**: *Steblé* s.n. (P). **St Lucia**: *G.R. Proctor* 21625 (BM). **St Vincent**: *H.G.A. von Eggers* 6808 (P).

**TRINIDAD.***W.E. Broadway* 6662 (BM, K, MO); *A.C. Jermy* 2456 (BM). **Tobago**: *W.E. Broadway* 4283 (BM, K).

#### Typification.

There are two collections by Parker labelled *Ipomoea demerariana* at Kew. The plant from Demerara is *I. philomega* whereas the plant from Barbados is a species of *Operculina*. As Choisy only cites the Demerara plant this should be treated as the type.

#### Notes.

A vigorous liana reaching at least 10 m in height, this species is usually easily identified by its woody stems (and peduncles), abaxially pubescent leaves and relatively small corolla. The elliptic, rounded, often reddish sepals are especially distinctive. It is most likely to be confused with *Ipomoea chondrosepala* but the inflorescences are many-flowered, often paniculate in form, the sepals opague and often reddish and the corolla shorter and often slightly constricted below the limb.

Var. marowynensis represents a form with a densely pubescent to subtomentose indumentum recorded from Surinam and French Guyana. (Ooststroom 1936: 221). Similar plants are found in Ecuador, sometimes with white flowers; examples include *A.J. Pérez et al.* 6685 (QCA) and *J. Jaramillo & F. Coello* 2592 (QCA) from Napo. Further study is needed to establish the status of these plants.

### 353. Ipomoea amazonica

Taxon classificationPlantaeSolanalesConvolvulaceae

(D.F. Austin & Staples) J.R.I. Wood & Scotland, Kew Bull. 70 (31): 27. 2015. (Wood et al. 2015: 27)

Turbina amazonica D.F. Austin & Staples, Bull. Torrey Bot. Club 118: 270. 1991. (Austin and Staples 1991: 270).
Calystegia glaziovii Dammer., Bot. Jahrb. Syst. 23(5), Beibl. 57: 41. 1897. (Dammer 1897: 41), non Ipomoea glazioviiDammer (1897). Type. BRAZIL. “environs de Rio Janeiro”, *A.F.M. Glaziou* 13009 (isotypes K00612827, P).

#### Type.

BRAZIL. Amapá. *D.F. Austin, C.E. Nauman, B. Rabelo, C. Rosario & M.R. Santos* 7389 (holotype MG; isotypes FAU, now in FTG, NY, MO, US).

#### Description.

Twining perennial, stem tomentose. Leaves petiolate, 3.5–9 × 3–7 cm, ovate-deltoid, obtuse and mucronate, base cordate with narrow sinus and rounded auricles, margin slightly undulate, softly tomentose on both surfaces, abaxially grey; petioles 1–2 cm, tomentose. Inflorescence a dense cluster of up to 10 flowers at apex of a long peduncle; peduncles 3–10 cm, tomentose; bracteoles 5–18 × 2–4 mm, ovate-rhomboid, tomentose, persistent; pedicels 5–10 mm; sepals tomentellous, accrescent in fruit, unequal, outer 8–12 × 5–8 mm, oblong-ovate, acute, base subcordate, inner 5–8 mm, oblong-ovate with broad scarious margins; corolla magenta, 5–6 cm long, funnel-shaped, glabrous except for a few hairs at apex of midpetaline bands in bud, limb c. 3 cm diam. Capsules 10–15 × 4–5 mm, ovoid, glabrous; seeds reported as usually one, oblong-ellipsoid, c. 10 mm long.

#### Illustration.

Austin and Staples (1991: 271).

#### Distribution.

A rare species of seasonally flooded lowland areas in the Amazon basin in Bolivia, Brazil and Colombia. It may be more common in the Amazonian regions of both Bolivia and Brazil than the few collections suggest.

**BRAZIL. Amapá**: *N.A. Rosa & M.R. Santos* 4309 (MG, NY); 12 km NE of Macapá, *D.F. Austin* 7389 (RB). **Amazonas**: Mun. Humaitá, *L.O.A. Teixeira et al*. 1329 (MO, NY, RB). **Mato Grosso**: Barra do Garças-Xavantina road, *D.R. Hunt & Ferreira Ramos* 5946 (K); Rio Suia Missú, c. 20 km N of ferry and 50 km NNW of base camp, *R.M. Harley & R. Souza* 11139 (K, P). **Pará**: Santarém, *Spruce* (K). **Rondônia**: *G. Prance et al.* 5966 (MG, NY).

**BOLIVIA. La Paz**: Iturralde, NE of confluence of Río Madidi with Río Inambari, *B.M. Torke et al.* 540 (LPB). **Pando**: Abuna, Río Negro confluence with Río Abuna, *A. Gentry & A. Perry* 77997 (MO, LPB). Federico Román, *L. Vargas et al.* 980 (F). **Santa Cruz**: Velasco, Campos de San Ramón, *S.R.P. Halloy et al.* 4291 (NY); PNNKM, Lago Caimán, *N. Ritter et al.* 4348 (MO).

**COLOMBIA. Vaupés**: Río Kubiyú, *J.L. Zarucchi* 1429 (K, COL, GH).

#### Note.

Very distinctive because of its rather small velvety leaves and oblong-cordate velvety sepals and persistent bracteoles.

### 354. Ipomoea racemosa

Taxon classificationPlantaeSolanalesConvolvulaceae

Poir., Encycl. [J. Lamarck et al.], Suppl. 4: 633. 1816. (Poiret 1814–17: 633)

Convolvulus racemosus (Poir.) Spreng., Syst. Veg. 1: 600. 1825 [pub. 1824]. (Sprengel 1824: 600).
Exogonium racemosum (Poir.) Choisy, Mém. Soc. Phys. Genève 8: 50 [128]. 1838. (Choisy 1838: 50 [128]).
Rivea racemosa (Poir.) Hallier f., Bot. Jahrb. Syst. 18: 158. 1894 [pub. 1893]. (Hallier 1893b: 158).
Quamoclit racemosa (Poir.) Roberty, Candollea 14: 41. 1952. (Roberty 1952: 41).
Turbina racemosa (Poir.) D. Austin, Ann. Missouri Bot. Gard. 64: 331. 1977 [pub. 1978]. (Austin 1978a: 331).
Ipomoea bracteata Rudolphi ex Ledeb., Neues J. Bot. 2: 292. 1807. (Ledebour 1807: 292), nom. illeg., non Ipomoea bracteata Cav. (1799). Type. “Santo Domingo”, Rudolphi s.n. (not found at MW, whereabouts unknown).
Pharbitis bracteata Choisy in A.P. de Candolle, Prodr. 9: 344. 1845. (Choisy 1845: 344). Type. Based on Ipomoea bracteata Rudolph ex Ledeb.
Ipomoea rudolphii Roem. & Schult., Syst. Veg. 4: 222. 1819. (Roemer and Schultes 1819: 222). Type. Based on Ipomoea bracteata Rudolph ex Ledeb.
Exogonium rudolphii (Roem. & Schult.) House, Bull. Torrey Bot. Club 35: 99. 1908. (House 1908a: 99).
Turbina rudolphii (Roem. & Schult.) O’Donell, Lilloa 30: 64. 1960. (O’Donell 1960: 64).
Convolvulus altissimus Spreng., Syst. Veg. 1: 613. 1825 [pub. 1824]. (Sprengel 1824: 613). Type. “Santo Domingo”, *C.L.G. Bertero*s.n. (Probable holotype P00391959, isotypes MO, MPU).
Ipomoea altissima (Spreng.) G. Don, Gen. Hist. 4: 273. 1838. (Don 1838: 264).
Exogonium wrightii House, Bull. Torrey Bot. Club 35: 99. 1908. (House 1908a: 99). Type. CUBA. *C. Wright* 1650 (holotype GH00054449, isotypes BR, G, MO, P).
Ipomoea wrightii (House) Alain, Mem. Soc. Cub. Hist. Nat. “Felipe Poey” 22: 123. 1955. (Liogier 1955: 122), nom. illeg., non Ipomoea wrightii A. Gray

#### Type.

[HAITI], “Sainte Dominique”, *Riedlé* s.n. (? F–Webb, fragment P00391958).

#### Description.

Liana, stems woody below, bark very pale, appressed sericeous when young. Leaves petiolate, 1–5 × 2–3.2 cm, ovate or oblong-ovate, obtuse, cordate, adaxially subglabrous to pilose, abaxially paler, pilose or sericeous; petioles 1.5–3.2 cm, pubescent. Inflorescence of axillary or terminal pedunculate cymes; peduncles 6–20 cm; bracteoles 1.5–4 cm, linear-lanceolate to oblong elliptic, coloured reddish to mauve, sericeous when young; pedicels 5–40 mm; sepals subequal, resembling the bracteoles, 18–25 × 7 mm, ovate to lanceolate, obtuse to acuminate, mucronulate, sericeous, glabrescent; corolla 3.5–5 cm long, salverform, limb 4 cm wide, red, purple or lavender, pubescent, stamens exserted, reddish. Capsules ovoid, apiculate; seeds usually 1, shortly pilose.

#### Illustration.

Figure 4A.

#### Distribution.

Endemic to the islands of Cuba and Hispaniola, growing in disturbed scrubby forest.

**CUBA.***C. Wright* 3096 (BM, NY, P); *J. Bisse et al*. (HAJB 21185); *L. Boise & H. Lipold* (HAJB20975). **Holguín**: Sabanaso, *E.L. Ekman* 6570 (BM, NY, S). **Villa Clara**: *A. Luna* 800 (NY).

**HAITI.** Massif des Matheux, *E.L.Ekman* H9168 (NY, S); La Brande to Mt. Balance, *G.V. Nash & N. Taylor* 1696 (NY).

**DOMINICAN REPUBLIC.** Valle de San Juan, *E.L. Ekman* H13530 (S); ibid., *R.A. & E.S. Howard* 8863 (BM, S); ibid., *A. Liogier* 12452 (NY); Baorhuco, *H. von Türckheim* 3598 (BM, NY); ibid., *M. Mejía et al.* 1062 (NY); Azua, *M. Mejia* 8327 (MO, NY); *M. Fuertes* 1883 (P).

#### Note.

The placement of this species is uncertain as we have been unable to sequence any example successfully. It is one of two Caribbean endemics that do not belong to Clade A2, the other being *Ipomoea jamaicensis*.

### 355. Ipomoea incarnata

Taxon classificationPlantaeSolanalesConvolvulaceae

(Vahl) Choisy in A.P. de Candolle, Prodr. 9: 359. 1845. (Choisy 1845: 359)

Convolvulus incarnatus Vahl, Eclog. 2: 12. 1798. (Vahl 1798: 12). Type. CURAÇAO. *Von Rohr* (lectotype C10009675, designated by Wood et al. 2015: 33).
Ipomoea monosperma Spreng. ex Choisy in A.P. de Candolle, Prodr. 9: 382. 1845. (Choisy 1845: 382). Type. COLOMBIA. Santa Marta, *C.L.G. Bertero*s.n. (holotype TO).
Ipomoea linearifolia Hook. f., Trans. Linn. Soc., London 20: 204. 1847. (Hooker, JD 1847: 204). Type. ECUADOR. Galápagos Islands, *C. Darwin*s.n. (holotype K00612875).
Ipomoea kinbergii Andersson, Vet. Akad. Handl. Stockholm 1853: 212. 1855. (Andersson 1855: 212). Type. ECUADOR. Galápagos Islands, *Andersson*s.n. (holotype S12-2024, isotype BR).
Ipomoea hilarifolia Rusby, Descr. S. Amer. Pl. 103. 1920. (Rusby 1920: 103). Type. COLOMBIA. Santa Marta, *H.H. Smith* 2109 (holotype NY00319192, isotypes BM, E, K, L, MICH, P, U, WIS).

#### Type.

Based on *Convolvulus incarnatus* Vahl

#### Description.

Creeping (rarely twining) perennial herb, stems glabrous. Leaves petiolate, 3.5–6 × 1–2 cm, deltoid, acute and apiculate, base sagittate with wide-spreading, usually acute auricles to hastate, both surfaces glabrous, abaxially paler, somewhat reticulate; petioles 1–3 cm. Inflorescence of 1(–2) shortly pedunculate flowers; peduncles 0.6–1.7 cm; bracteoles minute, filiform, caducous; pedicels 2.2–4 cm, prominently nerved; sepals subequal, 17–21 × 4–5 mm, lanceolate, acuminate to a fine aristate point, glabrous, chartaceous, veins prominent, inner sepals with scarious margins; corolla 7–8 cm long, funnel-shaped, pink, glabrous, limb 6–7 cm diam. Capsules ovoid, 9–10 mm long, glabrous; seeds 4–5 mm long, shortly pubescent.

#### Illustration.

Figures 5E, 170.

**Figure 170.**
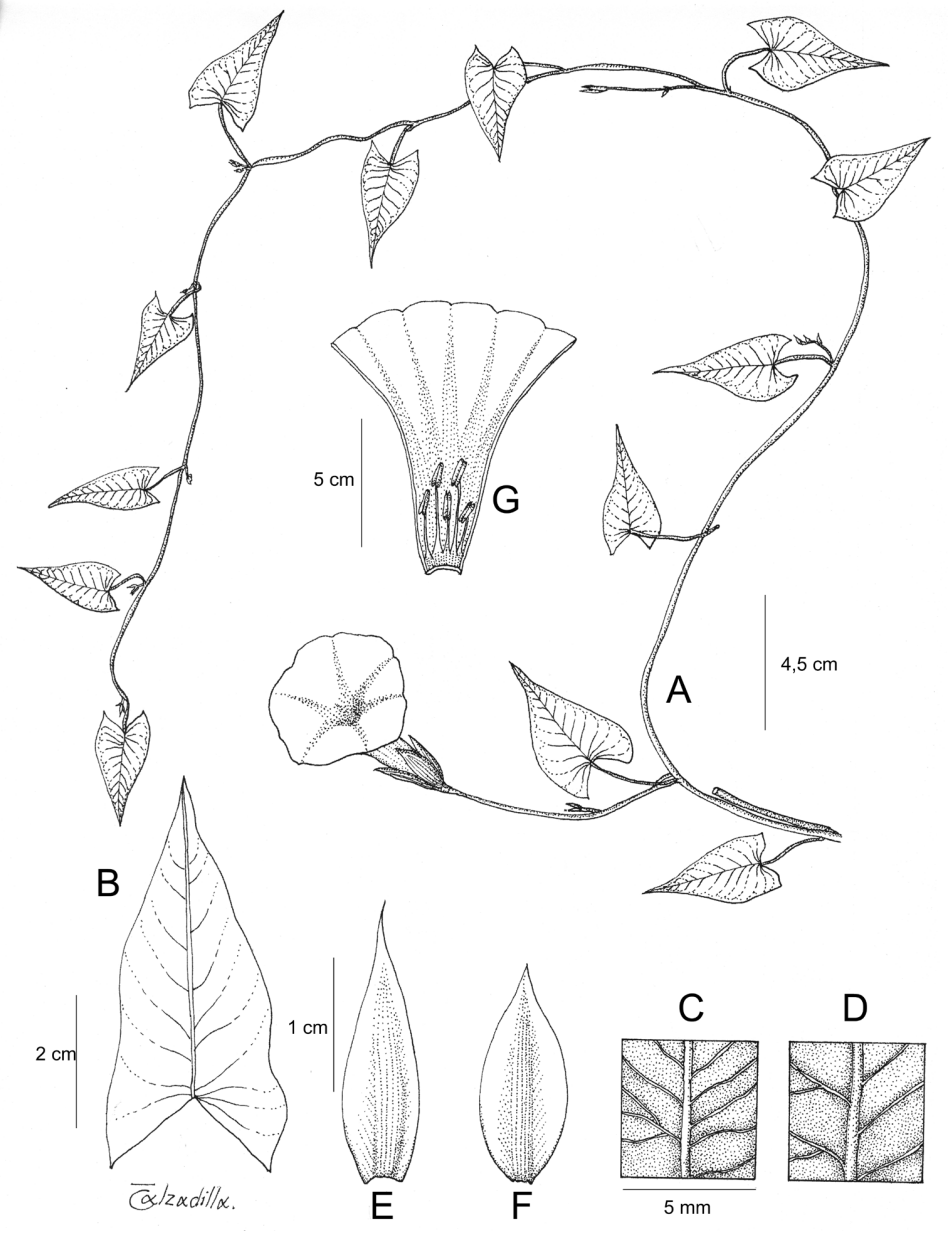
*Ipomoea incarnata*. **A** habit **B** leaf **C** adaxial leaf surface **D** abaxial leaf surface **E** outer sepal **F** inner sepal **G** corolla opened up to show stamens. Drawn by Eliana Calzadilla from *Wood et al.* 28031.

#### Distribution.

An indicator of very arid scrub, occurring in disjunct areas of South America, perhaps most common in the caatinga of the Brazilian state of Bahia.

**BRAZIL.** Caatinga region of the north east. **Bahia**: 21 km S of N. Senhora, dos Milagres, *A. Krapovickas* 10086 (CTES); Bom Jesus da Lapa, *R.M. Harley et al.* 21571 (CEPEC, K, NY); Ibotirama, *L. Coradin et al.* 6574 (CEN, K, MO); *H.S. Irwin* 32640 (NY). **Ceará**: Est. Eco. Aiuaba, *J.R. Lemos et al.* 235 (EAC, K); ibid., 240 (K). **Pernambuco**: *E.M. Carneiro 29* (ASE). **Rio Grande do Norte**: Ceará-Mirim, *A.B. Jardim* 315 (UFRN); Mossoró, Chapada do Apodi, *G.C. Pinto* 336/83 (RB). **Roraima**: Cantá, *R.C. Forzza* 8336 (RB). **Sergipe**: São Miguel do Aleixo, *G. Viana* 1589 (ASE).

**BOLIVIA.** Very arid Andean valleys. **Cochabamba**: Campero, c. 3 km east of Peña Colorada, *J.R.I. Wood* 20382 (BOLV, K, LPB). **Tarija**: Gran Chaco, between Villamontes and Palos Blancos, *J.R.I. Wood et al.* 27597 (OXF, LPB, USZ).

**PERU.** Common in coastal desert. **Arequipa**: Caravelí, *Martinet* 346 (P). **Cajamarca**: Río Chamaya, *T. Croat* 58371 (MO, USM). **La Libertad**: *M. Morales et al.* 3844 (USM). **Lambayeque**: Portachuelo de Olmos, *R. Ferreyra* 17785 (MO). **Lima**: *E. Asplund* 10886 (S); *D. Stafford* 44 (K); *C. Vargas* 4777 (CUZ); Chosica to Matacuna, *Y. Mexia* 4091 (MO). **Piura**: Talara, *A. Sagástegui* 10915 (MO, NY). **Tumbes**: Cancas, *A. Weberbauer* 7755 (BM, S).

**ECUADOR. Galápagos**: *F.R. Fosberg* 44927 (K, MO); *H.H. Van der Werff* 1082 (K, S); *G. Harling* 5528 (S). **Guayas**: Chanduy, *R. Spruce* 6500 (BM, K); *E. Asplund* 5640 (S); *G. Harling* 3109 (MO, S); *L.B. Holm-Nielsen* 2144 (AAU, F, MO). **Loja**: Zapotepampa, *B. Merino et al.* 4866 (LOJA).

**COLOMBIA.** Common in the arid NE coastal area. **Bolívar**: Cartagena, Mamonal, *R. Alvarado* 85 (COL). **Cesar**: Poponte, *C. Allen* 920 (K). **La Guajira**: *M.T. Dawe* 574 (K); Riohacha, *O. Haught* 4425 (COL, K); *J. Cuatrecasas* 25439 (COL). **Magdalena**: Santa Marta, *H.H. Smith* 1566 (K). **Norte de Santander**: Cúcuta-La Garrita, *R. Echeverry* 320 (COL).

**VENEZUELA. Anzoátegui**: 15 km E of Piritú, *T. Croat* 54391 (MO). **Carabobo**: Valencia-Maracay, *A.H.G. Alston* 6296 (BM, S); **Mérida**: Los Guaimaros, *L.E. Ruiz-Terán et al.* 12644 (MO). **Sucre**: Las Gonzales, *L.J. Dorr & L.C. Barnett* 7670 (NY); Playa Cachimena, *J. Steyermark* 108168 (MO). **Táchira**: *J.A. Steyermark et al.* 120210 (MO). **Zulia**: *L. Aristeguieta* 4955 (MO).

**NETHERLANDS ANTILLES: Aruba**: *A.L. Stoffers* 1999 (K, NY). **Bonaire**: *H. G. Hallier* 7108 (NY). **Curaçao**: *E.P. Killip* 21043 (NY).

#### Note.

A very distinct species quite unlike any other, and easily recognised by the elongate, lanceolate, finely acute sepals which are very prominently veined. *Ipomoea linearifolia* represents a form in which the leaf is reduced to a linear blade.

### 356. Ipomoea maurandioides

Taxon classificationPlantaeSolanalesConvolvulaceae

Meisn. in Martius et al., Fl. Brasil. 7: 275. 1869. (Meisner 1869: 275)

Ipomoea serpens var. albiflora Hallier f., Bull. Herb. Boiss. 7 (5), append. 1: 49. 1899. (Hallier 1899c: 49). Type. PARAGUAY. [Paraguarí], near Pirayú, *E. Hassler* 248 (holotype G, not found).
Ipomoea subtomentosa forma albiflora (Hallier f.) O’Donell, Arq. Mus. Paranaense 9: 241. 1952. (O’Donell 1952: 241).
Ipomoea serpens var. subtomentosa Chodat & Hassl., Bull. Herb. Boissier, sér. 2, 5: 694. (Chodat and Hassler 1905: 694). Type. PARAGUAY. [Cordillera], Tobatí, *E. Hassler* 6109 (lectotype G00175189, designated here; isolectotypes BM000089475, G00175188).
Ipomoea subtomentosa (Chodat & Hassl.) O’Donell, Arq. Mus. Paranaense 9: 239. 1952. (O’Donell 1952: 239).
Ipomoea maurandioides var. subtomentosa (Chodat & Hassl.) J.R.I. Wood & Scotland, Kew Bull. 70(31): 33. 2015. (Wood et al. 2015: 33).
Ipomoea serpens forma crassifolia Chodat & Hassler, Bull. Herb. Boissier, sér. 2, 5: 694. 1905 (Chodat and Hassler 1905: 694). Type. PARAGUAY. Cordillera, *E. Hassler* 6316 (isotypes BM, K, S12-2163).
Ipomoea carajasensis D.F. Austin, Acta Amazonica 11: 291. 1981. (Austin 1981: 291). Type. BRAZIL. Pará, Marabá, Serra dos Carajas, 700 m, *P. Cavalcante* 2115 (holotype MG).

#### Type.

BRAZIL. Rio Grande do Sul, Porto Alegre, *F. Sello* 3619 (B†, image F!, isotype NY00319201).

#### Description.

Trailing or twining herb from central tap root, stems glabrous to thinly pubescent. Leaves petiolate, 3–5 × 1–5 cm, narrowly ovate-deltoid, acute, sagittate or cordate, the auricles acute to obtuse (rarely rounded), green on both surfaces, glabrous or, rarely thinly pubescent; petioles 1–2(–3.5) cm. Inflorescence of axillary, pedunculate, 1–3-flowered cymes; peduncles 0.5–4.5; bracteoles minute, c. 1 mm long, deltoid, caducous; secondary peduncles (if present) 7–17 mm; pedicels 5–21 mm; sepals unequal, glabrous, outer 5–8 mm, broadly oblong-lanceolate or oblong-ovate, obtuse, greenish-scarious, 3-veined; inner 9–12 mm, oblong-oblanceolate, rounded and often mucronulate, with broad scarious margins; corolla 4–6 cm long, pink, funnel-shaped, glabrous, limb 3.5–4 cm diam., unlobed. Capsules 12 × 6 mm, ovoid, glabrous; seeds 6 × 2.5 mm, blackish, tomentellous.

**Figure 171.**
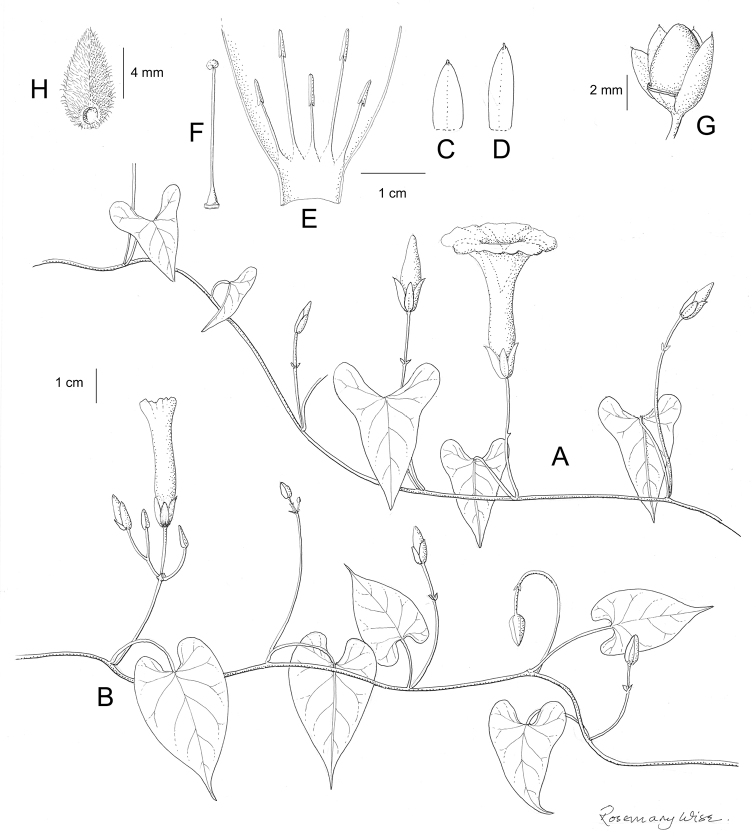
*Ipomoea maurandioides*. **A** habit with solitary flowers **B** habit with cymose inflorescence **C** outer sepal **D** inner sepal **E** corolla opened out to show stamens **F** ovary and style **G** capsule **H** seed. Drawn by Rosemary Wise **A** from *Petersen* 14655; **B–F** from *Wood & Williams* 27842; **G, H** from *Wood & Pozo* 25056.

**Illustrations.**O’Donell (1959b: 188); Figures 5G, 9E, 171, 173H–M.

#### Distribution.

Locally abundant in open, dry sandy and rocky cerrado and campo rupestre but especially characteristic of rock outcrops; northern Argentina, eastern Paraguay, eastern Bolivia and scattered locations in Brazil.

**ARGENTINA. Corrientes**: *T.S. Ibarrola* 2545 (LIL). **Misiones**: *B.S. Bertoni* 2568 (LIL).

**PARAGUAY. Amambay**: *T.M. Pedersen* 14655 (G); Cerro Corá, *N. Soria & Ortiz* 1953 (FCG, G); ibid., *E. Zardini et al.* 4165 (FCQ, MO); ibid., *I. Basualdo* 6339 (FCQ). **Central**: *Teague* 552 (BM). **Concepción**: Rancho Esperanza, *R. Degen* 2467 (FCQ). **Cordillera**: Limpio–Emboscada, *C. Ezcurra & F. Mereles* 1790 (FCQ, SI); Tobatí, *R. Degen & E. Zardini* 589 (FCQ). **Paraguarí**: *L. Bernardi* 18715 (G); La Colmena, *F. Mereles & G. Parini* 7810 (FCQ); P.N. Ybycu’í, *E. Zardini & P. Aquino* 29011 (PY). **San Luis**: *K. Fiebrig* 5340 (K).

**BRAZIL. Bahia**: Mun. Palmeiras, *M.L. Guedes et al.* PCD 2036 (ALCB, K); *Sano et al.* 14538 (K, USP); Sierra de Caitité, *R.M. Harley* 21280 (CEPEC, K). **Ceará**: Chapada de Ibiapaba, *A. Fernandes* s.n. (EAC). **Minas Gerais**: *L. Rossi et al.* 6987 (K); *R.C. Forzza et al.* 2692 (K); *R. Simão-Bianchini et al.* CFCR 11665 (SPF, K), 11503 (SPF, K); *M.M. Arbo et al.* 4168 (CTES); *H.S. Irwin et al.* 21918 (NY), 22892 (NY). **Pará**: *C.R. Sperling et al.* 5610 (MG, NY). **Rio Grande do Sul**: Type of *Ipomoea maurandioides*. Also Mato Grosso, Mato Grosso do Sul and Paraná fide Flora do Brasil (2020), in all of which it might be expected.

**BOLIVIA. Santa Cruz**: Germán Busch, camino a Rincón del Tigre, *D. Soto & I. Linneo* 1303 (K, LPB, USZ); Chiquitos, Valle de la Luna. Serranía de San José, *J.R.I. Wood et al.* 22871 (HSB, K, LPB); Santiago de Chiquitos, *J.R.I. Wood & D. Villarroel* 25571 (K, LPB, USZ, UB); entre Quimome y El Tinto, *J.R.I. Wood & P. Pozo* 25056 (K, LPB, UB, USZ); Cordillera, *A. Fuentes & G. Navarro* 2086 (LPB, USZ); Ángel Sandoval, Las Petas *J.R.I. Wood et al.* 24826 (K, LPB, UB, USZ); Velasco, Cerro Pelao, *J.R.I. Wood & H. Huaylla* 20780 (HSB, K, LPB, USZ); 10 km S. de San Rafael *M. Atahuachi et al.* 1435 (BOLV, LPB).

#### Notes.

This species is stored in many herbaria under the name *Ipomoea serpens* Meisn. but this is a later homonym of *I. serpens* L. (1759) and, in any case, the type material represents *I. paludicola*.

It is a relatively slender plant, not unlike a robust specimen of *Convolvulus arvensis* L., often trailing and growing around rocks, and recognised by its habit combined with the very unequal sepals, the inner sepals rounded and much longer than the ribbed outer sepals. The inflorescence commonly consists of solitary flowers but a cymose inflorescence with several flowers is not uncommon. Plants are usually completely glabrous but plants with pubescent leaves occur sporadically throughout its range and can be recognised as var. subtomentosa. Some Brazilian examples, such as *Harley* 21280 (K) or *Sano et al.* 14538 (K) are especially hirsute.

It is commonly confused with *Ipomoea paranaensis* and may intergrade with that species but the sepals are consistently shorter.

### 357. Ipomoea colombiana

Taxon classificationPlantaeSolanalesConvolvulaceae

O’Donell, Lilloa 26: 365. 1953. (O’Donell 1953a: 365)

#### Type.

COLOMBIA. Huila, Natagaima, *H.H. Rusby & F.W.Pennell* 268 (holotype NY).

#### Description.

Glabrous trailing perennial with woody tap root, resembling *I. maurandioides*, stems slender. Leaves petiolate, 1.3–5 × 0.5–2.5 cm, ovate, apex acute and mucronate, base hastate to sagittate with acute or obtuse auricles, the sinus ±triangular. Inflorescence of solitary or paired, pedunculate flowers from the leaf axils; peduncles 1.5–3.5 cm; bracteoles 1.5–2.5 mm, ovate, relatively persistent; pedicels 7–9 mm, becoming reflexed; sepals unequal, outer 7–10 × 2–3 mm, oblong-lanceolate, acute and mucronate, inner sepals 10–11 × 3 mm, acuminate, mucronate; corolla 2.5–3.5 cm long, pink, narrowly funnel-shaped. Capsules 8 mm, subglobose, glabrous; seeds 5–6 × 4–5 mm, white-tomentose.

#### Distribution.

Endemic to the dry Upper Magdalena valley where it grows around 500 m. **C**OLOMBIA. **Huila**: *Mason* 13807 (US).

#### Note.

Similar in facies to *Ipomoea maurandioides* but leaves sagittate to hastate and inner sepals gradually narrowed to a mucronate apex. There appear to be no recent collections and it is difficult to assess this species without more material.

### 358. Ipomoea aequiloba

Taxon classificationPlantaeSolanalesConvolvulaceae

J.R.I. Wood & Scotland, Kew Bull. 72(9): 22. 2017. (Wood and Scotland 2017b: 22)

#### Type.

BRAZIL. Tocantins, Mun. Tocantinopolis, km 18 estrada vecinal á Ferrovia Norte Sul, 6°38'50"N, 47°29'56"W, 190 m, 21 Feb. 2005, *G. Pereira-Silva et al.* 9483 (holotype CEN).

#### Description.

Slender trailing perennial; glabrous in all parts. Leaves shortly petiolate, 0.8–2.5 × 0.1–0.4 cm, sagittate, appearing subequally trilobed, the central lobe linear to very narrowly oblong, acute, the two linear acute auricles, resembling, ±equalling or slightly shorter than the central lobe, both surfaces glabrous; petioles 0.6–3 cm. Inflorescence of solitary axillary pedunculate flowers; peduncles 15–20 mm, commonly bent at a sharp angle at apex; bracteoles scale-like c. 1 mm long; pedicels 6–13 mm, slightly thickened upwards; sepals unequal, lanceolate, finely acuminate, glabrous, outer pair unequal 4.5–8 × 2–2.5 mm; inner 13–14 × 3 mm; corolla 6.5 cm long, funnel-shaped, pink, glabrous; limb c. 4.5 cm diam., the midpetaline bands ending in a tiny tooth. Capsules and seeds not seen.

#### Illustration.

Figure 172.

**Figure 172.**
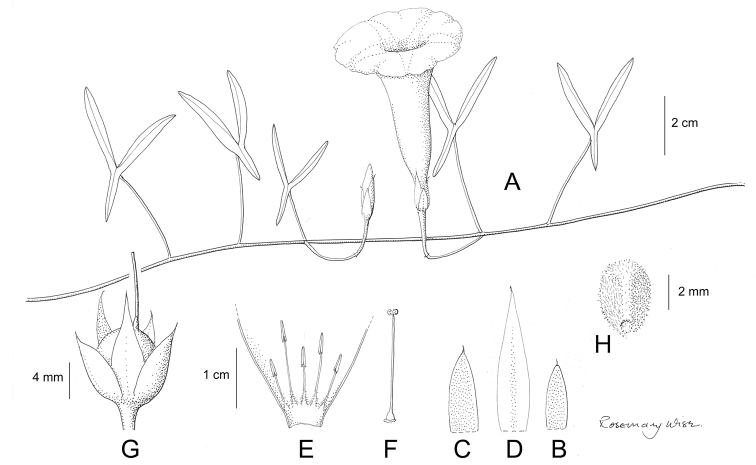
*Ipomoea aequiloba*. **A** habit **B** outer sepal **C** middle sepal **D** inner sepal **E** corolla opened out **F** ovary and style **G** capsule **E** seed. Drawn by Rosemary Wise **A** from *Macedo* 86, **B–H** from *Hatschbach* 42084.

#### Distribution.

Scattered in the Cerrado biome of Brazil, eastern Paraguay and NE Argentina.

**ARGENTINA. Misiones**: Dept. Candelaria, Colonia Tacuaruzu, *H. Keller et al.* 13355 (CTES, OXF).

**PARAGUAY. Concepción**: N of Arroyo Tagatiya-Guazu, *E. Zardini & T. Tilleria* 38683 (MO).

**BRAZIL. Bahia**: 10 km N. de Barreiras, *G. Hatschbach* 42084 (CTES, FTG). **Goiás**: São Domingos, *A.A. Santos* 2197 (CEN). **Maranhão**: Mun. Estreito, *G. Pereira-Silva & G.A. Moreira* 12442 (CEN). **Mato Grosso do Sul**: 68 km W of Jardim, *A. Krapovickas & A. Schinini* 32751 (CTES). **Minas Gerais**: Ituiutaba, *A. Macedo* 86 (K, US), 4141 (BM). **Tocantins**: Trans-Amazonian highway, *T. Plowman et al.* 9277 (MO, MG, NY, RB); Palmeiras do Tocantins, *G. Pereira-Silva & G.A. Moreira* 12546 (CEN).

#### Note.

Clearly related to to both *Ipomoea maurandioides* and *I. mucronatoproducta* and distinguished from *I. maurandioides* by the sepals lanceolate and finely acuminate, rather than the inner sepals oblong-oblanceolate and mucronate and from *I. mucronatoproducta* by the midpetaline bands terminating in a small tooth rather than in a long fine point up to 6 mm in length. It is easily distinguished from both by the distinctive, superficially 3-lobed leaves in which the two auricles are more or less equal to the blade.

### 359. Ipomoea mucronatoproducta

Taxon classificationPlantaeSolanalesConvolvulaceae

J.R.I. Wood & Scotland, Kew Bull. 70 (31): 34. 2015. (Wood et al. 2015: 34)

#### Type.

BOLIVIA. Santa Cruz, Prov. Germán Busch, Rincón del Tigre, portón de la entrada a la Misión, sobre el camino hacia Carmen Rivero Tórrez, *J.R.I. Wood & D. Villarroel* 25474 (holotype USZ; isotypes K, LPB, UB).

#### Description.

Glabrous trailing herb, probably perennial, stems to 1.5 m long. Leaves petiolate, narrowly deltoid, 1.5–3.2 × 0.4–1 cm (measured above intersection with petiole), apex obtuse and minutely mucronate, base strongly sagittate, the auricles deltoid, lanceolate, acute, basally asymmetric, 1–2.5 × 0.2–0.6 cm so leaves sometimes appearing 3-lobed, both surfaces glabrous; petioles 1.1–2.7 cm. Inflorescence of solitary or paired, axillary, pedunculate flowers; peduncles 2.6–4 cm; bracteoles 2–3 × 1 mm, ovate, acuminate, caducous; secondary peduncles (when present) 10 mm; pedicels 6–16 mm, thickened upwards; sepals unequal, oblong-lanceolate, acuminate to a fine aristate point, outer shorter, 11–12 × 2–2.5 mm, inner 15–16 mm; corolla 7–8 cm long, narrowly funnel-shaped with a long narrow basal tube, glabrous, tube deep pink inside, limb 5–6 cm diam., pale pink, unlobed, the midpetaline bands terminating in a fine point 5–6 mm long; Capsules (immature) ovoid, 7 × 6 mm, glabrous; seeds not known.

#### Illustration.

Figures 8R, 173A–G.

**Figure 173.**
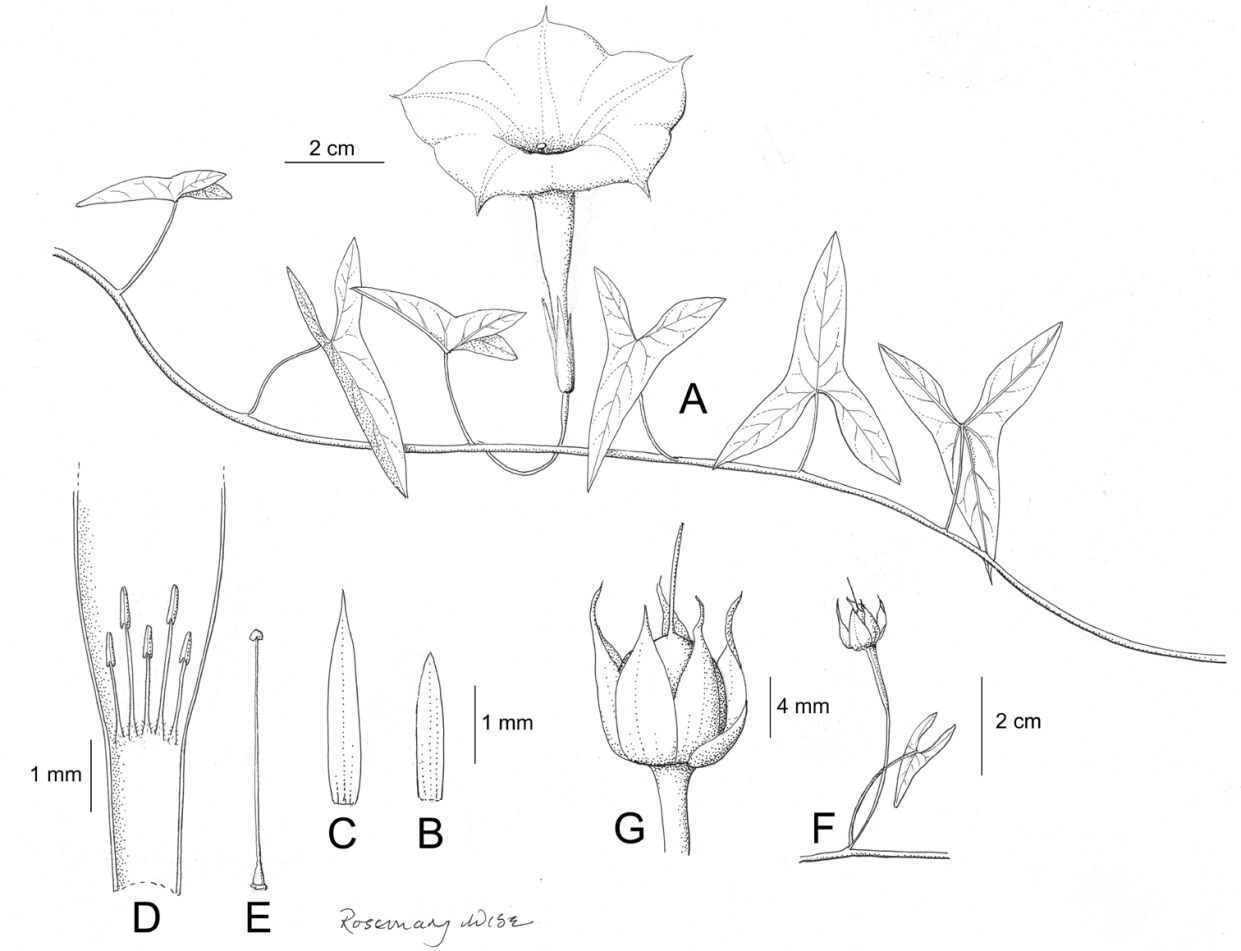
**A–G***Ipomoea mucronatoproducta*. **A** habit **B** outer sepal **C** inner sepal **D** corolla opened out to show stamens **E** ovary and style **F**. shoot with your fruiting inflorescence **G** calyx with young fruit. Drawn by Rosemary Wise from *Wood & Villarroel* 25474.

#### Distribution.

A characteristic species of seasonally flooded campo around the Pantanal with several records from Mato Grosso do Sul and eastern Bolivia.

**BRAZIL. Mato Grosso**: 68 km W of Jardim, *A. Krapovickas & A. Schinini* 327519 (FTG). **Mato Grosso do Sul**: Mun. Corumbá, Porto Esperança, *B. Lutz* s.n. (R); Mun. Corumbá, Faz. Acurizal, *A. Pott et al.* 3642 (CPAP); Mun. Miranda, Est. Caiman, *A. Pott et al.* 7944 (CPAP); Mun. Bonito, Lagoa das Pedras, *V.J. Pott et al.* 4156 (CPAP); Mun. Bela Vista, *G. Hatschbach et al.* 74293 (MBM).

**BOLIVIA. Germán Busch**: Rincón del Tigre, *J.R.I. Wood et al.* 27242 (K, LPB, USZ); *M. Atahuachi et al.* 1887(LPB); 30 km S. of Rincón del Tigre, *J.R.I. Wood et al.* 28824 (USZ).

#### Note.

*Ipomoea mucronatoproducta* sometimes grows with and is similar in habit and leaf shape to *I. maurandioides*. In the field it is readily distinguished by the corolla lobes which terminate in a long fine point 5–6 mm in length. Herbarium specimens are best identified by the finely acuminate sepals, the inner ones reaching 15 mm in length.

### 360. Ipomoea paranaensis

Taxon classificationPlantaeSolanalesConvolvulaceae

Hoehne, Boletim de Agricultura (São Paulo), 35(1): 475. 1934. (Hoehne 1934: 475)

Ipomoea ramboi O’Donell, Lilloa 30: 48. 1960. (O’Donell 1960: 48). Type. BRAZIL. Rio Grande do Sul, Nonoai, *B. Rambo* 28183 (holotype LIL001280).
?Ipomoea kunthiana var. sagittata Meisn. in Martius et al., Fl. Brasil.7: 253. 1869. (Meisner 1869: 253). Type. Not specified.

#### Type.

BRAZIL. Paraná, Ponta Grossa, *F.C. Hoehne* 23230 (holotype SP000577).

#### Description.

Trailing perennial herb, glabrous in all parts; rootstock, thick, fleshy. Leaves petiolate, 3.5–7 × 2–4.5 ovate to suborbicular, rounded, obtuse or retuse, sometimes mucronulate, base cordate with rounded auricles, abaxially veins prominent; petioles 1–2.5(–4) cm. Inflorescence of solitary, axillary flowers; peduncles 0.5–8 cm; bracteoles 2–4 mm, lanceolate-filiform, apiculate; pedicels 0.5–3 cm, thickened upwards, sometimes rugose; sepals unequal, outer sepals 10–15 × 5–6 mm, broadly or narrowly ovate or elliptic, acuminate, inner sepals similar but larger, 15–28 × 7–8 mm; corolla 6–9 cm long, funnel-shaped, pink, glabrous, limb c. 5–6 cm diam., apparently unlobed; ovary glabrous. Capsules and seeds unknown.

#### Illustration.

Figure 174.

**Figure 174.**
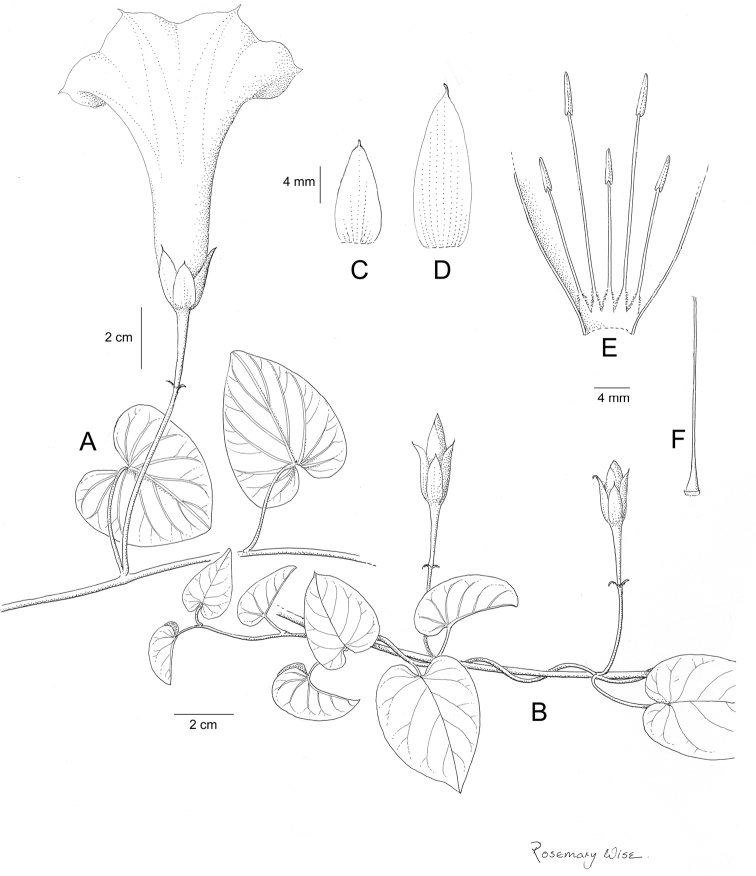
*Ipomoea paranaensis*. **A** habit **B** twining habit **C** outer sepal **D** inner sepal **E** corolla opened out to show stamens **F** ovary and style. Drawn by Rosemary Wise **A** from *Dusen* 7306; **B** from Dusen 1327a; **C–F** from *Moreira & Guimarães* 456.

#### Distribution.

A grassland species of southern Brazil and adjacent areas of Argentina. **ARGENTINA. Misiones**: Dept. General Belgrano, Cementerio Campiñas de América, *H. Keller* 3733 (CTES).

**BRAZIL. Minas Gerais**: *P. Clausen* s.n. (K). **Paraná**: *J.M. Silva et al.* 8290 (MBM); Turma, *G. Jaussan* 1327 (GH, MO, S); Vila Velha-Ponta Grossa, *H. Moreira & O. Guimarães* 456 (US); Ponta Grossa, Parque Vila Velha, *G. Hatschbach* 13109 (US); Itaperuçú, *P. Dusen* 7157 (S, GH, NY); Serrinha *P. Dusen* 7306 (K, MO, P, S); Piraquara, *G. Tessmann* (MBM265879). **Rio Grande do Sul**: type of *Ipomoea ramboi*. **Santa Catarina**: Chapecó, *L.B. Smith & R.M. Klein* 9341 (US); Joaçaba, campos of Rio Iraní, *L.B. Smith & R.M. Klein* 9838 (US); Mafra, Tingui-Mafra, *L.B. Smith & R.M. Klein* 10632 (K, US); Abelardo, *L.B. Smith & R.M. Klein* 13302 (US).

#### Note.

This species is most likely to be confused with *Ipomoea maurandioides* but differs in the much longer sepals and the distinctive broadly ovate, often obtuse leaves with rounded auricles.

### 361. Ipomoea variifolia

Taxon classificationPlantaeSolanalesConvolvulaceae

Meisn. in Martius et al., Fl. Brasil. 7: 275 (Meisner 1869: 275)

#### Type.

URUGUAY. Pr. Calderón, *F. Sello* 688 (B†, photo F, isotype NY00319238).

#### Description.

Slender twining or perhaps trailing herb; all parts glabrous. Leaves petiolate, 2–3.5 × 0.2–0.8 cm, oblong, auricles 9–12 × 3–5 mm, sagittate, often bilobed, apex acute, margins undulate, base broadly cordate and briefly cuneate onto the petiole, glabrous, abaxially slightly paler; petioles 7–14 mm. Inflorescence of solitary axillary flowers; peduncles 1.5–2.8 cm; bracteoles 2 mm, lanceolate, apiculate; pedicels 8–10 mm; sepals unequal, oblong-lanceolate, acuminate to an apiculate point, outer 10 × 3 mm, inner 15–16 × 3.5 mm; corolla 6.5–7 cm long, funnel-shaped, pink, limb c. 3.5 cm diam., undulate. Capsules and seeds not seen.

#### Distribution.

Known from two collections from Uruguay and southern Brazil, presumably growing in grassland.

**URUGUAY.** Type collection.

**BRAZIL. Santa Catarina**: Mun. Porto União, east of Valôes (Irineópolis) on road to Canoinhas, *L.B. Smith & P. R. Retz* 8631 (US).

#### Note.

A poorly known species apparently related to *Ipomoea paranaensis* but distinguished by the very distinctive leaves. Superficially it resembles a species of *Convolvulus* but the corolla is immediately recognizable as an *Ipomoea*.

### 362. Ipomoea tacuaremboensis

Taxon classificationPlantaeSolanalesConvolvulaceae

Arechav., Anales Mus. Nac. Montevideo 7: 195. 1911. (Arechavaleta y Balpardo 1911: 195)

Ipomoea tacuaremboensis forma foliosa Arechav., Anales Mus. Nac. Montevideo 7: 197 (1911). (Arechavaleta y Balpardo 1911: 197). Type. URUGUAY. Rivera, sine data (lectotype MVM, No. 1728 ex Herb. M.B. Berro, designated here).

#### Type.

URUGUAY. “Tacuarembó, Valle Edén, region Tambores, febrero”, *J. Arechavaleta* 5483A (renumbered 458) (lectotype MVM, designated here).

#### Description.

Decumbent perennial, stems angled, muricate, glabrous, at least 50 cm long. Leaves shortly petiolate, 4–11 × 0.2–1(–2) cm, narrowly oblong or very narrowly lanceolate, acuminate and mucronate, base hastate to sagittate, glabrous; petioles 8–12 mm. Inflorescence of solitary, axillary flowers; peduncles 0–2 mm; bracteoles 3–6 mm, filiform; pedicels 5–12 mm; sepals unequal, glabrous; outer sepals 10–12 × 5 mm, oblong-ovate, acute, shortly mucronate, inner 15–19 × 8 mm, ovate, acuminate, mucronate, the apex often bent; corolla 5.5–7 cm long, pink, funnel-shaped, glabrous; limb c. 3 cm diam. Capsules 11 × 8 mm, ovoid with persistent style, glabrous; seeds tomentellous.

#### Distribution.

Apparently very rare in “campo”, presumably some kind of grassland in the border region of Uruguay and Brazil.

**URUGUAY**: Gruta de Las Cuervas, *M.B. Berro* 4823 (K).

**BRAZIL. Rio Grande do Sul**: 55 km W of Rosario do Sul, *Krapovickas & Cristóbal 34234* (CTES, MO).

#### Lectotypification.

In selecting lectotypes, we have designated *Arechavaleta* 5483A as the lectotype of the type form as this is annotated by Arechavaleta as this species. The specimen designated as lectotype of forma foliosa is chosen because it appears to be the only possible specimen at MVM and is remarkable for the large number of leaves although there is no annotation to indicate Arechavalata considered it the type.

#### Note.

This species is sometimes treated as a synonym of *Ipomoea kunthiana* (Austin et al. 2015) but the leaves are different and we prefer to treat it as distinct for the time being. It is presumably related to *Ipomoea paranaensis* but is readily distinguished by the narrowly oblong leaves, muricate stems and very short peduncles.

### 363. Ipomoea squamisepala

Taxon classificationPlantaeSolanalesConvolvulaceae

O’Donell, Lilloa 23: 453. 1950. (O’Donell 1950a: 453)

Ipomoea angulata Mart. ex Choisy in A.P. de Candolle, Prodr. 9: 371. 1845. (Choisy 1845: 371), non Ipomoea angulata Lam. (1791). Type. BRAZIL. J.B. Pohl 1646 (holotype M0184962, isotype ?BR).
Ipomoea angulata var. latifolia Meisn. in Martius et al., Fl. Brasil. 7: 248. 1869. (Meisner 1869: 248), nom. illeg., autonym.
Ipomoea angulata var. gnidioides Meisn. in Martius et al., Fl. Brasil. 7: 248. 1869. (Meisner 1869: 248). Type. BRAZIL. [Goiás], Serra Dourada, J.B. Pohls.n. (lectotype BR0000005307449, designated here).
Ipomoea squamisepala var. gnidioides (Meisn.) O’Donell, Lilloa 23: 453. 1950. (O’Donell 1950a: 453).
Ipomoea angulata var. linearis Meisn. in Martius et al., Fl. Brasil. 7: 248. 1869. (Meisner 1869: 248). Type. BRAZIL. Goiás, L. Riedel [2757] (NY00319142, lectotype designated here; LE01025971 isolectotype).

#### Type.

Based on *Ipomoea angulata* Mart. ex Choisy

#### Description.

Erect undershrub from a xylopodium to c. 1 m, stems very woody, somewhat ridged, glabrous; plant drying blackish. Leaves shortly petiolate, (2–)4–6 × (0.2–)0.5–1.5(–3) cm, linear-oblong, oblong-elliptic or oblanceolate, obtuse to acute and apiculate, cuneate at base, glabrous; petiole 0–5 mm, poorly defined. Inflorescence racemose, terminal, typically elongate to 40 cm, sometimes branched but sometimes much reduced, often dense, formed of shortly pedunculate cymes from the upper leaf axils; peduncles 0–1.5 cm, erect; bracteoles fugacious (not seen); secondary peduncles c. 2 cm, often rhachis-like; pedicels 3–8 mm; sepals very unequal, obovate-elliptic, rigid, glabrous, outer 2–4 × 2 mm long, obtuse, white-margined, inner 5–7 × 3–4 mm, rounded, margins scarious; corolla 2–4 cm long, funnel-shaped, white or lilac, glabrous, limb c. 2.5–3 cm diam. Capsules and seeds not seen.

#### Illustration.

Figures 7G, 175.

**Figure 175.**
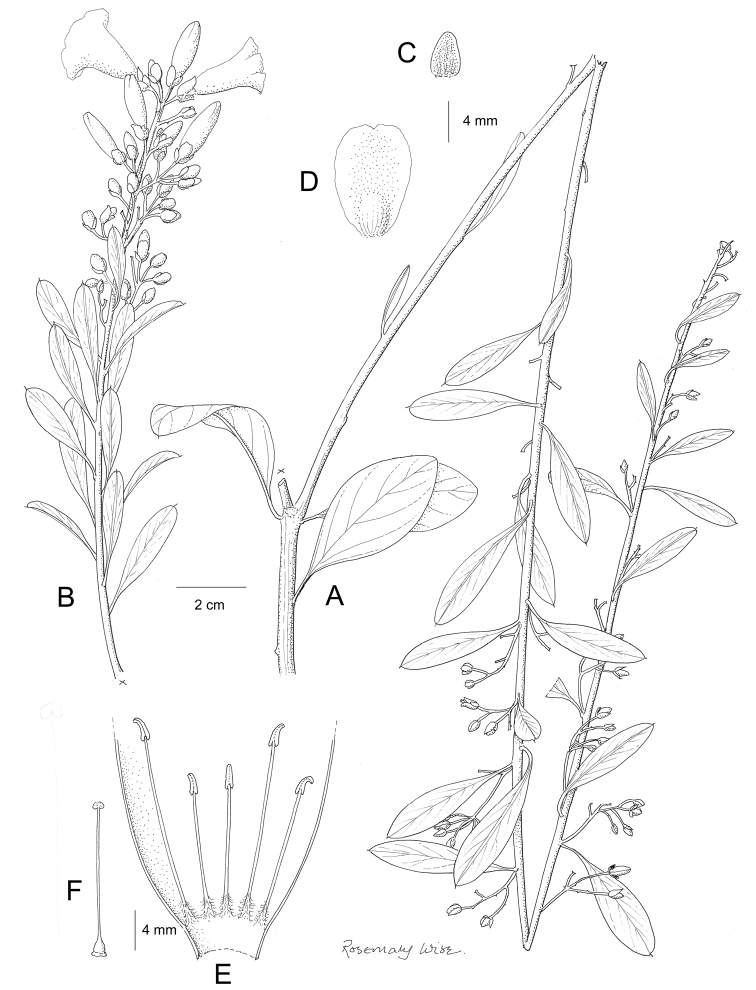
*Ipomoea squamisepala***A** habit **B** inflorescence **C** outer sepal **D** inner sepal **E** corolla opened up to show stamens **F** ovary and style. Drawn by Rosemary Wise from *Kirkbride* 5271.

#### Distribution.

A typical cerrado species, which is locally common in Brazil but known elsewhere only from a single location in eastern Bolivia.

**BRAZIL. Bahia**: Maracás, *E.B. dos Santos* 295 (NY). **Dist. Fed.**: *J.M. Pires et al.* 9110 (S, UB); Chapada da Contagem, *H.S. Irwin & Soderstrom* 5295 (NY, S); Bacia do Rio São Bartolomeu, *E.P. Heringer* 6588 (MO). **Goiás**: Formosa, *H.S. Irwin et al.* 14280 (MO, NY); Luziania, *G. Pereira-Silva et al.* 7541 (CEN). **Mato Grosso**: *Malme* s.n. [12/5/1903] (S); Cuiabá, *G. Hatschbach* 32042 (MBM, NY, S); ENE of Barra de Garças, *W.R. Anderson* 9690 (NY); Rio Turvu, Xavantina, *R. de Santos et al.* 1634 (K, P, RB). **Minas Gerais**: *P. Clausen s.n.* (BM, K, NY); Salinas, *Weddell* 2185 (P); Perdizes, *S. Mendes* 634 (HUFU). **Tocantins**: Serra de Ararais, *G. Gardner* 5033 p.p. (BM, K); Palmeiropolis, *G. Pereira-Silva* 10760 (CEN).

**BOLIVIA. Santa Cruz**: Ángel Sandoval: Santo Corazón, Sunsas-Boca Bella, *A. Fuentes et al.* 1776 (ARIZ, BOLV, MO, USZ).

#### Lectotypification.

In designating a lectotype of Ipomoea angulata var. linearis, we have chosen the NY specimen as it appears to have a label in Meisner’s handwriting annotated as “β linearis nob. (29./12./67.)”

#### Notes.

Distinctive because an erect subshrub with white or pale lilac flowers, the leaves at least 0.5 cm wide and the sepals very unequal. It flowers late in the rainy season unlike most cerrado species.

Although most specimens are readily assigned to either *I. squamisepala* or *I. pinifolia*, there is no clear molecular support for their monophyly and some specimens (Ipomoea angulata var. linearis) are somewhat intermediate in their leaf shapes.

### 364. Ipomoea pinifolia

Taxon classificationPlantaeSolanalesConvolvulaceae

Meisn. in Martius et al., Fl. Brasil. 7: 250. 1869. (Meisner 1869: 250)

#### Type.

BRAZIL. *W.J. Burchell* 6700-7 (lectotype BR0000005837731, designated by Wood et al. 2015: 35, isolectotype K!).

#### Description.

Wiry perennial of cerrado, occasionally leafless, rootstock a xylopodium, stems glabrous, woody, often simple and erect to 1.5 m but sometimes branched and then branches spreading or twining apically. Leaves sessile, very variable in length 2–14 × 0.1–0.3 cm, linear-filiform, acute, glabrous. Inflorescence of 1(–5)-flowered axillary cymes from the upper leaf axils, sometimes clustered apically but more commonly forming a long narrow raceme-like inflorescence up to 30 cm long; peduncles 0–8(–21) mm; bracteoles caducous, scale-like, secondary peduncles (if present) up to 4 mm; pedicels 7–10(–15) mm; sepals coriaceous, convex, very unequal, glabrous, outermost 2–6 mm, elliptic to suborbicular, obtuse to rounded, often minutely mucronate, inner 7–12 mm, oblong to elliptic, obtuse to rounded, margins broad, scarious; corolla 3–4.5 mm long, glabrous, pink, gradually widened from base, the limb 3–3.5 cm diam., undulate, the midpetaline bands ending in teeth. Capsules glabrous; seeds reported to be pilose.

#### Illustration.

Figure 176.

**Figure 176.**
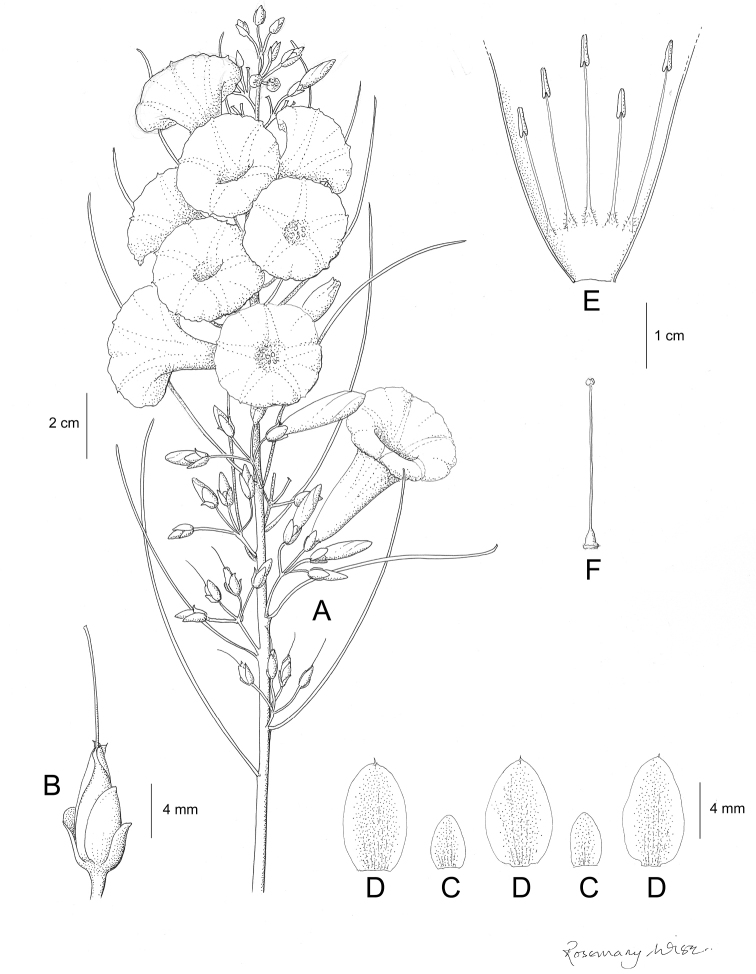
*Ipomoea pinifolia*. **A** inflorescence with leaves **B** calyx **C** outer sepals **D** inner sepals **E** corolla opened out to show stamens **F** ovary and style. Drawn by Rosemary Wise from *Azevedo et al.* 1172.

#### Distribution.

A characteristic species of the cerrado, which is locally common in central Brazil extending to a single area in Bolivia.

**BRAZIL. Dist. Fed.**: *J.F. Pastore* 307 (CEN); Rio Descoberto, *H.S. Irwin* 11050 (NY). **Goiás**: Serra Dourada, *B.R. Silva et al.* 1172 (F, RB, SPF); Niquelândia, *R.D. Reeves* 3006 (CEN); Alto Paraíso, *C. Proença & M.A. Silva* 1177 (UB); ibid., *T.B. Cavalcanti et al.* 38 (MBM, K, SPF), Minacu, *T.B. Cavalcanti* 1129 (CEN, RB). **Mato Grosso**: São José da Serra, *G. Hatschbach* 32025 (MBM, NY, S); Serra de Ricardo Franco, *M.F. Simon* 2195 (RB); Buriti, *Malme* s.n. [8 June 1903] (S); Sangradura, *A. Krapovickas et al*. 40235 (CTES, CEN); Chapada de Guimaraes, *A. Dubs* 1201 (K, Z). **Mato Grosso do Sul**: Rio Verde, Campo Grande-Cuiabá, *G. Hatschbach* 31952 (K, MBM, NY, RB). **Tocantins**: Serra das Ararais, *G. Gardner* 5033 p.p. (BM, K, W); Palmeiropolis, *G. Pereira-Silva* 13444 (CEN).

**BOLIVIA. Santa Cruz**: Velasco, P. N. Noel Kempff Mercado, *B. Mostacedo et al.* 1858 (MO, USZ); *S. Jiménez & E. Gutiérrez* 1385 (FTG, MO).

#### Note.

In habit, very unequal sepals and the form of its inflorescence resembling a linear-leaved form of *Ipomoea squamisepala* but differing additionally in the larger pink corolla and larger inner sepals.

### 365. Ipomoea graminifolia

Taxon classificationPlantaeSolanalesConvolvulaceae

J.R.I. Wood & Scotland, Phytokeys 88: 20. 2017. (Wood et al. 2017d: 20)

#### Type.

BRAZIL. Goiás, Fazenda Agua Fria, Alto Paraíso de Goias, cerca 10 km en direção a Teresina de Goias, 14 04 217S, 47 30 336 W, 1448 m, 20 Feb. 2001, *C. Munhoz, N. Rodrigues & K.M.O. Ramos* 2567 (holotype MO, isotypes?).

#### Description.

Completely glabrous, slender, probably clambering perennial herb, stems thin, wiry, slightly woody. Leaves sessile, 2.5–5.5 × 0.05–0.1 cm. linear-filiform, acute, minutely apiculate. Inflorescence of solitary axillary flowers; peduncles 8–18 mm; bracteoles deltoid, 1mm long, caducous; pedicels 6–8 mm, thickened upwards; sepals unequal, 5–6 × 2 mm, broadly lanceolate, acute and mucronate, margin narrow, scarious; inner 7–9 × 2 mm, oblong-lanceolate, acute, margins broad, scarious; corolla 3–3.5 cm long, funnel-shaped, pink, glabrous, limb 2.5–3 cm diam., undulate, the midpetaline bands ending in acute points; stamens included. Capsules and seeds unknown.

#### Illustration.

Figure 177.

**Figure 177.**
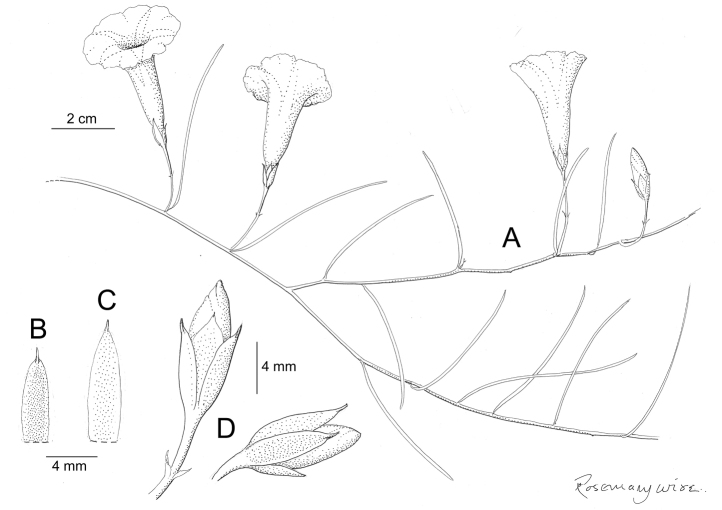
*Ipomoea graminifolia*. **A** habit **B** outer sepal **C** inner sepal **D** buds showing calyx. Drawn by Rosemary Wise from *C. Munhoz et al. 2567*.

#### Distribution.

High altitude endemic of campo limpo úmedo at 1400 m, only known from the type collection.

**BRAZIL. Goiás**: the type collection.

#### Note.

Similar to *Ipomoea procumbens* in being glabrous in all parts and with solitary axillary flowers and similar shaped unequal sepals. However, *Ipomoea graminifolia* differs in the much smaller calyx and corolla, the wiry stems, and the sessile, filiform leaves.

### 366. Ipomoea procumbens

Taxon classificationPlantaeSolanalesConvolvulaceae

Mart. ex Choisy in A.P. de Candolle, Prodr. 9: 351. 1845. (Choisy 1845: 351)

Ipomoea procumbens var. adenophylla Choisy in A.P. de Candolle, Prodr. 9: 351. 1845. (Choisy 1845: 351). Type. BRAZIL. F.C. Raben 277 (lectotype BR0000006972707, designated here).
Ipomoea kunthiana Meisn. in Martius et al., Fl. Brasil. 7: 253. 1869. (Meisner 1869: 253). Type. BRAZIL. Rio Grande do Sul, *F. Sello* 1523 (holotype B†, photo F).
Ipomoea procumbens var. longepedunculata Chodat & Hassl., Bull. Herb. Boiss., ser. 2 5: 692. 1905. (Chodat and Hassler 1905: 692). Type. PARAGUAY. Paraguarí, *E. Hassler* 5867 (lectotype G00175034, designated here; isolectotypes G, GH, K, MO, MPU, P, S, UC).
Ipomoea procumbens var. elliptica Chodat & Hassl., Bull. Herb. Boiss., ser. 2 5: 692. 1905. (Chodat and Hassler 1905: 692). Type. PARAGUAY. Canendiyú, Ipe hú [Ypé Jhu], Sierra de Maracayú), *E. Hassler* 5074 (lectotype G00175032, designated here; isolectotypes BM, G, P).

#### Type.

BRAZIL. Minas Gerais, *Martius* 964 (holotype M-0184989).

#### Description.

Prostrate or decumbent (rarely twining) herb from a woody xylopodium, glabrous or nearly so in all parts, stems somewhat woody. Leaves shortly petiolate, 4–11 × 0.2–1.5 cm, narrowly oblong to oblong-elliptic or oblanceolate, acute, base attenuate, cuneate, broadly cuneate or rounded; petioles 5–10 (–25) mm, straight and relatively stout. Inflorescence of solitary or (rarely) paired, pedunculate, axillary flowers; peduncles 0.3–3.5 cm, very variable in length, often short; bracteoles 2–3 mm, ovate, caducous; pedicels 7–16 mm, thickened upwards; sepals unequal, scarious-margined, somewhat accrescent in fruit, outer 6–11 × 4–5 mm, ovate or oblong-ovate, acute to obtuse and mucronate, inner 12–15 × 5–6 mm, oblong-elliptic, acute to obtuse; corolla 5.5–9 cm long, funnel-shaped, gradually widened from a narrow base, pink, glabrous, limb unlobed, c.3.5 cm diam. Capsules 13–15 × 7 mm, ovoid, shortly apiculate, glabrous; seeds 8 × 4 mm, minutely tomentellous.

#### Illustration.

O’Donell (1959b: 171) as *Ipomoea kunthiana*.

#### Distribution.

Locally common in cerrados and pampas, possibly stimulated by burning. NE Argentina, eastern Paraguay, Noel Kempff Park in Bolivia and central and southern Brazil.

**ARGENTINA. Corrientes**: *J. Paula-Souza* 7120 (ESA); Ituzaingó, *C. Cristóbal & A. Krapovickas* 1793 (CTES). **Misiones**: *E.L. Ekman* 1419 (GH, S); San Ignacio, *G.J. Schwarz* 5097 (GH. LIL, RB).

**PARAGUAY. Alto Paraná**: *J.E. Montes* 9879 (LIL). **Caaguazú**: *B. Balansa* 1048a (P); *E. Hassler* 9320 (BM, K); S. of Río Yhú, *Fernández Casas & J. Molero* 6441 (MO). **Canindeyú**: 25 km W of Curuguaty, *J.R.I. Wood & G. González* 28464 (FCQ). **Cordillera**: Tobatí, *E. Hassler* 7014 (BM, GH). **Guairá**: Villarica, *E. Hassler* 8713 (P). **Itapuá**: Encarnación, *L. Jiménez* 37 (SCP). **Misiones**: San Juan Bautista, *E. Lurvey* 387 (PY). **Paraguarí**: type of Ipomoea procumbens var. longepedunculata.

**BRAZIL. Bahia**: *P. T. Sano et al.* 14818 (IBUSP, K); Rio de Contas, *N. Roque et al.* 14893 (RB); Serra do Sincorá, *R.M. Harley et al.* 20725 (CEPEC, K, NY). **Dist. Fed.**: Campus do Universidad, *A. Gentry* 21441 (MO); Reserva IBGE, *M.A. da Silva* 4797 (IBGE, MO); *E. Pereira* 4816 (HB, K). **Goiás**: *A.C. Brade* 5564 (S); *A. Krapovickas & C. Cristóbal* 30186 (CTES, MBM); Serra de Caldas Nuevas, *E.P. Heringer* 13138 (NY); Goiânia, *J.R. Pirani et al.* 2089 (NY); Serra dos Cristais, *H.S. Irwin et al.* 13616 (NY); Chapada deVeadeiros, *H.S. Irwin et al.* 24571 (NY). **Mato Grosso do Sul**: Amambai, *W.G. García* 13978 (UEC). **Minas Gerais**: *P. Clausen* (K); *R. Simão-Bianchini* 1209 (CTES, SP); Mun. Perdizes, *E.K.O. Hattori et al.* 268 (F, MBM); ibid., *P.C. Duarte* 205 (HUFU); Sierra da Piedade, *L.R. Landrum* 4289 (NY) ; Serra de Espinhaço, *H.S. Irwin* et al. 23694 (MO, NY); Itacambira, *M.L. Kawasaki et al.* SPF36193 (K). **Paraná**: Fortaleza, *G. Hatschbach* 23225 (F, K, MBM, MO, NY); Jaguariaíva, *G. Hatschbach* 14003 (MBM, P); ibid., *P. Dusen* 16443 (MO). **Rio Grande do Sul**: *A. Bornmüller* 337 (GH); *Malme* 1005 (S); *P.P.A. Ferreira et al.* 640 (S); *E. Barbosa* 2532 (RB); Cacharia do Sul, *Palacios-Cuezzo* 11212 (LIL); *C. Gaudichaud* 3099 (P). **Rondônia**: Vilhena, *M.G. Silva & A. Pinheiro* 4165 (K, NY). **São Paulo**: Mun. São Roque, *S. Tsugara & Y. Otsuka* B-2234 (MO); Faz. Bocaina, *A.F.M. Glaziou* 8189 (P); San José dos Campos, *I. Mimura* 307 (K, NY); *A. Usteri* 133 (K).

**BOLIVIA. Santa Cruz**: PN Noel Kempff Mercado, Las Gamas, *Guardia et al.* 196 (USZ).

#### Notes.

Distinguished by the linear or oblong leaves which are usually cuneate at the base, rarely subtruncate but never cordate or sagittate.

We agree with O’Donell (1959b: 172) that *Ipomoea kunthiana* and *I. procumbens* cannot be separated. This can be confirmed by reference to the type specimens shown in Jstor (www.jstor.org). In general plants called *I. kunthiana* come from the southern part of the species range and have oblong-elliptic rather than oblong leaves but many intermediates are found.

*Ipomoea procumbens* forms a complex of species with *I. rupestris* and *I. granulosa*, none of which is satisfactorily resolved using *ITS* and all of which are highly variable.

### 367. Ipomoea rupestris

Taxon classificationPlantaeSolanalesConvolvulaceae

Sim.-Bianch. & Pirani, Hoehnea 32 (2): 296. 2005. (Simão Bianchini and Pirani 2005: 296)

#### Type.

BRAZIL. Minas Gerais, Mun. Santana de Riacho, *Simao-Bianchini* 11704 (holotype SP; isotypes NY, K, SPF).

#### Description.

Glabrous ascending or erect subshrub to 60 cm, with woody, tuberous xylopodium; stems glabrous, somewhat woody, bark pale brown. Leaves petiolate, 3–7 × 1–2.8 cm, broadly oblong to oblong-elliptic, obtuse and mucronate, base broadly cuneate, margin undulate to crenate, abaxially paler; petioles 0.4 –1.7 cm. Inflorescence of leafy branched, axillary, few-flowered cymes, in erect plants mostly arising in the upper leaf axils; peduncles 0.5–3 cm; bracteoles 2 mm, triangular, caducous; secondary peduncle 2–6 mm, often scabrid; pedicels 0.5–2.5; sepals unequal, outer 7–11 × 4–5 mm, ovate-elliptic, rounded to retuse, mucronate, margins scarious, inner 9–14 mm, broadly oblong, obtuse to retuse, margins scarious; corolla 4–6.5 cm long, funnel-shaped, pink, glabrous, limb 4–5 cm diam., undulate. Capsules (immature) ovoid, apiculate, glabrous; seeds not seen.

#### Distribution.

Cerrado and campo rupestre between 1000–1380 m, endemic to the planalto of central Brazil.

**BRAZIL. Bahia**: Abaíra, Boa Vista, *B. Stannard & R. Queiroz* 51763 (K, MO, NY); ibid., Campo de Ouro Fino, *R.M. Harley et al.* 51092 (K, HUEFS); Umbaranas, *L.P. de Queiroz et al.* 5218 (K). **Goiás**: Niquelândia, *M.L. Fonseca & Barros* 809 (RB, OXF); ibid., *A. Macedo* 4477* (S, US); Chapada de Veadeiros, Alto Paraíso, *T. Cavalcanti et al.* 1319* (CEN); *H.S. Irwin et al.* 24669* (FTG, NY). **Minas Gerais**: Serra do Cipó, *E. Pereira* 8918 (HB, K, RB); Serra de Mutuca, Lagôa Seca, *L. O. Williams & V. Assis* 5580 (GH); Serra do Cipo, *M.M. Arbo et al.* 4688* (CTES, FTG, K); ibid., *A.B. Joly et al.* 1061 (E); ibid., *U.C.S. Silva et al.* 33 *(HUEFS); Santana do Riacho, *A. Rapini et al*. 1628* (HUEFS); Serra do Espinhaço, *W.R. Anderson et al.* 36332* (FTG, NY, SP); ibid., *H.S. Irwin et al*. 20107* (NY); Santana de Pirapama, *W. Milliken et al.* 4305 (SPF, K); Grãu Mogol, *J.R. Pirani et al.* 850 (SPF, K). (* erect forms)

#### Notes.

This species holds together despite the varied habit because of its broadly oblong to oblong-elliptic leaves which are usually undulate to crenate on the margins and because of the usually branched inflorescence. In related species the flowers are solitary–very rarely paired in *I. procumbens*. Erect specimens cited above are indicated with an asterisk*; unmarked collections are of decumbent plants.

*Queiroz et al.* 5218 (K, HUEFS) from Bahia is odd as the inflorescence is on lateral branches with flowers mostly arising in the axils of distinct bracts resembling small leaves.

### 368. Ipomoea granulosa

Taxon classificationPlantaeSolanalesConvolvulaceae

Chodat & Hassl., Bull. Herb. Boiss., ser. 2, 5: 687. 1905. (Chodat and Hassler 1905: 687)

Ipomoea stenophylla forma glabrata Chodat & Hassl., Bull. Herb. Boiss., ser. 2, 5: 690.1905. (Chodat and Hassler 1905: 690). Type. PARAGUAY. [Canendiyú], Ipé hu [Ypé Jhu], Sierra de Maracayú: *E. Hassler* 5023 (isotypes BM, G, NY).

#### Type.

PARAGUAY. [Canendiyú], Ipe hú [Ypé Jhu], Sierra de Maracayú, *E. Hassler* 5045 (holotype G00175177, isotypes BM, F, GH, K, MPU, NY, P).

#### Description.

Undershrub from a xylopodium; stems erect, slender, wiry and somewhat woody, pale brown, glabrous, granulose, 10–15 cm high. Leaves subsessile, imbricate, 4–11.5 × 0.3–2.2 cm, linear, oblong or ovate, acute and mucronate, base tapering, cuneate, truncate to subcordate, glabrous, abaxially veins prominent; petioles 2–3 mm. Inflorescence of solitary axillary flowers; peduncles 0–2 mm, almost suppressed; bracteoles caducous, ovate, c. 1 mm; pedicels 4–10 mm, slightly thickened upwards, sometimes granulose; sepals slightly unequal, ovate, acute, (obtuse and mucronate in type), outer 10–14 × 3–6 mm, inner 13–16 × 8 mm, broader and slightly longer, margins scarious; corolla 6–8 cm long, pink, funnel-shaped, glabrous, limb 3–4.5 cm, the midpetaline bands ending in a small tooth. Capsules (immature), ovoid, apiculate, glabrous; seeds not seen.

#### Distribution.

Cerrados of eastern Paraguay and central Brazil.

**PARAGUAY. Canendiyú**: Mbaracayú Natural Reserve, Aguará ñu, *E. Zardini & S. Benítez* 51141 (ARIZ); ibid., *E. Zardini & S. Benítez* 51445 (ARIZ). **Amambay**: Sierra de Amambay, *T. Rojas in Hassler* 9826 (BM, K, P); P.N. Cerro Corá, *I. Basualdo* 4876 (FCQ, MO); Pedro Juan Caballero, *A. Krapovickas et al.* 45900 (CTES, K); ibid., *G. Hatschbach* 48501 (ARIZ, MBM, MO). **Concepción**: San Luis, *A. Schinini et al.* 35866 (CTES).

**BRAZIL. Mato Grosso do Sul**: 22 km de Ponta Porã para Antonio João, *G. Hatschbach et al.* 59080 (MBM). **Minas Gerais**: Serra do Cipo, *M.M. Arbo et al.* 4627 (CTES, FTG, SPF); ibid., Santana do Riacho, *D.C. Zappi et al.* 1531 (K); Pirapama, *D.C. Zappi et al.* 1999 (K); Presidente Joscelino, *V. C. Souza* CFRC13928 (K); Santana do Riacho, *A. Costa* (RB); ibid., *A. Rapini et al.* 1627 (HUEFS, OXF).

#### Notes.

Extraordinarily variable in terms of leaf shape (linear to ovate) and leaf size (3–4 cm long v. > 10 cm) as also in sepal size (6–7 mm v. 13–15 mm) and apex (finely acuminate to rounded). However the differences are not geographically marked and each of the three populations is variable within itself. The species is held together by the combination of granulose stems, subsessile imbricate leaves, very short peduncles, slightly unequal sepals and glabrous corollas. Molecular studies suggest this species is very closely related to and perhaps not distinct from *Ipomoea rupestris*.

*Hassler* 5023a from Ipé hu, Sierra de Maracayú is a different species with thinly pubescent corolla, stems, peduncles and sepals. The stems are not granulose and at least one leaf is forked. It is probably a form of *Ipomoea campestris* Meisn.

### 369. Ipomoea chondrosepala

Taxon classificationPlantaeSolanalesConvolvulaceae

Hallier f., Bull. Herb. Boiss. 7: 49. 1899. (Hallier 1899b: 49)

Ipomoea loefgrenii Hoehne, Anexos Mem. Inst. Butantan, Secc. Bot. i. VI: 75. 1922. (Hoehne 1922: 75). Type. BRAZIL. São Paulo, *A. Löfgren* 4334 (holotype SP).

#### Type.

PARAGUAY. Villarrica, *B. Balansa* 1072 (lectotype P03536110, designated by Wood et al. 2015: 51, isolectoypes G, K).

#### Description.

Liana climbing to at least 5 m, rarely trailing; stems rea; glabrous in all vegetative parts. Leaves petiolate, coriaceous, 4–10 × 3–7.5 cm, ovate, base shallowly cordate, apex acute and shortly mucronate, both surface glabrous; petioles 2–4.5 cm. Inflorescence often borne on leafy axillary shoots, c. 8–12 cm long; peduncles 0.2–5.5(–14) cm; bracteoles resembling tiny leaves; secondary and tertiary peduncles 8–12 mm; pedicels 2–3 cm, thicker than peduncles; sepals subequal, 13–17 × 6–10 mm, inner slightly longer, oblong elliptic, rounded, transparent, margins scarious, somewhat accrescent in fruit; corolla c. 6 cm long, narrowly funnel-shaped, dark pink, glabrous; filaments inserted c. 8 mm above the base, 10–13 mm long, only slightly unequal, anthers 5 mm; style white, c. 2.3 cm long, ovary glabrous. Capsules 15–20 × 7–10 mm, ovoid to ellipsoid, acute, angled, 4-seeded; seeds 5–10 × 4 mm (immature), the angles with silky hairs10–12 mm long.

#### Illustration.

Figures 141G, 178.

**Figure 178.**
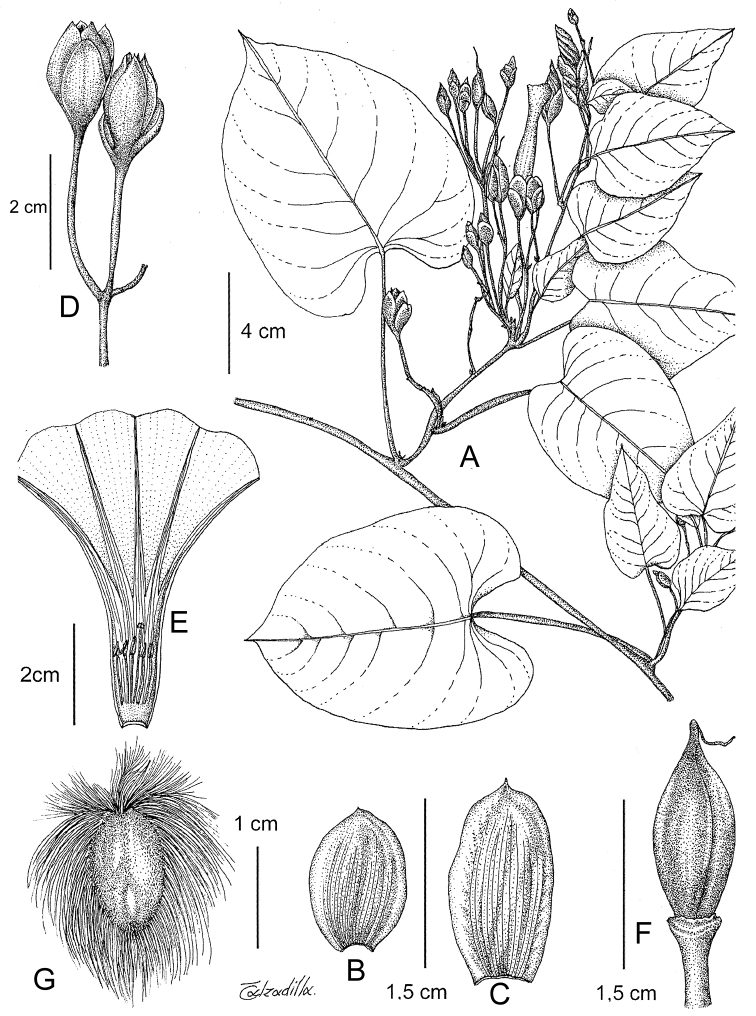
*Ipomoea chondrosepala***A** habit **B** outer sepal **C** inner sepal **D** part of inflorescence **E** corolla showing stamens and style **F** capsule **G** seed. Drawn by Eliana Calzadilla from *Wood et al.* 28286.

#### Distribution.

Seasonally moist forest in scattered locations from Paraguay, the São Paulo region of Brazil and the Santa Cruz area of Bolivia north to Colombia and Venezuela.

**PARAGUAY. Canindeyú**: camino de Lagunita a Horqueta-mi, *B. Jiménez & M. Peña* 1237 (BM, CTES, PY); Reserva Mbaracayú, *I, Basualdo* 4181 (FCQ); camino Curuguaty-Ygatimi, *J.R.I. Wood & G. González* 28469 (FCQ). **Guairá**: Independencia, Arroyo Guazú, *A. Schinini et al.* 28003 (CTES, FCQ). **Paraguarí**: P.N. Ybycuí on trail to Arroyo Corrientes, *E. Zardini & R. Velázquez* 12113 (MO, PY). **San Pedro**: Primavera, *A.L. Woolston* 821 (K, S); Laguna Blanca, *F. González Parini et al.* 1718 (FCQ).

**BRAZIL. Rondônia**: Cacoal, *Ladislao Araujo S. et al.* 823 (CEN); Ariquemes, *L. O. A. Teixeira* 503 (NY, RB). **Minas Gerais**: Serra do Espinhaço, *W.R. Anderson et al.* 35357 (FTG). **São Paulo**: type of *Ipomoea loefgrenii*.

**BOLIVIA. Cochabamba**: Chapare, *M. Bang* 1278 (GH, K, NY, MO, US). **La Paz**: Sud Yungas, *Seidel & Schulte* 2424 (K, LPB). **Santa Cruz**: Ibañez, Reserva Arubaí, 8 km de Terebinto, *D. Villarroel & I. Linneo* 599 (USZ); Angostura, *M. Nee & M. Sundoe* 52209 (LPB); Ichilo, P.N. Amboró, near Camp. Mataracú, *M. Nee & L. Bohs* 49535 (NY, USZ); near Hotel El Cafetal, Candelaria, Buenavista, *J.R.I. Wood et al.* 28286 (LPB, OXF, LPB).

**PERU.** Carretera al Marañón, 20 km del Abra de Porculla, *R. Ferreyra* 9139 (USM). **Madre de Dios**: *S.F. Smith* 1642 (MO); Río Acre, *E. Ule* 9704 (K). **Ucayali**: *Graham & Schunke* 1648 (ARIZ).

**ECUADOR. Napo**: *F. Hurtado* 572 (FTG, MO); Reserva Jatun Sacha, *C. Cerón* 859 (QCNE); Yasuri, Río Tiputini, *R. Burnham* 1303 (MICH, QCA). **Orellana**: *A. Herrera & W. Guerrero* 141 (MO, ARIZ); Res. Étnico Huaorani, *B. Freire & D. Naranjo* 539 (QCNE). **Pastaza**: *F. Hurtado et al.* 1379 (FTG, MO); *H. Lugo* 327 (GB, MO).).

**COLOMBIA. Quindio**: *E. André* 2140 (K).

**VENEZUELA. Tachira**: *J. Steyermark & R. Liesner* 119068 (MO).

#### Note.

Most collections from Amazonian Peru and Brazil have sepals with very prominent scarious margins.

### 370. Ipomoea longirostra

Taxon classificationPlantaeSolanalesConvolvulaceae

J.R.I. Wood & Scotland, Phytokeys 88: 23. 2017. (Wood et al. 2017d: 23)

#### Type.

BRAZIL. Minas Gerais, Lima Duarte, P.N. Estadual do Ibitipoca, prov. Rio do Salto, 21°42'80"S, [43°47'W] (longitude missing from label), 1200 m, 9 March 2003, fl., fr., *R.C. Forzza, L.C.S. Assis. J.G. Jardim, R. Lima, L. Menini Neto, E. Lucas, B.R. Silva, S. Edwards & D. Zappi* 3031 (holotype RB; isotypes K, NY).

#### Description.

Twining perennial of unknown height, glabrous in all vegetative parts. Leaves petiolate, 3–4 × 1.3–2.2 cm, deltoid, finely acuminate, shortly mucronate, base truncate to cordate with rounded auricles, margin denticulate, abaxially paler with prominent veins; petioles very slender, curved, 9–17 mm. Inflorescence of solitary pedunculate, axillary flowers; peduncles 10–15 mm; bracteoles caducous, not seen; pedicels noticeably stouter than peduncles 12–15 mm; sepals subequal, elliptic, glabrous, margins scarious, outer 8–11 × 4–6 mm, obtuse, inner 9–12 × 6–7 mm, rounded, usually c. 0.5 mm longer and 1 mm wider than outer sepals; corolla c. 5.5 cm long, pink, glabrous, funnel-shaped, limb 3–3.5 cm diam. Capsules 13 × 6–7 mm, conical, glabrous, strongly rostrate, the apex 4–5 mm long, persistent.

#### Illustration.

Figure 179.

**Figure 179.**
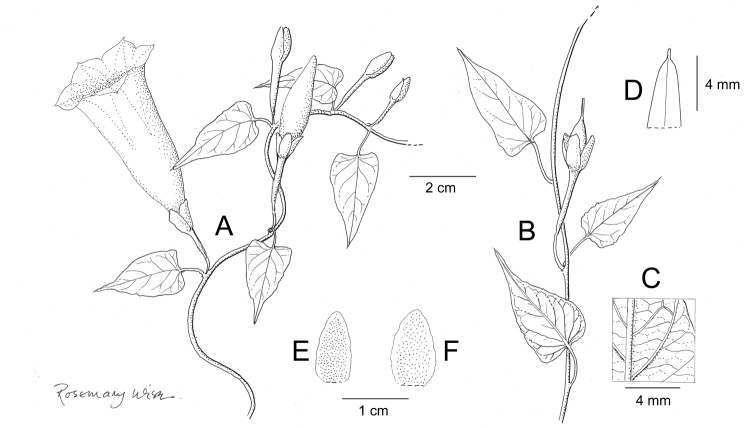
*Ipomoea longirostra*. **A** Habit showing inflorescence **B** habit showing rostrate capsule **C** abaxial leaf surface **D** leaf apex **E** outer sepal **F** inner sepal. Drawn by Rosemary Wise from *Forzza et al.* 303.

#### Distribution.

Endemic to the area of the type locality in the P.N. Estadual do Ibitipoca in Minas Gerais.

**BRAZIL. Minas Gerais**: P.N. Estadual do Ibitipoca, prov. Rio do Salto, *R.C. Forzza et al.* 4362 (NY, RB).

#### Notes.

Almost certainly related to *Ipomoea procumbens*, *I. longirostra* is distinguished by its ovate-deltoid, basally truncate leaves which are borne on slender pedicels. The subequal sepals are ovate-elliptic with distinct scarious margins, rather different from the lanceolate to ovate, usually acute to acuminate sepals of *I. procumbens*. The strongly rostrate capsule of the new species is also striking.

*C.R. Sperling et al. 6050* (FTG, K, MG, NY) from Serra dos Carajás in Pará State may belong here but the inflorescence is branched and no fruit was seen.

### 371. Ipomoea syringifolia

Taxon classificationPlantaeSolanalesConvolvulaceae

Meisn. in Martius et al., Fl. Brasil. 7: 270. 1869. (Meisner 1869: 270)

#### Type.

BRAZIL. Minas Gerais, Caldas, *A.F. Regnell* III 199 bis (S12-2168, lectotype designated here; isolectotype S).

#### Description.

Perennial, liana-like climber reaching many metres, stems glabrous, woody. Leaves petiolate, 3.5–9 × 1.5–4 cm, ovate, shortly acuminate, subtruncate to shallowly cordate, glabrous, abaxially glaucous; petioles 1–4 cm, slender. Inflorescence of shortly pedunculate axillary cymes, often laxly racemose in form and pendulous; peduncles 1–4 cm, very slender; bracteoles caducous, not seen; pedicels 1.5–2.5 cm, often exceeding peduncles; sepals unequal, glabrous, scarious-margined, outer sepals 6–9 × 4 mm, elliptic, obtuse, inner 9–10 × 4–5 mm, broadly elliptic, rounded; corolla 4–5 cm long, lemon-yellow, glabrous, abruptly widened above base so appearing inflated, limb c. 3 cm diam., shallowly lobed. Capsules ovoid, 12–13 × 7–8 mm, glabrous; seeds 5 × 3 mm, pilose with reddish hairs 6–8 mm long.

#### Illustration.

**Figure 180.**
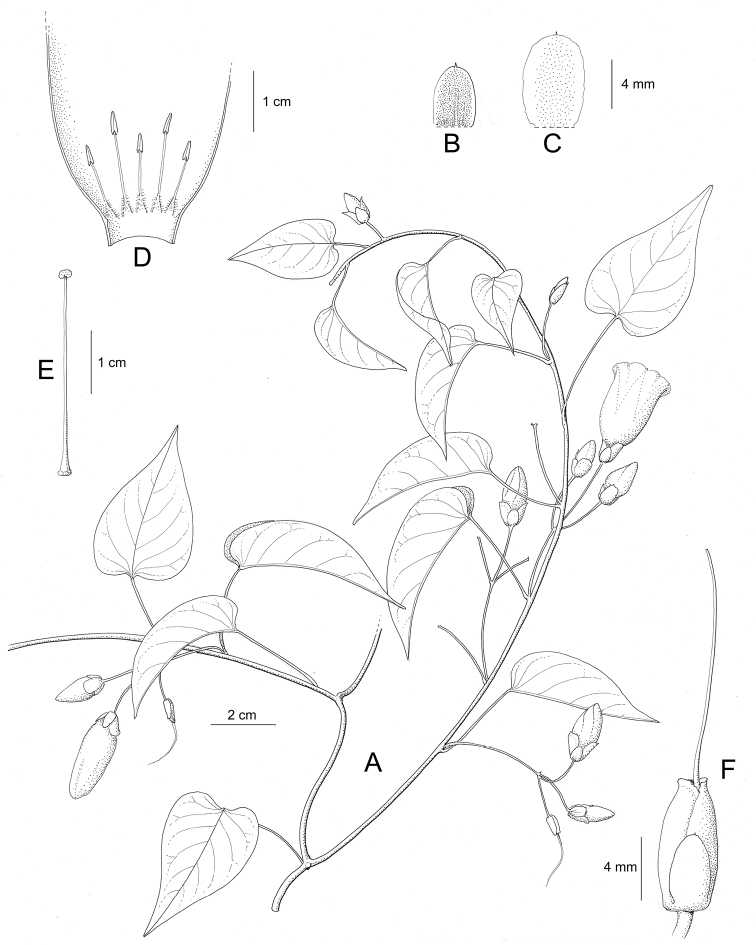
*Ipomoea syringifolia*. **A** habit **B** outer sepal **C** inner sepal **D** corolla opened out to show stamens **E** ovary and style **F** fruiting calyx. Drawn by Rosemary Wise from *Stutz de Ortega* 1426.

O’Donell (1959b: 248); Figures 6D, 8N, 180.

#### Distribution.

Atlantic forest and Paraná forest relics; southern Brazil and neighbouring parts of Paraguay and Argentina.

**ARGENTINA. Misiones**: Dept. Eldorado, *S.G. Tressens et al.* 5570 (CTES, K); Dept. San Pedro, *H. Keller & Franco* 9717 (CTES, MO).

**PARAGUAY. Alto Paraná**: *Stutz de Ortega* 1426 (G).

**BRAZIL. Espirito Santo**: Anchieta, *A. Stival-Santos* 555 (RB). **Minas Gerais**: *A. Glaziou* 18382 (K); Viçosa Agric. College, *Y. Mexia* 4430 (BM, K, MO, S); Caldas, *C.W. Mosén* 4494 (S). **Paraná**: Mun. Cel. Vivida, *G. Hatschbach* 26375 (CTES, MBM, K, S); Rio Branco do Sul, *J.M. Silva & G.L. Esteves* 1304 (MBM); Therezina, *P. Dusen* 11146 (GH, NY, S); Faz. Reserva, *J.C. Lindeman & J.H. de Haas* 4684 (K). **Rio de Janeiro**: Petropolis, *C. Goes & Constantino* 3 (RB); Organ Mts, *J. Miers* s.n. (BM); *A. Glaziou* 8819 (K). **Santa Catarina**: *F. Plaumann* 433 (RB). **São Paulo**: *Heiner* 432 (S); *J. Weir* 506 (K); *Martius* s.n. (M).

#### Typification.

None of the original syntypes in LE, K, M and S are very satisfactory, either lacking corollas or with badly eaten leaves. The lectotype selected here is probably the best from a not very high quality selection of specimens.

#### Note.

This is a distinctive species because of the very lax inflorescence and the pendulous, yellow-green campanulate corollas which are abruptly inflated at the apex of the calyx.

### 372. Ipomoea decemcornuta

Taxon classificationPlantaeSolanalesConvolvulaceae

O’Donell, Lilloa 26: 366. 1953. (O’Donell 1953a: 366)

#### Type.

MEXICO. Est. México, Temascaltepec, Nanchititla, *G.B. Hinton* 4991 (holotype US00111386, isotypes GBH, GH, F, K, MICH).

#### Description.

Climbing herb, glabrous or with a few hairs at the nodes. Leaves petiolate, 5.5–17 × 4.5–12.5 cm, ovate, cordate, finely acuminate, terminating in a long hair-point, adaxially with a few appressed hairs, abaxially glabrous; petioles 3.5–6 cm. Inflorescence of pedunculate axillary cymes; peduncles 2–6 cm, winged; bracteoles 1–3 mm, oblong-lanceolate, caducous; pedicels 3–4.5 mm; sepals glabrous, outer 2.5–3 mm, oblong-elliptic, obtuse and mucronate, abaxially with three wings terminating in mucros c. 3 mm long, inner 3–3.5 mm, elliptic, obtuse with a single wing terminating in a mucro, middle sepal 2-winged; corolla 2.5–3 cm long, funnel-shaped, tube white, shallowly lobed, lobes probably purple, glabrous. Capsules subglobose, > 3 mm wide, rostrate, glabrous; seeds not known.

#### Distribution.

Endemic to central Mexico, occurring in a few scattered localities between 1000 and 2000 m.

**MEXICO. Est. México**: type collection. **Michoacán**: Puerto Zarzamora, Coalcomán, *G.B. Hinton* 12271 (K). **Oaxaca**: km 662, Piedra Larga a Miahuatlan, *R. Cedrillo* 1825 (MEXU). **Sinaloa**: Sierra Surutato, *H.S. Gentry* 6477 (ARIZ, MEXU).

#### Note.

The strongly winged peduncles are very distinct as are the dentate (sometimes described as winged) sepals. The latter suggests a connection with *Ipomoea tentaculifera* and forms of *I. pedicellaris*, rather than the Quamoclit Clade, in which it has been sometimes placed. The funnel-shaped, purplish corolla with a white tube and included stamens and 2-locular ovary also rule out the latter. The placement here is uncertain, being based on an incomplete molecular sequence.

### 373. Ipomoea tenera

Taxon classificationPlantaeSolanalesConvolvulaceae

Meisn. in Martius et al., Fl. Brasil. 7: 289. 1869. (Meisner 1869: 289)

#### Type.

BRAZIL. Rio São Francisco, Salgado, Minas Gerais, *Martius* s.n. (lectotype M0184955, designated by Delgado Junior et al. 2017).

#### Description.

Slender, probably annual, glabrous twining herb. Leaves petiolate, divided into 5 separate leaflets, leaflets 3–6 × 0.2–0.7 cm, linear, apiculate, acuminate at both ends; petioles 1.5–2 cm. Flowers solitary, axillary; peduncle slender, 1–4 cm, often coiled and often bent 90° at apex; bracteoles 2 mm, linear; pedicels 5–7 mm; sepals unequal, outer sepals 7 mm, ovate, acuminate, margin strongly fimbriate below, base abruptly truncate to sagittate; inner sepals not seen; corolla 2–2.5 cm long, narrowly funnel-shaped with narrow tube, c. 0.5 cm diam., glabrous, pink. Capsules globose, glabrous; seeds not seen.

#### Distribution.

Endemic to the semi-arid NE of Brazil, where it appears to be uncommon.

**BRAZIL. Bahia**: Rio São Francisco, *L.P. de Queiroz* 16215 (HUEFS). **Ceará**: Fazenda Iracema, Quixadá, *E. Nunes* s.n. (EAC, RB). **Minas Gerais**: type collection. **Paraíba**: Sousa, *B. Pickel* 3894 (F, IPA). **Pernambuco**: Petrolina, *E.P. Heringer* 176 (PEUFR, RB, UB). **Rio Grande do Norte**: Serra Negra do Norte, Est. Eco, do Seridó, *R.T. Queiroz* 327 (SP, UFRN).

#### Note.

The coiled, or at least sharply bent, pedicels suggest a close relationship with *Ipomoea heptaphylla* but this species is easily distinguished by the fimbriate outer sepals. We have not been able to examine the inner sepals or the seeds, which are not described above.

### 374. Ipomoea heptaphylla

Taxon classificationPlantaeSolanalesConvolvulaceae

Sweet, Hort. Brit., ed. 2: 372. 1830. (Sweet 1830: 372)

Convolvulus heptaphyllus Roxb., Fl. Ind., ed. 2, 2: 66. 1824. (Roxburgh 1824: 66), nom. illeg., non Convolvulus heptaphyllus Rottler & Willd.(1803). Type. Icon. no. 1950 by Roxburgh (K, lectotype, designated by Verdcourt 1961: 11).
Ipomoea radicans Bertero ex Choisy in A.P. de Candolle, Prodr. 9: 387. 1845. (Choisy 1845: 387), nom. illeg., non Ipomoea radicans Blume (1826). Type. JAMAICA. *Bertero*s.n. (wherabouts uncertain, ?TO).
Ipomoea capillifolia Bertero ex Choisy in A.P. de Candolle, Prodr. 9: 388. 1845. (Choisy 1845: 337), nom.nud.
Ipomoea wrightii A. Gray, Syn. Fl. N. Amer. 2: 213. 1878. (Gray 1878: 213). Type. UNITED STATES. Texas. *C. Wright*s.n. (holotype GH00054467, isotype GH).
Ipomoea spiralis House, Muhlenbergia 3: 40 1907. (House 1907a: 40). Type. MEXICO. *E. Palmer* 24 (isotype US).
Ipomoea gracilipes Hassl., Fedde, Repert. Spec. Nov. Regni Veg.9: 158. 1911. Hassler (1911: 158). Type. PARAGUAY. zwischen Río Apa und Río Aquidaban, Rojas in Hassler 10907, *K. Fiebrig* 4936, 5744 (syntypes BM, GH, K000612826!, M).
Ipomoea pulchella var. lineariloba Hassl., Fedde, Repert. Spec. Nov. Regni Veg.9: 158. 1911. (Hassler 1911: 158). Type. PARAGUAY. Gran Chaco, Santa Elisa, *E. Hassler* 2762 (lectotype G00175236, designated here; isolectotypes BM, G, K, P).
Ipomoea pulchella auct., non Roth (1821), which is I. cairica (L.) Sweet (Verdcourt, 1961).

#### Type.

Based on *Convolvulus heptaphyllus* Roxb.

#### Description.

Twining annual herb, plant completely glabrous in all parts. Leaves petiolate, divided into 5–7 separate sessile leaflets, leaflets 3–7 × 0.3–1 cm, narrowly lanceolate, acuminate at both ends; petioles 2.5–5.5 cm. Flowers solitary (rarely paired), axillary, pedunculate; peduncles slender, flexuose and sometimes coiled, 3–6 cm long; bracteoles minute, c. 1 mm, scale-like, caducous; pedicels 5–8 mm, stouter than peduncles; sepals subequal, 5–7 mm, scarious-margined, outer 4–5 × 2.5–3 mm, ovate, obtuse, abaxially slightly muricate, inner 5–6 × 3 mm, broadly oblong, rounded; corolla 1.7–2.2 cm long, funnel-shaped, pink, glabrous; limb c. 1 cm diam. Capsules 10 × 7 mm, ovoid, glabrous; seeds 5 × 2.5 mm, tomentose.

#### Illustration.

**Figure 181.**
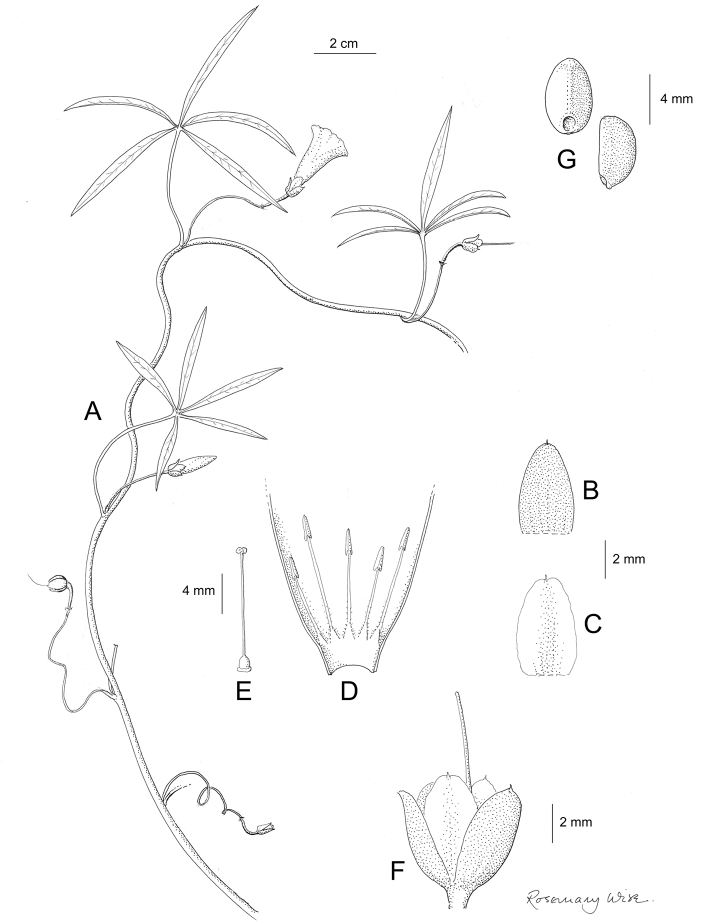
*Ipomoea heptaphylla*. **A** habit **B** outer sepal **C** inner sepal **D** corolla opened up to show stamens **E** ovary and style **F** fruiting calyx **G** seeds. Drawn by Rosemary Wise **A–F** from *Wood & Soto* 27105; **G** from *De Egea & Peña-Chocarro* 272.

Figure 181; Acevedo-Rodríguez (2005: 183) as *Ipomoea wrightii*.

#### Distribution.

Widely distributed throughout the neotropics but scattered, often ephemeral, never very common and unrecorded in some areas, for example Colombia, where it might be expected to occur. It seems to favour dry parts of islands and seasonally dry areas such as the Brazilian Caatinga and the Chaco region.

**PARAGUAY. Alto Paraguay**: Gabino Mendoza-Lagarenza, *R. Degen & F. Mereles* 3288 (FCQ); Capitan Pablo Lagerenza, *A. Charpin & L. Ramella* 21584 (G). **Boquerón**: *Krapovickas et al.* 45288 (CTES). **Misiones**: IslaYacyretá, *S. Keel et al.* 1365 (FCQ). **Presidente Hayes**: Santa Asunçion, *J. de Egea & M. Peña-Chocarro* 272 (BM, FCQ). **San Pedro**: *A. Krapovickas & C. Cristóbal 44907* (CTES).

**BRAZIL. Bahia**: Lagoa da Eugenia, *R.M. Harley et al.* 16282 (K, MO, NY, RB); João Dorado, *L.V. Vasconcelos* 462 (RB). **Ceará**: Ipaumirim, *J.L. Costa-Lima* 1208 (HUES, RB); Penaforte, *A.P.B. Santos* 2 (HVASF). **Mato Grosso**: Caceres, 9 km ENE de Porto Esperidiao, *A. Krapovickas et al.* 40113 (CTES); Barão de Melgaço, *G. Martinelli* 18598 (RB). **Minas Gerais**: Barbacena, *A.F.M. Glaziou* 13028 (BM, K, NY, P); *G. Hatschbach et al.* 52183 (CTES). **Paraíba**: Cajazeiras-Brejo das Freiras, *C. Miranda* s.n. (JPB). **Pernambuco**: *G. Gardner* s.n. [May 1838] (BM, K); Pedra Furada, *M. Grillo* 68 (PEUFR). **Piauí**: *A. Krapovickas et al.* 38612 (CTES). **Rio Grande do Norte**: José de Penha, *J.L. Costa-Lima* 1362 (RB).

**BOLIVIA. Santa Cruz**: Chiquitos, El Tinto, *J.R.I. Wood & D. Soto* 27105 (K, LPB, USZ); Cordillera, P.N. Kaa-Iya, *A. Fuentes & G. Navarro* 2524 (LPB, USZ); Germán Busch, *R. Frey et al.* 507 (K, MO).

**PERU. Lambayeque**: *C. Abad & J. Orrillo* s.n. (USM); East side of Chiclayo, *J. Hudson* 946 (MO). **Tumbes**: *A. Sagástegui* 14597 (MO).

**ECUADOR. Galapagos Islands**: *Fagerlind & Wibom* 2807 (S); San Cristóbal, *C. Huttel* 1766 (QCA). **Guayas**: *G. Harling & L. Andersson* 14616 (GB).

**VENEZUELA. Anzoátegui**: *W.A. Díaz* 6724 (MO). **Falcón**: *R.C. Wingfield* 7189 (MO).

**COSTA RICA.** Bagaces, P.N. Palo Verde, *U. Chavarría* 1046 (MO).

**EL SALVADOR.** Ahuachapan, *J.M. Rosales* 2309 (MO).

**GUATEMALA.** Petén, P.N.Tikal, *C.L. Lundell* 16907 (MO).

**MEXICO. Campeche**: La Tuxpeña, *C.L. Lundell* 979 (K, MO, US). **Jalisco**: La Huerta, Rancho Cuixmala, *E. Lott et al.* 2869 (F, MEXU, MO, NY). **Sonora**: Bácum, *R. Felger & F.W. Rechenbacher* 85-1264 (ARIZ, MEXU, TEX).

**UNITED STATES. Alabama**: Houston, Dotham, *J.R. McDonald* 8102 (IBE). **Arkansas**: Drew, *R. D. Thomas et al.* 158031 (MISS). **Georgia**: Calhoun Co., *J.R. Allison* 9468 (GA). **Florida**: fide Wunderlin and Hansen (2011: 392). **Louisiana**: Tendal, *D. Dixon* 4298 (VSC). **Mississippi**: Lowndes, *J.D. Bryson* 20417 (ARIZ, MMNS). **Tennessee**: *R. Kral* 64384 (FSU). **Texas**: type of *Ipomoea wrightii*.

**CUBA. Cienfuegos**: *R.A. Howard* 5398 (NY). **La Habana**: *Bro. León* 13711 (HAC, HAJB, NY). **Oriente**: *E.L. Ekman* 1412 (S), 7364 (NY, S). **Villa Clara**: *J.G. Jack* 6711 (A, NY, S).

**JAMAICA.***C.D. Adams* 11896 (BM, MO); *G.R. Proctor* 38167 (MO, NY).

**HAITI.** Port-au-Prince, *E.L. Ekman* H2074 (NY, S).

**PUERTO RICO.***P. Sintenis* 3619 (BM, K, S).

**LESSER ANTILLES. Antigua**: *H.E. Box* 1201 (BM, MO). **Guadeloupe**: *A. Duss* 4115 (NY). **Barbados**: fide Gooding et al. (1965).

**NETHERLANDS ANTILLES. Aruba**: *R.A. Howard* 20303 (NY). **Bonaire**: fide Proosdij (2012). **Curaçao**: *A.S.J. van Proosdij et al.* 568 (NY, U).

#### Notes.

Distinguished from other species with 5-foliolate leaves, by the annual habit, small flowers and slender flexuose peduncles.

The plant from which the type of this species was drawn appeared amongst cultivated material in the Calcutta Botanic Garden (Roxburgh 1824) but the species is otherwise unknown in the Old World.

### 375. Ipomoea macedoi

Taxon classificationPlantaeSolanalesConvolvulaceae

Hoehne, Arq. Bot. Estado São Paulo 2: 110. 1950. (Hoehne 1950: 110)

#### Type.

BRAZIL. Minas Gerais, Cachoeira Dourada do Rio Paranaiba em Ituiutaba, 9 May 1948, *A. Macedo* 1066 (holotype SP000576, isotypes BM, S, SPF).

#### Description.

Slender twining or trailing herb, probably annual, stems glabrous. Leaves petiolate, 3(–5)-foliate with distinct truncate(and very briefly cuneate) base, lateral lobes oblong-lanceolate, obtuse with a basal obtuse to acute auricle/lobe, central lobe narrowly oblong-elliptic, obtuse, mucronate, adaxially glabrous, abaxially paler, glabrous to thinly pilose, esp. on veins; petioles 3–5 cm, thinly pilose with multicellular hairs. Flowers solitary (rarely paired); peduncle very short, 0–3 mm, glabrous; bracteoles 5–8 mm, filiform, persistent; pedicels 5–15 mm, thinly pilose; outer sepals 13–20 × 8–10 mm, ovate, acute, base cordate and auriculate, inner similar but smaller, both glabrous to thinly pilose; corolla c. 2.5 cm long, white, glabrous. Capsules subglobose, 9 mm, glabrous, the style somewhat persistent; seeds unknown.

#### Illustration.

Hoehne (1950: t. 46).

#### Distribution.

Endemic to the Brazilian planalto found very locally in cerrado.

**BRAZIL. Minas Gerais**: Municipio Ituiutaba, Fazenda San [Terejuba], *A. Macedo* 1807 (MO, RB, SP69893).

#### Note.

Very distinct because of the truncate base to the 3-lobed leaves.

### 376. Ipomoea apodiensis

Taxon classificationPlantaeSolanalesConvolvulaceae

J.R.I. Wood & Scotland sp. nov.

urn:lsid:ipni.org:names:77208083-1

#### Type.

BRAZIL. Rio Grande do Norte, Felipe Guerra, Cachoeira do Roncador, -5,57943333S, -37,67805556W, 56 m., 21 Apr 2016, *M. Marinho, A.S. Soares & L.O.F. Sousa: 250* (holotype PEUFR).

**Diagnosis**. Differs from *Ipomoea macedoi* by the entire or shallowly 3-lobed leaves, which often appear more or less entire with broad lateral teeth, the base cordate (not all leaves 3–5-lobed, the base truncate and the lobes deeply cut and oblong-elliptic in outline), by the longer pedicels 2.2–7 cm in length, the longer peduncles 1–3 cm long and by the much longer pale pink corolla 4–5 cm in length.

#### Illustration.

Morais et al. (2017: 74).

#### Distribution.

Endemic to Rio Grande do Norte where it is found at low altitudes on the Chapada de Apodi and at the Cachoeira do Roncador in Felipe Guerra.

**BRAZIL. Rio Grande do Norte**: several specimens cited by Morais et al. 2017.

#### Note.

This species was originally published as the first record of *Ipomoea macedoi* from NE Brazil (Morais et al. 2017). However, the description and the accompanying images make it clear that it is a distinct species and is here published as such.

### 377. Ipomoea pantanalensis

Taxon classificationPlantaeSolanalesConvolvulaceae

J.R.I. Wood & C. Urbanetz, Kew Bull. 71 (6): 2. 2016. (Wood et al. 2016a: 2)

#### Type.

BRAZIL. Mato Grosso do Sul, Mun. Corumbá, Fazenda Nhumirim, caminho para o Caronal, Nhecolandia, 90 m, 18°59'S, 56°39'W, 31 Jan. 1990, *A. Pott & O.C. de Souza* 5475 (holotype CPAP, isotypes MBM, SP).

#### Description.

Slender herb, probably perennial; stems sometimes creeping and rooting at the nodes, sometimes ascending and twining up to c. 30 cm, glabrous. Leaves petiolate, sometimes dimorphic; petioles 0.8–3 cm, glabrous or with a few scattered hairs; lamina glabrous or thinly pubescent, abaxially pale green, base cuneate, occasionally ovate-deltoid, 1 –5.6 × 1.7–4.5 cm, acute, more commonly digitately 3–5-lobed to near the base with lobes 1–4.8 × 0.1–0.6 cm, linear or lanceolate, acute. Inflorescence of solitary axillary flowers; peduncles 1–3 cm; bracteoles persistent, 4 × 0.5 mm, ciliate; pedicels 1–3 cm, often dark red, thinly pilose; sepals very unequal, outer sepals 15–24 × 3–6 mm, deltoid, acute to shortly mucronate, base truncate with a simple or notched lateral tooth, margin ciliate, inner sepals 10–18 × 3–4 mm, similar in shape but lacking the distinct lateral teeth, abaxially pubescent in the central area, margins glabrous; corolla 3.8–5.5 cm long, pink, funnel-shaped, glabrous; limb c. 2.5 cm diam., the lobes apiculate; stamens included; ovary glabrous. Capsules and seeds not seen.

#### Illustration.

Figures 3F, 182.

**Figure 182.**
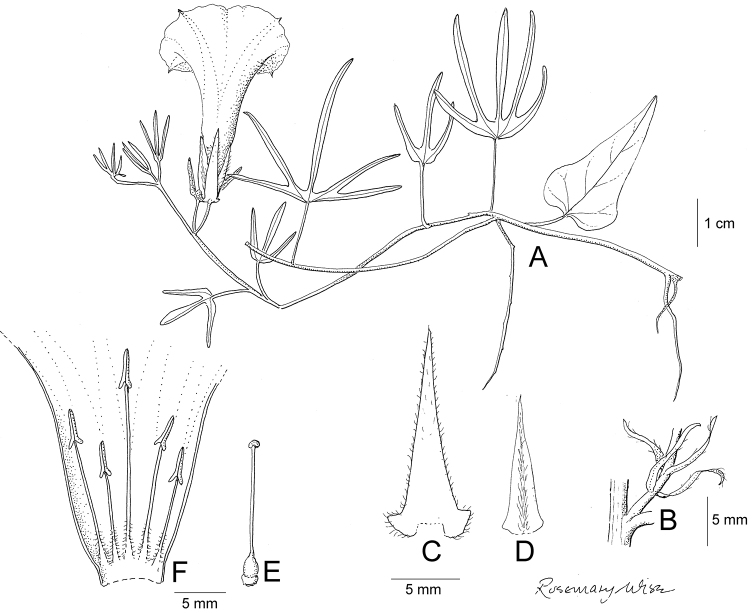
*Ipomoea pantanalensis*. **A** habit **B** peduncle with bracteoles **C** outer sepal **D** inner sepal **E** ovary & style **F** corolla opened up to show stamens. Drawn by Rosemary Wise from *A. Pott et al.* 6399.

#### Distribution.

Known certainly from a few collections from the Corumbá region but perhaps also in Piauí.

**BRAZIL. Mato Grosso do Sul**: *A. Pott et al.* 6399 (CPAP, K).

#### Notes.

Very distinctive when both leaf forms present but also easily distinguished by the truncate base of the outer sepals.

A specimen from Piauí, Castelo do Piauí, *J.M. Costa & D.P. Coutinho* 204 (HUEFS, TEPB) appears to belong to this species. It is described as a creeping herb and has the same distinctive sepals but differs in the broader, oblong-elliptic, more hirsute leaf lobes. Without further collections it is impossible to say whether this is a distinct species or merely a form of *Ipomoea pantanalensis*.

### 378. Ipomoea subrevoluta

Taxon classificationPlantaeSolanalesConvolvulaceae

Choisy Prodr. [A.P. de Candolle] 9: 386. 1845. (Choisy 1845: 386)

Ipomoea dactylophylla Griseb, Cat. Pl. Cub. 203. 1866. (Grisebach 1866: 203). Type. CUBA. *C. Wright* 3093 [1650] (holotype GOET, isotypes BM, GH, HAC, K, MO, P, YU).
Ipomoea subrevoluta var. induta Hassl., Repert. Spec. Nov. Regni Veg. 9: 159. 1911. (Hassler 1911: 159). Type. PARAGUAY. [Concepción], zwischen Río Apa and Río Aquidaban, *K. Fiebrig* 4975 (lectotype G00175185, designated here; isolectotypes BM, G, GH, K, L, P).
Ipomoea subrevoluta forma acutiloba Hassl. [as var. genuina Hassl. forma acutiloba ], Repert. Spec. Nov. Regni Veg. 9: 159. 1911. (Hassler 1911: 159). Type. PARAGUAY. Chaco, *K. Fiebrig* 1288 (lectotype G00175186, designated here; isolectotypes G, K, M).

#### Type.

GUYANA. *C.S. Parker* s.n. in Herb. Lindley (holotype CGE14419!, isotypes K!).

#### Description.

Twining perennial herb, completely glabrous in all parts. Leaves petiolate, divided into 5(–7) separate sessile leaflets, leaflets 2. 5–6 × 0.1–0.4(–0.7) cm, linear to narrowly oblong, apiculate, acuminate at base; petioles 0.5–5 cm. Inflorescence of 1(–3)-flowered, axillary, pedunculate cymes; peduncles slender, 0.8 –1.8 cm, often flexuose; bracteoles 1.5 mm, deltoid, caducous; pedicels 1–1.5 cm, stouter than peduncles; sepals subequal, 5–6 × 2–3 mm, ovate, shortly apiculate, pale green; corolla 4–6 cm long, funnel-shaped, pink, glabrous, limb c. 4 cm diam., unlobed. Capsules 12–14 cm long, ovoid, glabrous; seeds 5–6 mm, dark brown, nearly glabrous.

#### Illustration.

O’Donell (1959b: 246); Figure 58J–L.

#### Distribution.

Widely distributed in wetlands in tropical South America from Colombia and the Guianas south to northern Argentina but usually in small quantity in scattered populations; also present on the Isla de Juventud [Pinos], Cuba, perhaps an ancient introduction by birds. Characteristic of small streams with moving water in open areas below 500 m.

**ARGENTINA. Corrientes**: Dept. Mercedes, *S.G. Tressens et al.* 3683 (CTES, K). **Misiones**: *B. Berteroni* 5831 (LIL).

**PARAGUAY.** Concepción. Type of Ipomoea subrevoluta var. induta.

**BRAZIL. Amapá**: Río Urucaua, *J.M. Peres & L. Westra* 48887 (NY). **Bahia**: Oeste, Formosa do Rio Preto, *A.B. Xavier & M.L. Guedes* 289 (ALCB). **Mato Grosso**: *G.T. Prance* 26063 (NY); Poconé, *A. Macedo* 697 (NY). **Mato Grosso do Sul**: Corumbá, *P. da Silva & M. Moreira* 20 (CPAP); Rio Paraguai, Pantanal de Cáceres, *V.J. Pott* 2045 (CPAP, CTES). **Paraná**: Río Paraná, *J.C. Lindeman & de Haas* 4391 (NY). **Pernambuco**: Rio São Francisco, Cabrobó, *M.V. Meiado* 847 (HVASF). **Rio Grande do Norte**: Chapada do Apodi, *E.C. Tomaz & A.S. Pontes* 37 (UFRN). **Tocantins**: Lagoa do Raimuno, *E.R. Santos* 1956 (HUTU). Records from Amazonian Brazil in Flora do Brasil 2020 under construction may or may not be correct.

**FRENCH GUIANA.***Cremers* 5229 (P); *J.J. de Granville* 9146 (P).

**SURINAM.***W.R. Hostman* 538 (BM).

**GUYANA.** Moreru Lake, *R.J.A. Goodland* 1064 (MO, NY).

**BOLIVIA. Beni**: Ballivián, 40 km N. of Santa Rosa, *S.G. Beck* 20707 (LPB); Cercado, Laguna Suárez, *N. Ritter & M. Ritter* 3367 (BOLV, LPB); Marbán, Laguna Bolivia, *López al.* 83 (LPB); Moxos, P.N. Isiboro Sécure, *E. Gutiérrez & G. Navarro* 1641 (USZ). **Pando**: Manuripi, Conquista, *E. de la Sota* 993 (LIL). **Santa Cruz**: Ñuflo de Chávez, Concepción, *T.J. Killeen* 2403 (FTG, LPB, NY, F, USZ); Perseverancia, *I. G. Vargas* 589 (USZ); Ángel Sandoval, A.N.M.I. San Matías, *A. M. Carrión & E. Rivera* 790 (USZ); Velasco, El Refugio, *R. Guillén & S. Coria* 1585 (ARIZ, MO, USZ); Santa Rosa de la Roca, *J.R.I. Wood et al.* 27813 (K.LPB, USZ).

**PERU. Loreto**: Reserva Nac. Pacaya-Samiria, *C. del Carpio* 2276 (MO, USM).

**COLOMBIA. Antioquia**: *E. Rentería* 1930 (COL). **Chocó**: *J. León* 645(COL). **Córdoba**: Montería: *B. Anderson* 1929 (COL, K); **Magdalena**: *M.T. Dawe* 460 (K). **Córdoba**: Montería: *B. Anderson* 1929 (COL, K).

**VENEZUELA. Delta Amacuro**: Antonio Díaz, *J. Steyermark et al.* 114812 (K, MO).

**CUBA.** Isla de Pinos, *E.L. Ekman* 12283 (S).

**TRINIDAD.** Fide Hill and Sandwith (1953).

#### Note.

Usually easily recognised by the very short sepals combined with the 5-foliolate leaves and relatively large glabrous flower.

••• Clade D (species 379–388) comprises a small clade of entirely American species. All species are herbaceous but show no other obvious common character.

### 379. Ipomoea bahiensis

Taxon classificationPlantaeSolanalesConvolvulaceae

Willd. ex Roem. & Schult., Syst. Veg. 4: 769. 1819. (Roemer and Schultes 1819: 769)

Ipomoea salzmannii Choisy, Mém. Soc. Phys. Genève 8(1): 59 [137]. 1838. (Choisy 1838: 59 [137]). Type. BRAZIL. Salzmanns.n. (lectotype M0184904, designated here).
Ipomoea salzmannii var. uniflora Choisy in A.P. de Candolle, Prodr. 9: 379. 1845. (Choisy 1845: 379). Type. BRAZIL. Minas Gerais, Salgodo, *Martius*s.n. (holotype M0184905).
Ipomoea bahiensis var. uniflora (Choisy) Meisn. in Martius et al., Fl. Brasil. 7: 269. 1869. (Meisner 1869: 269).
Ipomoea bahiensis var. sagittifolia Meisn. in Martius et al., Fl. Brasil. 7: 269. 1869. (Meisner 1869: 269). Type. BRAZIL. Rio São Francisco, *Gardner* 1359 (lectotype K000944834, designated here).
Quamoclit rochai Hoehne, Anexos Mem. Inst. Butantan, Bot. 1, fasc. 6: 79. 1922. (Hoehne 1922: 79). Type. BRAZIL. Ceará, *da Rocha* 4090 (holotype SP).
Ipomoea rochai nom. nud., in synon with Quamoclit rochai.

#### Type.

BRAZIL. *T. Hoffmannsegg* s.n. (holotype B-W 03753-010).

#### Description.

Trailing or climbing perennial herb to 1.5 cm, stems glabrous. Leaves petiolate, 3–8 × 0.8–5.5 cm, ovate-deltoid, acuminate and mucronate, base cordate with rounded to acute auricles, glabrous or puberulent, abaxially pale green; petioles 0.5–2 cm. Inflorescence of few-flowered, dense, pedunculate axillary cymes; peduncles 0.5–4(–12) cm long, often very short, puberulent or glabrous; bracteoles 1.5–6 × 0.5–1.5 mm, ovate, acute, scarious except for green midrib, caducous; pedicels 3–7 mm; sepals unequal, somewhat variable in structure, glabrous, fleshy, white or pale green with darker spots and green apex, abaxially often with a prominent tooth-like appendage; outer sepals 6–7 × 3 mm, obovate or elliptic, obtuse, inner 9–10 × 4 mm, suborbicular-obovate, rounded to truncate with prominent angles, margin scarious; corolla 4–5.5 cm long, white, lilac or pink, glabrous, funnel-shaped, limb c. 4 cm diam., unlobed. Capsules subglobose, 7–8 mm, shortly rostrate, glabrous; seeds 5 × 3 mm, lanate on margins, pubescent on faces.

#### Illustration.

**Figure 183.**
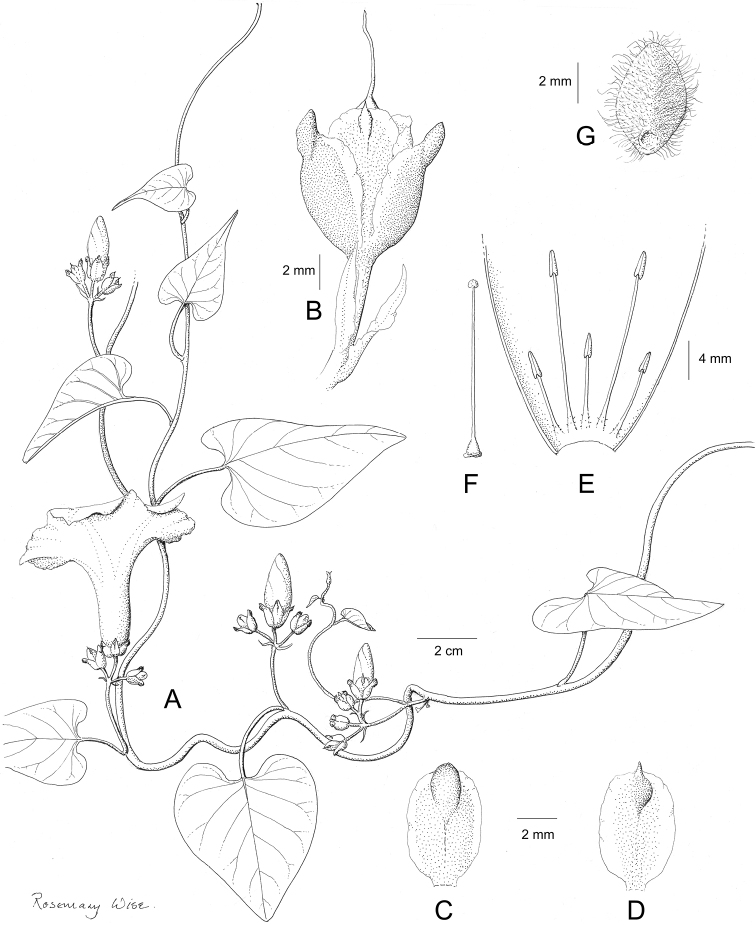
*Ipomoea bahiensis*. **A** habit **B** calyx **C** outer sepal **D** inner sepal **E** corolla opened out to show stamens **F** ovary and style **G** seed. Drawn by Rosemary Wise **A–F** from *Wood et al.* 27797; **G** from *Wood et al.* 27893.

Figures 2F, 9D; 183.

#### Distribution.

*Ipomoea bahiensis* is widespread in eastern Bolivia and Brazil and is typical of disturbed bushy places. It is especially common in NE Brazil but apparently absent south of about 18° latitude except in the Rio de Janeiro region. For an account of its mass flowering after fire in eastern Bolivia, see Wood (2019).

**BRAZIL. Alagoas**: *G. Gardner* 1359 (K). **Amazonas**: Itacoatiara, *W.J. Lowe* 4274 (K). **Bahia**: Feira de Santana, *I.M. Fernandes* 2 (K); Ibotirama, Upper São Francisco River, *R.M. Harley* et al. 22014 (K); Bom Jesus da Lapa, *R.M. Harley* et al. 21532 (K); Salvador, Itapoã, *Bautista & Pinto* 802 (K). **Ceará**: *Drouet* 2546 (S); Quixerí, Chapada do Apodi, *M.A. Figueiredo et al.* 626 (K); near limits with Pernambuco, *L. Duarte & A. Castellanos* 33393 (HB, K). **Goiás**: Minacu, *G. Pereira-Silva* 5373 (CEN). **Mato Grosso**: *C.A.M. Lindman* 3543 (S); Cáceres-Cuaibá, *W. Werneck* 64 (CEN, K); Xavantina–São Felix, *R.R. de Santos et al.* 1799 (K); Novo Mundo, *G.S. Henicka et al.* 22 (K). **Pará**: Serra do Piría, *R.C. Forzza et al.* 5867 (K); Nazaré de Para, *R. Spruce* 206 (K); Bragança-Viseu, *G. Prance & T. Pennington* 2074 (K). **Paraíba**: Santa Rita, *M.F. Agra & G. Gois* 647 (K). **Pernambuco**: *B.J. Pickel* 3622 (NY); Triunfo, *F.V. Silva & A.M. Miranda* 51 (HUEFS). **Piauí**: *G. Gardner* 2453 (K). **Rio de Janeiro**: *D. Sucre* 3965 (RB); Ilhas Cagarras, *M.G. Bovini et al.* 3635 (FHO, RB). **Rio Grande do Norte**: *F. Colla* 23 (UFRN). **Sergipe**: *M.R. França* 8 (ASE). **Tocantins**: Pedro Afonso, *K.G. Kissmann* (SP, K).

**FRENCH GUIANA.** Monts d’Arawa, *J.-J. de Granville et al.* 15048 (CAY, K).

**BOLIVIA. Santa Cruz**: Ángel Sandoval, Santo Corazón, *A. Fuentes & C. Cabrera* 1903 (USZ); Chiquitos, Santiago de Chiquitos, *J.R.I. Wood & D. Soto* 27327 (K, LPB, USZ); Germán Busch, Santa Ana–Carmen Rivero Torrez, *J.R.I. Wood et al.* 27893 (K, LPB, USZ); Ñuflo de Chávez, south of Concepción, *J.R.I. Wood et al.* 26205 (K, LPB, UB, USZ); Velasco, San Ignacio, *J.R.I. Wood & B. Williams* 27841 (K, LPB, USZ).

#### Note.

*Ipomoea bahiensis* has unique sepals. These are fleshy, very pale, spotted near the base and with prominent green tips, these often with a distinct tooth-like appendage. The exact structure appears to be rather variable and difficult to describe accurately even with the aid of photographs showing details. The compact, shortly pedunculate cymes are also distinctive.

### 380. Ipomoea squamosa

Taxon classificationPlantaeSolanalesConvolvulaceae

Choisy in A.P. de Candolle, Prodr. 9: 376. 1845. (Choisy 1845: 376)

Ipomoea morelii Duchass. & Walp., Linnaea 23: 752. 1850. (Duchassaing and Walpers 1850–51: 752). Type. PANAMA. *Duchassaing*s.n. (lectotype P04066969, designated here).
Ipomoea squamosa var. petiolaris Meisn. in Martius et al., Fl. Brasil. 7: 269. 1869. (Meisner 1869: 269). Type. BRAZIL. Bahia, Camamú, *Martius* 76 (holotype M0184961).
Convolvulus mattogrossensis Kuntze, Rev. Gen. 3(2): 214. 1898. (Kuntze 1898: 214). Type. BRAZIL. Mato Grosso, Cáceres, Villa Maria, *O. Kuntze*s.n. (isotype NY00318923).
Ipomoea mattogrossensis (Kuntze) K. Schum., Just’s Bot, Jahresber. 26: 383. 1900. (Schumann 1900: 383).
Ipomoea trinitensis Urban, Sym. Antill. 3(2): 346. 1902. (Urban1902–3: 346). Type. TRINIDAD. Mount Pleasant, *Finlay*s.n. (presumed holotype TRIN2945).
Ipomoea callida House, Muhlenbergia, 3: 42. 1907. (House 1907b: 42). Type. HONDURAS. Puerto Sierra, *P. Wilson* 534 (holotype NY00319074).
Ipomoea wilsonii House, Muhlenbergia, 3: 42. 1907. (House 1907b: 42). Type. HONDURAS. Puerto Sierra, *P. Wilson* 530 (holotype NY00547076).
Ipomoea squamosa var. villosa Ooststr., Rec. Trav. Bot. Neerl. 30: 211. 1933. (Ooststroom 1933: 211). Type. PERU. Huánuco, A. Weberbauer 3635 (holotype B†).
Ipomoea vestalii Standl., Contrib. Arnold Arbor. 5: 130. 1933. (Standley 1933: 130). Type. PANAMA. Barro Colorado Island, Shattuck 785 (holotype F0054904).

#### Type.

BRAZIL. Para, *Martius* 76 (lectotype M0184961, designated here).

#### Description.

Twining perennial herb or small liana, stems glabrous to thinly pubescent. Leaves petiolate, ovate, shortly acuminate, usually cordate with rounded to obtuse auricles, sometimes sagittate with acute auricles, glabrous except on the veins to subtomentose (var. villosa) on both surfaces, abaxially paler, prominently veined; petioles 3–6 (–12) cm, usually pubescent. Inflorescence of many-flowered pedunculate axillary cymes, the cymes often dense with shortly pedicellate, undeveloped flowers on the lateral branches; peduncles 4–12 cm, straight, usually pubescent; bracteoles 2–3 mm, ovate, caducous; secondary peduncles 4–16 mm; pedicels 4–15 mm, noticeably more slender than peduncles, glabrous; sepals unequal, glabrous, scarious-margined, accrescent in fruit, at anthesis outer 4–6 × 3–5 mm, obovate to suborbicular, obtuse, inner 7–10 × 5–8 mm, obovate to broadly elliptic, rounded, often nearly completely scarious; corolla 5.5–6.5 cm long, funnel-shaped, pink with dark centre, glabrous, limb 4.5 cm diam., undulate. Capsules 10–12 × 10–12 mm, broadly ovoid to subglobose, rostrate, glabrous; seeds woolly with long hairs.

#### Illustration.

**Figure 184.**
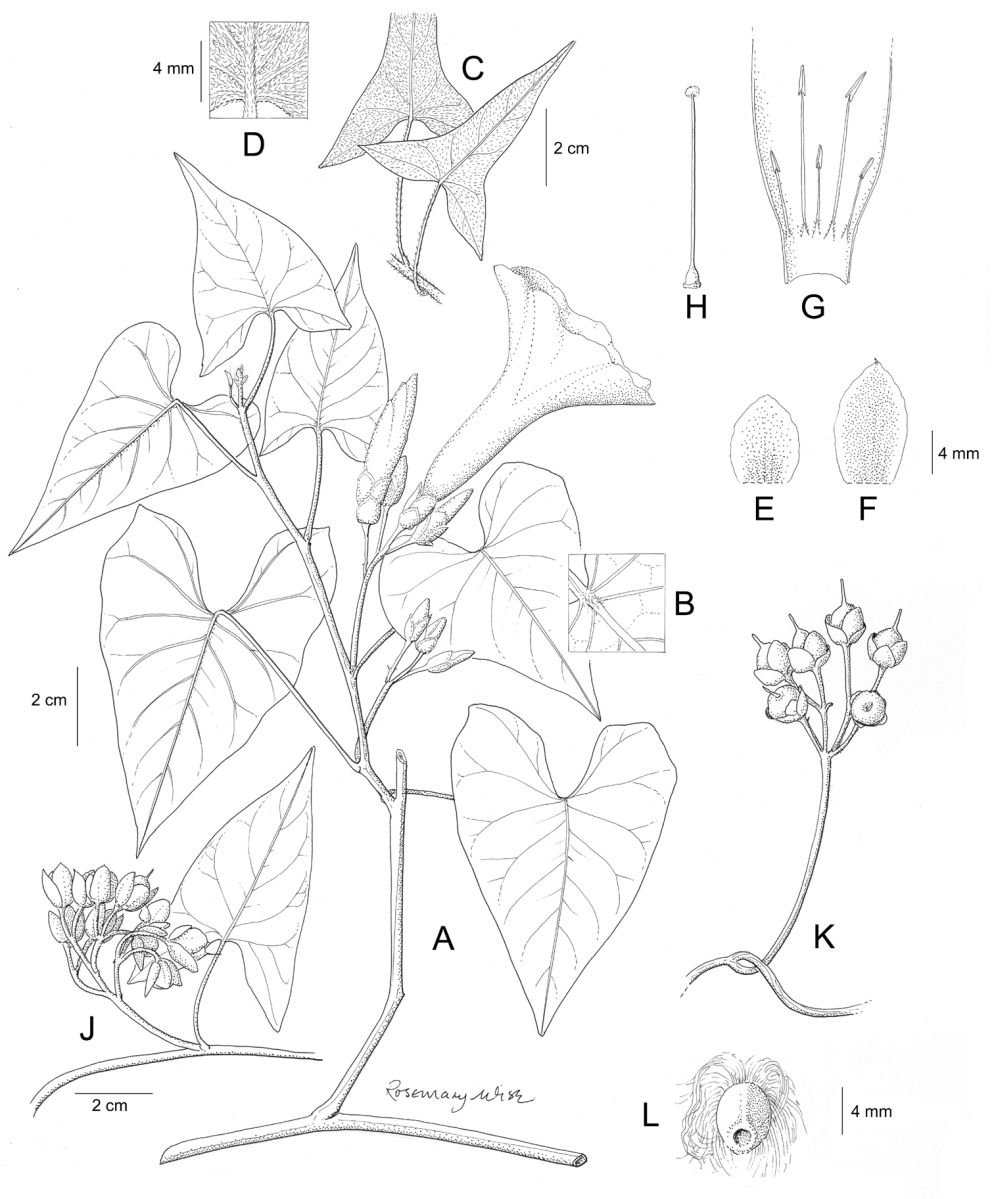
*Ipomoea squamosa*. **A** habit **B** abaxial leaf surface **C** leaf (var. villosa) **D** abaxial leaf surface (var. villosa) **E** outer sepal **F** inner sepal **G** corolla opened out to show stamens **H** ovary and style **J** fruiting inflorescence **K** capsules **L** seed. Drawn by Rosemary Wise **A, B, E–H, L** from *Hampshire & C. Whitefoord* 670; **C, D** from *Molina* 20647; **J***Proctor* 38825; **K** from *Proctor* 38839.

Figure 184; Austin (1998: 402).

#### Distribution.

Widely distributed in the neotropics and characteristic of moist lowland forest from southern Mexico south to Bolivia and Brazil at around 16°S.

**BRAZIL. Amapá**: *D.F. Austin et al.* 6964 (NY). **Amazonas**: *P. Acevedo-Rodríquez et al.* 81659 (NY). **Bahia**: *M.M. Arbo et al.* 7175 (CTES, NY). **Maranhão**: *G. Prance & Silva* 58577 (NY, S). **Mato Grosso**: *G. Prance et al.* 26075 (NY). **Minas Gerais**: Ituiutaba, *A. Macedo* 773 (BM). **Pará**: *C. Ferreira et al.* 1339 (NY, MO). **Roraima**: *G. Prance et al.* 9242 (NY, S). **Tocantins**: *G. Prance & Silva* 58462 (NY).

**FRENCH GUIANA.** Kanuku Mountains, *M.J. Jansen-Jacobs et al.* 352 (P); Rapunini, *M.J. Jansen-Jacobs et al.* 3772 (P).

**GUYANA.***A.C. Smith* 2464 (NY, P, S); *A.S. Hitchcock* 17584 (NY, S).

**BOLIVIA. Beni**: Cercado, Trinidad airport, *M. Atahuachi et al.* 1371, (BOLV). **Cochabamba**: P.N. Carrasco, Yanamayo, *M. Zarate et al.* 6417 (BOLV, USZ). **La Paz**: Iturralde, camino a Ixiamas, *L. Vargas et al.* 1327 (LPB, MO); Larecaja, Mapiri, *O. Buchtien* 1963 (US); 43 km from Guanay towards Mapiri, *S.G. Beck* 29480 (LPB, K) – var. villosa; Sud Yungas, Río Bopi, *C. White* 625 (NY). **Pando**: Suárez, NW of Cobija, *M. Mendoza & Rivadeneira* 2598 p.p. (US, K). **Santa Cruz**: Ichilo, c. 1 km W of San Carlos *J.R.I. Wood et al.* 28293 (K, LPB, USZ); Velasco, 5–7 km S of Río Iténez and 15 km SE of Flor de Oro, *M. Toledo* 87 (NY, USZ); PNNKM; camino entre Los Fierros and Aserradero Moira, *M. Saldias et al.* 2907 (ARIZ, BOLV, MO, USZ).

**PERU. Cusco**: La Convención, Kiteni, *W. Galiano et al.* 6691 (MO, OXF). **Loreto**: Aguaitia, *F. Woytkowski* 34456 (F, S, USM); ibid., *T. Croat* 20842 (MO); Río Ucayali, *H. Tuomisto & K. Ruokolainen* 52 (USM). **Pasco**: Oxapampa, Panjil, *D.N. Smith & R. Foster* 2403 (MO, OXF). **San Martín**: Río Huallaga, *G. Klug* 4356 (BM, K, S); *R. Ferreyra* 7755 (USM).

**ECUADOR. Napo**: Est. Sacha, *C.E. & M. Cerón* 4595 (QCA). **Orellana**: Canton Joya de las Sachas, *C. Montalvo & P. Paredes* 483 (Q). **Pastaza**: Arajuno, *E. Freire et al.* 3463 (MO).

**COLOMBIA. Amazonas**: Río Putumayo con Río Igaraparana, *R.E. Schultes* 3991 (COL, K). **Bolívar**: Gambote, *Dugand* 3350 (COL). **Cesar**: Poponte, *C. Allen* 895 (MO). **Chocó**: *C. Feddema* 1909 (S); Baudó, *Fuchs & Zanella* 22278 (COL, K, MO, S). **Córdoba**: Monteria-Lorica, *Franco* 2167 (COL). **Guaviare**: San José de Guaviare, *J. Cuatrecasas* 7660 (COL). **Meta**: Río Guejar, Los Micos, *J.M. Idrobo* 1229 (COL).

**VENEZUELA. Amazonas**: Río Negro, *B. Stergios & G. Aymard* 7690 (MO). **Apure**: Muñoz, *G. Aymard et al.* 5685 (MO). **Aragua**: Tovar, *A. Fendler* 939 (K). **Bolívar**: *R. Liesner & B. Holst* 20132 (MO). **Miranda**: *K.R. Robertson & D.F. Austin* 215 (MO). **Sucre**: *J. Steyermark et al.* 121787 (MO).

**PANAMA.** Gamboa, *H. Pittier* 2601 (BM, US); Bocas del Toro, *R.J. Hampshire & C. Whitefoord* 670 (BM); Chagres, *A. Fendler* 242 (K).

**COSTA RICA.** El General, *A.F. Skutch* 4121 (K, S); Alajuela, Upala, *M. Chavarria & N. Zamora* 606 (K, MO); Puntarenas, Golfito, *M. Chavarria & N. Zamora* 680 (K, MO); Heredia, Cuenca del Sarapiquí, *B. Hammel* 20854 (F).

**NICARAGUA.** Atlántico Sur, El Recreo, *D. Soza et al.* 451 (MO); ibid., El Rama-Pearl Lagoon, *W. D. Stevens* 29213 (MO).

**HONDURAS.** Guamil, *P.R. House* 1822 (BM); Olancho, Las Marias-La Colonia, *S. Blackmore & G.L. Heath* 1650 (BM); Puerto Lempira, *G.R. Proctor* 38825 (BM); La Mosquitia, Mocorón, *C. Nelson & E. Vargas* 5055 (MO); Roatan Island, *A. Molina* 20647 (NY) – var. villosa.

**EL SALVADOR.** Cabañas, Illobasco, *G. Davidse et al.* 37099; Lago de Ninfas, Juayua, *G. Davidse et al.* 37458 (BM, MO); Sierra Apaneca, *A. Molina & E. Montalvo* 21789 (BM, F).

**BELIZE.** Temash River, *W.A. Schipp* 898 (BM, K, S); Stann Creek, *D.R. Hunt* 384 (BM).

**GUATEMALA.** Izabal, *J.A. Steyermark* 42036 (F).

**MEXICO. Chiapas**: *E.W. Nelson* 3499 (US). **Guerrero**: *M.T. Germán et al.* 257 (MO). Tampico, *E. Palmer* 509 (K). **Veracruz**: *R.E. Gereau et al.* 2188 (MO).

**DOMINICAN REPUBLIC.** Santo Domingo city, *E.L. Ekman* H11170 (S). Apparently the only record fide Liogier (1994).

**TRINIDAD.***W.E. Broadway* 7824 (NY).

#### Notes.

The unequal scarious-margined sepals distinguish this species from all similar species except *Ipomoea cryptica* with which it has been confused so not all collections named as *I. squamosa* in different herbaria have been accepted above. The two species are extraordinarily similar although not closely related. In Bolivia, the leaves of *Ipomoea squamosa* are always with a few hairs at least on the veins beneath, the corolla is slightly larger (5.5–6.5 cm in length) and the outer sepals are at least half the length of the inner sepals. The leaves of *Ipomoea squamosa* are commonly sagittate, which seems never to be the case with *I. cryptica*.

Although most specimens of *Ipomoea squamosa* are at most thinly pubescent, the occasional specimen with subtomentose leaves occurs. These can be recognised as var. villosa Ooststr.

### 381. Ipomoea anisomeres

Taxon classificationPlantaeSolanalesConvolvulaceae

B.L. Rob. & Bartlett, Proc. Amer. Acad. Arts 43: 57. 1907. (Robinson and Bartlett 1907: 57)

Ipomoea anisomeres var. sagittiformis L.O. Williams, Fieldiana, Bot. 32: 185. 1970. (Williams 1970a: 185). Type. GUATEMALA. Izabal, *J. Steyermark* 38485 (holotype F0054821).

#### Type.

GUATEMALA. *C.C. Deam* 318 (lectotype GH00054484).

#### Description.

Entirely glabrous, twining perennial or liana; stems often granulose. Leaves petiolate, 3–8 × 1.5–6 cm, oblong-ovate to ovate, acute, base cordate, the auricles acute or rounded, abaxially paler; petioles 2–6.5 cm. Inflorescence of rather dense axillary pedunculate cymes; peduncles 5–10 cm; bracteoles ovate, c. 2 mm, caducous; pedicels short, 0.5–1.7 cm; sepals unequal, outer 1–3 × 2–3 mm, suborbicular to elliptic, the margin scarious, inner 7–8 × 3–4 mm, oblong-elliptic, rounded; corolla 5–6 cm long, funnel shaped, white with a purple tube, glabrous, limb c. 5 cm diam., the midpetaline bands terminating in small teeth. Capsules ovoid, 8–9 × 6–7 mm, glabrous, rostrate, the persistent style 4–5 mm long; seeds 7 × 4 mm, densely white-pubescent.

#### Distribution.

Lowland forests in Central America south to northern Peru.

**PERU. San Martín**: near Juanjui, *A. Gentry et al.* 37646 (MO, USM).

**COLOMBIA. Córdoba**: Montería, *B. Anderson* 1835 (K). **Magdalena**: Naranjo, *E. André* 371 (K).

**VENEZUELA.** Fide Hokche et al. (2008).

**PANAMA.***H. Pittier* 2704 (S).

**COSTA RICA.** Guanacaste, NW of Paloverde*N. Garwood et al.* 553 (BM).

**NICARAGUA.** Chontales, Puente Monato, *W.D. Stevens* 19059 (BM, MO); ibid., Cuapa, *W.D. Stevens* 6065 (BM, MO).

**HONDURAS.** Copán Ruins–Santa Rita, *A. Molina* 24693 (F); Santa Bárbara, Lago de Yojoa, *S. Blackmore & M. Chorley* 3712 (MO).

**BELIZE.** Orange Walk, Tower Hill, *A.H. Gentry* 8517 (FTG, MO).

**GUATEMALA.** Petén, P.N. Tikal, *R. Tun Ortíz* 693 (BM, MO); *Friedrichsthal* s.n. (K).

**MEXICO. Campeche**: Champotón, *E. & H. de Cabrera* 15203 (BM, IEB, MEXU, MO). **Chiapas**: Ocosingo, *E.M. Martínez* 17829 (MO). **Oaxaca**: Tuxtepec, *R.E. Gereau et al.* 2226 (MEXU). **Quintana Roo**: fide Austin et al. (2012). **Tabasco**: Macuspana, *M. A. Magaña & A. Guadarrama* 2357 (IEB). **Tamaulipas**: Tampico, *E. Palmer* 248 (BM, US). **Veracruz**: *C.R. Orcutt* 2997 (BM, K, MO); Temporal-Pánuco, *F. Chiang* 398 (F, MEXU, MO); Tempoal, *H. Puig* 4057 (MEXU, P). **Yucatán**: entrada a Chunchucmil, *M. Peña-Chocarro & Tun* 417 (BM, MO, UADY).

#### Notes.

Very close to *Ipomoea squamosa*, differing in being always glabrous with shorter outer sepals and a white corolla limb. The seeds are densely uniformly pubescent, rather than woolly.

We have been cautious in accepting South American records of this species, which may have been confused with *Ipomoea cryptica* as well as with *I. squamosa*.

### 382. Ipomoea acanthocarpa

Taxon classificationPlantaeSolanalesConvolvulaceae

(Choisy) Aschers. & Schweinf., Beitr. Fl. Aethiop. 277. 1867. (Ascherson and Schweinfurth 1867: 277)

Calonyction acanthocarpum Choisy in A.P. de Candolle, Prodr. 9: 346. 1845. (Choisy 1845: 346). Type. SUDAN. Kordofan, *T. Kotschy* 269 (isotype K000097122).
Ipomoea piurensis O’Donell, Lilloa 26: 382. 1953. (O’Donell 1953a: 382). Type. PERU. *O. Haught* 142 (holotype US00111444).
Ipomoea piurensis forma rosea O’Donell, Lilloa 26: 383. 1953. (O’Donell 1953a: 383). Type. BRAZIL. Pará, Rio Itacaiuna, Froes & Black s.n. (holotype LIL001283).

#### Type.

Based on *Calonyction acanthocarpum* Choisy

#### Description.

Glabrous twining herb. Leaves petiolate, 2–11 × 1.5–8 cm, ovate-deltoid, shortly and often abruptly acuminate or acute, cordate, auricles rounded to acute, often with a distinct tooth and sometimes shallowly bilobed, abaxially with prominent venation; petioles 1–8 cm. Inflorescence of few-flowered, somewhat congested, pedunculate cymes; peduncles 1–6 cm, often stout and somewhat swollen upwards, sometimes warty; bracteoles 2–3 mm, scale-like, caducous; pedicels 2–5 mm, sometimes warty; sepals slightly unequal, 5–10 × 3.5–7 mm, the margins white, outer ovate, acute to mucronate, usually conspicuously warty but otherwise glabrous, inner obtuse and mucronate, smooth, slightly larger; corolla 2–3 cm long, funnel-shaped, pink or white, glabrous, limb c. 2.5 cm diam., the midpetaline bands terminating in mucros. Capsules 9–10 mm, subglobose, rostrate with prominent persistent style, glabrous; seeds 5.5 mm long, grey, long-pilose.

#### Illustration.

**Figure 185.**
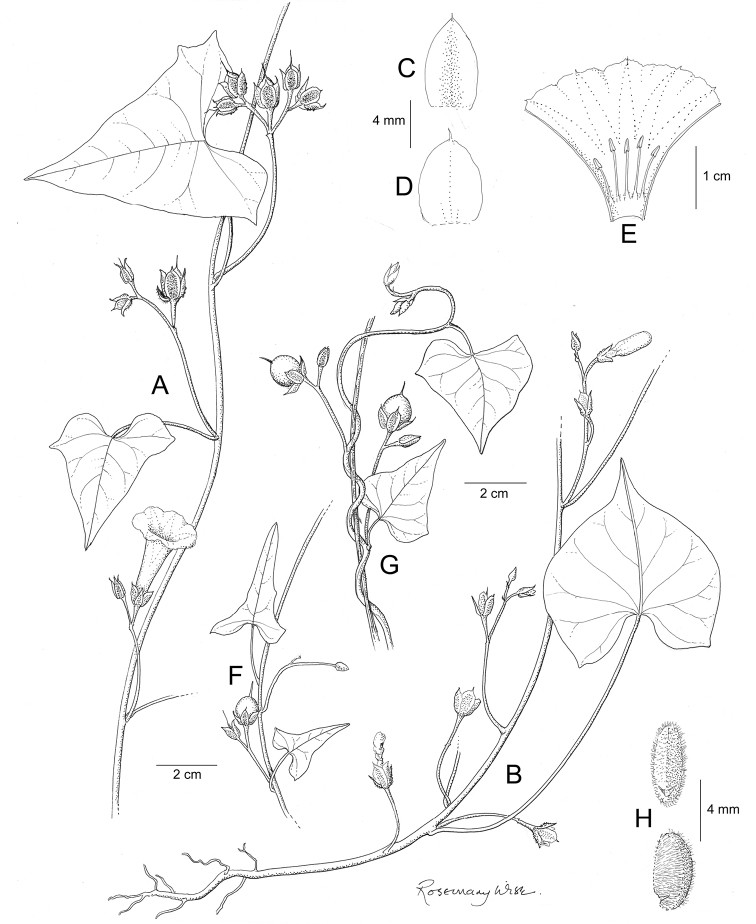
*Ipomoea acanthocarpa*. **A** habit **B** habit **C** outer sepal **D** inner sepal **E** corolla opened out to show stamens **F** habit with capsules **G** habit with capsules **H** seeds. Drawn by Rosemary Wise **A** from *Montes* 1362; **B** from *Cerón* 18749; **C–E** from *Wash* 143; **F** from *Smith* 2313; **G, H** from *Wurdack & Monachino* 39830.

Austin (1998: 402) as *Ipomoea piurensis*; Figures 11H, 167D, 185.

#### Distribution.

In South America this species extends in an arc from Bolivia through Peru to southern Colombia and then eastwards to Guyana and north east Brazil where it is especially common. There is an isolated record from Costa Rica. In Africa it is widely distributed across the Sahel region from Senegal and Sierra Leone east to Sudan and Ethiopia. In India it has recently been discovered in Gujerat (Kattee et al. 2019), confirming its essentially Sahara-Sindian distribution in the Old World.

**BRAZIL. Bahia**: Feira de Santana, *L.P. de Queiroz* 1721 (HUEFS, RB); *Aona & Costa* 3247 (HUEFS). **Ceará**: Caucaia, *E.B. Souza* 257 (EAC). **Paraíba**: *J. Falçao et al.* 1116 (RB); *R. Simão-Bianchini* 1752 (ASE). **Pernambuco**: Afrânio-Aboclo, *E.P. Heringer* 216 (RB); Petrolina, *C.T.V. Diaz* 172 (RB); Tapera, *B.J. Pickel* 3649 (NY); Archipeligo de Fernando do Noronho. *M. Miranda et al.* 946 (PEUFR), 1019 (PEUFR). **Rio Grande do Norte**: Serra Negra do Norte, *R.T. Queiroz* 267, 406 (UFRN). **Sergipe**: Canindé de São Francisco, *R. A. Silva et al.* 261 (PEUFR, RB). **Tocantins**: Porto Nacional, *E.R. Santos* 2 (HUEFS). Maranhão fide Flora do Brasil (2020).

**FRENCH GUIANA.** Mana, *G. Léotard* 1319 (CAY).

**GUYANA.** Rupununi, Charwair Creek, *A.C. Smith* 2313 (MO, S); ibid., Makawau Creek, *T. Henkel et al.* 3373 (K, US). .

**BOLIVIA. Beni**: Cercado, Ibiato, *M.T. Martinez & M. Adler* 9 (K, LPB, USZ). **Pando**: *E. de la Sota* 977 (LIL).

**PERU. Lambayeque**: Garraspiña, *C. Abad & J. Laos* s.n. (USM); between Jayanca and Motupe, *R. Ferreyra* 9054 (USM). **Piura**: *L’Emperaire* 5282 (P).

**ECUADOR. Guayas**: *E. Asplund* 16682 (K, NY, S, US); Isla Puná, *J.E. Madsen* 63158 (QCA, QCNE). **Loja**: San Pedro de Vilcabamba, *A. Balcazar* 182 (LOJA). **Manibí**: Puerto López, P.N. Machalilla, *C.E. Cerón* 18749 (ARIZ, MO).

**COLOMBIA. Nariño**: Pasto, *H. Martínez* 29 (COL).

**VENEZUELA. Amazonas**: fide Austin (1982b). **Anzoátegui**: Sucre, *A. Castillo & A de Franca* 2641 (MO); **Bolívar**: Cerro Borja, *J.J. Wurdack & J.V. Monachino* 39830 (MO, NY). **Guárico**: Est. Biol. de Los Llanos, *R.A. Montes* 1362 (MO).

**COSTA RICA.** Guanacaste, Bagaces, *U. Chavarría* 1344 (BM), ibid., 1349 (MO, BM).

#### Notes.

Molecular studies (Muñoz-Rodríguez et al. 2019) indicate that *Ipomoea acanthocarpa* is of American origin and has colonised Africa by long-distance dispersal. The name “*acanthocarpa*” presumably refers to the spine-like rostrate apex of the capsule.

This species is sometimes confused with *Ipomoea dumetorum* because of the lateral tooth which is often present near the base of the leaf and because of the white-margined sepals which are characteristic of both species. However, *I. acanthocarpa* is a lowland species, its sepals lack the dark spots of *I. dumetorum* and the inflorescence is rather compact with very short pedicels. The seeds are long pilose, not minutely tomentellous.

### 383. Ipomoea longeramosa

Taxon classificationPlantaeSolanalesConvolvulaceae

Choisy in A.P. de Candolle, Prodr. 9: 384. 1845. (Choisy 1845: 384)

Ipomoea geranioides Meisn. in Martius et al., Fl. Brasil. 7: 276. 1869. (Meisner 1869: 276). Type. BRAZIL. Mato Grosso, Cuiabá, L. Riedel 945, (lectotype NY00319188, designated by Wood and Scotland 2017c: 6), isolectotype LE).
Ipomoea punctata C. Wright in Sauvalle, Anales Acad. Cien. Med. Habana 7: 44–45. 1870. (Sauvalle 1870: 44), nom. illeg., non Ipomoea punctata Pers (1805). Type. CUBA. [Sancti Spiritus], en las sabanas del potrero Manatí, Trinidad, *C. Wright 3645* [1632] (lectotype K000612812, designated by Wood and Scotland 2017c: 6, isolectotypes GH, HAC).
Ipomoea flavopurpurea Urban, Symb. Antill. 3 (2): 345. 1902. (Urban 1902–3: 345). Type. Based on I. punctata C. Wright
Ipomoea dajabonensis Alain, Anales. Acad. Cien. Rep. Dom. 3: 68. 1978. (Liogier 1978: 68). Type. DOMINICAN REPUBLIC. En manigua a la orilla de la carretera de Dajabón, *A & P. Liogier* 27239 (isotype B10 0242101).

#### Type.

BRAZIL. Minas Gerais, Morro do Lobo, *Martius* s.n. (holotype M0185026, isotype M0185027).

#### Description.

Slender herb climbing to 70 cm, possibly annual, stems thinly pilose. Leaves petiolate, 3–4 cm long, 5-lobed to near base, base broadly cuneate, segments oblong to obovate, narrowed at base, minutely retuse and mucronulate, glabrous, punctate abaxially at least when young; petioles 3–4.5 cm, pilose. Inflorescence of solitary or paired, axillary flowers; peduncles 2–5 cm, glabrous; bracteoles 2 mm, filiform; pedicels notably thicker than peduncle, 10–15 mm; sepals nearly equal or inner slightly longer, 6–11 × 2–3 mm, lanceolate or oblong, finely acuminate, mucronate, glabrous or with a few spreading trichomes and spinules near base, margin narrowly scarious; corolla 2–3 cm long, cream with lavender centre, funnel-shaped to subcampanulate, glabrous; limb 2.5 cm, obscurely lobed, midpetaline bands ending in a tooth. Capsules 7–8 mm, glabrous, subglobose, slender style shortly persistent; seeds densely shortly pilose.

#### Illustration.

**Figure 186.**
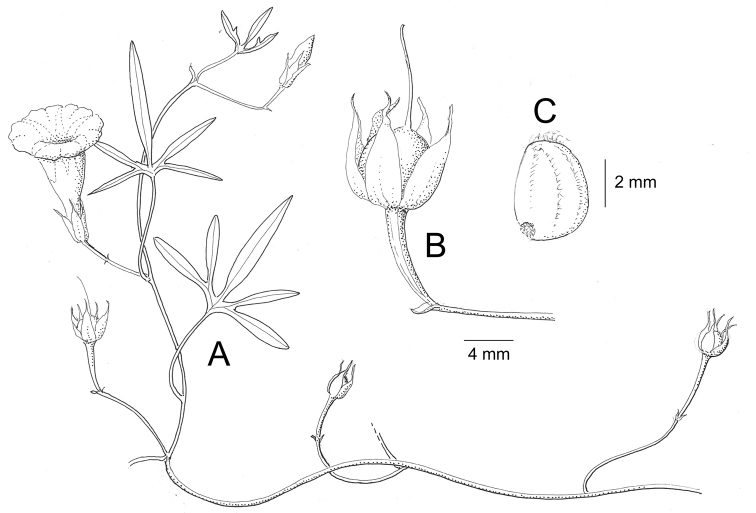
*Ipomoea longeramosa***A** habit **B** fruit **C** seed. Drawn by Rosemary Wise from *B. Pickersgill et al*. RU72-400.

Liogier (1994: 113) as *Ipomoea dajabonensis*; Figures 141C, 186.

#### Distribution.

Relatively frequent in the Caatinga region of NE Brazil; elsewhere rare and very scattered in occurrence both in other parts of Brazil, as well as in Guyana, Bolivia, Venezuela, Cuba and the Dominican Republic, and known from single records in four of these countries.

**BRAZIL. Acre**: Rio Branco, entre Surumu & Miriam, *E. Ule* 8286 (K, S). **Alagoas**: *R.P. Lyra-Lemos* 4830 (IPA). **Amazonas**: Rio Branco, *J.G. Kuhlmann* 720 (RB). **Bahia**: *D.V. Braga et al.* (IPA73962): Salvador, *L.R. Noblick* 1476 (HUEFS). **Ceará**: Serra da Ema, *A. Löfgren* 524 (S); Serra Apody, *A. Löfgren* 740 (S); *J. Santino de Assis* 379 (RB); Serra das Almas, *F.S. Araujo* 1522 (HUEFS). **Mato Grosso**: Type of *Ipomoea geranioides*. **Mato Grosso do Sul**: Mun. Corumbá, Lagoa do Jocadigo, *A. Pott et al.* 4742 (CPAP); Faz. Vale de Esperanza, *A. Pott et al.* 4838 (CPAP). **Minas Gerais**: Type of *Ipomoea longeramosa*. **Paraíba**: Regiones secas, *Coêlho de Moraes* 2108 (K, MO); São José dos Cadeiros, *R.M.T. Costa & M.F.M. de Brito* 136 (JPB); Santa Teresinha, *B. Laine* 16 (IPA). **Pernambuco**: Floresta, *A.C.B. Lins e Silva* 217 (PEUFR); P.N. do Catimbau, *G.C. Delgado Junior* 695 (RB). **Rio Grande do Norte**: 5 km from Currais Novas, *B. Pickersgill et al*. RU72-400 (K); *J.L. Costa-Lima* 220 (UFRN). **Sergipe**: *A.M. Miranda and M. Grillo* 4401 (UFPRE); Canindé de São Francisco, *R. Simão-Bianchini* 1743 (ASE).

**GUYANA.***R. Schomburgk* (K).

**BOLIVIA. Santa Cruz**: Velasco, 3 km N of San Rafael, *J.R.I. Wood et al.* 24770 (K, LPB, USZ).

**VENEZUELA.** Anzoategui: *E. Holt & W. Gehringer* 156 (VEN) fide Austin (1982b: 168)

**CUBA. [Granma**]: Aeropuerto Río Cauto, *Catasus* 2/95 (HAC40737). **Las Tunas**: Victoria, *J. Acuña & Montenegro* (HAJB17153). **Sancti Spiritus**: Trinidad, carrera de Casilda a Playa Aneón, *J. Bisse et al.* (HAJB34707). **Villa Clara**: Santa Clara, Casilda, *E.L. Ekman* 18876 (S).

**DOMINICAN REPULIC.** Type of *Ipomoea dajabonensis*.

#### Note.

An apparently easily overlooked annual herb distinguished by the palmately-lobed, abaxially punctate leaves, the yellowish corolla with a dark centre and the acuminate, mucronate sepals.

### 384. Ipomoea kraholandica

Taxon classificationPlantaeSolanalesConvolvulaceae

J.R.I. Wood & Scotland, Phytokeys 88: 21. 2017. (Wood et al. 2017d: 21)

#### Type.

BRAZIL. Tocantins, Mun. Itacajá, Reserva Indígena Krahó, Aldea Pedra Blanca, 9 May 2000, *A.A. Santos, A. Reatto, E. de Souza Martins, L. Rovênia, M. de Andrade & L. Moreira Rodrigues* 719 (CEN).

#### Description.

Slender twining herb of unknown height; stems glabrous. Leaves petiolate, 2–3.5 × 1–3 cm, 3-lobed with the central lobe lanceolate, entire, the laterals 2–3-lobed, the first second lobe bent forwards and the third lobe bent backwards, base truncate, apex finely acuminate; petioles 0.7–2 cm. Inflorescence of solitary, axillary flowers; peduncles very short, 0–3 mm, thinly pubescent; bracteoles, 1–3 mm, relatively persistent, thinly ciliate; pedicels 6–12 mm, thickened upwards, pubescent; sepals subequal, 11–12 × 1.5–2.5 mm, narrowly lanceolate, finely acuminate, mucronate, outer pubescent, inner pubescent with broad glabrous margins; corolla c. 2.5 cm long, funnel-shaped, pink, glabrous, midpetaline bands terminating in a prominent tooth, c. 2.5 cm diam. Capsules 10 × 5 mm, ovoid, glabrous; seeds 5 × 2 mm, dark grey, minutely tomentellous.

#### Illustration.

Figure 187.

**Figure 187.**
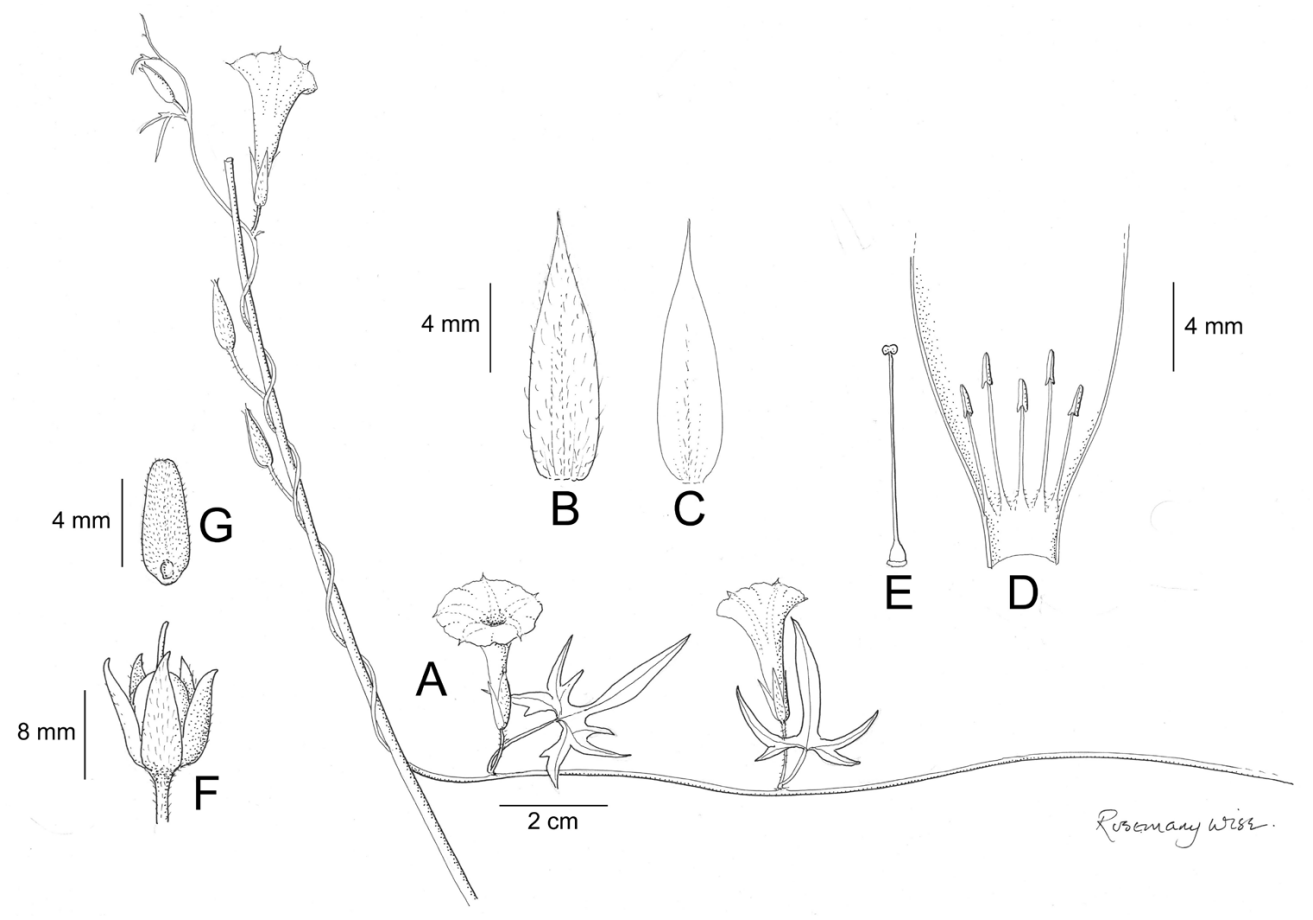
*Ipomoea kraholandica*. **A** habit **B** outer sepal **C** inner sepal **D** corolla opened out to show stamens **E** ovary and style **F** capsule and calyx **G** seed. Drawn by Rosemary Wise from *Santos et al.* 719.

#### Distribution.

Only known from the type. Locally abundant in disturbed ground on sand. **BRAZIL. Tocantins**: the type.

#### Note.

Very distinct because of the unusual leaf shape, solitary flowers with suppressed peduncles and narrowly lanceolate, pubescent sepals.

### 385. Ipomoea leprieurii

Taxon classificationPlantaeSolanalesConvolvulaceae

D.F. Austin, Acta Amazonica 11(2): 291 1981. (Austin 1981: 291)

Merremia linearifolia Hallier f., Jahrb. Hamburg. Wiss. Anst. 16, Beiheft 3: 36. 1899. (Hallier 1899b: 36), non Ipomoea linearifolia Hook. (1847). Type. FRENCH GUIANA. *F.M.R. Leprieur*s.n. (isotype G00227883).

#### Type.

Based on *Merremia linearifolia* Hallier f.

#### Description.

Erect or decumbent perennial from an often somewhat tufted woody rootstock with several stems from base, stems glabrous or obscurely pubescent, sometimes rooting at the nodes. Leaves petiolate, 4–12 × 0.2–0.7 cm, linear or linear-lanceolate, finely acuminate, acute, apiculate, base cuneate to subrounded, glabrous; petioles 0.5–2.5 cm long, glabrous to sparsely pilose. Inflorescence of solitary (rarely paired) flowers from the uppermost leaf axils, or apparently terminal; peduncles 0.5–4 cm; bracteoles filiform, 2–5 mm; pedicels 7–10 mm; sepals subequal, 4–7 mm long, lanceolate, finely acuminate, glabrous, margins scarious; corolla 3–4 cm long, pink, funnel-shaped from pale tube 10–15 mm long, glabrous, limb c. 3 cm diam. unlobed but toothed at tips of midpetaline bands. Capsules 7–8 mm, glabrous, globose, usually 2-seeded; seeds 4.5 × 3 mm, minutely pubescent.

#### Distribution.

French Guiana and Amapá State in Brazil. On granite outcrops and inselbergs in savanna.

**BRAZIL. Amapá**: Cidade da Pedras, Vila Porto Grande, *D.F. Austin et al.* 7089 (FTG, MG, MO, NY RB); 2 km de acampamento, Montanha de Pedra. *D.F. Austin et al.* 7342 (FTG. MG, NY); Rio Araguari, *J.M. Pires et al.* 50968 (NY, FTG); 14 km SSE of Oiapogue, *D.C. Daly & J. Cardoso* 3805 (NY, MG, FTG); Rio Oiapoque, granite outcrop, *W.A. Egler* 47645 (MG, FTG).

**FRENCH GUIANA.** Inselberg Mont Chauve, *J.F. Villiers & C. Sarthou* 6095 (P); Fleuve Oyapock, *Oldeman* 2569 (P); Mont. des Mouragues, *C. Sarthou* 229 (FTG); Savanne de Virginie, Mataroni River, *S.A. Mori et al.* 25290 (ARIZ, NY); Roche Touatou, Bassin de l’Aoyapock, *J.J. Granville & G. Cremers* 12965 (CAY, K, OXF).

#### Note.

A very unusual species because of the finely acuminate, linear to linear-lanceolate leaves, subequal, lanceolate filiform sepals and glabrous corolla. The placement of this species is uncertain.

### 386. Ipomoea eriocalyx

Taxon classificationPlantaeSolanalesConvolvulaceae

(Mart. ex Choisy) Meisn. in Martius et al., Fl. Brasil. 7: 226. 1869. (Meisner 1869: 226)

Pharbitis eriocalyx Mart. ex Choisy in A.P. de Candolle, Prodr. 9: 342. 1845. (Choisy 1845: 342). Type. BRAZIL. Bahia, Soteropolin, *Martius* 2162 (M0184877, lectotype, designated here).
Batatas triloba Choisy, Mém. Soc. Phys. Genève 8(1): 49 [127]. 1838. (Choisy 1838: 49 [127]), non Ipomoea triloba L. (1753). Type. BRAZIL. Rio de Janeiro, P. Lund 770 (holotype G-DC 00135165),
Convolvulus hewittaceus Kuntze, Rev. Gen. 3: 213. (Kuntze 1898: 213). Type. BRAZIL. Mato Grosso, *O. Kuntze*s.n. (holotype B†, isotype NY0621768).
Jacquemontia hewittacea (Kuntze) K. Schum., Bot. Jahrsber. (Just) 26 (1): 383. 1900. (Schumann 1900: 383).
Ipomoea hewittacea (Kuntze) J.R.I. Wood & Scotland, Kew Bull. 70 (31): 38. 2015. (Wood et al. 2015: 38).
Ipomoea piresii O’Donell, Arq. Mus. Paranaense 9: 229. 1952. (O’Donell 1952: 229). Type. BRAZIL. Maranhão, *J.M. Pires* 1989 (holotype LIL001279, isotypes IAN, P, US

#### Type.

Based on *Pharbitis eriocalyx* Mart. ex Choisy

#### Description.

Perennial twining herb to 2 m, stems scabrous, pubescent or pilose, the hairs swollen at base. Leaves shortly petiolate, 2–8 × 1.5–5.5 cm, entire or 3-lobed, lanceolate to ovate, slightly constricted above base, apex acute to finely acuminate, mucronate, base cordate to sagittate with narrow sinus, auricles acute to rounded, sometimes shallowly bifurcate, both surfaces thinly pubescent to tomentose but pubescence denser on veins and margins, abaxially paler; petioles 1–7 cm, pubescent to densely pilose. Inflorescence of 1–4-flowered clusters at apex of long, axillary peduncles; peduncles 2.5–15 cm, pubescent to pilose; bracteoles 10–21 × 1–4 mm, linear-lanceolate to broadly lanceolate, acute to acuminate, pubescent, persistent; pedicels very short, 1–6 mm, thinly pilose; so bracteoles ±appressed to the calyx; sepals slightly unequal, pilose and ciliate, outer 2–3 mm longer than inner, 13–16 × 3–5 mm, lanceolate to ovate, finely acuminate, inner 10–11 × 2–3 mm, lanceolate, margins scarious; corolla 3.5–7 cm long, funnel-shaped, pink, pilose on midpetaline bands, limb 2.5–5 cm diam., undulate. Capsules globose, 7–8 × 7–8 mm, glabrous; seeds 4.5 × 3 mm, obovoid, minutely scabrous.

#### Illustration.

Figures 6E, 188.

**Figure 188.**
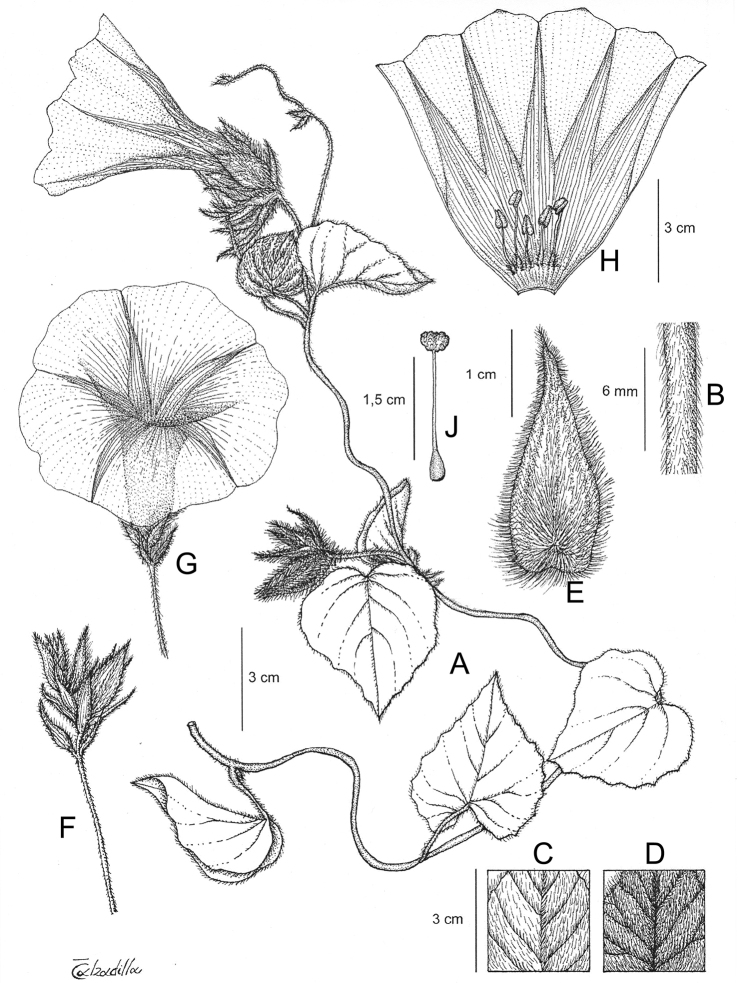
*Ipomoea eriocalyx*. **A** habit **B** stem **C** adaxial leaf surface **D** abaxial leaf surface **E** bracteole **F** inflorescence with bracteoles and sepals **G** flower **H** corolla opened out to show stamens **J** ovary and style. Drawn by Eliana Calzadilla **A, B, E, F, H–J** from *Pott* 4332; **C, D** from *Pott et al.* 2938; **G** from *Gasparini* s.n.

#### Distribution.

An uncommon species of swampy grassland at low altitudes principally in Brazil, but also found in Bolivia and Colombia, occurring in scattered populations around the edges of the Amazonian region.

**BRAZIL.***J.B. Pohl* 5208 (W). **Alagoas**: Mun. Satuba, *M.N. Rodrigues et al.* 1329 (SP). **Amazonas**: Humaitá, *A. Janssen* 351(RB). **Bahia**: *C. Gaudichaud* 10 (P); *C.E.F. von Glocker* 247 (NY); Salvador, *J.R.L. da Paz & M.J. Oliveira* 4 (HUEFS, SP); *G. Gardner* 893 (K, BM); Itacaré, *L.V. Vasconcelas et al.* 466 (HUEFS). **Maranhão**: Lorêto, Ilha de Balsa, *G. & L. Eiten* 4396 (K). **Mato Grosso**: Poconé, *Stapf et al. 414* (HUEFS); Mun. Luciara, *J. Pirani* 1260 (ARIZ, FTG). **Mato Grosso do Sul**: Faz. Nhumirim, Nhecolândia, *A. Pott et al*. 2938 (CPAP); ibid., *A. Pott* 4332 (MBM, CPAP). **Minas Gerais**: Pirapora, Rio São Francisco, *A. Krapovickas & C. Cristóbal* 42864 (CTES). **Pará**: *A. Ducke* 8416 (MG). **Piauí**: Priri, *A.S.F. Castro* 738 (EAC). **Rondônia**: Cerejeiras, *G. Martinelli* 14454 (RB). **Sergipe**: *A.C. Barreto* 80 (RB). **Tocantins**: Rio Araguaia, *N.T. Silva* 4847 (NY).

**BOLIVIA. Beni**: Cercado, Ibiato, *M.T. Martinez* 34 (K, PB, USZ). **Santa Cruz**: Ángel Sandoval, *J.R.I. Wood et al.* 24825 (K, LPB, UB, USZ); Velasco, El Refugio, *T. Killeen & R. Guillén* 6699 (MO); ibid., *J.R.I. Wood et al.* 26374 (K, LPB, UB, USZ); Santa Rosa de la Roca, *J.R.I. Wood et al.* 27814 (OXF, K, LPB, USZ).

**COLOMBIA. [Tolima**]: Chaparral, *Goudot* s.n. (K).

#### Note.

A very distinctive species because of the subcapitate inflorescence with persistent lanceolate to ovate bracteoles, densely pilose sepals, and globose capsule. However, it is extremely variable especially in indumentum and leaf shape so it is difficult to believe all specimens belong to the same species unless a range of specimens is examined. Leaves vary from ovate, cordate with rounded auricles to lanceolate sagittate with simple or bifurcate acute auricles. Margins may be entire or with one or more irregular large teeth. Indumentum varies from simply pubescent to densely tomentose. Consequently we can see no reason to maintain *Ipomoea hewittacea* as a separate species and have united it with *I. eriocalyx*, the type of which is very similar to many specimens identified as *I. hewittacea* or *I. piresii*.

### 387. Ipomoea deminuta

Taxon classificationPlantaeSolanalesConvolvulaceae

J.R.I. Wood & Scotland, Kew Bull. 72 (10): 9. 2017. (Wood and Scotland 2017b: 9)

#### Type.

BOLIVIA. Velasco, Flor de Oro, *E. Gutiérrez, R. Quevedo & F. Mamani* 1152 (holotype MO04639930).

#### Description.

Slender twining herb of unknown height; stems pubescent. Leaves petiolate, 1.5–2.7 × 0.4–1.2 cm, lanceolate-deltoid, obtuse to acute, mucronate, base cordate, auricles variable, rounded, acute, or rounded with a prominent tooth, adaxially tomentose, abaxially grey-tomentose; petioles 3–7 mm, densely pubescent. Inflorescence of very shortly pedunculate axillary flowers; peduncles 2–3 mm, densely pubescent; bracteoles 3–4 × 0.5–1 mm, filiform, tomentose, persistent, ±appressed to calyx; pedicels 0–1 mm; sepals subequal, 7–8 × 1–1.5 mm, lanceolate, acute, densely pubescent, inner slightly narrower with scarious glabrous margins; corolla 2–2.5 cm long, pale pink, funnel-shaped, glabrous, limb c. 1 cm diam. Capsules and seeds not seen.

#### Illustration.

Figure 189.

**Figure 189.**
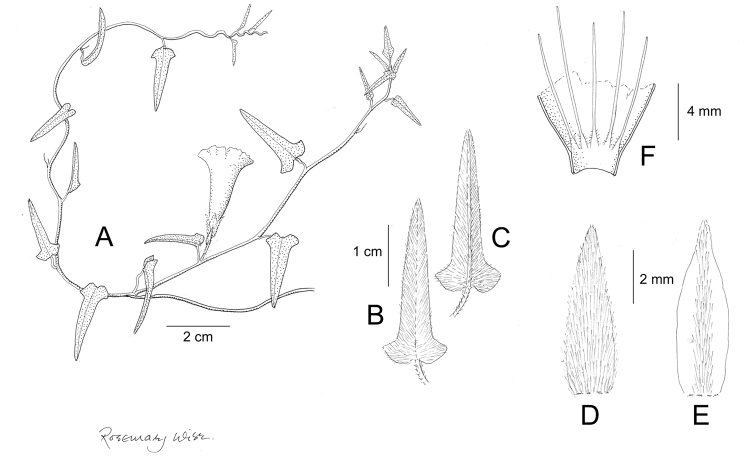
*Ipomoea deminuta*. **A** habit **B** adaxial leaf Surface **C** abaxial leaf Surface **D** outer sepal **E** inner sepal **F** corolla opened out to show insertion of filaments. Drawn by Rosemary Wise from *E. Gutiérrez et al.* 1152.

#### Distribution.

Endemic to the Noel Kempff Mercado National Park and only known from the type. It grows in seasonally flooded pampa.

**BOLIVIA. Santa Cruz**: type collection.

#### Note.

This very slender species is unlike any *Ipomoea* known to us. The small corolla, small tomentose lanceolate-deltoid leaves, solitary flowers, short peduncles, persistent bracteoles appressed to the calyx and suppressed pedicels all serve it separate it from all known species. We have not been able to sequence this species but it is probably related to *I. eriocalyx*.

### 388. Ipomoea imperati

Taxon classificationPlantaeSolanalesConvolvulaceae

(Vahl) Griseb., Cat. Pl. Cub. 203 (1866). (Grisebach 1866: 203)

Convolvulus imperati Vahl, Symb, Bot. 1: 17 (1790). Type. ITALY. Unnumbered illustration by Imperati cited as “Convolvulus marino” in Imperato, Hist. Nat. 671 (1672), lectotype, designated by La Valva and Sábato (1983: 114).
Convolvulus littoralis L., Syst. Nat., ed. 10, 924. 1759. Type. Icon in Plumier, Pl. Amer. 79, t. 90. F. 2, (1756), lectotype designated by Austin (1975b: 199).
Batatas littoralis (L.) Choisy, Mém. Soc. Phys., Genève 6: 46 [124]. 1838. (Choisy 1838: 46 [124]).
Ipomoea littoralis (L.) Boiss. Fl. Orient. 4: 112.1875 (Boissier 1875: 112), nom. illeg., non Ipomoea littoralis Blume 1826.
Convolvulus sinuatus Petagna, Inst. Bot. 2: 352. 1787. (Petagna 1787: 352), non Ipomoea sinuata Ortega (1798). Type. ITALY. Portici, Herb. Petagna (lectotype designated by La Valva and Sábato 1983).
Convolvulus stolonifer Cirillo, Pl. Rar. Neap. 1: 14. 1788. (Cirillo 1788: 14), nom. illeg. superfl. Type. Based on Convolvulus sinuatus Petagna.
Ipomoea stolonifera (Cirillo) J.F. Gmel., Syst. Nat., ed. 3, 2: 345. 1791. (Gmelin 1791: 345).
Convolvulus arenarius Vahl, Symb. Bot. 1: 18. 1790. (Vahl 1790: 18). Type. AZORES. No collector named. (whereabouts uncertain).
Ipomoea arenaria (Vahl) Roem. & Schult. Syst. Veg. 4: 247. 1819. (Roemer and Schultes 1819: 247).
Convolvulus acetosifolius Vahl, Ecl 1: 18. 1798. (Vahl 1798: 18). Type. Central America, (lectotype Plumier 1693: t. 105, designated here).
Ipomoea acetosifolia (Vahl) Roem. & Schult., Syst. Veg. 4: 246. 1819. (Roemer and Schultes 1819: 246).
Batatas acetosifolia (Vahl) Choisy, Mém. Soc. Phys. Genève 8(1): 46 [124]. 1838. (Choisy 1838: 46 [124]).
Ipomoea deppeana G. Don, Gen. Hist. 4: 276. 1838. (Don 1838: 276). Type. Based on Convolvulus sinuatus Petagna
Ipomoea carnosa R.Br., Prodr. 485.1810. (Brown, R 1810: 485). Type. AUSTRALIA. Carpentaria Island, *R. Brown* 2749 (holotype BM000630205).
Ipomoea acetosifolia var. longifolia Glaz. Bull. Soc. Bot. France 57, mém. 3e: 483. 1910. Glaziou 1910: 483). Type. BRAZIL. Rio de Janeiro, *A.F.M. Glaziou* 9977 (holotype P03866537).
Ipomoea denticulata auct., sensu Choisy (1845).

#### Type.

Based on *Convolvulus imperati* Vahl

#### Description.

Perennial herb; stems trailing, rooting at the nodes, glabrous, up to 5 m long. Leaves petiolate, slightly succulent, 1.5–3 × 0.8–2 cm, rather small and variable, linear, lanceolate or characteristically shortly oblong (± rectangular) or 3–5-lobed with the terminal larger than the laterals, apex obtuse or retuse, base truncate or very shallowly cordate, margin entire, undulate; petioles 0.5–4.5 cm. Flowers solitary (rarely 2–3), axillary, pedunculate; peduncles 0.5–2.5 cm; bracteoles 2 mm, lanceolate, acuminate, caducous; pedicels 8–15 mm, thickened upwards; sepals unequal, glabrous, oblong-oblanceolate, acute or obtuse, outer 7–12 mm, mucronate with mucro bent outwards, inner sepals 12–15 mm, pale and somewhat scarious; Corolla 3.5–4 cm long, funnel-shaped, white with a yellowish tube, glabrous, limb unlobed. Capsules subglobose, 10–12 mm, glabrous; seeds 7–8 × 4 mm, tomentose with longer hairs on margins.

#### Illustration.

Acevedo-Rodríguez (2005: 170); Figure 161C.

#### Distribution.

Pantropical on sand by the sea but rather scattered in occurrence. In the Americas on the Pacific coast from Ecuador and the Galapagos north to Mexico (Baja California and Sonora), thus avoiding the relatively cool Peruvian coast; on the Atlantic coast from Rio Grande do Sul north to Georgia in the United States and the Bahamas; also in the Caribbean but not recorded from most smaller islands.

**BRAZIL. Alagoas**: *S. Tsugaru et al.* B1465 (NY). **Bahia**: *J.S. Blanchet* 1419 (BM, P); *R.M. Harley et al.* 17139 (K, NY). **Espirito Santo**: *P.R. Bamps* 5049 (NY). **Maranhão**: *G. Gardner* 6072 (BM, K). **Pará**: *R. Spruce* 138 (K, P). **Paraíba**: *M.F. Agra* 1522 (K). **Paraná**: *G. Hatschbach* 14386 (K); *P. Dusen* 13607 (S). **Pernambuco**: *A. Cassio Sevilha et al.* 2486 (CEN). **Rio de Janeiro**: *B.M. J. Lutz* 1367 (K, NY, R); *G. Gardner* 5557 (BM); *Miers* 3692 (K). **Rio Grande do Norte**: *M. Martins* 370 (VIES). **Rio Grande do Sul**: *P.P.A. Ferreira* 219 (ICN). **Santa Catarina**: *R. Pozner* 163 (SI). **Sergipe**: Pirambu, *M. Ramos & E. Santos* 21 (ASE).

**FRENCH GUIANA.***P. Sagot* 806 (BM, K); *von Rohr* s.n. (BM, C); *C. Sastre* 1319 (P); *T. Deroin* 137 (P).

**SURINAM.** Fide Ooststroom (1932: 95).

**GUYANA.***S.A. Harris* EC25 (K).

**ECUADOR. Galapagos**: *H. Van der Werff* 2317 (K, NY, S). **Esmeraldas**: *J.L. Clarke* 1721 (MO). **Manabí**: *H.F.A. von Eggers* 15090 (K, P, US).

**COLOMBIA. Atlántico**: *G. Dugand* 4828 (COL). **Antioquia**: *C. Feddema* 2000 (NY). **Chocó**: *A. Gentry & Juncosa* 40942 (COL, MO). **Magdalena**: *H.H. Smith* 2669 (K, MO, P, S). **Nariño**: *J.M. Idrobo* 1428 (COL).

**VENEZUELA. Delta Amacuro**: *J. Steyermark* 114924 (MO). **Falcón**: *J. Steyermark et al.* 111134 (MO). **Miranda**: *L. Aristiguieta* 4149 (MO).

**PANAMA.** Chagres, *A. Fendler* 240 (K, MO); Canal area, *W.G. D’Arcy* 249 (MO); ibid., *A. Gentry* 4851 (F).

**COSTA RICA.** Limón, *B. Hammel et al.* 19670 (MO).

**NICARAGUA.** Río San Juan, *E.B. Nelson* 5279 (F, GU, MO).

**HONDURAS.** Roatán, *C.H. Nelson & E. Romero* 4515 (MO).

**BELIZE.***W.A. Schipp* 497 (NY, S); *D.R. Stoddart* 436 (P).

**GUATEMALA.***J. Steyermark* 39843 (F).

**MEXICO. Baja California Sur**: *J.J. Pérez* 71 (HCIB); *M. Domínguez* 646 (IEB). **Campeche**: *E.F. & H. Cabrera* 13405 (MEXU, MO). **Guerrero**: *L. Lozada* 4472 (IEB). **Jalisco**: *E.J. Lott* 2554 (MO). **Quintana Roo**: *E.F. & H. Cabrera* 4338 (MO). **Sinaloa**: *T.R. Van Devender et al.* 2007-1318 (ARIZ). **Sonora**: *S.F. Friedman* 37-96 (ASU). **Tabasco**: *M.A. Magaña* 479 (MO, XAL). **Tamaulipas**: *C.G. Pringle* 6358 (MO, P, S). **Veracruz**: *L.I. Nevling & A. Gómez-Pompa* 2452 (F).

**UNITED STATES. Alabama**: *S.M. Tracy* 6492 (BM). **Florida**: *F. Rugel* 311; *A.H. Curtiss* 2156 (BM, K). **Georgia**: *H. Holland* 228 (GA). **Louisiana**: *S.M. Tracy* 122 (BM). **Mississippi**: *D. Demaree* 33535 (S). **Texas**: *G. Gust & J.R. Stone* 320 (MO).

**BAHAMAS.***P. Wilson* 7279 (K, NY), 7541 (K, NY); *D.C. Correll* 46286 (NY).

**CUBA.***J. Acuña* (HAJB10676); *J. Bisse et al.* (HAJB48561); *C. Wright* 3090 (BM, K, MO, S); *E.L. Ekman* 104 (NY, S).

**CAYMAN ISLANDS.***D.R. Stoddart* 7034 (BM).

**JAMAICA.***G.R. Proctor* 21393 (BM).

**HAITI.***E.L. Ekman* H5179 (K, NY, S).

**DOMINICAN REPUBLIC.***E.L. Ekman* H12226 (S); *A.H. Liogier* 12313 (NY).

**PUERTO RICO.***R.J. Wagner* 1778 (BM); *P. Sintenis* 976 (BM, K, P, S); *J.A. Shafer* 2399 (NY).

**LESSER ANTILLES. St Lucia**: *R.A. Howard et al.* 19987 (A, BM, NY). **Guadeloupe**: *A. Duss* 3966 (NY). **Martinique**: fide Powell (1979). **St Vincent**: fide Powell (1979).

**TRINIDAD.***W.E. Broadway* 8013(BM, MO), 9120 (BM, K, MO). **Tobago**: *H.F.A. von Eggers* 5900 (K, P).

**HAWAII.***Faurie* 1034 (BM).

#### Note.

A very distinctive species because of its habitat (maritime sands), whitish corolla and unusual, although very variable, small leaves.

••• Clade E (species 389–392; Figure 1) consists of the following four species, of which only one is certainly of New World origin (*I. habeliana*). All other species in the clade are either African or of uncertain origin suggesting the clade is essentially African with *I. habeliana* having evolved from *I. violacea* in the Galapagos Islands.

### 389. Ipomoea violacea

Taxon classificationPlantaeSolanalesConvolvulaceae

L., Sp. Pl. 161. 1753. (Linnaeus 1753: 161)

Convolvulus violaceus (L.) Spreng. Syst. Veg. 1: 599. 1825 [pub. 1824]. (Sprengel 1824: 599).
Pharbitis violacea (L.) Bojer, Hort. Maurit. 227. 1837. (Bojer 1837: 227).
Calonyction comospermum Bojer, Hort. Maurit, 228. 1837. (Bojer 1837: 228), nom. illeg. superfl. Type. Based on Ipomoea violacea L.
Convolvulus grandiflorus Jacq., Hort. Bot. Vindobon. 3: 39. 1776. (Jacquin 1776: 39). Type. A plant cultivated at Vienna from seed collected in Martinique, apparently, not preserved, lectotype Icon, t. 69 in Jacquin, Hort. Bot. Vindobonensis vol. 3 (1776).
Calonyction grandiflorum (Jacq.) Choisy, Mém. Soc. Phys. Genève 6: 442 [60]. 1834. (Choisy 1834: 442 [60]).
Calonyction jacquinii G. Don, Gen. Hist. 4: 264. 1838. (Don 1838: 264), nom. illeg., superfl. for Calonyction grandiflorum (Jacq.) Choisy
Ipomoea grandiflora (Jacq.) Hallier f., Bot. Jahrb. Syst. 18: 153. 1894 [pub. 1893]. (Hallier 1893a: 153), nom. illeg., non. Lamarck (1791).
Operculina grandiflora (Jacq.) House, Muhlenbergia 5: 69. 1909. (House 1909a: 69).
Ipomoea longiflora R.Br., Prodr. 484. 1810. (Brown, R 1810: 484), nom. illeg., non Ipomoea longiflora Willd. (1809). Type. AUSTRALIA. Queensland, Sweer’s Island, Gulf of Carpentaria, *R. Brown* 2741 (holotype BM000630203).
Ipomoea macrantha Roem. & Schult. Syst. Veg. 4: 451. 1819. (Roemer and Schultes 1819: 451). Type. Based on Ipomoea longiflora R.Br.
Convolvulus longiflorus (R.Br.) Spreng., Syst. Veg. 1: 595 1825 [pub. 1824]. (Sprengel 1824: 595). Type. Based on Ipomoea longiflora R.Br.
Calonyction longiflorum (R. Br.) Hasskarl, Cat. Pl. Bogor. 140. 1844. (Hasskarl 1844: 140).
Calonyction speciosum var. laeve Choisy in A.P. de Candolle, Prodr. 9: 345. 1845. (Choisy1845: 390). Type. Based on Ipomoea longiflora R.Br.
Convolvulus tuba Schldtl., Linnaea 6: 735. 1831. (Schlechtendal 1831: 735). Type. U.S. VIRGIN ISLANDS. Saint Thomas, *C. Ehrenberg* (holotype HAL0037520).
Ipomoea tuba (Schldtl.) G. Don, Gen. Hist. 4: 271. 1838. (Don 1838: 271).
Calonyction tuba (Schldtl.) Colla, Att. Sci. Ital. 150. 1840. (Colla 1840: 150).
Ipomoea glaberrima Bojer ex Bouton, J. Bot. (Hooker) 1: 357. 1834. (Bouton 1834: 357). Type. SEYCHELLES. *Bojer*s.n. (holotype K000097304).

#### Type.

Icon in Plumier, Codex Boerhaavianus, t. sub n. 851 (lectotype, designated by Manitz 1977: 269).

#### Description.

Vigorous, glabrous trailing or climbing perennial, stems woody to 10 m. Leaves petiolate, 5–16 × 5–14 cm, ovate to suborbicular (rarely 3-lobed), shortly acuminate, mucronulate, base cordate with rounded auricles, glabrous, prominently reticulate below; petioles 3.5–11 cm. Flowers opening at night, usually solitary (rarely up to 3), pedunculate from the leaf axils, peduncles 2.5–10 cm; bracteoles 1–2 mm, scale-like, caducous; pedicels 2–4 cm, noticeably thickened upwards; sepals subequal, 16–23 mm, suborbicular to elliptic, obtuse, sometimes mucronulate, glabrous; corolla hypocrateriform, with long cylindrical tube 5–9 cm in length and spreading limb c. 4–8 cm diam., white except for yellow lines on lobes, glabrous, stamens included or shortly exserted. Capsules 20–25 mm, compressed globose, glabrous; seeds 10–12 × 8 mm, blackish, puberulent except for shaggy hairs on the margins.

#### Illustration.

Figures 11G, 191; Proctor (2012: 550); Acevedo-Rodríguez (2005: 183); Bosser and Heine (2000: 42); Deroin (2001: 255).

**Figure 190.**
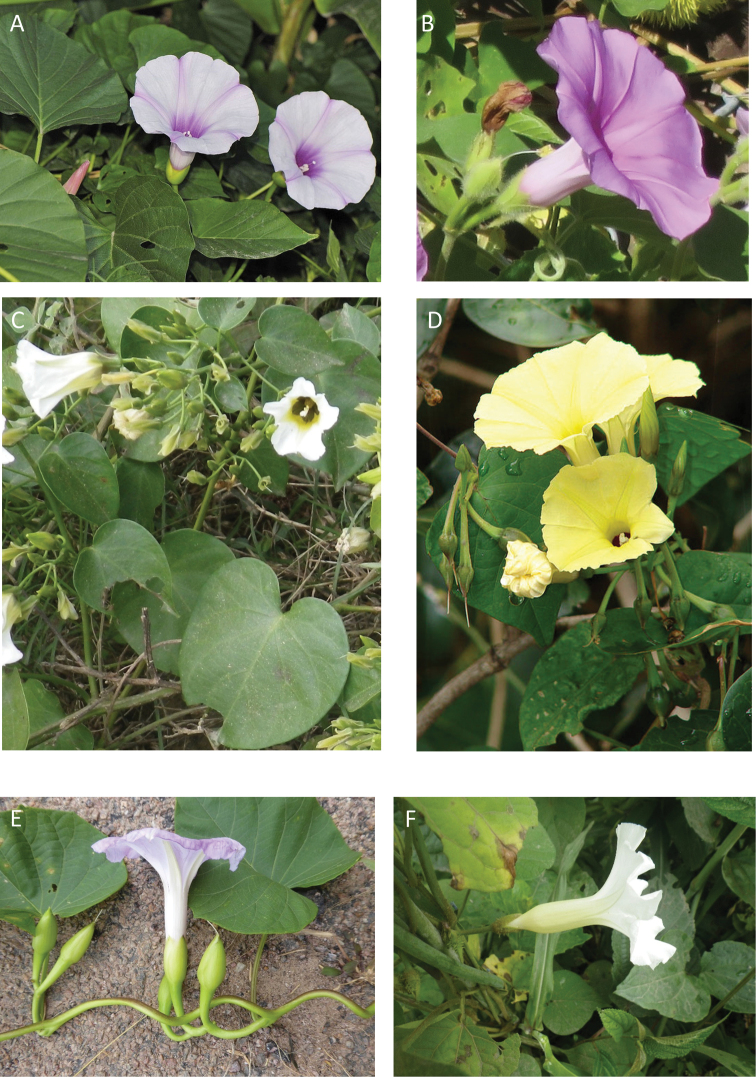
Photographs of *Ipomoea* species. **A***I. tiliifolia***B***I. rubens***C***I. corymbosa***D***I. ochracea***E***I. peruviana***F***I. echinocalyx*. **A**http://tropical.theferns.info/**B** John Pink **C** John Wood **D** Starr Environmental **E** Maira Martinez **F** John Wood.

**Figure 191.**
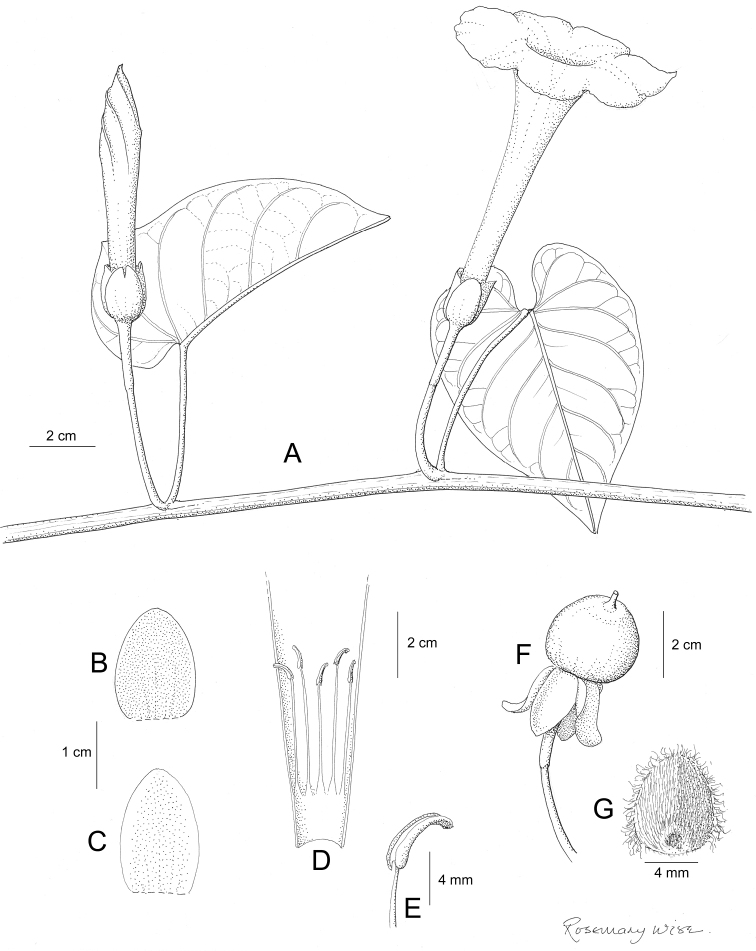
*Ipomoea violacea*. **A** habit **B** outer sepal **C** inner sepal **D** corolla opened out to show stamens **E** anther **F** capsule **G** seed. Drawn by Rosemary Wise **A** from *Proctor* 28930; **B, C** from *Stoddart* 9140; **D–G** from *Stearn* 322.

#### Distribution.

Pantropical on or near seashores, growing in mangrove swamp and less commonly on beaches. In the Americas, scattered and never very abundant but most common around the Caribbean. Nearly absent from the Pacific coast, including the Galapagos Islands, and only present in the Choco of Colombia.

**BRAZIL. Bahia**: Ilha dos Frades, *M.L. Guedes et al.* 19920 (ALCB). **Paraíba**: *L.A. Pereira* 299 (JPB). **Pernambuco**: Fernando Do Noronho, *Ridley, Lea & Ramage* 92 (BM); *A.M. Miranda* 842 (PEUFR), 4130 (RB).

**FRENCH GUIANA.** Cayenne, *R. Girault* 1569 (CAY).

**SURINAM.***G.J. H. Amshoff* 1969 (MO).

**GUYANA.** Fide Austin and Huáman (1996).

**COLOMBIA. Antioquia**: *F.J. Roldán & J. Betancur* 525 (MO). **Chocó**: Capulganá, *W.G. D’Arcy* 14192 (MO). **San Andrés Island**: *Torres* 214 (COL).

**VENEZUELA. Dist. Fed.**: *R. Liesner & J. Steyermark* 12314 (MO).

**PANAMA.** San Blas Islands, *J.A. Duke* 8516 (MO).

**BELIZE.***F.R. Fosberg & Sachet* 53896 (MO).

**MEXICO. Campeche**: *E.F. Cabrera* 13440 (BM, MEXU, MO). **Quintana Roo**: *E.F. & H. de Cabrera* 6406 (MO). **Yucatán**: *E.F. & H. de Cabrera* 10424 (MEXU, MO).

**UNITED STATES. Florida**: fide Wunderlin and Hansen (2011: 392).

**BAHAMAS.***R.A. & E.S. Howard* 10091 (S); *D.S. & H.B. Correll* 48929 (MO, NY).

**TURKS & CAICOS ISLANDS.***M.D. Sanchez et al.* 10 (K).

**CUBA.***J. Acuña* (HAJB15863); *López Figeiras* 49 (HAJB): Las Villas: *A. González* 242 (BM); *A. Gentry* 51014 (MO); *J.A. Shafer* 2698 (NY).

**CAYMAN ISLANDS.***G.R. Proctor* 28930 (BM); *M.A, Brunt* 1746 (BM, MO).

**JAMAICA.***W. Stearn* 322 (BM), 733(BM); *D.R. Stoddart & S.M. Head* 9132 (BM).

**HAITI.***E.L. Ekman* H4166 (NY, S); *E.C. Leonard* 13956 (NY).

**DOMINICAN REPUBLIC.***H.A. Allard* 14340 (S); *E.L. Ekman* H10927 (S); *B.A. Lavastre* 827 (NY); *M. Mejía & Ramírez* 9852 (NY); *P.A. Poiteau* s.n. (P).

**PUERTO RICO.***P. Sintenis* 5697 (S); *A.H. Liogier* 35797 (MO, NY); *G. Breckon et al.* 4480 (NY).

**LESSER ANTILLES. U.S. Virgin Islands**: St Croix, *F.R. Fosberg* 59208 (BM), 55339 (MO, NY, P); St John: *P. Acevedo-Rodríguez & A. Siaca* 4007 (NY). **Netherlands Antilles**: St Eustatius fide Powell (1979). **St Kitts**: *G.R. Proctor* 18512 (BM). **Guadeloupe**: *C. Le Gallo* 2176 (NY). **Martinique**: *F.E. Egler* 39-189 (NY). **St Lucia**: fide Powell (1979). **St Vincent**: *R.A. Howard* 11019 (A, BM). **Grenadines**: *P. Beard* 1404 (MO). **Barbados**: *E.G.B. Gooding* s.n. [9/1940] (BM).

**TRINIDAD.** Fide Hill and Sandwith (1953). **Tobago**: fide Hill and Sandwith (1953).

**NETHERLANDS ANTILLES. Aruba**: *A. Van Proosdij et al.* 799 (MO, NY). **Curaçao**: *N.L. Britton & J.A. Shafer* 2942 (NY).

**HAWAII.** Fide www.starrenvironmental.com.

#### Note.

Despite the epithet *violacea*, this species is usually white-flowered. Pale lilac forms occur occasionally.

### 390. Ipomoea habeliana

Taxon classificationPlantaeSolanalesConvolvulaceae

Oliv., Icon. Pl. 11: 80, t. 1099. 1871. (Oliver 1871: 80)

#### Type.

ECUADOR. Galapagos Islands, Hood [Española] Island, *Habel* s.n. (holotype K000612879).

#### Description.

Scrambling liana with white latex, to c. 8 m in height; stems stout, woody, glabrous. Leaves characteristically held erect, petiolate, 6–15 × 1.8–3.5 cm, lanceolate to ovate-lanceolate, acute and long-mucronate, base broadly cuneate, both surfaces glabrous, abaxially reticulate; petioles 2.2–6.5 cm. Inflorescence of 1-several flowers in axillary compound cymes, peduncles 1–6 cm, stout, occasionally with reflexed spinules; bracteoles not seen; pedicels 8–22 mm long, thickened upwards; sepals unequal, glabrous, outer 1.2–2 × 0.7–0.8 cm, ovate, obtuse, mucronulate, inner 1.6–2.3 cm, oblong-ovate, truncate, margins scarious; corolla opening at night, glabrous, tube cylindrical, 7–9 cm long, c. 0.7 cm wide, greenish, limb 4 cm–6 cm, white, undulate. Capsules 2.2 × 1.4 cm, ellipsoid, beaked, glabrous; seeds 11 × 7 mm, densely pilose on the margin with brownish hairs c. 5 mm long.

#### Illustration.

Figures 8E, 192.

**Figure 192.**
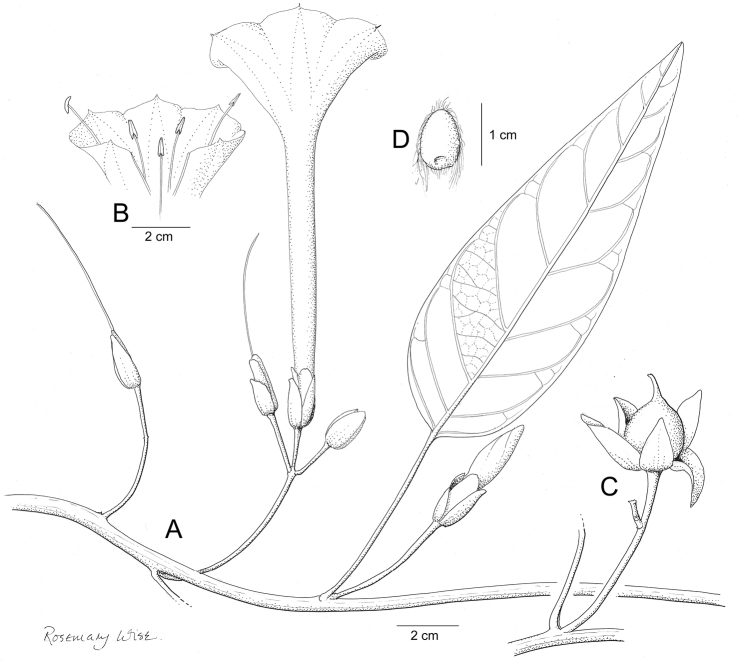
*Ipomoea habeliana*. **A** habit **B** corolla limb and anthers **C** capsule **D** seed. Drawn by Rosemary Wise **A, B** from *Bentley* 203; **C, D** from sin. data ex Herb. Hooker.

#### Distribution.

Endemic to the Galapagos Islands.

**ECUADOR. Galapagos Islands**: Santa Cruz Island, *H.J.F. Schimpf* 67 (BM, MO); *P.S. Bentley* 203 (K, MO); *T.W.J. Taylor* 90 (K).

#### Note.

Its nearest relative appears to be *Ipomoea violacea*, rather than any American species. It is probably pollinated by moths as the white flowers open in the evening.

### 391. Ipomoea aquatica

Taxon classificationPlantaeSolanalesConvolvulaceae

Forssk., Fl. Aegypt-Arab. 44. 1775. (Forsskal 1775: 44)

Convolvulus repens Vahl, Symb. Bot. 1: 17. 1790. (Vahl 1790: 17), nom. illeg., non Convolvulus repens L. (1753). Type. Based on Ipomoea aquatica Forssk.
Ipomoea subdentata Miq., Fl. Ned. Ind. 2: 614. 1857. (Miquel 1856–58 : 614). Type. INDONESIA. Java, Socrakarta, *T. Horsfield* (isotype U0001409).
Ipomoea natans Dinter & Suess., Mitt. Bot. Staatssamml. München 1: 112. 1952. (Suessenguth and Merxmüller 1952: 112). Type. NAMIBIA, Niengana, Okavanga, *K. Dinter* 7236 (holotype M, n.v., isotype PRE0125418-0).
Ipomoea reptans auct., non (L.) Poir.

#### Type.

YEMEN. Zabid, *Forsskal* s.n. (holotype C10002419).

#### Description.

Aquatic perennial, stems floating or creeping over mud and rooting at the nodes, several metres long, hollow, glabrous. Leaves petiolate, 3.5–12(–17) × 1–6 cm, deltoid, lanceolate, ovate or oblong, acute to acuminate, base hastate to weakly sagittate, the auricles usually acute, sometimes bifid, both surfaces glabrous; petioles 3–12(–17) cm. Inflorescence of lax, few-flowered, pedunculate axillary cymes, peduncles 1.5–9 cm, glabrous except for pilose base; bracteoles 1–2 mm, ovate; pedicels 2–5 cm, slender and very variable in length in the same plant; sepals subequal, outer 7–8 mm, elliptic, obtuse, mucronate, inner sepals c. 8 mm, ovate-elliptic, acute, margins sometimes scarious; corolla 4–5 cm long, funnel-shaped, pale pink or lavender with darker centre, occasionally white, glabrous, limb c. 2.5 cm diam. Capsules ovoid to subglobose, shortly rostrate, c. 10 × 8 mm, woody, glabrous, tardily dehiscent; seeds densely pubescent.

#### Illustration.

Deroin (2001: 171); Figure 167E.

#### Distribution.

Pantropical plant of Old World origin growing in muddy swamp and on lake margins. In the Americas it is well naturalised and sometimes regarded as invasive, as in Florida, Cuba and Guyana, but not recorded from many areas where it might be expected including the Dominican Republic.

**BRAZIL. Amazonas**: *S.A. Mori* 21889 (NY); *W. Junk* 40 (RB).

**FRENCH GUIANA.** Mana, *G. Léotard* s.n. (photo).

**SURINAM.** Corantijne River, *J. Lanjouw* 56 (K, RB), 603 (MO); Paramaribo, *B.E. Hammel & S. Koemar* 21202 (MO).

**GUYANA.***Jenman* 4837 (K); 5860 (K); *Harrison* 1661 (K); *D.H. Davis* 304 (K); *A.S. Hitchcock* 16690 (NY, S); Georgetown, *K.F. Robertson & D.F. Austin* 329 (MO).

**PERU. Loreto**: Iquitos, *T. Croat* 20105 (MO, P, RB); ibid., *A. Gentry et al.* 22130 (F, MO, USM); Maynas, Punchana, *M. Rimachi* 11086 (USM).

**ECUADOR. Guayas**: *C.H. & P. Dodson* 11235 (MO).

**COLOMBIA. Amazonas**: *R.E. Schultes et al.* 24129 (GH). **Córdoba**: Purisima, *F.J. Roldán* 1649 (MO).

**PANAMA.***V. Dunlap* 404 (F).

**COSTA RICA.***B. Hammel & Pérez* 24406 (MO).

**BELIZE.** Jones Lagoon, *P. Gentle* 1481 (K, MICH, MO).

**UNITED STATES. Florida**: Pinellas Co., *D.W. Hall* 1736 (BM). **Mississippi**: *C.T. Bryson* 16229 (VS).

**CUBA. La Habana**: *A.H. Curtiss* 685 (BM, K, MO, NY, P). **Matanzas**: *Bro. Alain* 3912 (NY). **Pinar del Río**: *P. Wilson* 9277 (K, NY). **Villa Clara**: *Bro. León* 9422 (NY).

**JAMAICA.***G.R. Proctor* 33066 (BM), 37950 (MO, NY); *W. Stearn* 391 (BM).

**HAITI.** St Louis du Nord, *E.L. Ekman* H5182 (K, NY, S).

**DOMINICAN REPUBLIC.** Doubtfully present, not included by Liogier (1994) but cited by Austin and Huámam (1996).

**PUERTO RICO.** Fide Acevedo-Rodríguez and Strong (2012).

**LESSER ANTILLES. Guadeloupe**: *G.R. Proctor* 19949 (BM); *A. Duss* 3502 (NY, P); *A. Raynal-Roques* 21883 (P). **Martinique**: *Stehlé* s.n. (P).

**TRINIDAD.***W.E. Broadway* 9102 (BM, K).

**NETHERLANDS ANTILLES. Aruba**: *A. Van Proosdij* 804 (MO, NY).

**HAWAII.** Maui, *C.R. Annable* 3892 (NY); Oahu, *W. Hillebrand* s.n. (BM); *O. Degener* 5999 (K); *Faurie* 1033 (BM, P).

#### Notes.

Popular in SE Asia as a stir-fried vegetable but not generally eaten in the Americas. White and pink flowered varieties are sometimes noted.

Usually easily identified by its aquatic habitat. The stems root at the nodes on mud but become free-floating on water. The leaves are variable but often narrowly lanceolate and sagittate.

### 392. Ipomoea cairica

Taxon classificationPlantaeSolanalesConvolvulaceae

(L.) Sweet, Hort. Brit., 2: 287. 1826. (Sweet 1826: 287)

Convolvulus cairicus L., Syst. Nat. (ed. 10) 2: 922. 1759. (Linnaeus 1759a: 922). Type. Icon, t. 70, Vesling in De Plantis Aegypti (Alpino 1640), lectotype, designated by Bosser and Heine 2000: 32).
Ipomoea palmata Forssk, Fl. Aegypt-Arab. 43. 1775. (Forsskal 1775: 43). Type. EGYPT. Rosetta, *P. Forsskal*s.n. (lectotype C10002422, designated here).
Convolvulus tuberculatus Desr., Encycl. 3: 545. 1792 [dated1789]. (Desrousseaux 1792: 545). Type. URUGUAY. Montevideo, Commerson s.n. (holotype P-LAM00357568).
Ipomoea tuberculata (Desr.) Roem. & Schult., Syst. Veg. 4: 208. 1819. (Roemer and Schultes 1819: 208).
Modesta tuberculata (Desr.) Raf., Fl. Tellur. 4: 76. 1836 [pub. 1838]. (Rafinesque 1838a: 76).
Ipomoea senegalensis Lam., Tabl. Encycl. 1: 464. 1793. (Lamarck 1793: 464). Type. SENEGAL. Roussillons.n. (holotype P-LAM00357478).
Batatas senegalensis (Lam.) G. Don, Gen. Hist. 4: 261. 1838. (Don 1838: 261).
Convolvulus quinquelobus Vahl, Symb. Bot. 3: 1794. (Vahl 1794: 52), Type. U.S. VIRGIN ISLANDS. St Croix, *H. West* (holotype C10009663).
Ipomoea quinqueloba (Vahl) Roem. & Schult., Syst. Veg. 4: 208. 1819. (Roemer and Schultes 1819: 208).
Ipomoea pentaphylla Cav., Icon. 3: 29. 1795. (Cavanilles 1795–1796: 29), nom. illeg., non Ipomoea pentaphylla Jacq. (1789). Type. URUGUAY. L. Née s.n. (lectotype MA 475852, designated here).
Ipomoea stipulacea Jacq., Pl. Hort. Schoenbr. 2: 39, t. 199. 1797. (Jacquin 1797b: 39). Type. Cultivated plant from Mauritius, apparently not preserved.
Ipomoea stipulacea forma pluriflora Meisn. in Martius et al., Fl. Brasil. 7: 288. 1869. (Meisner 1869: 288), nom. illeg. superfl., autonymic variety.
Convolvulus heptaphyllus Willd., Ges. Naturf. Freunde Berlin Neue Schriften 4: 196. 1803. Rottler 1803: 196). Type. INDIA. Madras, Marmelon, *Rottler*s.n. (holotype B-W3721).
Ipomoea cavanillesii Roem. & Schult., Syst. Veg. 4: 214. 1819. (Roemer and Schultes 1819: 214). Type. Based on Ipomoea pentaphylla Cav.
Convolvulus cavanillesii (Roem. & Schult.) Spreng., Syst. Veg. 1: 590. 1825 [pub. 1824]. (Sprengel 1824: 590).
Batatas cavanillesii (Roem. & Schult.) G. Don, Gen. Hist. 4: 262. 1838. (Don 1838: 262).
Ipomoea vesiculosa P. Beauv., Flore d’Oware 2: 73. 1819. (Beauvois 1808–20 73). Type. NIGERIA. Oware, *P. de Beauvois* (holotype G00415171, isotype G).
Ipomoea pulchella Roth, Nov. Pl. Sp. 115. 1821. (Roth and Heyne 1821: 115). Type. INDIA. *Heyne* in *Wallich* 1353B (K-W001112855, lectotype designated here).
Ipomoea heptaphylla Voigt, Hort. Suburb. Calcutt. 360. 1845. (Voigt 1845: 360). Type. Based on Ipomoea pulchella Roth.
Convolvulus lymphaticus Vell. Fl. Flumin.70, t. 47. 1825 [pub. 1829]. (Vellozo 1829: 70). Type. BRAZIL. (lectotype, original parchment plate of Flora Fluminensis in the manuscript section of the Biblioteca Nacional, Rio de Janeiro [cat. No.: mss1198651-047], designated here; later published in Vellozo, Fl. Flum. Icon. 2: t. 47 1827. [pub. 1831]).
Ipomoea tuberculata var. abbreviata Choisy in A.P. de Candolle, Prodr. 9: 387. 1845. (Choisy 1845: 387). Type. BRAZIL. Rio de Janeiro, *Martius* 981 (lectotype M0184898, designated here).
Ipomoea stipulacea forma uniflora Meisn. in Martius et al., Fl. Brasil. 7: 288. 1869. (Meisner 1869: 288). Type. Based on Ipomoea tuberculata var. abbreviata Choisy
Ipomoea cairica var. uniflora (Meisn.) Hoehne, Anexos Mem. Inst. Butantan, Secc. Bot. 1, Fasc. 6: 77. 1922. (Hoehne 1922: 77).
Ipomoea bouvetii Duchass. & Walp., Linnaea 23: 752. 1850 [pub. 1851]. (Duchassaing and Walpers 1850–51: 752). Type. Guadeloupe (lectotype P00622231, designated here).
Convolvulus paniculatus Naves in Blanco, Fl. Filip., ed. 3, 1: 131. 1877. (Blanco 1877–80: 131). Type. Icon, t. 32 in Blanco, Fl. Filip., ed. 3., lectotype, designated here).
Ipomoea tuberculata var. trichosperma Hilleb., Fl.Hawaii Islands 315 (1888). (Hillebrand 1888: 316). Type. HAWAII. “common on all islands”, no specimen cited.
Ipomoea tuberculata var. lineariloba Hillebr., Fl.Hawaii Islands 316 (1888). (Hillebrand 1888: 316). Type. HAWAII. South coast of Molokai, no specimen cited.
Ipomoea cairica var. lineariloba (Hillebr.) Deg. & Ooststr. in O.Deg., Fl. Hawaiiensis, fam. 307. 1938. (Degener 1932–1940; fam. 307).
Ipomoea palmata var. gracillima Collett & Hemsl, J. Linn. Soc.Bot. 28: 96.1890. (Collett and Hemsley 1890: 97). Type. MYANMAR (BURMA), Meiktila *H. Collett* 40 (lectotype K000830810, designated here).
Ipomoea gracillima (Collett & Hemsl.) Prain. J. Asiat. Soc. Bengal 63(2): 111. 1894. (Prain 1894: 111), comb. illeg., non Ipomoea gracillimaPeter (1891).
Ipomoea cairica var. gracillima (Collett & Hemsl,) C. Y. Wu, Rep. Stud. Pl. Trop. Subtrop. Yunnan 1: 120. 1965. (Wu 1965: 120).
Ipomoea cairica var. hederacea Hallier f., Bull. Herb. Boiss. 6: 546.1898. (Hallier 1898b: 546). Type. MADAGASCAR. *G.F. Scott Elliot* 3018 (holotype K000097177).
Ipomoea rosea var. pluripartita Hassl., Trab. Mus. Farmacol. 21: 98. 1909. (Hassler 1909: 98). Type. PARAGUAY/ARGENTINA. Río Pilcomayo, T. Rojas 184 (isotype S12-2031).
Ipomoea cairica var. obtusata Hoehne, Anexos Mem. Inst. Butantan, Secc. Bot. 1, Fasc. 6: 77. 1922. (Hoehne 1922: 77). Type. BRAZIL. São Paulo, Praia Grande, *A. Löfgren* 4108 (holotype SP000572).
Ipomoea funaria Larrañaga, Escr. Larrañaga 2: 78. 1923. (Larrañaga 1923: 78). Type. URUGUAY, not specified.
Ipomoea palmata var. semine-glabra Blatter & Hallberg, J. Bombay Nat. Hist. Soc. 26: 546. 1919. (Blatter and Hallberg 1919: 546). Type. INDIA. Vinjorai, *E. Blatter* 6675 (holotype BLATT).
Ipomoea cairica var. semine-glabra (Blatter & Hallberg) Bhandari, Fl. Indian Desert 253 (1978)

#### Type.

Based on *Convolvulus cairicus* L.

#### Description.

Twining perennial herb to 3 m, stems glabrous, often muricate. Leaves petiolate, digitately divided into 5–7 leaflets, the laterals sometimes joined at base, leaflets 1–5 × 0.3–1 cm, lanceolate or oblong-lanceolate, acute and mucronate, glabrous; petioles with stipule-like outgrowths at base, 1–5 cm. Flowers usually solitary, sometimes in shortly pedunculate, 2–3-flowered axillary cymes; peduncles 0.3–1 cm; bracteoles 1–2 mm, oblong, caducous; pedicels 0.3–2.5 cm; sepals slightly unequal, glabrous with scarious margins, outer 5–7 × 4 mm oblong-ovate, acute, often abaxially rugose, inner 6–8 mm, broadly ovate-elliptic, obtuse; corolla 4.5–7 cm long, funnel-shaped, pink, glabrous, limb 4 cm diam., unlobed. Capsules 1–1.3 cm, subglobose, glabrous; seeds 5–6 mm, tomentellous with longer caducous marginal hairs, rarely subglabrous.

**Illustrations.**O’Donell (1959b: 129); Bosser and Heine (2000: 33); Deroin (2001: 179); Figure 167B.

#### Distribution.

A species of Old World origin, now widespread throughout tropical and subtropical regions up to least 2600 m, but much more common in some regions than others, such as northern Argentina, eastern Paraguay and southern Brazil; unexpectedly absent in others, such as Hispaniola (Liogier 1994). It is well naturalised in waste places, usually near settlements but is also cultivated so occurring in and around gardens.

**URUGUAY.***W.G. Herter* 271 (MO, P), 1373 (S), 1878 (S).

**ARGENTINA. Catamarca**: *I. Brizuela* 88 (RB). **Córdoba**: *H.H. Bartlett* 20092 (P). **Corrientes**: *T.M. Pedersen* 7347 (C, P, S); *Huidobo* 2171 (BM). **Formosa**: Est. Guayacolec, *H. Maturo & D. Prado* 74 (BM, FCQ). **Jujuy**: *J. Araque & F.A. Barklay* Ar520 (P). **Misiones**: *E.L. Ekman* 1428 (S); *G.J. Schwarz* 5239 (LIL, P, RB).

**PARAGUAY.***Jorgensen* 4035 (MO, S). **Alto Paraná**: *Itaipú Binacional* 62 (MO). **Central**: *L. R. Landrum et al.* 8559 (ARIZ, FCQ); *B. Balansa* 1059 (K). **Cordillera**: *N. Soria* 2223 (FCQ). **Itapúa**: *Pin et al.* 646 (PY); Isla Yaciretá, *M. Peña Chocarro et al.* 1805 (BM, FCQ). **Paraguarí**: P.N.Ybyciú, *Schmeda* 351 (FCQ). **Pres. Hayes**: Río Negro on route to Fortin Gen. Bruguez, *E. Zardini and da Silva* 43193 (MO, PY); Puente Remanso, *F. Mereles* 1616 (FCQ).

**BRAZIL. Bahia**: Ilhéus, *J.L. Hage & E.B. dos Santos* 1586 (K). **Dist. Fed.**: *M.P. Ferreira* 14 (HUFU). **Espirito Santo**: *Boudet Fernandes* 1622 (MO). **Mato Grosso**: *Saddi* 3496 (RB). **Minas Gerais**: *W.N. Gonçales* s.n. (RB). **Paraná**: Tomazina, *J.C. Lindeman & J.H. de Haas* 3139 (K). **Rio de Janeiro**: *L. Riedel* 690 (K); *L.C. Giordano & L.H. de Andrade* 37 (K, RB); Raza Island, *J. Banks & D. Solander* s.n. [1768] (BM). **Rio Grande do Sul**: Rio Pardo, *Palacios Cuezzo* s.n. [10/2/1948] (K, W). **Santa Catarina**: *F. Mueller* 440 (K). **São Paulo**: *G.O. Joaquim* 113 (RB).

**GUYANA.***A.S. Hitchcock* 16723 (GH, NY, S); *Parker* s.n. (K).

**BOLIVIA. Cochabamba**: *J.R.I. Wood* 20391 (BOLV, K, LPB). **La Paz**: *L. Cayola et al.* 960 (BOLV, LPB, MO). **Santa Cruz**: *M. Nee* 47897 (NY, MO, USZ). **Tarija**: *L. Bohs* 2074 (GH, LPB).

**PERU. Lima**: Canta, *G. Vilcapoma* 8010 (USM). **Pasco**: Oxapampa, Nueva Bema, *R. Vásquez et al.* 36422 (MO, OXF, USM).

**COLOMBIA. Chocó**: *R. Fonnegra* 6738 (MO). **Santander**: *J.H. Langenheim* 3005 (COL).

**VENEZUELA. Carabobo**: *B. Trujillo* 18023 (MO). **Mirana**: *G. Morillo* (MO).

**MEXICO. Colima**: Manzanillo, *E. Palmer* 1631 (K). **Guanajuato**: Apaseo El Alto, *R. Carranza & E. Pérez* 4991 (IEB). **Guerrero**: *E. García* 43 (IEB). **Michoacán**: Morelia, R. Pedraza 308 (IEB). **Oaxaca**: El Mogotón, *I. López* 89 (IEB). **Sonora**: fide Felger et al. (2012).

**UNITED STATES. Florida**: *H. Moldenke* 278 (K, S); *A.H. Curtiss* 6496 (E, K).

**CUBA.***H. Manitz* s.n. [2/11/1989] (HAGB); *E.L. Ekman* 861 (S); *J.G. Jack* 5322 (A, P).

**CAYMAN ISLANDS.***G.R. Proctor* 11967 (BM)

**JAMAICA.***G.R. Proctor* 17470 (BM), 19662 (B).

**LESSER ANTILLES. Guadeloupe**: fide Powell (1979). **Grenada**: fide Powell (1979). **Barbados**: *E.G.B. Gooding* 389 (BM).

**TRINIDAD.***J. Becker* 528 (K, P). **Tobago**: *Clement & Ryves* 93/184 (BM); *W.E. Broadway* 4134 (S).

**HAWAII.***A.A. Heller* 2045 (BM); *Faurie* 1028 (BM); *B. Panahi* 398 (K); *Rock* s.n. (K).

#### Notes.

Readily identified by the 5–7-foliolate leaves and the nearly unique, stipule-like outgrowths at the base of the petiole. It is, however, extremely variable, especially so in Hawaii. Many plants from Hawaii have elliptic leaflets up to 3.5 cm wide and correspondingly robust pseudo-stipules. There also occurs in Hawaii a var. lineariloba with very long narrow elliptic leaflets, *Rock* s.n. (K) being a good example of this variety. Var. hederifolia with lobed leaves, in which the leaflets are partially fused is also present in Hawaii. It is one of a number of recognised Old World varieties.

We have found a specimen (*W.G. Herter* 99285 from Miguelete near Montevideo in Uruguay) at S labelled as an isotype of Ipomoea cairica forma obscura but have been unable to trace the publication of this name.

••• Species 393–419 This is the large, essentially Old World Clade (OWC), containing a small number of naturally occurring New World species as well as several Old World species which are ancient or recent introductions to the New World.

### 393. Ipomoea nervosa

Taxon classificationPlantaeSolanalesConvolvulaceae

(Burm. f.) J.R.I. Wood & Scotland, Nature Plants 2019, suppl. inf.: 29. (Muñoz-Rodríguez et al. 2019, suppl. inf.: 29)

Convolvulus nervosus Burm. f., Fl. Indica 48: 1768. (Burman, NL 1768: 48). Type. INDIA. Coromandel, Outgaerden [*Van Outgaarden*] s.n. (lectotype G-PREL, designated by Staples and Jacquemoud 2005: 60).
Lettsomia nervosa (Burm. f.) Roxb., Fl. Ind. 2: 78. 1824. (Roxburgh 1824: 78).
Argyreia nervosa (Burm. f.) Bojer, Hortus Maurit. 224. 1837. (Bojer 1837: 224).
Rivea nervosa (Burm. f.) Hallier f., Bull. Herb. Boiss., 5: 381. 1897. (Hallier 1897a: 381).
Convolvulus speciosus L.f., Suppl. Pl. 137. 1781 [pub. 1782]. Type. BRAZIL. Vandelli, LINN-HL218-23. (leaf only).
Ipomoea speciosa (L.f.) Pers., Syn. Pl. 1: 183. 1805. (Persoon 1805: 183).
Argyreia speciosa (L.f.) Sweet, Hort. Brit. 289, 1827. (Sweet 1826: 289).
Samudra speciosa (L.f.) Raf., Fl. Tellur. 4: 72.1838. (Rafinesque 1838a: 72).
Ipomoea valerii Standl. & L.O. Williams, Ceiba 3: 55. 1952. (Standley and Williams 1952a: 55). Type. HONDURAS. Morazán. Sabanagrande, *J. Valerio-R*. 3272 (holotype US00111484 (Received from EAP), isotype F).

#### Type.

Based on *Convolvulus nervosus* Burm. f.

#### Description.

Twining liana climbing to several metres, stems, stout, white-sericeous, latex white. Leaves petiolate, large, 9–17 × 8–15 cm, ovate cordate, apex acute to rounded and shortly mucronate, adaxially green, glabrous, abaxially white tomentose; petioles 3–10 cm, white sericeous. Inflorescence of long-pedunculate, bracteolate cymes, often compact; peduncles 15–21 cm, sericeous; bracteoles 2.5–6 × 1.8–3.2 cm, ovate, to broadly oblong-elliptic, long-acuminate, papery, pale yellow-green, sericeous, deciduous; secondary peduncles 1 cm; pedicels 2–6 mm, sericeous; sepals 12–16 × 10–11 mm, elliptic-obovate, mucronate, sericeous; corolla 5–6 cm long, dark pink, sericeous, abruptly widened above a short basal tube, funnel-shaped; limb lobed, c. 4 cm diam. Capsules c. 2 × 1.5 cm, subglobose, glabrous, partially enclosed by the strongly accrescent sepals, which can reach up to 2.5 × 2.5 cm; seeds 6 × 4 mm, shortly tomentose.

#### Illustration.

Austin (1975b: 215); Liogier (1994: 115); Bosser and Heine (2000: 25); Deroin (2001: 137) all as *Argyreia nervosa*.

#### Distribution.

Native of Asia of imprecise origin. Most records even from the Old World are of cultivated plants. In the Neotropics it is sometimes cultivated for its flowers, principally around the Caribbean and is occasionally reported as an escape.

**BRAZIL.** Type of *Convolvulus speciosus* L.f.

**PANAMA.** Fide Austin (1975b).

**HONDURAS.** Type of *Ipomoea valerii* Standl. & L.O. Williams.

**CUBA.** La Habana, *E.L. Ekman* 1254 (S)

**DOMINICAN REPUBLIC.***E.L. Ekman* H15355 (S); *W. Allard* 15732 (S).

**JAMAICA.***D. Hummel* 29/4/1958 (S).

#### Note.

This is the only representative of the large, entirely Old World Argyreia Clade that occurs in the Neotropics.

• Species 394–397 form a clade of morphologically very similar species, native to the neotropics. Molecular studies using *ITS* strongly suggest that *Ipomoea abutiloides*, *I. pearceana* and *I. sericosepala* are sisters of the African species *Ipomoea shirensis* Oliv., which is very similar morphologically to *I. sericosepala*.

### 394. Ipomoea abutiloides

Taxon classificationPlantaeSolanalesConvolvulaceae

(Kunth) G. Don, Gen. Hist. 4: 273. 1838. (Don 1838: 273)

Convolvulus abutiloides Kunth, Nov. Gen. Sp. 3: 106. 1818 [pub.1819]. (Kunth 1819: 106). Type. ECUADOR. Guayaquil, Bonplands.n. (holotype P00670760).
Rivea abutiloides (Kunth) Hallier f., Bot. Jahrb. 18: 158. 1893. (Hallier 1893b: 158).
Turbina abutiloides (Kunth) O’Donell, Lilloa 23: 505. 1950. (O’Donell 1950b: 505).
Ipomoea floribunda Moric., Pl. Nouv. Amer. 46, t. 31. 1838. (Moricand 1834–47). Type. BRAZIL. Bahia, Blanchet 926 (holotype G00222174).
Ipomoea floribunda var. blanchetii Meisn. in Martius et al., Fl. Brasil. 7: 262. 1869, nom. illeg., autonymic variety. (Meisner 1969: 262).
Ipomoea abutiloides var. kunthiana Kuntze, Revis. Gen. Pl. 2: 443. 1891, nom. illeg., autonymic variety (Kuntze 1891: 443).
Ipomoea abutiloides var. hartwegiana Kuntze, Revis. Gen. Pl. 2: 444. 1891. (Kuntze 1891: 444). Type. ECUADOR. [Guayas]. Guayaquil, *Hartweg*s.n. (lectotype K000370540 ex Herb. Bentham from Guayaquil annotated var. hartwegiana, designated here).

#### Type.

Based on *Convolvulus abutiloides* Kunth

#### Description.

Liana climbing high over shrubs to 7 m, stems white-tomentose, especially when young, roots tuberous. Leaves petiolate, 3–10 × 3–11 cm, broadly ovate, base truncate to subcordate, apex retuse, rounded or obtuse, adaxially pubescent, abaxially grey-tomentose; petioles (1–)3–6(–10) cm, pubescent to tomentose. Inflorescence of axillary and terminal cymes, the later compound and often paniculate or racemose in form, sometimes distinctly leafy; peduncles 2–11 cm, tomentose; bracteoles 2–9 mm, linear, tomentose, soon caducous; short (c. 5 mm), secondary and tertiary peduncles often present; pedicels 5–25 mm, tomentose; calyx narrow and ±cylindrical, sepals subequal, 10–14 × 4–7 mm, oblong-obovate, obtuse to rounded, drying brown, glabrous or nearly so, inner c. 2 mm longer than outer, the margins broad and scarious; corolla 5–7 cm long, funnel-shaped, pink, pubescent in bud, glabrescent, limb 4–5 cm, weakly lobed. Capsules glabrous, ovoid, 14–17 × 6–7 mm; seeds reported as usually solitary, 9–10 mm long, minutely tomentellous.

#### Illustration.

Figure 193.

**Figure 193.**
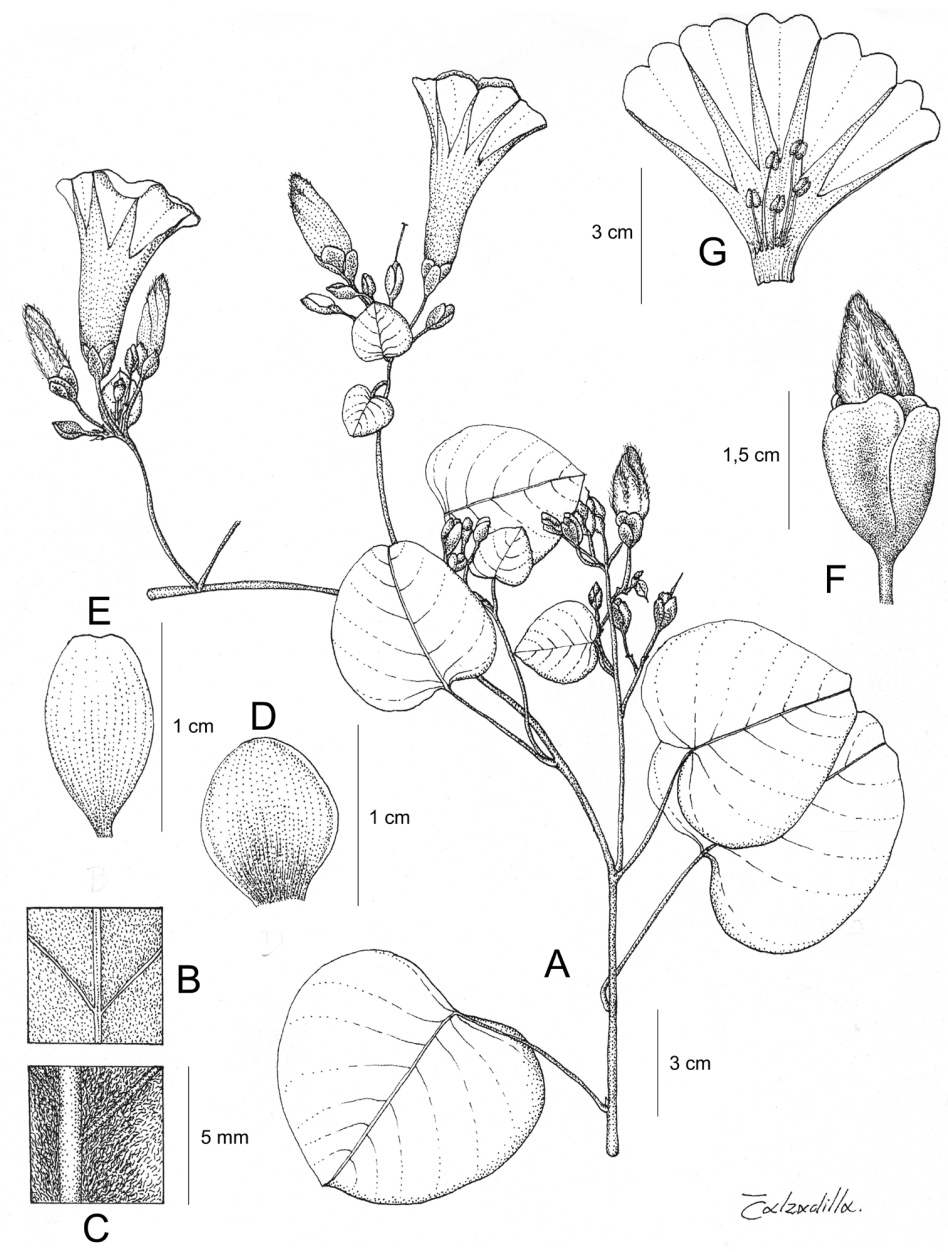
*Ipomoea abutiloides*. **A** habit **B** adaxial leaf surface **C** abaxial leaf surface **D** outer sepal **E** inner sepal **F** bud **G** corolla opened up to show stamens. Drawn by Eliana Calzadilla. **A** from *Wood et al.* 26090; **B–G** from *Wood et al.* 26136.

#### Distribution.

Scattered in seasonally dry tropical forest below 1000 m in tropical South America, most common in the Chiquitano dry forest of eastern Bolivia, around Guayaquil and in northern Colombia and Venezuela.

**PARAGUAY. Alto Paraguay**: Gabino Mendoza, *F. Mereles & R. Degen* 5946 (CTES, FCQ); trayecto a Cerro Chovoreca, *F. Mereles* 6608 (CTES, FCQ).

**BRAZIL. Bahia**: Morro do Chapéu, *A. Oliveira et al.* 144 (HUEFS, K); Ilhéus. *J. Hage & H.S. Brito* 1037 (K, CEPEC); Serra de Jatobá, *R.M. Harley et al.* 22022 (CEPEC, K). **Goiás**: *H.S. Irwin et al.* 15745 (MO). **Mato Grosso do Sul**: Corumbá, *Dorrien Smith* 32 (K); Rio Verde, *G. Hatschbach* 33952 (MBM, MO). **Minas Gerais**: *W.R. Anderson et al.* 37194 (NY).

**BOLIVIA. Chuquisaca**: Siles, *M. Serrano* 1506 (HSB). **Santa Cruz**: Chiquitos, Limoncito, *J.R.I. Wood & M. Mendoza* 27315 (K, LB, USZ); Cordillera, Santa Cruz-Abapó, *M. Nee* 48677 (K, MO, NY, USZ); Ibañez, *M. Nee* 48723 (NY, USZ). Ñuflo de Chávez, W of Concepción, *J.R.I. Wood & D. Soto* 27516 (K, LPB, USZ); Velasco, San Juancito, *J.R.I. Wood et al.* 26136 (K, LPB, USZ, UB).

**PERU. Amazonas**: Luya, Camporedondo, *J. Campos et al.* 3695 (MO, OXF).

**ECUADOR. El Oro**: *G. Harling & L. Andersson* 14313 (MO). **Guayas**: *G. Harling* 3009 (S); *E. Asplund* 15212 (K, S); *R. Spruce* 6494 (BM, K); *C.H. & P.M. Dodson* 11355 (MO). **Loja**: Macará, *F. Vivar* 1247 (LOJA). **Manabí**: Jipijapa, *M. Montesdeoca et al.* 641 (QAP).

**COLOMBIA. Bolívar**: *E.P. Killip & A.C. Smith* 14244 (BM), 14447 (GH, NY, S). **Cesar**: *A. Gentry et al.* 60692 (MO). **Sucre**: Coloso, *A. Gentry & H. Cuadros* 68163 (MO).

**VENEZUELA.** Maracay & Caracas, *P. Vogl & K. Suessenguth* 98 (BM, BR). **Aragua**: Tovar, *A. Fendler* 931(K, MO); El Consejo-La Victoria, *Ll. Williams & A.H.G. Alston***325** (BM, S). **Barinas**: Río Curbatí: *L. Bernardi* 1700 (K). **Falcón**: *J. A. Steyermark* 94702 (MO). **Portuguesa**: Guanare, *G. Aymard* 4287 (MO).

**PANAMA.** Coclé, *M.D. Correya* 405 (MO); Los Santos, *W.H. Lewis et al.* 2949 (MO)

#### Note.

This species is distinguished from *Ipomoea sericosepala* by the glabrous sepals and we are unaware of other distinguishing features. The following collections with very sparsely pubescent sepals were made where the range of the two species overlaps. They merit further investigation and may be hybrids:

**BOLIVIA. Santa Cruz**: Cordillera, Abapo, c. 35 km hacia Camiri, *M. Mendoza et al.* 2725 (USZ); Camiri, *M. Mendoza et al.* 2736 (USZ); Río Grande Bridge S of Abapó, *J.R.I. Wood et al.* 28017 (LPB, K, OXF).

### 395. Ipomoea sericosepala

Taxon classificationPlantaeSolanalesConvolvulaceae

J.R.I. Wood & Scotland, Kew Bull. 70(31): 21. 2015. (Wood et al. 2015: 21)

Rivea cordata Choisy in A.P. de Candolle, Prodr. 9: 326. 1845. (Choisy 1845: 326), non Ipomoea cordata L. B. Sm. & B.G. Schub. (1939). Type. BRAZIL. Minas Gerais, San Francisco prope Salgado, *Martius*s.n. (lectotype M0184947, designated by Austin and Staples 1991: 272).
Turbina cordata (Choisy) Austin & Staples, J. Arnold Arbor. 64: 488. 1983. (Austin and Staples 1983: 64).
Ipomoea martii Meisn. in Martius et al., Fl. Brasil. 7: 258. 1869. (Meisner 1869: 258), *nom. illeg*. Type. as for Rivea cordata.

#### Type.

Based on *Rivea cordata* Choisy

#### Description.

Liana climbing high over shrubs to 7 m, stems white-tomentose, especially when young, latex white. Leaves petiolate, 4–8 × 8–9 cm, broadly ovate, apex acute and mucronate or (less commonly) obtuse or retuse, base truncate to shallowly cordate, adaxially glabrous, glabrescent or shortly pubescent, abaxially grey-sericeous with long silky hairs; petioles 1–6 cm, tomentose. Inflorescence of pedunculate axillary cymes, these often leafy and appearing to be side branches; peduncles 3–13 cm; bracts resembling small leaves; bracteoles c. 5 mm long, linear-lanceolate, abaxially sericeous, caducous; secondary peduncles up to 9 cm long; pedicels 6–32 mm; sepals unequal, outer 8–10 × 3–4 mm, oblong, obtuse and sometimes mucronate, sericeous, inner 11–14 × 6 mm, elliptic-obovate, rounded, mucronate, sericeous, the margins broad, scarious, glabrous; corolla 5–7 cm long, funnel-shaped, pink, sericeous with long silky hairs, limb c. 5 cm diam., shallowly lobed. Capsules ovoid, 14–18 × 7–10 mm, glabrous; seeds 1–2, narrowly ellipsoid, 8–10 mm, tomentellous.

#### Illustration.

Figures 7E, 9G.

#### Distribution.

Restricted to scattered locations in Brazil and Bolivia. In Brazil it is far more common than *Ipomoea abutiloides* and is especially so in the state of Bahia, where it is typical of caatinga vegetation. In Bolivia it is much less common than *I. abutiloides* and with the single exception of a population on an inselberg near San José Campamento in Velasco, it is restricted to the western Chaco and Serrano Chaqueño scrub and dry forest along the Río Grande Valley entering the Andes.

**BRAZIL. Bahia**: Correntina, *R.M. Harley et al.* 21811 (CEPEC, K); Curaça, *G.C.P. Pinto & S.B. da Silva* 13413 (K); Maracás, *A. de Carvalho et al.* (CEPEC, K); Caetité, *M.L. Guedes et al.* (ALCB, K). **Ceará**: *Löfgren* 260 (S); Est. Biológica da Aiuaba, *J.R. Lemos & P. Matías* 155 (USP, K); Serra de Maranguape, *A. Ducke* 2541 (K). **Dist. Fed.**: *B.A.S. Pereira* 213 (IBGE, K, MO), *E.P. Heringer* 1393 (K); *H.S. Irwin* 13160 (MO, NY). **Goiás**: Corumbá de Goiás, *E.P. Heringer et al*. 1228 (MO, NY), 16982 (K, IBGE). **Minas Gerais**: *A. Glaziou* 19673 (K, P); *Y. Mexia* 5568 (K, MO, S); *A. Macedo* 304 (S), 1781 (BM, MO); *L.O Williams & V. Assis* 5899 (GH, MO). **Mato Grosso**: Nova Xavantina, *G.F. Arbocz* 3704 (ESA). **Paraíba**: *M.F. Agra et al.* 4068 (MO). **Pernambuco**: *L.S. Figueirêdo & W.M. Andrade* 415 (K, PEUFR); *S. Tsugaru et al.* B-1430 (MO); *A.P. Fontana et al.* 9176 (RB). **Rio Grande do Norte**: *J.G. Jardim* 6211 (UFRN). **São Paulo**: *W. Hoehne* 12742 (SP, K). **Sergipe**: *R. Simao-Bianchini* 1756 (ASE).

**BOLIVIA. Chuquisaca**: Luis Calvo, Muyupampa, *J.A. Peñaranda & J.G. Tudela* 1116 (MO, OXF); Oropeza, Río Chico valley, *J. Gutiérrez* 406 (HSB, K); Zudañez, Mojocoya, *J.R.I. Wood & H. Huaylla* 21549 (K, LPB). **Cochabamba**: Campero, Valle de Tunas Pampa, *J.R.I. Wood & M. Mendoza* 21517 (K, LPB). **Santa Cruz**: Cordillera, Boyuibe, *J.R.I. Wood et al.* 20107 (HSB, K, LPB, USZ); Vallegrande, Río Grande Valley, *J.R.I. Wood et al.* 22793 (K, LPB); Velasco, San José de Campamiento, *R. Guillén et al.* 4272 (ARIZ, NY, USZ). **Tarija**: Gran Chaco, *M. Nee & I. Linneo* 54033 (MO, NY, USZ).

#### Notes.

*Ipomoea sericosepala* and *I. abutiloides* are similar in their liana habit, leaves grey-tomentose or sericeous beneath, their oblong-elliptic sepals and their inflorescences which have a tendency to become leafy and racemose or even paniculate towards the branch tips. They are best distinguished by the sepal indumentum, *I. sericosepala* having sericeous sepals while those of *I. abutiloides* are glabrous. The two species intergrade in the Abapó area of Bolivia where their ranges overlap.

Records from Peru (Austin and Staples 1991: 273) are mostly errors for the very similar *Ipomoea pearceana* Kuntze, which differs in little more than the relatively persistent elongate- oblong bracteoles.

### 396. Ipomoea pearceana

Taxon classificationPlantaeSolanalesConvolvulaceae

Kuntze, Rev. Gen. Pl. 2: 443. 1891. (Kuntze 1891: 443)

#### Type.

PERU. [Cusco/Apurimac], common in the valley of the Apurimac, 8–9000 ft, [Jan. 1867], *R. Pearce* 1867 (lectotype K000612918, designated here; isolectotype BM).

#### Description.

Shrub 2–3 m high, white latex present; stems woody, sericeous. Leaves petiolate, 4–9 × 4–12 cm, broadly ovate, very shortly acuminate, base shallowly cordate to subtruncate, margin white-ciliolate, adaxially glabrous, abaxially grey sericeous-tomentellous, veins prominent; petioles 3.5–6 cm, sericeous. Inflorescence of axillary, pedunculate cymes; peduncles 4–12 cm, white-sericeous; bracteoles 1.3–2.7 × 0.4–0.5 cm. lanceolate, finely acuminate, boat-shaped, adaxially glabrous, abaxially grey-sericeous; secondary peduncles 4–12 mm; pedicels 6–25 mm, white-sericeous; outer sepals oblong, cuneate at base, acute and strongly mucronate, 18–25 × 7–8 mm, densely sericeous becoming less so marginally, inner sepals 20–22 × 7 mm oblong-elliptic, obtuse, mucronate, the midrib and mucro sericeous, the margins nearly glabrous; corolla 5–6 cm long, pink, sericeous in bud, funnel-shaped, filaments pink. Capsules and seeds not seen.

#### Distribution.

Almost endemic to the Apurimac Valley in Peru at about 2100 m where it grows on steep slopes in dry forest.

**PERU. Apurimac**: Abancay: *C. Vargas* 1444 (CUZ); Grau, Karrancka, *C. Vargas* 5850 (CUZ); Canyon of Río Apurimac, *J. West* 3847 (GH, MO, UC). **Cusco**: Abancay-Cusco, *R.T. Pennington et al.* 1796 (E); Anta, *W.L. Galeano* 5086 (CUZ, MO); Sisal-Cunyac, *C. Vargas* 4877 (CUZ, K). **Huancavelica**: Tayacaja, Quichicapota-Mantaro Bridge, *H.E. Stork & O.B. Horton* 10407 (K).

#### Note.

This is a poorly known species close to *Ipomoea sericosepala* differing principally in the much longer sepals and bracteoles. Some specimens, such as *O. Tovar* 3837 (USM) from Tayacaja, might be interpreted as *I. sericosepala*.

### 397. Ipomoea velutinifolia

Taxon classificationPlantaeSolanalesConvolvulaceae

J.R.I. Wood & Scotland sp. nov.

#### Type.

BRAZIL. Maranhão: Mun. Grajaú, 4 km W of Mondelandia on path to Rio Grajau, *E.L. Taylor, C.S. Rosario & J.B.F. Silva* 1326 (holotype ARIZ, isotypes MG, ?NY).

#### Description.

Perennial climber, stems relatively stout, silky-velutinous. Leaves petiolate, 5–9 × 4–8 cm, ovate, apex acute, mucronate, base very broadly cuneate to subtruncate with rounded auricles, margin undulate, adaxially softly and densely pubescent, abaxially velvety-grey; petioles 2.5–4.5 cm, velvety-grey. Inflorescence of compound axillary cymes, these often racemose in form and sometimes distinctly leafy; peduncles 2.5–5 cm, velvety-grey, often extended as a rhachis and reaching 15 cm; secondary peduncles 0.5–2 cm, velvety-grey; bracteoles caducous, not seen; pedicels 10–12 mm, puberulent; sepals subequal, 7–8 × 6–8 mm, outer ovate, obtuse, inner suborbicular, rounded, abaxially velvety-grey, adaxially glabrous; corolla 4.5–6 cm long, sericeous, funnel-shaped, exterior white, interior pale pink; ovary pubescent.

#### Illustration.

Figure 194.

**Figure 194.**
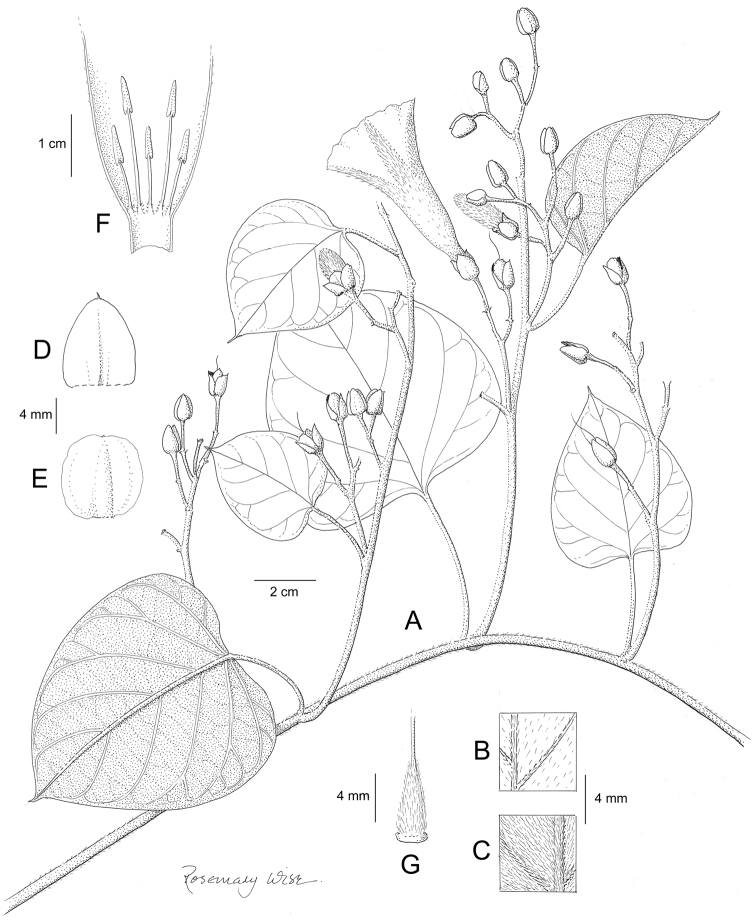
*Ipomoea velutinifolia*. **A** habit **B** adaxial leaf surface **C** abaxial leaf surface **D** outer sepal **E** inner sepal **F** corolla opened out to show stamens **G** ovary. Drawn by Rosemary Wise from *Taylor et al.* 1326.

#### Distribution.

Amazonian forest in disjunct locations of Brazil and Peru.

**BRAZIL. Maranhão**: type collection.

**PERU. Pasco**: Oxapampa, Palcazu Dist, San Cristóbal, *R. Vásquez et al.* 34378 (MO, USM); ibid., Comunidad Nativa Buenos Aires, *R. Vásquez et al.* 37328 (MO, OXF).

#### Validation.

This species was described by Wood & Scotland in Wood et al. (2017d: 32) but the phrase “species nova” was not included. This species is validated here.

#### Notes.

This species appears to be related to *Ipomoea sericosepala* because of the form of the inflorescence and the distribution of the indumentum on the corolla and almost all vegetative parts. It differs from *I. sericosepala* in the distinctive velvety-grey indumentum and in the shape and size of the sepals which are subequal, ovate, 7–8 × 6–8 mm. It differs from all related species in the densely pubescent ovary.

Its placement here is unconfirmed.

### 398. Ipomoea eriocarpa

Taxon classificationPlantaeSolanalesConvolvulaceae

R. Br., Prodr. 484. 1810. (Brown, R 1810: 484)

Convolvulus eriocarpus (R. Br.) Spreng., Syst. Veg. 1: 598. 1825 [pub. 1824]. (Sprengel 1824: 598).
Convolvulus hispidus Vahl, Symb. Bot. 3: 29. 1794, (Vahl 1794: 29). Type. “India Oriental”. Dahl (wherabouts unknown).
Ipomoea hispida (Vahl) Roem. & Schult., Syst. Veg. 4: 238. 1819. (Roemer and Schultes 1819: 238), nom. illeg., non I. hispida Zaccagni (1806).
Ipomoea sessiliflora Roth, Nov. Pl. Sp. 1821: 116. (Roth and Heyne 1821: 116.). Type. INDIA. *Heyne*s.n. (whereabouts unknown).
Convolvulus sessiliflorus (R.Br.) Spreng., Syst. Veg. 1: 599. 1825 [pub. 1824]. (Sprengel 1824: 598).
Ipomoea ligulata Bojer, Hortus Maurit. 229. 1837. (Bojer 1837: 229). Type. Not designated.
Ipomoea trematosperma Hochst ex Choisy in A.P. de Candolle, Prodr. 9: 367. 1845. (Choisy 1845: 367 sub I. sessiliflora Roth). Type. SUDAN. Kordofan, *K. Kotschy* 289 (BM, G, HBG).
Ipomoea horsfieldiana Miq., Fl. Ned. Ind. 2: 611. 1857 (Miquel 1856–58: 611). Type. INDONESIA. Java, Socrakarta, *T. Horsfield*s.n. (?holotype U0001410, isotype K).
Ipomoea hispida var. latifolia Kuntze, Rev. Gen. 2: 445. 1891. (Kuntze 1891: 445). Type. INDONESIA. Java, Probolingo, *O. Kuntze* 5994 (isotype NY00319255).
Ipomoea hispida var. angustifolia Kuntze, Rev. Gen. 2: 445. 1891. (Kuntze 1891: 445). Type. INDONESIA. Java, Willis Mountain, *O. Kuntze* 5844 (isotype NY00319254).
Ipomoea sindica Stapf, Bull. Misc. Inform. Kew 1894: 346. 1894. (Stapf 1894: 346). Type. INDIA. Sirhind, *T. Thomson* (K001081737, lectotype, designated here).

#### Type.

AUSTRALIA. *Banks & Solander* s.n. (holotype BM001040629).

#### Description.

Annual herb, stems twining or prostrate, pubescent or hispid, up to 2 m long. Leaves petiolate, 2.5–8 × 0.8–5 cm, ovate to narrowly oblong, base usually subhastate with rounded auricles, apex acute, both surfaces pilose to glabrescent; petioles 1–6 cm. Inflorescence of axillary subsessile or shortly pedunculate compact cymes; peduncles 0–15 mm; bracteoles linear; pedicels 2–5 mm; sepals subequal, 8–9 × 3–4 mm, ovate, acuminate, hispid-pilose, spreading in fruit; corolla 6–9 mm long, narrowly funnel-shaped, white, pink or mauve, hirsute, limb c. 1.5 cm diam. Capsules globose, 5–7 mm diam., pubescent, often enclosed by the calyx; seeds 2.5 mm, black, glabrous, punctate.

#### Illustration.

Bosser and Heine (2000: 40); Deroin (2001: 191).

#### Distribution.

A common Old World weedy species recorded as an adventive in the Caribbean region.

**PUERTO RICO.** Río Piedras, *J.A. Stevenson* 2278 (K, NY).

**LESSER ANTILLES. St Vincent**: “Rev. L. G.” (K).

#### Note.

A rather distinct Old World annual species because of its small hirsute flowers, ovate acuminate sepals that are spreading in fruit, and hirsute capsule. It is the only representative of an Old World Clade found in the Neotropics.

### 399. Ipomoea rubens

Taxon classificationPlantaeSolanalesConvolvulaceae

Choisy, Mém. Soc. Phys. Genève 6: 463[81]. 1834. (Choisy 1834: 463[81])

Ipomoea lilacina Blume, Bijdr. Fl. Ned. Ind. 13: 716. 1826. (Blume 1825–6: 716), nom. illeg., non Ipomoea lilacinaSchrank (1822). Type. JAVA. Batavia, *Blume* 1097 (holotype L0004203, possible isotype P).
Ipomoea riparia G. Don, Gen. Hist. 4: 265. 1838. (Don 1838: 265). Type. SÃO TOME AND PRÍNCIPE. (holotype BM000930417, fide Verdcourt 1963: 135, n.v.).
Ipomoea baclii Choisy, Mém. Soc. Phys. Genève 8(1): 60[138]. 1838. (Choisy 1838: 60[138]). Type. SENEGAL. Bacles.n. (holotype G00135884).
Ipomoea rubens var. lanata Choisy Prodr. [A.P. de Candolle] 9: 371. 1845. (Choisy 1845: 381). Type. INDIA. Pirgun, *Buchanan-Hamilton in Wallich* 2252 (isotype K).
Ipomoea lindleyi Choisy in A.P. de Candolle, Prodr. 9: 371. 1845. (Choisy 1845: 381). Type. MADAGASCAR. (holotype CGE00070, isotype BR).
Ipomoea parkeri Choisy in A.P. de Candolle, Prodr. 9: 381. 1845. (Choisy 1845: 381). Type. GUYANA. Demerara, *K.D. Parker* (holotype G 00135830, isotype K 000612844).
Pharbitis fragrans Bojer ex Choisy in A.P. de Candolle, Prodr. 9: 341. 1845. (Choisy 1845: 341). Type. MADAGASCAR. Foulepoint, *Bojer*s.n. (holotype G00135106).
Ipomoea fragrans (Bojer ex Choisy) Baker, Fl. Mauritius 209. 1877. (Baker 1877: 209).
Ipomoea parkeri var. subsericea Meisn. in Martius et al., Fl. Brasil. 7: 284. 1869. (Meisner 1869: 284). Type. SURINAM. Salem, *H.R. Wullschlaegel* 346 (BR0000005837717).
Ipomoea hellebarda Schweinf. ex Hallier f., Bot. Jahrb. Syst. 18: 142. 1893 (Hallier 1893b: 142). Type. SUDAN. Matemma, *G.A. Schweinfurth* 2176 (holotype B†?, isotype P00434209).
Ipomoea villicalyx N. E. Br., Trans. Proc. Bot. Soc. Edinb. 20: 64. 1894 (Brown, NE 1894: 64). Type. ARGENTINA or PARAGUAY. Gran Chaco, *E. Gibert* (lectotype K000612910, designated here).
Ipomoea oxyphylla Baker, Bull. Misc. Inf. Kew 1894: 71. 1894. (Baker 1894: 71). Type. ANGOLA. *Welwitsch* 6229 (holotype K000097183, isotypes LISU).
Ipomoea stuhlmannii Dammer, Pflanzenw. Ost-Afrikas 333. 1895. (Engler 1895: 333). Type. TANZANIA. Bukumbi, *Stuhlmann* 828 (holotype B†).
Ipomoea hovarum Rendle, J. Bot. 39: 58. 1901. (Rendle 1901: 58). Type. MADAGASCAR. *C.T. Hilsenberg & W.Bojer*s.n. (holotype BM).
Ipomoea brasseuriana De Wild. Ann. Mus. Congo Belge, Bot. sér. 4, [1(3)]: 115. 1903 (Wilderman 1902–3: 115). Type. CONGO. Environs du Lac Moero, *E. Verdick*s.n. (holotype BR0000008884886).
Ipomoea bonii Gagnep., Notul. Syst. (Paris) 3: 142. 1915. (Gagnepain 1915: 142). Type. VIETNAM. Hanoi, H.F. Bon 2816, 4233 (syntypes K, L, P).
Ipomoea garnieri Standl. & L.O. Williams, Ceiba 3: 128. 1952. (Standley and Williams 1952b: 128). Type. NICARAGUA. *A. Garnier* 110 (holotype F0054841).

#### Type.

INDIA. *Wallich* 1421 (lectotype G00227258, designated by Wood et al. 2015: 20, isolectotypes K-W, G).

#### Description.

Twining perennial herb, stems tomentose, to several metres long. Leaves petiolate, 4–8 × 3–5 cm, ovate-deltoid, often shallowly 3-lobed, cordate with rounded auricles, apex acute, adaxially pubescent, abaxially grey-tomentose; petioles 2–4 cm, grey-tomentose. Inflorescence of compact, axillary, pedunculate cymes; peduncles 3–12 cm, densely woolly-pilose; bracteoles 3–7 mm, linear, caducous; secondary peduncles (if present) 2–3 mm; pedicels 5–17 mm, pilose; sepals somewhat unequal, outer (8–)10–14 mm, accrescent to 16 mm in fruit, ovate-deltoid, acute (or obtuse), pilose, inner sepals 8–12 mm, obtuse, pilose, margins scarious; corolla 4–5.5 cm long, funnel-shaped, pink, sericeous-pubescent, limb 4–5 cm diam. Capsules globose, 8–13 × 11–12 mm, enclosed by sepals, glabrous; seeds 5–6 mm long, pilose.

**Illustration**: O’Donell (1959b: 231) as *Ipomoea riparia*; Deroin (2001: 241); Figure 190B.

#### Distribution.

A pantropical species originally described from India but with every appearance of being native in parts of the New World especially in the basin of the Paraguay-Paraná Rivers. It is a plant with a very distinct ecology, growing at low altitudes beside slow-moving tropical rivers, streams and small lakes but is very scattered in its distribution, being rare or absent from many parts of the neotropics, particularly north of the Isthmus of Panama.

**ARGENTINA. Chaco**: Resistencia, O’Donell 5578 (LIL). **Corrientes**: *T.M. Pedersen* 5557 (C, E, S), 6461 (C, S). **Entre Ríos**: *A. Burkart* 30083 (RB, SI) – requires confirmation. **Misiones**: Posadas, *C. O’Donell* 5601 (LIL); San Ignacio, *H.A. Keller & N.G. Paredes* 7095 (CTES, FCQ).

**PARAGUAY. Alto Paraguay**: Est. Miranda, *F. Mereles* 6824 (FCQ). **Central**: Río Salado on road to Limpio, *J.R.I. Wood et al.* 28141 (FCQ); Lago Ypacaraí, *F. Mereles* 459 (FCQ, MO). **Cordillera**: Ypacaraí, *E. Hassler* 12183 (BM, K, MO, S). **Itapuá**: *A. Pin et al.* 565 (PY); *B. Balansa* 1054 (P). **Presidente Hayes**: Puente Remanso, *K. Ericsson* 582 (MO, PY).

**BRAZIL. Amazonas**: Lago do Carão, *V.F. Kinupp* 1903 (INPA). **Mato Grosso**: *C.A.M. Lindman* 3195 (S); P. Estadual do Xingo, *D. Zappi et al.* 3157 (K, RB); Novo Mundo, *D. Sasaki et al.* 1474 (K). **Mato Grosso do Sul**: Faz. Acurizal, near Corumbá, *G. Schaller* 184 (NY); Cabeceira Grande, Rio Preto, *A.A. Santos & J.B. Pereira* 1806 (CEN). **Paraná**: *K.K. Kita* 304 (MBM). **São Paulo**: *V. Stranghetti* 297 (UEC). Also Acre, Pernambuco and Rio de Janeiro fide Flora do Brasil (2020).

**FRENCH GUIANA.** Savane Matiti, *G. Cremers* 14484a (CAY).

**GUYANA.***Jenman* 5531 (K); *D. Hancock* 50 (K).

**SURINAM.***J. Langouw* 1064 (K); *J. Langouw & J.C. Lindeman* 1430 (K).

**BOLIVIA. Beni**: Cercado, Laguna Suárez, *N. Ritter & M. Ritter* 3346 (BOLV, MO); Vaca Díaz, Riberalta, *J. Solomon* 16736 (LPB, MO). **Cochabamba**: Puerto Villarroel, *R. Chávez de Michel* 3269 (LPB). Pando: Río Negro, *Vargas et al.* 980 (LPB). **Santa Cruz**: Germán Busch, Puerto Suárez, *R. Frey et al.* 494 (MO, USZ); Ñuflo de Chávez, Sam Miguelito, *A. Fuentes* 1586 (LPB, NY, USZ); Velasco, El Refugio, *J.R.I. Wood & H. Huaylla* 20754 (HSB, K, LPB, USZ).

**PERU. Loreto**: Res. Nac. Pacaya-Samiria, *C. Del Cario* 2275 (MO).

**ECUADOR. Guayas**: *L.B. Holm-Nielsen & S. Jeppesen* 95 (AAU. MO, S). **Los Ríos**: *G. Harling* 435 (MO).

**COLOMBIA. Arauca**: *L.E.Forero & J.C. Betancour* 193 (COL, MO). **Chocó**: *H. León* 266 (COL). **Magdalena**: Chiriguana, *C. Allen* 48 (MO).

**VENEZUELA. Delta Amacuro**: Antonio Díaz, *J. Steyermark et al.* 114834 (MO).

**NICARAGUA.** Matagalpa, *P.P. Moreno* 4908 (MO).

**HONDURAS.** Lago Yojoa, *J.M. MacDougal et al.* 3093 (MO).

**MEXICO. Jalisco**: *E.J. Lott et al.* 2867 (MEXU). **Tabasco**: *A. Novelo et al.* 4127 (MO); **Veracruz**: Minatitlán, *M.A. Tenorio Torres* 2 (MEXU).

**TRINIDAD.***Crueger* (?) s.n, [4/10/1849] (K).

#### Note.

This superficially appears to belong to the *Jalapa* radiation (species 1–83) but molecular sequencing shows that it is an unrelated Old World species (Muñoz-Rodríguez et al. 2019). The pilose sepals and grey-tomentose leaves can lead to confusion with *Ipomoea longibarbis* but the corolla is shorter and the bracteoles much smaller. Moreover their habitats are quite different, *Ipomoea rubens* growing by water whereas *I. longibarbis* is a plant of very dry scrub.

• Species 400–417 form a neotropical clade nested within the Old World Clade (OWC). Although several species show obvious similarities to others in the clade, there is no obvious single over-riding morphological feature which characterises the group. It is noteworthy that *Ipomoea obscura* and *I. ochracea* belong to this clade although they are generally considered to be introductions from the Old World to the neotropics.

### 400. Ipomoea lindenii

Taxon classificationPlantaeSolanalesConvolvulaceae

M. Martens & Galeotti, Bull. Acad. Roy. Sci. Bruxelles 12: 264. 1845. (Martens and Galeotti 1845: 264)

Rivea lindenii (M. Martens & Galeotti) Hallier f., Bot. Jahrb. 18: 158. 1894 [pub. 1893]. (Hallier 1893b: 158).
Ipomoea cyanantha Griseb., Fl. Br. West Indian Islands 469. 1864 [pub. 1862]. (Grisebach 1862b: 469. Type. JAMAICA. Mountains of St Andrews, Purdies.n. (lectotype K00612707, designated by Wood and Scotland 2017c: 15).
Ipomoea brevipes Peter, Natürlichen Pflanzenfamilien 4 (3a): 30. 1897 [pub. 1891]. (Peter 1891: 30), nom. illeg., non Ipomoea brevipesChoisy (1845). Type. GUATEMALA. Retaluleu, *K.G. Bernoulli & Cario* 1885 (lectotype GOET005712, designated by Staples et al. 2012: 675).
Ipomoea pandurata Conzatti & L.C. Smith, Fl. Sinóp. Mex. 3: 48. 1895. (Conzatti and Smith 1895: 48), nom. illeg., non Ipomoea pandurata (L.) G. Mey. (1818). Type. MEXICO. Oaxaca, Jayacatlan, *L.C. Smith* 142 (holotype GH00054526).
Ipomoea sabulosa House, Ann. New York Acad. Sci. 18(6): 228. 1908. (House 1908b: 228). Type. Based on Ipomoea pandurata Conzatti & L.C. Smith
Ipomoea plicata Urb. ex House, Ann. New York Acad. Sci. 18(6): 226. 1908. (House 1908b: 226). Type. JAMAICA. Holly Mount. Mt. Diablo, *W. Harris* 8997 (holotype NY00111097, isotypes BM, K, F).
Ipomoea sabulosa var. hirtella House, Ann. New York Acad. Sci. 18(6): 228. 1908 (House 1908b: 228). Type. MEXICO. Chiapas, *E.W. Nelson* 3281 (holotype US00111462, isotype GH).
Ipomoea sabulosa var. mollicella House, Ann. New York Acad. Sci. 18(6): 228. 1908. 1908 (House 1908b: 228). Type. MEXICO. Oaxaca, *A.L. Smith* 640 (holotype GH00054539).
Ipomoea nicoyana House, Ann. New York Acad. Sci. 18: 231. 1908. (House 1908b: 231). Type. COSTA RICA. Nicoya, *A. Tónduz* 13671 (holotype NY00319121, isotypes BM, US).
Ipomoea armentalis L.O. Williams, Fieldiana, Bot. 32: 185.1970. (Williams 1970a: 185). Type. MEXICO. Chiapas, 3 miles south of Agucatenango, *D.E. Breedlove & P.H. Raven* 13435 (lectotype F0054823, designated here).
Ipomoea flavida L.O. Williams Fieldiana, Bot. 32: 190.1970. (Williams 1970a: 190). Type. GUATEMALA. Alta Verapaz, *H. von Türckheim* 3930 (holotype US00111393, isotypes F, M).

#### Type.

MEXICO. Veracruz, *H. Galeotti* 1360 (BR00006973308 lectotype, designated by Wood and Scotland 2017c: 15).

#### Description.

Vigorous twining perennial to 8 m; stems pubescent or glabrous, wiry, woody. Leaves rather shortly petiolate, 2.5–9.5(–16) × 1.5–8.5 cm, ovate, apex finely acuminate, mucronulate, often falcate, base shallowly cordate with rounded auricles, margins often somewhat undulate, abaxially paler, the veins prominent, usually glabrous, sometimes pubescent; petioles 1.7–8.5 cm, conspicuously slender, usually glabrous. Inflorescence of shortly pedunculate axillary cymes, sometimes subumbellate and sometimes developing on small side shoots; peduncles 0–15 mm; bracteoles not seen; pedicels 7–27 mm; sepals slightly unequal, outer 5–15 × (2–)3.5–5 mm, oblong-lanceolate, obtuse, margins scarious, glabrous, inner 10–18 × (2–)6 mm, oblong-ovate, obtuse to rounded, margins scarious; corolla 5–6 cm long, bluish or white to lemon-yellow, fragrant, narrowly funnel-shaped, ventricose above a short basal tube 1–1.5 cm long, glabrous except short hairs on the margins of the lobes, limb c. 4 cm diam. sometimes dark pink. Capsules 12–14 × 8–10 mm, broadly ovoid, glabrous, the style persistent as a 4–6 mm long mucro; seeds 6–7 × 3.5 mm, dark brown with long whitish or brownish hairs on margins.

#### Illustration.

Figure 195.

**Figure 195.**
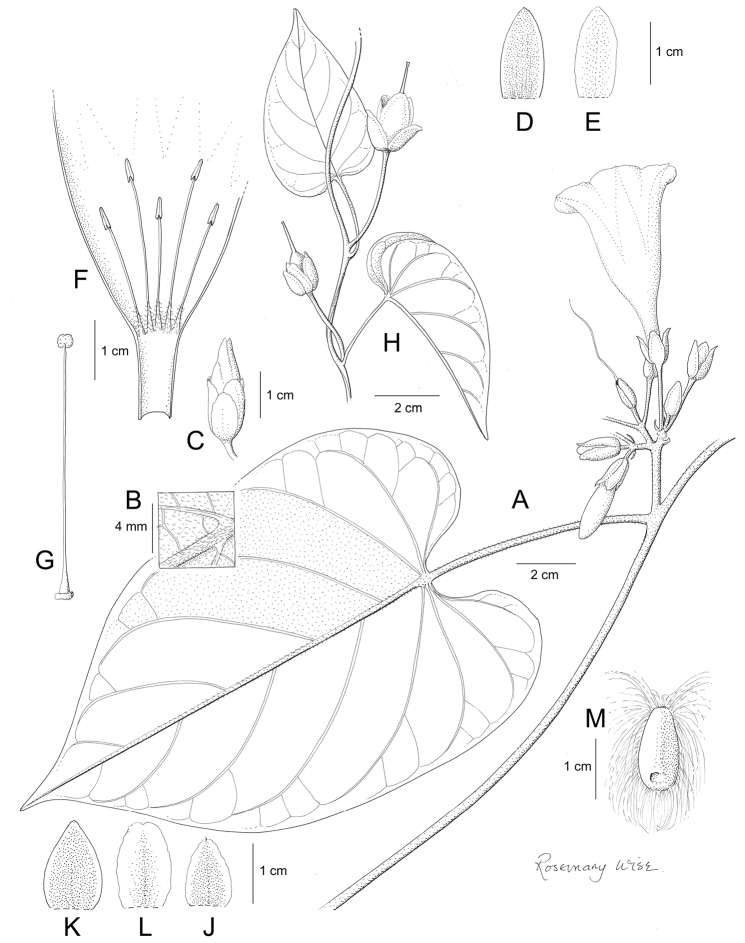
*Ipomoea lindenii*. **A** habit **B** abaxial leaf surface **C** bud **D** outer sepal **E** inner sepal. **F** corolla opened out to show stamens **G** ovary and style. **H** fruiting habit **J** outer sepal **K** middle sepal **L** inner sepal **M** seed. Drawn by Rosemary Wise **A, B, D, E** from *Hammel* 19361; **C, J–L** from *Wilkin* 441; **F, G** from *Hinton* 11561; **H** M from *Wilkin* 430.

#### Distribution.

Widely distributed in moist forest from the northern Andes of Peru, Colombia and Venezuela through Central America to southern Mexico, with isolated populations in Bolivia, Peru and Jamaica. It is found up to about 2000 m but most records are from below 1500 m.

**BOLIVIA. La Paz**: Inquisivi, Com. Khora–Mikilpirhua hacia Lakachaca, *N. Salinas* 3134 (LPB).

**PERU. Amazonas**: Mendoza-Arenal, *H. Van der Werff et al.* 16994 (MO). **Madre de Dios**: Manu, Río Salvación, *P. Nuñez* 6584 (F). **Pasco**: Oxapampa, *G. Castillo et al.* 1028 (MO, USM); ibid., *R. Rojas et al.* 1225 (USM); P.N. Yanachaga-Chemillen, *R. Rojas et al.* 7964 (MO, OXF). **San Martin**: Zepalación, near Moyobamba *G. Klug* 3603 (K, MO, S, US).

**COLOMBIA. Cundinamarca**: Laguna Verde, Zipacón, *L. Uribe* 5049 (COL). **Huila**: Vereda Cachaya, *G. Morales* 019 (COL). **Santander**: Virolín, *R. Torres* 2519 (COL).

**VENEZUELA.** Sine loc., *Moritz* 1243 (BM). **Lara**: *J. Steyermark & Espinoza* 111046 (VEN). **Mérida**: camino a La Carbonera, *F.J. Breteler* 3236 (MO, S, WAG); *G. Morillo* 14450 (OXF). **Portuguesa**: Cerro Córdoba, *J. Steyermark & R. Liesner 126887* (MO). **Trujillo**: Salta La Nevera, *J. Steyermark & Rabe 97195* (US, MO). **Yaracuy**: *J. Steyermark & Wessels-Boer* 100385 (VEN).

**PANAMA.** Isla de Coba, *J. Cuadras et al*. *7978* (K, MA); Chepo, *J.P. Folsom et al*. *6806* (FTG, MO); Canal area, *G. McPherson* 11854 (MO).

**COSTA RICA.** Alajuela, San Ramón, *B. Hammel 19361* (BM); Santa Elena, *P. Wilkin 441* (BM); San Luis, *V. Dryer* 1668 (F).

**NICARAGUA.** Chontales, *W.D. Stevens & O.M. Montiel* 33465 (MO); Jinotega, Reserva El Jaguar, *I. Coronado et al.* 5590 (HULE, MO).

**HONDURAS.** Copán Ruinas, *A. Molina* 24776 (F, MO); El Portillo-El Porvenir, *A & A.R. Molina* 25434 (F).

**EL SALVADOR.** Sonsonate, *R. Villacorta & M. Renderos* 02583 (MO).

**BELIZE.** Orange Walk, *T. Croat* 24979 (MO).

**GUATEMALA.** Baja Verapaz, *H. Von Türckheim 3930* (BM, F).

**MEXICO. Chiapas**: Ocosingo, *D.E. Breedlove* 27798 (MO). **Est. México & Dist. Fed.**: Temascaltepec, *G.B. Hinton 8592* (K). **Guerrero**: Montes de Oca, *G.B. Hinton* 11561 (K). **Michoacán**: *G.B. & J.C. Hinton* 16038 (GBH). **Oaxaca**: *S. Maya* 476 (MO); Pochutla, *A. Nava Zafra & J. Pascual* 188 (IEB). **Querétaro**: San Juan Bautista, *H. Rubio* 76 (IEB); Jalpán, *E. Carranza & E. Pérez 5212* (IEB). **Sinaloa**: *C.D. Johnson* 128-73 (MO). **Tabasco**: Villahermosa-Teapa, *M.A. Magaña* 2306 (IEB). **Veracruz**: *C.A. Purpus* 7586 (S).

**JAMAICA.** St Andrew, Chestervale, *G.R. Proctor* 25615 (BM); Troy, *W. Harris 9034* (BM, K), 12626 (NY); St Elizabeth, Chelsea, *E.T. Robertson* 5650 (BM); St Catherine, Hollymount, *C.D. Adams* 11692 (BM).

#### Notes.

*Ipomoea plicata* was published by House in Ann. New York Acad. Sci. 18(6): 226 not later than 11 May 1908. The same species was published by Urban on 20 May 2008 in Symbolae Antillanae 5: 471.

Generally nearly glabrous but the type of *Ipomoea nicoyana* is noticeably hairy. The sepals are variable in size. The corolla is also very variable in colour ranging from cream to dark blue or combinations of these colours. Despite the variation this species is usually easily recognised by the narrowly ovate or oblong-ovate sepals, very short peduncles and the unusual flower colour.

The collection from Bolivia is a fruiting specimen but appears correctly named. The record from Ecuador (*R. Benoist* 4798 (P) in Austin 1982a) is based on a misidentification. However, the occurrence of *I. lindenii* in Ecuador is expected.

### 401. Ipomoea clavata

Taxon classificationPlantaeSolanalesConvolvulaceae

(G. Don) Ooststr. ex J.F. Macbr., Publ. Field Mus. Nat. Hist. Bot. Ser. 11: 3. 1931. (Macbride 1931: 3)

Calonyction clavatum G. Don, Gen. Hist. 4: 264. 1838. (Don 1838: 264). Type. ECUADOR. Guayaquil, *Ruiz & Pavón*s.n. (holotype ?BM ex Herb. Lambert, n.v.; isotypes MA, OXF).
Convolvulus clavatus Pav. ex Choisy in A.P. de Candolle, Prodr. 9: 346. 1845. (Choisy 1845: 346).
Ipomoea lactescens Benth., Pl. Hartweg. 120. 1839. (Bentham 1839–57: 120). Type. ECUADOR. Guayaquil, *K.T. Hartweg* 676. (holotype K (not barcoded) ex Herb. Bentham, isotypes BM, K, OXF).
Operculina hirsuta Standl., J. Washington Acad. Sci. 14(11): 242. 1924. (Standley 1924: 241). Type. EL SALVADOR. *S. Calderón*, 1338 (holotype US00111353, isotype NY).
Ipomoea contrerasii L.O. Williams, *Fieldiana, Bot.* 32(12): 189. 1970. (Williams 1970a: 189). Type. GUATEMALA. Petén, Arroyo Paxcaman, Uaxactun, *E. Contreras* 3640 (holotype LL00372563, isotypes F, K, MO, S).

#### Type.

Based on *Calonyction clavatum* G. Don

#### Description.

Twining perennial to c. 5 m; stems with long, white, stiff, spreading hairs. Leaves petiolate, 6–12 × 5–10 cm, ovate, sometimes shallowly 3-lobed or with a single lateral lobe, shortly acuminate and mucronate, cordate with rounded auricles, margin often undulate, glabrous, paler beneath, thin in texture, main veins prominent beneath; petioles 4–4.5 cm, pilose. Inflorescence of 1(–2)-flowered, axillary, pedunculate cymes; peduncles 0.4–2.5 cm; bracteoles 2 mm, lanceolate, caducous; pedicels 2–4 cm, darker than peduncle, conspicuously thickened upwards, glabrous; sepals subequal, 23–28 × 10 mm, broadly lanceolate, acuminate, glabrous, margin broad, scarious; corolla 7.5–11 cm long, glabrous, broadly funnel-shaped, the tube white, limb blue, deeply lobed. Capsules ovoid, c. 2 cm long, glabrous; seeds 10–13 × 5 mm, shortly tomentose but with long yellowish marginal hairs.

#### Illustration.

Figures 11F, 196.

**Figure 196.**
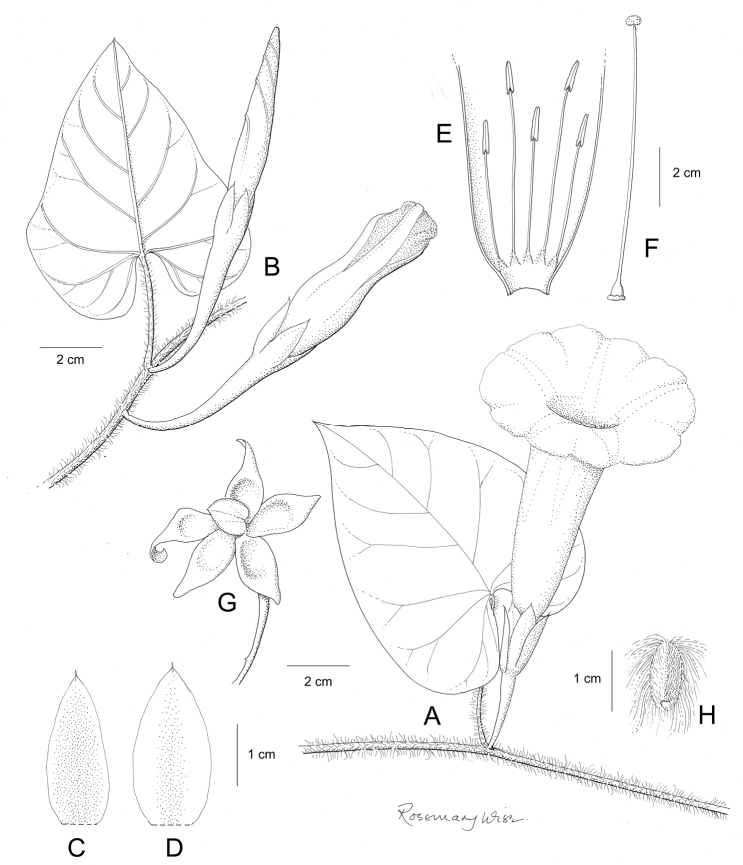
*Ipomoea clavata*. **A** habit with flower **B** habit with buds **C** outer sepal **D** inner sepal **E**corolla opened out to show stamens **F** ovary and style **G** fruiting calyx **H** seed. Drawn by Rosemary Wise **A** from *Hartweg* 671; **B** from photo by Fuentes; **C, D** from *Ruiz & Pavón* s.n.; **E–H** from *Fuentes & Miranda* 10895.

#### Distribution.

Scattered in disturbed bushy places in areas of good rainfall at low altitudes up to just over 1000 m along the Andean chain from northern Bolivia to southern Mexico:

**BOLIVIA. La Paz**: Sud Yungas, Alto Beni, *R. Seidel* 2455 (ARIZ, K, LPB); Madidi, *A. Fuentes & T. Miranda* 10895 (CTES, OXF, LPB, MO, USZ).

**PERU. Amazonas**: Bagua, *A. Gentry et al.* 22840 (MO); ibid., *F. de la Puente* 2443 (CIP). **Cajamarca**: San Ignacio, *J. Campos de la Cruz & O. Díaz* 2373 (MO). **Cusco**: La Convención, Maranura, *L. Valenzuela et al.* 3115 (MO, OXF). **Junín**: Satipo-La Merced, *T. Croat & M. Sizemore* 81993 (MO). **Loreto**: Via Nauta-Iquitos, *C. Díaz & N. Jaramillo* 1269 (MO). **Pasco**: Oxapampa, Pozuzo, *R. Vásquez et al.* 35838 (MO, OXF). **San Martín**: Río Huallaga, Chazuta, *G. Klug* 4076 (BM, K, MO, S); ibid., Juan Jui, *G. Klug* 4308 (BM, K, MO, S).

**ECUADOR. Bolívar**: La Chorrera, *C. Játiva & C. Epling* 017 (MO, S). **El Oro**: *E. Asplund* 15766 (S). **Esmeraldas**: *J. Hudson* 744 (MO, RB). **Guayas**: type of *Ipomoea lactescens*. **Imbabura**: Cotacachi, *C.E. Cerón & C. Reyes* 67397 (Q, QAP). **Manabí**: *H. von Eggers 15458* (K).

**COLOMBIA. Cesar**: Poponte, *C. Allen* 803 (MO). **Cundinamarca**: La Mesa-San Javier, *García Barriga* 12048 (COL); Pacho, *L. Uribe* 1821 (COL). **Valle**: Hac. Hato Viejo, Vijes-Yotoco, *J.E. Ramos* 2752 (MO).

**COSTA RICA.** Guanacaste, Santa Cruz, *B. Hammel & I. Pérez* 24993 (CR, MO).

**NICARAGUA.** Chontales, Río San Juan, *P. Shank & A. Molina* 4595 (F, GH).

**EL SALVADOR.** Ahuachapán, Área Protegida Santa Rita, *J.M. Rosales* 1951 (BM, MO); Santa Ana, San Diego-La Barra, *D. Rodríguez et al.* 2077 (BM).

**BELIZE.** Corozal, *P. Gentle* 545 (F).

**GUATEMALA.** Petén, Lago Petén Itza, *B. Wallnöfer* 9496 (K, MO, W); ibid., Laguna Macanché, *R. Tun Ortíz* 611 (F, MO).

**MEXICO. Campeche**: Calamul, *E. Martínez et al.* 29709 (BM, MEXU); Tenabo, *F. de la Puente* 2939 (CIP). **Guerrero**: Montes de Oca, Vallecitos, *G.B. Hinton 9676* (K, MO), Atoyac, Galeana, *10916* (K, MO), Mina, *11608* (K, MO); Juan R. Escudero, Tirra Colorada, *H. Kruse* 746 (IEB). **Jalisco**: La Huerta, Chamela, *E. Lott & M. Butterwick 1518* (MO). **Michoacán**: El Camalote, *J.C. Soto Nuñez et al.* 7126 (IEB, MEXU). **Oaxaca**: Asunción Ixtaltepec, Cerro Timbón, *A. Saynes & A. Sánchez* 3416 (IEB). **Quintana Roo**: *P. Moreno* 536 (MEXU). **Sinaloa**: Concordia, *A. González* s.n. [1/11/1994] (IEB). **Veracruz**: *C.A. Purpus* 7783 (GH). **Yucatán**: Izamal, *G.F. Gaumer 984* (BM, E, F, K, MO).

#### Note.

An unmistakeable species because of its large blue flowers and pilose stems with very long white hairs.

### 402. Ipomoea cuscoensis

Taxon classificationPlantaeSolanalesConvolvulaceae

J.R.I. Wood & P. Muñoz, Phytokeys 88: 8. 2017. (Wood et al. 2017d: 8)

#### Type.

PERU. Cusco, Anta, Sisal, Limatambo, *C. Vargas* 14325 (holotype CUZ, isotype US).

Twining perennial of unknown height; stems glabrous. Leaves petiolate, 3–6 × 3–6.5 cm, 3–5-lobed, lobes elliptic in outline, apex acuminate to an an obtuse mucronate tip, base shallowly cordate, margin weakly crenate, both surfaces glabrous, abaxially paler with prominent whittish veins; petioles 1.3–3 cm. Inflorescence of pedunculate axillary cymes with up to c. 7 flowers; peduncles 4–6 cm; bracteoles caducous, not seen; pedicels 8–20 mm; calyx narrowly ovoid, sepals somewhat unequal, outer sepals 20–22 × 10 mm, ovate to ovate-elliptic, shortly mucronate, glabrous, margins scarious; inner sepals 15 × 8 mm, ellipsoid, mucronate, the scarious margins broad; corolla c. 6.5 cm long, campanulate, glabrous, deep pink, limb 3–4 cm diam. Capsules and seeds unknown.

#### Illustration.

**Figure 197.**
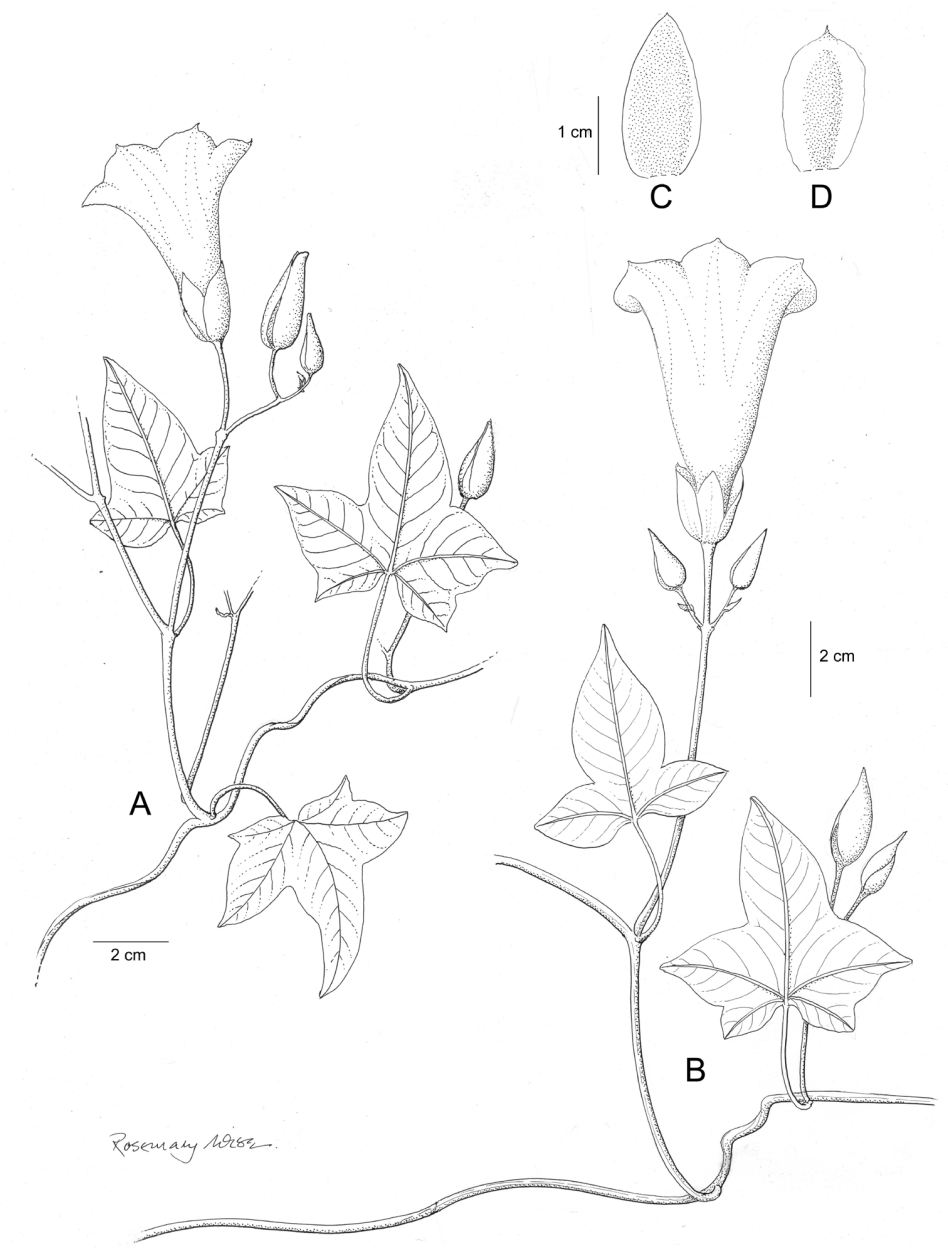
*Ipomoea cuscoensis*. **A** habit **B** habit **C** outer sepal **D** inner sepal. Drawn by Rosemary Wise from *Galiano et al.* 5146.

Figure 197; Wood et al. (2017d: 10).

#### Distribution.

Endemic to dry forest and scrub at 2300–2700 m in southern Peru.

**PERU. Apurimac**: Abancay, Cachora, *C. Vargas* 9083 (CUZ); Grau, *C. Vargas* 5826 (CUZ). **Cusco**: Anta, Mollepata, *W. Galiano et al*. 5146 (MO).

#### Note.

The deep pink or purple corolla is very striking.

### 403. Ipomoea corymbosa

Taxon classificationPlantaeSolanalesConvolvulaceae

(L.) Roth ex Roem. & Schult., Syst. Veg. 4: 232. 1819. (Roemer and Schultes 1819: 232)

Convolvulus corymbosus L., Syst. Nat., ed. 10, 2: 923. 1759. (Linnaeus 1759a: 923). Type. Icon in Burman, Pl. Amer. Plum. t. 89 f. 2, 1756, lectotype, designated by Stearn 1974: 7ff.).
Turbina corymbosa (L.) Raf., Fl. Tel. 4: 81. 1838. (Rafinesque 1838a: 81).
Rivea corymbosa (L.) Hallier f., Bot. Jahrb. Syst. 18(1–2): 157.1894[pub.1893]. (Hallier 1893b: 157).
Legendrea corymbosa (L.) Ooststr., Blumea 5(4): 355. 1943. (Ooststroom 1943: 355).
Ipomoea burmanii Choisy in A.P. de Candolle, Prodr. 9: 350. 1845. (Choisy1845: 350), nom. illeg. superfl. Type. Based on Convolvulus corymbosus L.
Convolvulus domingensis Desr. in Lam. Encycl. 3: 554. 1792 [dated1789]. (Desrousseaux 1792 554). Type. DOMINICAN REPUBLIC or HAITI. Saint Dominigue, Mazure (lectotype P03538776, designated here).
Quamoclit domingensis (Desr.) M. Gómez, Fl. Habana 346. 1899 [pub.1897]. (Gómez de la Maza y Jiménez 1897: 346).
Ipomoea domingensis (Desr.) House, Muhlenbergia 3: 38 1907. (House 1907a: 38).
Convolvulus laevicaulis Willd. ex Roem. & Schult., Syst. Veg. 4: 303. 1819. (Roemer and Schultes 1819: 303). Type. VENEZUELA. Cumana, Humboldt & Bonplands.n. (holotype B-W03705-010; isotype P).
Convolvulus prolifer Willd. ex Roem. & Schult., Syst. Veg. 4: 302. 1819. (Roemer and Schultes 1819: 302). Type. VENEZUELA. Caracas, Humboldt & Bonplands.n. (syntype B-W) & Cult. in Teneriffa, Willdenow s.n. (syntype B-W03698-010).
Convolvulus sidifolius Kunth Nov. Gen. Sp. 3: 99. 1818 [pub.1819]. (Kunth 1819: 99). Type. VENEZUELA. Sucre, Cumana, Humboldt & Bonpland 1226 (lectotype P00670745, designated by Austin and Staples (1991: 273); isolectotype B-W).
Ipomoea sidifolia (Kunth) Sweet, Hort. Brit., ed. 2: 372. 1830. (Sweet 1830: 372).
Convolvulus multiflorus Kunth, Nov. Gen. Sp. 3: 100. 1818 [pub. 1819]. (Kunth 1819: 100). Type. CUBA. La Habana, Humboldt & Bonpland 1306 (holotype P00670746, isotypes P).
Ipomoea cymosa Lindl., Edwards's Bot. Reg. 29: t. 24. 1843. (Lindley 1843: t. 24), nom. illeg., non Ipomoea cymosa (Desr.) Roem. & Schult. (1819). Type. Cultivated plant of unknown origin, apparently not preserved, lectotype t. 24 in Edwards’s Bot. Reg. 29 (1843), designated here.
Ipomoea antillana Millsp., Publ. Field Columb. Mus., Bot. Ser., 2(1): 84–85. 1900. (Millspaugh 1900: 84). Type. Based on Ipomoea cymosa Lindl.
Legendrea mollissima Webb & Berthel., Histoire Naturelle des Îles Canaries 2 (3): 27, t. 137. 1844 (Webb and Berthelot 1844–50: 27). Type. CANARY ISLANDS. Gran Canaria, Despreaux s.n. (holotype FI-Webb).
Rivea corymbosa var. mollissima (Webb & Berthel.) Hallier f., Bot. Jahrb. Syst. 18: 157. 1894 [pub.1893]. (Hallier 1893b: 157).
Legendrea corymbosa var. mollissima (Webb & Berthel.) Ooststr., Blumea 5(4): 355. 1943. (Ooststroom 1943: 355).
Turbina corymbosa forma mollissima (Webb & Berthel.) Stearn, Cuadernos de Botánica Canaria 21: 12. 1974. (Stearn 1974: 12).
Rivea corymbosa var. paniculata Hassl., Repert. Spec. Nov. Regni Veg.9: 151. 1911 (Hassler 1911: 151). Type. PARAGUAY. Amambay. T. Rojas in Hassler 10538 (lectotype G00175662, designated here; isolectotypes A, BM, G, K, NY, P, S).

#### Type.

Based on *Convolvulus corymbosus* L.

#### Description.

Liana climbing to about 7 m over shrubs and small trees; stems woody, usually glabrous. Leaves petiolate, 4–10 × 3–9 cm, ovate, cordate with rounded auricles, narrowed to an obtuse, shortly mucronate apex, glabrous or (rarely) pubescent, abaxially paler; petioles 2–5 cm. Inflorescence of lax compound cymes terminal on the main stem and on lateral branchlets 5–20 cm long; secondary peduncles 1–5 cm; bracteoles c. 2 mm, scale-like; pedicels 7–17 mm; sepals slightly unequal, oblong, obtuse, nearly completely scarious, glabrous, outer 10–11 mm, inner 11–14 mm; corolla 2.5–3 cm long, campanulate, cream with dark centre and yellow midpetaline bands, glabrous, limb c. 1.5–2 cm diam. Capsules narrowly ovoid, 11–14 × 3–4 mm, glabrous, style persistent; seeds 1–2, 4–5 mm diam., subglobose, tomentose.

#### Illustration.

Bosser and Heine (2000: 57) as *Turbina corymbosa*; Figures 9F, 190C.

#### Distribution.

Widespread throughout tropical America and introduced into the Old World but of uncertain status in several countries. It is locally frequent in disturbed bushy places usually near settlements at altitudes below about 1200 m but uncommon in much of South America, apparently absent from the Guianas and many Caribbean Islands, almost so from Paraguay and with few records in Colombia and Brazil. This patchy distribution suggests that it is not native throughout all of its range.

**PARAGUAY. Amambay**: type of Rivea corymbosa var. paniculata).

**BRAZIL. Bahía**: *Pinheiro* 1265 (RB). **Minas Gerais**: *A. Macedo* 2479 (BM, S). **Pará**: *J.M. Pires* 12423 (RB). **Paraná**: *G. Hatschbach* 17082 (MBM). **Rio de Janeiro**: *J.R. Mattos* 145 (RB). **São Paulo**: *M.R. Pietrobom-Silva* 3402 (IPA). Also Mato Grosso, Mato Grosso do Sul & Espirito Santo fide Flora do Brasil (2020).

**BOLIVIA. Beni**: Est. Biológica del Beni, *J. Balderrama* 417 (USZ). **Cochabamba**: Carrasco, Valle de Sajta, *Naessaeny* 67 (LPB). **La Paz**: Madidi, *L. Cayola et al.* 886 (LPB, MA, MO, USZ). **Santa Cruz**: Ibañez, *I. Linneo* 1161 (MO, OXF, USZ); Ñuflo de Chávez, *J.R.I. Wood & D. Soto* 27940 (OXF, LPB, USZ); Warnes, *M. Nee* 45160 (K, LPB, MO, NY, USZ).

**PERU. Cusco**: *C. Vargas* 16293 (CUZ). **Huánuco**: *F. Woytkowski 5394* (MO, P); *E. Asplund* 12397 (S). **Junín**: fide McPherson (1993). **Loreto**: *Ule* 6872 (K). **Pasco**: *J. Flores & M. Chuco* 924 (USM). **Madre de Dios**: Tambopata, *E. Succli & I. Huamantupa* 1967 (M). **Puno**: *P. & P.C. Muñoz* 5152 (CUZ). **San Martin**: *R. Spruce* 3931 (BM, K). **Tumbes**: *A. Gentry & C. Díaz* 58290 (MO). **Ucayali**: *A. Gentry & M. Horna* 29373 (MO).

**ECUADOR. Loja**: Pindal, *F. Vivar & B. Merino* 2124 (LOJA).

**COLOMBIA. Atlántico**: *A. Dugand* 4063 (COL). **Magdalena**: *H.H. Smith* 1623 (BM, COL, K, S).

**VENEZUELA. Bolívar**: *W.A. Díaz* 3110 (MO). **Dist. Fed.**: *T. Croat* 21573 (MO). **Falcón**: *T. Brown* 18 (K). **Lara**: *L. Aristegueita* 4937 (MO). **Miranda**: *H. Pittier* 11429 (K). **Portuguesa**: *H. Pittier* 12034 (MO). **Sucre**: *Humboldt & Bonpland* 1226 (P).

**PANAMA.***J.A. Duke* 15410 (MO).

**COSTA RICA.***Espinoza* 138 (BM, K, MO); *A. Tonduz* 8639 (BM).

**NICARAGUA.***F. Ortíz* 1674 (MO); *A.D. Moore* 2108 (BM).

**HONDURAS.***A. Molina* 25925 (BM).

**EL SALVADOR.***J.M. Tucker* 811 (K, UC); *A. Munro & K. Sidwell* 2784 (BM).

**BELIZE.***W.A. Schipp* 1128 (BM, K, S); *P.H. Gentle* 1838 (K, MICH).

**GUATEMALA.***Bartlett* 321 (S); *R. Tun Ortíz* 805 (BM, F).

**MEXICO. Campeche**: *Soto & Alvarez* 22691 (K, MEXU). **Chiapas**: *G. Aguilar et al.* 417 (BM, MEXU). **Est. México & Dist. Fed.**: *E. Bourgeau* 1265 (K, P, S); Temascaltepec, *G.B. Hinton* 2276 (BM, K, MO). **Guerrero**: *Y. Mexia* 8902 (S). **Hidalgo**: *G. Cruz* 2239 (K). **Jalisco**: *C.G. Pringle* 4549 (BM, MO, S). **Michoacán**: *G.B. Hinton* 13212 (K, S). **Nayarit**: *Y. Mexia* 812 (BM). **Oaxaca**: *E. Pérez-García & B. Reyes* 937 (MO). **Querétaro**: *P. Tenorio & C. Romero* 2286 (K). **Quintana Roo**: *E. Cabrera & H. Álvarez* 1620 (BM). **San Luis Potosí**: *C. Guzmán* 3283 (K). **Sinaloa**: *D.E. Breedlove* 35628 (MO). **Tabasco**: *A. Novelo et al.* 145 (BM, K, MEXU). **Tamaulipas**: Tampico, *E. Palmer* 82 (BM, US). **Veracruz**: *J.I. Calzada* 875 (K, MEXU); *M. Botteri* 557 (BM). **Yucatán**: *G.F. Gaumer* 2052 (BM, S).

**UNITED STATES. Florida**: *H. Moldenke* 428 (S); *D.S. Correll* 47693 (BM, Fairchild); **North Carolina**: *L. Kitching* s.n. [15/9/1906] (BM).

**BERMUDA.** Fide Britton (1918).

**BAHAMAS.***A.E. Wright* 13 (K); *A.H. Curtiss* 10 (BM, P).

**CUBA.***C. Wright* 1655 (P); *Bro. Alain* 9653 (HAC); *C.F. Baker* s.n. [14 Nov. 1904] (HAGB); *H. van Hermann* 304 (BM, NY, P).

**JAMAICA.***Prior 588* (K); *W.R. Robertson* 755 (K); *W. Stearn* 283 (BM).

**HAITI.***E.L. Ekman* H7220 (S); *L.R. Holdridge* 886 (BM).

**DOMINICAN REPUBLIC.***E.L. Ekman* H1111 (K, S); *E.J. Valeur* 282 (S); Barahona, *M. Fuertes* 1416 (P).

**PUERTO RICO.***C.M. Taylor & S. Miller* 10408 (K, MO); *R.J. Wagner* 1085 (BM).

**LESSER ANTILLES. Nevis**: *G.R. Proctor* 19310 (BM). **Antigua**: *H.E. Box* 1202 (BM). **Montserrat**: *R.A. Howard* 19673 (BM). **Guadeloupe**: *A. Duss* 4179 (MO). **Martinique**: fide Powell (1979). **St Lucia**: *Velez* 3310 (K). **Barbados**: *McIntosh* 184a (P).

**TRINIDAD.** Fide Hill and Sandwith (1953). **Tobago**: *W.E.Broadway* 24/2/1913 (BM, P).

#### Typification.

The protologue of *Convolvulus domingensis* simply states that the plant was collected in Sainte Dominique and was kept in the Jussieu herbarium. P03538776 appears to be the only possible specimen that fits these specifications and is here selected as lectotype. P00391965 cannot be part of the material seen by Desrousseaux as it was collected by Poiteau, who first arrived in Hispaniola some years after *Convolvulus domingensis* was published.

#### Note.

A conspicuously woody liana with a campanulate corolla and oblong scarious sepals, the inflorescence often subracemose or corymbose in appearance. The ripe fruit is distinctive as the sepals are persistent, become papery and spread outwards so aiding dispersal by the wind. The seeds have hallucinogenic properties (Stearn 1976). *Ipomoea corymbosa* can be confused with *Ipomoea reticulata* which has similar coloured flowers and grows in similar habitats but the sepals of *I. reticulata* are elliptic, less than 8 mm long.

### 404. Ipomoea sidifolia

Taxon classificationPlantaeSolanalesConvolvulaceae

Schrad., Gött, Gel. Anz. 1821(2): 719. 1821. (Schrader 1821: 719)

Ipomoea tubata Nees, Flora 4: 301. 1821. (Nees ab Esenbeck 1821: 301). Type. BRAZIL. *Prinz von Neuwied* (holotype of unknown whereabouts, isotype GOET 000810).

#### Type.

BRAZIL. *Prinz von Neuwied* (lectotype GOET 000810, designated here).

#### Description.

Liana to 10 m; stems subtomentose, woody. Leaves petiolate, 3–11 × 2.5–8 cm, ovate, shortly acuminate, cordate with broad sinus, adaxially green, tomentellous, abaxially grey-tomentose; petioles 1.5–5 cm, tomentellous. Inflorescence of few-flowered axillary cymes, peduncles 2–5.5 cm, white-tomentellous; bracteoles 5–7 mm, oblong-lanceolate, white-tomentellous, tardily deciduous; secondary peduncles up to 1.5 cm; pedicels (5–)15–45 mm, often long and straight, white-tomentose; outer sepals unequal, outer 10–15 × 6–7 mm, ovate, acute, abruptly narrowed to subtruncate at base, often undulate to fimbriate-margined, white-tomentose, inner sepals 16–17 × 7 mm, broadly oblong, obtuse, tomentellous with broad scarious margins; corolla hypocrateriform, with cylindrical basal tube 3.5–4.5 cm in length and spreading, deep pink, lobes, pilose on the exterior, especially on tube and midpetaline bands, limb 5.5–6 cm diam.; stamens exserted, equal. Capsules 16 × 12 mm conical, shortly rostrate, tomentellous; seeds 8–9 × 5 mm, long-pilose.

#### Illustration.

Figure 198.

**Figure 198.**
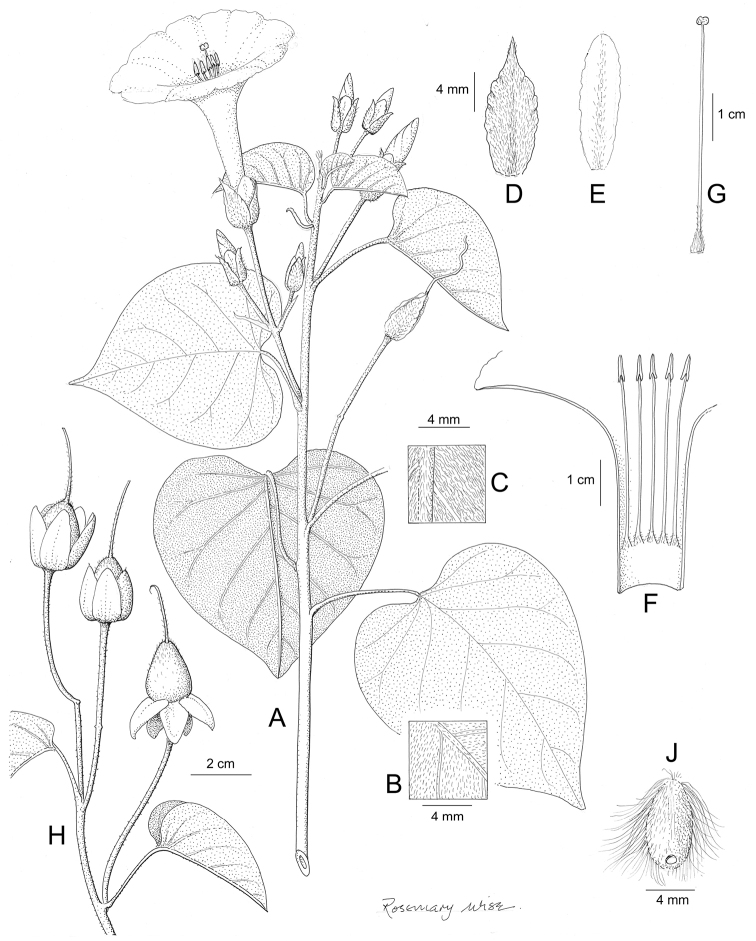
*Ipomoea sidifolia*. **A** habit **B** adaxial leaf surface **C** abaxial leaf surface **D** outer sepal **E** inner sepal **F** corolla opened out to show stamens **G** ovary and style **H** fruiting inflorescence with capsules **J** seed. Drawn by Rosemary Wise **A–E** from *Pohl* 2745; **F–J** from *Zappi & Taylor* 2266.

#### Distribution.

Endemic to the cerrado biome in Brazil, where it is common in Minas Gerais and Goiás.

**BRAZIL. Bahia**: Ilhéus, *T.S. Santos et al.* 3215 (K, RB). **Dist. Fed.**: Bacia do Rio Bartolomeu, *E.P. Heringer* 4501 (IBGE, K); Córrego Forquilha, *B.A.S. Pereira* 261 (IBGE, K); Rio Contagem, *H.S. Irwin et al.* 15703 (E, K, NY). **Goiás**: Santa Cruz, *J.B. Pohl* 2745 (OXF, W); Goiânia, *G. Hatschbach* 36992 (MBM, MO, NY); Huapolis, *A. Macedo* 3282 (MO, S). **Minas Gerais**: Serra do Cipó, *D. Zappi & N. Taylor* 2266 (SPF, K); Uberlandia, *B.C. Vargas* 114 (HUFU); Delfinópolis, Est. Carmen Silvia, *A.C.B. Silva* 374 (RB). **Pernambuco**: Sanharó, *Andrade Lima* 66-4538 (RB); Mun. De Brejo da Madre de Deus, *L.F. Silva et al.* 201 (K, PEUFR). **São Paulo**: Jeriquara, *Mattos & Bicalho* 11610 (SP).

#### Typification.

This species has long been known as *Ipomoea tubata* but the name *Ipomoea sidifolia* was published in the same year and has precedence so should be adopted for this species. Both names were based on the same collection. In order to avoid ambiguity the specimen at GOET is here chosen as lectotype even though it is not annotated either by Schrader or by Nees. It is only extant example of the original material that we are aware of.

#### Note.

A very distinctive species because of its liana habit, hypocrateriform corolla, exserted stamens and tomentellous ovary and capsule. It is the only species we have observed in which the lower half of the style is pubescent.

### 405. Ipomoea daturiflora

Taxon classificationPlantaeSolanalesConvolvulaceae

Meisn. in Martius et al., Fl. Brasil. 7: 273. 1869. (Meisner 1869: 273)

#### Type.

BRAZIL. Rio de Janeiro, Serra Farmarati, 1832, *L. Riedel s.n.* (sheet numbered 119 with collector’s label attached and annotated Ipomoea datureflora [sic], LE, lectotype, designated here).

#### Description.

Perennial twining or trailing herb to 3 m, stems pilose. Leaves petiolate, 8–15 × 10–12, ovate, acute with a prominent mucro up to 6 mm long, base cordate, adaxially pubescent, abaxially paler and densely pubescent; petioles 4–9 cm, pilose. Inflorescence of few-flowered axillary cymes; peduncles1.5–11 cm, pilose; bracteoles 12–20 mm, linear-filiform, deciduous; pedicels 1–6 cm, pilose; sepals slightly unequal, outer 21–34 × 7–8 mm, lanceolate, acuminate, mucronate, ciliate and pilose towards base, inner slightly broader, ovate, mucronate, glabrous, with scarious margins; corolla 8–10 cm long, funnel-shaped, pink, glabrous; limb c. 4 cm diam. Capsules 12 × 15, compressed globose, rostrate; seeds not seen.

#### Distribution.

A Brazilian endemic principally recorded from around Rio de Janeiro in disturbed bushy places.

**BRAZIL. Espirito Santo**: Santa Teresa, Pedra de Onça, *R.C. Forzza* 7538 (RB). **Piauí**: Ipiranga de Piauí, *Queiroz et al. 10195* (OXF, HUEFS). **Rio de Janeiro**: *T. Plowman & de Lima 12898* (F); *A.L. Menescal 118* (RB); *Giordano et al. 2260* (RB); Serra dos Orgãos, *P. Occhioni 8077* (MBM). It is also recorded from Minas Gerais (Flora do Brasil 2020 under construction) although this seems improbable. The record from Piauí is very disjunct, but appears correct.

#### Note.

This species is distinctive because of its very long pedicels and, especially, the elongate sepals.

### 406. Ipomoea chiquitensis

Taxon classificationPlantaeSolanalesConvolvulaceae

J.R.I. Wood & Scotland, Kew Bull. 70 (31): 18. 2015. (Wood et al. 2015: 18)

#### Type.

BOLIVIA. Santa Cruz, Velasco, 6–10 km N de San Rafael en el camino a San Miguel, *J.R.I. Wood & D. Soto* 27388 (holotype USZ; isotypes K, LPB).

#### Description.

Very slender, possibly annual, twining herb reaching no more than 1 m in height, stems glabrous. Leaves petiolate, 2.5–5.5 × 1.5–4.5 cm, ovate, cordate with rounded auricles, becoming truncate upwards, apex acute and minutely mucronate, margin entire, adaxially thinly pilose, abaxially glabrous; petioles, 0.5–3 cm long, diminishing in length upwards, pubescent. Inflorescence of very shortly pedunculate 1–2-flowered cymes from the leaf axils; peduncles 3–7 mm, elongating in fruit to 20 mm, glabrous; bracteoles filiform, 1 mm; pedicels 3–7 mm, pubescent; sepals subequal, 5–6 × 2.5 mm (accrescent to 6.5 mm), ovate, acute terminating in an aristate point, pilose with scattered long multicellular hairs, margins slightly paler, inner sepals slightly shorter and paler, nearly glabrous; corolla c. 2.2 cm long, uniformly pink, glabrous, funnel-shaped, midpetaline bands ending in a small white tooth; Capsules 6 × 3 mm, glabrous, ovoid, rostrate, the style base persistent as a pyramidal point 1.5 mm long.

#### Illustration.

Figure 199.

**Figure 199.**
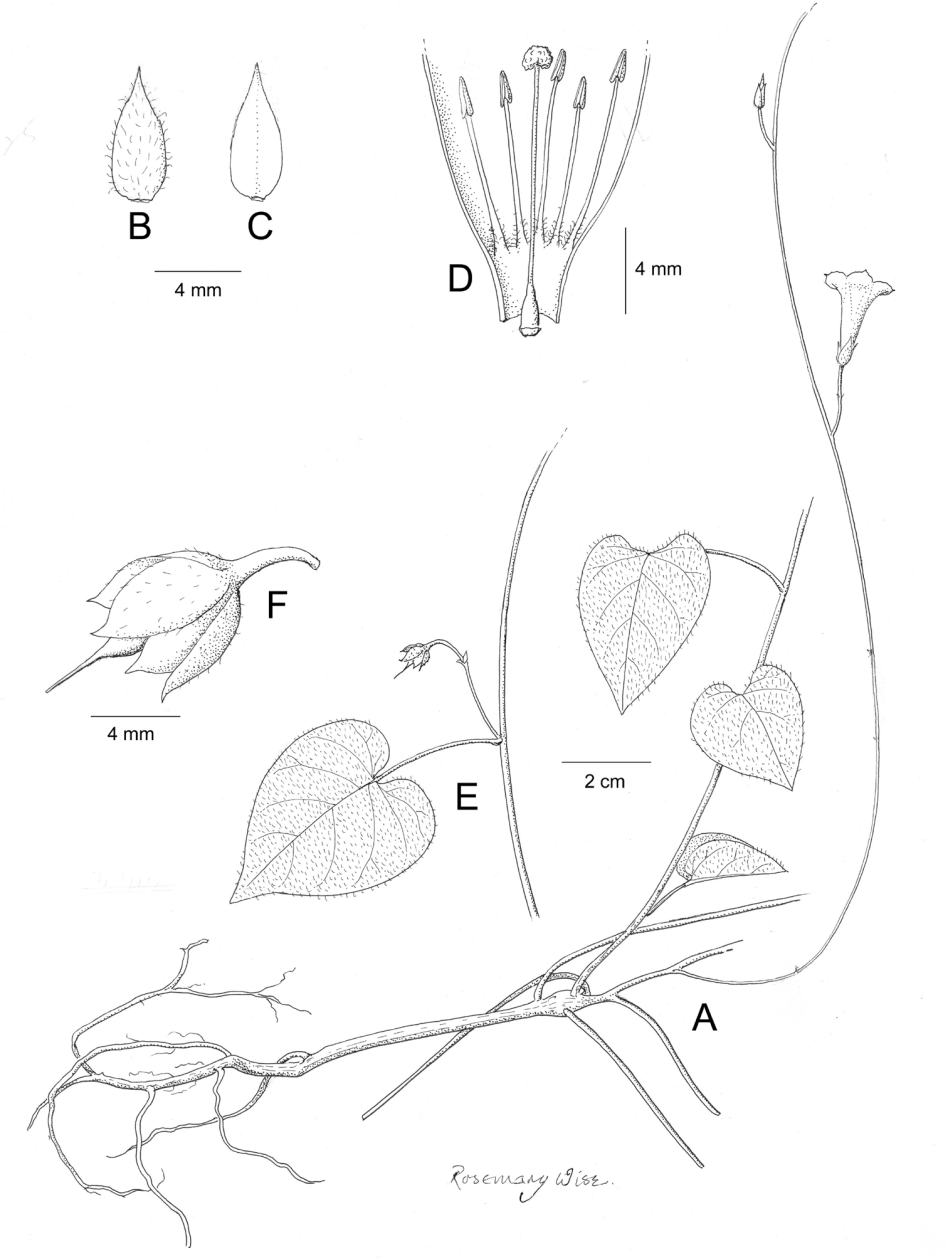
*Ipomoea chiquitensis*. **A** habit **B** outer sepal **C** inner sepal **D** corolla opened up to show stamens, ovary and style **E** shoot with young fruiting inflorescence **F** calyx with young fruit. Drawn by Rosemary Wise from *Wood & Soto* 27388.

#### Distribution.

Granite rock platforms at low altitudes in two very disjunct regions in Brazil and Bolivia.

**BRAZIL. Ceará**: Mun. Piripiri, 4°231352'S, 41°51'21"W, 158 m, *J.A.A.M. Lourenço* 124 (PEUFR). Piauí and Rio Grande do Norte fide Sousa-Santos et al. (2018).

**BOLIVIA. Santa Cruz**: San Rafael de Velasco, type cllection.

#### Note.

*Ipomoea chiquitensis* is a distinctive species readily recognised by the very small, shortly pedunculate flowers, adaxially pilose leaves and small pointed pilose sepals. The small glabrous pink corolla (c. 2.2 cm long) is only matched by that of *I. dumetorum*, *I. deminuta* and some species in the Batatas Clade (A3), such as *I. ramosissima*, but is readily distinguished from all of these by the distinctive ovate, acute sepals.

### 407. Ipomoea melancholica

Taxon classificationPlantaeSolanalesConvolvulaceae

J.R.I. Wood & Buril, Kew Bull. 72 (44): 9. 2017. (Wood et al. 2017b)

#### Type.

BRAZIL. Alagoas, Quebrangulo, REBIO Pedra Talhada, 6 Sept. 2012, *B.S. Amorin, J.L. Costa-Lima, W.M. Pora, V.S. Sampaio, M.A. Chagas* 1658 (holotype JPB, isotype UFP).

#### Description.

Slender twining herb of unknown height; stems pilose. Leaves petiolate, 4.5–10 × 3.5–8.5 cm, ovate and entire, undulate to shallowly 3-lobed, base cordate with rounded auricles, apex shortly acuminate, obtuse and mucronulate, adaxially thinly pubescent, abaxially paler, glabrous; margins ciliolate; petioles 1–8.5 cm pilose. Inflorescence of solitary, pedunculate flowers from the leaf axils; peduncles 3–11 mm; bracteoles 1–2 mm, lanceolate; pedicels 9–13 mm, thickened upwards, slightly winged, often recurved, thinly pubescent; sepals subequal, 7–8 × 1.75 mm, lanceolate, acuminate, pubescent and ciliate; corolla 2.5–3 cm long, pink, narrowly funnel-shaped, apparently glabrous, midpetaline bands ending in small teeth, limb c. 1.5 cm diam.; style globose. Capsules 10 × 7 mm, ovoid, shortly rostrate, glabrous; seeds 4, 5 × 2.5 mm, grey, densely tomentose.

#### Illustration.

Wood et al. (2017b: 7).

#### Distribution.

Endemic to Mata Atlântica in NE Brazil.

**BRAZIL. Alagoas**: *G.A. Gomes-Costa* 166 (JPB, UFP). **Ceará**: Inaçio de Azevedo. *J. Eugenio* 1007 (GH), Serra das Almes, *F.S. Araujo* 1424 (HUEFS).

#### Note.

This species has been interpreted as a form of *Ipomoea acanthocarpa* (Choisy) Aschers & Schweinf. but differs in the solitary flowers and very shortly rostrate capsule. It has also been identified as *I. minutiflora* (M. Martens & Galeotti) House but differs in its larger solitary pink flowers and larger capsules. It might also be thought to be a depauperate species from the Pharbitis Clade such as *I. indica* (Burm.) Merrill but the 4-seeded capsules and small sepals rule that out. The species seems closest to *I. chiquitensis*. Both species have leaves adaxially pubescent but abaxially glabrous and both have similar-sized, acuminate sepals with white margins as well as deflexed fruiting peduncles. However, *I. chiquitensis* always has entire leaves, the stem is glabrous but the leaves and sepals are much more hirsute, and the capsule is much more prominently rostrate. The position of *I. melancholica* here cannot be confirmed as it is only inferred from its morphology.

### 408. Ipomoea crinicalyx

Taxon classificationPlantaeSolanalesConvolvulaceae

S. Moore, Trans. Linn. Soc. London, Bot. 4: 402. 1895. (Moore 1895: 402)

Convolvulus crinicalyx (S. Moore) Kuntze, Rev. Gen. 3(2): 213. 1898. (Kuntze 1898: 213).
Ipomoea seleri Millsp., Bot. Jahrb. Syst. 36, Beibl. 80: 23. 1905 (Millspaugh 1905: 23). Type. MEXICO. Yucatán, Ticul, an Hecken, E & C. Seler 3862 (holotype B?†).

#### Type.

BRAZIL. Mato Grosso, *S. Moore* 953 (holotype BM000953162).

#### Description.

Twining perennial herb, stems glabrous or puberulent. Leaves petiolate, 3–9 × 3–9 cm, broadly ovate, cordate with broad sinus, acuminate, glabrous or shortly adpressed pubescent; petioles 1–6 cm. Inflorescence of pedunculate axillary cymes; peduncles 0.5–8 cm; bracteoles very variable sometimes small, linear, caducous, sometimes large, expanded and leaf-like; secondary peduncles (if present), 2–6 mm; pedicels 8–21 mm; sepals slightly unequal, oblong-ovate, acute, covered in soft spines otherwise glabrous, puberulent, or, frequently, farinose, outer 12–14 × 4–5 mm, inner 14–15 × 5–6 mm, the scarious margins and upper part spineless; corolla 5.5–8 cm long, pink, glabrous outside, limb 4–5 cm, unlobed. Capsules ovoid, glabrous, 14–15 × 12 mm with stout rostrate apex 5 mm long; seeds c. 5 mm long, flattened ellipsoid, minutely tomentellous with long, dense, brownish marginal hairs.

#### Illustration.

**Figure 200.**
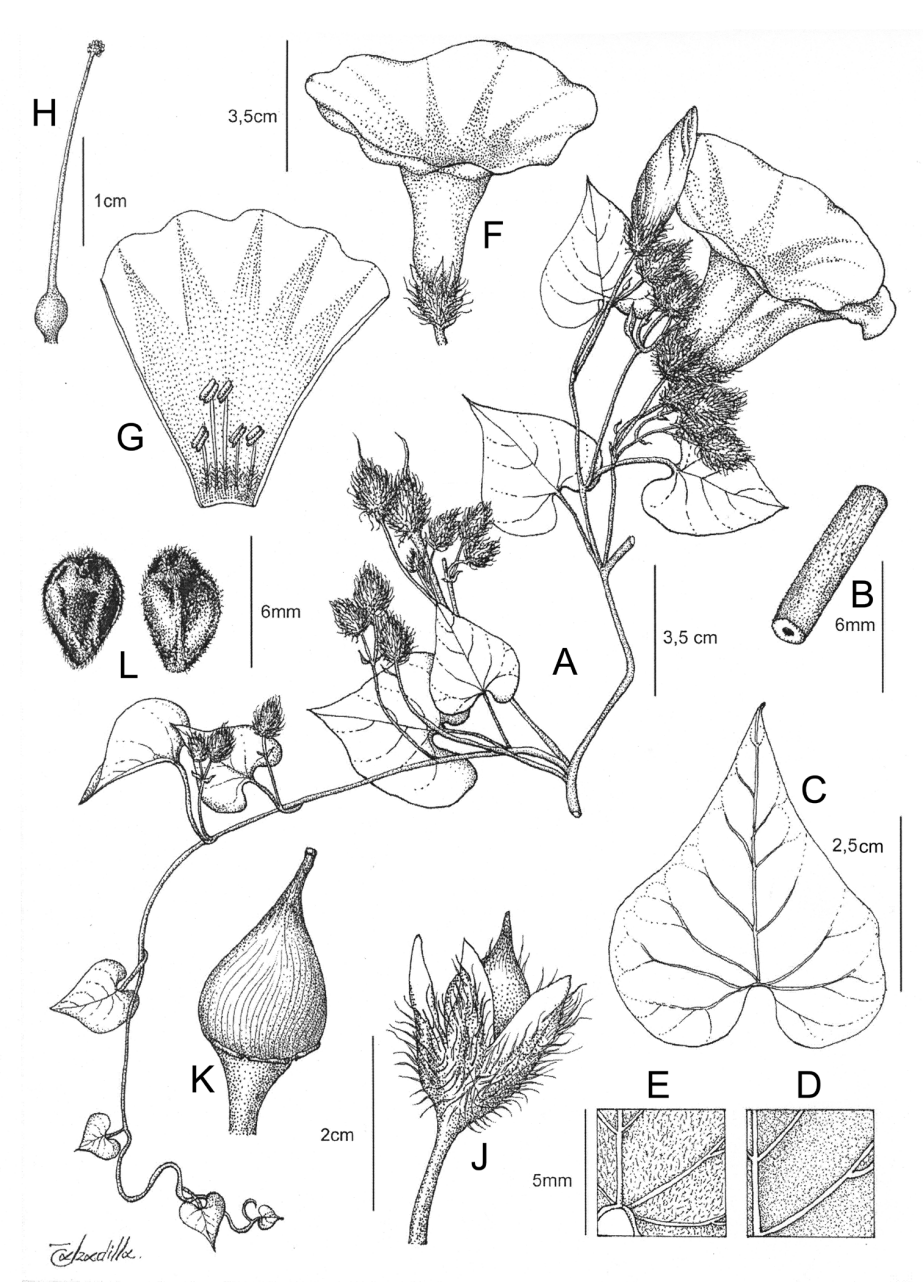
*Ipomoea crinicalyx*. **A** habit **B** stem **C** leaf **D** adaxial leaf surface **E** abaxial leaf surface **F** flower **G** corolla opened out to show stamens **H** ovary and style. **J** calyx and capsule **K** capsule **L** seeds. Drawn by Eliana Calzadilla **A–E** from *Wood & Mamani* 13495; **F–J** from *Wood et al.* 24462; **J–L** from *Fuentes* 2992.

O’Donell (1959b: 140). Figures 2D, 200.

#### Distribution.

A species with an amphitropical distribution being found in Mexico as well as in South America, where it has a typical Chaco distribution. In South America, it is characteristic of Chaco forest and scrub.

**ARGENTINA. Jujuy**: Ledesma, *A. Krapovickas & G. Seijo* 47735 (CTES); *Legname & Cuezzo* 8202 (CTES, LIL). **Salta**: Orán, *O. Morrone et al. 4045* (MO, SI).

**PARAGUAY. Alto Paraguay**: *F. Mereles* 6572 (FCQ).

**BRAZIL. Mato Grosso do Sul**: Corumbá región, *A. Pott 7769* (CPAP, CTES); *P.C. Silva & E.L.M. Assis* 18 (CPAP).

**BOLIVIA. Chuquisaca**: Boeto, below Nuevo Mundo, *J.R.I. Wood et al*. *22336* (K, LPB); Zudañez, El Palmar, *J. Gutiérrez et al.* 2645 (HSB). **Santa Cruz**: Chiquitos, Valle de Tucavaca, *J.R.I. Wood et al*. 24462 (K, LPB, UB, USZ); Cordillera, P.N. Kaa-Iya, *A. Fuentes* 2992 (USZ). **Tarija**: Gran Chaco, Villamontes-Palos Blancos, *J.R.I. Wood et al*. *27606* (OXF, LPB, USZ).

**NICARAGUA.** Managua, *S. Holt* 6204 (HULE, MO).

**BELIZE.** Cayo, San Luis, *J.D. Dwyer et al.* 410 (MO).

**GUATEMALA.** Petén, San José, *B. Wallnöfer* 9502 (MO, W); ibid., Cuxú, *R. Tun Ortíz* 510 (BM, F, S); Santa Elena, *R. Tun Ortíz* 1061 (BM, F); Lake Petén Itzá, *Contreras* 5494 (BM, F).

**MEXICO. Campeche**: Hopelchén, *E. Martínez et al.* 31358 (BM, MEXU); Calkiní, Tunkashe, *E. & H. de Cabrera* 15837 (IEB). **Chiapas**: *A. Espejo* 5868 (MEXU). **Guerrero**: Petatlán, *E. Langlassé* 631 (K). **Jalisco**: *S.H. Bullock* 2000 (K). **Michoacán**: Apatzingán, *Leavenworth* 444 (MO); La Huacana, Sierra Las Cruces, *V.W. Steinmann et al.* 5227 (IEB); Aguila, *E. Carranza & I. Silva* 6658 (IEB). **Quintana Roo**: Chetumal, *E. Cabrera & J.L. Godínez* 4492 (MO); Puerto Morelos, *O. Téllez & E. Cabrera* 1880 (BM, MEXU). **Yucatán**: Sayil, *E.& H. de Cabrera* 10322 (MEXU, MO); Izamal, *G.F. Gaumer* 547 (BM, C, K, S); ibid., *A. Schott* 905 (BM).

#### Note.

The presence of soft spines on the sepals makes this species unmistakeable and only likely to be confused with the following four species. From *I. echinocalyx* it is distinguished by the longer peduncles, shorter sepals, less hairy leaves and pink corolla.

### 409. Ipomoea echinocalyx

Taxon classificationPlantaeSolanalesConvolvulaceae

Meisn. in Martius et al., Fl. Brasil. 7: 223. 1869. (Meisner 1869: 223)

#### Type.

BRAZIL. Minas Gerais, Lagoa Santa, *E. Warming* (holotype BR00005307579).

#### Description.

Twining perennial herb reaching 4 m; stems thinly to densely pubescent. Leaves petiolate, 7–20 × 6–15 cm, ovate, cordate with rounded auricles, shortly acuminate, both surfaces pubescent but abaxially more densely so and paler; petioles 5–18 cm, pubescent. Inflorescence of 1–3-flowered, axillary cymes; peduncles 0–4 mm; bracteoles deltoid, up to 8 mm long; pedicels 15–40 mm, unequal in length, thinly pilose; sepals unequal, outer 15–25 × 3–4 mm, lanceolate or narrowly ovate, acuminate, covered in soft spines, which diminish towards the apex, thinly pilose with white hairs, inner sepals 12–16 mm, lanceolate, terminating in a long mucro, thinly pilose but nearly spineless, margins scarious; corolla c. 7 cm long, funnel-shaped, cream or white, glabrous outside, limb slightly lobed, c. 5 cm diam. Capsules and seeds unknown.

#### Illustration.

Figure 190F.

#### Distribution.

Central Brazil and Bolivia, apparently infrequent in both countries.

**BRAZIL. Minas Gerais**: Viçosa, *Y. Mexia 4428* (F, K, MO, NY, S); São Pedro do Suaçuí, *G. Davidse et al. 11483* (MO).

**BOLIVIA. La Paz**: Caranavi-Alto Beni, *J.R.I. Wood & T. F. Daniel 18388* (HSB, K, LPB); Sud Yungas, *G. Quintana et al.* 1124 (LPB). **Santa Cruz**: Amboró Park, Río San Rafael, *I. G. Vargas et al. 2132* (OXF, MO, NY).

#### Note.

Obviously related to *Ipomoea crinicalyx* but distinguished by the near absence of peduncles, much longer outer sepals and white or cream flowers. Additionally *I. echinocalyx* is a much more hirsute plant with fewer flowers in each cyme.

### 410. Ipomoea silvicola

Taxon classificationPlantaeSolanalesConvolvulaceae

House, Bot. Gaz 43: 411. 1907. (House 1907b: 411)

#### Type.

GUATEMALA. Santa Rosa, Río de Las Cañas, *Heyde & Lux* in *Donnell Smith* 4022 (holotype US00111471, isotypes BM, GH, K, NY).

#### Description.

Twining perennial herb, stems glabrous or puberulent. Leaves petiolate, 3–12 × 3–11 cm, broadly ovate, cordate with broad sinus, acuminate, adaxially glabrous or shortly adpressed pilose, abaxially densely adpressed pilose; petioles 1–8 cm. Inflorescence of pedunculate 2-flowered cymes, borne on short branchlets 0.5–1.5 cm long with reduced leaves; peduncles 0–4 mm, tomentose; bracteoles 5–6 mm, filiform, caducous; secondary peduncles (if present), 2–6 mm; pedicels 20–40 mm; sepals unequal, outer 30–35 × 4–5 mmlanceolate, acuminate, covered in soft spines but apically spineless, pilose throughout with white hairs, inner 20–23 × 5–6 mm, margins broad, scarious, the spines restricted to the midrib area; corolla 7–8 cm long, pink, glabrous outside, limb 4–5 cm, unlobed. Capsules and seeds not seen.

#### Distribution.

Woodland borders at around 1000–1500 m in Central America, apparently common in Honduras and Guatemala.

**HONDURAS.** Ocotepeque, *A. Molina* 22264 (F, MO); ibid., 22151 (BM).

**GUATEMALA.** Capertillo, Valle del Fuego, *O. Salvin* (K); Sacatepéquez, *T. Croat* 41947 (MO).

**MEXICO. Chiapas**: Solusuchiapa, *D.E. Breedlove* 19938 (DUKE, MO); Yajalón, Los Pinos, A. Shilom Ton 4941 (MO). **Oaxaca**: Santa María Chimalapa, *H. Hernández* 554 (MEXU, MO); Totontepec, *J. Rivera Reyes* 1303 (MEXU, MO). **Veracruz**: fide McDonald (1994). **Yucatán**: *F.C. Cabrera* 1413 (MO).

#### Notes.

The plate accompanying the protologue is incorrect and is of *Ipomoea lozanii*. The correct plate is Figure 4, labelled *Ipomoea collina*.

Very similar to *Ipomoea echinocalyx* but differing in the more densely pubescent to subtomentose leaves, especially the whitish abaxial surface. Most distinct are the long, lanceolate finely acuminate outer sepals which can reach 35 mm in length and which are naked of spines in the upper half but are pilose throughout. The cymes are usually 2-flowered, borne on short branchlets 0.5–1.5 cm long with reduced leaves, the pilose pedicels 2–4 cm long.

### 411. Ipomoea altoamazonica

Taxon classificationPlantaeSolanalesConvolvulaceae

J.R.I. Wood & Scotland, Kew. Bull. 72(10): 4. 2017. (Wood and Scotland 2017b: 4)

#### Type.

PERU. Cusco, Paucartambo, Chontachaca a Pillahuata, 700 m, *P. Nuñez 8087* (holotype MO3518513, isotype CUZ19924).

#### Description.

Twining perennial herb 1–2 m high, growing over shrubs; stems pilose. Leaves petiolate, 7–11 × 7–9.5 cm, 3–lobed to half way or slightly less, base cordate with rounded auricles, lobes ovate, apex shortly acuminate and mucronate, both surfaces densely pubescent with somewhat asperous long hairs; petioles 4–12 cm, pilose. Inflorescence of up to 5-flowered axillary cymes; peduncles 1.3–5.3 cm, pilose; bracteoles 3–11 × 0.5–1 mm, filiform to linear, pilose; secondary peduncles (if present) 8–10 mm; pedicels 22–33 mm, pilose; sepals unequal, outer 14–17 × 5–6 mm, oblong-ovate, obtuse, abaxially covered in scattered long white hairs mixed with soft spines, both 3–4 mm long, inner sepals 11–13 × 4–5 mm, ovate, mucronate, glabrous and spineless, margins scarious; corolla c. 5 cm long, funnel-shaped, white, glabrous; limb c. 4 cm diam. Capsules and seeds not seen.

#### Illustration.

Figure 201.

**Figure 201.**
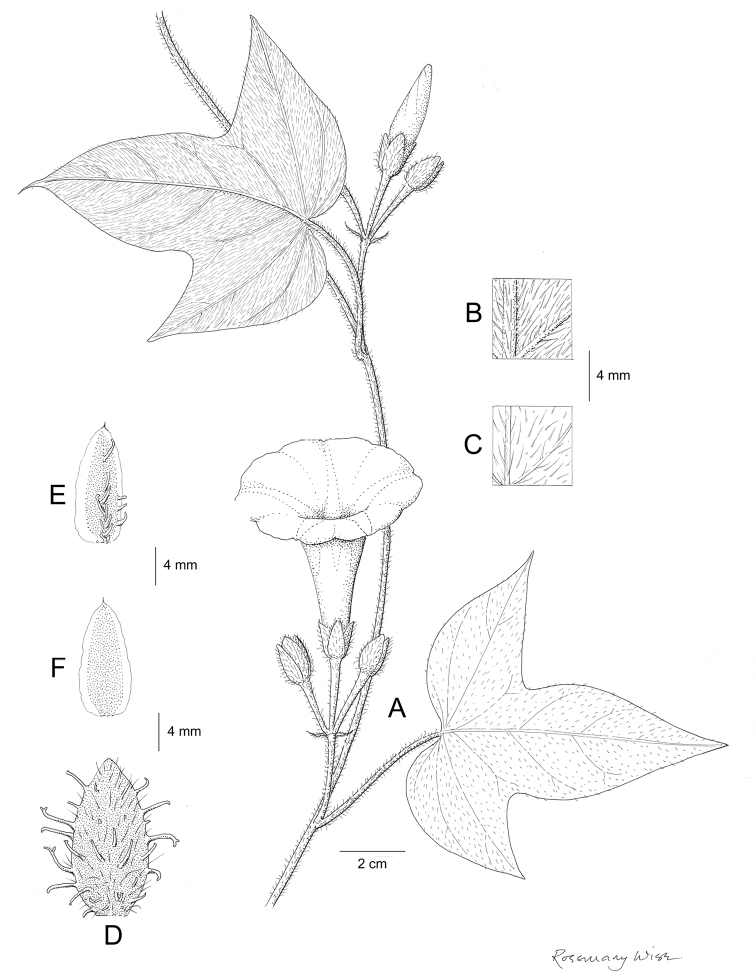
*Ipomoea altoamazonica*. **A** habit **B** adaxial leaf surface **C** abaxial leaf surface **D** outer sepal **E** middle sepal **F** inner sepal. Drawn by Rosemary Wise **A** from *D.C. Daly et al*. 10707; **B, C** from *D.C. Daly et al*. 15012; **D–F** from *Percy Nuñez* 887.

#### Distribution.

A plant of lowland forest areas, endemic to the upper Amazon watershed on the borders of Peru and Brazil.

**BRAZIL. Acre**: Marechal Thaumaturgo, basin of Rio Jurúa, *D.C. Daly et al.* 10707 (ARIZ). **PERU. Cusco**: Convención, Echarate, *P. Nuñez et al.* 19679 (CUZ, ?US). **Huancavelica**: Quintobamba, *O. Tovar* 4143 (USM). **Ucayali**: *J. Schunke V. & J.G. Graham* 15012 (ARIZ).

#### Note.

Clearly part of a complex of species with *Ipomoea crinicalyx*, *I. silvicola* and *I. echinocalyx* but is immediately distinguished from these by the 3-lobed leaves. Additionally the dense pilose indumentum and white flowers distinguish it from *I. crinicalyx*, and the pedunculate cymes from *I. silvicola* and *I. echinocalyx*.

### 412. Ipomoea ochracea

Taxon classificationPlantaeSolanalesConvolvulaceae

(Lindl.) G. Don, Gen. Hist. 4: 270. 1838. (Don 1838: 270)

Convolvulus ochraceus Lindl., Bot. Reg. 13, t. 1060. 1827. (Lindley 1827a: t. 1060). Type. A cultivated plant grown from seed collected by Murray in Ghana (holotype CGE00014).
Convolvulus trichocalyx Schum. & Thonn., Beskr. Guin. Pl. 91. 1827 (Schumacher and Thonning 1827: 91). Type. “GUINEA”, Iserts.n. (syntype C) & *Thonning* 6 (syntypes C, P-JU).
Ipomoea trichocalyx (Schum. & Thonn.) G. Don, Gen. Hist. 4: 275. 1838. (Don 1838: 275).
Ipomoea afra Choisy in A.P. de Candolle, Prodr. 9: 380. 1845. (Choisy 1845: 380). Type. “GUINEA”, specimen sent by Vahl (holotype P-JU).
Ipomoea kentrocarpa A. Rich., Tent. Fl. Abyss. 2: 70. 1851. (Richard 1851: 70). Type. ETHIOPIA. Near Dochli, *Schimper* 1420 (isotypes BM, P, S, TUB).
Ipomoea stocksii C.B. Clarke, Fl. Brit. India 4: 207. 1883. (Clarke 1883: 207), nom. illeg., non Ipomoea stocksii C.B. Clarke 1883: 204. Type. INDIA. Malabar and Concan, *Stocks*s.n. (lectotype K000830816, designated here).
Ipomoea clarkei Hook. f., Fl. Brit. Ind. 4: 734. 1885. (J.D. Hooker 1885: 734). Type. Based on Ipomoea stocksii C.B. Clarke (1883: 207).
Ipomoea ophthalmantha Hallier f., Jahrb. Syst. 18: 141.1894 [pub.1893]. (Hallier 1893b: 141). Type. TANZANIA, Tabora District, Boehm 253 (holotype B†).
Ipomoea curtissii House, Ann. New York Acad. Sci, 18: 257. 1908. (House 1908b: 257). Type. CUBA. *A.H. Curtiss* 562 (holotype NY n.v., isotypes BM, F, GH, L, M, US).
Ipomoea ochracea var. curtissii (House) Stearn, Proc. Linn. Soc. London 170: 145. 1959. (Stearn 1959: 145).

#### Type.

Based on *Convolvulus ochraceus* Lindl.

#### Description.

Twining or trailing perennial herb; stems rather slender, pubescent but usually glabrescent, up to 2 m long. Leaves petiolate, 2–7 × 1.3–6 cm, ovate, acute or shortly acuminate, base cordate with rounded auricles, adaxially glabrous, abaxially paler, glabrous or shortly pubescent on the veins; petioles 0.7–3.2 cm, glabrous or pubescent. Inflorescence of few-flowered, shortly pedunculate axillary cymes; peduncles 1–4 cm, glabrous, pubescent or thinly pilose; bracteoles 1–1.5 mm, lanceolate; secondary peduncles 0.5–1.5 cm; pedicels 7–22 mm, often bent or recurved, glabrous or, less commonly, pubescent or pilose; sepals 5–6 × 2–3 mm, glabrous, often wrinkled/muricate, margins scarious, usually glabrous but occasionally pilose with long white trichomes, slightly unequal, outer ovate, acute to shortly acuminate or mucronate, inner ovate-elliptic obtuse, occasionally mucronate; corolla 3–4 cm long, pale yellow with purple base to the inside of the tube, narrowly funnel-shaped, glabrous, limb often weakly lobed, 3–4 cm diam. Capsules 10 × 7 mm, ovoid, glabrous; seeds 4 × 2.5 mm, minutely tomentellous, sometimes glabrous (var. curtissii).

#### Illustration.

**Figure 202.**
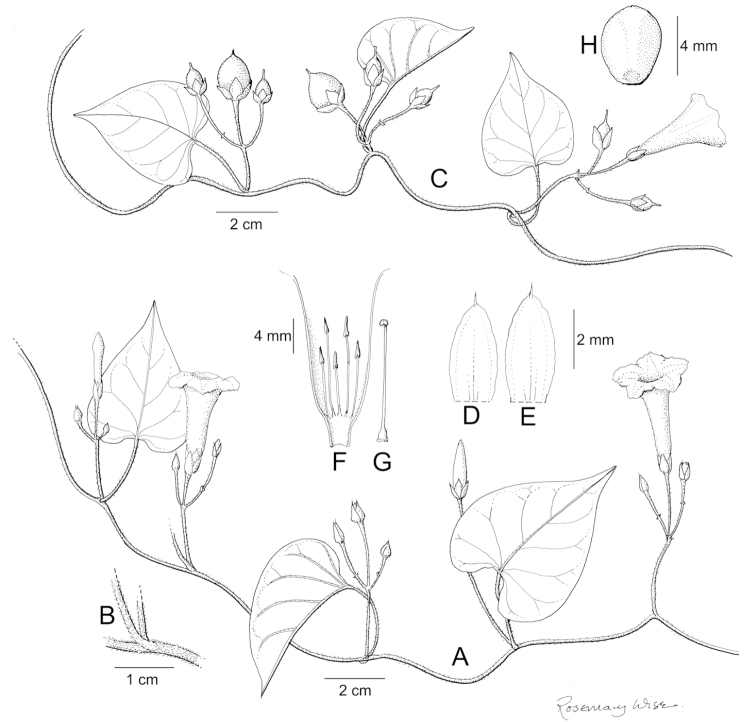
*Ipomoea ochracea*. **A** habit **B** stem **C** habit (fruiting) **D** outer sepal **E** inner sepal **F** corolla opened out to show stamens **G** ovary and style. **H** seed. Drawn by Rosemary Wise **A** from *Robertson* 7646; **B–H** from *Stearn* 163.

Acevedo-Rodríguez (2005: 174); Bosser and Heine (2000: 55); Figures 190D, 202.

#### Distribution.

Generally thought to be an African species introduced to the Caribbean region and locally common in disturbed places, especially in Cuba and Jamaica.

**BRAZIL.***A.F.M. Glaziou* 4890 (MO, S, US).

**VENEZUELA. Dist. Fed.**: *A. Castillo* 1471 (MO).

**CUBA.***A.H. Curtiss* 562 (HAC, K), *Bro. Alain & López Figuieras* 7036 (HAC, HAGB); *E.K. Ekman* 18200 (BM, S).

**JAMAICA.***W. Harris* 12319 (K, NY, S), *W. Robertson* 763b, 764b (BM); St Andrew, *W. Stearn* 39 (BM); St Catherine, *W. Stearn* 163 (BM).

**PUERTO RICO.***J.I. Otero* 478 (MO); *P. Acevedo-Rodríguez* s.n. [11/1/1996] (K, US).

**LESSER ANTILLES. U.S. Virgin Islands**: St Croix: *V.W. Steinmann* 2252 (BM, IEB); St John: *P. Acevedo-Rodríquez* 3096 (MO). **St. Lucia**: *R. Graveson* 320 (MO).

**HAWAII.***F.R. Fosberg* 57420 (BM, US).

#### Notes.

Plants from Cuba and Jamaica with glabrous seeds have been treated as var. curtissii (House) Stearn, but this variation has also been reported from the Old World tropics where it is generally unrecognised.

*Ipomoea ochracea* and *I. obscura* differ in nothing more than the size of their corolla and this makes the interpretation of *Ipomoea clarkei* somewhat difficult. The lectotype of *I. clarkei* has a few corollas in poor condition about 25–28 mm in length, thus essentially intermediate between *I. ochracea* and *I. obscura*, although larger than generally in *I. obscura*. A collection by P.S. Kanitkar from Junnar, Pune at K named *I. clarkei* is certainly *I. ochracea* and it seems best to treat *I. clarkei* as a synonym of *I. ochracea*. Clarke described the seeds as glabrous but annotated the lectotype with a note that the mature seeds were puberulous. There are no seeds attached to the specimen today.

### 413. Ipomoea obscura

Taxon classificationPlantaeSolanalesConvolvulaceae

(L.) Ker-Gawl., Bot. Reg. 3: t. 239. 1817. (Ker-Gawler 1817: t. 239)

Convolvulus obscurus L., Sp. Pl., ed. 2, 2: 220. 1762. (Linnaeus 1762: 220). Type. Icon in Dillenius, Hort. Eltham. 1: 99, t. 83, f. 95 (1832), designated by Meeuse (1958: 746).
Ipomoea curassavica All., Auct. Syn. 10. 1773. (Allioni 1773: 10). Type. Cultivated plant grown from seed collected in Curaçao (holotype TO).
Ipomoea luteola R. Br., Prodr. 485. 1810. (Brown, R 1810: 485). Type. AUSTRALIA. Queensland, Keppell Bay, *R.Brown* 2744 (lectotype BM001040635, designated here).
Ipomoea insuavis Blume Cat. Gen. Buitenzorg 50. 1823. (Blume 1823: 50). Type. INDONESIA. Java, Buitenzorg (no type cited).
Ipomoea fragilis Choisy in A.P. de Candolle, Prodr. 9: 372. 1845. (Choisy 1845: 372). Type. SOUTH AFRICA. Cape, Maadji Mountain, *W.J. Burchell* 2362 (lectotype G, designated by Meeuse and Welman (2000: 99), not seen, isolectotypes GH, K).
Ipomoea obscura var. fragilis (Choisy) Meeuse, in Dyer, Fl. Pl. Afr. 31: pl. 1222. (Meeuse 1956: pl.1222).
Ipomoea acutiflora A. Rich., Tent. Fl. Abyss. 2: 7. 1851. (Richard 1851: 7). Type. ETHIOPIA. Choa, *Quartin Dillon & Petit*s.n. (syntypes P).
Ipomoea longipes Engl., Bot. Jahrb. Syst. 10(3): 246. 1888. (Engler 1888: 246), nom. illeg., non Ipomoea longipesGarcke (1849). Type. SOUTH AFRICA. Griqualand West, *R. Marloth* 981 (holotype B†, isotype K000097277).
Ipomoea inconspicua Baker, Bull. Misc. Inform. Kew 1894 (86): 71. (Baker 1894: 71). Type. MALAWI. Nakulambe, *Buchanan 1881* (holotype K000097201).
Ipomoea saltiana Rendle, J. Bot. 32: 178. 1894. (Rendle 1894: 178). Type. ETHIOPIA, sine loc., Salt s.n. (holotype BM000930428).
Ipomoea demissa Hallier f., Jahrb. Syst. 18: 129 1893[pub. 1894]. (Hallier 1893b: 129). Type. TANZANIA, Tabora District, Boehm 83 (B†).
Ipomoea obscura var. demissa (Hallier f.) Verdc., Kew Bull. 33: 165. 1978. (Verdcourt 1978: 165).
Ipomoea fragilis var. pubescens Hallier f., Bull. Herb. Boiss.7: 51. 1899. (Hallier 1899a: 51). Type. Based on Ipomoea longipes Engl. and I. inconspicua Baker
Ipomoea obscura var. indica Hallier f., Bot. Jahrb. 28: 39. 1899. (Hallier 1899e: 39), nom. illeg., autonymic var.
Ipomoea obscura var. abyssinica Hallier f., Bot. Jahrb. 28: 39. 1899. (Hallier 1899e: 39). Type. ETHIOPIA. Gandia, *Schimper 801* (holotype B†, isotypes BR, G, K, S, W).
Ipomoea obscura var. sagittifolia Verdc., Kew Bull. 13: 210. 1958. (Verdcourt 1958: 210). Type. TANZANIA. Kahama District, Morgan 10 (holotype BM).

#### Type.

Based on *Convolvulus obscurus* L.

#### Description.

Twining or trailing perennial herb; stems rather slender, pilose or glabrescent, up to 1.2 m long. Leaves petiolate, 2.5–9 × 0.5–7.5 cm, ovate, shortly acuminate, base cordate with rounded auricles, rarely sagittate (var. sagittifolia), glabrous or shortly pubescent on both surfaces; petioles 1–8 cm, glabrous or pubescent. Inflorescence of few-flowered, shortly pedunculate axillary cymes; peduncles 3.5–4 cm, glabrous, pubescent or thinly pilose; bracteoles 1–1.5 mm, lanceolate; secondary peduncles 0.5–1.5 cm; pedicels 10–20 mm, often bent or recurved, glabrous or, less commonly, pubescent or pilose; sepals 4–8 × 2–4 mm, glabrous, often wrinkled or muricate, margins scarious, usually glabrous but occasionally pilose with long white trichomes, slightly unequal, outer ovate, acute to shortly acuminate or mucronate, inner ovate-elliptic obtuse, occasionally mucronate; corolla 1.5–2.5 cm long, white, yellow or orange with purple base to the inside of the tube, narrowly funnel-shaped, glabrous, limb often weakly lobed, 3–4 cm diam. Capsules 18 × 12 mm, globose, glabrous; seeds 4–5 × 2.5–3.5 mm, minutely tomentellous.

#### Illustration.

Bosser and Heine (2000: 55); Deroin (2001: 223).

#### Distribution.

Generally thought to be an African species introduced into the Caribbean region.

**JAMAICA.***W. Stearn* 164 (S).

**DOMINICAN REPUBLIC.***H.A. Allard* 13214 (S).

**LESSER ANTILLES. Antigua**: *H.E. Box* 1308 (BM, K). **Barbados**: *A.McIntosh* 351 (K). **Guadeloupe**: *Raynal-Roques & Jérémie* 21118 (K, P); Marie Galante fide Powell (1979). **Dominica**: *C. Whitefoord* 5505 (BM).

**HAWAII.** Kauai, *U.J. Faurie* 1042 (BM); *Judd et al.* s.n. [25/9/1937] (K).

#### Notes.

This hardly differs from *Ipomoea ochracea* except in the smaller dimensions of its corolla. It is, however, much more widespread in the Old World Tropics than *Ipomoea ochracea*.

Ipomoea obscura var. fragilis differs from the type in the concolorous pale yellow colour of the corolla. It is southern African in distribution.

### 414. Ipomoea pedicellaris

Taxon classificationPlantaeSolanalesConvolvulaceae

Benth., Bot. Voy. Sulphur 135. 1844 [pub. 1845]. (Bentham 1845: 135)

Ipomoea grayi Rose, Contrib. U.S. Natl, Herb. 1(4): 107. 1891. (Rose 1891: 107). Type. MEXICO. Chihuahua, *E. Palmer* 710 (lectotype US00111395, partially designated by Austin (1997: 151) and redesignated here; isolectotypes GH, K, NY).
Ipomoea saxorum Standl. & Steyerm., Publ. Field Mus. Nat. Hist., Bot. Ser. 23(2): 81. 1944. (Standley and Steyermark 1944: 81). Type. GUATEMALA. Chiquimula, gorge of Río Chiquimula, between Santa Bárbara and Petapilla, *J.A. Steyermark* 30254 (holotype F0054895).
Ipomoea breedlovei L.O. Williams, Fieldiana, Bot. 32(12): 188. 1970. (Williams 1970a: 188). Type. MEXICO. Chiapas, 9 km N of Tuxtla Gutiérrez, *D.E. Breedlove & P. Raven* 13871 (holotype F0054629, isotypes CAS, F).

#### Type.

HONDURAS. Valle, Gulf of Fonseca, *Sinclair* s.n. (lectotype K000612726, partially designated by C. Nelson, 1996: 58 and redesignated here).

#### Description.

Twining herb or liana to 6 m, stems glabrous or pubescent. Leaves petiolate, 2.5–10.5 × 2–9.5 cm, ovate, shortly acuminate, cordate with rounded auricles (rarely 3-lobed), glabrous or pubescent, abaxially paler; petioles 3–7.5 cm. Inflorescence of subumbellate, branched, usually many-flowered axillary cymes, peduncles 2.5–6 cm, often stout; bracteoles caducous; secondary, tertiary and quaternary peduncles often present, always short, 0.5–1.5 cm; pedicels 15–45 mm, often long; sepals unequal, glabrous, outer 5–8 × 4 mm, oblong-ovate, obtuse or acute, often muricate or midvein forming a narrow wing near base, inner 7–10 mm, ovate, obtuse or rounded, scarious upwards; corolla 6–8 cm long, broadly funnel-shaped, red to pale pink, pubescent at the apex of the midpetaline bands; limb wide, 6–7 cm diam. Capsules 15 × 12–13 mm, ovoid, glabrous; seeds 7 mm long, minutely pubescent.

#### Illustration.

Figure 203.

**Figure 203.**
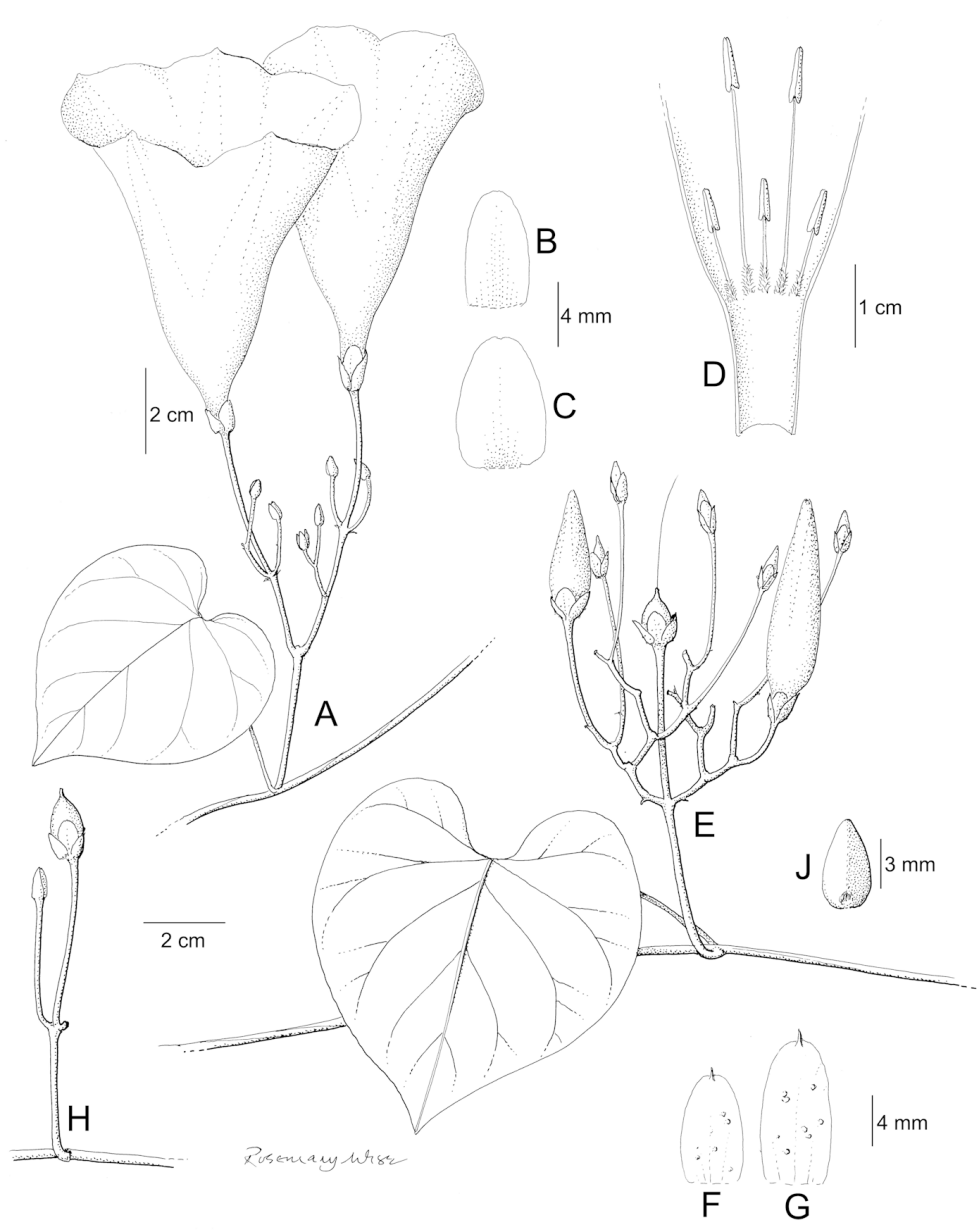
*Ipomoea pedicellaris*. **A** habit at anthesis **B** outer sepal **C** inner sepal **D** corolla opened out to show stamens **E** habit when fruiting **F** outer sepal **G** inner sepal **H** cyme with capsule **J** seed. Drawn by Rosemary Wise **A–C** from *Hinton* 1254; **D–G** from *Hancock* 46; **H–J** from *Palmer* 154.

#### Distribution.

From northern Mexico south to El Salvador and Honduras, principally in drier areas mostly between 200 and 1200 m. It mostly grows by streams or in gallery forest in deciduous tropical forest.

**HONDURAS.** Gulf of Fonseca: type collection.

**EL SALVADOR.** Ahuachapán, Área Protegida Santa Rita, *J.M. Rosales* 1948 (BM, B, MEXU, MO).

**GUATEMALA.** Cañon El Tapón, Huehuetenango, *A. Molina* 30134 (F).

**MEXICO. Chiapas**: Tonala, *C.A. Purpus* 6913 (BM, MO); Tenejapa, Río Chik Ha’, *D. Breedlove* 12640 (F). **Chihuahua**: *E. Palmer* 102 (K). **Guerrero**: Acapulco, *Hancock* 46 (K); Galeana, Tecpan, *G.B. Hinton* 10888 (GBH, K, MO); Acapulco, *E. Palmer* 154 (K, MO). **Hidalgo**: Zacualtipán, *E. Matuda* 38686 (MO). **Jalisco**: *E.J. Lott* 1585 (MO). **Michoacán**: Coalcomán, Villa Victoria, *G.B. Hinton* 12544 (GBH, K, MO); Aguililla, *E. Carranza & I. Silva* 6825 (IEB). **Morelos**: Atlacahualoya, *G. Flores & E. Cabrera* 335 (MEXU). **Nayarit**: Tepic, *E. Palmer* 1997 (P, US); Rosamorada, *E. Ruíz Sánchez & A. Castro* 486 (IEB). **Oaxaca**: Tehuantepec, *M. Elorsa* 5303 (IEB), 5334 (IEB, MO). **San Luis Potosí**: Rascon, *C.A. Purpus* 5406 (BM). **Sinaloa**: El Fuerte, La Constancia, *J. Ortega* 5486 (K); Imala, *E. Palmer* 1704 (S); Mun. Cosalá, Mineral de Nuestra Señora, c. 12 km E of Cosalá, *Rito Vega et al.* 2112 (MEXU). **Sonora**: San Bernardo, Río Mayo, *H.S. Gentry* 1616 (K, MO, S); *T.R. Van Devender & Dimmitt* 91-755 (ARIZ). **Tamaulipas**: *R. Kral* 27371 (MO). **Veracruz**: Rinconada, *Dorantes et al.* 01710 (BM, MEXU).

#### Typification.

Nelson (1996: 58) designated the Sinclair collection from the Gulf of Fonseca at K as lectotype but there are two sheets at Kew from the same location, neither annotated by Nelson. We have, therefore, redesignated the more complete sheet as lectotype. Similarly Austin (1997: 151) designated *Palmer* 710 (US) as lectotype of *Ipomoea grayi* but as there are three sheets of this number at US, we have redesignated the more complete sheet as lectotype.

#### Note.

**Figure 204.**
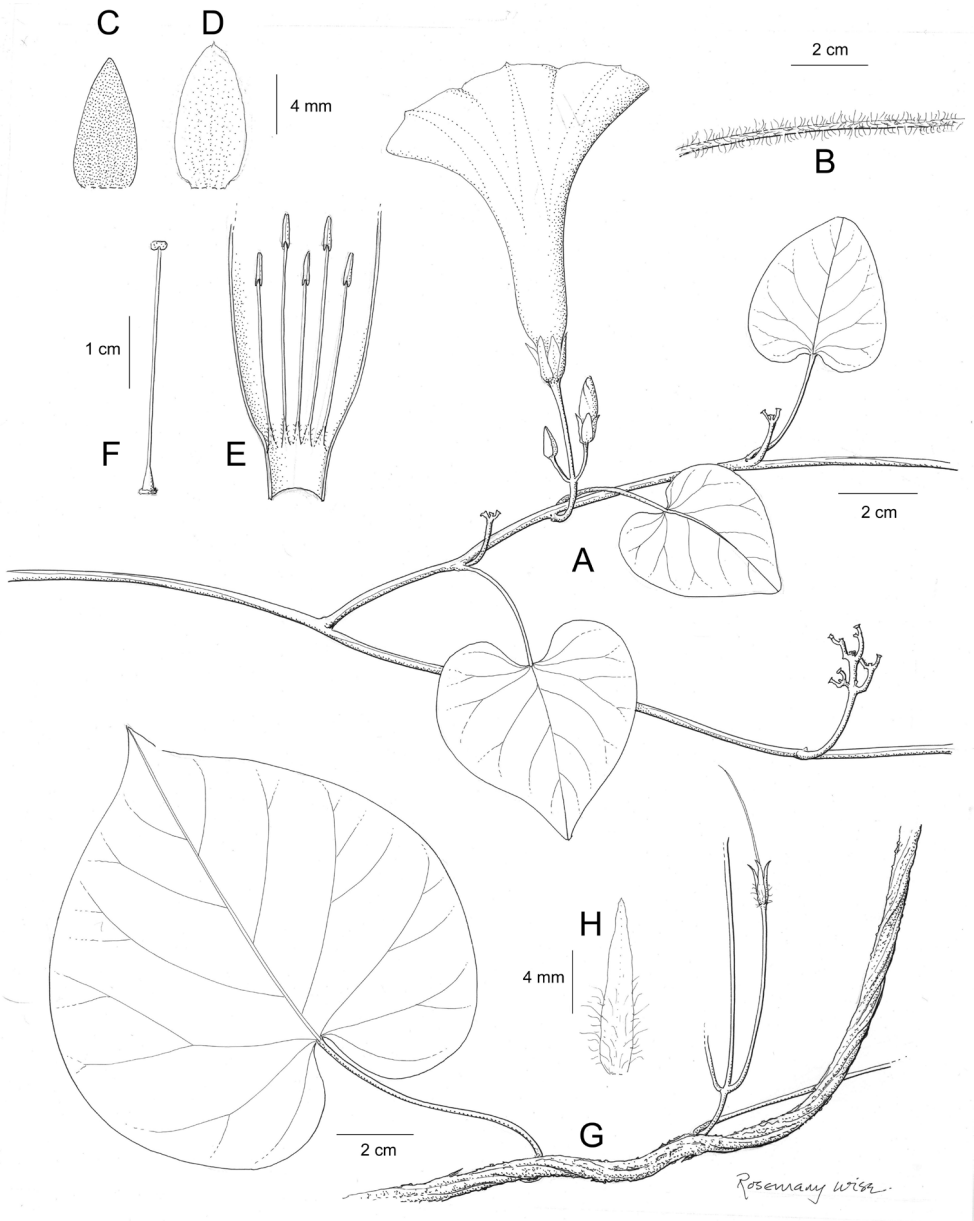
*Ipomoea regnellii*. **A** habit **B** stem **C** outer sepal **D** inner sepal **E** corolla opened out to show stamens **F** ovary and style **G** older stem **H** outer sepal, narrow form. Drawn by Rosemary Wise **A, C, D** from *Lugo* 1963; **B, E, F** from *Asplund* 15897; **G, H** from *Bohlin et al.* 1290.

The outer sepals are often muricate and the inflorescence has distinctive long pedicels, similar in form to *Ipomoea regnellii*, but often somewhat broader, although always glabrous. Occasionally the murication on the sepals develops into fleshy appendages similar to those seen in *I. tentaculifera*, most conspicuously in *Rito Vega et al.* 2112 (MEXU). At first sight this appears to be a distinct species and might perhaps merit recognition as a variety but seems to be only an extreme variation of a tendency occasionally found in other specimens of *I. pedicellaris*. The small mucro at the apex of the sepals is sometimes present (Figure 204F, G) and sometimes absent (Figure 203B, C).

### 415. Ipomoea tentaculifera

Taxon classificationPlantaeSolanalesConvolvulaceae

Greenm., Proc. Amer. Acad. Arts 33: 482. 1898. (Greenman 1898: 482)

#### Type.

MEXICO. Oaxaca, *C.G. Pringle* 6702 (holotype GH, isotypes AC, BM, BR, CM, E, F, GOET, K, M, MEXU, MICH, MO, MSC, NDG, NY, PH, S, UC, US).

#### Description.

Perennial herb, stems glabrous. Leaves petiolate, 6–11 × 2.5–6.5 cm, ovate, long-acuminate, cordate, glabrous, paler beneath; petioles 3.5–8 cm. Inflorescence of long-pedunculate solitary flowers; peduncles 7–12 cm; bracteoles caducous, not seen; pedicels 45–75 mm, noticeably thicker than peduncles and widened below calyx; sepals slightly unequal, outer 6–7 × 4.5 mm, ovate, obtuse, covered in soft fleshy spines on the abaxial surface, but otherwise glabrous, inner c. 8 × 5 mm, obovate, truncate, shortly mucronate, scarious-margined, soft spines only present near base; corolla 6–6.5 cm long, deep pink, glabrous, funnel-shaped, limb unlobed, 4–5 cm diam. Capsules and seeds not seen.

#### Illustration.

Figure 3C.

#### Distribution.

Pine woodland from 1500 to 2000 m. Endemic to Oaxaca.

**MEXICO. Oaxaca**: Abasolo, Santa Rosa Buenavista, *A. Saynes* 627 (IEB); San Felipe Tejalapa, *M. Cruz* 133 (IEB).

#### Note.

Resembling *Ipomoea crinicalyx* and allies in the fleshy spines on the calyx but the long thick pedicels are somewhat reminiscent of *I. setosa*. The solitary flowers and ovate, cordate, glabrous, spineless leaves render this species very distinct.

### 416. Ipomoea regnellii

Taxon classificationPlantaeSolanalesConvolvulaceae

Meisn. in Martius et al., Fl. Brasil. 7: 266. 1869. (Meisner 1869: 266)

Ipomoea warmingii Meisn. in Martius et al., Fl. Brasil. 7: 272. 1869. (Meisner 1869: 272). Type. BRAZIL. Minas Gerais, *E. Warming* 1764 (holotype BR00005793334, isotype C).
Ipomoea ophiodes Standl. & Steyermark, Field Mus. Nat. Hist., Bot. Ser. 23; 82. 1944. (Standley and Steyermark 1944: 82). Type. GUATEMALA. Santa Rosa, Región de La Morenita, Dec. 1940, *P. C. Standley* 78884 (holotype F0054857).

#### Type.

BRAZIL. Minas Gerais, Caldas, *A.F. Regnell* (lectotype BR000005793693, designated by O’Donell, 1952: 236).

#### Description.

Twining perennial herb, stems thinly pubescent to densely long-pilose, older parts sometimes with flaking bark. Leaves petiolate, 4–15 × 3–12 cm, ovate to suborbicular, cordate with rounded auricles, shortly acuminate to an obtuse and mucronate apex, adaxially thinly puberulent to subtomentose, abaxially weakly to densely tomentose; petioles 1.5–11 cm, thinly pubescent to tomentose. Inflorescence of shortly pedunculate axillary, many-flowered umbellate cymes, peduncles (0.3–)1–5.5 cm; bracteoles 1.5–2 mm, lanceolate, caducous; secondary peduncles 6–8 mm; pedicels 8–45 mm, relatively long, glabrous or pilose; sepals slightly unequal, lanceolate or oblong-lanceolate, obtuse and broadly mucronate to acuminate, pale green, thinly to densely pubescent, outer 7–11 × 1–3 mm, margins often ciliate, the inner 9–13 × 3–4 mm, often with scarious margins; corolla 5–9 cm long, funnel-shaped, pink or violet, densely short pubescent, limb c. 4 cm diam. Capsules subglobose to ellipsoid, 9–12 × 7–9 mm, rostrate (mucro 5 mm), glabrous; seeds 6 mm, pubescent on the angles (immature).

**Illustration**: Figures 6H, 204.

#### Distribution.

Widely distributed in moist forest regions of tropical America below about 1500 m from Bolivia north to Guatemala, but principally in the Andean foothills, and apparently relatively rare elsewhere as in Brazil and Central America.

**BRAZIL. Acre**: *E. Forero et al.* 6399 (MO, NY, R). **Goiás**: *R.C. Mendonça et al.* 4203 (RB). **Minas Gerais**: *C.W.Mosén* 1911 (S). **São Paulo**: *V.C. Souza et al.* 4986 (K, SPF).

**BOLIVIA. Beni**: Ballivián, Rurrenabague, *D. Williams* 955 (K, LIL, LPB, NY, MO, OXF, USZ). **Cochabamba**: Chapare, Cordillera de Mosetenes, *M. Kessler et al.* 13263 (LPB). **La Paz**: Sud Yungas, Río Bopi, *B.A. Krukoff* 10078 (F, GH, K, MICH, NY, MO, US). **Pando**: Madre de Dios, *D. Rocabado & E. Calzadilla* 949 (USZ). **Santa Cruz**: Ichilo, Urubó, *J.R.I. Wood & D. Soto* 27953 (OXF, K, LPB, USZ).

**PERU.** Sine loc., *Lechler* 2616 (K). **Cusco**: *C. Vargas* 16533 (CUZ); Paucartambo, *I. Huamantupa* 3514 (MO, OXF). **Madre de Dios**: *P. Nuñez* 6108 (MO, FTG). **Piura**: Ayabaca, *F. de La Puente 3148* (CIP). **Puno**: *P. Nuñez & C. Muñoz* 5329 (MO). **Tumbes**: *Díaz et al. 4849* (MA), *4098* (MA).

**ECUADOR. Esmeraldas**: *B. Løjtnant & U. Molau* (AAH). **Guayas**: *G. Tipaz et al*. 909 (FTG, MO); *E. Asplund* 15897 (F, K, NY, S, US). **El Oro**: *L. Albert* 1181 (S). **Loja**: *J.-E. Bohlin et al.* 1290 (GB). **Los Ríos**: *C.H. Dodson et al*. 8416 (MO); Río Pelenque, *A. Gentry* 9561 (MO). **Manabí**: *G. Harling & L. Andersson* 18845 (FTG); *A.S. Hitchcock* 20025 (US). **Napo**: *R. Marles* EE95 (F); Est. Biol. Jatun Sacha, *B.C. Bennett et al.* 207-SFS (QCNE). **Sucumbios**: Gonzalo Pizarro, Rio Dashiño, *A.P. Yañez et al.* 985 (QCA).

**COLOMBIA. Boyacá**: *M.T. Dawe* 913 (K). **Cauca**: *K. von Sneidern* 1111 (S). **Meta**: Río Meta, *T. Sprague* 30 (BM, K); Villavicencio, *J. Triana* 3805 p.p. (BM); ibid., *A.H.G. Alston* 7587 (BM); Sierra La Macarena, *W.R. Philipson et al.* 1642 (BM). **Putumayo**: *H.G. Barclay* 4698 (COL, MO).

**VENEZUELA. Bolívar**: El Dorado, *A. Gentry et al. 9561* (MO) – requires confirmation.

**COSTA RICA.** Puntarenas, *M.M. Chavarria* 735 (MO); *B. Hammel* 18629 (CR, MO). **HONDURAS.***I.C. Piñeda* 40 (MO).

**EL SALVADOR.***J. Hjalmarson* 1853 (S); Ataco, *J.L. Linares* 3768 (MEXU); Lake Illopango, *K. Sidwell et al.* 579 (BM).

**GUATEMALA.** Chiquimula, Jocotán, *J. Kufer* 275 (BM, MSB).

#### Notes.

Usually readily identified by the pubescent leaves and corolla, combined with the narrow, lanceolate, obtuse sepals and many-flowered pedunculate inflorescence.

We have united *Ipomoea ophiodes* with *I. regnellii* as we cannot see any consistent differences between the two species whose distribution complements each other. *Ipomoea ophiodes* is reported to have very pilose stems, few-flowered cymes, acuminate sepals and perhaps a narrower, more violet corolla (Figure 204 B, G, H). It is frequent in coastal Ecuador and parts of Central America and may prove to merit some kind of recognition.

### 417. Ipomoea chapadensis

Taxon classificationPlantaeSolanalesConvolvulaceae

J.R.I. Wood & L.V. Vasconc., Kew Bull. 72 (8): 2. 2017. (Wood et al. 2017a: 2)

#### Type.

BRAZIL. Bahia, Bonito, estrada Bonito para o assentamento Eugênio Lira, 11°59'54"S, 41°04'24"W, 10 Aug. 2014, *L. P de Queiroz, J.R.I. Wood & H. Huaylla* 15972 (holotype HUEFS, isotype OXF).

#### Description.

Twining perennial herb to 2 m; stems glabrous or thinly hirsute with short spreading hairs, less commonly tomentose. Leaves petiolate, 2–9(–13) × 1–4(–6) cm, narrowly (or rarely broadly) ovate, acute or acuminate and mucronate, base cordate with a narrow sinus and rounded auricles, both surfaces glabrous or tomentellous on the veins, adaxially with scattered hairs or hair bases only on both surfaces, rarely both surfaces tomentose; petioles 0.5–5 cm, the indumentum similar to that of the leaves. Inflorescence of solitary (rarely paired) axillary flowers; peduncles 0–5 mm often completely suppressed, glabrous to tomentose; bracteoles 2–3 × 0.5 mm, lanceolate, acuminate, persistent; pedicels 11–30 mm, thickened upwards, glabrous to tomentellous; sepals subequal, narrowly oblong-elliptic, acute and shortly mucronate, densely but very shortly puberulent, the margins slightly scarious, outer 7–11 × 3–3.5 mm, inner 9–13 × 4 mm, obtuse to rounded and mucronate; corolla 4.5–5(–7) cm long, funnel-shaped, pink, puberulent; limb 3.5(–5) cm diam., undulate. Capsules 10–11 × 7–9 mm, ovoid, rostrate, the style base 4–5 mm long, glabrous; seeds 7 × 4 mm, densely white-tomentose.

#### Illustration.

Figure 205.

**Figure 205.**
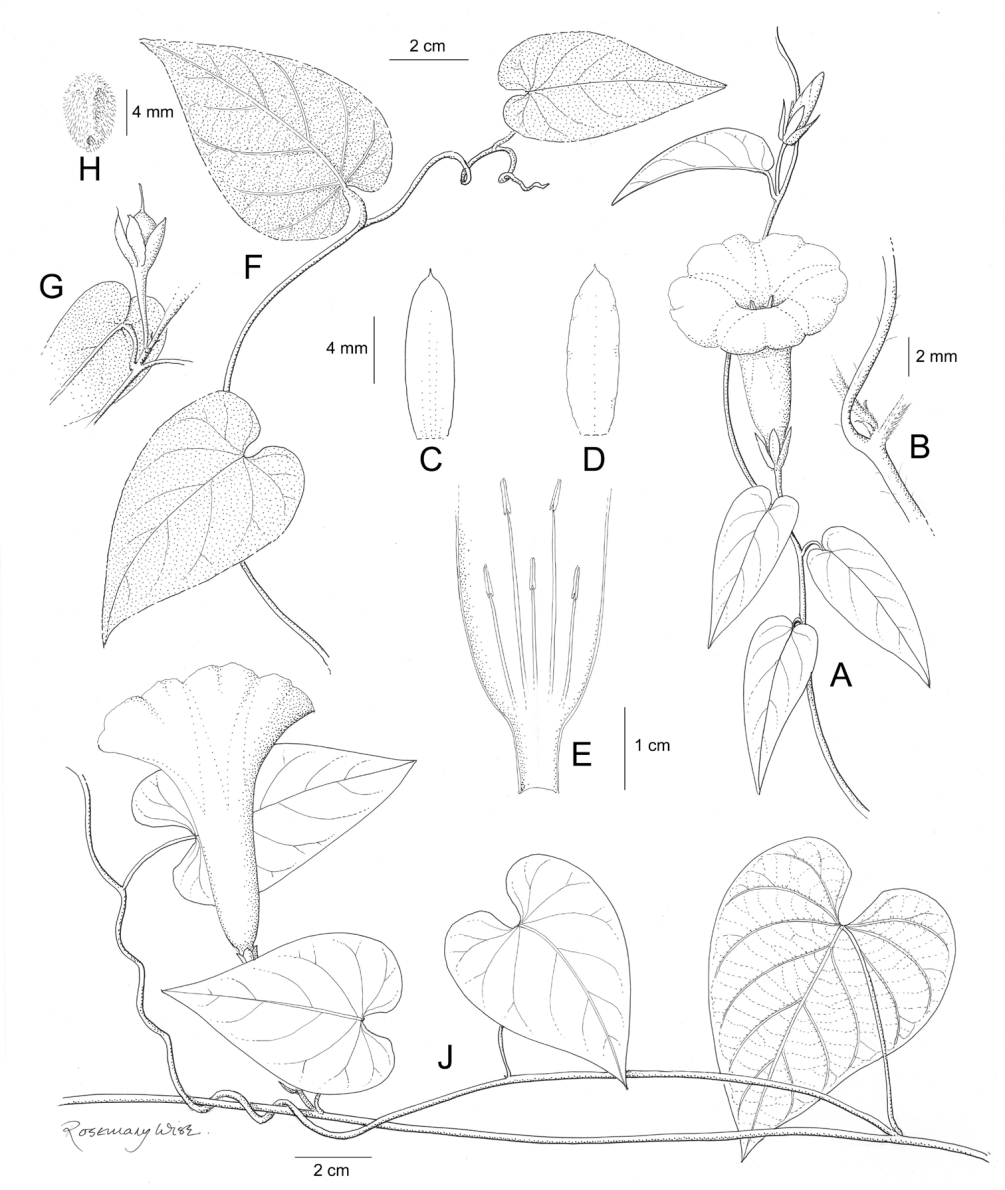
*Ipomoea chapadensis*. **A** habit **B** base of peduncle with bracteoles **C** outer sepal **D** inner sepal **E** corolla opened up to show stamens **F** habit with more pubescent indumentum **G** calyx with fruit **H** seed **J** habit with broader leaves. Drawn by Rosemary Wise **A, B** from *E. Souza et al.*121; **C, D** from *Almeida-Silva & Barros* 294; **E** J from *E. Melo* 12022; **F** from *Projeto Chapada Diamantina* 2598; **G** from *T. Cavalcanti* 330; **H** from *Lima & Lima* 291.

#### Distribution.

Endemic to the Chapada Diamantina in Bahia where it occurs along the borders of caatinga and cerrado vegetation.

**BRAZIL. Bahia**: Mun. Lençois, *R.M. Harley et al.* 14124 (K, SPF); Mun. Jacobina, *B. Stannard et al*. 2598 (ALCB, HUEFS, K); Mun. Palmeiras, *L.V. Vasconcelos et al.* 412 (HUEFS, SP); Mun. Rio de Contas, *R.M. Harley et al.* 25707 (CEPEC, K, SPF).

#### Note.

Very distinctive is the usually 1-flowered inflorescence with the peduncle almost completely suppressed so the bracteoles appear to be stipules. This serves to separate this species easily from *Ipomoea regnellii* to which it is obviously related. It is very variable in indumentum, some specimens (*Harley et al.* 25707, *Cavalcanti et al.* 330, *Junqeira & Andrade* 92) being markedly tomentose, others like *Souza et al.* 121 being glabrous. Intermediate states are often found. *Melo* 12022 (HUEFS) from the Mata Atlântica in Bahia is somewhat atypical because of its larger corolla and broadly ovate leaves, but the very short peduncle and solitary flower strongly suggest it is correctly placed.

### 418. Ipomoea tiliifolia

Taxon classificationPlantaeSolanalesConvolvulaceae

(Desr.) Roem. & Schult. Syst. Veg. 4: 229. 1819. (Roemer and Schultes 1819: 229)

Convolvulus tiliifolius [“*tilaefolius*”] Desr. in Lam., Encycl. 3: 544. 1792. (Desrousseaux 1792: 544). Type. Maurice [MAURITIUS]. *Commerson*s.n. (holotype P-LAM-00357542, isotypes G, MPU).
Rivea tiliifolia (Desr.) Choisy, Mém. Soc. Phys. Genève 6: 407[25]. 1834. (Choisy 1834: 407[25]).
Amphione tiliifolia (Desr.) Raf., Fl. Tellur. 4: 79. 1836 [1838]. (Rafinesque 1838a: 79).
Argyreia tiliifolia (Desr.) Wight, Icon. Pl. Ind. 4 (2): 12, t 1358. 1848. (Wight 1848: 12).
Stictocardia tiliifolia (Desr.) Hallier f., Bot. Jahrb. Syst. 18: 159. 1894 [pub. 1893]. (Hallier 1893b: 159).
Ipomoea benghalensis Roem. & Schult. Syst. Veg. 4: 229. 1819. (Roemer and Schultes 1819: 229). Type. INDIA. *Heyne*s.n. (wherabouts unknown, possibly B†).
Convolvulus gangeticus Roxb., Fl. Ind., 2: 46, 1824. (Roxburgh 1824; 46), nom. illeg., non Convolvulus gangeticus L. (1756). Type. INDIA. River Ganges, (lectotype *Roxburgh* Icon no. 1793 (K), designated here).
Ipomoea gangetica (Roxb.) Sweet, Hortus Brit., ed. 2: 288. 1830. (Sweet 1830: 288).
Ipomoea pulchra Blume, Bijdr. Fl. Ned. Ind. 716. 1826. (Blume 1825–26: 716). Type. INDONESIA. Moluccas, *C.L. Blume* 297 (holotype L0004259).
Convolvulus melanostictus Schltdl., Linnaea 6: 737. 1831. (Schlechtendal 1831: 737). Type. U.S. VIRGIN ISLANDS. St Thomas. *C. Ehrenberg*s.n. (holotype HAL0037540).
Ipomoea campanulata var. illustris C.B. Clarke, Fl. Brit. India 4: 211. 1883. (Clarke 1883: 211). Type. MYANMAR. *C. Parish*s.n. (lectotype K000830812, designated here).
Ipomoea campanulata auct., non L.

#### Type.

Based on *Convolvulus tiliifolius* Desr.

#### Description.

Twining liana to c. 12 m in height; stems densely white-pubescent/floccose when young, glabrescent, somewhat woody when old. Leaves petiolate, 5–15 × 4.5–15 cm, suborbicular to subreniform, apex rounded to retuse, sometimes mucronate, base cordate with rounded auricles, adaxially pubescent to puberulent, grey-green, abaxially dotted with dark glands, densely and softly white-pubescent when young, somewhat glabrescent and becoming greenish; petioles 5–15 cm, densely pubescent. Inflorescence of solitary (rarely 2–3) pedunculate flowers from the leaf axils; peduncles 1–9 cm, pubescent, somewhat glabrescent; bracteoles caducous; secondary peduncles 2–3 mm, white tomentose; pedicels 8–20 mm, elongating to 2.5 cm in fruit, shortly white-tomentose; sepals subequal, pubescent, broadly ovate, suborbicular, 11–15 × 11 mm, markedly accrescent in fruit to 40 × 35 mm and somewhat glabrescent; corolla 5.5–9 cm long, funnel-shaped, pink, pubescent towards the tips of the midpetaline bands; limb weakly lobed, 4.5–5 cm diam. Capsules 1.5–2 × 2–2.5 cm, compressed globose, indehiscent, enclosed by accrescent sepals; seeds ellipsoid, 9 × 6 mm, shortly but densely puberulent to subtomentose.

**Illustrations.** Figures 9H, 11P, 190A; Austin (1975b: 217) as *Stictocardia campanulata*; Liogier (1994: 113); Bosser and Heine (2000: 23); Deroin (2001: 151) all as *Stictocardia tiliifolia*.

#### Distribution.

Native of tropical Asia but long naturalised throughout the tropics, particularly on the shores of oceanic islands. In the New World reported as well-naturalised in the Galapagos Islands and near the sea in Central America and on some Caribbean Islands as well as Hawaii. Some of the records below may be of cultivated plants.

**ECUADOR. Galapagos Islands**: *T.W.J. Taylor* 108 (K); *G. Harling* 5610 (S); *U. & E. Eliason* (S); *H. Van der Werff* 1282A (MO).

**PANAMA.***B.C. Seeman* 174 (BM, K); *J.F. Macbride* 2614 (F, US).

**COSTA RICA.** Puntarenas, Isla de Coca, *F.J. Quesada* 1103 (BM, K, MO); ibid., Nicoya, *B. Hammel* 16796 (CR, MO).

**NICARAGUA.***W. Robleto* 1603 (MO).

**EL SALVADOR.** La Libertad, *R. Aparicio & R. Rivera* 127 (MO).

**GUATEMALA.***Friedrichstahl* s.n. (K).

**UNITED STATES. Florida**: *J.K. Small & C.A. Mosier* 6002 (K).

**CUBA.***E.L. Ekman* 3720 (S); *A.H. Liogier* 14397 (NY).

**HAITI.***E.L. Ekman* H5181 (S), H2923 (S).

**DOMINICAN REPUBLIC.***E.L. Ekman* H10929 (K, S).

**PUERTO RICO.***A. Liogier* et al. 40410 (NY); *F. Axelrod et al*. 3326 (NY).

**LESSER ANTILLES. U.S. Virgin Islands**: St John, *P. Acevedo-Rodríguez* 3120 (MO, NY). **U.K. Virgin Islands**: Guana Island, *G.R. Proctor* 43448 (NY). **Antigua**: *H.E. Box* 1299 (BM). **Montserrat**: *J.A. Shafer* 64 (NY); *G.R. Proctor* 18931 (BM). **Martinique**: *Hahn* 628 (BM). **Dominica**: *Imray* 230 (K); *C. Whitefoord* 5400 (BM). **Guadeloupe**: *A. Duss* 2476 (NY); Marie Galante, *G.R. Proctor* 20264 (BM). **St Lucia**: sine data (BM). **St Vincent**: *H.H. Smith* 1610 (K, NY). **Barbados**: fide Gooding et al. (1965).

**HAWAII.***Faurie* 1041 (BM); *G.W. Barclay* s.n. (BM).

#### Lectotypifiation.

There are no extant original specimens of *Convolvulus gangeticus* so we have selected Roxburgh’s painting (Icon no. 1793) as lectotype. This is preserved at Kew and can be seen online at http://apps.kew.org/floraindica/displayImages.do?index=1

#### Notes.

*Ipomoea tiliifolia* is a robust liana, usually with solitary pink flowers, pubescent on the exterior. The sepals are strongly accrescent around the large subglobose capsule. The dark glands on the abaxial leaf surface are also distinctive.

*Ipomoea tiliifolia* is the only representarive of the Stictocardia Clade found in the neotropics. It has usually been treated in a separate genus as *Stictocardia tiliifolia* based on the presence of gland dots on vegetative parts and the corolla, on the strongly accrescent sepals and by the separation of the fruit into exocarp and endocarp. The thin exocarp breaks up easily when dry leaving a lantern-shaped, 4-valved endocarp. Although this structure appears to be unique to species previously placed in *Stictocardia*, molecular studies do not support its retention as a separate genus. (Austin and Sebsebe Demissew1997, Ooststroom 1943, Muñoz-Rodríguez et al. 2019).

### 419. Ipomoea involucrata

Taxon classificationPlantaeSolanalesConvolvulaceae

P. Beauv., Fl. Owar 2: 52. 1817. (Beauvois 1817: 52)

Convolvulus perfoliatus Schumach. & Thonn., Beskr. Guin. Pl. 89. 1827. (Schumacher and Thonning 1827: pl. 89). Type. GHANA. *Thonning*s.n. (syntypes C).
Ipomoea operosa C.H. Wright, Bull. Misc. Inf., Kew 1897: 275. 1897. (Wright 1897: 275). Type. MALAWI. Zomba, Whyte (syntype K000097217) and *Kirk*s.n. (syntype K000097216).
Ipomoea involucrata var. operosa Verdc., Kew Bull. 13: 206.1958. (Verdcourt 1958: 206).
Ipomoea involucrata var. burtii Verdc., Kew Bull. 13: 206.1958. (Verdcourt 1958: 206).Type. TANZANIA. Kondo anear Sambala, *B.D. Burtt* 2155 (holotype K000097096, isotype EA).
Ipomoea austinii Infante-Betancour, Caldasia 36 (2): 248. 2014. (Infante-Betancour 2014: 248). Type. COLOMBIA. Cesar, San Alberto, *M. Carillo-F* 557 (holotype COL).

#### Type.

NIGERIA, Oware, *P. de Beauvois* (holotype G00023040).

#### Description.

Variable trailing or twining annual or short-lived perennial herb; stems rather slender, pubescent to asperous-pilose in all parts. Leaves petiolate, 2.5–6(–10) × 1.5–5(–8.5) cm, ovate, acute or obtuse and mucronate, cordate with rounded auricles, both surfaces pubescent to tomentose, abaxially paler; petioles 2–7 cm, pubescent. Inflorescence of axillary pedunculate, involucrate heads; peduncles 2.5–10 cm, densely pubescent to tomentose; outer bracteoles united at base to form a boat-shaped involucre around the flowers, sessile, 2.5–6.5 × 0.8–1.5 cm, each ovate, acuminate, folded, pubescent; inner bracteoles much smaller, obovate to linear-oblong; pedicels very short; flowers few to many; sepals unequal, 6–15 × 1.5–4 mm, acute to acuminate, pubescent to setose, outer lanceolate, inner 2–3 mm shorter, ovate; corolla 3.5–4.5 cm long, funnel-shaped, pink, less commonly white, pubescent; limb 3–4 cm diam., entire. Capsules 6 mm, globose, glabrous; seeds glabrous or shortly pubescent, c. 4 mm long.

#### Illustration.

Infante-Betancour (2014: 249) as *Ipomoea austinii*.

#### Distribution.

Common in West and Central Africa but a recent introduction in the neotropics in a Colombian oil palm plantation.

**COLOMBIA.** Cesar: type of *Ipomoea austinii*.

#### Note.

Probably an adventive in the New World but may become established.

We have not seen *M. Carillo-F* 557 but the shape and measurements of the sepals accompanying the protologue of *Ipomoea austinii* (Infante-Betancour 2014) clearly indicate that it is *I. involucrata*, rather than the very similar *I. pileata* Roxb. The drawing of the inflorescence, however, is very stylised and difficult to interpret.

••• The following four species are unplaced within *Ipomoea*. We are unable to make even a guess at their likely placement.

### 420. Ipomoea dolichopoda

Taxon classificationPlantaeSolanalesConvolvulaceae

J.R.I. Wood & R. Degen, Phytokeys 88: 14. 2017. (Wood et al. 2017d: 14)

#### Type.

PARAGUAY. Caazapá, Castor Cue, 26°10'S, 55°20'W, *I. Basualdo* 002775 (holotype FCQ, isotype MO).

#### Description.

Trailing herb, probably perennial; stems thinly pilose with white hairs. Leaves petiolate, 4–6.5 × 0.8–1.5 cm, slightly oblique, oblong, base cuneate, apex obtuse and mucronate, margins ciliate, adaxially glabrous, abaxially pilose on the veins; petioles 7–8 mm, thinly pilose. Inflorescence of pedunculate axillary cymes with 1–4 flowers borne on long secondary peduncles; primary peduncles 0.3–1.2 cm; secondary peduncles 7–12 cm, thinly pilose; bracteoles 9–12 × 1 mm, filiform, persistent until anthesis; pedicels 8–15 mm, pilose; sepals 10–14 × 3–4 mm, ovate, finely acuminate to a mucronate apex, base rounded to truncate, outer sepals pilose except at margins, inner sepals slightly shorter with glabrous, scarious margins; corolla c. 5.5 cm long, broadly funnel-shaped, glabrous in bud, pink, limb c. 3.5 cm diam. Capsules and seeds unknown.

#### Illustration.

Figure 206.

**Figure 206.**
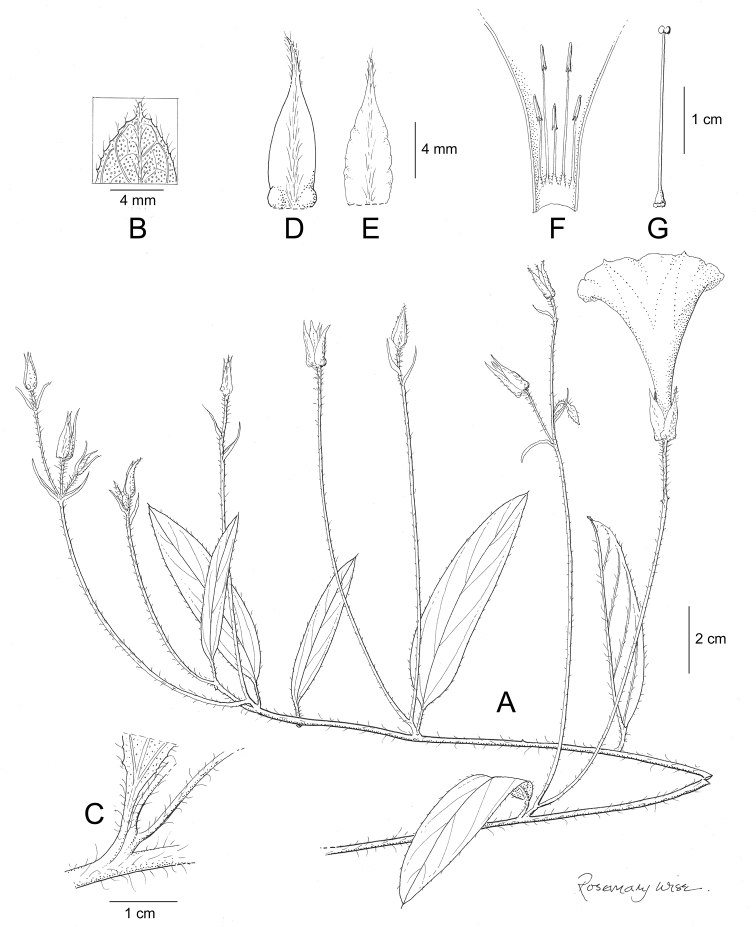
*Ipomoea dolichopoda*. **A** habit **B** leaf apex **C** leaf base showing peduncle **D** Outer sepal **E** inner sepal **F** corolla opened out to show stamens **G** ovary and style. Drawn by Rosemary Wise from *I. Basualdo* 002775.

#### Distribution.

Only known from the type collection which was found in “praderas”, presumably some kind of cerrado grassland in eastern Paraguay.

**PARAGUAY.** Caazapá: type collection.

#### Note.

This species bears a strong superficial resemblance to *Ipomoea attenuata* but differs in the glabrous corolla. Both species have somewhat similar oblong, shortly petiolate leaves and ovate sepals with a distinct truncate base and acuminate apex. *Ipomoea dolichopoda*, however, can be distinguished at first glance by the long white hairs, which are scattered over all vegetative parts. It is also distinct in the very short primary peduncles combined with the very long secondary peduncles, a combination that in our experience is unique in *Ipomoea*.

### 421. Ipomoea discoidea

Taxon classificationPlantaeSolanalesConvolvulaceae

González-Martínez & J. Jiménez Ram., Brittonia 67: 320. 2015. (Jiménez Ramírez and González-Martínez. 2015: 320)

#### Type.

MEXICO. Guerrero, Mun. Chilpancingo de los Bravos, *C.A. González-Martínez & S. Ríos-Carrasco* 761 (holotype FCME151016, isotype FCME).

#### Description.

Slender annual night-flowering twining herb, stems glabrous, up to 5 m long. Leaves petiolate, 6–10 × 4–7 cm, ovate, acuminate and mucronate, base cordate with obtuse auricles, both surfaces glabrous; petioles 3–5 cm long, setose below the junction with the leaf. Inflorescence of pedunculate, axillary 2-flowered cymes; peduncles up to 9 cm long, setose at the base, gland-dotted, sticky; bracteoles ovate, c. 1.5 mm long, caducous; secondary peduncles 5–13 mm; pedicels 7–8.5 mm, somewhat accrescent in fruit, sticky-glandular, otherwise glabrous; receptacle forming a swollen disc at base of flowers; sepals subequal, 4–5 × 2–2.5 mm, ovate-deltoid, acute, glandular, becoming reflexed in fruit; corolla 4.5–5.3 cm long, cylindrical-hypocrateriform, greenish-white, glabrous, limb c. 5 cm diam., unlobed; stamens weakly exserted. Capsules ovoid, c. 2 × 1.5 cm, rostrate, glabrous; seeds c. 10 × 7 mm, ellipsoid, pubescent and with long white marginal hairs 11–12 mm long.

#### Illustration.

Jiménez Ramírez and González-Martínez (2015: 321).

#### Distribution.

Endemic to Mexico growing at around 800–1000 m in semi-deciduous forest.

**MEXICO. Guerrero**: type locality. **Jalisco**: Mun. El Limon, Cerro el Carrizal, *A. Flores* 3679 (MEXU).

#### Note.

Very distinctive because of the setose peduncles and the disc-like receptacle. The peduncles, pedicels and sepals are reported to be sticky glandular. The annual habit combined with the hypocrateriform white corolla is also unusual.

### 422. Ipomoea fasciculata

Taxon classificationPlantaeSolanalesConvolvulaceae

J.R.I. Wood & Scotland, Phytokeys 88: 18. 2017. (Wood et al. 2017d: 18)

#### Type.

BRAZIL. Pernambuco, Agrestina, Inselberg Pedra Cabeça de Velho, *P. Gomes, M. Alves & B. Maciel* 658 (holotype RB00601358, isotype UFP).

#### Description.

Climbing perennial, stem minutely puberulent, glabrescent. Leaves petiolate, 2.5–5.5 × 2.4–5 cm, ovate, cordate with rounded auricles, apex acute, margin undulate to minutely denticulate, both surfaces shortly puberulent but more densely so on the abaxial veins, abaxially paler; petioles 1–4.5 cm, thinly puberulent. Inflorescence of pedunculate axillary cymes reduced to pedunculate clusters; peduncles 3.5–5.5 cm, puberulent; bracteoles 5 × 1.5 mm, lanceolate, acuminate, scarious, caducous; pedicels 1–3 mm, pubescent; sepals slightly unequal, glabrous; outer 5–6 × 3 mm, ovate, mucronate, abaxially slightly muricate, margin scarious, inner 6–8 × 4–6 mm. oblong-elliptic, rounded and mucronate, entirely scarious except central area; corolla c. 5 cm long, pink, funnel-shaped, glabrous, limb 3 cm diam.; stamens included; longer filaments 12–15 mm, shorter c. 10 mm. Capsules ovoid, rostrate, 5 × 3 mm with 1 mm long mucro, glabrous; seeds not seen.

#### Illustration.

Figure 207.

**Figure 207.**
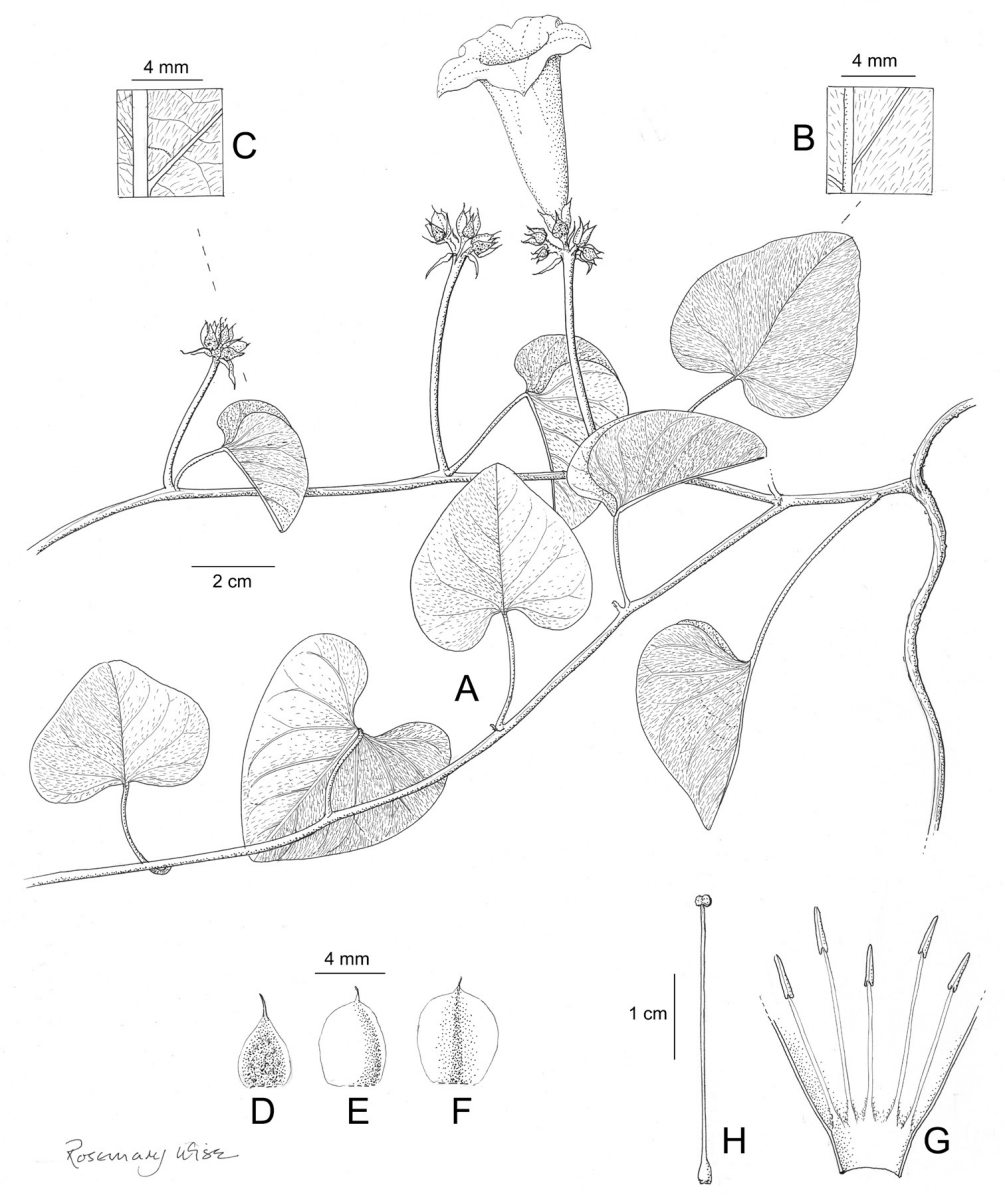
*Ipomoea fasciculata*. **A** habit **B** adaxial leaf surface **C** abaxial leaf surface **D** outer sepal **E** middle sepal **F** inner sepal **G** corolla opened out to show stamens **H** ovary, style and stigma. Drawn by Rosemary Wise from *P. Gomes et al.* 658.

#### Distribution.

Only known from a single inselberg in north eastern Brazil.

**BRAZIL**. Pernambuco: Only known from the type collection.

#### Note.

Distinct because of clustered flowers, prominently mucronate, small, scarious sepals and small, rostrate capsules.

### 423. Ipomoea scopulina

Taxon classificationPlantaeSolanalesConvolvulaceae

J.R.I. Wood & Scotland, Phytokeys 88: 27. 2017. (Wood et al. 2017d: 27)

#### Type.

BRAZIL. Espirito Santo, Pancas, Pedra da Colina, 19°13'51"S, 40°52'35"W, 700 m, *D.P. Saraiva, J. Silva, K.V. Hmeljeviski & R.C. Forzza* 47 (holotype RB 00591205).

#### Description.

Liana of unknown height; stems woody, pale grey, glabrous. Leaves petiolate, 4–7 × 3–5 cm, ovate, shortly acuminate, base cordate with rounded auricles, margin undulate, adaxially glabrous, abaxially paler, somewhat reticulate, the main veins obscurely puberulent; petioles 1.5–2.5 cm, glabrous or obscurely puberulent upwards. Inflorescence borne on woody branchlets, the axillary cymes subracemose in form, apparently arising after the leaves have fallen; peduncles 6–7 mm long, somewhat woody, glabrous apart from a few scattered hairs; bracteoles deltoid, c. 1 mm long, glabrous, caducous; secondary peduncles 2–7 mm long; pedicels 6–10 mm long, glabrous; sepals slightly unequal, outer 6–7 × 3–3.5 mm, broadly lanceolate, subacute, glabrous, margin scarious, inner similar but obtuse and with broader scarious margins; corolla 3.5–4 cm long, suburceolate, glabrous, reported to be “white”, tube subcylindrical, c. 4 mm wide at base, widened to 10 mm in the middle, constricted upwards, c. 6 mm wide at mouth, lobes broadly ovate, c. 2 × 3.5 mm; ovary presumably glabrous, style c. 2.2 cm, stigma biglobose. Capsules and seeds not seen.

#### Illustration.

Figure 208.

**Figure 208.**
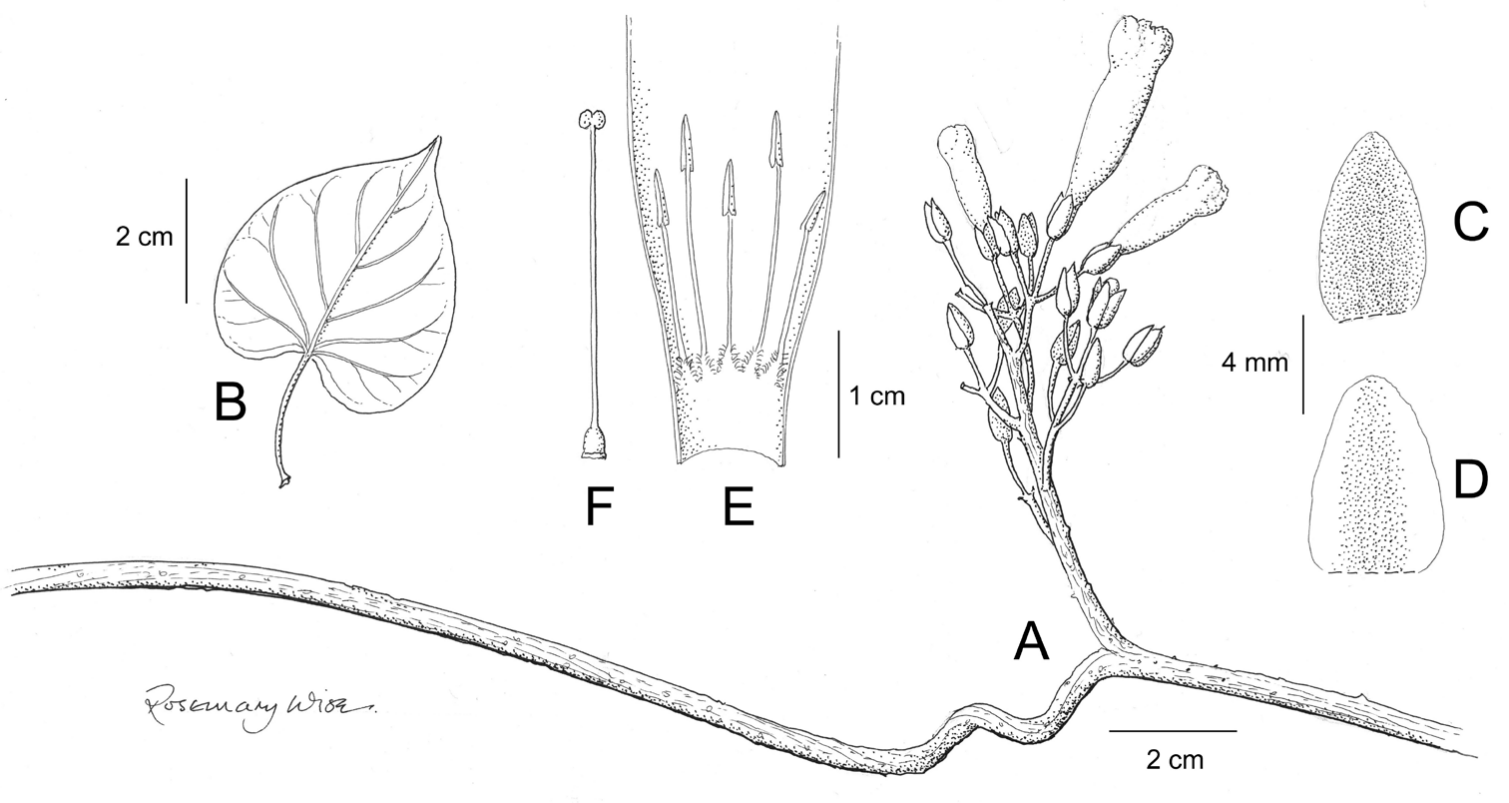
*Ipomoea scopulina*. **A** habit **B** leaf **C** outer sepal **D** inner sepal **E** corolla opened out to show stamens **F** ovary and style. Drawn by Rosemary Wise from *D.P. Saraiva et al.* 47.

#### Distribution.

Only known from a single granite sugarloaf inselberg in Espirito Santo State in Brazil.

**BRAZIL. Espirito Santo**: Only known from the type collection.

#### Note.

Species of *Ipomoea* with tubular suburceolate corollas are rare in Brazil. The only two comparable Brazilian species are *I. longistaminea* and *I. ana-mariae*. Both have oblong-elliptic, coriaceous, somewhat convex sepals very different from the broadly lanceolate subacute sepals of *I. scopulina*.

### 424. Ipomoea vespertilia

Taxon classificationPlantaeSolanalesConvolvulaceae

D. Santos, G. C. Delgado Junior & Buril, Brittonia 71: 191. 2019. (Santos et al. 2019: 191)

#### Type.

BRAZIL. Ceará, Sobral, on the ascent of Meruoca Mountain, 40°20'58"S, 3°41'10"W, *F. D. S. Santos* 506 (holotype EAC, isotypes HUVA, K, PEUFR, RB).

#### Description.

Liana to 6 m, roots tuberculate. Leaves petiolate, 3.5–18.5 × 2.7–14.8 cm, ovate, 3.5–18.5 × 2.7–14.8 cm; adaxially sparsely pubescent to glabrescent, abaxially paler, sparsely pubescent or with hairs restricted to the veins, deciduous at anthesis; petiole 2–11.5 cm long,pubescent or glabrescent. Inflorescence of compound cymes with up to 25 flowers; peduncles 3–20.5 cm, canescent, glabrescent; bracteoles 10–15 mm long, often deciduous; pedicels 5–10 mm long, canescent; sepals equal, 6–8 × 5–7 mm, ovate, oblong to elliptic, slightly convex, apex obtuse to rounded, canescent; corolla 2.3–4.5 cm long, narrowly funnel-shaped, white, midpetaline bands canescent, greenish; anthers held at mouth of corolla; ovary ca. 3mm long, conical, glabrous, 4-locular with the loci uniovulate. Capsules 0.9–1 cm long, ellipsoid, glabrous; Seeds 6–7 mm long, with hairs 3–5 (−8) mm, restricted to the margins and dorsal region.

#### Illustration.

Santos et al. 2019.

#### Distribution.

Endemic to the caatinga region of NE Brazil.

**BRAZIL. Ceará** and **Paraibá** fide Santos et al. (2019).

#### Notes.

We have not had the opportunity to evaluate specimens of this species and have not carried out any molecular sequences. The description of the species suggests it is closely related to *Ipomoea marcellia* but the 4-locular, uniovulate ovary would suggest it belongs to the Quamoclit Clade (312–327). In consequence, this species is placed in this section of the monograph.

J.A. Queiroz et al. (2015) describe how corolla morphology has diverged from *Ipomoea marcellia* in response to different pollinators.

### 425. Ipomoea

Taxon classificationPlantaeSolanalesConvolvulaceae

sp. D (E. Jara 990)

#### Description.

Perennial twining herb with wiry stems to 1.2 m, latex white, rootstock swollen, purplish. Leaves petiolate, 2–3.8 × 0.3–1 cm, lanceolate, base rounded to cuneate, apex acute and aristate, both surfaces glabrous; petiole 4–10 mm, weakly winged near base. Inflorescence of very shortly pedunculate axillary cymes with up to 4 flowers; peduncles 3–5 mm long, muricate; bracteoles not seen; pedicels 10–15 mm long; sepals unequal, coriaceous, glabrous, outer 5–10 mm, ovate, obtuse, inner oblong, rounded, 8–12 × 4–6 mm; corolla 5.5–6 cm long, deep pink, hypocrateriform, limb 4–5 cm diam., anthers exserted. Capsules 5–10 × 8–15 mm, compressed-globose; seeds 3–5 × 5–10 mm, pubescent.

#### Distribution.

Endemic to the Marañon valley where it grows in seasonally dry deciduous scrub at 2360 m.

**PERU. Ancash**: Prov. Antonio Raimondi, Dist. Chingas, Hanan Raqui, *E. Jara* 990 (USM).

**Notes**. A description is being prepared by the discoverers of this species but initial molecular studies have not confirmed its placement.

The roots are eaten raw by people in the neighbourhood of the collection locality.

## Excluded species

The following list does not include species treated as synonyms of recognised species of *Ipomoea*, nor any discussed in the main species treatment. It aims to include all American species described in *Ipomoea*, *Batatas*, *Exogonium* or *Quamoclit* of uncertain identity or transferred to other genera. It should be noted that we have generally accepted the identification of species listed as transferred to other genera by Austin and Huáman (1996) or Austin et al. (2015) and our determinations do not indicate we have revised these transfers thoroughly.

***Batatas bonariensis*** Lindl., Bot. Reg. 24: 55. 1838. (Lindley 1838b: 55). Type. Not cited. Uncertain species. From the description, this appears to be *Ipomoea platensis* Ker-Gawl. but it is not possible to confirm this.

***Batatas cissoides*** (L.) Choisy, Mém. Soc. Phys. Genève 6: 437 [55]. 1834. (Choisy 1834: 437[55]) = *Distimake cissoides* (Lam.) A.R. Simões & Staples

***Batatas pentaphylla*** (L.) Choisy, Mém. Soc. Phys. Genève 6: 436 [54]. 1834. (Choisy 1834: 436[54]), nom. superfl. = *Distimake aegyptius* (L.) A.R. Simões & Staples

***Batatas quinquefolia*** (L.) Choisy, Mém. Soc. Phys., Genève 6: 49 [127]. 1838. (Choisy 1838: 49 [127]) = *Distimake quinquefolius* (L.) A.R. Simões & Staples

***Batatas tomentosa*** Choisy in A.P. de Candolle, Prodr. 9: 337. 1845. (Choisy 1845: 337) = *Merremia tomentosa* (Choisy) Hallier f.

**Batatas tomentosa var. elongata** Choisy in A.P. de Candolle, Prodr. 9: 337. 1845. (Choisy 1845: 337) = *Distimake digitatus* (Spreng.) A.R. Simões & Staples var. (Merremia digitata var. elongata (Choisy) D.F. Austin & Staples)

***Calystegia cymosa*** Ledeb., Ind. Hort. Dorpat. 4. 1821. (Ledebour 1822 4). Type. BRAZIL. (wherabouts unknown, not found at LE). See *Ipomoea ledebourii*.

***Calystegia discolor*** Dammer, Bot. Jahrb. Syst. 23, beibl. 57: 42. 1897 (Dammer 1897: 42), non *Ipomoea discolor*Kunth (1819). Type. BRAZIL. *A.F.M. Glaziou* 11260 (isotype K00612831) = *Ipomoea* sp. indet. The material at Kew has not been matched certainly with any known species, but see page 209.

***Convolvulus ebracteatus*** Desr., Encycl. 3: 541. 1792 [dated 1789]. (Desrousseaux 1792: 541). Type. A plant of unknown origin cultivated “au Jardin du Roi” (holotype P-LAM00357567). See *Ipomoea ebracteata*.

***Convolvulus ipomoea*** Vell., Fl. Flumin.72, t. 56. 1825 [pub. 1829]. (Vellozo 1829: 71). Type. BRAZIL. (lectotype, original parchment plate of Flora Fluminensis in the manuscript section of the Biblioteca Nacional, Rio de Janeiro [cat. no.: mss1198651-056], designated here; later published in Vellozo, Fl. Flum. Icon. 2: t. 56 1827. [pub. 1831]). Uncertain species, the illustration represents a plant of the Batatas Clade, possibly *Ipomoea australis*.

***Convolvulus pendulus*** Silva Manso, Enum. das Subst. Braz. 17. 1836. (Manso 1836: 17). Type. BRAZIL. Minas Gerais, Mun. Rio Manso by Rio Grande, collector not cited (whereabouts unknown) = *Ipomoea utilis* Choisy (see below).

***Convolvulus polyrrhizos*** Silva Manso, Enum. das Subst. Braz. 18. 1836. (Manso 1836: 18). Type. Not cited. = *Ipomoea polyrrhizos* (Silva Manso) Choisy (see below).

***Convolvulus puniceus*** Silva Manso, Enum. das Subst. Braz. 18. 1836. (Manso 1836: 18). Type. Not cited. = *Ipomoea punicea* (Silva Manso) Choisy (see below).

***Convolvulus triqueter*** Vell., Fl. Flumin.71, t. 53. 1825 [pub. 1829]. (Vellozo 1829: 74), nom. illeg., non *Convolvulus triqueter* L. Type. BRAZIL. (lectotype, original parchment plate of Flora Fluminensis in the manuscript section of the Biblioteca Nacional, Rio de Janeiro [cat. No.: mss1198651-053], designated here; later published in Vellozo, Fl. Flum. Icon. 2: t. 53 1827. [pub. 1831]). Uncertain species, possibly *Ipomoea squamosa* Choisy.

***Convolvulus ventricorus***, Silva Manso, Enum. das Subst. Braz. 20. 1836. (Manso 1836: 20). Type. Not cited.

Uncertain species, presumably an *Ipomoea*. It was reported as being used as a horse purgative and described as a twining plant with tomentose oblong-cordate, obtuse leaves, canescent beneath, a 2-flowered peduncle and a ventricose corolla with an elongate tube.

***Exogonium solanifolium*** (L.) Britton, Brooklyn Bot. Gard. Mem. 1: 82. 1918. (Britton 1918b: 82) = *Jacquemontia solanifolia* (L.) Hallier f.

***Exogonium velutifolium*** House, Bull. Torrey Bot. Club 35: 100. 1908 (House 1908a: 100) = *Holographis velutifolia* (House) T.F. Daniel (Acanthaceae)

***Ipomoea amparoana*** Pilg., Repert. Spec. Nov. Regni Veg. 17: 125. 1921. (Pilger 1921: 125). Type. COSTA RICA. A cultivated plant, *Amparo de Zeledon* s.n. (?B†).

Uncertain species. A plant from Clade A2 resembling *I. batatoides* but buds pubescent. No known species from this region fits these characteristics.

***Ipomoea angustifolia*** Jacq., Collectanea 2, 1789 [dated1788]. (Jacquin 1789: 2) = *Xenostegia tridentata* (Jacq.) D.F. Austin & Staples

***Ipomoea angustifolia*** D. Parodi, Contr. Fl. Parag. 21. 1877. (Parodi 1877: 21), nomen nudum.

***Ipomoea aristulata*** M. Martens & Galeotti, Bull. Acad. Roy. Sci. Bruxelles 12: 263. 1845. (Martens and Galeotti 1845: 263). Type. MEXICO. Michoacán, Morelia, *H. Galeotti* 1383 (two supposed syntypes BR).

While the two specimens filed at BR under the name *Ipomoea aristulata* are clearly *Ipomoea capillacea*, they are not in accord with the protologue which describes a twining plant with ovate, cordate leaves. It seems the unnumbered specimens may have been filed incorrectly under this name. *Ipomoea aristulata* is an uncertain species.

***Ipomoea bisperma*** Larrañaga, Escritos Damaso Antonio Larranaga 2: 78.1923. (Larrañaga 1923: 78). Type. Not specified.

Unknown species presumably from Uruguay. The description as “loculis monospermis” suggests this is *I. hederifolia* L. or *I. indivisa* (Vell.) Hallier f.

***Ipomoea bronsonii*** J.N. Gerard, Garden & Forest 5: 345. 1892. (Gerard 1892: 345). Type. Cultivated sterile plant of Cuban origin, apparently not preserved.

Unknown species

***Ipomoea caesia*** Hoffmanns., Verz. Pfl.-Kult. Nachtr. 2: 140. 1826. (Hoffmannsegg 1826: 140). Type. BRAZIL. (whereabouts unknown).

Uncertain species of the Pharbitis Clade, probably *I. purpurea* or *I. indica*.

***Ipomoea cephalantha*** Dammer, Bot. Jahrb. Syst. 23, Beibl. 57: 39. 1897. (Dammer 1897: 39) = *Jacquemontia hallieriana* Ooststr.

***Ipomoea cernua*** (Moric.) Hassl., Repert. Spec. Nov. Regni Veg. 9: 156. 1911. (Hassler 1911: 156) = *Aniseia martinicensis* (Jacq.) Choisy

***Ipomoea cernua*** forma ***acutifolia*** Hassl., Repert. Spec. Nov. Regni Veg. 9: 156. 1911. (Hassler 1911: 156) = *Aniseia cernua* Moric.

***Ipomoea cernua*** forma ***chacoensis*** Hassl., Repert. Spec. Nov. Regni Veg. 9: 156. 1911. (Hassler 1911: 156) = *Aniseia argentina* (N.E.Br.) O’Donell

***Ipomoea cernua*** forma ***grandiflora*** Hassl. (as I. cernua var. meisneri forma grandiflora), Repert. Spec. Nov. Regni Veg. 9: 156. 1911. (Hassler 1911: 156) = *Aniseia martinicensis* (Jacq.) Choisy

***Ipomoea cernua*** var. ***meisneri*** Hassl., Repert. Spec. Nov. Regni Veg. 9: 156. 1911. (Hassler 1911: 156) = *Aniseia martinicensis* (Jacq.) Choisy

***Ipomoea cernua*** var. ***obtusifolia*** Hassl., Repert. Spec. Nov. Regni Veg. 9: 156. 1911. (Hassler 1911: 156) = Aniseia martinicensis var. ambigua Hallier f.

***Ipomoea cernua*** forma ***palmirensis*** Arechav., Anales Mus. Nac. Montevideo 7: 192. 1911. (Arechavaleta y Balpardo 1911: 195). 1911: 192) = *Aniseia argentina* (N.E.Br.) O’Donell

***Ipomoea cernua*** forma ***paraguariensis*** Hassl. (as I. cernua var. meisneri f. paraguariensis), Repert. Spec. Nov. Regni Veg. 9: 156. 1911. (Hassler 1911: 156) = Aniseia martinicensis var. ambigua Hallier f.

***Ipomoea cernua*** forma ***platensis*** Hassl., Repert. Spec. Nov. Regni Veg. 9: 156. 1911. (Hassler 1911: 156) = *Aniseia argentina* (N.E.Br.) O’Donell

***Ipomoea cernua*** subforma ***subsericea*** Hassl., Repert. Spec. Nov. Regni Veg. 9: 156. 1911. (Hassler 1911: 156) = *Aniseia cernua* Moric.

***Ipomoea cernua*** forma ***yapeyuana*** Arechav., Anales Mus. Nac. Montevideo 7: 193. 1911. (Arechavaleta y Balpardo 1911: 193) = *Aniseia argentina* (N.E.Br.) O’Donell

***Ipomoea chryseides*** Ker Gawl. Bot. Reg. 4: t. 270 1818. (Ker-Gawler 1818a: t. 270) = *Merremia chryseides* (Ker Gawl.) Hallier f.

***Ipomoea cissoides*** (Lam.) Griseb., Fl. Brit. W.I. 473. 1864 [pub. 1862]. (Grisebach 1862b: 473), including infraspecific taxa = *Distimake cissoides* (Lam.) A.R. Simões & Staples

***Ipomoea codonantha*** Benth., Pl. Hartw. 120. 1843. (Bentham 1839–57 : 46). = *Operculina codonantha* (Benth.) Hallier f.

***Ipomoea compressa*** Guss., Ind. Sem. Hort. Boccadifalco 7. 1825 (Gussone 1825: 7). Type. Plant grown from seeds sent from “India” (whereabouts unknown).

Uncertain species, possibly not of American origin. The description is too vague to allow identification.

***Ipomoea contorquens*** Choisy in A.P. de Candolle, Prodr. 9: 385. 1845. (Choisy 1845: 385) and infraspecific taxa = *Distimake digitatus* (Spreng.) A.R. Simões & Staples var. (Merremia digitata var. elongata (Choisy) D.F. Austin & Staples)

***Ipomoea coptica*** (L.) Roth ex Roem. & Schult., Syst. Veg. 4: 208. 1819. (Roemer and Schultes 1819: 208).

This African species is reported to occur as an adventive in Hawaii and The United States (St John, 1973), but no specimens have been traced and no mention of this species is made in later publications, such as Wagner et al. (1999).

***Ipomoea cordobana*** Peter, Peter, Nat. Pflanzenfam. [Engler & Prantl] 4(3a): 36. 1891. (Peter 1891: 36) = *Convolvulus bonariensis* Cav.

***Ipomoea corralinensis*** Choisy in A.P. de Candolle, Prodr. 9: 361. 1845. (Choisy 1845: 361) = *Merremia* sp. fide Austin et al. (2015).

***Ipomoea crotonifolia*** Gardner, London J. Bot. 1: 180. 1842. (Gardner 1842c: 180) = *Jacquemontia holosericea* (Weinm.) O’Donell

***Ipomoea cruckshanksii*** Choisy in A.P. de Candolle, Prodr. 9: 389. 1845. (Choisy 1845: 389). = *Evolvulus alsinoides* L.

***Ipomoea cumanensis*** (Kunth) G. Don, Gen. Hist. 4: 273. 1838 (Don 1838: 273) = *Jacquemontia cumanensis* (Kunth) Kuntze

***Ipomoea dasysperma*** var. ***disperma*** Ram. Goyena, Fl. Nicarag. 2: 652. 1911 (Ramírez Goyena 1911: 652). Type. NICARAGUA. No specimen cited.

Unknown species with yellow flowers and two black seeds, possibly *Camonea umbellata* (L.) A.R. Simões & Staples

***Ipomoea digitata*** D. Parodi, Contr. Fl. Parag. 26. 1877. (Parodi 1877: 26), nom. illeg., non *Ipomoea digitata* L. (1759). Type. PARAGUAY. Asunçion and Cordillera = A plant of Clade A2, possibly *I. platensis* Ker-Gawl.

***Ipomoea discoidesperma*** Donn.-Sm, Bot. Gaz. 14: 27. 1889. (Donnell Smith 1889: 27) = *Merremia discoidesperma* (Donn.-Sm.) O’Donell

***Ipomoea dissecta*** (Jacq.) Pursh, Fl. Amer. Sept. 1: 145. 1813. (Pursh 1813: 145), non *Ipomoea dissecta* Willd. (1794), including infraspecific taxa = *Distimake dissectus* (Jacq.) A.R. Simões & Staples

***Ipomoea distans*** Choisy in A.P. de Candolle, Prodr. 9: 378. 1845. (Choisy 1845: 378). Type. FRENCH GUIANA. *Perrottet* s.n. (P, not found).

An uncertain species of Clade A2, possibly *I. batatoides*.

***Ipomoea ebracteata*** (Desr.) Choisy in A.P. de Candolle, Prodr. 9: 377. 1845. (Choisy 1845: 377). Based on *Convolvulus ebracteatus*, which is probably *Ipomoea biflora* (L.) Pers.

No specimen of *Ipomoea biflora* has been seen from the New World and the specimens cited by Choisy (1845) probably represent a different species.

***Ipomoea elegans*** A. Dietr., Allg. Gartenzeitung (Otto & Dietrich) 4: 313. 1836 (Dietrich 1836: 313).

Uncertain species, probably *I. platensis* Ker-Gawl. (see Wood and Scotland 2017a: 9).

***Ipomoea emetica*** Choisy in A.P. de Candolle, Prodr. 9: 376. 1845. (Choisy 1845: 376). Type. MEXICO. Holotype, icon, Sessé & Moçiño, Icones Florae Mexicanae 37 sub “Ipomoea sagittata”.

Uncertain species. Choisy based his description solely on the painting prepared by Sessé and Moçiño and saw neither the original specimen (now lost) nor Sessé and Moçiño’s manuscript description of *Ipomoea sagittata*. The description of solitary flowers, sagittate leaves and thickened rootstock used as an emetic suggests this is *Ipomoea simulans*, but there can be no certainty as the picture lacks diagnostic details. House (1908b: 241) and Matuda (1966a: 85) treated it, not unreasonably, as conspecific with *Ipomoea caudata* but it has subsequently been treated as the correct name for *I. decasperma* (Austin and Huáman 1996) or as a distinct species related to *I. decasperma* (Carranza 2007). We can see no reason for either of these decisions and material named *I. emetica* at IEB by Carranza seems to belong to *I. decasperma*. We regard *Ipomoea emetica* as a name of uncertain application, which is best abandoned (Wood and McDonald 2018).

***Ipomoea erecta*** Michx., in Lam., Journ. Hist. Nat. i. 410. 1792. (Michaux 1792: 410) = *Ipomopsis rubra* (L.) Wherry (Polemoniaceae)

***Ipomoea ericoides*** Meisn. in Martius et al., Fl. Brasil. 7: 251. 1869 (Meisner 1869: 251). = *Distimake digitatus* (Spreng.) A.R. Simões & Staples var. (Merremia digitata var. ericoides (Meisn.) D.F. Austin & Staples)

***Ipomoea eriocephala*** Moric., Pl. Nouv. Amer. 43, t. 29. 1837. (Moricand 1834–47: 43) = *Odonellia eriocephala* (Moric.) K.R. Robertson

***Ipomoea erythraea*** Sessé & Moçiño ex Choisy in A.P. de Candolle, Prodr. 9: 335. 1845. (Choisy 1845: 335), nom.nud. = *Ipomoea* sp. of the Quamoclit Clade.

***Ipomoea evolvuloides Moric.***, Pl. Nouv. Amer. 47. 1837. (Moricand 1834–47: 47) = *Jacquemontia evolvuloides* (Moric.) Meisn.

***Ipomoea evolvuloides*** var. ***grandiflora*** Choisy in A.P. de Candolle, Prodr. 9: 361. 1845. (Choisy 1845: 361) = *Jacquemontia evolvuloides* (Moric.) Meisn.

***Ipomoea falkioides*** Griseb., Cat. Pl. Cub. 206. 1866. (Grisebach 1866: 206). Type. CUBA. “Orientalis”, *C. Wright s.n.* (isotypes GH, K, NY, YU).

A slender erect, subscapose herb 3–5 cm high with a pubescent stem. Leaves mostly at base of stem, 0.7–1.5 × 0.6–1.1 cm, ovate, cordate, obtuse, margin undulate, glabrous, abaxially punctate; petioles 2–5 mm, pubescent. Inflorescence of solitary, pedunculate axillary flowers; peduncles 1.5–3.5 cm; bracteoles and pedicels apparently absent; sepals 3 × 0.5 mm, oblong, acute, green and foliose in texture, sparsely and minutely hispidulous; corolla broadly funnel shaped, 1 cm long, ?white, glabrous apart from a few hairs at apex of midpetaline bands.

Uncertain species only known from fragile type collections. We have not been able to revise the pollen or see the stigmas clearly, so it is difficult to evaluate its generic position. However, it is quite unlike any *Ipomoea* known to us and has something of the appearance of a depauperate specimen of *Jacquemontia*.

***Ipomoea ferruginea*** (Vahl) Roem. & Schult., Syst. Veg. 4: 240. 1819. (Roemer and Schultes 1819: 240) = *Jacquemontia cumanensis* (Kunth) Kuntze

***Ipomoea filiformis*** Jacq., Enum. Syst. Pl. 13. 1760. (Jacquin 1760: 13) = *Jacquemontia solanifolia* (L.) Hallier f.

***Ipomoea filipedunculata*** Rusby, Bull. Torrey Bot. Club 26(3): 150. 1899. (Rusby 1899: 150) = *Jacquemontia blanchetii* Moric.

***Ipomoea flagellaris*** Choisy, Mém. Soc. Phys., Genève 6: 60 [138]. 1838. (Choisy 1838: 60 [138]) = *Distimake flagellaris* (Choisy) A.R. Simões & Staples

***Ipomoea flammea*** Nees, Flora 4: 301. 1821. (Nees ab Esenbeck 1821: 301) = *Merremia* sp.

***Ipomoea floribunda*** (Kunth) G. Don, Gen. Hist. 4: 267. 1838. (Don 1838: 267) = *Jacquemontia floribunda* (Kunth) Hallier f.

***Ipomoea fulva*** Bertol., Hort. Bot. Bonon. 1826: 5. 1826. (Bertoloni 1826: 5) = Distimake dissectus var. edentata (Meisn.) Petrongari & Sim.-Bianch.

***Ipomoea fusca*** Meisn. in Martius et al., Fl. Brasil. 7: 247. 1869. (Meisner 1869: 247) = *Jacquemontia fusca* (Meisn.) Hallier f.

***Ipomoea gabrielii*** Choisy in A.P. de Candolle, Prodr. 9: 378. 1845. (Choisy 1845: 378) = *Jacquemontia gabrielii* (Choisy) Buril.

***Ipomoea glabra*** (Aubl.) Choisy in A.P. de Candolle, Prodr. 9: 362. 1845. (Choisy 1845: 362) = *Distimake macrocalyx* (Ruiz & Pav.) A.R. Simões & Staples

**Ipomoea glabra var. septenata** (Choisy) Meisn. in Martius et al., Fl. Brasil. 7: 287. 1869. (Meisner 1869: 287) = *Ipomoea septenata* Choisy (see below).

***Ipomoea glaucifolia*** L., Sp. Pl. 1: 161. 1753. (Linnaeus 1753: 161), nom. rej = probably *Convolvulus equitans* Benth.

***Ipomoea glaziovii*** Dammer, Bot. Jahrb. Syst. 23, Beibl. 57: 40. 1897. (Dammer 1897: 40) = *Distimake tuberosus* (L.) A.R. Simões & Staples

***Ipomoea glutinosa*** Dammer, Bot. Jahrb. Syst. 23, Beibl. 57: 39. 1897. (Dammer 1897: 39) = *Jacquemontia* sp.

***Ipomoea graminiformis*** var. ***densiflora*** Chodat & Hassl., Bull. Herb. Boissier ser. 2, 5: 690. 1905. (Chodat and Hassler 1905: 690) = *Jacquemontia densiflora* (Chodat & Hassl.) Hassl.

***Ipomoea graminiformis*** forma ***minor*** Chodat & Hassl., Bull. Herb. Boissier ser. 2, 5: 690. 1905. (Chodat and Hassler 1905: 690) = *Jacquemontia densifolia* Chodat & Hassler”

***Ipomoea grandidentata*** C.H. Thomps., Trans. Acad. Sci. St. Louis 20: 18. 1911. (Thompson 1911: 18) = *Merremia grandidentata* (C.H. Thomps.) Staples & A.R. Simões

***Ipomoea grandiflora*** D. Parodi, Contr. Fl. Parag. 21. 1877. (Parodi 1877: 21), nom. illeg., non *Ipomoea grandiflora* Lam. (1793). Type. PARAGUAY. Cordillera (whereabouts unknown).

An uncertain species but probably *I. maurandioides*, which is common in Cordillera. The description of a slender, glabrous, often 1-flowered plant with emarginate (presumably sagittate) leaf bases fits well.

***Ipomoea granulata*** D. Parodi, Contr. Fl. Parag. 17. 1877. (Parodi 1877: 17). Type. PARAGUAY. [Canindeyú] Río Igatimi (whereabouts unknown).

An uncertain species, but very probably *I. granulosa* as the stem is described as granulose and the leaves narrowly elliptical and subsessile as in *I. granulosa*, which is known from Canindeyú. We hesitate to use the name *I. granulata* without seeing authentic material.

***Ipomoea guyanensis*** (Aubl.) Choisy in A.P. de Candolle, Prodr. 9: 366. 1845. (Choisy 1845: 366) = *Jacquemontia guyanensis* (Aubl.) Meisn.

***Ipomoea hamiltonii*** G. Don, Gen. Hist. 4: 268. 1838. (Don 1838: 268) = *Operculina hamiltonii* (G. Don) D.F. Austin & Staples

***Ipomoea hassleriana*** Chodat & Hassl., Bull. Herb. Boissier ser. 2, 5: 693. 1905. (Chodat and Hassler 1905: 693), including infraspecific taxa = *Distimake hasslerianus* (Chodat & Hassl.) A.R. Simões & Staples

***Ipomoea havanensis*** (Jacq.) Choisy in A.P. de Candolle, Prodr. 9: 368. 1845. (Choisy 1845: 368) = *Jacquemontia havanensis* (Jacq.) Urban

***Ipomoea hermanniae*** (L’Hérit.) G. Don, Gen. Hist. 4. 276. 1838 (Don 1838: 276) = *Convolvulus hermanniae* L’Hérit.

***Ipomoea heterophylla*** Schrank, Denkschr. Bot. Ges. Regensb. 2: 3. 1822. (Schrank 1822: 31). Type. Cultivated plant sent by Martius from Brazil.

Uncertain species. The 5-lobed dentate leaves suggest this is a species of *Merremia* or *Distimake*.

***Ipomoea hirtiflora*** M. Martens & Galeotti, Bull. Acad. Roy. Sci. Bruxelles 12: 267. 1845. (Martens and Galeotti 1845: 267) = *Odonella hirtiflora* (M. Martens & Galeotti) K.R. Robertson

***Ipomoea hispaniolae*** G. Don, Gen. Hist. 4: 280. 1838. (Don 1838: 280) = *Distimake quinquefolius* (L.) A.R. Simões & Staples

***Ipomoea hispida*** D. Parodi, Contr. Fl. Parag. 18. 1877. (Parodi 1877: 18), nom. illeg., non *Ipomoea hispida* Zuccagni (1810). Type. PARAGUAY. “Ñanduracaí” (whereabouts unknown).

Uncertain species, the protologue is insufficient to suggest a possible identification.

***Ipomoea holosericea*** Weinm., Syll. Pl. Nov. 2: 17. 1828. (Weinmann 1828: 17) = *Jacquemontia holosericea* (Weinm.) O’Donell

***Ipomoea hostmannii*** Meisn. in Martius et al., Fl. Brasil. 7: 290. 1869. (Meisner 1869: 290) = *Distimake macrocalyx* (Ruiz & Pav.) A.R. Simões & Staples

***Ipomoea humilis*** Raf. New Flora 4: 57. 1838. (Rafinesque 1838b: 57). Type. Not specified, a plant occurring in Florida and Cuba (Herb. Collins, whereabouts unknown).

Uncertain species. A slender annual herb with palmately 5-lobed leaves, sepals ciliate, corolla red. Possibly *I. heptaphylla*.

***Ipomoea igatimiana*** D. Parodi, Contr. Fl. Parag. 17. 1877. (Parodi 1877: 17). Type. PARAGUAY. [Canindeyú] Río Igatimi (whereabouts unknown).

An uncertain species, possibly *I. setifera* because of the paired, persistent bracts and verruculose calyx, or pehaps *I. fimbriosepala*.

***Ipomoea imbricata*** D. Parodi, Contr. Fl. Parag. 16. 1877. (Parodi 1877: 16), nom. illeg, non *Ipomoea imbricata* Roth (1821). Type. PARAGUAY. [Canindeyú] Río Igatimi (whereabouts unknown).

An uncertain species but probably *Ipomoea paludosa*, because of the erect simple stems and imbricate leaves.

***Ipomoea jamesonii*** Choisy in A.P. de Candolle, Prodr. 9: 367. 1845. (Choisy 1845: 367) = *Aniseia luxurians* (Moric.) Athiê-Souza & Buril

***Ipomoea juncea*** Choisy in A.P. de Candolle, Prodr. 9: 355. 1845. (Choisy 1845: 355) = *Distimake aturensis* (Kunth) A.R. Simões & Staples

***Ipomoea kunthiana*** var. ***pubescens*** Meisn. in Martius et al., Fl. Brasil. 7: 253. 1869. (Meisner 1869: 253). Type. BRAZIL. Location not specified.

Uncertain species. *Ipomoea procumbens* (incl. *I. kunthiana*) is always glabrous so this must presumably be another species, which we are unable to identify.

***Ipomoea lanceolata*** G. Don, Gen. Hist. iv. 282. 1838. (Don 1838: 282) = *Aniseia martinicensis* (Jacq.) Choisy

***Ipomoea lasioclados*** Choisy, Choisy in A.P. de Candolle, Prodr. 9: 357. 1845. (Choisy 1845: 357) = *Jacquemontia lasioclados* (Choisy) O’Donell

***Ipomoea ledebourii*** Choisy in A.P. de Candolle, Prodr. 9: 388. 1845. (Choisy 1845: 388). Type. BRAZIL. Based on *Calystegia cymosa* Ledeb. in Index Hort. Dorpat 4 (1821).

An uncertain species. We have not been able to trace a specimen and the description lacks sufficient detail to allow identification. Although Choisy states that *Ipomoea ledebourii* is based on *Calystegia rugosa* Ledeb., this must be an error as he quotes Ledebour’s original description of *Calystegia cymosa* word for word and, in any case, this is the only *Calystegia* species mentioned by Ledebour on page 4.

***Ipomoea leiocalyx*** Bruns, Mitt. Inst. Bot. Hamburg 8: 66. 1929. (Bruns 1929: 66), nom. nud. Based on *Günther & Buchtien* 164 (B†).

***Ipomoea lindmanii*** Urb., Symb. Antill. 9: 248. 1924. (1924: 248). Type. CUBA (east). Mir, *E. Ekman* 7508 (holotype S07-4660).

Uncertain species. The type is a sterile shoot and no flowering or fruiting material has ever been found and it cannot even be certain that it is a species of *Ipomoea* (Wood and Scotland 2017c).

***Ipomoea linoides*** Choisy in A.P. de Candolle, Prodr. 9: 354. 1845. (Choisy 1845: 354), including infraspecific taxa = *Jacquemontia linoides* (Choisy) Meisn.

***Ipomoea livescens*** (Schlecht. ex Kunze) Meisn. in Martius et al., Fl. Brasil. 7: 224. 1869. (Meisner 1869: 224). Type. Based on *Pharbitis livescens* (see below).

***Ipomoea longiflora*** Larrañaga, Escritos Damaso Antonio Larranaga 2: 78.1923. (Larrañaga 1923: 78). Type. Not specified. Unknown species from Uruguay, not identifiable from the protologue

***Ipomoea longipes*** Garke, Linnaea 22: 66. 1849. (Garcke 1849: 66) = *Camonea umbellata* (L.) A.R. Simões & Staples

***Ipomoea lundii*** Choisy, Mém. Soc. Phys., Genève 6: 56 [134]. 1838. (Choisy 1838: 6 [134]) = *Bonamia agrostopolis* (Vell.) Hallier f.

***Ipomoea luxurians*** Moric., Pl. Nouv. Amer. 58. t. 39. 1839. (Moricand 1834–47: 58) = *Aniseia luxurians* (Moric.) Athiê-Souza & Buril

***Ipomoea malvaeoides*** var. ***oblongifolia*** Hallier f. in Pilger, Bot. Jahrb. 30: 185 (1902). Type. BRAZIL. Mato Grosso, Cuyabá, *Pilger* 318.

Uncertain species, possibly *I. haenkeana*, based on the cited flowering season and dry habitat, the entire, oblong, abaxially tomentellous leaves, paniculate inflorescence and shortly tomentose sepals.

***Ipomoea nealleyi*** Coult., Contr. U.S. Natl. Herb. 2: 46. 1890. (Coulter 1890: 46). = ? *Antirrhinum maurandioides* A. Gray

***Ipomoea nematophylla*** Urb., Symb. Ant. 5: 473 (1908). (Urban 1908: 473). Type. HAITI. Petite Rivière de Bayonnais, *W. Buch* 900 (?B†)

A glabrous plant with simple linear-filiform leaves, the margins incurved, but otherwise similar to the variable *I. nematoloba*. Urban claimed the corolla was 2.5 cm long but was unable to observe it fully. Uncertain species. In the absence of a type specimen it is very uncertain how this species differs from the very variable *I. nematoloba* except by the leaf shape. Urban identified *E.L. Ekman* 9452 (S) as this species but it is leafless and in fruit. No other more recent collection has been seen that approximates to the type.

***Ipomoea nigricans*** Gardner, London J. Bot. 1: 180. 1842. (Gardner 1842c: Distimake dissectus var. edentatus (Meisn.) Petrongari & Sim.-Bianch.

***Ipomoea nutans*** Choisy in A.P. de Candolle, Prodr. 9: 368. 1845. (Choisy 1845: 368) = *Calycobolus nutans* (Choisy) D.F. Austin

***Ipomoea nyctaginea*** Choisy in A.P. de Candolle, Prodr. 9: 369. 1845. (Choisy 1845: 369) = *Ipomoea* sp. (African species of the Astripomoea Clade)

***Ipomoea nyctelea*** L., Sp. Pl. 1: 160. 1753 (Linnaeus 1753: 160) = *Ellisia nyctelea* (L.) L. (Hydrophyllaceae)

***Ipomoea operculata*** Mart., Reise Bras. (Spix & Mart.) 1: 547. 1823. (Spix and Martius 1823: 547) = *Operculina macrocarpa* (L.) Urb.

***Ipomoea ornithopoda*** B.L. Rob., Proc. Amer. Acad. Arts 27: 183. 1892 (Robinson 1892: 183) = *Operculina pinnatifida* (Kunth) O’Donell

***Ipomoea ottoensis*** Choisy in A.P. de Candolle, Prodr. 9: 378. 1845. (Choisy 1845: 378) = *Convolvulus crenatifolius* Ruiz & Pav.

***Ipomoea ovalifolia*** (Vahl ex H. West) Choisy, Mém. Soc. Phys. Genève 6: 449 [67]. 1834. (Choisy 1834: 449 [67]) = *Jacquemontia ovalifolia* (Vahl ex H. West) Hallier f.

***Ipomoea ovalifolia*** var. ***pubescens*** Choisy in A.P. de Candolle, Prodr. 9: 357. 1845. (Choisy 1845: 357) = *Jacquemontia ovalifolia* (Vahl ex H. West) Hallier f.

***Ipomoea ovalifolia*** var. ***tomentosa*** Choisy in A.P. de Candolle, Prodr. 9: 357. 1845. (Choisy 1845: 357) = *Jacquemontia sandwicensis* A. Gray

***Ipomoea palmeri*** S. Watson, Proc. Amer. Acad. Arts 24: 63. 1889 (Watson 1889: 63) = *Distimake palmeri* (S. Watson) A.R. Simões & Staples.

***Ipomoea palmeri*** var. ***platyphylla*** Fernald, Proc. Amer. Acad. Arts 33(5): 90. 1897. (Fernald 1897: 90) = *Merremia platyphylla* (Fernald) O’Donell

***Ipomoea papillosa*** Bertol., Hort. Bot. Bonon. 1826: 5. 1826. (Bertoloni 1826: 5). Type. A cultivated plant of Brazilian origin (whereabouts unknown).

Uncertain species.

***Ipomoea patula*** Choisy in A.P. de Candolle, Prodr. 9: 368. 1845. (Choisy 1845: 368) = *Ipomoea crassipes* Hook., an African species. See Wood and Scotland (2017a: 9).

***Ipomoea patula*** var. ***selloana*** Meisn. in Martius et al., Fl. Brasil. 7: 240. 1869. (Meisner 1869: 240). Type. BRAZIL. *F. Sello* s.n. (whereabouts unknown).

An unidentified species of *Ipomoea* (Wood and Scotland 2017a: 10).

***Ipomoea pendula*** (Silva Manso) Stellfeld, Tribuna Farm., Curitiba 13: 86. 1945. (Stellfeld 1945: 86), nom. illeg, non *Ipomoea pendula* R. Br. (1810). Type. Based on *Convolvulus pendulus* Silva Manso = *Ipomoea utilis*Choisy (1845: 375).

***Ipomoea pentaphylla*** (L.) Jacq., Collectanea 2: 297. 1789 [dated1788]. (Jacquin 1789: 297) = *Distimake aegyptius* (L.) A.R. Simões & Staples

***Ipomoea perryana*** Duchass. & Walp., Linnaea 23: 751. 1850 [pub. 1851]. (Duchassaing and Walpers 1850–1851: 751) = *Odonellia hirtiflora* (M.Martens & Galeotti) K.R. Robertson

***Ipomoea pilosa*** Cav., Icon. [Cavanilles] 4. 11. 1797. (Cavanilles 1797–1798: 11) = *Distimake aegyptius* (L.) A.R. Simões & Staples

***Ipomoea pinnatifida*** (Kunth) G. Don, Gen. Hist. 4: 280. 1838. (Don 1838: 280) = *Operculina pinnatifida* (Kunth) O’Donell

***Ipomoea polyanthes*** Roem. & Schult., Syst. Veg. 4: 234. 1819. (Roemer and Schultes 1819: 234) = *Camonea umbellata* (L.) A.R. Simões & Staples

***Ipomoea polymorpha*** var. ***glabra*** Griseb., Abh. Königl. Ges. Wiss. Göttingen 24: 264. 1879. (Grisebach 1879: 264) = *Convolvulus laciniatus* Desr.

***Ipomoea polyrrhizos*** (Silva Manso) Choisy in A.P. de Candolle, Prodr. 9: 356. 1845. (Choisy 1845: 356).

Uncertain species. The description of an erect, sericeous plant with oblong-obovate, mucronate leaves, 3-flowered peduncles, a rounded, sericeous calyx and a terminal, inflorescence of pale rose flowers and pilose seeds strongly suggests a cerrado species such as *Ipomoea haenkeana* but it is impossible to be completely certain which species is indicated from the protologue,

***Ipomoea portobellensis*** Beurl., Kongl Vetensk. Acad. Handl. 40: 139. 1854 [pub. 1856]. (Beurling 1856: 139) = *Camonea umbellata* (L.) A.R. Simões & Staples

***Ipomoea potentilloides*** Meisn. in Martius et al., Fl. Brasil. 7: 230. 1869. (Meisner 1869: 230) = *Distimake quinquefolius* (L.) A.R. Simões & Staples

***Ipomoea primuliflora*** G. Don, Gen. Hist. iv. 270. 1838. (Don 1838: 270) = *Camonea umbellata* (L.) A.R. Simões & Staples

***Ipomoea prostrata*** Meisn. in Martius et al., Fl. Brasil. 7: 254. 1869. (Meisner 1869: 254) = *Jacquemontia warmingii* O’Donell

***Ipomoea prostrata*** var. ***longepedunculata*** Chodat & Hassl., Bull. Herb. Boissier, sér. 2, 5: 692. 1905 (Chodat and Hassler 1905: 692) = *Jacquemontia* sp.

***Ipomoea pterodes*** Choisy in A.P. de Candolle, Prodr. 9: 361. 1845. (Choisy 1845: 361) = *Operculina hamiltonii* (G. Don) D.F. Austin & Staples

***Ipomoea pubescens*** Hornem. Hort. Bot. Hafn. i. 195. 1813. (Hornemann 1813: 195). Type. Specimen of unknown origin, cultivated at C, a syntype sent by Willdenow.

Uncertain species, possibly *I. pubescens* (L.) Roth

***Ipomoea punicea*** (Silva Manso) Choisy in A.P. de Candolle, Prodr. 9: 355. 1845. (Choisy 1845: 355).

Uncertain species. The description in the protologue of “an erect, hirsute plant covered in white hairs with decussate, lanceolate, subsessile, obtuse leaves, short 1–5-flowered peduncles, pilose, obovate, acute sepals and purple flowers” suggests this might be *I. hirsutissima*, *I. aurifolia* or a similar species.

***Ipomoea quinquefolia*** L., Sp. Pl. 1: 162. 1753 (Linnaeus 1753: 162) = *Distimake quinquefolius* (L.) A.R. Simões & Staples

***Ipomoea quinqueloba*** Sessé & Moçiño, Pl. Nov. Hisp. 27 1888. (Sessé and Moçiño 1887–1890: 27). Type. MEXICO (not found at MA).

Uncertain species.

***Ipomoea quinquepartita*** (Vahl) Roem. & Schult., Syst. Veg. 4: 247. 1819. (Roemer and Schultes 1819: 247) = *Jacquemontia obcordata* (Millsp.) House fide Austin et al. (2015).

***Ipomoea repens*** (L.) Lam., Tabl. Encycl. 1: 467. 1793. (Lamarck 1793: 467) = *Calystegia sepium* (L.) R.Br.

***Ipomoea rhodocalyx*** A. Gray ex S. Watson, Proc. Amer. Acad. Arts 22: 439. 1887. (Watson 1887: 439) = *Operculina pteripes* (G. Don) O’Donell

***Ipomoea robusta*** Urb., Symb. Antill. 9: 424. 1925. (Urban 1925: 424). Type. CUBA. Pinar del Río, *E. Ekman* 18220 (holotype S07-4777).

Uncertain species. The type is a relatively unremarkable sterile shoot. While this could be a species of *Ipomoea*, it cannot be matched with any known species. (Wood and Scotland 2017c).

***Ipomoea ruderaria*** (Kunth) G. Don, Gen. Hist. 4: 267. 1838. (Don 1838: 267) = *Jacquemontia havanensis* (Jacq.) Urb.

***Ipomoea sagittata*** Sessé & Moçiño Pl. Nov. Hisp. 27 1888. (Sessé and Moçiño 1887–1890: 27), nom. illeg., non *Ipomoea sagittata*Poiret (1789). Type. MEXICO. “Sancti Angeli montibus prope Mexicum” (not found at MA).

Uncertain species, possibly *Ipomoea simulans* D. Hanb. See also *Ipomoea emetica*.

***Ipomoea sagittifera*** (Kunth) G. Don, Gen. Hist. 4: 273. 1838. (Don 1838: 273) = *Camonea umbellata* (L.) A.R. Simões & Staples

***Ipomoea salicifolia*** Desr. ex Steud., Nomencl. Bot. 1: 819. 1840. (Steudel 1840: 819) = *Aniseia martinicensis* (Jacq.) Choisy

***Ipomoea scabra*** Schult., Obs. Bot. 37. 1809. (Schultes 1809: 37), nom. illeg. non *Ipomoea scabra*Forsskal (1775). Type. Specimen sent to Willdenow by Witiskiewicz.

If this specimen is B-W03763-01, this species is I. purpurea var. diversifolia as identified by Hallier bur the peduncles are not “subuniflorus” as stated by Schultes. The protologue and the specimen may not correspond.

***Ipomoea scabrida*** Roem. & Schult., Syst. Veg. 4: 223. 1819. (Roemer and Schultes 1819: 223). Type. Based on *I. scabra* Schult. (see above).

***Ipomoea schizoloma*** Kunze, Del. Sem. Hort. Lips. 2. 1845. (Kunze 1845: 2). Type. A cultivated plant grown at Leipzig with seeds from “Bonaria” (? Buenos Aires) (?LZ†). Annual resembling *I. orizabensis*. Stems hirsute; calyx black-strigose at base, Cymes 3–4 flowered; corolla tube red.

Uncertain species with no extant type.

***Ipomoea selloi*** Meisn. in Martius et al., Fl. Brasil. 7: 271. 1869. (Meisner 1869: 271) = *Jacquemontia selloi* (Meisn.) Hallier f.

***Ipomoea selloi*** var. ***rufescens*** Meisn. in Martius et al., Fl. Brasil. 7: 271. 1869. (Meisner 1869: 271) = *Jacquemontia selloi* (Meisn.) Hallier f.

***Ipomoea septenata*** Choisy in A.P. de Candolle, Prodr. 9: 362. 1845. (Choisy 1845: 362). Based on *Convolvulus glaber* = *Distimake macrocalyx* (Ruiz & Pav.) A.R. Simões & Staples

***Ipomoea sericantha*** Griseb., Fl. Brit. W.I. [Grisebach] 471. 1862. (Grisebach 1862b: 471) = *Aniseia luxurians* (Moric.) Athiê-Souza & Buril

***Ipomoea sericantha*** Miq., Stirp. Surinam. Select. 131. 1851 (Miquel 1851: 131) = *Operculina sericantha* (Miq.) Ooststr.

***Ipomoea sericea*** Spreng. ex Choisy in A.P. de Candolle, Prodr. 9: 368. 1845. (Choisy 1845: 368) = *Aniseia luxurians* (Moric.) Athiê-Souza & Buril

***Ipomoea sericea*** D. Parodi, Contr. Fl. Parag. 18. 1877. (Parodi 1877: 18), nom. illeg., non *Ipomoea sericea* (L.) Blume (1826). Type. PARAGUAY. [Canindeyú] Río Igatimi (whereabouts unknown).

An uncertain species, but the description of shortly petiolate, ovate, basally rounded and abaxially pilose leaves with solitary flowers suggests it could be *I. chodatiana*, especially as the type of this species was collected at the same locality.

***Ipomoea serpyllifolia*** (Kunth) G. Don, Gen. Hist. 4: 267. 1838. (Don 1838: 267) = *Jacquemontia serpyllifolia* (Kunth) Urb.

***Ipomoea serrata*** Choisy, Mém. Soc. Phys., Genève 6: 41 [135]. 1838. (Choisy 1838: 41 [135]), including infraspecific taxa = *Jacquemontia serrata* (Choisy) Meisn.

***Ipomoea sidifolia*** Choisy, Mém. Soc. Phys. Genève 6: 459 [77]. 1834. (Choisy 1834: 459 [77)], nom. illeg., non *Ipomoea sidifolia* Schrad. (1821). Type. Based on various disparate elements including *Convolvulus sidifolius* Kunth and *C. domingensis* Desr.

***Ipomoea silvana*** Choisy in A.P. de Candolle, Prodr. 9: 374. 1845. (Choisy 1845: 374) = Operculina turpethum var. ventricosa (Bertero) Staples & D. Austin

***Ipomoea sinaloensis*** Brandegee, Zoë 5: 217. 1905. (Brandegee 1905: 217) = *Distimake aegyptius* (L.) A.R. Simões & Staples

***Ipomoea sinuata*** Ortega, Nov. Pl. Descr. Dec. 84. 1798. Ortega 1797–1800: 84), including infraspecific taxa = *Distimake dissectus* (Jacq.) A.R. Simões & Staples

***Ipomoea solanifolia*** L., Sp. Pl. 1: 161. 1753. (Linnaeus 1753: 161) = *Jacquemontia solanifolia* (L.) Hallier f.

***Ipomoea soldanellifolia*** Schrank, Syll. Pl. Nov. 1: 198. 1824. (Schrank 1824: 198). Type. BRAZIL. “inter Bahiam und Maragnonum”, *Martius* s.n. (whereabouts unknown).

Uncertain species with orbicular, pubescent leaves and blue flowers.

***Ipomoea sphaerostigma*** (Cav.) Steud., Nomencl. Bot. 1: 819. 1840 (Steudel 1840: 819) = *Jacquemontia sphaerostigma* (Cav.) Rusby

***Ipomoea spiciflora*** Choisy, Mém. Soc. Phys., Genève 6: 54 [148]. 1838. (Choisy 1838: 54 [148]) = *Jacquemontia spiciflora* (Choisy) Hallier f.

***Ipomoea stellata*** D. Parodi, Contr. Fl. Parag. 18. 1877. (Parodi 1877: 18). Type. PARAGUAY. [Canindeyú] Río Igatimi (whereabouts unknown).

Uncertain species. The description of a plant with stellate hairs indicates that this must either be *Ipomoea bonariensis* or *I. homotrichoidea*. The former is more common in Paraguay but only the latter is definitely recorded from Canindeyú.

***Ipomoea sulcata*** D. Parodi, Contr. Fl. Parag. 19. 1877. (Parodi 1877: 19). Type. PARAGUAY. [Canindeyú] Río Igatimi (whereabouts unknown).

Uncertain species. The description of a glabrous prostrate plant with shortly petiolate, elliptic, rounded leaves combined with a pubescent calyx and large solitary flowers does not fit any Paraguayan species known to us.

***Ipomoea superba*** Schrank ex Colla, Hort. Ripul. append. 2: 350–351. 1826. (Colla 1826a: 350–351). Type. A cultivated plant of unknown origin sent to Colla by Schrank.

Uncertain species, possibly *Ipomoea orizabensis*.

***Ipomoea tamnifolia*** L., Sp. Pl. 1: 162. 1753. (Linnaeus 1753: 162) = *Jacquemontia tamnifolia* (L.) Griseb.

***Ipomoea terminalis*** Choisy, Mém. Soc. Phys., Genève 6: 54 [132]. 1838. (Choisy 1838: 54 [132]) = *Bonamia agrostopolis* (Vell.) Hallier f.

***Ipomoea tomentosa*** Choisy, Mém. Soc. Phys., Genève 6: 55 [133]. 1838. (Choisy 1838: 55 [133]) = *Merremia tomentosa* (Choisy) Hallier f.

***Ipomoea tortugensis*** Peter, Nat. Pflanzenfam. 4, 3a: 31. 1891. (Peter 1891: 31) = *Distimake aegyptius* (L.) A.R. Simões & Staples

***Ipomoea trichocephala*** G. Don, Gen. Hist. 4: 269. 1838 (Don 1838: 269) = *Jacquemontia tamnifolia* (L.) Raf.

***Ipomoea triflora*** Maria & Velasco, La Naturaleza 1: 338, 1870. (Maria and Velasco 1870: 338) nom. illeg., non *Ipomoea triflora* Forsskal (1776). Type. MEXICO. Querétaro, no type known.

Uncertain species, possibly related to *I. orizabensis*.

***Ipomoea triloba*** var. ***glaberrima*** Meisn. in Martius et al., Fl. Brasil. 7: 277. 1869. (Meisner 1869: 277). Type. BRAZIL. Various types cited.

Uncertain species. Probably a form of *I. australis* or *I. grandifolia* with a glabrous capsule.

***Ipomoea triquetra*** (Vahl) Roem. & Schult., Syst. Veg., 4: 231. 1819. (Roemer and Schultes 1819: 231), including infraspecific taxa = *Operculina turpethum* (L.) Silva Manso

***Ipomoea tuberosa*** L., Sp. Pl. 1: 160. 1753. (Linnaeus 1753: 160), including infraspecific taxa = *Distimake tuberosus* (L.) A.R. Simões & Staples

***Ipomoea tweediei*** Hook., Bot. Mag. 69: t. 3978. 1842. (Hooker WJ 1842: t. 3978).

An *Ipomoea* of uncertain application. The type collection at K consists of two plants, one possibly *I. grandifolia*, the other possibly *I. indivisa* or *I. rubriflora* but the illustration (Bot. Mag. t. 3978) and description do not fit either. This species is sometimes treated as a synonym of *I. aristolochiifolia* but neither the illustration nor the protologue fits (O’Donell 1959b: 115–116).

***Ipomoea umbellata*** L., Syst. Nat., ed. 10, 2: 924. 1759. (Linnaeus 1759a: 924) = *Camonea umbellata* (L.) A.R. Simões & Staples

***Ipomoea umbellifera*** Choisy, Choisy in A.P. de Candolle, Prodr. 9: 89. 1845. (Choisy 1845: 389), nom. illeg. superfl. *Camonea umbellata* (L.) A.R. Simões & Staples

***Ipomoea uniflora*** Sessé & Moçiño, Fl. Mexic. 39 (La Naturaleza, Ser. 2, 2, append. 42). 1893. (Sessé y Lacasta and Moçiño 1891–97: 39). Type. MEXICO. *Sessé & Moçiño* s.n. (whereabouts unknown)

Uncertain species, possibly *I. ternifolia*.

***Ipomoea utilis*** Choisy in A.P. de Candolle, Prodr. 9: 375. 1845. (Choisy 1845: 375) Type. BRAZIL. Based on *Convolvulus pendulus* Silva Manso (see above).

Uncertain species of Clade A1. It is described as having tuberous ovoid-fusiform roots with oblong pendulous secondary tubers, oblong-cordate, acute, shortly petiolate leaves, a scabrous calyx with a truncate base, solitary sericeous flowers borne on short peduncles equalling the petioles

***Ipomoea variifolia*** var. ***saxatilis*** Pilger, Bot. Jahrb. 30: 185. 1902. (Pilger 1902: 185). Type. BRAZIL. Mato Grosso, Cuyabá, *Pilger* 514 (?B†).

Uncertain species. A sterile plant with 3-lobed leaves, possibly not *Ipomoea* and certainly not *I. variifolia*.

***Ipomoea velloziana*** Mart., Flora 21, Beibl. 2: 64. 1838. (Martius 1838: 64) = *Jacquemontia velloziana* (Mart.) O’Donell

***Ipomoea ventricosa*** (Bertero) G. Don, Gen. Hist. 4: 274. 1838. (Don 1838: 274) = Operculina turpethum var. ventricosa (Bertero) Staples & D.F. Austin

***Ipomoea ventricosa*** (Silva Manso) Stellfeld, Trib. Farm., Bras. 13: 86. 1945. (Stellfeld 1945: 86) = Operculina turpethum var. ventricosa (Bertero) Staples & D.F. Austin

***Ipomoea verticillata*** L., Syst. Nat. (ed. 10) 2: 924. 1759. (Linnaeus 1759b: 924) = *Jacquemontia verticillata* (L.) Urb.

***Ipomoea vespertina*** Colla, Hort. Rip. append. 3: 40 [153]. 1826. (Colla 1826b: 40 [153]). Type. A cultivated plant from Brazil, not found at TO. An uncertain species.

***Ipomoea viridis*** Choisy in A.P. de Candolle, Prodr. 9: 374. 1845. (Choisy 1845: 374). Type. BRAZIL. Minas Gerais, Taparoca, *Martius* 1181 s.n. (location unknown)

Uncertain species. The plant has cordate, acuminate, abaxially sericeous-tomentose leaves, a white corolla with a green base borne in 5-flowered cymes. Based on *Rambo* 44809 and 52115 O’Donell suggested this was the species currently known as *Ipomoea sulina* P.P.A. Ferreira & Miotto. This seems unlikely given the distance separating Rio Grande do Sul from Minas Gerais and, in any case, it is impossible to be certain in the absence of type material as Choisy’s description could fit several species.

***Ipomoea walpersiana*** Urb., Symb. Antill. 3(2): 345. 1902. (Urban 1902–3: 345). Type. GUADELOUPE. *Duchassaing* s.n. (holotype ?B†).

Described as a liana with glabrous stems, ovate-deltoid leaves with a truncate to cordate base and an inflorescence of many-flowered cymes, the peduncles 3–15 cm and the pedicels 15–20 mm. The bracteoles are noted as leaf-like, the sepals glabrous, rounded and very unequal, the outer 4–6 mm, the inner 7.5–8 mm, the corolla 5 cm long, funnel-shaped with an entire limb and the capsule globose with shortly tomentose seeds with pilose margins. It was reported to be close to *Ipomoea philomega* but not all details fit that species and in the absence of a type specimen it is impossible to be sure of its identity.

***Ipomoea yetira*** D. Parodi, Contr. Fl. Parag. 17. 1877. (Parodi 1877: 17). Type. PARAGUAY. Wet pastures, San Pedro (whereabouts unknown). An uncertain species, possibly *I. paludicola* or *I. asarifolia* because of the habitat and the transversely rugose sepals. Both species are recorded from San Pedro.

***Pharbitis livescens*** Schlecht. ex Kunze., Linnaea 20: 32. (Kunze 1847: 32). Type. Plant cultivated from seed sent by Bescke from Brazil.

Uncertain species, possibly a form of *Ipomoea indica*. It was described as a pubescent perennial with 3-lobed leaves, abaxially purple, and 2–4-flowered cymes with oblong-ovate bracteoles.

***Pharbitis ostrina*** Lindl. Bot. Reg. 28: t. 51. 1842. (Lindley 1842: t. 51). Type. Not specified, but the illustration was reported to be based on a Cuban plant and shows a plant of Clade A2 but with a 3-lobed stigma and triloculate ovary, elements that are contradictory. The roots were apparently tuberous.

Excluding the ovary and style the plant could be *Ipomoea mauritiana* but the ovary and style suggest a plant from the Pharbitis Clade.

***Quamoclit solanifolia*** (L.) Choisy in A.P. de Candolle, Prodr. 9: 335. 1845. (Choisy 1845: 335) = *Jacquemontia solanifolia* (L.) Hallier f.

***Stomadena violacea*** Raf., Fl. Tellur. 2: 12 1836 [pub.1837]. (Rafinesque 1837: 12). An unidentified species of *Ipomoea*.

## Nomenclatural correction to Muñoz-Rodríguez et al. (2019), supplementary information

The basionym of *Ipomoea luzonensis* (Hallier f.) J.R.I. Wood & Scotland was not correctly cited in Muñoz-Rodríguez et al. (2019: 28). This is *Rivea luzonensis* Hallier f., Bull. Herb. Boiss. 6(9): 714 (1898) so the valid combination *Ipomoea luzonensis* (Hallier f.) J.R.I. Wood & Scotland, comb. nov. based on *Rivea luzonensis* Hallier f., is made here. This species was previously known as *Argyreia luzonensis* (Hallier f.) Ooststr., Blumea 5: 379 (1943).

The nom. nov. *Ipomoea chengyiwuiensis* should be corrected to *Ipomoea chengyiwuana* J.R.I. Wood & Scotland, nom. nov., based on *Argyreia eriocephala* C.Y.Wu, Yunnan Trop. Subtrop. Fl. Res. Rep. 1: 125 (1965), non *Ipomoea eriocephala* Moric.

The combination *Ipomoea philippinensis* (Merr.) J.R.I.Wood & Scotland (Munoz-Rodriguez et al. (2019: 30) is an illegitimate later homonym of *I. philippinensis* Choisy, Mém. Soc. Phys. Genève 6: 475 (1833). *Argyreia philippinensis* (Merr.) Ooststr. with basionym: *Lettsomia philippinensis* Merr. in Philipp. J. Sci. 26: 488 (1925) is here renamed *Ipomoea merrillii* J.R.I.Wood & Scotland, nom. nov.

Ipomoea baccata J.R.I.Wood & Scotland var. minor (C.B.Clarke) J.R.I.Wood & Scotland (Munoz-Rodriguez et al. (2019: 30) was not validly published as a new combination, as reference was not made to the original basionym, Lettsomia setosa var. minor C.B.Clarke in Hook.f., Fl. Brit. India 4: 194 (1883). The following combination is here validated by reference to this basionym Ipomoea baccata J.R.I.Wood & Scotland var. minor (C.B.Clarke) J.R.I.Wood & Scotland, comb. nov.

We thank Jonathan Krieger (International Plant Names Index at Kew) for help with these issues.
